# Supplementary figures and images for: Trends in disease incidence and survival and their effect on mortality in Scotland: nationwide cohort study of linked hospital admission and death records 2001–2016
Source: BMJ Open. 2020 Mar 25;10(3):e034299. doi: 10.1136/bmjopen-2019-034299 (PMC7170664; doi:10.1136/bmjopen-2019-034299)

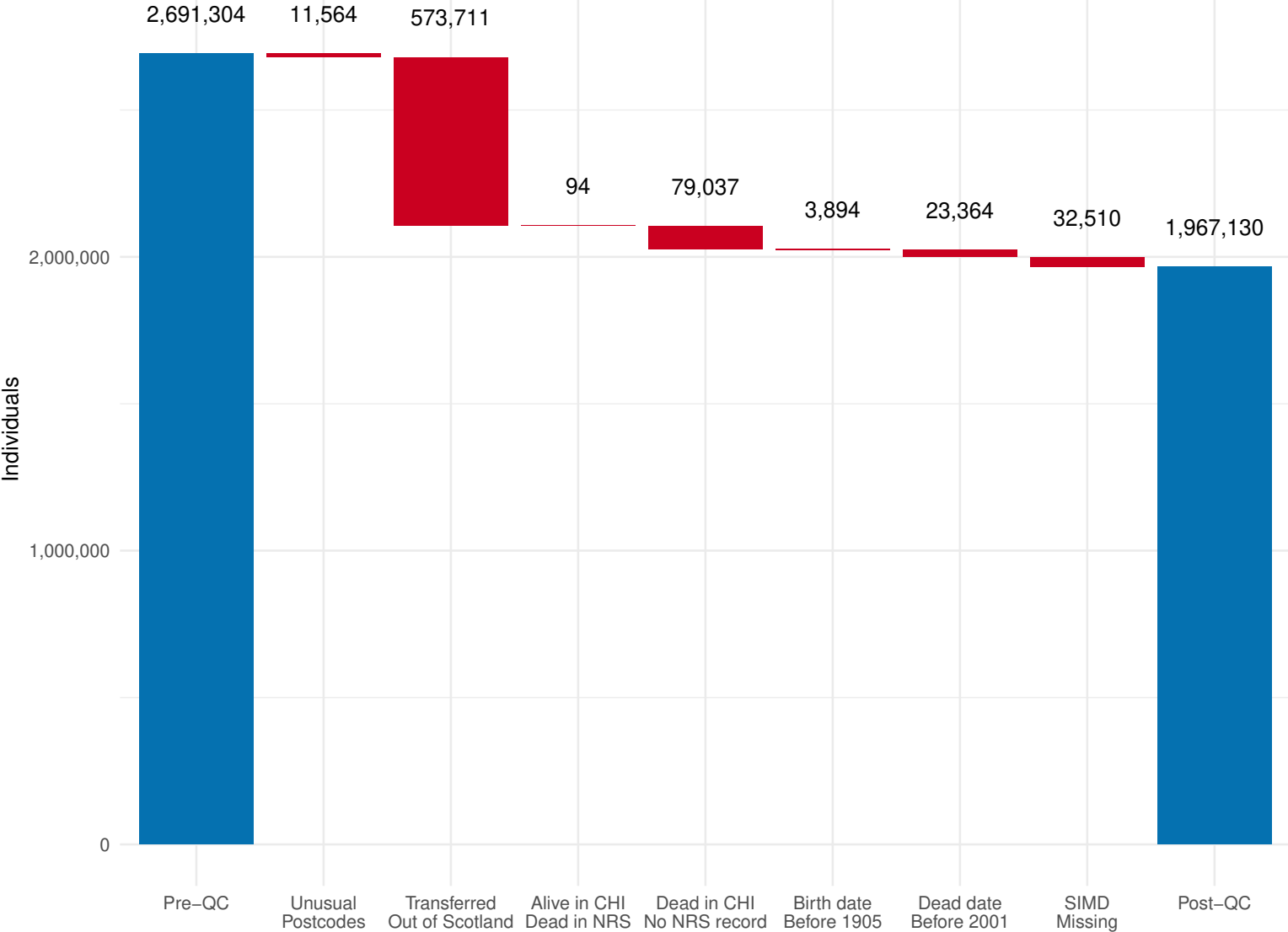

Supplement: Supplementary data [file bmjopen-2019-034299supp001.pdf]

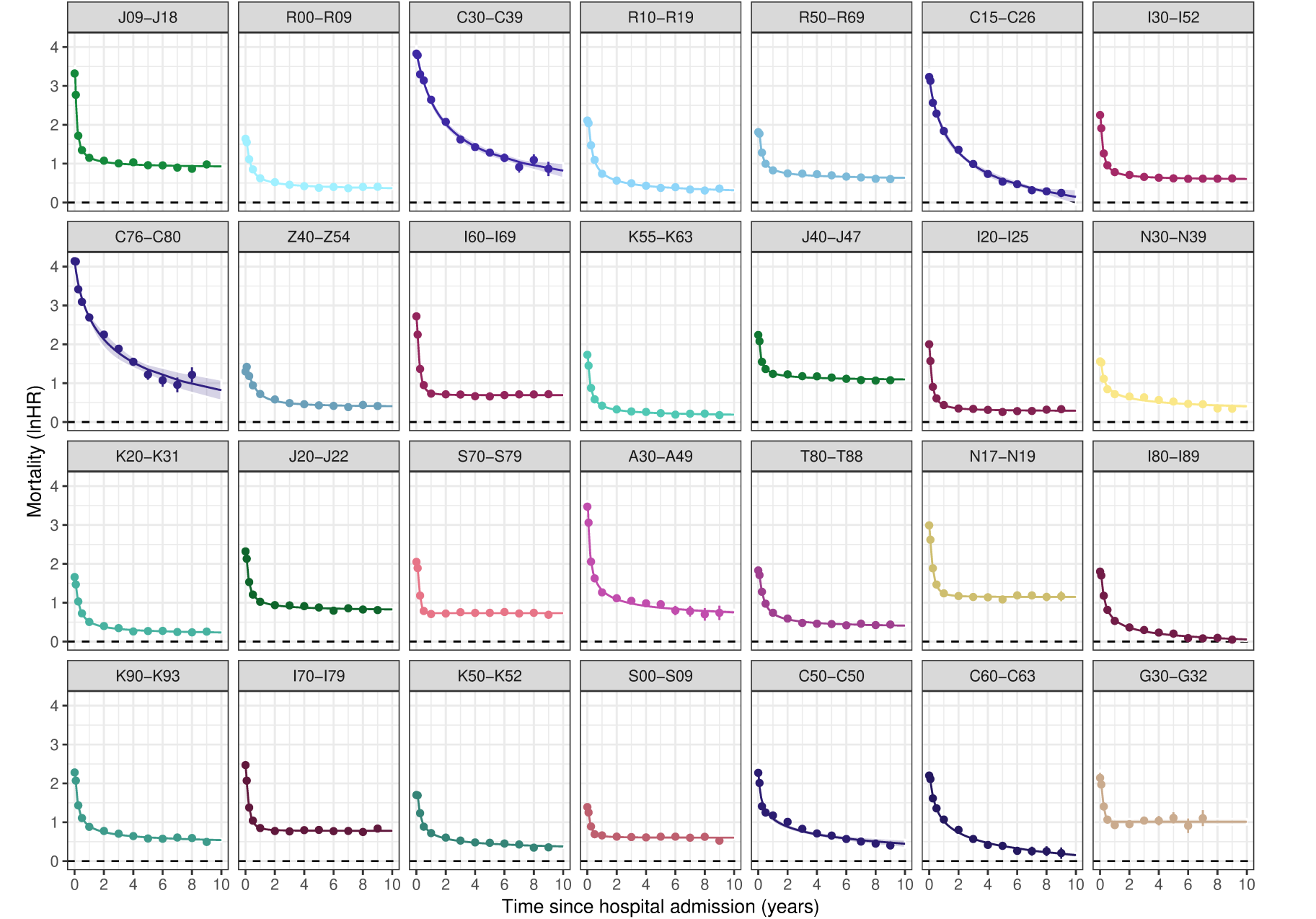

Supplement: Supplementary data [file bmjopen-2019-034299supp005.pdf]

**A** Improvements in hospital admission rate  
Change in lnHR from one decade to the next

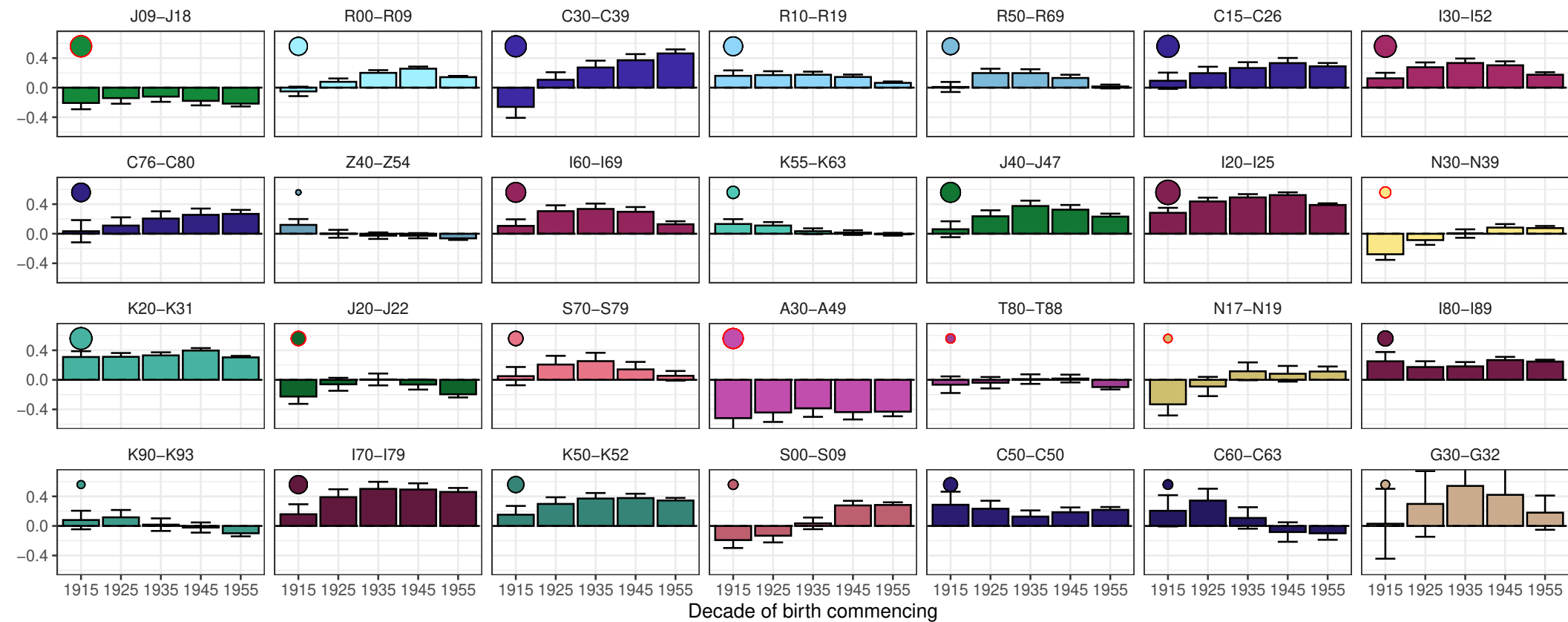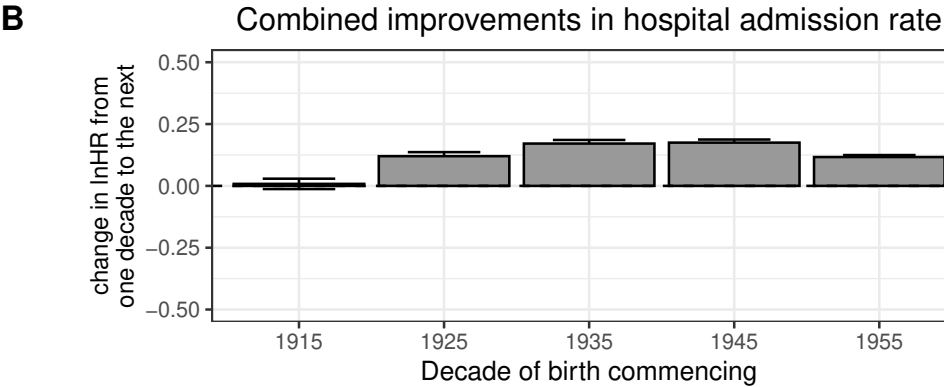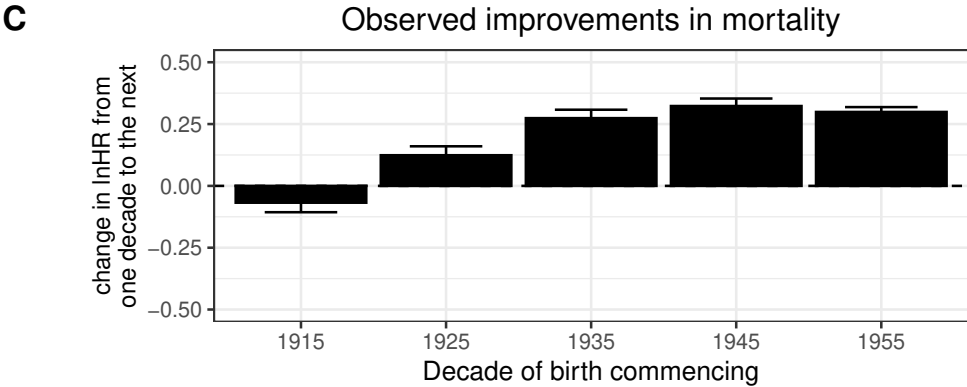

Supplement: Supplementary data [file bmjopen-2019-034299supp006.pdf]

| Effect  | lnHR  | SE    |
|---------|-------|-------|
| Female  | -0.35 | 0.003 |
| 1915    | 0.95  | 0.015 |
| 1925    | 1.02  | 0.013 |
| 1935    | 0.90  | 0.013 |
| 1945    | 0.62  | 0.012 |
| 1955    | 0.30  | 0.010 |
| SIMD 2  | 0.07  | 0.007 |
| SIMD 3  | 0.16  | 0.006 |
| SIMD 4  | 0.22  | 0.006 |
| SIMD 5  | 0.29  | 0.006 |
| SIMD 6  | 0.35  | 0.006 |
| SIMD 7  | 0.41  | 0.006 |
| SIMD 8  | 0.49  | 0.006 |
| SIMD 9  | 0.59  | 0.006 |
| SIMD 10 | 0.73  | 0.006 |

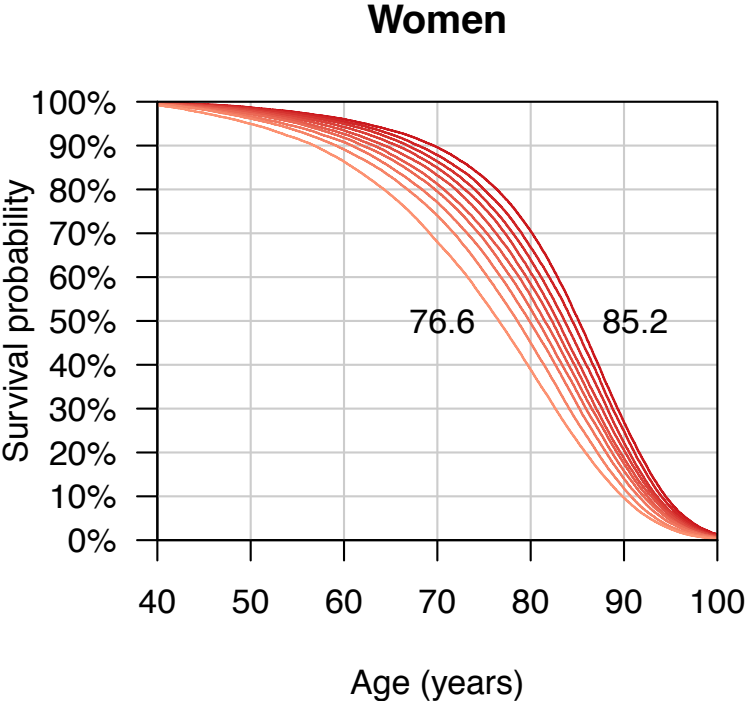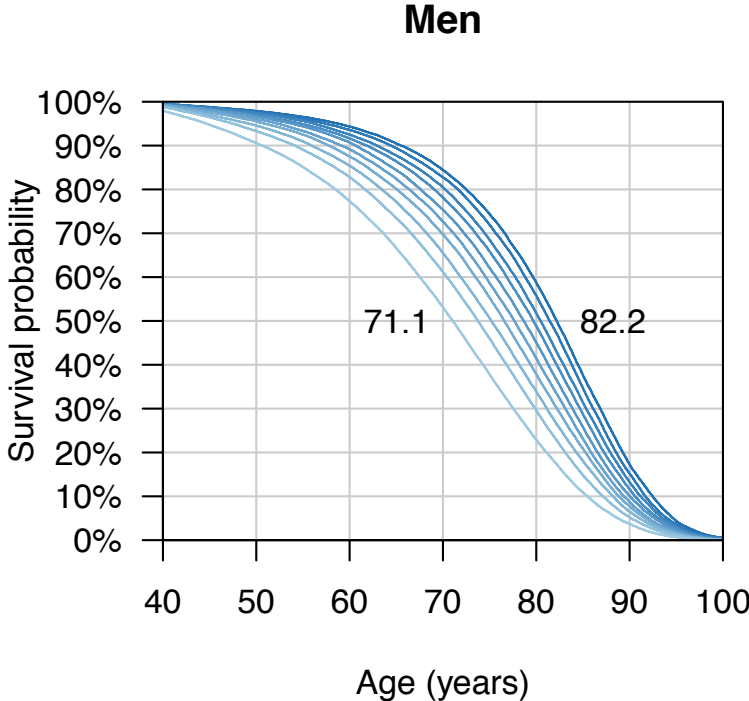

Supplement: Supplementary data [file bmjopen-2019-034299supp010.pdf]

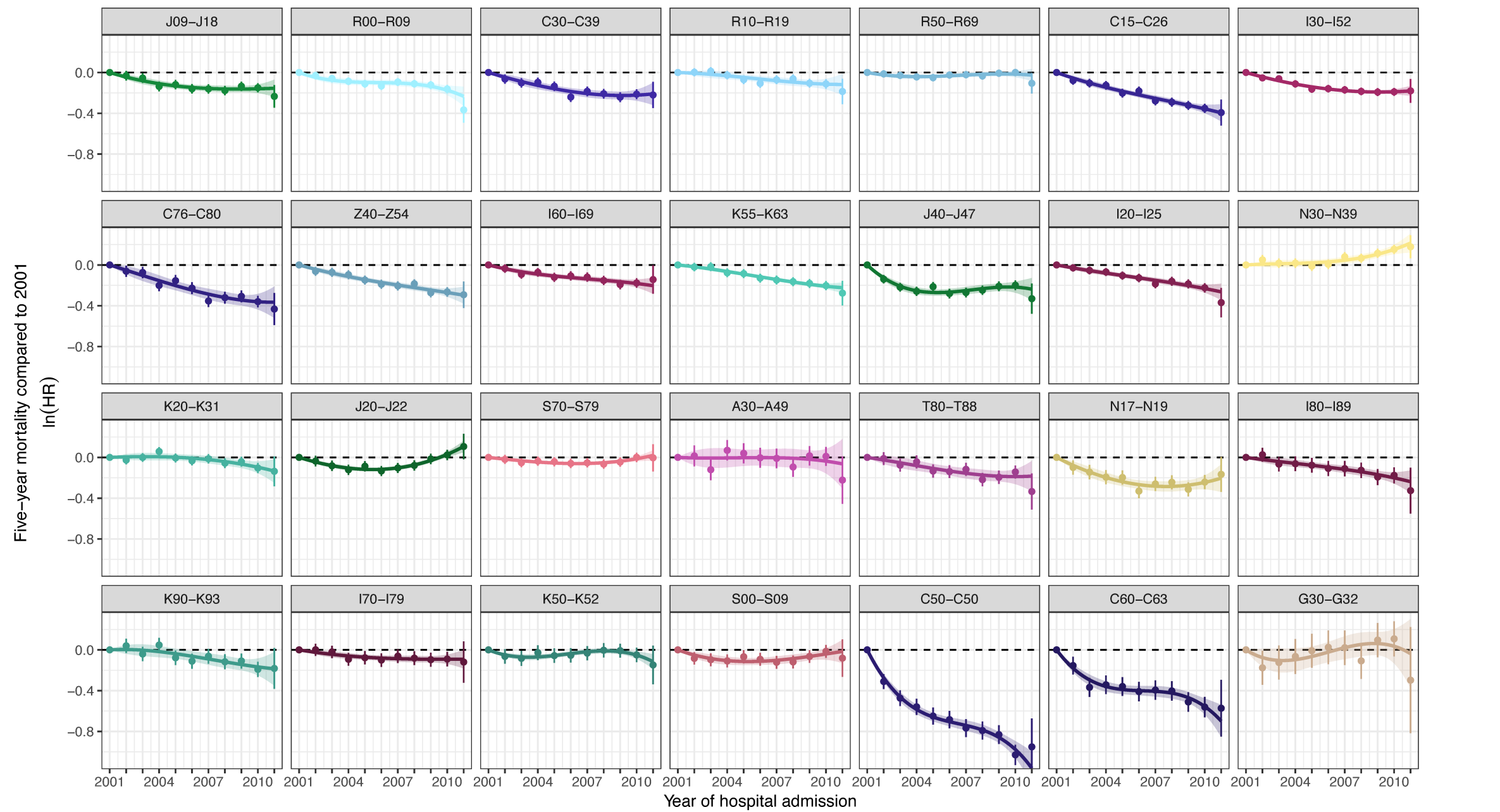

Supplement: Supplementary data [file bmjopen-2019-034299supp013.pdf]

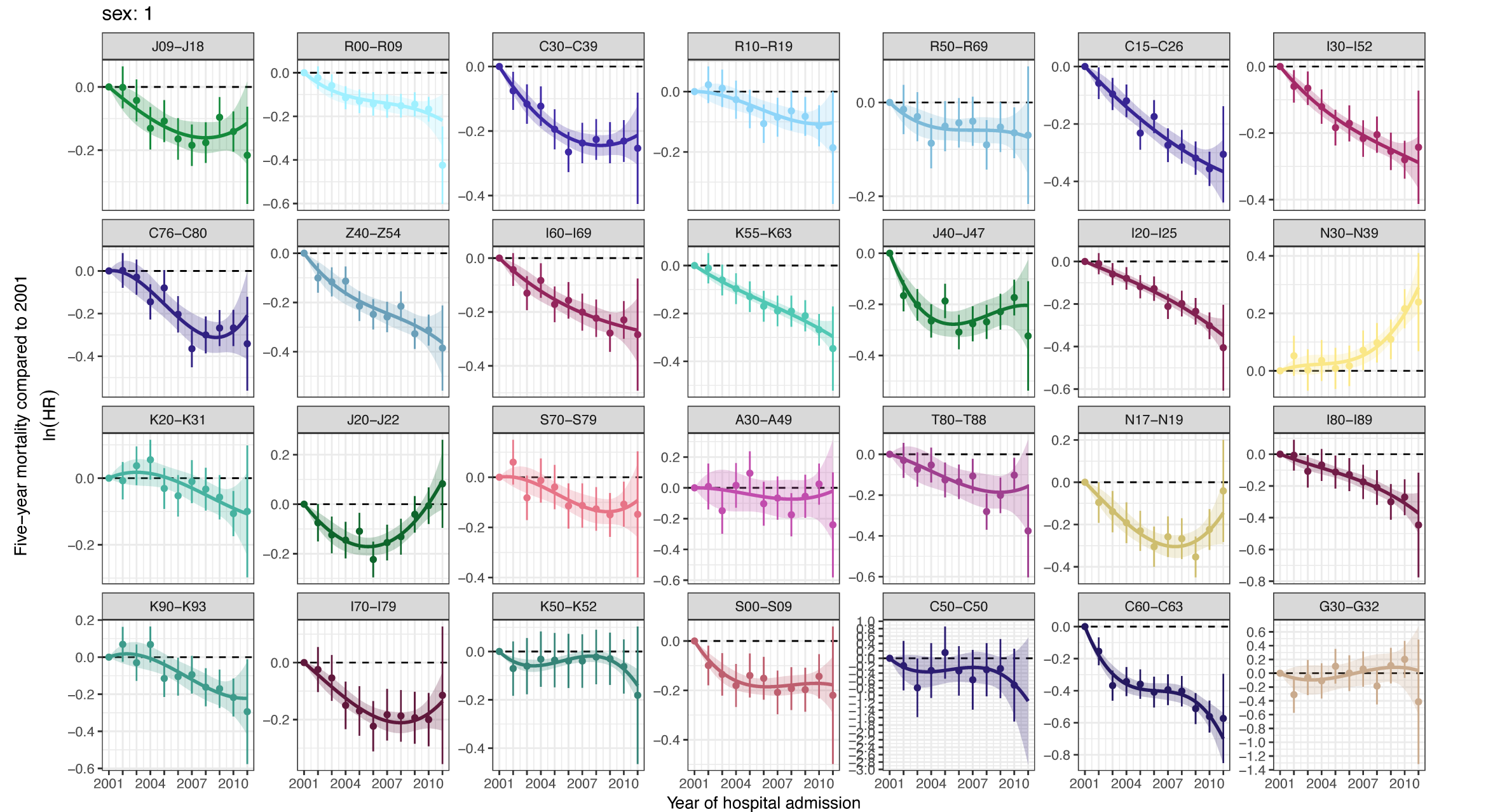

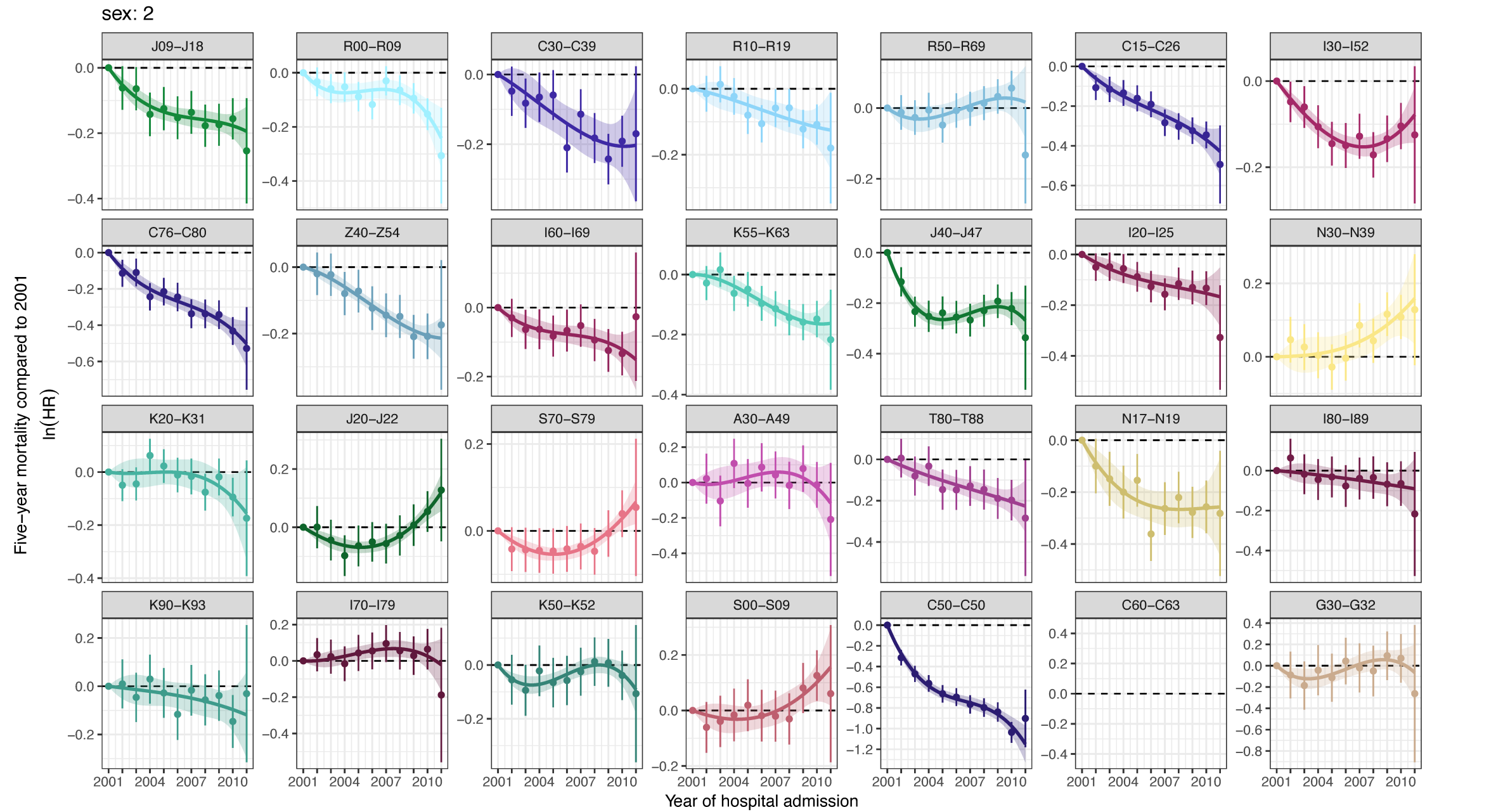

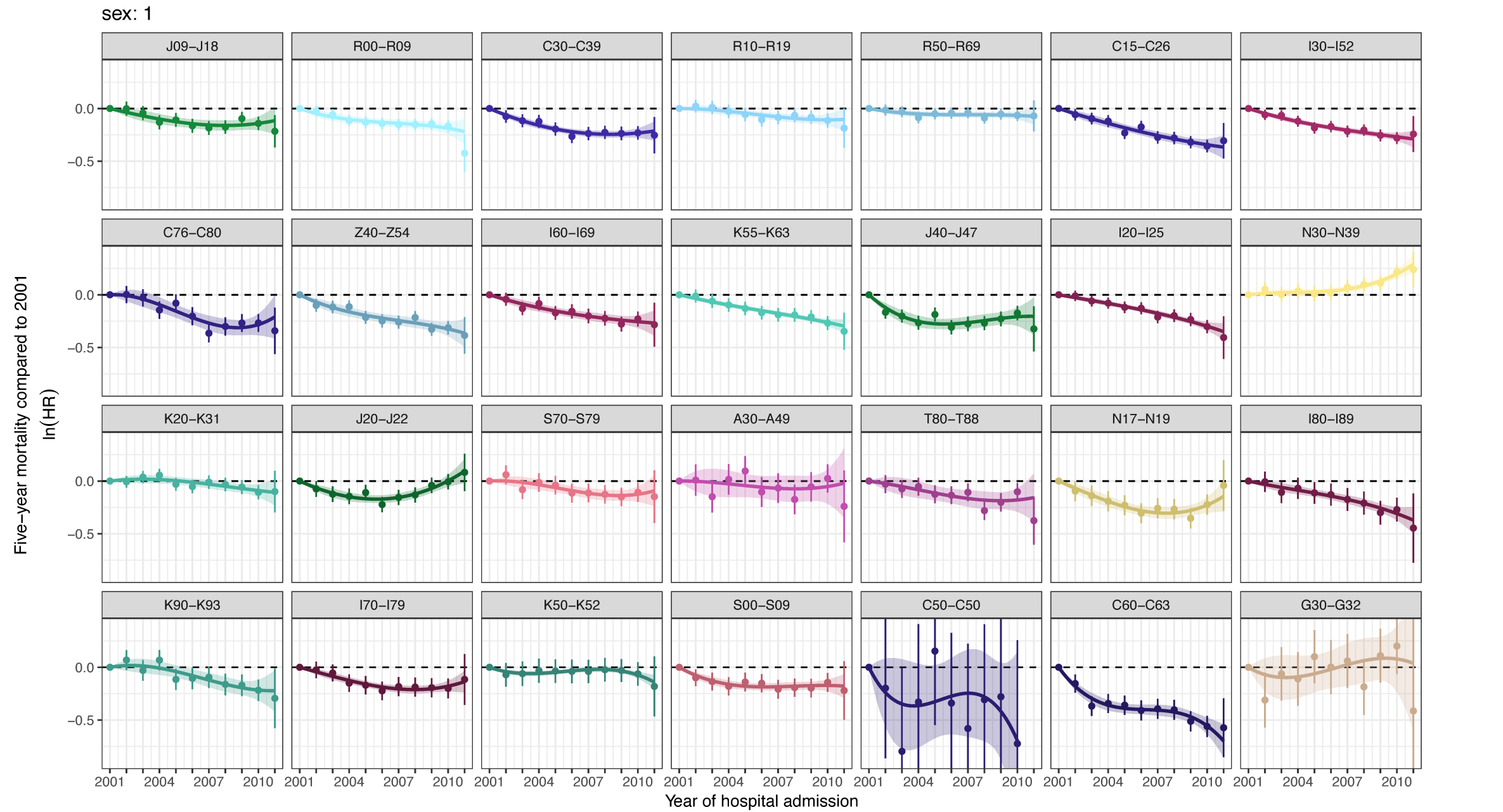

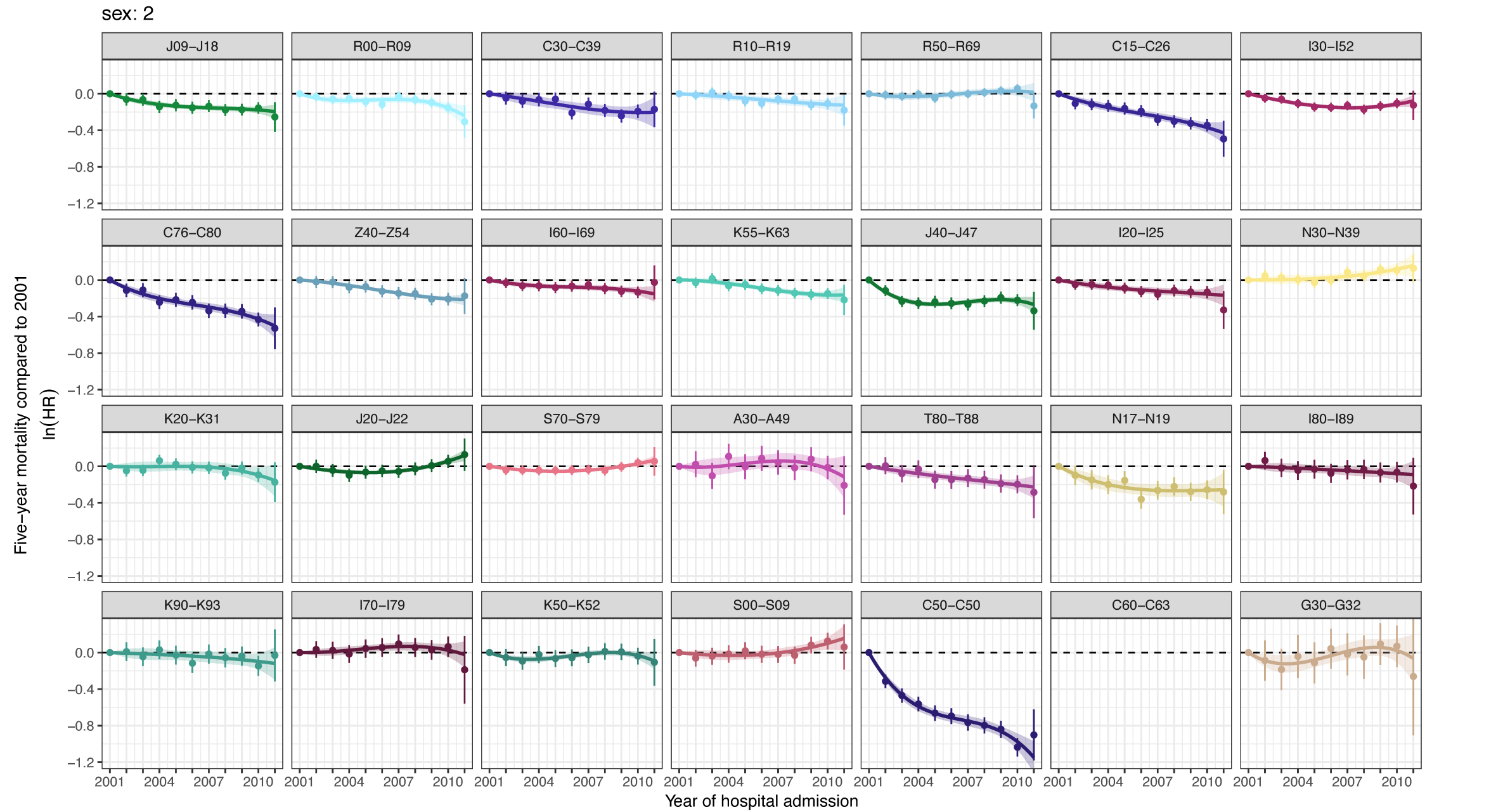

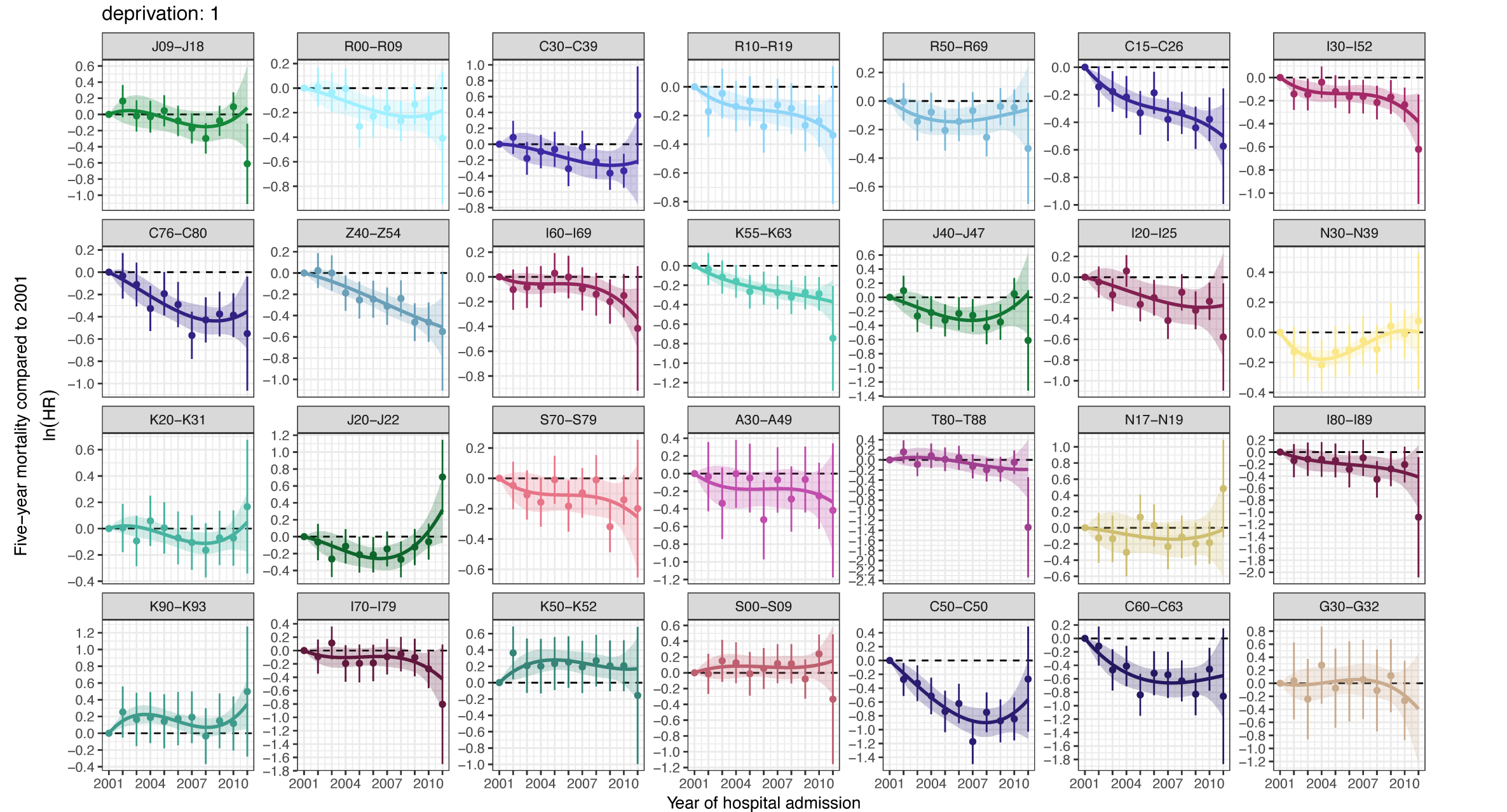

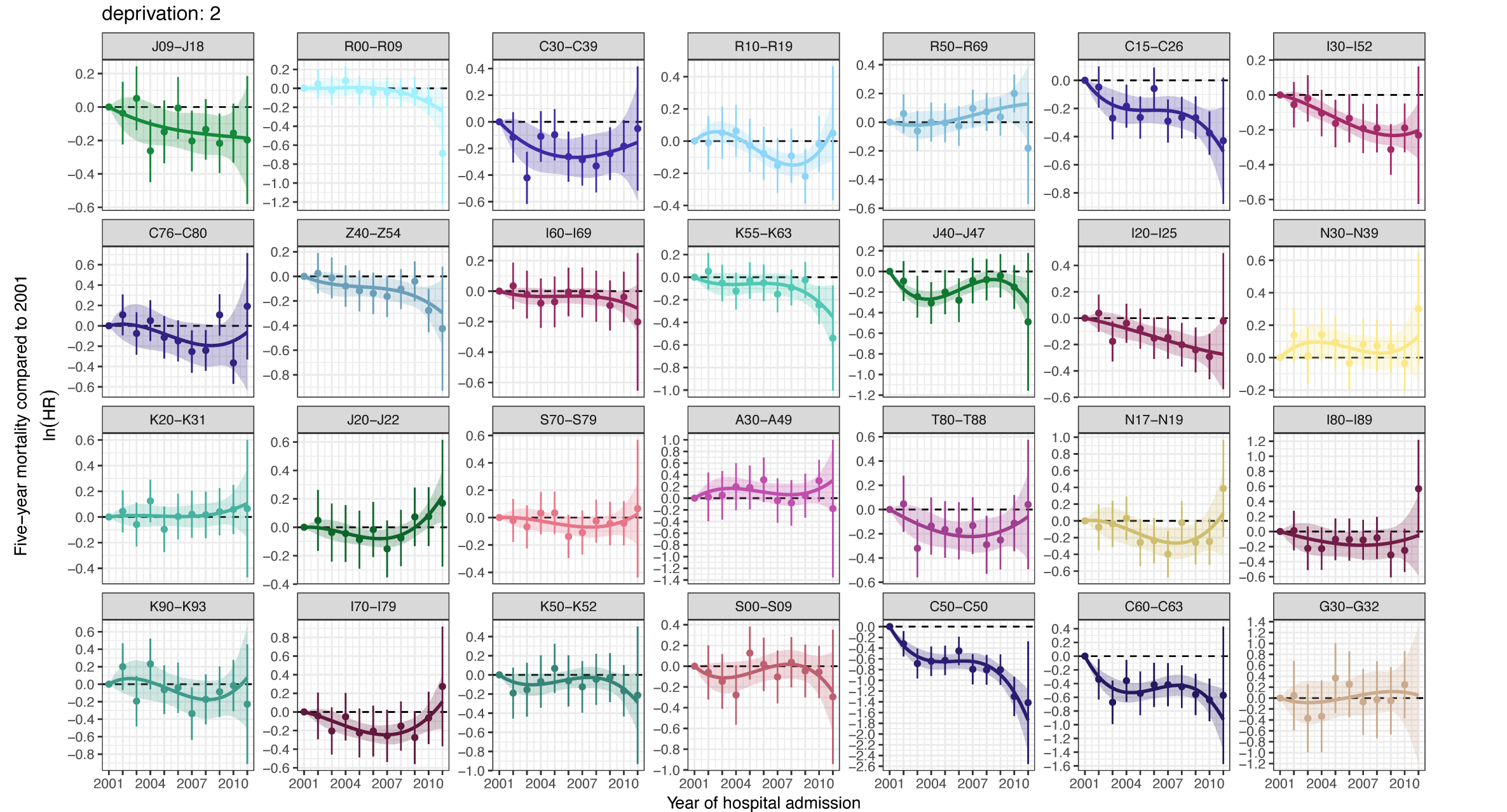

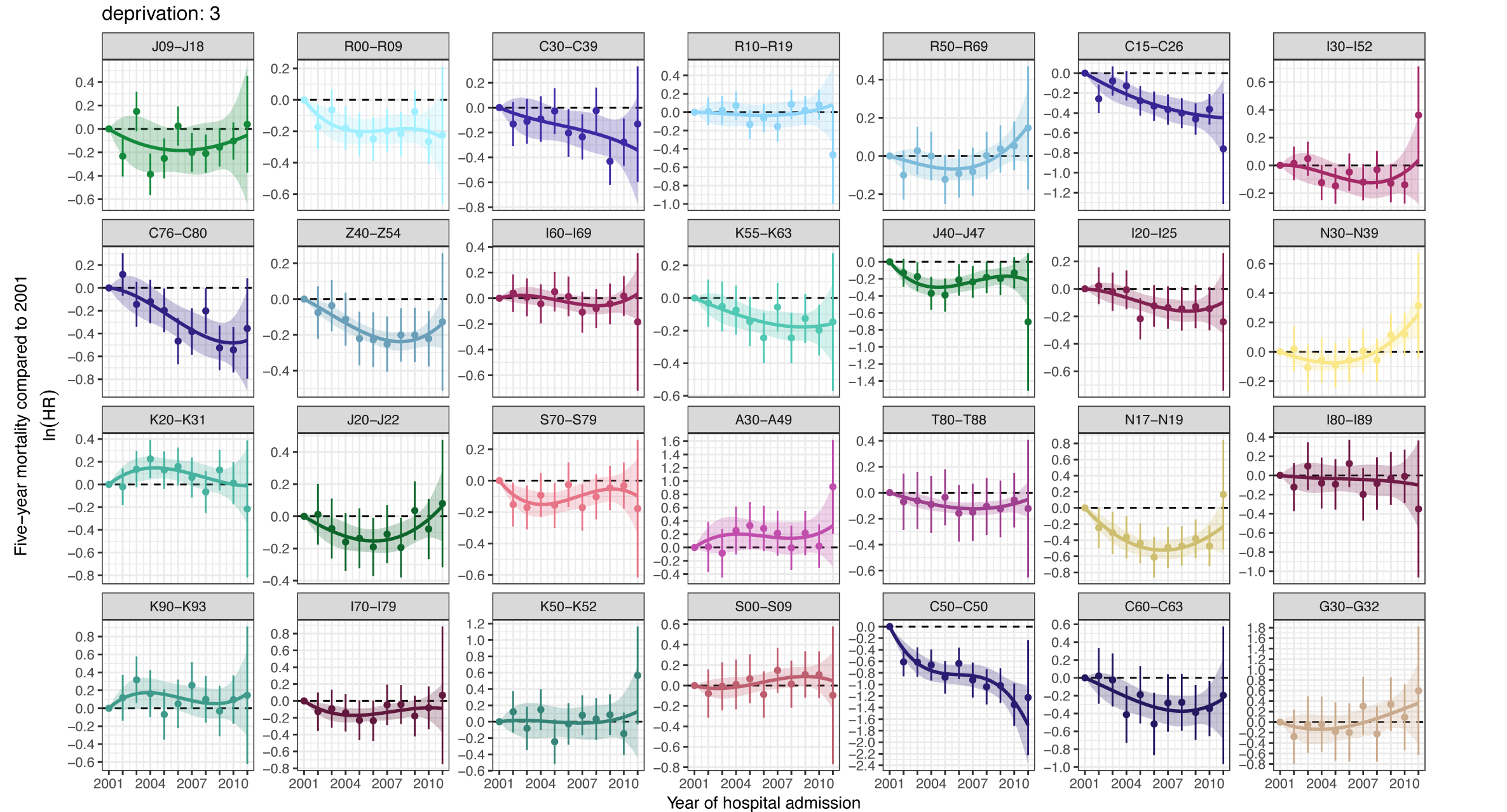

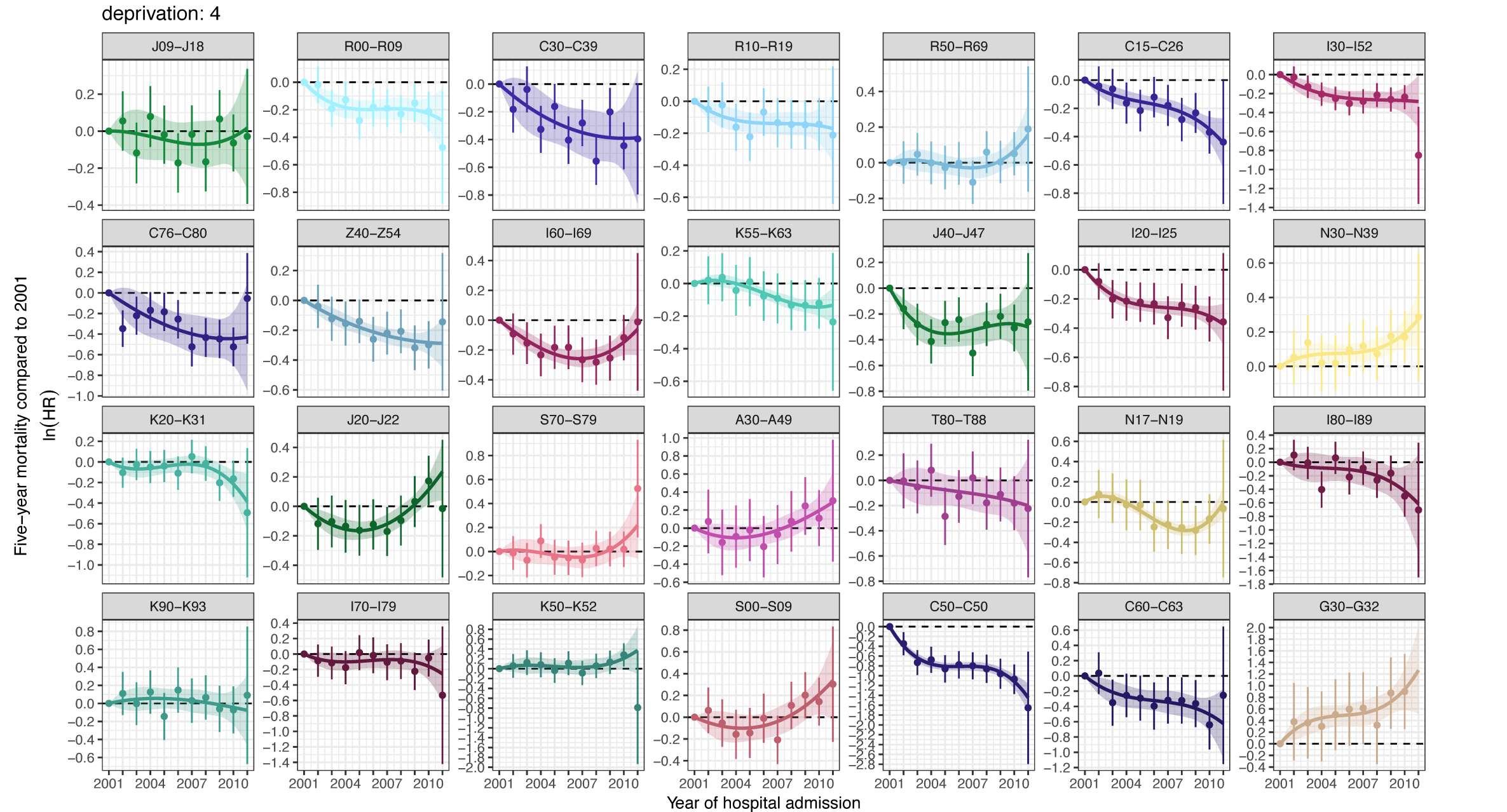

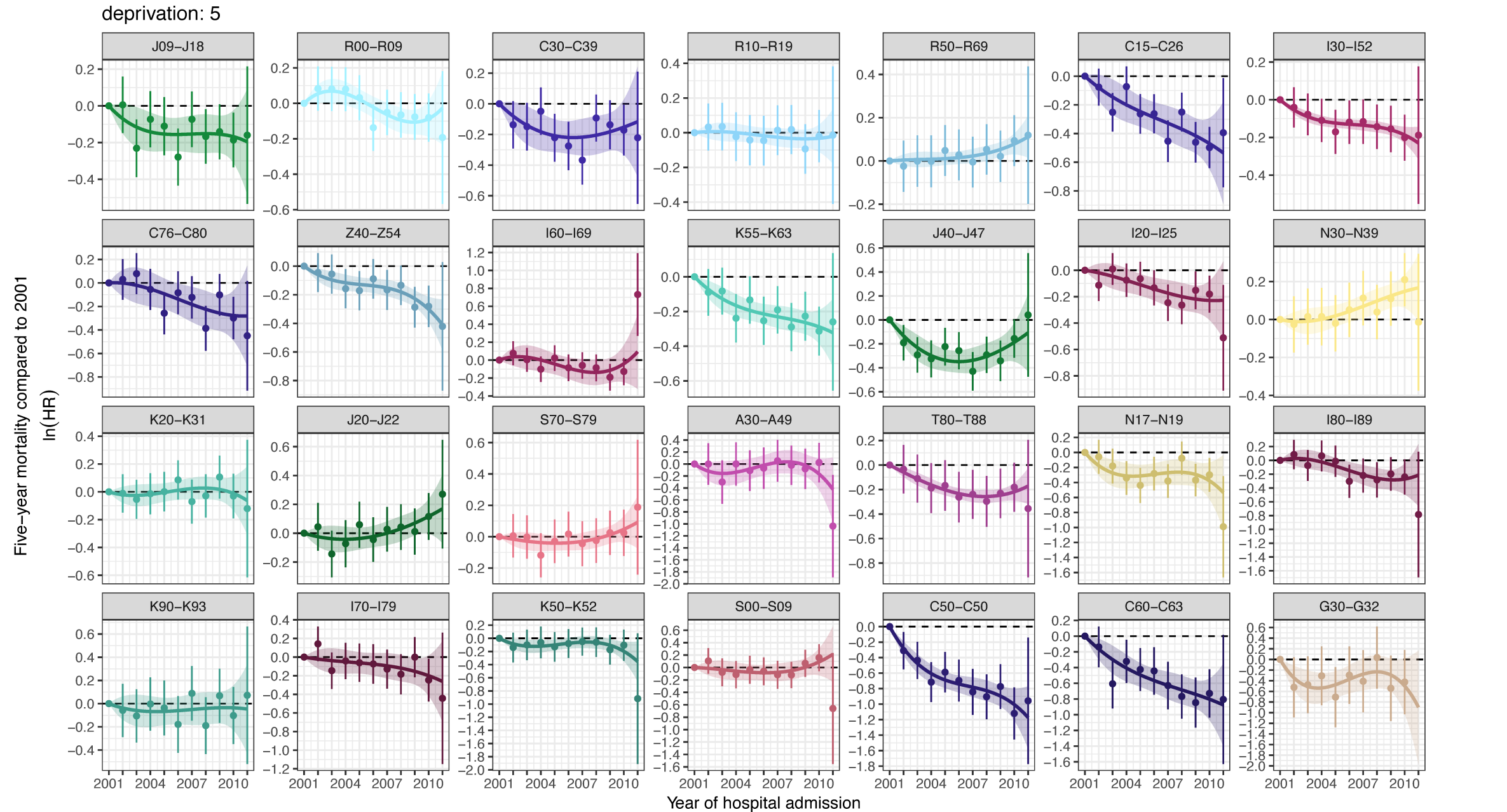

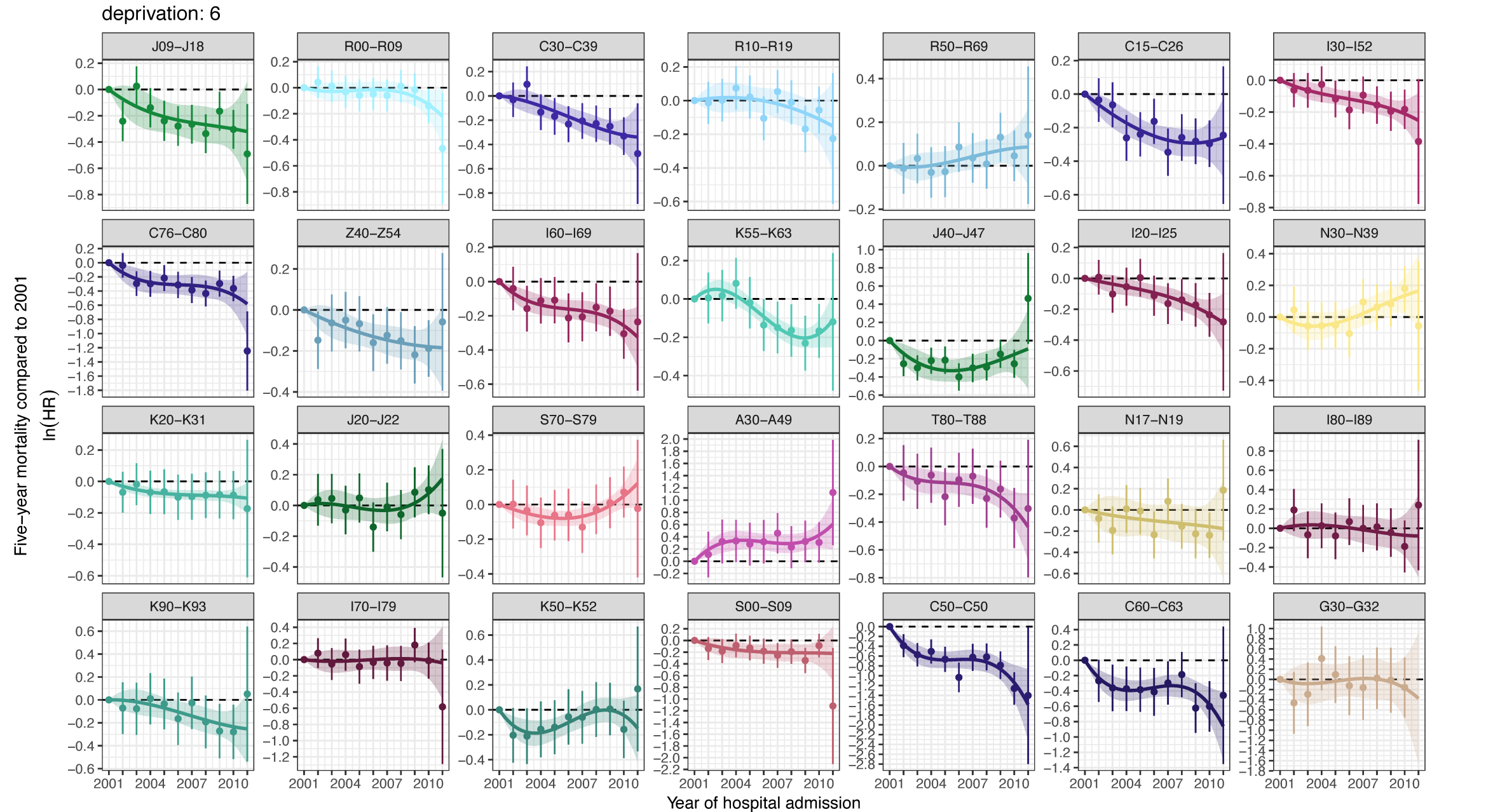

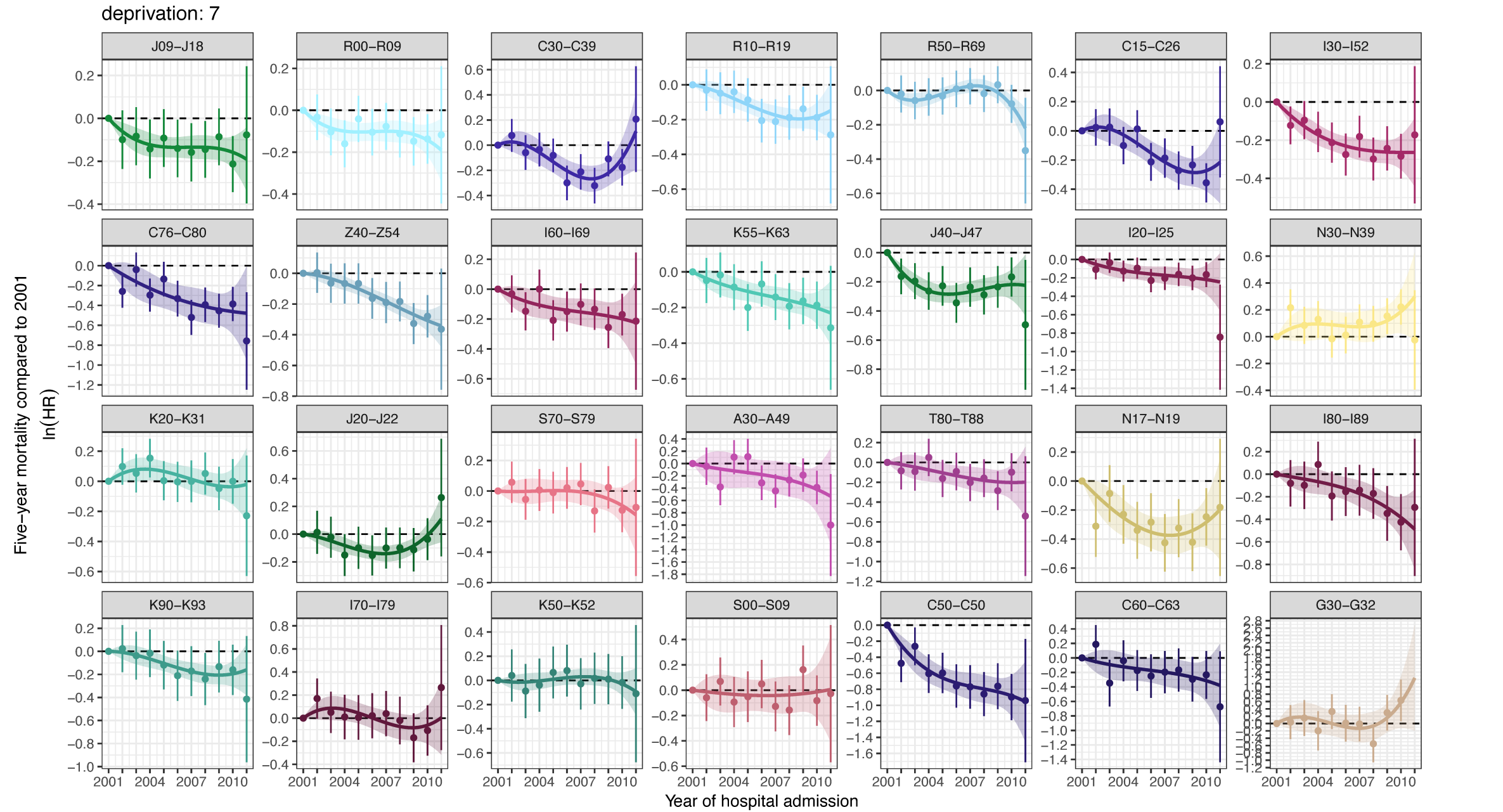

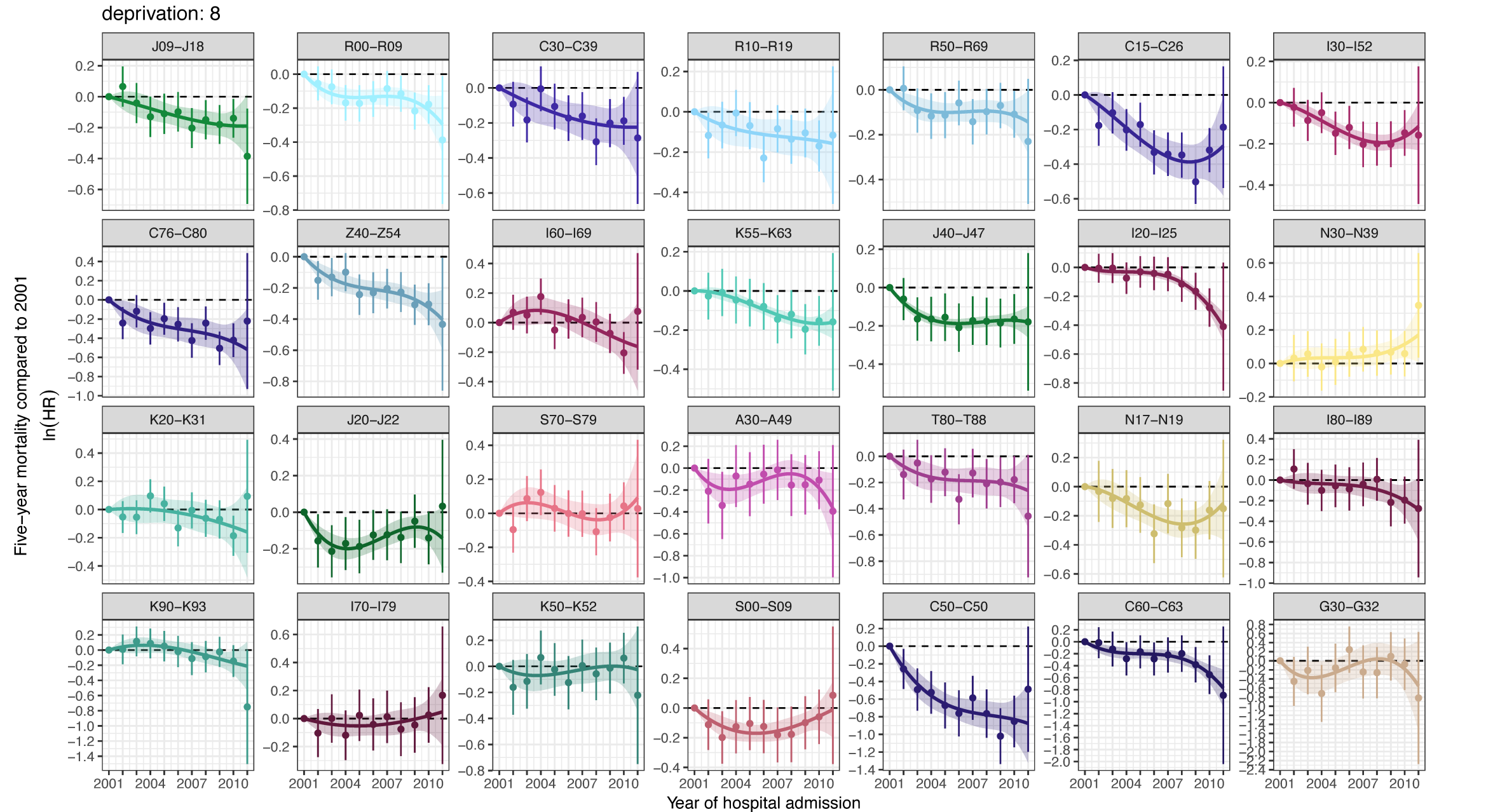

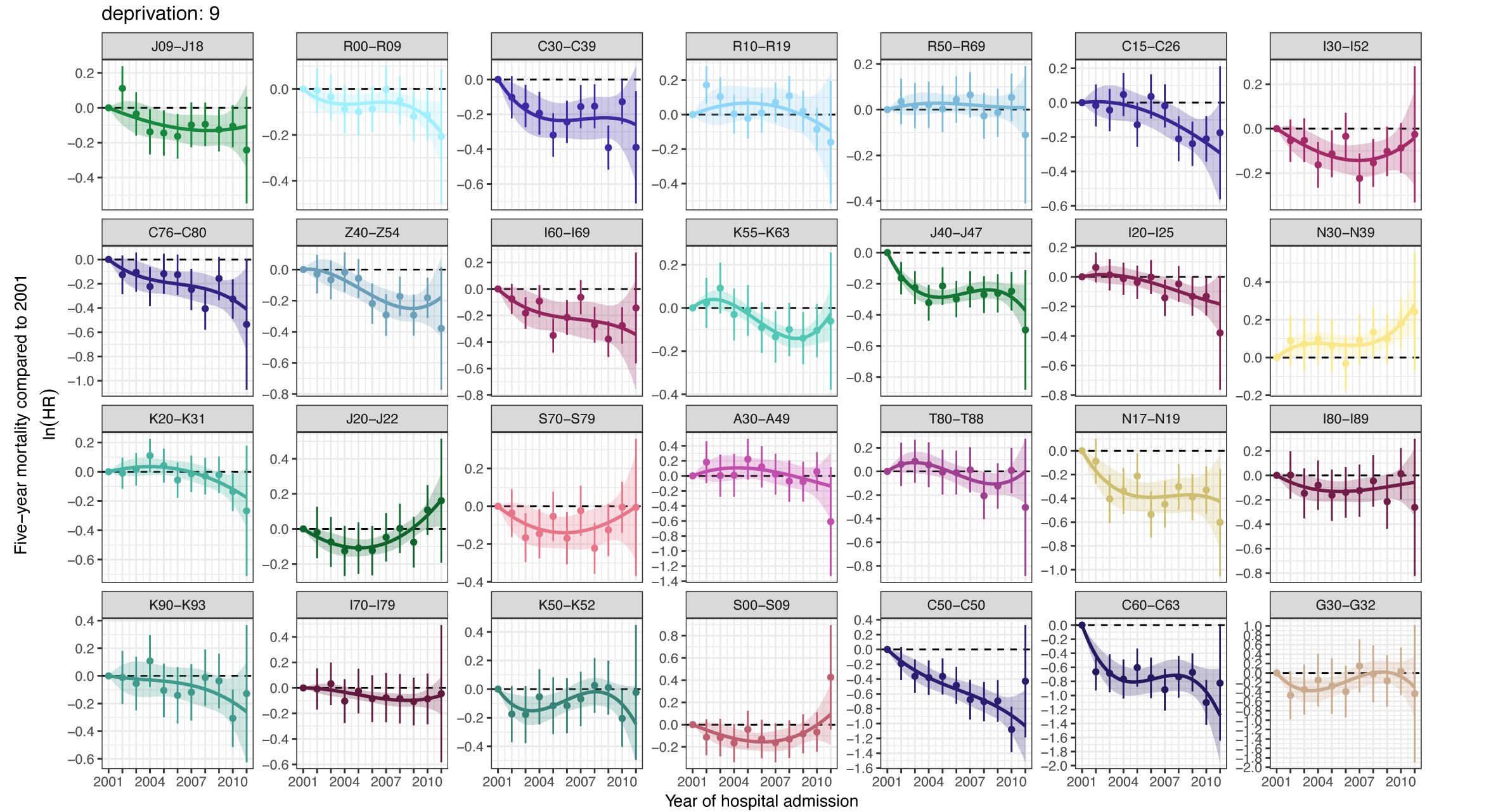

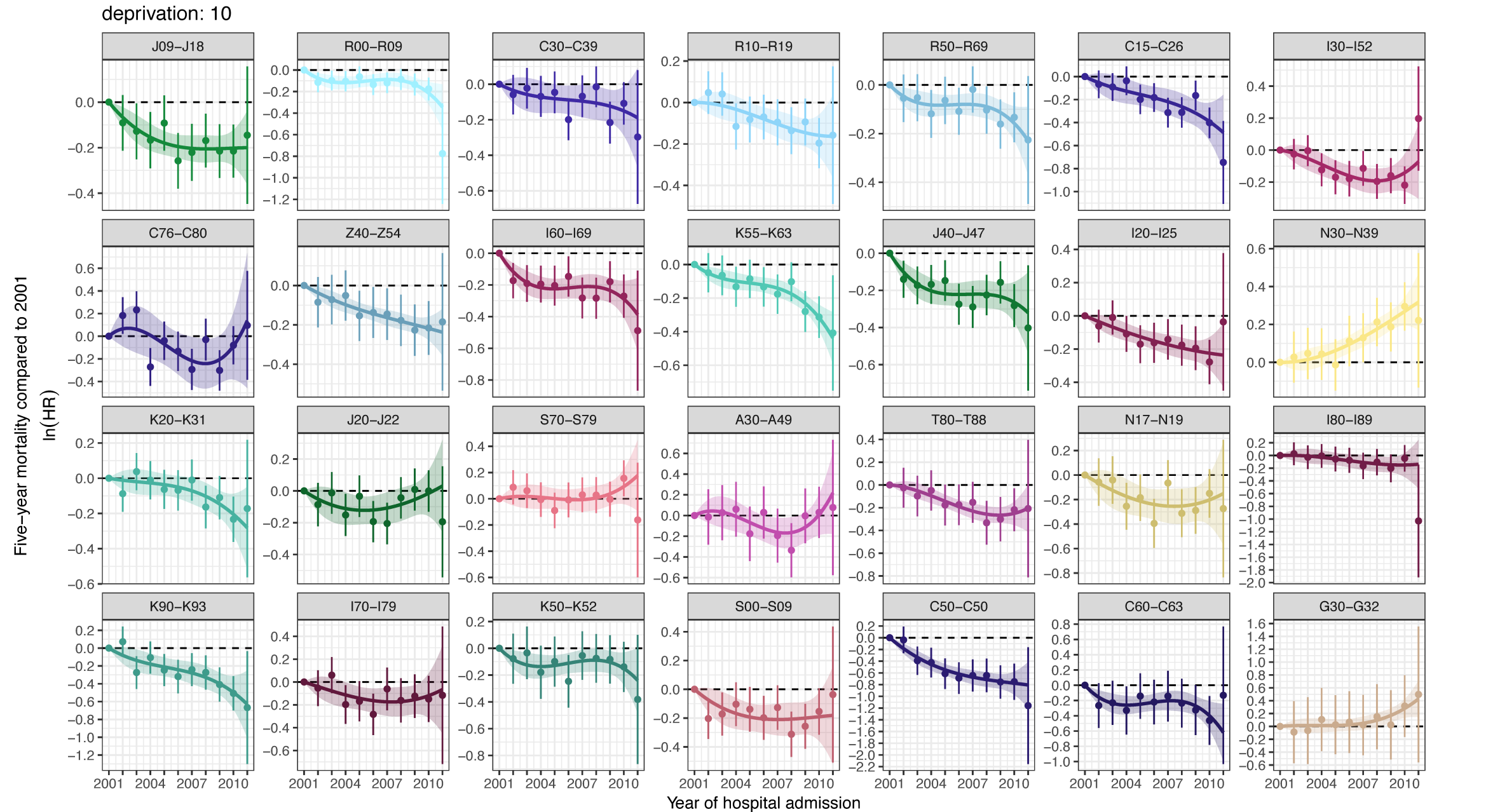

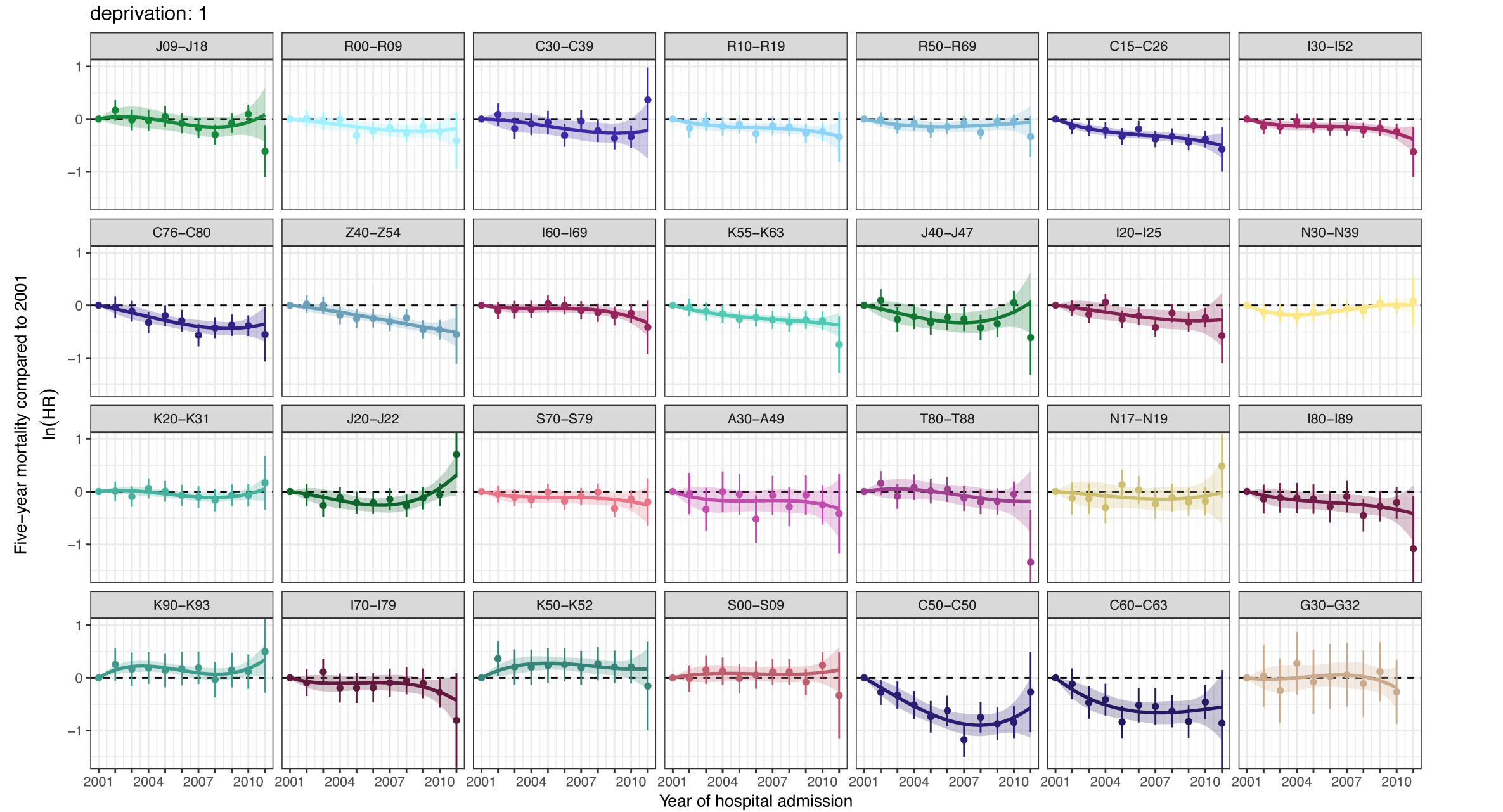

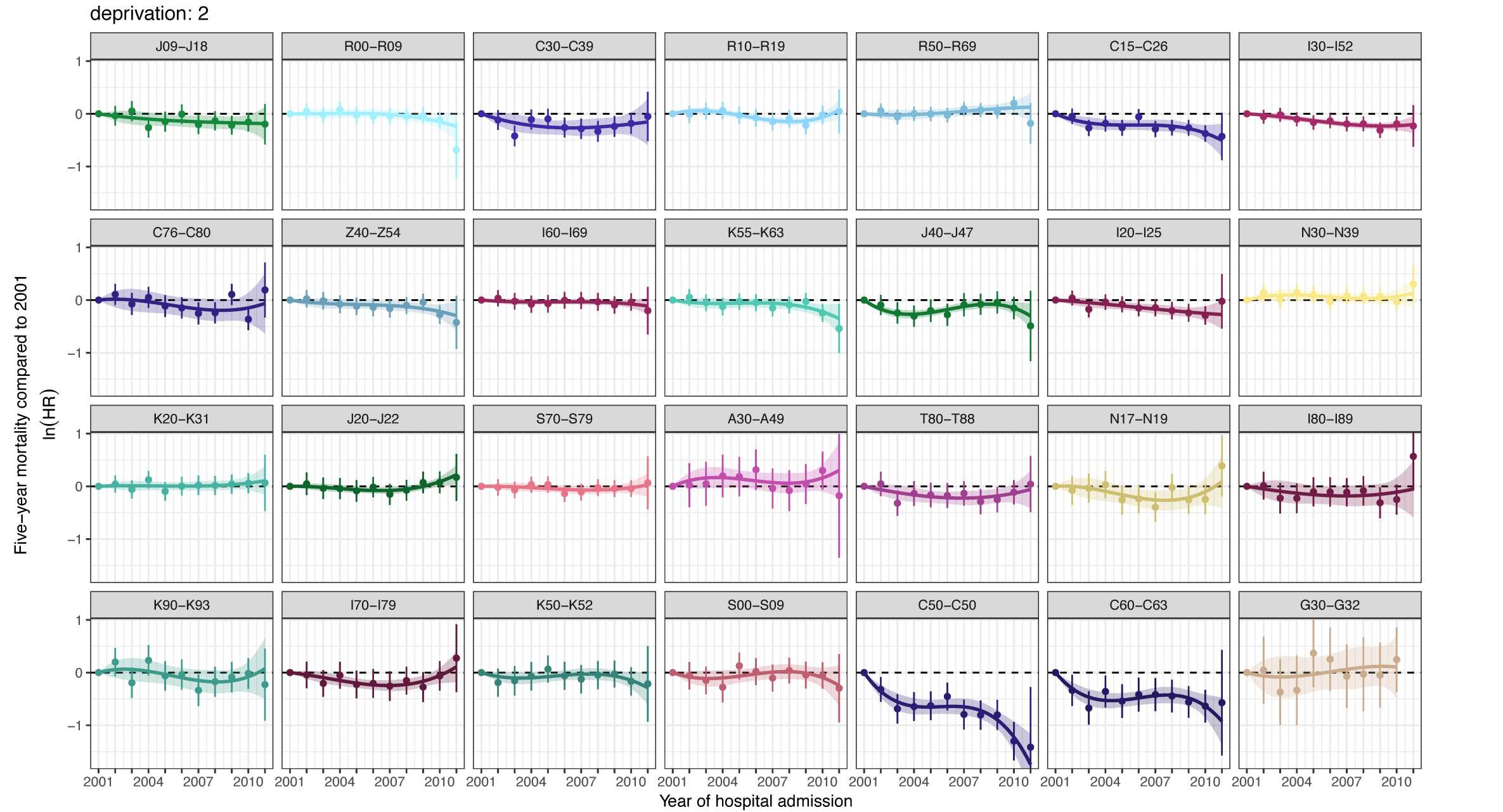

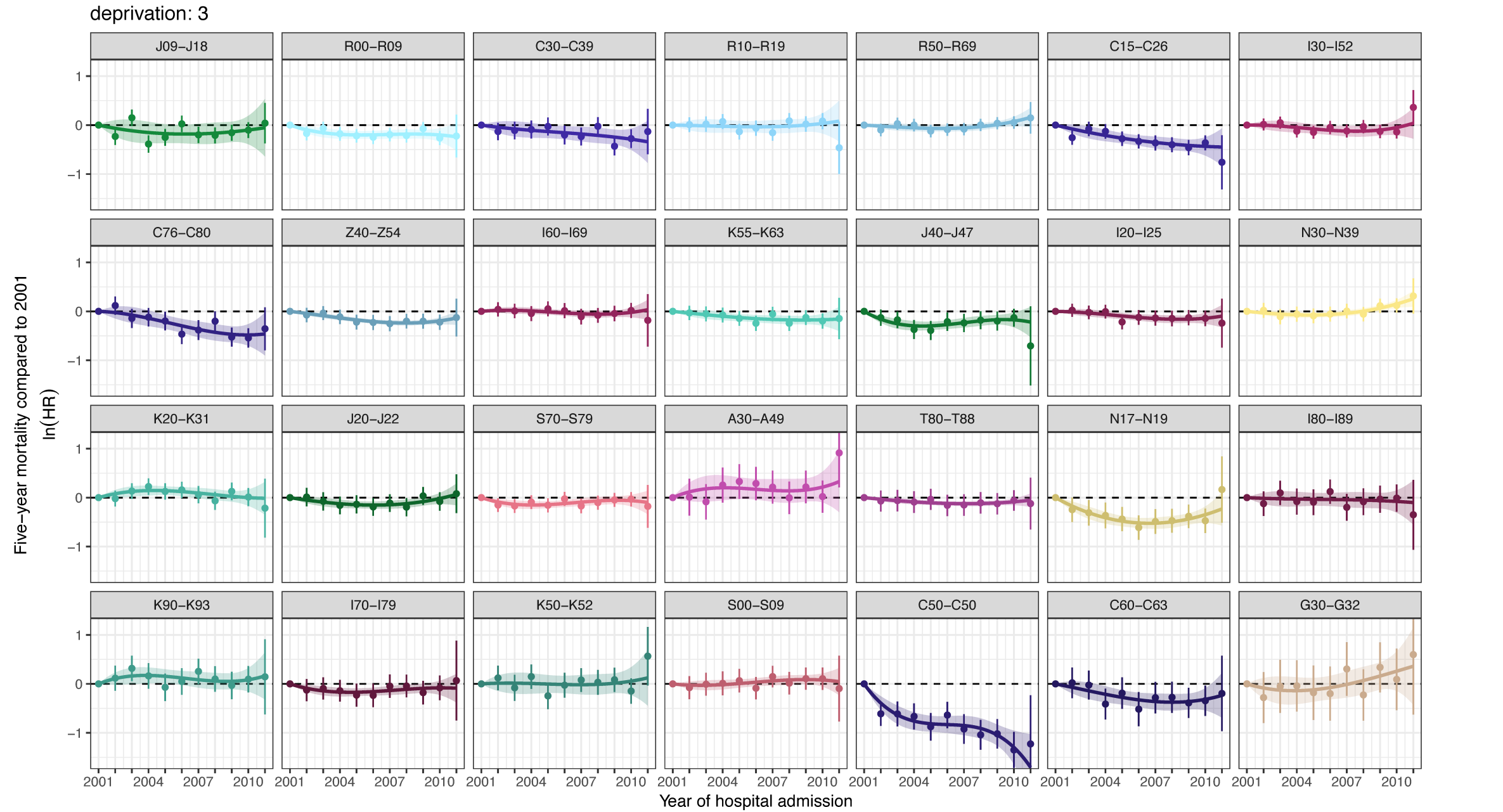

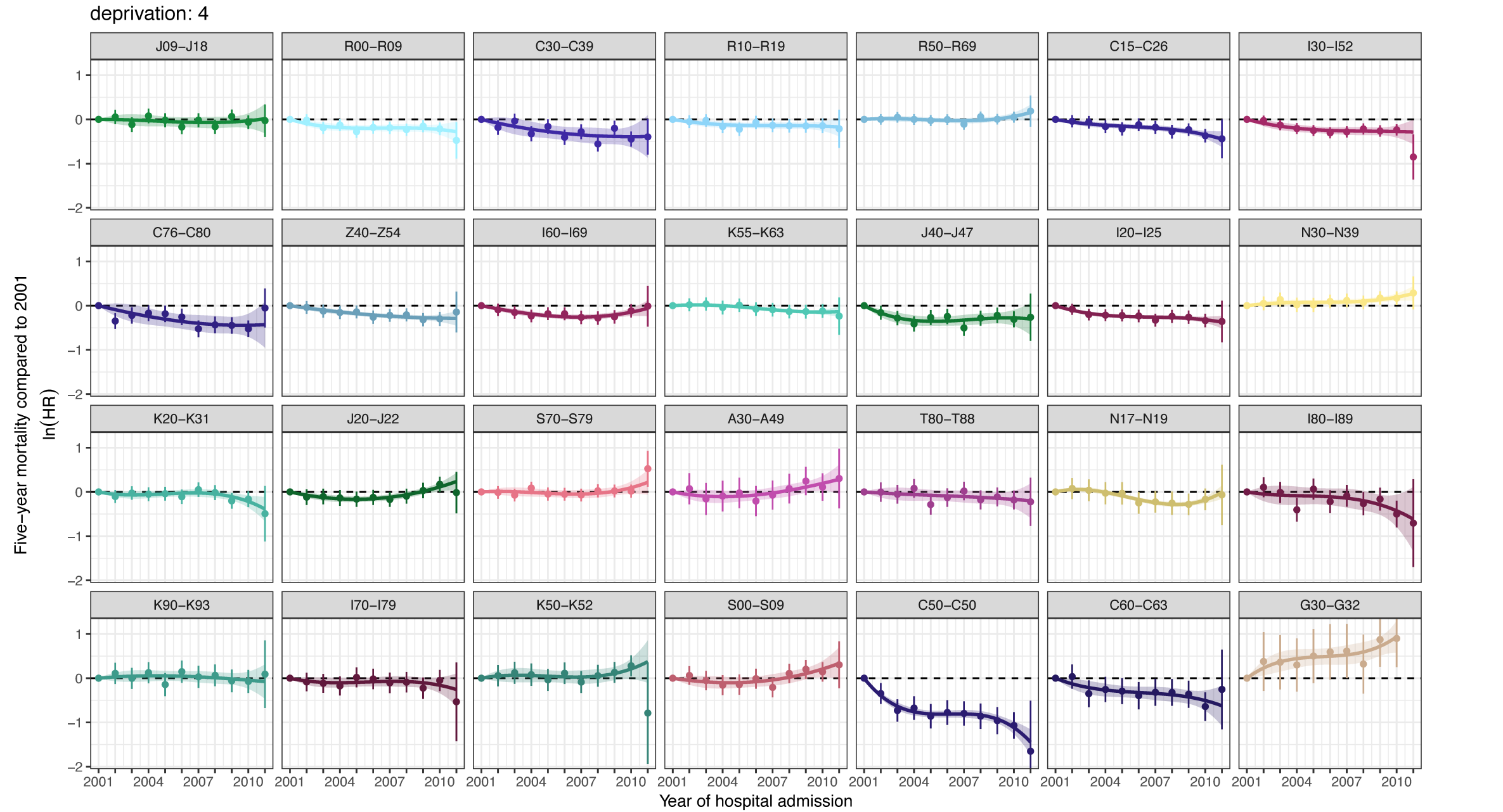

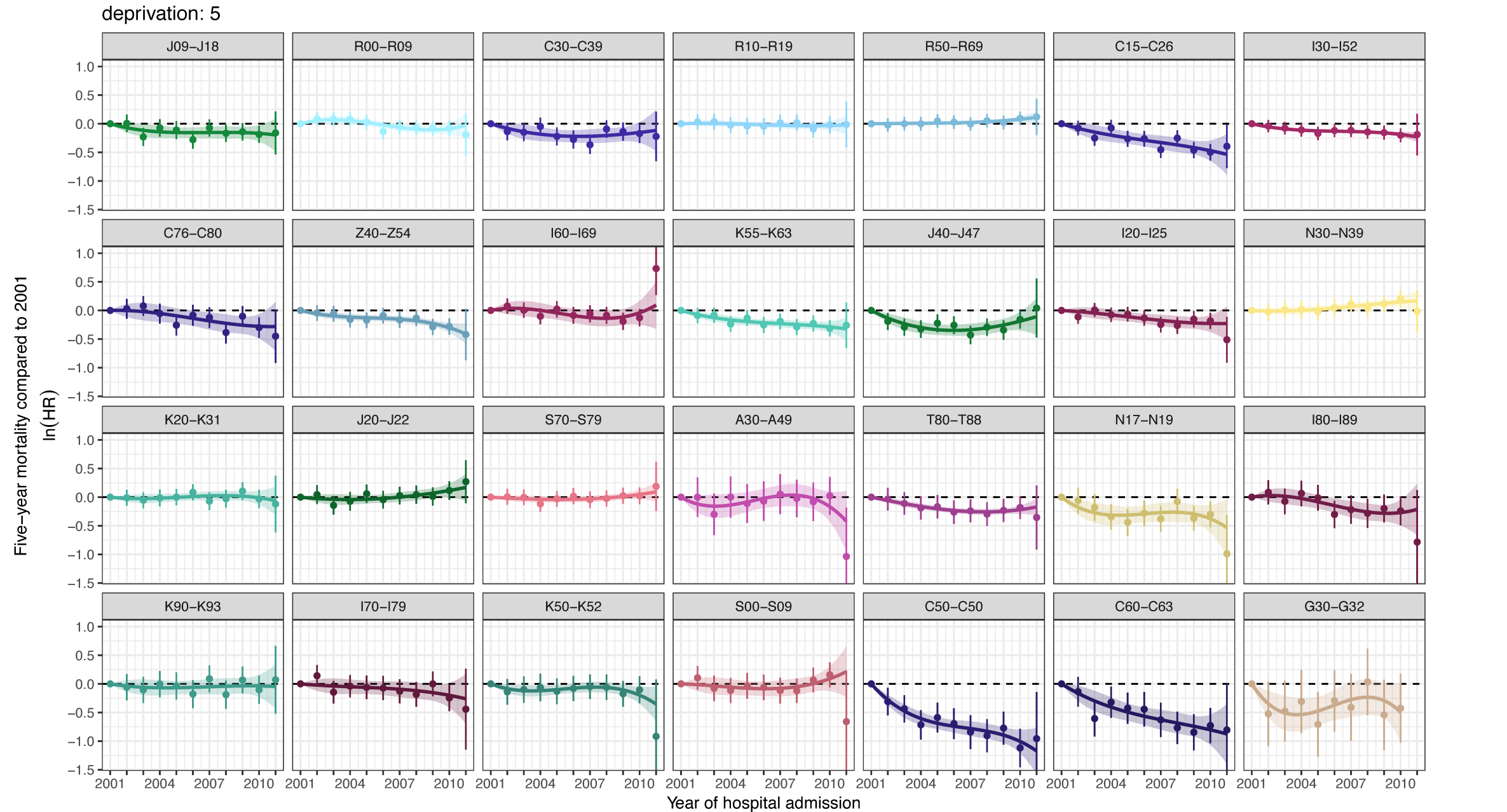

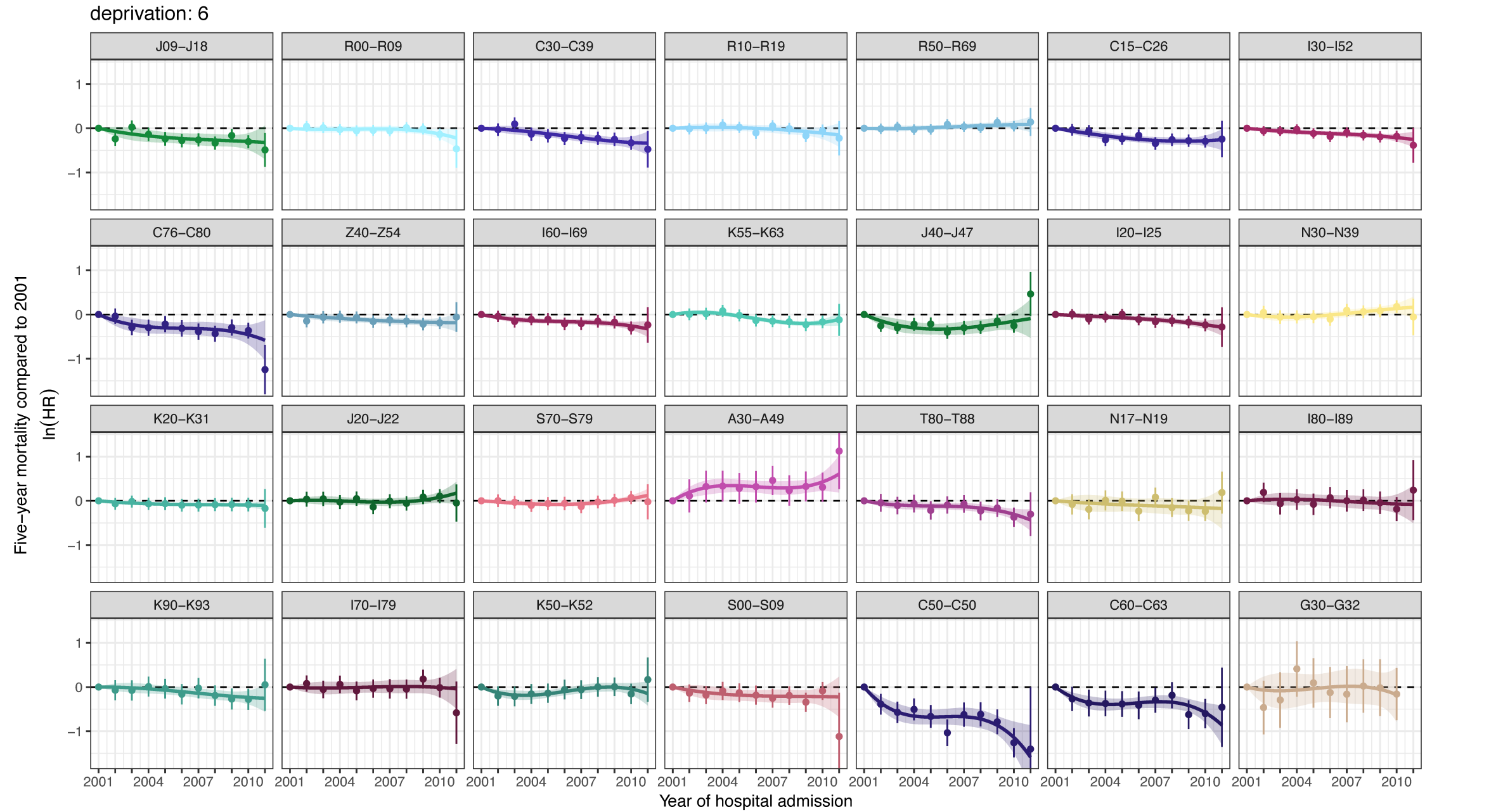

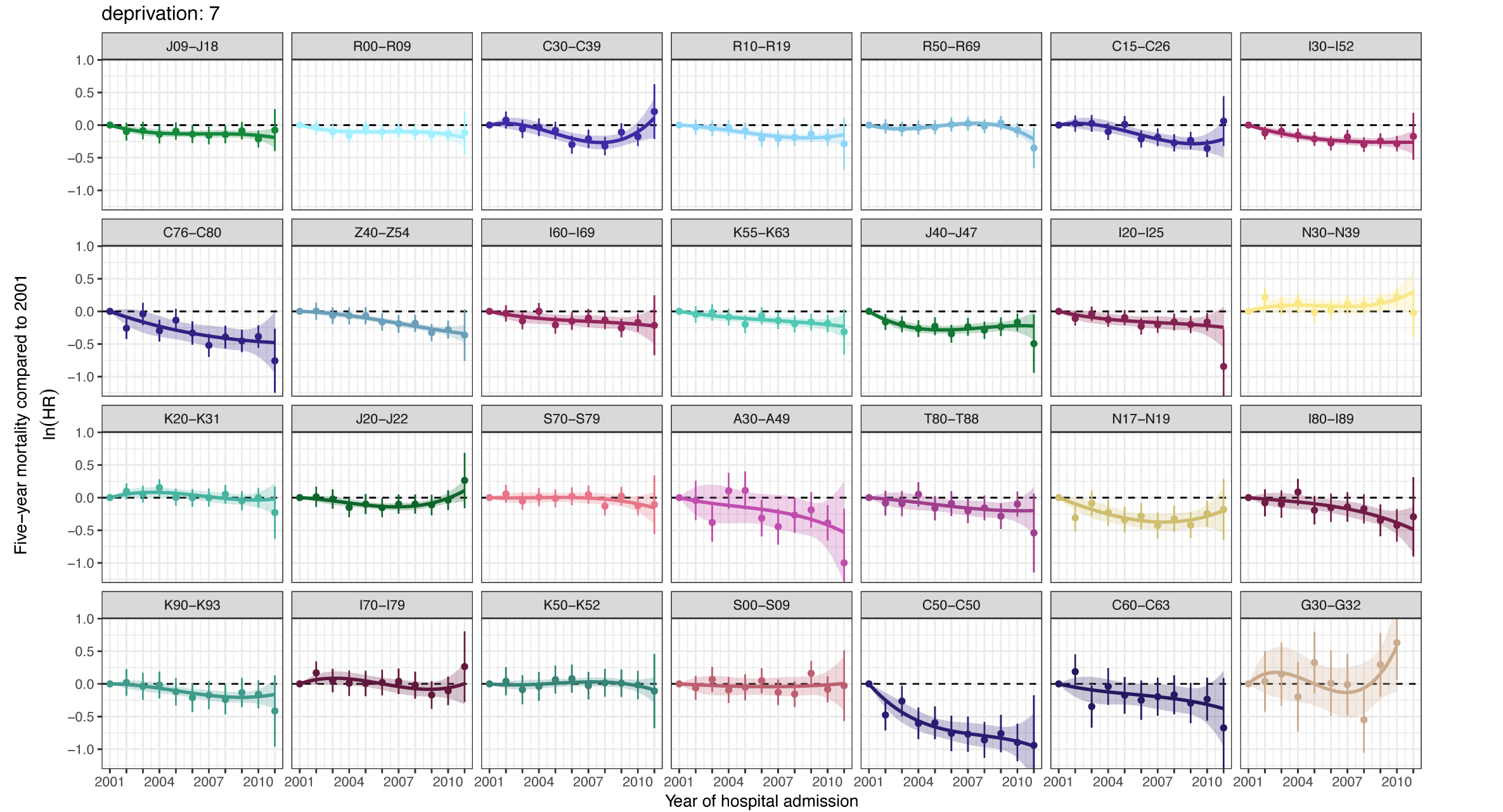

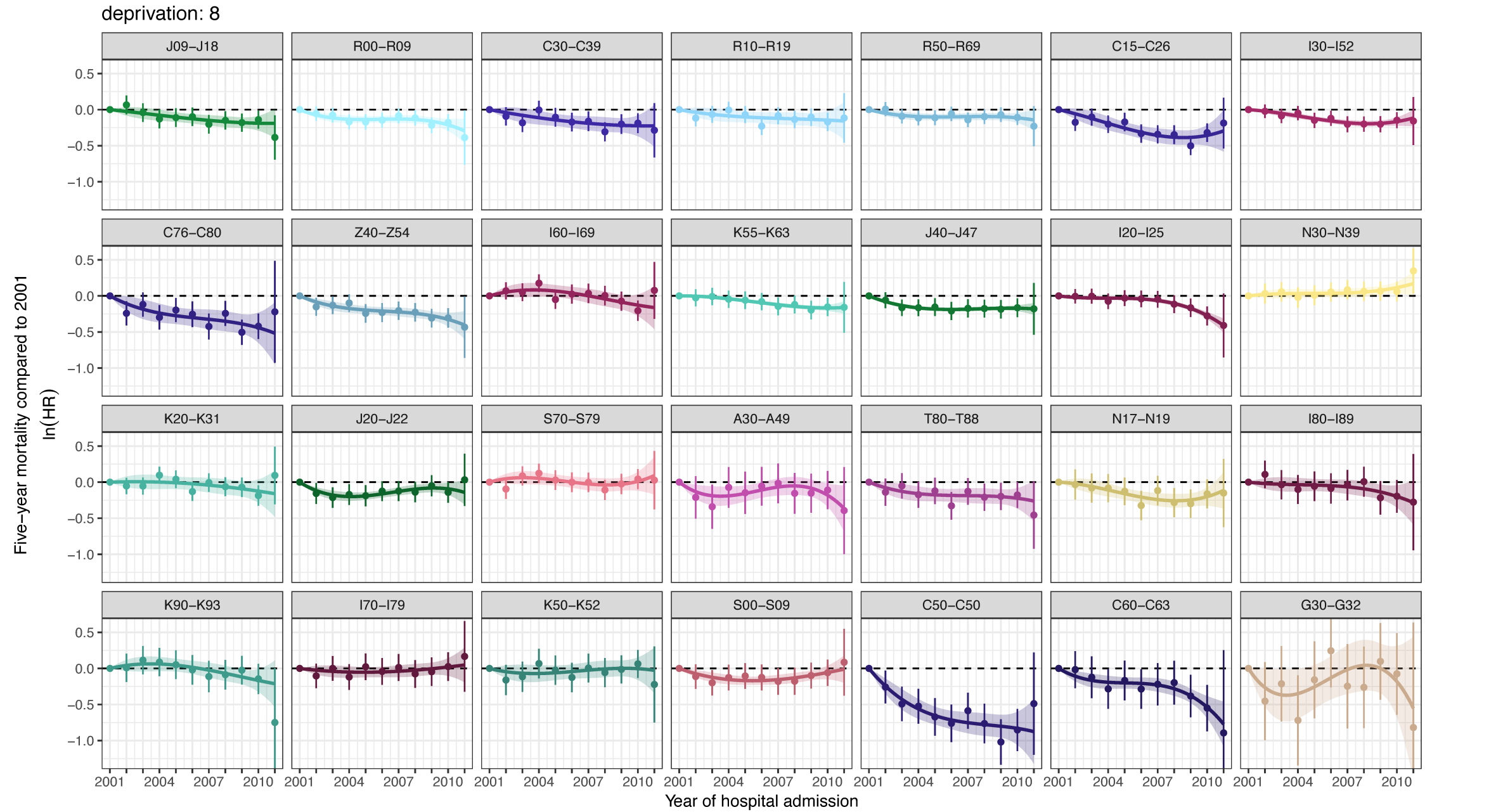

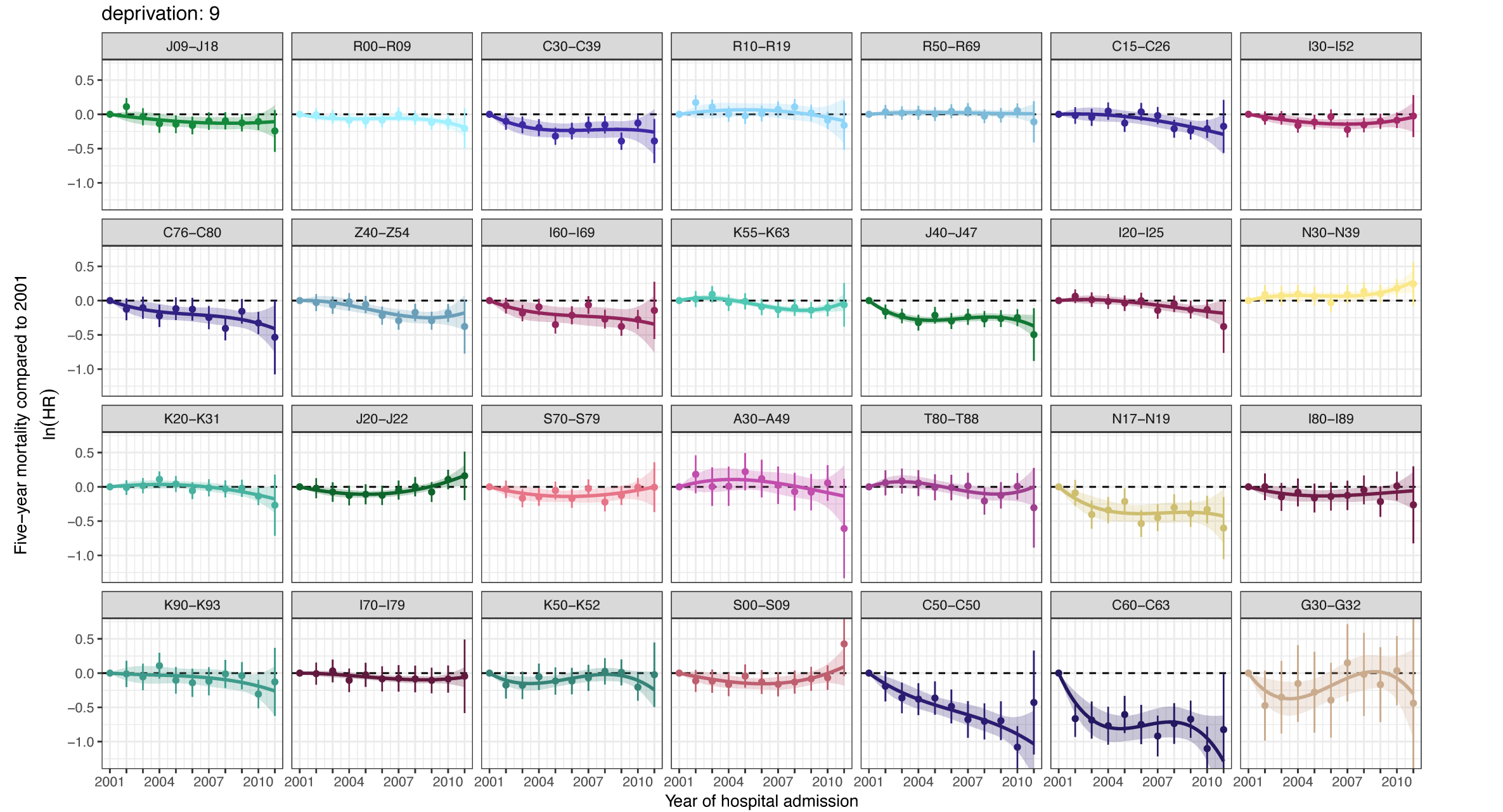

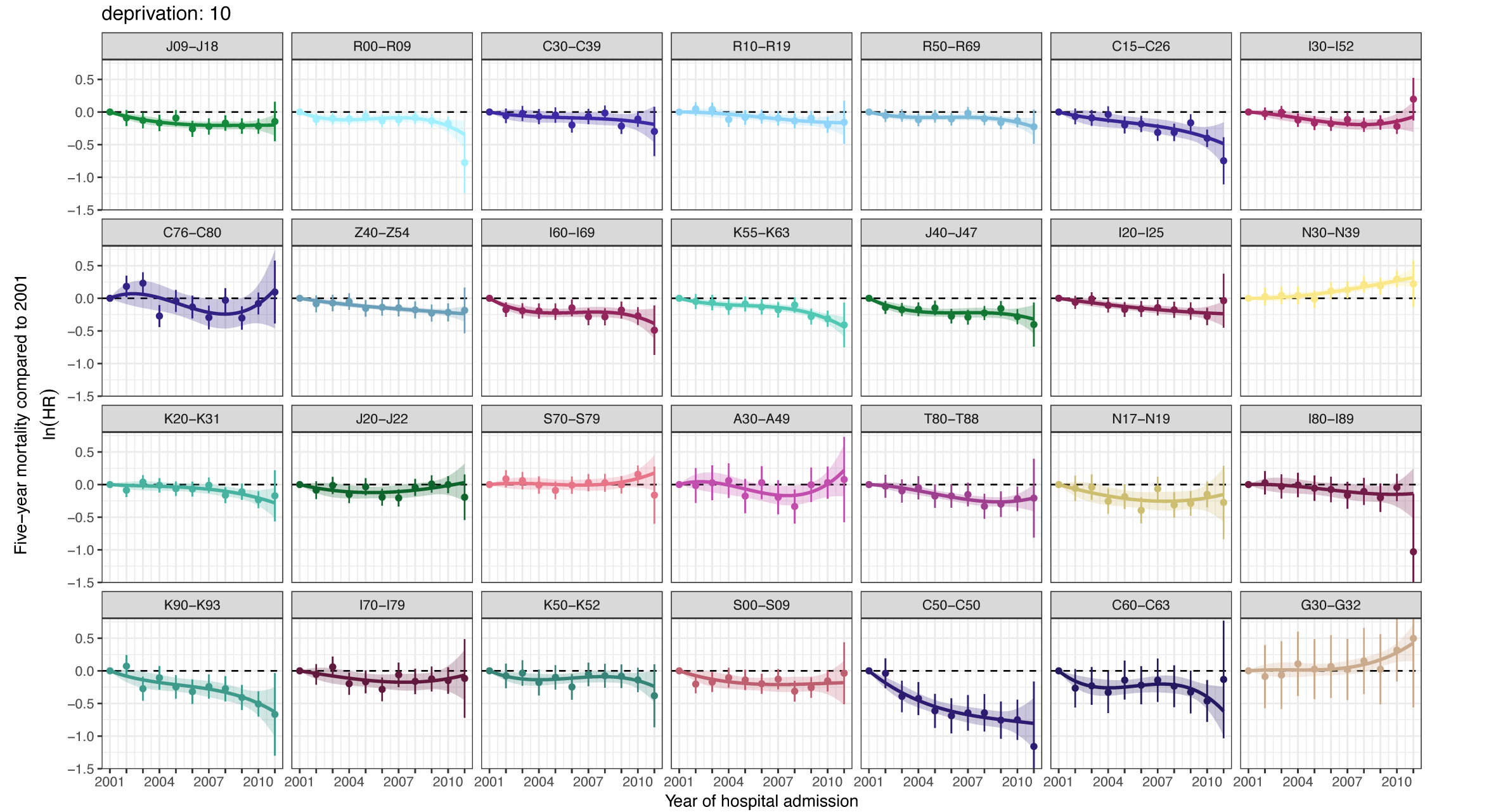

Supplement: Supplementary data [file bmjopen-2019-034299supp014.pdf]

Impact

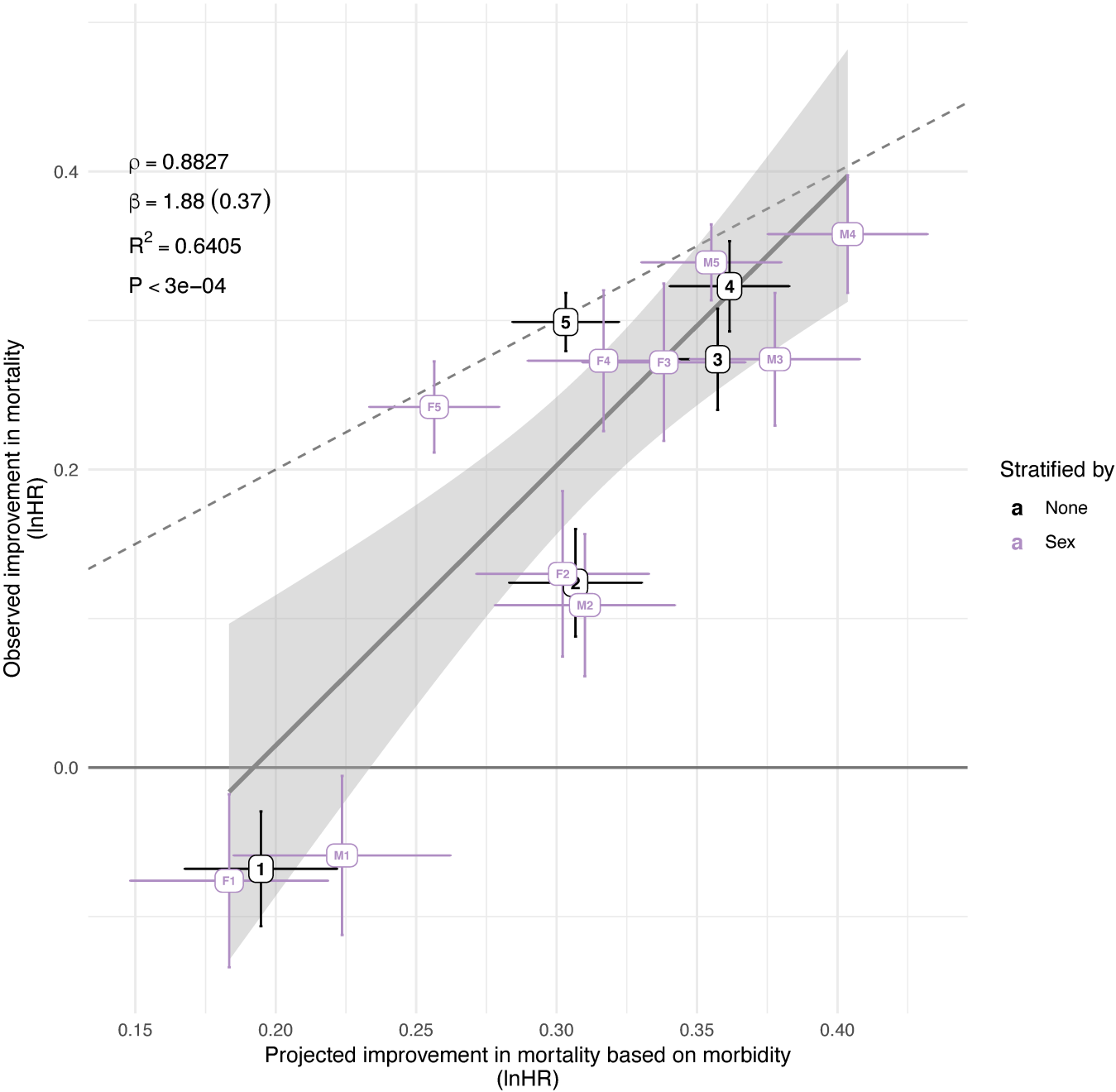

Supplement: Supplementary data [file bmjopen-2019-034299supp017.pdf]
