# Supplementary material for: Trends in disease incidence and survival and their effect on mortality in Scotland: nationwide cohort study of linked hospital admission and death records 2001–2016
Source: BMJ Open. 2020 Mar 25;10(3):e034299. doi: 10.1136/bmjopen-2019-034299 (PMC7170664; doi:10.1136/bmjopen-2019-034299)
Supplement: Supplementary data [file bmjopen-2019-034299supp007.pdf]

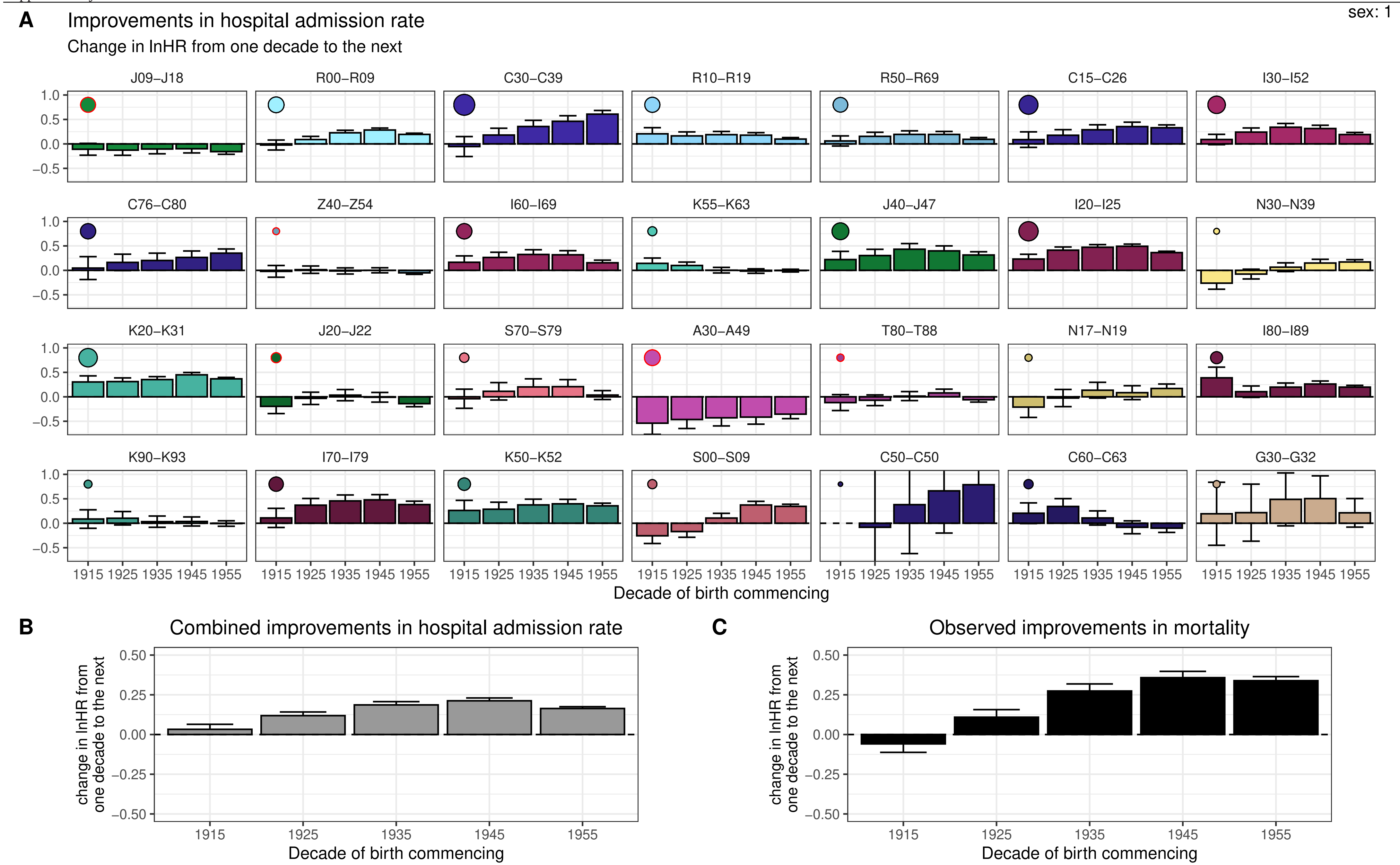
sex: 1
A
Improvements in hospital admission rate
Change in lnHR from one decade to the next
J09–J18
R00–R09
C30–C39
R10–R19
R50–R69
C15–C26
I30–I52
C76–C80
Z40–Z54
I60–I69
K55–K63
J40–J47
I20–I25
N30–N39
K20–K31
J20–J22
S70–S79
A30–A49
T80–T88
N17–N19
I80–I89
K90–K93
I70–I79
K50–K52
S00–S09
C50–C50
C60–C63
G30–G32
1.0
0.5
0.0
−0.5
1915 1925 1935 1945 1955
Decade of birth commencing
B
Combined improvements in hospital admission rate
change in lnHR from one decade to the next
0.50
0.25
0.00
−0.25
−0.50
1915 1925 1935 1945 1955
Decade of birth commencing
C
Observed improvements in mortality
change in lnHR from one decade to the next
0.50
0.25
0.00
−0.25
−0.50
1915 1925 1935 1945 1955
Decade of birth commencing

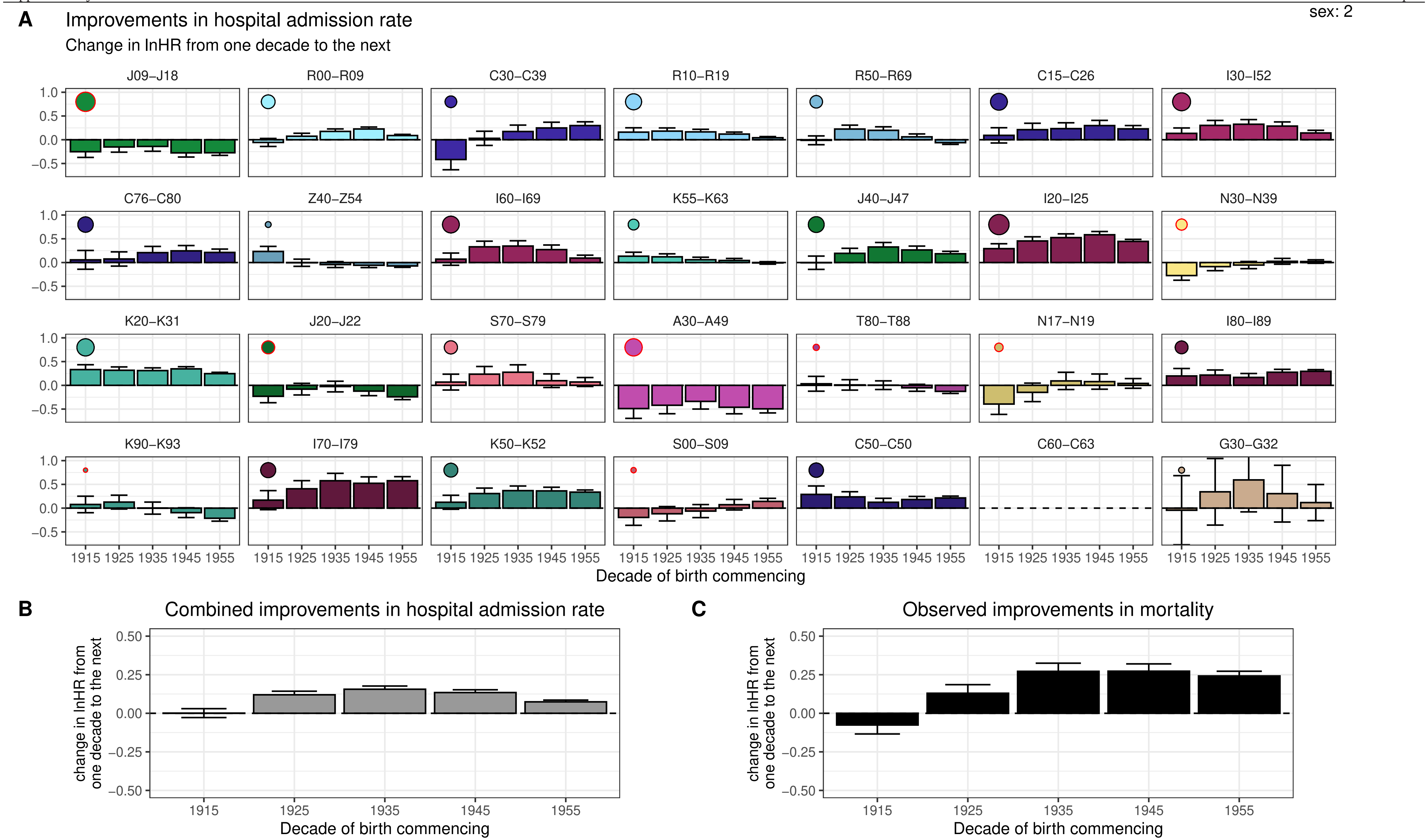
sex: 2
A
Improvements in hospital admission rate
Change in lnHR from one decade to the next
J09–J18
R00–R09
C30–C39
R10–R19
R50–R69
C15–C26
I30–I52
C76–C80
Z40–Z54
I60–I69
K55–K63
J40–J47
I20–I25
N30–N39
K20–K31
J20–J22
S70–S79
A30–A49
T80–T88
N17–N19
I80–I89
K90–K93
I70–I79
K50–K52
S00–S09
C50–C50
C60–C63
G30–G32
1.0
0.5
0.0
−0.5
1915 1925 1935 1945 1955
Decade of birth commencing
B
Combined improvements in hospital admission rate
change in lnHR from one decade to the next
0.50
0.25
0.00
−0.25
−0.50
1915
1925
1935
1945
1955
Decade of birth commencing
C
Observed improvements in mortality
change in lnHR from one decade to the next
Decade of birth commencing

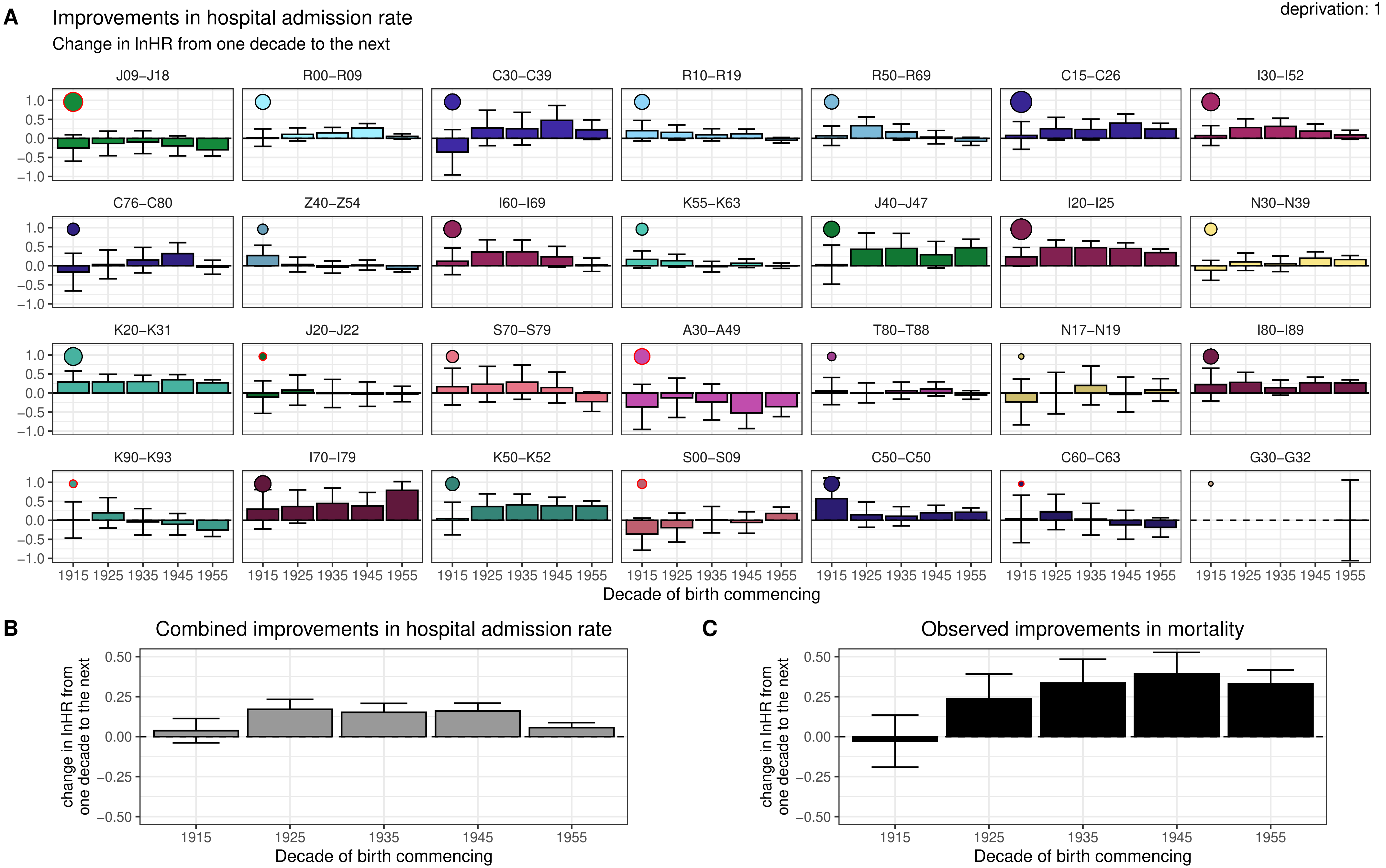

deprivation: 1
A
Improvements in hospital admission rate
Change in lnHR from one decade to the next
J09–J18
R00–R09
C30–C39
R10–R19
R50–R69
C15–C26
I30–I52
C76–C80
Z40–Z54
I60–I69
K55–K63
J40–J47
I20–I25
N30–N39
K20–K31
J20–J22
S70–S79
A30–A49
T80–T88
N17–N19
I80–I89
K90–K93
I70–I79
K50–K52
S00–S09
C50–C50
C60–C63
G30–G32
1.0
0.5
0.0
−0.5
−1.0
1915 1925 1935 1945 1955
Decade of birth commencing
B
Combined improvements in hospital admission rate
change in lnHR from one decade to the next
0.50
0.25
0.00
−0.25
−0.50
1915
1925
1935
1945
1955
Decade of birth commencing
C
Observed improvements in mortality
change in lnHR from one decade to the next
Decade of birth commencing

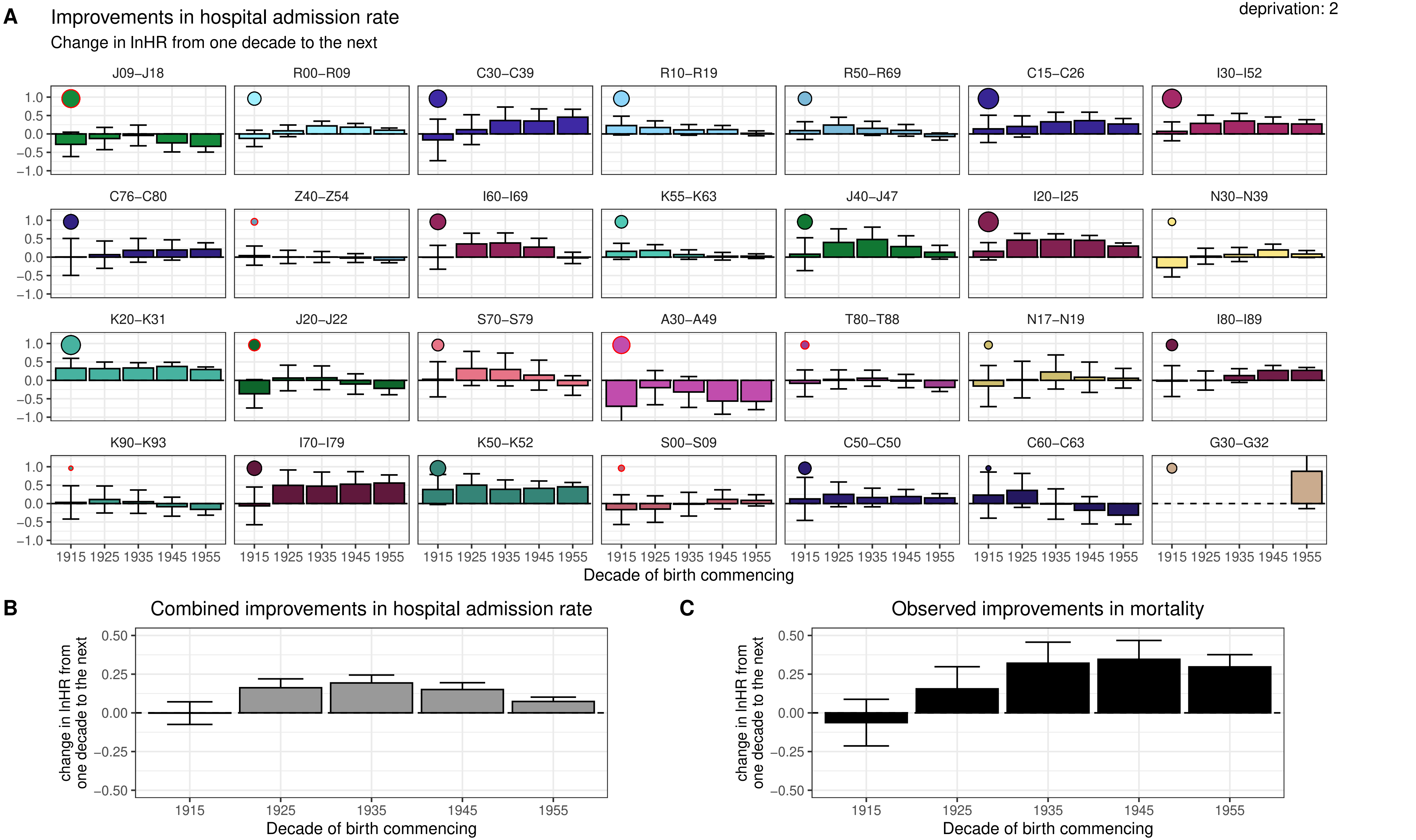

A
Improvements in hospital admission rate
Change in lnHR from one decade to the next
deprivation: 2
J09–J18
R00–R09
C30–C39
R10–R19
R50–R69
C15–C26
I30–I52
C76–C80
Z40–Z54
I60–I69
K55–K63
J40–J47
I20–I25
N30–N39
K20–K31
J20–J22
S70–S79
A30–A49
T80–T88
N17–N19
I80–I89
K90–K93
I70–I79
K50–K52
S00–S09
C50–C50
C60–C63
G30–G32
1.0
0.5
0.0
−0.5
−1.0
1915 1925 1935 1945 1955
Decade of birth commencing
B
Combined improvements in hospital admission rate
change in lnHR from one decade to the next
0.50
0.25
0.00
−0.25
−0.50
1915 1925 1935 1945 1955
Decade of birth commencing
C
Observed improvements in mortality
change in lnHR from one decade to the next
0.50
0.25
0.00
−0.25
−0.50
1915 1925 1935 1945 1955
Decade of birth commencing

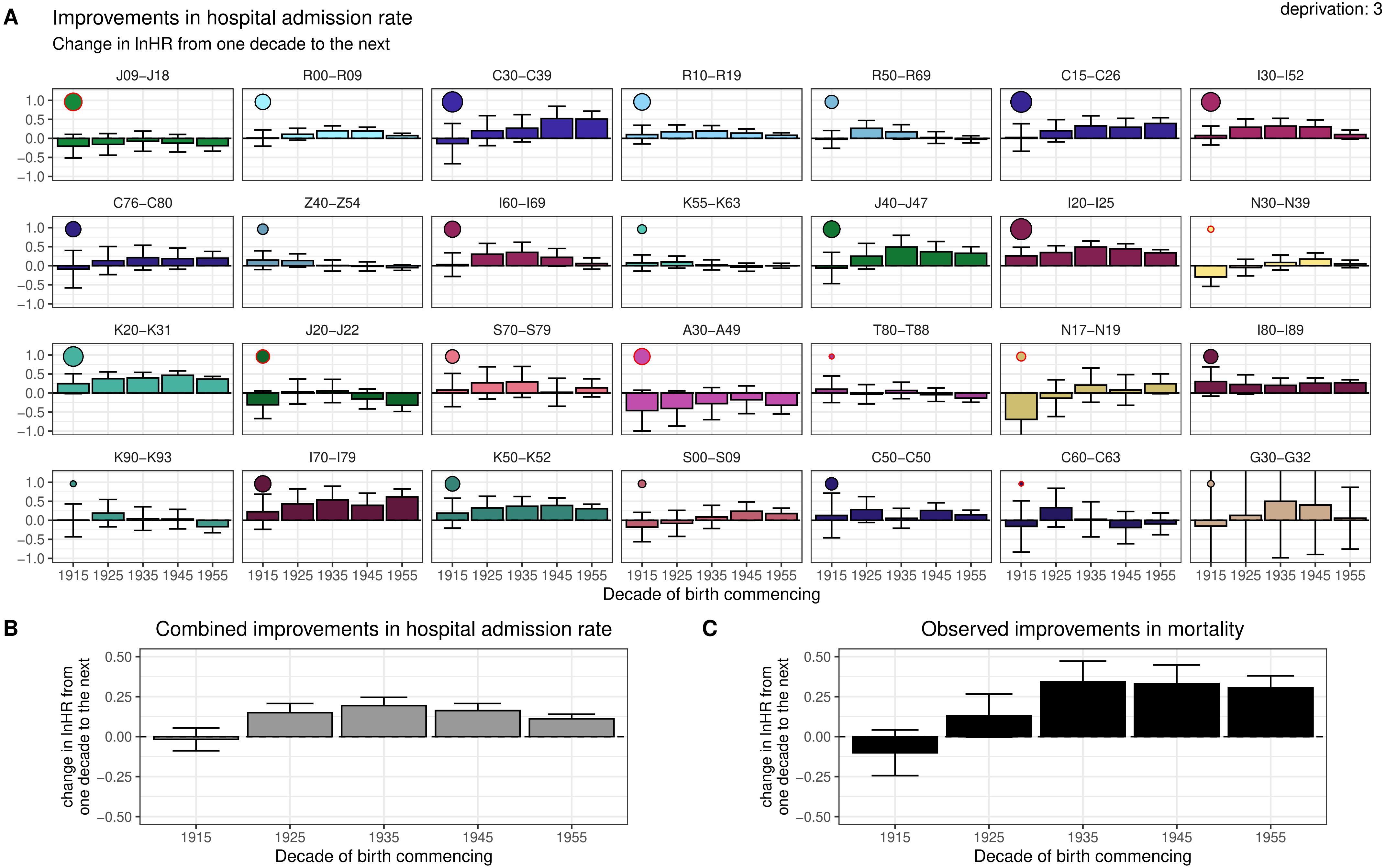
A
Improvements in hospital admission rate
Change in lnHR from one decade to the next
deprivation: 3
J09–J18
R00–R09
C30–C39
R10–R19
R50–R69
C15–C26
I30–I52
C76–C80
Z40–Z54
I60–I69
K55–K63
J40–J47
I20–I25
N30–N39
K20–K31
J20–J22
S70–S79
A30–A49
T80–T88
N17–N19
I80–I89
K90–K93
I70–I79
K50–K52
S00–S09
C50–C50
C60–C63
G30–G32
1.0
0.5
0.0
−0.5
−1.0
1915 1925 1935 1945 1955
Decade of birth commencing
B
Combined improvements in hospital admission rate
change in lnHR from one decade to the next
0.50
0.25
0.00
−0.25
−0.50
1915
1925
1935
1945
1955
Decade of birth commencing
C
Observed improvements in mortality
change in lnHR from one decade to the next
0.50
0.25
0.00
−0.25
−0.50
1915
1925
1935
1945
1955
Decade of birth commencing

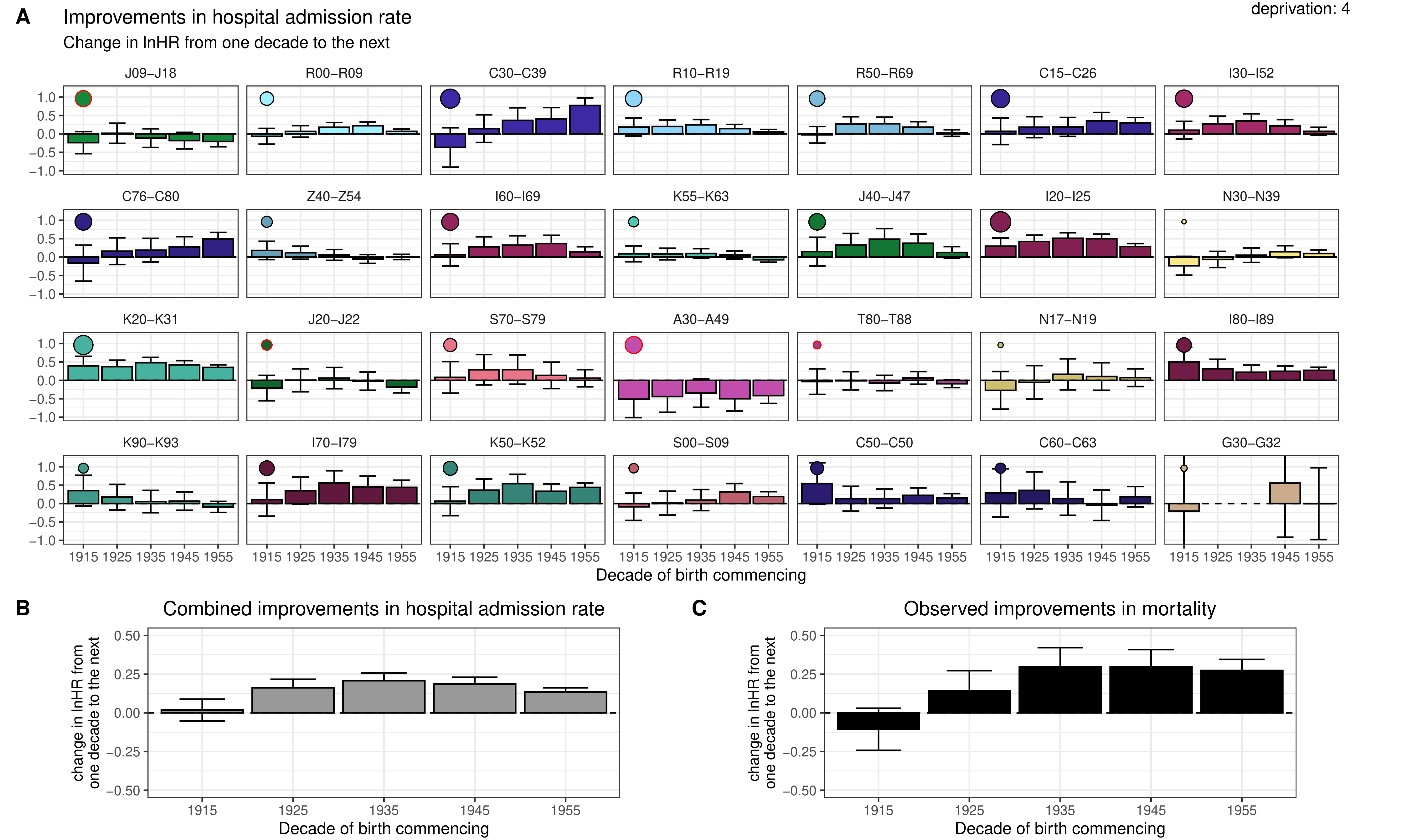
deprivation: 4
A
Improvements in hospital admission rate
Change in lnHR from one decade to the next
J09–J18
R00–R09
C30–C39
R10–R19
R50–R69
C15–C26
I30–I52
C76–C80
Z40–Z54
I60–I69
K55–K63
J40–J47
I20–I25
N30–N39
K20–K31
J20–J22
S70–S79
A30–A49
T80–T88
N17–N19
I80–I89
K90–K93
I70–I79
K50–K52
S00–S09
C50–C50
C60–C63
G30–G32
1.0
0.5
0.0
−0.5
−1.0
1915 1925 1935 1945 1955
Decade of birth commencing
B
Combined improvements in hospital admission rate
change in lnHR from one decade to the next
0.50
0.25
0.00
−0.25
−0.50
1915
1925
1935
1945
1955
Decade of birth commencing
C
Observed improvements in mortality
change in lnHR from one decade to the next
0.50
0.25
0.00
−0.25
−0.50
1915
1925
1935
1945
1955
Decade of birth commencing

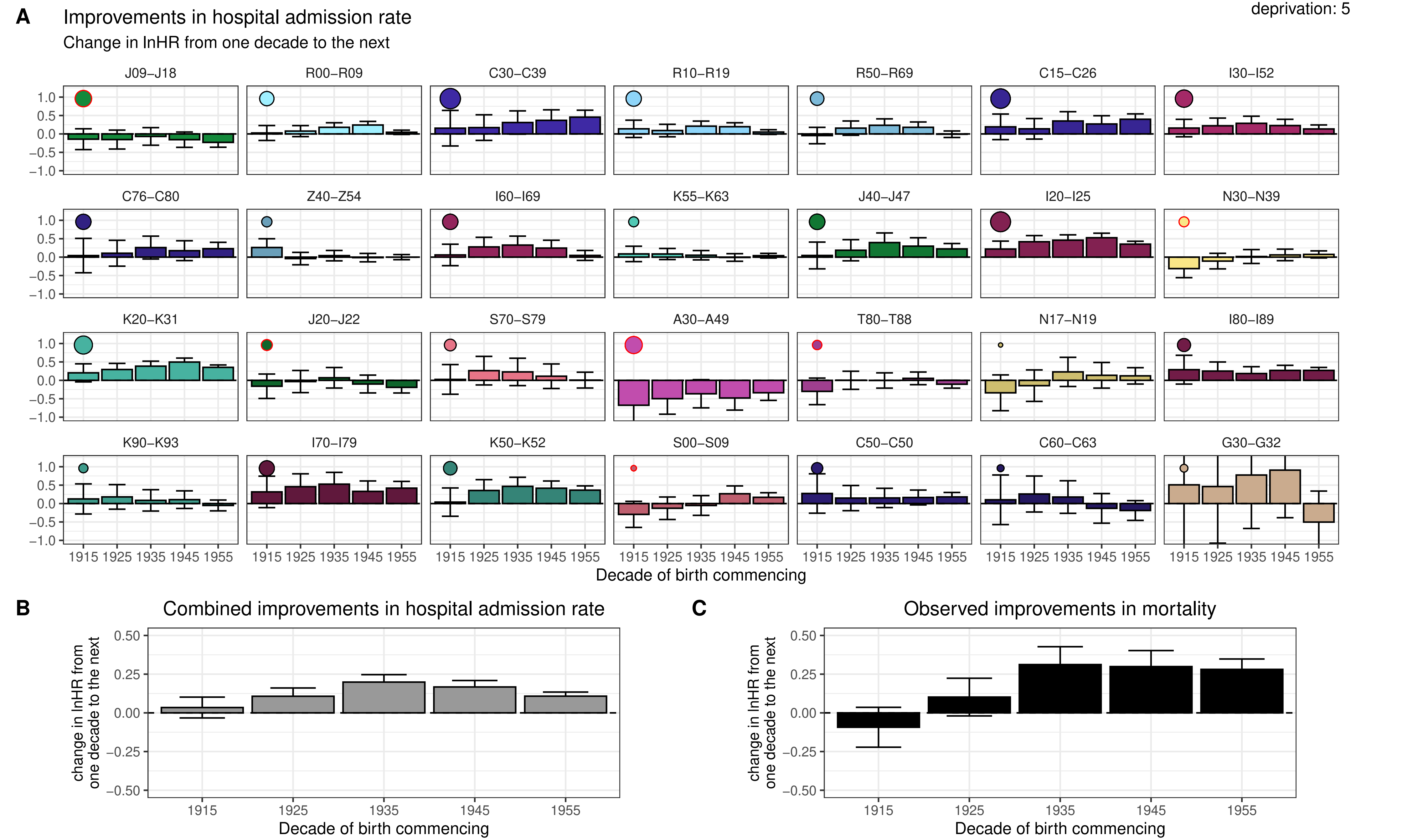

A
Improvements in hospital admission rate
Change in lnHR from one decade to the next
deprivation: 5
J09–J18
R00–R09
C30–C39
R10–R19
R50–R69
C15–C26
I30–I52
C76–C80
Z40–Z54
I60–I69
K55–K63
J40–J47
I20–I25
N30–N39
K20–K31
J20–J22
S70–S79
A30–A49
T80–T88
N17–N19
I80–I89
K90–K93
I70–I79
K50–K52
S00–S09
C50–C50
C60–C63
G30–G32
1.0
0.5
0.0
−0.5
−1.0
1915 1925 1935 1945 1955
Decade of birth commencing
B
Combined improvements in hospital admission rate
change in lnHR from one decade to the next
0.50
0.25
0.00
−0.25
−0.50
1915 1925 1935 1945 1955
Decade of birth commencing
C
Observed improvements in mortality
change in lnHR from one decade to the next
0.50
0.25
0.00
−0.25
−0.50
1915 1925 1935 1945 1955
Decade of birth commencing

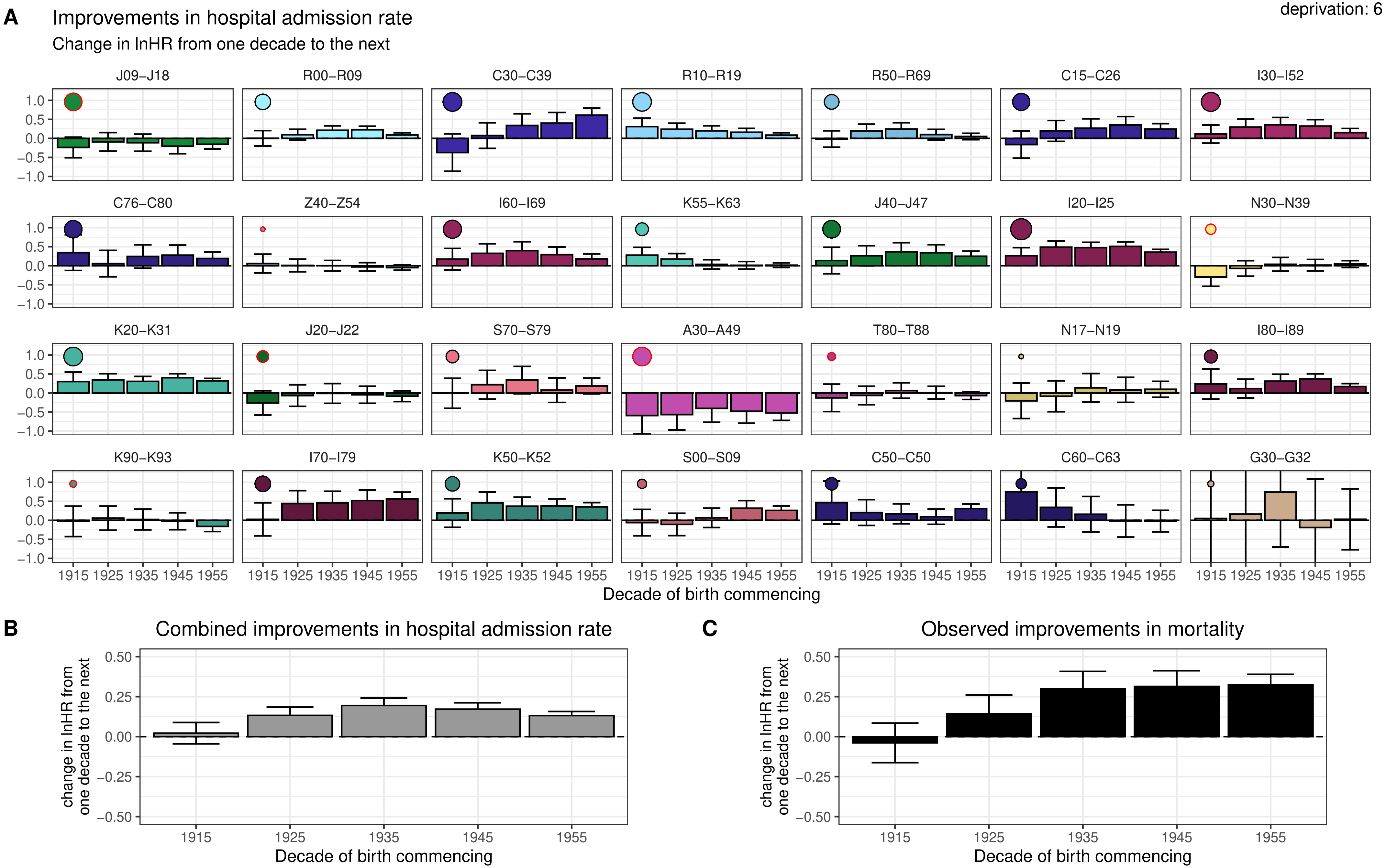

deprivation: 6
A
Improvements in hospital admission rate
Change in lnHR from one decade to the next
J09–J18
R00–R09
C30–C39
R10–R19
R50–R69
C15–C26
I30–I52
C76–C80
Z40–Z54
I60–I69
K55–K63
J40–J47
I20–I25
N30–N39
K20–K31
J20–J22
S70–S79
A30–A49
T80–T88
N17–N19
I80–I89
K90–K93
I70–I79
K50–K52
S00–S09
C50–C50
C60–C63
G30–G32
1.0
0.5
0.0
−0.5
−1.0
1915 1925 1935 1945 1955
Decade of birth commencing
B
Combined improvements in hospital admission rate
change in lnHR from one decade to the next
0.50
0.25
0.00
−0.25
−0.50
1915 1925 1935 1945 1955
Decade of birth commencing
C
Observed improvements in mortality
change in lnHR from one decade to the next
0.50
0.25
0.00
−0.25
−0.50
1915 1925 1935 1945 1955
Decade of birth commencing

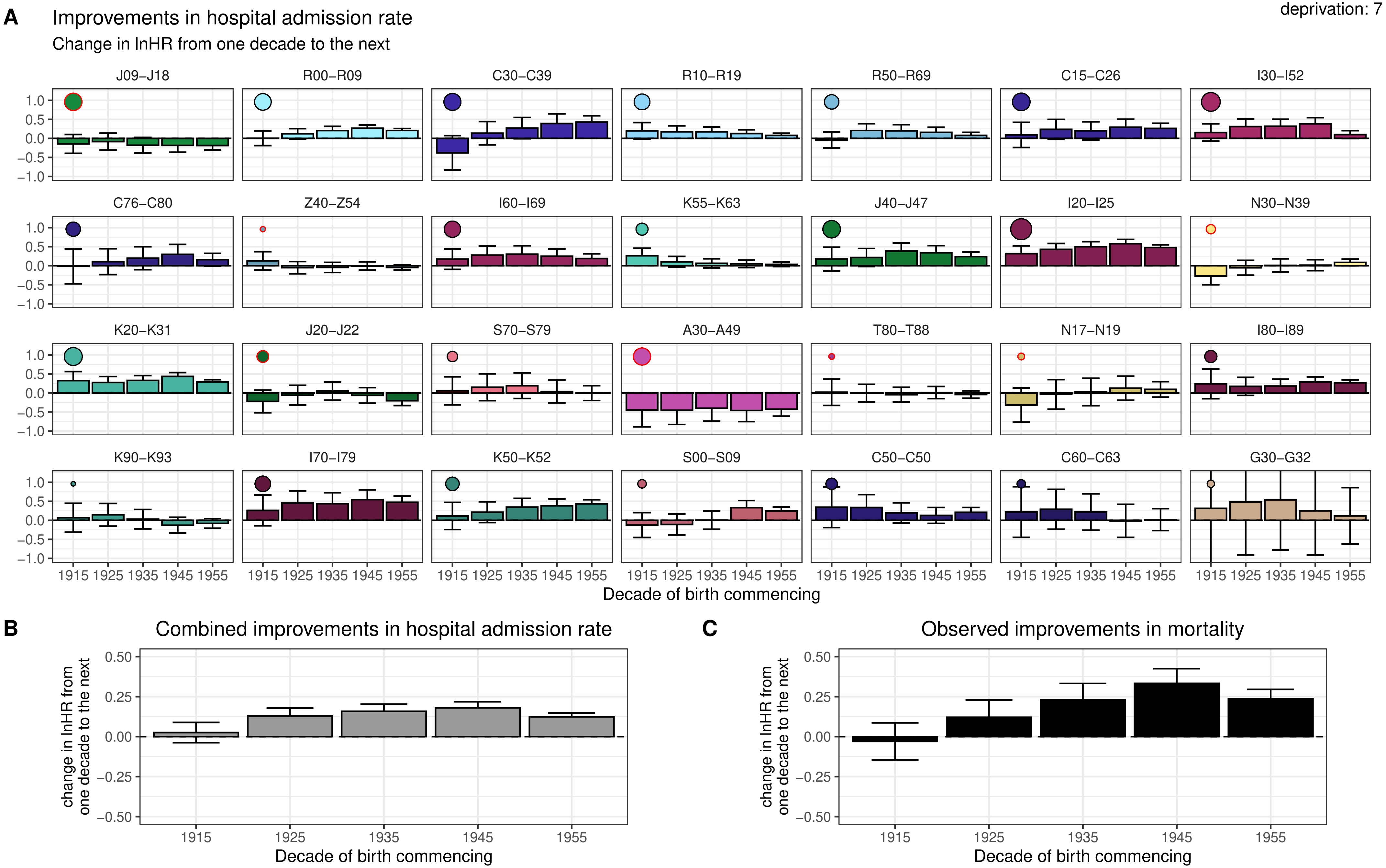
A
Improvements in hospital admission rate
Change in lnHR from one decade to the next
deprivation: 7
J09–J18
R00–R09
C30–C39
R10–R19
R50–R69
C15–C26
I30–I52
C76–C80
Z40–Z54
I60–I69
K55–K63
J40–J47
I20–I25
N30–N39
K20–K31
J20–J22
S70–S79
A30–A49
T80–T88
N17–N19
I80–I89
K90–K93
I70–I79
K50–K52
S00–S09
C50–C50
C60–C63
G30–G32
1.0
0.5
0.0
−0.5
−1.0
1915 1925 1935 1945 1955
Decade of birth commencing
B
Combined improvements in hospital admission rate
change in lnHR from one decade to the next
0.50
0.25
0.00
−0.25
−0.50
1915
1925
1935
1945
1955
Decade of birth commencing
C
Observed improvements in mortality
change in lnHR from one decade to the next
0.50
0.25
0.00
−0.25
−0.50
1915
1925
1935
1945
1955
Decade of birth commencing

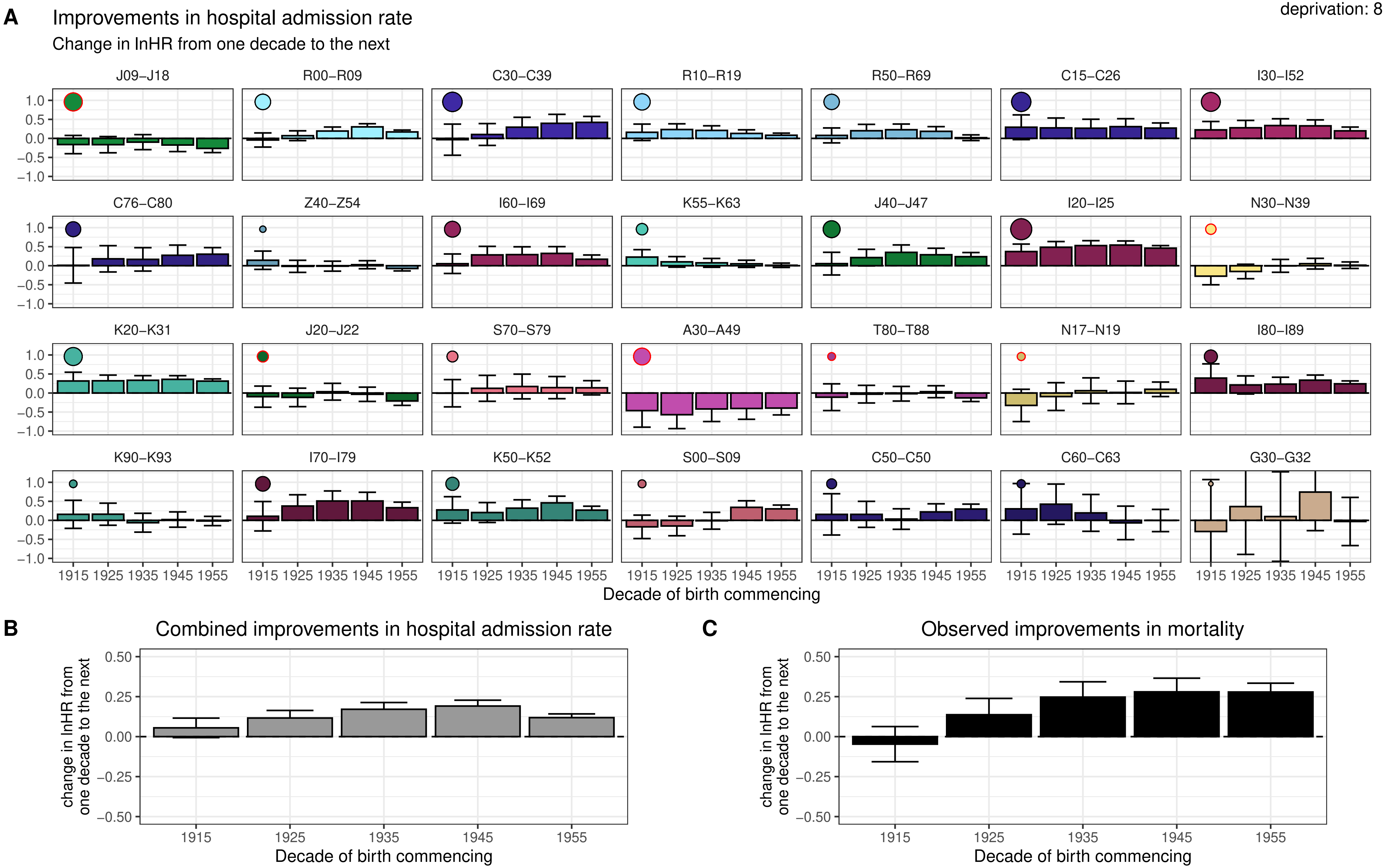
A
Improvements in hospital admission rate
Change in lnHR from one decade to the next
deprivation: 8
J09–J18
R00–R09
C30–C39
R10–R19
R50–R69
C15–C26
I30–I52
C76–C80
Z40–Z54
I60–I69
K55–K63
J40–J47
I20–I25
N30–N39
K20–K31
J20–J22
S70–S79
A30–A49
T80–T88
N17–N19
I80–I89
K90–K93
I70–I79
K50–K52
S00–S09
C50–C50
C60–C63
G30–G32
1915 1925 1935 1945 1955
Decade of birth commencing
B
Combined improvements in hospital admission rate
change in lnHR from one decade to the next
Decade of birth commencing
C
Observed improvements in mortality
change in lnHR from one decade to the next
Decade of birth commencing

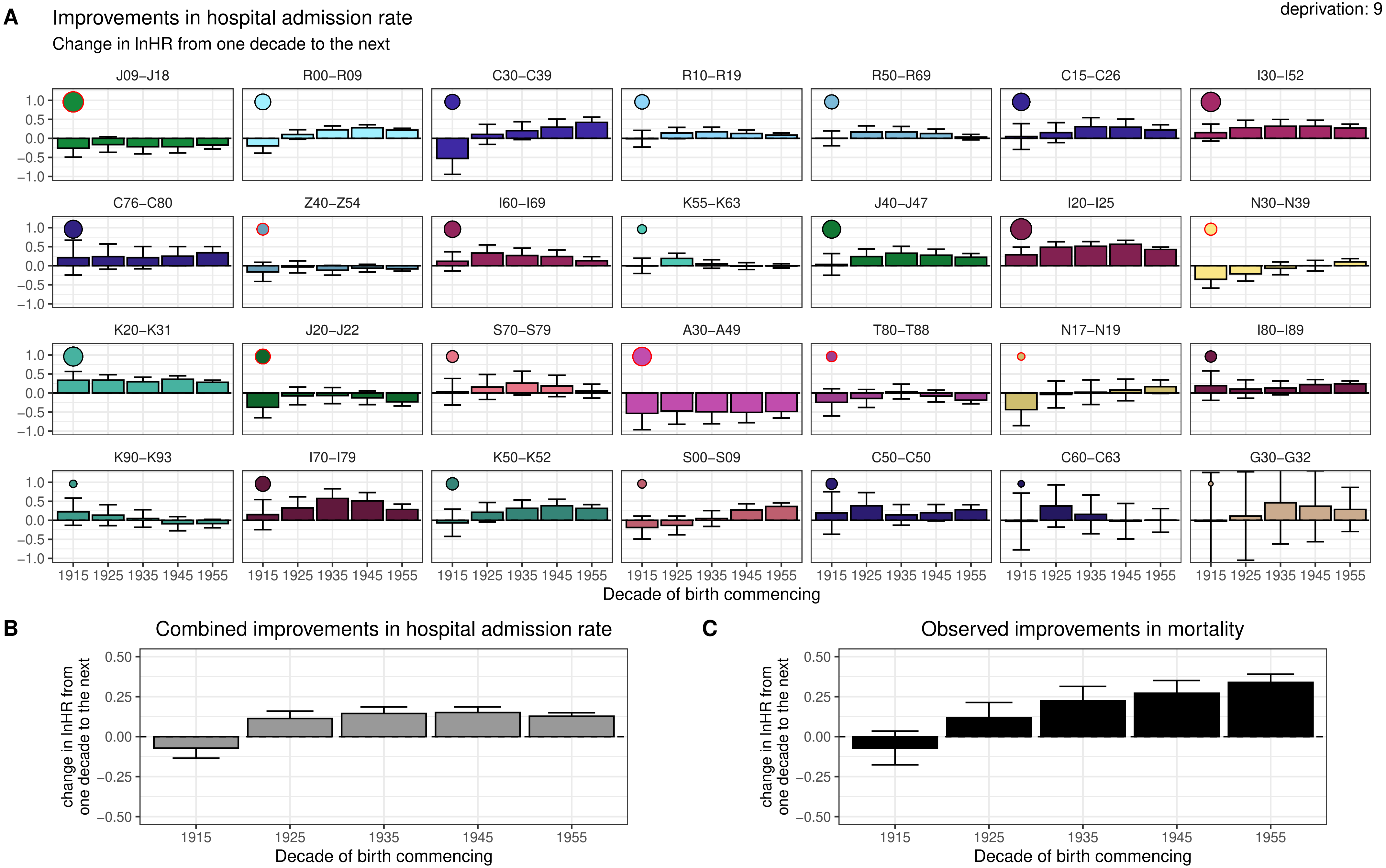
A
Improvements in hospital admission rate
Change in lnHR from one decade to the next
deprivation: 9
J09–J18
R00–R09
C30–C39
R10–R19
R50–R69
C15–C26
I30–I52
C76–C80
Z40–Z54
I60–I69
K55–K63
J40–J47
I20–I25
N30–N39
K20–K31
J20–J22
S70–S79
A30–A49
T80–T88
N17–N19
I80–I89
K90–K93
I70–I79
K50–K52
S00–S09
C50–C50
C60–C63
G30–G32
1.0
0.5
0.0
−0.5
−1.0
1915 1925 1935 1945 1955
Decade of birth commencing
B
Combined improvements in hospital admission rate
C
Observed improvements in mortality
change in lnHR from one decade to the next
0.50
0.25
0.00
−0.25
−0.50
1915
1925
1935
1945
1955
Decade of birth commencing

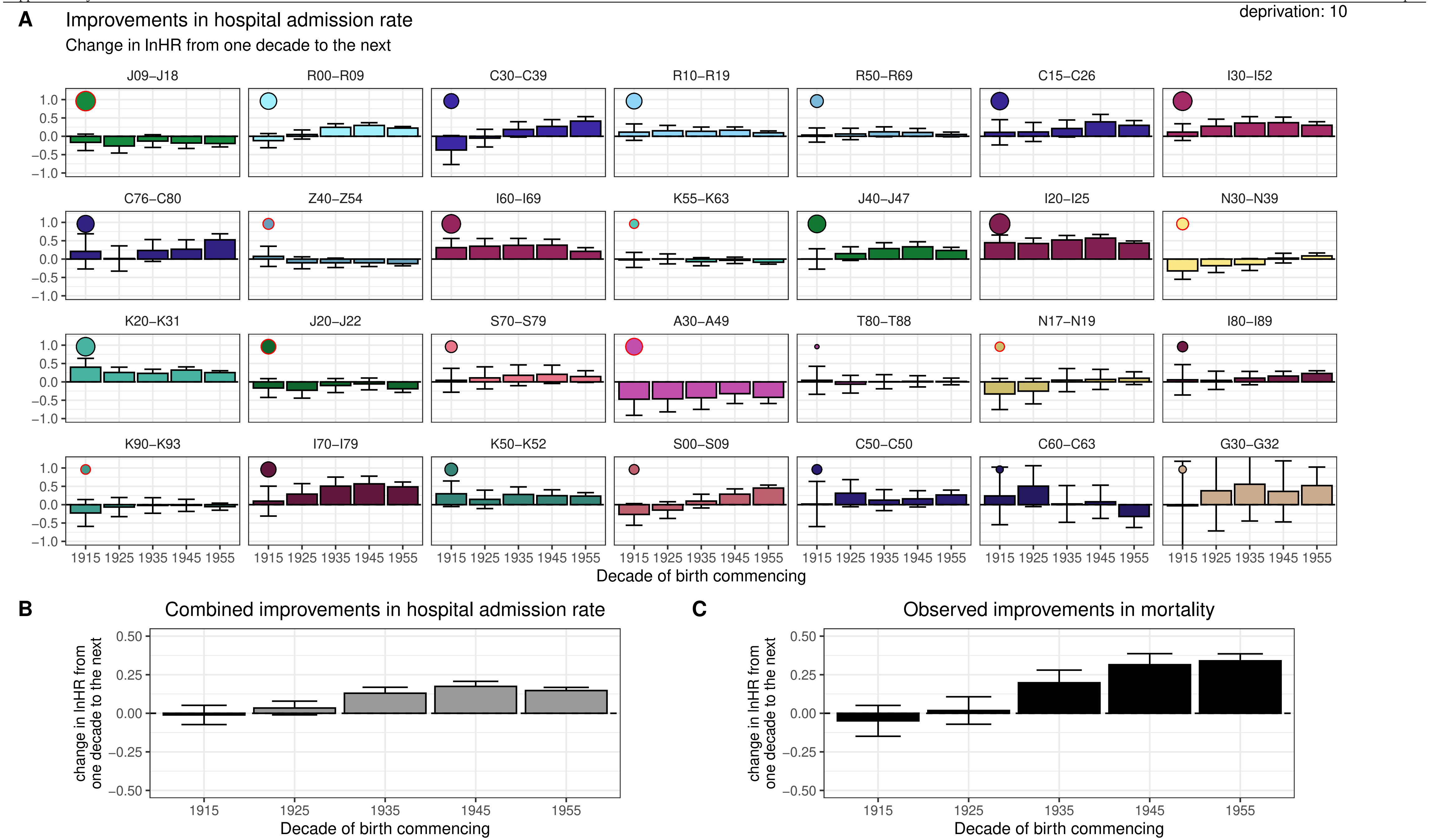
deprivation: 10
A
Improvements in hospital admission rate
Change in lnHR from one decade to the next
J09–J18
R00–R09
C30–C39
R10–R19
R50–R69
C15–C26
I30–I52
C76–C80
Z40–Z54
I60–I69
K55–K63
J40–J47
I20–I25
N30–N39
K20–K31
J20–J22
S70–S79
A30–A49
T80–T88
N17–N19
I80–I89
K90–K93
I70–I79
K50–K52
S00–S09
C50–C50
C60–C63
G30–G32
1.0
0.5
0.0
−0.5
−1.0
1915 1925 1935 1945 1955
Decade of birth commencing
B
Combined improvements in hospital admission rate
change in lnHR from one decade to the next
0.50
0.25
0.00
−0.25
−0.50
1915
1925
1935
1945
1955
Decade of birth commencing
C
Observed improvements in mortality
change in lnHR from one decade to the next
Decade of birth commencing
