# Supplementary material for: Trends in disease incidence and survival and their effect on mortality in Scotland: nationwide cohort study of linked hospital admission and death records 2001–2016
Source: BMJ Open. 2020 Mar 25;10(3):e034299. doi: 10.1136/bmjopen-2019-034299 (PMC7170664; doi:10.1136/bmjopen-2019-034299)
Supplement: Supplementary data [file bmjopen-2019-034299supp016.pdf]

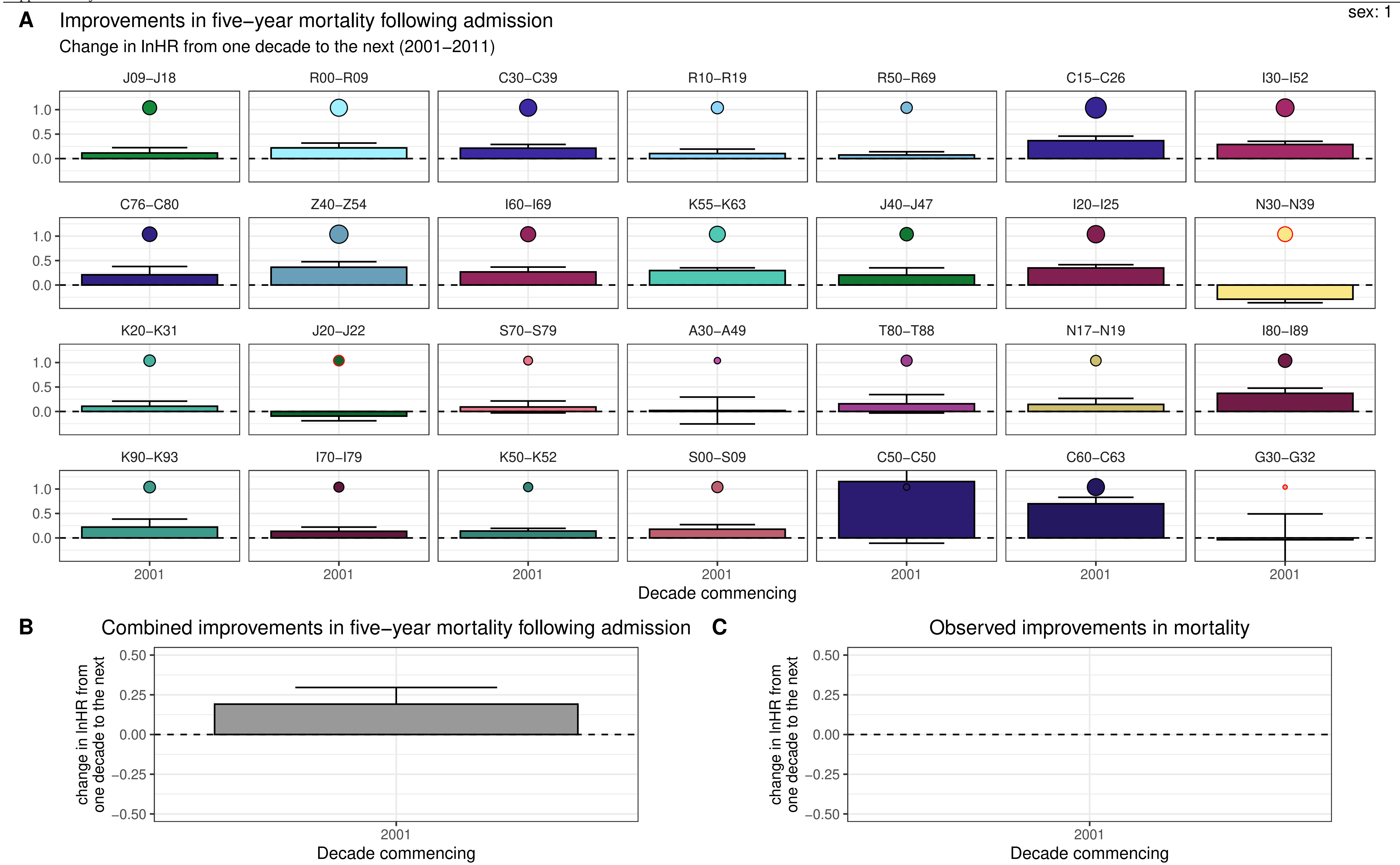
sex: 1
A
Improvements in five−year mortality following admission
Change in lnHR from one decade to the next (2001−2011)
J09−J18
R00−R09
C30−C39
R10−R19
R50−R69
C15−C26
I30−I52
C76−C80
Z40−Z54
I60−I69
K55−K63
J40−J47
I20−I25
N30−N39
K20−K31
J20−J22
S70−S79
A30−A49
T80−T88
N17−N19
I80−I89
K90−K93
I70−I79
K50−K52
S00−S09
C50−C50
C60−C63
G30−G32
1.0
0.5
0.0
2001
Decade commencing
B
Combined improvements in five−year mortality following admission
change in lnHR from one decade to the next
0.50
0.25
0.00
−0.25
−0.50
2001
Decade commencing
C
Observed improvements in mortality
change in lnHR from one decade to the next
0.50
0.25
0.00
−0.25
−0.50
2001
Decade commencing

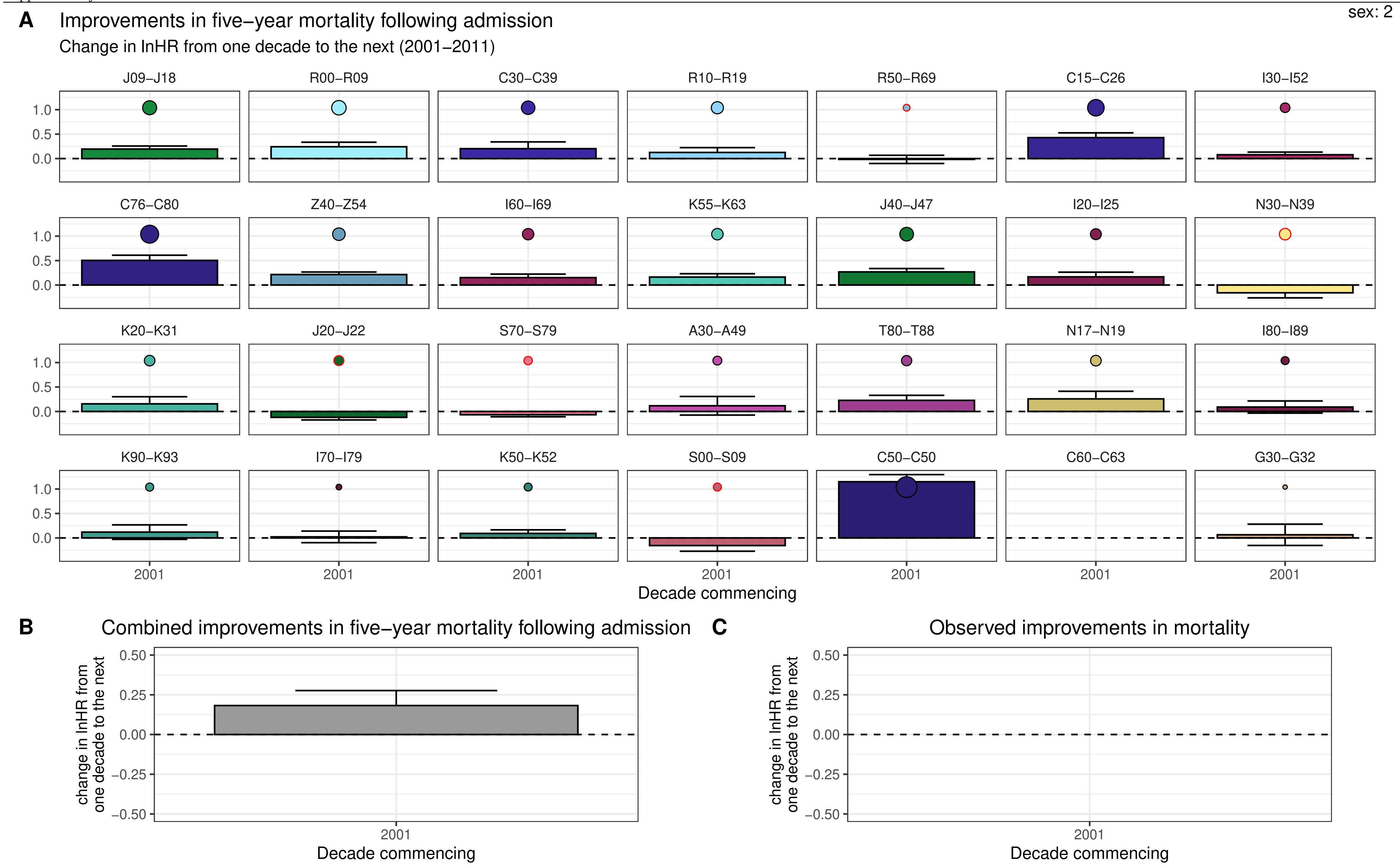
sex: 2
A
Improvements in five−year mortality following admission
Change in lnHR from one decade to the next (2001−2011)
J09−J18
R00−R09
C30−C39
R10−R19
R50−R69
C15−C26
I30−I52
C76−C80
Z40−Z54
I60−I69
K55−K63
J40−J47
I20−I25
N30−N39
K20−K31
J20−J22
S70−S79
A30−A49
T80−T88
N17−N19
I80−I89
K90−K93
I70−I79
K50−K52
S00−S09
C50−C50
C60−C63
G30−G32
1.0
0.5
0.0
2001
Decade commencing
B
Combined improvements in five−year mortality following admission
change in lnHR from one decade to the next
0.50
0.25
0.00
−0.25
−0.50
2001
Decade commencing
C
Observed improvements in mortality
change in lnHR from one decade to the next
0.50
0.25
0.00
−0.25
−0.50
2001
Decade commencing

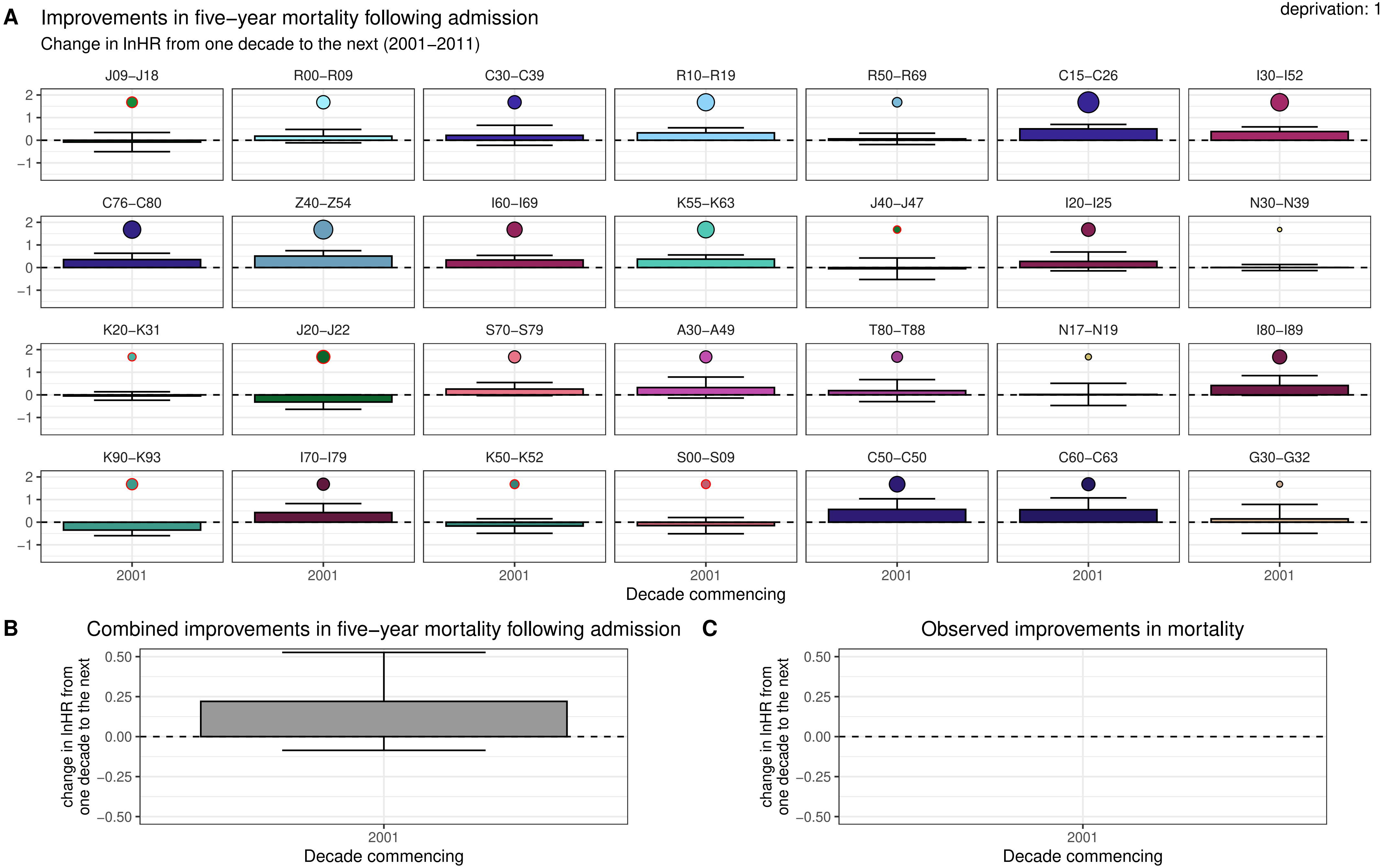
deprivation: 1
A
Improvements in five−year mortality following admission
Change in lnHR from one decade to the next (2001−2011)
J09−J18
R00−R09
C30−C39
R10−R19
R50−R69
C15−C26
I30−I52
C76−C80
Z40−Z54
I60−I69
K55−K63
J40−J47
I20−I25
N30−N39
K20−K31
J20−J22
S70−S79
A30−A49
T80−T88
N17−N19
I80−I89
K90−K93
I70−I79
K50−K52
S00−S09
C50−C50
C60−C63
G30−G32
2
1
0
−1
2001
Decade commencing
B
Combined improvements in five−year mortality following admission
change in lnHR from one decade to the next
0.50
0.25
0.00
−0.25
−0.50
2001
Decade commencing
C
Observed improvements in mortality
change in lnHR from one decade to the next
0.50
0.25
0.00
−0.25
−0.50
2001
Decade commencing

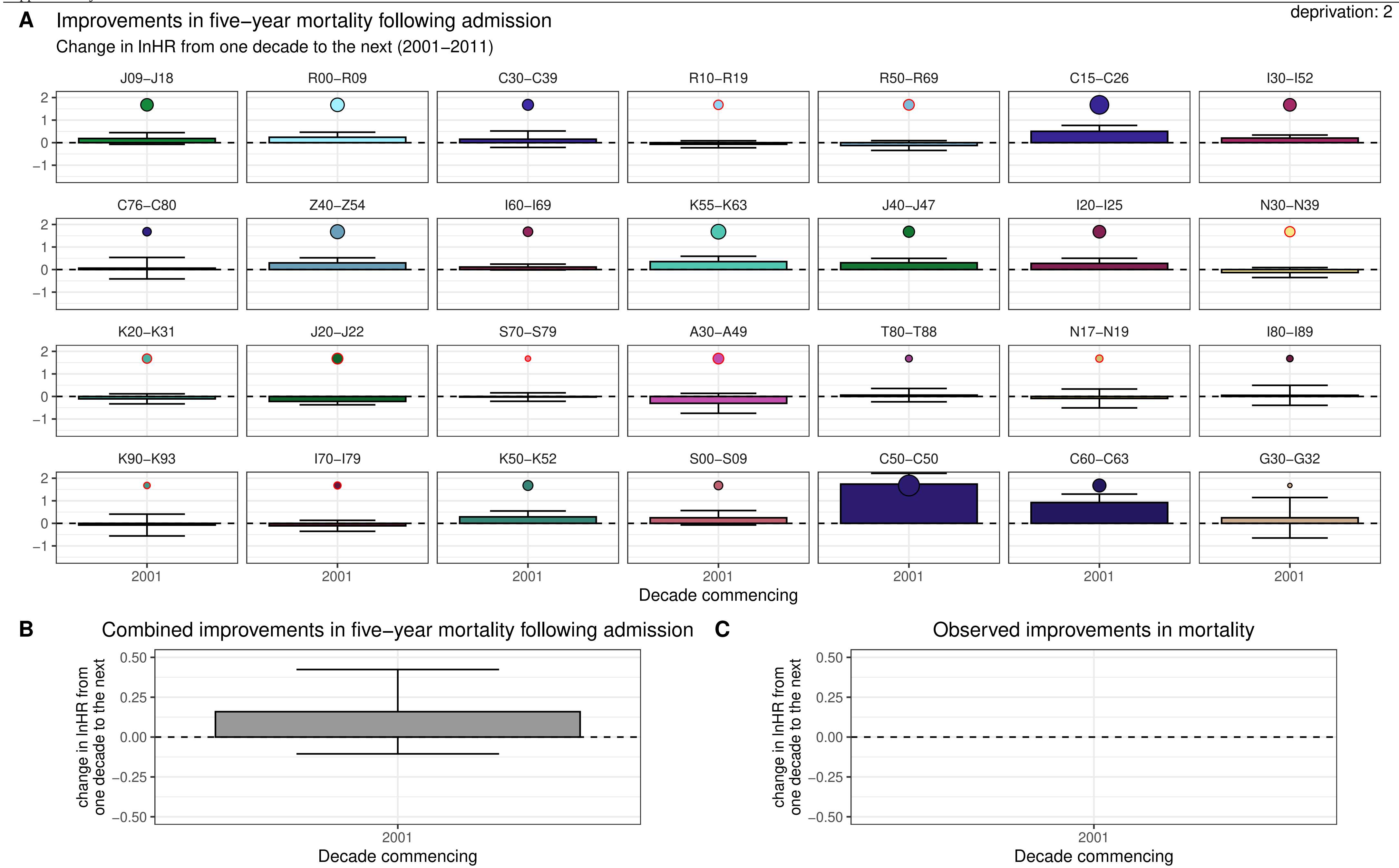
deprivation: 2
A
Improvements in five−year mortality following admission
Change in lnHR from one decade to the next (2001−2011)
J09−J18
R00−R09
C30−C39
R10−R19
R50−R69
C15−C26
I30−I52
C76−C80
Z40−Z54
I60−I69
K55−K63
J40−J47
I20−I25
N30−N39
K20−K31
J20−J22
S70−S79
A30−A49
T80−T88
N17−N19
I80−I89
K90−K93
I70−I79
K50−K52
S00−S09
C50−C50
C60−C63
G30−G32
2
1
0
−1
2001
Decade commencing
B
Combined improvements in five−year mortality following admission
change in lnHR from one decade to the next
0.50
0.25
0.00
−0.25
−0.50
2001
Decade commencing
C
Observed improvements in mortality
change in lnHR from one decade to the next
0.50
0.25
0.00
−0.25
−0.50
2001
Decade commencing

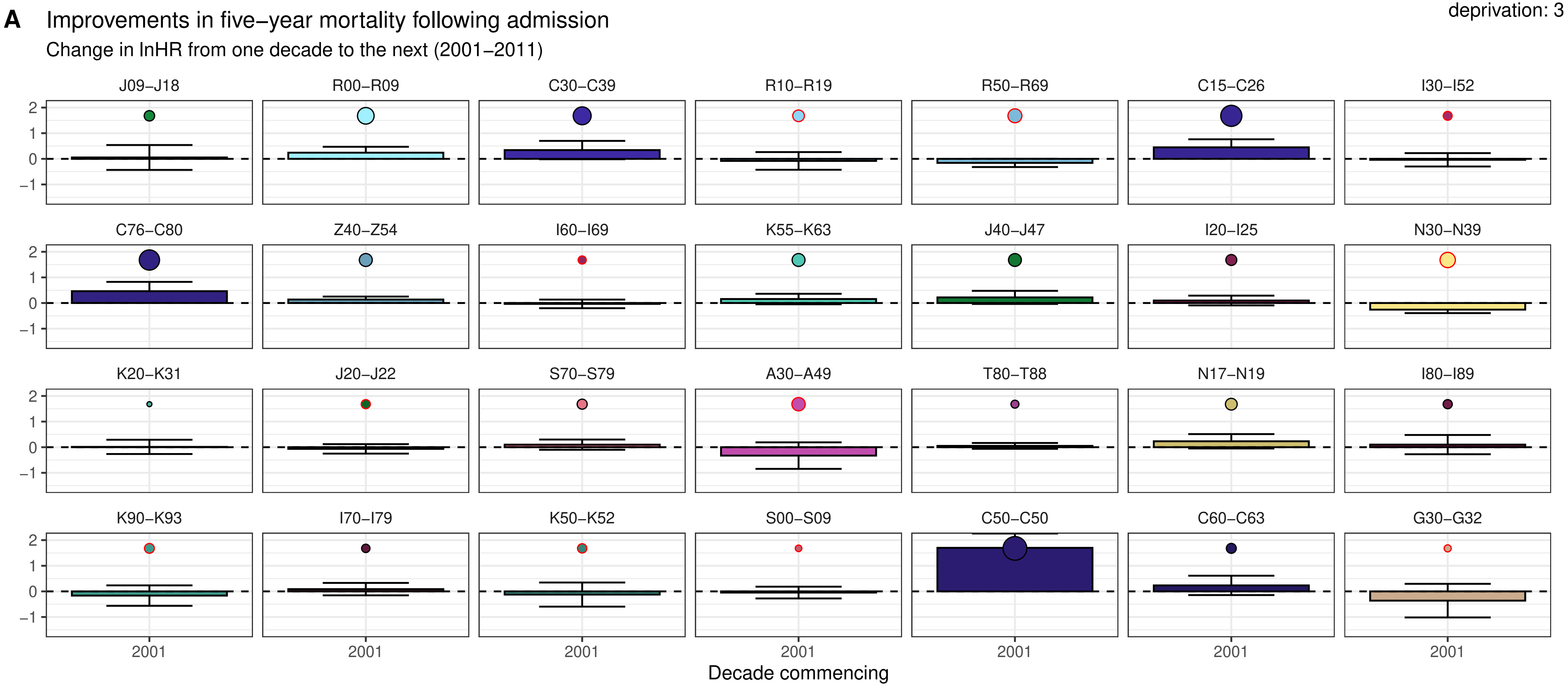
A
Improvements in five−year mortality following admission
Change in lnHR from one decade to the next (2001−2011)
deprivation: 3
J09−J18
R00−R09
C30−C39
R10−R19
R50−R69
C15−C26
I30−I52
C76−C80
Z40−Z54
I60−I69
K55−K63
J40−J47
I20−I25
N30−N39
K20−K31
J20−J22
S70−S79
A30−A49
T80−T88
N17−N19
I80−I89
K90−K93
I70−I79
K50−K52
S00−S09
C50−C50
C60−C63
G30−G32
2
1
0
−1
2001
Decade commencing

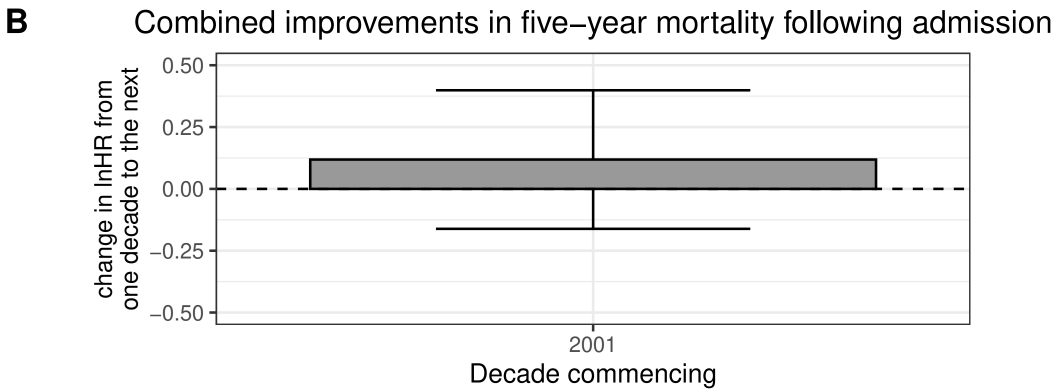
B
Combined improvements in five−year mortality following admission
change in lnHR from one decade to the next
0.50
0.25
0.00
−0.25
−0.50
2001
Decade commencing

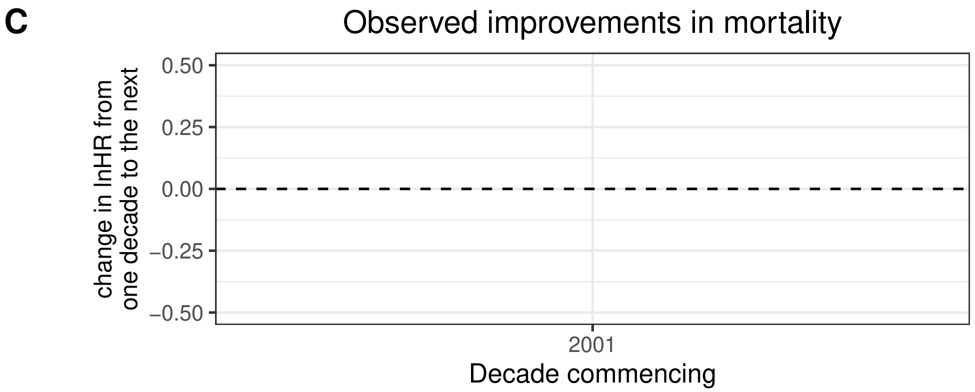
C
Observed improvements in mortality
change in lnHR from one decade to the next
0.50
0.25
0.00
−0.25
−0.50
2001
Decade commencing

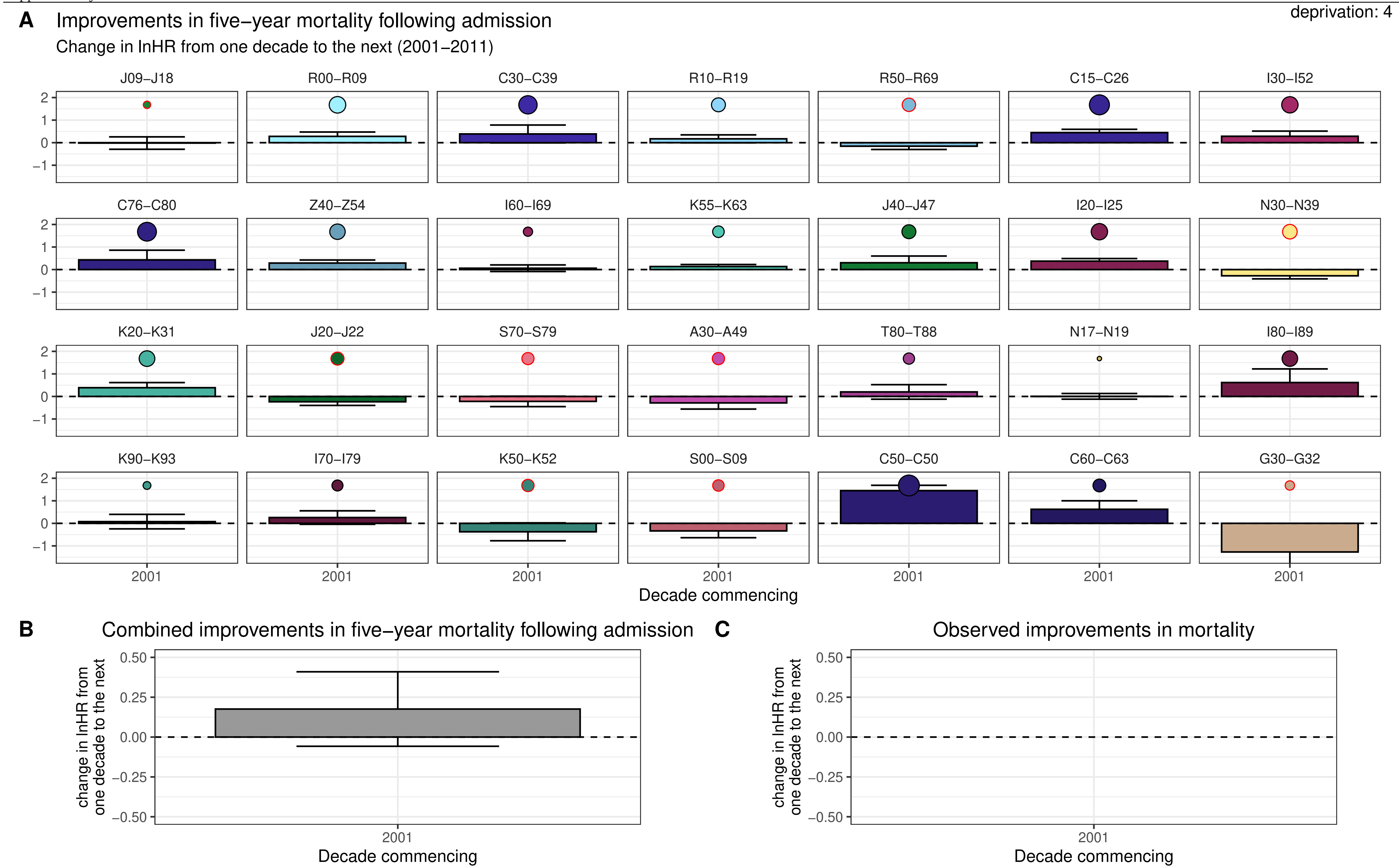
deprivation: 4
A
Improvements in five−year mortality following admission
Change in lnHR from one decade to the next (2001−2011)
J09−J18
R00−R09
C30−C39
R10−R19
R50−R69
C15−C26
I30−I52
C76−C80
Z40−Z54
I60−I69
K55−K63
J40−J47
I20−I25
N30−N39
K20−K31
J20−J22
S70−S79
A30−A49
T80−T88
N17−N19
I80−I89
K90−K93
I70−I79
K50−K52
S00−S09
C50−C50
C60−C63
G30−G32
2
1
0
−1
2001
Decade commencing
B
Combined improvements in five−year mortality following admission
change in lnHR from one decade to the next
0.50
0.25
0.00
−0.25
−0.50
2001
Decade commencing
C
Observed improvements in mortality
change in lnHR from one decade to the next
0.50
0.25
0.00
−0.25
−0.50
2001
Decade commencing

deprivation: 5

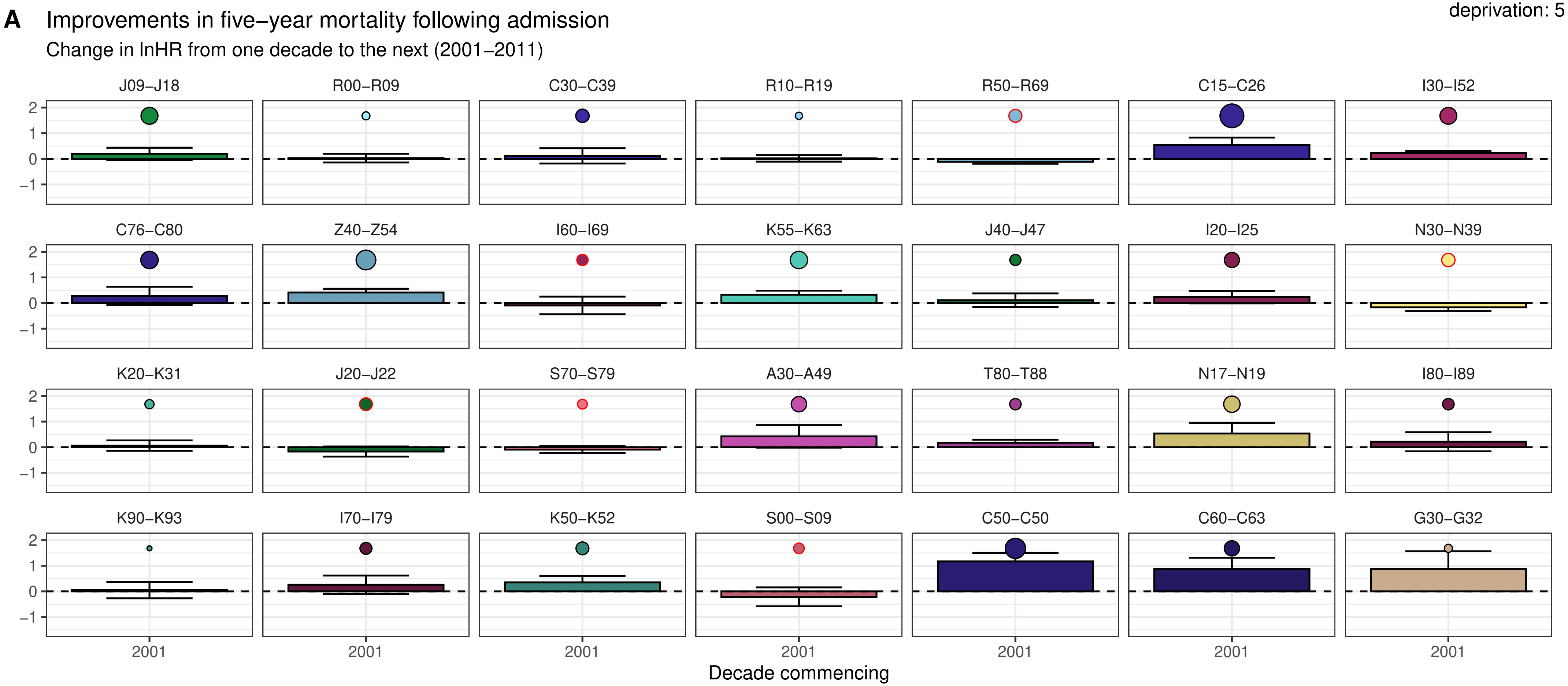

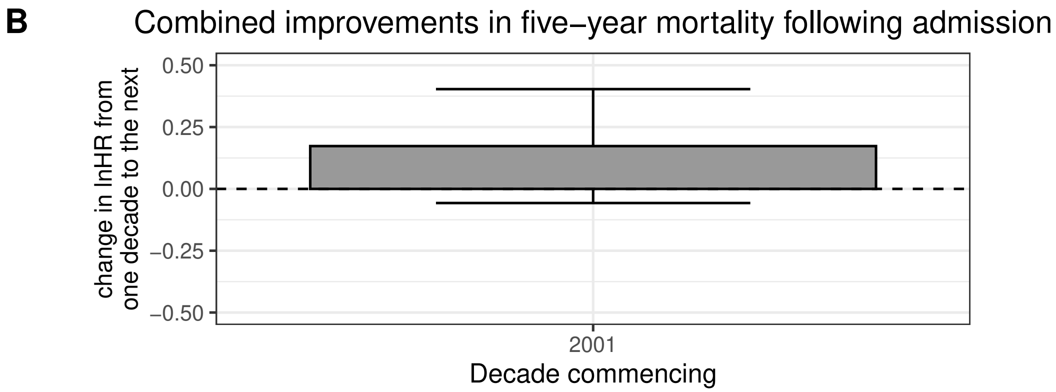

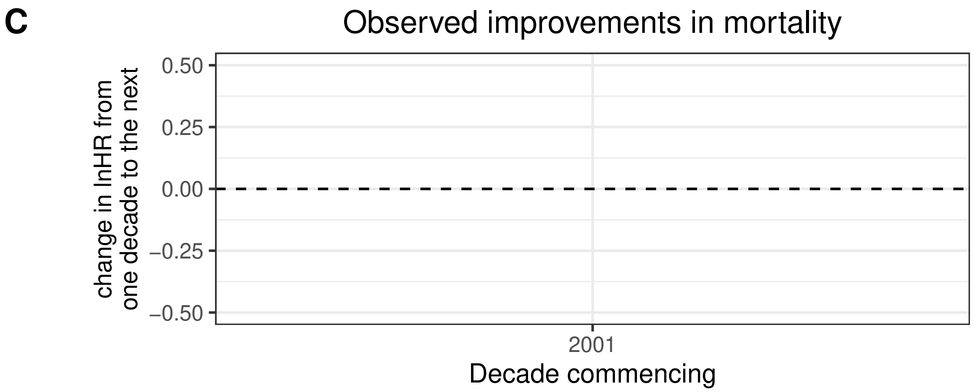

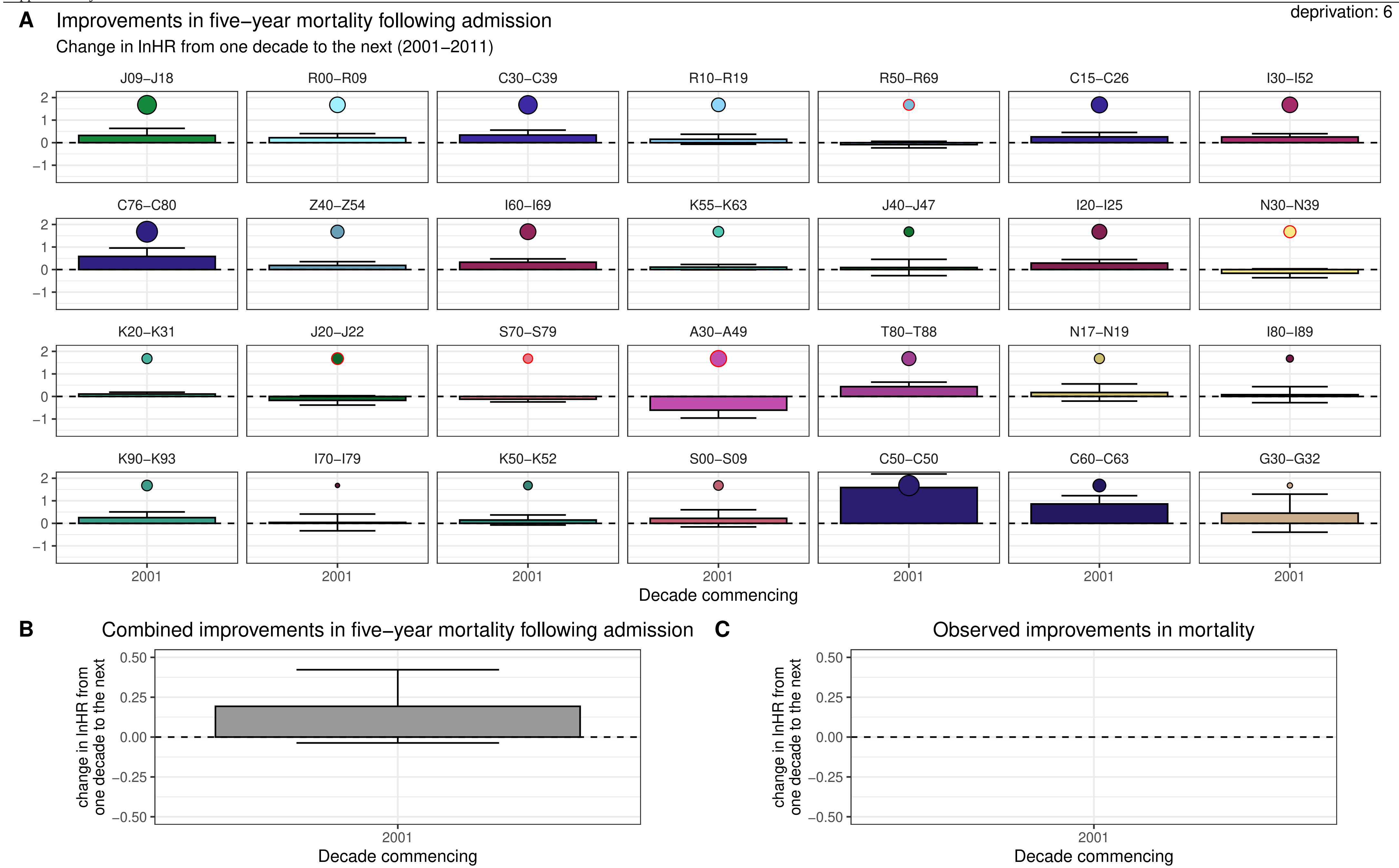
deprivation: 6
A Improvements in five−year mortality following admission
Change in lnHR from one decade to the next (2001−2011)
J09−J18
R00−R09
C30−C39
R10−R19
R50−R69
C15−C26
I30−I52
C76−C80
Z40−Z54
I60−I69
K55−K63
J40−J47
I20−I25
N30−N39
K20−K31
J20−J22
S70−S79
A30−A49
T80−T88
N17−N19
I80−I89
K90−K93
I70−I79
K50−K52
S00−S09
C50−C50
C60−C63
G30−G32
2
1
0
−1
2001
Decade commencing
B Combined improvements in five−year mortality following admission
change in lnHR from one decade to the next
0.50
0.25
0.00
−0.25
−0.50
2001
Decade commencing
C Observed improvements in mortality
change in lnHR from one decade to the next
0.50
0.25
0.00
−0.25
−0.50
2001
Decade commencing

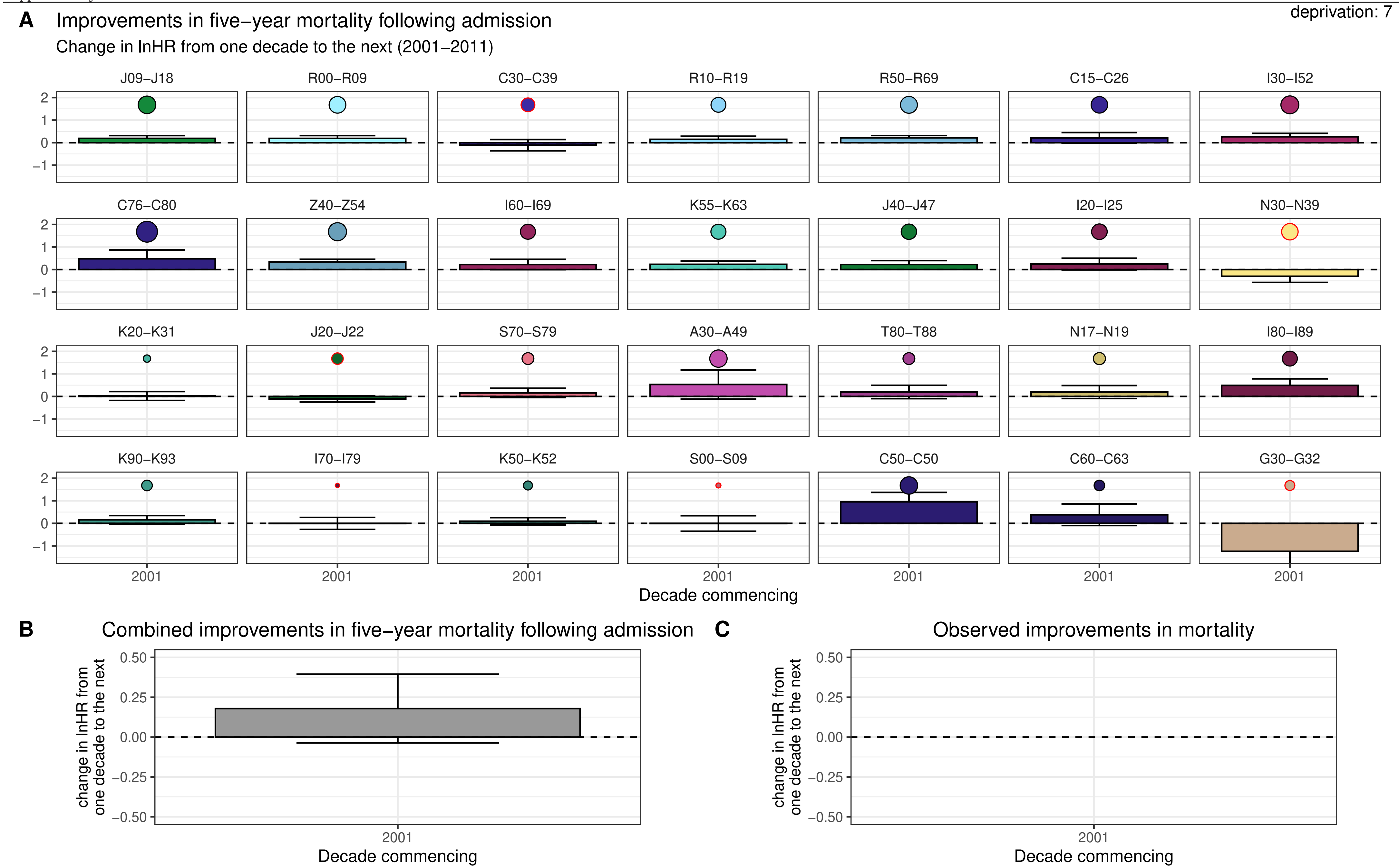
deprivation: 7
A
Improvements in five−year mortality following admission
Change in lnHR from one decade to the next (2001−2011)
J09−J18
R00−R09
C30−C39
R10−R19
R50−R69
C15−C26
I30−I52
C76−C80
Z40−Z54
I60−I69
K55−K63
J40−J47
I20−I25
N30−N39
K20−K31
J20−J22
S70−S79
A30−A49
T80−T88
N17−N19
I80−I89
K90−K93
I70−I79
K50−K52
S00−S09
C50−C50
C60−C63
G30−G32
2001
Decade commencing
B
Combined improvements in five−year mortality following admission
change in lnHR from one decade to the next
Decade commencing
C
Observed improvements in mortality
change in lnHR from one decade to the next
Decade commencing

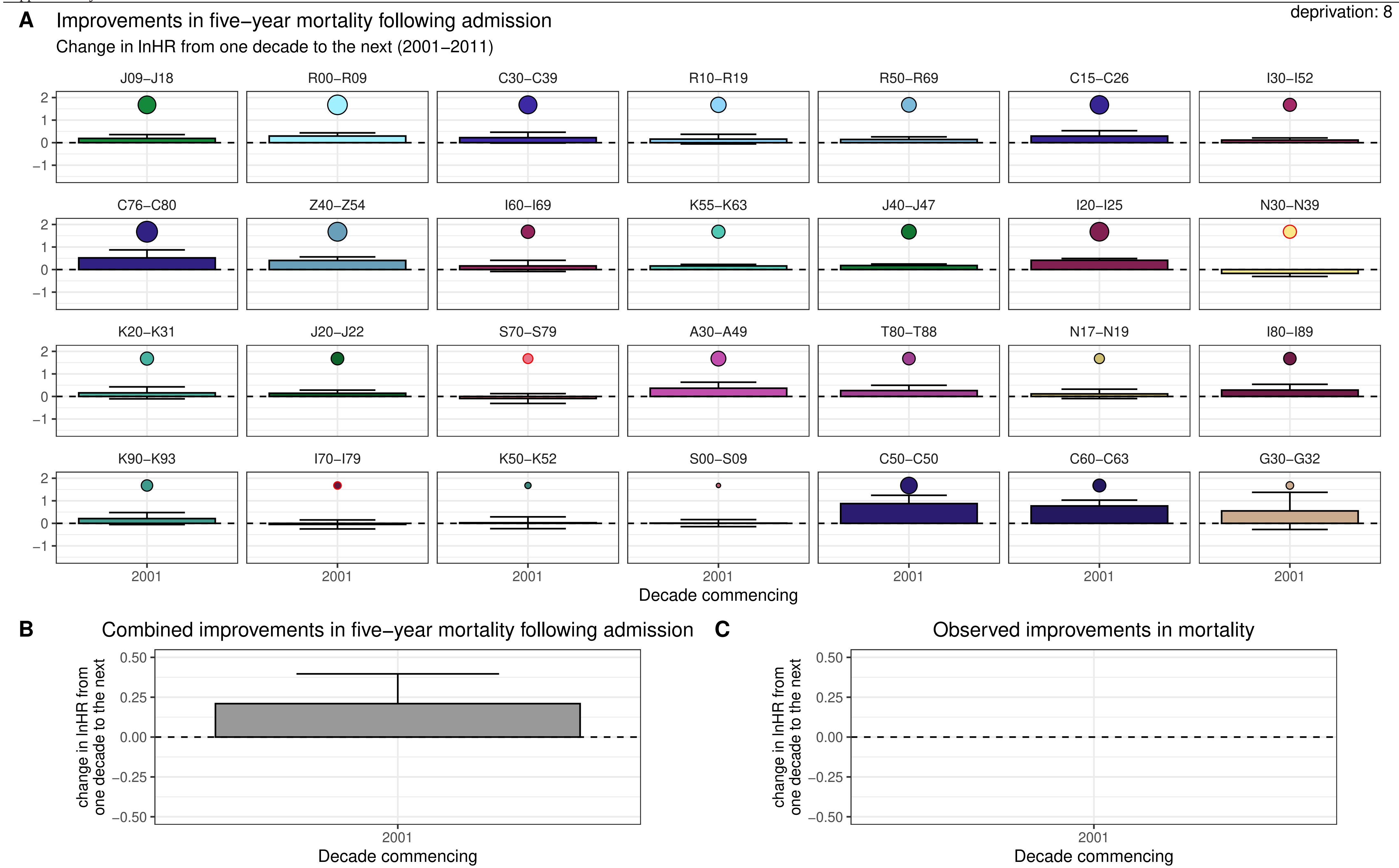

deprivation: 8
A
Improvements in five−year mortality following admission
Change in lnHR from one decade to the next (2001−2011)
J09−J18
R00−R09
C30−C39
R10−R19
R50−R69
C15−C26
I30−I52
C76−C80
Z40−Z54
I60−I69
K55−K63
J40−J47
I20−I25
N30−N39
K20−K31
J20−J22
S70−S79
A30−A49
T80−T88
N17−N19
I80−I89
K90−K93
I70−I79
K50−K52
S00−S09
C50−C50
C60−C63
G30−G32
2
1
0
−1
2001
Decade commencing
B
Combined improvements in five−year mortality following admission
change in lnHR from one decade to the next
0.50
0.25
0.00
−0.25
−0.50
2001
Decade commencing
C
Observed improvements in mortality
change in lnHR from one decade to the next
0.50
0.25
0.00
−0.25
−0.50
2001
Decade commencing

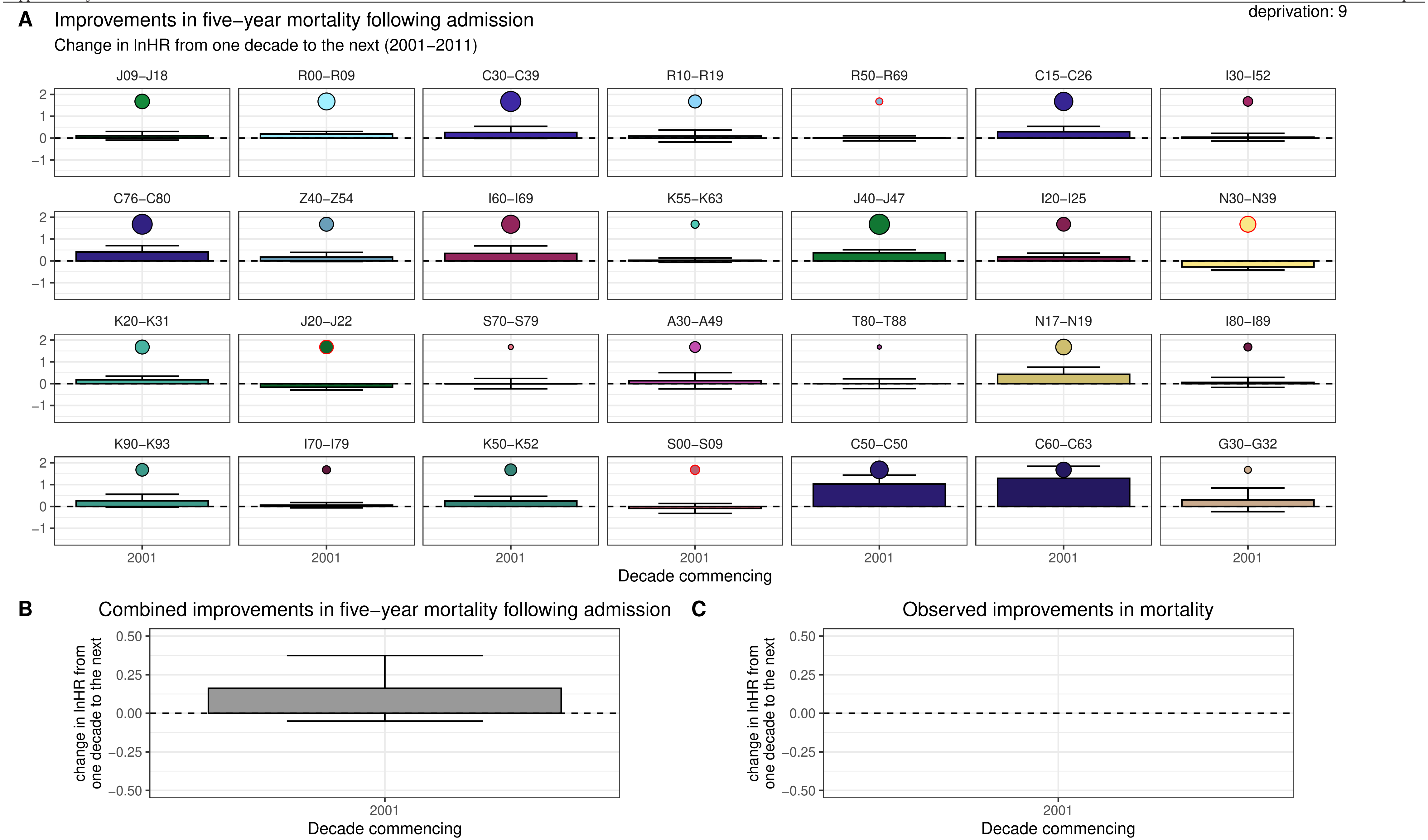
deprivation: 9
A
Improvements in five−year mortality following admission
Change in lnHR from one decade to the next (2001−2011)
J09−J18
R00−R09
C30−C39
R10−R19
R50−R69
C15−C26
I30−I52
C76−C80
Z40−Z54
I60−I69
K55−K63
J40−J47
I20−I25
N30−N39
K20−K31
J20−J22
S70−S79
A30−A49
T80−T88
N17−N19
I80−I89
K90−K93
I70−I79
K50−K52
S00−S09
C50−C50
C60−C63
G30−G32
2
1
0
−1
2001
Decade commencing
B
Combined improvements in five−year mortality following admission
change in lnHR from one decade to the next
0.50
0.25
0.00
−0.25
−0.50
2001
Decade commencing
C
Observed improvements in mortality
change in lnHR from one decade to the next
0.50
0.25
0.00
−0.25
−0.50
2001
Decade commencing

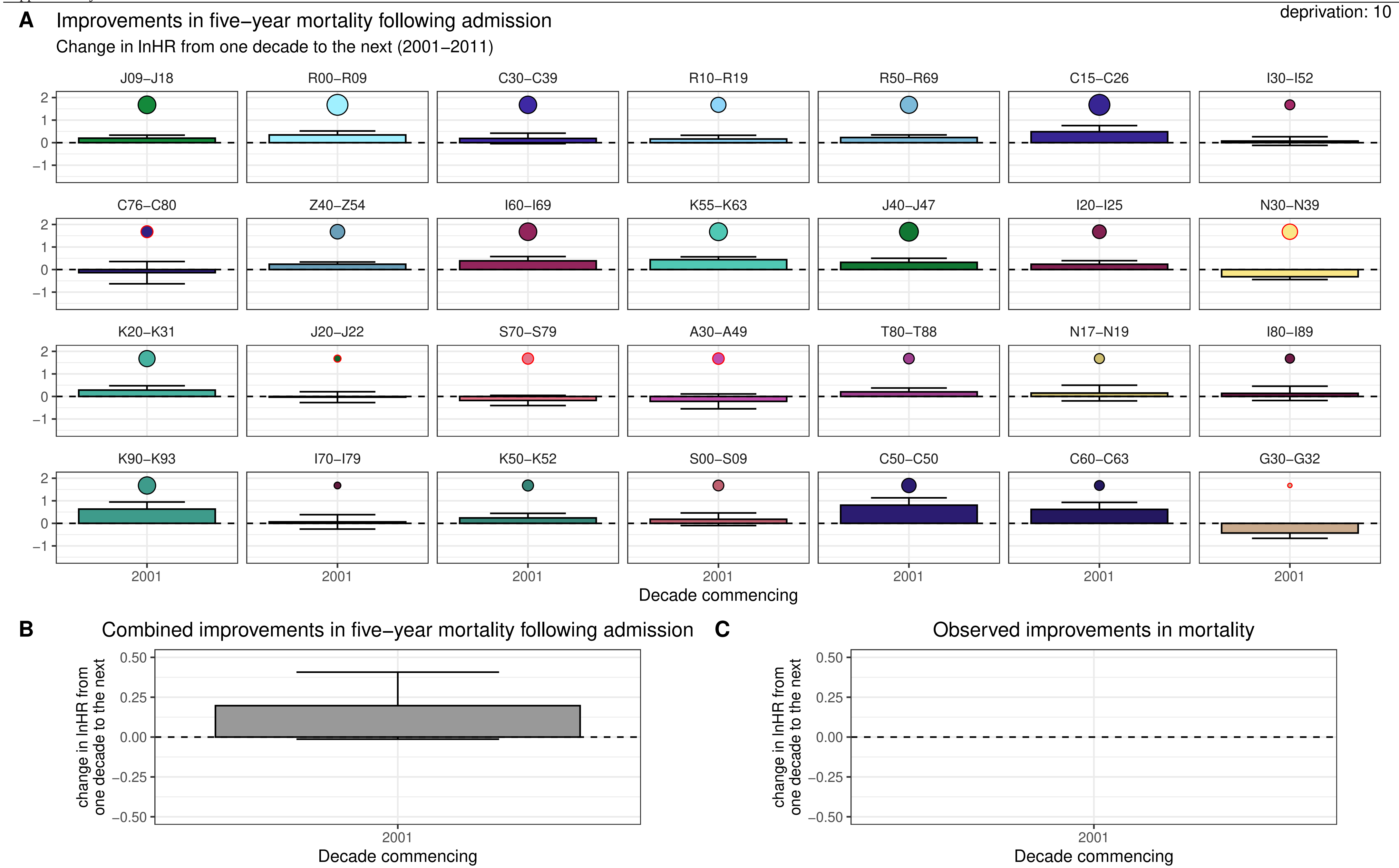
deprivation: 10
A
Improvements in five−year mortality following admission
Change in lnHR from one decade to the next (2001−2011)
J09−J18
R00−R09
C30−C39
R10−R19
R50−R69
C15−C26
I30−I52
C76−C80
Z40−Z54
I60−I69
K55−K63
J40−J47
I20−I25
N30−N39
K20−K31
J20−J22
S70−S79
A30−A49
T80−T88
N17−N19
I80−I89
K90−K93
I70−I79
K50−K52
S00−S09
C50−C50
C60−C63
G30−G32
2
1
0
−1
2001
Decade commencing
B
Combined improvements in five−year mortality following admission
change in lnHR from one decade to the next
0.50
0.25
0.00
−0.25
−0.50
2001
Decade commencing
C
Observed improvements in mortality
change in lnHR from one decade to the next
0.50
0.25
0.00
−0.25
−0.50
2001
Decade commencing
