# Supplementary material for: Trends in disease incidence and survival and their effect on mortality in Scotland: nationwide cohort study of linked hospital admission and death records 2001–2016
Source: BMJ Open. 2020 Mar 25;10(3):e034299. doi: 10.1136/bmjopen-2019-034299 (PMC7170664; doi:10.1136/bmjopen-2019-034299)
Supplement: Supplementary data [file bmjopen-2019-034299supp020.pdf]

Study population

Whilst necessary given the data structure, excluding all migrants during the study period will in many cases have excluded healthy people moving for economic or lifestyle opportunity. When health status influences the propensity to migrate, it forms a competing risk for both mortality and morbidity, which can bias results[1]. Due to the lack of mortality records on migrants, we are unable to assess the magnitude of this bias. Nevertheless, as we are principally concerned in the trend of morbidity and mortality, only changes in migration biases —not their absolute level—should affect our results materially.

Definition of diseases

We used recorded main position coding of first hospital admission of a disease block as the index event, excluding secondary diagnoses and any subsequent admissions for a disease within the same block. Hospital admission is only a proxy for the incidence of severe disease, and likely works better for chronic conditions like breast cancer where there is a single disease process that can result in a number of hospital admissions and may result in mortality in the timeframe of the study. It works perhaps less well for acute conditions, such as influenza, which can have distinct occurrences over the study period. Missing subsequent episodes of this type likely understates the burden of such diseases, although we expect trends in incidence and severity to be less affected by underreporting. Hospital admissions also have difficulty to capture chronic conditions which are managed in general practice or the community but eventually lead to death, such as dementia and multiple sclerosis. Although the CHI database captures this community illness through GP records, we only sought approval to access CHI data on patient date of birth and deprivation for this study, as GP records come with their own drawbacks (intractable scale, lack of precision, and heterogeneity between practices) and hospital admissions capture the incidence of most serious diseases. Future research may integrate GP records with our results to improve trend estimates of the diseases we were unable to model accurately.

Grouping of diseases into categories was another practical necessity; however, this grouping prevents us from assessing the trends of individual diseases, including the possibility of opposing trends offsetting each other. For example, the improvement seen in "General symptoms and signs involving the circulatory and respiratory systems" (R00-R09) does not reveal whether trends in "Pain in throat and chest" (R07) and "Abnormalities of breathing" (R06), the two most common diagnoses within the category (Table 2—Source Data 2), were directionally concordant.

Considerations with hospital admission data

Association between hospital admission for a condition and the latter occurrence of death does not necessarily imply causation, and may be confounded by other health risks, such as socioeconomic status, a second disease, or behavioural factors, although our analysis stratified by socioeconomic status may partly mitigate these effects. Should a patient have been admitted for several diseases prior to death, they would have been recorded as dying with all of those diseases. This is a strength in that diseases causing other diseases and in turn death are captured, although diseases not leading to death (DNLD) are also potentially captured as a cause of death. The latter is mitigated by the fact that subjects without DNLD should have been subject to similar other morbidity and mortality risks. The effect of DNLD across the population is thus properly measured. In any case, modelling comorbidities

simultaneously or modelling the causal effects of one morbidity on another and consequently on death has been beyond the scope of this study. Where an unanalysed comorbidity (for example, lung cancer amongst heart disease patients) is reducing across a dimension (such as over time, or increasing socioeconomic status), the reduction in the competing risk (lung cancer) will have led to a decrease in measured mortality amongst those with heart disease, but this would be counterbalanced to an unknown degree by a similar effect amongst those without heart disease. Future work might seek to consider this point using competing risk regression or site-specific survival.

By relying on routine data, we have been able to create a very large dataset, at the expense of being unable to externally check individual fields, such as primary diagnosis, where there are known to be inaccuracies[2]. However, our conclusions will be less affected if these inaccuracies are reasonably stable over time.

Changes in hospital admission rates could have been influenced by changes in referrals, waiting times, and disease screenings, especially if these changes differed by sex or socioeconomic status. For example, hospital waiting times in Scotland have fluctuated during the study period[3], coinciding with an increase in the demand for private healthcare[4], and an increase in the likelihood of patients' disease being managed in community care[5]. However, less than 10% of Scottish patients have private healthcare insurance and the NSS has not made a concerted change in hospital access for acute diseases[6]. Differences in access may well contribute to socioeconomic differences, but we would see them as a way in which the impact of deprivation on health is mediated rather than as a reason to discount the importance of deprivation as a health determinant. Conversely, changes in disease screening are likely to influence both hospital admission rates and subsequent survival. The UK has a long-established breast cancer screening programme, which evolved during the study period: the upper age for women to be screened was extended from 64 years old to 70 years old in 2004 and attendance rates fluctuated between 71%-76%, with its highest point in 2008[7]. The rate of overdiagnosis is around 11%[8], but whether this has changed over the study period is unknown.

Any increases in rate of screening and overdiagnosis would be reflected in our study by an improvement in disease survival and a deterioration in the incidence. One such example is the observed increase in incidence of influenza and pneumonia, which was offset by increased survival and may reflect changes in coding practices and/or frequency of referral[9]. For breast cancer, we observe an improvement in both incidence and survival, and this improvement is similar in shape those of prostate cancer, which does not have a national screening programme. As such, a degree of caution is needed in interpreting the exact partition between disease incidence and survival, but changes due to screening will have offset each other in terms of the overall change in morbidity.

Modelling assumptions

Our model assumed that (age-adjusted) incidence is a function of year of birth, but that mortality post-incidence is a function of the year of incidence. This is clearly an oversimplification, but necessary given that year of birth and year of incidence are completely confounded for a given age at incidence. Furthermore, we have used a simple definition of burden of disease: the total number of cases multiplied by the excess hazard across all ages. More complex definitions might have looked at Years of Life Lost (YLL) and taken into account

any age-related pattern in incidence. However, our measure is simple and is predominantly used to weight the diseases and especially their improvements to calculate an overall improvement in all-disease impact, rather than as an absolute measure of burden.

Our model also assumed disease incidence and survival hazards were proportionate. Disease status hazards were not always proportional across the baseline (age defined) hazard. Burden is thus sensitive to the population's age distribution, but is captured for the population under study (as the mean hazard increase across ages is captured). Furthermore, trends in hazard ratios measured should be robust given 1) the reasonably steady age distribution and 2) the shapes of mortality and incidence curves were constant over the study period. Conversely, recently doubt was cast on the effect of using decade of birth groupings in a standardised mortality analysis[10] due to changing patterns of births within the decade: that concern does not apply here as the Cox baseline hazard used full (not rounded) age information, with decade of birth fitted as a covariate. Using the cox model also allowed us to infer a baseline hazard across the whole range of ages (50+) , despite individuals only having been observed for 15 years, akin to constructing a period mortality table. However, further complexity was introduced by then calculating hazard ratios between successive decades of birth, relying upon a five year overlap in observed ages over 15 years in subjects born 10 years apart. Whilst if all the model assumptions hold, this is correct, it should not be used unthinkingly to infer trends in outcomes and especially differences between decades of birth prior to the study start. As such, there may be survivor biases in attempts to infer whole-of-life decade of birth inferences from our study. Nonetheless, we believe inferences of trends in disease and mortality and causal links remain valid with the period of study.

References

1 Andersen PK, Geskus RB, De witte T, *et al.* Competing risks in epidemiology: Possibilities and pitfalls. *Int J Epidemiol* 2012;**41**:861–70. doi:10.1093/ije/dyr213

2 ISD Scotland. Assessment of SMR01 Data Scotland 2014-2015. 2012.

3 Information Services Division. Inpatient and Day Case Waiting Times Trends: Long term trends. 1 April 1997 to 30 September 2018. 2019. https://beta.isdscotland.org/find-publications-and-data/healthcare-resources/waiting-times/inpatient-and-day-case-waiting-times-trends/ (accessed 20 Jan 2020).

4 Bíró A, Hellowell M. Public–private sector interactions and the demand for supplementary health insurance in the United Kingdom. *Health Policy (New York)* 2016;**120**:840–7. doi:10.1016/j.healthpol.2016.05.002

5 Information Services Devision. National Service Scotland: Acute Hospital Activity and NHS Beds Information. 2018.

6 Steel D, Cylus J. United Kingdom (Scotland): Health system review. *Health Syst Transit* 2012;**14**:xv–xxii, 1–150.http://www.ncbi.nlm.nih.gov/pubmed/23579054 (accessed 20 Jan 2020).

7 Information Services Division. Scottish Breast Screening Programme. 2018. https://www.isdscotland.org/Health-Topics/Cancer/Breast-Screening/ (accessed 20 Jan 2020).

8 Marmot MG, Altman DG, Cameron DA, *et al.* The benefits and harms of breast cancer screening: An independent review. *Br J Cancer* 2013;**108**:2205–40. doi:10.1038/bjc.2013.177

9 Nair H, Watts AT, Williams LJ, *et al.* Pneumonia hospitalisations in Scotland following the introduction of pneumococcal conjugate vaccination in young children. *BMC Infect*

Dis 2016;**16**:1–11. doi:10.1186/s12879-016-1693-x

10 Minton J, Green M, McCartney G, *et al.* Two cheers for a small giant? Why we need better ways of seeing data: A commentary on: ‘Rising morbidity and mortality in midlife among White non-Hispanic Americans in the 21st century’. *Int J Epidemiol* 2017;**46**:356–61. doi:10.1093/ije/dyw095
