# Supplementary material for: Trends in disease incidence and survival and their effect on mortality in Scotland: nationwide cohort study of linked hospital admission and death records 2001–2016
Source: BMJ Open. 2020 Mar 25;10(3):e034299. doi: 10.1136/bmjopen-2019-034299 (PMC7170664; doi:10.1136/bmjopen-2019-034299)
Supplement: Author's manuscript [file bmjopen-2019-034299.draft_revisions.pdf]

BMJ Open is committed to open peer review. As part of this commitment we make the peer review history of every article we publish publicly available.

When an article is published we post the peer reviewers' comments and the authors' responses online. We also post the versions of the paper that were used during peer review. These are the versions that the peer review comments apply to.

The versions of the paper that follow are the versions that were submitted during the peer review process. They are not the versions of record or the final published versions. They should not be cited or distributed as the published version of this manuscript.

BMJ Open is an open access journal and the full, final, typeset and author-corrected version of record of the manuscript is available on our site with no access controls, subscription charges or pay-per-view fees (http://bmjopen.bmj.com).

If you have any questions on BMJ Open's open peer review process please email
info.bmjopen@bmj.com

BMJ Open

Improvements in the incidence and survival of cancer and cardiovascular but not infectious disease have driven recent mortality improvements in Scotland: nationwide cohort study of linked hospital admission and death records 2001–2016

Journal:	*BMJ Open*
Manuscript ID	bmjopen-2019-034299
Article Type:	Original research
Date Submitted by the Author:	16-Sep-2019
Complete List of Authors:	Timmers, Paul; The University of Edinburgh Usher Institute of Population Health Sciences and Informatics, Centre for Global Health Research<br>Kerssens, Joannes; NHS National Services Scotland, Information Services Division<br>Minton, Jon; NHS Health Scotland, Public Health Observatory<br>Grant, Ian; NHS National Services Scotland, Information Services Division<br>Wilson, James; The University of Edinburgh Usher Institute of Population Health Sciences and Informatics, Centre for Global Health Research; The University of Edinburgh MRC Institute of Genetics and Molecular Medicine, Human Genetics Unit<br>Campbell, Harry; The University of Edinburgh Usher Institute of Population Health Sciences and Informatics, Centre for Global Health Research<br>Fischbacher, Colin; NHS National Services Scotland, Information Services Division<br>Joshi, Peter; The University of Edinburgh Usher Institute of Population Health Sciences and Informatics, Centre for Global Health Research
Keywords:	EPIDEMIOLOGY, PUBLIC HEALTH, Public health < INFECTIOUS DISEASES

SCHOLARONE™
Manuscripts

I, the Submitting Author has the right to grant and does grant on behalf of all authors of the Work (as defined in the below author licence), an exclusive licence and/or a non-exclusive licence for contributions from authors who are: i) UK Crown employees; ii) where BMJ has agreed a CC-BY licence shall apply, and/or iii) in accordance with the terms applicable for US Federal Government officers or employees acting as part of their official duties; on a worldwide, perpetual, irrevocable, royalty-free basis to BMJ Publishing Group Ltd ("BMJ") its licensees and where the relevant Journal is co-owned by BMJ to the co-owners of the Journal, to publish the Work in this journal and any other BMJ products and to exploit all rights, as set out in our licence.

The Submitting Author accepts and understands that any supply made under these terms is made by BMJ to the Submitting Author unless you are acting as an employee on behalf of your employer or a postgraduate student of an affiliated institution which is paying any applicable article publishing charge ("APC") for Open Access articles. Where the Submitting Author wishes to make the Work available on an Open Access basis (and intends to pay the relevant APC), the terms of reuse of such Open Access shall be governed by a Creative Commons licence – details of these licences and which Creative Commons licence will apply to this Work are set out in our licence referred to above.

Other than as permitted in any relevant BMJ Author's Self Archiving Policies, I confirm this Work has not been accepted for publication elsewhere, is not being considered for publication elsewhere and does not duplicate material already published. I confirm all authors consent to publication of this Work and authorise the granting of this licence.

For peer review only

Improvements in the incidence and survival of cancer and cardiovascular but not infectious disease have driven recent mortality improvements in Scotland: nationwide cohort study of linked hospital admission and death records 2001–2016

Paul RHJ Timmers[1], Joannes J Kerssens[2], Jon W Minton[3], Ian Grant[2], James F Wilson[1,4], Harry Campbell[1], Colin M Fischbacher[2], Peter K Joshi[1]

1 Centre for Global Health Research, Usher Institute of Population Health Sciences and Informatics, University of Edinburgh, Edinburgh, UK

2 Information Services Division, NHS National Services Scotland, Edinburgh, UK

3 Public Health Observatory, NHS Health Scotland, Edinburgh, UK

4 MRC Human Genetics Unit, Institute of Genetics and Molecular Medicine, University of Edinburgh, Edinburgh, UK

Corresponding author:

Paul Timmers, paul.timmers@ed.ac.uk

For peer review only - http://bmjopen.bmj.com/site/about/guidelines.xhtml

Abstract

Objectives: Identifying the causes and future trends underpinning improvements in Scottish mortality and quantify the relative contributions of disease incidence and survival.

Design: Population-based study.

Setting: Linked secondary care and mortality records across Scotland.

Participants: 1,967,130 individuals born 1905–1965 and resident in Scotland 2001–2016.

Main outcome measures: Hospital admission rates and survival within five years post-admission for 28 diseases, stratified by sex and socioeconomic status.

Results: The three hospital admission diagnoses associated with most deaths were “Influenza and pneumonia”, “Symptoms and signs involving circulatory and respiratory systems”, and “Malignant neoplasm of respiratory and intrathoracic organs”. Using disease trends, we modelled a mean mortality hazard ratio of 0.737 (95% CI 0.730–0.745) across decades of birth, equivalent to a life extension of ~3 years per decade. This improvement was 61% (30%–93%) accounted for by improved disease survival after hospitalisation (principally cancer) with the remainder accounted for by lowered hospitalisation incidence (principally heart disease and cancer). In contrast, deteriorations in infectious disease incidence and survival increased mortality by 9% (~3.3 months per decade). Disease-driven mortality improvements were slightly greater for men than women (due to greater falls in disease incidence), and generally similar across socioeconomic deciles. We project mortality improvements will continue over the next decade but will slow by 21% because much progress in disease survival has already been achieved.

Conclusion: Morbidity improvements broadly explain observed improvements in mortality, with progress on prevention and treatment of heart disease and cancer contributing most. The gaps between men and women’s health are closing, but those between socioeconomic groups

For peer review only - http://bmjopen.bmj.com/site/about/guidelines.xhtml

46 are not. A slowing trend in morbidity improvements may explain the stalling in improvements of period life expectancies observed recently in the UK. However, these could be offset if we accelerate improvements in the diseases accounting for most deaths and counteract recent deteriorations in infectious disease.

For peer review only

For peer review only - http://bmjopen.bmj.com/site/about/guidelines.xhtml

Strengths and limitations of this study

- The individual-level linkage of hospital and death records in this population-wide dataset allows for direct modelling of improvements in 28 disease categories in terms of improvements in disease incidence and subsequent survival, stratified by sex and socioeconomic status.

- Exclusion of migrating individuals means changes in disease are unaffected by population shifts, and allow for diseases to be compared with each other and summarised into trends in mortality based on morbidity.

- Hospital admission diagnosis and subsequent survival avoid issues with cause of death recording; however, they do not provide evidence of the causal effect of disease on mortality and may in some cases track changes in underlying frailty.

- This study is limited to the assessment of diseases which result in a hospital admission prior to death.

For peer review only

Introduction

In recent decades, there has been a substantial improvement in life expectancies at birth in the UK(1). More recently, several studies have suggested that there has been slowdown in improvements in the USA, UK, France, Germany, Sweden, the Netherlands and other Organisation for Economic Co-operation and Development countries; however, the causes are less clear, with speculation that they may arise from slowing improvements in cardiovascular disease, increased influenza mortality and/or pressure on health and social care services(1–8). Understanding trends in disease incidence and subsequent survival could illuminate such trends in mortality, and disentangling how and how much different diseases contribute has the potential to reveal whether investment in healthcare and research is directed at the most urgent diseases and most affected individuals.

Through its electronic Data Research and Innovation Service (eDRIS), Scotland has linkage of historical individual death and electronic health records in a controlled environment, with specific study approvals by the Public Benefit and Privacy Panel. This allows direct modelling at an individual level of the incidence of disease and subsequent death or survival of subjects. Furthermore, because historic records are available and the whole population is covered, a retrospective cohort study can be constructed (with inherent representativeness of the initial sample, with very complete levels of follow-up, and without survivor bias).

Here, we use population-wide data between 2001 and 2016 on residents of Scotland born before 1966 to explore how trends in longevity were driven by different trends in broad classes of disease incidence or survival, and highlight diseases which have shown more or less improvement in their contribution to overall mortality. We partition overall mortality by sex and socioeconomic status and, assuming past disease improvements continue to the same extent in the future, use these results to project future improvements in mortality and their changing sources.

For peer review only

For peer review only - http://bmjopen.bmj.com/site/about/guidelines.xhtml

Methods

Data sources

We received administration and care records from NHS National Services Scotland (NSS) from 2001. All methods and results are reported in line with RECORD guidelines(9).

Community Health Index dataset

Records were extracted from the historic and current Community Health Index (CHI) dataset. This is a register of all patients in NHS Scotland and is fed by eight regional databases (e.g, GP database, cancer screening). The register is considered complete from 2001 onwards. The CHI number, contained in the dataset, is effectively a patient identifier and added to other health datasets to make linkage possible, for instance between hospital admissions, death records and the Scottish cancer registry(10). Our extract consisted of 2,691,304 de-identified records, constituting the identified population of Scotland in 2001 who had been born between 1905 and 1965. The Scottish Index of Multiple Deprivation (SIMD)(11) was used to quantify socioeconomic status, determined by individuals' full postcode, and subsequently converted into deciles. The dataset we received contained only records with district-level postcodes and SIMD deciles, of which we excluded individuals with district codes with less than 5,000 individuals (thereby excluding anomalous postcodes, often with special meanings, such as "marketing campaign"; N = 11,564). We also excluded individuals missing from the CHI database in 2016, but not recorded as dead (and therefore likely transferred out of Scotland; N = 573,711), individuals with record discrepancies between the CHI and National Records of Scotland databases (N = 79,131), and individuals with records outside of the study dates or missing information on socioeconomic class (N = 59,767), giving 1,967,130 individuals for analysis after quality control (S Figure 1). Characteristics of the excluded individuals were similar to the rest of the population, except for postcode exclusions and database transfers, which were missing socioeconomic information and death records, respectively, as expected (S Figure 1—Source Data 1).

NRS death records

We received 1,477,796 death records from the National Registry of Scotland, of which 699,093 could be matched to the CHI database before quality control. Of the matched records, 176,197

For peer review only - http://bmjopen.bmj.com/site/about/guidelines.xhtml

belonged to individuals that were excluded during CHI quality control, leaving 602,506 total deaths for analysis (Table 1).

Acute hospital admission

Health records were also linked to 30,054,191 acute hospital admissions, of which 17,264,379 were dated between 2001 and 2016 and could be matched (Table 1—Source Data 1 & 2).

Disease classification

The main diagnosis of acute hospital admission records was used to classify records into disease categories, which corresponded to disease blocks as described in the chapters of the ICD10(12). In order to model the effect of disease incidence, we used only the first admission of disease for each individual, excluding subsequent visits to the hospital for the same disease. The term "incidence" is used throughout this study to refer to the first recorded hospital admission of the disease during the study period.

Design

Mortality trends were modelled using morbidity trends: we first determined the major disease categories (ICD10 blocks) associated with the most lives lost by taking into account the frequency of the disease (as measured by hospitalisation) and its effect on survival (as measured by the subsequent all-cause mortality of patients admitted for the disease compared to the mortality of everyone else). We combined these two measures to calculate a burden of death weighting for each block. We then looked at how the age-adjusted trends in hospitalisation rates (as a proxy for incidence) changed for each disease, by decade of birth, projecting that if incidence of a disease fell by a given percentage, its contribution to mortality would fall similarly. We then calculated a weighted average change in hospitalisation rates, reflecting the expected effect of all measured disease incidence changes on mortality rates, driven by decade of birth. Similarly, we looked at how the (age-adjusted) 5-year survival rates following first hospitalisation changed by year of hospitalisation. For each block this again gives a contribution towards reduced mortality, and the weighted average, the expected effect of changes in survival of the combined diseases on overall mortality. Adding these effects (and noting we assessed changes in survival from incidences over one decade), gives the expected effect on overall mortality from decade of birth to subsequent decade of birth from the effect of changes in disease incidence and survival, under the (necessarily simplified) model that

For peer review only - http://bmjopen.bmj.com/site/about/guidelines.xhtml

incidence is a function of birth cohort and survival post incidence is a function of year of incidence.

Statistical analysis

Mortality

A Cox proportional hazards model using NRS mortality data – fitting sex, decade of birth, and deprivation – was used to quantify mortality in the Scottish population during the study period. The same analysis was run stratified by sex and deprivation. Unless otherwise stated, (for example median age differences in Kaplan-Meier curves), years of life of a hazard effect have been calculated by multiplying the lnHR by 10 (ref. 13). Only individuals with complete records were included in the analysis.

Morbidity

We grouped the main diagnoses of each NSS hospital admission into categories, as laid out by the ICD10 Chapters, and included only the first instance of admission for a category per individual (discarding subsequent repeat visits to hospital for the same disease). Analysis was restricted to more common disease blocks. Visual inspection suggested a pragmatic threshold of at least 15,000 first-time admissions (see Table 2—Source Data 2 for all disease categories meeting this threshold).

Effects on the incidence of hospitalisation for the more common disease blocks was quantified using Cox proportional hazard models based on age, with events defined as the first incidence of hospitalisation. We fitted sex, deprivation, and decade of birth as covariates. Again, the same analyses were performed stratifying by sex and deprivation.

In order to quantify all-cause mortality in the five years following hospitalisation, person-time of individuals was divided into phases, corresponding to the study start until hospitalisation, the first five years after hospitalisation, and the remaining time in the study. For example, an individual admitted to hospital in 2004 for ischaemic heart disease I20-25 (IHD) and surviving until 2010 would contribute three phases to the model: one for the period until hospitalisation (no event), one for the first 5 years after hospitalisation (no event), and one >5 years after

For peer review only - http://bmjopen.bmj.com/site/about/guidelines.xhtml

hospitalisation (event after 1 year). The status of each phase was fitted as a covariate in a Cox proportional hazards model(14) with death as the event, adjusting for sex and deprivation:

$$h(x) = h_0(x)\ e^{\beta_1 X_1\ +\ \beta_2 X_2\ +\ \beta_3 X_3\ +\ \beta_4 X_4} \ (1)$$

Where h_0 is the baseline hazard, x the patient age, and X_1-X_4 the covariates sex, deprivation, and logically coded phase status (0-5 years True/False and >5 years True/False), with corresponding effect sizes β_1-β_4. This yielded estimates of the proportional hazard of status (0-5 and >5 years) after hospitalisation compared to pre-hospitalisation mortality. The same model was run, stratified by sex and deprivation.

Burden

For disease blocks with at least 15,000 first admissions during the study period, the relative mortality burden of each disease block was calculated as the excess mortality (lnHR) in the 5 years after hospital admission (Equation 1) multiplied by the number of first-time admissions for the disease block, as follows:

$$N_{firstadmission}/N_{total}\ *\ h_{(0,5)} \ (2)$$

Where $N_{firstadmission}$ is the total number of first hospital admissions of the disease during the study period, N_{total} is the total number of individuals in the study, and $h_{(0,5)}$ is the mortality of individuals in the first five years following hospitalisation compared to individuals who were never hospitalised for the disease, measured in lnHR. The resulting value was then scaled to [0-1] and provides a relative measure of the number of lives lost due to the disease, with higher values indicating a disease which is more common or associated with higher subsequent mortality, and lower values indicating a disease which is rare or associated with lower subsequent mortality. Whilst this measure may in principle be affected by differing age patterns on incidence, it was judged sufficient for our purpose – to establish broad relative weightings of the importance of each disease.

To maintain a feasible computational burden within the national safe haven, subsequent analysis was restricted to the 25 blocks with the highest burden of death on the population (Table 2). We added C50-C50 malignant neoplasm of the breast, C60-C63, malignant neoplasms of male genital organs, and G30-G32 other diseases of the nervous system to this list, out of specific interest: in the sex-specific effects and awareness of the limitations of our method for Alzheimer's disease (see discussion). All further analyses were performed on

For peer review only

For peer review only - http://bmjopen.bmj.com/site/about/guidelines.xhtml

these top 28 blocks (T-28). The use of (first) hospitalisation for a disease as our definition of incidence is imperfect (e.g. for Alzheimer's disease where hospitalisation following incidence is rare or delayed, and even first diagnosis in the community will often be preceded by a long latent period)(15).

Disease survival

Improvements in disease outcomes by ICD10 block were calculated by comparing 5-year all-cause mortality (Equation 1) following hospitalisation in 2001 with 5-year all-cause mortality following hospitalisation in 2011. We calculated 5-year mortality following first-time hospitalisation every year from 2001 to 2011 (i.e. mortality of patients admitted in that year) and fitted a 3rd order polynomial regression to these estimates to calculate the underlying change in mortality following hospitalisation between 2001 and 2011.

Mortality estimates from morbidity

Estimates of the improvement in incidence of hospitalisation between decades of birth was combined into an overall improvement by performing a weighted sum of all diseases, with weights derived from the relative burden of death of each disease (see above).

Ethical approval

This study was approved by the Public Benefit and Privacy Panel for Health and Social Care under application number 1617-0255/Joshi. As clinical records are provided without explicit patient consent, the panel requires the public benefit of the research to clearly outweigh any impact on individual patient privacy, and appropriate safeguards and security to be in place to protect patients. The panel granted access to de-identified patient data, accessible only through the National Safe Haven and only by University of Edinburgh researchers with data safeguarding qualifications. In addition, all results were reviewed by eDRIS before extraction from the safe haven to ensure no potentially identifiable information was made public.

Patient and public involvement

Patients and the public were not involved in the study or its design, beyond their contribution of health records. Due to the retrospective study design and anonymised nature of the records, it was not feasible to contact individual patients nor involve them in the dissemination of results.

For peer review only

Results

Mortality

The population consisted of 1,967,130 Scottish individuals aged 35 years or older at the start of the study period (1 December 2000). 53.3% were female, 78.5% had been admitted to hospital at least once within the study period, and 30.6% died over the course of the study (31 January 2016). See Table 1 for detailed population characteristics.

		N			Age Entry			Age Exit			Hospital Visits		
Sex	Dead	Individuals	Admitted	Hospital Visits	Mean	Median	SD	Mean	Median	SD	Mean	Median	SD
Male	FALSE	633,953	429,659	2,315,915	50.9	49.4	10.3	65.6	64.3	9.8	3.7	2.0	6.8
Male	TRUE	283,835	283,835	2,742,686	68.0	69.3	11.7	75.6	77.1	11.4	9.7	7.0	11.4
Female	FALSE	730,671	511,632	2,764,040	52.7	51.1	11.6	67.4	66.1	10.8	3.8	2.0	7.0
Female	TRUE	318,671	318,671	2,895,443	72.1	73.8	12.0	79.7	81.7	11.5	9.1	6.0	11.0
Both	FALSE	1,364,624	941,291	5,079,955	51.9	50.3	11.1	66.6	65.3	10.4	3.7	2.0	6.9
Both	TRUE	602,506	602,506	5,638,129	70.1	71.7	12.0	77.8	79.5	11.6	9.4	6.0	11.2
Both	ALL	1,967,130	1,543,797	10,718,084	57.5	55.4	14.1	70.0	69.2	11.9	5.4	3.0	8.8

Table 1: Description of the data. The population included almost 2 million individuals (one-third of whom died during the study). See Table 1—Source Data 1 for descriptives by deprivation including ICD10 codes. N – Number of records; Admitted – Individuals admitted to hospital at least once; Hospital Visits – All records of visiting the hospital; Age Entry – Age at the start of the study period (1 December 2000); Age Exit – Age at the end of the study period (31 January 2016) or at the end of life.

Quantifying mortality effects using Cox proportional hazard models, we observed statistically significant associations ($P < 1x10^{-26}$) between mortality and sex, deprivation and decade of birth (S Figure 2). Women showed lower overall age-adjusted mortality rates compared to men (70.7%; 95% CI 70.3%–71.1%), corresponding to an expectation of life of 3.5 years longer than their male counterparts, whilst individuals from the most deprived areas (top decile) suffered mortality rates more than twice as severe (207%; 95% CI 204%–209%) as those from the least deprived areas (bottom decile), corresponding to a difference in around 7 years of life. Median survival of men and women in the most deprived areas was 71.1 and 76.6 years, respectively, compared to 82.2 and 85.2 in the least deprived areas. A wide gap between the most deprived decile and the adjacent one for men is apparent visually: the difference in median survival between deprivation deciles 1 to 9 is roughly constant (0.82/1.05 years per decile for women/men), but moving from the 9th to 10th deprivation decile has a greater effect, especially for men (1.99/2.67 years for women/men). Lastly, individuals born in the decade commencing 1935 had age-adjusted mortality rates 245% (95% CI 239%–251%) of those born three decades later, corresponding to a difference in life expectancy of around 9 years of life.

For peer review only - http://bmjopen.bmj.com/site/about/guidelines.xhtml

For peer review only

Morbidities and consequent mortality

Multiplying total number of hospitalisations during the study period (as a proxy for disease prevalence) by 5-year mortality after hospital admission (as a proxy for disease severity) provided a weight for the death burden of hospitalisation of each ICD10 block. We restricted our analyses to 28 of the top disease blocks for burden of death (T-28, see methods). Among the T-28, total cases of disease incidence (i.e. first-time admissions) during the study period ranged from 33,613 (A30-A49, Other bacterial disease) to 225,504 (R00-R09, Symptoms and signs involving the circulatory and respiratory systems) (Table 2). Per-person total cases of disease incidence (not age-adjusted) were 68.0% higher for the most deprived decile (188,905 individuals with 331,701 first-time admissions) compared to the least deprived decile (187,193 individuals with 195,617 first-time admissions). Between sexes, per-person incidence was 2.2% higher for men (917,788 individuals with 1,257,417 first-time admissions) compared to women (1,049,342 individuals with 1,407,223 first-time admissions) (Table 2—Source Data 1). In the first five years, all-cause mortality HRs ranged from 21 for patients admitted for C76-C80 (Malignant neoplasms of ill defined, secondary and unspecified sites; 23,490 cases) compared to all-cause mortality for those not admitted for C76-C80, to 1.8 for those admitted for K20-K31 (Diseases of oesophagus, stomach and duodenum; 128,000 cases) compared to all-cause mortality for those not admitted for K20-K31 during the study period.

For peer review only - http://bmjopen.bmj.com/site/about/guidelines.xhtml

ICD10	Disease	Hospital Visits	Mortality	Weight	Incidence	Survival	Combined	Ratio
J09-J18	Influenza and pneumonia	110,985	1.66	0.068	-0.17 (0.03)	0.15 (0.04)	-0.02 (0.05)	0.47 (0.13)
R00-R09	Symptoms and signs involving the circulatory and respiratory systems	225,504	0.73	0.061	0.13 (0.02)	0.23 (0.04)	0.36 (0.05)	0.65 (0.14)
C30-C39	Malignant neoplasm of respiratory and intrathoracic organs	54,178	3.05	0.061	0.19 (0.05)	0.21 (0.05)	0.40 (0.07)	0.52 (0.15)
R10-R19	Symptoms and signs involving the digestive system and abdomen	174,055	0.94	0.060	0.14 (0.02)	0.12 (0.04)	0.26 (0.05)	0.45 (0.19)
R50-R69	General symptoms and signs	157,357	0.98	0.057	0.11 (0.03)	0.03 (0.03)	0.14 (0.04)	0.19 (0.19)
C15-C26	Malignant neoplasms of digestive organs	71,981	2.10	0.056	0.24 (0.04)	0.40 (0.04)	0.63 (0.05)	0.63 (0.08)
I30-I52	Other forms of heart disease	142,898	0.99	0.052	0.24 (0.03)	0.18 (0.02)	0.42 (0.04)	0.43 (0.06)
C76-C80	Malignant neoplasms of ill defined, secondary and unspecified sites	39,339	3.26	0.047	0.17 (0.05)	0.37 (0.07)	0.54 (0.08)	0.68 (0.16)
Z40-Z54	Persons encountering health services for specific procedures and health care	157,841	0.76	0.044	0.00 (0.02)	0.30 (0.04)	0.30 (0.04)	1.00 (0.19)
I60-I69	Cerebrovascular diseases	100,907	1.12	0.042	0.23 (0.04)	0.20 (0.03)	0.44 (0.05)	0.46 (0.09)
K55-K63	Other diseases of intestines	206,178	0.54	0.041	0.06 (0.02)	0.22 (0.02)	0.28 (0.03)	0.80 (0.12)
J40-J47	Chronic lower respiratory diseases	78,467	1.38	0.040	0.25 (0.04)	0.24 (0.05)	0.48 (0.06)	0.49 (0.11)
I20-I25	Ischaemic heart diseases	175,605	0.61	0.039	0.42 (0.02)	0.26 (0.03)	0.69 (0.04)	0.38 (0.05)
N30-N39	Other diseases of the urinary system	126,329	0.84	0.039	-0.04 (0.03)	-0.21 (0.04)	-0.26 (0.05)	0.84 (0.22)
K20-K31	Diseases of oesophagus, stomach and duodenum	172,206	0.60	0.038	0.33 (0.02)	0.13 (0.05)	0.46 (0.05)	0.29 (0.11)
J20-J22	Other acute lower respiratory infections	77,520	1.27	0.036	-0.11 (0.04)	-0.11 (0.02)	-0.22 (0.05)	0.50 (0.15)
S70-S79	Injuries to the hip and thigh	78,231	0.97	0.028	0.14 (0.05)	-0.02 (0.02)	0.12 (0.06)	0.12 (0.14)
A30-A49	Other bacterial diseases	33,613	1.89	0.023	-0.44 (0.06)	0.06 (0.10)	-0.38 (0.12)	0.13 (0.21)
T80-T88	Complications of surgical and medical care, not elsewhere classified	75,217	0.84	0.023	-0.04 (0.03)	0.18 (0.06)	0.15 (0.07)	0.84 (0.40)
N17-N19	Renal failure	37,213	1.64	0.022	-0.02 (0.06)	0.20 (0.06)	0.18 (0.08)	0.90 (0.42)
I80-I89	Diseases of veins, lymphatic vessels and lymph nodes, not elsewhere classified	84,073	0.66	0.021	0.22 (0.03)	0.24 (0.05)	0.46 (0.06)	0.52 (0.12)
K90-K93	Other diseases of the digestive system	47,091	1.09	0.019	0.02 (0.04)	0.19 (0.07)	0.20 (0.08)	0.91 (0.50)
I70-I79	Diseases of arteries, arterioles and capillaries	47,410	1.08	0.019	0.40 (0.05)	0.09 (0.04)	0.49 (0.06)	0.19 (0.08)
K50-K52	Non infective enteritis and colitis	59,183	0.82	0.018	0.31 (0.04)	0.12 (0.03)	0.42 (0.05)	0.27 (0.08)
S00-S09	Injuries to the head	64,925	0.74	0.018	0.05 (0.04)	0.02 (0.05)	0.07 (0.06)	0.22 (0.72)
C50-C50	Malignant neoplasm of breast	39,358	1.17	0.017	0.21 (0.05)	1.16 (0.07)	1.37 (0.09)	0.85 (0.07)
C60-C63	Malignant neoplasms of male genital organs	22,312	1.17	0.010	0.09 (0.08)	0.70 (0.07)	0.79 (0.10)	0.88 (0.14)
G30-G32	Other degenerative diseases of the central nervous system	4,655	1.19	0.002	0.29 (0.20)	0.04 (0.14)	0.25 (0.23)	0.11 (0.44)
TOTAL		2,664,631	1.02	1.000	0.12 (0.01)	0.19 (0.04)	0.30 (0.01)	0.61 (0.16)

Table 2: Relative mortality burden of hospital admission by disease grouping and improvements in hospitalisation incidence and survival. ICD10 – Diseases contained within the disease grouping, coded by International Classification of Disease Codes, Tenth Revision. See Table 1—Source Data 2 for counts of 3-letter ICD10 records within each ICD10 block. Disease – Description of the diseases within the disease grouping. Hospital visits – Number of first-time admissions with main diagnosis falling within the disease block. Mortality – Mortality compared to individuals who had not yet or ever been admitted for the disease group (in lnHR). Weight – Relative burden of death, calculated as hospital admissions multiplied by the mortality in the first five years after admission and scaled to [0-1]. Incidence – Improvements in disease incidence as captured by mean differences in hospital admissions between decades of birth (lnHR), with negative numbers indicating a deterioration. Survival – Improvements in 5-year mortality hazard following admission between 2001 and 2011 (lnHR). Combined – linear combination of improvements in disease incidence and survival. Ratio – the ratio of improvements in disease survival to incidence of hospital admission. Standard errors are listed in parentheses. See Table 2—Source Data 1 for these data by sex and deprivation. See Table 2—Source Data 2 for the relative burdens of all disease groupings with more than 15,000 first-time hospital admissions.

For peer review only - http://bmjopen.bmj.com/site/about/guidelines.xhtml

Apart from sex-specific cancers, we observe significant differences in burden of death between men and women for injuries to the hip and thigh (S70-S79) and head (S00-S09), with the former having a higher burden in women due to more female cases and the latter having a higher burden in men due to more male cases. For both disease blocks, the effect of hospitalisation on subsequent mortality is greater in men than women (S70-S79 HR men: 3.19, women 2.44; S00-S09 HR men: 2.32, women 1.88 HR). Strikingly, 5-year mortality after hospital admission for IHD is higher for women (HR 2.01/1.70 women/men), but this is offset by the lower prevalence of hospitalisation in women (Table 2—Source Data 1).

Trends in disease

To understand changes in disease survival rates, we next modelled the effects of a disease on all-cause mortality by year of hospital admission for admissions between 2001 and 2011 and 5-year survival subsequent to admission. We find an overall improvement over time in patient survival following hospitalisation, with a median decline between 2001 and 2011 in the 5-year HR of 16.8% for admitted cases across the T-28 diseases. The biggest improvements were for malignant neoplasms of the breast (C50) and male genital organs (C60-C63), which have seen 68.7% (95% CI 64.1%–72.7%) and 50.2% (95% CI 42.9%–56.6%) declines in the 5-year hazard ratio between 2001 and 2011 mortality, respectively. On the other hand, other acute lower respiratory infections (J20-J22) and other diseases of the urinary system (N30-N39) have seen increases in mortality hazard of 11.3% (95% CI 7.0%–15.7%) and 24.0% (95% CI 14.6%–34.1%), respectively (Table 2, S Figure 3).

We next modelled age-adjusted incidence of hospitalisation for a disease by birth decade, under the simplified model that incidence is a cohort rather than period effect – essentially modelling that current incidence is the effect of (previous) lifetime exposures, rather than current exposures. We find disease incidence has fallen decade on decade of birth for cancers, cardiovascular, and intestinal diseases, but this improvement appears to have slowed down in the last decade of birth (1955-1965) considered. Age-adjusted incidence of influenza and pneumonia (J09-J18) and other bacterial diseases (A30-A49) has worsened by decade on decade of birth, over the whole range of births considered (S Figure 4).

For peer review only

For peer review only - http://bmjopen.bmj.com/site/about/guidelines.xhtml

When taking both trends in incidence and survival into account – adding 1) the average age-adjusted incidence rate reductions between decade on decade of birth to 2) the 2001-2011 reductions in 5-year disease mortality – we observe the death burden of cancers is declining most (Figure 1). Notably, breast and prostate cancers have seen the largest improvement of all disease categories in the last decade. Other diseases of the urinary system (N30-N39), other bacterial diseases (A30-A49), other acute lower respiratory infections (J20-J22), and influenza and pneumonia (J09-J18) have all seen increases in their effect on age-adjusted all-cause mortality.

Overall, we see broad consistency in the scale of improvements across decades of birth, except for malignant neoplasms of respiratory and thoracic organs (C30-C39), where we see greater decade-on-decade improvements amongst later decades (Figure 2). Averaging these individual disease effects on death, using burden of death weightings, we can then compare the modelled death rates with those observed, and see broad correspondence, with the 1935 and 1945 decades, showing the greatest improvements. Overall, our morbidity model suggests an average decade of birth on decade of birth lnHR of death of 0.30 (~3 years of life) (Table 2).

The shape of disease modelled mortality improvements by decade of birth broadly tracks the observed changes. Furthermore, when stratified by sex a similar effect is seen: apparent distinctions in projected morbidity driven mortality are associated with distinctions with observed mortality (correlation 0.89); i.e. mortality trends in the study can largely be explained by trends in disease incidence and survival (S Table 1; S Figure 6). Across sex and deprivation strata, taking into account disease survival improvements between 2001 and 2011 and all improvements in disease incidence between decades of birth, we find the largest reductions in death are due to improvements in ischaemic heart diseases (I20-I25), malignant neoplasms of digestive organs (C15-C26), and malignant neoplasm of respiratory and intrathoracic organs (C30-C39), while the largest increases in death are due to other bacterial diseases (A30-A49) and influenza and pneumonia (J09-J18). In addition, the deterioration in other diseases of the urinary system (N30-N39) morbidity shows a consistent increase with

For peer review only - http://bmjopen.bmj.com/site/about/guidelines.xhtml

deprivation, while other diseases of the digestive system (K90-K93) shows consistently larger improvements in more deprived classes (Table 2—Source Data 1).

Overall, we estimate 61.2% (95% CI 29.9%–92.6%) of the improvement in mortality rates was due to improvements in survival following hospital admission, with the balance arising from reduced (age-adjusted) admission rates (Table 2). Improved outcomes for cancers were particularly driven by post-admission survival, especially C60-C63 (88% of mortality improvement attributable to survival rather than incidence), C50 (85%) and C76-80 (68%), whereas for cardiovascular diseases, the balance was more even I80-89 (52%), I60-I69 (46%), I30-I32 (43%), I20-I25 (38%).

As previously noted, disease severity was defined as the log hazard ratio for subsequent all-cause mortality among those with a previous admission for an index group of conditions compared with those with no such admission. We regarded the rate of improvement in disease severity over time as being constant if there was the same relative fall in log hazard rate over successive time periods (so for example we regarded a fall in lnHR from 0.6 to 0.3 as equivalent to a fall from 0.3 to 0.15). Assuming the improvements in survival following hospitalisation continue for the coming decade, and differences between incidence in birth cohorts remains the same, we project a 21% slowing of improvements in mortality (-0.242 lnHR c.f. -0.305 lnHR; Table 3). Essentially, at least arithmetically, the population mortality benefits from improved cancer treatments in 2001-2011 will be hard to repeat as so much benefit has already accrued. Admittedly, this is a consequence of our model: essentially judging it equally difficult to reduce 50 excess deaths following cancer hospital admission associated to 25, as it was to reduce from 100 to 50, and as such should be considered speculative. On the other hand - our model is clearly valid *in extremis*: if all excess cancer deaths were eliminated, no further cancer driven improvement in mortality would be possible.

For peer review only

		Current Improvements			Projected Improvements		
Stratified	Group	Hospital Admission Rate (lnHR)	Five Year Mortality After Admission (lnHR)	Combined (lnHR)	Hospital Admission Rate (lnHR)	Five Year Mortality After Admission (lnHR)	Combined (lnHR)
None		-0.1182	-0.1866	-0.3047	-0.1182	-0.1235	-0.2418
Sex	M	-0.1428	-0.1913	-0.3340	-0.1428	-0.1273	-0.2701
Sex	F	-0.0971	-0.1823	-0.2794	-0.0971	-0.1130	-0.2101
SIMD	1	-0.1154	-0.2204	-0.3360	-0.1154	-0.1280	-0.2433
SIMD	2	-0.1157	-0.1592	-0.2743	-0.1157	-0.0456	-0.1610
SIMD	3	-0.1202	-0.1186	-0.2392	-0.1202	-0.0370	-0.1572
SIMD	4	-0.1418	-0.1759	-0.3201	-0.1418	-0.0541	-0.1982
SIMD	5	-0.1231	-0.1731	-0.2958	-0.1231	-0.0914	-0.2145
SIMD	6	-0.1305	-0.1928	-0.3227	-0.1305	-0.0998	-0.2303
SIMD	7	-0.1233	-0.1787	-0.3044	-0.1233	-0.1109	-0.2343
SIMD	8	-0.1305	-0.2097	-0.3402	-0.1305	-0.1422	-0.2726
SIMD	9	-0.0926	-0.1619	-0.2546	-0.0926	-0.1033	-0.1959
SIMD	10	-0.0950	-0.1970	-0.2920	-0.0950	-0.1092	-0.2043

Table 3: Mean (over birth decades) decade of birth on decade of birth improvements in morbidity for the study period, and projections into the subsequent decade by sex and deprivation. Mortality improvements were estimated from morbidity records by combining the mean improvement in hospitalisation rate across birth cohorts and the improvement in disease severity between 2001 and 2011. This was then projected forward assuming improvements in age-adjusted hospitalisation rate between birth cohorts remained constant and improvements in severity remained proportional to the (now reduced) overall mortality of the disease group.

For peer review only

Discussion

In a study of 1,967,120 lives and 10,718,084 hospital admissions, we observed a median age at death of 82.2/85.2 for men/women in the highest socioeconomic decile, and 11.1/8.6 years less for the lowest decile. Cancers (C), cardiovascular disease (I), respiratory diseases (J), and unclassified symptoms and signs (R) were the principal ICD10 chapters recurring in the top 28 disease blocks where hospital admission was associated with the greatest subsequent all-cause mortality, which was a product of the rate of first hospital admission with group of conditions and of all-cause mortality in the five years following admission with that condition. Specifically, our top five causes of hospitalisations associated with subsequent burden of all-cause deaths were, in descending order, 'Influenza and pneumonia' (more common and with higher subsequent mortality than the average T-28 disease), 'Symptoms and signs involving the circulatory and respiratory systems' (common), 'Malignant neoplasm of respiratory and intrathoracic organs' (higher mortality) ,'Symptoms and signs involving the digestive system and abdomen' (common), and 'General symptoms and signs' (common and higher mortality). Whilst the latter might appear a benign diagnosis, our results suggest it is a fairly strong and frequent marker of subsequent all-cause mortality.

Across decades of birth, we modelled a reduction in mortality hazard of 0.737 (95% CI 0.730–0.745) due to improvements in morbidity, which broadly tracked improvements in observed mortality. The modelled improvement was 61% accounted for by reduction in excess mortality subsequent to admission and 39% accounted for by a fall in incidences of disease (as measured by hospital admission rates). The important (i.e. burden-of-death weighted) improvements in incidence were driven by cancers and heart disease, whilst improvement in outcomes following admission were mostly driven by cancer, particularly breast and prostate cancer. In contrast, we found deteriorations in the incidence of bacterial disease and in mortality following admission for respiratory and urinary infections. Levels of morbidity and mortality varied strongly across socioeconomic groups, but patterns in **changes** of such were generally less apparent. Men showed greater rates of improvement in mortality and morbidity than women, with lung and throat cancers contributing most to male improvements and IHD contributing most to female improvements.

For peer review only

For peer review only - http://bmjopen.bmj.com/site/about/guidelines.xhtml

In conclusion, we find trends in morbidity appear to partly explain trends in mortality. The progress in prevention and cure within oncology and prevention of heart disease account for the greatest parts of mortality improvement in 2001-2016, and our model suggests mortality improvements may slow, simply because the absolute effect of progress in treatment of these diseases will be difficult to repeat. However, there is scope for further improvements in life expectancy, especially if new progress is made in the treatment of other diseases associated with death, or if prevention initiatives accelerate.

Strengths and weaknesses of the study

This study has avoided some of the known issues with cause of death recording(16) since it does not use cause-specific mortality and tracks wider disease effects and subsequent mortality (such as frailty) beyond direct causes of death, by combining hospitalisation and death records. Implicit tracking of underlying causes through an associated effect (admission to hospital for a disease) may improve estimates of trends in mortality, even if the underlying cause is obscure. We are also able to partition trends in deaths due to a disease based on trends in prevalence and incidence, which has been done for IHD(17), but not simultaneously across diseases in the same dataset. Also, our results are unaffected by population shifts as we excluded immigrants into Scotland after 2001, and instead reflect trends within the defined groups. Combined with the scale of our data, this consistent tracking has enabled us to make like-for-like comparisons of the mortality outcomes of different disease classes across socioeconomic groups and their trends over time.

However, this study also has a number of limitations. Whilst necessary given the data structure, excluding all migrants during the study period will in many cases have excluded healthy people moving for economic or lifestyle opportunity. When health status influences the propensity to migrate, it forms a competing risk for both mortality and morbidity, which can bias results(18). Nevertheless, as we are principally concerned in the trend of morbidity and mortality, only changes in migration biases – not their absolute level – should affect our results materially.

For peer review only - http://bmjopen.bmj.com/site/about/guidelines.xhtml

Secondly, we used recorded main position coding of first hospital admission of a disease block as the index event. Hospital admission is only a proxy for the incidence of severe disease, and likely works better for chronic conditions like breast cancer where there is a single disease process that can result in a number of hospital admissions and may result in mortality in the timeframe of the study. It works perhaps less well for acute conditions, such as influenza, which can have distinct occurrences over the study period, and it fails to capture chronic conditions which are managed in general practice or the community but eventually lead to death, such as dementia and multiple sclerosis. Also, association between hospital admission for a condition and the latter occurrence of death does not necessarily imply causation, and may be confounded by other health risks, such as socioeconomic status, a second disease, or behavioural factors, although our analysis stratified by socioeconomic status may partly mitigate these effects. Modelling comorbidities simultaneously or modelling the causal effects of one morbidity on another and consequently on death has been beyond the scope of this study. In addition, our model assumed that (age-adjusted) incidence is a function of year of birth, but that mortality post-incidence is a function of the year of incidence. This is clearly an oversimplification, but necessary given that year of birth and year of incidence are completely confounded for a given age at incidence.

By relying on routine data, we have been able to create a very large dataset, at the expense of being unable to externally check individual fields, such as primary diagnosis, where there are known to be inaccuracies(19). However, our conclusions will be less affected if these inaccuracies are reasonably stable over time. Furthermore, we have used a simple definition of burden of disease: the total number of cases multiplied by the excess hazard across all ages. More complex definitions might have looked at Years of Life Lost (YLL) and taken into account any age-related pattern in incidence. However, our measure is simple and is predominantly used to weight the diseases and especially their improvements to calculate an overall improvement in all-disease impact, rather than as an absolute measure of burden.

Lastly, our model assumed disease incidence and survival hazards were proportionate. Incidence of hospital admission and death used "age" as the baseline hazard determinant, while mortality following admission used "time since admission". In practice we do not expect

For peer review only - http://bmjopen.bmj.com/site/about/guidelines.xhtml

the risks analysed to be truly proportionate, nonetheless we consider the analysis of trends to be revealing. Conversely, recently doubt was cast on the effect of using decade of birth groupings in a standardised mortality analysis(20) due to changing patterns of births within the decade: that concern does not apply here as the Cox baseline hazard used full (not rounded) age information, with decade of birth fitted as a covariate.

Changes in hospital admission policy have likely affected changes in observed hospitalisation rate. Conversely, survival rates could be influenced if for example, only more serious cases were admitted. One example from our study is the observed increase in incidence of influenza and pneumonia, which was offset by increased survival and may therefore reflect changes in coding practices and/or frequency of referral(21). Some degree of caution is needed in observing the changes in morbidity, although such biases will have tended to offset each other in terms of the overall change in the mortality arising from morbidity for such a disease block. However, clearly the partition of effect between incidence and survival can be affected.

Strengths and weaknesses in relation to other studies

There was a degree of correspondence in the principal burdens assessed here and a recent study by the Scottish Burden of Disease study (SBD)(22). This study used the same population and the same study period but assessed YLL (weighting young deaths more as opposed to our method which counted all deaths equally), included individuals younger than 35 years old, and used different disease groupings. Their principal burdens were IHD (ranked 13th in our list of burdens), tracheal, bronchus and lung cancers (3rd), chronic obstructive pulmonary disease (12th), stroke (10th), and Alzheimer's disease (–). Aside from Alzheimer's disease, discussed below, much of the distinction appears to arise from our observation of an association between death and admissions with indistinct diagnosis (not considered a valid specific cause of death by SBD). In the case of influenza and pneumonia, differences arise due to our study identifying a marker of frailty as well as a direct cause of death, combined with SBD grouping influenza and pneumonia under lower respiratory infections. A relative strength of our study stems from usage of incident morbidity (as marked by hospitalisation) in advance of death, based on recorded diagnosis at the time of hospital visit, thus tracking remote effects such as long term frailty rather than cause of death (which has known limited accuracy, particularly at older ages(16)). However, the principal strength arises from the ability

For peer review only - http://bmjopen.bmj.com/site/about/guidelines.xhtml

to distinguish trends in incidence of morbidity from trends in subsequent survival. On the other hand, a relative weakness is that we are reliant on hospital admission as a marker of incidence; therefore, diagnosed or latent (presumably milder) cases in the absence of admission are not visible to us, leading for example to significant discrepancy with SBD in the apparent relative burden of Alzheimer's disease, likely due to an understatement of its importance in our results.

The closing gap in mortality between the sexes and its widening across social classes observed in our study is consistent with recent findings from the Office of National Statistics, summarised by Torjesen(23), which looked at socioeconomic deprivation in England and Wales. Similarly, a recent study of health inequality in England found rising levels of lifespan inequality across socioeconomic groupings arising from increasing inequalities across a broad span of causes of death(24). These studies had the advantage of a larger sample size (~7.5 million deaths cf. 600,000 in our study) and could therefore track trends in mortality and cause of death between stratified groups more accurately. However, Scotland's unique linkage of death records and electronic health records through eDRIS allowed us to directly model changes disease mortality at an individual level (avoiding issues with cause of death recordings and shifts in population demographics). Our study has the advantage of partly explaining these trends in mortality inequality through changes in disease incidence and survival: men experienced greater improvements in incidence of lung cancer and survival following heart disease hospitalisation compared to women, while more socially deprived individuals (men and women combined) suffered worse deteriorations in infectious disease, especially for the incidence and survival of hospitalisation for urinary tract infections. However, in contrast to Bennet *et al.*(24), we do not find a clear pattern in overall morbidity improvements across socioeconomic deciles in Scotland, and we do not observe a widening inequality in cancer, respiratory and Alzheimer's disease morbidity within our study population, although we are underpowered to detect the latter and our disease groupings were not identical.

Lastly, a recent study of coronary heart disease mortality in Scotland, using a sophisticated model to apportion improvement between prevention and treatment, found improvements for coronary heart disease between 2001-2010 were similar across social classes, and reported 33%–61% of these improvements could be attributed to advances in treatment(17). Given the

For peer review only

For peer review only - http://bmjopen.bmj.com/site/about/guidelines.xhtml

very different methods, albeit studying the same population, there is reasonable concordance with our own study: we find roughly equal improvements in heart disease across social classes and estimate 38% (95% CI 28%–48%) of these improvements stem from increased survival after hospitalisation for ischaemic heart disease. Hotchkiss *et al.*(17) are able to further partition improvements by uptake of primary and secondary prevention drugs and treatments. Such detailed analysis of specific diseases has been beyond the scope of our study.

Implications for clinicians and policymakers

Much of the improvements in mortality observed in Scotland between 2001–2016 can be attributed to reductions in morbidity, as captured by hospital admissions. While this study examined mortality and morbidity in the Scottish population only, there is a substantial concordance in mortality trends across high-income countries(7), as well as similarities in disease-related mortality trends between Scotland and the rest of the UK(6), warranting similar studies to be performed in other high-income countries. It is a testament to healthcare services that the majority of mortality improvements appear to stem from advances in disease survival post-admission. Observed improvements in cancer incidence and survival – especially breast and prostate – coincide with a continued effort within Scotland(25), the UK(26), and other high-income nations(27) to improve prevention and care of these diseases. However, the rapid advances in survival of both heart disease and cancer modelled by our study between 2001 and 2011 will be hard to continue to the same extent, as so much progress has already been made. At the same time, the observed deteriorations in infectious disease coincide with global increases in antimicrobial resistance(28) and emphasise the need to prioritise research in this area: infectious disease will become a larger contributor to mortality and may contribute to a widening of health inequalities between socioeconomic classes. If these current trends in morbidity continue, we expect morbidity-driven improvements in mortality to slow down. However, the life expectancy gap between Scotland and other high-income countries(29) suggests further mortality improvements are possible. The rate of this improvement will hinge upon whether advances in all major diseases categories – especially infectious disease – can catch up with the progress we have recently seen on heart disease and cancer, and whether preventable deaths from external causes (such as suicide and drug-related deaths), which cannot be accurately tracked using hospital admissions, decrease rather than rise.

For peer review only

For peer review only - http://bmjopen.bmj.com/site/about/guidelines.xhtml

Funding

The study was funded by the Lloyds Banking Group, for the creation, curation and dissemination of knowledge in the public interest, in particular to improve estimates of future population size and morbidity and mortality rates to facilitate healthcare and other government planning. All analyses stratified by socioeconomic deprivation (Table 2, Table 3, Supplementary Table 1, and Supplementary Data Files 1-5) were confidentially re-analysed using 10 socioeconomic groups specified by Lloyds, rather than the Scottish Index of Multiple Deprivation. No Lloyds employees were granted access to individual patient data. Data access was granted to University of Edinburgh researchers only and only through the national safe haven by the Public Benefit and Privacy Panel for Health and Social Care under application number 1617-0255/Joshi.

The study and its design were conceived by the last author. The funder reviewed the design and said that adding stratification by socioeconomic status was a key requirement for meaningful analysis and their funding was conditional upon this, a request to which the authors readily agreed. The funder was kept informed of interim analyses and reviewed the draft manuscript, occasionally suggesting additional analyses or requesting clarifications. The funders were not permitted to and did not request the removal of any results.

The last author and authors affiliated with eDRIS and NHS Scotland were compensated by the University of Edinburgh using the grant from Lloyds Banking Group, in line with normal pricing for the work undertaken. PRHJT was funded by the MRC Doctoral Training Programme (MR/N013166/1) and the University of Edinburgh College of Medicine and Veterinary Medicine. JFW was funded by the MRC QTL in health and disease. Apart from Lloyds Banking Group, the funders had no role in the study design, data collection, analysis, and interpretation, or the decision to submit the work for publication.

Acknowledgements

We would like to thank Lloyds for the funding and in particular Craig Butler and Stuart McDonald for their engagement with the project. We would also like to thank Steve Pavis and

For peer review only - http://bmjopen.bmj.com/site/about/guidelines.xhtml

Doug Kidd for their guidance with the Public Benefit and Privacy Panel for Health and Social Care.

Competing interests

PKJ reports grants from Lloyds Banking Group, PLC, during the conduct of the study. This grant was awarded to the University of Edinburgh and used in part to pay for research costs of JJK and CMF. PKJ also reports shares in Lloyds Banking Group, PLC, as part of a diversified portfolio. The remaining authors declare no competing interests.

Author contributions

PRHJT—Formal Analysis, Software, Visualisation, Writing - Original Draft, Writing - Review & Editing. JJK—Data Curation, Writing - Original Draft. JWM – Writing - Review & Editing. IG—Writing - Review & Editing. JFW—Supervision, Writing - Review & Editing. HC—Writing - Review & Editing. CMF—Conceptualization, Writing - Review & Editing. PJK—Conceptualization, Formal Analysis, Funding Acquisition, Investigation, Methodology, Project Administration, Software, Supervision, Validation, Writing - Original Draft, Writing - Review & Editing.

Data sharing

Individual de-identified participant data cannot be shared. Statistical code and technical details are available upon request from the corresponding author.

For peer review only - http://bmjopen.bmj.com/site/about/guidelines.xhtml

References

1. Sanders S. National life tables, UK: 2015 to 2017. Off Natl Stat Stat Bull. 2018;1–11.

2. Lafortune, Gaetan; Balestat G. OECD Health Working Papers No.26. Trends Sev Disabil Among Elder People. 2007;(26):81.

3. Hiam L, Dorling D, Harrison D, McKee M. What caused the spike in mortality in England and Wales in January 2015? J R Soc Med. 2017;110(4):131–7.

4. Office for National Statistics. Changing trends in mortality in England and Wales:1990 to 2017 (Experimental Statistics). 2018;2017(June):1–24.

5. Dyer O. US life expectancy falls for third year in a row. BMJ. 2018;363(December):k5118.

6. Palin J. Mortality improvements in decline. The Actuary. 2017 Aug;

7. Fenton L, Minton J, Ramsay J, Kaye-Bardgett M, Fischbacher C, Wyper GM, et al. Recent adverse mortality trends in Scotland: comparison with other high-income countries. bioRxiv. 2019;542449.

8. Fenton L, Wyper G, McCartney G, Minton J. Socioeconomic inequality in recent adverse mortality trends in Scotland. bioRxiv. 2019;542472.

9. Nie X, Peng X. The reporting of studies conducted using observational routinely-collected health data (RECORD) statement. Chinese J Evidence-Based Med. 2017;17(4):475–87.

10. Fleming M, Kirby B, Penny KI. Record linkage in Scotland and its applications to health research. J Clin Nurs. 2012;21(19–20):2711–21.

11. The Scottish Government. SIMD16 Technical Notes. 2016;

12. World Health Organisation. International Statistical Classification of Diseases and Related Health Problems Tenth Revision Volume 2. World Heal Organ. 2004;

13. Joshi PK, Pirastu N, Kentistou KA, Fischer K, Hofer E, Schraut KE, et al. Genome-wide meta-analysis associates HLA-DQA1/DRB1 and LPA and lifestyle factors with human longevity. Nat Commun. 2017;8(1).

14. Cox DR. Regression Models and Life-Tables. J R Stat Soc Ser B Stat Methodol. 1972;34:187.

For peer review only - http://bmjopen.bmj.com/site/about/guidelines.xhtml

15. Duboisa B, Padovanib A, Scheltensc P, Rossid A, Agnello GD. Timely diagnosis for alzheimer's disease: A literature review on benefits and challenges. J Alzheimer's Dis. 2015;49(3):617–31.

16. Smith Sehdev AE, Hutchins GM. Problems with proper completion and accuracy of the COD statement. Arch Intern Med. 2001;161(2):277–84.

17. Hotchkiss JW, Davies CA, Dundas R, Hawkins N, Jhund PS, Scholes S, et al. Explaining trends in Scottish coronary heart disease mortality between 2000 and 2010 using IMPACTSEC model: Retrospective analysis using routine data. BMJ. 2014;348(February):1–15.

18. Andersen PK, Geskus RB, De witte T, Putter H. Competing risks in epidemiology: Possibilities and pitfalls. Int J Epidemiol. 2012;41(3):861–70.

19. ISD Scotland. Assessment of SMR01 Data Scotland 2014-2015. 2012.

20. Minton J, Green M, McCartney G, Shaw R, Vanderbloemen L, Pickett K. Two cheers for a small giant? Why we need better ways of seeing data: A commentary on: "Rising morbidity and mortality in midlife among White non-Hispanic Americans in the 21st century." Int J Epidemiol. 2017;46(1):356–61.

21. Nair H, Watts AT, Williams LJ, Omer SB, Simpson CR, Willocks LJ, et al. Pneumonia hospitalisations in Scotland following the introduction of pneumococcal conjugate vaccination in young children. BMC Infect Dis. 2016;16(1):1–11.

22. Mesalles-Naranjo O, Grant I, Wyper GMA, Stockton D, Dobbie R, McFadden M, et al. Trends and inequalities in the burden of mortality in Scotland 2000–2015. PLoS One. 2018;13(8):1–17.

23. Torjesen I. Inequalities in life expectancy are widening, data confirm. Bmj. 2018;1017(March):k1017.

24. Bennett JE, Pearson-Stuttard J, Kontis V, Capewell S, Wolfe I, Ezzati M. Contributions of diseases and injuries to widening life expectancy inequalities in England from 2001 to 2016: a population-based analysis of vital registration data. Lancet Public Heal. 2018;3(12):e586–97.

25. NHS Scotland. Better Cancer Care: An Action Plan. The Scottish Government. Edinburgh; 2008.

For peer review only - http://bmjopen.bmj.com/site/about/guidelines.xhtml

26. Department of Health. Improving Outcomes : A Strategy for Cancer. 2011;(January):101.

27. Harbeck N, Gnant M. Breast cancer. In: Lancet (London, England). Elsevier; 2017. p. 1134–50.

28. O'Neill J. The Review on Antimicrobial Resistance. Vol. 33, Wellcome Trust & UK Government. 2016.

29. Whyte B. Scottish mortality in a European Context 1950 – 2000. An analysis of comparative mortality trends. Heart Disease. 2006.

For peer review only

For peer review only - http://bmjopen.bmj.com/site/about/guidelines.xhtml

List of Tables and Figures

Figure 1: Modelled decade-of-birth upon previous decade-of-birth hospitalisations and survival show large improvements in cancer survival and heart disease incidence but deteriorations in infectious disease. Bars represent the mean improvements in hospital admission rate across decades of birth (darker bars), added to changes from 2001 to 2011 in 5-year survival rates following hospital admission (lighter bars). Both measures are expressed in age-adjusted lnHR. For definitions of each ICD10 block, see Table 2. Width of the bars represents the relative burden of death of each disease based on total first-time hospital admissions and 5-year mortality; as such, the total area of each bar represents the relative contribution to improvements – or deteriorations – in population mortality. Error bars are standard errors of the Cox model coefficient. G30–G32 had too few hospital admissions to accurately model improvements (Survival: lnHR 0.04, SE 0.14; Hospital admission lnHR 0.29, SE 0.20). Z40-Z54 only showed improvements in survival.

Figure 2: Modelled decade-of-birth upon previous decade-of-birth mortality reductions due to morbidity changes broadly track observed trends in mortality. Panels represent the combined improvements in hospital admission rate for each ICD10 disease block (for definitions see Table 2) and 5-year mortality rates following hospital admission, expressed in age-adjusted lnHR, split by decade of birth - under the model where change in incidence of disease is modelled by decade of birth and added to the survival effect is the change in subsequent 5 year survival rates from incidences in 2001 and 2011. Dots represent the relative contribution of the disease to the overall improvements in morbidity driven mortality. This is summarised by EST-DTH: modelled trend in deaths based on morbidity- grey bars and calculated as the weighted effect of the coloured bars), with larger dots indicating a greater contribution to morbidity improvements. A red circle around the dot indicates a negative contribution (i.e. deterioration). The black bars (ACT-DTH: observed trend in actual deaths) represent the improvements in mortality observed from death records, by decade of birth, for comparison. Diseases have been ordered by their burden of death (Table 2), so smaller bars in early panels may have similar effect on the grey bar average (indicated by the dot size) as larger bars in later panels. See Supplementary Data File 2 for this graph stratified by sex and deprivation.

For peer review only - http://bmjopen.bmj.com/site/about/guidelines.xhtml

Table 1: Description of the data. The population included almost 2 million individuals (one-third of whom died during the study). Over 90% of deceased individuals had a hospital admission record. Median projected age at death (under Kaplan-Meier curves) for males is 71.1 and 82.2, in the most and least deprived areas, respectively (females 76.6 and 85.2). See Table 1—Source Data 1 for descriptives by deprivation including ICD10 codes. N – Number of records; Admitted – Individuals admitted to hospital at least once; Hospital Visits – All records of visiting the hospital; Age Entry – Age at the start of the study period (1 December 2000); Age Exit – Age at the end of the study period (31 January 2016) or at the end of life.

Table 1—Source Data 1: Study descriptives by sex, deprivation, and deceased status (Excel Document). SIMD – Scottish Index of Multiple Deprivation; Age Entry – Age at the start of the study period (1 December 2000); Age Exit – Age at the end of study period (31 January 2016) or at the end of life; Admissions – Records of hospital admission, where Count represents the number of individuals with at least one hospital admission record, Mean/Median/SD represent the mean/median/standard deviation of number of hospital admission records per individual, and Sum represents the total number of admission records during the study period across all individuals; A-Z – First letter of the ICD10 main condition recorded at all hospital visits; SD – Standard Deviation

Table 1—Source Data 2: Count of records within each ICD10 block (Plain Text Document). Main diagnoses of hospital admission records were grouped into ICD 10 chapter. For each ICD 10 chapter, counts of the 3-letter ICD 10 subcategories with 10 or more admissions are reported.

Table 2: Relative mortality burden of hospital admission by disease grouping and improvements in hospitalisation incidence and survival. ICD10 – Diseases contained within the disease grouping, coded by International Classification of Disease Codes, Tenth Revision. See Table 1—Source Data 2 for counts of 3-letter ICD10 records within each ICD10 block. Disease – Description of the diseases within the disease grouping. Hospital visits – Number of first-time admissions with main diagnosis falling within the disease block. Mortality –

For peer review only - http://bmjopen.bmj.com/site/about/guidelines.xhtml

Mortality compared to individuals who had not yet or ever been admitted for the disease group (in lnHR). Weight – Relative burden of death, calculated as hospital admissions multiplied by the mortality in the first five years after admission and scaled to [0-1]. Incidence – Improvements in disease incidence as captured by mean differences in hospital admissions between decades of birth (lnHR), with negative numbers indicating a deterioration. Survival – Improvements in 5-year mortality hazard following admission between 2001–2011 (lnHR). Combined – linear combination of improvements in disease incidence and survival. Ratio – the ratio of improvements in disease survival to incidence of hospital admission. Standard errors are listed in parentheses. See Table 2—Source Data 1 for these data by sex and deprivation. See Table 2—Source Data 2 for the relative burdens of all disease groupings with more than 15,000 first-time hospital admissions.

Table 2—Source Data 1: Disease mortality burden by sex and deprivation (Excel Document). Contains sheets for 1) total incident cases, 2) mortality in the first 5 years following disease admission, 3) mortality after 5 years following disease admission, 4) the ratio of 0-5 and >5 year mortalities, 5) the relative burden of disease, and 6) the weight of each disease, by sex and deprivation. SIMD – Decile of Scottish Index of Multiple Deprivation, from least deprived (1) to most deprived (10).

Table 2—Source Data 2: Relative mortality burden of all disease groupings with at least 15,000 first-time admissions (Excel Document). ICD10 – Diseases contained within the disease grouping, coded by International Classification of Disease Codes, Tenth Revision. See (Table 1—Source Data 2 - Disease Count) for counts of 3-letter ICD10 records within each ICD10 block. Disease – Description of the diseases within the disease grouping. Hospital visits – Number of first-time admissions with main diagnosis falling within the disease block. Mortality – Mortality compared to individuals who had not yet or ever been admitted for the disease group. Relative Burden of Death – Ratio of hospital admissions to total individuals, multiplied by the mortality in the first five years after admission. Weight - burden of death rescaled to a total value of 1.

For peer review only - http://bmjopen.bmj.com/site/about/guidelines.xhtml

Table 3: Mean (over birth decades) decade of birth on decade of birth improvements in morbidity for the study period, and projections into the subsequent decade by sex and deprivation. Mortality improvements were estimated from morbidity records by combining the mean improvement in hospitalisation rate across birth cohorts and the improvement in disease severity between 2001 and 2011. This was then projected forward assuming improvements in age-adjusted hospitalisation rate between birth cohorts remained constant and improvements in severity remained proportional to the (now reduced) overall mortality of the disease group.

For peer review only

For peer review only - http://bmjopen.bmj.com/site/about/guidelines.xhtml

Supplementary Material

S Figure 1: Waterfall chart of individuals excluded from the study during quality control. We started with ~2.7 million primary care records from the NSS Community Health Index (CHI). Records were excluded from individuals with uncommon district postcodes (N < 5000), individuals missing from the CHI database in 2016 (and therefore likely transferred out of Scotland), individuals with record discrepancies between the CHI and National Records of Scotland databases, and individuals with records outside of the study dates or missing information on socioeconomic class.

S Figure 1—Source Data 1: Details of quality control performed on the dataset (Excel Document). Primary care records from the National Health Services Scotland underwent multiple rounds of quality control. For each step, descriptives of the excluded individuals are provided. Unusual postcode – District-level postcodes shared between fewer than 5,000 individuals (anomalous postcodes, often with special meanings, such as "marketing campaign"). Transferred out of Scotland – Individuals absent from NSS records at the end of the study period, suggesting transfer out of Scotland or otherwise poor tracking. CHI – Community Health Index. NRS – National Records of Scotland.

S Figure 2: Kaplan-Meier survival curves show ~10 year differences in median survival between top and bottom deprivation deciles. Empirical survival curves were calculated from 1,967,130 individuals and 647,752 mortality records between 2001–2016, with median survival annotated for the least and most deprived deciles. The accompanying table quantifies the relative mortality of the sexes, birth cohorts, and deprivation deciles in log hazard ratios, with SIMD 1 males from the 1965 birth cohort as reference. See S Figure 2—Source Data 1 for numerical details of the Kaplan-Meier curves including median survival of each decile.

S Figure 2—Source Data 1: Kaplan-Meier survival curves show ~10 year differences in median survival between top and bottom deprivation deciles (Excel Document). SIMD – Scottish Index for Multiple Deprivation decile; Records – Total number of individuals in the decile; N Max – Total number of uncensored individuals during the study period; Events –

For peer review only - http://bmjopen.bmj.com/site/about/guidelines.xhtml

Number of individuals who died during the study period; Mean – Mean survival time; SE – Standard error of the mean survival time; Median – median survival time (i.e. number of years 50% of individuals survive); 95% CI – 95% confidence interval around the median survival time (years).

S Figure 3: Age-adjusted reductions in excess mortality following hospital admission between 2001–2011. Dots represent excess mortality in the first five years after hospital admission compared non-admissions, compared to the excess mortality in 2001, expressed in age-adjusted ln HR. The black dots represent five-year mortality following survival to the current year. Fitted are fixed intercept 3rd power polynomials, weighted by inverse variance of estimates. The polynomial fitted value at 2011 with its standard error are used in morbidity driven mortality improvement models. See Supplementary Data File 3 for this graph stratified by sex and deprivation.

S Figure 4: Age-adjusted reductions in hospital admission rate by decade of birth. Bars represent the improvements in hospital admission rate since the previous decade of birth, expressed in age-adjusted lnHR. Dots represent the relative contribution of the disease category to the overall (ie. thee burden of death weighted) improvements in mortality due to changing hospital admission rates. Larger dots indicate a greater contribution. A red circle around the dot indicates a negative contribution (i.e. deterioration). These overall improvements are shown in the grey bars - which represent the modelled effect of morbidity incidence on mortality. The black bars represent the improvements in mortality observed from death records, by decade of birth. See Supplementary Data File 4 for this graph stratified by sex and deprivation.

For peer review only

For peer review only - http://bmjopen.bmj.com/site/about/guidelines.xhtml

S Figure 5: Age-adjusted improvements in survival following hospitalisation Bars represent the age adjusted ln HR of survival for admissions in 2011 compared with 2001. Dots represent the relative contribution of the disease category to the overall (i.e. the burden of death weighted) improvements in mortality due to changing hospital admission rates. Larger dots indicate a greater contribution. A red circle around the dot indicates a negative contribution (i.e. deterioration). These overall improvements are shown in the grey bar - which represents the modelled effect of morbidity survival on mortality 2001 vs 2011. (As is obvious, the effect of death on death itself - first panel - is unchanged) See Supplementary Data File 5 for this graph stratified by sex and deprivation.

S Figure 6: Improvements in mortality projected from changes in disease morbidity partially explain observed improvements in mortality. Improvements in morbidity, based on reductions in hospitalisation rate and 5-year mortality, were calculated for individuals stratified by sex and decade of birth. The dotted line represents perfect concordance between projected and observed mortality improvements, with points above this line indicating mortality improvements have been greater than projected from disease improvements, and points below this line indicating the opposite. The solid line is the regression of projected mortality on observed mortality and includes all shown data points, weighted by their uncertainty in both directions. Annotated are the regression slope (standard error), correlation, variance explained, and significance. Concordance between projected and observed mortality appears to increase with later decades of birth. M – Male; F – Female; 1 – improvements of the cohort born in the decade after 1915 compared with the cohort born in the decade before; 2 – 1925; 3 – 1935; 4 – 1945; 5 – 1955.

S Table 1: Total Improvements in mortality and morbidity across all birth decades by sex and deprivation (Excel Document). Mortality improvements were estimated from morbidity records by combining the improvement in hospitalisation rate for the 1955–1965 cohort compared to the 1905–1915 cohort and the improvement in disease severity between 2001 and 2011. Actual mortality improvements of the 1955–1965 cohort vs. 1905–1915 cohort was calculated directly from mortality records. SIMD – Scottish Index of Multiple Deprivation. Lives Saved –

For peer review only - http://bmjopen.bmj.com/site/about/guidelines.xhtml

Disease groups which have benefited the most and least from the improvements in morbidity, by taking into account the burden of death and its improvement.

Supplementary Data File 1 (Excel Document): Improvements in incidence of hospitalisation and 5-year mortality following hospitalisation by sex and deprivation. Contains improvements in hospital admission rate, disease survival, and combined impact on mortality for each disease grouping, stratified by sex and deprivation.

Supplementary Data File 2 (PDF document): Age-adjusted reductions in combined hospital admission rate and survival following hospitalisation by decade of birth, stratified by sex and deprivation

Supplementary Data File 3 (PDF document): Age-adjusted reductions in excess mortality following hospital admission between 2001–2011, stratified by sex and deprivation.

Supplementary Data File 4 (PDF Document): Age-adjusted reductions in hospital admission rate by decade of birth, stratified by sex and deprivation.

Supplementary Data File 5 (PDF Document): Age-adjusted increases in survival following hospitalisation, stratified by sex and deprivation.

For peer review only

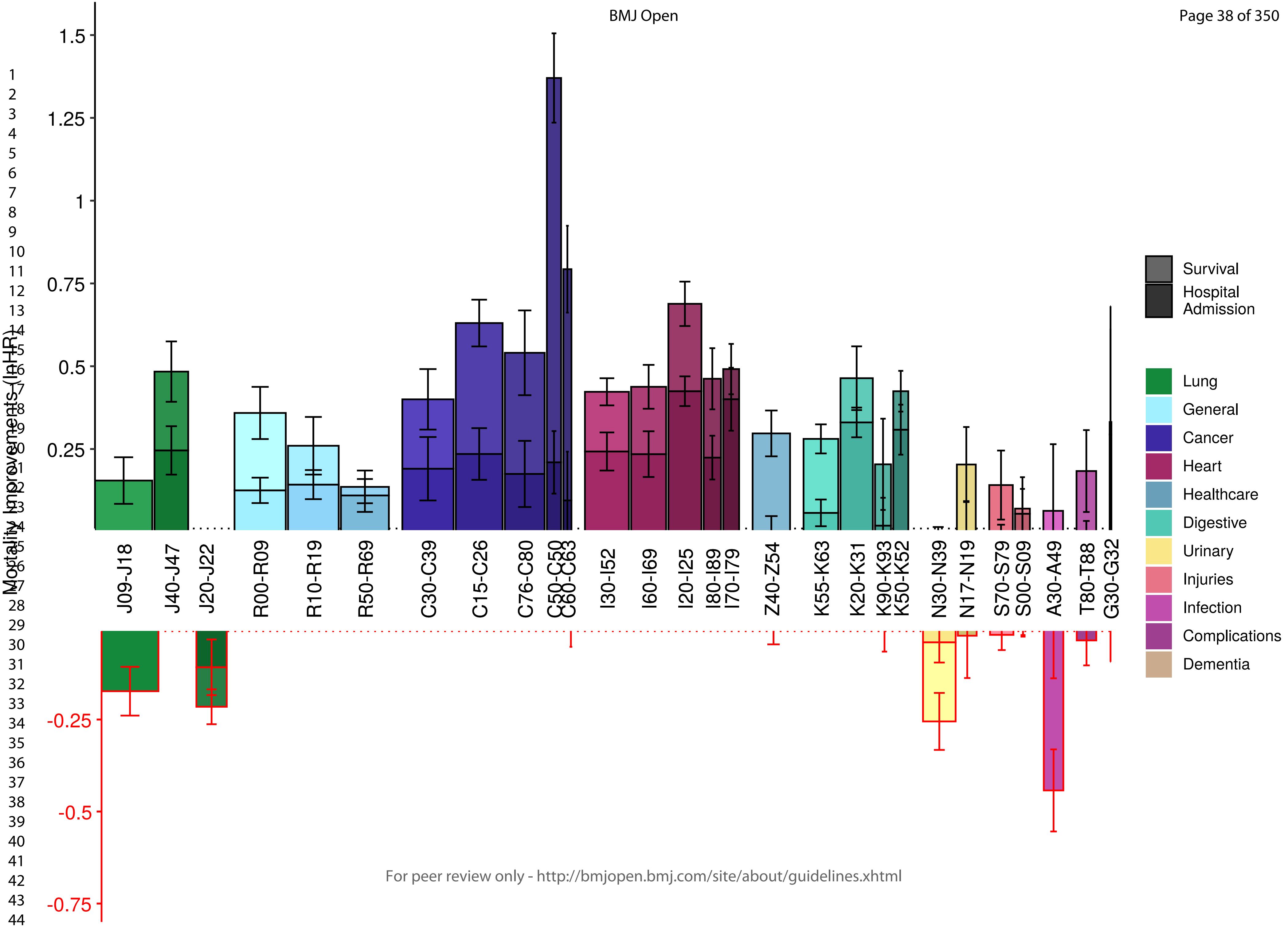
Mortality Improvements (lnHR)
1.5
1.25
1
0.75
0.5
0.25
-0.25
-0.5
-0.75
J09-J18
J40-J47
J20-J22
R00-R09
R10-R19
R50-R69
C30-C39
C15-C26
C76-C80
C50-C50
C60-C63
I30-I52
I60-I69
I20-I25
I80-I89
I70-I79
Z40-Z54
K55-K63
K20-K31
K90-K93
K50-K52
N30-N39
N17-N19
S70-S79
S00-S09
A30-A49
T80-T88
G30-G32
Survival
Hospital Admission
Lung
General
Cancer
Heart
Healthcare
Digestive
Urinary
Injuries
Infection
Complications
Dementia

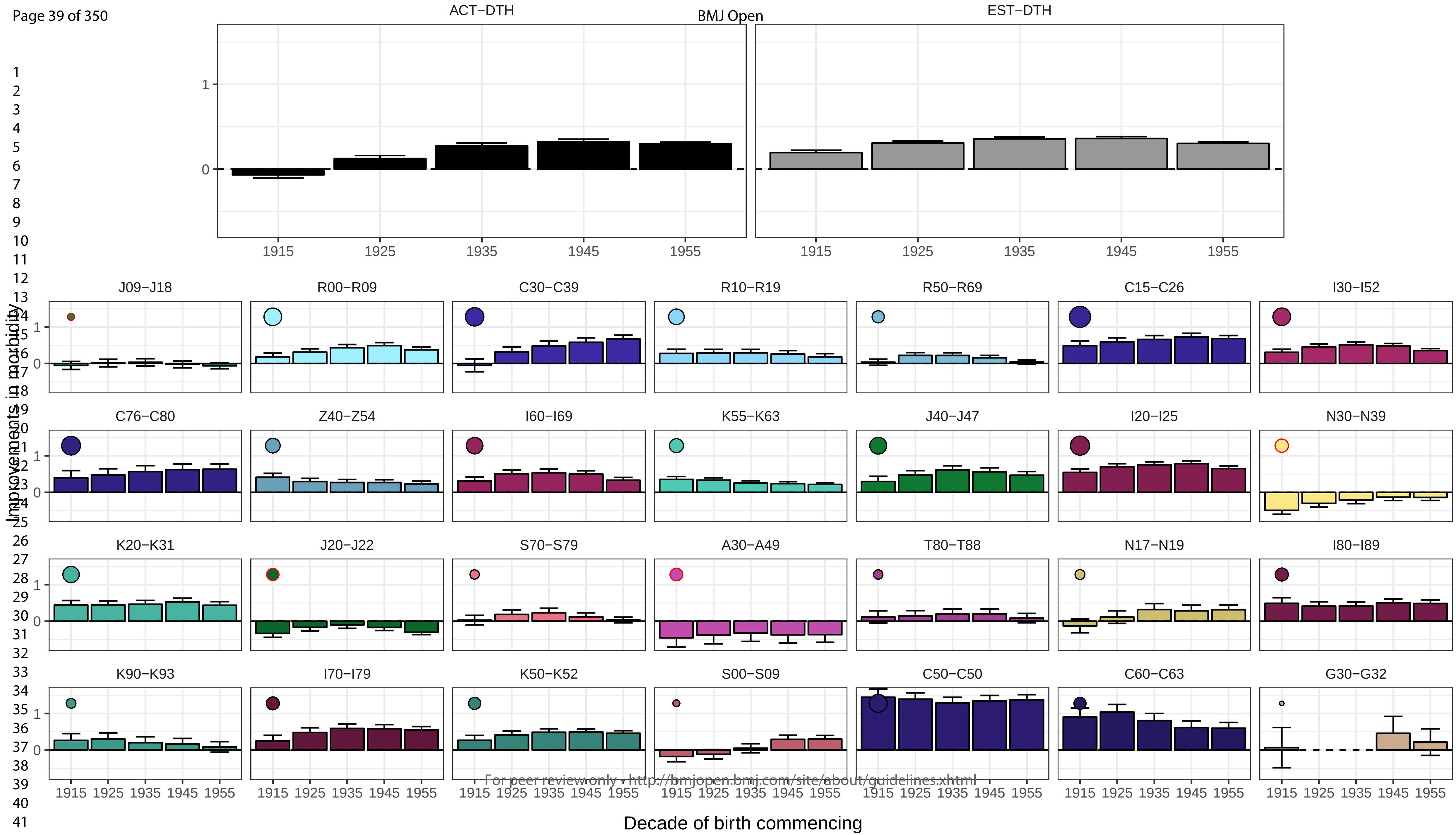

For peer review only - http://bmjopen.bmj.com/site/about/guidelines.xhtml

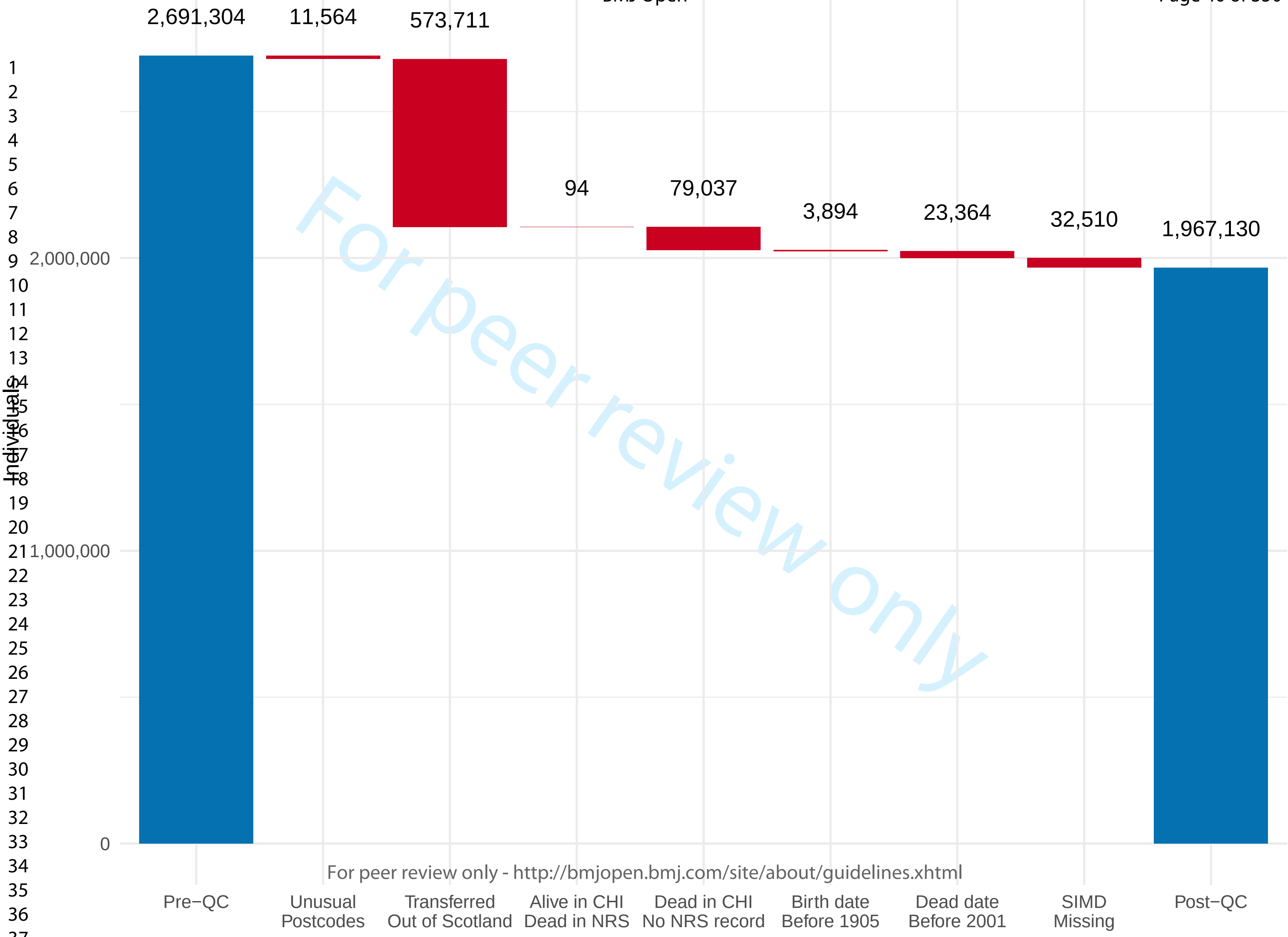

For peer review only

Effect	lnHR	SE
Female	-0.35	0.003
1915	0.95	0.015
1925	1.02	0.013
1935	0.90	0.013
1945	0.62	0.012
1955	0.30	0.010
SIMD 2	0.07	0.007
SIMD 3	0.16	0.006
SIMD 4	0.22	0.006
SIMD 5	0.29	0.006
SIMD 6	0.35	0.006
SIMD 7	0.41	0.006
SIMD 8	0.49	0.006
SIMD 9	0.59	0.006
SIMD 10	0.73	0.006

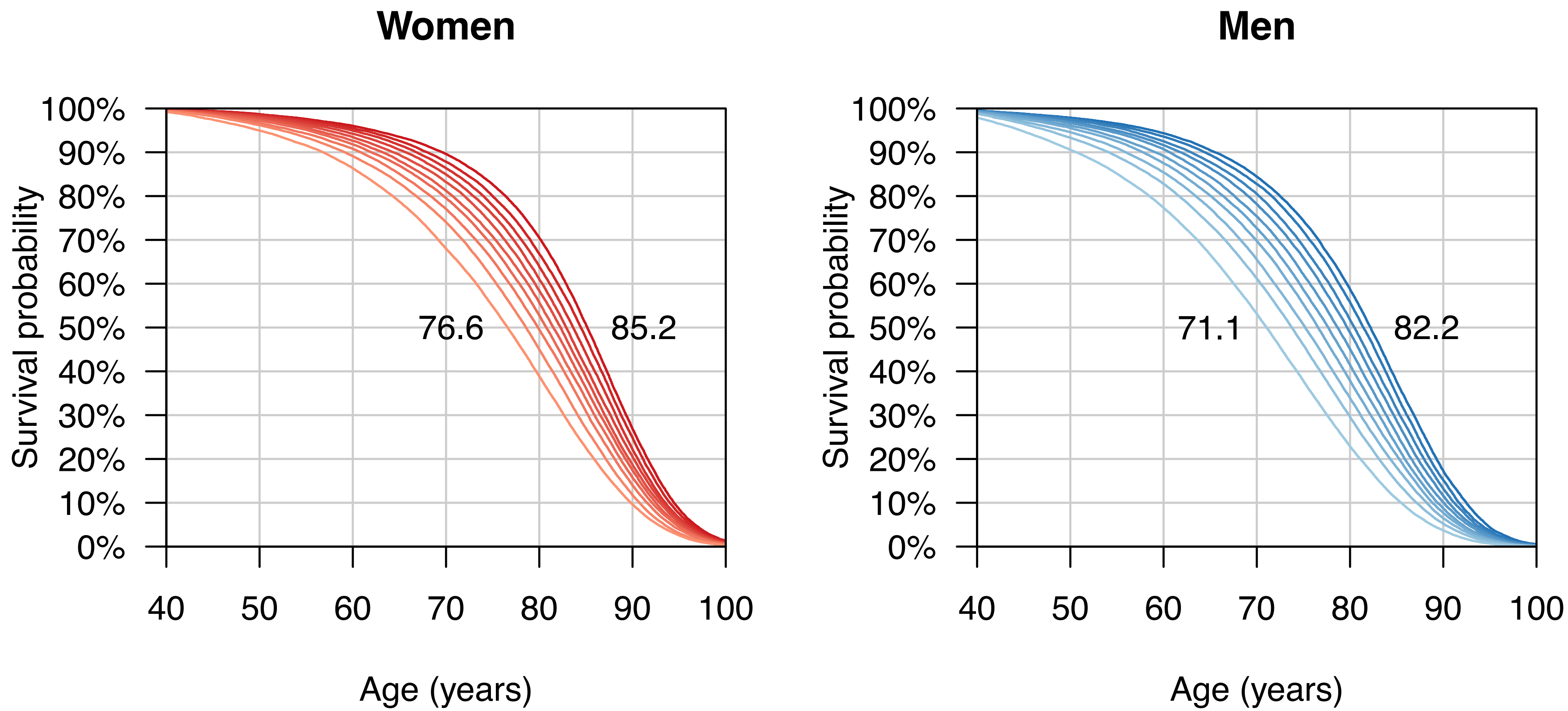

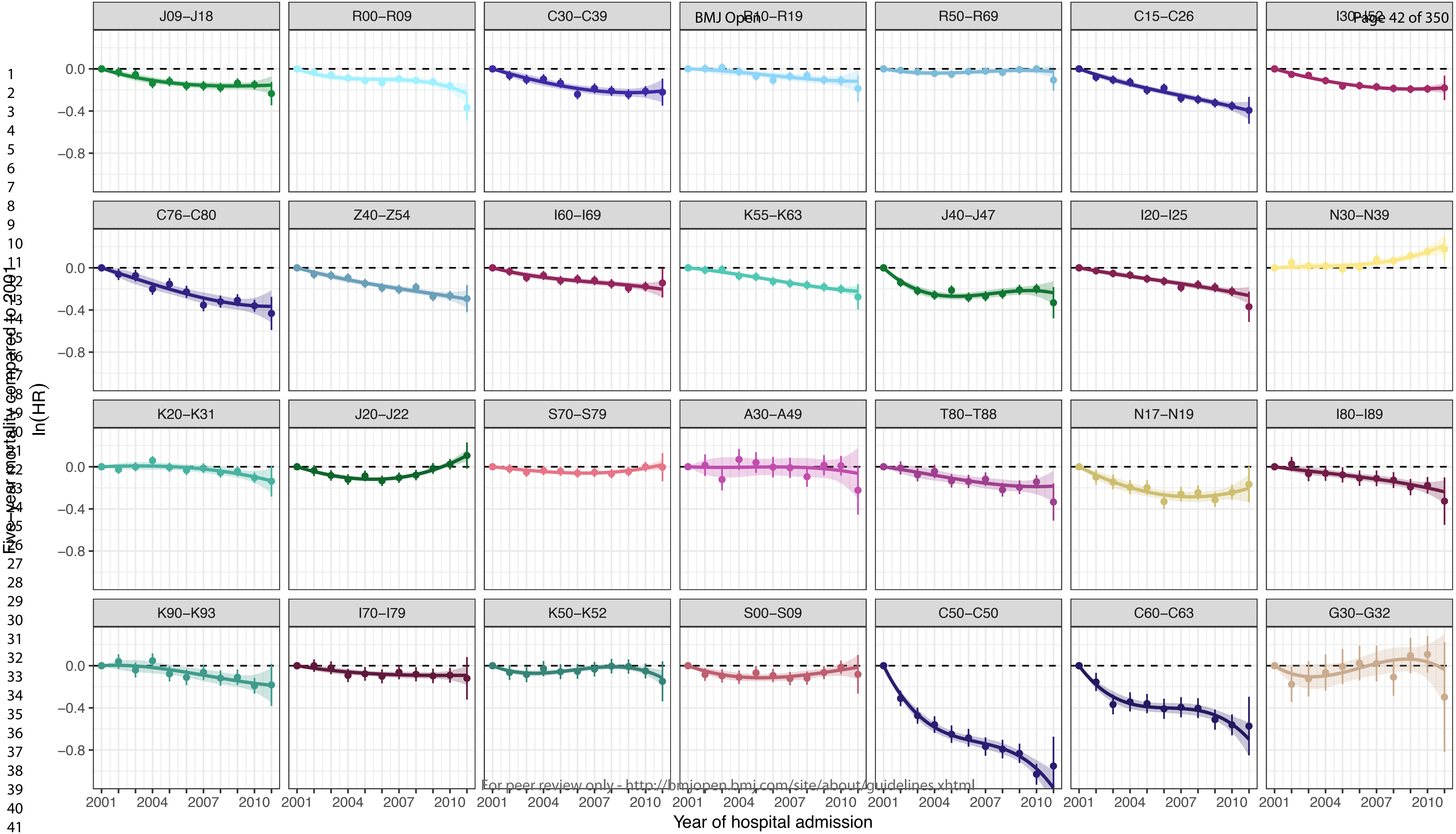
J09–J18
R00–R09
C30–C39
R10–R19
R50–R69
C15–C26
I30–I52
C76–C80
Z40–Z54
I60–I69
K55–K63
J40–J47
I20–I25
N30–N39
K20–K31
J20–J22
S70–S79
A30–A49
T80–T88
N17–N19
I80–I89
K90–K93
I70–I79
K50–K52
S00–S09
C50–C50
C60–C63
G30–G32
Five−year mortality compared to 2001
ln(HR)
0.0
−0.4
−0.8
2001
2004
2007
2010
Year of hospital admission

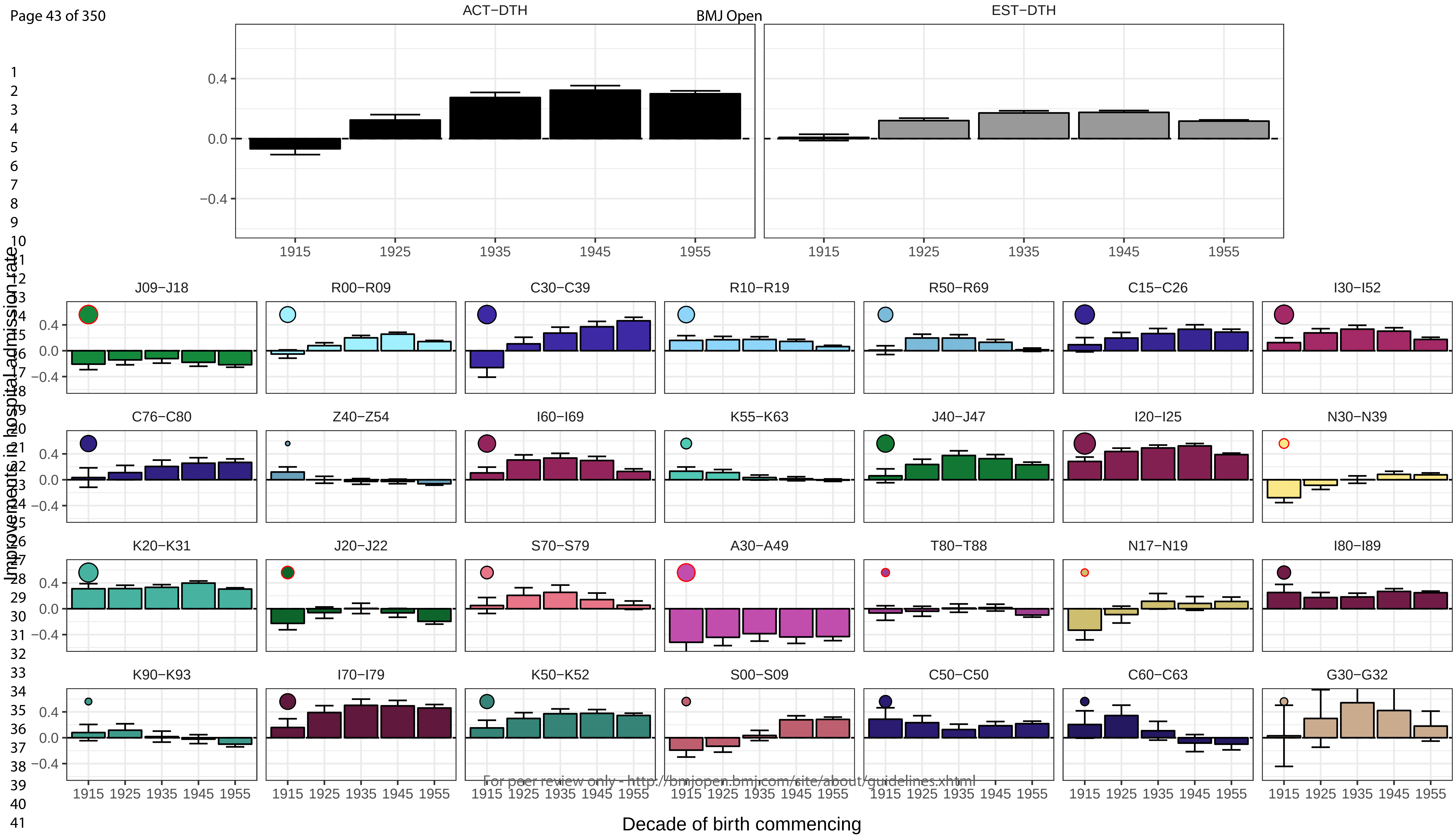

For peer review only - http://bmjopen.bmj.com/site/about/guidelines.xhtml

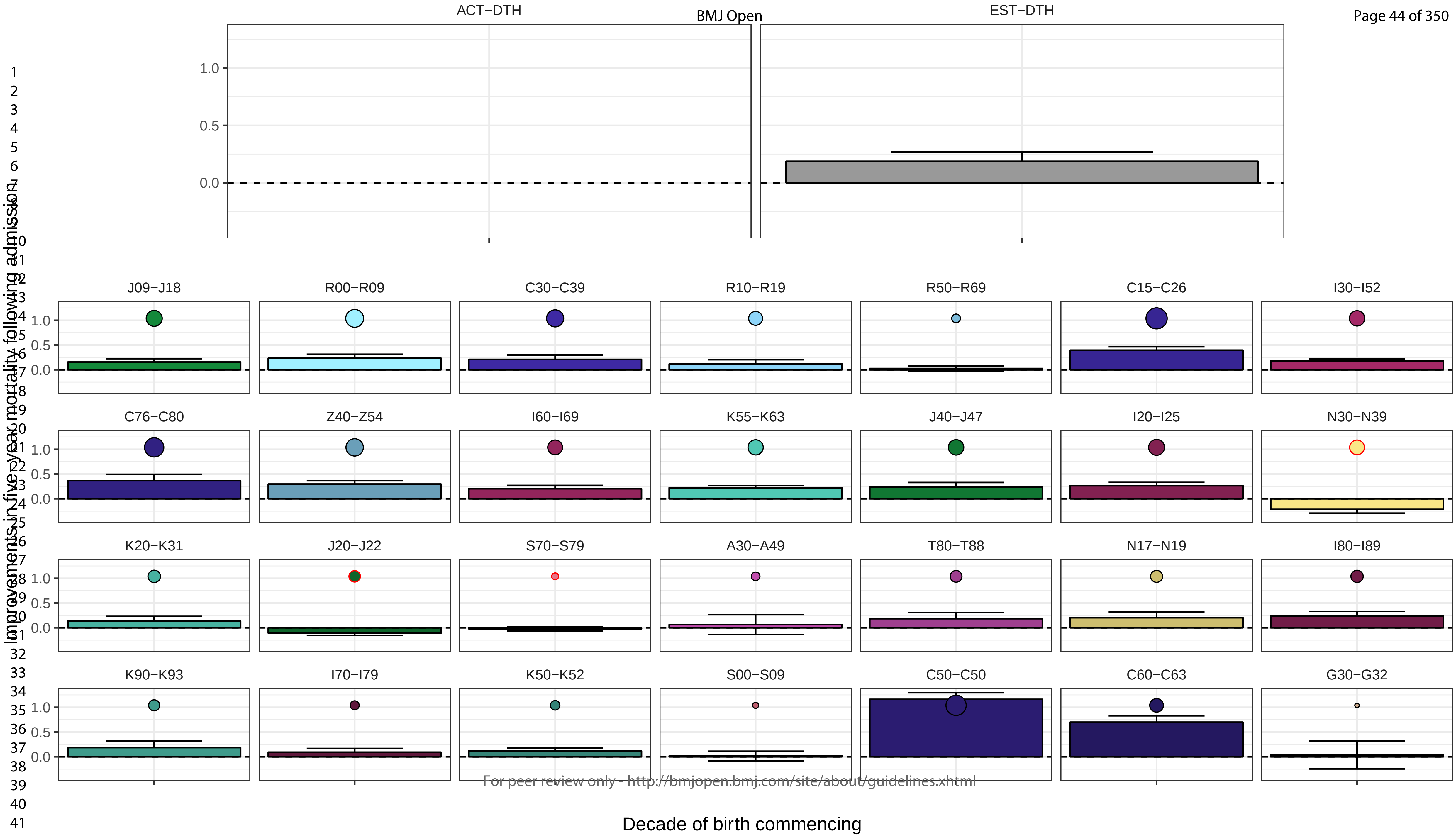
ACT−DTH
EST−DTH
J09−J18
R00−R09
C30−C39
R10−R19
R50−R69
C15−C26
I30−I52
C76−C80
Z40−Z54
I60−I69
K55−K63
J40−J47
I20−I25
N30−N39
K20−K31
J20−J22
S70−S79
A30−A49
T80−T88
N17−N19
I80−I89
K90−K93
I70−I79
K50−K52
S00−S09
C50−C50
C60−C63
G30−G32
Improvements in five−year mortality following admission
Decade of birth commencing
1.0
0.5
0.0

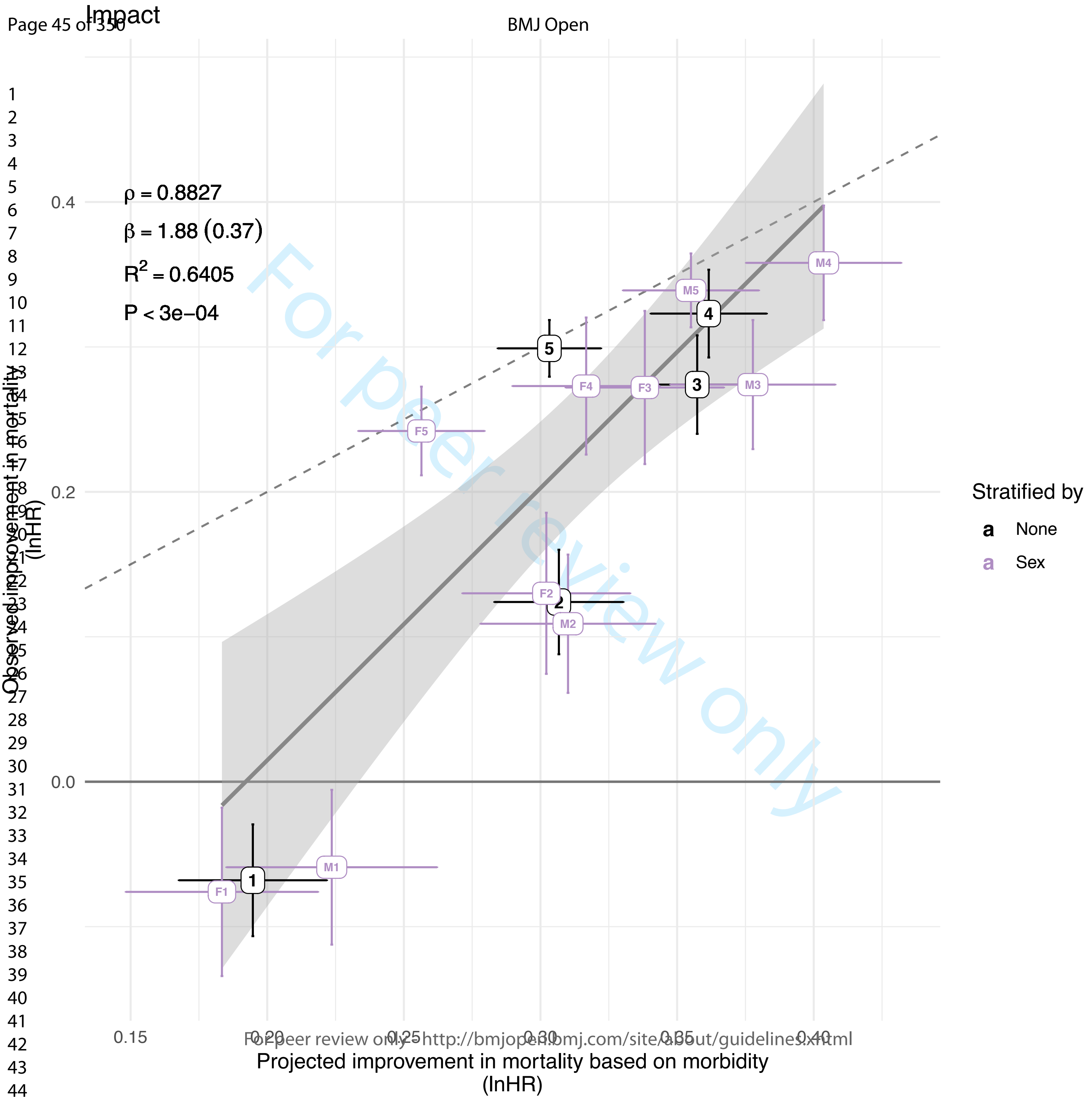

For peer review only

Stratified	Group	Mortality Improvements Observed From Death Records		Mortality Improvements Estimated from Morbidity Records		Lives Saved	
		lnHR	SE	lnHR	SE	Most	Least
None		0.95	0.0145	0.78	0.0124	I20-I25	A30-A49
Sex	M	1.02	0.0207	0.91	0.0176	C30-C39	A30-A49
	F	0.84	0.0215	0.67	0.0157	I20-I25	J09-J18
SIMD	1	1.27	0.0601	0.80	0.0449	C15-C26	J09-J18
	2	1.06	0.0560	0.74	0.0416	C15-C26	A30-A49
	3	1.01	0.0530	0.72	0.0433	C15-C26	A30-A49
	4	0.91	0.0506	0.89	0.0391	I20-I25	A30-A49
	5	0.90	0.0481	0.79	0.0374	C15-C26	A30-A49
	6	1.04	0.0465	0.85	0.0375	I20-I25	A30-A49
	7	0.89	0.0437	0.80	0.0351	I20-I25	J09-J18
	8	0.90	0.0416	0.86	0.0328	I20-I25	J09-J18
	9	0.88	0.0402	0.63	0.0355	I20-I25	J09-J18
	10	0.82	0.0387	0.67	0.0355	I20-I25	J09-J18

For peer review only

	Stratum			Individuals			
Statistic	Sex	SIMD	Dead	Age Entry	Age Exit	Deaths	Admissions
Count	Male	1	FALSE	67057	67057	0	42873
Count	Male	1	TRUE	19773	19773	19773	19773
Count	Male	2	FALSE	68578	68578	0	45638
Count	Male	2	TRUE	20904	20904	20904	20904
Count	Male	3	FALSE	65778	65778	0	43758
Count	Male	3	TRUE	23315	23315	23315	23315
Count	Male	4	FALSE	66278	66278	0	44121
Count	Male	4	TRUE	25194	25194	25194	25194
Count	Male	5	FALSE	64481	64481	0	43801
Count	Male	5	TRUE	27744	27744	27744	27744
Count	Male	6	FALSE	62624	62624	0	42915
Count	Male	6	TRUE	28861	28861	28861	28861
Count	Male	7	FALSE	62320	62320	0	43099
Count	Male	7	TRUE	31493	31493	31493	31493
Count	Male	8	FALSE	61306	61306	0	42554
Count	Male	8	TRUE	33924	33924	33924	33924
Count	Male	9	FALSE	58922	58922	0	41091
Count	Male	9	TRUE	35176	35176	35176	35176
Count	Male	10	FALSE	53644	53644	0	37873
Count	Male	10	TRUE	35580	35580	35580	35580
Count	Male	ALL	FALSE	630988	630988	0	427723
Count	Male	ALL	TRUE	281964	281964	281964	281964
Count	Female	1	FALSE	77585	77585	0	50045
Count	Female	1	TRUE	22778	22778	22778	22778
Count	Female	2	FALSE	78528	78528	0	52875
Count	Female	2	TRUE	23969	23969	23969	23969
Count	Female	3	FALSE	75176	75176	0	50644
Count	Female	3	TRUE	27550	27550	27550	27550
Count	Female	4	FALSE	74392	74392	0	50628
Count	Female	4	TRUE	29002	29002	29002	29002
Count	Female	5	FALSE	73595	73595	0	51276
Count	Female	5	TRUE	31315	31315	31315	31315
Count	Female	6	FALSE	72764	72764	0	51705
Count	Female	6	TRUE	31769	31769	31769	31769
Count	Female	7	FALSE	73363	73363	0	52755
Count	Female	7	TRUE	35711	35711	35711	35711
Count	Female	8	FALSE	72232	72232	0	52514
Count	Female	8	TRUE	37975	37975	37975	37975
Count	Female	9	FALSE	68635	68635	0	50778
Count	Female	9	TRUE	39108	39108	39108	39108
Count	Female	10	FALSE	61711	61711	0	46441
Count	Female	10	TRUE	37970	37970	37970	37970
Count	Female	ALL	FALSE	727981	727981	0	509661
Count	Female	ALL	TRUE	317147	317147	317147	317147
Count	ALL	1	FALSE	144642	144642	0	92918
Count	ALL	1	TRUE	42551	42551	42551	42551
Count	ALL	2	FALSE	147106	147106	0	98513
Count	ALL	2	TRUE	44873	44873	44873	44873

For peer review only

Count	ALL	3	FALSE	140954	140954	0	94402
Count	ALL	3	TRUE	50865	50865	50865	50865
Count	ALL	4	FALSE	140670	140670	0	94749
Count	ALL	4	TRUE	54196	54196	54196	54196
Count	ALL	5	FALSE	138076	138076	0	95077
Count	ALL	5	TRUE	59059	59059	59059	59059
Count	ALL	6	FALSE	135388	135388	0	94620
Count	ALL	6	TRUE	60630	60630	60630	60630
Count	ALL	7	FALSE	135683	135683	0	95854
Count	ALL	7	TRUE	67204	67204	67204	67204
Count	ALL	8	FALSE	133538	133538	0	95068
Count	ALL	8	TRUE	71899	71899	71899	71899
Count	ALL	9	FALSE	127557	127557	0	91869
Count	ALL	9	TRUE	74284	74284	74284	74284
Count	ALL	10	FALSE	115355	115355	0	84314
Count	ALL	10	TRUE	73550	73550	73550	73550
Count	ALL	ALL	FALSE	1358969	1358969	0	937384
Count	ALL	ALL	TRUE	599111	599111	599111	599111
Count	ALL	ALL	ALL	1958080	1958080	599111	1536495
Mean	Male	1	FALSE	51.42597	66.28053	0	3.022682196
Mean	Male	1	TRUE	70.28643	78.18031	1	9.638395792
Mean	Male	2	FALSE	51.03837	65.89842	0	3.247674181
Mean	Male	2	TRUE	69.47959	77.38318	1	9.913796403
Mean	Male	3	FALSE	51.20473	65.95515	0	3.275213597
Mean	Male	3	TRUE	69.67985	77.50238	1	9.254685825
Mean	Male	4	FALSE	51.15935	65.89322	0	3.264507076
Mean	Male	4	TRUE	69.26146	77.05243	1	9.325474319
Mean	Male	5	FALSE	51.15548	65.85526	0	3.488391929
Mean	Male	5	TRUE	68.88615	76.58978	1	9.352292388
Mean	Male	6	FALSE	51.04404	65.69063	0	3.74961676
Mean	Male	6	TRUE	68.37291	75.99292	1	9.493572641
Mean	Male	7	FALSE	51.12552	65.71495	0	3.856530809
Mean	Male	7	TRUE	67.96991	75.5622	1	9.641507637
Mean	Male	8	FALSE	50.78795	65.35291	0	4.040371252
Mean	Male	8	TRUE	67.42248	74.89572	1	9.831594152
Mean	Male	9	FALSE	50.5558	65.03233	0	4.281575642
Mean	Male	9	TRUE	66.58443	73.99249	1	9.876563566
Mean	Male	10	FALSE	49.7487	64.14061	0	4.579542913
Mean	Male	10	TRUE	64.88422	72.25591	1	10.06306914
Mean	Male	ALL	FALSE	50.95171	65.61792	0	3.649746112
Mean	Male	ALL	TRUE	68.0044	75.62636	1	9.66304209
Mean	Female	1	FALSE	52.84354	67.62372	0	2.971747116
Mean	Female	1	TRUE	74.03348	81.87075	1	8.8515234
Mean	Female	2	FALSE	52.34058	67.14119	0	3.217845864
Mean	Female	2	TRUE	73.2013	81.08391	1	8.994993533
Mean	Female	3	FALSE	52.74256	67.43342	0	3.257941364
Mean	Female	3	TRUE	73.65572	81.44229	1	8.581088929
Mean	Female	4	FALSE	52.8636	67.55455	0	3.346475427
Mean	Female	4	TRUE	73.11994	80.85057	1	8.586511275
Mean	Female	5	FALSE	52.93602	67.58846	0	3.533813438

For peer review only

Mean	Female	5	TRUE	72.87527	80.5496	1	8.608430465
Mean	Female	6	FALSE	52.78292	67.47188	0	3.865799021
Mean	Female	6	TRUE	72.03465	79.66025	1	8.980893324
Mean	Female	7	FALSE	53.06696	67.72345	0	4.08361163
Mean	Female	7	TRUE	71.87808	79.50544	1	9.199602363
Mean	Female	8	FALSE	52.88178	67.52256	0	4.25892956
Mean	Female	8	TRUE	71.50472	79.04223	1	9.278314681
Mean	Female	9	FALSE	52.54855	67.1636	0	4.582224812
Mean	Female	9	TRUE	70.73319	78.21541	1	9.421167025
Mean	Female	10	FALSE	51.786	66.35218	0	5.038761323
Mean	Female	10	TRUE	69.64389	77.0918	1	9.833394785
Mean	Female	ALL	FALSE	52.69305	67.37505	0	3.779152203
Mean	Female	ALL	TRUE	72.06165	79.70097	1	9.081690825
Mean	ALL	1	FALSE	52.18634	67.00101	0	2.99536096
Mean	ALL	1	TRUE	72.29227	80.15584	1	9.217174684
Mean	ALL	2	FALSE	51.73351	66.56184	0	3.231751254
Mean	ALL	2	TRUE	71.46755	79.35993	1	9.423016068
Mean	ALL	3	FALSE	52.02491	66.74356	0	3.266001674
Mean	ALL	3	TRUE	71.8333	79.63635	1	8.88984567
Mean	ALL	4	FALSE	52.06063	66.7718	0	3.307855264
Mean	ALL	4	TRUE	71.32626	79.08493	1	8.930031737
Mean	ALL	5	FALSE	52.10451	66.77906	0	3.512601756
Mean	ALL	5	TRUE	71.00131	78.6894	1	8.957872636
Mean	ALL	6	FALSE	51.9786	66.64796	0	3.812058676
Mean	ALL	6	TRUE	70.29159	77.91453	1	9.224938149
Mean	ALL	7	FALSE	52.17525	66.80093	0	3.979312073
Mean	ALL	7	TRUE	70.04664	77.65757	1	9.406687102
Mean	ALL	8	FALSE	51.92052	66.5265	0	4.158591562
Mean	ALL	8	TRUE	69.5786	77.08579	1	9.539367724
Mean	ALL	9	FALSE	51.62805	66.17911	0	4.443346896
Mean	ALL	9	TRUE	68.76861	76.21571	1	9.636812773
Mean	ALL	10	FALSE	50.83859	65.32373	0	4.825209137
Mean	ALL	10	TRUE	67.34138	74.75243	1	9.94450034
Mean	ALL	ALL	FALSE	51.88452	66.55919	0	3.719067175
Mean	ALL	ALL	TRUE	70.15216	77.78331	1	9.355296431
Mean	ALL	ALL	ALL	57.47384	69.99342	0.305969	5.443576361
Median	Male	1	FALSE	50.09	65.08	0	1
Median	Male	1	TRUE	71.67	79.75	1	7
Median	Male	2	FALSE	49.84	64.83	0	1
Median	Male	2	TRUE	70.92	79	1	7
Median	Male	3	FALSE	49.84	64.83	0	1
Median	Male	3	TRUE	71.17	79.17	1	6
Median	Male	4	FALSE	49.75	64.75	0	1
Median	Male	4	TRUE	70.67	78.66	1	6
Median	Male	5	FALSE	49.75	64.66	0	2
Median	Male	5	TRUE	70.25	78.09	1	6
Median	Male	6	FALSE	49.59	64.5	0	2
Median	Male	6	TRUE	69.67	77.5	1	7
Median	Male	7	FALSE	49.67	64.5	0	2
Median	Male	7	TRUE	69.25	76.92	1	7

For peer review only

Median	Male	8	FALSE	49.17	64	0	2
Median	Male	8	TRUE	68.67	76.33	1	7
Median	Male	9	FALSE	48.84	63.58	0	2
Median	Male	9	TRUE	67.84	75.33	1	7
Median	Male	10	FALSE	47.67	62.41	0	2
Median	Male	10	TRUE	66	73.42	1	7
Median	Male	ALL	FALSE	49.42	64.33	0	2
Median	Male	ALL	TRUE	69.34	77.09	1	7
Median	Female	1	FALSE	51.25	66.41	0	1
Median	Female	1	TRUE	75.92	84	1	6
Median	Female	2	FALSE	50.75	65.83	0	1
Median	Female	2	TRUE	75	83.33	1	6
Median	Female	3	FALSE	51	66.08	0	1
Median	Female	3	TRUE	75.59	83.59	1	6
Median	Female	4	FALSE	51.25	66.41	0	2
Median	Female	4	TRUE	74.84	82.84	1	6
Median	Female	5	FALSE	51.34	66.41	0	2
Median	Female	5	TRUE	74.67	82.58	1	6
Median	Female	6	FALSE	51.25	66.33	0	2
Median	Female	6	TRUE	73.67	81.5	1	6
Median	Female	7	FALSE	51.59	66.58	0	2
Median	Female	7	TRUE	73.5	81.33	1	6
Median	Female	8	FALSE	51.25	66.25	0	2
Median	Female	8	TRUE	73.09	80.84	1	6
Median	Female	9	FALSE	50.84	65.91	0	2
Median	Female	9	TRUE	72.25	79.92	1	6
Median	Female	10	FALSE	49.67	64.58	0	3
Median	Female	10	TRUE	71.09	78.83	1	7
Median	Female	ALL	FALSE	51.09	66.16	0	2
Median	Female	ALL	TRUE	73.84	81.67	1	6
Median	ALL	1	FALSE	50.67	65.75	0	1
Median	ALL	1	TRUE	73.92	82	1	6
Median	ALL	2	FALSE	50.25	65.33	0	1
Median	ALL	2	TRUE	73.09	81.25	1	6
Median	ALL	3	FALSE	50.42	65.5	0	1
Median	ALL	3	TRUE	73.59	81.58	1	6
Median	ALL	4	FALSE	50.5	65.5	0	2
Median	ALL	4	TRUE	72.92	80.84	1	6
Median	ALL	5	FALSE	50.59	65.58	0	2
Median	ALL	5	TRUE	72.59	80.5	1	6
Median	ALL	6	FALSE	50.5	65.41	0	2
Median	ALL	6	TRUE	71.75	79.59	1	6
Median	ALL	7	FALSE	50.67	65.58	0	2
Median	ALL	7	TRUE	71.5	79.25	1	6
Median	ALL	8	FALSE	50.25	65.16	0	2
Median	ALL	8	TRUE	71	78.67	1	6
Median	ALL	9	FALSE	49.92	64.75	0	2
Median	ALL	9	TRUE	70.17	77.75	1	6
Median	ALL	10	FALSE	48.67	63.5	0	2
Median	ALL	10	TRUE	68.59	76.16	1	7

For peer review only

Median	ALL	ALL	FALSE	50.25	65.25	0	2
Median	ALL	ALL	TRUE	71.67	79.5	1	6
Median	ALL	ALL	ALL	55.42	69.16	0	3
SD	Male	1	FALSE	10.29335	9.895881	0	5.999965825
SD	Male	1	TRUE	11.20425	10.84432	0	11.56477008
SD	Male	2	FALSE	10.02753	9.654498	0	5.937700309
SD	Male	2	TRUE	11.41291	11.05364	0	12.00967705
SD	Male	3	FALSE	10.3327	9.804563	0	5.99970456
SD	Male	3	TRUE	11.48086	11.07249	0	10.36340142
SD	Male	4	FALSE	10.33222	9.816467	0	5.706537374
SD	Male	4	TRUE	11.45496	10.99963	0	11.33085377
SD	Male	5	FALSE	10.36481	9.818106	0	6.255840399
SD	Male	5	TRUE	11.5018	11.04789	0	11.10793953
SD	Male	6	FALSE	10.37469	9.804766	0	7.046345911
SD	Male	6	TRUE	11.53179	11.13248	0	10.85321017
SD	Male	7	FALSE	10.43387	9.812677	0	6.503949308
SD	Male	7	TRUE	11.56689	11.14452	0	11.39198387
SD	Male	8	FALSE	10.38086	9.799004	0	7.39389737
SD	Male	8	TRUE	11.7453	11.31354	0	11.60403991
SD	Male	9	FALSE	10.3025	9.69833	0	7.552972389
SD	Male	9	TRUE	11.84546	11.4105	0	11.86501435
SD	Male	10	FALSE	10.09947	9.450429	0	9.092688632
SD	Male	10	TRUE	12.11585	11.79579	0	11.63274414
SD	Male	ALL	FALSE	10.30438	9.777146	0	6.769454973
SD	Male	ALL	TRUE	11.73831	11.36275	0	11.40272153
SD	Female	1	FALSE	11.57404	10.81761	0	6.140900821
SD	Female	1	TRUE	11.61165	11.17618	0	10.80280577
SD	Female	2	FALSE	11.29791	10.57343	0	6.300878901
SD	Female	2	TRUE	11.91055	11.4771	0	11.22758745
SD	Female	3	FALSE	11.72025	10.80205	0	6.099846813
SD	Female	3	TRUE	11.71637	11.27446	0	10.18372078
SD	Female	4	FALSE	11.69384	10.81516	0	5.929254745
SD	Female	4	TRUE	11.79621	11.33437	0	10.83215537
SD	Female	5	FALSE	11.75032	10.84331	0	6.169119002
SD	Female	5	TRUE	11.80434	11.35569	0	11.05748387
SD	Female	6	FALSE	11.56321	10.77253	0	6.785243756
SD	Female	6	TRUE	11.85046	11.40183	0	10.89387782
SD	Female	7	FALSE	11.63108	10.81993	0	7.980228565
SD	Female	7	TRUE	11.8501	11.33384	0	11.96747814
SD	Female	8	FALSE	11.68272	10.877	0	7.409969424
SD	Female	8	TRUE	11.88115	11.38483	0	11.05675793
SD	Female	9	FALSE	11.59512	10.75171	0	8.314135343
SD	Female	9	TRUE	12.04586	11.55406	0	10.93156282
SD	Female	10	FALSE	11.56579	10.69242	0	8.178867841
SD	Female	10	TRUE	12.40504	11.98232	0	10.94713744
SD	Female	ALL	FALSE	11.61171	10.78305	0	6.973080412
SD	Female	ALL	TRUE	11.98467	11.54057	0	11.02783173
SD	ALL	1	FALSE	11.02152	10.42197	0	6.076001052
SD	ALL	1	TRUE	11.57586	11.1757	0	11.17011719
SD	ALL	2	FALSE	10.74405	10.17427	0	6.1342454

For peer review only

SD	ALL	2	TRUE	11.82785	11.43173	0	11.60740759
SD	ALL	3	FALSE	11.12081	10.37474	0	6.053305018
SD	ALL	3	TRUE	11.77672	11.35324	0	10.27185504
SD	ALL	4	FALSE	11.10577	10.38973	0	5.825503356
SD	ALL	4	TRUE	11.79675	11.33927	0	11.07281312
SD	ALL	5	FALSE	11.16016	10.41308	0	6.209787135
SD	ALL	5	TRUE	11.8318	11.3849	0	11.0873384
SD	ALL	6	FALSE	11.06337	10.37421	0	6.907461287
SD	ALL	6	TRUE	11.84182	11.42213	0	10.87746257
SD	ALL	7	FALSE	11.1393	10.41761	0	7.339977794
SD	ALL	7	TRUE	11.87932	11.4163	0	11.703307
SD	ALL	8	FALSE	11.15289	10.45202	0	7.403368695
SD	ALL	8	TRUE	11.99159	11.53837	0	11.32156955
SD	ALL	9	FALSE	11.06155	10.33328	0	7.972949385
SD	ALL	9	TRUE	12.1295	11.67815	0	11.38532278
SD	ALL	10	FALSE	10.95563	10.19362	0	8.618896162
SD	ALL	10	TRUE	12.49441	12.13544	0	11.28451096
SD	ALL	ALL	FALSE	11.05815	10.36529	0	6.879584137
SD	ALL	ALL	TRUE	12.04087	11.63632	0	11.20957831
SD	ALL	ALL	ALL	14.14536	11.94771	0.460817	8.834008915
Sum	Male	1	FALSE	3448471	4444574	0	202692
Sum	Male	1	TRUE	1389774	1545859	19773	190580
Sum	Male	2	FALSE	3500109	4519182	0	222719
Sum	Male	2	TRUE	1452401	1617618	20904	207238
Sum	Male	3	FALSE	3368144	4338398	0	215437
Sum	Male	3	TRUE	1624586	1806968	23315	215773
Sum	Male	4	FALSE	3390740	4367271	0	216365
Sum	Male	4	TRUE	1744973	1941259	25194	234946
Sum	Male	5	FALSE	3298556	4246413	0	224935
Sum	Male	5	TRUE	1911177	2124907	27744	259470
Sum	Male	6	FALSE	3196582	4113810	0	234816
Sum	Male	6	TRUE	1973311	2193232	28861	273994
Sum	Male	7	FALSE	3186142	4095356	0	240339
Sum	Male	7	TRUE	2140576	2379681	31493	303640
Sum	Male	8	FALSE	3113606	4006526	0	247699
Sum	Male	8	TRUE	2287240	2540762	33924	333527
Sum	Male	9	FALSE	2978849	3831835	0	252279
Sum	Male	9	TRUE	2342174	2602760	35176	347418
Sum	Male	10	FALSE	2668719	3440759	0	245665
Sum	Male	10	TRUE	2308580	2570865	35580	358044
Sum	Male	ALL	FALSE	32149919	41404123	0	2302946
Sum	Male	ALL	TRUE	19174792	21323910	281964	2724630
Sum	Female	1	FALSE	4099866	5246586	0	230563
Sum	Female	1	TRUE	1686335	1864852	22778	201620
Sum	Female	2	FALSE	4110201	5272464	0	252691
Sum	Female	2	TRUE	1754562	1943500	23969	215601
Sum	Female	3	FALSE	3964975	5069375	0	244919
Sum	Female	3	TRUE	2029215	2243735	27550	236409
Sum	Female	4	FALSE	3932629	5025518	0	248951
Sum	Female	4	TRUE	2120625	2344828	29002	249026

For peer review only

Sum	Female	5	FALSE	3895827	4974173	0	260071
Sum	Female	5	TRUE	2282089	2522411	31315	269573
Sum	Female	6	FALSE	3840696	4909524	0	281291
Sum	Female	6	TRUE	2288469	2530726	31769	285314
Sum	Female	7	FALSE	3893152	4968395	0	299586
Sum	Female	7	TRUE	2566838	2839219	35711	328527
Sum	Female	8	FALSE	3819757	4877290	0	307631
Sum	Female	8	TRUE	2715392	3001629	37975	352344
Sum	Female	9	FALSE	3606670	4609774	0	314501
Sum	Female	9	TRUE	2766234	3058848	39108	368443
Sum	Female	10	FALSE	3195766	4094660	0	310947
Sum	Female	10	TRUE	2644378	2927176	37970	373374
Sum	Female	ALL	FALSE	38359538	49047757	0	2751151
Sum	Female	ALL	TRUE	22854136	25276924	317147	2880231
Sum	ALL	1	FALSE	7548337	9691160	0	433255
Sum	ALL	1	TRUE	3076108	3410711	42551	392200
Sum	ALL	2	FALSE	7610310	9791646	0	475410
Sum	ALL	2	TRUE	3206963	3561118	44873	422839
Sum	ALL	3	FALSE	7333119	9407772	0	460356
Sum	ALL	3	TRUE	3653801	4050703	50865	452182
Sum	ALL	4	FALSE	7323369	9392789	0	465316
Sum	ALL	4	TRUE	3865598	4286087	54196	483972
Sum	ALL	5	FALSE	7194383	9220586	0	485006
Sum	ALL	5	TRUE	4193267	4647317	59059	529043
Sum	ALL	6	FALSE	7037278	9023334	0	516107
Sum	ALL	6	TRUE	4261779	4723958	60630	559308
Sum	ALL	7	FALSE	7079294	9063751	0	539925
Sum	ALL	7	TRUE	4707414	5218899	67204	632167
Sum	ALL	8	FALSE	6933363	8883815	0	555330
Sum	ALL	8	TRUE	5002632	5542391	71899	685871
Sum	ALL	9	FALSE	6585519	8441609	0	566780
Sum	ALL	9	TRUE	5108407	5661608	74284	715861
Sum	ALL	10	FALSE	5864485	7535418	0	556612
Sum	ALL	10	TRUE	4952959	5498041	73550	731418
Sum	ALL	ALL	FALSE	70509457	90451880	0	5054097
Sum	ALL	ALL	TRUE	42028928	46600834	599111	5604861
Sum	ALL	ALL	ALL	1.13E+08	1.37E+08	599111	10658958

For peer review only

A	B	C	D	E	F	G	H	I
768	381	4912	4307	751	355	2680	6290	11876
1187	200	8254	2540	915	796	1577	2963	8557
947	442	5057	4910	864	387	2786	6419	12800
1307	205	8489	2889	1107	873	1714	3289	9226
904	398	4465	4753	806	466	2809	6089	12413
1439	219	8672	2994	1227	1074	1951	3659	10152
932	422	4324	4713	861	536	2993	6120	12650
1579	227	9537	3301	1410	1170	2087	3921	11082
989	388	4315	4880	943	606	2953	6114	12756
1688	278	10341	3586	1601	1328	2166	4407	12340
1023	430	4169	4982	1011	708	3052	6168	12721
1747	251	10747	3912	1678	1402	2281	4473	12687
1114	455	4091	4988	1114	845	3098	6299	13064
1946	318	11547	4154	1806	1677	2338	4835	13848
1156	434	3943	4850	1076	950	3191	6165	13071
2182	373	12407	4563	2183	1686	2435	5192	14854
1222	422	3744	4958	1209	1106	2900	6021	13041
2250	390	12585	4612	2186	2028	2551	5123	15077
1193	417	3183	4661	1239	1398	2721	5225	12022
2159	362	12620	4635	2238	2454	2508	4779	14900
10248	4189	42203	48002	9874	7357	29183	60910	126414
17484	2823	105199	37186	16351	14488	21608	42641	122723
1193	453	6203	6623	1059	447	3348	9096	8959
1370	230	7227	2504	1178	1175	1674	3717	8838
1291	517	6381	7534	1198	502	3694	9132	9766
1461	254	7507	2939	1336	1158	1748	4249	9326
1265	484	5911	7155	1220	539	3689	8872	9649
1670	269	8030	3221	1612	1542	2172	4964	10847
1279	456	5846	7166	1255	566	3781	9320	9851
1852	241	8746	3503	1679	1442	2161	5254	11583
1399	526	5765	7377	1375	610	3965	9118	10271
1885	278	9268	3889	1845	1615	2220	5813	12493
1533	541	5842	7419	1425	719	4167	9503	10746
2063	342	9884	3962	1943	1601	2226	5822	12998
1688	572	5873	7713	1572	812	4256	9891	11324
2537	354	10894	4538	2260	1651	2432	6734	14576
1771	560	5610	7678	1631	826	4388	9918	11506
2692	355	11410	4741	2439	1745	2600	6868	15761
1797	630	5387	7669	1673	813	4169	9473	11668
2801	392	11941	5022	2586	1843	2524	6954	15978
1815	573	4705	7256	1634	967	4132	8244	11054
2755	389	11506	5042	2657	1837	2475	6532	15461
15031	5312	57523	73590	14042	6801	39589	92567	104794
21086	3104	96413	39361	19535	15609	22232	56907	127861
1961	834	11115	10930	1810	802	6028	15386	20835
2557	430	15481	5044	2093	1971	3251	6680	17395
2238	959	11438	12444	2062	889	6480	15551	22566
2768	459	15996	5828	2443	2031	3462	7538	18552

For peer review only

2169	882	10376	11908	2026	1005	6498	14961	22062
3109	488	16702	6215	2839	2616	4123	8623	20999
2211	878	10170	11879	2116	1102	6774	15440	22501
3431	468	18283	6804	3089	2612	4248	9175	22665
2388	914	10080	12257	2318	1216	6918	15232	23027
3573	556	19609	7475	3446	2943	4386	10220	24833
2556	971	10011	12401	2436	1427	7219	15671	23467
3810	593	20631	7874	3621	3003	4507	10295	25685
2802	1027	9964	12701	2686	1657	7354	16190	24388
4483	672	22441	8692	4066	3328	4770	11569	28424
2927	994	9553	12528	2707	1776	7579	16083	24577
4874	728	23817	9304	4622	3431	5035	12060	30615
3019	1052	9131	12627	2882	1919	7069	15494	24709
5051	782	24526	9634	4772	3871	5075	12077	31055
3008	990	7888	11917	2873	2365	6853	13469	23076
4914	751	24126	9677	4895	4291	4983	11311	30361
25279	9501	99726	121592	23916	14158	68772	153477	231208
38570	5927	201612	76547	35886	30097	43840	99548	250584
63849	15428	301338	198139	59802	44255	112612	253025	481792
0.019029	0.008366	0.296464	0.132738	0.063856	0.010036	0.087776	0.163816	0.501603
0.112325	0.015779	2.710868	0.485055	0.11369	0.07227	0.160977	0.24842	1.415971
0.022617	0.008807	0.289772	0.143326	0.06995	0.010966	0.069439	0.163784	0.545146
0.115385	0.015499	2.649254	0.46594	0.139399	0.078119	0.156477	0.26593	1.485314
0.023123	0.009091	0.260938	0.142251	0.058941	0.012755	0.082581	0.158305	0.561221
0.116406	0.013682	2.105469	0.420459	0.125842	0.082222	0.162428	0.25653	1.447609
0.023613	0.008826	0.250822	0.135354	0.048764	0.015254	0.079423	0.162286	0.561619
0.118004	0.014646	2.139875	0.446972	0.126816	0.088593	0.173057	0.258435	1.45741
0.025946	0.01174	0.259069	0.140429	0.064143	0.020626	0.087064	0.166095	0.593369
0.110871	0.016076	2.060734	0.41454	0.129578	0.091011	0.156538	0.259624	1.49034
0.027801	0.011194	0.261513	0.171324	0.083083	0.024384	0.093015	0.168114	0.63118
0.114653	0.015661	2.079796	0.432487	0.129691	0.097017	0.168324	0.257233	1.462631
0.030568	0.010398	0.259499	0.166528	0.074358	0.028514	0.092121	0.181386	0.651621
0.118376	0.015591	2.043121	0.401137	0.151843	0.106341	0.151494	0.25339	1.511383
0.034271	0.010342	0.264101	0.161436	0.08805	0.034744	0.094281	0.178808	0.675105
0.125604	0.023464	2.019308	0.427249	0.152901	0.10721	0.152075	0.2528	1.532219
0.037813	0.011354	0.262483	0.162842	0.091036	0.042989	0.092359	0.180764	0.713808
0.12662	0.017938	1.968189	0.429242	0.144445	0.1221	0.148368	0.234506	1.504236
0.040769	0.0118	0.24575	0.176907	0.103423	0.06435	0.094046	0.169395	0.728991
0.120573	0.019084	1.943761	0.391821	0.168325	0.151124	0.144182	0.218353	1.417566
0.028127	0.010127	0.265669	0.152462	0.073648	0.025395	0.086834	0.168986	0.611913
0.11859	0.017091	2.129421	0.427519	0.140685	0.103538	0.156336	0.24884	1.47538
0.02485	0.007463	0.386544	0.149617	0.043127	0.010157	0.075414	0.205207	0.276445
0.103345	0.016595	2.489595	0.330582	0.108614	0.097594	0.148872	0.265914	1.129072
0.028321	0.00922	0.371995	0.154607	0.0394	0.010735	0.097812	0.207455	0.302987
0.109642	0.01698	2.330469	0.361675	0.10597	0.088156	0.15374	0.290083	1.130001
0.026884	0.008567	0.355313	0.161674	0.044948	0.013887	0.084588	0.210147	0.318626
0.103666	0.014955	1.971688	0.345336	0.122831	0.098984	0.153103	0.298911	1.135644
0.027503	0.00781	0.347994	0.164507	0.042545	0.013886	0.08927	0.22118	0.335103
0.11151	0.011965	2.015551	0.305082	0.116647	0.084891	0.15392	0.295635	1.168264
0.032217	0.008886	0.343108	0.166805	0.049501	0.016401	0.092778	0.216781	0.353407

0.109149	0.013284	1.806131	0.382341	0.119527	0.089861	0.139039	0.300655	1.171004
0.03554	0.009648	0.374814	0.182659	0.056594	0.019254	0.103581	0.23021	0.37836
0.120274	0.016903	1.955334	0.349051	0.128396	0.09141	0.146841	0.30108	1.211212
0.040811	0.010278	0.353175	0.183035	0.077055	0.022682	0.104726	0.242656	0.413737
0.131248	0.014393	1.88715	0.364594	0.141749	0.087228	0.137437	0.308532	1.246199
0.043291	0.010619	0.356352	0.195038	0.067768	0.022442	0.106504	0.240267	0.429713
0.129849	0.014957	1.802238	0.375958	0.139855	0.08661	0.13788	0.299197	1.270072
0.046274	0.011889	0.363109	0.202841	0.073243	0.022438	0.114344	0.242748	0.465652
0.135701	0.01524	1.825074	0.358341	0.149816	0.08364	0.127237	0.289225	1.275468
0.055128	0.013855	0.354005	0.217028	0.078381	0.031664	0.126785	0.238126	0.497237
0.137556	0.016223	1.782723	0.341348	0.156492	0.094101	0.137398	0.278378	1.293258
0.035544	0.00972	0.36092	0.176569	0.056536	0.017988	0.098828	0.224844	0.373224
0.121266	0.015107	1.951515	0.352817	0.131728	0.089835	0.142249	0.293429	1.213447
0.022151	0.007882	0.344782	0.141791	0.052737	0.010101	0.081145	0.186018	0.38083
0.107518	0.016216	2.592419	0.402364	0.110973	0.085826	0.154497	0.257785	1.262391
0.025662	0.009028	0.333664	0.149348	0.053642	0.010843	0.084585	0.187096	0.415877
0.112317	0.01629	2.478974	0.410247	0.121543	0.08348	0.155015	0.278831	1.295523
0.025129	0.008811	0.311272	0.15261	0.051478	0.013359	0.083651	0.185954	0.431836
0.109506	0.014371	2.033009	0.37977	0.124211	0.091301	0.157377	0.279485	1.27864
0.02567	0.008289	0.302211	0.150771	0.045475	0.01453	0.084631	0.193431	0.441828
0.114529	0.013211	2.073345	0.371042	0.121374	0.086612	0.162816	0.278342	1.302679
0.029288	0.010219	0.303862	0.154487	0.056339	0.018374	0.09011	0.193111	0.465468
0.109958	0.014596	1.925735	0.397467	0.124249	0.090401	0.14726	0.28138	1.321018
0.03196	0.010363	0.322407	0.177416	0.068847	0.021627	0.098694	0.201488	0.495302
0.117599	0.016312	2.01458	0.388768	0.129012	0.094079	0.157067	0.280208	1.330892
0.036106	0.010333	0.310149	0.175453	0.075816	0.025361	0.098936	0.214515	0.522998
0.125216	0.014954	1.96024	0.381718	0.146479	0.096185	0.144024	0.282692	1.370469
0.03915	0.010491	0.314001	0.179612	0.077079	0.028089	0.100893	0.212052	0.54237
0.127846	0.018971	1.904658	0.400159	0.14601	0.09633	0.144578	0.277306	1.393761
0.042365	0.011642	0.316627	0.184365	0.081462	0.031931	0.104189	0.214116	0.580282
0.131401	0.016518	1.892844	0.391915	0.147273	0.101852	0.137244	0.263314	1.383797
0.04845	0.012899	0.303663	0.19837	0.090026	0.046864	0.11156	0.206164	0.605011
0.129341	0.017607	1.860625	0.365765	0.162216	0.121686	0.14068	0.249341	1.353392
0.0321	0.009909	0.316694	0.165376	0.064481	0.021427	0.093259	0.198908	0.484051
0.120006	0.01604	2.035244	0.387975	0.135943	0.096284	0.148879	0.272444	1.336722
0.058997	0.011785	0.842516	0.233484	0.086346	0.044331	0.110277	0.221408	0.744941
0	0	0	0	0	0	0	0	0
0	0	0	0	0	0	0	0	0
0	0	0	0	0	0	0	0	0
0	0	0	0	0	0	0	0	0
0	0	0	0	0	0	0	0	0
0	0	0	0	0	0	0	0	0
0	0	0	0	0	0	0	0	0
0	0	0	0	0	0	0	0	0
0	0	0	0	0	0	0	0	0
0	0	0	0	0	0	0	0	0
0	0	0	0	0	0	0	0	0
0	0	0	0	0	0	0	0	0
0	0	0	0	0	0	0	0	0
0	0	0	0	0	0	0	0	0

For peer review only

0	0	0	0	0	0	0	0	0
0	0	0	0	0	0	0	0	0
0	0	0	0	0	0	0	0	0
0	0	0	0	0	0	0	0	0
0	0	0	0	0	0	0	0	0
0	0	0	0	0	0	0	0	0
0	0	0	0	0	0	0	0	0
0	0	0	0	0	0	0	0	0
0	0	0	0	0	0	0	0	0
0	0	0	0	0	0	0	0	0
0	0	0	0	0	0	0	0	0
0	0	0	0	0	0	0	0	0
0	0	0	0	0	0	0	0	0
0	0	0	0	0	0	0	0	0
0	0	0	0	0	0	0	0	0
0	0	0	0	0	0	0	0	0
0	0	0	0	0	0	0	0	0
0	0	0	0	0	0	0	0	0
0	0	0	0	0	0	0	0	0
0	0	0	0	0	0	0	0	0
0	0	0	0	0	0	0	0	0
0	0	0	0	0	0	0	0	0
0	0	0	0	0	0	0	0	0
0	0	0	0	0	0	0	0	0
0	0	0	0	0	0	0	0	0
0	0	0	0	0	0	0	0	0
0	0	0	0	0	0	0	0	0
0	0	0	0	0	0	0	0	0
0	0	0	0	0	0	0	0	0
0	0	0	0	0	0	0	0	0
0	0	0	0	0	0	0	0	0
0	0	0	0	0	0	0	0	0
0	0	0	0	0	0	0	0	0
0	0	0	0	0	0	0	0	0
0	0	0	0	0	0	0	0	0
0	0	0	0	0	0	0	0	0
0	0	0	0	0	0	0	0	0
0	0	0	0	0	0	0	0	0
0	0	0	0	0	0	0	0	0
0	0	0	0	0	0	0	0	0
0	0	0	0	0	0	0	0	0
0	0	0	0	0	0	0	0	0
0	0	0	0	0	0	0	0	0
0	0	0	0	0	0	0	0	0
0	0	0	0	0	0	0	0	0
0	0	0	0	0	0	0	0	0
0	0	0	0	0	0	0	0	0
0	0	0	0	0	0	0	0	0
0	0	0	0	0	0	0	0	0
0	0	0	0	0	0	0	0	0

For peer review only

0	0	0	0	0	0	0	0	0
0	0	0	0	0	0	0	0	0
0	0	0	0	0	0	0	0	0
0.230792	0.175965	2.333179	1.333495	1.60647	0.220566	2.134165	0.614141	1.519509
0.571489	0.187765	7.643945	4.751326	1.113488	0.418428	0.826387	0.716548	2.650713
0.231018	0.12976	2.25699	1.678535	1.608845	0.227771	0.541433	0.640879	1.613277
0.556336	0.184945	8.029279	3.608501	1.97123	0.478572	0.752077	0.782734	2.753447
0.266392	0.165634	2.262611	1.505966	1.529528	0.212216	1.147733	0.582474	1.648018
0.58012	0.179912	5.805775	4.041207	1.43015	0.460845	0.822182	0.699535	2.68662
0.247749	0.155172	2.054445	1.573823	1.077153	0.231162	0.876693	0.642487	1.647871
0.57778	0.199988	5.990244	4.505199	1.052615	0.572815	1.140847	0.705978	2.763253
0.261331	0.910854	2.062573	1.117822	1.30602	0.366319	1.600913	0.661005	1.731801
0.540771	0.216038	5.729766	3.378423	0.833186	0.521989	0.976514	0.720675	2.921626
0.292358	0.349403	1.996117	2.782163	1.545755	0.431645	0.996915	0.62131	1.821876
0.586331	0.343584	6.2395	3.792923	0.840741	0.593168	0.997021	0.754343	2.723394
0.281285	0.232587	1.913255	1.707741	1.486339	0.358611	1.164636	0.699992	1.836605
0.582371	0.19661	5.859959	3.326421	1.454984	0.626505	0.91125	0.706926	2.946004
0.321459	0.163331	2.314019	2.475764	1.668335	0.433376	0.931178	0.700122	1.894178
0.765409	1.274043	6.251932	3.285676	0.995937	0.695793	1.43561	0.709614	3.069226
0.334273	0.243425	2.077137	1.434603	1.545043	0.532511	0.768026	0.684113	2.300365
0.615184	0.269337	5.820038	3.542813	0.907194	0.711217	0.869848	0.696148	3.048583
0.373463	0.182341	1.843753	2.744422	1.788316	0.616253	0.656313	0.651455	3.02197
0.699619	0.386397	5.88204	3.726093	1.358842	0.919547	0.804935	0.654621	2.824426
0.284529	0.351715	2.124434	1.898406	1.521973	0.378522	1.185093	0.649821	1.920883
0.620343	0.505404	6.266448	3.763073	1.214683	0.641376	0.987165	0.711962	2.864078
0.29701	0.10858	2.891878	1.457956	1.078003	0.1635	0.69937	0.679934	1.096574
0.490446	0.232735	8.071861	2.888655	0.890533	0.506707	1.022772	0.708715	2.241084
0.298246	0.154371	2.560022	1.075344	0.875203	0.167377	2.825476	0.713345	1.210603
0.538871	0.252498	7.868462	3.395343	0.638288	0.47732	1.055346	0.746712	2.223521
0.25997	0.122846	2.601747	1.890895	0.917357	0.332469	0.704351	0.732196	1.594599
0.50075	0.195955	6.776997	3.13512	1.519512	0.558693	0.799565	0.823391	2.207223
0.261158	0.122617	2.577808	1.34893	0.683076	0.236381	0.821406	0.725898	1.301769
0.527947	0.162398	6.926511	2.329253	0.719958	0.476726	1.256148	0.767455	2.315119
0.295508	0.116462	2.556902	1.134573	0.900374	0.349183	0.789523	0.728222	1.315024
0.712235	0.176949	5.829402	4.757346	0.797975	0.494239	0.905876	0.751281	2.294817
0.300582	0.129127	2.8398	1.824138	0.923	0.310565	0.855584	0.745438	1.669348
0.558452	0.21496	6.376546	3.297313	0.927307	0.527944	0.894174	0.798772	2.315047
0.350719	0.133335	2.4566	1.211907	3.230293	0.387579	0.838621	0.790819	1.999533
0.609039	0.163842	6.27416	4.392373	1.491322	0.504566	0.819659	0.770184	2.955601
0.372335	0.146907	2.465375	2.213014	1.136021	0.294312	0.703398	0.738853	1.641226
0.575219	0.225647	5.919553	3.227816	0.964179	0.511163	0.730428	0.789482	2.755117
0.348778	0.142121	2.606975	1.917113	1.133772	0.303294	1.050497	0.745342	1.763048
0.615573	0.184202	6.038163	2.823089	0.980768	0.462781	0.717078	0.743733	2.545122
0.668872	0.214948	2.373401	1.832936	1.094112	0.365151	0.978128	0.766222	1.624463
0.610998	0.200608	5.816483	2.351283	0.847634	0.531521	0.992959	0.710322	2.639924
0.355575	0.140415	2.602486	1.623215	1.382264	0.297838	1.214474	0.736248	1.53847
0.583764	0.200746	6.509998	3.347996	1.025351	0.505765	0.917217	0.76208	2.496747
0.268365	0.143802	2.647933	1.40165	1.34903	0.192076	1.540763	0.650586	1.314541
0.529663	0.21302	7.876587	3.868175	1.000329	0.467927	0.936658	0.712411	2.444166
0.269018	0.143424	2.423813	1.389519	1.271129	0.197839	2.097256	0.68087	1.417827

For peer review only

0.547078	0.223581	7.945286	3.496607	1.424084	0.477924	0.926494	0.763791	2.490775
0.262992	0.1444	2.449779	1.722026	1.24121	0.282788	0.93772	0.667024	1.624257
0.538617	0.18877	6.350573	3.579192	1.479208	0.516212	0.810016	0.769382	2.443615
0.254935	0.13891	2.346306	1.459282	0.890743	0.233937	0.847915	0.688485	1.479313
0.551677	0.18085	6.508282	3.513321	0.890205	0.523591	1.203949	0.739739	2.537399
0.280084	0.628231	2.339458	1.126854	1.108461	0.357293	1.236575	0.698094	1.5286
0.637452	0.196287	5.784157	4.166795	0.814714	0.50746	0.939754	0.737339	2.612943
0.29683	0.255794	2.486043	2.317043	1.250293	0.371516	0.923661	0.691491	1.746111
0.571895	0.283558	6.311935	3.542108	0.887147	0.559943	0.944581	0.778241	2.520809
0.320739	0.185632	2.224068	1.460715	2.580056	0.374562	1.001642	0.751086	1.930048
0.59672	0.179942	6.084023	3.929037	1.474402	0.565071	0.863812	0.741718	2.954051
0.349926	0.154663	2.397508	2.337361	1.405688	0.364848	0.815923	0.721977	1.766101
0.671698	0.890373	6.079567	3.255322	0.979307	0.605418	1.119933	0.753206	2.910477
0.342179	0.19555	2.377472	1.711331	1.339558	0.424955	0.930793	0.718371	2.03277
0.615402	0.228507	5.936263	3.18442	0.946639	0.593838	0.793161	0.722103	2.79717
0.551587	0.200447	2.144109	2.302221	1.45868	0.498195	0.844034	0.715964	2.381565
0.655417	0.304962	5.848802	3.093687	1.124348	0.74545	0.906883	0.684596	2.731422
0.324549	0.260766	2.3929	1.756401	1.448834	0.337727	1.200936	0.698008	1.730693
0.601258	0.376231	6.397141	3.549591	1.118465	0.573637	0.950803	0.739251	2.679105
0.430529	0.300851	4.137926	2.450846	1.356722	0.425479	1.130586	0.711688	2.1046
1276	561	19880	8901	4282	673	5886	10985	33636
2221	312	53602	9591	2248	1429	3183	4912	27998
1551	604	19872	9829	4797	752	4762	11232	37385
2412	324	55380	9740	2914	1633	3271	5559	31049
1521	598	17164	9357	3877	839	5432	10413	36916
2714	319	49089	9803	2934	1917	3787	5981	33751
1565	585	16624	8971	3232	1011	5264	10756	37223
2973	369	53912	11261	3195	2232	4360	6511	36718
1673	757	16705	9055	4136	1330	5614	10710	38261
3076	446	57173	11501	3595	2525	4343	7203	41348
1741	701	16377	10729	5203	1527	5825	10528	39527
3309	452	60025	12482	3743	2800	4858	7424	42213
1905	648	16172	10378	4634	1777	5741	11304	40609
3728	491	64344	12633	4782	3349	4771	7980	47598
2101	634	16191	9897	5398	2130	5780	10962	41388
4261	796	68503	14494	5187	3637	5159	8576	51979
2228	669	15466	9595	5364	2533	5442	10651	42059
4454	631	69233	15099	5081	4295	5219	8249	52913
2187	633	13183	9490	5548	3452	5045	9087	39106
4290	679	69159	13941	5989	5377	5130	7769	50437
17748	6390	167634	96202	46471	16024	54791	106628	386110
33438	4819	600420	120545	39668	29194	44081	70164	416004
1928	579	29990	11608	3346	788	5851	15921	21448
2354	378	56708	7530	2474	2223	3391	6057	25718
2224	724	29212	12141	3094	843	7681	16291	23793
2628	407	55859	8669	2540	2113	3685	6953	27085
2021	644	26711	12154	3379	1044	6359	15798	23953
2856	412	54320	9514	3384	2727	4218	8235	31287
2046	581	25888	12238	3165	1033	6641	16454	24929
3234	347	58455	8848	3383	2462	4464	8574	33882

For peer review only

2371	654	25251	12276	3643	1207	6828	15954	26009
3418	416	56559	11973	3743	2814	4354	9415	36670
2586	702	27273	13291	4118	1401	7537	16751	27531
3821	537	62119	11089	4079	2904	4665	9565	38479
2994	754	25910	13428	5653	1664	7683	17802	30353
4687	514	67392	13020	5062	3115	4908	11018	44503
3127	767	25740	14088	4895	1621	7693	17355	31039
4931	568	68440	14277	5311	3289	5236	11362	48231
3176	816	24922	13922	5027	1540	7848	16661	31960
5307	596	71375	14014	5859	3271	4976	11311	49881
3402	855	21846	13393	4837	1954	7824	14695	30685
5223	616	67690	12961	5942	3573	5217	10570	49105
25875	7076	262743	128539	41157	13095	71945	163682	271700
38459	4791	618917	111895	41777	28491	45114	93060	384841
3204	1140	49870	20509	7628	1461	11737	26906	55084
4575	690	110310	17121	4722	3652	6574	10969	53716
3775	1328	49084	21970	7891	1595	12443	27523	61178
5040	731	111239	18409	5454	3746	6956	12512	58134
3542	1242	43875	21511	7256	1883	11791	26211	60869
5570	731	103409	19317	6318	4644	8005	14216	65038
3611	1166	42512	21209	6397	2044	11905	27210	62152
6207	716	112367	20109	6578	4694	8824	15085	70600
4044	1411	41956	21331	7779	2537	12442	26664	64270
6494	862	113732	23474	7338	5339	8697	16618	78018
4327	1403	43650	24020	9321	2928	13362	27279	67058
7130	989	122144	23571	7822	5704	9523	16989	80692
4899	1402	42082	23806	10287	3441	13424	29106	70962
8415	1005	131736	25653	9844	6464	9679	18998	92101
5228	1401	41931	23985	10293	3751	13473	28317	72427
9192	1364	136943	28771	10498	6926	10395	19938	100210
5404	1485	40388	23517	10391	4073	13290	27312	74019
9761	1227	140608	29113	10940	7566	10195	19560	102794
5589	1488	35029	22883	10385	5406	12869	23782	69791
9513	1295	136849	26902	11931	8950	10347	18339	99542
43623	13466	430377	224741	87628	29119	126736	270310	657810
71897	9610	1219337	232440	81445	57685	89195	163224	800845
115520	23076	1649714	457181	169073	86804	215931	433534	1458655

For peer review only

Hospital ICD10 Admissions

J	K	L	M	N	O	P	Q	R
3574	15522	2986	9518	7240	<10	<10	210	12744
5800	6409	1260	2721	4188	<10	<10	76	8567
4172	17435	3413	10205	7851	<10	<10	234	14252
6321	7222	1424	2967	4501	<10	<10	70	9348
4290	16820	3123	10482	7367	<10	<10	228	13495
7428	7826	1553	3116	5034	<10	<10	80	10365
4328	16820	3501	10468	7357	<10	<10	220	13365
8088	8405	1659	3529	5118	<10	<10	73	11053
4570	17133	3449	10425	7519	<10	<10	237	13854
9010	9637	1859	3756	5699	<10	<10	88	12361
4770	17098	3578	10312	7367	<10	<10	255	14358
9482	10113	2139	3961	5823	<10	<10	104	13156
5072	17155	3765	10248	7560	<10	<10	229	14886
10914	11080	2259	4130	6228	<10	<10	92	14383
5264	17079	3762	9765	7431	<10	<10	287	15658
12021	12020	2414	4344	6685	<10	<10	97	15843
5374	16918	3593	9383	7192	<10	<10	207	16036
12512	12806	2433	4280	6595	<10	<10	93	16556
5290	16069	3390	8216	6531	<10	<10	217	15737
13235	13238	2476	4203	6240	<10	<10	107	17407
46704	168049	34560	99022	73415	<10	<10	2324	144385
94811	98756	19476	37007	56111	<10	<10	880	129039
3695	15668	3016	12344	11755	428	<10	265	14018
5758	6615	1456	3668	4440	<10	<10	78	9189
4214	18276	3422	13063	12985	398	<10	269	16138
6364	7401	1557	4092	4747	<10	<10	60	10271
4254	17462	3327	12865	12628	356	<10	248	15210
7538	8421	1885	4760	5515	<10	<10	63	11733
4516	17454	3460	13002	12538	380	<10	265	15145
8160	8888	1947	4837	5645	<10	<10	59	12232
4985	18743	3704	13127	12852	342	<10	285	16016
9023	10000	2129	5063	6041	<10	<10	85	13231
5339	19675	3816	13413	13155	363	<10	272	17521
9504	10306	2216	5181	6149	<10	<10	84	13696
5982	20433	3962	13937	13694	311	<10	296	18596
11324	11659	2475	5828	7087	<10	<10	102	15600
6568	20650	4051	13628	13687	298	<10	292	19408
12587	12819	2592	6001	7511	<10	<10	106	17011
6972	20815	3880	13074	13245	302	<10	313	19694
13302	13588	2760	6026	7719	17	<10	100	17666
7353	20193	3721	11834	12551	314	<10	273	20172
13852	13685	2674	5641	7805	<10	<10	119	17916
53878	189369	36359	130287	129090	3492	<10	2778	171918
97412	103382	21691	51097	62659	70	<10	856	138545
7269	31190	6002	21862	18995	428	<10	475	26762
11558	13024	2716	6389	8628	<10	<10	154	17756
8386	35711	6835	23268	20836	398	<10	503	30390
12685	14623	2981	7059	9248	<10	<10	130	19619

For peer review only

8544	34282	6450	23347	19995	356	<10	476	28705
14966	16247	3438	7876	10549	<10	<10	143	22098
8844	34274	6961	23470	19895	380	<10	485	28510
16248	17293	3606	8366	10763	<10	<10	132	23285
9555	35876	7153	23552	20371	342	<10	522	29870
18033	19637	3988	8819	11740	<10	<10	173	25592
10109	36773	7394	23725	20522	363	<10	527	31879
18986	20419	4355	9142	11972	<10	<10	188	26852
11054	37588	7727	24185	21254	311	<10	525	33482
22238	22739	4734	9958	13315	<10	<10	194	29983
11832	37729	7813	23393	21118	298	<10	579	35066
24608	24839	5006	10345	14196	<10	<10	203	32854
12346	37733	7473	22457	20437	302	<10	520	35730
25814	26394	5193	10306	14314	17	<10	193	34222
12643	36262	7111	20050	19082	314	<10	490	35909
27087	26923	5150	9844	14045	<10	<10	226	35323
100582	357418	70919	229309	202505	3492	<10	5102	316303
192223	202138	41167	88104	118770	70	<10	1736	267584
292805	559556	112086	317413	321275	3562	<10	6838	583887
0.103106	0.449245	0.066272	0.244329	0.1964	<0.005	<0.005	0.005443	0.333522
0.825064	0.799019	0.117382	0.256815	0.471856	<0.005	<0.005	0.006676	1.007788
0.116801	0.50853	0.072341	0.261746	0.213115	<0.005	<0.005	0.005949	0.378955
0.876387	0.868925	0.124378	0.262294	0.513777	<0.005	<0.005	0.005645	1.059223
0.127459	0.50941	0.069354	0.293426	0.209538	<0.005	<0.005	0.006187	0.371948
0.943213	0.827193	0.128973	0.248938	0.48857	<0.005	<0.005	0.006262	1.042033
0.128293	0.509732	0.077914	0.286943	0.204774	<0.005	<0.005	0.005537	0.36789
0.936493	0.84024	0.112844	0.255735	0.490236	<0.005	<0.005	0.006113	1.036953
0.150075	0.542005	0.07945	0.292474	0.216482	<0.005	<0.005	0.006467	0.408322
1.002127	0.876081	0.124171	0.248775	0.4898	<0.005	<0.005	0.005046	1.05439
0.163611	0.576616	0.087219	0.309626	0.229289	<0.005	<0.005	0.006914	0.460606
1.025363	0.904993	0.139011	0.253491	0.466893	<0.005	<0.005	0.00589	1.104432
0.177712	0.598219	0.094769	0.302359	0.23206	<0.005	<0.005	0.006162	0.484194
1.1373	0.916045	0.142032	0.240784	0.464135	<0.005	<0.005	<0.005	1.15505
0.196376	0.63183	0.102616	0.295469	0.241624	<0.005	<0.005	0.007405	0.543046
1.183557	0.948031	0.158501	0.235084	0.455371	<0.005	<0.005	<0.005	1.221937
0.22635	0.661671	0.116968	0.299362	0.252045	<0.005	<0.005	0.006432	0.613727
1.238316	0.995508	0.169462	0.23172	0.453121	<0.005	<0.005	0.005458	1.257875
0.262434	0.730333	0.1158	0.294311	0.251007	<0.005	<0.005	0.006748	0.720863
1.377909	1.076363	0.13932	0.217482	0.423384	<0.005	<0.005	<0.005	1.385638
0.162068	0.566621	0.087192	0.287305	0.223537	<0.005	<0.005	0.006301	0.460666
1.085841	0.919153	0.138301	0.242843	0.467946	<0.005	<0.005	0.005221	1.152608
0.100045	0.398569	0.05541	0.317355	0.248566	0.006277	<0.005	0.006857	0.324457
0.713452	0.683335	0.116648	0.333392	0.427167	<0.005	<0.005	0.005839	0.930898
0.114252	0.467311	0.064779	0.334225	0.271037	0.006036	<0.005	0.005425	0.378056
0.763987	0.749426	0.125162	0.350912	0.427385	<0.005	<0.005	<0.005	1.021486
0.120823	0.480206	0.063824	0.341	0.270339	0.005427	<0.005	0.006026	0.380786
0.767913	0.733612	0.117132	0.335572	0.430018	<0.005	<0.005	<0.005	1.008566
0.135297	0.493897	0.069403	0.348183	0.275809	0.00621	<0.005	0.006036	0.385485
0.824185	0.73457	0.124578	0.322392	0.436866	<0.005	<0.005	<0.005	0.968864
0.14891	0.536382	0.077206	0.367036	0.285454	0.005585	<0.005	0.006278	0.425749

0.872872	0.770972	0.157401	0.330161	0.43088	<0.005	<0.005	0.005109	1.001022
0.174289	0.592876	0.082046	0.383775	0.313782	0.005745	<0.005	0.006171	0.488071
0.967767	0.812081	0.132519	0.337688	0.440398	<0.005	<0.005	<0.005	1.061349
0.20434	0.629663	0.082712	0.393877	0.332565	<0.005	<0.005	0.006556	0.527132
1.082272	0.836353	0.168576	0.339727	0.448797	<0.005	<0.005	0.005489	1.097281
0.235851	0.653976	0.099928	0.401872	0.343352	<0.005	<0.005	0.006175	0.582221
1.168611	0.891718	0.151942	0.329717	0.452192	<0.005	<0.005	<0.005	1.13738
0.280251	0.719152	0.113616	0.430946	0.349064	<0.005	<0.005	0.006513	0.646303
1.229621	0.92109	0.172292	0.331953	0.456198	<0.005	<0.005	<0.005	1.19303
0.364327	0.807457	0.104876	0.42174	0.384162	0.006028	<0.005	0.007276	0.79976
1.462865	0.973189	0.165051	0.322307	0.493758	<0.005	<0.005	0.005109	1.3138
0.183065	0.571027	0.080435	0.372084	0.30511	0.005591	<0.005	0.006311	0.485522
1.022747	0.825135	0.146402	0.332779	0.446995	<0.005	<0.005	<0.005	1.08958
0.101464	0.422063	0.060446	0.2835	0.224382	<0.005	<0.005	0.006202	0.32866
0.765317	0.737092	0.116989	0.297807	0.447933	<0.005	<0.005	0.006228	0.966628
0.115441	0.486527	0.068304	0.300436	0.244035	<0.005	<0.005	0.005669	0.378475
0.816348	0.805094	0.124797	0.309629	0.467631	<0.005	<0.005	<0.005	1.039066
0.12392	0.493835	0.066405	0.318799	0.241965	<0.005	<0.005	0.006101	0.376662
0.848265	0.776506	0.12256	0.295862	0.456856	<0.005	<0.005	<0.005	1.023906
0.131997	0.501358	0.073413	0.319329	0.24234	<0.005	<0.005	0.005801	0.377195
0.876393	0.783693	0.119123	0.291405	0.461676	<0.005	<0.005	<0.005	1.000517
0.149454	0.539008	0.078254	0.332216	0.253245	<0.005	<0.005	0.006366	0.417611
0.933592	0.820349	0.14179	0.291928	0.458558	<0.005	<0.005	0.00508	1.026093
0.16935	0.585355	0.084439	0.349477	0.274699	<0.005	<0.005	0.006515	0.475367
0.995184	0.856309	0.135609	0.297608	0.45301	<0.005	<0.005	0.005129	1.081857
0.19211	0.615221	0.08825	0.351842	0.286403	<0.005	<0.005	0.006375	0.507411
1.108059	0.873698	0.156137	0.293361	0.455985	<0.005	<0.005	<0.005	1.124353
0.217728	0.643809	0.101162	0.353023	0.29665	<0.005	<0.005	0.00674	0.564236
1.175663	0.918288	0.155037	0.285067	0.453692	<0.005	<0.005	<0.005	1.177276
0.255353	0.6926	0.115164	0.370164	0.304248	<0.005	<0.005	0.006476	0.631255
1.233738	0.95633	0.170952	0.284489	0.454741	<0.005	<0.005	<0.005	1.223736
0.316943	0.771592	0.109956	0.362481	0.32224	<0.005	<0.005	0.00703	0.763071
1.421768	1.0231	0.152604	0.271598	0.459714	<0.005	<0.005	<0.005	1.348552
0.173316	0.568981	0.083572	0.33272	0.267234	<0.005	<0.005	0.006306	0.473981
1.052441	0.869383	0.14259	0.290452	0.456855	<0.005	<0.005	<0.005	1.119243
0.442301	0.660895	0.10163	0.319787	0.325252	<0.005	<0.005	0.00586	0.671411
0	0	0	0	0	0	0	0	0
0	0	0	0	0	0	0	0	0
0	0	0	0	0	0	0	0	0
0	0	0	0	0	0	0	0	0
0	0	0	0	0	0	0	0	0
0	0	0	0	0	0	0	0	0
0	0	0	0	0	0	0	0	0
0	0	0	0	0	0	0	0	0
0	0	0	0	0	0	0	0	0
0	0	0	0	0	0	0	0	0
0	0	0	0	0	0	0	0	0
0	0	0	0	0	0	0	0	0
0	0	0	0	0	0	0	0	0
0	0	0	0	0	0	0	0	0

For peer review only

0	0	0	0	0	0	0	0	0
0	0	0	0	0	0	0	0	0
0	0	0	0	0	0	0	0	0
0	0	0	0	0	0	0	0	0
0	0	0	0	0	0	0	0	0
0	0	0	0	0	0	0	0	0
0	0	0	0	0	0	0	0	0
0	0	0	0	0	0	0	0	0
0	0	0	0	0	0	0	0	0
0	0	0	0	0	0	0	0	0
0	0	0	0	0	0	0	0	0
0	0	0	0	0	0	0	0	0
0	0	0	0	0	0	0	0	0
0	0	0	0	0	0	0	0	0
0	0	0	0	0	0	0	0	0
0	0	0	0	0	0	0	0	0
0	0	0	0	0	0	0	0	0
0	0	0	0	0	0	0	0	0
0	0	0	0	0	0	0	0	0
0	0	0	0	0	0	0	0	0
0	0	0	0	0	0	0	0	0
0	0	0	0	0	0	0	0	0
0	0	0	0	0	0	0	0	0
0	0	0	0	0	0	0	0	0
0	0	0	0	0	0	0	0	0
0	0	0	0	0	0	0	0	0
0	0	0	0	0	0	0	0	0
0	0	0	0	0	0	0	0	0
0	0	0	0	0	0	0	0	0
0	0	0	0	0	0	0	0	0
0	0	0	0	0	0	0	0	0
0	0	0	0	0	0	0	0	0
0	0	0	0	0	0	0	0	0
0	0	0	0	0	0	0	0	0
0	0	0	0	0	0	0	0	0
0	0	0	0	0	0	0	0	0
0	0	0	0	0	0	0	0	0
0	0	0	0	0	0	0	0	0
0	0	0	0	0	0	0	0	0
0	0	0	0	0	0	0	0	0
0	0	0	0	0	0	0	0	0
0	0	0	0	0	0	0	0	0
0	0	0	0	0	0	0	0	0
0	0	0	0	0	0	0	0	0
0	0	0	0	0	0	0	0	0
0	0	0	0	0	0	0	0	0
0	0	0	0	0	0	0	0	0
0	0	0	0	0	0	0	0	0
0	0	0	0	0	0	0	0	0
0	0	0	0	0	0	0	0	0

For peer review only

0	0	0	0	0	0	0	0	0
0	0	0	0	0	0	0	0	0
0	0	0	0	0	0	0	0	0
0.652815	1.517797	0.421715	0.883727	0.833143	0	0	0.122668	0.966247
2.112886	1.923708	1.051587	1.157877	1.302078	0	0	0.142078	1.870596
0.682466	1.401731	0.468593	0.981801	1.046524	0	0.003819	0.137978	1.145634
2.255003	2.126606	0.723463	1.084323	2.456414	0	0	0.128905	1.840903
0.721251	1.372239	0.495868	1.239466	0.852438	0	0	0.140718	1.083048
2.454465	1.960216	1.155292	0.933473	1.513284	0	0	0.167113	1.899766
0.783967	1.411689	0.450412	1.096371	0.855711	0	0	0.126045	1.106398
2.392983	2.009523	0.615244	0.897902	3.395122	0	0	0.176752	2.061826
1.010021	1.439853	0.741578	1.008903	0.975849	0	0.003938	0.159106	1.234371
2.854021	2.010175	1.128982	0.916414	4.268842	0	0	0.115061	1.898868
0.930506	1.554899	0.551886	1.372313	0.940704	0	0	0.149946	1.405429
2.683278	2.122427	0.892843	0.954391	1.494337	0	0	0.12775	2.002556
0.932285	1.597401	0.576757	1.134737	0.906604	0	0	0.133918	1.375213
3.008426	2.162549	0.946319	0.907705	3.525022	0	0	0.09799	2.478793
1.137399	1.731373	1.374992	1.161053	0.992179	0	0	0.137357	1.582533
3.028649	2.268945	1.712743	0.885946	1.34979	0	0.005429	0.085414	2.766562
1.232513	1.769006	1.662386	1.198346	1.027844	0	0	0.151511	1.775059
3.351433	2.312607	3.459342	1.430406	1.427267	0	0.021984	0.190384	2.472362
1.529114	2.051147	1.625881	1.298484	1.096122	0	0	0.147531	2.375456
3.683731	2.521988	0.9954	0.881289	1.412724	0	0	0.087174	2.789855
0.979422	1.585925	0.935821	1.140629	0.953177	0	0.00178	0.140777	1.435829
2.914442	2.17945	1.593329	1.020296	2.433226	0	0.00799	0.134731	2.303514
0.72109	1.218097	0.382294	2.478803	0.930838	0.089822	0.00359	0.219334	0.987924
2.167329	1.684347	0.614509	1.722866	1.266272	0.017528	0	0.129203	1.690673
0.795336	1.301211	0.494954	1.482452	0.979662	0.092034	0	0.119196	1.1288
2.207334	1.936438	1.269844	1.415297	1.415421	0.024166	0	0.088723	1.813781
0.836271	1.433258	0.434308	1.350182	0.876618	0.083631	0	0.153716	1.133847
2.205972	1.775914	0.609167	1.070776	1.519992	0.025559	0	0.107537	1.862705
0.898315	1.423929	0.521231	1.317526	0.943291	0.099137	0	0.135721	1.192197
2.383527	1.716992	0.777358	1.334759	4.314707	0.01313	0	0.101824	1.774899
0.921587	1.426723	0.597884	2.172975	0.910433	0.089439	0	0.135194	1.270178
2.442602	1.843041	2.965403	1.199804	3.151915	0.013841	0	0.147273	1.789454
1.027899	1.575103	0.765534	1.571088	1.092594	0.08691	0	0.134904	1.370871
2.711194	2.626119	0.783994	1.239248	3.262258	0.015867	0	0.166092	2.03061
1.261428	1.675143	0.579802	2.015062	2.126381	0.079036	0.003692	0.139021	1.451518
3.10808	2.286125	2.523567	1.918958	1.349405	0.011832	0	0.17796	2.022224
1.286477	1.691271	1.364564	1.894098	1.232514	0.07992	0	0.122913	1.552252
3.227416	3.154326	1.887995	1.431505	1.362653	0.012569	0	0.088797	2.086734
1.526945	1.768019	2.206822	2.877347	1.103227	0.077417	0	0.112852	1.717121
3.315194	2.345742	1.976407	1.919271	1.389957	0.027226	0.005057	0.114452	2.198046
1.758752	2.206486	0.667961	2.00773	1.172344	0.09053	0	0.14833	2.199161
3.931403	2.151794	1.95585	1.438418	1.481314	0.017776	0	0.116238	2.377977
1.132428	1.581662	0.948623	1.967317	1.186826	0.087104	0.001658	0.145469	1.424145
2.927259	2.253336	1.802843	1.511628	2.268316	0.018622	0.001776	0.128042	2.009902
0.690277	1.365474	0.401087	1.912908	0.887264	0.065859	0.002629	0.181054	0.977941
2.1429	1.800443	0.846165	1.487734	1.283213	0.012825	0	0.135337	1.776942
0.744848	1.349156	0.482857	1.274292	1.011791	0.06731	0.002607	0.128295	1.136675

For peer review only

2.230348	2.028102	1.051249	1.272629	1.970483	0.017663	0	0.109298	1.826542
0.7847	1.405183	0.46406	1.299901	0.865947	0.061136	0	0.147792	1.110435
2.324794	1.863222	0.901551	1.011072	1.517187	0.018811	0	0.138077	1.879839
0.84637	1.418192	0.48916	1.218717	0.903778	0.07216	0	0.131251	1.152597
2.388562	1.859445	0.706656	1.152932	3.914242	0.009605	0	0.141679	1.913924
0.963892	1.432867	0.66884	1.730162	0.942173	0.065356	0.002691	0.146846	1.253609
2.644623	1.924063	2.293822	1.07677	3.718714	0.010079	0	0.133114	1.84184
0.98406	1.565805	0.67517	1.482916	1.026	0.063778	0	0.14206	1.387025
2.698073	2.400004	0.837573	1.113568	2.576708	0.011486	0	0.149076	2.017402
1.122376	1.639962	0.578434	1.670015	1.680701	0.058167	0.002715	0.1367	1.417138
3.061884	2.229407	1.950341	1.531428	2.605855	0.008625	0	0.146042	2.247924
1.220454	1.709828	1.369357	1.6007	1.129686	0.058826	0	0.129745	1.566342
3.13519	2.772165	1.807415	1.206186	1.35659	0.009135	0.003729	0.087217	2.431648
1.398916	1.7687	1.974075	2.263269	1.070156	0.05684	0	0.132122	1.744192
3.332384	2.330391	2.779059	1.706059	1.407739	0.019756	0.015566	0.155113	2.332179
1.656701	2.135991	1.211616	1.715963	1.139466	0.066283	0	0.147959	2.283167
3.813811	2.338759	1.566611	1.202739	1.448956	0.012773	0	0.103206	2.585661
1.064176	1.583644	0.942706	1.636814	1.08538	0.063813	0.001716	0.143309	1.429635
2.921402	2.219363	1.707447	1.304435	2.347394	0.013549	0.005631	0.131233	2.153308
1.887168	1.807433	1.228631	1.542861	1.584677	0.053703	0.003427	0.139727	1.710442
6914	30125	4444	16384	13170	<10	<10	365	22365
16314	15799	2321	5078	9330	<10	<10	132	19927
8010	34874	4961	17950	14615	<10	<10	408	25988
18320	18164	2600	5483	10740	<10	<10	118	22142
8384	33508	4562	19301	13783	<10	<10	407	24466
21991	19286	3007	5804	11391	<10	<10	146	24295
8503	33784	5164	19018	13572	<10	<10	367	24383
23594	21169	2843	6443	12351	<10	<10	154	26125
9677	34949	5123	18859	13959	<10	<10	417	26329
27803	24306	3445	6902	13589	<10	<10	140	29253
10246	36110	5462	19390	14359	<10	<10	433	28845
29593	26119	4012	7316	13475	<10	<10	170	31875
11075	37281	5906	18843	14462	<10	<10	384	30175
35817	28849	4473	7583	14617	<10	<10	139	36376
12039	38735	6291	18114	14813	<10	<10	454	33292
40151	32161	5377	7975	15448	<10	<10	132	41453
13337	38987	6892	17639	14851	<10	<10	379	36162
43559	35018	5961	8151	15939	<10	<10	192	44247
14078	39178	6212	15788	13465	<10	<10	362	38670
49026	38297	4957	7738	15064	<10	<10	149	49301
102263	357531	55017	181286	141049	<10	<10	3976	290675
306168	259168	38996	68473	131944	<10	<10	1472	324994
7762	30923	4299	24622	19285	487	<10	532	25173
16251	15565	2657	7594	9730	<10	<10	133	21204
8972	36697	5087	26246	21284	474	<10	426	29688
18312	17963	3000	8411	10244	<10	<10	89	24484
9083	36100	4798	25635	20323	408	<10	453	28626
21156	20211	3227	9245	11847	10	<10	107	27786
10065	36742	5163	25902	20518	462	<10	449	28677
23903	21304	3613	9350	12670	<10	<10	95	28099

For peer review only

10959	39475	5682	27012	21008	411	<10	462	31333
27334	24143	4929	10339	13493	<10	<10	160	31347
12682	43140	5970	27925	22832	418	<10	449	35514
30745	25799	4210	10728	13991	<10	<10	141	33718
14991	46194	6068	28896	24398	356	<10	481	38672
38649	29867	6020	12132	16027	<10	<10	196	39185
17036	47238	7218	29028	24801	345	<10	446	42055
44378	33863	5770	12521	17172	<10	<10	148	43192
19235	49359	7798	29578	23958	337	<10	447	44359
48088	36022	6738	12982	17841	21	<10	169	46657
22483	49829	6472	26026	23707	372	<10	449	49354
55545	36952	6267	12238	18748	<10	<10	194	49885
133268	415697	58555	270870	222114	4070	<10	4594	353451
324361	261689	46431	105540	141763	82	<10	1432	345557
14676	61048	8743	41006	32455	487	<10	897	47538
32565	31364	4978	12672	19060	<10	<10	265	41131
16982	71571	10048	44196	35899	474	<10	834	55676
36632	36127	5600	13894	20984	<10	<10	207	46626
17467	69608	9360	44936	34106	408	<10	860	53092
43147	39497	6234	15049	23238	10	<10	253	52081
18568	70526	10327	44920	34090	462	<10	816	53060
47497	42473	6456	15793	25021	<10	<10	249	54224
20636	74424	10805	45871	34967	411	<10	879	57662
55137	48449	8374	17241	27082	<10	<10	300	60600
22928	79250	11432	47315	37191	418	<10	882	64359
60338	51918	8222	18044	27466	<10	<10	311	65593
26066	83475	11974	47739	38860	356	<10	865	68847
74466	58716	10493	19715	30644	<10	<10	335	75561
29075	85973	13509	47142	39614	345	<10	900	75347
84529	66024	11147	20496	32620	<10	<10	280	84645
32572	88346	14690	47217	38809	337	<10	826	80521
91647	71040	12699	21133	33780	21	<10	361	90904
36561	89007	12684	41814	37172	372	<10	811	88024
104571	75249	11224	19976	33812	<10	<10	343	99186
235531	773228	113572	452156	363163	4070	<10	8570	644126
630529	520857	85427	174013	273707	82	<10	2904	670551
866060	1294085	198999	626169	636870	4152	11	11474	1314677

For peer review only

S	T	W	Z
4597	2569	<10	7030
3009	1829	<10	3399
4984	2815	<10	7987
3272	1963	<10	3833
5257	2817	<10	7671
3793	2207	<10	4382
5721	3055	<10	7823
4054	2356	<10	4551
5921	3034	<10	7860
4431	2507	<10	5135
5864	3058	<10	7965
4672	2684	<10	5294
6105	3400	<10	8007
5219	2869	<10	5601
6517	3437	<10	7687
5561	3108	<10	5882
6572	3382	<10	7705
6251	3287	<10	5903
6984	3377	<10	6971
7081	3381	<10	5690
58522	30944	<10	76706
47343	26191	<10	49670
6576	2991	<10	6703
6528	1670	<10	2874
6769	3103	<10	7876
6819	1799	<10	3203
6623	3159	<10	7621
7897	1965	<10	3834
6814	3268	<10	7587
8075	2084	<10	3920
6937	3483	<10	8237
8486	2303	<10	4388
6949	3705	<10	8489
8128	2424	<10	4356
7419	4016	<10	8773
9176	2625	<10	4931
7409	4146	<10	8569
9610	2840	<10	4972
7232	4206	<10	8681
9617	2833	<10	5016
6921	4165	<10	7985
9284	3017	<10	4685
69649	36242	<10	80521
83620	23560	<10	42179
11173	5560	<10	13733
9537	3499	<10	6273
11753	5918	<10	15863
10091	3762	<10	7036

For peer review only

11880	5976	<10	15292
11690	4172	<10	8216
12535	6323	<10	15410
12129	4440	<10	8471
12858	6517	<10	16097
12917	4810	<10	9523
12813	6763	<10	16454
12800	5108	<10	9650
13524	7416	<10	16780
14395	5494	<10	10532
13926	7583	<10	16256
15171	5948	<10	10854
13804	7588	<10	16386
15868	6120	<10	10919
13905	7542	<10	14956
16365	6398	<10	10375
128171	67186	<10	157227
130963	49751	<10	91849
259134	116937	<10	249076
0.097708	0.06669	<0.005	0.275721
0.289991	0.191119	<0.005	0.337329
0.099959	0.074193	<0.005	0.288904
0.294202	0.190825	<0.005	0.346824
0.114278	0.075116	<0.005	0.284542
0.307656	0.183187	<0.005	0.348016
0.124838	0.083738	<0.005	0.283742
0.302096	0.187108	<0.005	0.333849
0.135017	0.085483	<0.005	0.300495
0.304859	0.177191	<0.005	0.340542
0.140154	0.088576	<0.005	0.314319
0.307439	0.184055	<0.005	0.344513
0.148203	0.10053	<0.005	0.314253
0.316356	0.183628	<0.005	0.329089
0.167977	0.104704	<0.005	0.299925
0.321189	0.185385	<0.005	0.325787
0.178117	0.109416	<0.005	0.318391
0.33864	0.181317	<0.005	0.309359
0.215905	0.124021	<0.005	0.315423
0.387071	0.189376	<0.005	0.287521
0.139927	0.090167	<0.005	0.299173
0.321112	0.18502	<0.005	0.327574
0.132912	0.065812	<0.005	0.211849
0.598867	0.143691	<0.005	0.207744
0.128438	0.06995	<0.005	0.231395
0.587592	0.149068	<0.005	0.229171
0.134192	0.073973	<0.005	0.232624
0.57637	0.134519	<0.005	0.228022
0.142314	0.077697	<0.005	0.229735
0.549204	0.137508	<0.005	0.221502
0.145186	0.085685	<0.005	0.244656

For peer review only

0.545426	0.138688	<0.005	0.224717
0.153001	0.094937	<0.005	0.253941
0.530391	0.145016	<0.005	0.228462
0.16248	0.10215	<0.005	0.262989
0.523508	0.145193	<0.005	0.233738
0.16735	0.10865	<0.005	0.251419
0.518394	0.145964	<0.005	0.221725
0.172128	0.117549	<0.005	0.269329
0.496701	0.141608	<0.005	0.214048
0.187633	0.135989	<0.005	0.275468
0.492257	0.163208	<0.005	0.204161
0.151504	0.091975	<0.005	0.246183
0.5359	0.144895	<0.005	0.221084
0.116591	0.066219	<0.005	0.24132
0.455336	0.165731	<0.005	0.267961
0.115162	0.071928	<0.005	0.258037
0.450917	0.16852	<0.005	0.283979
0.124899	0.074507	<0.005	0.256689
0.4532	0.156827	<0.005	0.283024
0.13408	0.080543	<0.005	0.254884
0.434331	0.160565	<0.005	0.273729
0.140437	0.085591	<0.005	0.270381
0.432415	0.156775	<0.005	0.279128
0.147059	0.091995	<0.005	0.281325
0.424262	0.163599	<0.005	0.283704
0.155922	0.101405	<0.005	0.286039
0.426433	0.163205	<0.005	0.278421
0.167638	0.106839	<0.005	0.273131
0.425347	0.164564	<0.005	0.270824
0.174894	0.113792	<0.005	0.291273
0.421854	0.160411	<0.005	0.259181
0.20078	0.130423	<0.005	0.293415
0.441373	0.175867	<0.005	0.244487
0.146128	0.091135	<0.005	0.270362
0.434813	0.163779	<0.005	0.271202
0.234457	0.113362	<0.005	0.270689
0	0	0	0
0	0	0	0
0	0	0	0
0	0	0	0
0	0	0	0
0	0	0	0
0	0	0	0
0	0	0	0
0	0	0	0
0	0	0	0
0	0	0	0
0	0	0	0
0	0	0	0
0	0	0	0

For peer review only

0	0	0	0
0	0	0	0
0	0	0	0
0	0	0	0
0	0	0	0
0	0	0	0
0	0	0	0
0	0	0	0
0	0	0	0
0	0	0	0
0	0	0	0
0	0	0	0
0	0	0	0
0	0	0	0
0	0	0	0
0	0	0	0
0	0	0	0
0	0	0	0
0	0	0	0
0	0	0	0
0	0	0	0
0	0	0	0
0	0	0	0
0	0	0	0
0	0	0	0
0	0	0	0
0	0	0	0
0	0	0	0
0	0	0	0
0	0	0	0
0	0	0	0
0	0	0	0
0	0	0	0
0	0	0	0
0	0	0	0
0	0	0	0
0	0	0	0
0	0	0	0
0	0	0	0
0	0	0	0
0	0	0	0
0	0	0	0
0	0	0	0
0	0	0	0
0	0	0	0
0	0	0	0
0	0	0	0
0	0	0	0
0	0	0	0
0	0	0	0

For peer review only

0	0	0	0
0	0	0	0
0	0	0	0
0.452561	0.501099	0	1.015114
0.883435	0.859217	0	1.17199
0.444013	0.579965	0	1.016633
0.875295	0.91519	0	1.142975
0.48958	0.503629	0	0.998756
0.91601	0.773771	0	1.118949
0.517787	0.700835	0	0.943663
0.895743	0.856374	0	1.118775
0.582714	0.577048	0	1.04093
0.925037	0.817414	0	1.182216
0.551007	0.599379	0	1.057303
0.919397	0.836003	0	1.402595
0.584068	0.623172	0	1.066382
0.928218	0.829354	0	1.126752
0.655159	0.641227	0	1.020794
0.972431	0.893714	0	1.152691
0.673613	0.664812	0	1.088933
0.977706	0.847549	0	1.113713
0.774231	0.76413	0	1.111306
1.06189	0.841695	0.005301	1.028374
0.575382	0.616805	0	1.035528
0.946384	0.847414	0.001883	1.156544
0.555135	0.473955	0	0.843832
1.264402	0.797015	0	0.805896
0.524303	0.535171	0	0.840878
1.237932	0.809716	0.006459	0.942173
0.540335	0.550836	0	0.879939
1.187445	0.740187	0	0.851566
0.574892	0.544419	0	0.803126
1.144657	0.688806	0	0.882908
0.594744	0.572889	0	0.79594
1.164369	0.687963	0	0.870149
0.61597	0.601573	0	0.847763
1.193543	0.736007	0.00561	0.867729
0.638488	0.730223	0	0.887514
1.176618	0.736232	0	0.950438
0.643939	0.720277	0	0.830772
1.164566	0.800766	0	0.897453
0.659376	0.743708	0	0.91776
1.151351	0.814308	0	0.895907
0.745563	0.857604	0	0.955907
1.145206	0.816963	0	0.837557
0.608903	0.637149	0	0.860874
1.178711	0.765953	0.002511	0.882827
0.510452	0.486726	0	0.927346
1.114532	0.826831	0	0.995037
0.488723	0.556504	0	0.926893

For peer review only

1.094018	0.860704	0.004721	1.042191
0.517363	0.529329	0	0.937242
1.079919	0.756148	0	0.985001
0.548795	0.623033	0	0.871804
1.043701	0.771634	0	1.001063
0.589176	0.574833	0	0.91739
1.065476	0.751796	0	1.030221
0.586848	0.600565	0	0.949025
1.077585	0.785432	0.004061	1.155131
0.61413	0.683138	0	0.972349
1.072421	0.781483	0	1.037885
0.649112	0.685119	0	0.921001
1.082664	0.846118	0	1.027133
0.665994	0.708366	0	0.99825
1.075526	0.830446	0	1.006066
0.759157	0.815488	0	1.028805
1.106927	0.829117	0.003687	0.935663
0.593602	0.627785	0	0.94495
1.080967	0.805567	0.002238	1.022214
0.787254	0.687897	0.001238	0.975805
6552	4472	<10	11821
5734	3779	<10	6670
6855	5088	<10	13185
6150	3989	<10	7250
7517	4941	<10	12451
7173	4271	<10	8114
8274	5550	<10	12519
7611	4714	<10	8411
8706	5512	<10	13162
8458	4916	<10	9448
8777	5547	<10	13489
8873	5312	<10	9943
9236	6265	<10	13544
9963	5783	<10	10364
10298	6419	<10	12763
10896	6289	<10	11052
10495	6447	<10	13083
11912	6378	<10	10882
11582	6653	<10	11946
13772	6738	<10	10230
88292	56894	<10	127963
90542	52169	<10	92364
10312	5106	<10	10602
13641	3273	<10	4732
10086	5493	<10	12235
14084	3573	<10	5493
10088	5561	<10	11781
15879	3706	<10	6282
10587	5780	<10	11631
15928	3988	<10	6424

For peer review only

10685	6306	<10	12545
17080	4343	<10	7037
11133	6908	<10	13130
16850	4607	<10	7258
11920	7494	<10	13874
18695	5185	<10	8347
12088	7848	<10	13203
19686	5543	<10	8420
11814	8068	<10	13676
19425	5538	<10	8371
11579	8392	<10	12793
18691	6197	<10	7752
110292	66956	<10	125470
169959	45953	<10	70116
16864	9578	<10	22423
19375	7052	<10	11402
16941	10581	<10	25420
20234	7562	<10	12743
17605	10502	<10	24232
23052	7977	<10	14396
18861	11330	<10	24150
23539	8702	<10	14835
19391	11818	<10	25707
25538	9259	<10	16485
19910	12455	<10	26619
25723	9919	<10	17201
21156	13759	<10	27418
28658	10968	<10	18711
22386	14267	<10	25966
30582	11832	<10	19472
22309	14515	<10	26759
31337	11916	<10	19253
23161	15045	<10	24739
32463	12935	<10	17982
198584	123850	<10	253433
260501	98122	<10	162480
459085	221972	<10	415913

For peer review only

code_block	code	cases
A00-A09	A00	<10
A00-A09	A01	61
A00-A09	A02	741
A00-A09	A03	51
A00-A09	A04	14398
A00-A09	A05	160
A00-A09	A06	39
A00-A09	A07	136
A00-A09	A08	7594
A00-A09	A09	21348
A15-A19	A15	798
A15-A19	A16	1305
A15-A19	A17	119
A15-A19	A18	474
A15-A19	A19	101
A20-A28	A20	<10
A20-A28	A21	<10
A20-A28	A22	<10
A20-A28	A23	<10
A20-A28	A24	<10
A20-A28	A25	<10
A20-A28	A26	<10
A20-A28	A27	21
A20-A28	A28	<10
A30-A49	A30	<10
A30-A49	A31	809
A30-A49	A32	139
A30-A49	A33	<10
A30-A49	A34	<10
A30-A49	A35	<10
A30-A49	A36	<10
A30-A49	A37	15
A30-A49	A38	12
A30-A49	A39	292
A30-A49	A40	1812
A30-A49	A41	59137
A30-A49	A42	111
A30-A49	A43	<10
A30-A49	A44	<10
A30-A49	A45	<10
A30-A49	A46	718
A30-A49	A47	<10
A30-A49	A48	757
A30-A49	A49	3528
A50-A64	A50	<10

For peer review only

A50-A64	A51	17
A50-A64	A52	163
A50-A64	A53	99
A50-A64	A54	22
A50-A64	A55	<10
A50-A64	A56	<10
A50-A64	A57	<10
A50-A64	A58	<10
A50-A64	A59	<10
A50-A64	A60	39
A50-A64	A61	<10
A50-A64	A62	<10
A50-A64	A63	774
A50-A64	A64	<10
A65-A69	A65	<10
A65-A69	A66	<10
A65-A69	A67	<10
A65-A69	A68	<10
A65-A69	A69	366
A70-A74	A70	13
A70-A74	A71	<10
A70-A74	A72	<10
A70-A74	A73	<10
A70-A74	A74	<10
A75-A79	A75	<10
A75-A79	A76	<10
A75-A79	A77	<10
A75-A79	A78	<10
A75-A79	A79	<10
A80-A89	A80	<10
A80-A89	A81	253
A80-A89	A82	<10
A80-A89	A83	<10
A80-A89	A84	<10
A80-A89	A85	19
A80-A89	A86	428
A80-A89	A87	538
A80-A89	A88	<10
A80-A89	A89	30
A90-A99	A90	26
A90-A99	A91	<10
A90-A99	A92	<10
A90-A99	A93	<10
A90-A99	A94	<10
A90-A99	A95	<10
A90-A99	A96	<10

For peer review only

A90-A99	A97	<10
A90-A99	A98	<10
A90-A99	A99	<10
B00-B09	B00	862
B00-B09	B01	331
B00-B09	B02	4021
B00-B09	B03	<10
B00-B09	B04	<10
B00-B09	B05	<10
B00-B09	B06	<10
B00-B09	B07	1228
B00-B09	B08	83
B00-B09	B09	29
B15-B19	B15	56
B15-B19	B16	170
B15-B19	B17	441
B15-B19	B18	894
B15-B19	B19	102
B20-B24	B20	898
B20-B24	B21	228
B20-B24	B22	196
B20-B24	B23	339
B20-B24	B24	561
B25-B34	B25	242
B25-B34	B26	40
B25-B34	B27	119
B25-B34	B28	<10
B25-B34	B29	<10
B25-B34	B30	29
B25-B34	B31	<10
B25-B34	B32	<10
B25-B34	B33	54
B25-B34	B34	7442
B35-B49	B35	259
B35-B49	B36	35
B35-B49	B37	2789
B35-B49	B38	<10
B35-B49	B39	<10
B35-B49	B40	<10
B35-B49	B41	<10
B35-B49	B42	<10
B35-B49	B43	<10
B35-B49	B44	917
B35-B49	B45	25
B35-B49	B46	<10
B35-B49	B47	36

For peer review only

B35-B49	B48	<10
B35-B49	B49	27
B50-B64	B50	102
B50-B64	B51	21
B50-B64	B52	<10
B50-B64	B53	<10
B50-B64	B54	31
B50-B64	B55	<10
B50-B64	B56	<10
B50-B64	B57	<10
B50-B64	B58	38
B50-B64	B59	413
B50-B64	B60	27
B50-B64	B61	<10
B50-B64	B62	<10
B50-B64	B63	<10
B50-B64	B64	<10
B65-B83	B65	<10
B65-B83	B66	<10
B65-B83	B67	12
B65-B83	B68	<10
B65-B83	B69	<10
B65-B83	B70	<10
B65-B83	B71	<10
B65-B83	B72	<10
B65-B83	B73	<10
B65-B83	B74	<10
B65-B83	B75	<10
B65-B83	B76	<10
B65-B83	B77	<10
B65-B83	B78	<10
B65-B83	B79	<10
B65-B83	B80	75
B65-B83	B81	<10
B65-B83	B82	15
B65-B83	B83	14
B85-B89	B85	<10
B85-B89	B86	66
B85-B89	B87	<10
B85-B89	B88	<10
B85-B89	B89	<10
B95-B98	B95	<10
B95-B98	B96	<10
B95-B98	B97	<10
B95-B98	B98	<10
B99-B99	B99	337

For peer review only

C00-C14	C00	600
C00-C14	C01	3393
C00-C14	C02	4936
C00-C14	C03	669
C00-C14	C04	2675
C00-C14	C05	1534
C00-C14	C06	2317
C00-C14	C07	1180
C00-C14	C08	398
C00-C14	C09	3780
C00-C14	C10	3029
C00-C14	C11	1236
C00-C14	C12	1907
C00-C14	C13	1739
C00-C14	C14	1984
C15-C26	C15	64713
C15-C26	C16	45622
C15-C26	C17	4248
C15-C26	C18	148739
C15-C26	C19	33263
C15-C26	C20	62367
C15-C26	C21	3890
C15-C26	C22	16066
C15-C26	C23	2790
C15-C26	C24	4795
C15-C26	C25	40633
C15-C26	C26	2466
C30-C39	C30	653
C30-C39	C31	844
C30-C39	C32	11590
C30-C39	C33	302
C30-C39	C34	207455
C30-C39	C35	<10
C30-C39	C36	<10
C30-C39	C37	426
C30-C39	C38	757
C30-C39	C39	23
C40-C41	C40	460
C40-C41	C41	1853
C43-C44	C43	11618
C43-C44	C44	54189
C45-C49	C45	8961
C45-C49	C46	140
C45-C49	C47	220
C45-C49	C48	6732
C45-C49	C49	4676

For peer review only

C50-C50	C50	230331
C51-C58	C51	4015
C51-C58	C52	1427
C51-C58	C53	12393
C51-C58	C54	21614
C51-C58	C55	2368
C51-C58	C56	67234
C51-C58	C57	993
C51-C58	C58	35
C60-C63	C60	1614
C60-C63	C61	62292
C60-C63	C62	2342
C60-C63	C63	131
C64-C68	C64	21359
C64-C68	C65	1624
C64-C68	C66	1785
C64-C68	C67	57717
C64-C68	C68	562
C69-C72	C69	1176
C69-C72	C70	279
C69-C72	C71	15898
C69-C72	C72	275
C73-C75	C73	3695
C73-C75	C74	689
C73-C75	C75	560
C76-C80	C76	2462
C76-C80	C77	8892
C76-C80	C78	45065
C76-C80	C79	39153
C76-C80	C80	20089
C81-C96	C81	9092
C81-C96	C82	20760
C81-C96	C83	33853
C81-C96	C84	4401
C81-C96	C85	40124
C81-C96	C86	526
C81-C96	C87	<10
C81-C96	C88	4846
C81-C96	C89	<10
C81-C96	C90	88383
C81-C96	C91	38832
C81-C96	C92	39801
C81-C96	C93	950
C81-C96	C94	759
C81-C96	C95	602
C81-C96	C96	229

For peer review only

C97-C97	C97	49
D00-D09	D00	945
D00-D09	D01	689
D00-D09	D02	1191
D00-D09	D03	2279
D00-D09	D04	2430
D00-D09	D05	5369
D00-D09	D06	580
D00-D09	D07	1998
D00-D09	D08	<10
D00-D09	D09	4379
D10-D36	D10	1456
D10-D36	D11	2221
D10-D36	D12	62890
D10-D36	D13	4961
D10-D36	D14	1926
D10-D36	D15	751
D10-D36	D16	727
D10-D36	D17	13392
D10-D36	D18	3327
D10-D36	D19	43
D10-D36	D20	70
D10-D36	D21	1388
D10-D36	D22	4922
D10-D36	D23	7143
D10-D36	D24	3472
D10-D36	D25	11816
D10-D36	D26	561
D10-D36	D27	4312
D10-D36	D28	334
D10-D36	D29	326
D10-D36	D30	978
D10-D36	D31	126
D10-D36	D32	4464
D10-D36	D33	1352
D10-D36	D34	809
D10-D36	D35	3410
D10-D36	D36	1975
D37-D48	D37	4921
D37-D48	D38	1248
D37-D48	D39	617
D37-D48	D40	209
D37-D48	D41	27615
D37-D48	D42	117
D37-D48	D43	1589
D37-D48	D44	558

For peer review only

D37-D48	D45	13580
D37-D48	D46	48236
D37-D48	D47	17317
D37-D48	D48	2027
D50-D53	D50	54494
D50-D53	D51	1368
D50-D53	D52	2161
D50-D53	D53	698
D55-D59	D55	33
D55-D59	D56	110
D55-D59	D57	37
D55-D59	D58	658
D55-D59	D59	4905
D60-D64	D60	1142
D60-D64	D61	9688
D60-D64	D62	135
D60-D64	D63	679
D60-D64	D64	67005
D65-D69	D65	166
D65-D69	D66	995
D65-D69	D67	175
D65-D69	D68	2853
D65-D69	D69	11338
D70-D77	D70	9311
D70-D77	D71	22
D70-D77	D72	575
D70-D77	D73	596
D70-D77	D74	19
D70-D77	D75	7842
D70-D77	D76	253
D70-D77	D77	<10
D80-D89	D80	4390
D80-D89	D81	66
D80-D89	D82	236
D80-D89	D83	2783
D80-D89	D84	563
D80-D89	D85	<10
D80-D89	D86	1783
D80-D89	D87	<10
D80-D89	D88	<10
D80-D89	D89	1883
E00-E07	E00	<10
E00-E07	E01	24
E00-E07	E02	<10
E00-E07	E03	985
E00-E07	E04	3761

For peer review only

E00-E07	E05	2536
E00-E07	E06	336
E00-E07	E07	229
E10-E14	E10	14740
E10-E14	E11	22918
E10-E14	E12	<10
E10-E14	E13	176
E10-E14	E14	3406
E15-E16	E15	82
E15-E16	E16	12554
E20-E35	E20	75
E20-E35	E21	1694
E20-E35	E22	1362
E20-E35	E23	640
E20-E35	E24	464
E20-E35	E25	<10
E20-E35	E26	124
E20-E35	E27	1248
E20-E35	E28	41
E20-E35	E29	460
E20-E35	E30	<10
E20-E35	E31	<10
E20-E35	E32	88
E20-E35	E33	<10
E20-E35	E34	292
E20-E35	E35	<10
E40-E46	E40	<10
E40-E46	E41	14
E40-E46	E42	<10
E40-E46	E43	56
E40-E46	E44	13
E40-E46	E45	<10
E40-E46	E46	839
E50-E64	E50	<10
E50-E64	E51	239
E50-E64	E52	<10
E50-E64	E53	1140
E50-E64	E54	<10
E50-E64	E55	58
E50-E64	E56	33
E50-E64	E57	<10
E50-E64	E58	17
E50-E64	E59	<10
E50-E64	E60	<10
E50-E64	E61	1001
E50-E64	E62	<10

For peer review only

E50-E64	E63	77
E50-E64	E64	<10
E65-E68	E65	122
E65-E68	E66	2216
E65-E68	E67	<10
E65-E68	E68	<10
E70-E90	E70	17
E70-E90	E71	20
E70-E90	E72	68
E70-E90	E73	40
E70-E90	E74	83
E70-E90	E75	107
E70-E90	E76	<10
E70-E90	E77	11
E70-E90	E78	513
E70-E90	E79	<10
E70-E90	E80	539
E70-E90	E81	<10
E70-E90	E82	<10
E70-E90	E83	60601
E70-E90	E84	395
E70-E90	E85	1311
E70-E90	E86	11524
E70-E90	E87	22513
E70-E90	E88	417
E70-E90	E89	126
E70-E90	E90	<10
F00-F09	F00	31
F00-F09	F01	5843
F00-F09	F02	14
F00-F09	F03	12131
F00-F09	F04	17
F00-F09	F05	19521
F00-F09	F06	2237
F00-F09	F07	108
F00-F09	F08	<10
F00-F09	F09	20
F10-F19	F10	36876
F10-F19	F11	200
F10-F19	F12	<10
F10-F19	F13	72
F10-F19	F14	<10
F10-F19	F15	23
F10-F19	F16	<10
F10-F19	F17	41
F10-F19	F18	<10

For peer review only

F10-F19	F19	53
F20-F29	F20	344
F20-F29	F21	<10
F20-F29	F22	177
F20-F29	F23	206
F20-F29	F24	<10
F20-F29	F25	104
F20-F29	F26	<10
F20-F29	F27	<10
F20-F29	F28	24
F20-F29	F29	235
F30-F39	F30	134
F30-F39	F31	305
F30-F39	F32	2675
F30-F39	F33	336
F30-F39	F34	29
F30-F39	F35	<10
F30-F39	F36	<10
F30-F39	F37	<10
F30-F39	F38	10
F30-F39	F39	17
F40-F48	F40	20
F40-F48	F41	4692
F40-F48	F42	15
F40-F48	F43	176
F40-F48	F44	296
F40-F48	F45	1107
F40-F48	F46	<10
F40-F48	F47	<10
F40-F48	F48	37
F50-F59	F50	154
F50-F59	F51	26
F50-F59	F52	131
F50-F59	F53	<10
F50-F59	F54	<10
F50-F59	F55	<10
F50-F59	F56	<10
F50-F59	F57	<10
F50-F59	F58	<10
F50-F59	F59	<10
F60-F69	F60	69
F60-F69	F61	<10
F60-F69	F62	11
F60-F69	F63	<10
F60-F69	F64	13
F60-F69	F65	<10

For peer review only

F60-F69	F66	<10
F60-F69	F67	<10
F60-F69	F68	<10
F60-F69	F69	19
F70-F79	F70	13
F70-F79	F71	<10
F70-F79	F72	<10
F70-F79	F73	<10
F70-F79	F74	<10
F70-F79	F75	<10
F70-F79	F76	<10
F70-F79	F77	<10
F70-F79	F78	<10
F70-F79	F79	15
F80-F89	F80	77
F80-F89	F81	23
F80-F89	F82	<10
F80-F89	F83	<10
F80-F89	F84	<10
F80-F89	F85	<10
F80-F89	F86	<10
F80-F89	F87	<10
F80-F89	F88	<10
F80-F89	F89	<10
F90-F98	F90	<10
F90-F98	F91	24
F90-F98	F92	<10
F90-F98	F93	<10
F90-F98	F94	<10
F90-F98	F95	<10
F90-F98	F96	<10
F90-F98	F97	<10
F90-F98	F98	11
F99-F99	F99	37
G00-G09	G00	1102
G00-G09	G01	<10
G00-G09	G02	<10
G00-G09	G03	420
G00-G09	G04	941
G00-G09	G05	<10
G00-G09	G06	1201
G00-G09	G07	<10
G00-G09	G08	218
G00-G09	G09	<10
G10-G13	G10	353
G10-G13	G11	454

For peer review only

G10-G13	G12	4387
G10-G13	G13	<10
G20-G26	G20	9724
G20-G26	G21	369
G20-G26	G22	<10
G20-G26	G23	170
G20-G26	G24	445
G20-G26	G25	1114
G20-G26	G26	<10
G30-G32	G30	4330
G30-G32	G31	3362
G30-G32	G32	<10
G35-G37	G35	16934
G35-G37	G36	73
G35-G37	G37	1839
G40-G47	G40	23766
G40-G47	G41	2591
G40-G47	G42	<10
G40-G47	G43	6460
G40-G47	G44	1789
G40-G47	G45	32971
G40-G47	G46	39
G40-G47	G47	15847
G50-G59	G50	2341
G50-G59	G51	3318
G50-G59	G52	168
G50-G59	G53	<10
G50-G59	G54	1004
G50-G59	G55	163
G50-G59	G56	41073
G50-G59	G57	2967
G50-G59	G58	1209
G50-G59	G59	<10
G60-G64	G60	896
G60-G64	G61	5878
G60-G64	G62	7294
G60-G64	G63	<10
G60-G64	G64	<10
G70-G73	G70	3090
G70-G73	G71	568
G70-G73	G72	564
G70-G73	G73	<10
G80-G83	G80	311
G80-G83	G81	4763
G80-G83	G82	1154
G80-G83	G83	1780

For peer review only

G90-G99	G90	737
G90-G99	G91	1299
G90-G99	G92	29
G90-G99	G93	4315
G90-G99	G94	<10
G90-G99	G95	3757
G90-G99	G96	525
G90-G99	G97	351
G90-G99	G98	124
G90-G99	G99	92
H00-H06	H00	923
H00-H06	H01	601
H00-H06	H02	20890
H00-H06	H03	<10
H00-H06	H04	6139
H00-H06	H05	1044
H00-H06	H06	70
H10-H13	H10	303
H10-H13	H11	1577
H10-H13	H12	<10
H10-H13	H13	<10
H15-H22	H15	248
H15-H22	H16	2751
H15-H22	H17	175
H15-H22	H18	2158
H15-H22	H19	<10
H15-H22	H20	1021
H15-H22	H21	352
H15-H22	H22	<10
H25-H28	H25	36162
H25-H28	H26	283044
H25-H28	H27	604
H25-H28	H28	<10
H30-H36	H30	128
H30-H36	H31	267
H30-H36	H32	<10
H30-H36	H33	8905
H30-H36	H34	1428
H30-H36	H35	23741
H30-H36	H36	507
H40-H42	H40	10279
H40-H42	H41	<10
H40-H42	H42	<10
H43-H45	H43	3417
H43-H45	H44	862
H43-H45	H45	19

For peer review only

H46-H48	H46	240
H46-H48	H47	366
H46-H48	H48	<10
H49-H52	H49	823
H49-H52	H50	1638
H49-H52	H51	94
H49-H52	H52	356
H53-H54	H53	2822
H53-H54	H54	437
H55-H59	H55	76
H55-H59	H56	<10
H55-H59	H57	910
H55-H59	H58	<10
H55-H59	H59	410
H60-H62	H60	1833
H60-H62	H61	2628
H60-H62	H62	<10
H65-H75	H65	2479
H65-H75	H66	2455
H65-H75	H67	<10
H65-H75	H68	78
H65-H75	H69	167
H65-H75	H70	238
H65-H75	H71	1245
H65-H75	H72	2070
H65-H75	H73	431
H65-H75	H74	769
H65-H75	H75	<10
H80-H83	H80	586
H80-H83	H81	2443
H80-H83	H82	<10
H80-H83	H83	3864
H90-H95	H90	791
H90-H95	H91	1229
H90-H95	H92	1177
H90-H95	H93	325
H90-H95	H94	<10
H90-H95	H95	195
I00-I02I00	28	
I00-I02I01	40	
I00-I02I02	<10	
I05-I09I05	2332	
I05-I09I06	170	
I05-I09I07	191	
I05-I09I08	5145	
I05-I09I09	160	

For peer review only

I10-I15I10	9211
I10-I15I11	1102
I10-I15I12	4488
I10-I15I13	344
I10-I15I14	<10
I10-I15I15	86
I20-I25I20	118638
I20-I25I21	202146
I20-I25I22	17939
I20-I25I23	304
I20-I25I24	16721
I20-I25I25	173177
I26-I28I26	35328
I26-I28I27	5307
I26-I28I28	81
I30-I52I30	1529
I30-I52I31	4778
I30-I52I32	<10
I30-I52I33	3800
I30-I52I34	7695
I30-I52I35	34980
I30-I52I36	168
I30-I52I37	79
I30-I52I38	1102
I30-I52I39	<10
I30-I52I40	166
I30-I52I41	<10
I30-I52I42	6027
I30-I52I43	11
I30-I52I44	16546
I30-I52I45	4403
I30-I52I46	10938
I30-I52I47	20735
I30-I52I48	119012
I30-I52I49	9265
I30-I52I50	139263
I30-I52I51	3702
I30-I52I52	<10
I60-I69I60	13545
I60-I69I61	21689
I60-I69I62	10954
I60-I69I63	98595
I60-I69I64	66512
I60-I69I65	8453
I60-I69I66	2288
I60-I69I67	16855

For peer review only

I60-I69I68	<10	
I60-I69I69	<10	
I70-I79I70	10337	
I70-I79I71	26253	
I70-I79I72	3723	
I70-I79I73	54493	
I70-I79I74	7634	
I70-I79I75	<10	
I70-I79I76	<10	
I70-I79I77	10398	
I70-I79I78	6099	
I70-I79I79	64	
I80-I89I80	24269	
I80-I89I81	303	
I80-I89I82	1156	
I80-I89I83	35793	
I80-I89I84	44665	
I80-I89I85	13775	
I80-I89I86	1600	
I80-I89I87	2400	
I80-I89I88	216	
I80-I89I89	1727	
I95-I99I95	21488	
I95-I99I96	<10	
I95-I99I97	400	
I95-I99I98	465	
I95-I99I99	1748	
J00-J06	J00	179
J00-J06	J01	415
J00-J06	J02	1525
J00-J06	J03	1743
J00-J06	J04	719
J00-J06	J05	422
J00-J06	J06	4633
J09-J18	J09	71
J09-J18	J10	3581
J09-J18	J11	914
J09-J18	J12	715
J09-J18	J13	3705
J09-J18	J14	1247
J09-J18	J15	7603
J09-J18	J16	166
J09-J18	J17	30
J09-J18	J18	222976
J20-J22	J20	955
J20-J22	J21	294

For peer review only

J20-J22	J22	142163
J30-J39	J30	204
J30-J39	J31	1031
J30-J39	J32	3119
J30-J39	J33	8790
J30-J39	J34	11726
J30-J39	J35	1249
J30-J39	J36	1191
J30-J39	J37	669
J30-J39	J38	10097
J30-J39	J39	3391
J40-J47	J40	1332
J40-J47	J41	80
J40-J47	J42	568
J40-J47	J43	4124
J40-J47	J44	272560
J40-J47	J45	31051
J40-J47	J46	1830
J40-J47	J47	10247
J60-J70	J60	41
J60-J70	J61	306
J60-J70	J62	48
J60-J70	J63	<10
J60-J70	J64	44
J60-J70	J65	<10
J60-J70	J66	<10
J60-J70	J67	663
J60-J70	J68	50
J60-J70	J69	29080
J60-J70	J70	356
J80-J84	J80	833
J80-J84	J81	12546
J80-J84	J82	234
J80-J84	J83	<10
J80-J84	J84	13248
J85-J86	J85	769
J85-J86	J86	4185
J90-J94	J90	25422
J90-J94	J91	274
J90-J94	J92	765
J90-J94	J93	8133
J90-J94	J94	1237
J95-J99	J95	1907
J95-J99	J96	15107
J95-J99	J97	<10
J95-J99	J98	10366

For peer review only

J95-J99	J99	15
K00-K14	K00	213
K00-K14	K01	2001
K00-K14	K02	7171
K00-K14	K03	98
K00-K14	K04	3080
K00-K14	K05	676
K00-K14	K06	669
K00-K14	K07	1047
K00-K14	K08	2333
K00-K14	K09	1024
K00-K14	K10	1536
K00-K14	K11	3597
K00-K14	K12	1701
K00-K14	K13	3908
K00-K14	K14	2507
K20-K31	K20	18489
K20-K31	K21	47676
K20-K31	K22	61869
K20-K31	K23	<10
K20-K31	K24	<10
K20-K31	K25	19140
K20-K31	K26	22841
K20-K31	K27	1453
K20-K31	K28	340
K20-K31	K29	80588
K20-K31	K30	19305
K20-K31	K31	15963
K35-K38	K35	10590
K35-K38	K36	233
K35-K38	K37	1179
K35-K38	K38	373
K40-K46	K40	61120
K40-K46	K41	4613
K40-K46	K42	13014
K40-K46	K43	20112
K40-K46	K44	56940
K40-K46	K45	770
K40-K46	K46	869
K50-K52	K50	22827
K50-K52	K51	19728
K50-K52	K52	69630
K55-K63	K55	16090
K55-K63	K56	40625
K55-K63	K57	126292
K55-K63	K58	5743

For peer review only

K55-K63	K59	52671
K55-K63	K60	11881
K55-K63	K61	8608
K55-K63	K62	67372
K55-K63	K63	37732
K65-K67	K65	6797
K65-K67	K66	3646
K65-K67	K67	<10
K70-K77	K70	36852
K70-K77	K71	343
K70-K77	K72	4934
K70-K77	K73	547
K70-K77	K74	8727
K70-K77	K75	3887
K70-K77	K76	8283
K70-K77	K77	<10
K80-K87	K80	111477
K80-K87	K81	23462
K80-K87	K82	3488
K80-K87	K83	18526
K80-K87	K84	<10
K80-K87	K85	26402
K80-K87	K86	13035
K80-K87	K87	<10
K90-K93	K90	3082
K90-K93	K91	9128
K90-K93	K92	69215
K90-K93	K93	<10
L00-L08	L00	18
L00-L08	L01	98
L00-L08	L02	12283
L00-L08	L03	70241
L00-L08	L04	202
L00-L08	L05	2072
L00-L08	L06	<10
L00-L08	L07	<10
L00-L08	L08	3880
L10-L14	L10	211
L10-L14	L11	100
L10-L14	L12	1308
L10-L14	L13	81
L10-L14	L14	<10
L20-L30	L20	373
L20-L30	L21	54
L20-L30	L22	<10
L20-L30	L23	150

For peer review only

L20-L30	L24	37
L20-L30	L25	157
L20-L30	L26	33
L20-L30	L27	784
L20-L30	L28	623
L20-L30	L29	948
L20-L30	L30	4352
L40-L45	L40	12087
L40-L45	L41	15
L40-L45	L42	<10
L40-L45	L43	647
L40-L45	L44	217
L40-L45	L45	<10
L50-L54	L50	798
L50-L54	L51	447
L50-L54	L52	117
L50-L54	L53	1109
L50-L54	L54	<10
L55-L59	L55	27
L55-L59	L56	441
L55-L59	L57	5458
L55-L59	L58	19
L55-L59	L59	113
L60-L75	L60	3250
L60-L75	L61	<10
L60-L75	L62	<10
L60-L75	L63	20
L60-L75	L64	<10
L60-L75	L65	47
L60-L75	L66	23
L60-L75	L67	<10
L60-L75	L68	32
L60-L75	L69	<10
L60-L75	L70	72
L60-L75	L71	671
L60-L75	L72	21898
L60-L75	L73	1783
L60-L75	L74	28
L60-L75	L75	14
L80-L99	L80	<10
L80-L99	L81	1039
L80-L99	L82	3535
L80-L99	L83	81
L80-L99	L84	210
L80-L99	L85	1123
L80-L99	L86	<10

For peer review only

L80-L99	L87	24
L80-L99	L88	463
L80-L99	L89	2891
L80-L99	L90	5435
L80-L99	L91	1594
L80-L99	L92	1104
L80-L99	L93	214
L80-L99	L94	319
L80-L99	L95	590
L80-L99	L96	<10
L80-L99	L97	28410
L80-L99	L98	8618
L80-L99	L99	<10
M00-M03	M00	5098
M00-M03	M01	<10
M00-M03	M02	248
M00-M03	M03	<10
M05-M14	M05	7371
M05-M14	M06	44100
M05-M14	M07	195
M05-M14	M08	156
M05-M14	M09	<10
M05-M14	M10	7801
M05-M14	M11	1083
M05-M14	M12	377
M05-M14	M13	5923
M05-M14	M14	<10
M15-M19	M15	1379
M15-M19	M16	72426
M15-M19	M17	80381
M15-M19	M18	4508
M15-M19	M19	19803
M20-M25	M20	24631
M20-M25	M21	2203
M20-M25	M22	755
M20-M25	M23	24731
M20-M25	M24	3350
M20-M25	M25	41115
M30-M36	M30	463
M30-M36	M31	5184
M30-M36	M32	1509
M30-M36	M33	1719
M30-M36	M34	4583
M30-M36	M35	2937
M30-M36	M36	<10
M40-M43	M40	147

For peer review only

M40-M43	M41	590
M40-M43	M42	<10
M40-M43	M43	1631
M45-M49	M45	1501
M45-M49	M46	3176
M45-M49	M47	6933
M45-M49	M48	10222
M45-M49	M49	16
M50-M54	M50	3098
M50-M54	M51	12249
M50-M54	M52	<10
M50-M54	M53	1234
M50-M54	M54	61197
M60-M63	M60	1550
M60-M63	M61	35
M60-M63	M62	5276
M60-M63	M63	<10
M65-M68	M65	9817
M65-M68	M66	1893
M65-M68	M67	6696
M65-M68	M68	<10
M70-M79	M70	4448
M70-M79	M71	2375
M70-M79	M72	19694
M70-M79	M73	<10
M70-M79	M74	<10
M70-M79	M75	20973
M70-M79	M76	736
M70-M79	M77	3176
M70-M79	M78	<10
M70-M79	M79	54568
M80-M85	M80	6497
M80-M85	M81	9212
M80-M85	M82	<10
M80-M85	M83	85
M80-M85	M84	8467
M80-M85	M85	455
M86-M90	M86	5691
M86-M90	M87	2272
M86-M90	M88	1151
M86-M90	M89	2704
M86-M90	M90	194
M91-M94	M91	38
M91-M94	M92	84
M91-M94	M93	300
M91-M94	M94	1470

For peer review only

M95-M99	M95	907
M95-M99	M96	2265
M95-M99	M97	<10
M95-M99	M98	<10
M95-M99	M99	<10
N00-N08	N00	158
N00-N08	N01	22
N00-N08	N02	8982
N00-N08	N03	611
N00-N08	N04	1296
N00-N08	N05	966
N00-N08	N06	17
N00-N08	N07	<10
N00-N08	N08	<10
N10-N16	N10	1183
N10-N16	N11	842
N10-N16	N12	3853
N10-N16	N13	16606
N10-N16	N14	100
N10-N16	N15	638
N10-N16	N16	<10
N17-N19	N17	49846
N17-N19	N18	41153
N17-N19	N19	7323
N20-N23	N20	39818
N20-N23	N21	5370
N20-N23	N22	<10
N20-N23	N23	7058
N25-N29	N25	275
N25-N29	N26	198
N25-N29	N27	31
N25-N29	N28	4123
N25-N29	N29	<10
N30-N39	N30	16966
N30-N39	N31	7352
N30-N39	N32	21571
N30-N39	N33	<10
N30-N39	N34	448
N30-N39	N35	17761
N30-N39	N36	1774
N30-N39	N37	<10
N30-N39	N38	<10
N30-N39	N39	185262
N40-N51	N40	30726
N40-N51	N41	2847
N40-N51	N42	1244

For peer review only

N40-N51	N43	5185
N40-N51	N44	61
N40-N51	N45	4217
N40-N51	N46	86
N40-N51	N47	9463
N40-N51	N48	5493
N40-N51	N49	1684
N40-N51	N50	6038
N40-N51	N51	<10
N60-N64	N60	3459
N60-N64	N61	2338
N60-N64	N62	1002
N60-N64	N63	1826
N60-N64	N64	2365
N70-N77	N70	844
N70-N77	N71	595
N70-N77	N72	259
N70-N77	N73	1742
N70-N77	N74	<10
N70-N77	N75	1513
N70-N77	N76	2140
N70-N77	N77	<10
N80-N98	N80	3008
N80-N98	N81	32139
N80-N98	N82	2451
N80-N98	N83	7052
N80-N98	N84	13289
N80-N98	N85	3244
N80-N98	N86	253
N80-N98	N87	555
N80-N98	N88	558
N80-N98	N89	1945
N80-N98	N90	3232
N80-N98	N91	105
N80-N98	N92	22483
N80-N98	N93	6963
N80-N98	N94	2318
N80-N98	N95	21071
N80-N98	N96	<10
N80-N98	N97	598
N80-N98	N98	12
N99-N99	N99	1952
O00-O08	O00	202
O00-O08	O01	17
O00-O08	O02	783
O00-O08	O03	675

For peer review only

O00-O08	O04	2376
O00-O08	O05	<10
O00-O08	O06	<10
O00-O08	O07	10
O00-O08	O08	30
O10-O16	O10	<10
O10-O16	O11	<10
O10-O16	O12	<10
O10-O16	O13	<10
O10-O16	O14	<10
O10-O16	O15	<10
O10-O16	O16	<10
O20-O29	O20	131
O20-O29	O21	30
O20-O29	O22	<10
O20-O29	O23	<10
O20-O29	O24	<10
O20-O29	O25	<10
O20-O29	O26	<10
O20-O29	O27	<10
O20-O29	O28	<10
O20-O29	O29	<10
O30-O48	O30	<10
O30-O48	O31	<10
O30-O48	O32	<10
O30-O48	O33	<10
O30-O48	O34	<10
O30-O48	O35	<10
O30-O48	O36	<10
O30-O48	O37	<10
O30-O48	O38	<10
O30-O48	O39	<10
O30-O48	O40	<10
O30-O48	O41	<10
O30-O48	O42	<10
O30-O48	O43	<10
O30-O48	O44	<10
O30-O48	O45	<10
O30-O48	O46	<10
O30-O48	O47	<10
O30-O48	O48	<10
O60-O75	O60	<10
O60-O75	O61	<10
O60-O75	O62	<10
O60-O75	O63	<10
O60-O75	O64	<10

For peer review only

O60-O75	O65	<10
O60-O75	O66	<10
O60-O75	O67	<10
O60-O75	O68	<10
O60-O75	O69	<10
O60-O75	O70	<10
O60-O75	O71	<10
O60-O75	O72	16
O60-O75	O73	<10
O60-O75	O74	<10
O60-O75	O75	<10
O80-O84	O80	<10
O80-O84	O81	<10
O80-O84	O82	<10
O80-O84	O83	<10
O80-O84	O84	<10
O85-O92	O85	<10
O85-O92	O86	<10
O85-O92	O87	<10
O85-O92	O88	<10
O85-O92	O89	<10
O85-O92	O90	<10
O85-O92	O91	<10
O85-O92	O92	<10
O95-O99	O95	<10
O95-O99	O96	<10
O95-O99	O97	<10
O95-O99	O98	<10
O95-O99	O99	<10
P05-P08	P05	<10
P05-P08	P06	<10
P05-P08	P07	<10
P05-P08	P08	<10
P10-P15	P10	<10
P10-P15	P11	<10
P10-P15	P12	<10
P10-P15	P13	<10
P10-P15	P14	<10
P10-P15	P15	<10
P20-P29	P20	<10
P20-P29	P21	<10
P20-P29	P22	<10
P20-P29	P23	<10
P20-P29	P24	<10
P20-P29	P25	<10
P20-P29	P26	<10

For peer review only

P20-P29	P27	<10
P20-P29	P28	<10
P20-P29	P29	<10
P35-P39	P35	<10
P35-P39	P36	<10
P35-P39	P37	<10
P35-P39	P38	<10
P35-P39	P39	<10
P50-P61	P50	<10
P50-P61	P51	<10
P50-P61	P52	<10
P50-P61	P53	<10
P50-P61	P54	<10
P50-P61	P55	<10
P50-P61	P56	<10
P50-P61	P57	<10
P50-P61	P58	<10
P50-P61	P59	<10
P50-P61	P60	<10
P50-P61	P61	<10
P80-P83	P80	<10
P80-P83	P81	<10
P80-P83	P82	<10
P80-P83	P83	<10
Q00-Q07	Q00	<10
Q00-Q07	Q01	14
Q00-Q07	Q02	13
Q00-Q07	Q03	90
Q00-Q07	Q04	124
Q00-Q07	Q05	114
Q00-Q07	Q06	74
Q00-Q07	Q07	236
Q10-Q18	Q10	54
Q10-Q18	Q11	<10
Q10-Q18	Q12	72
Q10-Q18	Q13	15
Q10-Q18	Q14	<10
Q10-Q18	Q15	29
Q10-Q18	Q16	14
Q10-Q18	Q17	92
Q10-Q18	Q18	352
Q20-Q28	Q20	15
Q20-Q28	Q21	1461
Q20-Q28	Q22	100
Q20-Q28	Q23	420
Q20-Q28	Q24	163

For peer review only

Q20-Q28	Q25	187
Q20-Q28	Q26	47
Q20-Q28	Q27	741
Q20-Q28	Q28	744
Q30-Q34	Q30	37
Q30-Q34	Q31	130
Q30-Q34	Q32	<10
Q30-Q34	Q33	33
Q30-Q34	Q34	12
Q35-Q37	Q35	25
Q35-Q37	Q36	10
Q35-Q37	Q37	11
Q38-Q45	Q38	1018
Q38-Q45	Q39	185
Q38-Q45	Q40	67
Q38-Q45	Q41	<10
Q38-Q45	Q42	<10
Q38-Q45	Q43	329
Q38-Q45	Q44	158
Q38-Q45	Q45	39
Q50-Q56	Q50	73
Q50-Q56	Q51	25
Q50-Q56	Q52	89
Q50-Q56	Q53	121
Q50-Q56	Q54	215
Q50-Q56	Q55	88
Q50-Q56	Q56	<10
Q60-Q64	Q60	16
Q60-Q64	Q61	839
Q60-Q64	Q62	128
Q60-Q64	Q63	67
Q60-Q64	Q64	81
Q65-Q79	Q65	201
Q65-Q79	Q66	283
Q65-Q79	Q67	90
Q65-Q79	Q68	19
Q65-Q79	Q69	<10
Q65-Q79	Q70	15
Q65-Q79	Q71	<10
Q65-Q79	Q72	<10
Q65-Q79	Q73	<10
Q65-Q79	Q74	65
Q65-Q79	Q75	17
Q65-Q79	Q76	64
Q65-Q79	Q77	51
Q65-Q79	Q78	82

For peer review only

Q65-Q79	Q79	79
Q80-Q89	Q80	<10
Q80-Q89	Q81	15
Q80-Q89	Q82	646
Q80-Q89	Q83	91
Q80-Q89	Q84	<10
Q80-Q89	Q85	413
Q80-Q89	Q86	<10
Q80-Q89	Q87	96
Q80-Q89	Q88	<10
Q80-Q89	Q89	240
Q90-Q99	Q90	133
Q90-Q99	Q91	<10
Q90-Q99	Q92	<10
Q90-Q99	Q93	<10
Q90-Q99	Q94	<10
Q90-Q99	Q95	<10
Q90-Q99	Q96	19
Q90-Q99	Q97	<10
Q90-Q99	Q98	67
Q90-Q99	Q99	17
R00-R09	R00	21072
R00-R09	R01	65
R00-R09	R02	3009
R00-R09	R03	143
R00-R09	R04	39227
R00-R09	R05	5669
R00-R09	R06	54686
R00-R09	R07	301798
R00-R09	R08	<10
R00-R09	R09	3392
R10-R19	R10	151132
R10-R19	R11	39828
R10-R19	R12	2189
R10-R19	R13	27099
R10-R19	R14	1969
R10-R19	R15	4868
R10-R19	R16	628
R10-R19	R17	5908
R10-R19	R18	9937
R10-R19	R19	31403
R20-R23	R20	5419
R20-R23	R21	2314
R20-R23	R22	5833
R20-R23	R23	972
R25-R29	R25	1817

For peer review only

R25-R29	R26	61809
R25-R29	R27	828
R25-R29	R28	<10
R25-R29	R29	27490
R30-R39	R30	1643
R30-R39	R31	55796
R30-R39	R32	5670
R30-R39	R33	35863
R30-R39	R34	151
R30-R39	R35	5426
R30-R39	R36	86
R30-R39	R37	<10
R30-R39	R38	<10
R30-R39	R39	15982
R40-R46	R40	4355
R40-R46	R41	45481
R40-R46	R42	20685
R40-R46	R43	34
R40-R46	R44	1634
R40-R46	R45	895
R40-R46	R46	668
R47-R49	R47	4910
R47-R49	R48	18
R47-R49	R49	2963
R50-R69	R50	8353
R50-R69	R51	30605
R50-R69	R52	3116
R50-R69	R53	13671
R50-R69	R54	57428
R50-R69	R55	108422
R50-R69	R56	27660
R50-R69	R57	2049
R50-R69	R58	573
R50-R69	R59	4932
R50-R69	R60	7080
R50-R69	R61	683
R50-R69	R62	38
R50-R69	R63	15747
R50-R69	R64	169
R50-R69	R65	<10
R50-R69	R66	<10
R50-R69	R67	<10
R50-R69	R68	1693
R50-R69	R69	3781
R70-R79	R70	123
R70-R79	R71	68

For peer review only

R70-R79	R72	100
R70-R79	R73	3558
R70-R79	R74	186
R70-R79	R75	<10
R70-R79	R76	716
R70-R79	R77	147
R70-R79	R78	154
R70-R79	R79	5184
R80-R82	R80	354
R80-R82	R81	10
R80-R82	R82	310
R83-R89	R83	<10
R83-R89	R84	145
R83-R89	R85	33
R83-R89	R86	21
R83-R89	R87	442
R83-R89	R88	<10
R83-R89	R89	26
R90-R94	R90	841
R90-R94	R91	18713
R90-R94	R92	130
R90-R94	R93	5822
R90-R94	R94	4510
R95-R99	R95	<10
R95-R99	R96	<10
R95-R99	R97	<10
R95-R99	R98	<10
R95-R99	R99	1944
S00-S09	S00	18337
S00-S09	S01	24541
S00-S09	S02	8599
S00-S09	S03	129
S00-S09	S04	47
S00-S09	S05	1488
S00-S09	S06	13727
S00-S09	S07	<10
S00-S09	S08	34
S00-S09	S09	35949
S10-S19	S10	379
S10-S19	S11	306
S10-S19	S12	3539
S10-S19	S13	470
S10-S19	S14	602
S10-S19	S15	24
S10-S19	S16	29
S10-S19	S17	<10

For peer review only

S10-S19	S18	<10
S10-S19	S19	652
S20-S29	S20	2820
S20-S29	S21	531
S20-S29	S22	14287
S20-S29	S23	83
S20-S29	S24	49
S20-S29	S25	41
S20-S29	S26	20
S20-S29	S27	3099
S20-S29	S28	54
S20-S29	S29	1343
S30-S39	S30	2998
S30-S39	S31	921
S30-S39	S32	22852
S30-S39	S33	91
S30-S39	S34	63
S30-S39	S35	86
S30-S39	S36	994
S30-S39	S37	430
S30-S39	S38	53
S30-S39	S39	3506
S40-S49	S40	1428
S40-S49	S41	429
S40-S49	S42	28092
S40-S49	S43	4615
S40-S49	S44	65
S40-S49	S45	50
S40-S49	S46	619
S40-S49	S47	27
S40-S49	S48	<10
S40-S49	S49	1309
S50-S59	S50	638
S50-S59	S51	1654
S50-S59	S52	37213
S50-S59	S53	491
S50-S59	S54	68
S50-S59	S55	43
S50-S59	S56	543
S50-S59	S57	24
S50-S59	S58	<10
S50-S59	S59	294
S60-S69	S60	820
S60-S69	S61	8375
S60-S69	S62	7364
S60-S69	S63	1407

For peer review only

S60-S69	S64	977
S60-S69	S65	126
S60-S69	S66	2279
S60-S69	S67	583
S60-S69	S68	1374
S60-S69	S69	964
S70-S79	S70	4456
S70-S79	S71	298
S70-S79	S72	128265
S70-S79	S73	1110
S70-S79	S74	22
S70-S79	S75	66
S70-S79	S76	1289
S70-S79	S77	22
S70-S79	S78	11
S70-S79	S79	6696
S80-S89	S80	3855
S80-S89	S81	3644
S80-S89	S82	38308
S80-S89	S83	2082
S80-S89	S84	19
S80-S89	S85	62
S80-S89	S86	1397
S80-S89	S87	78
S80-S89	S88	53
S80-S89	S89	2775
S90-S99	S90	923
S90-S99	S91	686
S90-S99	S92	4814
S90-S99	S93	823
S90-S99	S94	<10
S90-S99	S95	25
S90-S99	S96	118
S90-S99	S97	78
S90-S99	S98	72
S90-S99	S99	1056
T00-T07	T00	537
T00-T07	T01	126
T00-T07	T02	419
T00-T07	T03	22
T00-T07	T04	38
T00-T07	T05	<10
T00-T07	T06	29
T00-T07	T07	55
T08-T14	T08	270
T08-T14	T09	458

For peer review only

T08-T14	T10	20
T08-T14	T11	329
T08-T14	T12	<10
T08-T14	T13	690
T08-T14	T14	1916
T15-T19	T15	86
T15-T19	T16	84
T15-T19	T17	1763
T15-T19	T18	4624
T15-T19	T19	126
T20-T25	T20	649
T20-T25	T21	823
T20-T25	T22	489
T20-T25	T23	606
T20-T25	T24	545
T20-T25	T25	363
T26-T28	T26	59
T26-T28	T27	11
T26-T28	T28	31
T29-T32	T29	225
T29-T32	T30	78
T29-T32	T31	63
T29-T32	T32	<10
T33-T35	T33	<10
T33-T35	T34	27
T33-T35	T35	10
T36-T50	T36	344
T36-T50	T37	200
T36-T50	T38	1043
T36-T50	T39	13526
T36-T50	T40	6647
T36-T50	T41	49
T36-T50	T42	7832
T36-T50	T43	8970
T36-T50	T44	1166
T36-T50	T45	1213
T36-T50	T46	1791
T36-T50	T47	178
T36-T50	T48	147
T36-T50	T49	104
T36-T50	T50	3942
T51-T65	T51	432
T51-T65	T52	125
T51-T65	T53	10
T51-T65	T54	162
T51-T65	T55	18

For peer review only

T51-T65	T56	234
T51-T65	T57	<10
T51-T65	T58	223
T51-T65	T59	1598
T51-T65	T60	84
T51-T65	T61	24
T51-T65	T62	120
T51-T65	T63	257
T51-T65	T64	<10
T51-T65	T65	323
T66-T78	T66	139
T66-T78	T67	33
T66-T78	T68	1088
T66-T78	T69	19
T66-T78	T70	94
T66-T78	T71	110
T66-T78	T72	<10
T66-T78	T73	21
T66-T78	T74	10
T66-T78	T75	267
T66-T78	T76	<10
T66-T78	T77	<10
T66-T78	T78	4680
T79-T79	T79	1904
T80-T88	T80	679
T80-T88	T81	46443
T80-T88	T82	25827
T80-T88	T83	13664
T80-T88	T84	44484
T80-T88	T85	15471
T80-T88	T86	1883
T80-T88	T87	1659
T80-T88	T88	2050
W00-W19	W00	<10
W00-W19	W01	<10
W00-W19	W02	<10
W00-W19	W03	<10
W00-W19	W04	<10
W00-W19	W05	<10
W00-W19	W06	<10
W00-W19	W07	<10
W00-W19	W08	<10
W00-W19	W09	<10
W00-W19	W10	<10
W00-W19	W11	<10
W00-W19	W12	<10

For peer review only

W00-W19	W13	<10
W00-W19	W14	<10
W00-W19	W15	<10
W00-W19	W16	<10
W00-W19	W17	<10
W00-W19	W18	<10
W00-W19	W19	<10
Z00-Z13	Z00	542
Z00-Z13	Z01	293
Z00-Z13	Z02	<10
Z00-Z13	Z03	12713
Z00-Z13	Z04	5106
Z00-Z13	Z05	<10
Z00-Z13	Z06	<10
Z00-Z13	Z07	<10
Z00-Z13	Z08	63043
Z00-Z13	Z09	68414
Z00-Z13	Z10	<10
Z00-Z13	Z11	507
Z00-Z13	Z12	9653
Z00-Z13	Z13	8682
Z20-Z29	Z20	<10
Z20-Z29	Z21	<10
Z20-Z29	Z22	15
Z20-Z29	Z23	<10
Z20-Z29	Z24	<10
Z20-Z29	Z25	<10
Z20-Z29	Z26	<10
Z20-Z29	Z27	<10
Z20-Z29	Z28	<10
Z20-Z29	Z29	1198
Z30-Z39	Z30	13783
Z30-Z39	Z31	437
Z30-Z39	Z32	<10
Z30-Z39	Z33	<10
Z30-Z39	Z34	<10
Z30-Z39	Z35	<10
Z30-Z39	Z36	<10
Z30-Z39	Z37	<10
Z30-Z39	Z38	<10
Z30-Z39	Z39	<10
Z40-Z54	Z40	1433
Z40-Z54	Z41	2832
Z40-Z54	Z42	11095
Z40-Z54	Z43	12974
Z40-Z54	Z44	679

For peer review only

Z40-Z54	Z45	12442
Z40-Z54	Z46	23792
Z40-Z54	Z47	12123
Z40-Z54	Z48	4384
Z40-Z54	Z49	1307
Z40-Z54	Z50	7458
Z40-Z54	Z51	3707
Z40-Z54	Z52	1044
Z40-Z54	Z53	128753
Z40-Z54	Z54	3016
Z55-Z65	Z55	<10
Z55-Z65	Z56	<10
Z55-Z65	Z57	<10
Z55-Z65	Z58	<10
Z55-Z65	Z59	43
Z55-Z65	Z60	196
Z55-Z65	Z61	<10
Z55-Z65	Z62	<10
Z55-Z65	Z63	34
Z55-Z65	Z64	<10
Z55-Z65	Z65	<10
Z70-Z76	Z70	<10
Z70-Z76	Z71	706
Z70-Z76	Z72	103
Z70-Z76	Z73	215
Z70-Z76	Z74	4252
Z70-Z76	Z75	6190
Z70-Z76	Z76	100
Z80-Z99	Z80	<10
Z80-Z99	Z81	<10
Z80-Z99	Z82	<10
Z80-Z99	Z83	<10
Z80-Z99	Z84	<10
Z80-Z99	Z85	550
Z80-Z99	Z86	<10
Z80-Z99	Z87	<10
Z80-Z99	Z88	<10
Z80-Z99	Z89	<10
Z80-Z99	Z90	51
Z80-Z99	Z91	69
Z80-Z99	Z92	17
Z80-Z99	Z93	<10
Z80-Z99	Z94	<10
Z80-Z99	Z95	<10
Z80-Z99	Z96	<10
Z80-Z99	Z97	<10

For peer review only

Z80-Z99	Z98	133
Z80-Z99	Z99	<10

For peer review only

ICD10	Unstratified	Male	Female	SIMD 1	SIMD 2	SIMD 3	SIMD 4
J09-J18	110,985	54,896	56,089	7,252	7,776	8,751	9,459
R00-R09	225,504	109,642	115,862	15,272	18,020	18,360	18,633
C30-C39	54,178	29,657	24,521	2,895	3,368	3,643	4,210
R10-R19	174,055	68,258	105,797	12,079	14,415	14,372	14,847
R50-R69	157,357	71,014	86,343	11,363	12,493	13,392	14,408
C15-C26	71,981	40,064	31,917	6,269	6,442	6,434	6,747
I30-I52	142,898	73,086	69,812	11,666	12,084	12,791	13,737
C76-C80	39,339	18,077	21,262	3,490	3,437	3,549	3,828
Z40-Z54	157,841	77,906	79,935	12,101	13,893	14,324	14,538
I60-I69	100,907	47,573	53,334	7,498	8,086	8,810	9,378
K55-K63	206,178	91,114	115,064	16,228	18,523	18,172	18,214
J40-J47	78,467	33,777	44,690	3,337	4,159	5,065	5,654
I20-I25	175,605	104,230	71,375	12,769	14,306	15,055	15,919
N30-N39	126,329	51,830	74,499	10,422	11,152	11,750	11,300
K20-K31	172,206	80,887	91,319	11,156	14,273	14,354	14,637
J20-J22	77,520	35,833	41,687	4,647	5,420	6,174	6,656
S70-S79	78,231	22,042	56,189	6,346	6,608	7,494	7,665
A30-A49	33,613	15,982	17,631	2,244	2,533	2,688	3,109
T80-T88	75,217	37,899	37,318	6,647	6,827	7,006	7,368
N17-N19	37,213	19,032	18,181	2,462	2,760	3,053	3,327
I80-I89	84,073	39,544	44,529	7,315	8,149	7,999	7,857
K90-K93	47,091	23,934	23,157	3,048	3,542	3,754	4,054
I70-I79	47,410	27,229	20,181	3,235	3,392	3,922	4,307
K50-K52	59,183	22,753	36,430	4,277	4,976	5,123	5,226
S00-S09	64,925	36,484	28,441	4,311	4,563	4,997	5,435
C50-C50	39,358	339	39,019	4,213	4,168	3,981	4,077
C60-C63	22,312	22,311	<10	2,663	2,499	2,329	2,326
G30-G32	4,655	2,024	2,631	412	431	473	430
TOTAL	2,664,631	1,257,417	<1,407,223	195,617	218,295	227,815	237,346

For peer review only

SIMD 5	SIMD 6	SIMD 7	SIMD 8	SIMD 9	SIMD 10
10,358	11,004	12,978	13,930	14,406	15,071
20,418	22,175	25,017	27,572	28,979	31,058
4,779	5,418	6,287	7,040	7,831	8,707
16,364	18,102	19,207	20,624	21,429	22,616
14,842	15,727	17,003	18,829	19,170	20,130
7,147	7,280	7,966	8,129	7,844	7,723
14,379	14,312	15,845	16,391	16,281	15,412
4,028	4,029	4,251	4,216	4,389	4,122
15,932	16,360	17,423	17,777	18,091	17,402
9,805	10,290	10,952	11,903	12,069	12,116
19,789	20,934	22,228	23,380	24,145	24,565
6,762	7,675	9,291	10,862	11,983	13,679
17,142	18,025	19,688	20,874	21,235	20,592
12,142	12,490	13,844	14,379	14,377	14,473
16,561	17,478	18,855	20,361	21,734	22,797
7,380	7,662	8,743	9,513	10,049	11,276
7,939	7,703	8,451	8,705	8,716	8,604
3,195	3,363	3,929	4,164	4,298	4,090
7,477	7,579	8,212	8,293	8,020	7,788
3,578	3,802	4,254	4,650	4,738	4,589
8,384	8,603	8,884	8,948	8,943	8,991
4,311	4,639	5,270	5,536	6,098	6,839
4,698	4,926	5,415	5,809	5,821	5,885
5,609	5,907	6,494	6,936	7,135	7,500
5,927	6,164	6,991	7,960	8,607	9,970
3,958	3,966	3,939	3,869	3,787	3,400
2,347	2,177	2,120	2,113	1,996	1,742
414	423	483	488	537	564
255,665	268,213	294,020	313,251	322,708	331,701

For peer review only

ICD10	Jnstratifiec	Male	Female	SIMD 1	SIMD 2	SIMD 3	SIMD 4	SIMD 5
J09-J18	1.66	1.68	1.66	1.74	1.64	1.65	1.60	1.65
R00-R09	0.73	0.76	0.71	0.71	0.70	0.73	0.74	0.75
C30-C39	3.05	2.92	3.23	3.12	3.19	3.17	3.10	3.19
R10-R19	0.94	1.00	0.89	1.02	0.97	0.96	0.94	0.94
R50-R69	0.98	1.07	0.91	0.98	0.95	0.92	0.95	0.97
C15-C26	2.10	2.09	2.10	2.15	2.18	2.11	2.10	2.08
I30-I52	0.99	0.98	1.01	0.95	1.00	0.91	0.96	1.00
C76-C80	3.26	3.18	3.35	3.41	3.38	3.34	3.30	3.19
Z40-Z54	0.76	0.77	0.76	0.84	0.80	0.80	0.77	0.76
I60-I69	1.12	1.03	1.19	1.15	1.16	1.06	1.15	1.14
K55-K63	0.54	0.56	0.53	0.61	0.58	0.54	0.56	0.56
J40-J47	1.38	1.36	1.41	1.41	1.38	1.37	1.40	1.41
I20-I25	0.61	0.53	0.70	0.61	0.63	0.62	0.63	0.62
N30-N39	0.84	0.83	0.84	0.88	0.88	0.84	0.87	0.86
K20-K31	0.60	0.68	0.54	0.63	0.64	0.55	0.59	0.57
J20-J22	1.27	1.35	1.21	1.29	1.28	1.21	1.25	1.24
S70-S79	0.97	1.16	0.89	0.92	0.96	0.92	0.94	0.95
A30-A49	1.89	1.83	1.96	2.05	1.94	1.93	1.84	1.91
T80-T88	0.84	0.88	0.80	0.84	0.88	0.83	0.85	0.86
N17-N19	1.64	1.63	1.66	1.77	1.70	1.63	1.63	1.61
I80-I89	0.66	0.65	0.67	0.77	0.70	0.61	0.67	0.63
K90-K93	1.09	1.16	1.02	1.11	1.08	1.02	1.08	1.07
I70-I79	1.08	1.01	1.18	1.13	1.10	1.03	1.05	1.05
K50-K52	0.82	0.87	0.78	0.73	0.83	0.83	0.84	0.80
S00-S09	0.74	0.84	0.63	0.78	0.68	0.68	0.73	0.70
C50-C50	1.17	1.53	1.17	1.31	1.30	1.21	1.20	1.15
C60-C63	1.17	1.18	NA	1.29	1.18	1.21	1.29	1.23
G30-G32	1.19	1.39	1.06	1.33	1.21	1.16	1.23	1.19

For peer review only

SIMD 6	SIMD 7	SIMD 8	SIMD 9	SIMD 10
1.67	1.69	1.71	1.68	1.66
0.75	0.76	0.72	0.71	0.70
3.06	3.06	2.96	2.96	2.92
0.94	0.92	0.94	0.92	0.90
1.01	1.01	1.00	1.00	1.00
2.13	2.07	2.09	2.08	2.01
1.02	1.05	1.03	1.00	0.98
3.29	3.21	3.15	3.26	3.19
0.77	0.75	0.74	0.70	0.73
1.14	1.12	1.09	1.11	1.05
0.57	0.53	0.54	0.51	0.48
1.43	1.39	1.40	1.37	1.33
0.62	0.62	0.60	0.56	0.53
0.88	0.85	0.81	0.81	0.76
0.60	0.60	0.61	0.60	0.62
1.29	1.30	1.29	1.27	1.31
0.95	1.01	1.00	0.98	0.99
1.96	1.94	1.83	1.82	1.82
0.85	0.83	0.81	0.83	0.82
1.65	1.72	1.64	1.56	1.58
0.62	0.62	0.67	0.65	0.70
1.11	1.13	1.11	1.07	1.09
1.08	1.08	1.10	1.06	1.06
0.84	0.88	0.82	0.79	0.81
0.71	0.76	0.73	0.76	0.76
1.16	1.09	1.12	1.12	1.07
1.16	1.16	1.16	1.14	0.98
1.26	1.26	1.18	1.11	1.07

For peer review only

ICD10	Jnstratifiec	Male	Female	SIMD 1	SIMD 2	SIMD 3	SIMD 4	SIMD 5
J09-J18	0.0340	0.0354	0.0329	0.0308	0.0287	0.0319	0.0312	0.0327
R00-R09	0.0304	0.0319	0.0290	0.0266	0.0283	0.0295	0.0286	0.0293
C30-C39	0.0304	0.0331	0.0281	0.0220	0.0241	0.0255	0.0270	0.0293
R10-R19	0.0300	0.0262	0.0334	0.0300	0.0314	0.0305	0.0288	0.0294
R50-R69	0.0284	0.0291	0.0278	0.0272	0.0266	0.0272	0.0282	0.0275
C15-C26	0.0277	0.0320	0.0238	0.0328	0.0314	0.0300	0.0293	0.0286
I30-I52	0.0261	0.0274	0.0249	0.0270	0.0271	0.0259	0.0272	0.0275
C76-C80	0.0236	0.0220	0.0252	0.0290	0.0260	0.0262	0.0261	0.0246
Z40-Z54	0.0222	0.0230	0.0216	0.0248	0.0250	0.0254	0.0230	0.0233
I60-I69	0.0207	0.0187	0.0225	0.0210	0.0210	0.0206	0.0223	0.0215
K55-K63	0.0206	0.0197	0.0215	0.0243	0.0240	0.0216	0.0212	0.0214
J40-J47	0.0200	0.0176	0.0223	0.0115	0.0129	0.0154	0.0164	0.0184
I20-I25	0.0196	0.0211	0.0176	0.0190	0.0203	0.0206	0.0208	0.0206
N30-N39	0.0195	0.0165	0.0223	0.0224	0.0219	0.0219	0.0203	0.0200
K20-K31	0.0192	0.0209	0.0173	0.0172	0.0206	0.0174	0.0178	0.0182
J20-J22	0.0181	0.0186	0.0179	0.0147	0.0156	0.0166	0.0172	0.0176
S70-S79	0.0140	0.0098	0.0176	0.0143	0.0142	0.0153	0.0149	0.0145
A30-A49	0.0117	0.0112	0.0122	0.0112	0.0110	0.0115	0.0118	0.0117
T80-T88	0.0117	0.0128	0.0106	0.0136	0.0134	0.0129	0.0129	0.0124
N17-N19	0.0112	0.0119	0.0107	0.0106	0.0105	0.0110	0.0112	0.0111
I80-I89	0.0102	0.0099	0.0106	0.0138	0.0129	0.0108	0.0109	0.0102
K90-K93	0.0095	0.0106	0.0084	0.0083	0.0086	0.0085	0.0090	0.0088
I70-I79	0.0094	0.0105	0.0084	0.0089	0.0083	0.0090	0.0094	0.0095
K50-K52	0.0089	0.0076	0.0101	0.0076	0.0093	0.0094	0.0090	0.0086
S00-S09	0.0088	0.0117	0.0063	0.0082	0.0069	0.0075	0.0082	0.0080
C50-C50	0.0084	NA	0.0161	0.0135	0.0122	0.0106	0.0101	0.0087
C60-C63	0.0048	0.0101	NA	0.0084	0.0066	0.0062	0.0062	0.0055
G30-G32	0.0010	0.0011	0.0010	0.0013	0.0012	0.0012	0.0011	0.0009
TOTAL	1.0000	1.0000	1.0000	1.0000	1.0000	1.0000	1.0000	1.0000

For peer review only

SIMD 6	SIMD 7	SIMD 8	SIMD 9	SIMD 10
0.0331	0.0360	0.0371	0.0370	0.0376
0.0301	0.0314	0.0309	0.0317	0.0330
0.0299	0.0316	0.0325	0.0356	0.0383
0.0308	0.0291	0.0302	0.0301	0.0306
0.0286	0.0282	0.0294	0.0293	0.0302
0.0280	0.0271	0.0265	0.0250	0.0234
0.0263	0.0272	0.0263	0.0249	0.0228
0.0239	0.0224	0.0207	0.0219	0.0198
0.0228	0.0215	0.0205	0.0194	0.0192
0.0211	0.0202	0.0203	0.0205	0.0192
0.0215	0.0194	0.0196	0.0189	0.0176
0.0198	0.0212	0.0236	0.0252	0.0274
0.0203	0.0202	0.0197	0.0183	0.0165
0.0199	0.0193	0.0181	0.0178	0.0166
0.0188	0.0186	0.0193	0.0200	0.0213
0.0178	0.0186	0.0192	0.0196	0.0223
0.0133	0.0140	0.0136	0.0131	0.0128
0.0119	0.0125	0.0119	0.0120	0.0112
0.0116	0.0111	0.0105	0.0102	0.0097
0.0113	0.0120	0.0119	0.0113	0.0109
0.0097	0.0090	0.0093	0.0089	0.0095
0.0093	0.0097	0.0096	0.0100	0.0113
0.0096	0.0096	0.0099	0.0095	0.0094
0.0090	0.0093	0.0089	0.0087	0.0092
0.0079	0.0087	0.0091	0.0100	0.0113
0.0083	0.0070	0.0067	0.0065	0.0055
0.0046	0.0040	0.0038	0.0035	0.0026
0.0010	0.0010	0.0009	0.0009	0.0009
1.0000	1.0000	1.0000	1.0000	1.0000

For peer review only

ICD10	Unstratified	Male	Female	SIMD 1	SIMD 2	SIMD 3
J09-J18	-0.19 (0.01)	-0.13 (0.02)	-0.24 (0.02)	-0.23 (0.06)	-0.25 (0.05)	-0.16 (0.05)
R00-R09	0.16 (0.01)	0.20 (0.01)	0.12 (0.01)	0.11 (0.03)	0.12 (0.02)	0.11 (0.02)
C30-C39	0.32 (0.02)	0.44 (0.03)	0.19 (0.03)	0.24 (0.09)	0.34 (0.07)	0.39 (0.07)
R10-R19	0.11 (0.01)	0.13 (0.01)	0.09 (0.01)	0.03 (0.03)	0.07 (0.03)	0.11 (0.03)
R50-R69	0.08 (0.01)	0.13 (0.01)	0.03 (0.01)	0.03 (0.04)	0.03 (0.03)	0.04 (0.03)
C15-C26	0.27 (0.02)	0.29 (0.02)	0.23 (0.02)	0.26 (0.05)	0.28 (0.05)	0.31 (0.05)
I30-I52	0.23 (0.01)	0.23 (0.01)	0.21 (0.02)	0.16 (0.04)	0.27 (0.04)	0.19 (0.04)
C76-C80	0.22 (0.02)	0.27 (0.03)	0.19 (0.03)	0.06 (0.06)	0.18 (0.06)	0.17 (0.06)
Z40-Z54	-0.04 (0.01)	-0.03 (0.01)	-0.05 (0.01)	-0.03 (0.03)	-0.05 (0.03)	-0.01 (0.03)
I60-I69	0.21 (0.01)	0.22 (0.02)	0.19 (0.02)	0.17 (0.06)	0.14 (0.05)	0.16 (0.05)
K55-K63	0.02 (0.01)	0.01 (0.01)	0.03 (0.01)	0.03 (0.03)	0.05 (0.02)	0.01 (0.02)
J40-J47	0.26 (0.01)	0.34 (0.02)	0.21 (0.02)	0.39 (0.08)	0.24 (0.07)	0.32 (0.06)
I20-I25	0.43 (0.01)	0.40 (0.01)	0.47 (0.02)	0.39 (0.03)	0.36 (0.03)	0.38 (0.03)
N30-N39	0.03 (0.01)	0.09 (0.02)	-0.02 (0.01)	0.12 (0.04)	0.07 (0.03)	0.04 (0.04)
K20-K31	0.33 (0.01)	0.38 (0.01)	0.28 (0.01)	0.29 (0.03)	0.32 (0.03)	0.39 (0.03)
J20-J22	-0.13 (0.02)	-0.08 (0.02)	-0.17 (0.02)	-0.02 (0.07)	-0.14 (0.06)	-0.20 (0.06)
S70-S79	0.12 (0.02)	0.09 (0.03)	0.13 (0.03)	0.02 (0.09)	0.06 (0.09)	0.15 (0.08)
A30-A49	-0.43 (0.02)	-0.40 (0.03)	-0.46 (0.03)	-0.35 (0.09)	-0.50 (0.08)	-0.31 (0.08)
T80-T88	-0.05 (0.01)	-0.03 (0.02)	-0.08 (0.02)	0.01 (0.04)	-0.10 (0.04)	-0.07 (0.04)
N17-N19	0.04 (0.02)	0.09 (0.03)	-0.01 (0.03)	0.03 (0.10)	0.06 (0.09)	0.07 (0.09)
I80-I89	0.24 (0.01)	0.21 (0.01)	0.27 (0.01)	0.25 (0.03)	0.23 (0.03)	0.26 (0.03)
K90-K93	-0.04 (0.02)	0.02 (0.02)	-0.11 (0.02)	-0.14 (0.06)	-0.08 (0.06)	-0.06 (0.06)
I70-I79	0.44 (0.02)	0.39 (0.02)	0.51 (0.03)	0.56 (0.08)	0.48 (0.08)	0.50 (0.07)
K50-K52	0.34 (0.01)	0.36 (0.02)	0.33 (0.02)	0.36 (0.05)	0.44 (0.05)	0.33 (0.04)
S00-S09	0.19 (0.01)	0.26 (0.02)	0.05 (0.02)	0.04 (0.06)	0.04 (0.06)	0.13 (0.05)
C50-C50	0.20 (0.01)	NA	0.20 (0.02)	0.20 (0.05)	0.17 (0.04)	0.17 (0.05)
C60-C63	0.02 (0.03)	0.02 (0.03)	NA	-0.07 (0.09)	-0.12 (0.08)	-0.04 (0.10)
G30-G32	0.27 (0.08)	0.30 (0.10)	0.23 (0.13)	NA	NA	0.16 (0.28)
TOTAL	0.13 (0.00)	0.16 (0.00)	0.10 (0.00)	0.10 (0.01)	0.11 (0.01)	0.13 (0.01)

For peer review only

SIMD 4	SIMD 5	SIMD 6	SIMD 7	SIMD 8	SIMD 9
-0.16 (0.05)	-0.18 (0.05)	-0.16 (0.04)	-0.17 (0.04)	-0.20 (0.04)	-0.19 (0.04)
0.11 (0.02)	0.10 (0.02)	0.13 (0.02)	0.20 (0.02)	0.18 (0.02)	0.21 (0.02)
0.48 (0.07)	0.36 (0.06)	0.39 (0.06)	0.31 (0.06)	0.32 (0.05)	0.27 (0.05)
0.11 (0.03)	0.11 (0.02)	0.14 (0.02)	0.11 (0.02)	0.12 (0.02)	0.11 (0.02)
0.11 (0.03)	0.07 (0.03)	0.09 (0.03)	0.11 (0.03)	0.10 (0.03)	0.08 (0.03)
0.26 (0.05)	0.32 (0.05)	0.23 (0.05)	0.24 (0.05)	0.28 (0.05)	0.23 (0.05)
0.17 (0.04)	0.19 (0.04)	0.23 (0.04)	0.22 (0.04)	0.25 (0.03)	0.28 (0.03)
0.33 (0.06)	0.20 (0.06)	0.21 (0.06)	0.17 (0.06)	0.24 (0.06)	0.29 (0.06)
0.02 (0.03)	0.01 (0.03)	-0.03 (0.03)	-0.03 (0.02)	-0.03 (0.02)	-0.08 (0.02)
0.22 (0.05)	0.15 (0.05)	0.25 (0.04)	0.23 (0.04)	0.22 (0.04)	0.19 (0.04)
-0.00 (0.02)	0.04 (0.02)	0.04 (0.02)	0.06 (0.02)	0.05 (0.02)	0.02 (0.02)
0.25 (0.06)	0.24 (0.05)	0.28 (0.05)	0.27 (0.04)	0.25 (0.04)	0.24 (0.04)
0.38 (0.03)	0.40 (0.03)	0.41 (0.03)	0.49 (0.03)	0.48 (0.02)	0.46 (0.02)
0.06 (0.04)	0.02 (0.03)	0.00 (0.03)	0.02 (0.03)	-0.02 (0.03)	-0.00 (0.03)
0.38 (0.03)	0.37 (0.02)	0.34 (0.02)	0.32 (0.02)	0.33 (0.02)	0.31 (0.02)
-0.10 (0.05)	-0.12 (0.05)	-0.08 (0.05)	-0.13 (0.04)	-0.12 (0.04)	-0.18 (0.04)
0.14 (0.08)	0.09 (0.07)	0.17 (0.07)	0.06 (0.07)	0.12 (0.06)	0.12 (0.06)
-0.43 (0.07)	-0.41 (0.07)	-0.51 (0.07)	-0.43 (0.06)	-0.43 (0.06)	-0.49 (0.06)
-0.05 (0.04)	-0.06 (0.04)	-0.04 (0.04)	-0.02 (0.04)	-0.07 (0.04)	-0.14 (0.04)
0.04 (0.08)	0.06 (0.08)	0.05 (0.07)	0.04 (0.07)	0.01 (0.07)	0.05 (0.06)
0.27 (0.03)	0.26 (0.03)	0.22 (0.03)	0.26 (0.03)	0.26 (0.03)	0.22 (0.03)
0.01 (0.06)	0.03 (0.05)	-0.08 (0.05)	-0.05 (0.05)	0.01 (0.05)	-0.03 (0.04)
0.42 (0.07)	0.41 (0.06)	0.48 (0.06)	0.46 (0.06)	0.38 (0.05)	0.37 (0.05)
0.41 (0.05)	0.37 (0.04)	0.36 (0.04)	0.38 (0.04)	0.31 (0.04)	0.30 (0.04)
0.17 (0.05)	0.10 (0.05)	0.20 (0.04)	0.18 (0.04)	0.21 (0.04)	0.24 (0.04)
0.17 (0.05)	0.17 (0.05)	0.25 (0.05)	0.21 (0.05)	0.23 (0.05)	0.25 (0.05)
0.16 (0.09)	-0.04 (0.09)	0.12 (0.10)	0.10 (0.10)	0.10 (0.10)	0.07 (0.10)
NA	0.18 (0.28)	0.11 (0.27)	0.27 (0.25)	0.15 (0.22)	0.27 (0.20)
0.15 (0.01)	0.13 (0.01)	0.14 (0.01)	0.13 (0.01)	0.14 (0.01)	0.12 (0.01)

SIMD 10
-0.19 (0.03)
0.22 (0.02)
0.26 (0.04)
0.12 (0.02)
0.07 (0.02)
0.27 (0.05)
0.30 (0.03)
0.36 (0.06)
-0.11 (0.02)
0.29 (0.04)
-0.06 (0.02)
0.24 (0.03)
0.47 (0.02)
-0.01 (0.03)
0.27 (0.02)
-0.15 (0.04)
0.15 (0.05)
-0.41 (0.06)
0.01 (0.03)
0.01 (0.06)
0.19 (0.03)
-0.05 (0.04)
0.46 (0.05)
0.24 (0.04)
0.31 (0.03)
0.22 (0.05)
-0.04 (0.10)
0.43 (0.18)
0.13 (0.01)

For peer review only

ICD10	Unstratified	Male	Female	SIMD 1	SIMD 2	SIMD 3
J09-J18	0.15 (0.04)	0.11 (0.06)	0.19 (0.03)	-0.08 (0.22)	0.19 (0.13)	0.05 (0.25)
R00-R09	0.23 (0.04)	0.22 (0.05)	0.24 (0.05)	0.18 (0.15)	0.24 (0.11)	0.24 (0.12)
C30-C39	0.21 (0.05)	0.21 (0.04)	0.20 (0.07)	0.22 (0.23)	0.15 (0.19)	0.34 (0.18)
R10-R19	0.12 (0.04)	0.10 (0.05)	0.13 (0.05)	0.33 (0.11)	-0.07 (0.08)	-0.08 (0.18)
R50-R69	0.03 (0.03)	0.07 (0.03)	-0.02 (0.04)	0.06 (0.13)	-0.13 (0.11)	-0.16 (0.08)
C15-C26	0.40 (0.04)	0.37 (0.05)	0.43 (0.05)	0.50 (0.10)	0.50 (0.13)	0.45 (0.16)
I30-I52	0.18 (0.02)	0.29 (0.03)	0.08 (0.03)	0.38 (0.11)	0.20 (0.07)	-0.04 (0.13)
C76-C80	0.37 (0.07)	0.21 (0.09)	0.50 (0.05)	0.35 (0.14)	0.06 (0.24)	0.46 (0.19)
Z40-Z54	0.30 (0.04)	0.36 (0.06)	0.21 (0.03)	0.51 (0.12)	0.30 (0.12)	0.14 (0.06)
I60-I69	0.20 (0.03)	0.27 (0.05)	0.15 (0.04)	0.34 (0.10)	0.11 (0.07)	-0.03 (0.09)
K55-K63	0.22 (0.02)	0.30 (0.03)	0.16 (0.03)	0.37 (0.10)	0.35 (0.12)	0.16 (0.10)
J40-J47	0.24 (0.05)	0.20 (0.08)	0.27 (0.04)	-0.05 (0.24)	0.30 (0.10)	0.22 (0.13)
I20-I25	0.26 (0.03)	0.35 (0.03)	0.17 (0.05)	0.27 (0.21)	0.27 (0.12)	0.10 (0.10)
N30-N39	-0.21 (0.04)	-0.29 (0.04)	-0.16 (0.05)	0.00 (0.07)	-0.13 (0.11)	-0.26 (0.07)
K20-K31	0.13 (0.05)	0.11 (0.05)	0.16 (0.07)	-0.05 (0.10)	-0.11 (0.11)	0.01 (0.14)
J20-J22	-0.11 (0.02)	-0.09 (0.05)	-0.12 (0.03)	-0.32 (0.16)	-0.22 (0.08)	-0.07 (0.09)
S70-S79	-0.02 (0.02)	0.09 (0.06)	-0.07 (0.02)	0.26 (0.15)	-0.03 (0.10)	0.10 (0.10)
A30-A49	0.06 (0.10)	0.02 (0.14)	0.12 (0.10)	0.32 (0.24)	-0.30 (0.23)	-0.33 (0.26)
T80-T88	0.18 (0.06)	0.16 (0.10)	0.23 (0.05)	0.19 (0.25)	0.06 (0.15)	0.05 (0.06)
N17-N19	0.20 (0.06)	0.14 (0.06)	0.26 (0.08)	0.02 (0.25)	-0.09 (0.21)	0.23 (0.14)
I80-I89	0.24 (0.05)	0.37 (0.05)	0.09 (0.06)	0.42 (0.22)	0.05 (0.23)	0.10 (0.19)
K90-K93	0.19 (0.07)	0.22 (0.08)	0.12 (0.08)	-0.35 (0.13)	-0.08 (0.25)	-0.16 (0.20)
I70-I79	0.09 (0.04)	0.14 (0.04)	0.02 (0.06)	0.43 (0.20)	-0.11 (0.12)	0.09 (0.12)
K50-K52	0.12 (0.03)	0.14 (0.03)	0.09 (0.04)	-0.17 (0.16)	0.29 (0.13)	-0.12 (0.24)
S00-S09	0.02 (0.05)	0.18 (0.05)	-0.16 (0.06)	-0.15 (0.18)	0.25 (0.16)	-0.05 (0.12)
C50-C50	1.16 (0.07)	1.15 (0.64)	1.15 (0.07)	0.56 (0.24)	1.74 (0.24)	1.70 (0.28)
C60-C63	0.70 (0.07)	0.70 (0.07)	NA	0.55 (0.27)	0.92 (0.19)	0.23 (0.19)
G30-G32	0.04 (0.14)	-0.04 (0.27)	0.06 (0.11)	0.15 (0.33)	0.25 (0.46)	-0.36 (0.33)
TOTAL	0.19 (0.04)	0.19 (0.05)	0.18 (0.05)	0.22 (0.16)	0.16 (0.13)	0.12 (0.14)

For peer review only

SIMD 4	SIMD 5	SIMD 6	SIMD 7	SIMD 8	SIMD 9
-0.02 (0.14)	0.20 (0.12)	0.32 (0.16)	0.19 (0.06)	0.19 (0.09)	0.11 (0.10)
0.28 (0.10)	0.03 (0.09)	0.22 (0.09)	0.19 (0.06)	0.30 (0.07)	0.19 (0.06)
0.39 (0.20)	0.12 (0.15)	0.34 (0.11)	-0.11 (0.13)	0.22 (0.12)	0.26 (0.14)
0.17 (0.09)	0.02 (0.07)	0.15 (0.11)	0.15 (0.07)	0.16 (0.11)	0.09 (0.14)
-0.16 (0.07)	-0.11 (0.04)	-0.09 (0.07)	0.22 (0.05)	0.14 (0.06)	-0.01 (0.06)
0.45 (0.07)	0.54 (0.15)	0.26 (0.10)	0.22 (0.12)	0.29 (0.12)	0.29 (0.12)
0.28 (0.12)	0.23 (0.04)	0.25 (0.07)	0.26 (0.08)	0.12 (0.05)	0.04 (0.09)
0.43 (0.22)	0.28 (0.18)	0.58 (0.19)	0.48 (0.20)	0.52 (0.18)	0.41 (0.15)
0.29 (0.07)	0.41 (0.07)	0.18 (0.09)	0.34 (0.06)	0.40 (0.08)	0.18 (0.11)
0.06 (0.07)	-0.09 (0.18)	0.33 (0.08)	0.22 (0.12)	0.16 (0.13)	0.34 (0.18)
0.13 (0.05)	0.32 (0.08)	0.11 (0.06)	0.23 (0.08)	0.16 (0.04)	0.03 (0.05)
0.30 (0.15)	0.11 (0.14)	0.09 (0.18)	0.22 (0.09)	0.18 (0.03)	0.37 (0.07)
0.37 (0.06)	0.23 (0.12)	0.29 (0.08)	0.24 (0.13)	0.41 (0.04)	0.18 (0.09)
-0.28 (0.07)	-0.17 (0.07)	-0.16 (0.10)	-0.30 (0.14)	-0.17 (0.07)	-0.28 (0.07)
0.39 (0.12)	0.06 (0.10)	0.11 (0.04)	0.02 (0.10)	0.16 (0.14)	0.18 (0.09)
-0.24 (0.08)	-0.17 (0.10)	-0.18 (0.11)	-0.11 (0.07)	0.14 (0.07)	-0.16 (0.06)
-0.22 (0.12)	-0.09 (0.07)	-0.12 (0.06)	0.16 (0.11)	-0.09 (0.11)	0.00 (0.12)
-0.29 (0.14)	0.42 (0.22)	-0.61 (0.18)	0.53 (0.33)	0.37 (0.14)	0.13 (0.19)
0.20 (0.16)	0.17 (0.06)	0.43 (0.10)	0.20 (0.15)	0.26 (0.12)	0.00 (0.11)
0.01 (0.06)	0.53 (0.21)	0.18 (0.19)	0.20 (0.15)	0.11 (0.11)	0.43 (0.17)
0.62 (0.31)	0.21 (0.19)	0.08 (0.18)	0.49 (0.15)	0.28 (0.13)	0.06 (0.12)
0.08 (0.16)	0.05 (0.16)	0.25 (0.13)	0.16 (0.09)	0.21 (0.14)	0.26 (0.15)
0.26 (0.15)	0.26 (0.18)	0.04 (0.19)	-0.00 (0.14)	-0.05 (0.10)	0.06 (0.06)
-0.38 (0.20)	0.35 (0.13)	0.15 (0.11)	0.09 (0.08)	0.03 (0.13)	0.24 (0.11)
-0.34 (0.15)	-0.21 (0.19)	0.22 (0.19)	-0.01 (0.18)	0.01 (0.08)	-0.09 (0.12)
1.45 (0.12)	1.17 (0.17)	1.59 (0.31)	0.96 (0.21)	0.88 (0.19)	1.03 (0.21)
0.62 (0.19)	0.88 (0.22)	0.86 (0.19)	0.38 (0.24)	0.77 (0.13)	1.29 (0.28)
-1.27 (0.28)	0.88 (0.35)	0.45 (0.43)	-1.24 (0.59)	0.55 (0.42)	0.30 (0.28)
0.18 (0.12)	0.17 (0.12)	0.19 (0.12)	0.18 (0.11)	0.21 (0.10)	0.16 (0.11)

For peer review only

SIMD 10
0.20 (0.07)
0.34 (0.09)
0.19 (0.12)
0.16 (0.08)
0.23 (0.06)
0.49 (0.14)
0.07 (0.10)
-0.14 (0.25)
0.24 (0.05)
0.39 (0.10)
0.44 (0.07)
0.32 (0.09)
0.24 (0.08)
-0.32 (0.06)
0.28 (0.10)
-0.03 (0.12)
-0.18 (0.11)
-0.22 (0.17)
0.20 (0.09)
0.15 (0.18)
0.14 (0.16)
0.63 (0.16)
0.07 (0.16)
0.24 (0.10)
0.18 (0.14)
0.81 (0.17)
0.62 (0.16)
-0.43 (0.12)
0.20 (0.11)

For peer review only

ICD10	Unstratified	Male	Female	SIMD 1	SIMD 2	SIMD 3
J09-J18	-0.02 (0.02)	-0.01 (0.03)	-0.04 (0.03)	-0.28 (0.12)	-0.03 (0.08)	-0.10 (0.12)
R00-R09	0.37 (0.02)	0.39 (0.03)	0.35 (0.02)	0.31 (0.08)	0.35 (0.06)	0.36 (0.06)
C30-C39	0.47 (0.03)	0.62 (0.03)	0.34 (0.04)	0.43 (0.14)	0.44 (0.12)	0.67 (0.11)
R10-R19	0.26 (0.02)	0.26 (0.03)	0.25 (0.03)	0.41 (0.06)	0.03 (0.05)	0.06 (0.09)
R50-R69	0.13 (0.02)	0.21 (0.02)	0.05 (0.02)	0.15 (0.07)	-0.04 (0.06)	-0.09 (0.05)
C15-C26	0.65 (0.02)	0.64 (0.03)	0.65 (0.03)	0.76 (0.07)	0.78 (0.08)	0.72 (0.09)
I30-I52	0.42 (0.02)	0.53 (0.02)	0.30 (0.02)	0.57 (0.07)	0.47 (0.05)	0.18 (0.07)
C76-C80	0.56 (0.04)	0.44 (0.05)	0.68 (0.04)	0.42 (0.10)	0.21 (0.13)	0.61 (0.11)
Z40-Z54	0.28 (0.02)	0.35 (0.03)	0.19 (0.02)	0.52 (0.07)	0.28 (0.06)	0.15 (0.04)
I60-I69	0.43 (0.02)	0.51 (0.03)	0.36 (0.03)	0.53 (0.08)	0.27 (0.06)	0.14 (0.07)
K55-K63	0.26 (0.01)	0.32 (0.02)	0.22 (0.02)	0.42 (0.05)	0.43 (0.06)	0.18 (0.06)
J40-J47	0.50 (0.03)	0.54 (0.04)	0.48 (0.03)	0.30 (0.14)	0.56 (0.08)	0.52 (0.09)
I20-I25	0.70 (0.02)	0.76 (0.02)	0.64 (0.03)	0.67 (0.10)	0.65 (0.06)	0.48 (0.06)
N30-N39	-0.23 (0.02)	-0.23 (0.03)	-0.22 (0.03)	0.11 (0.05)	-0.09 (0.06)	-0.23 (0.05)
K20-K31	0.47 (0.02)	0.47 (0.03)	0.46 (0.04)	0.25 (0.06)	0.22 (0.06)	0.39 (0.07)
J20-J22	-0.23 (0.02)	-0.16 (0.03)	-0.28 (0.03)	-0.34 (0.11)	-0.35 (0.07)	-0.23 (0.07)
S70-S79	0.10 (0.02)	0.19 (0.04)	0.06 (0.03)	0.32 (0.11)	0.05 (0.10)	0.25 (0.09)
A30-A49	-0.38 (0.05)	-0.41 (0.07)	-0.33 (0.06)	-0.01 (0.15)	-0.78 (0.13)	-0.65 (0.15)
T80-T88	0.15 (0.03)	0.13 (0.05)	0.18 (0.03)	0.22 (0.12)	0.01 (0.08)	0.00 (0.05)
N17-N19	0.21 (0.04)	0.21 (0.04)	0.22 (0.05)	0.04 (0.16)	-0.03 (0.14)	0.25 (0.11)
I80-I89	0.46 (0.03)	0.58 (0.03)	0.33 (0.03)	0.65 (0.11)	0.20 (0.11)	0.35 (0.10)
K90-K93	0.19 (0.04)	0.26 (0.05)	0.07 (0.04)	-0.44 (0.09)	-0.10 (0.13)	-0.16 (0.11)
I70-I79	0.52 (0.03)	0.53 (0.03)	0.51 (0.04)	0.92 (0.13)	0.34 (0.10)	0.57 (0.09)
K50-K52	0.45 (0.02)	0.50 (0.03)	0.42 (0.03)	0.17 (0.10)	0.72 (0.08)	0.20 (0.12)
S00-S09	0.11 (0.03)	0.34 (0.03)	-0.15 (0.04)	-0.19 (0.11)	0.25 (0.10)	0.05 (0.08)
C50-C50	1.36 (0.04)	NA	1.35 (0.04)	0.78 (0.13)	1.92 (0.13)	1.88 (0.14)
C60-C63	0.76 (0.04)	0.76 (0.04)	NA	0.53 (0.15)	0.87 (0.13)	0.21 (0.13)
G30-G32	NA	0.28 (0.17)	0.30 (0.14)	NA	NA	NA
TOTAL	0.31 (0.01)	0.35 (0.01)	0.28 (0.01)	0.34 (0.02)	0.28 (0.02)	0.25 (0.02)

For peer review only

SIMD 4	SIMD 5	SIMD 6	SIMD 7	SIMD 8	SIMD 9
-0.17 (0.08)	0.04 (0.07)	0.16 (0.09)	0.03 (0.05)	0.01 (0.06)	-0.10 (0.06)
0.39 (0.05)	0.15 (0.05)	0.36 (0.05)	0.38 (0.04)	0.46 (0.04)	0.37 (0.03)
0.74 (0.12)	0.44 (0.10)	0.67 (0.08)	0.14 (0.08)	0.51 (0.08)	0.45 (0.08)
0.32 (0.05)	0.15 (0.04)	0.34 (0.06)	0.28 (0.04)	0.31 (0.06)	0.20 (0.07)
-0.02 (0.05)	-0.03 (0.04)	0.02 (0.05)	0.34 (0.04)	0.27 (0.04)	0.09 (0.04)
0.70 (0.06)	0.82 (0.09)	0.48 (0.07)	0.45 (0.07)	0.58 (0.07)	0.51 (0.08)
0.48 (0.07)	0.42 (0.04)	0.50 (0.05)	0.51 (0.05)	0.38 (0.04)	0.32 (0.06)
0.67 (0.12)	0.46 (0.11)	0.80 (0.11)	0.64 (0.11)	0.73 (0.11)	0.68 (0.09)
0.33 (0.04)	0.44 (0.05)	0.17 (0.05)	0.32 (0.04)	0.40 (0.05)	0.09 (0.06)
0.29 (0.06)	0.09 (0.09)	0.59 (0.06)	0.46 (0.07)	0.39 (0.07)	0.56 (0.09)
0.16 (0.03)	0.37 (0.05)	0.18 (0.04)	0.31 (0.04)	0.22 (0.03)	0.06 (0.03)
0.59 (0.09)	0.35 (0.08)	0.37 (0.10)	0.50 (0.06)	0.43 (0.04)	0.61 (0.05)
0.77 (0.04)	0.63 (0.06)	0.71 (0.05)	0.71 (0.07)	0.90 (0.03)	0.65 (0.05)
-0.24 (0.05)	-0.18 (0.05)	-0.19 (0.06)	-0.32 (0.07)	-0.21 (0.05)	-0.33 (0.05)
0.79 (0.06)	0.43 (0.06)	0.45 (0.03)	0.35 (0.05)	0.49 (0.07)	0.49 (0.05)
-0.32 (0.07)	-0.27 (0.07)	-0.26 (0.07)	-0.22 (0.06)	0.04 (0.05)	-0.34 (0.05)
-0.07 (0.10)	0.01 (0.08)	0.05 (0.08)	0.23 (0.08)	0.03 (0.08)	0.13 (0.08)
-0.72 (0.10)	-0.03 (0.13)	-1.12 (0.11)	0.10 (0.17)	-0.07 (0.09)	-0.36 (0.11)
0.17 (0.09)	0.12 (0.05)	0.41 (0.06)	0.18 (0.08)	0.21 (0.07)	-0.12 (0.07)
0.04 (0.09)	0.56 (0.13)	0.20 (0.12)	0.21 (0.10)	0.11 (0.08)	0.43 (0.10)
0.92 (0.15)	0.46 (0.10)	0.32 (0.09)	0.72 (0.08)	0.56 (0.07)	0.25 (0.07)
0.15 (0.10)	0.12 (0.10)	0.21 (0.08)	0.14 (0.07)	0.25 (0.08)	0.28 (0.09)
0.66 (0.10)	0.67 (0.11)	0.48 (0.11)	0.45 (0.09)	0.34 (0.07)	0.44 (0.06)
-0.01 (0.11)	0.71 (0.08)	0.51 (0.07)	0.44 (0.06)	0.34 (0.08)	0.52 (0.07)
-0.20 (0.09)	-0.19 (0.10)	0.34 (0.10)	0.09 (0.09)	0.16 (0.06)	0.04 (0.07)
1.64 (0.08)	1.34 (0.10)	1.82 (0.15)	1.18 (0.12)	1.06 (0.11)	1.27 (0.11)
0.79 (0.13)	0.89 (0.14)	1.03 (0.13)	0.50 (0.15)	0.89 (0.12)	1.38 (0.17)
NA	NA	NA	NA	0.73 (0.30)	0.57 (0.25)
0.33 (0.02)	0.30 (0.02)	0.33 (0.02)	0.31 (0.01)	0.35 (0.01)	0.27 (0.01)

For peer review only

SIMD 10
0.01 (0.05)
0.51 (0.05)
0.37 (0.07)
0.29 (0.05)
0.30 (0.04)
0.73 (0.08)
0.37 (0.06)
0.13 (0.13)
0.14 (0.04)
0.70 (0.06)
0.39 (0.04)
0.55 (0.06)
0.72 (0.05)
-0.37 (0.04)
0.56 (0.05)
-0.18 (0.07)
-0.04 (0.08)
-0.63 (0.10)
0.21 (0.06)
0.11 (0.10)
0.27 (0.09)
0.57 (0.09)
0.49 (0.09)
0.48 (0.06)
0.32 (0.08)
1.00 (0.10)
0.63 (0.13)
-0.00 (0.19)
0.31 (0.01)

For peer review only

ICD10	Unstratified	Male	Female	SIMD 1	SIMD 2	SIMD 3
J09-J18	0.45 (0.12)	0.47 (0.25)	0.45 (0.08)	0.26 (0.73)	0.43 (0.34)	0.25 (1.19)
R00-R09	0.60 (0.12)	0.52 (0.14)	0.67 (0.16)	0.62 (0.61)	0.67 (0.38)	0.68 (0.41)
C30-C39	0.39 (0.09)	0.33 (0.06)	0.52 (0.20)	0.48 (0.56)	0.31 (0.40)	0.47 (0.28)
R10-R19	0.53 (0.23)	0.43 (0.21)	0.59 (0.27)	0.92 (0.44)	0.51 (0.65)	0.42 (0.98)
R50-R69	0.25 (0.25)	0.36 (0.18)	0.39 (1.08)	0.67 (1.72)	0.79 (0.90)	0.80 (0.55)
C15-C26	0.60 (0.06)	0.55 (0.08)	0.65 (0.09)	0.66 (0.16)	0.64 (0.21)	0.59 (0.25)
I30-I52	0.44 (0.06)	0.55 (0.07)	0.27 (0.10)	0.70 (0.24)	0.43 (0.16)	0.16 (0.59)
C76-C80	0.62 (0.13)	0.44 (0.20)	0.72 (0.10)	0.86 (0.48)	0.26 (1.05)	0.73 (0.37)
Z40-Z54	0.89 (0.14)	0.93 (0.20)	0.82 (0.14)	0.94 (0.31)	0.87 (0.45)	0.93 (0.57)
I60-I69	0.49 (0.09)	0.54 (0.12)	0.45 (0.12)	0.67 (0.26)	0.45 (0.30)	0.18 (0.46)
K55-K63	0.92 (0.13)	0.97 (0.13)	0.84 (0.24)	0.93 (0.33)	0.87 (0.40)	0.96 (0.90)
J40-J47	0.48 (0.10)	0.38 (0.15)	0.56 (0.09)	0.12 (0.55)	0.56 (0.22)	0.41 (0.27)
I20-I25	0.38 (0.05)	0.46 (0.05)	0.26 (0.08)	0.41 (0.35)	0.43 (0.20)	0.21 (0.21)
N30-N39	0.89 (0.22)	0.76 (0.12)	0.89 (0.40)	0.01 (0.54)	0.65 (0.67)	0.87 (0.33)
K20-K31	0.29 (0.11)	0.22 (0.11)	0.35 (0.18)	0.15 (0.28)	0.25 (0.28)	0.03 (0.36)
J20-J22	0.45 (0.11)	0.53 (0.32)	0.41 (0.10)	0.94 (0.70)	0.62 (0.27)	0.25 (0.37)
S70-S79	0.14 (0.16)	0.50 (0.39)	0.34 (0.13)	0.92 (0.78)	0.32 (1.23)	0.40 (0.46)
A30-A49	0.13 (0.21)	0.05 (0.33)	0.20 (0.17)	0.48 (0.40)	0.38 (0.30)	0.51 (0.47)
T80-T88	0.77 (0.34)	0.86 (0.70)	0.75 (0.22)	0.96 (1.77)	0.38 (1.05)	0.44 (0.56)
N17-N19	0.83 (0.32)	0.61 (0.32)	0.96 (0.43)	0.38 (4.95)	0.59 (1.70)	0.78 (0.65)
I80-I89	0.50 (0.11)	0.64 (0.11)	0.25 (0.18)	0.62 (0.40)	0.18 (0.81)	0.28 (0.56)
K90-K93	0.83 (0.41)	0.91 (0.47)	0.51 (0.37)	0.72 (0.33)	0.49 (1.78)	0.75 (1.17)
I70-I79	0.17 (0.07)	0.26 (0.09)	0.04 (0.11)	0.43 (0.23)	0.19 (0.22)	0.15 (0.21)
K50-K52	0.25 (0.07)	0.28 (0.06)	0.22 (0.09)	0.32 (0.32)	0.40 (0.20)	0.28 (0.55)
S00-S09	0.08 (0.24)	0.41 (0.12)	0.74 (0.36)	0.80 (1.27)	0.87 (0.77)	0.26 (0.69)
C50-C50	0.85 (0.07)	NA	0.85 (0.07)	0.74 (0.39)	0.91 (0.17)	0.91 (0.21)
C60-C63	0.97 (0.14)	0.97 (0.14)	NA	0.89 (0.59)	0.89 (0.25)	0.85 (0.97)
G30-G32	0.12 (0.47)	0.11 (0.80)	0.22 (0.40)	NA	NA	0.69 (0.86)
TOTAL	0.59 (0.15)	0.54 (0.17)	0.65 (0.21)	0.68 (0.59)	0.59 (0.58)	0.48 (0.64)

For peer review only

SIMD 4	SIMD 5	SIMD 6	SIMD 7	SIMD 8	SIMD 9
0.10 (0.78)	0.53 (0.37)	0.67 (0.41)	0.53 (0.21)	0.48 (0.25)	0.35 (0.36)
0.72 (0.32)	0.21 (0.69)	0.63 (0.32)	0.48 (0.18)	0.62 (0.18)	0.48 (0.16)
0.45 (0.26)	0.24 (0.33)	0.47 (0.17)	0.26 (0.32)	0.41 (0.24)	0.49 (0.30)
0.60 (0.37)	0.17 (0.53)	0.53 (0.45)	0.57 (0.31)	0.57 (0.45)	0.47 (0.78)
0.59 (0.34)	0.61 (0.28)	0.48 (0.46)	0.66 (0.19)	0.59 (0.29)	0.11 (0.66)
0.63 (0.13)	0.63 (0.21)	0.53 (0.23)	0.47 (0.29)	0.51 (0.24)	0.56 (0.28)
0.63 (0.31)	0.55 (0.11)	0.53 (0.18)	0.55 (0.19)	0.31 (0.14)	0.13 (0.29)
0.57 (0.34)	0.59 (0.44)	0.74 (0.30)	0.73 (0.38)	0.68 (0.29)	0.59 (0.25)
0.94 (0.32)	0.97 (0.25)	0.86 (0.54)	0.91 (0.22)	0.93 (0.26)	0.68 (0.51)
0.21 (0.28)	0.38 (0.77)	0.57 (0.16)	0.50 (0.29)	0.43 (0.36)	0.64 (0.39)
0.99 (0.52)	0.89 (0.31)	0.72 (0.51)	0.80 (0.34)	0.78 (0.23)	0.61 (1.32)
0.54 (0.32)	0.31 (0.41)	0.25 (0.52)	0.45 (0.20)	0.42 (0.10)	0.61 (0.14)
0.50 (0.09)	0.36 (0.21)	0.41 (0.13)	0.34 (0.19)	0.46 (0.05)	0.28 (0.14)
0.82 (0.28)	0.91 (0.57)	0.99 (0.88)	0.93 (0.60)	0.90 (0.51)	0.99 (0.36)
0.50 (0.17)	0.14 (0.24)	0.24 (0.10)	0.06 (0.29)	0.33 (0.29)	0.37 (0.19)
0.70 (0.32)	0.59 (0.42)	0.68 (0.52)	0.46 (0.34)	0.54 (0.31)	0.47 (0.21)
0.61 (0.41)	0.50 (0.47)	0.42 (0.25)	0.72 (0.63)	0.42 (0.57)	0.02 (1.00)
0.40 (0.21)	0.51 (0.30)	0.54 (0.18)	0.55 (0.39)	0.46 (0.19)	0.21 (0.31)
0.81 (0.86)	0.75 (0.36)	0.92 (0.31)	0.89 (0.92)	0.79 (0.47)	0.00 (0.85)
0.12 (1.36)	0.89 (0.49)	0.78 (1.11)	0.84 (0.86)	0.89 (1.19)	0.89 (0.48)
0.70 (0.42)	0.45 (0.44)	0.26 (0.62)	0.66 (0.24)	0.52 (0.27)	0.21 (0.44)
0.86 (2.48)	0.59 (2.44)	0.76 (0.50)	0.78 (0.61)	0.95 (0.86)	0.88 (0.71)
0.38 (0.24)	0.39 (0.29)	0.08 (0.36)	0.01 (0.29)	0.11 (0.24)	0.14 (0.15)
0.48 (0.29)	0.49 (0.20)	0.29 (0.23)	0.20 (0.18)	0.08 (0.40)	0.45 (0.23)
0.67 (0.37)	0.67 (0.72)	0.53 (0.53)	0.03 (0.96)	0.05 (0.37)	0.28 (0.36)
0.89 (0.10)	0.87 (0.17)	0.87 (0.22)	0.82 (0.24)	0.79 (0.22)	0.80 (0.21)
0.79 (0.32)	0.96 (0.35)	0.88 (0.27)	0.80 (0.68)	0.89 (0.23)	0.95 (0.29)
NA	0.83 (0.49)	0.81 (1.07)	0.82 (0.52)	0.79 (0.80)	0.53 (0.57)
0.54 (0.42)	0.58 (0.45)	0.58 (0.41)	0.57 (0.41)	0.61 (0.32)	0.57 (0.44)

For peer review only

SIMD 10
0.51 (0.20)
0.61 (0.19)
0.42 (0.29)
0.58 (0.35)
0.77 (0.26)
0.64 (0.22)
0.19 (0.27)
0.28 (0.53)
0.68 (0.18)
0.57 (0.17)
0.87 (0.18)
0.57 (0.19)
0.33 (0.12)
0.98 (0.29)
0.51 (0.20)
0.17 (0.67)
0.55 (0.41)
0.35 (0.28)
0.97 (0.60)
0.93 (1.53)
0.42 (0.54)
0.92 (0.32)
0.12 (0.31)
0.50 (0.25)
0.37 (0.31)
0.78 (0.21)
0.93 (0.36)
0.50 (0.19)
0.60 (0.38)

For peer review only

ICD10	Disease
J09-J18	Influenza and pneumonia
R00-R09	Symptoms and signs involving the circulatory and respiratory systems
C30-C39	Malignant neoplasm of respiratory and intrathoracic organs
R10-R19	Symptoms and signs involving the digestive system and abdomen
R50-R69	General symptoms and signs
C15-C26	Malignant neoplasms of the digestive organs
I30-I52	Other forms of heart disease
C76-C80	Malignant neoplasms of ill defined, secondary and unspecified sites
Z40-Z54	Persons encountering health services for specific procedures and health care
I60-I69	Cerebrovascular diseases
K55-K63	Other diseases of intestines
J40-J47	Chronic lower respiratory diseases
I20-I25	Ischaemic heart diseases
N30-N39	Other diseases of the urinary system
K20-K31	Diseases of oesophagus, stomach and duodenum
J20-J22	Other acute lower respiratory infections
S70-S79	Injuries to the hip and thigh
A30-A49	Other bacterial diseases
T80-T88	Complications of surgical and medical care, not elsewhere classified
N17-N19	Renal failure
I80-I89	Diseases of veins, lymphatic vessels and lymph nodes, not elsewhere classified
K90-K93	Other diseases of the digestive system
I70-I79	Diseases of arteries, arterioles and capillaries
K50-K52	Non infective enteritis and colitis
S00-S09	Injuries to the head
R25-R29	Symptoms and signs involving the nervous and musculoskeletal systems
K70-K77	Diseases of liver
C50-C50	Malignant neoplasm of breast
R30-R39	Symptoms and signs involving the urinary system
D60-D64	Aplastic and other anaemias
K80-K87	Diseases of gallbladder, biliary tract and pancreas
L00-L08	Infections of the skin and subcutaneous tissue
R40-R46	Symptoms and signs involving cognition, perception, emotional state and behaviour
E70-E90	Metabolic disorders
C81-C96	Malignant neoplasm of lymphoid, haematopoietic and related tissue
R90-R94	Abnormal findings on diagnostic imaging and in function studies, without diagnosis
J60-J70	Lung diseases due to external agents
G40-G47	Episodic and paroxysmal disorders
Z00-Z13	Persons encountering health services for examination and investigation
C51-C58	Malignant neoplasms of female genital organs
J90-J94	Other diseases of the pleura
I26-I28	Pulmonary heart disease and diseases of pulmonary circulation
T36-T50	Poisoning by, adverse effects of and underdosing of drugs, medicaments and biological substan
C64-C68	Malignant neoplasm of urinary tract
J95-J99	Other diseases of the respiratory system
M50-M54	Other dorsopathies
M70-M79	Other soft tissue disorders
F10-F19	Mental and behavioral disorders due to psychoactive substance abuse
A00-A09	Intestinal infectious diseases

For peer review only

C60-C63	Malignant neoplasms of male genital organs
D37-D48	Neoplasms of uncertain or unknown behaviour
F00-F09	Mental disorders due to known physiological conditions
D50-D53	Nutritional anaemias
E10-E14	Diabetes mellitus
L80-L99	Other disorders of the skin and subcutaneous tissue
S80-S89	Injuries to the knee and lower leg
S40-S49	Injuries to the shoulder and upper arm
K00-K14	Diseases of oral cavity and salivary glands
S30-S39	Injuries to abdomen, lower back, lumbar spine,pelvis and external genitals
M20-M25	Other joint disorders
M05-M14	Inflammatory polyarthropathies
I95-I99	Other and unspecified disorders of the circulatory system
D10-D36	Benign neoplasms
S50-S59	Injuries to the elbow and forearm
J30-J39	Other diseases of the upper respiratory tract
C43-C44	Melanoma and other malignant neoplasms of skin
S60-S69	Injuries to the wrist, hand and fingers
N20-N23	Urolithiasis
K40-K46	Hernia
N40-N51	Diseases of male genital organs
L60-L75	Disorders of skin appendages
N80-N98	Noninflammatory disorders of female genital tract
H00-H06	Disorders of the eyelid, lacrimal system and orbit
H25-H28	Disorders of the lens
M65-M68	Disorders of synovium and tendon
G50-G59	Nerve, nerve root and plexus disorders
M15-M19	Arthrosis
TOTAL	

For peer review only

Hospital Visits	Mortality (lnHR)	Relative Burden of Death	Weight
110,985	1.66	0.0939	0.0497
225,504	0.73	0.0841	0.0445
54,178	3.05	0.0840	0.0445
174,055	0.94	0.0831	0.0440
157,357	0.98	0.0786	0.0416
71,981	2.10	0.0767	0.0406
142,898	0.99	0.0720	0.0382
39,339	3.26	0.0653	0.0346
157,841	0.77	0.0614	0.0325
100,907	1.12	0.0573	0.0304
206,178	0.54	0.0569	0.0302
78,467	1.39	0.0553	0.0293
175,605	0.61	0.0542	0.0287
126,329	0.84	0.0538	0.0285
172,206	0.61	0.0530	0.0281
77,520	1.27	0.0501	0.0266
78,231	0.97	0.0386	0.0204
33,613	1.89	0.0323	0.0171
75,217	0.84	0.0322	0.0171
37,213	1.64	0.0310	0.0164
84,073	0.66	0.0283	0.0150
47,091	1.09	0.0262	0.0139
47,410	1.08	0.0261	0.0138
59,183	0.82	0.0247	0.0131
64,925	0.74	0.0243	0.0129
43,879	1.07	0.0238	0.0126
20,000	2.31	0.0235	0.0124
39,358	1.16	0.0233	0.0123
83,268	0.51	0.0217	0.0115
36,646	1.15	0.0215	0.0114
88,750	0.47	0.0211	0.0112
49,670	0.83	0.0210	0.0111
43,698	0.94	0.0209	0.0111
27,143	1.41	0.0195	0.0103
21,056	1.81	0.0193	0.0102
23,144	1.63	0.0192	0.0102
15,076	2.49	0.0191	0.0101
46,888	0.71	0.0169	0.0089
87,800	0.37	0.0164	0.0087
16,066	1.99	0.0162	0.0086
17,344	1.84	0.0162	0.0086
21,339	1.36	0.0147	0.0078
24,568	1.16	0.0145	0.0077
22,227	1.27	0.0144	0.0076
16,069	1.75	0.0143	0.0076
43,086	0.64	0.0141	0.0075
74,798	0.36	0.0136	0.0072
15,859	1.65	0.0133	0.0071
26,830	0.98	0.0133	0.0070

For peer review only

22,312	1.17	0.0133	0.0070
23,442	1.07	0.0127	0.0067
20,555	1.16	0.0122	0.0064
30,807	0.72	0.0113	0.0060
16,979	1.30	0.0112	0.0059
29,041	0.74	0.0109	0.0058
37,008	0.50	0.0094	0.0050
23,854	0.67	0.0081	0.0043
24,895	0.61	0.0077	0.0041
19,420	0.74	0.0073	0.0039
71,525	0.18	0.0065	0.0034
20,818	0.58	0.0061	0.0032
14,817	0.77	0.0058	0.0031
98,998	0.11	0.0058	0.0031
32,200	0.30	0.0050	0.0026
31,730	0.29	0.0046	0.0025
41,279	0.18	0.0037	0.0020
20,735	0.30	0.0032	0.0017
23,572	0.17	0.0020	0.0011
116,941	0.03	0.0020	0.0010
50,936	-0.01	-0.0003	-0.0002
24,006	-0.03	-0.0004	-0.0002
89,156	-0.01	-0.0007	-0.0004
22,633	-0.07	-0.0008	-0.0004
196,082	-0.02	-0.0022	-0.0012
15,091	-0.36	-0.0027	-0.0015
36,734	-0.22	-0.0040	-0.0021
112,390	-0.44	-0.0251	-0.0133
4,770,824	0.78	1.8874	1.0000

For peer review only

Description	Descriptive	Male			
		N	Mean	Median	SD
Unusual Postcode	Birth Date	6,189	1938.7	1941.2	17.07
	Dead Date	233	2004.9	2002.7	5.38
	SIMD	162	4.5	4	2.27
Transferred out of Scotland	Birth Date	268,506	1947.1	1951.8	15.65
	Dead Date	491	1998.7	1997.6	5.88
	SIMD	225,372	5.1	5	3.02
Alive in CHI but Dead in NRS	Birth Date	50	1940.7	1942.8	11.09
	Dead Date	50	2004.9	2005	8.79
	SIMD	47	5.7	5	3.18
Dead in CHI but no NRS record	Birth Date	38,657	1938.5	1937.7	11.67
	Dead Date	0	0.0	0	0.00
	SIMD	37,570	5.8	6	2.84
Birth Date before 1905	Birth Date	526	1903.1	1903.5	1.83
	Dead Date	526	2002.4	2002	1.99
	SIMD	514	4.8	5	2.65
Dead Date before 2001	Birth Date	11,141	1927.8	1925.9	12.40
	Dead Date	11,141	2000.7	2000.8	0.62
	SIMD	10,897	6.2	7	2.80
Post QC	Birth Date	930,952	1944.7	1946.7	13.34
	Dead Date	316,490	2008.2	2007.9	4.50
	SIMD	917,788	5.6	6	2.86

For peer review only

Female			
N	Mean	Median	SD
5,375	1936.3	1937.7	18.75
296	2004.3	2002.3	4.86
172	4.4	4	2.34
305,205	1946.4	1951.6	16.55
599	1997.8	1996.7	5.85
257,719	5.2	5	3.01
44	1935.8	1933.4	10.55
44	2005.5	2006.4	9.68
44	5.1	5	2.84
40,380	1934.6	1933.6	12.06
0	0.0	0	0.00
39,268	5.8	6	2.82
3,368	1902.9	1903.4	1.89
3,368	2002.7	2002.2	1.97
3,285	5.0	5	2.83
12,223	1922.4	1920.2	11.70
12,223	2000.7	2000.8	0.64
11,900	6.1	6	2.79
1,068,688	1942.4	1944.3	14.73
354,499	2008.2	2007.9	4.47
1,049,342	5.6	6	2.85

For peer review only

SIMD	Sex	Records	N Max	Events	Mean	SE	Median	95% CI
1	Female	100,383	40,153	24,811	83.27	0.06	85.17	(85.00 - 85.25)
2	Female	102,599	42,224	25,838	82.45	0.07	84.42	(84.33 - 84.59)
3	Female	102,821	39,962	29,961	81.67	0.07	83.66	(83.50 - 83.75)
4	Female	103,757	39,061	31,518	80.93	0.07	82.91	(82.75 - 83.00)
5	Female	105,156	38,529	33,927	80.21	0.07	82.25	(82.16 - 82.42)
6	Female	104,629	38,337	34,087	79.45	0.07	81.50	(81.34 - 81.66)
7	Female	109,393	38,245	38,311	78.73	0.08	80.75	(80.59 - 80.92)
8	Female	110,980	38,602	40,759	77.79	0.08	79.84	(79.75 - 80.00)
9	Female	108,027	37,650	41,626	76.59	0.08	78.58	(78.42 - 78.75)
10	Female	101,597	36,901	41,221	74.76	0.08	76.59	(76.42 - 76.75)
1	Male	86,838	37,598	21,157	80.27	0.11	82.17	(82.00 - 82.33)
2	Male	89,592	39,461	22,285	79.46	0.09	81.42	(81.25 - 81.59)
3	Male	89,210	37,738	25,213	78.51	0.08	80.50	(80.33 - 80.59)
4	Male	91,885	38,420	27,322	77.67	0.10	79.67	(79.50 - 79.83)
5	Male	92,520	37,718	29,886	76.72	0.08	78.67	(78.50 - 78.83)
6	Male	91,619	36,850	31,135	75.74	0.08	77.67	(77.50 - 77.83)
7	Male	94,206	36,950	34,148	74.77	0.09	76.58	(76.42 - 76.75)
8	Male	96,038	37,801	36,772	73.50	0.09	75.17	(75.00 - 75.33)
9	Male	94,456	37,026	38,175	72.09	0.09	73.75	(73.58 - 73.92)
10	Male	91,424	37,172	39,600	69.57	0.12	71.08	(70.84 - 71.25)

For peer review only

block	strat_level	birth_decac	burden	severity	se_severity	variable	beta	se
ACT-DTH	-1	1915	NA	NA	NA	incidence	-0.068	########
ACT-DTH	-1	1915	NA	NA	NA	impact	-0.068	########
ACT-DTH	-1	1925	NA	NA	NA	incidence	0.124	########
ACT-DTH	-1	1925	NA	NA	NA	impact	0.124	########
ACT-DTH	-1	1935	NA	NA	NA	incidence	0.274	########
ACT-DTH	-1	1935	NA	NA	NA	impact	0.274	########
ACT-DTH	-1	1945	NA	NA	NA	incidence	0.323	########
ACT-DTH	-1	1945	NA	NA	NA	impact	0.323	########
ACT-DTH	-1	1955	NA	NA	NA	incidence	0.299	0.00997
ACT-DTH	-1	1955	NA	NA	NA	impact	0.299	0.00997
EST-DTH	-1	1915	1.3825	########	########	incidence	########	########
EST-DTH	-1	1915	1.3825	########	########	impact	########	########
EST-DTH	-1	1925	1.3825	########	########	incidence	########	########
EST-DTH	-1	1925	1.3825	########	########	impact	########	########
EST-DTH	-1	1935	1.3825	########	########	incidence	########	########
EST-DTH	-1	1935	1.3825	########	########	impact	########	########
EST-DTH	-1	1945	1.3825	########	########	incidence	########	########
EST-DTH	-1	1945	1.3825	########	########	impact	########	########
EST-DTH	-1	1955	1.3825	########	########	incidence	########	########
EST-DTH	-1	1955	1.3825	########	########	impact	########	########
J09-J18	-1	1915	0.09388	0.1548	0.0359	incidence	-0.20744	########
J09-J18	-1	1915	0.09388	0.1548	0.0359	impact	-0.05264	########
J09-J18	-1	1925	0.09388	0.1548	0.0359	incidence	-0.1417	########
J09-J18	-1	1925	0.09388	0.1548	0.0359	impact	0.0131	########
J09-J18	-1	1935	0.09388	0.1548	0.0359	incidence	-0.1213	########
J09-J18	-1	1935	0.09388	0.1548	0.0359	impact	0.0335	########
J09-J18	-1	1945	0.09388	0.1548	0.0359	incidence	-0.1787	########
J09-J18	-1	1945	0.09388	0.1548	0.0359	impact	-0.0239	########
J09-J18	-1	1955	0.09388	0.1548	0.0359	incidence	-0.2161	0.01972
J09-J18	-1	1955	0.09388	0.1548	0.0359	impact	-0.0613	########
R00-R09	-1	1915	0.08403	0.2337	0.0402	incidence	-0.051	########
R00-R09	-1	1915	0.08403	0.2337	0.0402	impact	0.1827	########
R00-R09	-1	1925	0.08403	0.2337	0.0402	incidence	0.079	########
R00-R09	-1	1925	0.08403	0.2337	0.0402	impact	0.3127	########
R00-R09	-1	1935	0.08403	0.2337	0.0402	incidence	0.19984	########
R00-R09	-1	1935	0.08403	0.2337	0.0402	impact	0.43354	########
R00-R09	-1	1945	0.08403	0.2337	0.0402	incidence	0.25636	########
R00-R09	-1	1945	0.08403	0.2337	0.0402	impact	0.49006	########
R00-R09	-1	1955	0.08403	0.2337	0.0402	incidence	0.1418	0.0087
R00-R09	-1	1955	0.08403	0.2337	0.0402	impact	0.3755	########
C30-C39	-1	1915	0.084	0.2096	0.0466	incidence	-0.26062	########
C30-C39	-1	1915	0.084	0.2096	0.0466	impact	-0.05102	########
C30-C39	-1	1925	0.084	0.2096	0.0466	incidence	0.1066	########
C30-C39	-1	1925	0.084	0.2096	0.0466	impact	0.3162	########
C30-C39	-1	1935	0.084	0.2096	0.0466	incidence	0.2727	########
C30-C39	-1	1935	0.084	0.2096	0.0466	impact	0.4823	########
C30-C39	-1	1945	0.084	0.2096	0.0466	incidence	0.3708	########
C30-C39	-1	1945	0.084	0.2096	0.0466	impact	0.5804	########
C30-C39	-1	1955	0.084	0.2096	0.0466	incidence	0.4629	0.0278
C30-C39	-1	1955	0.084	0.2096	0.0466	impact	0.6725	########
R10-R19	-1	1915	0.08308	0.1173	0.0444	incidence	0.15981	########
R10-R19	-1	1915	0.08308	0.1173	0.0444	impact	0.27711	########
R10-R19	-1	1925	0.08308	0.1173	0.0444	incidence	0.17	########
R10-R19	-1	1925	0.08308	0.1173	0.0444	impact	0.2873	########
R10-R19	-1	1935	0.08308	0.1173	0.0444	incidence	0.1744	########

R10-R19	-1	1935	0.08308	0.1173	0.0444	impact	0.2917	########
R10-R19	-1	1945	0.08308	0.1173	0.0444	incidence	0.1442	########
R10-R19	-1	1945	0.08308	0.1173	0.0444	impact	0.2615	########
R10-R19	-1	1955	0.08308	0.1173	0.0444	incidence	0.0644	0.00982
R10-R19	-1	1955	0.08308	0.1173	0.0444	impact	0.1817	########
R50-R69	-1	1915	0.07855	0.0259	0.0252	incidence	0.00907	########
R50-R69	-1	1915	0.07855	0.0259	0.0252	impact	0.03497	########
R50-R69	-1	1925	0.07855	0.0259	0.0252	incidence	0.1971	########
R50-R69	-1	1925	0.07855	0.0259	0.0252	impact	0.223	########
R50-R69	-1	1935	0.07855	0.0259	0.0252	incidence	0.1963	########
R50-R69	-1	1935	0.07855	0.0259	0.0252	impact	0.2222	########
R50-R69	-1	1945	0.07855	0.0259	0.0252	incidence	0.131	########
R50-R69	-1	1945	0.07855	0.0259	0.0252	impact	0.1569	########
R50-R69	-1	1955	0.07855	0.0259	0.0252	incidence	0.0154	0.01333
R50-R69	-1	1955	0.07855	0.0259	0.0252	impact	0.0413	########
C15-C26	-1	1915	0.07666	0.3954	0.036	incidence	0.093	########
C15-C26	-1	1915	0.07666	0.3954	0.036	impact	0.4884	########
C15-C26	-1	1925	0.07666	0.3954	0.036	incidence	0.196	########
C15-C26	-1	1925	0.07666	0.3954	0.036	impact	0.5914	########
C15-C26	-1	1935	0.07666	0.3954	0.036	incidence	0.26561	########
C15-C26	-1	1935	0.07666	0.3954	0.036	impact	0.66101	########
C15-C26	-1	1945	0.07666	0.3954	0.036	incidence	0.33219	########
C15-C26	-1	1945	0.07666	0.3954	0.036	impact	0.72759	########
C15-C26	-1	1955	0.07666	0.3954	0.036	incidence	0.2882	0.02297
C15-C26	-1	1955	0.07666	0.3954	0.036	impact	0.6836	########
I30-I52	-1	1915	0.07206	0.1802	0.0209	incidence	0.126	########
I30-I52	-1	1915	0.07206	0.1802	0.0209	impact	0.3062	########
I30-I52	-1	1925	0.07206	0.1802	0.0209	incidence	0.276	########
I30-I52	-1	1925	0.07206	0.1802	0.0209	impact	0.4562	########
I30-I52	-1	1935	0.07206	0.1802	0.0209	incidence	0.33362	########
I30-I52	-1	1935	0.07206	0.1802	0.0209	impact	0.51382	########
I30-I52	-1	1945	0.07206	0.1802	0.0209	incidence	0.30278	########
I30-I52	-1	1945	0.07206	0.1802	0.0209	impact	0.48298	########
I30-I52	-1	1955	0.07206	0.1802	0.0209	incidence	0.1746	0.01735
I30-I52	-1	1955	0.07206	0.1802	0.0209	impact	0.3548	########
C76-C80	-1	1915	0.06525	0.3658	0.0653	incidence	0.03395	########
C76-C80	-1	1915	0.06525	0.3658	0.0653	impact	0.39975	########
C76-C80	-1	1925	0.06525	0.3658	0.0653	incidence	0.1104	########
C76-C80	-1	1925	0.06525	0.3658	0.0653	impact	0.4762	########
C76-C80	-1	1935	0.06525	0.3658	0.0653	incidence	0.2055	########
C76-C80	-1	1935	0.06525	0.3658	0.0653	impact	0.5713	########
C76-C80	-1	1945	0.06525	0.3658	0.0653	incidence	0.2557	########
C76-C80	-1	1945	0.06525	0.3658	0.0653	impact	0.6215	########
C76-C80	-1	1955	0.06525	0.3658	0.0653	incidence	0.2683	0.02766
C76-C80	-1	1955	0.06525	0.3658	0.0653	impact	0.6341	########
Z40-Z54	-1	1915	0.06138	0.2963	0.0353	incidence	0.11957	########
Z40-Z54	-1	1915	0.06138	0.2963	0.0353	impact	0.41587	########
Z40-Z54	-1	1925	0.06138	0.2963	0.0353	incidence	-5.00E-04	########
Z40-Z54	-1	1925	0.06138	0.2963	0.0353	impact	0.2958	########
Z40-Z54	-1	1935	0.06138	0.2963	0.0353	incidence	-0.0261	########
Z40-Z54	-1	1935	0.06138	0.2963	0.0353	impact	0.2702	########
Z40-Z54	-1	1945	0.06138	0.2963	0.0353	incidence	-0.0263	########
Z40-Z54	-1	1945	0.06138	0.2963	0.0353	impact	0.27	########
Z40-Z54	-1	1955	0.06138	0.2963	0.0353	incidence	-0.0625	0.01091
Z40-Z54	-1	1955	0.06138	0.2963	0.0353	impact	0.2338	########
I60-I69	-1	1915	0.05735	0.2033	0.0338	incidence	0.1062	########

For peer review only

I60-I69	-1	1915	0.05735	0.2033	0.0338	impact	0.3095	########
I60-I69	-1	1925	0.05735	0.2033	0.0338	incidence	0.3056	########
I60-I69	-1	1925	0.05735	0.2033	0.0338	impact	0.5089	########
I60-I69	-1	1935	0.05735	0.2033	0.0338	incidence	0.3354	########
I60-I69	-1	1935	0.05735	0.2033	0.0338	impact	0.5387	########
I60-I69	-1	1945	0.05735	0.2033	0.0338	incidence	0.2974	########
I60-I69	-1	1945	0.05735	0.2033	0.0338	impact	0.5007	########
I60-I69	-1	1955	0.05735	0.2033	0.0338	incidence	0.1283	0.0206
I60-I69	-1	1955	0.05735	0.2033	0.0338	impact	0.3316	########
K55-K63	-1	1915	0.05691	0.2234	0.0224	incidence	0.132	########
K55-K63	-1	1915	0.05691	0.2234	0.0224	impact	0.3554	########
K55-K63	-1	1925	0.05691	0.2234	0.0224	incidence	0.111	########
K55-K63	-1	1925	0.05691	0.2234	0.0224	impact	0.3344	########
K55-K63	-1	1935	0.05691	0.2234	0.0224	incidence	0.03424	########
K55-K63	-1	1935	0.05691	0.2234	0.0224	impact	0.25764	########
K55-K63	-1	1945	0.05691	0.2234	0.0224	incidence	0.01546	########
K55-K63	-1	1945	0.05691	0.2234	0.0224	impact	0.23886	########
K55-K63	-1	1955	0.05691	0.2234	0.0224	incidence	-0.0067	0.00974
K55-K63	-1	1955	0.05691	0.2234	0.0224	impact	0.2167	########
J40-J47	-1	1915	0.05525	0.238	0.0465	incidence	0.061	########
J40-J47	-1	1915	0.05525	0.238	0.0465	impact	0.299	########
J40-J47	-1	1925	0.05525	0.238	0.0465	incidence	0.236	########
J40-J47	-1	1925	0.05525	0.238	0.0465	impact	0.474	########
J40-J47	-1	1935	0.05525	0.238	0.0465	incidence	0.37465	########
J40-J47	-1	1935	0.05525	0.238	0.0465	impact	0.61265	########
J40-J47	-1	1945	0.05525	0.238	0.0465	incidence	0.32565	########
J40-J47	-1	1945	0.05525	0.238	0.0465	impact	0.56365	########
J40-J47	-1	1955	0.05525	0.238	0.0465	incidence	0.2317	0.02062
J40-J47	-1	1955	0.05525	0.238	0.0465	impact	0.4697	########
I20-I25	-1	1915	0.05419	0.2639	0.0342	incidence	0.283	########
I20-I25	-1	1915	0.05419	0.2639	0.0342	impact	0.5469	########
I20-I25	-1	1925	0.05419	0.2639	0.0342	incidence	0.437	########
I20-I25	-1	1925	0.05419	0.2639	0.0342	impact	0.7009	########
I20-I25	-1	1935	0.05419	0.2639	0.0342	incidence	0.49165	########
I20-I25	-1	1935	0.05419	0.2639	0.0342	impact	0.75555	########
I20-I25	-1	1945	0.05419	0.2639	0.0342	incidence	0.52295	########
I20-I25	-1	1945	0.05419	0.2639	0.0342	impact	0.78685	########
I20-I25	-1	1955	0.05419	0.2639	0.0342	incidence	0.3884	0.01194
I20-I25	-1	1955	0.05419	0.2639	0.0342	impact	0.6523	########
N30-N39	-1	1915	0.05382	-0.2148	0.0395	incidence	-0.27757	########
N30-N39	-1	1915	0.05382	-0.2148	0.0395	impact	-0.49237	########
N30-N39	-1	1925	0.05382	-0.2148	0.0395	incidence	-0.0859	########
N30-N39	-1	1925	0.05382	-0.2148	0.0395	impact	-0.3007	########
N30-N39	-1	1935	0.05382	-0.2148	0.0395	incidence	0.0024	0.0292
N30-N39	-1	1935	0.05382	-0.2148	0.0395	impact	-0.2124	########
N30-N39	-1	1945	0.05382	-0.2148	0.0395	incidence	0.0832	########
N30-N39	-1	1945	0.05382	-0.2148	0.0395	impact	-0.1316	########
N30-N39	-1	1955	0.05382	-0.2148	0.0395	incidence	0.0762	0.01474
N30-N39	-1	1955	0.05382	-0.2148	0.0395	impact	-0.1386	########
K20-K31	-1	1915	0.05296	0.1335	0.0491	incidence	0.30913	########
K20-K31	-1	1915	0.05296	0.1335	0.0491	impact	0.44263	########
K20-K31	-1	1925	0.05296	0.1335	0.0491	incidence	0.3116	########
K20-K31	-1	1925	0.05296	0.1335	0.0491	impact	0.4451	########
K20-K31	-1	1935	0.05296	0.1335	0.0491	incidence	0.3308	########
K20-K31	-1	1935	0.05296	0.1335	0.0491	impact	0.4643	########
K20-K31	-1	1945	0.05296	0.1335	0.0491	incidence	0.3963	########

For peer review only

K20-K31	-1	1945	0.05296	0.1335	0.0491	impact	0.5298	########
K20-K31	-1	1955	0.05296	0.1335	0.0491	incidence	0.304	0.01011
K20-K31	-1	1955	0.05296	0.1335	0.0491	impact	0.4375	########
J20-J22	-1	1915	0.05013	-0.1073	0.0242	incidence	-0.22614	########
J20-J22	-1	1915	0.05013	-0.1073	0.0242	impact	-0.33344	########
J20-J22	-1	1925	0.05013	-0.1073	0.0242	incidence	-0.0604	########
J20-J22	-1	1925	0.05013	-0.1073	0.0242	impact	-0.1677	########
J20-J22	-1	1935	0.05013	-0.1073	0.0242	incidence	0.0052	########
J20-J22	-1	1935	0.05013	-0.1073	0.0242	impact	-0.1021	########
J20-J22	-1	1945	0.05013	-0.1073	0.0242	incidence	-0.0634	########
J20-J22	-1	1945	0.05013	-0.1073	0.0242	impact	-0.1707	########
J20-J22	-1	1955	0.05013	-0.1073	0.0242	incidence	-0.1954	0.02179
J20-J22	-1	1955	0.05013	-0.1073	0.0242	impact	-0.3027	########
S70-S79	-1	1915	0.03858	-0.0199	0.0211	incidence	0.05001	########
S70-S79	-1	1915	0.03858	-0.0199	0.0211	impact	0.03011	########
S70-S79	-1	1925	0.03858	-0.0199	0.0211	incidence	0.2069	########
S70-S79	-1	1925	0.03858	-0.0199	0.0211	impact	0.187	########
S70-S79	-1	1935	0.03858	-0.0199	0.0211	incidence	0.2531	########
S70-S79	-1	1935	0.03858	-0.0199	0.0211	impact	0.2332	########
S70-S79	-1	1945	0.03858	-0.0199	0.0211	incidence	0.1413	########
S70-S79	-1	1945	0.03858	-0.0199	0.0211	impact	0.1214	########
S70-S79	-1	1955	0.03858	-0.0199	0.0211	incidence	0.0546	0.03333
S70-S79	-1	1955	0.03858	-0.0199	0.0211	impact	0.0347	########
A30-A49	-1	1915	0.03224	0.0633	0.1027	incidence	-0.5186	########
A30-A49	-1	1915	0.03224	0.0633	0.1027	impact	-0.4553	########
A30-A49	-1	1925	0.03224	0.0633	0.1027	incidence	-0.4419	########
A30-A49	-1	1925	0.03224	0.0633	0.1027	impact	-0.3786	########
A30-A49	-1	1935	0.03224	0.0633	0.1027	incidence	-0.3852	########
A30-A49	-1	1935	0.03224	0.0633	0.1027	impact	-0.3219	########
A30-A49	-1	1945	0.03224	0.0633	0.1027	incidence	-0.4369	########
A30-A49	-1	1945	0.03224	0.0633	0.1027	impact	-0.3736	########
A30-A49	-1	1955	0.03224	0.0633	0.1027	incidence	-0.4306	0.0319
A30-A49	-1	1955	0.03224	0.0633	0.1027	impact	-0.3673	########
T80-T88	-1	1915	0.03223	0.1835	0.0631	incidence	-0.0659	########
T80-T88	-1	1915	0.03223	0.1835	0.0631	impact	0.1176	########
T80-T88	-1	1925	0.03223	0.1835	0.0631	incidence	-0.039	########
T80-T88	-1	1925	0.03223	0.1835	0.0631	impact	0.1445	########
T80-T88	-1	1935	0.03223	0.1835	0.0631	incidence	0.0098	########
T80-T88	-1	1935	0.03223	0.1835	0.0631	impact	0.1933	########
T80-T88	-1	1945	0.03223	0.1835	0.0631	incidence	0.0174	########
T80-T88	-1	1945	0.03223	0.1835	0.0631	impact	0.2009	########
T80-T88	-1	1955	0.03223	0.1835	0.0631	incidence	-0.0975	0.0165
T80-T88	-1	1955	0.03223	0.1835	0.0631	impact	0.086	########
N17-N19	-1	1915	0.03099	0.2032	0.0578	incidence	-0.3314	########
N17-N19	-1	1915	0.03099	0.2032	0.0578	impact	-0.1282	########
N17-N19	-1	1925	0.03099	0.2032	0.0578	incidence	-0.0903	########
N17-N19	-1	1925	0.03099	0.2032	0.0578	impact	0.1129	########
N17-N19	-1	1935	0.03099	0.2032	0.0578	incidence	0.1154	########
N17-N19	-1	1935	0.03099	0.2032	0.0578	impact	0.3186	########
N17-N19	-1	1945	0.03099	0.2032	0.0578	incidence	0.0823	########
N17-N19	-1	1945	0.03099	0.2032	0.0578	impact	0.2855	########
N17-N19	-1	1955	0.03099	0.2032	0.0578	incidence	0.1126	0.0346
N17-N19	-1	1955	0.03099	0.2032	0.0578	impact	0.3158	########
I80-I89	-1	1915	0.02834	0.2381	0.0471	incidence	0.25092	########
I80-I89	-1	1915	0.02834	0.2381	0.0471	impact	0.48902	########
I80-I89	-1	1925	0.02834	0.2381	0.0471	incidence	0.1729	########

For peer review only

I80-I89	-1	1925	0.02834	0.2381	0.0471	impact	0.411	########
I80-I89	-1	1935	0.02834	0.2381	0.0471	incidence	0.1818	########
I80-I89	-1	1935	0.02834	0.2381	0.0471	impact	0.4199	########
I80-I89	-1	1945	0.02834	0.2381	0.0471	incidence	0.2675	########
I80-I89	-1	1945	0.02834	0.2381	0.0471	impact	0.5056	########
I80-I89	-1	1955	0.02834	0.2381	0.0471	incidence	0.2474	0.01273
I80-I89	-1	1955	0.02834	0.2381	0.0471	impact	0.4855	########
K90-K93	-1	1915	0.02617	0.1852	0.0703	incidence	0.0801	########
K90-K93	-1	1915	0.02617	0.1852	0.0703	impact	0.2653	########
K90-K93	-1	1925	0.02617	0.1852	0.0703	incidence	0.1164	########
K90-K93	-1	1925	0.02617	0.1852	0.0703	impact	0.3016	########
K90-K93	-1	1935	0.02617	0.1852	0.0703	incidence	0.0173	########
K90-K93	-1	1935	0.02617	0.1852	0.0703	impact	0.2025	########
K90-K93	-1	1945	0.02617	0.1852	0.0703	incidence	-0.0215	########
K90-K93	-1	1945	0.02617	0.1852	0.0703	impact	0.1637	########
K90-K93	-1	1955	0.02617	0.1852	0.0703	incidence	-0.0991	0.0212
K90-K93	-1	1955	0.02617	0.1852	0.0703	impact	0.0861	########
I70-I79	-1	1915	0.02608	0.091	0.0388	incidence	0.15853	########
I70-I79	-1	1915	0.02608	0.091	0.0388	impact	0.24953	########
I70-I79	-1	1925	0.02608	0.091	0.0388	incidence	0.3895	########
I70-I79	-1	1925	0.02608	0.091	0.0388	impact	0.4805	########
I70-I79	-1	1935	0.02608	0.091	0.0388	incidence	0.5012	########
I70-I79	-1	1935	0.02608	0.091	0.0388	impact	0.5922	########
I70-I79	-1	1945	0.02608	0.091	0.0388	incidence	0.4928	########
I70-I79	-1	1945	0.02608	0.091	0.0388	impact	0.5838	########
I70-I79	-1	1955	0.02608	0.091	0.0388	incidence	0.46	0.02752
I70-I79	-1	1955	0.02608	0.091	0.0388	impact	0.551	########
K50-K52	-1	1915	0.02464	0.1161	0.0314	incidence	0.15185	########
K50-K52	-1	1915	0.02464	0.1161	0.0314	impact	0.26795	########
K50-K52	-1	1925	0.02464	0.1161	0.0314	incidence	0.2981	########
K50-K52	-1	1925	0.02464	0.1161	0.0314	impact	0.4142	########
K50-K52	-1	1935	0.02464	0.1161	0.0314	incidence	0.3709	########
K50-K52	-1	1935	0.02464	0.1161	0.0314	impact	0.487	########
K50-K52	-1	1945	0.02464	0.1161	0.0314	incidence	0.3769	########
K50-K52	-1	1945	0.02464	0.1161	0.0314	impact	0.493	########
K50-K52	-1	1955	0.02464	0.1161	0.0314	incidence	0.3444	0.01784
K50-K52	-1	1955	0.02464	0.1161	0.0314	impact	0.4605	########
S00-S09	-1	1915	0.02432	0.0155	0.0484	incidence	-0.1914	########
S00-S09	-1	1915	0.02432	0.0155	0.0484	impact	-0.1759	########
S00-S09	-1	1925	0.02432	0.0155	0.0484	incidence	-0.1319	########
S00-S09	-1	1925	0.02432	0.0155	0.0484	impact	-0.1164	########
S00-S09	-1	1935	0.02432	0.0155	0.0484	incidence	0.035	########
S00-S09	-1	1935	0.02432	0.0155	0.0484	impact	0.0505	########
S00-S09	-1	1945	0.02432	0.0155	0.0484	incidence	0.2775	########
S00-S09	-1	1945	0.02432	0.0155	0.0484	impact	0.293	########
S00-S09	-1	1955	0.02432	0.0155	0.0484	incidence	0.2837	0.0184
S00-S09	-1	1955	0.02432	0.0155	0.0484	impact	0.2992	########
C50-C50	-1	1915	0.02331	1.1606	0.0688	incidence	0.28644	########
C50-C50	-1	1915	0.02331	1.1606	0.0688	impact	1.44704	########
C50-C50	-1	1925	0.02331	1.1606	0.0688	incidence	0.2329	########
C50-C50	-1	1925	0.02331	1.1606	0.0688	impact	1.3935	########
C50-C50	-1	1935	0.02331	1.1606	0.0688	incidence	0.1266	########
C50-C50	-1	1935	0.02331	1.1606	0.0688	impact	1.2872	########
C50-C50	-1	1945	0.02331	1.1606	0.0688	incidence	0.1857	########
C50-C50	-1	1945	0.02331	1.1606	0.0688	impact	1.3463	########
C50-C50	-1	1955	0.02331	1.1606	0.0688	incidence	0.2174	0.01983

For peer review only

C50-C50	-1	1955	0.02331	1.1606	0.0688	impact	1.378	########
C60-C63	-1	1915	0.01328	0.6983	0.0669	incidence	0.2049	########
C60-C63	-1	1915	0.01328	0.6983	0.0669	impact	0.9032	########
C60-C63	-1	1925	0.01328	0.6983	0.0669	incidence	0.3433	########
C60-C63	-1	1925	0.01328	0.6983	0.0669	impact	1.0416	########
C60-C63	-1	1935	0.01328	0.6983	0.0669	incidence	0.108	########
C60-C63	-1	1935	0.01328	0.6983	0.0669	impact	0.8063	########
C60-C63	-1	1945	0.01328	0.6983	0.0669	incidence	-0.0828	########
C60-C63	-1	1945	0.01328	0.6983	0.0669	impact	0.6155	########
C60-C63	-1	1955	0.01328	0.6983	0.0669	incidence	-0.0995	0.0448
C60-C63	-1	1955	0.01328	0.6983	0.0669	impact	0.5988	########
G30-G32	-1	1915	0.00282	0.0375	0.1439	incidence	0.02977	########
G30-G32	-1	1915	0.00282	0.0375	0.1439	impact	0.06727	########
G30-G32	-1	1925	0.00282	0.0375	0.1439	incidence	0.2979	########
G30-G32	-1	1925	0.00282	0.0375	0.1439	impact	NA	NA
G30-G32	-1	1935	0.00282	0.0375	0.1439	incidence	0.54149	########
G30-G32	-1	1935	0.00282	0.0375	0.1439	impact	NA	NA
G30-G32	-1	1945	0.00282	0.0375	0.1439	incidence	0.42091	########
G30-G32	-1	1945	0.00282	0.0375	0.1439	impact	0.45841	########
G30-G32	-1	1955	0.00282	0.0375	0.1439	incidence	0.1798	0.11813
G30-G32	-1	1955	0.00282	0.0375	0.1439	impact	0.2173	########
ACT-DTH	1	1915	NA	NA	NA	incidence	-0.059	########
ACT-DTH	1	1915	NA	NA	NA	impact	-0.059	########
ACT-DTH	1	1925	NA	NA	NA	incidence	0.109	########
ACT-DTH	1	1925	NA	NA	NA	impact	0.109	########
ACT-DTH	1	1935	NA	NA	NA	incidence	0.274	########
ACT-DTH	1	1935	NA	NA	NA	impact	0.274	########
ACT-DTH	1	1945	NA	NA	NA	incidence	0.358	########
ACT-DTH	1	1945	NA	NA	NA	impact	0.358	########
ACT-DTH	1	1955	NA	NA	NA	incidence	0.339	0.013
ACT-DTH	1	1955	NA	NA	NA	impact	0.339	0.013
ACT-DTH	2	1915	NA	NA	NA	incidence	-0.076	########
ACT-DTH	2	1915	NA	NA	NA	impact	-0.076	########
ACT-DTH	2	1925	NA	NA	NA	incidence	0.13	########
ACT-DTH	2	1925	NA	NA	NA	impact	0.13	########
ACT-DTH	2	1935	NA	NA	NA	incidence	0.272	########
ACT-DTH	2	1935	NA	NA	NA	impact	0.272	########
ACT-DTH	2	1945	NA	NA	NA	incidence	0.273	########
ACT-DTH	2	1945	NA	NA	NA	impact	0.273	########
ACT-DTH	2	1955	NA	NA	NA	incidence	0.242	0.0156
ACT-DTH	2	1955	NA	NA	NA	impact	0.242	0.0156
EST-DTH	1	1915	1.425335	########	########	incidence	########	########
EST-DTH	1	1915	1.425335	########	########	impact	########	########
EST-DTH	1	1925	1.425335	########	########	incidence	########	########
EST-DTH	1	1925	1.425335	########	########	impact	########	########
EST-DTH	1	1935	1.425335	########	########	incidence	########	########
EST-DTH	1	1935	1.425335	########	########	impact	########	########
EST-DTH	1	1945	1.425335	########	########	incidence	########	########
EST-DTH	1	1945	1.425335	########	########	impact	########	########
EST-DTH	1	1955	1.425335	########	########	incidence	########	########
EST-DTH	1	1955	1.425335	########	########	impact	########	########
EST-DTH	2	1915	1.347046	########	########	incidence	########	########
EST-DTH	2	1915	1.347046	########	########	impact	########	########
EST-DTH	2	1925	1.347046	########	########	incidence	########	########
EST-DTH	2	1925	1.347046	########	########	impact	########	########
EST-DTH	2	1935	1.347046	########	########	incidence	########	########

For peer review only

EST-DTH	2	1935	1.347046	########	########	impact	########	########
EST-DTH	2	1945	1.347046	########	########	incidence	########	########
EST-DTH	2	1945	1.347046	########	########	impact	########	########
EST-DTH	2	1955	1.347046	########	########	incidence	########	########
EST-DTH	2	1955	1.347046	########	########	impact	########	########
J09-J18	1	1915	0.101	0.1144	0.0555	incidence	-0.1097	########
J09-J18	1	1915	0.101	0.1144	0.0555	impact	0.0047	########
J09-J18	1	1925	0.101	0.1144	0.0555	incidence	-0.12721	########
J09-J18	1	1925	0.101	0.1144	0.0555	impact	-0.01281	########
J09-J18	1	1935	0.101	0.1144	0.0555	incidence	-0.10449	########
J09-J18	1	1935	0.101	0.1144	0.0555	impact	0.00991	########
J09-J18	1	1945	0.101	0.1144	0.0555	incidence	-0.09908	########
J09-J18	1	1945	0.101	0.1144	0.0555	impact	0.01532	########
J09-J18	1	1955	0.101	0.1144	0.0555	incidence	-0.15792	0.0277
J09-J18	1	1955	0.101	0.1144	0.0555	impact	-0.04352	########
J09-J18	2	1915	0.0886	0.1945	0.032	incidence	-0.2518	########
J09-J18	2	1915	0.0886	0.1945	0.032	impact	-0.0573	########
J09-J18	2	1925	0.0886	0.1945	0.032	incidence	-0.15187	########
J09-J18	2	1925	0.0886	0.1945	0.032	impact	0.04263	########
J09-J18	2	1935	0.0886	0.1945	0.032	incidence	-0.14063	########
J09-J18	2	1935	0.0886	0.1945	0.032	impact	0.05387	########
J09-J18	2	1945	0.0886	0.1945	0.032	incidence	-0.27624	########
J09-J18	2	1945	0.0886	0.1945	0.032	impact	-0.08174	########
J09-J18	2	1955	0.0886	0.1945	0.032	incidence	-0.27376	0.0281
J09-J18	2	1955	0.0886	0.1945	0.032	impact	-0.07926	########
R00-R09	1	1915	0.0909	0.2189	0.0504	incidence	-0.0228	########
R00-R09	1	1915	0.0909	0.2189	0.0504	impact	0.1961	########
R00-R09	1	1925	0.0909	0.2189	0.0504	incidence	0.08933	########
R00-R09	1	1925	0.0909	0.2189	0.0504	impact	0.30823	########
R00-R09	1	1935	0.0909	0.2189	0.0504	incidence	0.22697	########
R00-R09	1	1935	0.0909	0.2189	0.0504	impact	0.44587	########
R00-R09	1	1945	0.0909	0.2189	0.0504	incidence	0.28377	########
R00-R09	1	1945	0.0909	0.2189	0.0504	impact	0.50267	########
R00-R09	1	1955	0.0909	0.2189	0.0504	incidence	0.19423	0.0122
R00-R09	1	1955	0.0909	0.2189	0.0504	impact	0.41313	########
R00-R09	2	1915	0.0782	0.2416	0.0473	incidence	-0.057	########
R00-R09	2	1915	0.0782	0.2416	0.0473	impact	0.1846	########
R00-R09	2	1925	0.0782	0.2416	0.0473	incidence	0.07366	########
R00-R09	2	1925	0.0782	0.2416	0.0473	impact	0.31526	########
R00-R09	2	1935	0.0782	0.2416	0.0473	incidence	0.17524	########
R00-R09	2	1935	0.0782	0.2416	0.0473	impact	0.41684	########
R00-R09	2	1945	0.0782	0.2416	0.0473	incidence	0.2264	########
R00-R09	2	1945	0.0782	0.2416	0.0473	impact	0.468	########
R00-R09	2	1955	0.0782	0.2416	0.0473	incidence	0.0886	0.0124
R00-R09	2	1955	0.0782	0.2416	0.0473	impact	0.3302	########
C30-C39	1	1915	0.0942	0.213	0.0389	incidence	-0.0538	########
C30-C39	1	1915	0.0942	0.213	0.0389	impact	0.1592	########
C30-C39	1	1925	0.0942	0.213	0.0389	incidence	0.18086	########
C30-C39	1	1925	0.0942	0.213	0.0389	impact	0.39386	########
C30-C39	1	1935	0.0942	0.213	0.0389	incidence	0.35554	########
C30-C39	1	1935	0.0942	0.213	0.0389	impact	0.56854	########
C30-C39	1	1945	0.0942	0.213	0.0389	incidence	0.46193	########
C30-C39	1	1945	0.0942	0.213	0.0389	impact	0.67493	########
C30-C39	1	1955	0.0942	0.213	0.0389	incidence	0.60807	0.0385
C30-C39	1	1955	0.0942	0.213	0.0389	impact	0.82107	########
C30-C39	2	1915	0.0756	0.2028	0.0706	incidence	-0.4167	########

For peer review only

C30-C39	2	1915	0.0756	0.2028	0.0706	impact	-0.2139	########
C30-C39	2	1925	0.0756	0.2028	0.0706	incidence	0.02981	########
C30-C39	2	1925	0.0756	0.2028	0.0706	impact	0.23261	########
C30-C39	2	1935	0.0756	0.2028	0.0706	incidence	0.17359	########
C30-C39	2	1935	0.0756	0.2028	0.0706	impact	0.37639	########
C30-C39	2	1945	0.0756	0.2028	0.0706	incidence	0.24838	########
C30-C39	2	1945	0.0756	0.2028	0.0706	impact	0.45118	########
C30-C39	2	1955	0.0756	0.2028	0.0706	incidence	0.29862	0.0403
C30-C39	2	1955	0.0756	0.2028	0.0706	impact	0.50142	########
R10-R19	1	1915	0.0746	0.103	0.0465	incidence	0.2061	########
R10-R19	1	1915	0.0746	0.103	0.0465	impact	0.3091	########
R10-R19	1	1925	0.0746	0.103	0.0465	incidence	0.16362	########
R10-R19	1	1925	0.0746	0.103	0.0465	impact	0.26662	########
R10-R19	1	1935	0.0746	0.103	0.0465	incidence	0.18888	########
R10-R19	1	1935	0.0746	0.103	0.0465	impact	0.29188	########
R10-R19	1	1945	0.0746	0.103	0.0465	incidence	0.17844	########
R10-R19	1	1945	0.0746	0.103	0.0465	impact	0.28144	########
R10-R19	1	1955	0.0746	0.103	0.0465	incidence	0.09856	0.0157
R10-R19	1	1955	0.0746	0.103	0.0465	impact	0.20156	########
R10-R19	2	1915	0.09	0.1253	0.05	incidence	0.1598	########
R10-R19	2	1915	0.09	0.1253	0.05	impact	0.2851	########
R10-R19	2	1925	0.09	0.1253	0.05	incidence	0.1815	########
R10-R19	2	1925	0.09	0.1253	0.05	impact	0.3068	########
R10-R19	2	1935	0.09	0.1253	0.05	incidence	0.1651	########
R10-R19	2	1935	0.09	0.1253	0.05	impact	0.2904	########
R10-R19	2	1945	0.09	0.1253	0.05	incidence	0.11918	########
R10-R19	2	1945	0.09	0.1253	0.05	impact	0.24448	########
R10-R19	2	1955	0.09	0.1253	0.05	incidence	0.04182	0.0126
R10-R19	2	1955	0.09	0.1253	0.05	impact	0.16712	########
R50-R69	1	1915	0.0829	0.0742	0.0334	incidence	0.0601	########
R50-R69	1	1915	0.0829	0.0742	0.0334	impact	0.1343	########
R50-R69	1	1925	0.0829	0.0742	0.0334	incidence	0.15465	########
R50-R69	1	1925	0.0829	0.0742	0.0334	impact	0.22885	########
R50-R69	1	1935	0.0829	0.0742	0.0334	incidence	0.19415	########
R50-R69	1	1935	0.0829	0.0742	0.0334	impact	0.26835	########
R50-R69	1	1945	0.0829	0.0742	0.0334	incidence	0.19373	########
R50-R69	1	1945	0.0829	0.0742	0.0334	impact	0.26793	########
R50-R69	1	1955	0.0829	0.0742	0.0334	incidence	0.09327	0.0189
R50-R69	1	1955	0.0829	0.0742	0.0334	impact	0.16747	########
R50-R69	2	1915	0.0748	-0.0166	0.0428	incidence	-0.0119	########
R50-R69	2	1915	0.0748	-0.0166	0.0428	impact	-0.0285	########
R50-R69	2	1925	0.0748	-0.0166	0.0428	incidence	0.22479	########
R50-R69	2	1925	0.0748	-0.0166	0.0428	impact	0.20819	########
R50-R69	2	1935	0.0748	-0.0166	0.0428	incidence	########	########
R50-R69	2	1935	0.0748	-0.0166	0.0428	impact	########	########
R50-R69	2	1945	0.0748	-0.0166	0.0428	incidence	########	########
R50-R69	2	1945	0.0748	-0.0166	0.0428	impact	########	########
R50-R69	2	1955	0.0748	-0.0166	0.0428	incidence	-0.06099	0.0188
R50-R69	2	1955	0.0748	-0.0166	0.0428	impact	-0.07759	########
C15-C26	1	1915	0.0913	0.366	0.0475	incidence	0.0876	########
C15-C26	1	1915	0.0913	0.366	0.0475	impact	0.4536	########
C15-C26	1	1925	0.0913	0.366	0.0475	incidence	0.17702	########
C15-C26	1	1925	0.0913	0.366	0.0475	impact	0.54302	########
C15-C26	1	1935	0.0913	0.366	0.0475	incidence	0.28948	########
C15-C26	1	1935	0.0913	0.366	0.0475	impact	0.65548	########
C15-C26	1	1945	0.0913	0.366	0.0475	incidence	0.35387	########

For peer review only

C15-C26	1	1945	0.0913	0.366	0.0475	impact	0.71987	########
C15-C26	1	1955	0.0913	0.366	0.0475	incidence	0.33013	0.03
C15-C26	1	1955	0.0913	0.366	0.0475	impact	0.69613	########
C15-C26	2	1915	0.064	0.4284	0.0499	incidence	0.0923	########
C15-C26	2	1915	0.064	0.4284	0.0499	impact	0.5207	########
C15-C26	2	1925	0.064	0.4284	0.0499	incidence	0.21209	########
C15-C26	2	1925	0.064	0.4284	0.0499	impact	0.64049	########
C15-C26	2	1935	0.064	0.4284	0.0499	incidence	0.23351	########
C15-C26	2	1935	0.064	0.4284	0.0499	impact	0.66191	########
C15-C26	2	1945	0.064	0.4284	0.0499	incidence	0.29841	########
C15-C26	2	1945	0.064	0.4284	0.0499	impact	0.72681	########
C15-C26	2	1955	0.064	0.4284	0.0499	incidence	0.22859	0.0359
C15-C26	2	1955	0.064	0.4284	0.0499	impact	0.65699	########
I30-I52	1	1915	0.078	0.2884	0.0332	incidence	0.09	########
I30-I52	1	1915	0.078	0.2884	0.0332	impact	0.3784	########
I30-I52	1	1925	0.078	0.2884	0.0332	incidence	0.24098	########
I30-I52	1	1925	0.078	0.2884	0.0332	impact	0.52938	########
I30-I52	1	1935	0.078	0.2884	0.0332	incidence	0.34122	########
I30-I52	1	1935	0.078	0.2884	0.0332	impact	0.62962	########
I30-I52	1	1945	0.078	0.2884	0.0332	incidence	0.31449	########
I30-I52	1	1945	0.078	0.2884	0.0332	impact	0.60289	########
I30-I52	1	1955	0.078	0.2884	0.0332	incidence	0.19151	0.0217
I30-I52	1	1955	0.078	0.2884	0.0332	impact	0.47991	########
I30-I52	2	1915	0.0671	0.0783	0.0275	incidence	0.1342	########
I30-I52	2	1915	0.0671	0.0783	0.0275	impact	0.2125	########
I30-I52	2	1925	0.0671	0.0783	0.0275	incidence	0.30216	########
I30-I52	2	1925	0.0671	0.0783	0.0275	impact	0.38046	########
I30-I52	2	1935	0.0671	0.0783	0.0275	incidence	0.32624	########
I30-I52	2	1935	0.0671	0.0783	0.0275	impact	0.40454	########
I30-I52	2	1945	0.0671	0.0783	0.0275	incidence	0.28752	########
I30-I52	2	1945	0.0671	0.0783	0.0275	impact	0.36582	########
I30-I52	2	1955	0.0671	0.0783	0.0275	incidence	0.14248	0.0288
I30-I52	2	1955	0.0671	0.0783	0.0275	impact	0.22078	########
C76-C80	1	1915	0.0626	0.2093	0.0866	incidence	0.0463	########
C76-C80	1	1915	0.0626	0.2093	0.0866	impact	0.2556	########
C76-C80	1	1925	0.0626	0.2093	0.0866	incidence	0.16267	########
C76-C80	1	1925	0.0626	0.2093	0.0866	impact	0.37197	########
C76-C80	1	1935	0.0626	0.2093	0.0866	incidence	0.20203	########
C76-C80	1	1935	0.0626	0.2093	0.0866	impact	0.41133	########
C76-C80	1	1945	0.0626	0.2093	0.0866	incidence	0.26424	########
C76-C80	1	1945	0.0626	0.2093	0.0866	impact	0.47354	########
C76-C80	1	1955	0.0626	0.2093	0.0866	incidence	0.35176	0.0435
C76-C80	1	1955	0.0626	0.2093	0.0866	impact	0.56106	########
C76-C80	2	1915	0.0679	0.5031	0.0541	incidence	0.0566	########
C76-C80	2	1915	0.0679	0.5031	0.0541	impact	0.5597	########
C76-C80	2	1925	0.0679	0.5031	0.0541	incidence	0.07494	########
C76-C80	2	1925	0.0679	0.5031	0.0541	impact	0.57804	########
C76-C80	2	1935	0.0679	0.5031	0.0541	incidence	0.20836	########
C76-C80	2	1935	0.0679	0.5031	0.0541	impact	0.71146	########
C76-C80	2	1945	0.0679	0.5031	0.0541	incidence	0.24605	########
C76-C80	2	1945	0.0679	0.5031	0.0541	impact	0.74915	########
C76-C80	2	1955	0.0679	0.5031	0.0541	incidence	0.21295	0.036
C76-C80	2	1955	0.0679	0.5031	0.0541	impact	0.71605	########
Z40-Z54	1	1915	0.0656	0.364	0.0574	incidence	-0.0204	########
Z40-Z54	1	1915	0.0656	0.364	0.0574	impact	0.3436	########
Z40-Z54	1	1925	0.0656	0.364	0.0574	incidence	0.0138	########

For peer review only

Z40-Z54	1	1925	0.0656	0.364	0.0574	impact	0.3778	########
Z40-Z54	1	1935	0.0656	0.364	0.0574	incidence	-0.0121	########
Z40-Z54	1	1935	0.0656	0.364	0.0574	impact	0.3519	########
Z40-Z54	1	1945	0.0656	0.364	0.0574	incidence	0.00148	########
Z40-Z54	1	1945	0.0656	0.364	0.0574	impact	0.36548	########
Z40-Z54	1	1955	0.0656	0.364	0.0574	incidence	-0.05008	0.0162
Z40-Z54	1	1955	0.0656	0.364	0.0574	impact	0.31392	########
Z40-Z54	2	1915	0.0583	0.2137	0.0266	incidence	0.2334	########
Z40-Z54	2	1915	0.0583	0.2137	0.0266	impact	0.4471	########
Z40-Z54	2	1925	0.0583	0.2137	0.0266	incidence	-0.00426	########
Z40-Z54	2	1925	0.0583	0.2137	0.0266	impact	0.20944	########
Z40-Z54	2	1935	0.0583	0.2137	0.0266	incidence	-0.04284	########
Z40-Z54	2	1935	0.0583	0.2137	0.0266	impact	0.17086	########
Z40-Z54	2	1945	0.0583	0.2137	0.0266	incidence	-0.05733	########
Z40-Z54	2	1945	0.0583	0.2137	0.0266	impact	0.15637	########
Z40-Z54	2	1955	0.0583	0.2137	0.0266	incidence	-0.07167	0.0148
Z40-Z54	2	1955	0.0583	0.2137	0.0266	impact	0.14203	########
I60-I69	1	1915	0.0534	0.2677	0.0509	incidence	0.1651	########
I60-I69	1	1915	0.0534	0.2677	0.0509	impact	0.4328	########
I60-I69	1	1925	0.0534	0.2677	0.0509	incidence	0.26358	########
I60-I69	1	1925	0.0534	0.2677	0.0509	impact	0.53128	########
I60-I69	1	1935	0.0534	0.2677	0.0509	incidence	0.32482	########
I60-I69	1	1935	0.0534	0.2677	0.0509	impact	0.59252	########
I60-I69	1	1945	0.0534	0.2677	0.0509	incidence	0.31811	########
I60-I69	1	1945	0.0534	0.2677	0.0509	impact	0.58581	########
I60-I69	1	1955	0.0534	0.2677	0.0509	incidence	0.15489	0.0274
I60-I69	1	1955	0.0534	0.2677	0.0509	impact	0.42259	########
I60-I69	2	1915	0.0605	0.151	0.0368	incidence	0.0712	########
I60-I69	2	1915	0.0605	0.151	0.0368	impact	0.2222	########
I60-I69	2	1925	0.0605	0.151	0.0368	incidence	0.33121	########
I60-I69	2	1925	0.0605	0.151	0.0368	impact	0.48221	########
I60-I69	2	1935	0.0605	0.151	0.0368	incidence	0.34719	########
I60-I69	2	1935	0.0605	0.151	0.0368	impact	0.49819	########
I60-I69	2	1945	0.0605	0.151	0.0368	incidence	0.2721	########
I60-I69	2	1945	0.0605	0.151	0.0368	impact	0.4231	########
I60-I69	2	1955	0.0605	0.151	0.0368	incidence	0.0939	0.0312
I60-I69	2	1955	0.0605	0.151	0.0368	impact	0.2449	########
K55-K63	1	1915	0.056	0.2964	0.0283	incidence	0.1428	########
K55-K63	1	1915	0.056	0.2964	0.0283	impact	0.4392	########
K55-K63	1	1925	0.056	0.2964	0.0283	incidence	0.09951	########
K55-K63	1	1925	0.056	0.2964	0.0283	impact	0.39591	########
K55-K63	1	1935	0.056	0.2964	0.0283	incidence	0.00349	########
K55-K63	1	1935	0.056	0.2964	0.0283	impact	0.29989	########
K55-K63	1	1945	0.056	0.2964	0.0283	incidence	-0.01481	########
K55-K63	1	1945	0.056	0.2964	0.0283	impact	0.28159	########
K55-K63	1	1955	0.056	0.2964	0.0283	incidence	-0.00509	0.0142
K55-K63	1	1955	0.056	0.2964	0.0283	impact	0.29131	########
K55-K63	2	1915	0.0578	0.1624	0.0349	incidence	0.1333	########
K55-K63	2	1915	0.0578	0.1624	0.0349	impact	0.2957	########
K55-K63	2	1925	0.0578	0.1624	0.0349	incidence	0.12058	########
K55-K63	2	1925	0.0578	0.1624	0.0349	impact	0.28298	########
K55-K63	2	1935	0.0578	0.1624	0.0349	incidence	0.05862	########
K55-K63	2	1935	0.0578	0.1624	0.0349	impact	0.22102	########
K55-K63	2	1945	0.0578	0.1624	0.0349	incidence	0.04294	########
K55-K63	2	1945	0.0578	0.1624	0.0349	impact	0.20534	########
K55-K63	2	1955	0.0578	0.1624	0.0349	incidence	-0.00794	0.0134

For peer review only

K55-K63	2	1955	0.0578	0.1624	0.0349	impact	0.15446	########
J40-J47	1	1915	0.0502	0.2043	0.0751	incidence	0.2205	########
J40-J47	1	1915	0.0502	0.2043	0.0751	impact	0.4248	########
J40-J47	1	1925	0.0502	0.2043	0.0751	incidence	0.30273	########
J40-J47	1	1925	0.0502	0.2043	0.0751	impact	0.50703	########
J40-J47	1	1935	0.0502	0.2043	0.0751	incidence	0.43187	########
J40-J47	1	1935	0.0502	0.2043	0.0751	impact	0.63617	########
J40-J47	1	1945	0.0502	0.2043	0.0751	incidence	0.39794	########
J40-J47	1	1945	0.0502	0.2043	0.0751	impact	0.60224	########
J40-J47	1	1955	0.0502	0.2043	0.0751	incidence	0.31406	0.0338
J40-J47	1	1955	0.0502	0.2043	0.0751	impact	0.51836	########
J40-J47	2	1915	0.0602	0.268	0.0353	incidence	-0.0041	########
J40-J47	2	1915	0.0602	0.268	0.0353	impact	0.2639	########
J40-J47	2	1925	0.0602	0.268	0.0353	incidence	0.19374	########
J40-J47	2	1925	0.0602	0.268	0.0353	impact	0.46174	########
J40-J47	2	1935	0.0602	0.268	0.0353	incidence	0.32766	########
J40-J47	2	1935	0.0602	0.268	0.0353	impact	0.59566	########
J40-J47	2	1945	0.0602	0.268	0.0353	incidence	0.26532	########
J40-J47	2	1945	0.0602	0.268	0.0353	impact	0.53332	########
J40-J47	2	1955	0.0602	0.268	0.0353	incidence	0.18468	0.0261
J40-J47	2	1955	0.0602	0.268	0.0353	impact	0.45268	########
I20-I25	1	1915	0.06	0.3489	0.034	incidence	0.2305	########
I20-I25	1	1915	0.06	0.3489	0.034	impact	0.5794	########
I20-I25	1	1925	0.06	0.3489	0.034	incidence	0.41256	########
I20-I25	1	1925	0.06	0.3489	0.034	impact	0.76146	########
I20-I25	1	1935	0.06	0.3489	0.034	incidence	0.47224	########
I20-I25	1	1935	0.06	0.3489	0.034	impact	0.82114	########
I20-I25	1	1945	0.06	0.3489	0.034	incidence	0.49211	########
I20-I25	1	1945	0.06	0.3489	0.034	impact	0.84101	########
I20-I25	1	1955	0.06	0.3489	0.034	incidence	0.36289	0.0142
I20-I25	1	1955	0.06	0.3489	0.034	impact	0.71179	########
I20-I25	2	1915	0.0475	0.1668	0.049	incidence	0.2925	########
I20-I25	2	1915	0.0475	0.1668	0.049	impact	0.4593	########
I20-I25	2	1925	0.0475	0.1668	0.049	incidence	0.4541	########
I20-I25	2	1925	0.0475	0.1668	0.049	impact	0.6209	########
I20-I25	2	1935	0.0475	0.1668	0.049	incidence	0.5249	########
I20-I25	2	1935	0.0475	0.1668	0.049	impact	0.6917	########
I20-I25	2	1945	0.0475	0.1668	0.049	incidence	0.58498	########
I20-I25	2	1945	0.0475	0.1668	0.049	impact	0.75178	########
I20-I25	2	1955	0.0475	0.1668	0.049	incidence	0.44502	0.022
I20-I25	2	1955	0.0475	0.1668	0.049	impact	0.61182	########
N30-N39	1	1915	0.0469	-0.2899	0.0369	incidence	-0.2618	########
N30-N39	1	1915	0.0469	-0.2899	0.0369	impact	-0.5517	########
N30-N39	1	1925	0.0469	-0.2899	0.0369	incidence	-0.07632	########
N30-N39	1	1925	0.0469	-0.2899	0.0369	impact	-0.36622	########
N30-N39	1	1935	0.0469	-0.2899	0.0369	incidence	0.06462	########
N30-N39	1	1935	0.0469	-0.2899	0.0369	impact	-0.22528	########
N30-N39	1	1945	0.0469	-0.2899	0.0369	incidence	0.14991	########
N30-N39	1	1945	0.0469	-0.2899	0.0369	impact	-0.13999	########
N30-N39	1	1955	0.0469	-0.2899	0.0369	incidence	0.17109	0.0242
N30-N39	1	1955	0.0469	-0.2899	0.0369	impact	-0.11881	########
N30-N39	2	1915	0.06	-0.1589	0.0528	incidence	-0.2739	########
N30-N39	2	1915	0.06	-0.1589	0.0528	impact	-0.4328	########
N30-N39	2	1925	0.06	-0.1589	0.0528	incidence	-0.08617	########
N30-N39	2	1925	0.06	-0.1589	0.0528	impact	-0.24507	########
N30-N39	2	1935	0.06	-0.1589	0.0528	incidence	-0.05313	########

For peer review only

N30-N39	2	1935	0.06	-0.1589	0.0528	impact	-0.21203	########
N30-N39	2	1945	0.06	-0.1589	0.0528	incidence	0.02599	########
N30-N39	2	1945	0.06	-0.1589	0.0528	impact	-0.13291	########
N30-N39	2	1955	0.06	-0.1589	0.0528	incidence	0.02101	0.0186
N30-N39	2	1955	0.06	-0.1589	0.0528	impact	-0.13789	########
K20-K31	1	1915	0.0595	0.1072	0.0529	incidence	0.3032	########
K20-K31	1	1915	0.0595	0.1072	0.0529	impact	0.4104	########
K20-K31	1	1925	0.0595	0.1072	0.0529	incidence	0.31114	########
K20-K31	1	1925	0.0595	0.1072	0.0529	impact	0.41834	########
K20-K31	1	1935	0.0595	0.1072	0.0529	incidence	0.35236	########
K20-K31	1	1935	0.0595	0.1072	0.0529	impact	0.45956	########
K20-K31	1	1945	0.0595	0.1072	0.0529	incidence	0.44923	########
K20-K31	1	1945	0.0595	0.1072	0.0529	impact	0.55643	########
K20-K31	1	1955	0.0595	0.1072	0.0529	incidence	0.36777	0.0147
K20-K31	1	1955	0.0595	0.1072	0.0529	impact	0.47497	########
K20-K31	2	1915	0.0466	0.1561	0.074	incidence	0.3316	########
K20-K31	2	1915	0.0466	0.1561	0.074	impact	0.4877	########
K20-K31	2	1925	0.0466	0.1561	0.074	incidence	0.31704	########
K20-K31	2	1925	0.0466	0.1561	0.074	impact	0.47314	########
K20-K31	2	1935	0.0466	0.1561	0.074	incidence	0.31066	########
K20-K31	2	1935	0.0466	0.1561	0.074	impact	0.46676	########
K20-K31	2	1945	0.0466	0.1561	0.074	incidence	0.34669	########
K20-K31	2	1945	0.0466	0.1561	0.074	impact	0.50279	########
K20-K31	2	1955	0.0466	0.1561	0.074	incidence	0.24631	0.014
K20-K31	2	1955	0.0466	0.1561	0.074	impact	0.40241	########
J20-J22	1	1915	0.0529	-0.0947	0.0489	incidence	-0.1956	########
J20-J22	1	1915	0.0529	-0.0947	0.0489	impact	-0.2903	########
J20-J22	1	1925	0.0529	-0.0947	0.0489	incidence	-0.03153	########
J20-J22	1	1925	0.0529	-0.0947	0.0489	impact	-0.12623	########
J20-J22	1	1935	0.0529	-0.0947	0.0489	incidence	0.03403	########
J20-J22	1	1935	0.0529	-0.0947	0.0489	impact	-0.06067	########
J20-J22	1	1945	0.0529	-0.0947	0.0489	incidence	-0.00971	########
J20-J22	1	1945	0.0529	-0.0947	0.0489	impact	-0.10441	########
J20-J22	1	1955	0.0529	-0.0947	0.0489	incidence	-0.14029	0.0318
J20-J22	1	1955	0.0529	-0.0947	0.0489	impact	-0.23499	########
J20-J22	2	1915	0.0481	-0.1198	0.0272	incidence	-0.2303	########
J20-J22	2	1915	0.0481	-0.1198	0.0272	impact	-0.3501	########
J20-J22	2	1925	0.0481	-0.1198	0.0272	incidence	-0.08025	########
J20-J22	2	1925	0.0481	-0.1198	0.0272	impact	-0.20005	########
J20-J22	2	1935	0.0481	-0.1198	0.0272	incidence	-0.02545	########
J20-J22	2	1935	0.0481	-0.1198	0.0272	impact	-0.14525	########
J20-J22	2	1945	0.0481	-0.1198	0.0272	incidence	-0.12188	########
J20-J22	2	1945	0.0481	-0.1198	0.0272	impact	-0.24168	########
J20-J22	2	1955	0.0481	-0.1198	0.0272	incidence	-0.24212	0.03
J20-J22	2	1955	0.0481	-0.1198	0.0272	impact	-0.36192	########
S70-S79	1	1915	0.0278	0.0925	0.0622	incidence	-0.0393	########
S70-S79	1	1915	0.0278	0.0925	0.0622	impact	0.0532	########
S70-S79	1	1925	0.0278	0.0925	0.0622	incidence	0.11241	########
S70-S79	1	1925	0.0278	0.0925	0.0622	impact	0.20491	########
S70-S79	1	1935	0.0278	0.0925	0.0622	incidence	0.20199	########
S70-S79	1	1935	0.0278	0.0925	0.0622	impact	0.29449	########
S70-S79	1	1945	0.0278	0.0925	0.0622	incidence	0.20731	########
S70-S79	1	1945	0.0278	0.0925	0.0622	impact	0.29981	########
S70-S79	1	1955	0.0278	0.0925	0.0622	incidence	0.03569	0.0464
S70-S79	1	1955	0.0278	0.0925	0.0622	impact	0.12819	########
S70-S79	2	1915	0.0474	-0.0664	0.0213	incidence	0.0683	########

For peer review only

S70-S79	2	1915	0.0474	-0.0664	0.0213	impact	0.0019	########
S70-S79	2	1925	0.0474	-0.0664	0.0213	incidence	0.23515	########
S70-S79	2	1925	0.0474	-0.0664	0.0213	impact	0.16875	########
S70-S79	2	1935	0.0474	-0.0664	0.0213	incidence	0.27575	########
S70-S79	2	1935	0.0474	-0.0664	0.0213	impact	0.20935	########
S70-S79	2	1945	0.0474	-0.0664	0.0213	incidence	0.09764	########
S70-S79	2	1945	0.0474	-0.0664	0.0213	impact	0.03124	########
S70-S79	2	1955	0.0474	-0.0664	0.0213	incidence	0.07036	0.0479
S70-S79	2	1955	0.0474	-0.0664	0.0213	impact	0.00396	########
A30-A49	1	1915	0.0318	0.02	0.1402	incidence	-0.5393	########
A30-A49	1	1915	0.0318	0.02	0.1402	impact	-0.5193	########
A30-A49	1	1925	0.0318	0.02	0.1402	incidence	-0.46406	########
A30-A49	1	1925	0.0318	0.02	0.1402	impact	-0.44406	########
A30-A49	1	1935	0.0318	0.02	0.1402	incidence	-0.42934	########
A30-A49	1	1935	0.0318	0.02	0.1402	impact	-0.40934	########
A30-A49	1	1945	0.0318	0.02	0.1402	incidence	-0.41551	########
A30-A49	1	1945	0.0318	0.02	0.1402	impact	-0.39551	########
A30-A49	1	1955	0.0318	0.02	0.1402	incidence	-0.35449	0.0468
A30-A49	1	1955	0.0318	0.02	0.1402	impact	-0.33449	########
A30-A49	2	1915	0.033	0.1166	0.097	incidence	-0.4889	########
A30-A49	2	1915	0.033	0.1166	0.097	impact	-0.3723	########
A30-A49	2	1925	0.033	0.1166	0.097	incidence	-0.41942	########
A30-A49	2	1925	0.033	0.1166	0.097	impact	-0.30282	########
A30-A49	2	1935	0.033	0.1166	0.097	incidence	-0.33818	########
A30-A49	2	1935	0.033	0.1166	0.097	impact	-0.22158	########
A30-A49	2	1945	0.033	0.1166	0.097	incidence	-0.46189	########
A30-A49	2	1945	0.033	0.1166	0.097	impact	-0.34529	########
A30-A49	2	1955	0.033	0.1166	0.097	incidence	-0.49511	0.0438
A30-A49	2	1955	0.033	0.1166	0.097	impact	-0.37851	########
T80-T88	1	1915	0.0365	0.1569	0.0961	incidence	-0.1183	########
T80-T88	1	1915	0.0365	0.1569	0.0961	impact	0.0386	########
T80-T88	1	1925	0.0365	0.1569	0.0961	incidence	-0.07091	########
T80-T88	1	1925	0.0365	0.1569	0.0961	impact	0.08599	########
T80-T88	1	1935	0.0365	0.1569	0.0961	incidence	0.01491	########
T80-T88	1	1935	0.0365	0.1569	0.0961	impact	0.17181	########
T80-T88	1	1945	0.0365	0.1569	0.0961	incidence	0.07947	########
T80-T88	1	1945	0.0365	0.1569	0.0961	impact	0.23637	########
T80-T88	1	1955	0.0365	0.1569	0.0961	incidence	-0.06007	0.0244
T80-T88	1	1955	0.0365	0.1569	0.0961	impact	0.09683	########
T80-T88	2	1915	0.0286	0.2264	0.053	incidence	0.0327	########
T80-T88	2	1915	0.0286	0.2264	0.053	impact	0.2591	########
T80-T88	2	1925	0.0286	0.2264	0.053	incidence	0.00919	########
T80-T88	2	1925	0.0286	0.2264	0.053	impact	0.23559	########
T80-T88	2	1935	0.0286	0.2264	0.053	incidence	0.00311	########
T80-T88	2	1935	0.0286	0.2264	0.053	impact	0.22951	########
T80-T88	2	1945	0.0286	0.2264	0.053	incidence	-0.05197	########
T80-T88	2	1945	0.0286	0.2264	0.053	impact	0.17443	########
T80-T88	2	1955	0.0286	0.2264	0.053	incidence	-0.12703	0.0225
T80-T88	2	1955	0.0286	0.2264	0.053	impact	0.09937	########
N17-N19	1	1915	0.0338	0.1446	0.0628	incidence	-0.2104	########
N17-N19	1	1915	0.0338	0.1446	0.0628	impact	-0.0658	########
N17-N19	1	1925	0.0338	0.1446	0.0628	incidence	-0.02535	########
N17-N19	1	1925	0.0338	0.1446	0.0628	impact	0.11925	########
N17-N19	1	1935	0.0338	0.1446	0.0628	incidence	0.13495	########
N17-N19	1	1935	0.0338	0.1446	0.0628	impact	0.27955	########
N17-N19	1	1945	0.0338	0.1446	0.0628	incidence	0.08463	########

For peer review only

N17-N19	1	1945	0.0338	0.1446	0.0628	impact	0.22923	########
N17-N19	1	1955	0.0338	0.1446	0.0628	incidence	0.17037	0.0464
N17-N19	1	1955	0.0338	0.1446	0.0628	impact	0.31497	########
N17-N19	2	1915	0.0287	0.2572	0.0793	incidence	-0.3939	########
N17-N19	2	1915	0.0287	0.2572	0.0793	impact	-0.1367	########
N17-N19	2	1925	0.0287	0.2572	0.0793	incidence	-0.14805	########
N17-N19	2	1925	0.0287	0.2572	0.0793	impact	0.10915	########
N17-N19	2	1935	0.0287	0.2572	0.0793	incidence	0.09315	########
N17-N19	2	1935	0.0287	0.2572	0.0793	impact	0.35035	########
N17-N19	2	1945	0.0287	0.2572	0.0793	incidence	0.07863	########
N17-N19	2	1945	0.0287	0.2572	0.0793	impact	0.33583	########
N17-N19	2	1955	0.0287	0.2572	0.0793	incidence	0.04037	0.0521
N17-N19	2	1955	0.0287	0.2572	0.0793	impact	0.29757	########
I80-I89	1	1915	0.0281	0.3714	0.0539	incidence	0.3881	########
I80-I89	1	1915	0.0281	0.3714	0.0539	impact	0.7595	########
I80-I89	1	1925	0.0281	0.3714	0.0539	incidence	0.10496	########
I80-I89	1	1925	0.0281	0.3714	0.0539	impact	0.47636	########
I80-I89	1	1935	0.0281	0.3714	0.0539	incidence	0.19824	########
I80-I89	1	1935	0.0281	0.3714	0.0539	impact	0.56964	########
I80-I89	1	1945	0.0281	0.3714	0.0539	incidence	0.26099	########
I80-I89	1	1945	0.0281	0.3714	0.0539	impact	0.63239	########
I80-I89	1	1955	0.0281	0.3714	0.0539	incidence	0.19801	0.0182
I80-I89	1	1955	0.0281	0.3714	0.0539	impact	0.56941	########
I80-I89	2	1915	0.0285	0.0909	0.0634	incidence	0.1976	########
I80-I89	2	1915	0.0285	0.0909	0.0634	impact	0.2885	########
I80-I89	2	1925	0.0285	0.0909	0.0634	incidence	0.21587	########
I80-I89	2	1925	0.0285	0.0909	0.0634	impact	0.30677	########
I80-I89	2	1935	0.0285	0.0909	0.0634	incidence	0.16693	########
I80-I89	2	1935	0.0285	0.0909	0.0634	impact	0.25783	########
I80-I89	2	1945	0.0285	0.0909	0.0634	incidence	0.27575	########
I80-I89	2	1945	0.0285	0.0909	0.0634	impact	0.36665	########
I80-I89	2	1955	0.0285	0.0909	0.0634	incidence	0.29525	0.0179
I80-I89	2	1955	0.0285	0.0909	0.0634	impact	0.38615	########
K90-K93	1	1915	0.0302	0.2217	0.0834	incidence	0.0864	########
K90-K93	1	1915	0.0302	0.2217	0.0834	impact	0.3081	########
K90-K93	1	1925	0.0302	0.2217	0.0834	incidence	0.10234	########
K90-K93	1	1925	0.0302	0.2217	0.0834	impact	0.32404	########
K90-K93	1	1935	0.0302	0.2217	0.0834	incidence	0.03256	########
K90-K93	1	1935	0.0302	0.2217	0.0834	impact	0.25426	########
K90-K93	1	1945	0.0302	0.2217	0.0834	incidence	0.03636	########
K90-K93	1	1945	0.0302	0.2217	0.0834	impact	0.25806	########
K90-K93	1	1955	0.0302	0.2217	0.0834	incidence	-0.00546	0.0286
K90-K93	1	1955	0.0302	0.2217	0.0834	impact	0.21624	########
K90-K93	2	1915	0.0226	0.1189	0.0755	incidence	0.0774	########
K90-K93	2	1915	0.0226	0.1189	0.0755	impact	0.1963	########
K90-K93	2	1925	0.0226	0.1189	0.0755	incidence	0.12696	########
K90-K93	2	1925	0.0226	0.1189	0.0755	impact	0.24586	########
K90-K93	2	1935	0.0226	0.1189	0.0755	incidence	0.00024	########
K90-K93	2	1935	0.0226	0.1189	0.0755	impact	0.11914	########
K90-K93	2	1945	0.0226	0.1189	0.0755	incidence	-0.09527	########
K90-K93	2	1945	0.0226	0.1189	0.0755	impact	0.02363	########
K90-K93	2	1955	0.0226	0.1189	0.0755	incidence	-0.21273	0.0316
K90-K93	2	1955	0.0226	0.1189	0.0755	impact	-0.09383	########
I70-I79	1	1915	0.03	0.1357	0.0437	incidence	0.1092	########
I70-I79	1	1915	0.03	0.1357	0.0437	impact	0.2449	########
I70-I79	1	1925	0.03	0.1357	0.0437	incidence	0.36925	########

For peer review only

I70-I79	1	1925	0.03	0.1357	0.0437	impact	0.50495	########
I70-I79	1	1935	0.03	0.1357	0.0437	incidence	0.45645	########
I70-I79	1	1935	0.03	0.1357	0.0437	impact	0.59215	########
I70-I79	1	1945	0.03	0.1357	0.0437	incidence	0.47805	########
I70-I79	1	1945	0.03	0.1357	0.0437	impact	0.61375	########
I70-I79	1	1955	0.03	0.1357	0.0437	incidence	0.38195	0.0354
I70-I79	1	1955	0.03	0.1357	0.0437	impact	0.51765	########
I70-I79	2	1915	0.0226	0.0218	0.0605	incidence	0.168	########
I70-I79	2	1915	0.0226	0.0218	0.0605	impact	0.1898	########
I70-I79	2	1925	0.0226	0.0218	0.0605	incidence	0.40739	########
I70-I79	2	1925	0.0226	0.0218	0.0605	impact	0.42919	########
I70-I79	2	1935	0.0226	0.0218	0.0605	incidence	0.57661	########
I70-I79	2	1935	0.0226	0.0218	0.0605	impact	0.59841	########
I70-I79	2	1945	0.0226	0.0218	0.0605	incidence	0.52262	########
I70-I79	2	1945	0.0226	0.0218	0.0605	impact	0.54442	########
I70-I79	2	1955	0.0226	0.0218	0.0605	incidence	0.57738	0.0435
I70-I79	2	1955	0.0226	0.0218	0.0605	impact	0.59918	########
K50-K52	1	1915	0.0216	0.1408	0.0282	incidence	0.2617	########
K50-K52	1	1915	0.0216	0.1408	0.0282	impact	0.4025	########
K50-K52	1	1925	0.0216	0.1408	0.0282	incidence	0.28652	########
K50-K52	1	1925	0.0216	0.1408	0.0282	impact	0.42732	########
K50-K52	1	1935	0.0216	0.1408	0.0282	incidence	0.37488	########
K50-K52	1	1935	0.0216	0.1408	0.0282	impact	0.51568	########
K50-K52	1	1945	0.0216	0.1408	0.0282	incidence	0.39694	########
K50-K52	1	1945	0.0216	0.1408	0.0282	impact	0.53774	########
K50-K52	1	1955	0.0216	0.1408	0.0282	incidence	0.35606	0.0276
K50-K52	1	1955	0.0216	0.1408	0.0282	impact	0.49686	########
K50-K52	2	1915	0.0273	0.092	0.0373	incidence	0.1237	########
K50-K52	2	1915	0.0273	0.092	0.0373	impact	0.2157	########
K50-K52	2	1925	0.0273	0.092	0.0373	incidence	0.30682	########
K50-K52	2	1925	0.0273	0.092	0.0373	impact	0.39882	########
K50-K52	2	1935	0.0273	0.092	0.0373	incidence	0.36828	########
K50-K52	2	1935	0.0273	0.092	0.0373	impact	0.46028	########
K50-K52	2	1945	0.0273	0.092	0.0373	incidence	0.36265	########
K50-K52	2	1945	0.0273	0.092	0.0373	impact	0.45465	########
K50-K52	2	1955	0.0273	0.092	0.0373	incidence	0.33635	0.0234
K50-K52	2	1955	0.0273	0.092	0.0373	impact	0.42835	########
S00-S09	1	1915	0.0332	0.1778	0.0483	incidence	-0.2562	########
S00-S09	1	1915	0.0332	0.1778	0.0483	impact	-0.0784	########
S00-S09	1	1925	0.0332	0.1778	0.0483	incidence	-0.1698	########
S00-S09	1	1925	0.0332	0.1778	0.0483	impact	0.008	########
S00-S09	1	1935	0.0332	0.1778	0.0483	incidence	0.1059	########
S00-S09	1	1935	0.0332	0.1778	0.0483	impact	0.2837	########
S00-S09	1	1945	0.0332	0.1778	0.0483	incidence	0.37286	########
S00-S09	1	1945	0.0332	0.1778	0.0483	impact	0.55066	########
S00-S09	1	1955	0.0332	0.1778	0.0483	incidence	0.34514	0.0222
S00-S09	1	1955	0.0332	0.1778	0.0483	impact	0.52294	########
S00-S09	2	1915	0.0171	-0.1563	0.0589	incidence	-0.1956	########
S00-S09	2	1915	0.0171	-0.1563	0.0589	impact	-0.3519	########
S00-S09	2	1925	0.0171	-0.1563	0.0589	incidence	-0.11854	########
S00-S09	2	1925	0.0171	-0.1563	0.0589	impact	-0.27484	########
S00-S09	2	1935	0.0171	-0.1563	0.0589	incidence	-0.06236	########
S00-S09	2	1935	0.0171	-0.1563	0.0589	impact	-0.21866	########
S00-S09	2	1945	0.0171	-0.1563	0.0589	incidence	0.0723	########
S00-S09	2	1945	0.0171	-0.1563	0.0589	impact	-0.084	########
S00-S09	2	1955	0.0171	-0.1563	0.0589	incidence	0.1417	0.0329

S00-S09	2	1955	0.0171	-0.1563	0.0589	impact	-0.0146	########
C50-C50	1	1915	0.000565	1.1527	0.6432	incidence	NA	NA
C50-C50	1	1915	0.000565	1.1527	0.6432	impact	NA	NA
C50-C50	1	1925	0.000565	1.1527	0.6432	incidence	-0.08219	########
C50-C50	1	1925	0.000565	1.1527	0.6432	impact	1.07051	########
C50-C50	1	1935	0.000565	1.1527	0.6432	incidence	0.37979	########
C50-C50	1	1935	0.000565	1.1527	0.6432	impact	1.53249	########
C50-C50	1	1945	0.000565	1.1527	0.6432	incidence	0.66213	########
C50-C50	1	1945	0.000565	1.1527	0.6432	impact	1.81483	########
C50-C50	1	1955	0.000565	1.1527	0.6432	incidence	0.78787	0.283
C50-C50	1	1955	0.000565	1.1527	0.6432	impact	1.94057	########
C50-C50	2	1915	0.0434	1.1487	0.0744	incidence	0.2888	########
C50-C50	2	1915	0.0434	1.1487	0.0744	impact	1.4375	########
C50-C50	2	1925	0.0434	1.1487	0.0744	incidence	0.23679	########
C50-C50	2	1925	0.0434	1.1487	0.0744	impact	1.38549	########
C50-C50	2	1935	0.0434	1.1487	0.0744	incidence	0.12331	########
C50-C50	2	1935	0.0434	1.1487	0.0744	impact	1.27201	########
C50-C50	2	1945	0.0434	1.1487	0.0744	incidence	0.18162	########
C50-C50	2	1945	0.0434	1.1487	0.0744	impact	1.33032	########
C50-C50	2	1955	0.0434	1.1487	0.0744	incidence	0.21438	0.0199
C50-C50	2	1955	0.0434	1.1487	0.0744	impact	1.36308	########
C60-C63	1	1915	0.0287	0.6987	0.0671	incidence	0.205	########
C60-C63	1	1915	0.0287	0.6987	0.0671	impact	0.9037	########
C60-C63	1	1925	0.0287	0.6987	0.0671	incidence	0.3432	########
C60-C63	1	1925	0.0287	0.6987	0.0671	impact	1.0419	########
C60-C63	1	1935	0.0287	0.6987	0.0671	incidence	0.1078	########
C60-C63	1	1935	0.0287	0.6987	0.0671	impact	0.8065	########
C60-C63	1	1945	0.0287	0.6987	0.0671	incidence	-0.0825	########
C60-C63	1	1945	0.0287	0.6987	0.0671	impact	0.6162	########
C60-C63	1	1955	0.0287	0.6987	0.0671	incidence	-0.0995	0.0448
C60-C63	1	1955	0.0287	0.6987	0.0671	impact	0.5992	########
C60-C63	2	1915	-3.57E-06	NA	NA	incidence	NA	0
C60-C63	2	1915	-3.57E-06	NA	NA	impact	NA	0
C60-C63	2	1925	-3.57E-06	NA	NA	incidence	NA	NA
C60-C63	2	1925	-3.57E-06	NA	NA	impact	NA	NA
C60-C63	2	1935	-3.57E-06	NA	NA	incidence	NA	NA
C60-C63	2	1935	-3.57E-06	NA	NA	impact	NA	NA
C60-C63	2	1945	-3.57E-06	NA	NA	incidence	NA	NA
C60-C63	2	1945	-3.57E-06	NA	NA	impact	NA	NA
C60-C63	2	1955	-3.57E-06	NA	NA	incidence	NA	NA
C60-C63	2	1955	-3.57E-06	NA	NA	impact	NA	NA
G30-G32	1	1915	0.00307	-0.0377	0.2699	incidence	0.1939	########
G30-G32	1	1915	0.00307	-0.0377	0.2699	impact	0.1562	########
G30-G32	1	1925	0.00307	-0.0377	0.2699	incidence	0.21645	########
G30-G32	1	1925	0.00307	-0.0377	0.2699	impact	0.17875	########
G30-G32	1	1935	0.00307	-0.0377	0.2699	incidence	0.48805	########
G30-G32	1	1935	0.00307	-0.0377	0.2699	impact	0.45035	########
G30-G32	1	1945	0.00307	-0.0377	0.2699	incidence	0.50373	########
G30-G32	1	1945	0.00307	-0.0377	0.2699	impact	0.46603	########
G30-G32	1	1955	0.00307	-0.0377	0.2699	incidence	0.21327	0.149
G30-G32	1	1955	0.00307	-0.0377	0.2699	impact	0.17557	########
G30-G32	2	1915	0.00265	0.064	0.1112	incidence	-0.0443	########
G30-G32	2	1915	0.00265	0.064	0.1112	impact	0.0197	########
G30-G32	2	1925	0.00265	0.064	0.1112	incidence	0.3435	########
G30-G32	2	1925	0.00265	0.064	0.1112	impact	0.4075	########
G30-G32	2	1935	0.00265	0.064	0.1112	incidence	0.5937	########

For peer review only

G30-G32	2	1935	0.00265	0.064	0.1112	impact	0.6577	########
G30-G32	2	1945	0.00265	0.064	0.1112	incidence	0.30487	########
G30-G32	2	1945	0.00265	0.064	0.1112	impact	0.36887	########
G30-G32	2	1955	0.00265	0.064	0.1112	incidence	0.11713	0.194
G30-G32	2	1955	0.00265	0.064	0.1112	impact	0.18113	########
ACT-DTH	1	1915	NA	NA	NA	incidence	-0.028	########
ACT-DTH	1	1915	NA	NA	NA	impact	-0.028	########
ACT-DTH	1	1925	NA	NA	NA	incidence	0.235	########
ACT-DTH	1	1925	NA	NA	NA	impact	0.235	########
ACT-DTH	1	1935	NA	NA	NA	incidence	0.335	########
ACT-DTH	1	1935	NA	NA	NA	impact	0.335	########
ACT-DTH	1	1945	NA	NA	NA	incidence	0.393	########
ACT-DTH	1	1945	NA	NA	NA	impact	0.393	########
ACT-DTH	1	1955	NA	NA	NA	incidence	0.33	0.0442
ACT-DTH	1	1955	NA	NA	NA	impact	0.33	0.0442
ACT-DTH	2	1915	NA	NA	NA	incidence	-0.063	########
ACT-DTH	2	1915	NA	NA	NA	impact	-0.063	########
ACT-DTH	2	1925	NA	NA	NA	incidence	0.155	########
ACT-DTH	2	1925	NA	NA	NA	impact	0.155	########
ACT-DTH	2	1935	NA	NA	NA	incidence	0.321	########
ACT-DTH	2	1935	NA	NA	NA	impact	0.321	########
ACT-DTH	2	1945	NA	NA	NA	incidence	0.346	########
ACT-DTH	2	1945	NA	NA	NA	impact	0.346	########
ACT-DTH	2	1955	NA	NA	NA	incidence	0.297	0.0403
ACT-DTH	2	1955	NA	NA	NA	impact	0.297	0.0403
ACT-DTH	3	1915	NA	NA	NA	incidence	-0.101	########
ACT-DTH	3	1915	NA	NA	NA	impact	-0.101	########
ACT-DTH	3	1925	NA	NA	NA	incidence	0.131	########
ACT-DTH	3	1925	NA	NA	NA	impact	0.131	########
ACT-DTH	3	1935	NA	NA	NA	incidence	0.343	########
ACT-DTH	3	1935	NA	NA	NA	impact	0.343	########
ACT-DTH	3	1945	NA	NA	NA	incidence	0.332	########
ACT-DTH	3	1945	NA	NA	NA	impact	0.332	########
ACT-DTH	3	1955	NA	NA	NA	incidence	0.305	0.0383
ACT-DTH	3	1955	NA	NA	NA	impact	0.305	0.0383
ACT-DTH	4	1915	NA	NA	NA	incidence	-0.106	########
ACT-DTH	4	1915	NA	NA	NA	impact	-0.106	########
ACT-DTH	4	1925	NA	NA	NA	incidence	0.144	########
ACT-DTH	4	1925	NA	NA	NA	impact	0.144	########
ACT-DTH	4	1935	NA	NA	NA	incidence	0.299	########
ACT-DTH	4	1935	NA	NA	NA	impact	0.299	########
ACT-DTH	4	1945	NA	NA	NA	incidence	0.299	########
ACT-DTH	4	1945	NA	NA	NA	impact	0.299	########
ACT-DTH	4	1955	NA	NA	NA	incidence	0.274	0.0363
ACT-DTH	4	1955	NA	NA	NA	impact	0.274	0.0363
ACT-DTH	5	1915	NA	NA	NA	incidence	-0.093	########
ACT-DTH	5	1915	NA	NA	NA	impact	-0.093	########
ACT-DTH	5	1925	NA	NA	NA	incidence	0.102	########
ACT-DTH	5	1925	NA	NA	NA	impact	0.102	########
ACT-DTH	5	1935	NA	NA	NA	incidence	0.312	########
ACT-DTH	5	1935	NA	NA	NA	impact	0.312	########
ACT-DTH	5	1945	NA	NA	NA	incidence	0.299	########
ACT-DTH	5	1945	NA	NA	NA	impact	0.299	########
ACT-DTH	5	1955	NA	NA	NA	incidence	0.281	0.0342
ACT-DTH	5	1955	NA	NA	NA	impact	0.281	0.0342
ACT-DTH	6	1915	NA	NA	NA	incidence	-0.039	########

For peer review only

ACT-DTH	6	1915	NA	NA	NA	impact	-0.039	########
ACT-DTH	6	1925	NA	NA	NA	incidence	0.144	########
ACT-DTH	6	1925	NA	NA	NA	impact	0.144	########
ACT-DTH	6	1935	NA	NA	NA	incidence	0.298	########
ACT-DTH	6	1935	NA	NA	NA	impact	0.298	########
ACT-DTH	6	1945	NA	NA	NA	incidence	0.314	########
ACT-DTH	6	1945	NA	NA	NA	impact	0.314	########
ACT-DTH	6	1955	NA	NA	NA	incidence	0.326	0.0324
ACT-DTH	6	1955	NA	NA	NA	impact	0.326	0.0324
ACT-DTH	7	1915	NA	NA	NA	incidence	-0.03	########
ACT-DTH	7	1915	NA	NA	NA	impact	-0.03	########
ACT-DTH	7	1925	NA	NA	NA	incidence	0.121	########
ACT-DTH	7	1925	NA	NA	NA	impact	0.121	########
ACT-DTH	7	1935	NA	NA	NA	incidence	0.23	########
ACT-DTH	7	1935	NA	NA	NA	impact	0.23	########
ACT-DTH	7	1945	NA	NA	NA	incidence	0.333	########
ACT-DTH	7	1945	NA	NA	NA	impact	0.333	########
ACT-DTH	7	1955	NA	NA	NA	incidence	0.236	0.0303
ACT-DTH	7	1955	NA	NA	NA	impact	0.236	0.0303
ACT-DTH	8	1915	NA	NA	NA	incidence	-0.047	########
ACT-DTH	8	1915	NA	NA	NA	impact	-0.047	########
ACT-DTH	8	1925	NA	NA	NA	incidence	0.137	########
ACT-DTH	8	1925	NA	NA	NA	impact	0.137	########
ACT-DTH	8	1935	NA	NA	NA	incidence	0.247	########
ACT-DTH	8	1935	NA	NA	NA	impact	0.247	########
ACT-DTH	8	1945	NA	NA	NA	incidence	0.28	########
ACT-DTH	8	1945	NA	NA	NA	impact	0.28	########
ACT-DTH	8	1955	NA	NA	NA	incidence	0.279	0.0281
ACT-DTH	8	1955	NA	NA	NA	impact	0.279	0.0281
ACT-DTH	9	1915	NA	NA	NA	incidence	-0.071	########
ACT-DTH	9	1915	NA	NA	NA	impact	-0.071	########
ACT-DTH	9	1925	NA	NA	NA	incidence	0.117	########
ACT-DTH	9	1925	NA	NA	NA	impact	0.117	########
ACT-DTH	9	1935	NA	NA	NA	incidence	0.224	########
ACT-DTH	9	1935	NA	NA	NA	impact	0.224	########
ACT-DTH	9	1945	NA	NA	NA	incidence	0.271	########
ACT-DTH	9	1945	NA	NA	NA	impact	0.271	########
ACT-DTH	9	1955	NA	NA	NA	incidence	0.339	0.0261
ACT-DTH	9	1955	NA	NA	NA	impact	0.339	0.0261
ACT-DTH	10	1915	NA	NA	NA	incidence	-0.049	########
ACT-DTH	10	1915	NA	NA	NA	impact	-0.049	########
ACT-DTH	10	1925	NA	NA	NA	incidence	0.018	########
ACT-DTH	10	1925	NA	NA	NA	impact	0.018	########
ACT-DTH	10	1935	NA	NA	NA	incidence	0.198	########
ACT-DTH	10	1935	NA	NA	NA	impact	0.198	########
ACT-DTH	10	1945	NA	NA	NA	incidence	0.315	########
ACT-DTH	10	1945	NA	NA	NA	impact	0.315	########
ACT-DTH	10	1955	NA	NA	NA	incidence	0.34	0.0232
ACT-DTH	10	1955	NA	NA	NA	impact	0.34	0.0232
EST-DTH	1	1915	1.09419	########	########	incidence	########	########
EST-DTH	1	1915	1.09419	########	########	impact	########	########
EST-DTH	1	1925	1.09419	########	########	incidence	########	########
EST-DTH	1	1925	1.09419	########	########	impact	########	########
EST-DTH	1	1935	1.09419	########	########	incidence	########	########
EST-DTH	1	1935	1.09419	########	########	impact	########	########
EST-DTH	1	1945	1.09419	########	########	incidence	########	########

For peer review only

EST-DTH	1	1945	1.09419	########	########	impact	########	########
EST-DTH	1	1955	1.09419	########	########	incidence	########	########
EST-DTH	1	1955	1.09419	########	########	impact	########	########
EST-DTH	2	1915	1.16032	########	########	incidence	########	########
EST-DTH	2	1915	1.16032	########	########	impact	########	########
EST-DTH	2	1925	1.16032	########	########	incidence	########	########
EST-DTH	2	1925	1.16032	########	########	impact	########	########
EST-DTH	2	1935	1.16032	########	########	incidence	########	########
EST-DTH	2	1935	1.16032	########	########	impact	########	########
EST-DTH	2	1945	1.16032	########	########	incidence	########	########
EST-DTH	2	1945	1.16032	########	########	impact	########	########
EST-DTH	2	1955	1.16032	########	########	incidence	########	########
EST-DTH	2	1955	1.16032	########	########	impact	########	########
EST-DTH	3	1915	1.17692	########	########	incidence	########	########
EST-DTH	3	1915	1.17692	########	########	impact	########	########
EST-DTH	3	1925	1.17692	########	########	incidence	########	########
EST-DTH	3	1925	1.17692	########	########	impact	########	########
EST-DTH	3	1935	1.17692	########	########	incidence	########	########
EST-DTH	3	1935	1.17692	########	########	impact	########	########
EST-DTH	3	1945	1.17692	########	########	incidence	########	########
EST-DTH	3	1945	1.17692	########	########	impact	########	########
EST-DTH	3	1955	1.17692	########	########	incidence	########	########
EST-DTH	3	1955	1.17692	########	########	impact	########	########
EST-DTH	4	1915	1.23779	########	########	incidence	########	########
EST-DTH	4	1915	1.23779	########	########	impact	########	########
EST-DTH	4	1925	1.23779	########	########	incidence	########	########
EST-DTH	4	1925	1.23779	########	########	impact	########	########
EST-DTH	4	1935	1.23779	########	########	incidence	########	########
EST-DTH	4	1935	1.23779	########	########	impact	########	########
EST-DTH	4	1945	1.23779	########	########	incidence	########	########
EST-DTH	4	1945	1.23779	########	########	impact	########	########
EST-DTH	4	1955	1.23779	########	########	incidence	########	########
EST-DTH	4	1955	1.23779	########	########	impact	########	########
EST-DTH	5	1915	1.31797	########	########	incidence	########	########
EST-DTH	5	1915	1.31797	########	########	impact	########	########
EST-DTH	5	1925	1.31797	########	########	incidence	########	########
EST-DTH	5	1925	1.31797	########	########	impact	########	########
EST-DTH	5	1935	1.31797	########	########	incidence	########	########
EST-DTH	5	1935	1.31797	########	########	impact	########	########
EST-DTH	5	1945	1.31797	########	########	incidence	########	########
EST-DTH	5	1945	1.31797	########	########	impact	########	########
EST-DTH	5	1955	1.31797	########	########	incidence	########	########
EST-DTH	5	1955	1.31797	########	########	impact	########	########
EST-DTH	6	1915	1.41303	########	########	incidence	########	########
EST-DTH	6	1915	1.41303	########	########	impact	########	########
EST-DTH	6	1925	1.41303	########	########	incidence	########	########
EST-DTH	6	1925	1.41303	########	########	impact	########	########
EST-DTH	6	1935	1.41303	########	########	incidence	########	########
EST-DTH	6	1935	1.41303	########	########	impact	########	########
EST-DTH	6	1945	1.41303	########	########	incidence	########	########
EST-DTH	6	1945	1.41303	########	########	impact	########	########
EST-DTH	6	1955	1.41303	########	########	incidence	########	########
EST-DTH	6	1955	1.41303	########	########	impact	########	########
EST-DTH	7	1915	1.49752	########	########	incidence	########	########
EST-DTH	7	1915	1.49752	########	########	impact	########	########
EST-DTH	7	1925	1.49752	########	########	incidence	########	########

For peer review only

EST-DTH	7	1925	1.49752	########	########	impact	########	########
EST-DTH	7	1935	1.49752	########	########	incidence	########	########
EST-DTH	7	1935	1.49752	########	########	impact	########	########
EST-DTH	7	1945	1.49752	########	########	incidence	########	########
EST-DTH	7	1945	1.49752	########	########	impact	########	########
EST-DTH	7	1955	1.49752	########	########	incidence	########	########
EST-DTH	7	1955	1.49752	########	########	impact	########	########
EST-DTH	8	1915	1.54785	########	########	incidence	########	########
EST-DTH	8	1915	1.54785	########	########	impact	########	########
EST-DTH	8	1925	1.54785	########	########	incidence	########	########
EST-DTH	8	1925	1.54785	########	########	impact	########	########
EST-DTH	8	1935	1.54785	########	########	incidence	########	########
EST-DTH	8	1935	1.54785	########	########	impact	########	########
EST-DTH	8	1945	1.54785	########	########	incidence	########	########
EST-DTH	8	1945	1.54785	########	########	impact	########	########
EST-DTH	8	1955	1.54785	########	########	incidence	########	########
EST-DTH	8	1955	1.54785	########	########	impact	########	########
EST-DTH	9	1915	1.61129	########	########	incidence	########	########
EST-DTH	9	1915	1.61129	########	########	impact	########	########
EST-DTH	9	1925	1.61129	########	########	incidence	########	########
EST-DTH	9	1925	1.61129	########	########	impact	########	########
EST-DTH	9	1935	1.61129	########	########	incidence	########	########
EST-DTH	9	1935	1.61129	########	########	impact	########	########
EST-DTH	9	1945	1.61129	########	########	incidence	########	########
EST-DTH	9	1945	1.61129	########	########	impact	########	########
EST-DTH	9	1955	1.61129	########	########	incidence	########	########
EST-DTH	9	1955	1.61129	########	########	impact	########	########
EST-DTH	10	1915	1.71953	########	########	incidence	########	########
EST-DTH	10	1915	1.71953	########	########	impact	########	########
EST-DTH	10	1925	1.71953	########	########	incidence	########	########
EST-DTH	10	1925	1.71953	########	########	impact	########	########
EST-DTH	10	1935	1.71953	########	########	incidence	########	########
EST-DTH	10	1935	1.71953	########	########	impact	########	########
EST-DTH	10	1945	1.71953	########	########	incidence	########	########
EST-DTH	10	1945	1.71953	########	########	impact	########	########
EST-DTH	10	1955	1.71953	########	########	incidence	########	########
EST-DTH	10	1955	1.71953	########	########	impact	########	########
J09-J18	1	1915	0.06736	-0.0811	0.2164	incidence	-0.25274	########
J09-J18	1	1915	0.06736	-0.0811	0.2164	impact	-0.33384	########
J09-J18	1	1925	0.06736	-0.0811	0.2164	incidence	-0.1329	########
J09-J18	1	1925	0.06736	-0.0811	0.2164	impact	-0.214	########
J09-J18	1	1935	0.06736	-0.0811	0.2164	incidence	-0.09771	########
J09-J18	1	1935	0.06736	-0.0811	0.2164	impact	-0.17881	########
J09-J18	1	1945	0.06736	-0.0811	0.2164	incidence	-0.196809	########
J09-J18	1	1945	0.06736	-0.0811	0.2164	impact	-0.277909	########
J09-J18	1	1955	0.06736	-0.0811	0.2164	incidence	-0.297931	0.085
J09-J18	1	1955	0.06736	-0.0811	0.2164	impact	-0.379031	########
J09-J18	2	1915	0.06656	0.186	0.1322	incidence	-0.28315	########
J09-J18	2	1915	0.06656	0.186	0.1322	impact	-0.09715	########
J09-J18	2	1925	0.06656	0.186	0.1322	incidence	-0.12495	########
J09-J18	2	1925	0.06656	0.186	0.1322	impact	0.06105	########
J09-J18	2	1935	0.06656	0.186	0.1322	incidence	-0.04126	########
J09-J18	2	1935	0.06656	0.186	0.1322	impact	0.14474	########
J09-J18	2	1945	0.06656	0.186	0.1322	incidence	-0.243141	########
J09-J18	2	1945	0.06656	0.186	0.1322	impact	-0.057141	########
J09-J18	2	1955	0.06656	0.186	0.1322	incidence	-0.337499	0.0795

For peer review only

J09-J18	2	1955	0.06656	0.186	0.1322	impact	-0.151499	########
J09-J18	3	1915	0.07515	0.0534	0.2479	incidence	-0.20366	########
J09-J18	3	1915	0.07515	0.0534	0.2479	impact	-0.15026	########
J09-J18	3	1925	0.07515	0.0534	0.2479	incidence	-0.15718	########
J09-J18	3	1925	0.07515	0.0534	0.2479	impact	-0.10378	########
J09-J18	3	1935	0.07515	0.0534	0.2479	incidence	-0.07526	########
J09-J18	3	1935	0.07515	0.0534	0.2479	impact	-0.02186	########
J09-J18	3	1945	0.07515	0.0534	0.2479	incidence	-0.125819	########
J09-J18	3	1945	0.07515	0.0534	0.2479	impact	-0.072419	########
J09-J18	3	1955	0.07515	0.0534	0.2479	incidence	-0.192171	0.0742
J09-J18	3	1955	0.07515	0.0534	0.2479	impact	-0.138771	########
J09-J18	4	1915	0.07726	-0.0173	0.1405	incidence	-0.23646	########
J09-J18	4	1915	0.07726	-0.0173	0.1405	impact	-0.25376	########
J09-J18	4	1925	0.07726	-0.0173	0.1405	incidence	0.01704	########
J09-J18	4	1925	0.07726	-0.0173	0.1405	impact	-0.00026	########
J09-J18	4	1935	0.07726	-0.0173	0.1405	incidence	-0.11377	########
J09-J18	4	1935	0.07726	-0.0173	0.1405	impact	-0.13107	########
J09-J18	4	1945	0.07726	-0.0173	0.1405	incidence	-0.181218	########
J09-J18	4	1945	0.07726	-0.0173	0.1405	impact	-0.198518	########
J09-J18	4	1955	0.07726	-0.0173	0.1405	incidence	-0.205792	0.0725
J09-J18	4	1955	0.07726	-0.0173	0.1405	impact	-0.223092	########
J09-J18	5	1915	0.0863	0.196	0.1213	incidence	-0.14255	########
J09-J18	5	1915	0.0863	0.196	0.1213	impact	0.05345	########
J09-J18	5	1925	0.0863	0.196	0.1213	incidence	-0.15324	########
J09-J18	5	1925	0.0863	0.196	0.1213	impact	0.04276	########
J09-J18	5	1935	0.0863	0.196	0.1213	incidence	-0.06779	########
J09-J18	5	1935	0.0863	0.196	0.1213	impact	0.12821	########
J09-J18	5	1945	0.0863	0.196	0.1213	incidence	-0.156126	########
J09-J18	5	1945	0.0863	0.196	0.1213	impact	0.039874	########
J09-J18	5	1955	0.0863	0.196	0.1213	incidence	-0.229254	0.0672
J09-J18	5	1955	0.0863	0.196	0.1213	impact	-0.033254	########
J09-J18	6	1915	0.09347	0.321	0.1602	incidence	-0.23769	########
J09-J18	6	1915	0.09347	0.321	0.1602	impact	0.08331	########
J09-J18	6	1925	0.09347	0.321	0.1602	incidence	-0.09018	########
J09-J18	6	1925	0.09347	0.321	0.1602	impact	0.23082	########
J09-J18	6	1935	0.09347	0.321	0.1602	incidence	-0.11295	########
J09-J18	6	1935	0.09347	0.321	0.1602	impact	0.20805	########
J09-J18	6	1945	0.09347	0.321	0.1602	incidence	-0.205507	########
J09-J18	6	1945	0.09347	0.321	0.1602	impact	0.115493	########
J09-J18	6	1955	0.09347	0.321	0.1602	incidence	-0.154313	0.0631
J09-J18	6	1955	0.09347	0.321	0.1602	impact	0.166687	########
J09-J18	7	1915	0.10779	0.191	0.0632	incidence	-0.14491	########
J09-J18	7	1915	0.10779	0.191	0.0632	impact	0.04609	########
J09-J18	7	1925	0.10779	0.191	0.0632	incidence	-0.08373	########
J09-J18	7	1925	0.10779	0.191	0.0632	impact	0.10727	########
J09-J18	7	1935	0.10779	0.191	0.0632	incidence	-0.17946	########
J09-J18	7	1935	0.10779	0.191	0.0632	impact	0.01154	########
J09-J18	7	1945	0.10779	0.191	0.0632	incidence	-0.184831	########
J09-J18	7	1945	0.10779	0.191	0.0632	impact	0.006169	########
J09-J18	7	1955	0.10779	0.191	0.0632	incidence	-0.188179	0.058
J09-J18	7	1955	0.10779	0.191	0.0632	impact	0.002821	########
J09-J18	8	1915	0.11486	0.19	0.0861	incidence	-0.16189	########
J09-J18	8	1915	0.11486	0.19	0.0861	impact	0.02811	########
J09-J18	8	1925	0.11486	0.19	0.0861	incidence	-0.16481	########
J09-J18	8	1925	0.11486	0.19	0.0861	impact	0.02519	########
J09-J18	8	1935	0.11486	0.19	0.0861	incidence	-0.09828	########

For peer review only

J09-J18	8	1935	0.11486	0.19	0.0861	impact	0.09172	########
J09-J18	8	1945	0.11486	0.19	0.0861	incidence	-0.173728	########
J09-J18	8	1945	0.11486	0.19	0.0861	impact	0.016272	########
J09-J18	8	1955	0.11486	0.19	0.0861	incidence	-0.263072	0.0559
J09-J18	8	1955	0.11486	0.19	0.0861	impact	-0.073072	########
J09-J18	9	1915	0.11924	0.106	0.1007	incidence	-0.25883	########
J09-J18	9	1915	0.11924	0.106	0.1007	impact	-0.15283	########
J09-J18	9	1925	0.11924	0.106	0.1007	incidence	-0.16269	########
J09-J18	9	1925	0.11924	0.106	0.1007	impact	-0.05669	########
J09-J18	9	1935	0.11924	0.106	0.1007	incidence	-0.21843	########
J09-J18	9	1935	0.11924	0.106	0.1007	impact	-0.11243	########
J09-J18	9	1945	0.11924	0.106	0.1007	incidence	-0.217941	########
J09-J18	9	1945	0.11924	0.106	0.1007	impact	-0.111941	########
J09-J18	9	1955	0.11924	0.106	0.1007	incidence	-0.173989	0.0523
J09-J18	9	1955	0.11924	0.106	0.1007	impact	-0.067989	########
J09-J18	10	1915	0.12938	0.2	0.0684	incidence	-0.1645	########
J09-J18	10	1915	0.12938	0.2	0.0684	impact	0.0355	########
J09-J18	10	1925	0.12938	0.2	0.0684	incidence	-0.26477	########
J09-J18	10	1925	0.12938	0.2	0.0684	impact	-0.06477	########
J09-J18	10	1935	0.12938	0.2	0.0684	incidence	-0.13044	########
J09-J18	10	1935	0.12938	0.2	0.0684	impact	0.06956	########
J09-J18	10	1945	0.12938	0.2	0.0684	incidence	-0.183303	########
J09-J18	10	1945	0.12938	0.2	0.0684	impact	0.016697	########
J09-J18	10	1955	0.12938	0.2	0.0684	incidence	-0.196577	0.0474
J09-J18	10	1955	0.12938	0.2	0.0684	impact	0.003423	########
R00-R09	1	1915	0.05824	0.182	0.1512	incidence	0.02092	########
R00-R09	1	1915	0.05824	0.182	0.1512	impact	0.20292	########
R00-R09	1	1925	0.05824	0.182	0.1512	incidence	0.10596	########
R00-R09	1	1925	0.05824	0.182	0.1512	impact	0.28796	########
R00-R09	1	1935	0.05824	0.182	0.1512	incidence	0.14444	########
R00-R09	1	1935	0.05824	0.182	0.1512	impact	0.32644	########
R00-R09	1	1945	0.05824	0.182	0.1512	incidence	0.276299	########
R00-R09	1	1945	0.05824	0.182	0.1512	impact	0.458299	########
R00-R09	1	1955	0.05824	0.182	0.1512	incidence	0.052211	0.035
R00-R09	1	1955	0.05824	0.182	0.1512	impact	0.234211	########
R00-R09	2	1915	0.06563	0.24	0.1145	incidence	-0.12135	########
R00-R09	2	1915	0.06563	0.24	0.1145	impact	0.11865	########
R00-R09	2	1925	0.06563	0.24	0.1145	incidence	0.08422	########
R00-R09	2	1925	0.06563	0.24	0.1145	impact	0.32422	########
R00-R09	2	1935	0.06563	0.24	0.1145	incidence	0.21745	########
R00-R09	2	1935	0.06563	0.24	0.1145	impact	0.45745	########
R00-R09	2	1945	0.06563	0.24	0.1145	incidence	0.183291	########
R00-R09	2	1945	0.06563	0.24	0.1145	impact	0.423291	########
R00-R09	2	1955	0.06563	0.24	0.1145	incidence	0.100679	0.0308
R00-R09	2	1955	0.06563	0.24	0.1145	impact	0.340679	########
R00-R09	3	1915	0.06941	0.24	0.1174	incidence	0.00995	########
R00-R09	3	1915	0.06941	0.24	0.1174	impact	0.24995	########
R00-R09	3	1925	0.06941	0.24	0.1174	incidence	0.10814	########
R00-R09	3	1925	0.06941	0.24	0.1174	impact	0.34814	########
R00-R09	3	1935	0.06941	0.24	0.1174	incidence	0.20023	########
R00-R09	3	1935	0.06941	0.24	0.1174	impact	0.44023	########
R00-R09	3	1945	0.06941	0.24	0.1174	incidence	0.191618	########
R00-R09	3	1945	0.06941	0.24	0.1174	impact	0.431618	########
R00-R09	3	1955	0.06941	0.24	0.1174	incidence	0.072652	0.0313
R00-R09	3	1955	0.06941	0.24	0.1174	impact	0.312652	########
R00-R09	4	1915	0.07076	0.279	0.0989	incidence	-0.06407	########

For peer review only

R00-R09	4	1915	0.07076	0.279	0.0989	impact	0.21493	########
R00-R09	4	1925	0.07076	0.279	0.0989	incidence	0.0683	########
R00-R09	4	1925	0.07076	0.279	0.0989	impact	0.3473	########
R00-R09	4	1935	0.07076	0.279	0.0989	incidence	0.18082	########
R00-R09	4	1935	0.07076	0.279	0.0989	impact	0.45982	########
R00-R09	4	1945	0.07076	0.279	0.0989	incidence	0.222224	########
R00-R09	4	1945	0.07076	0.279	0.0989	impact	0.501224	########
R00-R09	4	1955	0.07076	0.279	0.0989	incidence	0.069536	0.031
R00-R09	4	1955	0.07076	0.279	0.0989	impact	0.348536	########
R00-R09	5	1915	0.07736	0.0266	0.0866	incidence	0.02566	########
R00-R09	5	1915	0.07736	0.0266	0.0866	impact	0.05226	########
R00-R09	5	1925	0.07736	0.0266	0.0866	incidence	0.07666	########
R00-R09	5	1925	0.07736	0.0266	0.0866	impact	0.10326	########
R00-R09	5	1935	0.07736	0.0266	0.0866	incidence	0.17937	########
R00-R09	5	1935	0.07736	0.0266	0.0866	impact	0.20597	########
R00-R09	5	1945	0.07736	0.0266	0.0866	incidence	0.241856	########
R00-R09	5	1945	0.07736	0.0266	0.0866	impact	0.268456	########
R00-R09	5	1955	0.07736	0.0266	0.0866	incidence	0.044334	0.0298
R00-R09	5	1955	0.07736	0.0266	0.0866	impact	0.070934	########
R00-R09	6	1915	0.08497	0.22	0.0933	incidence	0.00207	########
R00-R09	6	1915	0.08497	0.22	0.0933	impact	0.22207	########
R00-R09	6	1925	0.08497	0.22	0.0933	incidence	0.09594	########
R00-R09	6	1925	0.08497	0.22	0.0933	impact	0.31594	########
R00-R09	6	1935	0.08497	0.22	0.0933	incidence	0.21254	########
R00-R09	6	1935	0.08497	0.22	0.0933	impact	0.43254	########
R00-R09	6	1945	0.08497	0.22	0.0933	incidence	0.228018	########
R00-R09	6	1945	0.08497	0.22	0.0933	impact	0.448018	########
R00-R09	6	1955	0.08497	0.22	0.0933	incidence	0.091182	0.0281
R00-R09	6	1955	0.08497	0.22	0.0933	impact	0.311182	########
R00-R09	7	1915	0.094	0.19	0.0637	incidence	0.00292	########
R00-R09	7	1915	0.094	0.19	0.0637	impact	0.19292	########
R00-R09	7	1925	0.094	0.19	0.0637	incidence	0.1214	########
R00-R09	7	1925	0.094	0.19	0.0637	impact	0.3114	########
R00-R09	7	1935	0.094	0.19	0.0637	incidence	0.20566	########
R00-R09	7	1935	0.094	0.19	0.0637	impact	0.39566	########
R00-R09	7	1945	0.094	0.19	0.0637	incidence	0.265992	########
R00-R09	7	1945	0.094	0.19	0.0637	impact	0.455992	########
R00-R09	7	1955	0.094	0.19	0.0637	incidence	0.207888	0.0265
R00-R09	7	1955	0.094	0.19	0.0637	impact	0.397888	########
R00-R09	8	1915	0.09576	0.295	0.0714	incidence	-0.04213	########
R00-R09	8	1915	0.09576	0.295	0.0714	impact	0.25287	########
R00-R09	8	1925	0.09576	0.295	0.0714	incidence	0.07058	########
R00-R09	8	1925	0.09576	0.295	0.0714	impact	0.36558	########
R00-R09	8	1935	0.09576	0.295	0.0714	incidence	0.1935	########
R00-R09	8	1935	0.09576	0.295	0.0714	impact	0.4885	########
R00-R09	8	1945	0.09576	0.295	0.0714	incidence	0.302638	########
R00-R09	8	1945	0.09576	0.295	0.0714	impact	0.597638	########
R00-R09	8	1955	0.09576	0.295	0.0714	incidence	0.169582	0.0251
R00-R09	8	1955	0.09576	0.295	0.0714	impact	0.464582	########
R00-R09	9	1915	0.10204	0.189	0.0585	incidence	-0.19682	########
R00-R09	9	1915	0.10204	0.189	0.0585	impact	-0.00782	########
R00-R09	9	1925	0.10204	0.189	0.0585	incidence	0.10251	########
R00-R09	9	1925	0.10204	0.189	0.0585	impact	0.29151	########
R00-R09	9	1935	0.10204	0.189	0.0585	incidence	0.22646	########
R00-R09	9	1935	0.10204	0.189	0.0585	impact	0.41546	########
R00-R09	9	1945	0.10204	0.189	0.0585	incidence	0.28041	########

For peer review only

R00-R09	9	1945	0.10204	0.189	0.0585	impact	0.46941	########
R00-R09	9	1955	0.10204	0.189	0.0585	incidence	0.21841	0.0236
R00-R09	9	1955	0.10204	0.189	0.0585	impact	0.40741	########
R00-R09	10	1915	0.11344	0.344	0.0891	incidence	-0.11833	########
R00-R09	10	1915	0.11344	0.344	0.0891	impact	0.22567	########
R00-R09	10	1925	0.11344	0.344	0.0891	incidence	0.04859	########
R00-R09	10	1925	0.11344	0.344	0.0891	impact	0.39259	########
R00-R09	10	1935	0.11344	0.344	0.0891	incidence	0.24401	########
R00-R09	10	1935	0.11344	0.344	0.0891	impact	0.58801	########
R00-R09	10	1945	0.11344	0.344	0.0891	incidence	0.295551	########
R00-R09	10	1945	0.11344	0.344	0.0891	impact	0.639551	########
R00-R09	10	1955	0.11344	0.344	0.0891	incidence	0.223149	0.0219
R00-R09	10	1955	0.11344	0.344	0.0891	impact	0.567149	########
C30-C39	1	1915	0.04823	0.218	0.226	incidence	-0.36311	########
C30-C39	1	1915	0.04823	0.218	0.226	impact	-0.14511	########
C30-C39	1	1925	0.04823	0.218	0.226	incidence	0.27449	########
C30-C39	1	1925	0.04823	0.218	0.226	impact	0.49249	########
C30-C39	1	1935	0.04823	0.218	0.226	incidence	0.25447	########
C30-C39	1	1935	0.04823	0.218	0.226	impact	0.47247	########
C30-C39	1	1945	0.04823	0.218	0.226	incidence	0.473859	########
C30-C39	1	1945	0.04823	0.218	0.226	impact	0.691859	########
C30-C39	1	1955	0.04823	0.218	0.226	incidence	0.226781	0.1312
C30-C39	1	1955	0.04823	0.218	0.226	impact	0.444781	########
C30-C39	2	1915	0.05585	0.154	0.1857	incidence	-0.16222	########
C30-C39	2	1915	0.05585	0.154	0.1857	impact	-0.00822	########
C30-C39	2	1925	0.05585	0.154	0.1857	incidence	0.11578	########
C30-C39	2	1925	0.05585	0.154	0.1857	impact	0.26978	########
C30-C39	2	1935	0.05585	0.154	0.1857	incidence	0.36382	########
C30-C39	2	1935	0.05585	0.154	0.1857	impact	0.51782	########
C30-C39	2	1945	0.05585	0.154	0.1857	incidence	0.351074	########
C30-C39	2	1945	0.05585	0.154	0.1857	impact	0.505074	########
C30-C39	2	1955	0.05585	0.154	0.1857	incidence	0.454386	0.1103
C30-C39	2	1955	0.05585	0.154	0.1857	impact	0.608386	########
C30-C39	3	1915	0.0601	0.342	0.1827	incidence	-0.13592	########
C30-C39	3	1915	0.0601	0.342	0.1827	impact	0.20608	########
C30-C39	3	1925	0.0601	0.342	0.1827	incidence	0.20359	########
C30-C39	3	1925	0.0601	0.342	0.1827	impact	0.54559	########
C30-C39	3	1935	0.0601	0.342	0.1827	incidence	0.26622	########
C30-C39	3	1935	0.0601	0.342	0.1827	impact	0.60822	########
C30-C39	3	1945	0.0601	0.342	0.1827	incidence	0.52238	########
C30-C39	3	1945	0.0601	0.342	0.1827	impact	0.86438	########
C30-C39	3	1955	0.0601	0.342	0.1827	incidence	0.50519	0.1072
C30-C39	3	1955	0.0601	0.342	0.1827	impact	0.84719	########
C30-C39	4	1915	0.06682	0.387	0.2018	incidence	-0.36573	########
C30-C39	4	1915	0.06682	0.387	0.2018	impact	0.02127	########
C30-C39	4	1925	0.06682	0.387	0.2018	incidence	0.14385	########
C30-C39	4	1925	0.06682	0.387	0.2018	impact	0.53085	########
C30-C39	4	1935	0.06682	0.387	0.2018	incidence	0.36827	########
C30-C39	4	1935	0.06682	0.387	0.2018	impact	0.75527	########
C30-C39	4	1945	0.06682	0.387	0.2018	incidence	0.405984	########
C30-C39	4	1945	0.06682	0.387	0.2018	impact	0.792984	########
C30-C39	4	1955	0.06682	0.387	0.2018	incidence	0.772006	0.1048
C30-C39	4	1955	0.06682	0.387	0.2018	impact	1.159006	########
C30-C39	5	1915	0.07719	0.116	0.1521	incidence	0.15615	########
C30-C39	5	1915	0.07719	0.116	0.1521	impact	0.27215	########
C30-C39	5	1925	0.07719	0.116	0.1521	incidence	0.17237	########

For peer review only

C30-C39	5	1925	0.07719	0.116	0.1521	impact	0.28837	########
C30-C39	5	1935	0.07719	0.116	0.1521	incidence	0.30958	########
C30-C39	5	1935	0.07719	0.116	0.1521	impact	0.42558	########
C30-C39	5	1945	0.07719	0.116	0.1521	incidence	0.369006	########
C30-C39	5	1945	0.07719	0.116	0.1521	impact	0.485006	########
C30-C39	5	1955	0.07719	0.116	0.1521	incidence	0.455934	0.0961
C30-C39	5	1955	0.07719	0.116	0.1521	impact	0.571934	########
C30-C39	6	1915	0.08462	0.339	0.1115	incidence	-0.37247	########
C30-C39	6	1915	0.08462	0.339	0.1115	impact	-0.03347	########
C30-C39	6	1925	0.08462	0.339	0.1115	incidence	0.07421	########
C30-C39	6	1925	0.08462	0.339	0.1115	impact	0.41321	########
C30-C39	6	1935	0.08462	0.339	0.1115	incidence	0.33879	########
C30-C39	6	1935	0.08462	0.339	0.1115	impact	0.67779	########
C30-C39	6	1945	0.08462	0.339	0.1115	incidence	0.400848	########
C30-C39	6	1945	0.08462	0.339	0.1115	impact	0.739848	########
C30-C39	6	1955	0.08462	0.339	0.1115	incidence	0.611222	0.0952
C30-C39	6	1955	0.08462	0.339	0.1115	impact	0.950222	########
C30-C39	7	1915	0.09461	-0.11	0.1279	incidence	-0.37855	########
C30-C39	7	1915	0.09461	-0.11	0.1279	impact	-0.48855	########
C30-C39	7	1925	0.09461	-0.11	0.1279	incidence	0.13657	########
C30-C39	7	1925	0.09461	-0.11	0.1279	impact	0.02657	########
C30-C39	7	1935	0.09461	-0.11	0.1279	incidence	0.27056	########
C30-C39	7	1935	0.09461	-0.11	0.1279	impact	0.16056	########
C30-C39	7	1945	0.09461	-0.11	0.1279	incidence	0.391158	########
C30-C39	7	1945	0.09461	-0.11	0.1279	impact	0.281158	########
C30-C39	7	1955	0.09461	-0.11	0.1279	incidence	0.426712	0.0857
C30-C39	7	1955	0.09461	-0.11	0.1279	impact	0.316712	########
C30-C39	8	1915	0.10066	0.224	0.1223	incidence	-0.03347	########
C30-C39	8	1915	0.10066	0.224	0.1223	impact	0.19053	########
C30-C39	8	1925	0.10066	0.224	0.1223	incidence	0.1025	########
C30-C39	8	1925	0.10066	0.224	0.1223	impact	0.3265	########
C30-C39	8	1935	0.10066	0.224	0.1223	incidence	0.29439	########
C30-C39	8	1935	0.10066	0.224	0.1223	impact	0.51839	########
C30-C39	8	1945	0.10066	0.224	0.1223	incidence	0.395962	########
C30-C39	8	1945	0.10066	0.224	0.1223	impact	0.619962	########
C30-C39	8	1955	0.10066	0.224	0.1223	incidence	0.421288	0.0795
C30-C39	8	1955	0.10066	0.224	0.1223	impact	0.645288	########
C30-C39	9	1915	0.11459	0.26	0.1417	incidence	-0.52792	########
C30-C39	9	1915	0.11459	0.26	0.1417	impact	-0.26792	########
C30-C39	9	1925	0.11459	0.26	0.1417	incidence	0.10558	########
C30-C39	9	1925	0.11459	0.26	0.1417	impact	0.36558	########
C30-C39	9	1935	0.11459	0.26	0.1417	incidence	0.20351	########
C30-C39	9	1935	0.11459	0.26	0.1417	impact	0.46351	########
C30-C39	9	1945	0.11459	0.26	0.1417	incidence	0.294619	########
C30-C39	9	1945	0.11459	0.26	0.1417	impact	0.554619	########
C30-C39	9	1955	0.11459	0.26	0.1417	incidence	0.420801	0.0711
C30-C39	9	1955	0.11459	0.26	0.1417	impact	0.680801	########
C30-C39	10	1915	0.13172	0.189	0.1183	incidence	-0.3745	########
C30-C39	10	1915	0.13172	0.189	0.1183	impact	-0.1855	########
C30-C39	10	1925	0.13172	0.189	0.1183	incidence	-0.05072	########
C30-C39	10	1925	0.13172	0.189	0.1183	impact	0.13828	########
C30-C39	10	1935	0.13172	0.189	0.1183	incidence	0.18749	########
C30-C39	10	1935	0.13172	0.189	0.1183	impact	0.37649	########
C30-C39	10	1945	0.13172	0.189	0.1183	incidence	0.268954	########
C30-C39	10	1945	0.13172	0.189	0.1183	impact	0.457954	########
C30-C39	10	1955	0.13172	0.189	0.1183	incidence	0.414586	0.0618

For peer review only

C30-C39	10	1955	0.13172	0.189	0.1183	impact	0.603586	########
R10-R19	1	1915	0.06568	0.327	0.1147	incidence	0.20386	########
R10-R19	1	1915	0.06568	0.327	0.1147	impact	0.53086	########
R10-R19	1	1925	0.06568	0.327	0.1147	incidence	0.15831	########
R10-R19	1	1925	0.06568	0.327	0.1147	impact	0.48531	########
R10-R19	1	1935	0.06568	0.327	0.1147	incidence	0.09563	########
R10-R19	1	1935	0.06568	0.327	0.1147	impact	0.42263	########
R10-R19	1	1945	0.06568	0.327	0.1147	incidence	0.120298	########
R10-R19	1	1945	0.06568	0.327	0.1147	impact	0.447298	########
R10-R19	1	1955	0.06568	0.327	0.1147	incidence	-0.050158	0.038
R10-R19	1	1955	0.06568	0.327	0.1147	impact	0.276842	########
R10-R19	2	1915	0.07283	-0.0707	0.0794	incidence	0.22686	########
R10-R19	2	1915	0.07283	-0.0707	0.0794	impact	0.15616	########
R10-R19	2	1925	0.07283	-0.0707	0.0794	incidence	0.17429	########
R10-R19	2	1925	0.07283	-0.0707	0.0794	impact	0.10359	########
R10-R19	2	1935	0.07283	-0.0707	0.0794	incidence	0.10994	########
R10-R19	2	1935	0.07283	-0.0707	0.0794	impact	0.03924	########
R10-R19	2	1945	0.07283	-0.0707	0.0794	incidence	0.118018	########
R10-R19	2	1945	0.07283	-0.0707	0.0794	impact	0.047318	########
R10-R19	2	1955	0.07283	-0.0707	0.0794	incidence	0.016632	0.0337
R10-R19	2	1955	0.07283	-0.0707	0.0794	impact	-0.054068	########
R10-R19	3	1915	0.07177	-0.0812	0.1762	incidence	0.09969	########
R10-R19	3	1915	0.07177	-0.0812	0.1762	impact	0.01849	########
R10-R19	3	1925	0.07177	-0.0812	0.1762	incidence	0.17384	########
R10-R19	3	1925	0.07177	-0.0812	0.1762	impact	0.09264	########
R10-R19	3	1935	0.07177	-0.0812	0.1762	incidence	0.19065	########
R10-R19	3	1935	0.07177	-0.0812	0.1762	impact	0.10945	########
R10-R19	3	1945	0.07177	-0.0812	0.1762	incidence	0.137405	########
R10-R19	3	1945	0.07177	-0.0812	0.1762	impact	0.056205	########
R10-R19	3	1955	0.07177	-0.0812	0.1762	incidence	0.081915	0.0341
R10-R19	3	1955	0.07177	-0.0812	0.1762	impact	0.000715	########
R10-R19	4	1915	0.07134	0.171	0.0898	incidence	0.18718	########
R10-R19	4	1915	0.07134	0.171	0.0898	impact	0.35818	########
R10-R19	4	1925	0.07134	0.171	0.0898	incidence	0.20175	########
R10-R19	4	1925	0.07134	0.171	0.0898	impact	0.37275	########
R10-R19	4	1935	0.07134	0.171	0.0898	incidence	0.2461	########
R10-R19	4	1935	0.07134	0.171	0.0898	impact	0.4171	########
R10-R19	4	1945	0.07134	0.171	0.0898	incidence	0.144978	########
R10-R19	4	1945	0.07134	0.171	0.0898	impact	0.315978	########
R10-R19	4	1955	0.07134	0.171	0.0898	incidence	0.054972	0.0344
R10-R19	4	1955	0.07134	0.171	0.0898	impact	0.225972	########
R10-R19	5	1915	0.07757	0.0212	0.0662	incidence	0.13867	########
R10-R19	5	1915	0.07757	0.0212	0.0662	impact	0.15987	########
R10-R19	5	1925	0.07757	0.0212	0.0662	incidence	0.09192	########
R10-R19	5	1925	0.07757	0.0212	0.0662	impact	0.11312	########
R10-R19	5	1935	0.07757	0.0212	0.0662	incidence	0.21039	########
R10-R19	5	1935	0.07757	0.0212	0.0662	impact	0.23159	########
R10-R19	5	1945	0.07757	0.0212	0.0662	incidence	0.195739	########
R10-R19	5	1945	0.07757	0.0212	0.0662	impact	0.216939	########
R10-R19	5	1955	0.07757	0.0212	0.0662	incidence	0.050801	0.0326
R10-R19	5	1955	0.07757	0.0212	0.0662	impact	0.072001	########
R10-R19	6	1915	0.08707	0.152	0.1132	incidence	0.30937	########
R10-R19	6	1915	0.08707	0.152	0.1132	impact	0.46137	########
R10-R19	6	1925	0.08707	0.152	0.1132	incidence	0.2395	########
R10-R19	6	1925	0.08707	0.152	0.1132	impact	0.3915	########
R10-R19	6	1935	0.08707	0.152	0.1132	incidence	0.20023	########

For peer review only

R10-R19	6	1935	0.08707	0.152	0.1132	impact	0.35223	########
R10-R19	6	1945	0.08707	0.152	0.1132	incidence	0.160791	########
R10-R19	6	1945	0.08707	0.152	0.1132	impact	0.312791	########
R10-R19	6	1955	0.08707	0.152	0.1132	incidence	0.085889	0.031
R10-R19	6	1955	0.08707	0.152	0.1132	impact	0.237889	########
R10-R19	7	1915	0.08726	0.148	0.0699	incidence	0.19817	########
R10-R19	7	1915	0.08726	0.148	0.0699	impact	0.34617	########
R10-R19	7	1925	0.08726	0.148	0.0699	incidence	0.17451	########
R10-R19	7	1925	0.08726	0.148	0.0699	impact	0.32251	########
R10-R19	7	1935	0.08726	0.148	0.0699	incidence	0.17351	########
R10-R19	7	1935	0.08726	0.148	0.0699	impact	0.32151	########
R10-R19	7	1945	0.08726	0.148	0.0699	incidence	0.127333	########
R10-R19	7	1945	0.08726	0.148	0.0699	impact	0.275333	########
R10-R19	7	1955	0.08726	0.148	0.0699	incidence	0.077947	0.0301
R10-R19	7	1955	0.08726	0.148	0.0699	impact	0.225947	########
R10-R19	8	1915	0.09335	0.158	0.1092	incidence	0.16015	########
R10-R19	8	1915	0.09335	0.158	0.1092	impact	0.31815	########
R10-R19	8	1925	0.09335	0.158	0.1092	incidence	0.23288	########
R10-R19	8	1925	0.09335	0.158	0.1092	impact	0.39088	########
R10-R19	8	1935	0.09335	0.158	0.1092	incidence	0.20888	########
R10-R19	8	1935	0.09335	0.158	0.1092	impact	0.36688	########
R10-R19	8	1945	0.09335	0.158	0.1092	incidence	0.129992	########
R10-R19	8	1945	0.09335	0.158	0.1092	impact	0.287992	########
R10-R19	8	1955	0.09335	0.158	0.1092	incidence	0.081818	0.0287
R10-R19	8	1955	0.09335	0.158	0.1092	impact	0.239818	########
R10-R19	9	1915	0.09705	0.0947	0.1415	incidence	-0.00856	########
R10-R19	9	1915	0.09705	0.0947	0.1415	impact	0.08614	########
R10-R19	9	1925	0.09705	0.0947	0.1415	incidence	0.14075	########
R10-R19	9	1925	0.09705	0.0947	0.1415	impact	0.23545	########
R10-R19	9	1935	0.09705	0.0947	0.1415	incidence	0.17524	########
R10-R19	9	1935	0.09705	0.0947	0.1415	impact	0.26994	########
R10-R19	9	1945	0.09705	0.0947	0.1415	incidence	0.129073	########
R10-R19	9	1945	0.09705	0.0947	0.1415	impact	0.223773	########
R10-R19	9	1955	0.09705	0.0947	0.1415	incidence	0.086667	0.0277
R10-R19	9	1955	0.09705	0.0947	0.1415	impact	0.181367	########
R10-R19	10	1915	0.10533	0.163	0.0842	incidence	0.11311	########
R10-R19	10	1915	0.10533	0.163	0.0842	impact	0.27611	########
R10-R19	10	1925	0.10533	0.163	0.0842	incidence	0.1499	########
R10-R19	10	1925	0.10533	0.163	0.0842	impact	0.3129	########
R10-R19	10	1935	0.10533	0.163	0.0842	incidence	0.13599	########
R10-R19	10	1935	0.10533	0.163	0.0842	impact	0.29899	########
R10-R19	10	1945	0.10533	0.163	0.0842	incidence	0.165848	########
R10-R19	10	1945	0.10533	0.163	0.0842	impact	0.328848	########
R10-R19	10	1955	0.10533	0.163	0.0842	incidence	0.094942	0.0259
R10-R19	10	1955	0.10533	0.163	0.0842	impact	0.257942	########
R50-R69	1	1915	0.05948	0.0606	0.1279	incidence	0.0698	########
R50-R69	1	1915	0.05948	0.0606	0.1279	impact	0.1304	########
R50-R69	1	1925	0.05948	0.0606	0.1279	incidence	0.33378	########
R50-R69	1	1925	0.05948	0.0606	0.1279	impact	0.39438	########
R50-R69	1	1935	0.05948	0.0606	0.1279	incidence	0.16848	########
R50-R69	1	1935	0.05948	0.0606	0.1279	impact	0.22908	########
R50-R69	1	1945	0.05948	0.0606	0.1279	incidence	0.032505	########
R50-R69	1	1945	0.05948	0.0606	0.1279	impact	0.093105	########
R50-R69	1	1955	0.05948	0.0606	0.1279	incidence	-0.078705	0.0543
R50-R69	1	1955	0.05948	0.0606	0.1279	impact	-0.018105	########
R50-R69	2	1915	0.06175	-0.126	0.1108	incidence	0.09048	########

For peer review only

R50-R69	2	1915	0.06175	-0.126	0.1108	impact	-0.03552	########
R50-R69	2	1925	0.06175	-0.126	0.1108	incidence	0.24078	########
R50-R69	2	1925	0.06175	-0.126	0.1108	impact	0.11478	########
R50-R69	2	1935	0.06175	-0.126	0.1108	incidence	0.15178	########
R50-R69	2	1935	0.06175	-0.126	0.1108	impact	0.02578	########
R50-R69	2	1945	0.06175	-0.126	0.1108	incidence	0.097603	########
R50-R69	2	1945	0.06175	-0.126	0.1108	impact	-0.028397	########
R50-R69	2	1955	0.06175	-0.126	0.1108	incidence	-0.070783	0.0492
R50-R69	2	1955	0.06175	-0.126	0.1108	impact	-0.196783	########
R50-R69	3	1915	0.06402	-0.158	0.0825	incidence	-0.02635	########
R50-R69	3	1915	0.06402	-0.158	0.0825	impact	-0.18435	########
R50-R69	3	1925	0.06402	-0.158	0.0825	incidence	0.26404	########
R50-R69	3	1925	0.06402	-0.158	0.0825	impact	0.10604	########
R50-R69	3	1935	0.06402	-0.158	0.0825	incidence	0.17384	########
R50-R69	3	1935	0.06402	-0.158	0.0825	impact	0.01584	########
R50-R69	3	1945	0.06402	-0.158	0.0825	incidence	0.023281	########
R50-R69	3	1945	0.06402	-0.158	0.0825	impact	-0.134719	########
R50-R69	3	1955	0.06402	-0.158	0.0825	incidence	-0.024391	0.0484
R50-R69	3	1955	0.06402	-0.158	0.0825	impact	-0.182391	########
R50-R69	4	1915	0.06989	-0.156	0.0743	incidence	-0.02477	########
R50-R69	4	1915	0.06989	-0.156	0.0743	impact	-0.18077	########
R50-R69	4	1925	0.06989	-0.156	0.0743	incidence	0.26841	########
R50-R69	4	1925	0.06989	-0.156	0.0743	impact	0.11241	########
R50-R69	4	1935	0.06989	-0.156	0.0743	incidence	0.2769	########
R50-R69	4	1935	0.06989	-0.156	0.0743	impact	0.1209	########
R50-R69	4	1945	0.06989	-0.156	0.0743	incidence	0.184001	########
R50-R69	4	1945	0.06989	-0.156	0.0743	impact	0.028001	########
R50-R69	4	1955	0.06989	-0.156	0.0743	incidence	0.023429	0.046
R50-R69	4	1955	0.06989	-0.156	0.0743	impact	-0.132571	########
R50-R69	5	1915	0.0726	-0.112	0.0409	incidence	-0.04379	########
R50-R69	5	1915	0.0726	-0.112	0.0409	impact	-0.15579	########
R50-R69	5	1925	0.0726	-0.112	0.0409	incidence	0.15768	########
R50-R69	5	1925	0.0726	-0.112	0.0409	impact	0.04568	########
R50-R69	5	1935	0.0726	-0.112	0.0409	incidence	0.23468	########
R50-R69	5	1935	0.0726	-0.112	0.0409	impact	0.12268	########
R50-R69	5	1945	0.0726	-0.112	0.0409	incidence	0.179245	########
R50-R69	5	1945	0.0726	-0.112	0.0409	impact	0.067245	########
R50-R69	5	1955	0.0726	-0.112	0.0409	incidence	-0.008065	0.0451
R50-R69	5	1955	0.0726	-0.112	0.0409	impact	-0.120065	########
R50-R69	6	1915	0.08086	-0.0859	0.074	incidence	-0.01488	########
R50-R69	6	1915	0.08086	-0.0859	0.074	impact	-0.10078	########
R50-R69	6	1925	0.08086	-0.0859	0.074	incidence	0.18986	########
R50-R69	6	1925	0.08086	-0.0859	0.074	impact	0.10396	########
R50-R69	6	1935	0.08086	-0.0859	0.074	incidence	0.24522	########
R50-R69	6	1935	0.08086	-0.0859	0.074	impact	0.15932	########
R50-R69	6	1945	0.08086	-0.0859	0.074	incidence	0.099416	########
R50-R69	6	1945	0.08086	-0.0859	0.074	impact	0.013516	########
R50-R69	6	1955	0.08086	-0.0859	0.074	incidence	0.050334	0.0429
R50-R69	6	1955	0.08086	-0.0859	0.074	impact	-0.035566	########
R50-R69	7	1915	0.08435	0.219	0.05	incidence	-0.04133	########
R50-R69	7	1915	0.08435	0.219	0.05	impact	0.17767	########
R50-R69	7	1925	0.08435	0.219	0.05	incidence	0.20765	########
R50-R69	7	1925	0.08435	0.219	0.05	impact	0.42665	########
R50-R69	7	1935	0.08435	0.219	0.05	incidence	0.19958	########
R50-R69	7	1935	0.08435	0.219	0.05	impact	0.41858	########
R50-R69	7	1945	0.08435	0.219	0.05	incidence	0.158688	########

For peer review only

R50-R69	7	1945	0.08435	0.219	0.05	impact	0.377688	########
R50-R69	7	1955	0.08435	0.219	0.05	incidence	0.078412	0.0415
R50-R69	7	1955	0.08435	0.219	0.05	impact	0.297412	########
R50-R69	8	1915	0.09114	0.143	0.0592	incidence	0.07823	########
R50-R69	8	1915	0.09114	0.143	0.0592	impact	0.22123	########
R50-R69	8	1925	0.09114	0.143	0.0592	incidence	0.20086	########
R50-R69	8	1925	0.09114	0.143	0.0592	impact	0.34386	########
R50-R69	8	1935	0.09114	0.143	0.0592	incidence	0.22708	########
R50-R69	8	1935	0.09114	0.143	0.0592	impact	0.37008	########
R50-R69	8	1945	0.09114	0.143	0.0592	incidence	0.185102	########
R50-R69	8	1945	0.09114	0.143	0.0592	impact	0.328102	########
R50-R69	8	1955	0.09114	0.143	0.0592	incidence	0.018028	0.0382
R50-R69	8	1955	0.09114	0.143	0.0592	impact	0.161028	########
R50-R69	9	1915	0.09458	-0.00997	0.0592	incidence	0.00186	########
R50-R69	9	1915	0.09458	-0.00997	0.0592	impact	-0.00811	########
R50-R69	9	1925	0.09458	-0.00997	0.0592	incidence	0.1654	########
R50-R69	9	1925	0.09458	-0.00997	0.0592	impact	0.15543	########
R50-R69	9	1935	0.09458	-0.00997	0.0592	incidence	0.16923	########
R50-R69	9	1935	0.09458	-0.00997	0.0592	impact	0.15926	########
R50-R69	9	1945	0.09458	-0.00997	0.0592	incidence	0.127822	########
R50-R69	9	1945	0.09458	-0.00997	0.0592	impact	0.117852	########
R50-R69	9	1955	0.09458	-0.00997	0.0592	incidence	0.034698	0.0364
R50-R69	9	1955	0.09458	-0.00997	0.0592	impact	0.024728	########
R50-R69	10	1915	0.10377	0.229	0.0593	incidence	0.03379	########
R50-R69	10	1915	0.10377	0.229	0.0593	impact	0.26279	########
R50-R69	10	1925	0.10377	0.229	0.0593	incidence	0.06358	########
R50-R69	10	1925	0.10377	0.229	0.0593	impact	0.29258	########
R50-R69	10	1935	0.10377	0.229	0.0593	incidence	0.12174	########
R50-R69	10	1935	0.10377	0.229	0.0593	impact	0.35074	########
R50-R69	10	1945	0.10377	0.229	0.0593	incidence	0.104863	########
R50-R69	10	1945	0.10377	0.229	0.0593	impact	0.333863	########
R50-R69	10	1955	0.10377	0.229	0.0593	incidence	0.050077	0.0329
R50-R69	10	1955	0.10377	0.229	0.0593	impact	0.279077	########
C15-C26	1	1915	0.07182	0.503	0.0999	incidence	0.07774	########
C15-C26	1	1915	0.07182	0.503	0.0999	impact	0.58074	########
C15-C26	1	1925	0.07182	0.503	0.0999	incidence	0.25467	########
C15-C26	1	1925	0.07182	0.503	0.0999	impact	0.75767	########
C15-C26	1	1935	0.07182	0.503	0.0999	incidence	0.23355	########
C15-C26	1	1935	0.07182	0.503	0.0999	impact	0.73655	########
C15-C26	1	1945	0.07182	0.503	0.0999	incidence	0.400112	########
C15-C26	1	1945	0.07182	0.503	0.0999	impact	0.903112	########
C15-C26	1	1955	0.07182	0.503	0.0999	incidence	0.242328	0.0794
C15-C26	1	1955	0.07182	0.503	0.0999	impact	0.745328	########
C15-C26	2	1915	0.07297	0.504	0.1334	incidence	0.13706	########
C15-C26	2	1915	0.07297	0.504	0.1334	impact	0.64106	########
C15-C26	2	1925	0.07297	0.504	0.1334	incidence	0.20281	########
C15-C26	2	1925	0.07297	0.504	0.1334	impact	0.70681	########
C15-C26	2	1935	0.07297	0.504	0.1334	incidence	0.32935	########
C15-C26	2	1935	0.07297	0.504	0.1334	impact	0.83335	########
C15-C26	2	1945	0.07297	0.504	0.1334	incidence	0.360394	########
C15-C26	2	1945	0.07297	0.504	0.1334	impact	0.864394	########
C15-C26	2	1955	0.07297	0.504	0.1334	incidence	0.269166	0.0761
C15-C26	2	1955	0.07297	0.504	0.1334	impact	0.773166	########
C15-C26	3	1915	0.0707	0.448	0.1607	incidence	0.02371	########
C15-C26	3	1915	0.0707	0.448	0.1607	impact	0.47171	########
C15-C26	3	1925	0.0707	0.448	0.1607	incidence	0.20086	########

For peer review only

C15-C26	3	1925	0.0707	0.448	0.1607	impact	0.64886	########
C15-C26	3	1935	0.0707	0.448	0.1607	incidence	0.32865	########
C15-C26	3	1935	0.0707	0.448	0.1607	impact	0.77665	########
C15-C26	3	1945	0.0707	0.448	0.1607	incidence	0.292453	########
C15-C26	3	1945	0.0707	0.448	0.1607	impact	0.740453	########
C15-C26	3	1955	0.0707	0.448	0.1607	incidence	0.392807	0.0772
C15-C26	3	1955	0.0707	0.448	0.1607	impact	0.840807	########
C15-C26	4	1915	0.07253	0.446	0.0749	incidence	0.07148	########
C15-C26	4	1915	0.07253	0.446	0.0749	impact	0.51748	########
C15-C26	4	1925	0.07253	0.446	0.0749	incidence	0.18377	########
C15-C26	4	1925	0.07253	0.446	0.0749	impact	0.62977	########
C15-C26	4	1935	0.07253	0.446	0.0749	incidence	0.19018	########
C15-C26	4	1935	0.07253	0.446	0.0749	impact	0.63618	########
C15-C26	4	1945	0.07253	0.446	0.0749	incidence	0.354925	########
C15-C26	4	1945	0.07253	0.446	0.0749	impact	0.800925	########
C15-C26	4	1955	0.07253	0.446	0.0749	incidence	0.297015	0.0758
C15-C26	4	1955	0.07253	0.446	0.0749	impact	0.743015	########
C15-C26	5	1915	0.07538	0.535	0.1503	incidence	0.19278	########
C15-C26	5	1915	0.07538	0.535	0.1503	impact	0.72778	########
C15-C26	5	1925	0.07538	0.535	0.1503	incidence	0.13871	########
C15-C26	5	1925	0.07538	0.535	0.1503	impact	0.67371	########
C15-C26	5	1935	0.07538	0.535	0.1503	incidence	0.35098	########
C15-C26	5	1935	0.07538	0.535	0.1503	impact	0.88598	########
C15-C26	5	1945	0.07538	0.535	0.1503	incidence	0.269528	########
C15-C26	5	1945	0.07538	0.535	0.1503	impact	0.804528	########
C15-C26	5	1955	0.07538	0.535	0.1503	incidence	0.398452	0.0746
C15-C26	5	1955	0.07538	0.535	0.1503	impact	0.933452	########
C15-C26	6	1915	0.07905	0.259	0.0994	incidence	-0.16212	########
C15-C26	6	1915	0.07905	0.259	0.0994	impact	0.09688	########
C15-C26	6	1925	0.07905	0.259	0.0994	incidence	0.1963	########
C15-C26	6	1925	0.07905	0.259	0.0994	impact	0.4553	########
C15-C26	6	1935	0.07905	0.259	0.0994	incidence	0.26634	########
C15-C26	6	1935	0.07905	0.259	0.0994	impact	0.52534	########
C15-C26	6	1945	0.07905	0.259	0.0994	incidence	0.352256	########
C15-C26	6	1945	0.07905	0.259	0.0994	impact	0.611256	########
C15-C26	6	1955	0.07905	0.259	0.0994	incidence	0.244964	0.073
C15-C26	6	1955	0.07905	0.259	0.0994	impact	0.503964	########
C15-C26	7	1915	0.08115	0.215	0.1191	incidence	0.09167	########
C15-C26	7	1915	0.08115	0.215	0.1191	impact	0.30667	########
C15-C26	7	1925	0.08115	0.215	0.1191	incidence	0.23726	########
C15-C26	7	1925	0.08115	0.215	0.1191	impact	0.45226	########
C15-C26	7	1935	0.08115	0.215	0.1191	incidence	0.19947	########
C15-C26	7	1935	0.08115	0.215	0.1191	impact	0.41447	########
C15-C26	7	1945	0.08115	0.215	0.1191	incidence	0.292953	########
C15-C26	7	1945	0.08115	0.215	0.1191	impact	0.507953	########
C15-C26	7	1955	0.08115	0.215	0.1191	incidence	0.262277	0.0693
C15-C26	7	1955	0.08115	0.215	0.1191	impact	0.477277	########
C15-C26	8	1915	0.08203	0.294	0.1213	incidence	0.29319	########
C15-C26	8	1915	0.08203	0.294	0.1213	impact	0.58719	########
C15-C26	8	1925	0.08203	0.294	0.1213	incidence	0.27641	########
C15-C26	8	1925	0.08203	0.294	0.1213	impact	0.57041	########
C15-C26	8	1935	0.08203	0.294	0.1213	incidence	0.26739	########
C15-C26	8	1935	0.08203	0.294	0.1213	impact	0.56139	########
C15-C26	8	1945	0.08203	0.294	0.1213	incidence	0.309029	########
C15-C26	8	1945	0.08203	0.294	0.1213	impact	0.603029	########
C15-C26	8	1955	0.08203	0.294	0.1213	incidence	0.270641	0.0689

For peer review only

C15-C26	8	1955	0.08203	0.294	0.1213	impact	0.564641	########
C15-C26	9	1915	0.0805	0.293	0.1242	incidence	0.04861	########
C15-C26	9	1915	0.0805	0.293	0.1242	impact	0.34161	########
C15-C26	9	1925	0.0805	0.293	0.1242	incidence	0.15249	########
C15-C26	9	1925	0.0805	0.293	0.1242	impact	0.44549	########
C15-C26	9	1935	0.0805	0.293	0.1242	incidence	0.30791	########
C15-C26	9	1935	0.0805	0.293	0.1242	impact	0.60091	########
C15-C26	9	1945	0.0805	0.293	0.1242	incidence	0.294787	########
C15-C26	9	1945	0.0805	0.293	0.1242	impact	0.587787	########
C15-C26	9	1955	0.0805	0.293	0.1242	incidence	0.223573	0.0691
C15-C26	9	1955	0.0805	0.293	0.1242	impact	0.516573	########
C15-C26	10	1915	0.08054	0.486	0.1388	incidence	0.10824	########
C15-C26	10	1915	0.08054	0.486	0.1388	impact	0.59424	########
C15-C26	10	1925	0.08054	0.486	0.1388	incidence	0.11775	########
C15-C26	10	1925	0.08054	0.486	0.1388	impact	0.60375	########
C15-C26	10	1935	0.08054	0.486	0.1388	incidence	0.21195	########
C15-C26	10	1935	0.08054	0.486	0.1388	impact	0.69795	########
C15-C26	10	1945	0.08054	0.486	0.1388	incidence	0.393053	########
C15-C26	10	1945	0.08054	0.486	0.1388	impact	0.879053	########
C15-C26	10	1955	0.08054	0.486	0.1388	incidence	0.298027	0.0667
C15-C26	10	1955	0.08054	0.486	0.1388	impact	0.784027	########
I30-I52	1	1915	0.05901	0.385	0.1052	incidence	0.0742	########
I30-I52	1	1915	0.05901	0.385	0.1052	impact	0.4592	########
I30-I52	1	1925	0.05901	0.385	0.1052	incidence	0.28391	########
I30-I52	1	1925	0.05901	0.385	0.1052	impact	0.66891	########
I30-I52	1	1935	0.05901	0.385	0.1052	incidence	0.31469	########
I30-I52	1	1935	0.05901	0.385	0.1052	impact	0.69969	########
I30-I52	1	1945	0.05901	0.385	0.1052	incidence	0.188708	########
I30-I52	1	1945	0.05901	0.385	0.1052	impact	0.573708	########
I30-I52	1	1955	0.05901	0.385	0.1052	incidence	0.092032	0.0615
I30-I52	1	1955	0.05901	0.385	0.1052	impact	0.477032	########
I30-I52	2	1915	0.06281	0.204	0.0691	incidence	0.07017	########
I30-I52	2	1915	0.06281	0.204	0.0691	impact	0.27417	########
I30-I52	2	1925	0.06281	0.204	0.0691	incidence	0.28593	########
I30-I52	2	1925	0.06281	0.204	0.0691	impact	0.48993	########
I30-I52	2	1935	0.06281	0.204	0.0691	incidence	0.35049	########
I30-I52	2	1935	0.06281	0.204	0.0691	impact	0.55449	########
I30-I52	2	1945	0.06281	0.204	0.0691	incidence	0.277257	########
I30-I52	2	1945	0.06281	0.204	0.0691	impact	0.481257	########
I30-I52	2	1955	0.06281	0.204	0.0691	incidence	0.270313	0.0598
I30-I52	2	1955	0.06281	0.204	0.0691	impact	0.474313	########
I30-I52	3	1915	0.06088	-0.0376	0.1333	incidence	0.07614	########
I30-I52	3	1915	0.06088	-0.0376	0.1333	impact	0.03854	########
I30-I52	3	1925	0.06088	-0.0376	0.1333	incidence	0.29304	########
I30-I52	3	1925	0.06088	-0.0376	0.1333	impact	0.25544	########
I30-I52	3	1935	0.06088	-0.0376	0.1333	incidence	0.3234	########
I30-I52	3	1935	0.06088	-0.0376	0.1333	impact	0.2858	########
I30-I52	3	1945	0.06088	-0.0376	0.1333	incidence	0.30371	########
I30-I52	3	1945	0.06088	-0.0376	0.1333	impact	0.26611	########
I30-I52	3	1955	0.06088	-0.0376	0.1333	incidence	0.10156	0.0583
I30-I52	3	1955	0.06088	-0.0376	0.1333	impact	0.06396	########
I30-I52	4	1915	0.06727	0.284	0.1168	incidence	0.10138	########
I30-I52	4	1915	0.06727	0.284	0.1168	impact	0.38538	########
I30-I52	4	1925	0.06727	0.284	0.1168	incidence	0.27111	########
I30-I52	4	1925	0.06727	0.284	0.1168	impact	0.55511	########
I30-I52	4	1935	0.06727	0.284	0.1168	incidence	0.35227	########

For peer review only

I30-I52	4	1935	0.06727	0.284	0.1168	impact	0.63627	########
I30-I52	4	1945	0.06727	0.284	0.1168	incidence	0.218173	########
I30-I52	4	1945	0.06727	0.284	0.1168	impact	0.502173	########
I30-I52	4	1955	0.06727	0.284	0.1168	incidence	0.070537	0.0559
I30-I52	4	1955	0.06727	0.284	0.1168	impact	0.354537	########
I30-I52	5	1915	0.07252	0.23	0.0366	incidence	0.15943	########
I30-I52	5	1915	0.07252	0.23	0.0366	impact	0.38943	########
I30-I52	5	1925	0.07252	0.23	0.0366	incidence	0.2209	########
I30-I52	5	1925	0.07252	0.23	0.0366	impact	0.4509	########
I30-I52	5	1935	0.07252	0.23	0.0366	incidence	0.28811	########
I30-I52	5	1935	0.07252	0.23	0.0366	impact	0.51811	########
I30-I52	5	1945	0.07252	0.23	0.0366	incidence	0.226504	########
I30-I52	5	1945	0.07252	0.23	0.0366	impact	0.456504	########
I30-I52	5	1955	0.07252	0.23	0.0366	incidence	0.134706	0.0558
I30-I52	5	1955	0.07252	0.23	0.0366	impact	0.364706	########
I30-I52	6	1915	0.07431	0.253	0.0737	incidence	0.11339	########
I30-I52	6	1915	0.07431	0.253	0.0737	impact	0.36639	########
I30-I52	6	1925	0.07431	0.253	0.0737	incidence	0.29687	########
I30-I52	6	1925	0.07431	0.253	0.0737	impact	0.54987	########
I30-I52	6	1935	0.07431	0.253	0.0737	incidence	0.35698	########
I30-I52	6	1935	0.07431	0.253	0.0737	impact	0.60998	########
I30-I52	6	1945	0.07431	0.253	0.0737	incidence	0.323109	########
I30-I52	6	1945	0.07431	0.253	0.0737	impact	0.576109	########
I30-I52	6	1955	0.07431	0.253	0.0737	incidence	0.151221	0.0559
I30-I52	6	1955	0.07431	0.253	0.0737	impact	0.404221	########
I30-I52	7	1915	0.0814	0.264	0.0761	incidence	0.15529	########
I30-I52	7	1915	0.0814	0.264	0.0761	impact	0.41929	########
I30-I52	7	1925	0.0814	0.264	0.0761	incidence	0.31119	########
I30-I52	7	1925	0.0814	0.264	0.0761	impact	0.57519	########
I30-I52	7	1935	0.0814	0.264	0.0761	incidence	0.31878	########
I30-I52	7	1935	0.0814	0.264	0.0761	impact	0.58278	########
I30-I52	7	1945	0.0814	0.264	0.0761	incidence	0.382809	########
I30-I52	7	1945	0.0814	0.264	0.0761	impact	0.646809	########
I30-I52	7	1955	0.0814	0.264	0.0761	incidence	0.099671	0.0534
I30-I52	7	1955	0.0814	0.264	0.0761	impact	0.363671	########
I30-I52	8	1915	0.08139	0.116	0.0487	incidence	0.22278	########
I30-I52	8	1915	0.08139	0.116	0.0487	impact	0.33878	########
I30-I52	8	1925	0.08139	0.116	0.0487	incidence	0.27883	########
I30-I52	8	1925	0.08139	0.116	0.0487	impact	0.39483	########
I30-I52	8	1935	0.08139	0.116	0.0487	incidence	0.33754	########
I30-I52	8	1935	0.08139	0.116	0.0487	impact	0.45354	########
I30-I52	8	1945	0.08139	0.116	0.0487	incidence	0.329227	########
I30-I52	8	1945	0.08139	0.116	0.0487	impact	0.445227	########
I30-I52	8	1955	0.08139	0.116	0.0487	incidence	0.197893	0.0512
I30-I52	8	1955	0.08139	0.116	0.0487	impact	0.313893	########
I30-I52	9	1915	0.08033	0.0407	0.0909	incidence	0.15166	########
I30-I52	9	1915	0.08033	0.0407	0.0909	impact	0.19236	########
I30-I52	9	1925	0.08033	0.0407	0.0909	incidence	0.28231	########
I30-I52	9	1925	0.08033	0.0407	0.0909	impact	0.32301	########
I30-I52	9	1935	0.08033	0.0407	0.0909	incidence	0.31957	########
I30-I52	9	1935	0.08033	0.0407	0.0909	impact	0.36027	########
I30-I52	9	1945	0.08033	0.0407	0.0909	incidence	0.31993	########
I30-I52	9	1945	0.08033	0.0407	0.0909	impact	0.36063	########
I30-I52	9	1955	0.08033	0.0407	0.0909	incidence	0.27352	0.0514
I30-I52	9	1955	0.08033	0.0407	0.0909	impact	0.31422	########
I30-I52	10	1915	0.07841	0.072	0.0981	incidence	0.11357	########

For peer review only

I30-I52	10	1915	0.07841	0.072	0.0981	impact	0.18557	########
I30-I52	10	1925	0.07841	0.072	0.0981	incidence	0.27465	########
I30-I52	10	1925	0.07841	0.072	0.0981	impact	0.34665	########
I30-I52	10	1935	0.07841	0.072	0.0981	incidence	0.36052	########
I30-I52	10	1935	0.07841	0.072	0.0981	impact	0.43252	########
I30-I52	10	1945	0.07841	0.072	0.0981	incidence	0.370911	########
I30-I52	10	1945	0.07841	0.072	0.0981	impact	0.442911	########
I30-I52	10	1955	0.07841	0.072	0.0981	incidence	0.300299	0.0493
I30-I52	10	1955	0.07841	0.072	0.0981	impact	0.372299	########
C76-C80	1	1915	0.06351	0.353	0.1424	incidence	-0.16709	########
C76-C80	1	1915	0.06351	0.353	0.1424	impact	0.18591	########
C76-C80	1	1925	0.06351	0.353	0.1424	incidence	0.0336	########
C76-C80	1	1925	0.06351	0.353	0.1424	impact	0.3866	########
C76-C80	1	1935	0.06351	0.353	0.1424	incidence	0.14633	########
C76-C80	1	1935	0.06351	0.353	0.1424	impact	0.49933	########
C76-C80	1	1945	0.06351	0.353	0.1424	incidence	0.318316	########
C76-C80	1	1945	0.06351	0.353	0.1424	impact	0.671316	########
C76-C80	1	1955	0.06351	0.353	0.1424	incidence	-0.040836	0.0938
C76-C80	1	1955	0.06351	0.353	0.1424	impact	0.312164	########
C76-C80	2	1915	0.06043	0.062	0.2428	incidence	0.00567	########
C76-C80	2	1915	0.06043	0.062	0.2428	impact	0.06767	########
C76-C80	2	1925	0.06043	0.062	0.2428	incidence	0.06689	########
C76-C80	2	1925	0.06043	0.062	0.2428	impact	0.12889	########
C76-C80	2	1935	0.06043	0.062	0.2428	incidence	0.18554	########
C76-C80	2	1935	0.06043	0.062	0.2428	impact	0.24754	########
C76-C80	2	1945	0.06043	0.062	0.2428	incidence	0.194471	########
C76-C80	2	1945	0.06043	0.062	0.2428	impact	0.256471	########
C76-C80	2	1955	0.06043	0.062	0.2428	incidence	0.215009	0.089
C76-C80	2	1955	0.06043	0.062	0.2428	impact	0.277009	########
C76-C80	3	1915	0.06169	0.461	0.1858	incidence	-0.09045	########
C76-C80	3	1915	0.06169	0.461	0.1858	impact	0.37055	########
C76-C80	3	1925	0.06169	0.461	0.1858	incidence	0.13537	########
C76-C80	3	1925	0.06169	0.461	0.1858	impact	0.59637	########
C76-C80	3	1935	0.06169	0.461	0.1858	incidence	0.21269	########
C76-C80	3	1935	0.06169	0.461	0.1858	impact	0.67369	########
C76-C80	3	1945	0.06169	0.461	0.1858	incidence	0.186059	########
C76-C80	3	1945	0.06169	0.461	0.1858	impact	0.647059	########
C76-C80	3	1955	0.06169	0.461	0.1858	incidence	0.197771	0.0906
C76-C80	3	1955	0.06169	0.461	0.1858	impact	0.658771	########
C76-C80	4	1915	0.06455	0.429	0.2195	incidence	-0.16288	########
C76-C80	4	1915	0.06455	0.429	0.2195	impact	0.26612	########
C76-C80	4	1925	0.06455	0.429	0.2195	incidence	0.16065	########
C76-C80	4	1925	0.06455	0.429	0.2195	impact	0.58965	########
C76-C80	4	1935	0.06455	0.429	0.2195	incidence	0.18959	########
C76-C80	4	1935	0.06455	0.429	0.2195	impact	0.61859	########
C76-C80	4	1945	0.06455	0.429	0.2195	incidence	0.277776	########
C76-C80	4	1945	0.06455	0.429	0.2195	impact	0.706776	########
C76-C80	4	1955	0.06455	0.429	0.2195	incidence	0.493504	0.0913
C76-C80	4	1955	0.06455	0.429	0.2195	impact	0.922504	########
C76-C80	5	1915	0.06494	0.28	0.1804	incidence	0.0424	########
C76-C80	5	1915	0.06494	0.28	0.1804	impact	0.3224	########
C76-C80	5	1925	0.06494	0.28	0.1804	incidence	0.10603	########
C76-C80	5	1925	0.06494	0.28	0.1804	impact	0.38603	########
C76-C80	5	1935	0.06494	0.28	0.1804	incidence	0.25956	########
C76-C80	5	1935	0.06494	0.28	0.1804	impact	0.53956	########
C76-C80	5	1945	0.06494	0.28	0.1804	incidence	0.175451	########

For peer review only

C76-C80	5	1945	0.06494	0.28	0.1804	impact	0.455451	########
C76-C80	5	1955	0.06494	0.28	0.1804	incidence	0.229619	0.0882
C76-C80	5	1955	0.06494	0.28	0.1804	impact	0.509619	########
C76-C80	6	1915	0.06744	0.582	0.1907	incidence	0.34348	########
C76-C80	6	1915	0.06744	0.582	0.1907	impact	0.92548	########
C76-C80	6	1925	0.06744	0.582	0.1907	incidence	0.0557	########
C76-C80	6	1925	0.06744	0.582	0.1907	impact	0.6377	########
C76-C80	6	1935	0.06744	0.582	0.1907	incidence	0.24247	########
C76-C80	6	1935	0.06744	0.582	0.1907	impact	0.82447	########
C76-C80	6	1945	0.06744	0.582	0.1907	incidence	0.277423	########
C76-C80	6	1945	0.06744	0.582	0.1907	impact	0.859423	########
C76-C80	6	1955	0.06744	0.582	0.1907	incidence	0.190157	0.0865
C76-C80	6	1955	0.06744	0.582	0.1907	impact	0.772157	########
C76-C80	7	1915	0.06698	0.479	0.1987	incidence	-0.01655	########
C76-C80	7	1915	0.06698	0.479	0.1987	impact	0.46245	########
C76-C80	7	1925	0.06698	0.479	0.1987	incidence	0.10669	########
C76-C80	7	1925	0.06698	0.479	0.1987	impact	0.58569	########
C76-C80	7	1935	0.06698	0.479	0.1987	incidence	0.19728	########
C76-C80	7	1935	0.06698	0.479	0.1987	impact	0.67628	########
C76-C80	7	1945	0.06698	0.479	0.1987	incidence	0.298944	########
C76-C80	7	1945	0.06698	0.479	0.1987	impact	0.777944	########
C76-C80	7	1955	0.06698	0.479	0.1987	incidence	0.159326	0.0846
C76-C80	7	1955	0.06698	0.479	0.1987	impact	0.638326	########
C76-C80	8	1915	0.06417	0.518	0.181	incidence	0.0111	########
C76-C80	8	1915	0.06417	0.518	0.181	impact	0.5291	########
C76-C80	8	1925	0.06417	0.518	0.181	incidence	0.18136	########
C76-C80	8	1925	0.06417	0.518	0.181	impact	0.69936	########
C76-C80	8	1935	0.06417	0.518	0.181	incidence	0.1664	########
C76-C80	8	1935	0.06417	0.518	0.181	impact	0.6844	########
C76-C80	8	1945	0.06417	0.518	0.181	incidence	0.273645	########
C76-C80	8	1945	0.06417	0.518	0.181	impact	0.791645	########
C76-C80	8	1955	0.06417	0.518	0.181	incidence	0.302835	0.0878
C76-C80	8	1955	0.06417	0.518	0.181	impact	0.820835	########
C76-C80	9	1915	0.07071	0.411	0.1457	incidence	0.21164	########
C76-C80	9	1915	0.07071	0.411	0.1457	impact	0.62264	########
C76-C80	9	1925	0.07071	0.411	0.1457	incidence	0.23906	########
C76-C80	9	1925	0.07071	0.411	0.1457	impact	0.65006	########
C76-C80	9	1935	0.07071	0.411	0.1457	incidence	0.21144	########
C76-C80	9	1935	0.07071	0.411	0.1457	impact	0.62244	########
C76-C80	9	1945	0.07071	0.411	0.1457	incidence	0.250318	########
C76-C80	9	1945	0.07071	0.411	0.1457	impact	0.661318	########
C76-C80	9	1955	0.07071	0.411	0.1457	incidence	0.341642	0.082
C76-C80	9	1955	0.07071	0.411	0.1457	impact	0.752642	########
C76-C80	10	1915	0.06817	-0.137	0.2519	incidence	0.20966	########
C76-C80	10	1915	0.06817	-0.137	0.2519	impact	0.07266	########
C76-C80	10	1925	0.06817	-0.137	0.2519	incidence	0.016	########
C76-C80	10	1925	0.06817	-0.137	0.2519	impact	-0.121	########
C76-C80	10	1935	0.06817	-0.137	0.2519	incidence	0.23443	########
C76-C80	10	1935	0.06817	-0.137	0.2519	impact	0.09743	########
C76-C80	10	1945	0.06817	-0.137	0.2519	incidence	0.268284	########
C76-C80	10	1945	0.06817	-0.137	0.2519	impact	0.131284	########
C76-C80	10	1955	0.06817	-0.137	0.2519	incidence	0.524956	0.0838
C76-C80	10	1955	0.06817	-0.137	0.2519	impact	0.387956	########
Z40-Z54	1	1915	0.05436	0.508	0.1214	incidence	0.26653	########
Z40-Z54	1	1915	0.05436	0.508	0.1214	impact	0.77453	########
Z40-Z54	1	1925	0.05436	0.508	0.1214	incidence	0.03265	########

For peer review only

Z40-Z54	1	1925	0.05436	0.508	0.1214	impact	0.54065	########
Z40-Z54	1	1935	0.05436	0.508	0.1214	incidence	-0.03664	########
Z40-Z54	1	1935	0.05436	0.508	0.1214	impact	0.47136	########
Z40-Z54	1	1945	0.05436	0.508	0.1214	incidence	0.015488	########
Z40-Z54	1	1945	0.05436	0.508	0.1214	impact	0.523488	########
Z40-Z54	1	1955	0.05436	0.508	0.1214	incidence	-0.084348	0.0406
Z40-Z54	1	1955	0.05436	0.508	0.1214	impact	0.423652	########
Z40-Z54	2	1915	0.05805	0.296	0.1157	incidence	0.04114	########
Z40-Z54	2	1915	0.05805	0.296	0.1157	impact	0.33714	########
Z40-Z54	2	1925	0.05805	0.296	0.1157	incidence	0.00632	########
Z40-Z54	2	1925	0.05805	0.296	0.1157	impact	0.30232	########
Z40-Z54	2	1935	0.05805	0.296	0.1157	incidence	0.00445	########
Z40-Z54	2	1935	0.05805	0.296	0.1157	impact	0.30045	########
Z40-Z54	2	1945	0.05805	0.296	0.1157	incidence	-0.025173	########
Z40-Z54	2	1945	0.05805	0.296	0.1157	impact	0.270827	########
Z40-Z54	2	1955	0.05805	0.296	0.1157	incidence	-0.080067	0.0372
Z40-Z54	2	1955	0.05805	0.296	0.1157	impact	0.215933	########
Z40-Z54	3	1915	0.05967	0.136	0.0591	incidence	0.14597	########
Z40-Z54	3	1915	0.05967	0.136	0.0591	impact	0.28197	########
Z40-Z54	3	1925	0.05967	0.136	0.0591	incidence	0.1359	########
Z40-Z54	3	1925	0.05967	0.136	0.0591	impact	0.2719	########
Z40-Z54	3	1935	0.05967	0.136	0.0591	incidence	0.00531	########
Z40-Z54	3	1935	0.05967	0.136	0.0591	impact	0.14131	########
Z40-Z54	3	1945	0.05967	0.136	0.0591	incidence	-0.017164	########
Z40-Z54	3	1945	0.05967	0.136	0.0591	impact	0.118836	########
Z40-Z54	3	1955	0.05967	0.136	0.0591	incidence	-0.051336	0.0373
Z40-Z54	3	1955	0.05967	0.136	0.0591	impact	0.084664	########
Z40-Z54	4	1915	0.05692	0.288	0.0692	incidence	0.18134	########
Z40-Z54	4	1915	0.05692	0.288	0.0692	impact	0.46934	########
Z40-Z54	4	1925	0.05692	0.288	0.0692	incidence	0.12067	########
Z40-Z54	4	1925	0.05692	0.288	0.0692	impact	0.40867	########
Z40-Z54	4	1935	0.05692	0.288	0.0692	incidence	0.05896	########
Z40-Z54	4	1935	0.05692	0.288	0.0692	impact	0.34696	########
Z40-Z54	4	1945	0.05692	0.288	0.0692	incidence	-0.049703	########
Z40-Z54	4	1945	0.05692	0.288	0.0692	impact	0.238297	########
Z40-Z54	4	1955	0.05692	0.288	0.0692	incidence	0.004753	0.0368
Z40-Z54	4	1955	0.05692	0.288	0.0692	impact	0.292753	########
Z40-Z54	5	1915	0.06141	0.41	0.0748	incidence	0.26153	########
Z40-Z54	5	1915	0.06141	0.41	0.0748	impact	0.67153	########
Z40-Z54	5	1925	0.06141	0.41	0.0748	incidence	-0.0388	########
Z40-Z54	5	1925	0.06141	0.41	0.0748	impact	0.3712	########
Z40-Z54	5	1935	0.06141	0.41	0.0748	incidence	0.04062	########
Z40-Z54	5	1935	0.06141	0.41	0.0748	impact	0.45062	########
Z40-Z54	5	1945	0.06141	0.41	0.0748	incidence	-0.01316	########
Z40-Z54	5	1945	0.06141	0.41	0.0748	impact	0.39684	########
Z40-Z54	5	1955	0.06141	0.41	0.0748	incidence	3.00E-04	0.0352
Z40-Z54	5	1955	0.06141	0.41	0.0748	impact	0.4103	########
Z40-Z54	6	1915	0.06444	0.184	0.0853	incidence	0.057	########
Z40-Z54	6	1915	0.06444	0.184	0.0853	impact	0.241	########
Z40-Z54	6	1925	0.06444	0.184	0.0853	incidence	0.00725	########
Z40-Z54	6	1925	0.06444	0.184	0.0853	impact	0.19125	########
Z40-Z54	6	1935	0.06444	0.184	0.0853	incidence	0.00151	########
Z40-Z54	6	1935	0.06444	0.184	0.0853	impact	0.18551	########
Z40-Z54	6	1945	0.06444	0.184	0.0853	incidence	-0.030004	########
Z40-Z54	6	1945	0.06444	0.184	0.0853	impact	0.153996	########
Z40-Z54	6	1955	0.06444	0.184	0.0853	incidence	-0.050006	0.0343

For peer review only

Z40-Z54	6	1955	0.06444	0.184	0.0853	impact	0.133994	########
Z40-Z54	7	1915	0.06444	0.342	0.0583	incidence	0.12862	########
Z40-Z54	7	1915	0.06444	0.342	0.0583	impact	0.47062	########
Z40-Z54	7	1925	0.06444	0.342	0.0583	incidence	-0.05119	########
Z40-Z54	7	1925	0.06444	0.342	0.0583	impact	0.29081	########
Z40-Z54	7	1935	0.06444	0.342	0.0583	incidence	-0.04509	########
Z40-Z54	7	1935	0.06444	0.342	0.0583	impact	0.29691	########
Z40-Z54	7	1945	0.06444	0.342	0.0583	incidence	-0.005859	########
Z40-Z54	7	1945	0.06444	0.342	0.0583	impact	0.336141	########
Z40-Z54	7	1955	0.06444	0.342	0.0583	incidence	-0.047461	0.033
Z40-Z54	7	1955	0.06444	0.342	0.0583	impact	0.294539	########
Z40-Z54	8	1915	0.06346	0.404	0.0808	incidence	0.14382	########
Z40-Z54	8	1915	0.06346	0.404	0.0808	impact	0.54782	########
Z40-Z54	8	1925	0.06346	0.404	0.0808	incidence	-0.0167	########
Z40-Z54	8	1925	0.06346	0.404	0.0808	impact	0.3873	########
Z40-Z54	8	1935	0.06346	0.404	0.0808	incidence	-0.0122	########
Z40-Z54	8	1935	0.06346	0.404	0.0808	impact	0.3918	########
Z40-Z54	8	1945	0.06346	0.404	0.0808	incidence	0.026182	########
Z40-Z54	8	1945	0.06346	0.404	0.0808	impact	0.430182	########
Z40-Z54	8	1955	0.06346	0.404	0.0808	incidence	-0.073292	0.0326
Z40-Z54	8	1955	0.06346	0.404	0.0808	impact	0.330708	########
Z40-Z54	9	1915	0.06254	0.178	0.1077	incidence	-0.16262	########
Z40-Z54	9	1915	0.06254	0.178	0.1077	impact	0.01538	########
Z40-Z54	9	1925	0.06254	0.178	0.1077	incidence	-0.03119	########
Z40-Z54	9	1925	0.06254	0.178	0.1077	impact	0.14681	########
Z40-Z54	9	1935	0.06254	0.178	0.1077	incidence	-0.12005	########
Z40-Z54	9	1935	0.06254	0.178	0.1077	impact	0.05795	########
Z40-Z54	9	1945	0.06254	0.178	0.1077	incidence	-0.065832	########
Z40-Z54	9	1945	0.06254	0.178	0.1077	impact	0.112168	########
Z40-Z54	9	1955	0.06254	0.178	0.1077	incidence	-0.082148	0.0315
Z40-Z54	9	1955	0.06254	0.178	0.1077	impact	0.095852	########
Z40-Z54	10	1915	0.06599	0.237	0.0504	incidence	0.07477	########
Z40-Z54	10	1915	0.06599	0.237	0.0504	impact	0.31177	########
Z40-Z54	10	1925	0.06599	0.237	0.0504	incidence	-0.10096	########
Z40-Z54	10	1925	0.06599	0.237	0.0504	impact	0.13604	########
Z40-Z54	10	1935	0.06599	0.237	0.0504	incidence	-0.10201	########
Z40-Z54	10	1935	0.06599	0.237	0.0504	impact	0.13499	########
Z40-Z54	10	1945	0.06599	0.237	0.0504	incidence	-0.102115	########
Z40-Z54	10	1945	0.06599	0.237	0.0504	impact	0.134885	########
Z40-Z54	10	1955	0.06599	0.237	0.0504	incidence	-0.123925	0.0304
Z40-Z54	10	1955	0.06599	0.237	0.0504	impact	0.113075	########
I60-I69	1	1915	0.04602	0.335	0.1035	incidence	0.11634	########
I60-I69	1	1915	0.04602	0.335	0.1035	impact	0.45134	########
I60-I69	1	1925	0.04602	0.335	0.1035	incidence	0.3588	########
I60-I69	1	1925	0.04602	0.335	0.1035	impact	0.6938	########
I60-I69	1	1935	0.04602	0.335	0.1035	incidence	0.36692	########
I60-I69	1	1935	0.04602	0.335	0.1035	impact	0.70192	########
I60-I69	1	1945	0.04602	0.335	0.1035	incidence	0.234405	########
I60-I69	1	1945	0.04602	0.335	0.1035	impact	0.569405	########
I60-I69	1	1955	0.04602	0.335	0.1035	incidence	0.024945	0.0901
I60-I69	1	1955	0.04602	0.335	0.1035	impact	0.359945	########
I60-I69	2	1915	0.04864	0.113	0.0658	incidence	-0.004	########
I60-I69	2	1915	0.04864	0.113	0.0658	impact	0.109	########
I60-I69	2	1925	0.04864	0.113	0.0658	incidence	0.35773	########
I60-I69	2	1925	0.04864	0.113	0.0658	impact	0.47073	########
I60-I69	2	1935	0.04864	0.113	0.0658	incidence	0.38417	########

For peer review only

I60-I69	2	1935	0.04864	0.113	0.0658	impact	0.49717	########
I60-I69	2	1945	0.04864	0.113	0.0658	incidence	0.271516	########
I60-I69	2	1945	0.04864	0.113	0.0658	impact	0.384516	########
I60-I69	2	1955	0.04864	0.113	0.0658	incidence	-0.020436	0.0787
I60-I69	2	1955	0.04864	0.113	0.0658	impact	0.092564	########
I60-I69	3	1915	0.04845	-0.0343	0.0859	incidence	0.0286	########
I60-I69	3	1915	0.04845	-0.0343	0.0859	impact	-0.0057	########
I60-I69	3	1925	0.04845	-0.0343	0.0859	incidence	0.30234	########
I60-I69	3	1925	0.04845	-0.0343	0.0859	impact	0.26804	########
I60-I69	3	1935	0.04845	-0.0343	0.0859	incidence	0.35101	########
I60-I69	3	1935	0.04845	-0.0343	0.0859	impact	0.31671	########
I60-I69	3	1945	0.04845	-0.0343	0.0859	incidence	0.218668	########
I60-I69	3	1945	0.04845	-0.0343	0.0859	impact	0.184368	########
I60-I69	3	1955	0.04845	-0.0343	0.0859	incidence	0.058432	0.0758
I60-I69	3	1955	0.04845	-0.0343	0.0859	impact	0.024132	########
I60-I69	4	1915	0.05517	0.0596	0.0747	incidence	0.06428	########
I60-I69	4	1915	0.05517	0.0596	0.0747	impact	0.12388	########
I60-I69	4	1925	0.05517	0.0596	0.0747	incidence	0.27982	########
I60-I69	4	1925	0.05517	0.0596	0.0747	impact	0.33942	########
I60-I69	4	1935	0.05517	0.0596	0.0747	incidence	0.32731	########
I60-I69	4	1935	0.05517	0.0596	0.0747	impact	0.38691	########
I60-I69	4	1945	0.05517	0.0596	0.0747	incidence	0.368247	########
I60-I69	4	1945	0.05517	0.0596	0.0747	impact	0.427847	########
I60-I69	4	1955	0.05517	0.0596	0.0747	incidence	0.140233	0.0734
I60-I69	4	1955	0.05517	0.0596	0.0747	impact	0.199833	########
I60-I69	5	1915	0.0566	-0.0936	0.1751	incidence	0.05974	########
I60-I69	5	1915	0.0566	-0.0936	0.1751	impact	-0.03386	########
I60-I69	5	1925	0.0566	-0.0936	0.1751	incidence	0.27714	########
I60-I69	5	1925	0.0566	-0.0936	0.1751	impact	0.18354	########
I60-I69	5	1935	0.0566	-0.0936	0.1751	incidence	0.32715	########
I60-I69	5	1935	0.0566	-0.0936	0.1751	impact	0.23355	########
I60-I69	5	1945	0.0566	-0.0936	0.1751	incidence	0.245696	########
I60-I69	5	1945	0.0566	-0.0936	0.1751	impact	0.152096	########
I60-I69	5	1955	0.0566	-0.0936	0.1751	incidence	0.046484	0.069
I60-I69	5	1955	0.0566	-0.0936	0.1751	impact	-0.047116	########
I60-I69	6	1915	0.05977	0.326	0.0758	incidence	0.17266	########
I60-I69	6	1915	0.05977	0.326	0.0758	impact	0.49866	########
I60-I69	6	1925	0.05977	0.326	0.0758	incidence	0.32483	########
I60-I69	6	1925	0.05977	0.326	0.0758	impact	0.65083	########
I60-I69	6	1935	0.05977	0.326	0.0758	incidence	0.39747	########
I60-I69	6	1935	0.05977	0.326	0.0758	impact	0.72347	########
I60-I69	6	1945	0.05977	0.326	0.0758	incidence	0.290796	########
I60-I69	6	1945	0.05977	0.326	0.0758	impact	0.616796	########
I60-I69	6	1955	0.05977	0.326	0.0758	incidence	0.179684	0.0661
I60-I69	6	1955	0.05977	0.326	0.0758	impact	0.505684	########
I60-I69	7	1915	0.06041	0.224	0.1172	incidence	0.17323	########
I60-I69	7	1915	0.06041	0.224	0.1172	impact	0.39723	########
I60-I69	7	1925	0.06041	0.224	0.1172	incidence	0.27813	########
I60-I69	7	1925	0.06041	0.224	0.1172	impact	0.50213	########
I60-I69	7	1935	0.06041	0.224	0.1172	incidence	0.30292	########
I60-I69	7	1935	0.06041	0.224	0.1172	impact	0.52692	########
I60-I69	7	1945	0.06041	0.224	0.1172	incidence	0.249883	########
I60-I69	7	1945	0.06041	0.224	0.1172	impact	0.473883	########
I60-I69	7	1955	0.06041	0.224	0.1172	incidence	0.189237	0.063
I60-I69	7	1955	0.06041	0.224	0.1172	impact	0.413237	########
I60-I69	8	1915	0.06284	0.162	0.1262	incidence	0.05217	########

For peer review only

I60-I69	8	1915	0.06284	0.162	0.1262	impact	0.21417	########
I60-I69	8	1925	0.06284	0.162	0.1262	incidence	0.28415	########
I60-I69	8	1925	0.06284	0.162	0.1262	impact	0.44615	########
I60-I69	8	1935	0.06284	0.162	0.1262	incidence	0.29143	########
I60-I69	8	1935	0.06284	0.162	0.1262	impact	0.45343	########
I60-I69	8	1945	0.06284	0.162	0.1262	incidence	0.323187	########
I60-I69	8	1945	0.06284	0.162	0.1262	impact	0.485187	########
I60-I69	8	1955	0.06284	0.162	0.1262	incidence	0.169693	0.0574
I60-I69	8	1955	0.06284	0.162	0.1262	impact	0.331693	########
I60-I69	9	1915	0.06622	0.344	0.1757	incidence	0.11531	########
I60-I69	9	1915	0.06622	0.344	0.1757	impact	0.45931	########
I60-I69	9	1925	0.06622	0.344	0.1757	incidence	0.33137	########
I60-I69	9	1925	0.06622	0.344	0.1757	impact	0.67537	########
I60-I69	9	1935	0.06622	0.344	0.1757	incidence	0.26723	########
I60-I69	9	1935	0.06622	0.344	0.1757	impact	0.61123	########
I60-I69	9	1945	0.06622	0.344	0.1757	incidence	0.240802	########
I60-I69	9	1945	0.06622	0.344	0.1757	impact	0.584802	########
I60-I69	9	1955	0.06622	0.344	0.1757	incidence	0.131778	0.0543
I60-I69	9	1955	0.06622	0.344	0.1757	impact	0.475778	########
I60-I69	10	1915	0.0661	0.387	0.0974	incidence	0.31221	########
I60-I69	10	1915	0.0661	0.387	0.0974	impact	0.69921	########
I60-I69	10	1925	0.0661	0.387	0.0974	incidence	0.35037	########
I60-I69	10	1925	0.0661	0.387	0.0974	impact	0.73737	########
I60-I69	10	1935	0.0661	0.387	0.0974	incidence	0.37446	########
I60-I69	10	1935	0.0661	0.387	0.0974	impact	0.76146	########
I60-I69	10	1945	0.0661	0.387	0.0974	incidence	0.379224	########
I60-I69	10	1945	0.0661	0.387	0.0974	impact	0.766224	########
I60-I69	10	1955	0.0661	0.387	0.0974	incidence	0.210386	0.0516
I60-I69	10	1955	0.0661	0.387	0.0974	impact	0.597386	########
K55-K63	1	1915	0.05313	0.372	0.0952	incidence	0.16481	########
K55-K63	1	1915	0.05313	0.372	0.0952	impact	0.53681	########
K55-K63	1	1925	0.05313	0.372	0.0952	incidence	0.1328	########
K55-K63	1	1925	0.05313	0.372	0.0952	impact	0.5048	########
K55-K63	1	1935	0.05313	0.372	0.0952	incidence	-0.02573	########
K55-K63	1	1935	0.05313	0.372	0.0952	impact	0.34627	########
K55-K63	1	1945	0.05313	0.372	0.0952	incidence	0.064532	########
K55-K63	1	1945	0.05313	0.372	0.0952	impact	0.436532	########
K55-K63	1	1955	0.05313	0.372	0.0952	incidence	-0.002582	0.0356
K55-K63	1	1955	0.05313	0.372	0.0952	impact	0.369418	########
K55-K63	2	1915	0.0558	0.352	0.1217	incidence	0.15724	########
K55-K63	2	1915	0.0558	0.352	0.1217	impact	0.50924	########
K55-K63	2	1925	0.0558	0.352	0.1217	incidence	0.18325	########
K55-K63	2	1925	0.0558	0.352	0.1217	impact	0.53525	########
K55-K63	2	1935	0.0558	0.352	0.1217	incidence	0.0696	########
K55-K63	2	1935	0.0558	0.352	0.1217	impact	0.4216	########
K55-K63	2	1945	0.0558	0.352	0.1217	incidence	0.02466	########
K55-K63	2	1945	0.0558	0.352	0.1217	impact	0.37666	########
K55-K63	2	1955	0.0558	0.352	0.1217	incidence	0.0265	0.0324
K55-K63	2	1955	0.0558	0.352	0.1217	impact	0.3785	########
K55-K63	3	1915	0.05091	0.156	0.1045	incidence	0.0715	########
K55-K63	3	1915	0.05091	0.156	0.1045	impact	0.2275	########
K55-K63	3	1925	0.05091	0.156	0.1045	incidence	0.09562	########
K55-K63	3	1925	0.05091	0.156	0.1045	impact	0.25162	########
K55-K63	3	1935	0.05091	0.156	0.1045	incidence	0.02416	########
K55-K63	3	1935	0.05091	0.156	0.1045	impact	0.18016	########
K55-K63	3	1945	0.05091	0.156	0.1045	incidence	-0.040384	########

For peer review only

K55-K63	3	1945	0.05091	0.156	0.1045	impact	0.115616	########
K55-K63	3	1955	0.05091	0.156	0.1045	incidence	-0.000796	0.0332
K55-K63	3	1955	0.05091	0.156	0.1045	impact	0.155204	########
K55-K63	4	1915	0.05251	0.133	0.0466	incidence	0.09006	########
K55-K63	4	1915	0.05251	0.133	0.0466	impact	0.22306	########
K55-K63	4	1925	0.05251	0.133	0.0466	incidence	0.08543	########
K55-K63	4	1925	0.05251	0.133	0.0466	impact	0.21843	########
K55-K63	4	1935	0.05251	0.133	0.0466	incidence	0.09841	########
K55-K63	4	1935	0.05251	0.133	0.0466	impact	0.23141	########
K55-K63	4	1945	0.05251	0.133	0.0466	incidence	0.059151	########
K55-K63	4	1945	0.05251	0.133	0.0466	impact	0.192151	########
K55-K63	4	1955	0.05251	0.133	0.0466	incidence	-0.071941	0.0335
K55-K63	4	1955	0.05251	0.133	0.0466	impact	0.061059	########
K55-K63	5	1915	0.05646	0.322	0.0821	incidence	0.08743	########
K55-K63	5	1915	0.05646	0.322	0.0821	impact	0.40943	########
K55-K63	5	1925	0.05646	0.322	0.0821	incidence	0.08515	########
K55-K63	5	1925	0.05646	0.322	0.0821	impact	0.40715	########
K55-K63	5	1935	0.05646	0.322	0.0821	incidence	0.05228	########
K55-K63	5	1935	0.05646	0.322	0.0821	impact	0.37428	########
K55-K63	5	1945	0.05646	0.322	0.0821	incidence	-0.008988	########
K55-K63	5	1945	0.05646	0.322	0.0821	impact	0.313012	########
K55-K63	5	1955	0.05646	0.322	0.0821	incidence	0.039858	0.032
K55-K63	5	1955	0.05646	0.322	0.0821	impact	0.361858	########
K55-K63	6	1915	0.0608	0.108	0.0607	incidence	0.27748	########
K55-K63	6	1915	0.0608	0.108	0.0607	impact	0.38548	########
K55-K63	6	1925	0.0608	0.108	0.0607	incidence	0.17274	########
K55-K63	6	1925	0.0608	0.108	0.0607	impact	0.28074	########
K55-K63	6	1935	0.0608	0.108	0.0607	incidence	0.03499	########
K55-K63	6	1935	0.0608	0.108	0.0607	impact	0.14299	########
K55-K63	6	1945	0.0608	0.108	0.0607	incidence	0.010616	########
K55-K63	6	1945	0.0608	0.108	0.0607	impact	0.118616	########
K55-K63	6	1955	0.0608	0.108	0.0607	incidence	0.013564	0.0308
K55-K63	6	1955	0.0608	0.108	0.0607	impact	0.121564	########
K55-K63	7	1915	0.05797	0.231	0.076	incidence	0.26153	########
K55-K63	7	1915	0.05797	0.231	0.076	impact	0.49253	########
K55-K63	7	1925	0.05797	0.231	0.076	incidence	0.10128	########
K55-K63	7	1925	0.05797	0.231	0.076	impact	0.33228	########
K55-K63	7	1935	0.05797	0.231	0.076	incidence	0.06319	########
K55-K63	7	1935	0.05797	0.231	0.076	impact	0.29419	########
K55-K63	7	1945	0.05797	0.231	0.076	incidence	0.04778	########
K55-K63	7	1945	0.05797	0.231	0.076	impact	0.27878	########
K55-K63	7	1955	0.05797	0.231	0.076	incidence	0.03564	0.0304
K55-K63	7	1955	0.05797	0.231	0.076	impact	0.26664	########
K55-K63	8	1915	0.06053	0.16	0.0355	incidence	0.22605	########
K55-K63	8	1915	0.06053	0.16	0.0355	impact	0.38605	########
K55-K63	8	1925	0.06053	0.16	0.0355	incidence	0.1011	########
K55-K63	8	1925	0.06053	0.16	0.0355	impact	0.2611	########
K55-K63	8	1935	0.06053	0.16	0.0355	incidence	0.075	########
K55-K63	8	1935	0.06053	0.16	0.0355	impact	0.235	########
K55-K63	8	1945	0.06053	0.16	0.0355	incidence	0.05034	########
K55-K63	8	1945	0.06053	0.16	0.0355	impact	0.21034	########
K55-K63	8	1955	0.06053	0.16	0.0355	incidence	0.01177	0.029
K55-K63	8	1955	0.06053	0.16	0.0355	impact	0.17177	########
K55-K63	9	1915	0.06093	0.0284	0.0519	incidence	-0.00377	########
K55-K63	9	1915	0.06093	0.0284	0.0519	impact	0.02463	########
K55-K63	9	1925	0.06093	0.0284	0.0519	incidence	0.19071	########

For peer review only

K55-K63	9	1925	0.06093	0.0284	0.0519	impact	0.21911	########
K55-K63	9	1935	0.06093	0.0284	0.0519	incidence	0.04469	########
K55-K63	9	1935	0.06093	0.0284	0.0519	impact	0.07309	########
K55-K63	9	1945	0.06093	0.0284	0.0519	incidence	-0.009761	########
K55-K63	9	1945	0.06093	0.0284	0.0519	impact	0.018639	########
K55-K63	9	1955	0.06093	0.0284	0.0519	incidence	-0.003819	0.0281
K55-K63	9	1955	0.06093	0.0284	0.0519	impact	0.024581	########
K55-K63	10	1915	0.06045	0.438	0.0651	incidence	-0.02273	########
K55-K63	10	1915	0.06045	0.438	0.0651	impact	0.41527	########
K55-K63	10	1925	0.06045	0.438	0.0651	incidence	0.00523	########
K55-K63	10	1925	0.06045	0.438	0.0651	impact	0.44323	########
K55-K63	10	1935	0.06045	0.438	0.0651	incidence	-0.07153	########
K55-K63	10	1935	0.06045	0.438	0.0651	impact	0.36647	########
K55-K63	10	1945	0.06045	0.438	0.0651	incidence	-0.034066	########
K55-K63	10	1945	0.06045	0.438	0.0651	impact	0.403934	########
K55-K63	10	1955	0.06045	0.438	0.0651	incidence	-0.085644	0.0265
K55-K63	10	1955	0.06045	0.438	0.0651	impact	0.352356	########
J40-J47	1	1915	0.02506	-0.0521	0.2423	incidence	0.02809	########
J40-J47	1	1915	0.02506	-0.0521	0.2423	impact	-0.02401	########
J40-J47	1	1925	0.02506	-0.0521	0.2423	incidence	0.43055	########
J40-J47	1	1925	0.02506	-0.0521	0.2423	impact	0.37845	########
J40-J47	1	1935	0.02506	-0.0521	0.2423	incidence	0.45318	########
J40-J47	1	1935	0.02506	-0.0521	0.2423	impact	0.40108	########
J40-J47	1	1945	0.02506	-0.0521	0.2423	incidence	0.289603	########
J40-J47	1	1945	0.02506	-0.0521	0.2423	impact	0.237503	########
J40-J47	1	1955	0.02506	-0.0521	0.2423	incidence	0.471947	0.1136
J40-J47	1	1955	0.02506	-0.0521	0.2423	impact	0.419847	########
J40-J47	2	1915	0.02988	0.302	0.0996	incidence	0.08036	########
J40-J47	2	1915	0.02988	0.302	0.0996	impact	0.38236	########
J40-J47	2	1925	0.02988	0.302	0.0996	incidence	0.39953	########
J40-J47	2	1925	0.02988	0.302	0.0996	impact	0.70153	########
J40-J47	2	1935	0.02988	0.302	0.0996	incidence	0.47901	########
J40-J47	2	1935	0.02988	0.302	0.0996	impact	0.78101	########
J40-J47	2	1945	0.02988	0.302	0.0996	incidence	0.286284	########
J40-J47	2	1945	0.02988	0.302	0.0996	impact	0.588284	########
J40-J47	2	1955	0.02988	0.302	0.0996	incidence	0.133186	0.0958
J40-J47	2	1955	0.02988	0.302	0.0996	impact	0.435186	########
J40-J47	3	1915	0.03616	0.219	0.1306	incidence	-0.05922	########
J40-J47	3	1915	0.03616	0.219	0.1306	impact	0.15978	########
J40-J47	3	1925	0.03616	0.219	0.1306	incidence	0.25046	########
J40-J47	3	1925	0.03616	0.219	0.1306	impact	0.46946	########
J40-J47	3	1935	0.03616	0.219	0.1306	incidence	0.49121	########
J40-J47	3	1935	0.03616	0.219	0.1306	impact	0.71021	########
J40-J47	3	1945	0.03616	0.219	0.1306	incidence	0.367141	########
J40-J47	3	1945	0.03616	0.219	0.1306	impact	0.586141	########
J40-J47	3	1955	0.03616	0.219	0.1306	incidence	0.326989	0.0881
J40-J47	3	1955	0.03616	0.219	0.1306	impact	0.545989	########
J40-J47	4	1915	0.04058	0.302	0.1523	incidence	0.15184	########
J40-J47	4	1915	0.04058	0.302	0.1523	impact	0.45384	########
J40-J47	4	1925	0.04058	0.302	0.1523	incidence	0.32878	########
J40-J47	4	1925	0.04058	0.302	0.1523	impact	0.63078	########
J40-J47	4	1935	0.04058	0.302	0.1523	incidence	0.48842	########
J40-J47	4	1935	0.04058	0.302	0.1523	impact	0.79042	########
J40-J47	4	1945	0.04058	0.302	0.1523	incidence	0.378132	########
J40-J47	4	1945	0.04058	0.302	0.1523	impact	0.680132	########
J40-J47	4	1955	0.04058	0.302	0.1523	incidence	0.126878	0.0816

For peer review only

J40-J47	4	1955	0.04058	0.302	0.1523	impact	0.428878	########
J40-J47	5	1915	0.04837	0.108	0.1366	incidence	0.04533	########
J40-J47	5	1915	0.04837	0.108	0.1366	impact	0.15333	########
J40-J47	5	1925	0.04837	0.108	0.1366	incidence	0.18723	########
J40-J47	5	1925	0.04837	0.108	0.1366	impact	0.29523	########
J40-J47	5	1935	0.04837	0.108	0.1366	incidence	0.39549	########
J40-J47	5	1935	0.04837	0.108	0.1366	impact	0.50349	########
J40-J47	5	1945	0.04837	0.108	0.1366	incidence	0.297718	########
J40-J47	5	1945	0.04837	0.108	0.1366	impact	0.405718	########
J40-J47	5	1955	0.04837	0.108	0.1366	incidence	0.223222	0.0747
J40-J47	5	1955	0.04837	0.108	0.1366	impact	0.331222	########
J40-J47	6	1915	0.056	0.0917	0.1844	incidence	0.13652	########
J40-J47	6	1915	0.056	0.0917	0.1844	impact	0.22822	########
J40-J47	6	1925	0.056	0.0917	0.1844	incidence	0.26147	########
J40-J47	6	1925	0.056	0.0917	0.1844	impact	0.35317	########
J40-J47	6	1935	0.056	0.0917	0.1844	incidence	0.36612	########
J40-J47	6	1935	0.056	0.0917	0.1844	impact	0.45782	########
J40-J47	6	1945	0.056	0.0917	0.1844	incidence	0.341374	########
J40-J47	6	1945	0.056	0.0917	0.1844	impact	0.433074	########
J40-J47	6	1955	0.056	0.0917	0.1844	incidence	0.247546	0.0686
J40-J47	6	1955	0.056	0.0917	0.1844	impact	0.339246	########
J40-J47	7	1915	0.06357	0.225	0.0885	incidence	0.17574	########
J40-J47	7	1915	0.06357	0.225	0.0885	impact	0.40074	########
J40-J47	7	1925	0.06357	0.225	0.0885	incidence	0.21502	########
J40-J47	7	1925	0.06357	0.225	0.0885	impact	0.44002	########
J40-J47	7	1935	0.06357	0.225	0.0885	incidence	0.38217	########
J40-J47	7	1935	0.06357	0.225	0.0885	impact	0.60717	########
J40-J47	7	1945	0.06357	0.225	0.0885	incidence	0.340247	########
J40-J47	7	1945	0.06357	0.225	0.0885	impact	0.565247	########
J40-J47	7	1955	0.06357	0.225	0.0885	incidence	0.238343	0.0609
J40-J47	7	1955	0.06357	0.225	0.0885	impact	0.463343	########
J40-J47	8	1915	0.07319	0.18	0.0345	incidence	0.05435	########
J40-J47	8	1915	0.07319	0.18	0.0345	impact	0.23435	########
J40-J47	8	1925	0.07319	0.18	0.0345	incidence	0.21284	########
J40-J47	8	1925	0.07319	0.18	0.0345	impact	0.39284	########
J40-J47	8	1935	0.07319	0.18	0.0345	incidence	0.35065	########
J40-J47	8	1935	0.07319	0.18	0.0345	impact	0.53065	########
J40-J47	8	1945	0.07319	0.18	0.0345	incidence	0.287578	########
J40-J47	8	1945	0.07319	0.18	0.0345	impact	0.467578	########
J40-J47	8	1955	0.07319	0.18	0.0345	incidence	0.236642	0.0558
J40-J47	8	1955	0.07319	0.18	0.0345	impact	0.416642	########
J40-J47	9	1915	0.08131	0.372	0.0696	incidence	0.03434	########
J40-J47	9	1915	0.08131	0.372	0.0696	impact	0.40634	########
J40-J47	9	1925	0.08131	0.372	0.0696	incidence	0.2394	########
J40-J47	9	1925	0.08131	0.372	0.0696	impact	0.6114	########
J40-J47	9	1935	0.08131	0.372	0.0696	incidence	0.32759	########
J40-J47	9	1935	0.08131	0.372	0.0696	impact	0.69959	########
J40-J47	9	1945	0.08131	0.372	0.0696	incidence	0.275387	########
J40-J47	9	1945	0.08131	0.372	0.0696	impact	0.647387	########
J40-J47	9	1955	0.08131	0.372	0.0696	incidence	0.221263	0.0507
J40-J47	9	1955	0.08131	0.372	0.0696	impact	0.593263	########
J40-J47	10	1915	0.09425	0.32	0.0923	incidence	0.00365	########
J40-J47	10	1915	0.09425	0.32	0.0923	impact	0.32365	########
J40-J47	10	1925	0.09425	0.32	0.0923	incidence	0.14901	########
J40-J47	10	1925	0.09425	0.32	0.0923	impact	0.46901	########
J40-J47	10	1935	0.09425	0.32	0.0923	incidence	0.28583	########

For peer review only

J40-J47	10	1935	0.09425	0.32	0.0923	impact	0.60583	########
J40-J47	10	1945	0.09425	0.32	0.0923	incidence	0.334637	########
J40-J47	10	1945	0.09425	0.32	0.0923	impact	0.654637	########
J40-J47	10	1955	0.09425	0.32	0.0923	incidence	0.234223	0.0441
J40-J47	10	1955	0.09425	0.32	0.0923	impact	0.554223	########
I20-I25	1	1915	0.04147	0.272	0.2127	incidence	0.23326	########
I20-I25	1	1915	0.04147	0.272	0.2127	impact	0.50526	########
I20-I25	1	1925	0.04147	0.272	0.2127	incidence	0.47813	########
I20-I25	1	1925	0.04147	0.272	0.2127	impact	0.75013	########
I20-I25	1	1935	0.04147	0.272	0.2127	incidence	0.47419	########
I20-I25	1	1935	0.04147	0.272	0.2127	impact	0.74619	########
I20-I25	1	1945	0.04147	0.272	0.2127	incidence	0.451977	########
I20-I25	1	1945	0.04147	0.272	0.2127	impact	0.723977	########
I20-I25	1	1955	0.04147	0.272	0.2127	incidence	0.344883	0.0492
I20-I25	1	1955	0.04147	0.272	0.2127	impact	0.616883	########
I20-I25	2	1915	0.04704	0.274	0.1164	incidence	0.15926	########
I20-I25	2	1915	0.04704	0.274	0.1164	impact	0.43326	########
I20-I25	2	1925	0.04704	0.274	0.1164	incidence	0.4622	########
I20-I25	2	1925	0.04704	0.274	0.1164	impact	0.7362	########
I20-I25	2	1935	0.04704	0.274	0.1164	incidence	0.47575	########
I20-I25	2	1935	0.04704	0.274	0.1164	impact	0.74975	########
I20-I25	2	1945	0.04704	0.274	0.1164	incidence	0.455276	########
I20-I25	2	1945	0.04704	0.274	0.1164	impact	0.729276	########
I20-I25	2	1955	0.04704	0.274	0.1164	incidence	0.299724	0.0433
I20-I25	2	1955	0.04704	0.274	0.1164	impact	0.573724	########
I20-I25	3	1915	0.04845	0.0976	0.0974	incidence	0.25766	########
I20-I25	3	1915	0.04845	0.0976	0.0974	impact	0.35526	########
I20-I25	3	1925	0.04845	0.0976	0.0974	incidence	0.34688	########
I20-I25	3	1925	0.04845	0.0976	0.0974	impact	0.44448	########
I20-I25	3	1935	0.04845	0.0976	0.0974	incidence	0.49087	########
I20-I25	3	1935	0.04845	0.0976	0.0974	impact	0.58847	########
I20-I25	3	1945	0.04845	0.0976	0.0974	incidence	0.444557	########
I20-I25	3	1945	0.04845	0.0976	0.0974	impact	0.542157	########
I20-I25	3	1955	0.04845	0.0976	0.0974	incidence	0.337513	0.0433
I20-I25	3	1955	0.04845	0.0976	0.0974	impact	0.435113	########
I20-I25	4	1915	0.05142	0.373	0.0591	incidence	0.29661	########
I20-I25	4	1915	0.05142	0.373	0.0591	impact	0.66961	########
I20-I25	4	1925	0.05142	0.373	0.0591	incidence	0.42579	########
I20-I25	4	1925	0.05142	0.373	0.0591	impact	0.79879	########
I20-I25	4	1935	0.05142	0.373	0.0591	incidence	0.5124	########
I20-I25	4	1935	0.05142	0.373	0.0591	impact	0.8854	########
I20-I25	4	1945	0.05142	0.373	0.0591	incidence	0.499935	########
I20-I25	4	1945	0.05142	0.373	0.0591	impact	0.872935	########
I20-I25	4	1955	0.05142	0.373	0.0591	incidence	0.288295	0.0406
I20-I25	4	1955	0.05142	0.373	0.0591	impact	0.661295	########
I20-I25	5	1915	0.0542	0.227	0.1244	incidence	0.21958	########
I20-I25	5	1915	0.0542	0.227	0.1244	impact	0.44658	########
I20-I25	5	1925	0.0542	0.227	0.1244	incidence	0.41766	########
I20-I25	5	1925	0.0542	0.227	0.1244	impact	0.64466	########
I20-I25	5	1935	0.0542	0.227	0.1244	incidence	0.46006	########
I20-I25	5	1935	0.0542	0.227	0.1244	impact	0.68706	########
I20-I25	5	1945	0.0542	0.227	0.1244	incidence	0.526283	########
I20-I25	5	1945	0.0542	0.227	0.1244	impact	0.753283	########
I20-I25	5	1955	0.0542	0.227	0.1244	incidence	0.353127	0.04
I20-I25	5	1955	0.0542	0.227	0.1244	impact	0.580127	########
I20-I25	6	1915	0.05741	0.288	0.0802	incidence	0.26279	########

I20-I25	6	1915	0.05741	0.288	0.0802	impact	0.55079	########
I20-I25	6	1925	0.05741	0.288	0.0802	incidence	0.4854	########
I20-I25	6	1925	0.05741	0.288	0.0802	impact	0.7734	########
I20-I25	6	1935	0.05741	0.288	0.0802	incidence	0.47584	########
I20-I25	6	1935	0.05741	0.288	0.0802	impact	0.76384	########
I20-I25	6	1945	0.05741	0.288	0.0802	incidence	0.506955	########
I20-I25	6	1945	0.05741	0.288	0.0802	impact	0.794955	########
I20-I25	6	1955	0.05741	0.288	0.0802	incidence	0.355925	0.0379
I20-I25	6	1955	0.05741	0.288	0.0802	impact	0.643925	########
I20-I25	7	1915	0.06044	0.245	0.1318	incidence	0.31751	########
I20-I25	7	1915	0.06044	0.245	0.1318	impact	0.56251	########
I20-I25	7	1925	0.06044	0.245	0.1318	incidence	0.42944	########
I20-I25	7	1925	0.06044	0.245	0.1318	impact	0.67444	########
I20-I25	7	1935	0.06044	0.245	0.1318	incidence	0.49845	########
I20-I25	7	1935	0.06044	0.245	0.1318	impact	0.74345	########
I20-I25	7	1945	0.06044	0.245	0.1318	incidence	0.577794	########
I20-I25	7	1945	0.06044	0.245	0.1318	impact	0.822794	########
I20-I25	7	1955	0.06044	0.245	0.1318	incidence	0.477466	0.0362
I20-I25	7	1955	0.06044	0.245	0.1318	impact	0.722466	########
I20-I25	8	1915	0.0609	0.411	0.041	incidence	0.37173	########
I20-I25	8	1915	0.0609	0.411	0.041	impact	0.78273	########
I20-I25	8	1925	0.0609	0.411	0.041	incidence	0.48465	########
I20-I25	8	1925	0.0609	0.411	0.041	impact	0.89565	########
I20-I25	8	1935	0.0609	0.411	0.041	incidence	0.52876	########
I20-I25	8	1935	0.0609	0.411	0.041	impact	0.93976	########
I20-I25	8	1945	0.0609	0.411	0.041	incidence	0.542288	########
I20-I25	8	1945	0.0609	0.411	0.041	impact	0.953288	########
I20-I25	8	1955	0.0609	0.411	0.041	incidence	0.461432	0.0346
I20-I25	8	1955	0.0609	0.411	0.041	impact	0.872432	########
I20-I25	9	1915	0.05894	0.183	0.0856	incidence	0.28777	########
I20-I25	9	1915	0.05894	0.183	0.0856	impact	0.47077	########
I20-I25	9	1925	0.05894	0.183	0.0856	incidence	0.48374	########
I20-I25	9	1925	0.05894	0.183	0.0856	impact	0.66674	########
I20-I25	9	1935	0.05894	0.183	0.0856	incidence	0.50974	########
I20-I25	9	1935	0.05894	0.183	0.0856	impact	0.69274	########
I20-I25	9	1945	0.05894	0.183	0.0856	incidence	0.563166	########
I20-I25	9	1945	0.05894	0.183	0.0856	impact	0.746166	########
I20-I25	9	1955	0.05894	0.183	0.0856	incidence	0.426194	0.0326
I20-I25	9	1955	0.05894	0.183	0.0856	impact	0.609194	########
I20-I25	10	1915	0.05665	0.236	0.0807	incidence	0.44614	########
I20-I25	10	1915	0.05665	0.236	0.0807	impact	0.68214	########
I20-I25	10	1925	0.05665	0.236	0.0807	incidence	0.42394	########
I20-I25	10	1925	0.05665	0.236	0.0807	impact	0.65994	########
I20-I25	10	1935	0.05665	0.236	0.0807	incidence	0.52125	########
I20-I25	10	1935	0.05665	0.236	0.0807	impact	0.75725	########
I20-I25	10	1945	0.05665	0.236	0.0807	incidence	0.571923	########
I20-I25	10	1945	0.05665	0.236	0.0807	impact	0.807923	########
I20-I25	10	1955	0.05665	0.236	0.0807	incidence	0.433867	0.0306
I20-I25	10	1955	0.05665	0.236	0.0807	impact	0.669867	########
N30-N39	1	1915	0.04904	0.00162	0.0681	incidence	-0.12334	########
N30-N39	1	1915	0.04904	0.00162	0.0681	impact	-0.12172	########
N30-N39	1	1925	0.04904	0.00162	0.0681	incidence	0.10281	########
N30-N39	1	1925	0.04904	0.00162	0.0681	impact	0.10443	########
N30-N39	1	1935	0.04904	0.00162	0.0681	incidence	0.05093	########
N30-N39	1	1935	0.04904	0.00162	0.0681	impact	0.05255	########
N30-N39	1	1945	0.04904	0.00162	0.0681	incidence	0.195006	########

For peer review only

N30-N39	1	1945	0.04904	0.00162	0.0681	impact	0.196626	########
N30-N39	1	1955	0.04904	0.00162	0.0681	incidence	0.161754	0.0536
N30-N39	1	1955	0.04904	0.00162	0.0681	impact	0.163374	########
N30-N39	2	1915	0.05089	-0.132	0.113	incidence	-0.28449	########
N30-N39	2	1915	0.05089	-0.132	0.113	impact	-0.41649	########
N30-N39	2	1925	0.05089	-0.132	0.113	incidence	0.02627	########
N30-N39	2	1925	0.05089	-0.132	0.113	impact	-0.10573	########
N30-N39	2	1935	0.05089	-0.132	0.113	incidence	0.0715	########
N30-N39	2	1935	0.05089	-0.132	0.113	impact	-0.0605	########
N30-N39	2	1945	0.05089	-0.132	0.113	incidence	0.195348	########
N30-N39	2	1945	0.05089	-0.132	0.113	impact	0.063348	########
N30-N39	2	1955	0.05089	-0.132	0.113	incidence	0.084222	0.0487
N30-N39	2	1955	0.05089	-0.132	0.113	impact	-0.047778	########
N30-N39	3	1915	0.05158	-0.258	0.0691	incidence	-0.29514	########
N30-N39	3	1915	0.05158	-0.258	0.0691	impact	-0.55314	########
N30-N39	3	1925	0.05158	-0.258	0.0691	incidence	-0.04983	########
N30-N39	3	1925	0.05158	-0.258	0.0691	impact	-0.30783	########
N30-N39	3	1935	0.05158	-0.258	0.0691	incidence	0.08558	########
N30-N39	3	1935	0.05158	-0.258	0.0691	impact	-0.17242	########
N30-N39	3	1945	0.05158	-0.258	0.0691	incidence	0.173524	########
N30-N39	3	1945	0.05158	-0.258	0.0691	impact	-0.084476	########
N30-N39	3	1955	0.05158	-0.258	0.0691	incidence	0.045926	0.0501
N30-N39	3	1955	0.05158	-0.258	0.0691	impact	-0.212074	########
N30-N39	4	1915	0.05025	-0.276	0.0693	incidence	-0.23343	########
N30-N39	4	1915	0.05025	-0.276	0.0693	impact	-0.50943	########
N30-N39	4	1925	0.05025	-0.276	0.0693	incidence	-0.06284	########
N30-N39	4	1925	0.05025	-0.276	0.0693	impact	-0.33884	########
N30-N39	4	1935	0.05025	-0.276	0.0693	incidence	0.05312	########
N30-N39	4	1935	0.05025	-0.276	0.0693	impact	-0.22288	########
N30-N39	4	1945	0.05025	-0.276	0.0693	incidence	0.147792	########
N30-N39	4	1945	0.05025	-0.276	0.0693	impact	-0.128208	########
N30-N39	4	1955	0.05025	-0.276	0.0693	incidence	0.097318	0.0512
N30-N39	4	1955	0.05025	-0.276	0.0693	impact	-0.178682	########
N30-N39	5	1915	0.05282	-0.167	0.074	incidence	-0.31168	########
N30-N39	5	1915	0.05282	-0.167	0.074	impact	-0.47868	########
N30-N39	5	1925	0.05282	-0.167	0.074	incidence	-0.10752	########
N30-N39	5	1925	0.05282	-0.167	0.074	impact	-0.27452	########
N30-N39	5	1935	0.05282	-0.167	0.074	incidence	0.0168	########
N30-N39	5	1935	0.05282	-0.167	0.074	impact	-0.1502	########
N30-N39	5	1945	0.05282	-0.167	0.074	incidence	0.060536	########
N30-N39	5	1945	0.05282	-0.167	0.074	impact	-0.106464	########
N30-N39	5	1955	0.05282	-0.167	0.074	incidence	0.073974	0.0488
N30-N39	5	1955	0.05282	-0.167	0.074	impact	-0.093026	########
N30-N39	6	1915	0.05613	-0.164	0.1009	incidence	-0.29688	########
N30-N39	6	1915	0.05613	-0.164	0.1009	impact	-0.46088	########
N30-N39	6	1925	0.05613	-0.164	0.1009	incidence	-0.07081	########
N30-N39	6	1925	0.05613	-0.164	0.1009	impact	-0.23481	########
N30-N39	6	1935	0.05613	-0.164	0.1009	incidence	0.03671	########
N30-N39	6	1935	0.05613	-0.164	0.1009	impact	-0.12729	########
N30-N39	6	1945	0.05613	-0.164	0.1009	incidence	0.015466	########
N30-N39	6	1945	0.05613	-0.164	0.1009	impact	-0.148534	########
N30-N39	6	1955	0.05613	-0.164	0.1009	incidence	0.042694	0.0463
N30-N39	6	1955	0.05613	-0.164	0.1009	impact	-0.121306	########
N30-N39	7	1915	0.05793	-0.298	0.139	incidence	-0.26962	########
N30-N39	7	1915	0.05793	-0.298	0.139	impact	-0.56762	########
N30-N39	7	1925	0.05793	-0.298	0.139	incidence	-0.05333	########

For peer review only

N30-N39	7	1925	0.05793	-0.298	0.139	impact	-0.35133	########
N30-N39	7	1935	0.05793	-0.298	0.139	incidence	0.00806	########
N30-N39	7	1935	0.05793	-0.298	0.139	impact	-0.28994	########
N30-N39	7	1945	0.05793	-0.298	0.139	incidence	0.014254	########
N30-N39	7	1945	0.05793	-0.298	0.139	impact	-0.283746	########
N30-N39	7	1955	0.05793	-0.298	0.139	incidence	0.086826	0.0448
N30-N39	7	1955	0.05793	-0.298	0.139	impact	-0.211174	########
N30-N39	8	1915	0.05605	-0.17	0.0693	incidence	-0.27473	########
N30-N39	8	1915	0.05605	-0.17	0.0693	impact	-0.44473	########
N30-N39	8	1925	0.05605	-0.17	0.0693	incidence	-0.15153	########
N30-N39	8	1925	0.05605	-0.17	0.0693	impact	-0.32153	########
N30-N39	8	1935	0.05605	-0.17	0.0693	incidence	-0.00365	########
N30-N39	8	1935	0.05605	-0.17	0.0693	impact	-0.17365	########
N30-N39	8	1945	0.05605	-0.17	0.0693	incidence	0.053115	########
N30-N39	8	1945	0.05605	-0.17	0.0693	impact	-0.116885	########
N30-N39	8	1955	0.05605	-0.17	0.0693	incidence	0.013585	0.0437
N30-N39	8	1955	0.05605	-0.17	0.0693	impact	-0.156415	########
N30-N39	9	1915	0.05751	-0.28	0.0692	incidence	-0.3588	########
N30-N39	9	1915	0.05751	-0.28	0.0692	impact	-0.6388	########
N30-N39	9	1925	0.05751	-0.28	0.0692	incidence	-0.2139	########
N30-N39	9	1925	0.05751	-0.28	0.0692	impact	-0.4939	########
N30-N39	9	1935	0.05751	-0.28	0.0692	incidence	-0.06928	########
N30-N39	9	1935	0.05751	-0.28	0.0692	impact	-0.34928	########
N30-N39	9	1945	0.05751	-0.28	0.0692	incidence	0.000867	########
N30-N39	9	1945	0.05751	-0.28	0.0692	impact	-0.279133	########
N30-N39	9	1955	0.05751	-0.28	0.0692	incidence	0.101903	0.0431
N30-N39	9	1955	0.05751	-0.28	0.0692	impact	-0.178097	########
N30-N39	10	1915	0.05699	-0.318	0.0632	incidence	-0.32226	########
N30-N39	10	1915	0.05699	-0.318	0.0632	impact	-0.64026	########
N30-N39	10	1925	0.05699	-0.318	0.0632	incidence	-0.18056	########
N30-N39	10	1925	0.05699	-0.318	0.0632	impact	-0.49856	########
N30-N39	10	1935	0.05699	-0.318	0.0632	incidence	-0.14799	########
N30-N39	10	1935	0.05699	-0.318	0.0632	impact	-0.46599	########
N30-N39	10	1945	0.05699	-0.318	0.0632	incidence	0.026327	########
N30-N39	10	1945	0.05699	-0.318	0.0632	impact	-0.291673	########
N30-N39	10	1955	0.05699	-0.318	0.0632	incidence	0.086963	0.0406
N30-N39	10	1955	0.05699	-0.318	0.0632	impact	-0.231037	########
K20-K31	1	1915	0.03766	-0.0496	0.0955	incidence	0.28819	########
K20-K31	1	1915	0.03766	-0.0496	0.0955	impact	0.23859	########
K20-K31	1	1925	0.03766	-0.0496	0.0955	incidence	0.29246	########
K20-K31	1	1925	0.03766	-0.0496	0.0955	impact	0.24286	########
K20-K31	1	1935	0.03766	-0.0496	0.0955	incidence	0.30091	########
K20-K31	1	1935	0.03766	-0.0496	0.0955	impact	0.25131	########
K20-K31	1	1945	0.03766	-0.0496	0.0955	incidence	0.350702	########
K20-K31	1	1945	0.03766	-0.0496	0.0955	impact	0.301102	########
K20-K31	1	1955	0.03766	-0.0496	0.0955	incidence	0.267258	0.0417
K20-K31	1	1955	0.03766	-0.0496	0.0955	impact	0.217658	########
K20-K31	2	1915	0.04783	-0.106	0.1136	incidence	0.33064	########
K20-K31	2	1915	0.04783	-0.106	0.1136	impact	0.22464	########
K20-K31	2	1925	0.04783	-0.106	0.1136	incidence	0.31824	########
K20-K31	2	1925	0.04783	-0.106	0.1136	impact	0.21224	########
K20-K31	2	1935	0.04783	-0.106	0.1136	incidence	0.33575	########
K20-K31	2	1935	0.04783	-0.106	0.1136	impact	0.22975	########
K20-K31	2	1945	0.04783	-0.106	0.1136	incidence	0.376857	########
K20-K31	2	1945	0.04783	-0.106	0.1136	impact	0.270857	########
K20-K31	2	1955	0.04783	-0.106	0.1136	incidence	0.294223	0.0353

K20-K31	2	1955	0.04783	-0.106	0.1136	impact	0.188223	########
K20-K31	3	1915	0.04096	0.0126	0.1419	incidence	0.24583	########
K20-K31	3	1915	0.04096	0.0126	0.1419	impact	0.25843	########
K20-K31	3	1925	0.04096	0.0126	0.1419	incidence	0.37706	########
K20-K31	3	1925	0.04096	0.0126	0.1419	impact	0.38966	########
K20-K31	3	1935	0.04096	0.0126	0.1419	incidence	0.39748	########
K20-K31	3	1935	0.04096	0.0126	0.1419	impact	0.41008	########
K20-K31	3	1945	0.04096	0.0126	0.1419	incidence	0.465997	########
K20-K31	3	1945	0.04096	0.0126	0.1419	impact	0.478597	########
K20-K31	3	1955	0.04096	0.0126	0.1419	incidence	0.365913	0.0359
K20-K31	3	1955	0.04096	0.0126	0.1419	impact	0.378513	########
K20-K31	4	1915	0.04399	0.388	0.1158	incidence	0.39214	########
K20-K31	4	1915	0.04399	0.388	0.1158	impact	0.78014	########
K20-K31	4	1925	0.04399	0.388	0.1158	incidence	0.37082	########
K20-K31	4	1925	0.04399	0.388	0.1158	impact	0.75882	########
K20-K31	4	1935	0.04399	0.388	0.1158	incidence	0.47949	########
K20-K31	4	1935	0.04399	0.388	0.1158	impact	0.86749	########
K20-K31	4	1945	0.04399	0.388	0.1158	incidence	0.420007	########
K20-K31	4	1945	0.04399	0.388	0.1158	impact	0.808007	########
K20-K31	4	1955	0.04399	0.388	0.1158	incidence	0.350973	0.0356
K20-K31	4	1955	0.04399	0.388	0.1158	impact	0.738973	########
K20-K31	5	1915	0.04809	0.0629	0.1036	incidence	0.20382	########
K20-K31	5	1915	0.04809	0.0629	0.1036	impact	0.26672	########
K20-K31	5	1925	0.04809	0.0629	0.1036	incidence	0.29443	########
K20-K31	5	1925	0.04809	0.0629	0.1036	impact	0.35733	########
K20-K31	5	1935	0.04809	0.0629	0.1036	incidence	0.38529	########
K20-K31	5	1935	0.04809	0.0629	0.1036	impact	0.44819	########
K20-K31	5	1945	0.04809	0.0629	0.1036	incidence	0.496742	########
K20-K31	5	1945	0.04809	0.0629	0.1036	impact	0.559642	########
K20-K31	5	1955	0.04809	0.0629	0.1036	incidence	0.352678	0.0332
K20-K31	5	1955	0.04809	0.0629	0.1036	impact	0.415578	########
K20-K31	6	1915	0.05308	0.107	0.0414	incidence	0.30135	########
K20-K31	6	1915	0.05308	0.107	0.0414	impact	0.40835	########
K20-K31	6	1925	0.05308	0.107	0.0414	incidence	0.34704	########
K20-K31	6	1925	0.05308	0.107	0.0414	impact	0.45404	########
K20-K31	6	1935	0.05308	0.107	0.0414	incidence	0.30595	########
K20-K31	6	1935	0.05308	0.107	0.0414	impact	0.41295	########
K20-K31	6	1945	0.05308	0.107	0.0414	incidence	0.403242	########
K20-K31	6	1945	0.05308	0.107	0.0414	impact	0.510242	########
K20-K31	6	1955	0.05308	0.107	0.0414	incidence	0.322328	0.0319
K20-K31	6	1955	0.05308	0.107	0.0414	impact	0.429328	########
K20-K31	7	1915	0.05566	0.0204	0.101	incidence	0.3267	########
K20-K31	7	1915	0.05566	0.0204	0.101	impact	0.3471	########
K20-K31	7	1925	0.05566	0.0204	0.101	incidence	0.2779	########
K20-K31	7	1925	0.05566	0.0204	0.101	impact	0.2983	########
K20-K31	7	1935	0.05566	0.0204	0.101	incidence	0.33179	########
K20-K31	7	1935	0.05566	0.0204	0.101	impact	0.35219	########
K20-K31	7	1945	0.05566	0.0204	0.101	incidence	0.436694	########
K20-K31	7	1945	0.05566	0.0204	0.101	impact	0.457094	########
K20-K31	7	1955	0.05566	0.0204	0.101	incidence	0.289526	0.0311
K20-K31	7	1955	0.05566	0.0204	0.101	impact	0.309926	########
K20-K31	8	1915	0.0597	0.16	0.1355	incidence	0.3168	########
K20-K31	8	1915	0.0597	0.16	0.1355	impact	0.4768	########
K20-K31	8	1925	0.0597	0.16	0.1355	incidence	0.32144	########
K20-K31	8	1925	0.0597	0.16	0.1355	impact	0.48144	########
K20-K31	8	1935	0.0597	0.16	0.1355	incidence	0.33486	########

For peer review only

K20-K31	8	1935	0.0597	0.16	0.1355	impact	0.49486	########
K20-K31	8	1945	0.0597	0.16	0.1355	incidence	0.3594	########
K20-K31	8	1945	0.0597	0.16	0.1355	impact	0.5194	########
K20-K31	8	1955	0.0597	0.16	0.1355	incidence	0.31297	0.0295
K20-K31	8	1955	0.0597	0.16	0.1355	impact	0.47297	########
K20-K31	9	1915	0.06451	0.176	0.086	incidence	0.33447	########
K20-K31	9	1915	0.06451	0.176	0.086	impact	0.51047	########
K20-K31	9	1925	0.06451	0.176	0.086	incidence	0.33696	########
K20-K31	9	1925	0.06451	0.176	0.086	impact	0.51296	########
K20-K31	9	1935	0.06451	0.176	0.086	incidence	0.2979	########
K20-K31	9	1935	0.06451	0.176	0.086	impact	0.4739	########
K20-K31	9	1945	0.06451	0.176	0.086	incidence	0.360789	########
K20-K31	9	1945	0.06451	0.176	0.086	impact	0.536789	########
K20-K31	9	1955	0.06451	0.176	0.086	incidence	0.281461	0.028
K20-K31	9	1955	0.06451	0.176	0.086	impact	0.457461	########
K20-K31	10	1915	0.07311	0.282	0.0986	incidence	0.40081	########
K20-K31	10	1915	0.07311	0.282	0.0986	impact	0.68281	########
K20-K31	10	1925	0.07311	0.282	0.0986	incidence	0.25662	########
K20-K31	10	1925	0.07311	0.282	0.0986	impact	0.53862	########
K20-K31	10	1935	0.07311	0.282	0.0986	incidence	0.23166	########
K20-K31	10	1935	0.07311	0.282	0.0986	impact	0.51366	########
K20-K31	10	1945	0.07311	0.282	0.0986	incidence	0.321451	########
K20-K31	10	1945	0.07311	0.282	0.0986	impact	0.603451	########
K20-K31	10	1955	0.07311	0.282	0.0986	incidence	0.252879	0.026
K20-K31	10	1955	0.07311	0.282	0.0986	impact	0.534879	########
J20-J22	1	1915	0.03207	-0.316	0.1645	incidence	-0.10658	########
J20-J22	1	1915	0.03207	-0.316	0.1645	impact	-0.42258	########
J20-J22	1	1925	0.03207	-0.316	0.1645	incidence	0.07548	########
J20-J22	1	1925	0.03207	-0.316	0.1645	impact	-0.24052	########
J20-J22	1	1935	0.03207	-0.316	0.1645	incidence	-0.01256	########
J20-J22	1	1935	0.03207	-0.316	0.1645	impact	-0.32856	########
J20-J22	1	1945	0.03207	-0.316	0.1645	incidence	-0.0302	########
J20-J22	1	1945	0.03207	-0.316	0.1645	impact	-0.3462	########
J20-J22	1	1955	0.03207	-0.316	0.1645	incidence	-0.02538	0.1033
J20-J22	1	1955	0.03207	-0.316	0.1645	impact	-0.34138	########
J20-J22	2	1915	0.03618	-0.221	0.0764	incidence	-0.36493	########
J20-J22	2	1915	0.03618	-0.221	0.0764	impact	-0.58593	########
J20-J22	2	1925	0.03618	-0.221	0.0764	incidence	0.06369	########
J20-J22	2	1925	0.03618	-0.221	0.0764	impact	-0.15731	########
J20-J22	2	1935	0.03618	-0.221	0.0764	incidence	0.07008	########
J20-J22	2	1935	0.03618	-0.221	0.0764	impact	-0.15092	########
J20-J22	2	1945	0.03618	-0.221	0.0764	incidence	-0.099865	########
J20-J22	2	1945	0.03618	-0.221	0.0764	impact	-0.320865	########
J20-J22	2	1955	0.03618	-0.221	0.0764	incidence	-0.217615	0.0884
J20-J22	2	1955	0.03618	-0.221	0.0764	impact	-0.438615	########
J20-J22	3	1915	0.03897	-0.0651	0.0942	incidence	-0.30909	########
J20-J22	3	1915	0.03897	-0.0651	0.0942	impact	-0.37419	########
J20-J22	3	1925	0.03897	-0.0651	0.0942	incidence	0.03984	########
J20-J22	3	1925	0.03897	-0.0651	0.0942	impact	-0.02526	########
J20-J22	3	1935	0.03897	-0.0651	0.0942	incidence	0.05339	########
J20-J22	3	1935	0.03897	-0.0651	0.0942	impact	-0.01171	########
J20-J22	3	1945	0.03897	-0.0651	0.0942	incidence	-0.153593	########
J20-J22	3	1945	0.03897	-0.0651	0.0942	impact	-0.218693	########
J20-J22	3	1955	0.03897	-0.0651	0.0942	incidence	-0.321427	0.084
J20-J22	3	1955	0.03897	-0.0651	0.0942	impact	-0.386527	########
J20-J22	4	1915	0.04249	-0.236	0.0827	incidence	-0.20901	########

For peer review only

J20-J22	4	1915	0.04249	-0.236	0.0827	impact	-0.44501	########
J20-J22	4	1925	0.04249	-0.236	0.0827	incidence	0.002	########
J20-J22	4	1925	0.04249	-0.236	0.0827	impact	-0.234	########
J20-J22	4	1935	0.04249	-0.236	0.0827	incidence	0.06139	########
J20-J22	4	1935	0.04249	-0.236	0.0827	impact	-0.17461	########
J20-J22	4	1945	0.04249	-0.236	0.0827	incidence	-0.020366	########
J20-J22	4	1945	0.04249	-0.236	0.0827	impact	-0.256366	########
J20-J22	4	1955	0.04249	-0.236	0.0827	incidence	-0.182524	0.08
J20-J22	4	1955	0.04249	-0.236	0.0827	impact	-0.418524	########
J20-J22	5	1915	0.04629	-0.169	0.1004	incidence	-0.16017	########
J20-J22	5	1915	0.04629	-0.169	0.1004	impact	-0.32917	########
J20-J22	5	1925	0.04629	-0.169	0.1004	incidence	-0.03276	########
J20-J22	5	1925	0.04629	-0.169	0.1004	impact	-0.20176	########
J20-J22	5	1935	0.04629	-0.169	0.1004	incidence	0.07003	########
J20-J22	5	1935	0.04629	-0.169	0.1004	impact	-0.09897	########
J20-J22	5	1945	0.04629	-0.169	0.1004	incidence	-0.09983	########
J20-J22	5	1945	0.04629	-0.169	0.1004	impact	-0.26883	########
J20-J22	5	1955	0.04629	-0.169	0.1004	incidence	-0.19055	0.0778
J20-J22	5	1955	0.04629	-0.169	0.1004	impact	-0.35955	########
J20-J22	6	1915	0.05025	-0.175	0.1064	incidence	-0.26043	########
J20-J22	6	1915	0.05025	-0.175	0.1064	impact	-0.43543	########
J20-J22	6	1925	0.05025	-0.175	0.1064	incidence	-0.06757	########
J20-J22	6	1925	0.05025	-0.175	0.1064	impact	-0.24257	########
J20-J22	6	1935	0.05025	-0.175	0.1064	incidence	-0.01151	########
J20-J22	6	1935	0.05025	-0.175	0.1064	impact	-0.18651	########
J20-J22	6	1945	0.05025	-0.175	0.1064	incidence	-0.045587	########
J20-J22	6	1945	0.05025	-0.175	0.1064	impact	-0.220587	########
J20-J22	6	1955	0.05025	-0.175	0.1064	incidence	-0.083313	0.0722
J20-J22	6	1955	0.05025	-0.175	0.1064	impact	-0.258313	########
J20-J22	7	1915	0.05574	-0.108	0.0707	incidence	-0.22184	########
J20-J22	7	1915	0.05574	-0.108	0.0707	impact	-0.32984	########
J20-J22	7	1925	0.05574	-0.108	0.0707	incidence	-0.05684	########
J20-J22	7	1925	0.05574	-0.108	0.0707	impact	-0.16484	########
J20-J22	7	1935	0.05574	-0.108	0.0707	incidence	0.04976	########
J20-J22	7	1935	0.05574	-0.108	0.0707	impact	-0.05824	########
J20-J22	7	1945	0.05574	-0.108	0.0707	incidence	-0.062303	########
J20-J22	7	1945	0.05574	-0.108	0.0707	impact	-0.170303	########
J20-J22	7	1955	0.05574	-0.108	0.0707	incidence	-0.201677	0.0652
J20-J22	7	1955	0.05574	-0.108	0.0707	impact	-0.309677	########
J20-J22	8	1915	0.05951	0.143	0.0706	incidence	-0.09538	########
J20-J22	8	1915	0.05951	0.143	0.0706	impact	0.04762	########
J20-J22	8	1925	0.05951	0.143	0.0706	incidence	-0.1151	########
J20-J22	8	1925	0.05951	0.143	0.0706	impact	0.0279	########
J20-J22	8	1935	0.05951	0.143	0.0706	incidence	0.03413	########
J20-J22	8	1935	0.05951	0.143	0.0706	impact	0.17713	########
J20-J22	8	1945	0.05951	0.143	0.0706	incidence	-0.03313	########
J20-J22	8	1945	0.05951	0.143	0.0706	impact	0.10987	########
J20-J22	8	1955	0.05951	0.143	0.0706	incidence	-0.20837	0.0594
J20-J22	8	1955	0.05951	0.143	0.0706	impact	-0.06537	########
J20-J22	9	1915	0.06303	-0.165	0.0647	incidence	-0.37625	########
J20-J22	9	1915	0.06303	-0.165	0.0647	impact	-0.54125	########
J20-J22	9	1925	0.06303	-0.165	0.0647	incidence	-0.07467	########
J20-J22	9	1925	0.06303	-0.165	0.0647	impact	-0.23967	########
J20-J22	9	1935	0.06303	-0.165	0.0647	incidence	-0.06752	########
J20-J22	9	1935	0.06303	-0.165	0.0647	impact	-0.23252	########
J20-J22	9	1945	0.06303	-0.165	0.0647	incidence	-0.12571	########

For peer review only

J20-J22	9	1945	0.06303	-0.165	0.0647	impact	-0.29071	########
J20-J22	9	1955	0.06303	-0.165	0.0647	incidence	-0.22928	0.0566
J20-J22	9	1955	0.06303	-0.165	0.0647	impact	-0.39428	########
J20-J22	10	1915	0.07659	-0.0308	0.1224	incidence	-0.16761	########
J20-J22	10	1915	0.07659	-0.0308	0.1224	impact	-0.19841	########
J20-J22	10	1925	0.07659	-0.0308	0.1224	incidence	-0.22863	########
J20-J22	10	1925	0.07659	-0.0308	0.1224	impact	-0.25943	########
J20-J22	10	1935	0.07659	-0.0308	0.1224	incidence	-0.09969	########
J20-J22	10	1935	0.07659	-0.0308	0.1224	impact	-0.13049	########
J20-J22	10	1945	0.07659	-0.0308	0.1224	incidence	-0.055496	########
J20-J22	10	1945	0.07659	-0.0308	0.1224	impact	-0.086296	########
J20-J22	10	1955	0.07659	-0.0308	0.1224	incidence	-0.188504	0.0503
J20-J22	10	1955	0.07659	-0.0308	0.1224	impact	-0.219304	########
S70-S79	1	1915	0.03132	0.257	0.148	incidence	0.16711	########
S70-S79	1	1915	0.03132	0.257	0.148	impact	0.42411	########
S70-S79	1	1925	0.03132	0.257	0.148	incidence	0.23069	########
S70-S79	1	1925	0.03132	0.257	0.148	impact	0.48769	########
S70-S79	1	1935	0.03132	0.257	0.148	incidence	0.28451	########
S70-S79	1	1935	0.03132	0.257	0.148	impact	0.54151	########
S70-S79	1	1945	0.03132	0.257	0.148	incidence	0.143054	########
S70-S79	1	1945	0.03132	0.257	0.148	impact	0.400054	########
S70-S79	1	1955	0.03132	0.257	0.148	incidence	-0.224944	0.1331
S70-S79	1	1955	0.03132	0.257	0.148	impact	0.032056	########
S70-S79	2	1915	0.03294	-0.027	0.0959	incidence	0.02873	########
S70-S79	2	1915	0.03294	-0.027	0.0959	impact	0.00173	########
S70-S79	2	1925	0.03294	-0.027	0.0959	incidence	0.32238	########
S70-S79	2	1925	0.03294	-0.027	0.0959	impact	0.29538	########
S70-S79	2	1935	0.03294	-0.027	0.0959	incidence	0.29486	########
S70-S79	2	1935	0.03294	-0.027	0.0959	impact	0.26786	########
S70-S79	2	1945	0.03294	-0.027	0.0959	incidence	0.142019	########
S70-S79	2	1945	0.03294	-0.027	0.0959	impact	0.115019	########
S70-S79	2	1955	0.03294	-0.027	0.0959	incidence	-0.140299	0.1356
S70-S79	2	1955	0.03294	-0.027	0.0959	impact	-0.167299	########
S70-S79	3	1915	0.03598	0.0994	0.1019	incidence	0.07825	########
S70-S79	3	1915	0.03598	0.0994	0.1019	impact	0.17765	########
S70-S79	3	1925	0.03598	0.0994	0.1019	incidence	0.26675	########
S70-S79	3	1925	0.03598	0.0994	0.1019	impact	0.36615	########
S70-S79	3	1935	0.03598	0.0994	0.1019	incidence	0.29044	########
S70-S79	3	1935	0.03598	0.0994	0.1019	impact	0.38984	########
S70-S79	3	1945	0.03598	0.0994	0.1019	incidence	0.019728	########
S70-S79	3	1945	0.03598	0.0994	0.1019	impact	0.119128	########
S70-S79	3	1955	0.03598	0.0994	0.1019	incidence	0.136512	0.1215
S70-S79	3	1955	0.03598	0.0994	0.1019	impact	0.235912	########
S70-S79	4	1915	0.03695	-0.219	0.1176	incidence	0.08103	########
S70-S79	4	1915	0.03695	-0.219	0.1176	impact	-0.13797	########
S70-S79	4	1925	0.03695	-0.219	0.1176	incidence	0.28882	########
S70-S79	4	1925	0.03695	-0.219	0.1176	impact	0.06982	########
S70-S79	4	1935	0.03695	-0.219	0.1176	incidence	0.29131	########
S70-S79	4	1935	0.03695	-0.219	0.1176	impact	0.07231	########
S70-S79	4	1945	0.03695	-0.219	0.1176	incidence	0.13551	########
S70-S79	4	1945	0.03695	-0.219	0.1176	impact	-0.08349	########
S70-S79	4	1955	0.03695	-0.219	0.1176	incidence	0.05755	0.119
S70-S79	4	1955	0.03695	-0.219	0.1176	impact	-0.16145	########
S70-S79	5	1915	0.03831	-0.0924	0.0706	incidence	0.02549	########
S70-S79	5	1915	0.03831	-0.0924	0.0706	impact	-0.06691	########
S70-S79	5	1925	0.03831	-0.0924	0.0706	incidence	0.26299	########

For peer review only

S70-S79	5	1925	0.03831	-0.0924	0.0706	impact	0.17059	########
S70-S79	5	1935	0.03831	-0.0924	0.0706	incidence	0.2289	########
S70-S79	5	1935	0.03831	-0.0924	0.0706	impact	0.1365	########
S70-S79	5	1945	0.03831	-0.0924	0.0706	incidence	0.112045	########
S70-S79	5	1945	0.03831	-0.0924	0.0706	impact	0.019645	########
S70-S79	5	1955	0.03831	-0.0924	0.0706	incidence	0.006325	0.1095
S70-S79	5	1955	0.03831	-0.0924	0.0706	impact	-0.086075	########
S70-S79	6	1915	0.03745	-0.122	0.0613	incidence	-0.00678	########
S70-S79	6	1915	0.03745	-0.122	0.0613	impact	-0.12878	########
S70-S79	6	1925	0.03745	-0.122	0.0613	incidence	0.21877	########
S70-S79	6	1925	0.03745	-0.122	0.0613	impact	0.09677	########
S70-S79	6	1935	0.03745	-0.122	0.0613	incidence	0.33895	########
S70-S79	6	1935	0.03745	-0.122	0.0613	impact	0.21695	########
S70-S79	6	1945	0.03745	-0.122	0.0613	incidence	0.075513	########
S70-S79	6	1945	0.03745	-0.122	0.0613	impact	-0.046487	########
S70-S79	6	1955	0.03745	-0.122	0.0613	incidence	0.185827	0.1062
S70-S79	6	1955	0.03745	-0.122	0.0613	impact	0.063827	########
S70-S79	7	1915	0.04192	0.157	0.1052	incidence	0.05741	########
S70-S79	7	1915	0.04192	0.157	0.1052	impact	0.21441	########
S70-S79	7	1925	0.04192	0.157	0.1052	incidence	0.15137	########
S70-S79	7	1925	0.04192	0.157	0.1052	impact	0.30837	########
S70-S79	7	1935	0.04192	0.157	0.1052	incidence	0.19178	########
S70-S79	7	1935	0.04192	0.157	0.1052	impact	0.34878	########
S70-S79	7	1945	0.04192	0.157	0.1052	incidence	0.04088	########
S70-S79	7	1945	0.04192	0.157	0.1052	impact	0.19788	########
S70-S79	7	1955	0.04192	0.157	0.1052	incidence	-0.00234	0.0994
S70-S79	7	1955	0.04192	0.157	0.1052	impact	0.15466	########
S70-S79	8	1915	0.04218	-0.0917	0.1111	incidence	-0.0055	########
S70-S79	8	1915	0.04218	-0.0917	0.1111	impact	-0.0972	########
S70-S79	8	1925	0.04218	-0.0917	0.1111	incidence	0.12344	########
S70-S79	8	1925	0.04218	-0.0917	0.1111	impact	0.03174	########
S70-S79	8	1935	0.04218	-0.0917	0.1111	incidence	0.17133	########
S70-S79	8	1935	0.04218	-0.0917	0.1111	impact	0.07963	########
S70-S79	8	1945	0.04218	-0.0917	0.1111	incidence	0.142365	########
S70-S79	8	1945	0.04218	-0.0917	0.1111	impact	0.050665	########
S70-S79	8	1955	0.04218	-0.0917	0.1111	incidence	0.137565	0.0955
S70-S79	8	1955	0.04218	-0.0917	0.1111	impact	0.045865	########
S70-S79	9	1915	0.04231	0.00191	0.1198	incidence	0.03261	########
S70-S79	9	1915	0.04231	0.00191	0.1198	impact	0.03452	########
S70-S79	9	1925	0.04231	0.00191	0.1198	incidence	0.15828	########
S70-S79	9	1925	0.04231	0.00191	0.1198	impact	0.16019	########
S70-S79	9	1935	0.04231	0.00191	0.1198	incidence	0.25988	########
S70-S79	9	1935	0.04231	0.00191	0.1198	impact	0.26179	########
S70-S79	9	1945	0.04231	0.00191	0.1198	incidence	0.186267	########
S70-S79	9	1945	0.04231	0.00191	0.1198	impact	0.188177	########
S70-S79	9	1955	0.04231	0.00191	0.1198	incidence	0.051193	0.093
S70-S79	9	1955	0.04231	0.00191	0.1198	impact	0.053103	########
S70-S79	10	1915	0.04409	-0.178	0.1147	incidence	0.04348	########
S70-S79	10	1915	0.04409	-0.178	0.1147	impact	-0.13452	########
S70-S79	10	1925	0.04409	-0.178	0.1147	incidence	0.10887	########
S70-S79	10	1925	0.04409	-0.178	0.1147	impact	-0.06913	########
S70-S79	10	1935	0.04409	-0.178	0.1147	incidence	0.17962	########
S70-S79	10	1935	0.04409	-0.178	0.1147	impact	0.00162	########
S70-S79	10	1945	0.04409	-0.178	0.1147	incidence	0.205817	########
S70-S79	10	1945	0.04409	-0.178	0.1147	impact	0.027817	########
S70-S79	10	1955	0.04409	-0.178	0.1147	incidence	0.145173	0.0812

For peer review only

S70-S79	10	1955	0.04409	-0.178	0.1147	impact	-0.032827	########
A30-A49	1	1915	0.02452	0.324	0.2369	incidence	-0.36484	########
A30-A49	1	1915	0.02452	0.324	0.2369	impact	-0.04084	########
A30-A49	1	1925	0.02452	0.324	0.2369	incidence	-0.12572	########
A30-A49	1	1925	0.02452	0.324	0.2369	impact	0.19828	########
A30-A49	1	1935	0.02452	0.324	0.2369	incidence	-0.23588	########
A30-A49	1	1935	0.02452	0.324	0.2369	impact	0.08812	########
A30-A49	1	1945	0.02452	0.324	0.2369	incidence	-0.523434	########
A30-A49	1	1945	0.02452	0.324	0.2369	impact	-0.199434	########
A30-A49	1	1955	0.02452	0.324	0.2369	incidence	-0.358476	0.1331
A30-A49	1	1955	0.02452	0.324	0.2369	impact	-0.034476	########
A30-A49	2	1915	0.02561	-0.304	0.2255	incidence	-0.70373	########
A30-A49	2	1915	0.02561	-0.304	0.2255	impact	-1.00773	########
A30-A49	2	1925	0.02561	-0.304	0.2255	incidence	-0.19828	########
A30-A49	2	1925	0.02561	-0.304	0.2255	impact	-0.50228	########
A30-A49	2	1935	0.02561	-0.304	0.2255	incidence	-0.31638	########
A30-A49	2	1935	0.02561	-0.304	0.2255	impact	-0.62038	########
A30-A49	2	1945	0.02561	-0.304	0.2255	incidence	-0.562626	########
A30-A49	2	1945	0.02561	-0.304	0.2255	impact	-0.866626	########
A30-A49	2	1955	0.02561	-0.304	0.2255	incidence	-0.571574	0.1142
A30-A49	2	1955	0.02561	-0.304	0.2255	impact	-0.875574	########
A30-A49	3	1915	0.02707	-0.328	0.2641	incidence	-0.46163	########
A30-A49	3	1915	0.02707	-0.328	0.2641	impact	-0.78963	########
A30-A49	3	1925	0.02707	-0.328	0.2641	incidence	-0.40633	########
A30-A49	3	1925	0.02707	-0.328	0.2641	impact	-0.73433	########
A30-A49	3	1935	0.02707	-0.328	0.2641	incidence	-0.27809	########
A30-A49	3	1935	0.02707	-0.328	0.2641	impact	-0.60609	########
A30-A49	3	1945	0.02707	-0.328	0.2641	incidence	-0.176485	########
A30-A49	3	1945	0.02707	-0.328	0.2641	impact	-0.504485	########
A30-A49	3	1955	0.02707	-0.328	0.2641	incidence	-0.320815	0.118
A30-A49	3	1955	0.02707	-0.328	0.2641	impact	-0.648815	########
A30-A49	4	1915	0.02927	-0.286	0.1399	incidence	-0.51216	########
A30-A49	4	1915	0.02927	-0.286	0.1399	impact	-0.79816	########
A30-A49	4	1925	0.02927	-0.286	0.1399	incidence	-0.43985	########
A30-A49	4	1925	0.02927	-0.286	0.1399	impact	-0.72585	########
A30-A49	4	1935	0.02927	-0.286	0.1399	incidence	-0.34437	########
A30-A49	4	1935	0.02927	-0.286	0.1399	impact	-0.63037	########
A30-A49	4	1945	0.02927	-0.286	0.1399	incidence	-0.499051	########
A30-A49	4	1945	0.02927	-0.286	0.1399	impact	-0.785051	########
A30-A49	4	1955	0.02927	-0.286	0.1399	incidence	-0.414419	0.1088
A30-A49	4	1955	0.02927	-0.286	0.1399	impact	-0.700419	########
A30-A49	5	1915	0.03087	0.423	0.2242	incidence	-0.67625	########
A30-A49	5	1915	0.03087	0.423	0.2242	impact	-0.25325	########
A30-A49	5	1925	0.03087	0.423	0.2242	incidence	-0.49728	########
A30-A49	5	1925	0.03087	0.423	0.2242	impact	-0.07428	########
A30-A49	5	1935	0.03087	0.423	0.2242	incidence	-0.36428	########
A30-A49	5	1935	0.03087	0.423	0.2242	impact	0.05872	########
A30-A49	5	1945	0.03087	0.423	0.2242	incidence	-0.478934	########
A30-A49	5	1945	0.03087	0.423	0.2242	impact	-0.055934	########
A30-A49	5	1955	0.03087	0.423	0.2242	incidence	-0.336496	0.106
A30-A49	5	1955	0.03087	0.423	0.2242	impact	0.086504	########
A30-A49	6	1915	0.03366	-0.608	0.1778	incidence	-0.59255	########
A30-A49	6	1915	0.03366	-0.608	0.1778	impact	-1.20055	########
A30-A49	6	1925	0.03366	-0.608	0.1778	incidence	-0.56719	########
A30-A49	6	1925	0.03366	-0.608	0.1778	impact	-1.17519	########
A30-A49	6	1935	0.03366	-0.608	0.1778	incidence	-0.40407	########

For peer review only

A30-A49	6	1935	0.03366	-0.608	0.1778	impact	-1.01207	########
A30-A49	6	1945	0.03366	-0.608	0.1778	incidence	-0.479749	########
A30-A49	6	1945	0.03366	-0.608	0.1778	impact	-1.08775	########
A30-A49	6	1955	0.03366	-0.608	0.1778	incidence	-0.521711	0.1011
A30-A49	6	1955	0.03366	-0.608	0.1778	impact	-1.12971	########
A30-A49	7	1915	0.03746	0.531	0.3313	incidence	-0.44288	########
A30-A49	7	1915	0.03746	0.531	0.3313	impact	0.08812	########
A30-A49	7	1925	0.03746	0.531	0.3313	incidence	-0.45213	########
A30-A49	7	1925	0.03746	0.531	0.3313	impact	0.07887	########
A30-A49	7	1935	0.03746	0.531	0.3313	incidence	-0.39859	########
A30-A49	7	1935	0.03746	0.531	0.3313	impact	0.13241	########
A30-A49	7	1945	0.03746	0.531	0.3313	incidence	-0.459816	########
A30-A49	7	1945	0.03746	0.531	0.3313	impact	0.071184	########
A30-A49	7	1955	0.03746	0.531	0.3313	incidence	-0.424244	0.0936
A30-A49	7	1955	0.03746	0.531	0.3313	impact	0.106756	########
A30-A49	8	1915	0.03675	0.366	0.1353	incidence	-0.462	########
A30-A49	8	1915	0.03675	0.366	0.1353	impact	-0.096	########
A30-A49	8	1925	0.03675	0.366	0.1353	incidence	-0.56972	########
A30-A49	8	1925	0.03675	0.366	0.1353	impact	-0.20372	########
A30-A49	8	1935	0.03675	0.366	0.1353	incidence	-0.41916	########
A30-A49	8	1935	0.03675	0.366	0.1353	impact	-0.05316	########
A30-A49	8	1945	0.03675	0.366	0.1353	incidence	-0.405884	########
A30-A49	8	1945	0.03675	0.366	0.1353	impact	-0.039884	########
A30-A49	8	1955	0.03675	0.366	0.1353	incidence	-0.395756	0.0917
A30-A49	8	1955	0.03675	0.366	0.1353	impact	-0.029756	########
A30-A49	9	1915	0.03863	0.134	0.1894	incidence	-0.53571	########
A30-A49	9	1915	0.03863	0.134	0.1894	impact	-0.40171	########
A30-A49	9	1925	0.03863	0.134	0.1894	incidence	-0.47106	########
A30-A49	9	1925	0.03863	0.134	0.1894	impact	-0.33706	########
A30-A49	9	1935	0.03863	0.134	0.1894	incidence	-0.49158	########
A30-A49	9	1935	0.03863	0.134	0.1894	impact	-0.35758	########
A30-A49	9	1945	0.03863	0.134	0.1894	incidence	-0.509548	########
A30-A49	9	1945	0.03863	0.134	0.1894	impact	-0.375548	########
A30-A49	9	1955	0.03863	0.134	0.1894	incidence	-0.486552	0.0863
A30-A49	9	1955	0.03863	0.134	0.1894	impact	-0.352552	########
A30-A49	10	1915	0.03867	-0.218	0.1681	incidence	-0.47161	########
A30-A49	10	1915	0.03867	-0.218	0.1681	impact	-0.68961	########
A30-A49	10	1925	0.03867	-0.218	0.1681	incidence	-0.46231	########
A30-A49	10	1925	0.03867	-0.218	0.1681	impact	-0.68031	########
A30-A49	10	1935	0.03867	-0.218	0.1681	incidence	-0.43353	########
A30-A49	10	1935	0.03867	-0.218	0.1681	impact	-0.65153	########
A30-A49	10	1945	0.03867	-0.218	0.1681	incidence	-0.321208	########
A30-A49	10	1945	0.03867	-0.218	0.1681	impact	-0.539208	########
A30-A49	10	1955	0.03867	-0.218	0.1681	incidence	-0.421432	0.0856
A30-A49	10	1955	0.03867	-0.218	0.1681	impact	-0.639432	########
T80-T88	1	1915	0.02975	0.189	0.248	incidence	0.05126	########
T80-T88	1	1915	0.02975	0.189	0.248	impact	0.24026	########
T80-T88	1	1925	0.02975	0.189	0.248	incidence	0.00497	########
T80-T88	1	1925	0.02975	0.189	0.248	impact	0.19397	########
T80-T88	1	1935	0.02975	0.189	0.248	incidence	0.06191	########
T80-T88	1	1935	0.02975	0.189	0.248	impact	0.25091	########
T80-T88	1	1945	0.02975	0.189	0.248	incidence	0.108431	########
T80-T88	1	1945	0.02975	0.189	0.248	impact	0.297431	########
T80-T88	1	1955	0.02975	0.189	0.248	incidence	-0.050501	0.0587
T80-T88	1	1955	0.02975	0.189	0.248	impact	0.138499	########
T80-T88	2	1915	0.03112	0.0583	0.1508	incidence	-0.08057	########

For peer review only

T80-T88	2	1915	0.03112	0.0583	0.1508	impact	-0.02227	########
T80-T88	2	1925	0.03112	0.0583	0.1508	incidence	0.02571	########
T80-T88	2	1925	0.03112	0.0583	0.1508	impact	0.08401	########
T80-T88	2	1935	0.03112	0.0583	0.1508	incidence	0.05884	########
T80-T88	2	1935	0.03112	0.0583	0.1508	impact	0.11714	########
T80-T88	2	1945	0.03112	0.0583	0.1508	incidence	-0.018378	########
T80-T88	2	1945	0.03112	0.0583	0.1508	impact	0.039922	########
T80-T88	2	1955	0.03112	0.0583	0.1508	incidence	-0.191182	0.057
T80-T88	2	1955	0.03112	0.0583	0.1508	impact	-0.132882	########
T80-T88	3	1915	0.03032	0.0515	0.0582	incidence	0.09987	########
T80-T88	3	1915	0.03032	0.0515	0.0582	impact	0.15137	########
T80-T88	3	1925	0.03032	0.0515	0.0582	incidence	-0.03343	########
T80-T88	3	1925	0.03032	0.0515	0.0582	impact	0.01807	########
T80-T88	3	1935	0.03032	0.0515	0.0582	incidence	0.06758	########
T80-T88	3	1935	0.03032	0.0515	0.0582	impact	0.11908	########
T80-T88	3	1945	0.03032	0.0515	0.0582	incidence	-0.043718	########
T80-T88	3	1945	0.03032	0.0515	0.0582	impact	0.007782	########
T80-T88	3	1955	0.03032	0.0515	0.0582	incidence	-0.132902	0.0562
T80-T88	3	1955	0.03032	0.0515	0.0582	impact	-0.081402	########
T80-T88	4	1915	0.03201	0.201	0.1644	incidence	-0.03409	########
T80-T88	4	1915	0.03201	0.201	0.1644	impact	0.16691	########
T80-T88	4	1925	0.03201	0.201	0.1644	incidence	-0.01214	########
T80-T88	4	1925	0.03201	0.201	0.1644	impact	0.18886	########
T80-T88	4	1935	0.03201	0.201	0.1644	incidence	-0.07175	########
T80-T88	4	1935	0.03201	0.201	0.1644	impact	0.12925	########
T80-T88	4	1945	0.03201	0.201	0.1644	incidence	0.065428	########
T80-T88	4	1945	0.03201	0.201	0.1644	impact	0.266428	########
T80-T88	4	1955	0.03201	0.201	0.1644	incidence	-0.091398	0.0541
T80-T88	4	1955	0.03201	0.201	0.1644	impact	0.109602	########
T80-T88	5	1915	0.0326	0.172	0.0617	incidence	-0.29949	########
T80-T88	5	1915	0.0326	0.172	0.0617	impact	-0.12749	########
T80-T88	5	1925	0.0326	0.172	0.0617	incidence	0.00144	########
T80-T88	5	1925	0.0326	0.172	0.0617	impact	0.17344	########
T80-T88	5	1935	0.0326	0.172	0.0617	incidence	-0.00359	########
T80-T88	5	1935	0.0326	0.172	0.0617	impact	0.16841	########
T80-T88	5	1945	0.0326	0.172	0.0617	incidence	0.053988	########
T80-T88	5	1945	0.0326	0.172	0.0617	impact	0.225988	########
T80-T88	5	1955	0.0326	0.172	0.0617	incidence	-0.104788	0.0543
T80-T88	5	1955	0.0326	0.172	0.0617	impact	0.067212	########
T80-T88	6	1915	0.03287	0.434	0.1027	incidence	-0.12677	########
T80-T88	6	1915	0.03287	0.434	0.1027	impact	0.30723	########
T80-T88	6	1925	0.03287	0.434	0.1027	incidence	-0.06414	########
T80-T88	6	1925	0.03287	0.434	0.1027	impact	0.36986	########
T80-T88	6	1935	0.03287	0.434	0.1027	incidence	0.06617	########
T80-T88	6	1935	0.03287	0.434	0.1027	impact	0.50017	########
T80-T88	6	1945	0.03287	0.434	0.1027	incidence	0.010706	########
T80-T88	6	1945	0.03287	0.434	0.1027	impact	0.444706	########
T80-T88	6	1955	0.03287	0.434	0.1027	incidence	-0.068166	0.0522
T80-T88	6	1955	0.03287	0.434	0.1027	impact	0.365834	########
T80-T88	7	1915	0.03332	0.198	0.1504	incidence	0.02111	########
T80-T88	7	1915	0.03332	0.198	0.1504	impact	0.21911	########
T80-T88	7	1925	0.03332	0.198	0.1504	incidence	-0.0045	########
T80-T88	7	1925	0.03332	0.198	0.1504	impact	0.1935	########
T80-T88	7	1935	0.03332	0.198	0.1504	incidence	-0.04415	########
T80-T88	7	1935	0.03332	0.198	0.1504	impact	0.15385	########
T80-T88	7	1945	0.03332	0.198	0.1504	incidence	0.012467	########

For peer review only

T80-T88	7	1945	0.03332	0.198	0.1504	impact	0.210467	########
T80-T88	7	1955	0.03332	0.198	0.1504	incidence	-0.039247	0.0497
T80-T88	7	1955	0.03332	0.198	0.1504	impact	0.158753	########
T80-T88	8	1915	0.03245	0.261	0.1197	incidence	-0.10966	########
T80-T88	8	1915	0.03245	0.261	0.1197	impact	0.15134	########
T80-T88	8	1925	0.03245	0.261	0.1197	incidence	-0.03016	########
T80-T88	8	1925	0.03245	0.261	0.1197	impact	0.23084	########
T80-T88	8	1935	0.03245	0.261	0.1197	incidence	-0.01862	########
T80-T88	8	1935	0.03245	0.261	0.1197	impact	0.24238	########
T80-T88	8	1945	0.03245	0.261	0.1197	incidence	0.035	########
T80-T88	8	1945	0.03245	0.261	0.1197	impact	0.296	########
T80-T88	8	1955	0.03245	0.261	0.1197	incidence	-0.1278	0.0488
T80-T88	8	1955	0.03245	0.261	0.1197	impact	0.1332	########
T80-T88	9	1915	0.03284	2.14E-05	0.1148	incidence	-0.24529	########
T80-T88	9	1915	0.03284	2.14E-05	0.1148	impact	########	########
T80-T88	9	1925	0.03284	2.14E-05	0.1148	incidence	-0.14386	########
T80-T88	9	1925	0.03284	2.14E-05	0.1148	impact	########	########
T80-T88	9	1935	0.03284	2.14E-05	0.1148	incidence	0.04033	########
T80-T88	9	1935	0.03284	2.14E-05	0.1148	impact	########	########
T80-T88	9	1945	0.03284	2.14E-05	0.1148	incidence	-0.079979	########
T80-T88	9	1945	0.03284	2.14E-05	0.1148	impact	########	########
T80-T88	9	1955	0.03284	2.14E-05	0.1148	incidence	-0.188831	0.0485
T80-T88	9	1955	0.03284	2.14E-05	0.1148	impact	########	########
T80-T88	10	1915	0.03329	0.204	0.0876	incidence	0.04348	########
T80-T88	10	1915	0.03329	0.204	0.0876	impact	0.24748	########
T80-T88	10	1925	0.03329	0.204	0.0876	incidence	-0.06386	########
T80-T88	10	1925	0.03329	0.204	0.0876	impact	0.14014	########
T80-T88	10	1935	0.03329	0.204	0.0876	incidence	0.00435	########
T80-T88	10	1935	0.03329	0.204	0.0876	impact	0.20835	########
T80-T88	10	1945	0.03329	0.204	0.0876	incidence	0.01559	########
T80-T88	10	1945	0.03329	0.204	0.0876	impact	0.21959	########
T80-T88	10	1955	0.03329	0.204	0.0876	incidence	0.01201	0.0468
T80-T88	10	1955	0.03329	0.204	0.0876	impact	0.21601	########
N17-N19	1	1915	0.02326	0.0207	0.2501	incidence	-0.23299	########
N17-N19	1	1915	0.02326	0.0207	0.2501	impact	-0.21229	########
N17-N19	1	1925	0.02326	0.0207	0.2501	incidence	-0.00241	########
N17-N19	1	1925	0.02326	0.0207	0.2501	impact	0.01829	########
N17-N19	1	1935	0.02326	0.0207	0.2501	incidence	0.19947	########
N17-N19	1	1935	0.02326	0.0207	0.2501	impact	0.22017	########
N17-N19	1	1945	0.02326	0.0207	0.2501	incidence	-0.037397	########
N17-N19	1	1945	0.02326	0.0207	0.2501	impact	-0.016697	########
N17-N19	1	1955	0.02326	0.0207	0.2501	incidence	0.083387	0.1503
N17-N19	1	1955	0.02326	0.0207	0.2501	impact	0.104087	########
N17-N19	2	1915	0.02443	-0.0883	0.2134	incidence	-0.15528	########
N17-N19	2	1915	0.02443	-0.0883	0.2134	impact	-0.24358	########
N17-N19	2	1925	0.02443	-0.0883	0.2134	incidence	0.02032	########
N17-N19	2	1925	0.02443	-0.0883	0.2134	impact	-0.06798	########
N17-N19	2	1935	0.02443	-0.0883	0.2134	incidence	0.22676	########
N17-N19	2	1935	0.02443	-0.0883	0.2134	impact	0.13846	########
N17-N19	2	1945	0.02443	-0.0883	0.2134	incidence	0.084195	########
N17-N19	2	1945	0.02443	-0.0883	0.2134	impact	-0.004105	########
N17-N19	2	1955	0.02443	-0.0883	0.2134	incidence	0.057155	0.1361
N17-N19	2	1955	0.02443	-0.0883	0.2134	impact	-0.031145	########
N17-N19	3	1915	0.02593	0.231	0.1435	incidence	-0.69375	########
N17-N19	3	1915	0.02593	0.231	0.1435	impact	-0.46275	########
N17-N19	3	1925	0.02593	0.231	0.1435	incidence	-0.13442	########

For peer review only

N17-N19	3	1925	0.02593	0.231	0.1435	impact	0.09658	########
N17-N19	3	1935	0.02593	0.231	0.1435	incidence	0.20841	########
N17-N19	3	1935	0.02593	0.231	0.1435	impact	0.43941	########
N17-N19	3	1945	0.02593	0.231	0.1435	incidence	0.080852	########
N17-N19	3	1945	0.02593	0.231	0.1435	impact	0.311852	########
N17-N19	3	1955	0.02593	0.231	0.1435	incidence	0.242648	0.1328
N17-N19	3	1955	0.02593	0.231	0.1435	impact	0.473648	########
N17-N19	4	1915	0.02765	0.00575	0.064	incidence	-0.2722	########
N17-N19	4	1915	0.02765	0.00575	0.064	impact	-0.26645	########
N17-N19	4	1925	0.02765	0.00575	0.064	incidence	-0.05292	########
N17-N19	4	1925	0.02765	0.00575	0.064	impact	-0.04717	########
N17-N19	4	1935	0.02765	0.00575	0.064	incidence	0.16418	########
N17-N19	4	1935	0.02765	0.00575	0.064	impact	0.16993	########
N17-N19	4	1945	0.02765	0.00575	0.064	incidence	0.104484	########
N17-N19	4	1945	0.02765	0.00575	0.064	impact	0.110234	########
N17-N19	4	1955	0.02765	0.00575	0.064	incidence	0.073166	0.123
N17-N19	4	1955	0.02765	0.00575	0.064	impact	0.078916	########
N17-N19	5	1915	0.02914	0.534	0.2105	incidence	-0.33817	########
N17-N19	5	1915	0.02914	0.534	0.2105	impact	0.19583	########
N17-N19	5	1925	0.02914	0.534	0.2105	incidence	-0.14442	########
N17-N19	5	1925	0.02914	0.534	0.2105	impact	0.38958	########
N17-N19	5	1935	0.02914	0.534	0.2105	incidence	0.22923	########
N17-N19	5	1935	0.02914	0.534	0.2105	impact	0.76323	########
N17-N19	5	1945	0.02914	0.534	0.2105	incidence	0.137246	########
N17-N19	5	1945	0.02914	0.534	0.2105	impact	0.671246	########
N17-N19	5	1955	0.02914	0.534	0.2105	incidence	0.121924	0.1133
N17-N19	5	1955	0.02914	0.534	0.2105	impact	0.655924	########
N17-N19	6	1915	0.03191	0.176	0.1943	incidence	-0.20389	########
N17-N19	6	1915	0.03191	0.176	0.1943	impact	-0.02789	########
N17-N19	6	1925	0.03191	0.176	0.1943	incidence	-0.08484	########
N17-N19	6	1925	0.03191	0.176	0.1943	impact	0.09116	########
N17-N19	6	1935	0.03191	0.176	0.1943	incidence	0.13669	########
N17-N19	6	1935	0.03191	0.176	0.1943	impact	0.31269	########
N17-N19	6	1945	0.03191	0.176	0.1943	incidence	0.083557	########
N17-N19	6	1945	0.03191	0.176	0.1943	impact	0.259557	########
N17-N19	6	1955	0.03191	0.176	0.1943	incidence	0.097993	0.1067
N17-N19	6	1955	0.03191	0.176	0.1943	impact	0.273993	########
N17-N19	7	1915	0.03594	0.196	0.1471	incidence	-0.31519	########
N17-N19	7	1915	0.03594	0.196	0.1471	impact	-0.11919	########
N17-N19	7	1925	0.03594	0.196	0.1471	incidence	-0.0358	########
N17-N19	7	1925	0.03594	0.196	0.1471	impact	0.1602	########
N17-N19	7	1935	0.03594	0.196	0.1471	incidence	0.02749	########
N17-N19	7	1935	0.03594	0.196	0.1471	impact	0.22349	########
N17-N19	7	1945	0.03594	0.196	0.1471	incidence	0.126935	########
N17-N19	7	1945	0.03594	0.196	0.1471	impact	0.322935	########
N17-N19	7	1955	0.03594	0.196	0.1471	incidence	0.096205	0.1036
N17-N19	7	1955	0.03594	0.196	0.1471	impact	0.292205	########
N17-N19	8	1915	0.03673	0.114	0.1066	incidence	-0.32584	########
N17-N19	8	1915	0.03673	0.114	0.1066	impact	-0.21184	########
N17-N19	8	1925	0.03673	0.114	0.1066	incidence	-0.09319	########
N17-N19	8	1925	0.03673	0.114	0.1066	impact	0.02081	########
N17-N19	8	1935	0.03673	0.114	0.1066	incidence	0.06106	########
N17-N19	8	1935	0.03673	0.114	0.1066	impact	0.17506	########
N17-N19	8	1945	0.03673	0.114	0.1066	incidence	0.015446	########
N17-N19	8	1945	0.03673	0.114	0.1066	impact	0.129446	########
N17-N19	8	1955	0.03673	0.114	0.1066	incidence	0.097994	0.0973

For peer review only

N17-N19	8	1955	0.03673	0.114	0.1066	impact	0.211994	########
N17-N19	9	1915	0.03657	0.428	0.1679	incidence	-0.43597	########
N17-N19	9	1915	0.03657	0.428	0.1679	impact	-0.00797	########
N17-N19	9	1925	0.03657	0.428	0.1679	incidence	-0.03788	########
N17-N19	9	1925	0.03657	0.428	0.1679	impact	0.39012	########
N17-N19	9	1935	0.03657	0.428	0.1679	incidence	0.02042	########
N17-N19	9	1935	0.03657	0.428	0.1679	impact	0.44842	########
N17-N19	9	1945	0.03657	0.428	0.1679	incidence	0.080302	########
N17-N19	9	1945	0.03657	0.428	0.1679	impact	0.508302	########
N17-N19	9	1955	0.03657	0.428	0.1679	incidence	0.166938	0.0916
N17-N19	9	1955	0.03657	0.428	0.1679	impact	0.594938	########
N17-N19	10	1915	0.03761	0.151	0.1776	incidence	-0.32981	########
N17-N19	10	1915	0.03761	0.151	0.1776	impact	-0.17881	########
N17-N19	10	1925	0.03761	0.151	0.1776	incidence	-0.25185	########
N17-N19	10	1925	0.03761	0.151	0.1776	impact	-0.10085	########
N17-N19	10	1935	0.03761	0.151	0.1776	incidence	0.04824	########
N17-N19	10	1935	0.03761	0.151	0.1776	impact	0.19924	########
N17-N19	10	1945	0.03761	0.151	0.1776	incidence	0.067258	########
N17-N19	10	1945	0.03761	0.151	0.1776	impact	0.218258	########
N17-N19	10	1955	0.03761	0.151	0.1776	incidence	0.100902	0.0885
N17-N19	10	1955	0.03761	0.151	0.1776	impact	0.251902	########
I80-I89	1	1915	0.03016	0.415	0.2227	incidence	0.22136	########
I80-I89	1	1915	0.03016	0.415	0.2227	impact	0.63636	########
I80-I89	1	1925	0.03016	0.415	0.2227	incidence	0.27978	########
I80-I89	1	1925	0.03016	0.415	0.2227	impact	0.69478	########
I80-I89	1	1935	0.03016	0.415	0.2227	incidence	0.14092	########
I80-I89	1	1935	0.03016	0.415	0.2227	impact	0.55592	########
I80-I89	1	1945	0.03016	0.415	0.2227	incidence	0.269865	########
I80-I89	1	1945	0.03016	0.415	0.2227	impact	0.684865	########
I80-I89	1	1955	0.03016	0.415	0.2227	incidence	0.262125	0.0439
I80-I89	1	1955	0.03016	0.415	0.2227	impact	0.677125	########
I80-I89	2	1915	0.02989	0.0507	0.2263	incidence	-0.02032	########
I80-I89	2	1915	0.02989	0.0507	0.2263	impact	0.03038	########
I80-I89	2	1925	0.02989	0.0507	0.2263	incidence	-0.0056	########
I80-I89	2	1925	0.02989	0.0507	0.2263	impact	0.0451	########
I80-I89	2	1935	0.02989	0.0507	0.2263	incidence	0.12943	########
I80-I89	2	1935	0.02989	0.0507	0.2263	impact	0.18013	########
I80-I89	2	1945	0.02989	0.0507	0.2263	incidence	0.267793	########
I80-I89	2	1945	0.02989	0.0507	0.2263	impact	0.318493	########
I80-I89	2	1955	0.02989	0.0507	0.2263	incidence	0.270367	0.0402
I80-I89	2	1955	0.02989	0.0507	0.2263	impact	0.321067	########
I80-I89	3	1915	0.02537	0.101	0.1917	incidence	0.30356	########
I80-I89	3	1915	0.02537	0.101	0.1917	impact	0.40456	########
I80-I89	3	1925	0.02537	0.101	0.1917	incidence	0.22408	########
I80-I89	3	1925	0.02537	0.101	0.1917	impact	0.32508	########
I80-I89	3	1935	0.02537	0.101	0.1917	incidence	0.20194	########
I80-I89	3	1935	0.02537	0.101	0.1917	impact	0.30294	########
I80-I89	3	1945	0.02537	0.101	0.1917	incidence	0.257849	########
I80-I89	3	1945	0.02537	0.101	0.1917	impact	0.358849	########
I80-I89	3	1955	0.02537	0.101	0.1917	incidence	0.267661	0.0416
I80-I89	3	1955	0.02537	0.101	0.1917	impact	0.368661	########
I80-I89	4	1915	0.02707	0.617	0.3081	incidence	0.4988	########
I80-I89	4	1915	0.02707	0.617	0.3081	impact	1.1158	########
I80-I89	4	1925	0.02707	0.617	0.3081	incidence	0.31534	########
I80-I89	4	1925	0.02707	0.617	0.3081	impact	0.93234	########
I80-I89	4	1935	0.02707	0.617	0.3081	incidence	0.2198	########

For peer review only

I80-I89	4	1935	0.02707	0.617	0.3081	impact	0.8368	########
I80-I89	4	1945	0.02707	0.617	0.3081	incidence	0.245986	########
I80-I89	4	1945	0.02707	0.617	0.3081	impact	0.862986	########
I80-I89	4	1955	0.02707	0.617	0.3081	incidence	0.272134	0.0424
I80-I89	4	1955	0.02707	0.617	0.3081	impact	0.889134	########
I80-I89	5	1915	0.02685	0.213	0.1902	incidence	0.28958	########
I80-I89	5	1915	0.02685	0.213	0.1902	impact	0.50258	########
I80-I89	5	1925	0.02685	0.213	0.1902	incidence	0.25119	########
I80-I89	5	1925	0.02685	0.213	0.1902	impact	0.46419	########
I80-I89	5	1935	0.02685	0.213	0.1902	incidence	0.18566	########
I80-I89	5	1935	0.02685	0.213	0.1902	impact	0.39866	########
I80-I89	5	1945	0.02685	0.213	0.1902	incidence	0.270045	########
I80-I89	5	1945	0.02685	0.213	0.1902	impact	0.483045	########
I80-I89	5	1955	0.02685	0.213	0.1902	incidence	0.270275	0.0409
I80-I89	5	1955	0.02685	0.213	0.1902	impact	0.483275	########
I80-I89	6	1915	0.0274	0.0794	0.1808	incidence	0.23578	########
I80-I89	6	1915	0.0274	0.0794	0.1808	impact	0.31518	########
I80-I89	6	1925	0.0274	0.0794	0.1808	incidence	0.11726	########
I80-I89	6	1925	0.0274	0.0794	0.1808	impact	0.19666	########
I80-I89	6	1935	0.0274	0.0794	0.1808	incidence	0.3108	########
I80-I89	6	1935	0.0274	0.0794	0.1808	impact	0.3902	########
I80-I89	6	1945	0.0274	0.0794	0.1808	incidence	0.369068	########
I80-I89	6	1945	0.0274	0.0794	0.1808	impact	0.448468	########
I80-I89	6	1955	0.0274	0.0794	0.1808	incidence	0.170332	0.0395
I80-I89	6	1955	0.0274	0.0794	0.1808	impact	0.249732	########
I80-I89	7	1915	0.02688	0.489	0.1499	incidence	0.24008	########
I80-I89	7	1915	0.02688	0.489	0.1499	impact	0.72908	########
I80-I89	7	1925	0.02688	0.489	0.1499	incidence	0.17455	########
I80-I89	7	1925	0.02688	0.489	0.1499	impact	0.66355	########
I80-I89	7	1935	0.02688	0.489	0.1499	incidence	0.18275	########
I80-I89	7	1935	0.02688	0.489	0.1499	impact	0.67175	########
I80-I89	7	1945	0.02688	0.489	0.1499	incidence	0.289567	########
I80-I89	7	1945	0.02688	0.489	0.1499	impact	0.778567	########
I80-I89	7	1955	0.02688	0.489	0.1499	incidence	0.267583	0.0401
I80-I89	7	1955	0.02688	0.489	0.1499	impact	0.756583	########
I80-I89	8	1915	0.02879	0.284	0.1301	incidence	0.39248	########
I80-I89	8	1915	0.02879	0.284	0.1301	impact	0.67648	########
I80-I89	8	1925	0.02879	0.284	0.1301	incidence	0.21198	########
I80-I89	8	1925	0.02879	0.284	0.1301	impact	0.49598	########
I80-I89	8	1935	0.02879	0.284	0.1301	incidence	0.23331	########
I80-I89	8	1935	0.02879	0.284	0.1301	impact	0.51731	########
I80-I89	8	1945	0.02879	0.284	0.1301	incidence	0.3376	########
I80-I89	8	1945	0.02879	0.284	0.1301	impact	0.6216	########
I80-I89	8	1955	0.02879	0.284	0.1301	incidence	0.24189	0.039
I80-I89	8	1955	0.02879	0.284	0.1301	impact	0.52589	########
I80-I89	9	1915	0.02884	0.057	0.1173	incidence	0.19349	########
I80-I89	9	1915	0.02884	0.057	0.1173	impact	0.25049	########
I80-I89	9	1925	0.02884	0.057	0.1173	incidence	0.10582	########
I80-I89	9	1925	0.02884	0.057	0.1173	impact	0.16282	########
I80-I89	9	1935	0.02884	0.057	0.1173	incidence	0.1327	########
I80-I89	9	1935	0.02884	0.057	0.1173	impact	0.1897	########
I80-I89	9	1945	0.02884	0.057	0.1173	incidence	0.220998	########
I80-I89	9	1945	0.02884	0.057	0.1173	impact	0.277998	########
I80-I89	9	1955	0.02884	0.057	0.1173	incidence	0.240402	0.0387
I80-I89	9	1955	0.02884	0.057	0.1173	impact	0.297402	########
I80-I89	10	1915	0.0327	0.138	0.1618	incidence	0.05683	########

For peer review only

I80-I89	10	1915	0.0327	0.138	0.1618	impact	0.19483	########
I80-I89	10	1925	0.0327	0.138	0.1618	incidence	0.04219	########
I80-I89	10	1925	0.0327	0.138	0.1618	impact	0.18019	########
I80-I89	10	1935	0.0327	0.138	0.1618	incidence	0.1034	########
I80-I89	10	1935	0.0327	0.138	0.1618	impact	0.2414	########
I80-I89	10	1945	0.0327	0.138	0.1618	incidence	0.158194	########
I80-I89	10	1945	0.0327	0.138	0.1618	impact	0.296194	########
I80-I89	10	1955	0.0327	0.138	0.1618	incidence	0.229036	0.0376
I80-I89	10	1955	0.0327	0.138	0.1618	impact	0.367036	########
K90-K93	1	1915	0.01809	-0.348	0.1264	incidence	0.0095	########
K90-K93	1	1915	0.01809	-0.348	0.1264	impact	-0.3385	########
K90-K93	1	1925	0.01809	-0.348	0.1264	incidence	0.1975	########
K90-K93	1	1925	0.01809	-0.348	0.1264	impact	-0.1505	########
K90-K93	1	1935	0.01809	-0.348	0.1264	incidence	-0.03937	########
K90-K93	1	1935	0.01809	-0.348	0.1264	impact	-0.38737	########
K90-K93	1	1945	0.01809	-0.348	0.1264	incidence	-0.103799	########
K90-K93	1	1945	0.01809	-0.348	0.1264	impact	-0.451799	########
K90-K93	1	1955	0.01809	-0.348	0.1264	incidence	-0.253941	0.0875
K90-K93	1	1955	0.01809	-0.348	0.1264	impact	-0.601941	########
K90-K93	2	1915	0.01987	-0.0753	0.2466	incidence	0.03124	########
K90-K93	2	1915	0.01987	-0.0753	0.2466	impact	-0.04406	########
K90-K93	2	1925	0.01987	-0.0753	0.2466	incidence	0.11048	########
K90-K93	2	1925	0.01987	-0.0753	0.2466	impact	0.03518	########
K90-K93	2	1935	0.01987	-0.0753	0.2466	incidence	0.05088	########
K90-K93	2	1935	0.01987	-0.0753	0.2466	impact	-0.02442	########
K90-K93	2	1945	0.01987	-0.0753	0.2466	incidence	-0.084923	########
K90-K93	2	1945	0.01987	-0.0753	0.2466	impact	-0.160223	########
K90-K93	2	1955	0.01987	-0.0753	0.2466	incidence	-0.158377	0.0802
K90-K93	2	1955	0.01987	-0.0753	0.2466	impact	-0.233677	########
K90-K93	3	1915	0.01998	-0.164	0.2035	incidence	-0.00093	########
K90-K93	3	1915	0.01998	-0.164	0.2035	impact	-0.16493	########
K90-K93	3	1925	0.01998	-0.164	0.2035	incidence	0.18896	########
K90-K93	3	1925	0.01998	-0.164	0.2035	impact	0.02496	########
K90-K93	3	1935	0.01998	-0.164	0.2035	incidence	0.04501	########
K90-K93	3	1935	0.01998	-0.164	0.2035	impact	-0.11899	########
K90-K93	3	1945	0.01998	-0.164	0.2035	incidence	0.032663	########
K90-K93	3	1945	0.01998	-0.164	0.2035	impact	-0.131337	########
K90-K93	3	1955	0.01998	-0.164	0.2035	incidence	-0.167653	0.0799
K90-K93	3	1955	0.01998	-0.164	0.2035	impact	-0.331653	########
K90-K93	4	1915	0.02234	0.0764	0.1642	incidence	0.35104	########
K90-K93	4	1915	0.02234	0.0764	0.1642	impact	0.42744	########
K90-K93	4	1925	0.02234	0.0764	0.1642	incidence	0.17381	########
K90-K93	4	1925	0.02234	0.0764	0.1642	impact	0.25021	########
K90-K93	4	1935	0.02234	0.0764	0.1642	incidence	0.05481	########
K90-K93	4	1935	0.02234	0.0764	0.1642	impact	0.13121	########
K90-K93	4	1945	0.02234	0.0764	0.1642	incidence	0.066199	########
K90-K93	4	1945	0.02234	0.0764	0.1642	impact	0.142599	########
K90-K93	4	1955	0.02234	0.0764	0.1642	incidence	-0.091469	0.0766
K90-K93	4	1955	0.02234	0.0764	0.1642	impact	-0.015069	########
K90-K93	5	1915	0.02331	0.0467	0.1623	incidence	0.12508	########
K90-K93	5	1915	0.02331	0.0467	0.1623	impact	0.17178	########
K90-K93	5	1925	0.02331	0.0467	0.1623	incidence	0.18062	########
K90-K93	5	1925	0.02331	0.0467	0.1623	impact	0.22732	########
K90-K93	5	1935	0.02331	0.0467	0.1623	incidence	0.08632	########
K90-K93	5	1935	0.02331	0.0467	0.1623	impact	0.13302	########
K90-K93	5	1945	0.02331	0.0467	0.1623	incidence	0.106037	########

For peer review only

K90-K93	5	1945	0.02331	0.0467	0.1623	impact	0.152737	########
K90-K93	5	1955	0.02331	0.0467	0.1623	incidence	-0.051087	0.075
K90-K93	5	1955	0.02331	0.0467	0.1623	impact	-0.004387	########
K90-K93	6	1915	0.02622	0.253	0.1297	incidence	-0.02606	########
K90-K93	6	1915	0.02622	0.253	0.1297	impact	0.22694	########
K90-K93	6	1925	0.02622	0.253	0.1297	incidence	0.05888	########
K90-K93	6	1925	0.02622	0.253	0.1297	impact	0.31188	########
K90-K93	6	1935	0.02622	0.253	0.1297	incidence	0.02345	########
K90-K93	6	1935	0.02622	0.253	0.1297	impact	0.27645	########
K90-K93	6	1945	0.02622	0.253	0.1297	incidence	-0.023928	########
K90-K93	6	1945	0.02622	0.253	0.1297	impact	0.229072	########
K90-K93	6	1955	0.02622	0.253	0.1297	incidence	-0.159752	0.0689
K90-K93	6	1955	0.02622	0.253	0.1297	impact	0.093248	########
K90-K93	7	1915	0.02917	0.16	0.0941	incidence	0.06862	########
K90-K93	7	1915	0.02917	0.16	0.0941	impact	0.22862	########
K90-K93	7	1925	0.02917	0.16	0.0941	incidence	0.14625	########
K90-K93	7	1925	0.02917	0.16	0.0941	impact	0.30625	########
K90-K93	7	1935	0.02917	0.16	0.0941	incidence	0.031	########
K90-K93	7	1935	0.02917	0.16	0.0941	impact	0.191	########
K90-K93	7	1945	0.02917	0.16	0.0941	incidence	-0.128034	########
K90-K93	7	1945	0.02917	0.16	0.0941	impact	0.031966	########
K90-K93	7	1955	0.02917	0.16	0.0941	incidence	-0.080326	0.0646
K90-K93	7	1955	0.02917	0.16	0.0941	impact	0.079674	########
K90-K93	8	1915	0.02958	0.212	0.1361	incidence	0.15799	########
K90-K93	8	1915	0.02958	0.212	0.1361	impact	0.36999	########
K90-K93	8	1925	0.02958	0.212	0.1361	incidence	0.16115	########
K90-K93	8	1925	0.02958	0.212	0.1361	impact	0.37315	########
K90-K93	8	1935	0.02958	0.212	0.1361	incidence	-0.06221	########
K90-K93	8	1935	0.02958	0.212	0.1361	impact	0.14979	########
K90-K93	8	1945	0.02958	0.212	0.1361	incidence	0.020329	########
K90-K93	8	1945	0.02958	0.212	0.1361	impact	0.232329	########
K90-K93	8	1955	0.02958	0.212	0.1361	incidence	-0.017259	0.0621
K90-K93	8	1955	0.02958	0.212	0.1361	impact	0.194741	########
K90-K93	9	1915	0.03216	0.26	0.1528	incidence	0.22643	########
K90-K93	9	1915	0.03216	0.26	0.1528	impact	0.48643	########
K90-K93	9	1925	0.03216	0.26	0.1528	incidence	0.1359	########
K90-K93	9	1925	0.03216	0.26	0.1528	impact	0.3959	########
K90-K93	9	1935	0.03216	0.26	0.1528	incidence	0.04865	########
K90-K93	9	1935	0.03216	0.26	0.1528	impact	0.30865	########
K90-K93	9	1945	0.03216	0.26	0.1528	incidence	-0.088222	########
K90-K93	9	1945	0.03216	0.26	0.1528	impact	0.171778	########
K90-K93	9	1955	0.03216	0.26	0.1528	incidence	-0.085138	0.0562
K90-K93	9	1955	0.03216	0.26	0.1528	impact	0.174862	########
K90-K93	10	1915	0.03873	0.631	0.1607	incidence	-0.22614	########
K90-K93	10	1915	0.03873	0.631	0.1607	impact	0.40486	########
K90-K93	10	1925	0.03873	0.631	0.1607	incidence	-0.06523	########
K90-K93	10	1925	0.03873	0.631	0.1607	impact	0.56577	########
K90-K93	10	1935	0.03873	0.631	0.1607	incidence	-0.02173	########
K90-K93	10	1935	0.03873	0.631	0.1607	impact	0.60927	########
K90-K93	10	1945	0.03873	0.631	0.1607	incidence	-0.018717	########
K90-K93	10	1945	0.03873	0.631	0.1607	impact	0.612283	########
K90-K93	10	1955	0.03873	0.631	0.1607	incidence	-0.054593	0.0491
K90-K93	10	1955	0.03873	0.631	0.1607	impact	0.576407	########
I70-I79	1	1915	0.01949	0.426	0.2025	incidence	0.29208	########
I70-I79	1	1915	0.01949	0.426	0.2025	impact	0.71808	########
I70-I79	1	1925	0.01949	0.426	0.2025	incidence	0.36252	########

For peer review only

I70-I79	1	1925	0.01949	0.426	0.2025	impact	0.78852	########
I70-I79	1	1935	0.01949	0.426	0.2025	incidence	0.44519	########
I70-I79	1	1935	0.01949	0.426	0.2025	impact	0.87119	########
I70-I79	1	1945	0.01949	0.426	0.2025	incidence	0.376434	########
I70-I79	1	1945	0.01949	0.426	0.2025	impact	0.802434	########
I70-I79	1	1955	0.01949	0.426	0.2025	incidence	0.788776	0.117
I70-I79	1	1955	0.01949	0.426	0.2025	impact	1.214776	########
I70-I79	2	1915	0.01936	-0.11	0.124	incidence	-0.06331	########
I70-I79	2	1915	0.01936	-0.11	0.124	impact	-0.17331	########
I70-I79	2	1925	0.01936	-0.11	0.124	incidence	0.49402	########
I70-I79	2	1925	0.01936	-0.11	0.124	impact	0.38402	########
I70-I79	2	1935	0.01936	-0.11	0.124	incidence	0.47266	########
I70-I79	2	1935	0.01936	-0.11	0.124	impact	0.36266	########
I70-I79	2	1945	0.01936	-0.11	0.124	incidence	0.525666	########
I70-I79	2	1945	0.01936	-0.11	0.124	impact	0.415666	########
I70-I79	2	1955	0.01936	-0.11	0.124	incidence	0.557344	0.1122
I70-I79	2	1955	0.01936	-0.11	0.124	impact	0.447344	########
I70-I79	3	1915	0.02112	0.088	0.1238	incidence	0.22488	########
I70-I79	3	1915	0.02112	0.088	0.1238	impact	0.31288	########
I70-I79	3	1925	0.02112	0.088	0.1238	incidence	0.43142	########
I70-I79	3	1925	0.02112	0.088	0.1238	impact	0.51942	########
I70-I79	3	1935	0.02112	0.088	0.1238	incidence	0.53508	########
I70-I79	3	1935	0.02112	0.088	0.1238	impact	0.62308	########
I70-I79	3	1945	0.02112	0.088	0.1238	incidence	0.394118	########
I70-I79	3	1945	0.02112	0.088	0.1238	impact	0.482118	########
I70-I79	3	1955	0.02112	0.088	0.1238	incidence	0.614432	0.1059
I70-I79	3	1955	0.02112	0.088	0.1238	impact	0.702432	########
I70-I79	4	1915	0.02318	0.257	0.1528	incidence	0.10749	########
I70-I79	4	1915	0.02318	0.257	0.1528	impact	0.36449	########
I70-I79	4	1925	0.02318	0.257	0.1528	incidence	0.34887	########
I70-I79	4	1925	0.02318	0.257	0.1528	impact	0.60587	########
I70-I79	4	1935	0.02318	0.257	0.1528	incidence	0.55614	########
I70-I79	4	1935	0.02318	0.257	0.1528	impact	0.81314	########
I70-I79	4	1945	0.02318	0.257	0.1528	incidence	0.4481	########
I70-I79	4	1945	0.02318	0.257	0.1528	impact	0.7051	########
I70-I79	4	1955	0.02318	0.257	0.1528	incidence	0.44037	0.0988
I70-I79	4	1955	0.02318	0.257	0.1528	impact	0.69737	########
I70-I79	5	1915	0.02498	0.261	0.1831	incidence	0.31652	########
I70-I79	5	1915	0.02498	0.261	0.1831	impact	0.57752	########
I70-I79	5	1925	0.02498	0.261	0.1831	incidence	0.45785	########
I70-I79	5	1925	0.02498	0.261	0.1831	impact	0.71885	########
I70-I79	5	1935	0.02498	0.261	0.1831	incidence	0.52672	########
I70-I79	5	1935	0.02498	0.261	0.1831	impact	0.78772	########
I70-I79	5	1945	0.02498	0.261	0.1831	incidence	0.330566	########
I70-I79	5	1945	0.02498	0.261	0.1831	impact	0.591566	########
I70-I79	5	1955	0.02498	0.261	0.1831	incidence	0.418824	0.0931
I70-I79	5	1955	0.02498	0.261	0.1831	impact	0.679824	########
I70-I79	6	1915	0.02721	0.0406	0.1895	incidence	0.02548	########
I70-I79	6	1915	0.02721	0.0406	0.1895	impact	0.06608	########
I70-I79	6	1925	0.02721	0.0406	0.1895	incidence	0.44258	########
I70-I79	6	1925	0.02721	0.0406	0.1895	impact	0.48318	########
I70-I79	6	1935	0.02721	0.0406	0.1895	incidence	0.45558	########
I70-I79	6	1935	0.02721	0.0406	0.1895	impact	0.49618	########
I70-I79	6	1945	0.02721	0.0406	0.1895	incidence	0.521651	########
I70-I79	6	1945	0.02721	0.0406	0.1895	impact	0.562251	########
I70-I79	6	1955	0.02721	0.0406	0.1895	incidence	0.564239	0.0905

For peer review only

I70-I79	6	1955	0.02721	0.0406	0.1895	impact	0.604839	########
I70-I79	7	1915	0.02886	-0.00355	0.135	incidence	0.26157	########
I70-I79	7	1915	0.02886	-0.00355	0.135	impact	0.25802	########
I70-I79	7	1925	0.02886	-0.00355	0.135	incidence	0.45291	########
I70-I79	7	1925	0.02886	-0.00355	0.135	impact	0.44936	########
I70-I79	7	1935	0.02886	-0.00355	0.135	incidence	0.43699	########
I70-I79	7	1935	0.02886	-0.00355	0.135	impact	0.43344	########
I70-I79	7	1945	0.02886	-0.00355	0.135	incidence	0.545891	########
I70-I79	7	1945	0.02886	-0.00355	0.135	impact	0.542341	########
I70-I79	7	1955	0.02886	-0.00355	0.135	incidence	0.475479	0.0837
I70-I79	7	1955	0.02886	-0.00355	0.135	impact	0.471929	########
I70-I79	8	1915	0.03078	-0.048	0.1019	incidence	0.10717	########
I70-I79	8	1915	0.03078	-0.048	0.1019	impact	0.05917	########
I70-I79	8	1925	0.03078	-0.048	0.1019	incidence	0.3756	########
I70-I79	8	1925	0.03078	-0.048	0.1019	impact	0.3276	########
I70-I79	8	1935	0.03078	-0.048	0.1019	incidence	0.50837	########
I70-I79	8	1935	0.03078	-0.048	0.1019	impact	0.46037	########
I70-I79	8	1945	0.03078	-0.048	0.1019	incidence	0.509014	########
I70-I79	8	1945	0.03078	-0.048	0.1019	impact	0.461014	########
I70-I79	8	1955	0.03078	-0.048	0.1019	incidence	0.331806	0.0744
I70-I79	8	1955	0.03078	-0.048	0.1019	impact	0.283806	########
I70-I79	9	1915	0.03059	0.0584	0.0619	incidence	0.15103	########
I70-I79	9	1915	0.03059	0.0584	0.0619	impact	0.20943	########
I70-I79	9	1925	0.03059	0.0584	0.0619	incidence	0.32817	########
I70-I79	9	1925	0.03059	0.0584	0.0619	impact	0.38657	########
I70-I79	9	1935	0.03059	0.0584	0.0619	incidence	0.57528	########
I70-I79	9	1935	0.03059	0.0584	0.0619	impact	0.63368	########
I70-I79	9	1945	0.03059	0.0584	0.0619	incidence	0.509837	########
I70-I79	9	1945	0.03059	0.0584	0.0619	impact	0.568237	########
I70-I79	9	1955	0.03059	0.0584	0.0619	incidence	0.285393	0.0716
I70-I79	9	1955	0.03059	0.0584	0.0619	impact	0.343793	########
I70-I79	10	1915	0.03226	0.0655	0.163	incidence	0.09688	########
I70-I79	10	1915	0.03226	0.0655	0.163	impact	0.16238	########
I70-I79	10	1925	0.03226	0.0655	0.163	incidence	0.28767	########
I70-I79	10	1925	0.03226	0.0655	0.163	impact	0.35317	########
I70-I79	10	1935	0.03226	0.0655	0.163	incidence	0.50534	########
I70-I79	10	1935	0.03226	0.0655	0.163	impact	0.57084	########
I70-I79	10	1945	0.03226	0.0655	0.163	incidence	0.567386	########
I70-I79	10	1945	0.03226	0.0655	0.163	impact	0.632886	########
I70-I79	10	1955	0.03226	0.0655	0.163	incidence	0.484944	0.0676
I70-I79	10	1955	0.03226	0.0655	0.163	impact	0.550444	########
K50-K52	1	1915	0.01668	-0.17	0.164	incidence	0.04693	########
K50-K52	1	1915	0.01668	-0.17	0.164	impact	-0.12307	########
K50-K52	1	1925	0.01668	-0.17	0.164	incidence	0.36177	########
K50-K52	1	1925	0.01668	-0.17	0.164	impact	0.19177	########
K50-K52	1	1935	0.01668	-0.17	0.164	incidence	0.40669	########
K50-K52	1	1935	0.01668	-0.17	0.164	impact	0.23669	########
K50-K52	1	1945	0.01668	-0.17	0.164	incidence	0.382692	########
K50-K52	1	1945	0.01668	-0.17	0.164	impact	0.212692	########
K50-K52	1	1955	0.01668	-0.17	0.164	incidence	0.375278	0.0676
K50-K52	1	1955	0.01668	-0.17	0.164	impact	0.205278	########
K50-K52	2	1915	0.02159	0.288	0.1331	incidence	0.38164	########
K50-K52	2	1915	0.02159	0.288	0.1331	impact	0.66964	########
K50-K52	2	1925	0.02159	0.288	0.1331	incidence	0.49965	########
K50-K52	2	1925	0.02159	0.288	0.1331	impact	0.78765	########
K50-K52	2	1935	0.02159	0.288	0.1331	incidence	0.38646	0.13

For peer review only

K50-K52	2	1935	0.02159	0.288	0.1331	impact	0.67446	########
K50-K52	2	1945	0.02159	0.288	0.1331	incidence	0.413404	########
K50-K52	2	1945	0.02159	0.288	0.1331	impact	0.701404	########
K50-K52	2	1955	0.02159	0.288	0.1331	incidence	0.453756	0.0604
K50-K52	2	1955	0.02159	0.288	0.1331	impact	0.741756	########
K50-K52	3	1915	0.02204	-0.124	0.24	incidence	0.1886	########
K50-K52	3	1915	0.02204	-0.124	0.24	impact	0.0646	########
K50-K52	3	1925	0.02204	-0.124	0.24	incidence	0.32598	########
K50-K52	3	1925	0.02204	-0.124	0.24	impact	0.20198	########
K50-K52	3	1935	0.02204	-0.124	0.24	incidence	0.37006	########
K50-K52	3	1935	0.02204	-0.124	0.24	impact	0.24606	########
K50-K52	3	1945	0.02204	-0.124	0.24	incidence	0.39043	########
K50-K52	3	1945	0.02204	-0.124	0.24	impact	0.26643	########
K50-K52	3	1955	0.02204	-0.124	0.24	incidence	0.30606	0.0603
K50-K52	3	1955	0.02204	-0.124	0.24	impact	0.18206	########
K50-K52	4	1915	0.02233	-0.375	0.2026	incidence	0.06475	########
K50-K52	4	1915	0.02233	-0.375	0.2026	impact	-0.31025	0.285412
K50-K52	4	1925	0.02233	-0.375	0.2026	incidence	0.36375	########
K50-K52	4	1925	0.02233	-0.375	0.2026	impact	-0.01125	########
K50-K52	4	1935	0.02233	-0.375	0.2026	incidence	0.54114	########
K50-K52	4	1935	0.02233	-0.375	0.2026	impact	0.16614	########
K50-K52	4	1945	0.02233	-0.375	0.2026	incidence	0.333	########
K50-K52	4	1945	0.02233	-0.375	0.2026	impact	-0.042	########
K50-K52	4	1955	0.02233	-0.375	0.2026	incidence	0.43961	0.0608
K50-K52	4	1955	0.02233	-0.375	0.2026	impact	0.06461	########
K50-K52	5	1915	0.02267	0.353	0.1282	incidence	0.03916	########
K50-K52	5	1915	0.02267	0.353	0.1282	impact	0.39216	########
K50-K52	5	1925	0.02267	0.353	0.1282	incidence	0.35487	########
K50-K52	5	1925	0.02267	0.353	0.1282	impact	0.70787	########
K50-K52	5	1935	0.02267	0.353	0.1282	incidence	0.46689	########
K50-K52	5	1935	0.02267	0.353	0.1282	impact	0.81989	########
K50-K52	5	1945	0.02267	0.353	0.1282	incidence	0.41676	########
K50-K52	5	1945	0.02267	0.353	0.1282	impact	0.76976	########
K50-K52	5	1955	0.02267	0.353	0.1282	incidence	0.36396	0.0593
K50-K52	5	1955	0.02267	0.353	0.1282	impact	0.71696	########
K50-K52	6	1915	0.02537	0.149	0.1138	incidence	0.19252	########
K50-K52	6	1915	0.02537	0.149	0.1138	impact	0.34152	########
K50-K52	6	1925	0.02537	0.149	0.1138	incidence	0.45853	########
K50-K52	6	1925	0.02537	0.149	0.1138	impact	0.60753	########
K50-K52	6	1935	0.02537	0.149	0.1138	incidence	0.37158	########
K50-K52	6	1935	0.02537	0.149	0.1138	impact	0.52058	########
K50-K52	6	1945	0.02537	0.149	0.1138	incidence	0.377346	########
K50-K52	6	1945	0.02537	0.149	0.1138	impact	0.526346	########
K50-K52	6	1955	0.02537	0.149	0.1138	incidence	0.354884	0.0569
K50-K52	6	1955	0.02537	0.149	0.1138	impact	0.503884	########
K50-K52	7	1915	0.028	0.0934	0.082	incidence	0.11498	########
K50-K52	7	1915	0.028	0.0934	0.082	impact	0.20838	########
K50-K52	7	1925	0.028	0.0934	0.082	incidence	0.21385	########
K50-K52	7	1925	0.028	0.0934	0.082	impact	0.30725	########
K50-K52	7	1935	0.028	0.0934	0.082	incidence	0.34847	########
K50-K52	7	1935	0.028	0.0934	0.082	impact	0.44187	########
K50-K52	7	1945	0.028	0.0934	0.082	incidence	0.382362	########
K50-K52	7	1945	0.028	0.0934	0.082	impact	0.475762	########
K50-K52	7	1955	0.028	0.0934	0.082	incidence	0.432868	0.0558
K50-K52	7	1955	0.028	0.0934	0.082	impact	0.526268	########
K50-K52	8	1915	0.02751	0.0265	0.1333	incidence	0.27406	########

For peer review only

K50-K52	8	1915	0.02751	0.0265	0.1333	impact	0.30056	########
K50-K52	8	1925	0.02751	0.0265	0.1333	incidence	0.20486	########
K50-K52	8	1925	0.02751	0.0265	0.1333	impact	0.23136	########
K50-K52	8	1935	0.02751	0.0265	0.1333	incidence	0.31974	########
K50-K52	8	1935	0.02751	0.0265	0.1333	impact	0.34624	########
K50-K52	8	1945	0.02751	0.0265	0.1333	incidence	0.459544	########
K50-K52	8	1945	0.02751	0.0265	0.1333	impact	0.486044	########
K50-K52	8	1955	0.02751	0.0265	0.1333	incidence	0.267476	0.0532
K50-K52	8	1955	0.02751	0.0265	0.1333	impact	0.293976	########
K50-K52	9	1915	0.02794	0.245	0.1126	incidence	-0.06529	########
K50-K52	9	1915	0.02794	0.245	0.1126	impact	0.17971	########
K50-K52	9	1925	0.02794	0.245	0.1126	incidence	0.21158	########
K50-K52	9	1925	0.02794	0.245	0.1126	impact	0.45658	########
K50-K52	9	1935	0.02794	0.245	0.1126	incidence	0.31543	########
K50-K52	9	1935	0.02794	0.245	0.1126	impact	0.56043	########
K50-K52	9	1945	0.02794	0.245	0.1126	incidence	0.38381	########
K50-K52	9	1945	0.02794	0.245	0.1126	impact	0.62881	########
K50-K52	9	1955	0.02794	0.245	0.1126	incidence	0.31434	0.0505
K50-K52	9	1955	0.02794	0.245	0.1126	impact	0.55934	########
K50-K52	10	1915	0.03151	0.24	0.1034	incidence	0.2971	########
K50-K52	10	1915	0.03151	0.24	0.1034	impact	0.5371	########
K50-K52	10	1925	0.03151	0.24	0.1034	incidence	0.14486	########
K50-K52	10	1925	0.03151	0.24	0.1034	impact	0.38486	########
K50-K52	10	1935	0.03151	0.24	0.1034	incidence	0.27869	########
K50-K52	10	1935	0.03151	0.24	0.1034	impact	0.51869	########
K50-K52	10	1945	0.03151	0.24	0.1034	incidence	0.245172	########
K50-K52	10	1945	0.03151	0.24	0.1034	impact	0.485172	########
K50-K52	10	1955	0.03151	0.24	0.1034	incidence	0.234218	0.0479
K50-K52	10	1955	0.03151	0.24	0.1034	impact	0.474218	0.113956
S00-S09	1	1915	0.01798	-0.152	0.183	incidence	-0.36309	########
S00-S09	1	1915	0.01798	-0.152	0.183	impact	-0.51509	########
S00-S09	1	1925	0.01798	-0.152	0.183	incidence	-0.19284	########
S00-S09	1	1925	0.01798	-0.152	0.183	impact	-0.34484	########
S00-S09	1	1935	0.01798	-0.152	0.183	incidence	0.018	########
S00-S09	1	1935	0.01798	-0.152	0.183	impact	-0.134	########
S00-S09	1	1945	0.01798	-0.152	0.183	incidence	-0.056748	########
S00-S09	1	1945	0.01798	-0.152	0.183	impact	-0.208748	########
S00-S09	1	1955	0.01798	-0.152	0.183	incidence	0.181508	0.0856
S00-S09	1	1955	0.01798	-0.152	0.183	impact	0.029508	########
S00-S09	2	1915	0.01603	0.248	0.1632	incidence	-0.16553	########
S00-S09	2	1915	0.01603	0.248	0.1632	impact	0.08247	########
S00-S09	2	1925	0.01603	0.248	0.1632	incidence	-0.15019	########
S00-S09	2	1925	0.01603	0.248	0.1632	impact	0.09781	########
S00-S09	2	1935	0.01603	0.248	0.1632	incidence	-0.01759	########
S00-S09	2	1935	0.01603	0.248	0.1632	impact	0.23041	########
S00-S09	2	1945	0.01603	0.248	0.1632	incidence	0.113794	########
S00-S09	2	1945	0.01603	0.248	0.1632	impact	0.361794	########
S00-S09	2	1955	0.01603	0.248	0.1632	incidence	0.087186	0.0778
S00-S09	2	1955	0.01603	0.248	0.1632	impact	0.335186	########
S00-S09	3	1915	0.01769	-0.0459	0.1171	incidence	-0.1755	########
S00-S09	3	1915	0.01769	-0.0459	0.1171	impact	-0.2214	########
S00-S09	3	1925	0.01769	-0.0459	0.1171	incidence	-0.08027	########
S00-S09	3	1925	0.01769	-0.0459	0.1171	impact	-0.12617	########
S00-S09	3	1935	0.01769	-0.0459	0.1171	incidence	0.08953	########
S00-S09	3	1935	0.01769	-0.0459	0.1171	impact	0.04363	########
S00-S09	3	1945	0.01769	-0.0459	0.1171	incidence	0.238871	########

For peer review only

S00-S09	3	1945	0.01769	-0.0459	0.1171	impact	0.192971	########
S00-S09	3	1955	0.01769	-0.0459	0.1171	incidence	0.178589	0.0723
S00-S09	3	1955	0.01769	-0.0459	0.1171	impact	0.132689	########
S00-S09	4	1915	0.0202	-0.336	0.1536	incidence	-0.08736	########
S00-S09	4	1915	0.0202	-0.336	0.1536	impact	-0.42336	########
S00-S09	4	1925	0.0202	-0.336	0.1536	incidence	0.01151	########
S00-S09	4	1925	0.0202	-0.336	0.1536	impact	-0.32449	########
S00-S09	4	1935	0.0202	-0.336	0.1536	incidence	0.09397	########
S00-S09	4	1935	0.0202	-0.336	0.1536	impact	-0.24203	########
S00-S09	4	1945	0.0202	-0.336	0.1536	incidence	0.316901	########
S00-S09	4	1945	0.0202	-0.336	0.1536	impact	-0.019099	########
S00-S09	4	1955	0.0202	-0.336	0.1536	incidence	0.190619	0.0675
S00-S09	4	1955	0.0202	-0.336	0.1536	impact	-0.145381	########
S00-S09	5	1915	0.02099	-0.213	0.1882	incidence	-0.29433	########
S00-S09	5	1915	0.02099	-0.213	0.1882	impact	-0.50733	########
S00-S09	5	1925	0.02099	-0.213	0.1882	incidence	-0.12783	########
S00-S09	5	1925	0.02099	-0.213	0.1882	impact	-0.34083	########
S00-S09	5	1935	0.02099	-0.213	0.1882	incidence	-0.05251	########
S00-S09	5	1935	0.02099	-0.213	0.1882	impact	-0.26551	########
S00-S09	5	1945	0.02099	-0.213	0.1882	incidence	0.266171	########
S00-S09	5	1945	0.02099	-0.213	0.1882	impact	0.053171	########
S00-S09	5	1955	0.02099	-0.213	0.1882	incidence	0.169469	0.0635
S00-S09	5	1955	0.02099	-0.213	0.1882	impact	-0.043531	########
S00-S09	6	1915	0.0223	0.222	0.1938	incidence	-0.05954	########
S00-S09	6	1915	0.0223	0.222	0.1938	impact	0.16246	########
S00-S09	6	1925	0.0223	0.222	0.1938	incidence	-0.10736	########
S00-S09	6	1925	0.0223	0.222	0.1938	impact	0.11464	########
S00-S09	6	1935	0.0223	0.222	0.1938	incidence	0.06683	########
S00-S09	6	1935	0.0223	0.222	0.1938	impact	0.28883	########
S00-S09	6	1945	0.0223	0.222	0.1938	incidence	0.31758	########
S00-S09	6	1945	0.0223	0.222	0.1938	impact	0.53958	########
S00-S09	6	1955	0.0223	0.222	0.1938	incidence	0.26111	0.0602
S00-S09	6	1955	0.0223	0.222	0.1938	impact	0.48311	########
S00-S09	7	1915	0.0262	-0.00577	0.1761	incidence	-0.12361	########
S00-S09	7	1915	0.0262	-0.00577	0.1761	impact	-0.12938	########
S00-S09	7	1925	0.0262	-0.00577	0.1761	incidence	-0.10853	########
S00-S09	7	1925	0.0262	-0.00577	0.1761	impact	-0.1143	########
S00-S09	7	1935	0.0262	-0.00577	0.1761	incidence	0.00336	########
S00-S09	7	1935	0.0262	-0.00577	0.1761	impact	-0.00241	########
S00-S09	7	1945	0.0262	-0.00577	0.1761	incidence	0.333403	########
S00-S09	7	1945	0.0262	-0.00577	0.1761	impact	0.327633	########
S00-S09	7	1955	0.0262	-0.00577	0.1761	incidence	0.242587	0.0561
S00-S09	7	1955	0.0262	-0.00577	0.1761	impact	0.236817	########
S00-S09	8	1915	0.02803	0.0104	0.0798	incidence	-0.17078	########
S00-S09	8	1915	0.02803	0.0104	0.0798	impact	-0.16038	########
S00-S09	8	1925	0.02803	0.0104	0.0798	incidence	-0.14808	########
S00-S09	8	1925	0.02803	0.0104	0.0798	impact	-0.13768	########
S00-S09	8	1935	0.02803	0.0104	0.0798	incidence	-0.01129	########
S00-S09	8	1935	0.02803	0.0104	0.0798	impact	-0.00089	########
S00-S09	8	1945	0.02803	0.0104	0.0798	incidence	0.341535	########
S00-S09	8	1945	0.02803	0.0104	0.0798	impact	0.351935	########
S00-S09	8	1955	0.02803	0.0104	0.0798	incidence	0.301275	0.0511
S00-S09	8	1955	0.02803	0.0104	0.0798	impact	0.311675	########
S00-S09	9	1915	0.03226	-0.093	0.1162	incidence	-0.18885	########
S00-S09	9	1915	0.03226	-0.093	0.1162	impact	-0.28185	########
S00-S09	9	1925	0.03226	-0.093	0.1162	incidence	-0.13306	########

For peer review only

S00-S09	9	1925	0.03226	-0.093	0.1162	impact	-0.22606	########
S00-S09	9	1935	0.03226	-0.093	0.1162	incidence	0.048	########
S00-S09	9	1935	0.03226	-0.093	0.1162	impact	-0.045	########
S00-S09	9	1945	0.03226	-0.093	0.1162	incidence	0.272973	########
S00-S09	9	1945	0.03226	-0.093	0.1162	impact	0.179973	########
S00-S09	9	1955	0.03226	-0.093	0.1162	incidence	0.364287	0.0477
S00-S09	9	1955	0.03226	-0.093	0.1162	impact	0.271287	########
S00-S09	10	1915	0.039	0.181	0.143	incidence	-0.26592	########
S00-S09	10	1915	0.039	0.181	0.143	impact	-0.08492	########
S00-S09	10	1925	0.039	0.181	0.143	incidence	-0.14767	########
S00-S09	10	1925	0.039	0.181	0.143	impact	0.03333	########
S00-S09	10	1935	0.039	0.181	0.143	incidence	0.09772	########
S00-S09	10	1935	0.039	0.181	0.143	impact	0.27872	########
S00-S09	10	1945	0.039	0.181	0.143	incidence	0.286395	########
S00-S09	10	1945	0.039	0.181	0.143	impact	0.467395	########
S00-S09	10	1955	0.039	0.181	0.143	incidence	0.452375	0.0416
S00-S09	10	1955	0.039	0.181	0.143	impact	0.633375	########
C50-C50	1	1915	0.02952	0.565	0.2398	incidence	0.57093	########
C50-C50	1	1915	0.02952	0.565	0.2398	impact	1.13593	########
C50-C50	1	1925	0.02952	0.565	0.2398	incidence	0.14759	########
C50-C50	1	1925	0.02952	0.565	0.2398	impact	0.71259	########
C50-C50	1	1935	0.02952	0.565	0.2398	incidence	0.10716	########
C50-C50	1	1935	0.02952	0.565	0.2398	impact	0.67216	########
C50-C50	1	1945	0.02952	0.565	0.2398	incidence	0.200385	########
C50-C50	1	1945	0.02952	0.565	0.2398	impact	0.765385	########
C50-C50	1	1955	0.02952	0.565	0.2398	incidence	0.212105	0.0594
C50-C50	1	1955	0.02952	0.565	0.2398	impact	0.777105	########
C50-C50	2	1915	0.02828	1.74	0.2432	incidence	0.12662	########
C50-C50	2	1915	0.02828	1.74	0.2432	impact	1.86662	########
C50-C50	2	1925	0.02828	1.74	0.2432	incidence	0.24929	########
C50-C50	2	1925	0.02828	1.74	0.2432	impact	1.98929	########
C50-C50	2	1935	0.02828	1.74	0.2432	incidence	0.16444	########
C50-C50	2	1935	0.02828	1.74	0.2432	impact	1.90444	########
C50-C50	2	1945	0.02828	1.74	0.2432	incidence	0.190072	########
C50-C50	2	1945	0.02828	1.74	0.2432	impact	1.930072	########
C50-C50	2	1955	0.02828	1.74	0.2432	incidence	0.153448	0.0589
C50-C50	2	1955	0.02828	1.74	0.2432	impact	1.893448	########
C50-C50	3	1915	0.02502	1.7	0.2848	incidence	0.12831	########
C50-C50	3	1915	0.02502	1.7	0.2848	impact	1.82831	########
C50-C50	3	1925	0.02502	1.7	0.2848	incidence	0.28344	########
C50-C50	3	1925	0.02502	1.7	0.2848	impact	1.98344	########
C50-C50	3	1935	0.02502	1.7	0.2848	incidence	0.05314	########
C50-C50	3	1935	0.02502	1.7	0.2848	impact	1.75314	########
C50-C50	3	1945	0.02502	1.7	0.2848	incidence	0.259794	########
C50-C50	3	1945	0.02502	1.7	0.2848	impact	1.959794	########
C50-C50	3	1955	0.02502	1.7	0.2848	incidence	0.143936	0.0619
C50-C50	3	1955	0.02502	1.7	0.2848	impact	1.843936	########
C50-C50	4	1915	0.02499	1.45	0.1209	incidence	0.54233	########
C50-C50	4	1915	0.02499	1.45	0.1209	impact	1.99233	########
C50-C50	4	1925	0.02499	1.45	0.1209	incidence	0.13248	########
C50-C50	4	1925	0.02499	1.45	0.1209	impact	1.58248	########
C50-C50	4	1935	0.02499	1.45	0.1209	incidence	0.13397	########
C50-C50	4	1935	0.02499	1.45	0.1209	impact	1.58397	########
C50-C50	4	1945	0.02499	1.45	0.1209	incidence	0.222236	########
C50-C50	4	1945	0.02499	1.45	0.1209	impact	1.672236	########
C50-C50	4	1955	0.02499	1.45	0.1209	incidence	0.149594	0.0616

For peer review only

C50-C50	4	1955	0.02499	1.45	0.1209	impact	1.599594	########
C50-C50	5	1915	0.02305	1.17	0.1704	incidence	0.27318	########
C50-C50	5	1915	0.02305	1.17	0.1704	impact	1.44318	########
C50-C50	5	1925	0.02305	1.17	0.1704	incidence	0.14826	########
C50-C50	5	1925	0.02305	1.17	0.1704	impact	1.31826	########
C50-C50	5	1935	0.02305	1.17	0.1704	incidence	0.15257	########
C50-C50	5	1935	0.02305	1.17	0.1704	impact	1.32257	########
C50-C50	5	1945	0.02305	1.17	0.1704	incidence	0.164898	########
C50-C50	5	1945	0.02305	1.17	0.1704	impact	1.334898	########
C50-C50	5	1955	0.02305	1.17	0.1704	incidence	0.180402	0.0617
C50-C50	5	1955	0.02305	1.17	0.1704	impact	1.350402	########
C50-C50	6	1915	0.02338	1.59	0.3056	incidence	0.46622	########
C50-C50	6	1915	0.02338	1.59	0.3056	impact	2.05622	########
C50-C50	6	1925	0.02338	1.59	0.3056	incidence	0.20515	########
C50-C50	6	1925	0.02338	1.59	0.3056	impact	1.79515	########
C50-C50	6	1935	0.02338	1.59	0.3056	incidence	0.17093	########
C50-C50	6	1935	0.02338	1.59	0.3056	impact	1.76093	########
C50-C50	6	1945	0.02338	1.59	0.3056	incidence	0.096701	########
C50-C50	6	1945	0.02338	1.59	0.3056	impact	1.686701	########
C50-C50	6	1955	0.02338	1.59	0.3056	incidence	0.308739	0.0615
C50-C50	6	1955	0.02338	1.59	0.3056	impact	1.898739	########
C50-C50	7	1915	0.02101	0.955	0.2127	incidence	0.34472	########
C50-C50	7	1915	0.02101	0.955	0.2127	impact	1.29972	########
C50-C50	7	1925	0.02101	0.955	0.2127	incidence	0.33799	########
C50-C50	7	1925	0.02101	0.955	0.2127	impact	1.29299	########
C50-C50	7	1935	0.02101	0.955	0.2127	incidence	0.19384	########
C50-C50	7	1935	0.02101	0.955	0.2127	impact	1.14884	########
C50-C50	7	1945	0.02101	0.955	0.2127	incidence	0.128898	########
C50-C50	7	1945	0.02101	0.955	0.2127	impact	1.083898	########
C50-C50	7	1955	0.02101	0.955	0.2127	incidence	0.211702	0.0642
C50-C50	7	1955	0.02101	0.955	0.2127	impact	1.166702	########
C50-C50	8	1915	0.02088	0.876	0.1875	incidence	0.15661	########
C50-C50	8	1915	0.02088	0.876	0.1875	impact	1.03261	########
C50-C50	8	1925	0.02088	0.876	0.1875	incidence	0.15711	########
C50-C50	8	1925	0.02088	0.876	0.1875	impact	1.03311	########
C50-C50	8	1935	0.02088	0.876	0.1875	incidence	0.03363	########
C50-C50	8	1935	0.02088	0.876	0.1875	impact	0.90963	########
C50-C50	8	1945	0.02088	0.876	0.1875	incidence	0.221135	########
C50-C50	8	1945	0.02088	0.876	0.1875	impact	1.097135	########
C50-C50	8	1955	0.02088	0.876	0.1875	incidence	0.297235	0.0659
C50-C50	8	1955	0.02088	0.876	0.1875	impact	1.173235	########
C50-C50	9	1915	0.02093	1.03	0.2063	incidence	0.19309	########
C50-C50	9	1915	0.02093	1.03	0.2063	impact	1.22309	########
C50-C50	9	1925	0.02093	1.03	0.2063	incidence	0.38261	########
C50-C50	9	1925	0.02093	1.03	0.2063	impact	1.41261	########
C50-C50	9	1935	0.02093	1.03	0.2063	incidence	0.14365	########
C50-C50	9	1935	0.02093	1.03	0.2063	impact	1.17365	########
C50-C50	9	1945	0.02093	1.03	0.2063	incidence	0.201289	########
C50-C50	9	1945	0.02093	1.03	0.2063	impact	1.231289	########
C50-C50	9	1955	0.02093	1.03	0.2063	incidence	0.280731	0.0664
C50-C50	9	1955	0.02093	1.03	0.2063	impact	1.310731	########
C50-C50	10	1915	0.01881	0.806	0.1659	incidence	0.01846	########
C50-C50	10	1915	0.01881	0.806	0.1659	impact	0.82446	########
C50-C50	10	1925	0.01881	0.806	0.1659	incidence	0.31314	########
C50-C50	10	1925	0.01881	0.806	0.1659	impact	1.11914	########
C50-C50	10	1935	0.01881	0.806	0.1659	incidence	0.12398	########

For peer review only

C50-C50	10	1935	0.01881	0.806	0.1659	impact	0.92998	########
C50-C50	10	1945	0.01881	0.806	0.1659	incidence	0.159037	########
C50-C50	10	1945	0.01881	0.806	0.1659	impact	0.965037	########
C50-C50	10	1955	0.01881	0.806	0.1659	incidence	0.264673	0.0676
C50-C50	10	1955	0.01881	0.806	0.1659	impact	1.070673	########
C60-C63	1	1915	0.01835	0.552	0.2666	incidence	0.0374	########
C60-C63	1	1915	0.01835	0.552	0.2666	impact	0.5894	########
C60-C63	1	1925	0.01835	0.552	0.2666	incidence	0.2197	########
C60-C63	1	1925	0.01835	0.552	0.2666	impact	0.7717	########
C60-C63	1	1935	0.01835	0.552	0.2666	incidence	0.02896	########
C60-C63	1	1935	0.01835	0.552	0.2666	impact	0.58096	########
C60-C63	1	1945	0.01835	0.552	0.2666	incidence	-0.11864	0.194
C60-C63	1	1945	0.01835	0.552	0.2666	impact	0.43336	########
C60-C63	1	1955	0.01835	0.552	0.2666	incidence	-0.18632	0.13
C60-C63	1	1955	0.01835	0.552	0.2666	impact	0.36568	########
C60-C63	2	1915	0.01534	0.923	0.1908	incidence	0.2296	########
C60-C63	2	1915	0.01534	0.923	0.1908	impact	1.1526	########
C60-C63	2	1925	0.01534	0.923	0.1908	incidence	0.3571	########
C60-C63	2	1925	0.01534	0.923	0.1908	impact	1.2801	########
C60-C63	2	1935	0.01534	0.923	0.1908	incidence	-0.01347	########
C60-C63	2	1935	0.01534	0.923	0.1908	impact	0.90953	########
C60-C63	2	1945	0.01534	0.923	0.1908	incidence	-0.18307	########
C60-C63	2	1945	0.01534	0.923	0.1908	impact	0.73993	########
C60-C63	2	1955	0.01534	0.923	0.1908	incidence	-0.31546	0.125
C60-C63	2	1955	0.01534	0.923	0.1908	impact	0.60754	########
C60-C63	3	1915	0.01468	0.234	0.193	incidence	-0.1596	########
C60-C63	3	1915	0.01468	0.234	0.193	impact	0.0744	########
C60-C63	3	1925	0.01468	0.234	0.193	incidence	0.3335	########
C60-C63	3	1925	0.01468	0.234	0.193	impact	0.5675	########
C60-C63	3	1935	0.01468	0.234	0.193	incidence	0.02698	########
C60-C63	3	1935	0.01468	0.234	0.193	impact	0.26098	########
C60-C63	3	1945	0.01468	0.234	0.193	incidence	-0.19047	########
C60-C63	3	1945	0.01468	0.234	0.193	impact	0.04353	########
C60-C63	3	1955	0.01468	0.234	0.193	incidence	-0.09351	0.145
C60-C63	3	1955	0.01468	0.234	0.193	impact	0.14049	########
C60-C63	4	1915	0.01534	0.624	0.1919	incidence	0.2886	########
C60-C63	4	1915	0.01534	0.624	0.1919	impact	0.9126	########
C60-C63	4	1925	0.01534	0.624	0.1919	incidence	0.3574	########
C60-C63	4	1925	0.01534	0.624	0.1919	impact	0.9814	########
C60-C63	4	1935	0.01534	0.624	0.1919	incidence	0.13604	########
C60-C63	4	1935	0.01534	0.624	0.1919	impact	0.76004	########
C60-C63	4	1945	0.01534	0.624	0.1919	incidence	-0.04672	########
C60-C63	4	1945	0.01534	0.624	0.1919	impact	0.57728	########
C60-C63	4	1955	0.01534	0.624	0.1919	incidence	0.18568	0.141
C60-C63	4	1955	0.01534	0.624	0.1919	impact	0.80968	########
C60-C63	5	1915	0.0146	0.876	0.2221	incidence	0.1024	########
C60-C63	5	1915	0.0146	0.876	0.2221	impact	0.9784	########
C60-C63	5	1925	0.0146	0.876	0.2221	incidence	0.2593	########
C60-C63	5	1925	0.0146	0.876	0.2221	impact	1.1353	########
C60-C63	5	1935	0.0146	0.876	0.2221	incidence	0.17694	########
C60-C63	5	1935	0.0146	0.876	0.2221	impact	1.05294	########
C60-C63	5	1945	0.0146	0.876	0.2221	incidence	-0.13092	########
C60-C63	5	1945	0.0146	0.876	0.2221	impact	0.74508	########
C60-C63	5	1955	0.0146	0.876	0.2221	incidence	-0.18902	0.137
C60-C63	5	1955	0.0146	0.876	0.2221	impact	0.68698	########
C60-C63	6	1915	0.01287	0.86	0.187	incidence	0.7519	########

For peer review only

C60-C63	6	1915	0.01287	0.86	0.187	impact	1.6119	########
C60-C63	6	1925	0.01287	0.86	0.187	incidence	0.3402	########
C60-C63	6	1925	0.01287	0.86	0.187	impact	1.2002	########
C60-C63	6	1935	0.01287	0.86	0.187	incidence	0.16037	########
C60-C63	6	1935	0.01287	0.86	0.187	impact	1.02037	########
C60-C63	6	1945	0.01287	0.86	0.187	incidence	-0.01612	########
C60-C63	6	1945	0.01287	0.86	0.187	impact	0.84388	########
C60-C63	6	1955	0.01287	0.86	0.187	incidence	-0.01925	0.144
C60-C63	6	1955	0.01287	0.86	0.187	impact	0.84075	########
C60-C63	7	1915	0.01208	0.38	0.244	incidence	0.2181	########
C60-C63	7	1915	0.01208	0.38	0.244	impact	0.5981	########
C60-C63	7	1925	0.01208	0.38	0.244	incidence	0.2901	########
C60-C63	7	1925	0.01208	0.38	0.244	impact	0.6701	########
C60-C63	7	1935	0.01208	0.38	0.244	incidence	0.21835	########
C60-C63	7	1935	0.01208	0.38	0.244	impact	0.59835	########
C60-C63	7	1945	0.01208	0.38	0.244	incidence	-0.01326	########
C60-C63	7	1945	0.01208	0.38	0.244	impact	0.36674	########
C60-C63	7	1955	0.01208	0.38	0.244	incidence	0.01891	0.147
C60-C63	7	1955	0.01208	0.38	0.244	impact	0.39891	########
C60-C63	8	1915	0.01184	0.772	0.1313	incidence	0.3018	########
C60-C63	8	1915	0.01184	0.772	0.1313	impact	1.0738	########
C60-C63	8	1925	0.01184	0.772	0.1313	incidence	0.4242	########
C60-C63	8	1925	0.01184	0.772	0.1313	impact	1.1962	########
C60-C63	8	1935	0.01184	0.772	0.1313	incidence	0.19544	########
C60-C63	8	1935	0.01184	0.772	0.1313	impact	0.96744	########
C60-C63	8	1945	0.01184	0.772	0.1313	incidence	-0.06707	########
C60-C63	8	1945	0.01184	0.772	0.1313	impact	0.70493	########
C60-C63	8	1955	0.01184	0.772	0.1313	incidence	-0.00437	0.149
C60-C63	8	1955	0.01184	0.772	0.1313	impact	0.76763	########
C60-C63	9	1915	0.01124	1.29	0.2817	incidence	-0.0299	########
C60-C63	9	1915	0.01124	1.29	0.2817	impact	1.2601	########
C60-C63	9	1925	0.01124	1.29	0.2817	incidence	0.3773	########
C60-C63	9	1925	0.01124	1.29	0.2817	impact	1.6673	########
C60-C63	9	1935	0.01124	1.29	0.2817	incidence	0.15734	########
C60-C63	9	1935	0.01124	1.29	0.2817	impact	1.44734	########
C60-C63	9	1945	0.01124	1.29	0.2817	incidence	-0.02303	########
C60-C63	9	1945	0.01124	1.29	0.2817	impact	1.26697	########
C60-C63	9	1955	0.01124	1.29	0.2817	incidence	-0.00231	0.159
C60-C63	9	1955	0.01124	1.29	0.2817	impact	1.28769	########
C60-C63	10	1915	0.00884	0.618	0.1586	incidence	0.2386	########
C60-C63	10	1915	0.00884	0.618	0.1586	impact	0.8566	########
C60-C63	10	1925	0.00884	0.618	0.1586	incidence	0.5046	########
C60-C63	10	1925	0.00884	0.618	0.1586	impact	1.1226	########
C60-C63	10	1935	0.00884	0.618	0.1586	incidence	0.02038	########
C60-C63	10	1935	0.00884	0.618	0.1586	impact	0.63838	########
C60-C63	10	1945	0.00884	0.618	0.1586	incidence	0.07874	########
C60-C63	10	1945	0.00884	0.618	0.1586	impact	0.69674	########
C60-C63	10	1955	0.00884	0.618	0.1586	incidence	-0.32212	0.153
C60-C63	10	1955	0.00884	0.618	0.1586	impact	0.29588	########
G30-G32	1	1915	0.00293	0.146	0.326	incidence	NA	NA
G30-G32	1	1915	0.00293	0.146	0.326	impact	NA	NA
G30-G32	1	1925	0.00293	0.146	0.326	incidence	NA	NA
G30-G32	1	1925	0.00293	0.146	0.326	impact	NA	NA
G30-G32	1	1935	0.00293	0.146	0.326	incidence	NA	NA
G30-G32	1	1935	0.00293	0.146	0.326	impact	NA	NA
G30-G32	1	1945	0.00293	0.146	0.326	incidence	NA	NA

For peer review only

G30-G32	1	1945	0.00293	0.146	0.326	impact	NA	NA
G30-G32	1	1955	0.00293	0.146	0.326	incidence	0.000132	0.5407
G30-G32	1	1955	0.00293	0.146	0.326	impact	NA	NA
G30-G32	2	1915	0.00272	0.248	0.4579	incidence	NA	NA
G30-G32	2	1915	0.00272	0.248	0.4579	impact	NA	NA
G30-G32	2	1925	0.00272	0.248	0.4579	incidence	NA	NA
G30-G32	2	1925	0.00272	0.248	0.4579	impact	NA	NA
G30-G32	2	1935	0.00272	0.248	0.4579	incidence	NA	NA
G30-G32	2	1935	0.00272	0.248	0.4579	impact	NA	NA
G30-G32	2	1945	0.00272	0.248	0.4579	incidence	NA	NA
G30-G32	2	1945	0.00272	0.248	0.4579	impact	NA	NA
G30-G32	2	1955	0.00272	0.248	0.4579	incidence	0.876307	0.5174
G30-G32	2	1955	0.00272	0.248	0.4579	impact	NA	NA
G30-G32	3	1915	0.00285	-0.361	0.3347	incidence	-0.15082	########
G30-G32	3	1915	0.00285	-0.361	0.3347	impact	-0.51182	########
G30-G32	3	1925	0.00285	-0.361	0.3347	incidence	0.13002	########
G30-G32	3	1925	0.00285	-0.361	0.3347	impact	NA	NA
G30-G32	3	1935	0.00285	-0.361	0.3347	incidence	0.50059	########
G30-G32	3	1935	0.00285	-0.361	0.3347	impact	NA	NA
G30-G32	3	1945	0.00285	-0.361	0.3347	incidence	0.402828	########
G30-G32	3	1945	0.00285	-0.361	0.3347	impact	0.041828	########
G30-G32	3	1955	0.00285	-0.361	0.3347	incidence	0.056312	0.4137
G30-G32	3	1955	0.00285	-0.361	0.3347	impact	-0.304688	########
G30-G32	4	1915	0.00271	-1.27	0.284	incidence	-0.20471	########
G30-G32	4	1915	0.00271	-1.27	0.284	impact	NA	NA
G30-G32	4	1925	0.00271	-1.27	0.284	incidence	NA	NA
G30-G32	4	1925	0.00271	-1.27	0.284	impact	NA	NA
G30-G32	4	1935	0.00271	-1.27	0.284	incidence	NA	NA
G30-G32	4	1935	0.00271	-1.27	0.284	impact	NA	NA
G30-G32	4	1945	0.00271	-1.27	0.284	incidence	0.555061	########
G30-G32	4	1945	0.00271	-1.27	0.284	impact	NA	NA
G30-G32	4	1955	0.00271	-1.27	0.284	incidence	-0.002811	0.4975
G30-G32	4	1955	0.00271	-1.27	0.284	impact	-1.27281	########
G30-G32	5	1915	0.0025	0.876	0.3529	incidence	0.5084	########
G30-G32	5	1915	0.0025	0.876	0.3529	impact	1.3844	########
G30-G32	5	1925	0.0025	0.876	0.3529	incidence	0.46193	########
G30-G32	5	1925	0.0025	0.876	0.3529	impact	NA	NA
G30-G32	5	1935	0.0025	0.876	0.3529	incidence	0.77429	########
G30-G32	5	1935	0.0025	0.876	0.3529	impact	1.65029	########
G30-G32	5	1945	0.0025	0.876	0.3529	incidence	0.90804	########
G30-G32	5	1945	0.0025	0.876	0.3529	impact	1.78404	########
G30-G32	5	1955	0.0025	0.876	0.3529	incidence	-0.50319	0.4297
G30-G32	5	1955	0.0025	0.876	0.3529	impact	0.37281	########
G30-G32	6	1915	0.00272	0.451	0.4303	incidence	0.049	########
G30-G32	6	1915	0.00272	0.451	0.4303	impact	NA	NA
G30-G32	6	1925	0.00272	0.451	0.4303	incidence	0.16399	########
G30-G32	6	1925	0.00272	0.451	0.4303	impact	NA	NA
G30-G32	6	1935	0.00272	0.451	0.4303	incidence	0.74241	########
G30-G32	6	1935	0.00272	0.451	0.4303	impact	NA	NA
G30-G32	6	1945	0.00272	0.451	0.4303	incidence	-0.188646	########
G30-G32	6	1945	0.00272	0.451	0.4303	impact	NA	NA
G30-G32	6	1955	0.00272	0.451	0.4303	incidence	0.026796	0.4083
G30-G32	6	1955	0.00272	0.451	0.4303	impact	NA	NA
G30-G32	7	1915	0.00298	-1.24	0.588	incidence	0.31403	########
G30-G32	7	1915	0.00298	-1.24	0.588	impact	NA	NA
G30-G32	7	1925	0.00298	-1.24	0.588	incidence	0.48051	########

For peer review only

G30-G32	7	1925	0.00298	-1.24	0.588	impact	NA	NA
G30-G32	7	1935	0.00298	-1.24	0.588	incidence	0.53712	########
G30-G32	7	1935	0.00298	-1.24	0.588	impact	NA	NA
G30-G32	7	1945	0.00298	-1.24	0.588	incidence	0.249681	########
G30-G32	7	1945	0.00298	-1.24	0.588	impact	NA	NA
G30-G32	7	1955	0.00298	-1.24	0.588	incidence	0.117089	0.3788
G30-G32	7	1955	0.00298	-1.24	0.588	impact	NA	NA
G30-G32	8	1915	0.00279	0.552	0.4202	incidence	-0.2937	########
G30-G32	8	1915	0.00279	0.552	0.4202	impact	0.2583	########
G30-G32	8	1925	0.00279	0.552	0.4202	incidence	0.36212	########
G30-G32	8	1925	0.00279	0.552	0.4202	impact	0.91412	########
G30-G32	8	1935	0.00279	0.552	0.4202	incidence	0.09948	########
G30-G32	8	1935	0.00279	0.552	0.4202	impact	0.65148	########
G30-G32	8	1945	0.00279	0.552	0.4202	incidence	0.743215	########
G30-G32	8	1945	0.00279	0.552	0.4202	impact	1.295215	########
G30-G32	8	1955	0.00279	0.552	0.4202	incidence	-0.030285	0.3229
G30-G32	8	1955	0.00279	0.552	0.4202	impact	0.521715	########
G30-G32	9	1915	0.00295	0.304	0.2768	incidence	-0.02172	########
G30-G32	9	1915	0.00295	0.304	0.2768	impact	0.28228	########
G30-G32	9	1925	0.00295	0.304	0.2768	incidence	0.11319	########
G30-G32	9	1925	0.00295	0.304	0.2768	impact	0.41719	########
G30-G32	9	1935	0.00295	0.304	0.2768	incidence	0.46207	########
G30-G32	9	1935	0.00295	0.304	0.2768	impact	0.76607	########
G30-G32	9	1945	0.00295	0.304	0.2768	incidence	0.370378	########
G30-G32	9	1945	0.00295	0.304	0.2768	impact	0.674378	########
G30-G32	9	1955	0.00295	0.304	0.2768	incidence	0.285492	0.2957
G30-G32	9	1955	0.00295	0.304	0.2768	impact	0.589492	########
G30-G32	10	1915	0.00313	-0.429	0.12	incidence	-0.03039	########
G30-G32	10	1915	0.00313	-0.429	0.12	impact	-0.45939	########
G30-G32	10	1925	0.00313	-0.429	0.12	incidence	0.37902	########
G30-G32	10	1925	0.00313	-0.429	0.12	impact	-0.04998	########
G30-G32	10	1935	0.00313	-0.429	0.12	incidence	0.5566	########
G30-G32	10	1935	0.00313	-0.429	0.12	impact	0.1276	########
G30-G32	10	1945	0.00313	-0.429	0.12	incidence	0.361747	########
G30-G32	10	1945	0.00313	-0.429	0.12	impact	-0.067253	########
G30-G32	10	1955	0.00313	-0.429	0.12	incidence	0.520203	0.2574
G30-G32	10	1955	0.00313	-0.429	0.12	impact	0.091203	########

For peer review only

strat_var
none
none
none
none
none
none
none
none
none
none
none
none
none
none
none
none
none
none
none
none
none
none
none
none
none
none
none
none
none
none
none
none
none
none
none
none
none
none
none
none
none
none
none
none
none
none
none
none
none
none
none
none
none
none
none
none
none

For peer review only

none
none
none
none
none
none
none
none
none
none
none
none
none
none
none
none
none
none
none
none
none
none
none
none
none
none
none
none
none
none
none
none
none
none
none
none
none
none
none
none
none
none
none
none
none
none
none
none
none
none
none
none
none
none
none
none
none
none

For peer review only

none
none
none
none
none
none
none
none
none
none
none
none
none
none
none
none
none
none
none
none
none
none
none
none
none
none
none
none
none
none
none
none
none
none
none
none
none
none
none
none
none
none
none
none
none
none
none
none
none
none
none
none
none
none
none
none
none
none

For peer review only

none
none
none
none
none
none
none
none
none
none
none
none
none
none
none
none
none
none
none
none
none
none
none
none
none
none
none
none
none
none
none
none
none
none
none
none
none
none
none
none
none
none
none
none
none
none
none
none
none
none
none
none
none
none
none
none
none
none

For peer review only

none
none
none
none
none
none
none
none
none
none
none
none
none
none
none
none
none
none
none
none
none
none
none
none
none
none
none
none
none
none
none
none
none
none
none
none
none
none
none
none
none
none
none
none
none
none
none
none
none
none
none
none
none
none
none
none
none
none

For peer review only

none
none
none
none
none
none
none
none
none
none
none
none
none
none
none
none
none
none
none
none
none
none
sex
sex
sex
sex
sex
sex
sex
sex
sex
sex
sex
sex
sex
sex
sex
sex
sex
sex
sex
sex
sex
sex
sex
sex
sex
sex
sex
sex
sex
sex
sex
sex
sex
sex
sex
sex

For peer review only

sex
sex
sex
sex
sex
sex
sex
sex
sex
sex
sex
sex
sex
sex
sex
sex
sex
sex
sex
sex
sex
sex
sex
sex
sex
sex
sex
sex
sex
sex
sex
sex
sex
sex
sex
sex
sex
sex
sex
sex
sex
sex
sex
sex
sex
sex
sex
sex
sex
sex
sex
sex
sex
sex
sex
sex
sex
sex

For peer review only

sex
sex
sex
sex
sex
sex
sex
sex
sex
sex
sex
sex
sex
sex
sex
sex
sex
sex
sex
sex
sex
sex
sex
sex
sex
sex
sex
sex
sex
sex
sex
sex
sex
sex
sex
sex
sex
sex
sex
sex
sex
sex
sex
sex
sex
sex
sex
sex
sex
sex
sex
sex
sex
sex
sex
sex
sex
sex

For peer review only

sex
sex
sex
sex
sex
sex
sex
sex
sex
sex
sex
sex
sex
sex
sex
sex
sex
sex
sex
sex
sex
sex
sex
sex
sex
sex
sex
sex
sex
sex
sex
sex
sex
sex
sex
sex
sex
sex
sex
sex
sex
sex
sex
sex
sex
sex
sex
sex
sex
sex
sex
sex
sex
sex
sex
sex
sex
sex

For peer review only

sex
sex
sex
sex
sex
sex
sex
sex
sex
sex
sex
sex
sex
sex
sex
sex
sex
sex
sex
sex
sex
sex
sex
sex
sex
sex
sex
sex
sex
sex
sex
sex
sex
sex
sex
sex
sex
sex
sex
sex
sex
sex
sex
sex
sex
sex
sex
sex
sex
sex
sex
sex
sex
sex
sex
sex
sex
sex

For peer review only

sex
sex
sex
sex
sex
sex
sex
sex
sex
sex
sex
sex
sex
sex
sex
sex
sex
sex
sex
sex
sex
sex
sex
sex
sex
sex
sex
sex
sex
sex
sex
sex
sex
sex
sex
sex
sex
sex
sex
sex
sex
sex
sex
sex
sex
sex
sex
sex
sex
sex
sex
sex
sex
sex
sex
sex
sex
sex

For peer review only

sex
sex
sex
sex
sex
sex
sex
sex
sex
sex
sex
sex
sex
sex
sex
sex
sex
sex
sex
sex
sex
sex
sex
sex
sex
sex
sex
sex
sex
sex
sex
sex
sex
sex
sex
sex
sex
sex
sex
sex
sex
sex
sex
sex
sex
sex
sex
sex
sex
sex
sex
sex
sex
sex
sex
sex
sex
sex

For peer review only

sex
sex
sex
sex
sex
sex
sex
sex
sex
sex
sex
sex
sex
sex
sex
sex
sex
sex
sex
sex
sex
sex
sex
sex
sex
sex
sex
sex
sex
sex
sex
sex
sex
sex
sex
sex
sex
sex
sex
sex
sex
sex
sex
sex
sex
sex
sex
sex
sex
sex
sex
sex
sex
sex
sex
sex
sex
sex

For peer review only

sex
sex
sex
sex
sex
sex
sex
sex
sex
sex
sex
sex
sex
sex
sex
sex
sex
sex
sex
sex
sex
sex
sex
sex
sex
sex
sex
sex
sex
sex
sex
sex
sex
sex
sex
sex
sex
sex
sex
sex
sex
sex
sex
sex
sex
sex
sex
sex
sex
sex
sex
sex
sex
sex
sex
sex
sex
sex

For peer review only

sex
sex
sex
sex
sex
sex
sex
sex
sex
sex
sex
sex
sex
sex
sex
sex
sex
sex
sex
sex
sex
sex
sex
sex
sex
sex
sex
sex
sex
sex
sex
sex
sex
sex
sex
sex
sex
sex
sex
sex
sex
sex
sex
sex
sex
sex
sex
sex
sex
sex
sex
sex
sex
sex
sex
sex
sex
sex

For peer review only

sex
sex
sex
sex
sex
sex
sex
sex
sex
sex
sex
sex
sex
sex
sex
sex
sex
sex
sex
sex
sex
sex
sex
sex
sex
sex
sex
sex
sex
sex
sex
sex
sex
sex
sex
sex
sex
sex
sex
sex
sex
sex
sex
sex
sex
sex
sex
sex
sex
sex
sex
sex
sex
sex
sex
sex
sex
sex

For peer review only

sex
sex
sex
sex
sex
deprivation
deprivation
deprivation
deprivation
deprivation
deprivation
deprivation
deprivation
deprivation
deprivation
deprivation
deprivation
deprivation
deprivation
deprivation
deprivation
deprivation
deprivation
deprivation
deprivation
deprivation
deprivation
deprivation
deprivation
deprivation
deprivation
deprivation
deprivation
deprivation
deprivation
deprivation
deprivation
deprivation
deprivation
deprivation
deprivation
deprivation
deprivation
deprivation
deprivation
deprivation
deprivation
deprivation
deprivation
deprivation
deprivation
deprivation
deprivation
deprivation
deprivation
deprivation
deprivation
deprivation

For peer review only

deprivation
deprivation
deprivation
deprivation
deprivation
deprivation
deprivation
deprivation
deprivation
deprivation
deprivation
deprivation
deprivation
deprivation
deprivation
deprivation
deprivation
deprivation
deprivation
deprivation
deprivation
deprivation
deprivation
deprivation
deprivation
deprivation
deprivation
deprivation
deprivation
deprivation
deprivation
deprivation
deprivation
deprivation
deprivation
deprivation
deprivation
deprivation
deprivation
deprivation
deprivation
deprivation
deprivation
deprivation
deprivation
deprivation
deprivation
deprivation
deprivation
deprivation
deprivation
deprivation
deprivation
deprivation
deprivation
deprivation
deprivation
deprivation

For peer review only

deprivation
deprivation
deprivation
deprivation
deprivation
deprivation
deprivation
deprivation
deprivation
deprivation
deprivation
deprivation
deprivation
deprivation
deprivation
deprivation
deprivation
deprivation
deprivation
deprivation
deprivation
deprivation
deprivation
deprivation
deprivation
deprivation
deprivation
deprivation
deprivation
deprivation
deprivation
deprivation
deprivation
deprivation
deprivation
deprivation
deprivation
deprivation
deprivation
deprivation
deprivation
deprivation
deprivation
deprivation
deprivation
deprivation
deprivation
deprivation
deprivation
deprivation
deprivation
deprivation
deprivation
deprivation
deprivation
deprivation
deprivation
deprivation

For peer review only

deprivation
deprivation
deprivation
deprivation
deprivation
deprivation
deprivation
deprivation
deprivation
deprivation
deprivation
deprivation
deprivation
deprivation
deprivation
deprivation
deprivation
deprivation
deprivation
deprivation
deprivation
deprivation
deprivation
deprivation
deprivation
deprivation
deprivation
deprivation
deprivation
deprivation
deprivation
deprivation
deprivation
deprivation
deprivation
deprivation
deprivation
deprivation
deprivation
deprivation
deprivation
deprivation
deprivation
deprivation
deprivation
deprivation
deprivation
deprivation
deprivation
deprivation
deprivation
deprivation
deprivation
deprivation
deprivation
deprivation
deprivation
deprivation

For peer review only

deprivation
deprivation
deprivation
deprivation
deprivation
deprivation
deprivation
deprivation
deprivation
deprivation
deprivation
deprivation
deprivation
deprivation
deprivation
deprivation
deprivation
deprivation
deprivation
deprivation
deprivation
deprivation
deprivation
deprivation
deprivation
deprivation
deprivation
deprivation
deprivation
deprivation
deprivation
deprivation
deprivation
deprivation
deprivation
deprivation
deprivation
deprivation
deprivation
deprivation
deprivation
deprivation
deprivation
deprivation
deprivation
deprivation
deprivation
deprivation
deprivation
deprivation
deprivation
deprivation
deprivation
deprivation
deprivation
deprivation
deprivation
deprivation

For peer review only

deprivation
deprivation
deprivation
deprivation
deprivation
deprivation
deprivation
deprivation
deprivation
deprivation
deprivation
deprivation
deprivation
deprivation
deprivation
deprivation
deprivation
deprivation
deprivation
deprivation
deprivation
deprivation
deprivation
deprivation
deprivation
deprivation
deprivation
deprivation
deprivation
deprivation
deprivation
deprivation
deprivation
deprivation
deprivation
deprivation
deprivation
deprivation
deprivation
deprivation
deprivation
deprivation
deprivation
deprivation
deprivation
deprivation
deprivation
deprivation
deprivation
deprivation
deprivation
deprivation
deprivation
deprivation
deprivation
deprivation
deprivation
deprivation

For peer review only

deprivation
deprivation
deprivation
deprivation
deprivation
deprivation
deprivation
deprivation
deprivation
deprivation
deprivation
deprivation
deprivation
deprivation
deprivation
deprivation
deprivation
deprivation
deprivation
deprivation
deprivation
deprivation
deprivation
deprivation
deprivation
deprivation
deprivation
deprivation
deprivation
deprivation
deprivation
deprivation
deprivation
deprivation
deprivation
deprivation
deprivation
deprivation
deprivation
deprivation
deprivation
deprivation
deprivation
deprivation
deprivation
deprivation
deprivation
deprivation
deprivation
deprivation
deprivation
deprivation
deprivation
deprivation
deprivation
deprivation
deprivation
deprivation

For peer review only

deprivation
deprivation
deprivation
deprivation
deprivation
deprivation
deprivation
deprivation
deprivation
deprivation
deprivation
deprivation
deprivation
deprivation
deprivation
deprivation
deprivation
deprivation
deprivation
deprivation
deprivation
deprivation
deprivation
deprivation
deprivation
deprivation
deprivation
deprivation
deprivation
deprivation
deprivation
deprivation
deprivation
deprivation
deprivation
deprivation
deprivation
deprivation
deprivation
deprivation
deprivation
deprivation
deprivation
deprivation
deprivation
deprivation
deprivation
deprivation
deprivation
deprivation
deprivation
deprivation
deprivation
deprivation
deprivation
deprivation
deprivation
deprivation

For peer review only

deprivation
deprivation
deprivation
deprivation
deprivation
deprivation
deprivation
deprivation
deprivation
deprivation
deprivation
deprivation
deprivation
deprivation
deprivation
deprivation
deprivation
deprivation
deprivation
deprivation
deprivation
deprivation
deprivation
deprivation
deprivation
deprivation
deprivation
deprivation
deprivation
deprivation
deprivation
deprivation
deprivation
deprivation
deprivation
deprivation
deprivation
deprivation
deprivation
deprivation
deprivation
deprivation
deprivation
deprivation
deprivation
deprivation
deprivation
deprivation
deprivation
deprivation
deprivation
deprivation
deprivation
deprivation
deprivation
deprivation
deprivation
deprivation

For peer review only

deprivation
deprivation
deprivation
deprivation
deprivation
deprivation
deprivation
deprivation
deprivation
deprivation
deprivation
deprivation
deprivation
deprivation
deprivation
deprivation
deprivation
deprivation
deprivation
deprivation
deprivation
deprivation
deprivation
deprivation
deprivation
deprivation
deprivation
deprivation
deprivation
deprivation
deprivation
deprivation
deprivation
deprivation
deprivation
deprivation
deprivation
deprivation
deprivation
deprivation
deprivation
deprivation
deprivation
deprivation
deprivation
deprivation
deprivation
deprivation
deprivation
deprivation
deprivation
deprivation
deprivation
deprivation
deprivation
deprivation
deprivation
deprivation

For peer review only

deprivation
deprivation
deprivation
deprivation
deprivation
deprivation
deprivation
deprivation
deprivation
deprivation
deprivation
deprivation
deprivation
deprivation
deprivation
deprivation
deprivation
deprivation
deprivation
deprivation
deprivation
deprivation
deprivation
deprivation
deprivation
deprivation
deprivation
deprivation
deprivation
deprivation
deprivation
deprivation
deprivation
deprivation
deprivation
deprivation
deprivation
deprivation
deprivation
deprivation
deprivation
deprivation
deprivation
deprivation
deprivation
deprivation
deprivation
deprivation
deprivation
deprivation
deprivation
deprivation
deprivation
deprivation
deprivation
deprivation
deprivation
deprivation

For peer review only

deprivation
deprivation
deprivation
deprivation
deprivation
deprivation
deprivation
deprivation
deprivation
deprivation
deprivation
deprivation
deprivation
deprivation
deprivation
deprivation
deprivation
deprivation
deprivation
deprivation
deprivation
deprivation
deprivation
deprivation
deprivation
deprivation
deprivation
deprivation
deprivation
deprivation
deprivation
deprivation
deprivation
deprivation
deprivation
deprivation
deprivation
deprivation
deprivation
deprivation
deprivation
deprivation
deprivation
deprivation
deprivation
deprivation
deprivation
deprivation
deprivation
deprivation
deprivation
deprivation
deprivation
deprivation
deprivation
deprivation
deprivation
deprivation

For peer review only

deprivation
deprivation
deprivation
deprivation
deprivation
deprivation
deprivation
deprivation
deprivation
deprivation
deprivation
deprivation
deprivation
deprivation
deprivation
deprivation
deprivation
deprivation
deprivation
deprivation
deprivation
deprivation
deprivation
deprivation
deprivation
deprivation
deprivation
deprivation
deprivation
deprivation
deprivation
deprivation
deprivation
deprivation
deprivation
deprivation
deprivation
deprivation
deprivation
deprivation
deprivation
deprivation
deprivation
deprivation
deprivation
deprivation
deprivation
deprivation
deprivation
deprivation
deprivation
deprivation
deprivation
deprivation
deprivation
deprivation
deprivation
deprivation

For peer review only

deprivation
deprivation
deprivation
deprivation
deprivation
deprivation
deprivation
deprivation
deprivation
deprivation
deprivation
deprivation
deprivation
deprivation
deprivation
deprivation
deprivation
deprivation
deprivation
deprivation
deprivation
deprivation
deprivation
deprivation
deprivation
deprivation
deprivation
deprivation
deprivation
deprivation
deprivation
deprivation
deprivation
deprivation
deprivation
deprivation
deprivation
deprivation
deprivation
deprivation
deprivation
deprivation
deprivation
deprivation
deprivation
deprivation
deprivation
deprivation
deprivation
deprivation
deprivation
deprivation
deprivation
deprivation
deprivation
deprivation
deprivation
deprivation

For peer review only

deprivation
deprivation
deprivation
deprivation
deprivation
deprivation
deprivation
deprivation
deprivation
deprivation
deprivation
deprivation
deprivation
deprivation
deprivation
deprivation
deprivation
deprivation
deprivation
deprivation
deprivation
deprivation
deprivation
deprivation
deprivation
deprivation
deprivation
deprivation
deprivation
deprivation
deprivation
deprivation
deprivation
deprivation
deprivation
deprivation
deprivation
deprivation
deprivation
deprivation
deprivation
deprivation
deprivation
deprivation
deprivation
deprivation
deprivation
deprivation
deprivation
deprivation
deprivation
deprivation
deprivation
deprivation
deprivation
deprivation
deprivation
deprivation

For peer review only

deprivation
deprivation
deprivation
deprivation
deprivation
deprivation
deprivation
deprivation
deprivation
deprivation
deprivation
deprivation
deprivation
deprivation
deprivation
deprivation
deprivation
deprivation
deprivation
deprivation
deprivation
deprivation
deprivation
deprivation
deprivation
deprivation
deprivation
deprivation
deprivation
deprivation
deprivation
deprivation
deprivation
deprivation
deprivation
deprivation
deprivation
deprivation
deprivation
deprivation
deprivation
deprivation
deprivation
deprivation
deprivation
deprivation
deprivation
deprivation
deprivation
deprivation
deprivation
deprivation
deprivation
deprivation
deprivation
deprivation
deprivation
deprivation

For peer review only

deprivation
deprivation
deprivation
deprivation
deprivation
deprivation
deprivation
deprivation
deprivation
deprivation
deprivation
deprivation
deprivation
deprivation
deprivation
deprivation
deprivation
deprivation
deprivation
deprivation
deprivation
deprivation
deprivation
deprivation
deprivation
deprivation
deprivation
deprivation
deprivation
deprivation
deprivation
deprivation
deprivation
deprivation
deprivation
deprivation
deprivation
deprivation
deprivation
deprivation
deprivation
deprivation
deprivation
deprivation
deprivation
deprivation
deprivation
deprivation
deprivation
deprivation
deprivation
deprivation
deprivation
deprivation
deprivation
deprivation
deprivation
deprivation

For peer review only

deprivation
deprivation
deprivation
deprivation
deprivation
deprivation
deprivation
deprivation
deprivation
deprivation
deprivation
deprivation
deprivation
deprivation
deprivation
deprivation
deprivation
deprivation
deprivation
deprivation
deprivation
deprivation
deprivation
deprivation
deprivation
deprivation
deprivation
deprivation
deprivation
deprivation
deprivation
deprivation
deprivation
deprivation
deprivation
deprivation
deprivation
deprivation
deprivation
deprivation
deprivation
deprivation
deprivation
deprivation
deprivation
deprivation
deprivation
deprivation
deprivation
deprivation
deprivation
deprivation
deprivation
deprivation
deprivation
deprivation
deprivation
deprivation

For peer review only

deprivation
deprivation
deprivation
deprivation
deprivation
deprivation
deprivation
deprivation
deprivation
deprivation
deprivation
deprivation
deprivation
deprivation
deprivation
deprivation
deprivation
deprivation
deprivation
deprivation
deprivation
deprivation
deprivation
deprivation
deprivation
deprivation
deprivation
deprivation
deprivation
deprivation
deprivation
deprivation
deprivation
deprivation
deprivation
deprivation
deprivation
deprivation
deprivation
deprivation
deprivation
deprivation
deprivation
deprivation
deprivation
deprivation
deprivation
deprivation
deprivation
deprivation
deprivation
deprivation
deprivation
deprivation
deprivation
deprivation
deprivation
deprivation

For peer review only

deprivation
deprivation
deprivation
deprivation
deprivation
deprivation
deprivation
deprivation
deprivation
deprivation
deprivation
deprivation
deprivation
deprivation
deprivation
deprivation
deprivation
deprivation
deprivation
deprivation
deprivation
deprivation
deprivation
deprivation
deprivation
deprivation
deprivation
deprivation
deprivation
deprivation
deprivation
deprivation
deprivation
deprivation
deprivation
deprivation
deprivation
deprivation
deprivation
deprivation
deprivation
deprivation
deprivation
deprivation
deprivation
deprivation
deprivation
deprivation
deprivation
deprivation
deprivation
deprivation
deprivation
deprivation
deprivation
deprivation
deprivation
deprivation

For peer review only

deprivation
deprivation
deprivation
deprivation
deprivation
deprivation
deprivation
deprivation
deprivation
deprivation
deprivation
deprivation
deprivation
deprivation
deprivation
deprivation
deprivation
deprivation
deprivation
deprivation
deprivation
deprivation
deprivation
deprivation
deprivation
deprivation
deprivation
deprivation
deprivation
deprivation
deprivation
deprivation
deprivation
deprivation
deprivation
deprivation
deprivation
deprivation
deprivation
deprivation
deprivation
deprivation
deprivation
deprivation
deprivation
deprivation
deprivation
deprivation
deprivation
deprivation
deprivation
deprivation
deprivation
deprivation
deprivation
deprivation
deprivation
deprivation

For peer review only

deprivation
deprivation
deprivation
deprivation
deprivation
deprivation
deprivation
deprivation
deprivation
deprivation
deprivation
deprivation
deprivation
deprivation
deprivation
deprivation
deprivation
deprivation
deprivation
deprivation
deprivation
deprivation
deprivation
deprivation
deprivation
deprivation
deprivation
deprivation
deprivation
deprivation
deprivation
deprivation
deprivation
deprivation
deprivation
deprivation
deprivation
deprivation
deprivation
deprivation
deprivation
deprivation
deprivation
deprivation
deprivation
deprivation
deprivation
deprivation
deprivation
deprivation
deprivation
deprivation
deprivation
deprivation
deprivation
deprivation
deprivation
deprivation

For peer review only

deprivation
deprivation
deprivation
deprivation
deprivation
deprivation
deprivation
deprivation
deprivation
deprivation
deprivation
deprivation
deprivation
deprivation
deprivation
deprivation
deprivation
deprivation
deprivation
deprivation
deprivation
deprivation
deprivation
deprivation
deprivation
deprivation
deprivation
deprivation
deprivation
deprivation
deprivation
deprivation
deprivation
deprivation
deprivation
deprivation
deprivation
deprivation
deprivation
deprivation
deprivation
deprivation
deprivation
deprivation
deprivation
deprivation
deprivation
deprivation
deprivation
deprivation
deprivation
deprivation
deprivation
deprivation
deprivation
deprivation
deprivation
deprivation

For peer review only

deprivation
deprivation
deprivation
deprivation
deprivation
deprivation
deprivation
deprivation
deprivation
deprivation
deprivation
deprivation
deprivation
deprivation
deprivation
deprivation
deprivation
deprivation
deprivation
deprivation
deprivation
deprivation
deprivation
deprivation
deprivation
deprivation
deprivation
deprivation
deprivation
deprivation
deprivation
deprivation
deprivation
deprivation
deprivation
deprivation
deprivation
deprivation
deprivation
deprivation
deprivation
deprivation
deprivation
deprivation
deprivation
deprivation
deprivation
deprivation
deprivation
deprivation
deprivation
deprivation
deprivation
deprivation
deprivation
deprivation
deprivation
deprivation

For peer review only

deprivation
deprivation
deprivation
deprivation
deprivation
deprivation
deprivation
deprivation
deprivation
deprivation
deprivation
deprivation
deprivation
deprivation
deprivation
deprivation
deprivation
deprivation
deprivation
deprivation
deprivation
deprivation
deprivation
deprivation
deprivation
deprivation
deprivation
deprivation
deprivation
deprivation
deprivation
deprivation
deprivation
deprivation
deprivation
deprivation
deprivation
deprivation
deprivation
deprivation
deprivation
deprivation
deprivation
deprivation
deprivation
deprivation
deprivation
deprivation
deprivation
deprivation
deprivation
deprivation
deprivation
deprivation
deprivation
deprivation
deprivation
deprivation

For peer review only

deprivation
deprivation
deprivation
deprivation
deprivation
deprivation
deprivation
deprivation
deprivation
deprivation
deprivation
deprivation
deprivation
deprivation
deprivation
deprivation
deprivation
deprivation
deprivation
deprivation
deprivation
deprivation
deprivation
deprivation
deprivation
deprivation
deprivation
deprivation
deprivation
deprivation
deprivation
deprivation
deprivation
deprivation
deprivation
deprivation
deprivation
deprivation
deprivation
deprivation
deprivation
deprivation
deprivation
deprivation
deprivation
deprivation
deprivation
deprivation
deprivation
deprivation
deprivation
deprivation
deprivation
deprivation
deprivation
deprivation
deprivation
deprivation

For peer review only

deprivation
deprivation
deprivation
deprivation
deprivation
deprivation
deprivation
deprivation
deprivation
deprivation
deprivation
deprivation
deprivation
deprivation
deprivation
deprivation
deprivation
deprivation
deprivation
deprivation
deprivation
deprivation
deprivation
deprivation
deprivation
deprivation
deprivation
deprivation
deprivation
deprivation
deprivation
deprivation
deprivation
deprivation
deprivation
deprivation
deprivation
deprivation
deprivation
deprivation
deprivation
deprivation
deprivation
deprivation
deprivation
deprivation
deprivation
deprivation
deprivation
deprivation
deprivation
deprivation
deprivation
deprivation
deprivation
deprivation
deprivation
deprivation

For peer review only

deprivation
deprivation
deprivation
deprivation
deprivation
deprivation
deprivation
deprivation
deprivation
deprivation
deprivation
deprivation
deprivation
deprivation
deprivation
deprivation
deprivation
deprivation
deprivation
deprivation
deprivation
deprivation
deprivation
deprivation
deprivation
deprivation
deprivation
deprivation
deprivation
deprivation
deprivation
deprivation
deprivation
deprivation
deprivation
deprivation
deprivation
deprivation
deprivation
deprivation
deprivation
deprivation
deprivation
deprivation
deprivation
deprivation
deprivation
deprivation
deprivation
deprivation
deprivation
deprivation
deprivation
deprivation
deprivation
deprivation
deprivation
deprivation

For peer review only

deprivation
deprivation
deprivation
deprivation
deprivation
deprivation
deprivation
deprivation
deprivation
deprivation
deprivation
deprivation
deprivation
deprivation
deprivation
deprivation
deprivation
deprivation
deprivation
deprivation
deprivation
deprivation
deprivation
deprivation
deprivation
deprivation
deprivation
deprivation
deprivation
deprivation
deprivation
deprivation
deprivation
deprivation
deprivation
deprivation
deprivation
deprivation
deprivation
deprivation
deprivation
deprivation
deprivation
deprivation
deprivation
deprivation
deprivation
deprivation
deprivation
deprivation
deprivation
deprivation
deprivation
deprivation
deprivation
deprivation
deprivation
deprivation

For peer review only

deprivation
deprivation
deprivation
deprivation
deprivation
deprivation
deprivation
deprivation
deprivation
deprivation
deprivation
deprivation
deprivation
deprivation
deprivation
deprivation
deprivation
deprivation
deprivation
deprivation
deprivation
deprivation
deprivation
deprivation
deprivation
deprivation
deprivation
deprivation
deprivation
deprivation
deprivation
deprivation
deprivation
deprivation
deprivation
deprivation
deprivation
deprivation
deprivation
deprivation
deprivation
deprivation
deprivation
deprivation
deprivation
deprivation
deprivation
deprivation
deprivation
deprivation
deprivation
deprivation
deprivation
deprivation
deprivation
deprivation
deprivation
deprivation

For peer review only

deprivation
deprivation
deprivation
deprivation
deprivation
deprivation
deprivation
deprivation
deprivation
deprivation
deprivation
deprivation
deprivation
deprivation
deprivation
deprivation
deprivation
deprivation
deprivation
deprivation
deprivation
deprivation
deprivation
deprivation
deprivation
deprivation
deprivation
deprivation
deprivation
deprivation
deprivation
deprivation
deprivation
deprivation
deprivation
deprivation
deprivation
deprivation
deprivation
deprivation
deprivation
deprivation
deprivation
deprivation
deprivation
deprivation
deprivation
deprivation
deprivation
deprivation
deprivation
deprivation
deprivation
deprivation
deprivation
deprivation
deprivation
deprivation

For peer review only

deprivation
deprivation
deprivation
deprivation
deprivation
deprivation
deprivation
deprivation
deprivation
deprivation
deprivation
deprivation
deprivation
deprivation
deprivation
deprivation
deprivation
deprivation
deprivation
deprivation
deprivation
deprivation
deprivation
deprivation
deprivation
deprivation
deprivation
deprivation
deprivation
deprivation
deprivation
deprivation
deprivation
deprivation
deprivation
deprivation
deprivation
deprivation
deprivation
deprivation
deprivation
deprivation
deprivation
deprivation
deprivation
deprivation
deprivation
deprivation
deprivation
deprivation
deprivation
deprivation
deprivation
deprivation
deprivation
deprivation
deprivation
deprivation

For peer review only

deprivation
deprivation
deprivation
deprivation
deprivation
deprivation
deprivation
deprivation
deprivation
deprivation
deprivation
deprivation
deprivation
deprivation
deprivation
deprivation
deprivation
deprivation
deprivation
deprivation
deprivation
deprivation
deprivation
deprivation
deprivation
deprivation
deprivation
deprivation
deprivation
deprivation
deprivation
deprivation
deprivation
deprivation
deprivation
deprivation
deprivation
deprivation
deprivation
deprivation
deprivation
deprivation
deprivation
deprivation
deprivation
deprivation
deprivation
deprivation
deprivation
deprivation
deprivation
deprivation
deprivation
deprivation
deprivation
deprivation
deprivation
deprivation

For peer review only

deprivation
deprivation
deprivation
deprivation
deprivation
deprivation
deprivation
deprivation
deprivation
deprivation
deprivation
deprivation
deprivation
deprivation
deprivation
deprivation
deprivation
deprivation
deprivation
deprivation
deprivation
deprivation
deprivation
deprivation
deprivation
deprivation
deprivation
deprivation
deprivation
deprivation
deprivation
deprivation
deprivation
deprivation
deprivation
deprivation
deprivation
deprivation
deprivation
deprivation
deprivation
deprivation
deprivation
deprivation
deprivation
deprivation
deprivation
deprivation
deprivation
deprivation
deprivation
deprivation
deprivation
deprivation
deprivation
deprivation
deprivation
deprivation

For peer review only

deprivation
deprivation
deprivation
deprivation
deprivation
deprivation
deprivation
deprivation
deprivation
deprivation
deprivation
deprivation
deprivation
deprivation
deprivation
deprivation
deprivation
deprivation
deprivation
deprivation
deprivation
deprivation
deprivation
deprivation
deprivation
deprivation
deprivation
deprivation
deprivation
deprivation
deprivation
deprivation
deprivation
deprivation
deprivation
deprivation
deprivation
deprivation
deprivation
deprivation
deprivation
deprivation
deprivation
deprivation
deprivation
deprivation
deprivation
deprivation
deprivation
deprivation
deprivation
deprivation
deprivation
deprivation
deprivation
deprivation
deprivation
deprivation

For peer review only

deprivation
deprivation
deprivation
deprivation
deprivation
deprivation
deprivation
deprivation
deprivation
deprivation
deprivation
deprivation
deprivation
deprivation
deprivation
deprivation
deprivation
deprivation
deprivation
deprivation
deprivation
deprivation
deprivation
deprivation
deprivation
deprivation
deprivation
deprivation
deprivation
deprivation
deprivation
deprivation
deprivation
deprivation
deprivation
deprivation
deprivation
deprivation
deprivation
deprivation
deprivation
deprivation
deprivation
deprivation
deprivation
deprivation
deprivation
deprivation
deprivation
deprivation
deprivation
deprivation
deprivation
deprivation
deprivation
deprivation
deprivation
deprivation

For peer review only

deprivation
deprivation
deprivation
deprivation
deprivation
deprivation
deprivation
deprivation
deprivation
deprivation
deprivation
deprivation
deprivation
deprivation
deprivation
deprivation
deprivation
deprivation
deprivation
deprivation
deprivation
deprivation
deprivation
deprivation
deprivation
deprivation
deprivation
deprivation
deprivation
deprivation
deprivation
deprivation
deprivation
deprivation
deprivation
deprivation
deprivation
deprivation
deprivation
deprivation
deprivation
deprivation
deprivation
deprivation
deprivation
deprivation
deprivation
deprivation
deprivation
deprivation
deprivation
deprivation
deprivation
deprivation
deprivation
deprivation
deprivation
deprivation

For peer review only

deprivation
deprivation
deprivation
deprivation
deprivation
deprivation
deprivation
deprivation
deprivation
deprivation
deprivation
deprivation
deprivation
deprivation
deprivation
deprivation
deprivation
deprivation
deprivation
deprivation
deprivation
deprivation
deprivation
deprivation
deprivation
deprivation
deprivation
deprivation
deprivation
deprivation
deprivation
deprivation
deprivation
deprivation
deprivation
deprivation
deprivation
deprivation
deprivation
deprivation
deprivation
deprivation
deprivation
deprivation
deprivation
deprivation
deprivation
deprivation
deprivation
deprivation
deprivation
deprivation
deprivation
deprivation
deprivation
deprivation
deprivation
deprivation

For peer review only

deprivation
deprivation
deprivation
deprivation
deprivation
deprivation
deprivation
deprivation
deprivation
deprivation
deprivation
deprivation
deprivation
deprivation
deprivation
deprivation
deprivation
deprivation
deprivation
deprivation
deprivation
deprivation
deprivation
deprivation
deprivation
deprivation
deprivation
deprivation
deprivation
deprivation
deprivation
deprivation
deprivation
deprivation
deprivation
deprivation
deprivation
deprivation
deprivation
deprivation
deprivation
deprivation
deprivation
deprivation
deprivation
deprivation
deprivation
deprivation
deprivation
deprivation
deprivation
deprivation
deprivation
deprivation
deprivation
deprivation
deprivation
deprivation

For peer review only

deprivation
deprivation
deprivation
deprivation
deprivation
deprivation
deprivation
deprivation
deprivation
deprivation
deprivation
deprivation
deprivation
deprivation
deprivation
deprivation
deprivation
deprivation
deprivation
deprivation
deprivation
deprivation
deprivation
deprivation
deprivation
deprivation
deprivation
deprivation
deprivation
deprivation
deprivation
deprivation
deprivation
deprivation
deprivation
deprivation
deprivation
deprivation
deprivation
deprivation
deprivation
deprivation
deprivation
deprivation
deprivation
deprivation
deprivation
deprivation
deprivation
deprivation
deprivation
deprivation
deprivation
deprivation
deprivation
deprivation
deprivation
deprivation

For peer review only

deprivation
deprivation
deprivation
deprivation
deprivation
deprivation
deprivation
deprivation
deprivation
deprivation
deprivation
deprivation
deprivation
deprivation
deprivation
deprivation
deprivation
deprivation
deprivation
deprivation
deprivation
deprivation
deprivation
deprivation
deprivation
deprivation
deprivation
deprivation
deprivation
deprivation
deprivation
deprivation
deprivation
deprivation
deprivation
deprivation
deprivation
deprivation
deprivation
deprivation
deprivation
deprivation
deprivation
deprivation
deprivation
deprivation
deprivation
deprivation
deprivation
deprivation
deprivation
deprivation
deprivation
deprivation
deprivation
deprivation
deprivation
deprivation

For peer review only

deprivation
deprivation
deprivation
deprivation
deprivation
deprivation
deprivation
deprivation
deprivation
deprivation
deprivation
deprivation
deprivation
deprivation
deprivation
deprivation
deprivation
deprivation
deprivation
deprivation
deprivation
deprivation
deprivation
deprivation
deprivation
deprivation
deprivation
deprivation
deprivation
deprivation
deprivation
deprivation
deprivation
deprivation
deprivation
deprivation
deprivation
deprivation
deprivation
deprivation
deprivation
deprivation
deprivation
deprivation
deprivation
deprivation
deprivation
deprivation
deprivation
deprivation
deprivation
deprivation
deprivation
deprivation
deprivation
deprivation
deprivation
deprivation

For peer review only

deprivation
deprivation
deprivation
deprivation
deprivation
deprivation
deprivation
deprivation
deprivation
deprivation
deprivation
deprivation
deprivation
deprivation
deprivation
deprivation
deprivation
deprivation
deprivation
deprivation
deprivation
deprivation
deprivation
deprivation
deprivation
deprivation
deprivation
deprivation
deprivation
deprivation
deprivation
deprivation
deprivation
deprivation
deprivation
deprivation
deprivation
deprivation
deprivation
deprivation
deprivation
deprivation
deprivation
deprivation
deprivation
deprivation
deprivation
deprivation
deprivation
deprivation
deprivation
deprivation
deprivation
deprivation
deprivation
deprivation
deprivation
deprivation

For peer review only

deprivation
deprivation
deprivation
deprivation
deprivation
deprivation
deprivation
deprivation
deprivation
deprivation
deprivation
deprivation
deprivation
deprivation
deprivation
deprivation
deprivation
deprivation
deprivation
deprivation
deprivation
deprivation
deprivation
deprivation
deprivation
deprivation
deprivation
deprivation
deprivation
deprivation
deprivation
deprivation
deprivation
deprivation
deprivation
deprivation
deprivation
deprivation
deprivation
deprivation
deprivation
deprivation
deprivation
deprivation
deprivation
deprivation
deprivation
deprivation
deprivation
deprivation
deprivation
deprivation
deprivation
deprivation
deprivation
deprivation
deprivation
deprivation

For peer review only

deprivation
deprivation
deprivation
deprivation
deprivation
deprivation
deprivation
deprivation
deprivation
deprivation
deprivation
deprivation
deprivation
deprivation
deprivation
deprivation
deprivation
deprivation
deprivation
deprivation
deprivation
deprivation
deprivation
deprivation
deprivation
deprivation
deprivation
deprivation
deprivation
deprivation
deprivation
deprivation
deprivation
deprivation
deprivation
deprivation
deprivation
deprivation
deprivation
deprivation
deprivation
deprivation
deprivation
deprivation
deprivation
deprivation
deprivation
deprivation
deprivation
deprivation
deprivation
deprivation
deprivation
deprivation
deprivation
deprivation
deprivation
deprivation

For peer review only

deprivation
deprivation
deprivation
deprivation
deprivation
deprivation
deprivation
deprivation
deprivation
deprivation
deprivation
deprivation
deprivation
deprivation
deprivation
deprivation
deprivation
deprivation
deprivation
deprivation
deprivation
deprivation
deprivation
deprivation
deprivation
deprivation
deprivation
deprivation
deprivation
deprivation
deprivation
deprivation
deprivation
deprivation
deprivation
deprivation
deprivation
deprivation
deprivation
deprivation
deprivation
deprivation
deprivation
deprivation
deprivation
deprivation
deprivation
deprivation
deprivation
deprivation
deprivation
deprivation
deprivation
deprivation
deprivation
deprivation
deprivation
deprivation

For peer review only

deprivation
deprivation
deprivation
deprivation
deprivation
deprivation
deprivation
deprivation
deprivation
deprivation
deprivation
deprivation
deprivation
deprivation
deprivation
deprivation
deprivation
deprivation
deprivation
deprivation
deprivation
deprivation
deprivation
deprivation
deprivation
deprivation
deprivation
deprivation
deprivation
deprivation
deprivation
deprivation
deprivation
deprivation
deprivation
deprivation
deprivation
deprivation
deprivation
deprivation
deprivation
deprivation
deprivation
deprivation
deprivation
deprivation
deprivation
deprivation
deprivation
deprivation
deprivation
deprivation
deprivation
deprivation
deprivation
deprivation
deprivation
deprivation

For peer review only

deprivation
deprivation
deprivation
deprivation
deprivation
deprivation
deprivation
deprivation
deprivation
deprivation
deprivation
deprivation
deprivation
deprivation
deprivation
deprivation
deprivation
deprivation
deprivation
deprivation
deprivation
deprivation
deprivation
deprivation
deprivation
deprivation
deprivation
deprivation
deprivation
deprivation
deprivation
deprivation
deprivation
deprivation
deprivation
deprivation
deprivation
deprivation
deprivation
deprivation
deprivation
deprivation
deprivation
deprivation
deprivation
deprivation
deprivation
deprivation
deprivation
deprivation
deprivation
deprivation
deprivation
deprivation
deprivation
deprivation
deprivation
deprivation

For peer review only

deprivation
deprivation
deprivation
deprivation
deprivation
deprivation
deprivation
deprivation
deprivation
deprivation
deprivation
deprivation
deprivation
deprivation
deprivation
deprivation
deprivation
deprivation
deprivation
deprivation
deprivation
deprivation
deprivation
deprivation
deprivation
deprivation
deprivation
deprivation
deprivation
deprivation
deprivation
deprivation
deprivation
deprivation
deprivation
deprivation
deprivation
deprivation
deprivation
deprivation
deprivation
deprivation
deprivation
deprivation
deprivation
deprivation
deprivation
deprivation
deprivation
deprivation
deprivation
deprivation
deprivation
deprivation
deprivation
deprivation
deprivation
deprivation

For peer review only

deprivation
deprivation
deprivation
deprivation
deprivation
deprivation
deprivation
deprivation
deprivation
deprivation
deprivation
deprivation
deprivation
deprivation
deprivation
deprivation
deprivation
deprivation
deprivation
deprivation
deprivation
deprivation
deprivation
deprivation
deprivation
deprivation
deprivation
deprivation
deprivation
deprivation
deprivation
deprivation
deprivation
deprivation
deprivation
deprivation
deprivation
deprivation
deprivation
deprivation
deprivation
deprivation
deprivation
deprivation
deprivation
deprivation
deprivation
deprivation
deprivation
deprivation
deprivation
deprivation
deprivation
deprivation
deprivation
deprivation
deprivation
deprivation

For peer review only

deprivation
deprivation
deprivation
deprivation
deprivation
deprivation
deprivation
deprivation
deprivation
deprivation
deprivation
deprivation
deprivation
deprivation
deprivation
deprivation
deprivation
deprivation
deprivation
deprivation
deprivation
deprivation
deprivation
deprivation
deprivation
deprivation
deprivation
deprivation
deprivation
deprivation
deprivation
deprivation
deprivation
deprivation
deprivation
deprivation
deprivation
deprivation
deprivation
deprivation
deprivation
deprivation
deprivation
deprivation
deprivation
deprivation
deprivation
deprivation
deprivation
deprivation
deprivation
deprivation
deprivation
deprivation
deprivation
deprivation
deprivation
deprivation

For peer review only

deprivation
deprivation
deprivation
deprivation
deprivation
deprivation
deprivation
deprivation
deprivation
deprivation
deprivation
deprivation
deprivation
deprivation
deprivation
deprivation
deprivation
deprivation
deprivation
deprivation
deprivation
deprivation
deprivation
deprivation
deprivation
deprivation
deprivation
deprivation
deprivation
deprivation
deprivation
deprivation
deprivation
deprivation
deprivation
deprivation
deprivation
deprivation
deprivation
deprivation
deprivation
deprivation
deprivation
deprivation
deprivation
deprivation
deprivation
deprivation
deprivation
deprivation
deprivation
deprivation
deprivation
deprivation
deprivation
deprivation
deprivation
deprivation

For peer review only

deprivation
deprivation
deprivation
deprivation
deprivation
deprivation
deprivation
deprivation
deprivation
deprivation
deprivation
deprivation
deprivation
deprivation
deprivation
deprivation
deprivation
deprivation
deprivation
deprivation
deprivation
deprivation
deprivation
deprivation
deprivation
deprivation
deprivation
deprivation
deprivation
deprivation
deprivation
deprivation
deprivation
deprivation
deprivation
deprivation
deprivation
deprivation
deprivation
deprivation
deprivation
deprivation
deprivation
deprivation
deprivation
deprivation
deprivation
deprivation
deprivation
deprivation
deprivation
deprivation
deprivation
deprivation
deprivation
deprivation
deprivation
deprivation

For peer review only

deprivation
deprivation
deprivation
deprivation
deprivation
deprivation
deprivation
deprivation
deprivation
deprivation
deprivation
deprivation
deprivation
deprivation
deprivation
deprivation
deprivation
deprivation
deprivation
deprivation
deprivation
deprivation
deprivation
deprivation
deprivation
deprivation
deprivation
deprivation
deprivation
deprivation
deprivation
deprivation
deprivation
deprivation
deprivation
deprivation
deprivation
deprivation

For peer review only

sex: 1

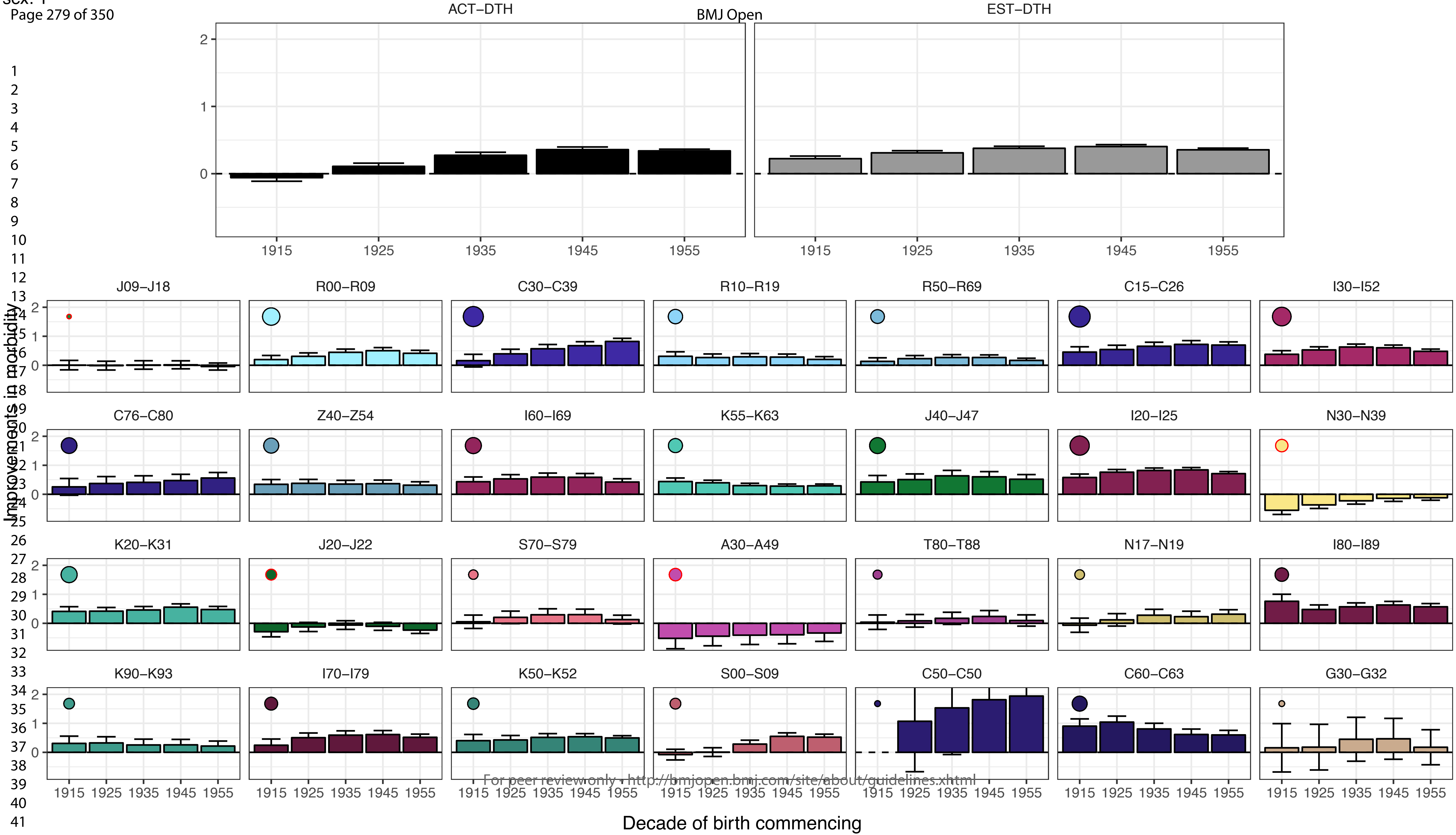

For peer review only - http://bmjopen.bmj.com/site/about/guidelines.xhtml

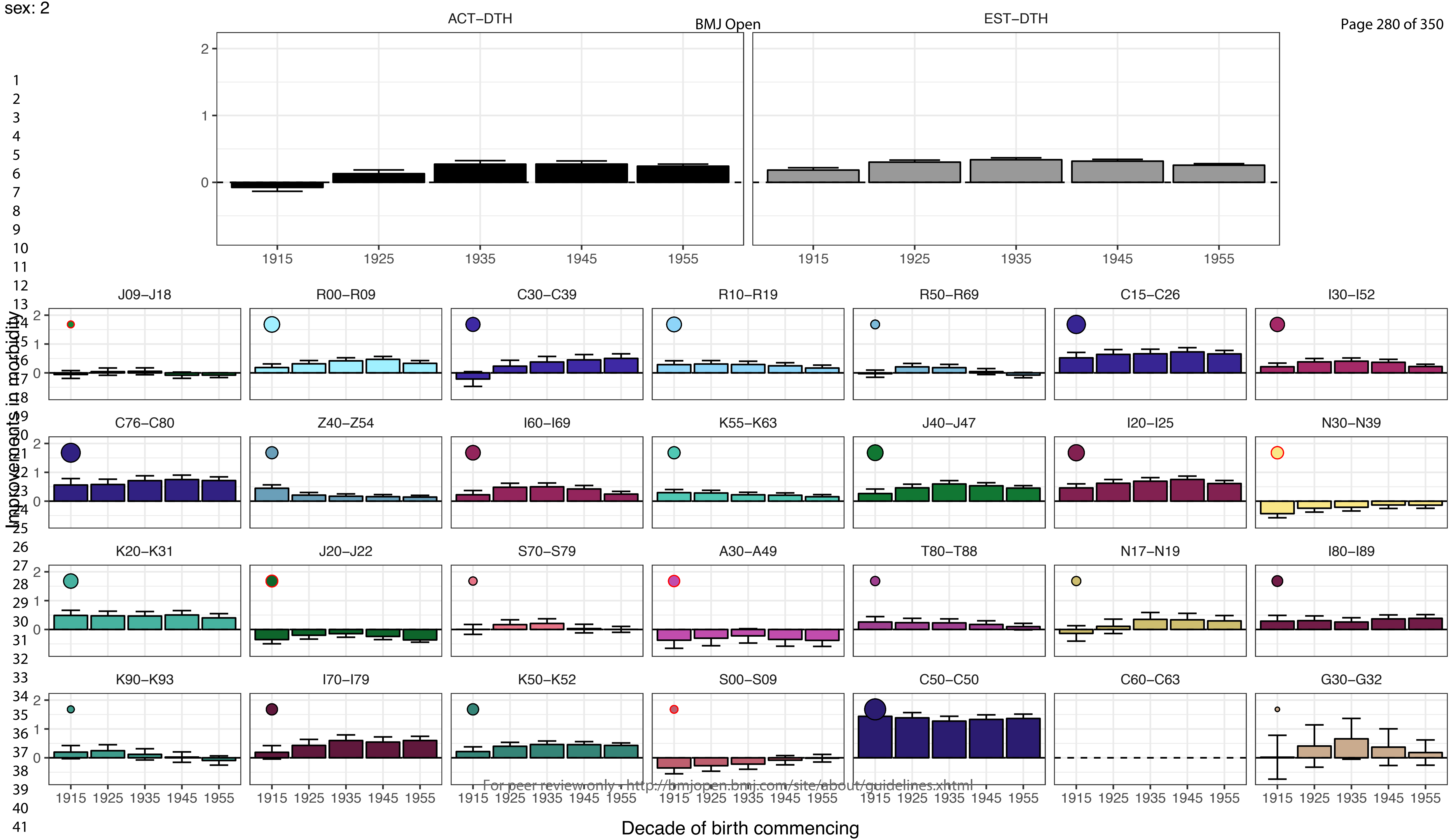
sex: 2
ACT–DTH
EST–DTH
J09–J18
R00–R09
C30–C39
R10–R19
R50–R69
C15–C26
I30–I52
C76–C80
Z40–Z54
I60–I69
K55–K63
J40–J47
I20–I25
N30–N39
K20–K31
J20–J22
S70–S79
A30–A49
T80–T88
N17–N19
I80–I89
K90–K93
I70–I79
K50–K52
S00–S09
C50–C50
C60–C63
G30–G32
Improvements in morbidity
Decade of birth commencing
1915 1925 1935 1945 1955

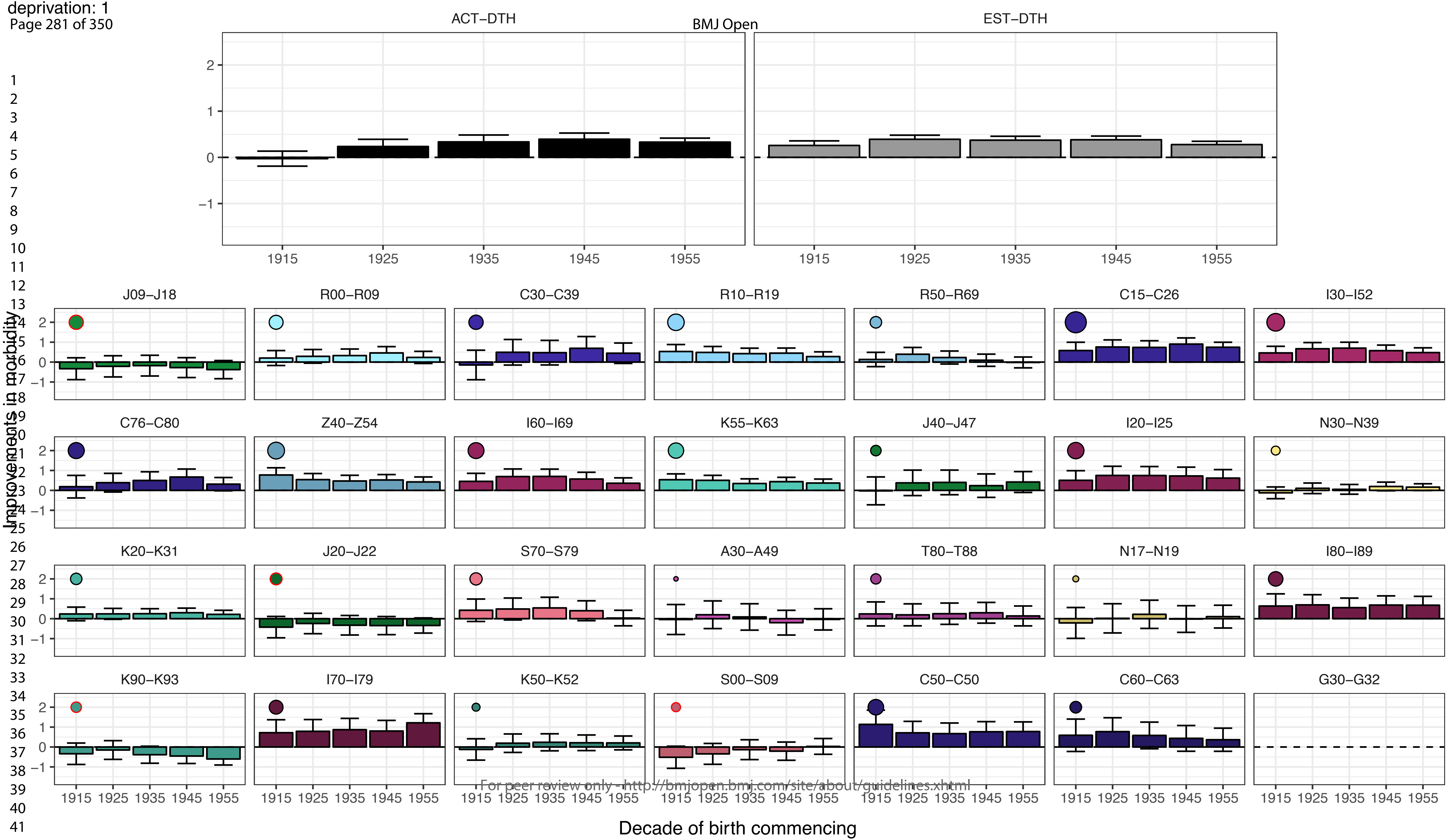

For peer review only - http://bmjopen.bmj.com/site/about/guidelines.xhtml

deprivation: 2

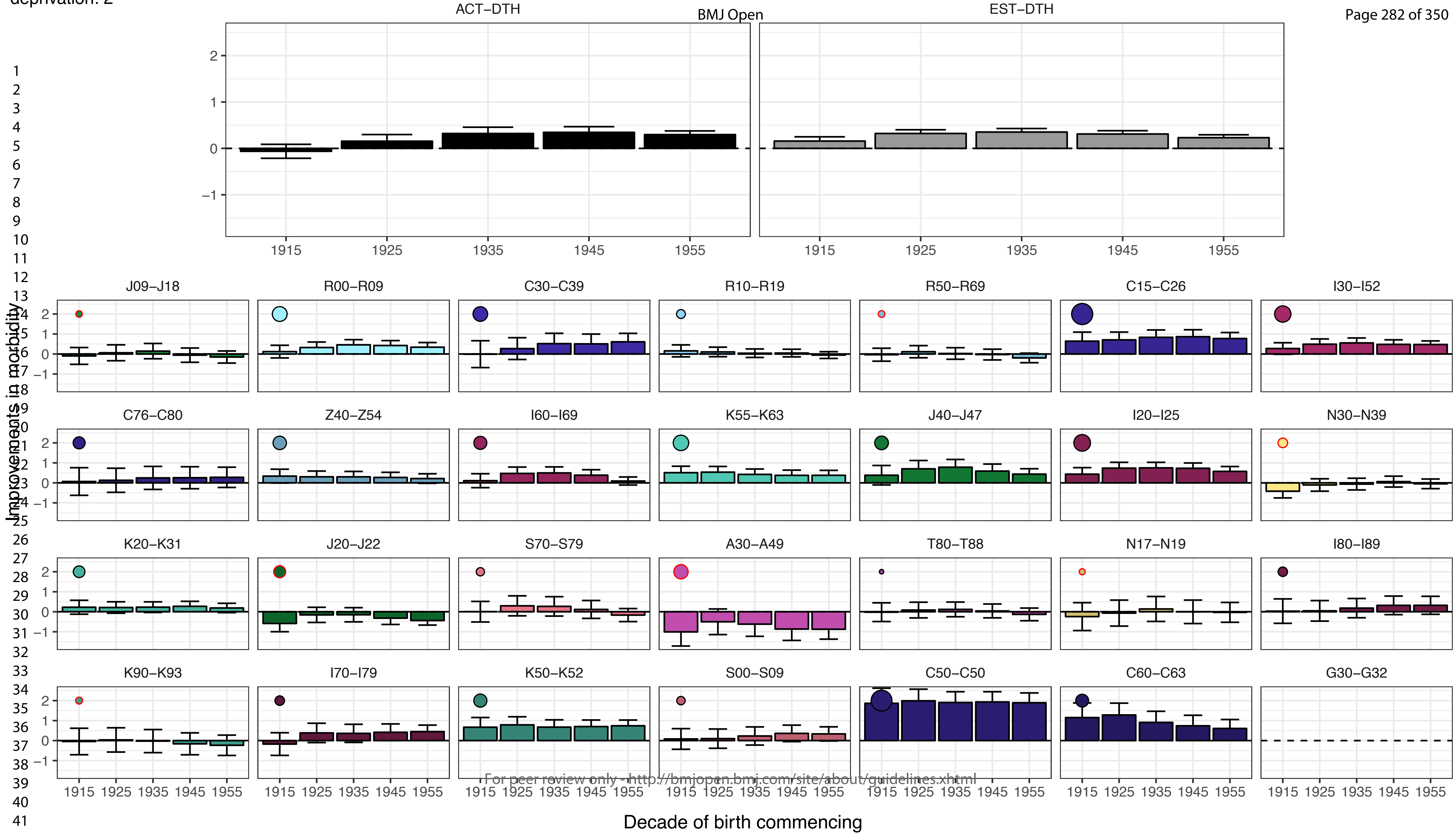

For peer review only - http://bmjopen.bmj.com/site/about/guidelines.xhtml

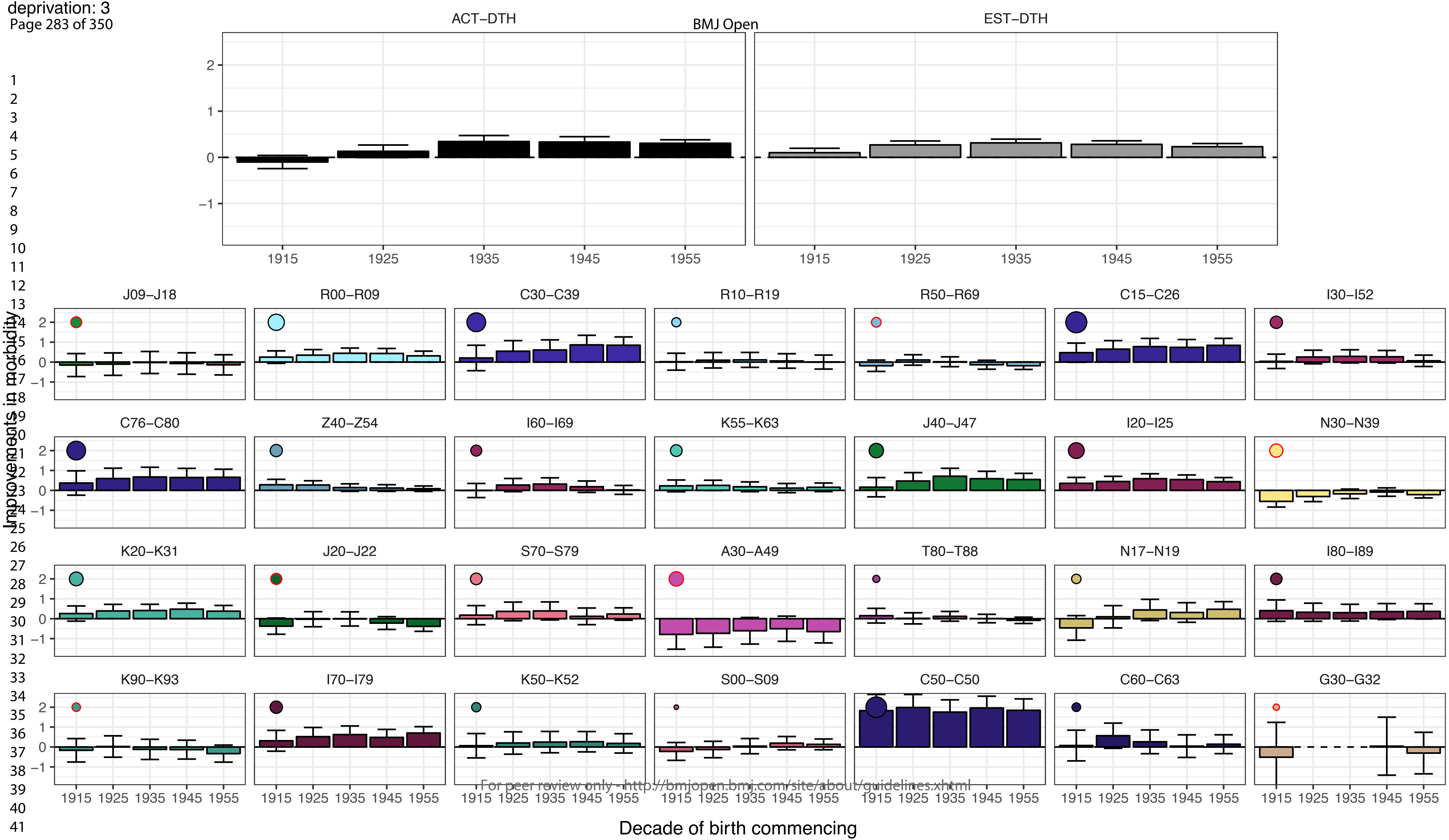

For peer review only - http://bmjopen.bmj.com/site/about/guidelines.xhtml

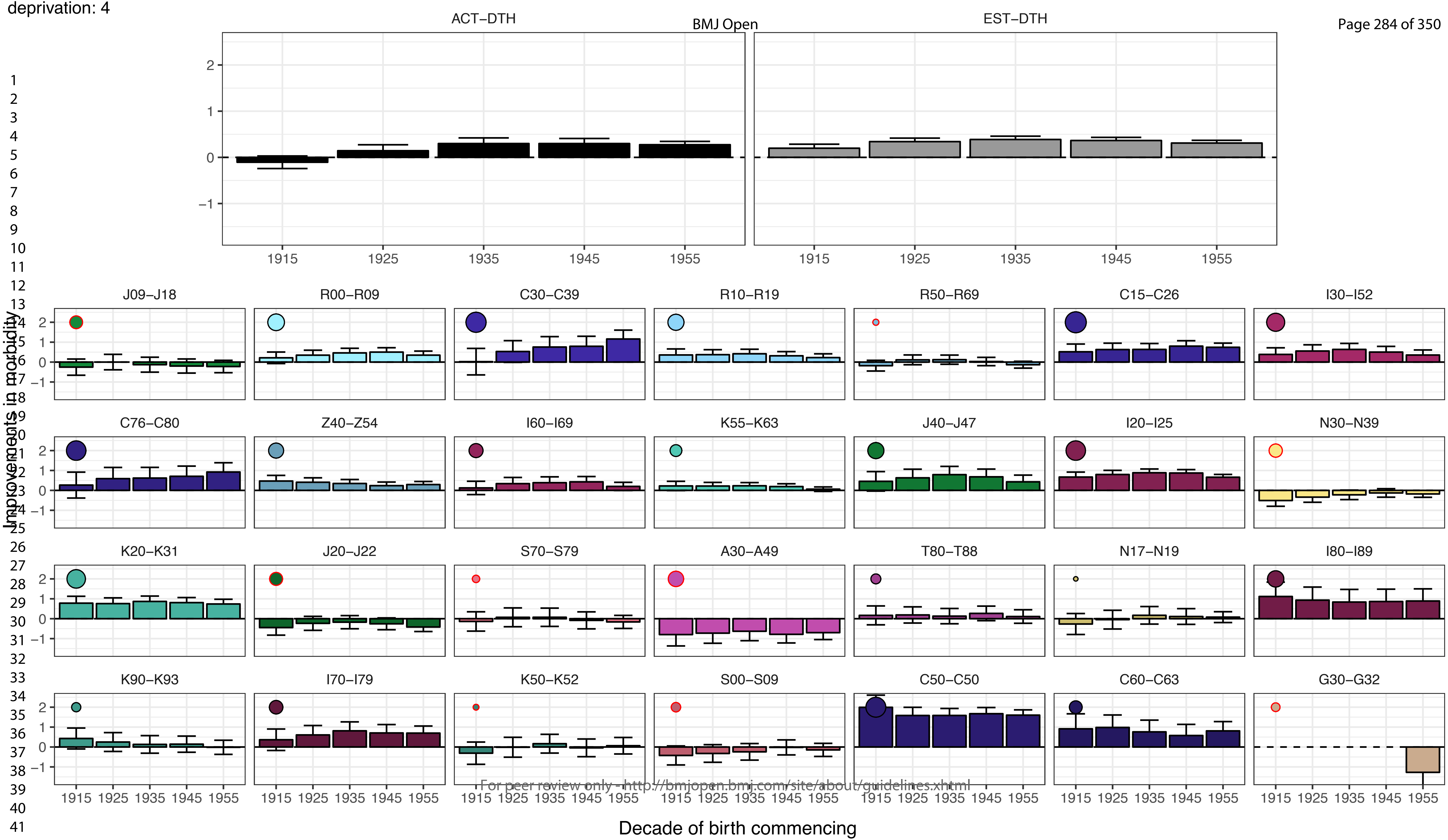

For peer review only - http://bmjopen.bmj.com/site/about/guidelines.xhtml

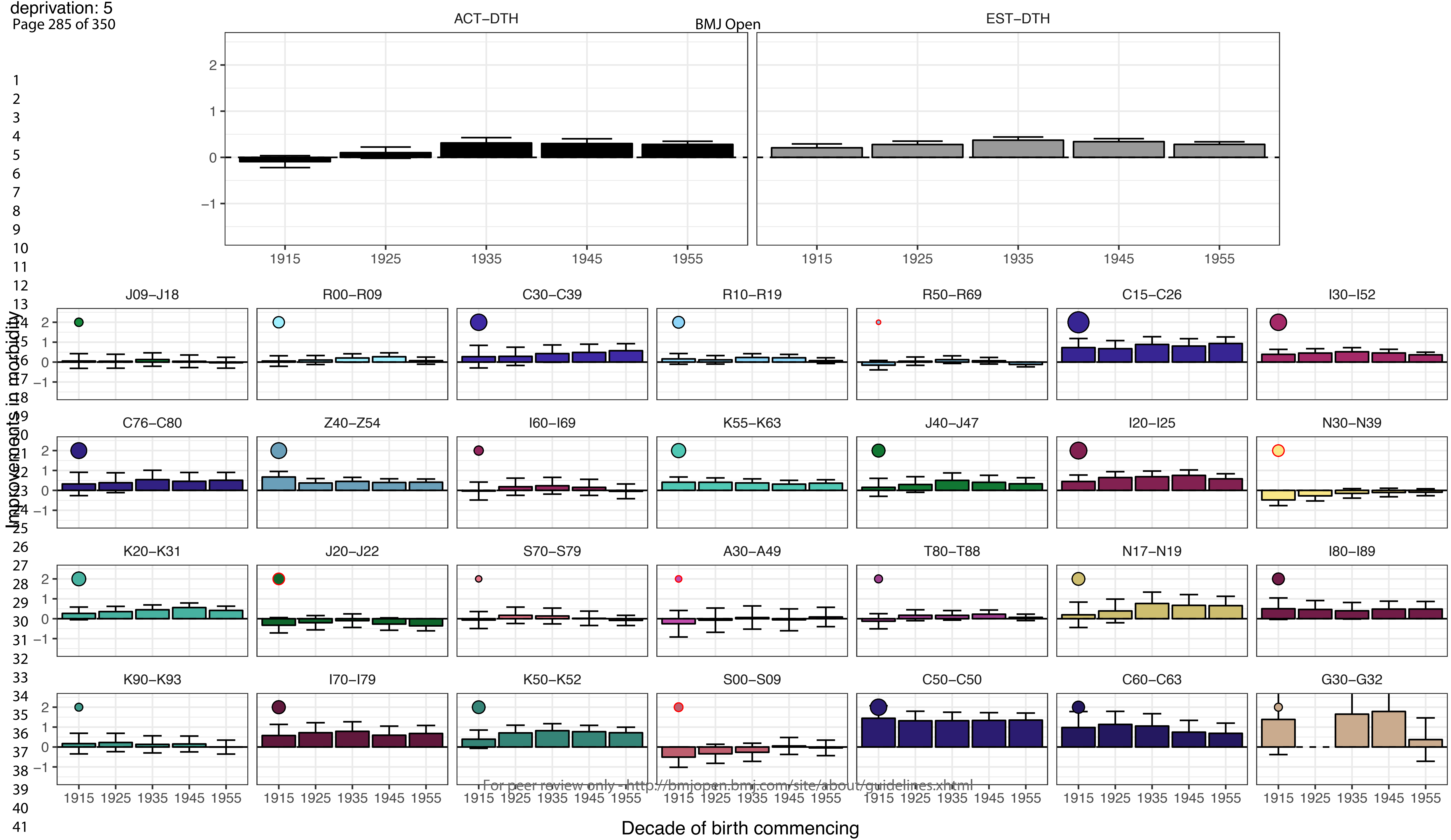

For peer review only - http://bmjopen.bmj.com/site/about/guidelines.xhtml

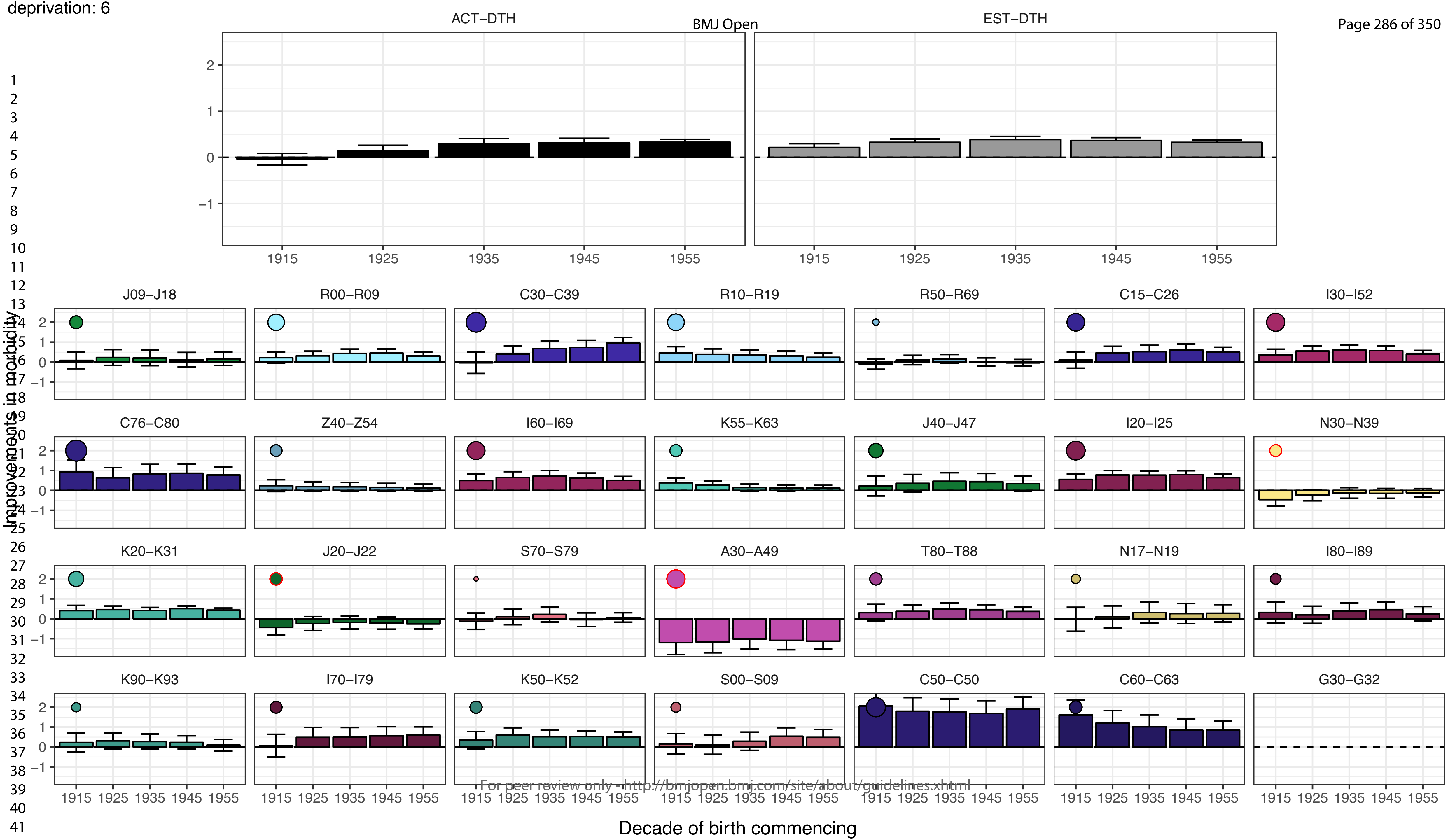

For peer review only - http://bmjopen.bmj.com/site/about/guidelines.xhtml

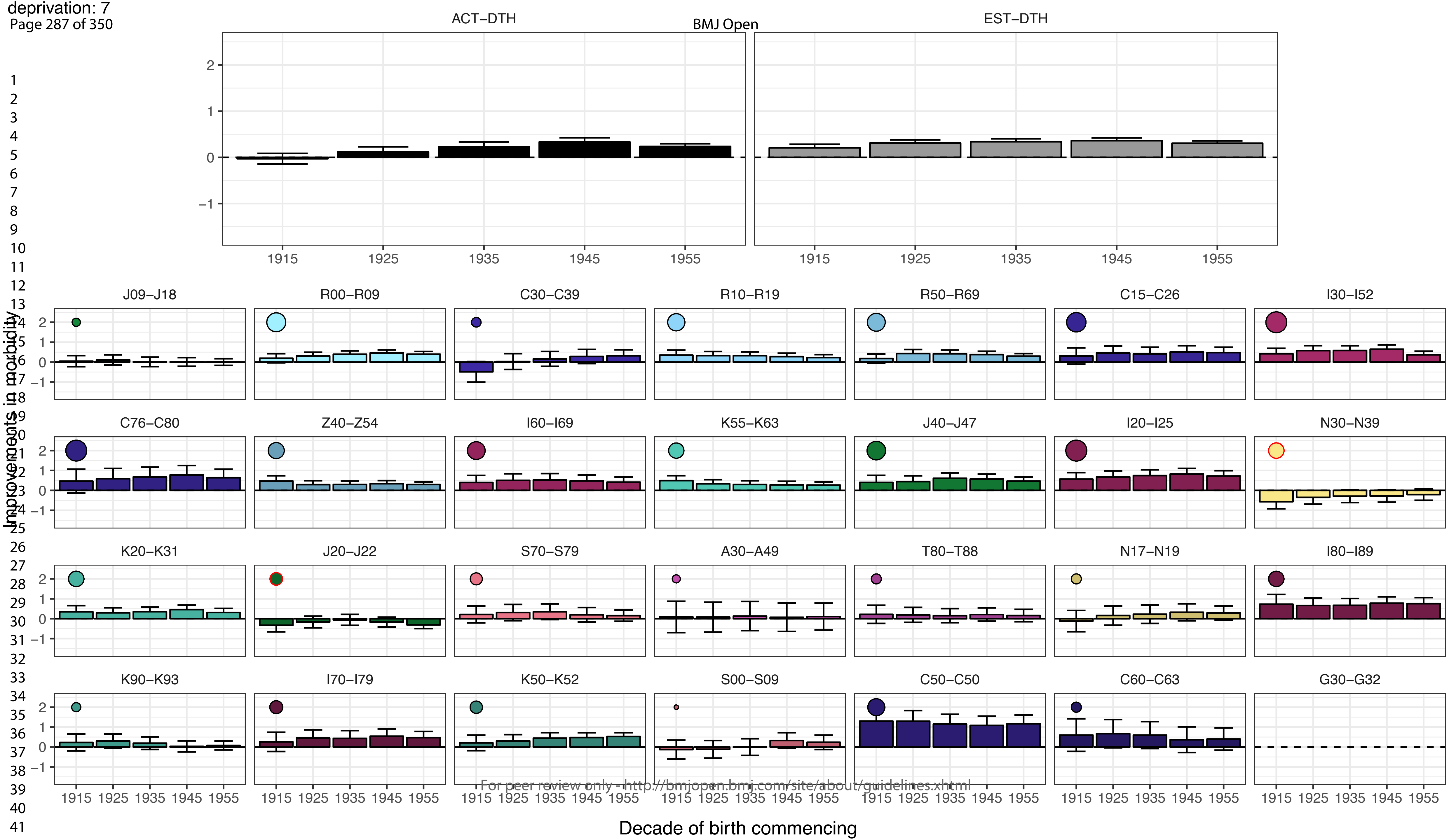

For peer review only - http://bmjopen.bmj.com/site/about/guidelines.xhtml

deprivation: 8

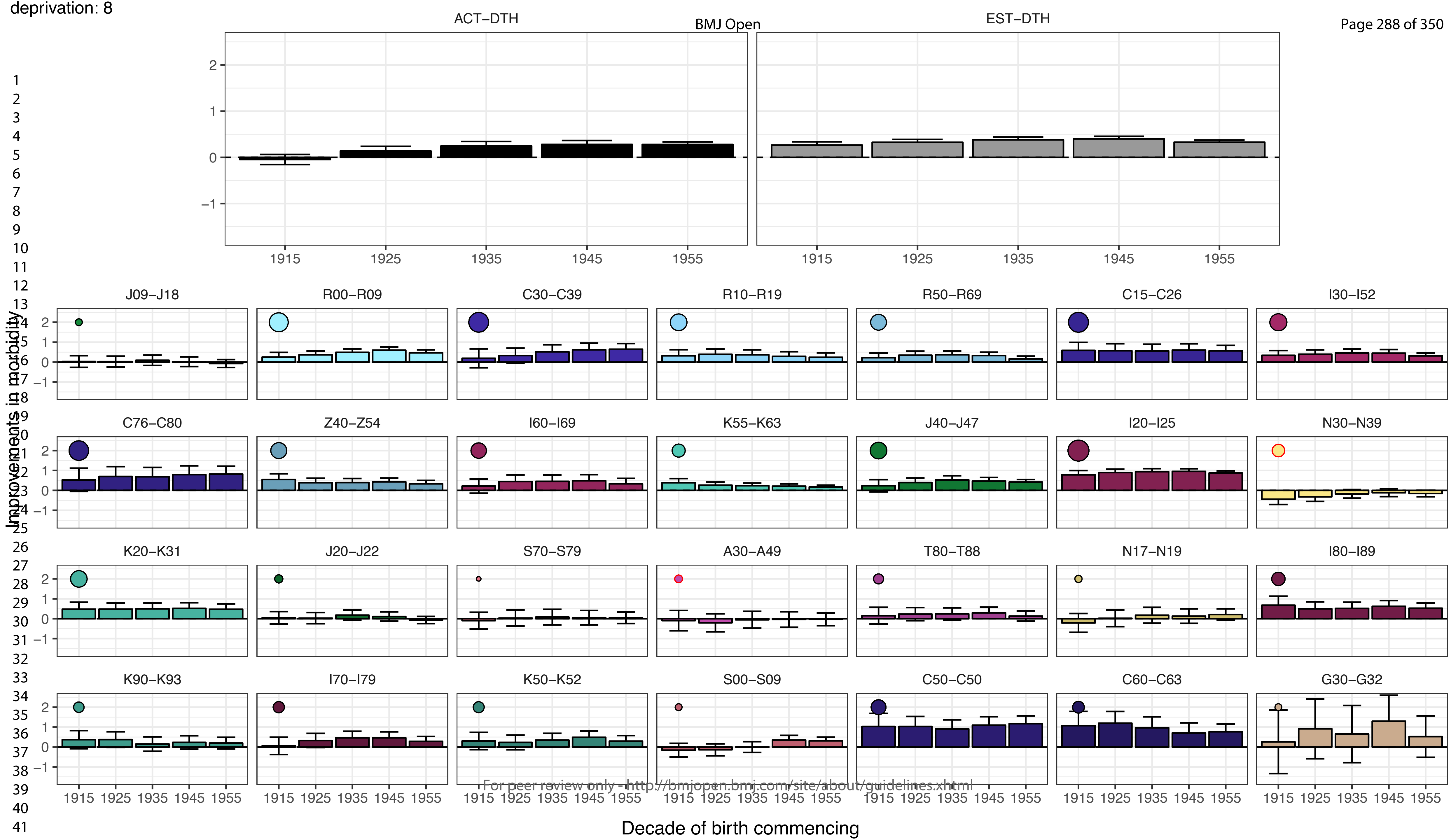

For peer review only - http://bmjopen.bmj.com/site/about/guidelines.xhtml

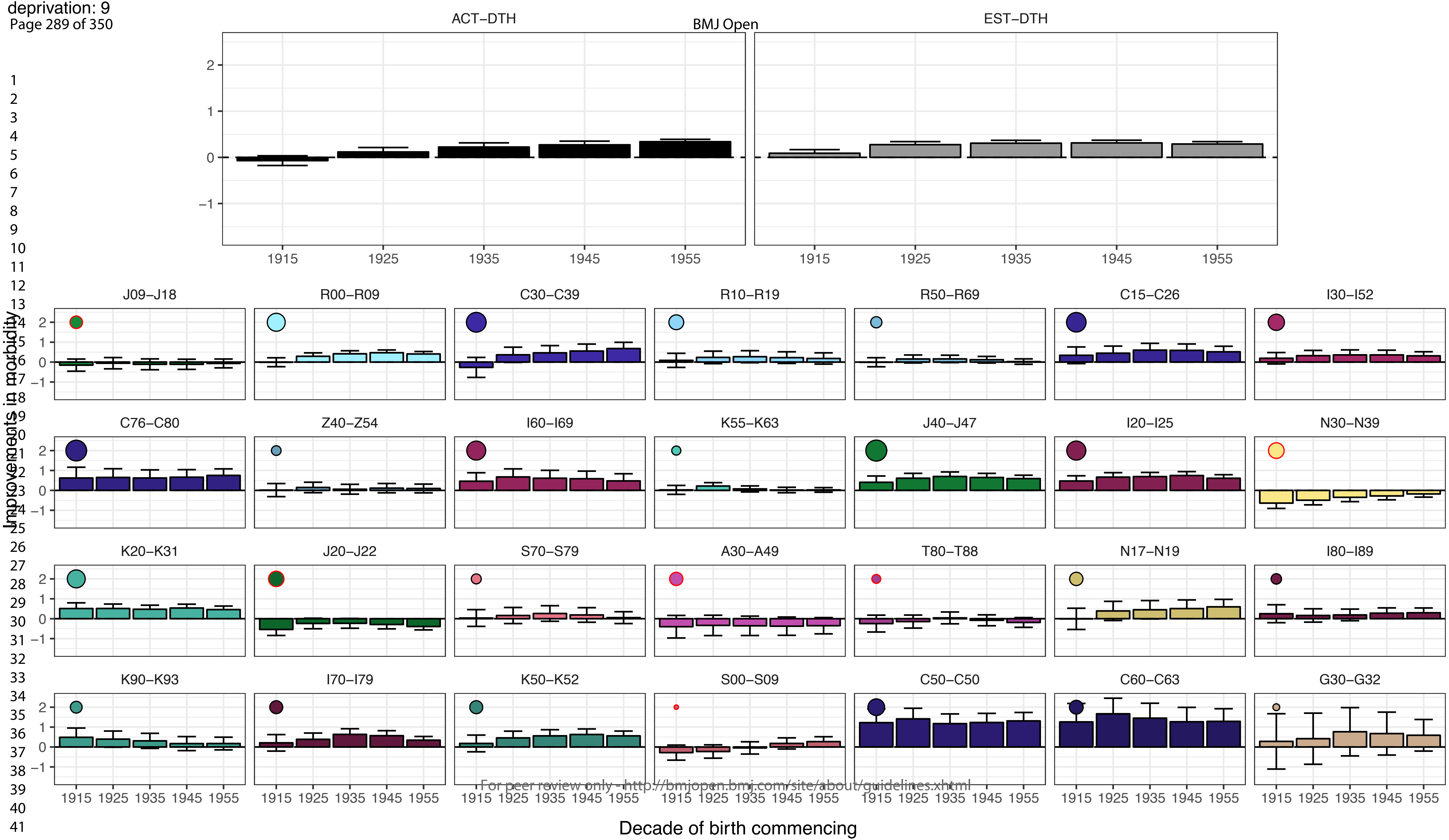

For peer review only - http://bmjopen.bmj.com/site/about/guidelines.xhtml

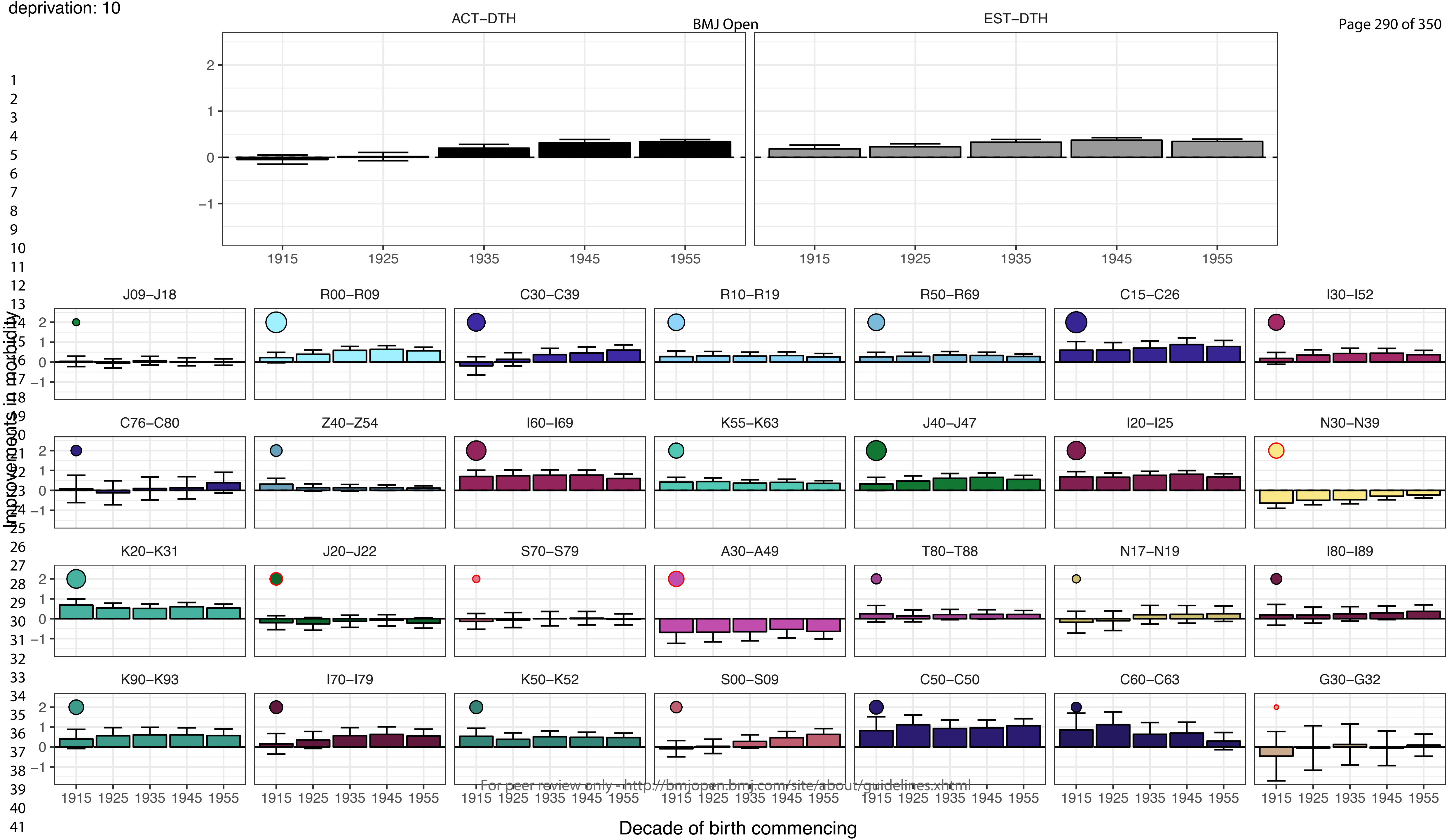

For peer review only - http://bmjopen.bmj.com/site/about/guidelines.xhtml

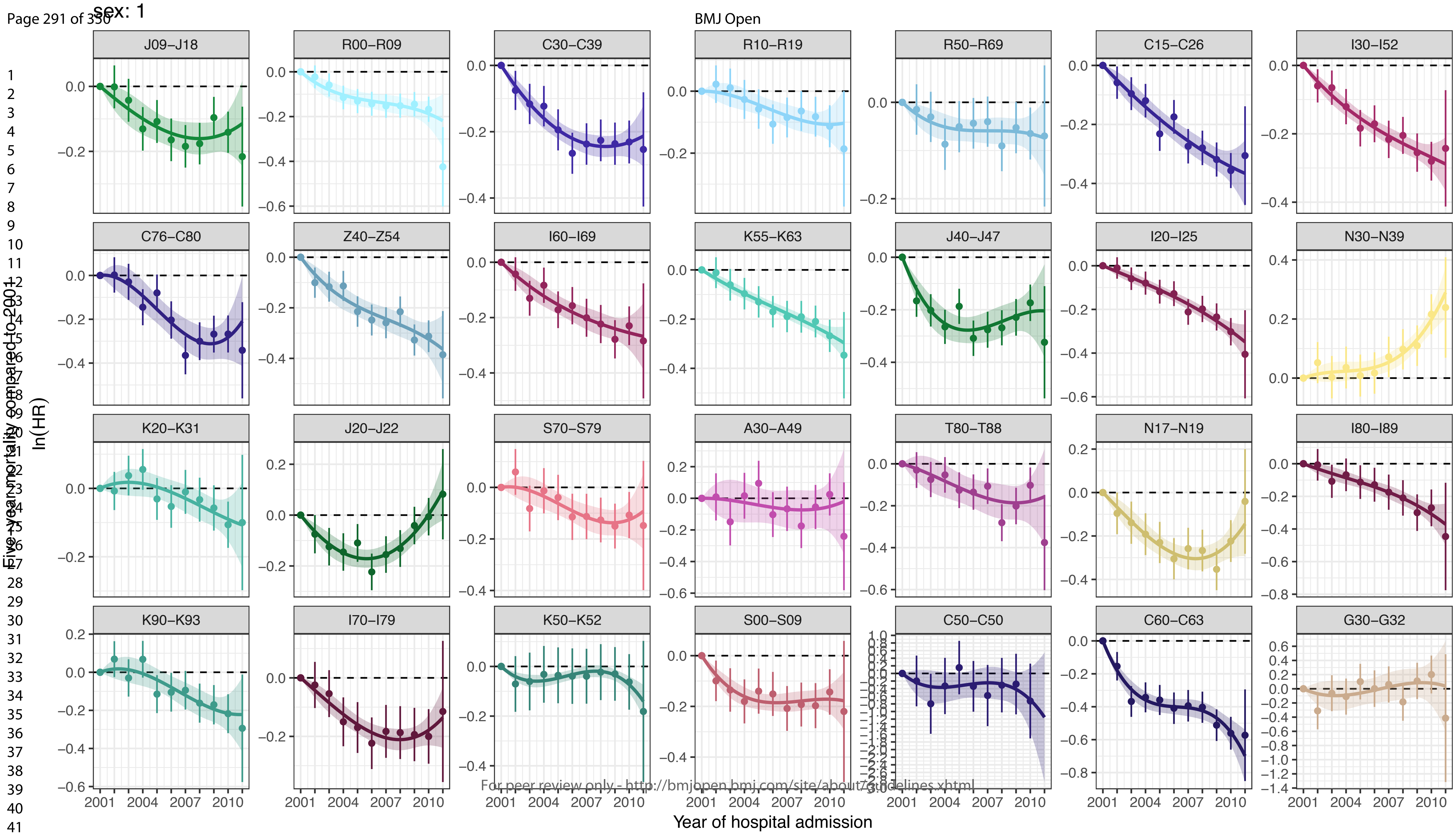

For peer review only - http://bmjopen.bmj.com/site/about/guidelines.xhtml

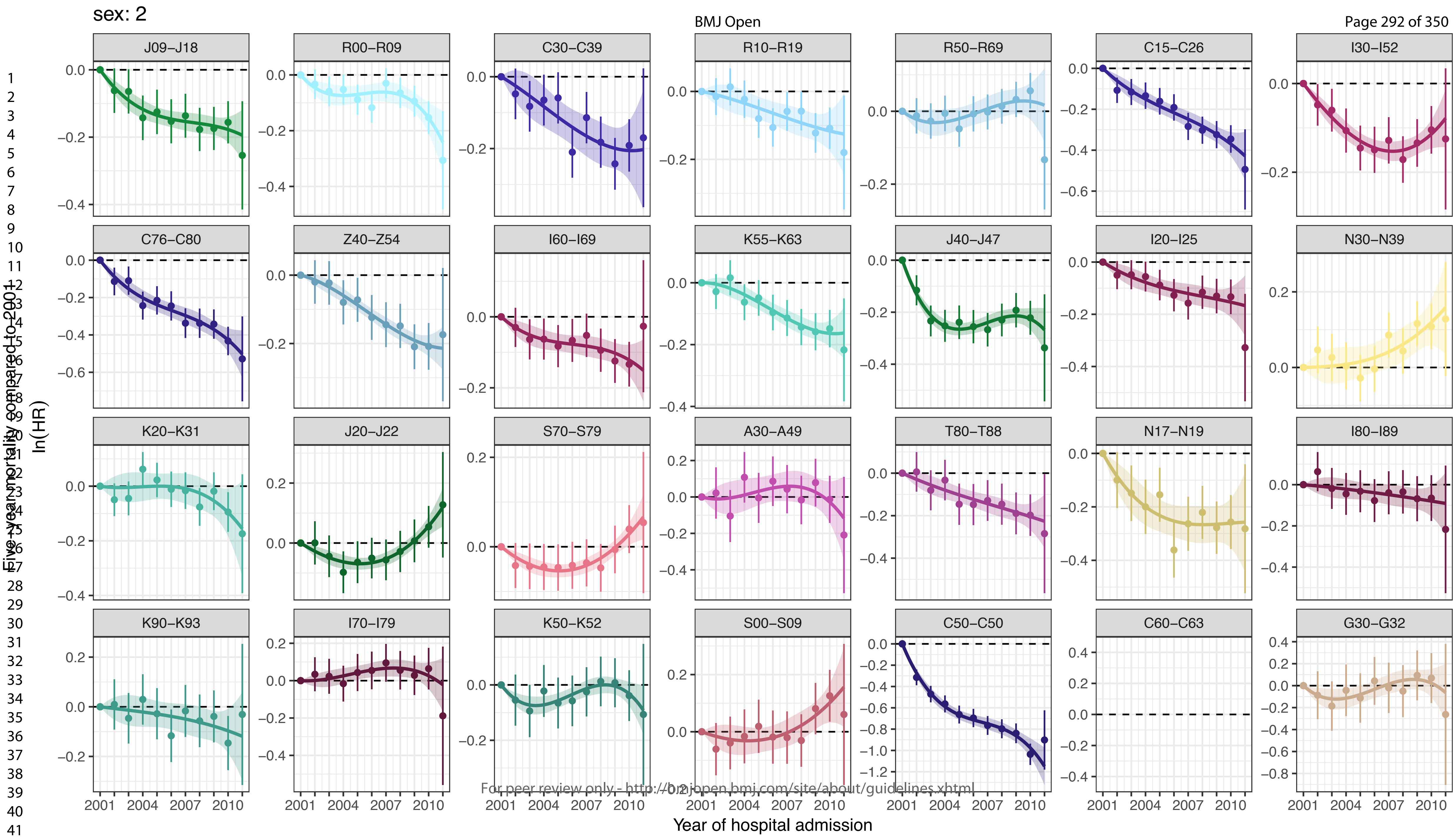

For peer review only - http://bmjopen.bmj.com/site/about/guidelines.xhtml

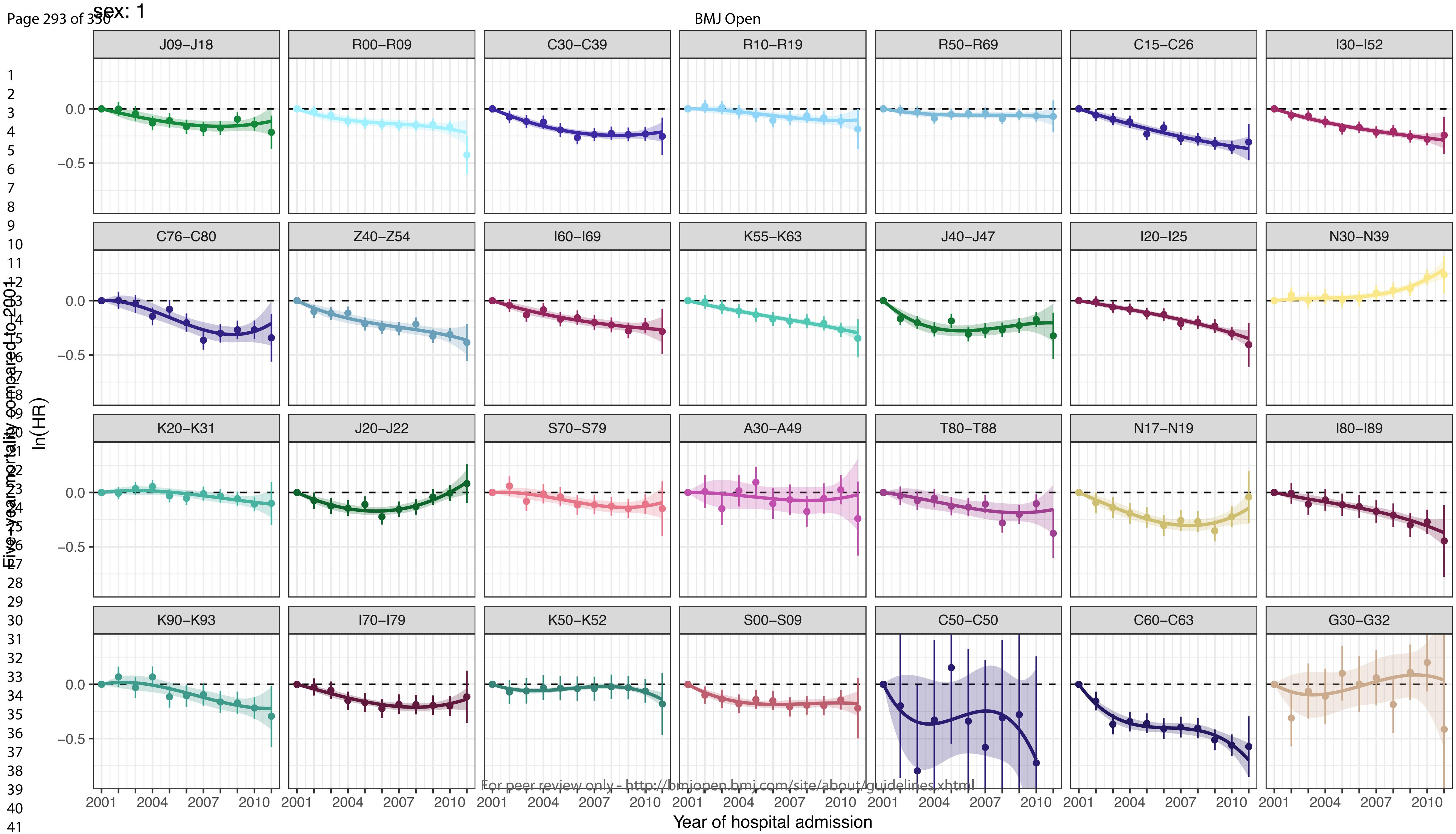
sex: 1
J09–J18
R00–R09
C30–C39
R10–R19
R50–R69
C15–C26
I30–I52
C76–C80
Z40–Z54
I60–I69
K55–K63
J40–J47
I20–I25
N30–N39
K20–K31
J20–J22
S70–S79
A30–A49
T80–T88
N17–N19
I80–I89
K90–K93
I70–I79
K50–K52
S00–S09
C50–C50
C60–C63
G30–G32
0.0
−0.5
2001
2004
2007
2010
Five-year mortality compared to 2001
ln(HR)
Year of hospital admission

sex: 2

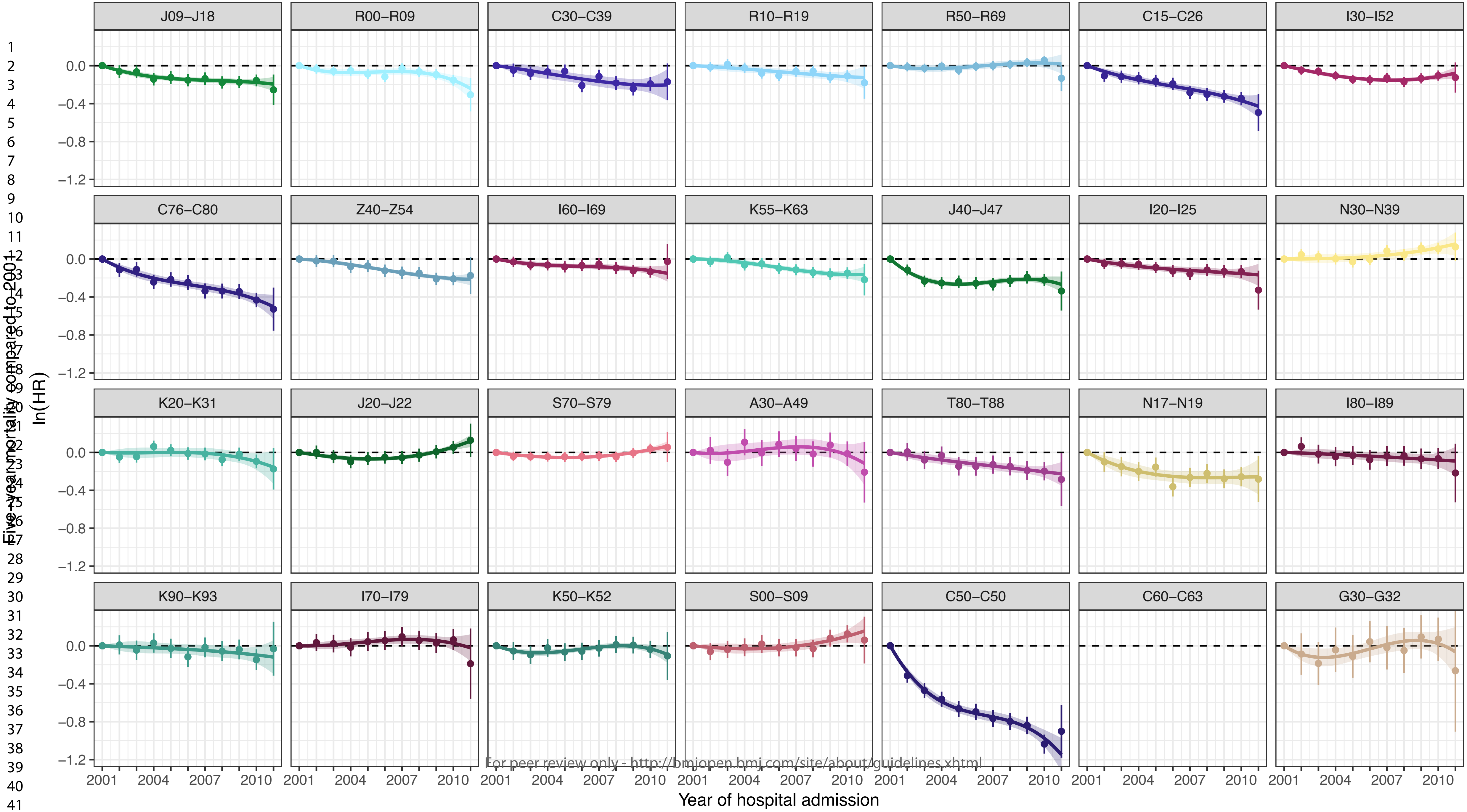

For peer review only - http://bmjopen.bmj.com/site/about/guidelines.xhtml

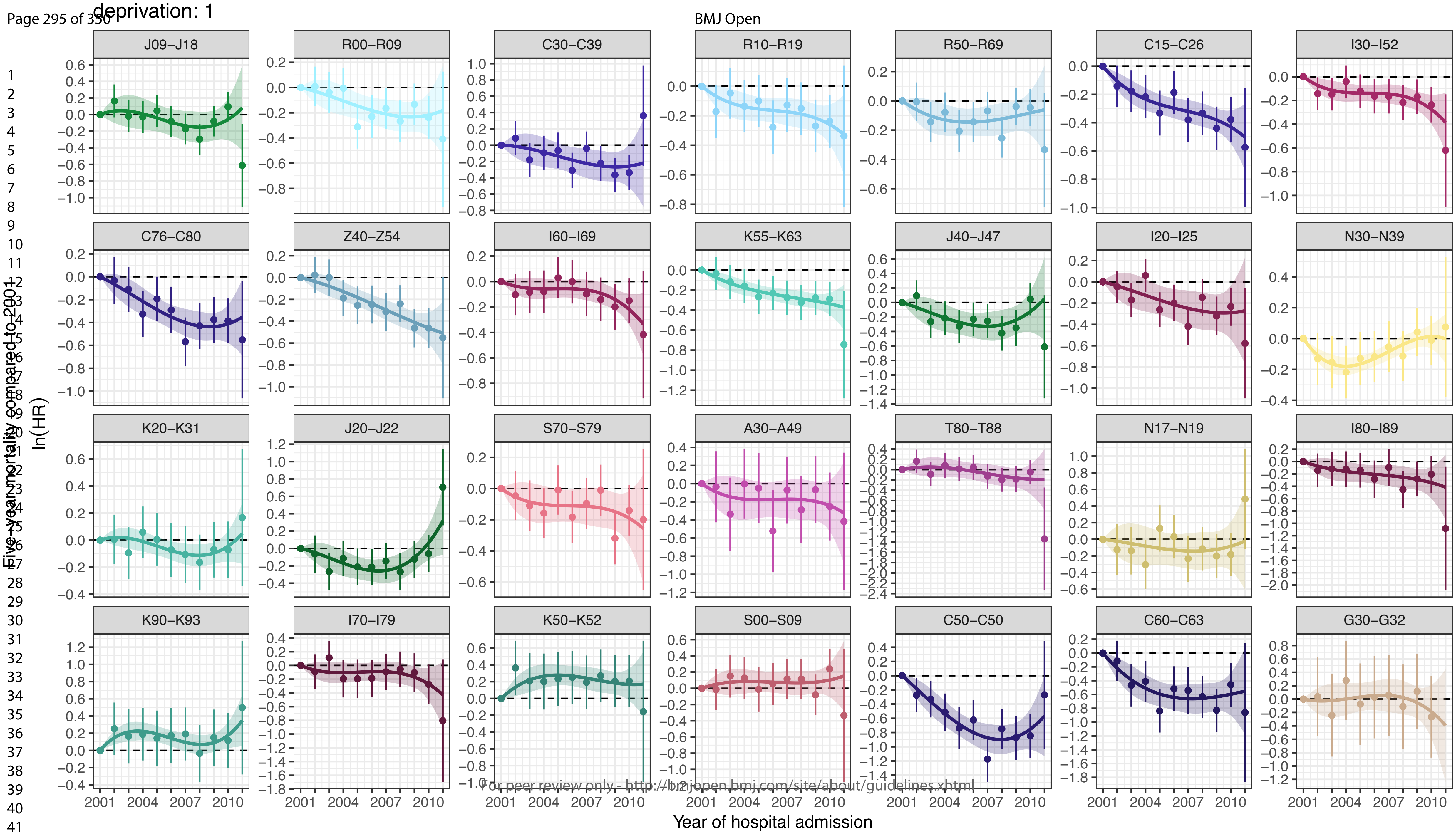
deprivation: 1
J09–J18
R00–R09
C30–C39
R10–R19
R50–R69
C15–C26
I30–I52
C76–C80
Z40–Z54
I60–I69
K55–K63
J40–J47
I20–I25
N30–N39
K20–K31
J20–J22
S70–S79
A30–A49
T80–T88
N17–N19
I80–I89
K90–K93
I70–I79
K50–K52
S00–S09
C50–C50
C60–C63
G30–G32
Five-year mortality compared to 2001
ln(HR)
Year of hospital admission

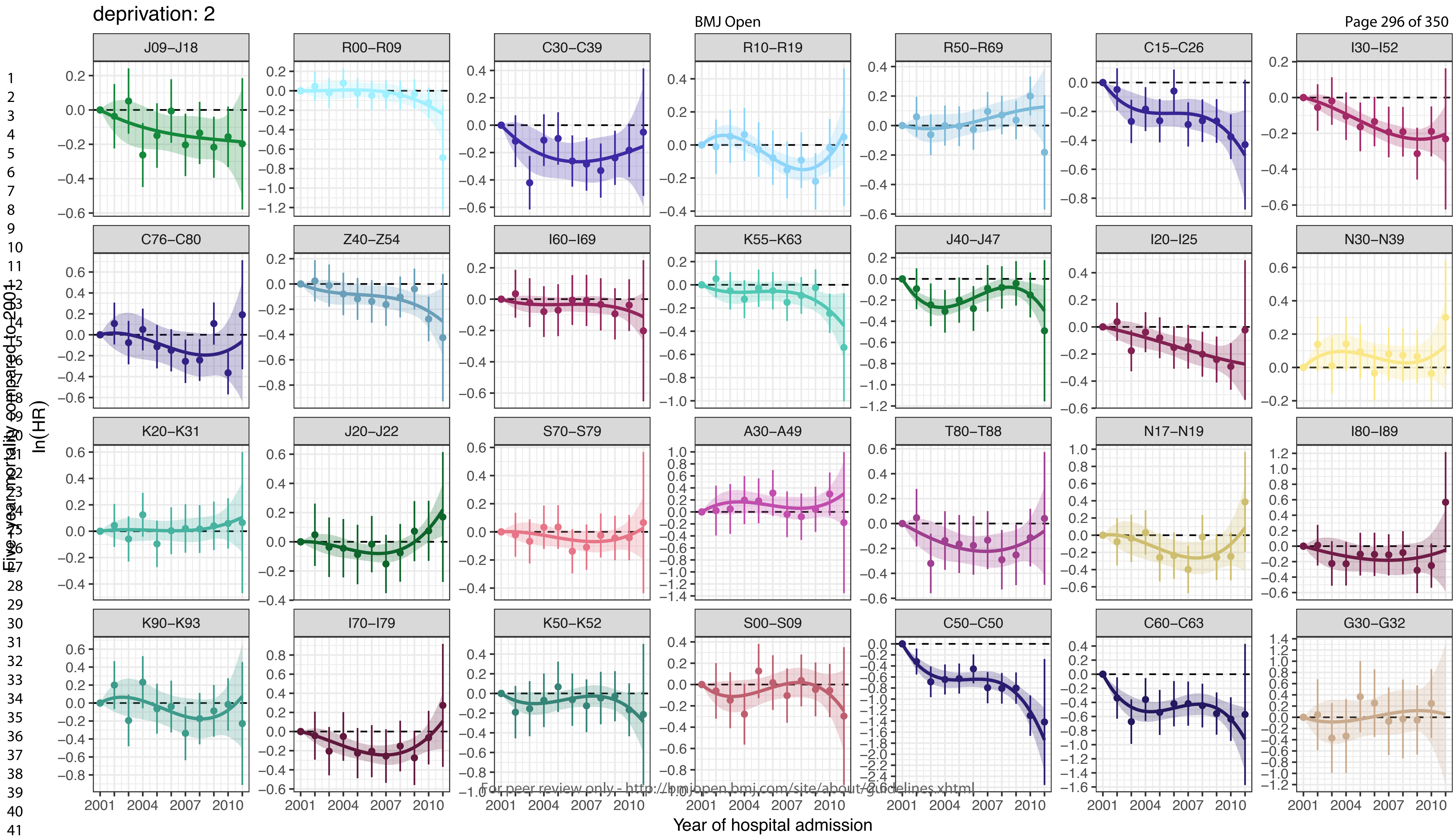
deprivation: 2
J09–J18
R00–R09
C30–C39
R10–R19
R50–R69
C15–C26
I30–I52
C76–C80
Z40–Z54
I60–I69
K55–K63
J40–J47
I20–I25
N30–N39
K20–K31
J20–J22
S70–S79
A30–A49
T80–T88
N17–N19
I80–I89
K90–K93
I70–I79
K50–K52
S00–S09
C50–C50
C60–C63
G30–G32
Five-year mortality compared to 2001
ln(HR)
Year of hospital admission

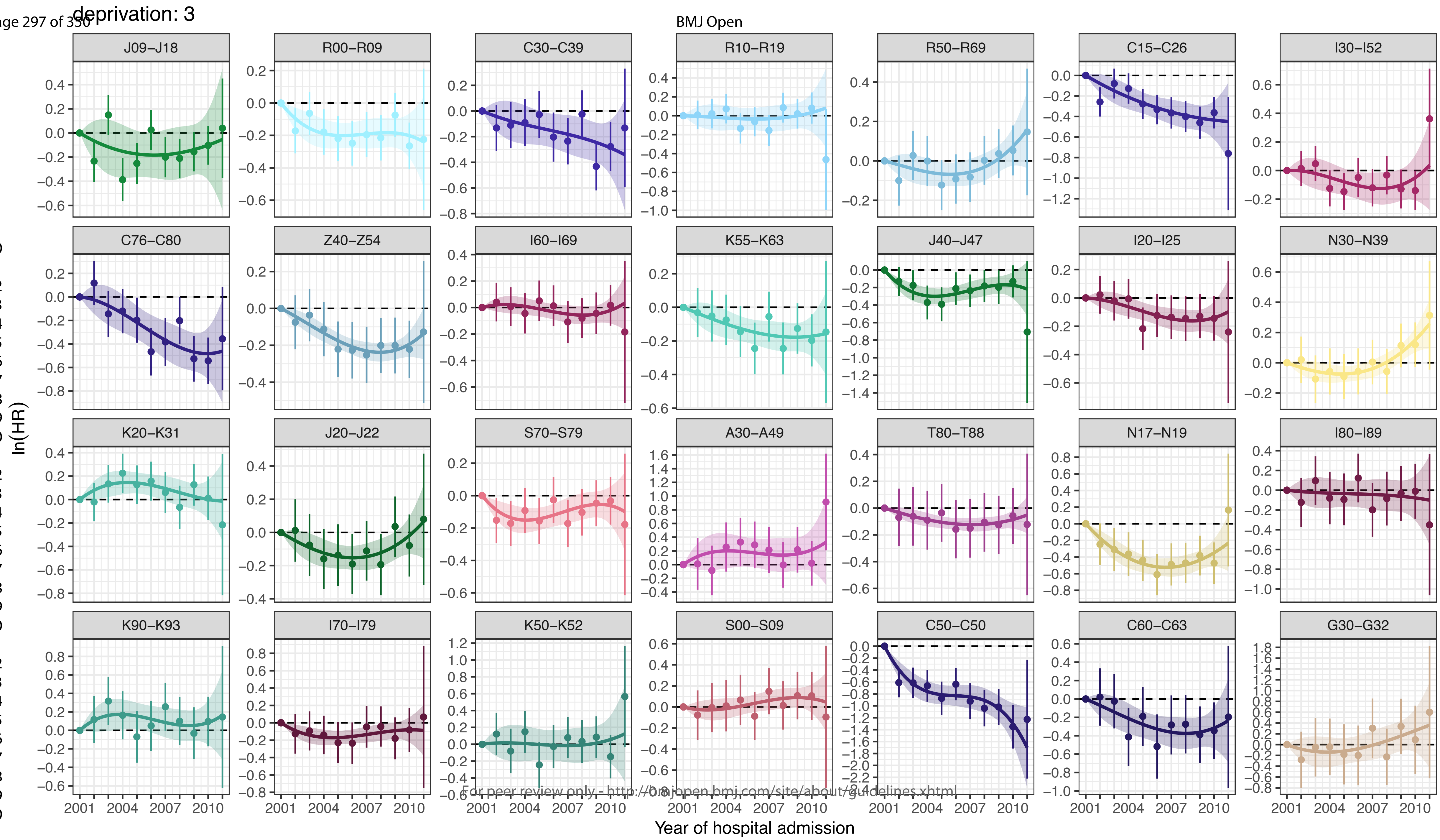

For peer review only - http://bmjopen.bmj.com/site/about/guidelines.xhtml

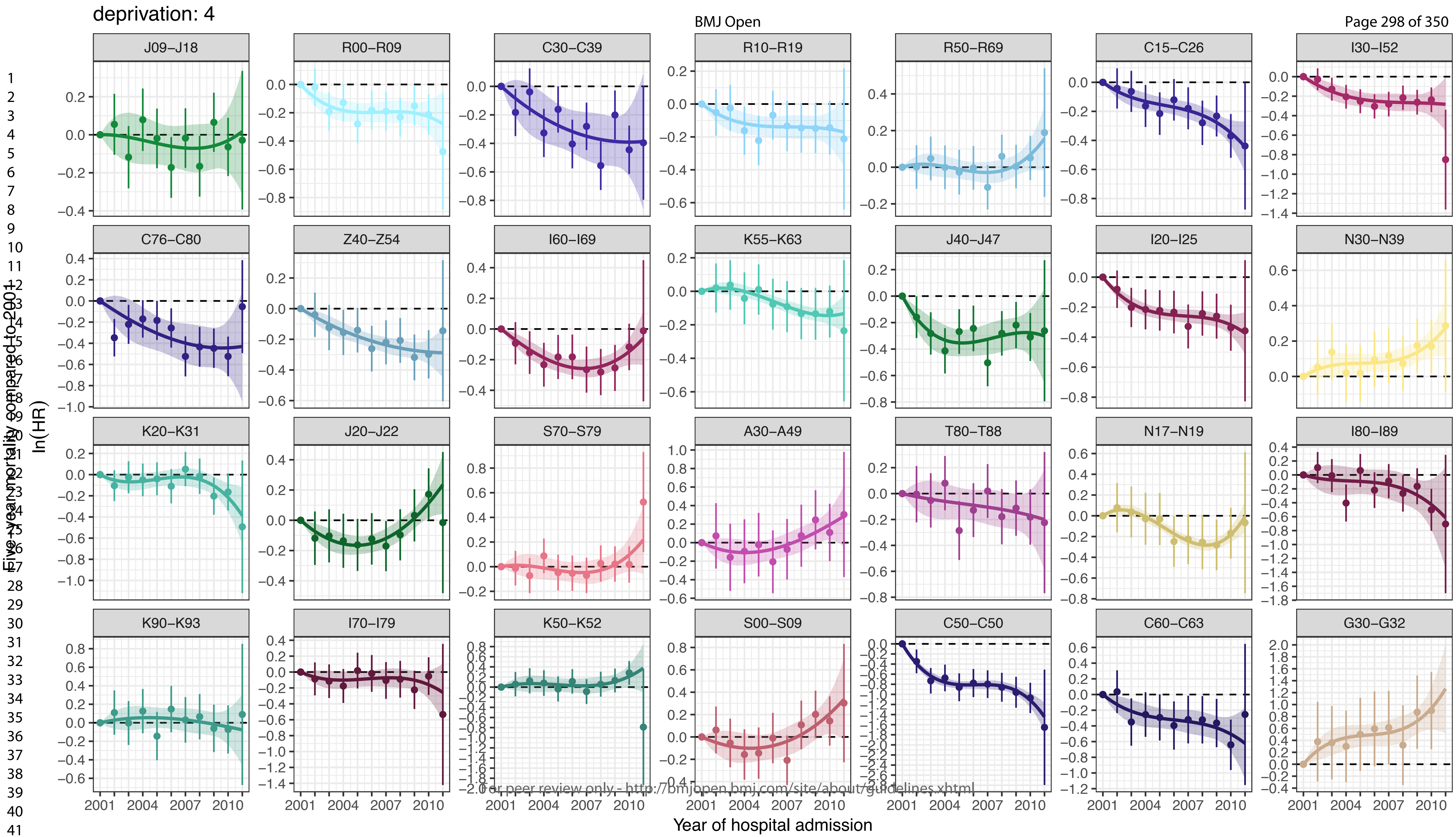
deprivation: 4
J09–J18
R00–R09
C30–C39
R10–R19
R50–R69
C15–C26
I30–I52
C76–C80
Z40–Z54
I60–I69
K55–K63
J40–J47
I20–I25
N30–N39
K20–K31
J20–J22
S70–S79
A30–A49
T80–T88
N17–N19
I80–I89
K90–K93
I70–I79
K50–K52
S00–S09
C50–C50
C60–C63
G30–G32
Five-year mortality compared to 2001
ln(HR)
Year of hospital admission

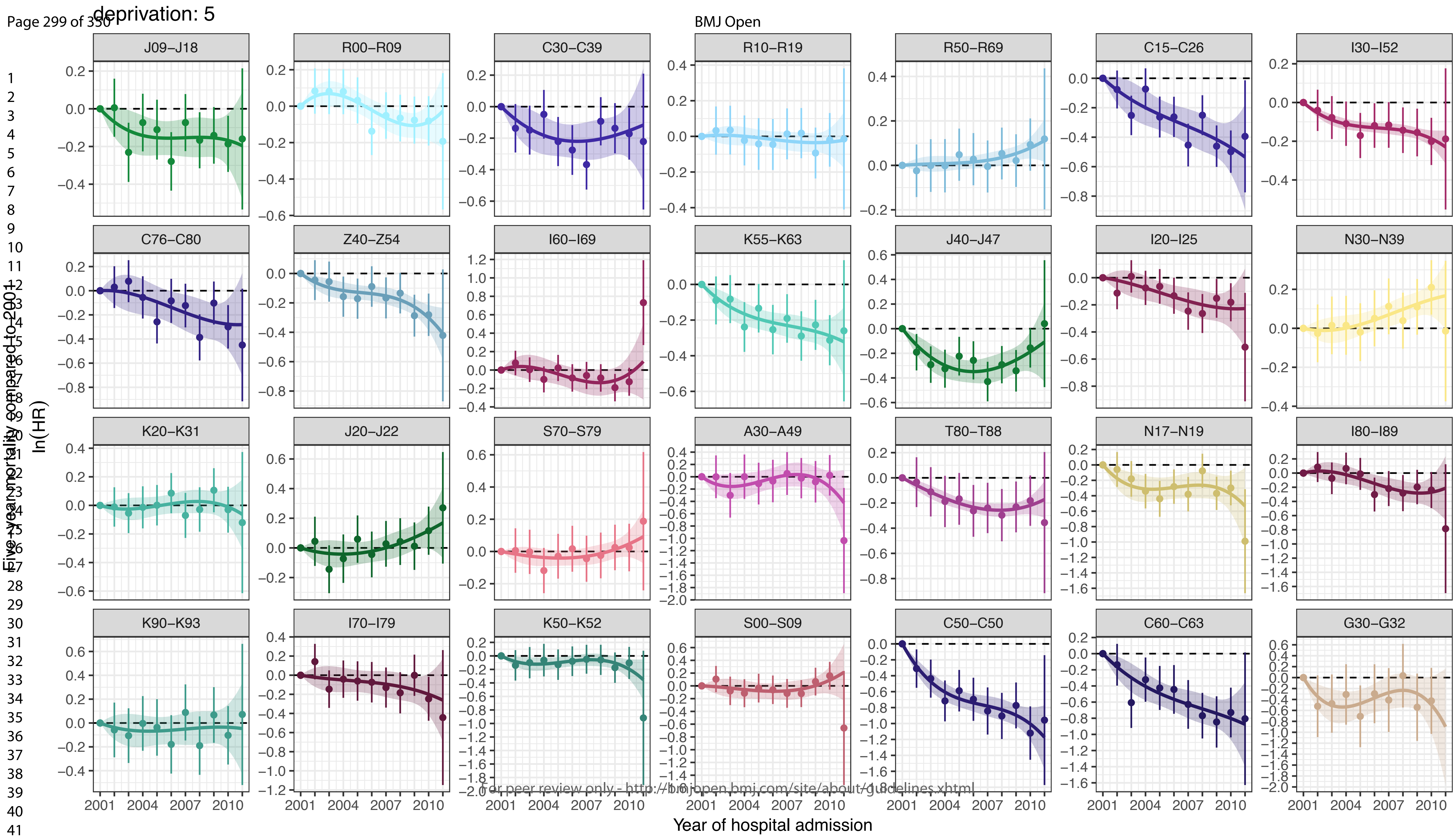

deprivation: 5
J09–J18
R00–R09
C30–C39
R10–R19
R50–R69
C15–C26
I30–I52
C76–C80
Z40–Z54
I60–I69
K55–K63
J40–J47
I20–I25
N30–N39
K20–K31
J20–J22
S70–S79
A30–A49
T80–T88
N17–N19
I80–I89
K90–K93
I70–I79
K50–K52
S00–S09
C50–C50
C60–C63
G30–G32
Five-year mortality compared to 2001
ln(HR)
Year of hospital admission
2001
2004
2007
2010

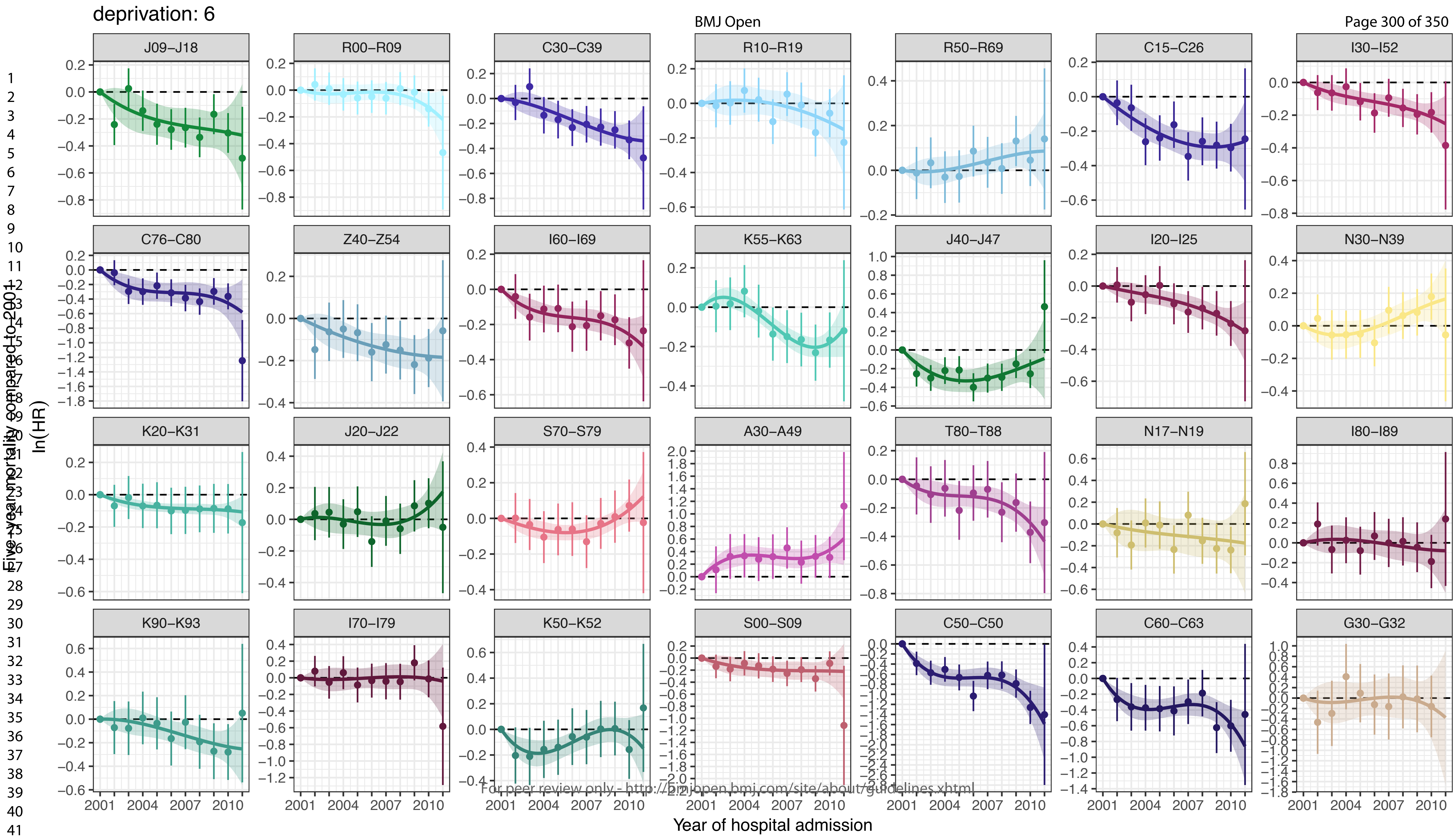
deprivation: 6
J09–J18
R00–R09
C30–C39
R10–R19
R50–R69
C15–C26
I30–I52
C76–C80
Z40–Z54
I60–I69
K55–K63
J40–J47
I20–I25
N30–N39
K20–K31
J20–J22
S70–S79
A30–A49
T80–T88
N17–N19
I80–I89
K90–K93
I70–I79
K50–K52
S00–S09
C50–C50
C60–C63
G30–G32
Five-year mortality compared to 2001
ln(HR)
2001
2004
2007
2010
Year of hospital admission

deprivation: 7

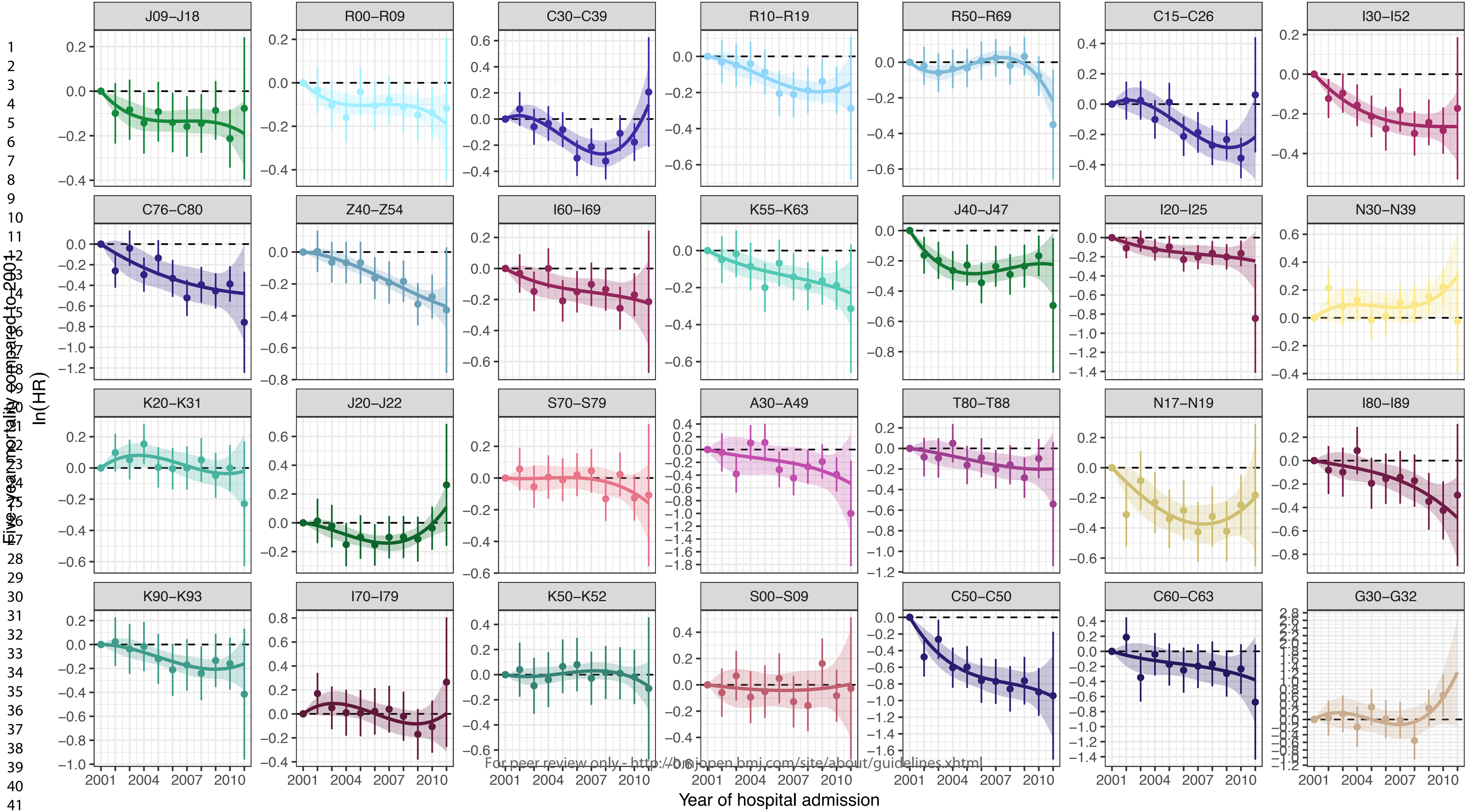

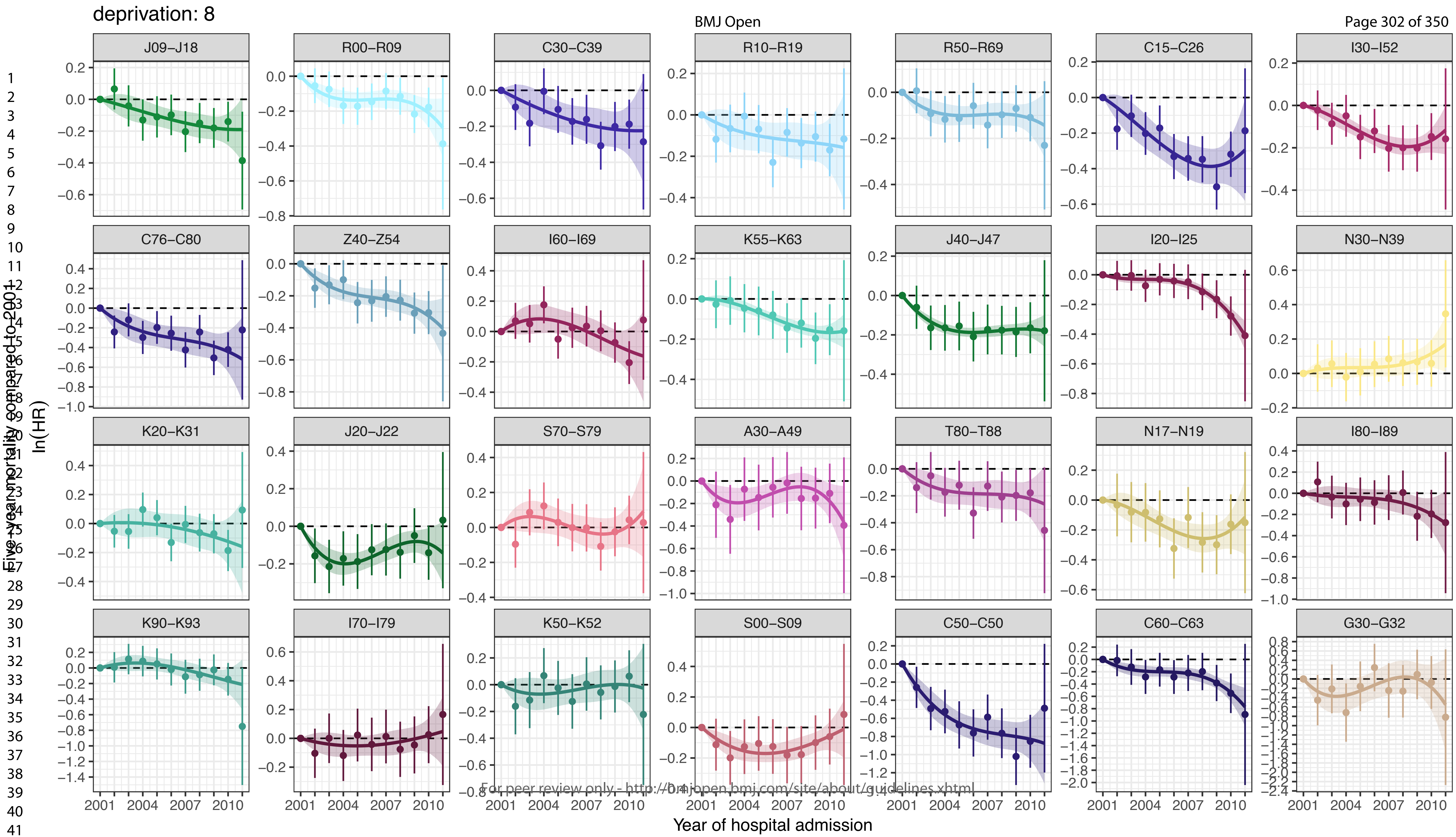

For peer review only - http://bmjopen.bmj.com/site/about/guidelines.xhtml

deprivation: 9

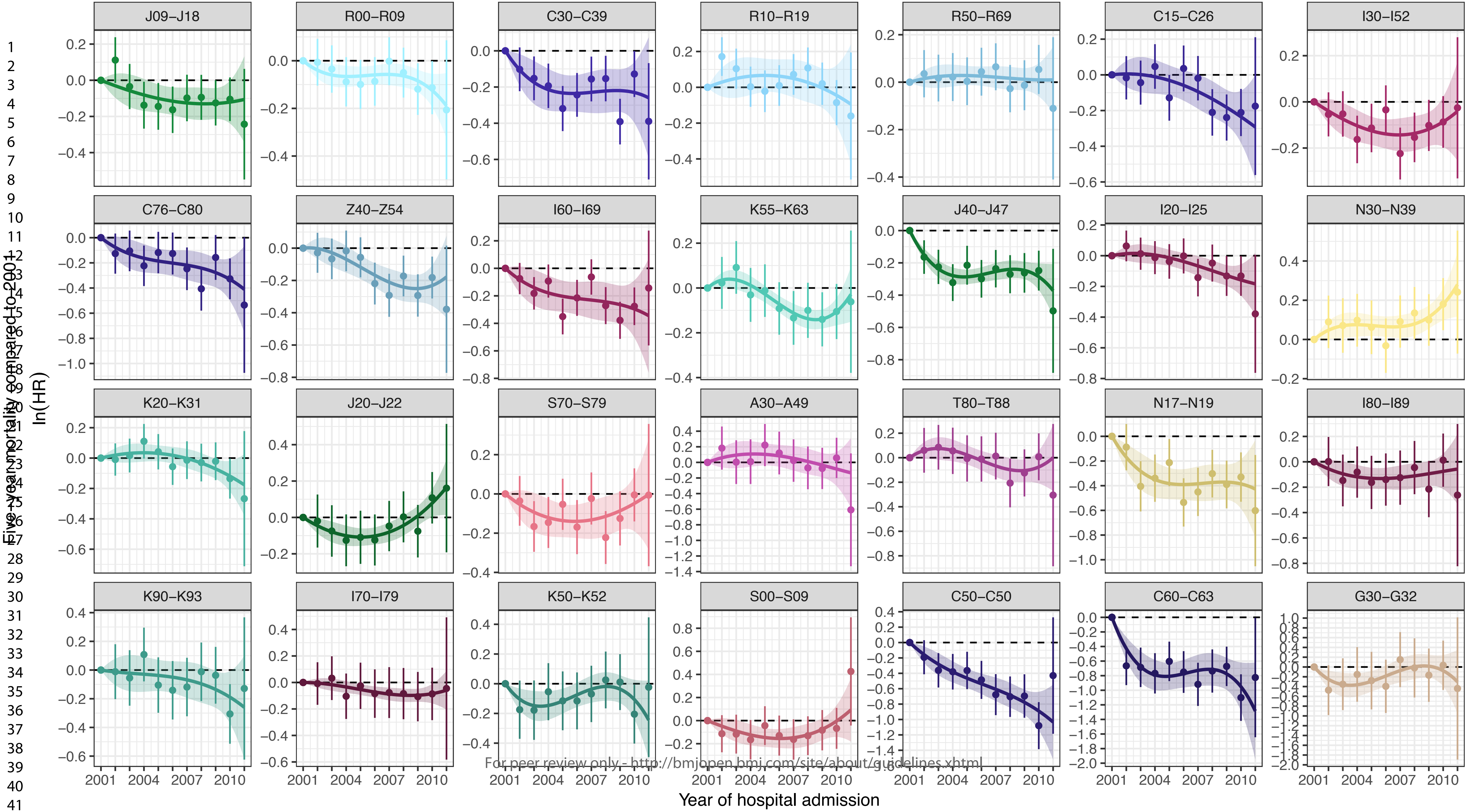

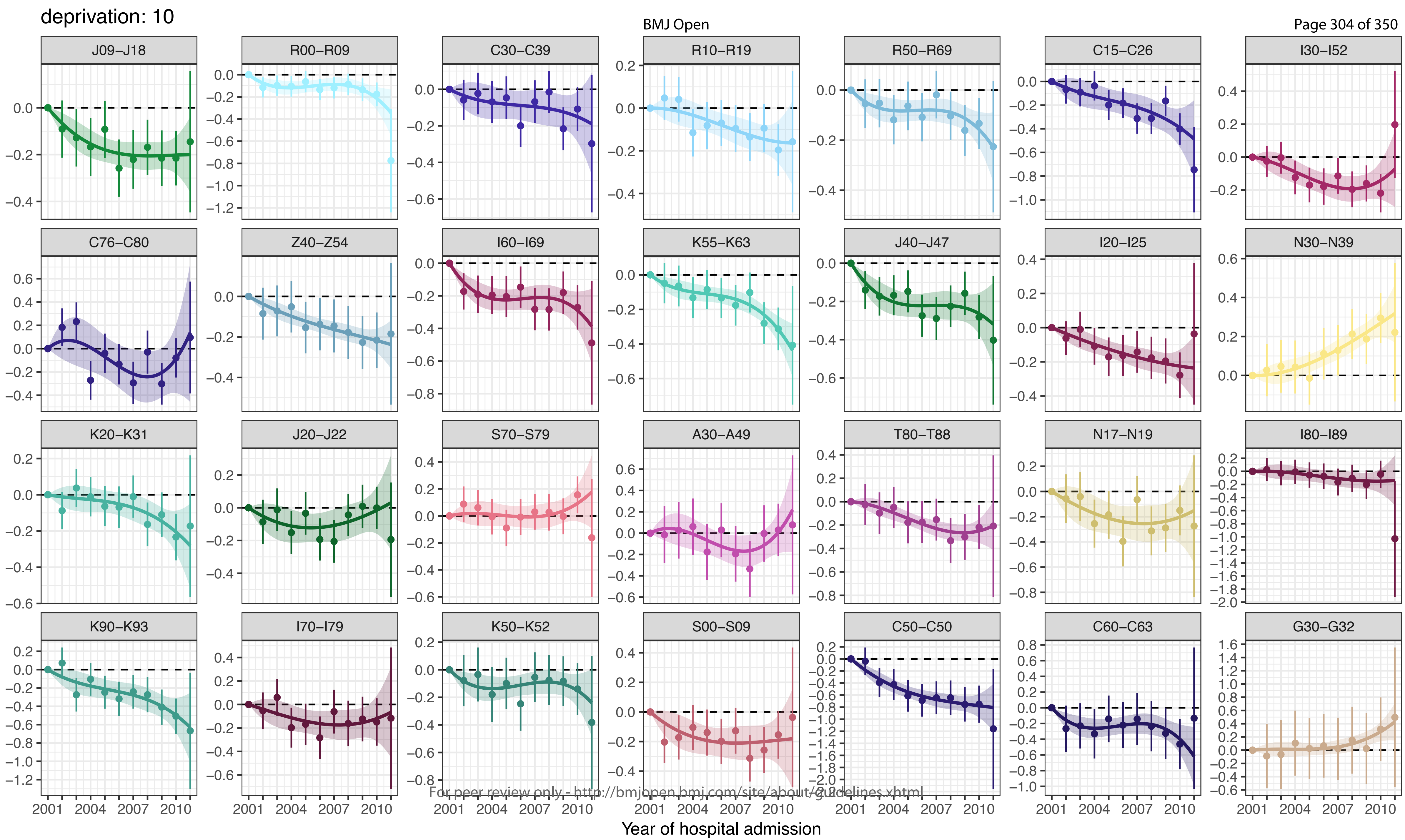
deprivation: 10
J09–J18
R00–R09
C30–C39
R10–R19
R50–R69
C15–C26
I30–I52
C76–C80
Z40–Z54
I60–I69
K55–K63
J40–J47
I20–I25
N30–N39
K20–K31
J20–J22
S70–S79
A30–A49
T80–T88
N17–N19
I80–I89
K90–K93
I70–I79
K50–K52
S00–S09
C50–C50
C60–C63
G30–G32
Five-year mortality compared to 2001
ln(HR)
Year of hospital admission

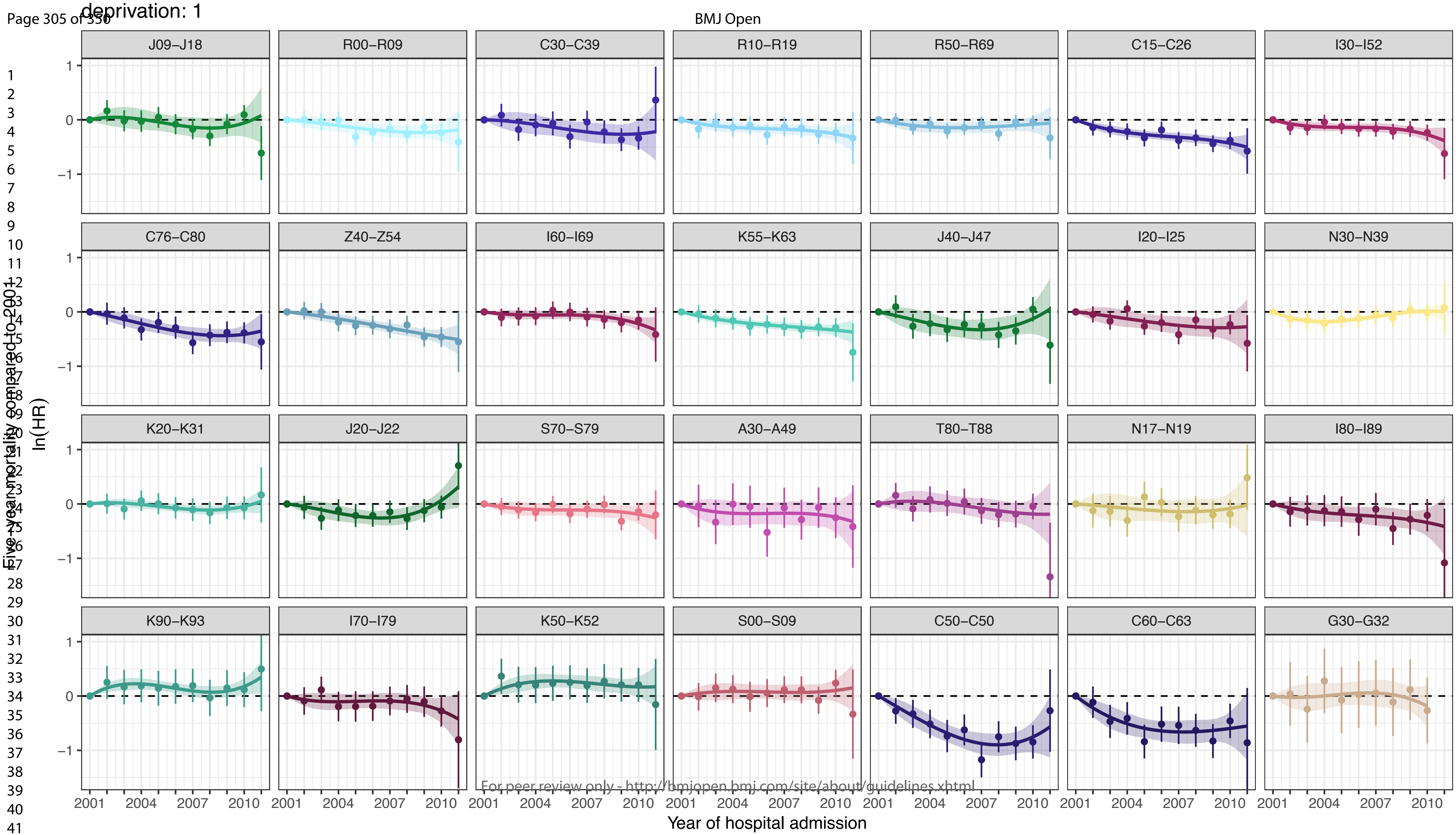

For peer review only - http://bmjopen.bmj.com/site/about/guidelines.xhtml

deprivation: 2

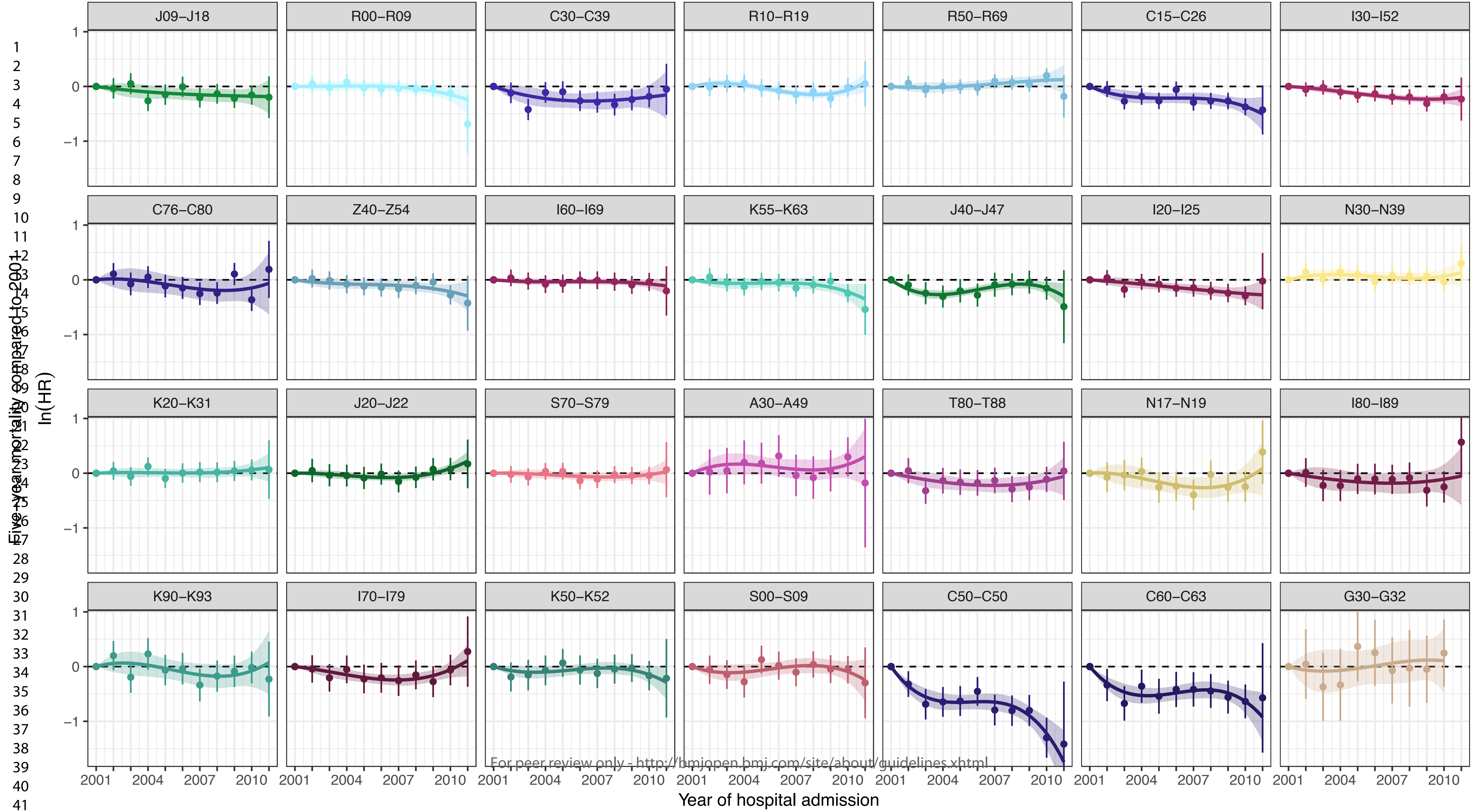

For peer review only - http://bmjopen.bmj.com/site/about/guidelines.xhtml

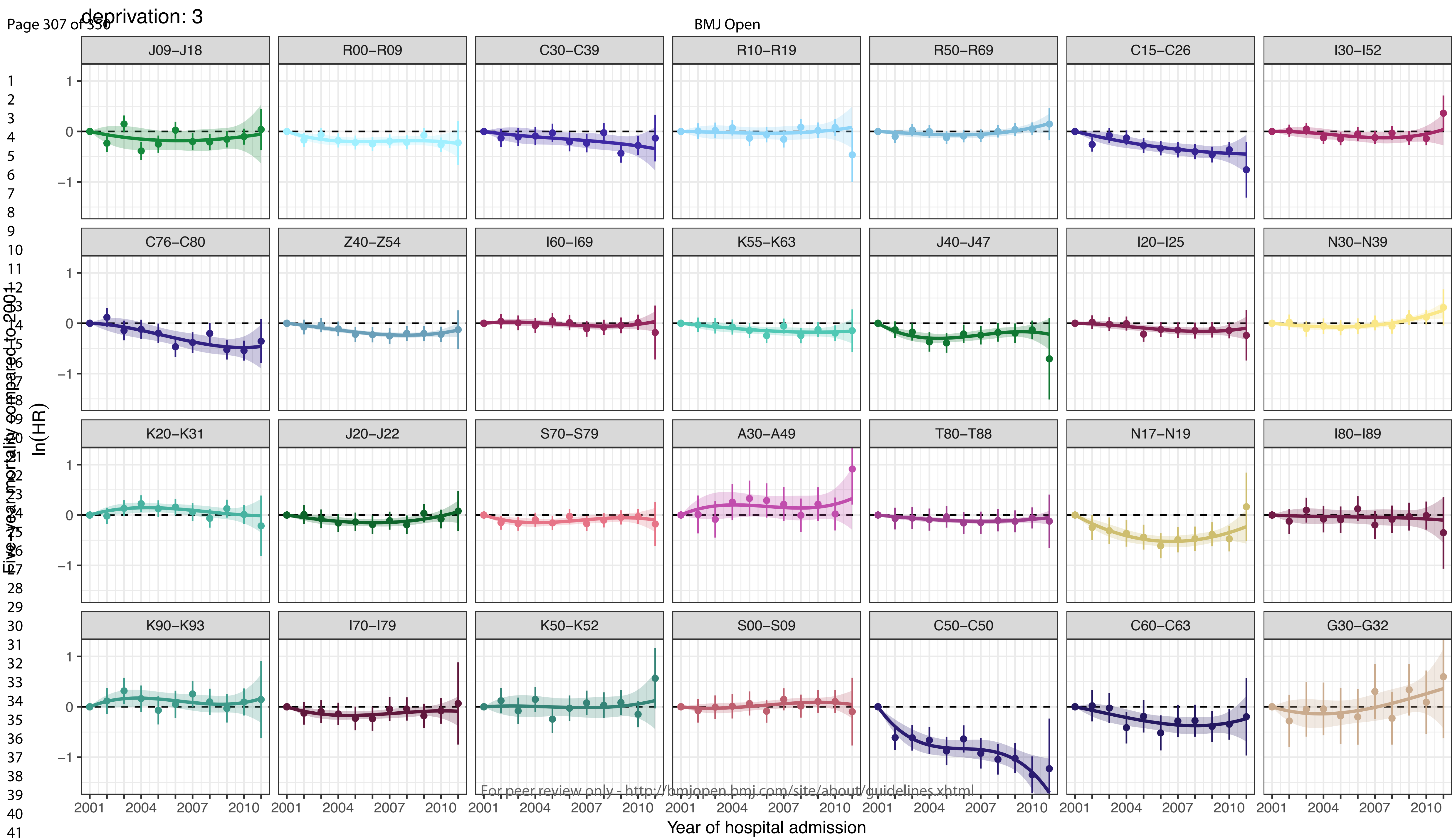
deprivation: 3
J09–J18
R00–R09
C30–C39
R10–R19
R50–R69
C15–C26
I30–I52
C76–C80
Z40–Z54
I60–I69
K55–K63
J40–J47
I20–I25
N30–N39
K20–K31
J20–J22
S70–S79
A30–A49
T80–T88
N17–N19
I80–I89
K90–K93
I70–I79
K50–K52
S00–S09
C50–C50
C60–C63
G30–G32
1
0
−1
2001
2004
2007
2010
Five-year mortality compared to 2001
ln(HR)
Year of hospital admission

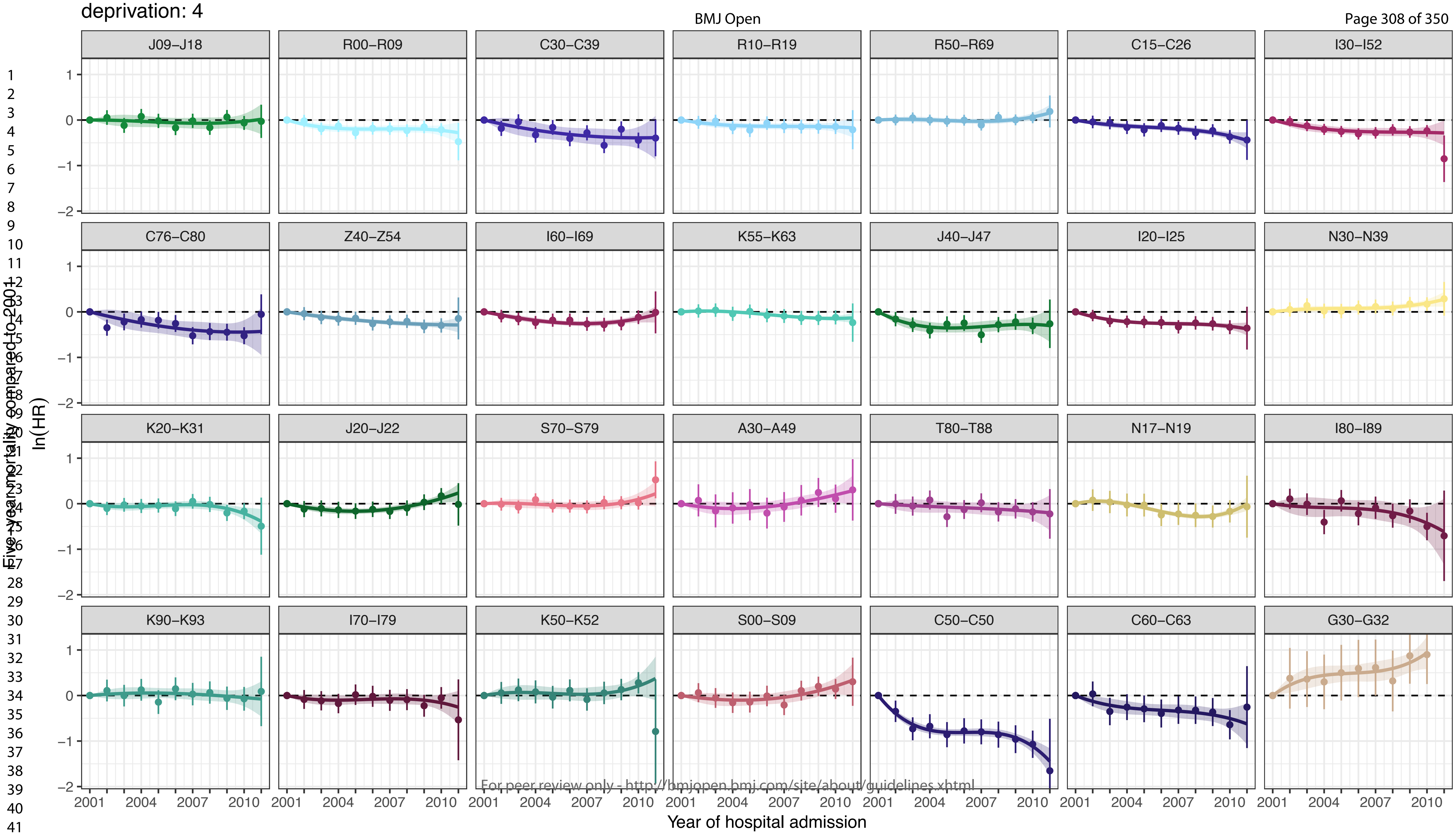
deprivation: 4
J09–J18
R00–R09
C30–C39
R10–R19
R50–R69
C15–C26
I30–I52
C76–C80
Z40–Z54
I60–I69
K55–K63
J40–J47
I20–I25
N30–N39
K20–K31
J20–J22
S70–S79
A30–A49
T80–T88
N17–N19
I80–I89
K90–K93
I70–I79
K50–K52
S00–S09
C50–C50
C60–C63
G30–G32
Five-year mortality compared to 2001
ln(HR)
Year of hospital admission
2001
2004
2007
2010
1
0
−1
−2

deprivation: 5

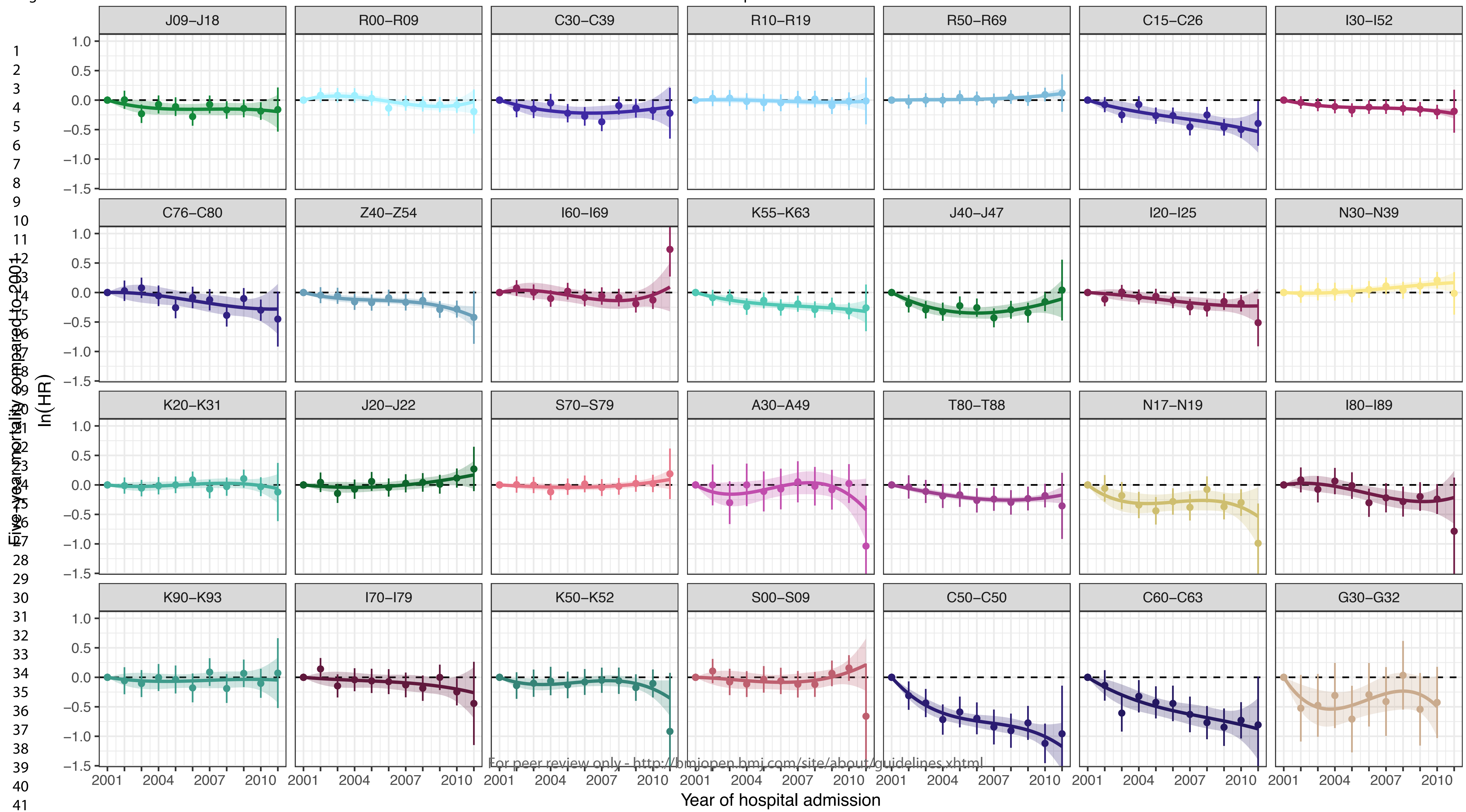

For peer review only - http://bmjopen.bmj.com/site/about/guidelines.xhtml

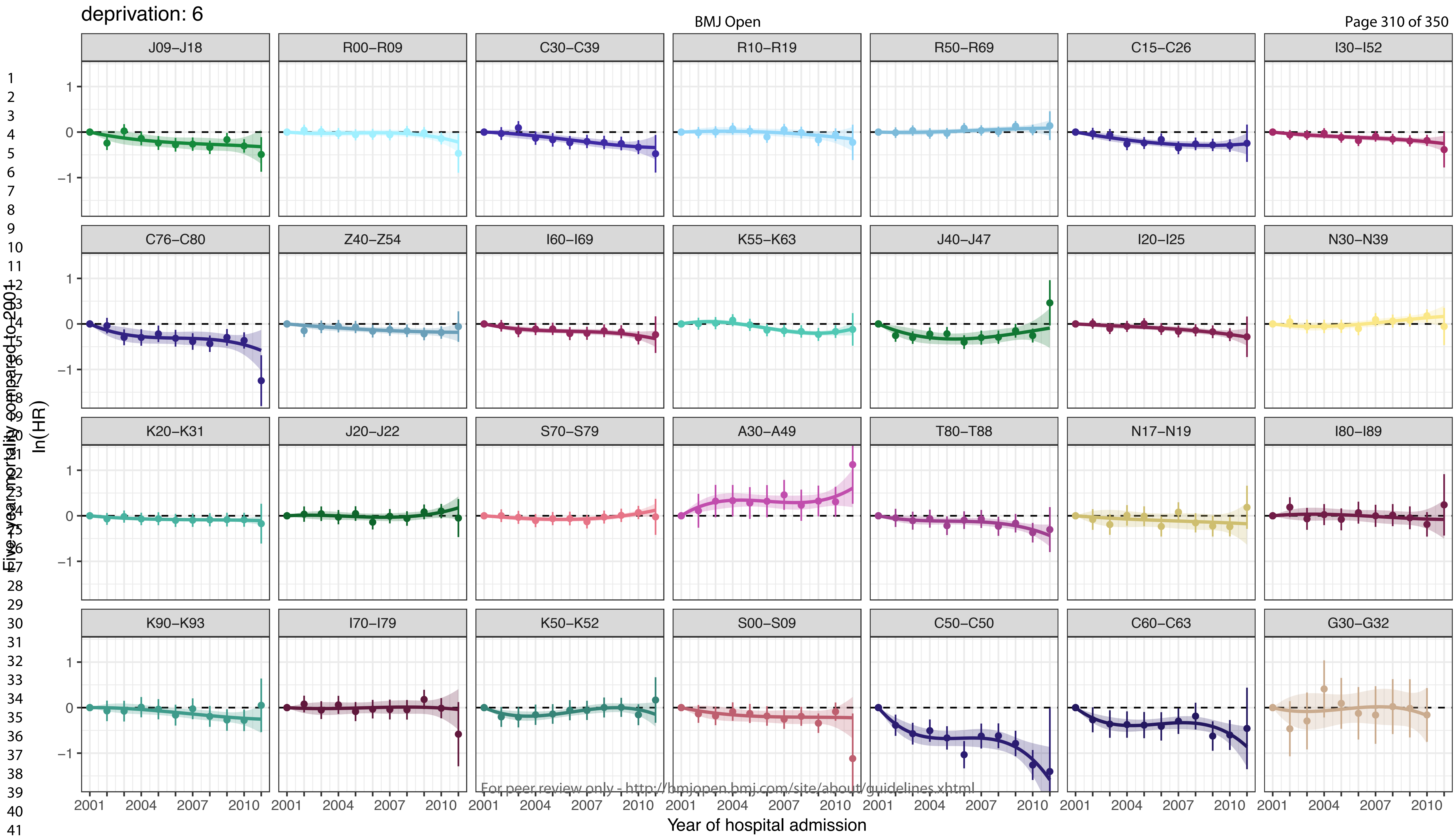

For peer review only - http://bmjopen.bmj.com/site/about/guidelines.xhtml

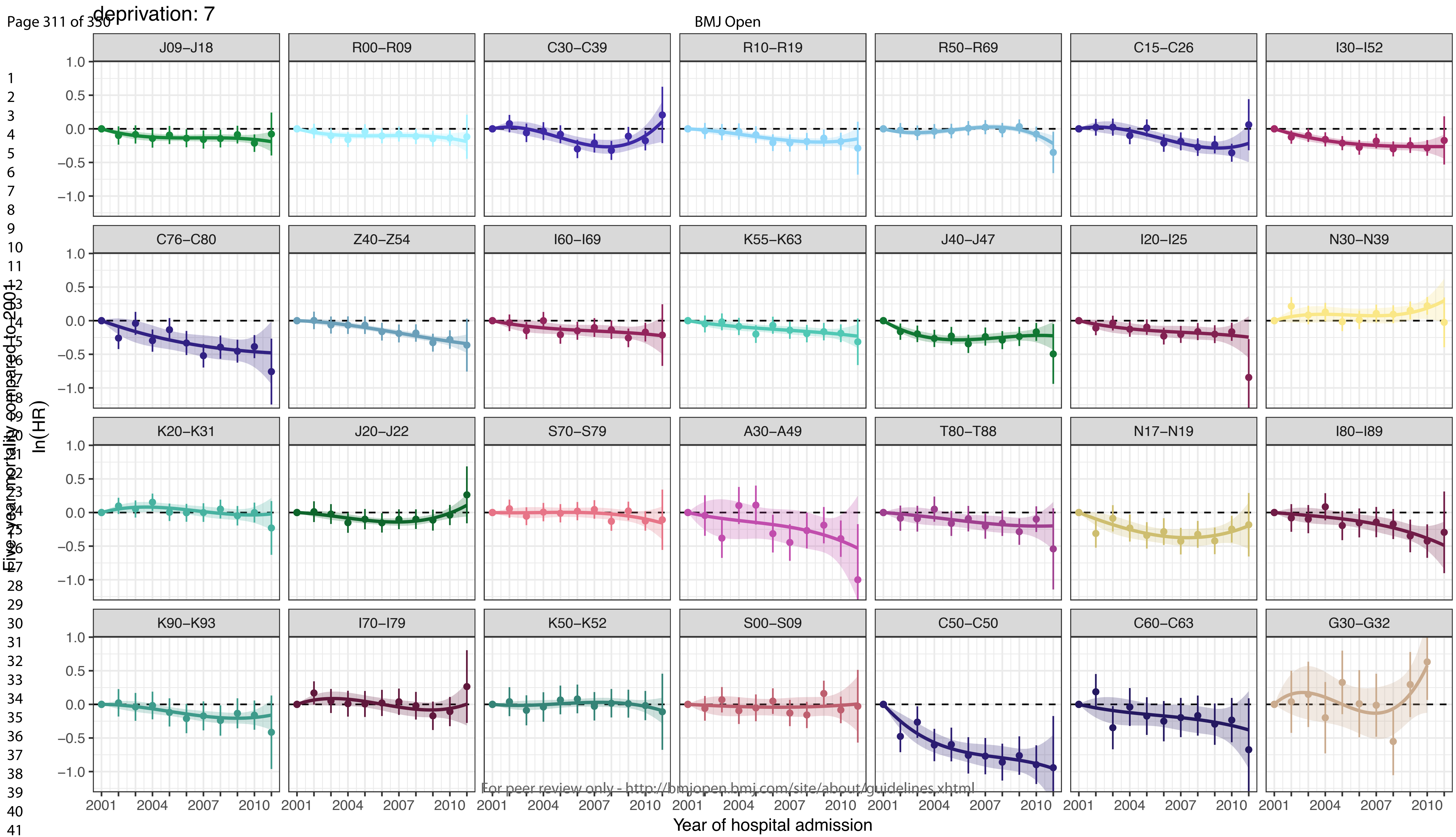

For peer review only - http://bmjopen.bmj.com/site/about/guidelines.xhtml

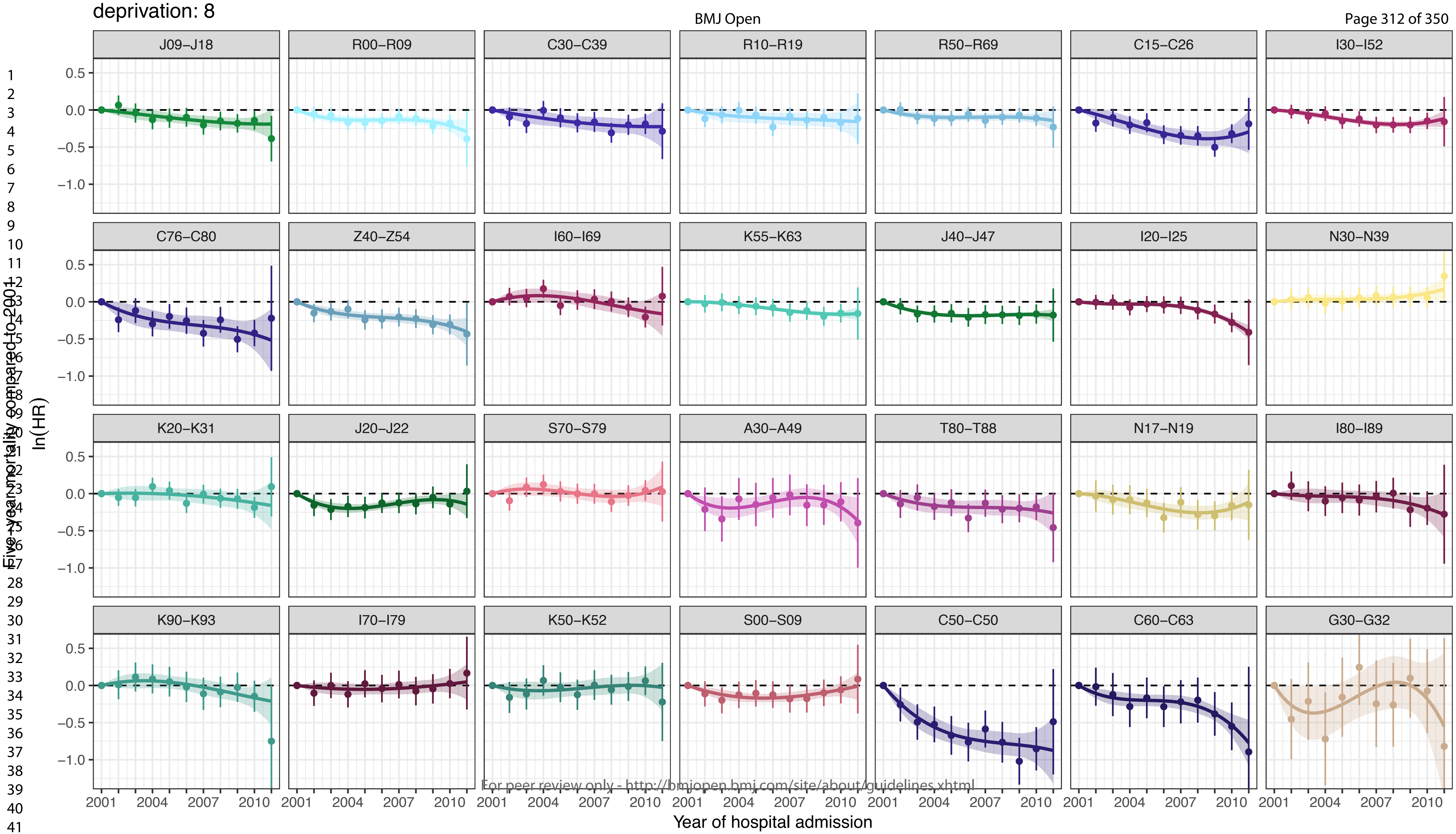

For peer review only - http://bmjopen.bmj.com/site/about/guidelines.xhtml

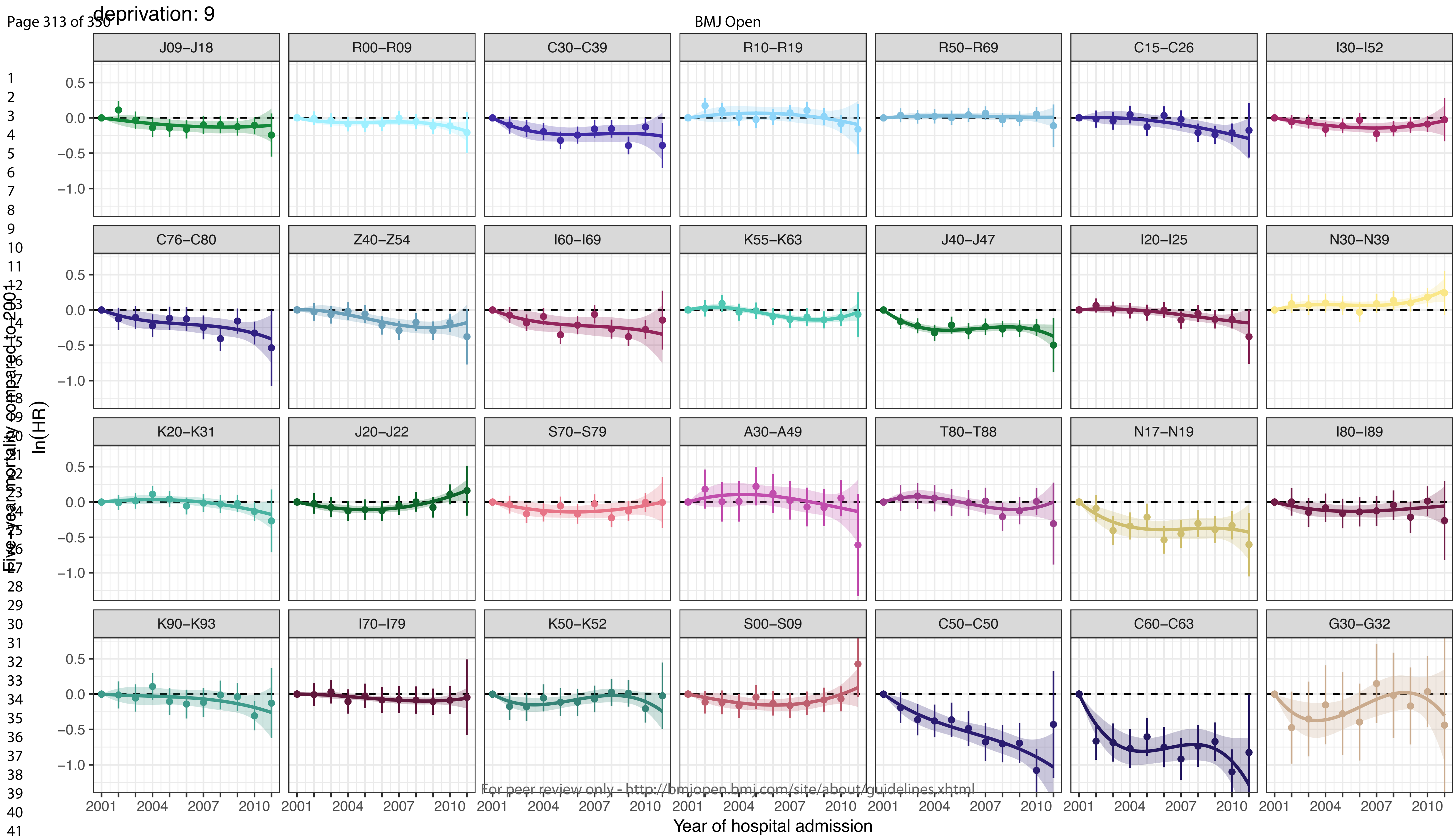

For peer review only - http://bmjopen.bmj.com/site/about/guidelines.xhtml

deprivation: 10

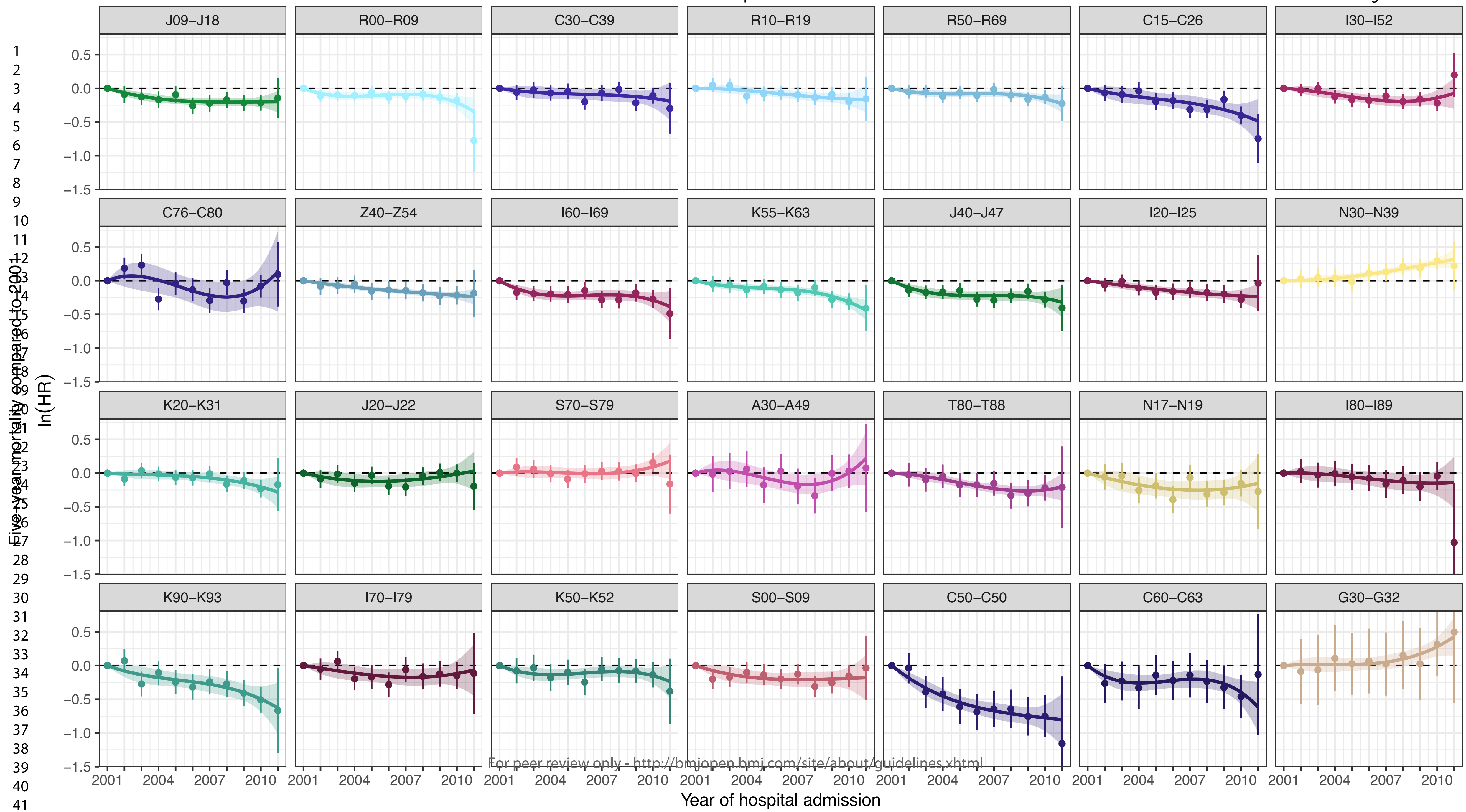

For peer review only - http://bmjopen.bmj.com/site/about/guidelines.xhtml

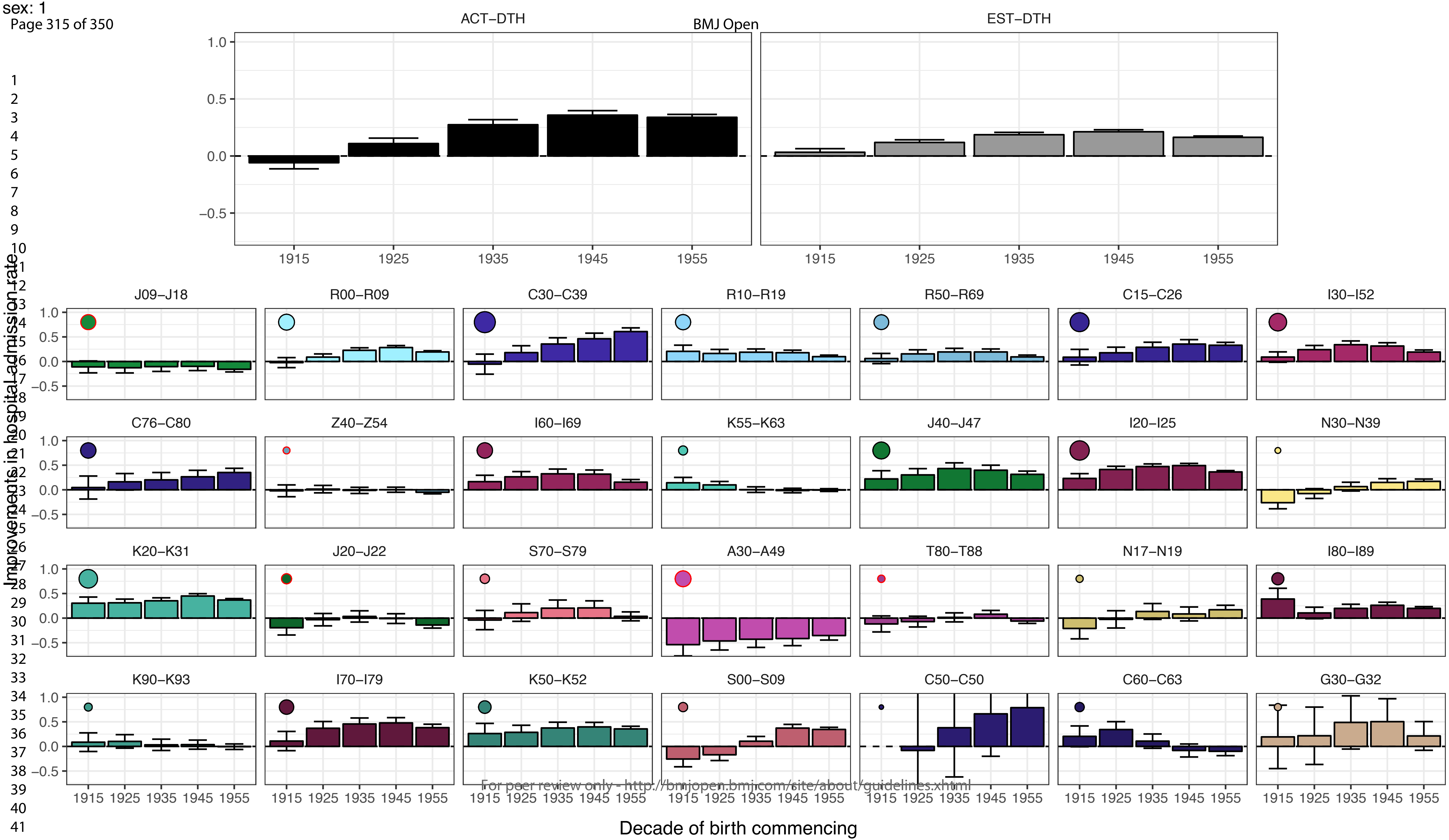

For peer review only - http://bmjopen.bmj.com/site/about/guidelines.xhtml

sex: 2

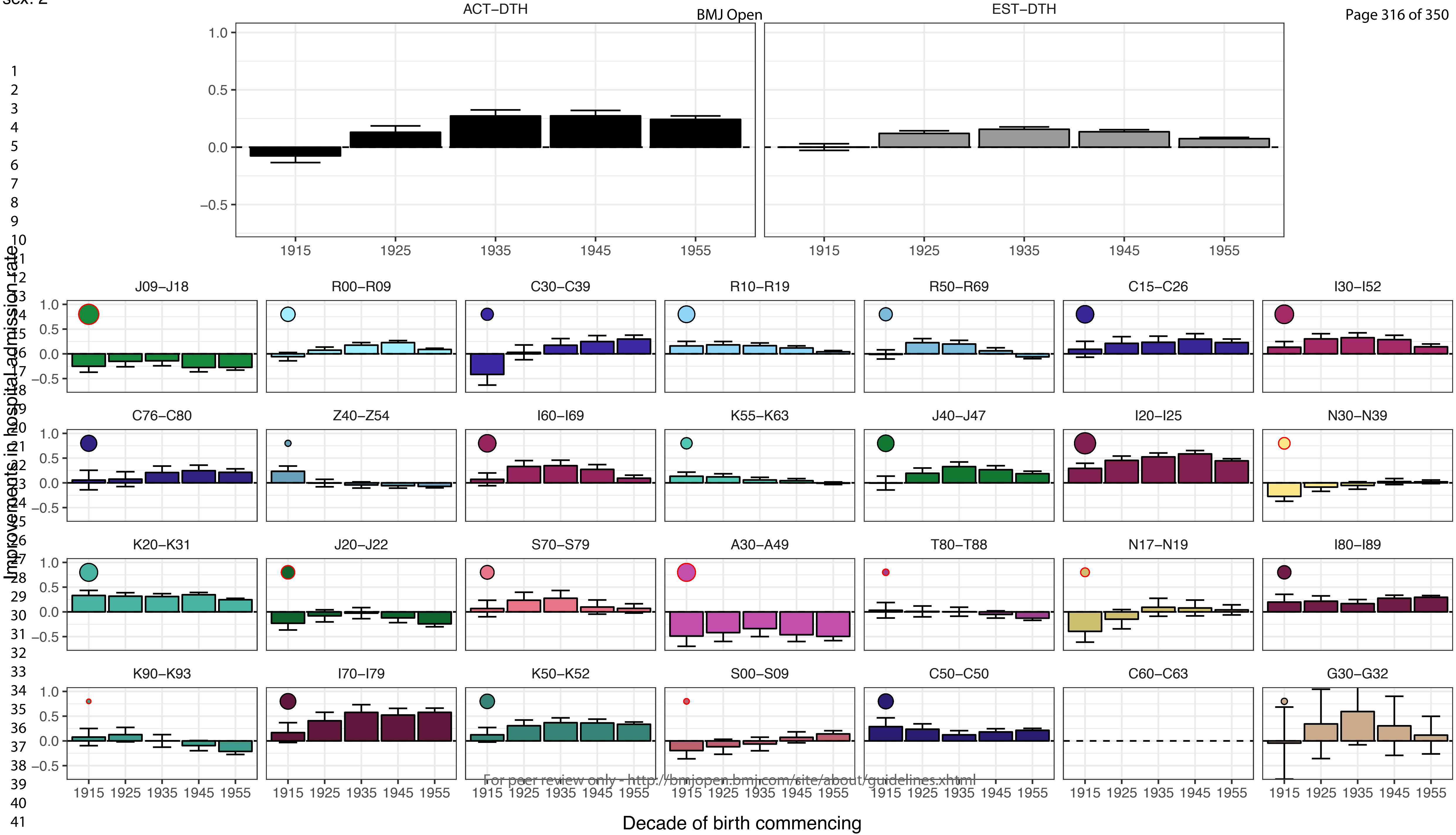

For peer review only - http://bmjopen.bmj.com/site/about/guidelines.xhtml

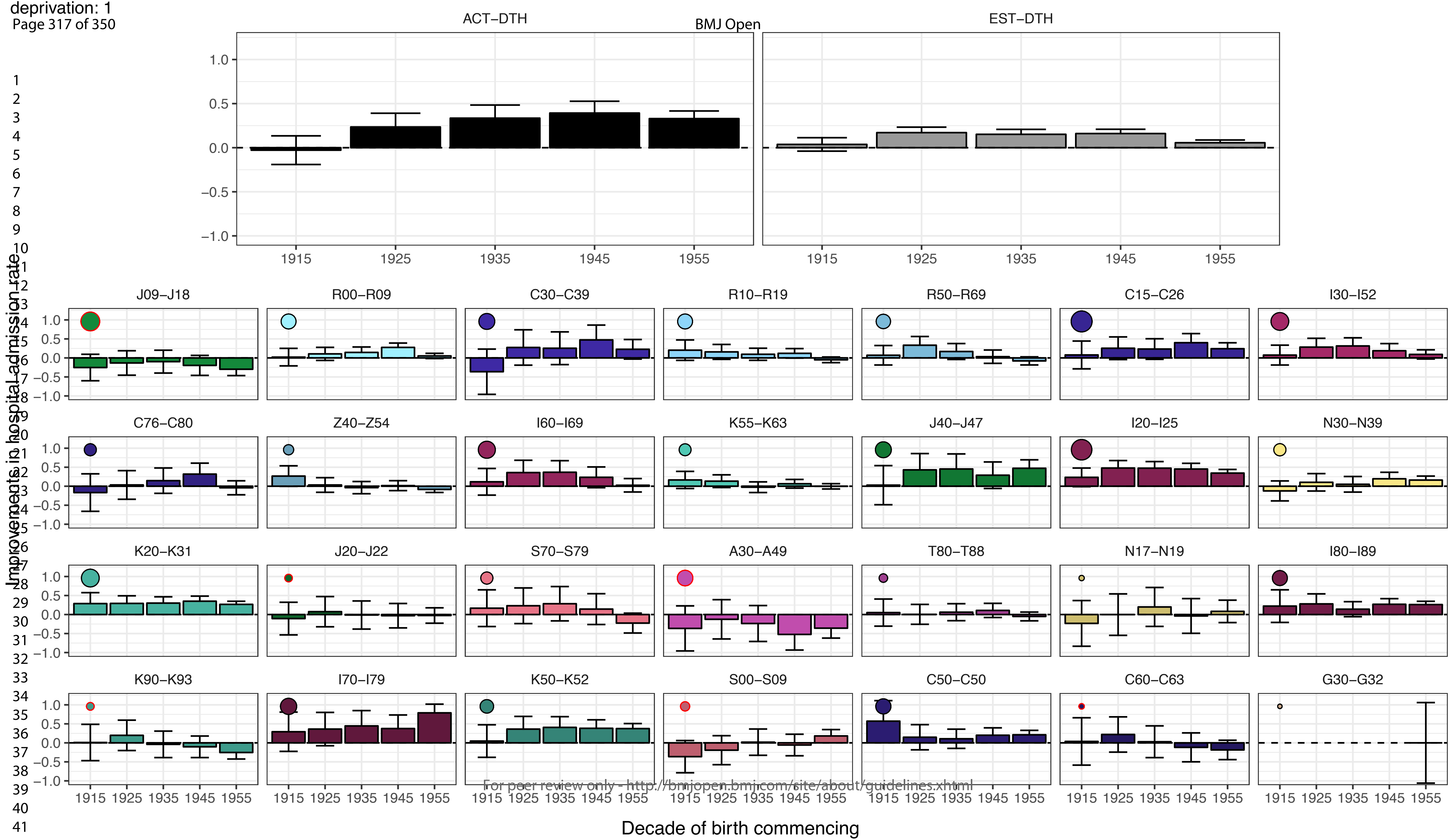

For peer review only - http://bmjopen.bmj.com/site/about/guidelines.xhtml

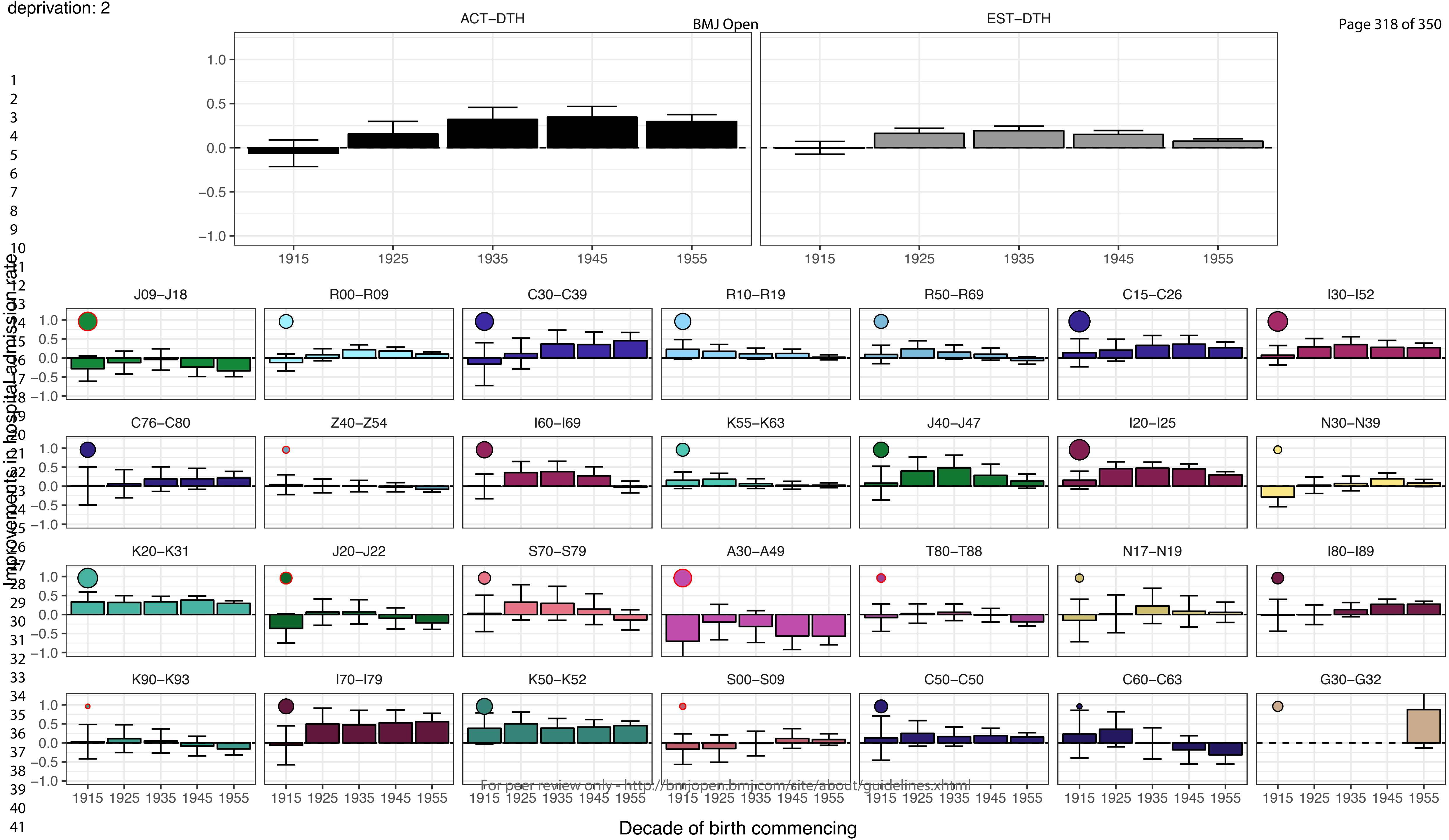

For peer review only - http://bmjopen.bmj.com/site/about/guidelines.xhtml

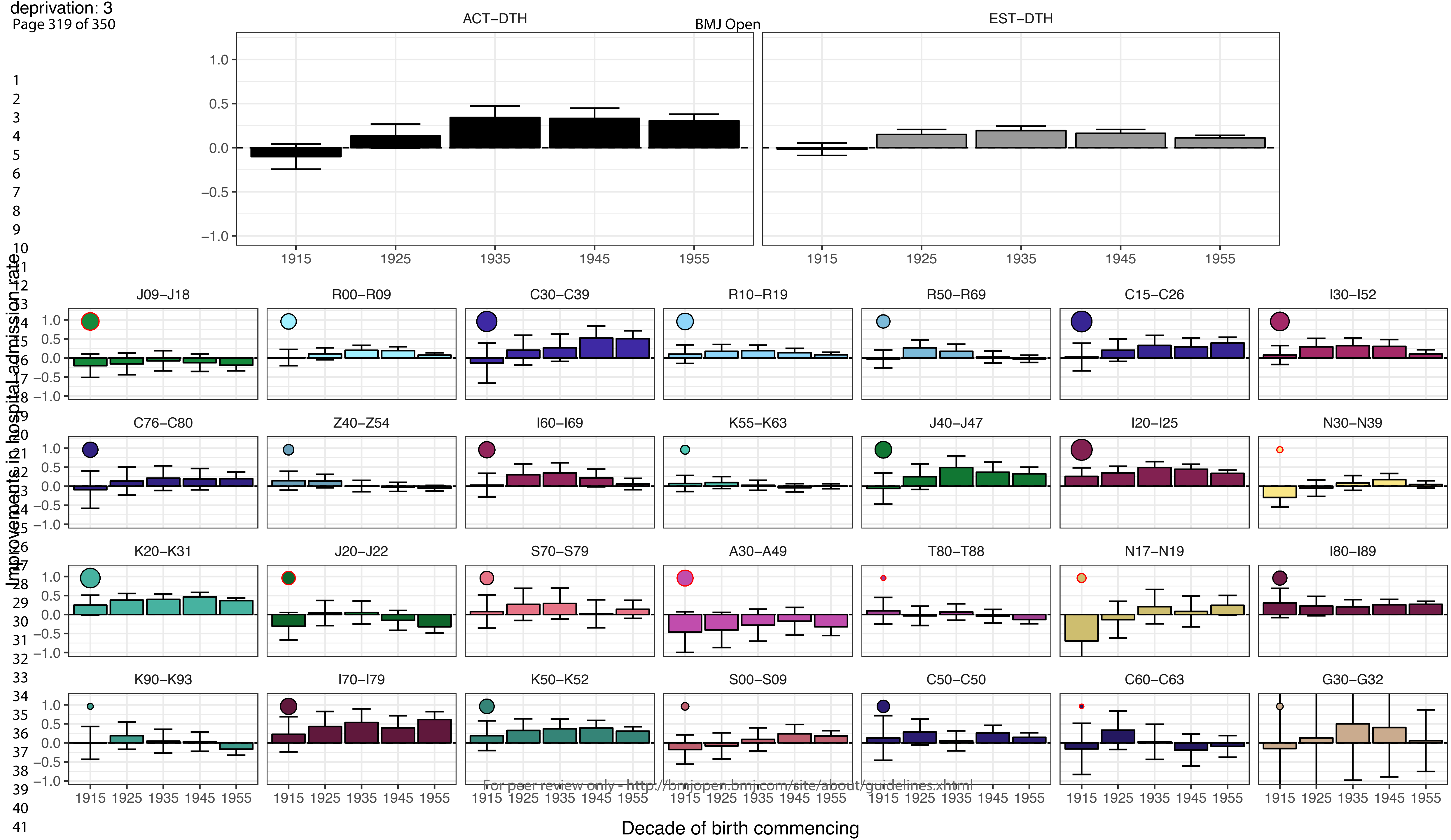

For peer review only - http://bmjopen.bmj.com/site/about/guidelines.xhtml

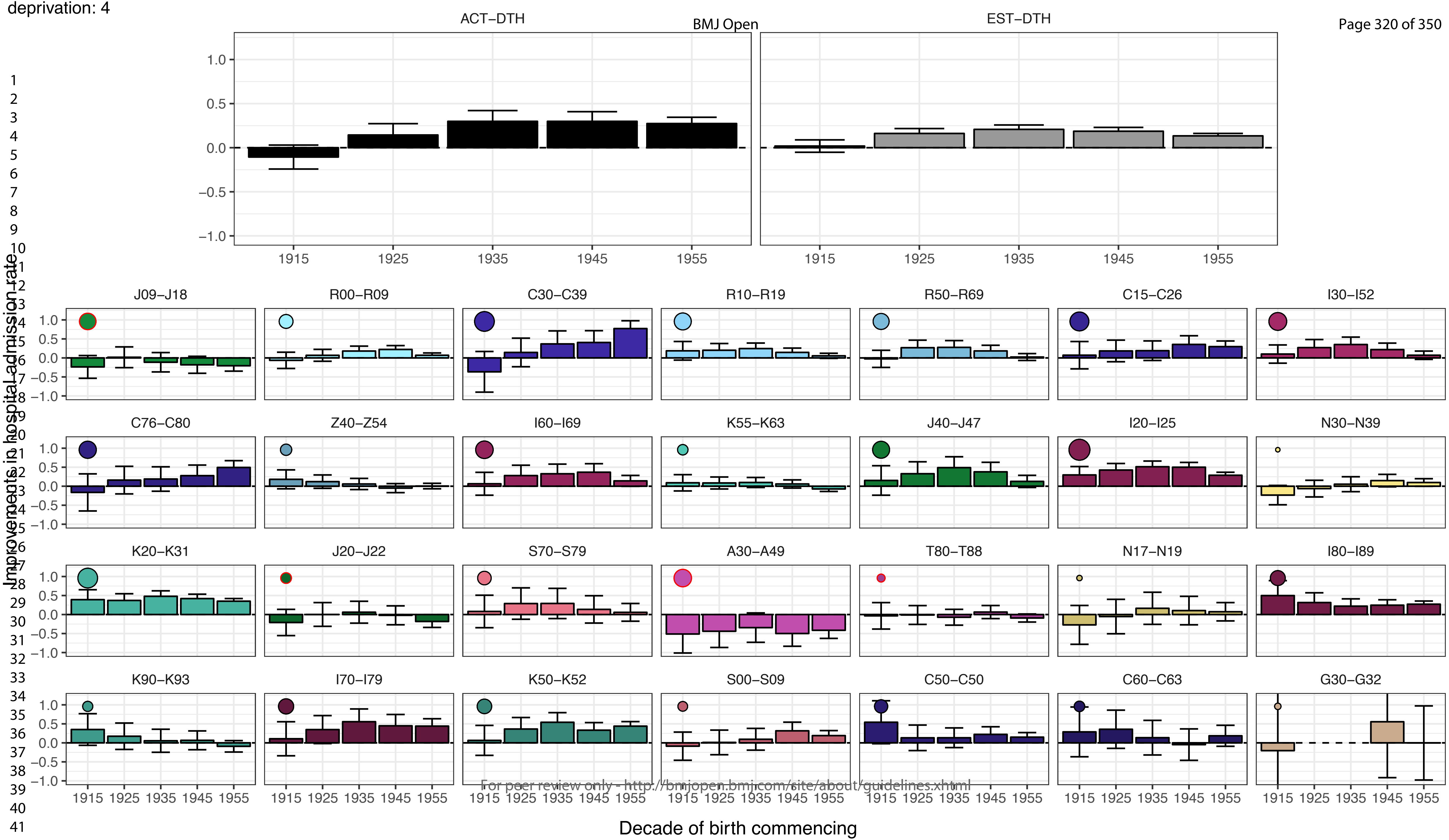

For peer review only - http://bmjopen.bmj.com/site/about/guidelines.xhtml

deprivation: 5

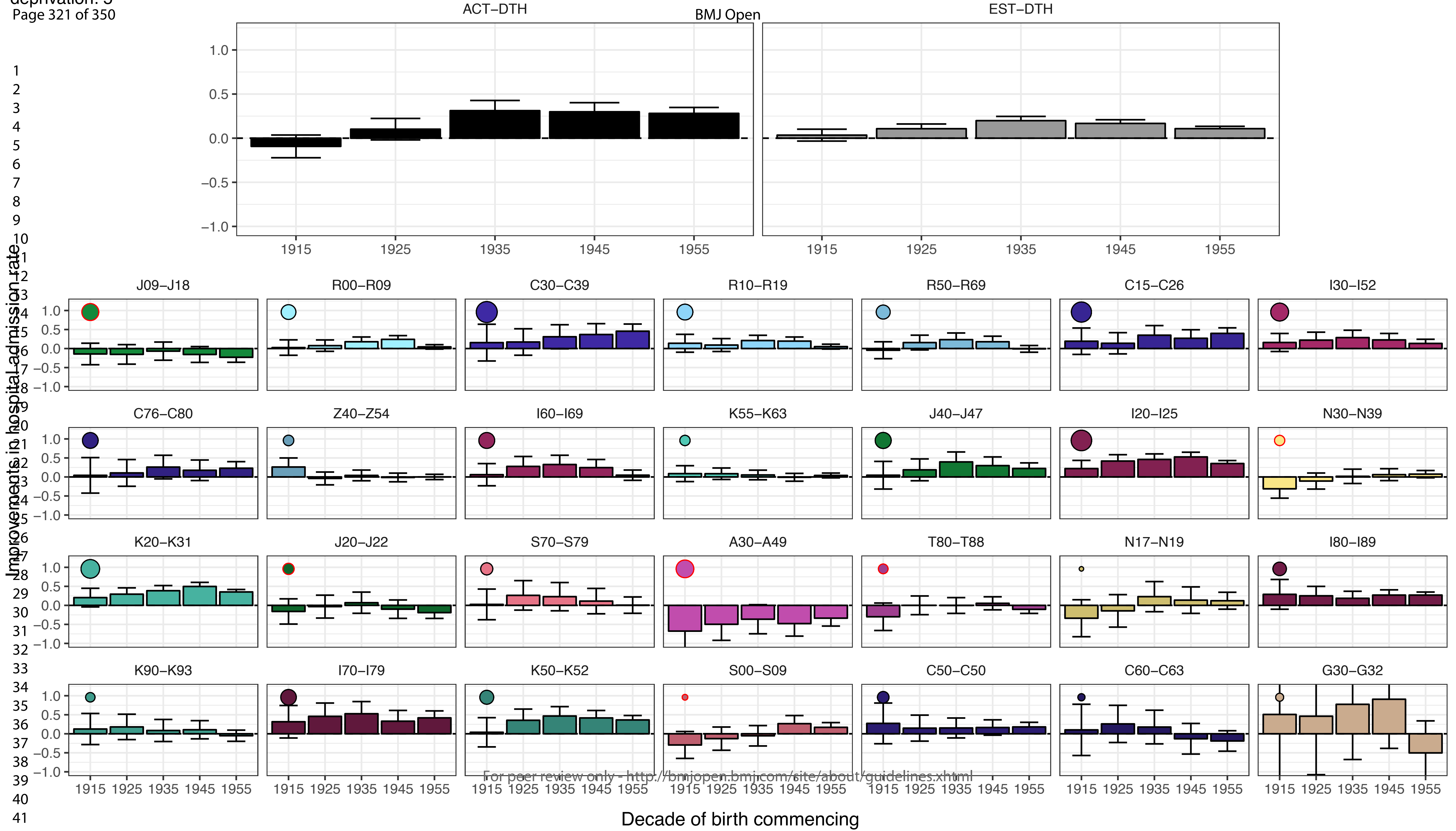

For peer review only - http://bmjopen.bmj.com/site/about/guidelines.xhtml

deprivation: 6

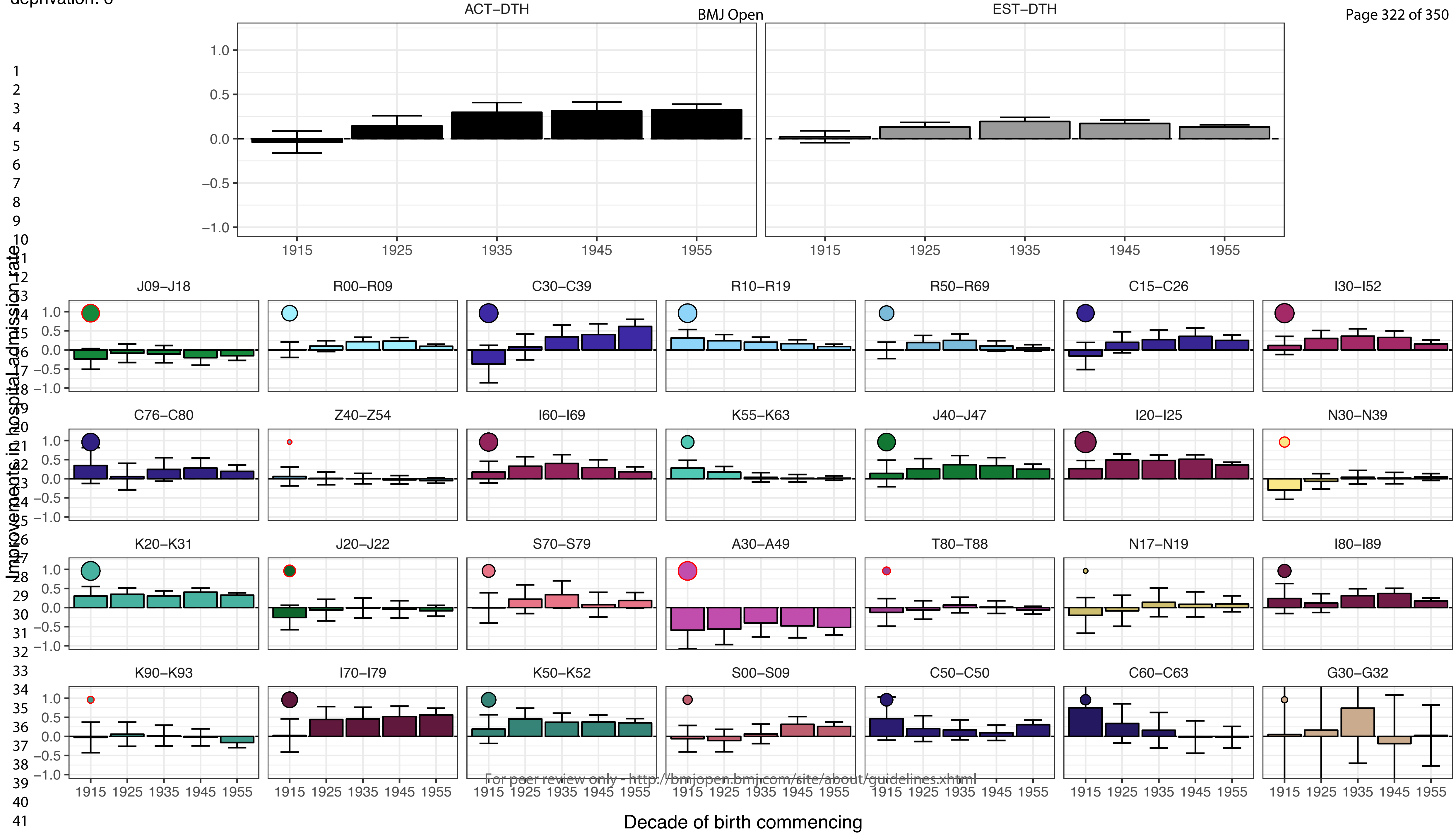

For peer review only - http://bmjopen.bmj.com/site/about/guidelines.xhtml

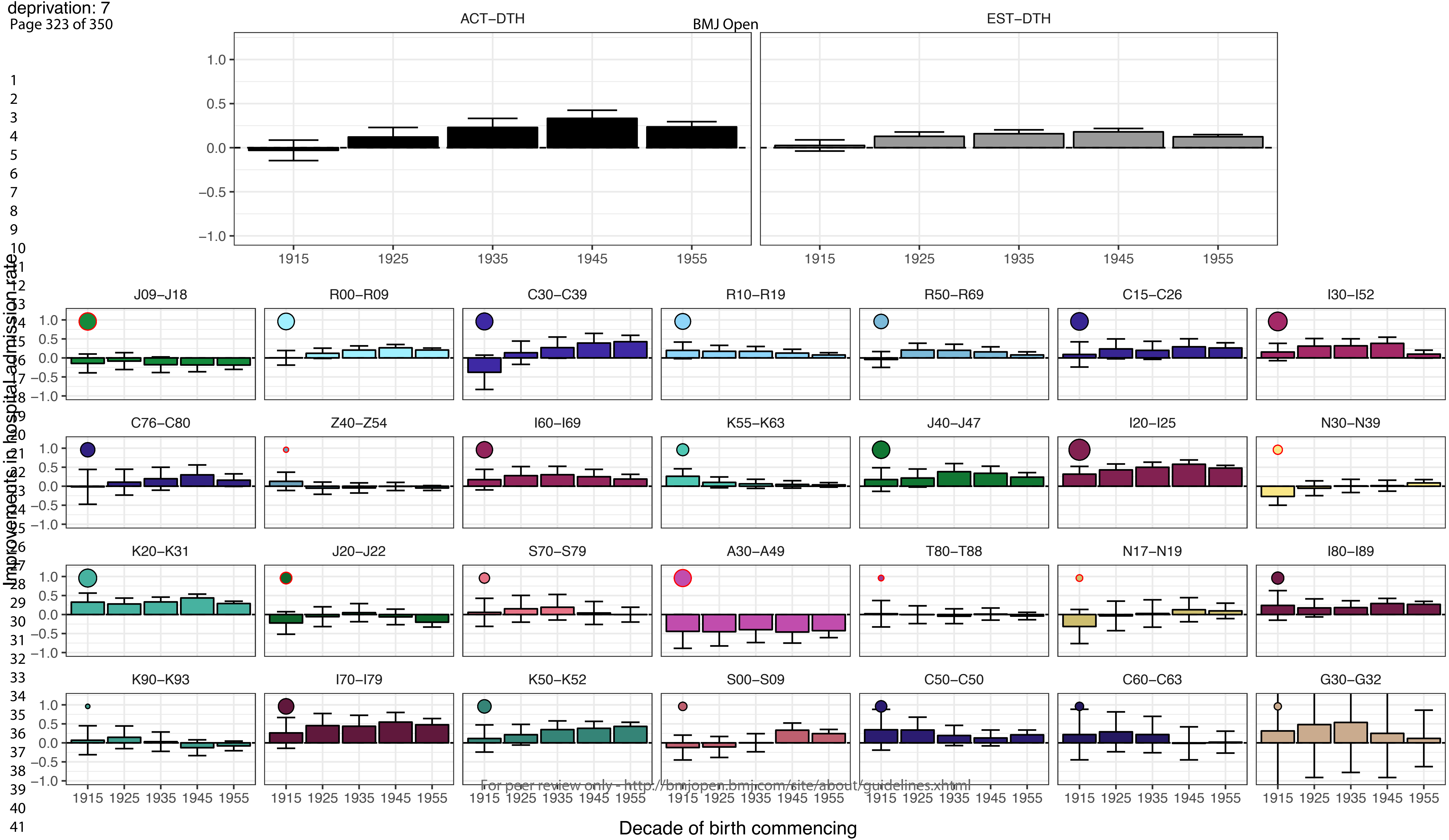

For peer review only - http://bmjopen.bmj.com/site/about/guidelines.xhtml

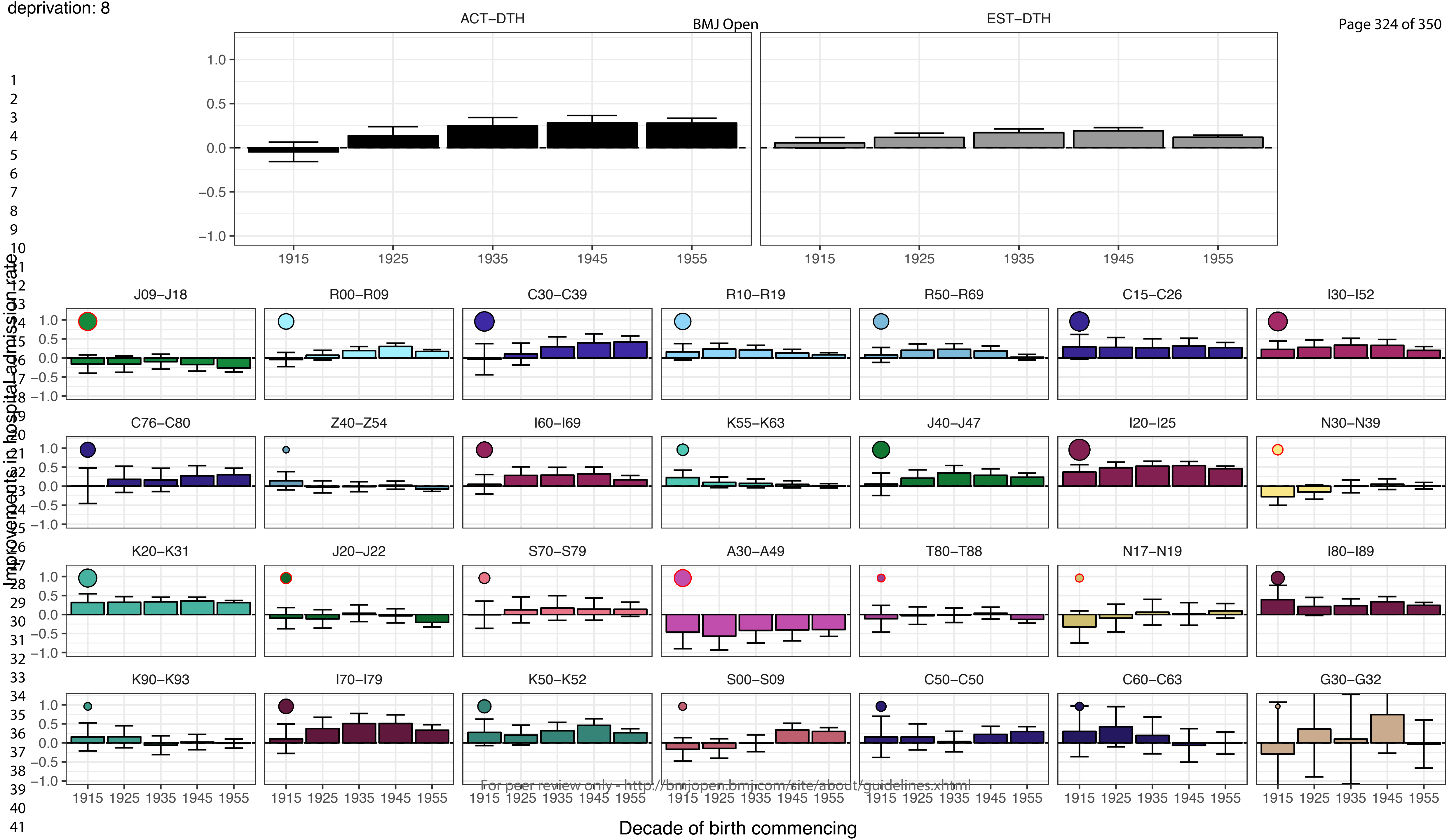

For peer review only - http://bmjopen.bmj.com/site/about/guidelines.xhtml

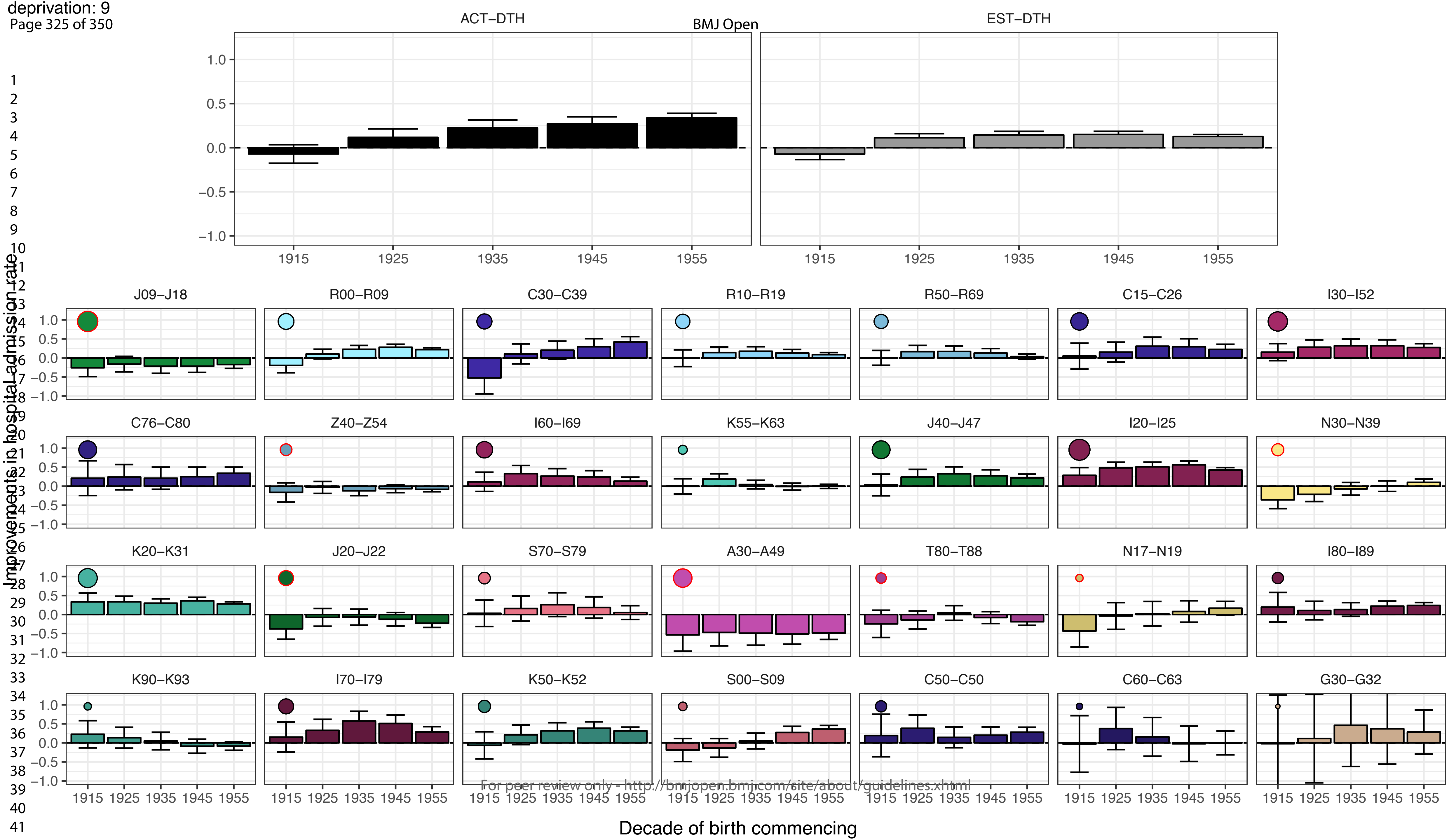

For peer review only - http://bmjopen.bmj.com/site/about/guidelines.xhtml

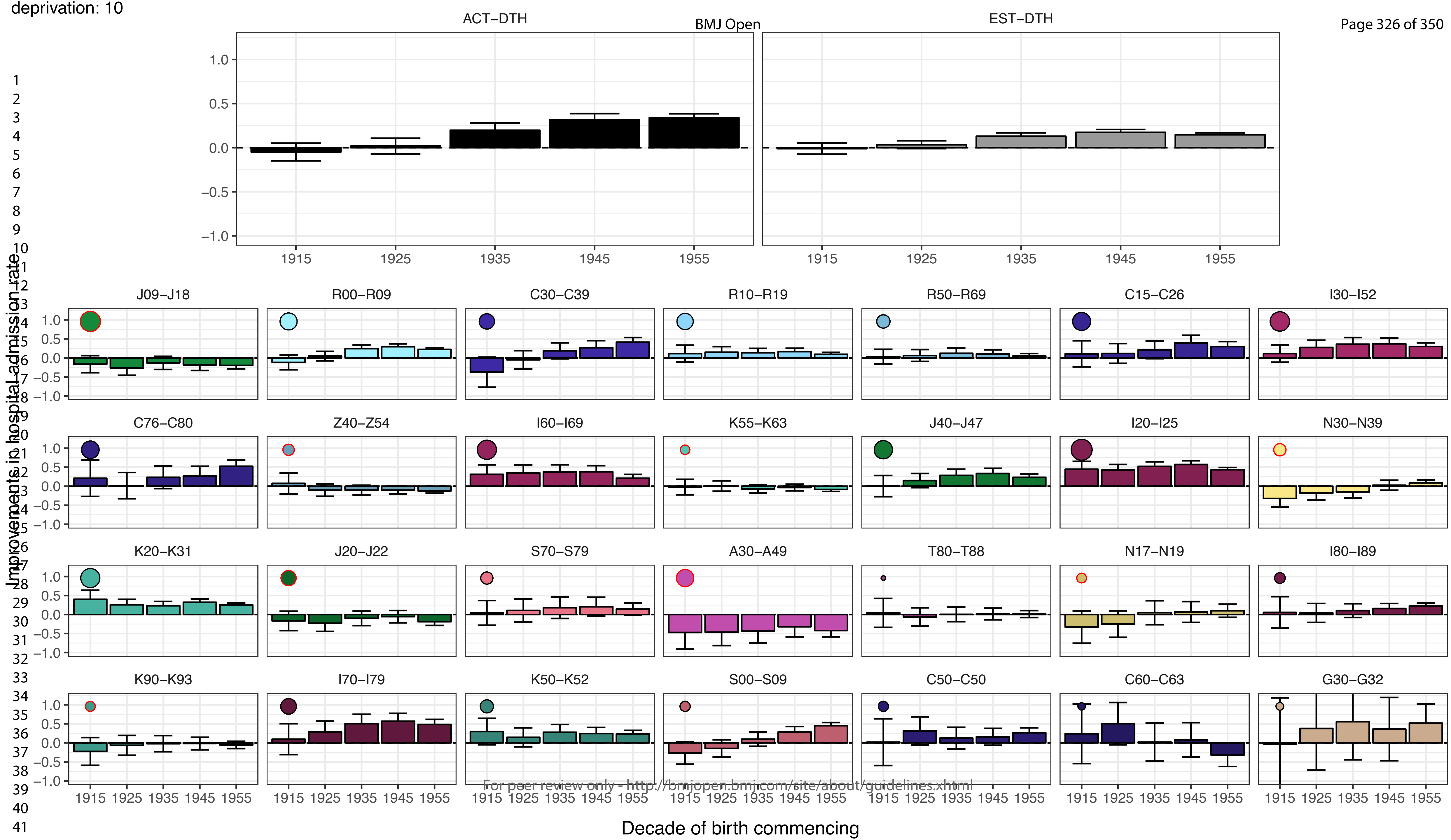

For peer review only - http://bmjopen.bmj.com/site/about/guidelines.xhtml

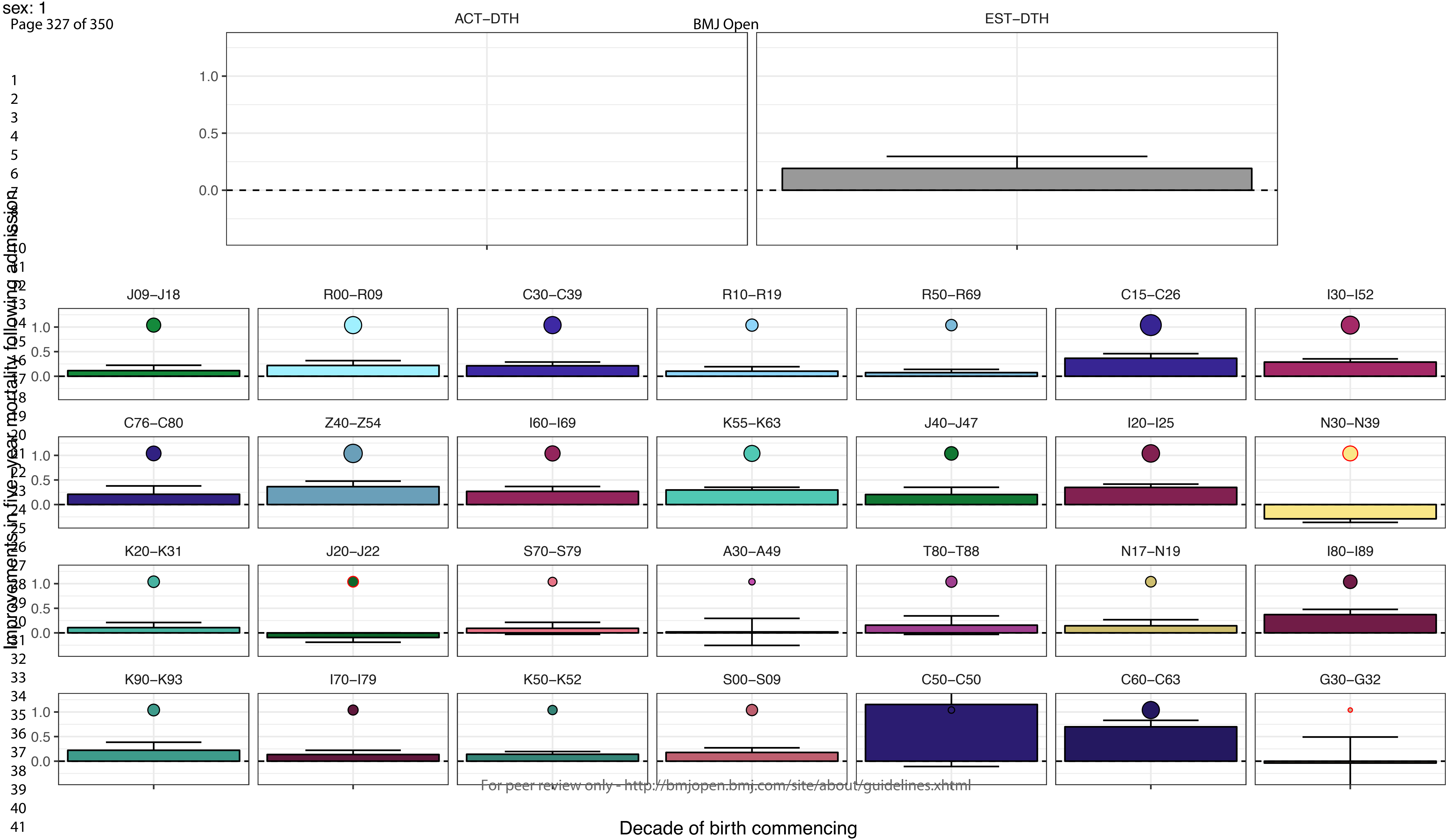

For peer review only - http://bmjopen.bmj.com/site/about/guidelines.xhtml

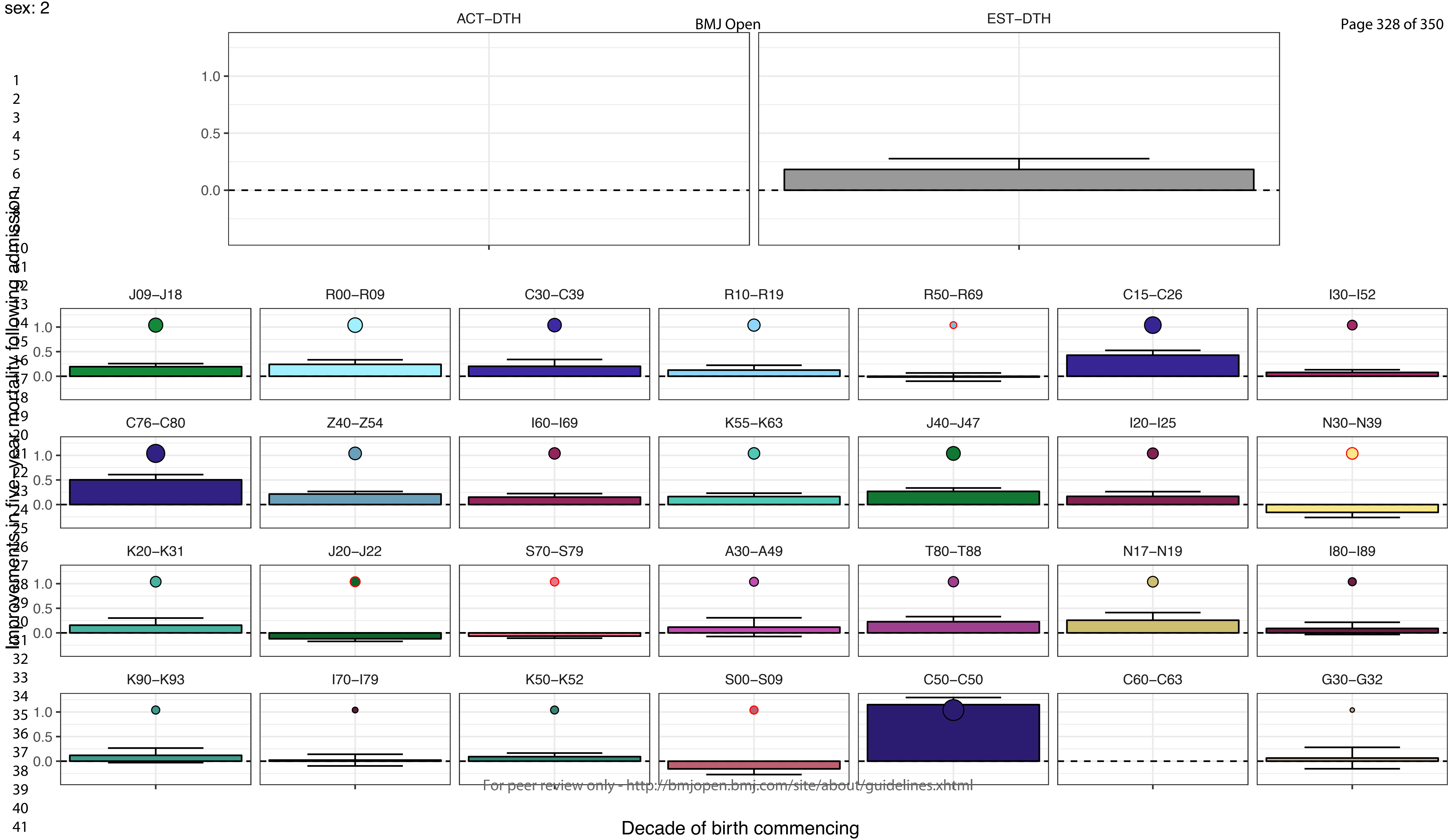

For peer review only - http://bmjopen.bmj.com/site/about/guidelines.xhtml

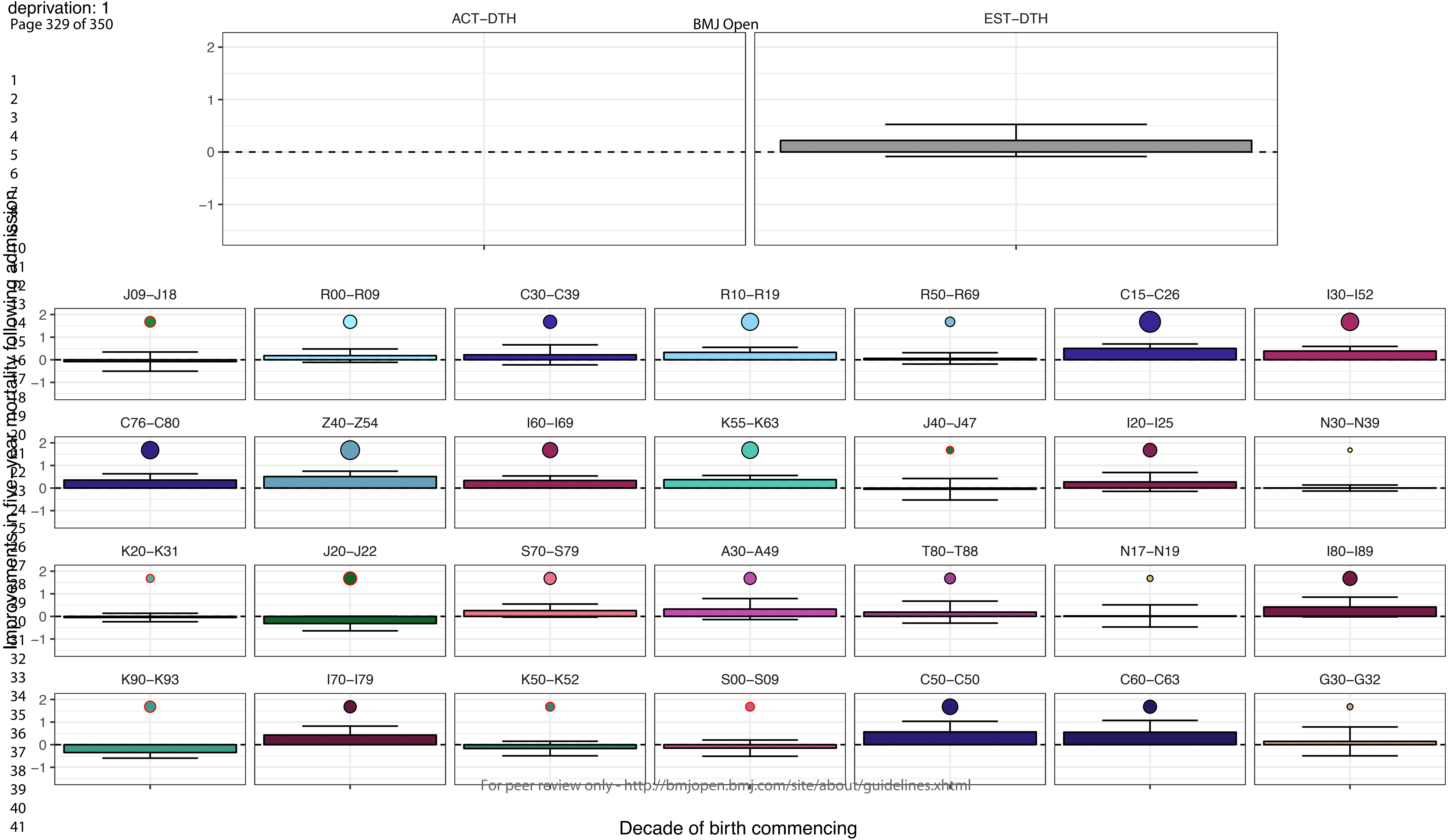
ACT−DTH
EST−DTH
J09−J18
R00−R09
C30−C39
R10−R19
R50−R69
C15−C26
I30−I52
C76−C80
Z40−Z54
I60−I69
K55−K63
J40−J47
I20−I25
N30−N39
K20−K31
J20−J22
S70−S79
A30−A49
T80−T88
N17−N19
I80−I89
K90−K93
I70−I79
K50−K52
S00−S09
C50−C50
C60−C63
G30−G32
Improvements in five−year mortality following admission
Decade of birth commencing

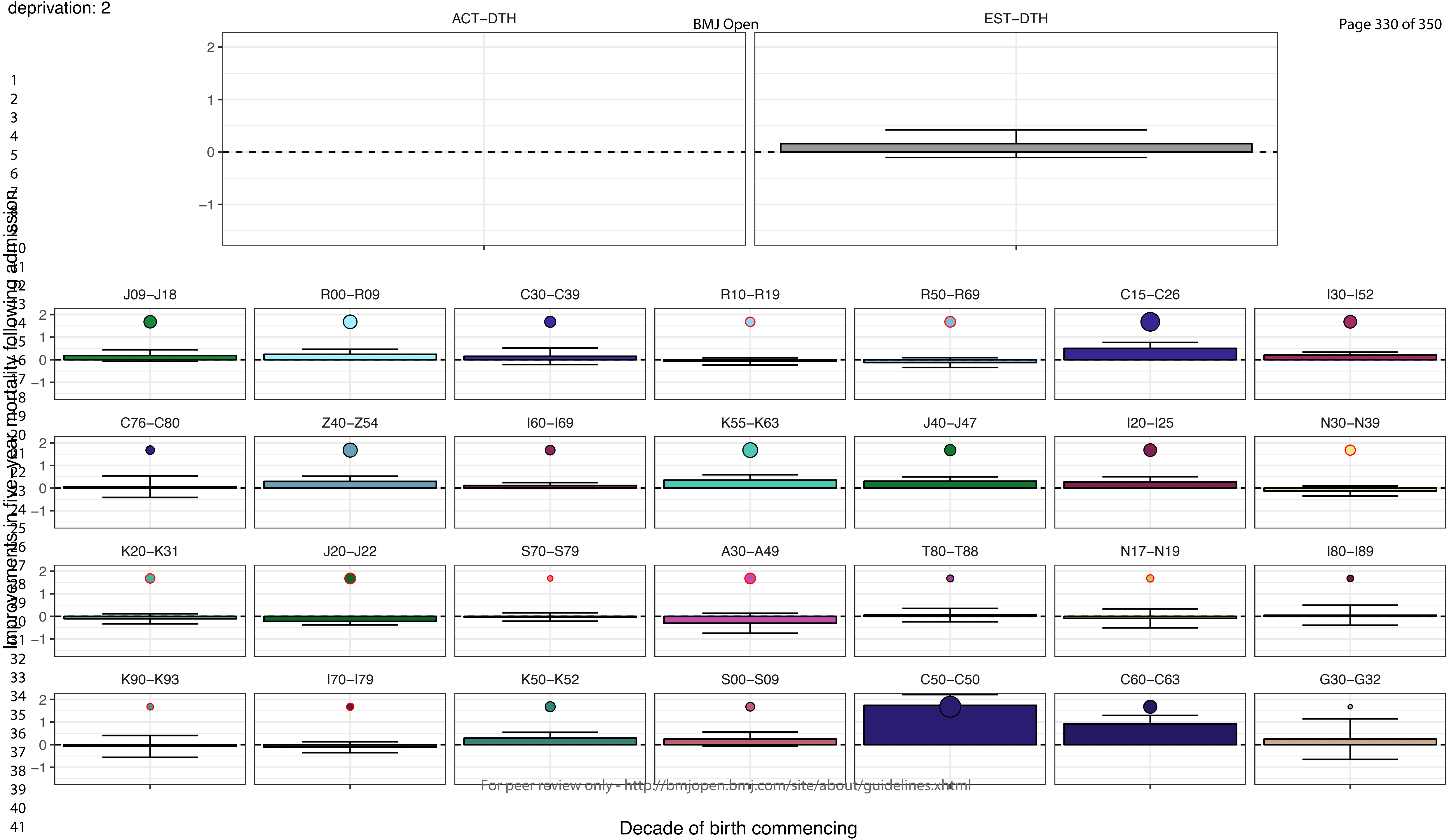

For peer review only - http://bmjopen.bmj.com/site/about/guidelines.xhtml

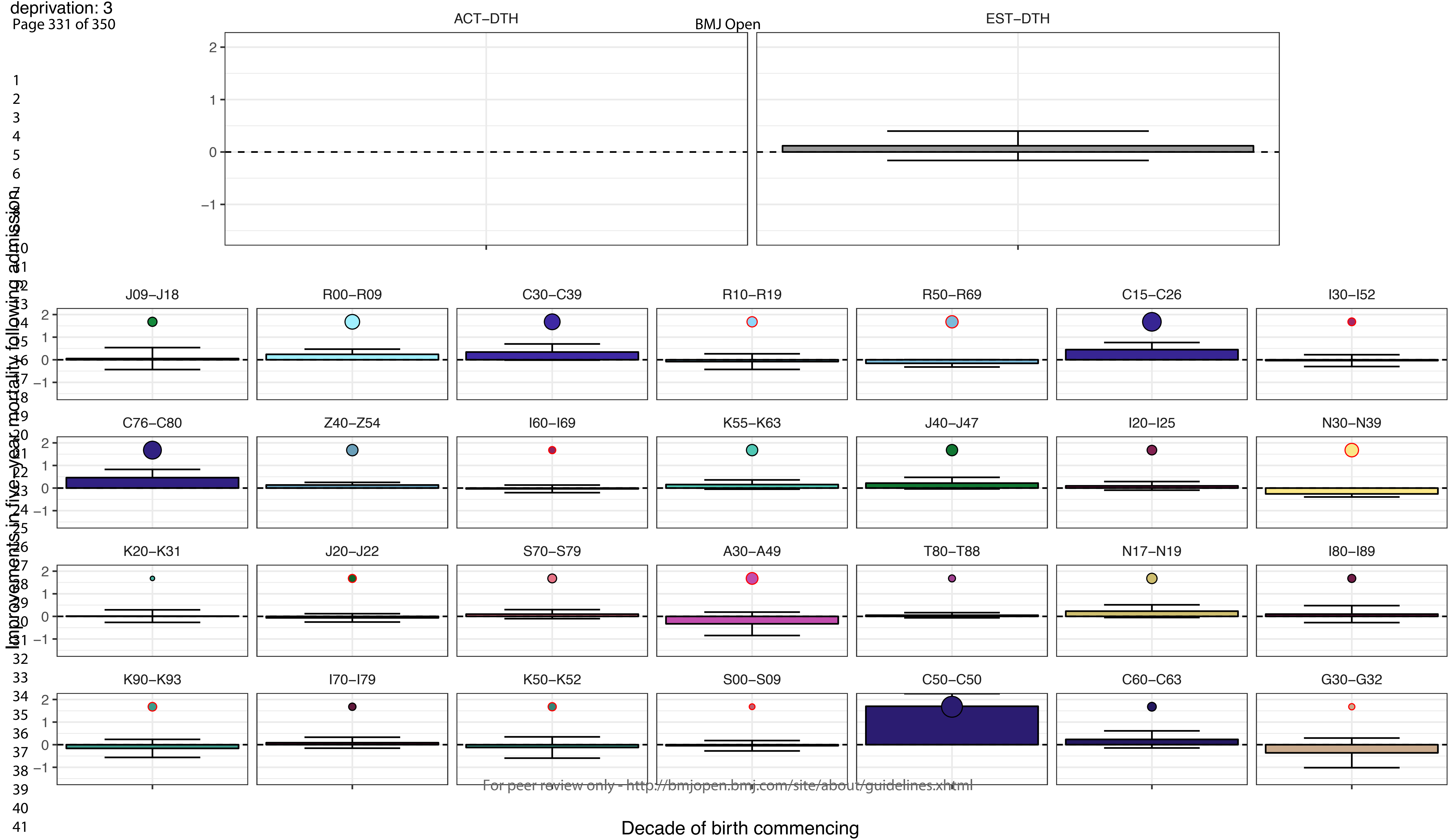

For peer review only - http://bmjopen.bmj.com/site/about/guidelines.xhtml

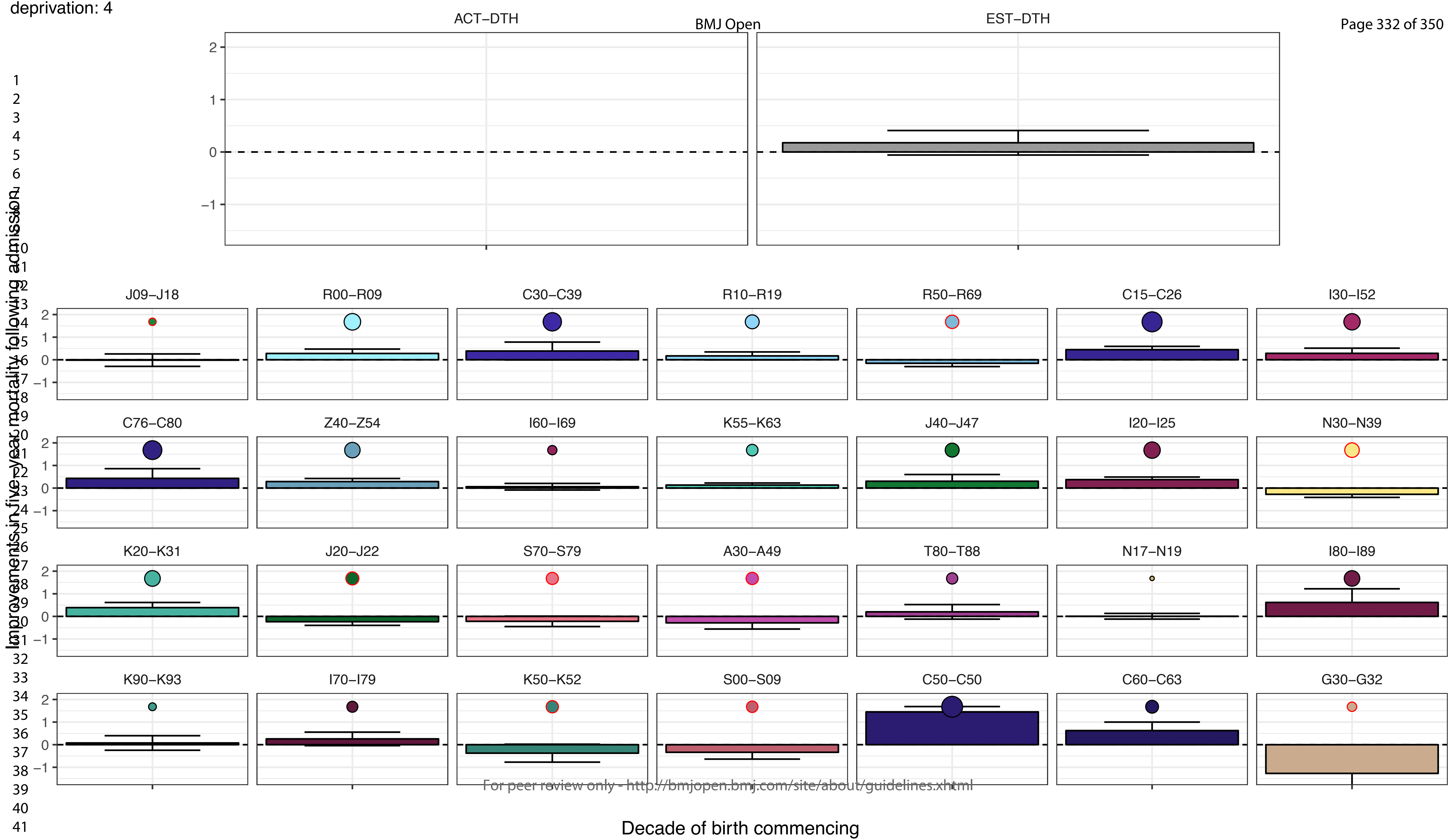

For peer review only - http://bmjopen.bmj.com/site/about/guidelines.xhtml

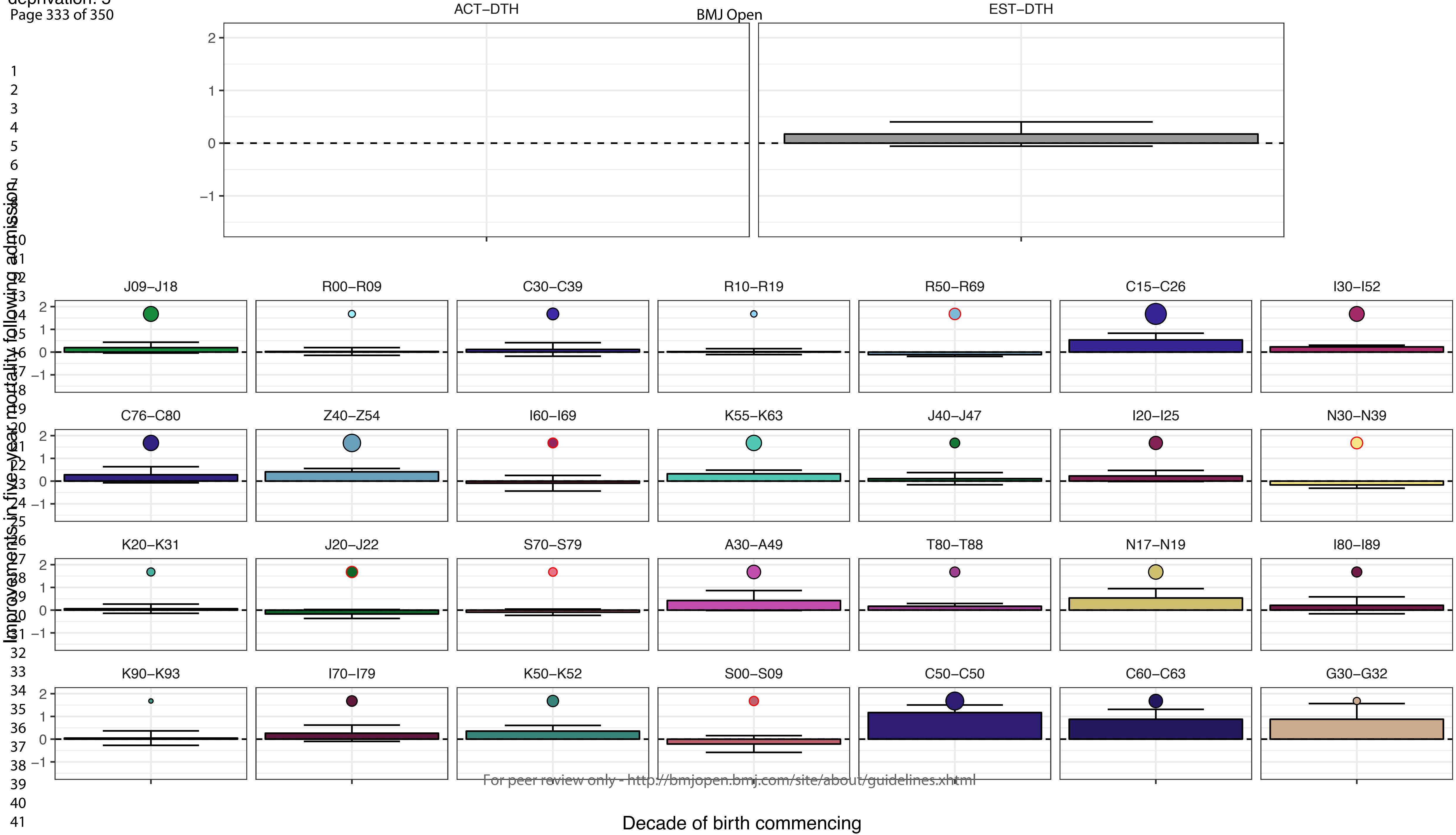

For peer review only - http://bmjopen.bmj.com/site/about/guidelines.xhtml

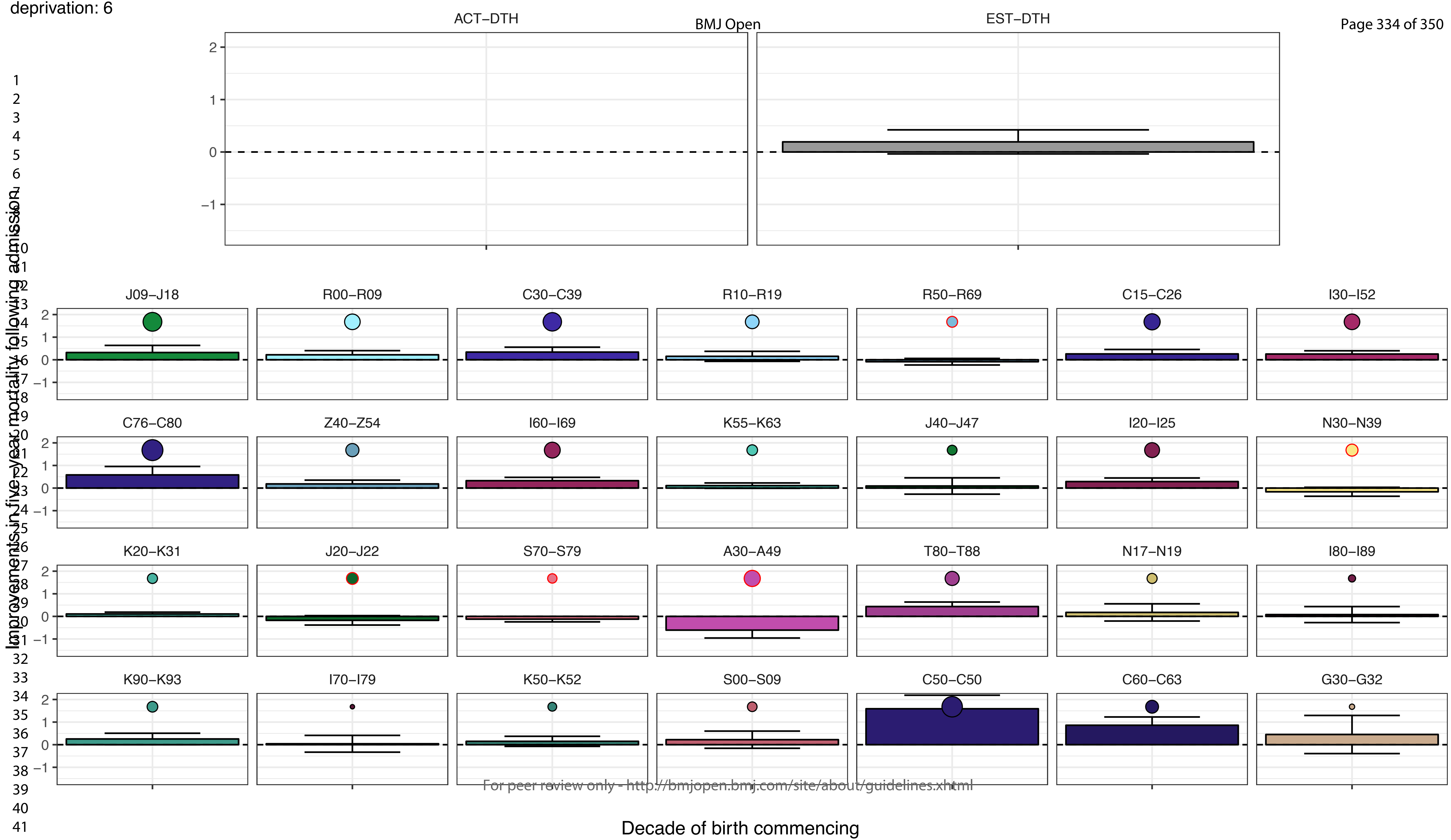
deprivation: 6
ACT–DTH
EST–DTH
J09–J18
R00–R09
C30–C39
R10–R19
R50–R69
C15–C26
I30–I52
C76–C80
Z40–Z54
I60–I69
K55–K63
J40–J47
I20–I25
N30–N39
K20–K31
J20–J22
S70–S79
A30–A49
T80–T88
N17–N19
I80–I89
K90–K93
I70–I79
K50–K52
S00–S09
C50–C50
C60–C63
G30–G32
Improvements in five-year mortality following admission
Decade of birth commencing

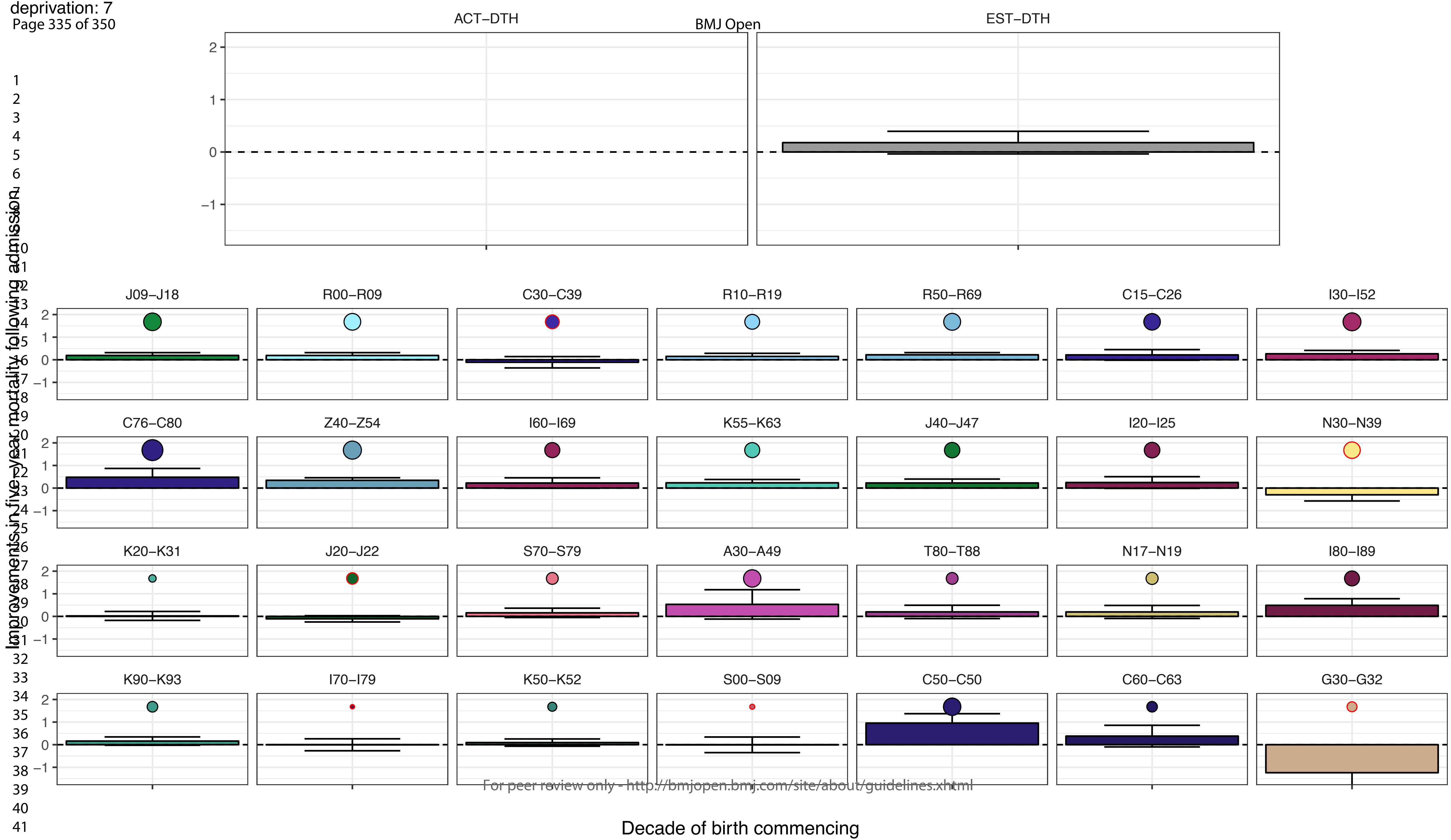
deprivation: 7
ACT−DTH
EST−DTH
J09−J18
R00−R09
C30−C39
R10−R19
R50−R69
C15−C26
I30−I52
C76−C80
Z40−Z54
I60−I69
K55−K63
J40−J47
I20−I25
N30−N39
K20−K31
J20−J22
S70−S79
A30−A49
T80−T88
N17−N19
I80−I89
K90−K93
I70−I79
K50−K52
S00−S09
C50−C50
C60−C63
G30−G32
Improvements in five−year mortality following admission
Decade of birth commencing

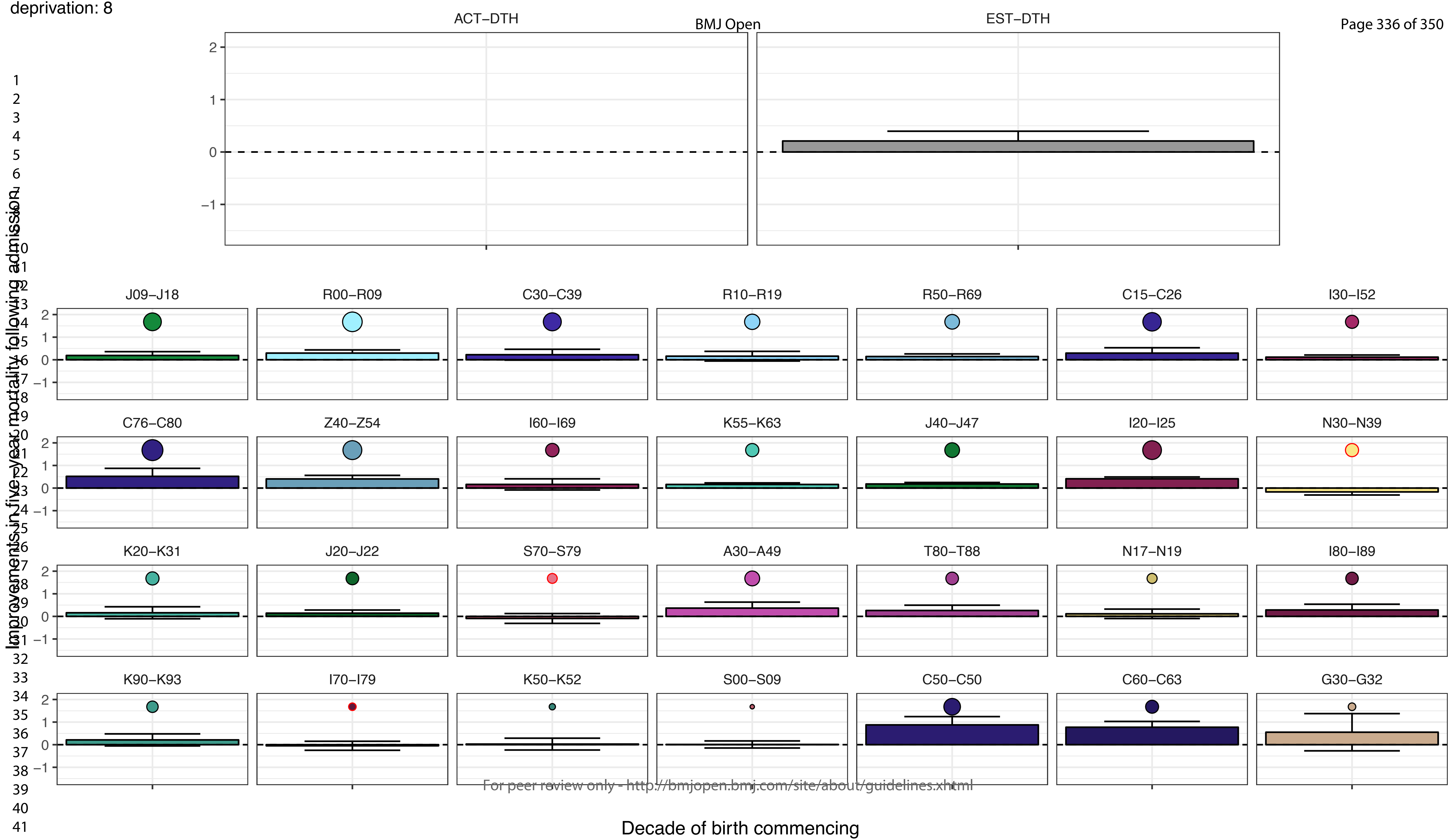

For peer review only - http://bmjopen.bmj.com/site/about/guidelines.xhtml

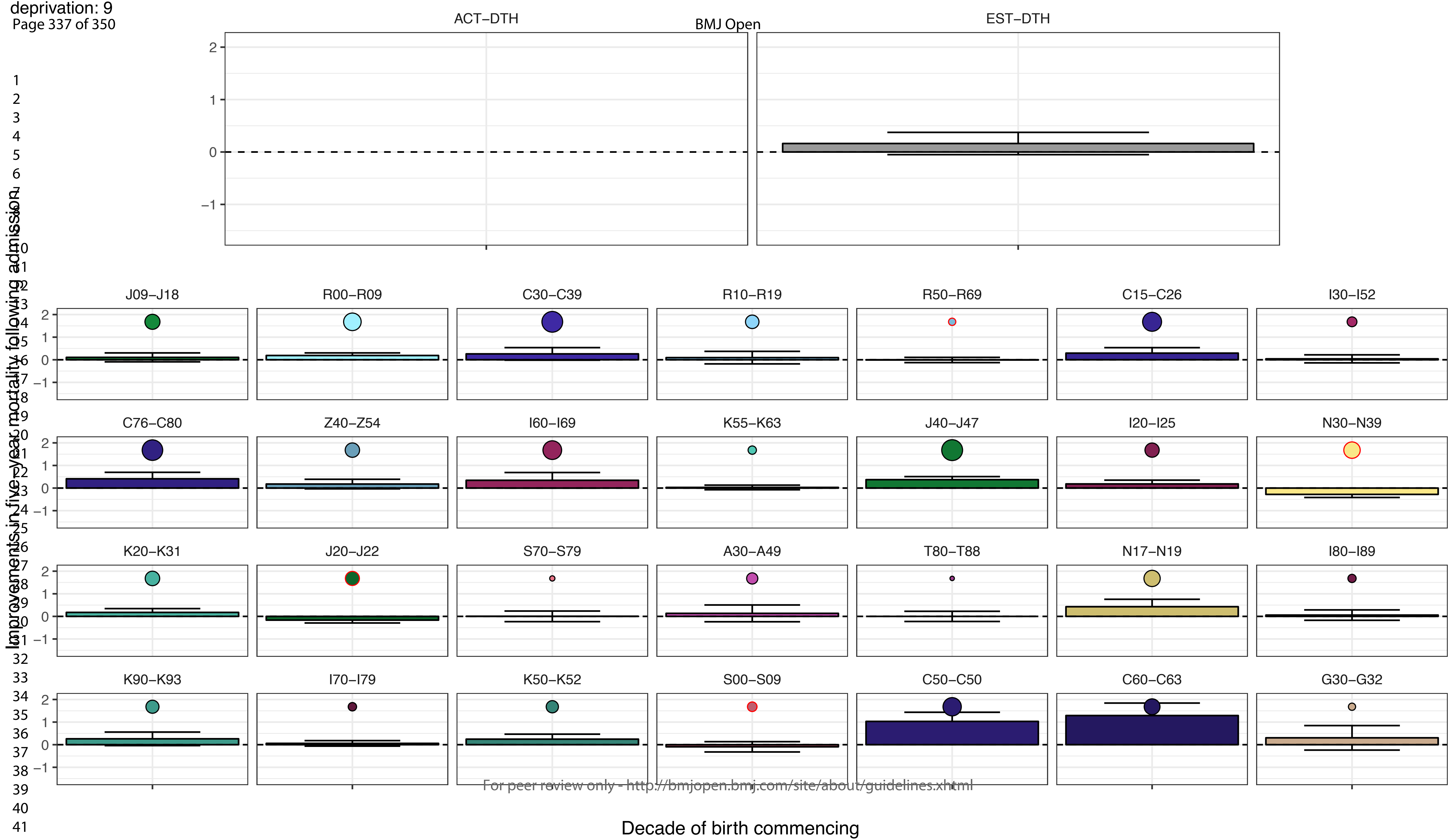

For peer review only - http://bmjopen.bmj.com/site/about/guidelines.xhtml

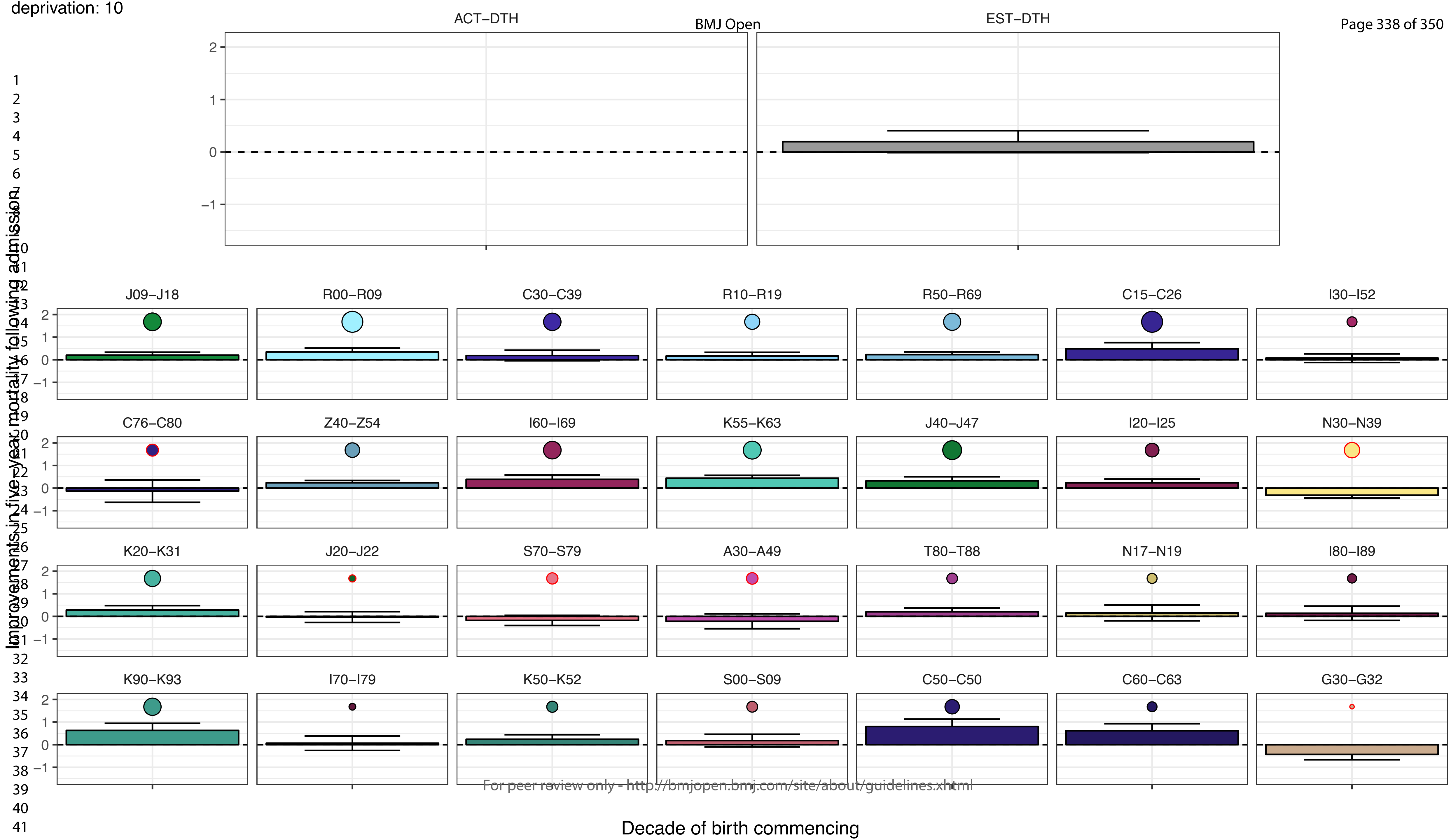
deprivation: 10
ACT−DTH
EST−DTH
J09−J18
R00−R09
C30−C39
R10−R19
R50−R69
C15−C26
I30−I52
C76−C80
Z40−Z54
I60−I69
K55−K63
J40−J47
I20−I25
N30−N39
K20−K31
J20−J22
S70−S79
A30−A49
T80−T88
N17−N19
I80−I89
K90−K93
I70−I79
K50−K52
S00−S09
C50−C50
C60−C63
G30−G32
Improvements in five−year mortality following admission
Decade of birth commencing

The RECORD statement – checklist of items, extended from the STROBE statement, that should be reported in observational studies using routinely collected health data.

	Item No.	STROBE items	Location in manuscript where items are reported	RECORD items	Location in manuscript where items are reported
**Title and abstract**					
	1	(a) Indicate the study's design with a commonly used term in the title or the abstract (b) Provide in the abstract an informative and balanced summary of what was done and what was found	Page 1:	RECORD 1.1: The type of data used should be specified in the title or abstract. When possible, the name of the databases used should be included.<br><br>RECORD 1.2: If applicable, the geographic region and timeframe within which the study took place should be reported in the title or abstract.<br><br>RECORD 1.3: If linkage between databases was conducted for the study, this should be clearly stated in the title or abstract.	Page 1: "nationwide cohort study of linked hospital admission and death records 2001–2016"
**Introduction**					
Background rationale	2	Explain the scientific background and rationale for the investigation being reported	Page 5: "Understanding the trends … has the potential to reveal whether investment in healthcare and research is directed at the most urgent diseases and most affected individuals."		

For peer review only

Objectives	3	State specific objectives, including any prespecified hypotheses	Page 5: "explore how trends in longevity were driven by different trends in broad classes of disease incidence or survival, and highlight diseases which have shown more or less improvement in their contribution to overall mortality"		
**Methods**					
Study Design	4	Present key elements of study design early in the paper	Page 6-8: Data Sources & Statistical Analysis		
Setting	5	Describe the setting, locations, and relevant dates, including periods of recruitment, exposure, follow-up, and data collection	Page 6: Data Sources		
Participants	6	*(a) Cohort study* - Give the eligibility criteria, and the sources and methods of selection of participants. Describe methods of follow-up *Case-control study* - Give the eligibility criteria, and the sources and methods of case ascertainment and control selection. Give the rationale for the choice of cases and controls *Cross-sectional study* - Give the eligibility criteria, and the sources and methods of selection of participants	Page 6: Data Sources "Our extract consisted … the identified population of Scotland in 2001 who had been born between 1905 and 1965"	RECORD 6.1: The methods of study population selection (such as codes or algorithms used to identify subjects) should be listed in detail. If this is not possible, an explanation should be provided.<br><br>RECORD 6.2: Any validation studies of the codes or algorithms used to select the population should be referenced. If validation was conducted for this study and not published elsewhere, detailed methods and results should be provided.	Page 6: Data Sources "The CHI number, contained in the dataset, is effectively a patient identifier and added to other health datasets to make linkage possible, for instance between hospital admissions, death records and the

For peer review only

		(b) Cohort study - For matched studies, give matching criteria and number of exposed and unexposed<br>*Case-control study* - For matched studies, give matching criteria and the number of controls per case	N/A<br><br>N/A	RECORD 6.3: If the study involved linkage of databases, consider use of a flow diagram or other graphical display to demonstrate the data linkage process, including the number of individuals with linked data at each stage.	Scottish cancer registry (reference to paper describing linkage)"
Variables	7	Clearly define all outcomes, exposures, predictors, potential confounders, and effect modifiers. Give diagnostic criteria, if applicable.	Page 6,7:<br>"The main diagnosis of acute hospital admission records was used to classify records into disease categories"<br><br>"fitting sex, decade of birth, and deprivation"	RECORD 7.1: A complete list of codes and algorithms used to classify exposures, outcomes, confounders, and effect modifiers should be provided. If these cannot be reported, an explanation should be provided.	"main diagnoses of each NNS hospital admission into categories, as laid out by the ICD10 Chapters"<br><br>"Analysis was restricted to more common disease blocks. Visual inspection suggested a pragmatic threshold of at least 15,000 first-time admissions (see Table for all disease categories meeting this threshold)."
Data sources/ measurement	8	For each variable of interest, give sources of data and details of methods of assessment (measurement).<br>Describe comparability of assessment methods if there is more than one group	Page 7, 8:<br>"A Cox proportional hazards model using NRS mortality data"<br><br>"We grouped the main diagnoses of each NSS hospital		

			admission into categories, as laid out by the ICD10 Chapters"<br><br>"relative mortality burden of each disease block was calculated as the excess mortality (lnHR) in the 5 years after hospital admission (Equation 1) multiplied by the number of first-time admissions for the disease block"		
Bias	9	Describe any efforts to address potential sources of bias	Page 8:<br>"Whilst this measure may in principle be affected by differing age patterns on incidence, it was judged sufficient for our purpose – to establish broad relative weightings of the importance of each disease."<br><br>"The use of (first) hospitalisation for a disease as our definition of incidence is imperfect"		

For peer review only

Study size	10	Explain how the study size was arrived at	Page 7: "we excluded individuals with district codes with less than 5,000 individuals (thereby excluding anomalous postcodes, often with special meanings, such as "marketing campaign"; N = 11,564). We also excluded individuals missing from the CHI database in 2016, but not recorded as dead (and therefore likely transferred out of Scotland; N = 573,711), individuals with record discrepancies between the CHI and National Records of Scotland databases (N = 79,131), and individuals with records outside of the study dates or missing information on socioeconomic class (N = 59,767), giving 1,967,130 individuals for analysis post-QC"		

For peer review only

Quantitative variables	11	Explain how quantitative variables were handled in the analyses. If applicable, describe which groupings were chosen, and why	Page 7:<br>"In order to quantify mortality in the 5 years following hospitalisation, person-time of individuals was divided into phases, corresponding to the study start until hospitalisation, the first 5 years after hospitalisation, and the remaining time in the study."<br><br>"The status of each phase was fitted as a covariate in a Cox proportional hazards model (ref) with death as the event, adjusting for sex and deprivation"		
Statistical methods	12	(a) Describe all statistical methods, including those used to control for confounding<br>(b) Describe any methods used to examine subgroups and interactions<br>(c) Explain how missing data were addressed<br>(d) *Cohort study* - If applicable, explain how loss to follow-up was addressed<br>*Case-control study* - If applicable, explain how	Page 7, 8:<br>"Cox proportional hazard models"<br><br>"we fit sex, deprivation, and decade of birth as covariates. Again, the same analyses were performed when stratifying by sex and deprivation"		

For peer review only

		matching of cases and controls was addressed<br>*Cross-sectional study* - If applicable, describe analytical methods taking account of sampling strategy<br>(e) Describe any sensitivity analyses	"only individuals with complete records were included in the analysis."		
Data access and cleaning methods		..		RECORD 12.1: Authors should describe the extent to which the investigators had access to the database population used to create the study population.<br><br>RECORD 12.2: Authors should provide information on the data cleaning methods used in the study.	Page 6:<br>"The dataset we received contained only records with district-level postcodes and SIMD deciles"<br><br>"excluded individuals…"
Linkage		..		RECORD 12.3: State whether the study included person-level, institutional-level, or other data linkage across two or more databases. The methods of linkage and methods of linkage quality evaluation should be provided.	Page 6:<br>"The CHI number, contained in the dataset, is effectively a patient identifier and added to other health datasets to make linkage possible, for instance between hospital admissions, death records and the Scottish cancer registry"
**Results**					

For peer review only

Participants	13	(a) Report the numbers of individuals at each stage of the study (*e.g.*, numbers potentially eligible, examined for eligibility, confirmed eligible, included in the study, completing follow-up, and analysed)<br>(b) Give reasons for non-participation at each stage.<br>(c) Consider use of a flow diagram	Page 6 & S Figure 1 & S Figure 1—Source Data 1	RECORD 13.1: Describe in detail the selection of the persons included in the study (*i.e.,* study population selection) including filtering based on data quality, data availability and linkage. The selection of included persons can be described in the text and/or by means of the study flow diagram.	Page 6:<br>"We also excluded individuals missing from the CHI database in 2016, but not recorded as dead (and therefore likely transferred out of Scotland; N = 573,711), individuals with record discrepancies between the CHI and National Records of Scotland databases (N = 79,131), and individuals with records outside of the study dates or missing information on socioeconomic class (N = 59,767),"
Descriptive data	14	(a) Give characteristics of study participants (*e.g.*, demographic, clinical, social) and information on exposures and potential confounders<br>(b) Indicate the number of participants with missing data for each variable of interest	Table 1, Table 1—Source Data 1, S Figure 1, S Figure 1—Source Data 1		

For peer review only

		(c) *Cohort study* - summarise follow-up time (*e.g.*, average and total amount)			
Outcome data	15	*Cohort study* - Report numbers of outcome events or summary measures over time<br>*Case-control study* - Report numbers in each exposure category, or summary measures of exposure<br>*Cross-sectional study* - Report numbers of outcome events or summary measures	Table 1, Table 1—Source Data 1		
Main results	16	(a) Give unadjusted estimates and, if applicable, confounder-adjusted estimates and their precision (e.g., 95% confidence interval). Make clear which confounders were adjusted for and why they were included<br>(b) Report category boundaries when continuous variables were categorized<br>(c) If relevant, consider translating estimates of relative risk into absolute risk for a meaningful time period	Page 9: "age-adjusted mortality rates compared to men (70.7%; 95% CI 70.3%–71.1%)"<br><br>N/A<br><br>Page 10: "68.0% higher for the most deprived decile (188,905 individuals with 331,701 first-time admissions)"		
Other analyses	17	Report other analyses done—e.g., analyses of subgroups and interactions, and sensitivity analyses	Page 12: "Across sex and deprivation strata"		
**Discussion**					
Key results	18	Summarise key results with reference to study objectives	Page 14:		

			"Cancers (C), cardiovascular disease (I), respiratory diseases (J), and unclassified symptoms and signs (R) were the principal ICD10 chapters recurring in the top 28 disease blocks associated with total death "<br><br>"we modelled a reduction in mortality hazard of 0.737 (95% CI 0.730–0.745) due to improvements in morbidity"		
Limitations	19	Discuss limitations of the study, taking into account sources of potential bias or imprecision. Discuss both direction and magnitude of any potential bias	Page 15:<br>"However, this study also has a number of limitations."	RECORD 19.1: Discuss the implications of using data that were not created or collected to answer the specific research question(s). Include discussion of misclassification bias, unmeasured confounding, missing data, and changing eligibility over time, as they pertain to the study being reported.	Page 15:<br>"By relying on routine data, we have been able to create a very large dataset, at the expense of being unable to externally check individual fields, such as primary diagnosis"<br><br>Page 16:<br>"a relative weakness is that we are reliant on hospital admission as a

For peer review only - http://bmjopen.bmj.com/site/about/guidelines.xhtml

					marker of incidence; therefore, diagnosed or latent (presumably milder) cases in the absence of admission are not visible to us”
Interpretation	20	Give a cautious overall interpretation of results considering objectives, limitations, multiplicity of analyses, results from similar studies, and other relevant evidence	Page 17:<br>“Much of the improvements in mortality observed in Scotland between 2001–2016 can be attributed to improved morbidity”<br><br>“coincide with a continued effort within Scotland, the UK, and other developed nations to improve prevention and care”<br><br>“global increases in antimicrobial resistance”		
Generalisability	21	Discuss the generalisability (external validity) of the study results	Page 17:<br>“While this study examined mortality and morbidity in the Scottish population only, there is a substantial concordance in		

For peer review only

			mortality trends across high-income countries, as well as similarities in disease-related mortality trends between Scotland and the rest of the UK[6], warranting similar studies to be performed in other high-income countries"		
**Other Information**					
Funding	22	Give the source of funding and the role of the funders for the present study and, if applicable, for the original study on which the present article is based	Page 18: "[Lloyds Banking Group] reviewed the design and said that adding stratification by socioeconomic status was a key requirement for meaningful analysis and their funding was conditional upon this, a request to which the authors readily agreed. The funder was kept informed of interim analyses and reviewed the draft manuscript, occasionally suggesting additional analyses or requesting clarifications. The		

For peer review only

			funders were not permitted to and did not request the removal of any results."		
Accessibility of protocol, raw data, and programming code		..		RECORD 22.1: Authors should provide information on how to access any supplemental information such as the study protocol, raw data, or programming code.	Page 18: "Individual de-identified participant data cannot be shared."

*Reference: Benchimol EI, Smeeth L, Guttmann A, Harron K, Moher D, Petersen I, Sørensen HT, von Elm E, Langan SM, the RECORD Working Committee. The REporting of studies Conducted using Observational Routinely-collected health Data (RECORD) Statement. *PLoS Medicine* 2015; in press.

*Checklist is protected under Creative Commons Attribution (CC BY) license.

For peer review only

BMJ Open

Trends in disease incidence and survival and their effect on mortality in Scotland: nationwide cohort study of linked hospital admission and death records 2001–2016

Journal:	*BMJ Open*
Manuscript ID	bmjopen-2019-034299.R1
Article Type:	Original research
Date Submitted by the Author:	05-Feb-2020
Complete List of Authors:	Timmers, Paul; The University of Edinburgh Usher Institute of Population Health Sciences and Informatics, Centre for Global Health Research<br>Kerssens, Joannes; NHS National Services Scotland, Information Services Division<br>Minton, Jon; NHS Health Scotland, Public Health Observatory<br>Grant, Ian; NHS National Services Scotland, Information Services Division<br>Wilson, James; The University of Edinburgh Usher Institute of Population Health Sciences and Informatics, Centre for Global Health Research; The University of Edinburgh MRC Institute of Genetics and Molecular Medicine, Human Genetics Unit<br>Campbell, Harry; The University of Edinburgh Usher Institute of Population Health Sciences and Informatics, Centre for Global Health Research<br>Fischbacher, Colin; NHS National Services Scotland, Information Services Division<br>Joshi, Peter; The University of Edinburgh Usher Institute of Population Health Sciences and Informatics, Centre for Global Health Research
**Primary Subject Heading**:	Public health
Secondary Subject Heading:	Public health, Epidemiology
Keywords:	EPIDEMIOLOGY, PUBLIC HEALTH, Public health < INFECTIOUS DISEASES

SCHOLARONE™
Manuscripts

I, the Submitting Author has the right to grant and does grant on behalf of all authors of the Work (as defined in the below author licence), an exclusive licence and/or a non-exclusive licence for contributions from authors who are: i) UK Crown employees; ii) where BMJ has agreed a CC-BY licence shall apply, and/or iii) in accordance with the terms applicable for US Federal Government officers or employees acting as part of their official duties; on a worldwide, perpetual, irrevocable, royalty-free basis to BMJ Publishing Group Ltd ("BMJ") its licensees and where the relevant Journal is co-owned by BMJ to the co-owners of the Journal, to publish the Work in this journal and any other BMJ products and to exploit all rights, as set out in our licence.

The Submitting Author accepts and understands that any supply made under these terms is made by BMJ to the Submitting Author unless you are acting as an employee on behalf of your employer or a postgraduate student of an affiliated institution which is paying any applicable article publishing charge ("APC") for Open Access articles. Where the Submitting Author wishes to make the Work available on an Open Access basis (and intends to pay the relevant APC), the terms of reuse of such Open Access shall be governed by a Creative Commons licence – details of these licences and which Creative Commons licence will apply to this Work are set out in our licence referred to above.

Other than as permitted in any relevant BMJ Author's Self Archiving Policies, I confirm this Work has not been accepted for publication elsewhere, is not being considered for publication elsewhere and does not duplicate material already published. I confirm all authors consent to publication of this Work and authorise the granting of this licence.

For peer review only

Trends in disease incidence and survival and their effect on mortality in Scotland: nationwide cohort study of linked hospital admission and death records 2001–2016

Paul RHJ Timmers[1], Joannes J Kerssens[2], Jon W Minton[3], Ian Grant[2], James F Wilson[1,4], Harry Campbell[1], Colin M Fischbacher[2], Peter K Joshi[1]

1 Centre for Global Health Research, Usher Institute of Population Health Sciences and Informatics, University of Edinburgh, Edinburgh, UK
2 Information Services Division, NHS National Services Scotland, Edinburgh, UK
3 Public Health Observatory, NHS Health Scotland, Edinburgh, UK
4 MRC Human Genetics Unit, Institute of Genetics and Molecular Medicine, University of Edinburgh, Edinburgh, UK

Corresponding author: Paul Timmers (paul.timmers@ed.ac.uk)

For peer review only

Abstract

Objectives: Identify causes and future trends underpinning Scottish mortality improvements and quantify the relative contributions of disease incidence and survival.

Design: Population-based study.

Setting: Linked secondary care and mortality records across Scotland.

Participants: 1,967,130 individuals born 1905–1965 and resident in Scotland 2001–2016.

Main outcome measures: Hospital admission rates and survival within five years post-admission for 28 diseases, stratified by sex and socioeconomic status.

Results: "Influenza and pneumonia", "Symptoms and signs involving circulatory and respiratory systems", and "Malignant neoplasm of respiratory and intrathoracic organs" were the hospital diagnosis groupings associated with most excess deaths, being both common and linked to high post-admission mortality. Using disease trends, we modelled a mean mortality hazard ratio of 0.737 (95% CI 0.730–0.745) from one decade of birth to the next, equivalent to a life extension of ~3 years per decade. This improvement was 61% (30%–93%) accounted for by improved disease survival after hospitalisation (principally cancer) with the remainder accounted for by lowered hospitalisation incidence (principally heart disease and cancer). In contrast, deteriorations in infectious disease incidence and survival increased mortality by 9% (~3.3 months per decade). Disease-driven mortality improvements were slightly greater for men than women (due to greater falls in disease incidence), and generally similar across socioeconomic deciles. We project mortality improvements will continue over the next decade but slow by 21% because much progress in disease survival has already been achieved.

Conclusion: Morbidity improvements broadly explain observed mortality improvements, with progress on prevention and treatment of heart disease and cancer contributing most. The male-female health gaps are closing, but those between socioeconomic groups are not. Slowing improvements in morbidity may explain recent stalling in improvements of UK period life expectancies. However, these could be offset if we accelerate improvements in the diseases accounting for most deaths and counteract recent deteriorations in infectious disease.

For peer review only - http://bmjopen.bmj.com/site/about/guidelines.xhtml

Strengths and limitations of this study

- The individual-level linkage of hospital and death records in this population-wide dataset allows for direct modelling of improvements in 28 disease categories in terms of improvements in disease incidence and subsequent survival, stratified by sex and socioeconomic status.

- Exclusion of migrating individuals means changes in disease are unaffected by population shifts, and allow for diseases to be compared with each other and summarised into trends in mortality based on morbidity.

- Hospital admission diagnosis and subsequent survival avoid issues with cause of death recording; however, they do not provide evidence of the causal effect of disease on mortality and may in some cases track changes in underlying frailty.

- This study is limited to the assessment of diseases which result in a hospital admission prior to death.

For peer review only

Introduction

In recent decades, there has been a substantial improvement in life expectancies at birth in the UK(1). More recently, several studies have suggested that there has been slowdown in improvements in the USA, UK, France, Germany, Sweden, the Netherlands and other Organisation for Economic Co-operation and Development countries; however, the causes are less clear, with speculation that they may arise from slowing improvements in cardiovascular disease, increased influenza mortality and/or pressure on health and social care services(1–8). Understanding trends in disease incidence and subsequent survival could illuminate such trends in mortality, and disentangling how and how much different diseases contribute has the potential to reveal whether investment in healthcare and research is directed at the most urgent diseases and most affected individuals.

Through its electronic Data Research and Innovation Service (eDRIS), Scotland has linkage of historical individual death and electronic health records in a controlled environment, with specific study approvals by the Public Benefit and Privacy Panel. This allows direct modelling at an individual level of the incidence of disease and subsequent death or survival of subjects. Furthermore, because historic records are available and the whole population is covered, a retrospective cohort study can be constructed (with inherent representativeness of the initial sample, with very complete levels of follow-up, and without survivor bias).

Here, we use population-wide data between 2001 and 2016 on residents of Scotland born before 1966 to explore how trends in longevity were driven by different trends in broad classes of disease incidence or survival, and highlight diseases which have shown more or less improvement in their contribution to overall mortality. We partition overall mortality by sex and socioeconomic status and, assuming past disease improvements continue to the same extent in the future, use these results to project future improvements in mortality and their changing sources.

For peer review only

Methods

All methods and results are reported in line with RECORD guidelines(9).

Data sources

We received ethical approval to access administration and care records from NHS National Services Scotland (NSS) from 2001 up to 2016. The final study population included all 1,967,130 individuals born between 1905 and 1965 who registered with the NSS, were resident in Scotland during the study period, and had complete and reliable records on their date of birth, socioeconomic status, and death (if applicable). Linkage and quality control of the data are described below.

Community Health Index dataset

Records were extracted from the historical and current Community Health Index (CHI) dataset. This is a register of all patients in NHS Scotland and is fed by eight regional databases (e.g, GP database, cancer screening). The register is considered complete from 2001 onwards. The CHI number, contained in the dataset, is effectively a patient identifier and added to other health datasets to make linkage possible, for instance between hospital admissions, death records and the Scottish cancer registry(10). Our extract consisted of 2,691,304 de-identified records, constituting the identified population of Scotland in 2001 who had been born between 1905 and 1965. The Scottish Index of Multiple Deprivation (SIMD)(11) was used to quantify socioeconomic status, determined by individuals' full postcode, and subsequently converted into deciles. The dataset we received contained only records with district-level postcodes and SIMD deciles, of which we excluded individuals with district codes with less than 5,000 individuals (thereby excluding anomalous postcodes, often with special meanings, such as "marketing campaign"; N = 11,564). We also excluded individuals missing from the CHI database in 2016, but not recorded as dead (and therefore likely transferred out of Scotland; N = 573,711), individuals with record discrepancies between the CHI and National Records of Scotland databases (N = 79,131), and individuals with records outside of the study dates or missing information on socioeconomic class (N = 59,767), giving 1,967,130 individuals for analysis after quality control (Supplementary File 1). Characteristics of the excluded individuals were similar to the rest of the population, except for postcode exclusions and database transfers, which were missing socioeconomic information and death records, respectively, as expected (Supplementary File 2).

National Registry of Scotland death records

We received 1,477,796 death records from the National Registry of Scotland (NRS) of all deaths occurring between 1990 and 2016, of which 699,093 could be matched to the CHI database before quality control. Unmatched records were usually for deaths occurring prior to the study start (2001). Of the matched records, 176,197 belonged to individuals that were excluded during CHI quality control, leaving 602,506 total deaths for analysis (Table 1).

Acute hospital admission

Health records were also linked to 30,054,191 acute hospital admissions, of which 17,264,379 were dated between 2001 and 2016 and could be matched (Supplementary File 3 & 4).

Disease classification

The main diagnosis of acute hospital admission records, excluding any secondary diagnoses, was used to classify records into disease categories, which corresponded to disease blocks as described in the chapters of the ICD10(12). In order to model the effect of disease incidence and avoid double counting of chronic conditions, we used only the first admission of a disease category for each individual, excluding subsequent visits to the hospital for diseases within the same category. The term "incidence"

For peer review only - http://bmjopen.bmj.com/site/about/guidelines.xhtml

is used throughout this study to refer to the first recorded hospital admission of any disease within the disease category during the study period.

Design

Mortality trends were modelled using morbidity trends: we first determined the major disease categories (ICD10 blocks) associated with the most lives lost by taking into account the frequency of the disease (as measured by hospitalisation) and its effect on survival (as measured by the subsequent all-cause mortality of patients admitted for the disease compared to the mortality of everyone else). The effect of disease incidence has previously been modelled based on one-, five-, or ten-year mortality(13); we chose five years as this captured the great part of excess mortality attributable to the incidence, rather than common underlying factors, although this does vary by disease (area under graphs in Supplementary File 5, in excess of asymptotic rates) while leaving a range of 10 years in our study to examine trends over time. We combined disease frequency and 5-year excess age-adjusted death rates to calculate a burden of death weighting for each disease block. We then looked at how the age-adjusted trends in hospitalisation rates (as a proxy for incidence) changed for each disease, by decade of birth, projecting that if incidence of a disease fell by a given percentage, its contribution to mortality would fall similarly. The use of a cohort model for the incidence of disease was driven by empirical investigation. Specifically, the distinctions we found by decade of birth in cancers, especially "Malignant neoplasm of respiratory and intrathoracic organs" (C30-C39) in Supplementary File 6 & 7, show a clear cohort effect. However, it should be recognised that the cohorts have only been observed over the study period (2001-2016). After calculating hospitalisation rates between decades of birth, we calculated their weighted average, reflecting the expected effect of all measured disease incidence changes on mortality rates, driven by decade of birth. Similarly, we looked at how the (age-adjusted) 5-year survival rates following first hospitalisation changed by year of hospitalisation. For each block this again gives a contribution towards reduced mortality, and the weighted average, the expected effect of changes in survival of the combined diseases on overall mortality. Adding these effects (and noting we assessed changes in survival from incidences over one decade), gives the expected effect on overall mortality from decade of birth to subsequent decade of birth from the effect of changes in disease incidence and survival, under the (necessarily simplified) model that incidence is a function of birth cohort and survival post incidence is a function of year of incidence.

Statistical analysis

Mortality

A Cox proportional hazards model using NRS mortality data – fitting sex, decade of birth, and deprivation – was used to quantify mortality in the Scottish population during the study period. The same analysis was run stratified by sex and deprivation. Unless otherwise stated, (for example median age differences in Kaplan-Meier curves), years of life of a hazard effect have been calculated by multiplying the $\log_e$ hazard ratio (lnHRl) by 10 (ref. 14). Only individuals with complete records were included in the analysis.

Morbidity

We grouped the main diagnoses of each NSS hospital admission into categories, as laid out by the ICD10 Chapters, and included only the first instance of admission for a category per individual (discarding subsequent repeat visits to hospital for a disease within the same disease category). Analysis was restricted to more common disease blocks. Visual inspection suggested a pragmatic threshold of at least 15,000 first-time admissions (see Supplementary File 8 for all disease categories meeting this threshold).

For peer review only - http://bmjopen.bmj.com/site/about/guidelines.xhtml

Effects on the incidence of hospitalisation for the more common disease blocks was quantified using Cox proportional hazard models based on age, with events defined as the first incidence of hospitalisation. We fitted sex, deprivation, and decade of birth as covariates. Again, the same analyses were performed stratifying by sex and deprivation.

In order to quantify all-cause mortality in the five years following hospitalisation, person-time of individuals was divided into phases, corresponding to the study start until hospitalisation, the first five years after hospitalisation, and the remaining time in the study. For example, an individual admitted to hospital in 2004 for ischaemic heart disease I20-25 (IHD) and surviving until 2010 would contribute three phases to the model: one for the period until hospitalisation (no event), one for the first 5 years after hospitalisation (no event), and one >5 years after hospitalisation (event after 1 year). The status of each phase was fitted as a covariate in a Cox proportional hazards model(15) with death as the event, adjusting for sex and deprivation:

$$h(x) = h_0(x)\, e^{\beta_1 X_1 \,+\, \beta_2 X_2 \,+\, \beta_3 X_3 \,+\, \beta_4 X_4} \quad (1)$$

Where h_0 is the baseline hazard, x the patient age, and X_1-X_4 the covariates sex, deprivation, and logically coded phase status (0-5 years True/False and >5 years True/False), with corresponding effect sizes β_1-β_4. This yielded estimates of the proportional hazard of status (0-5 and >5 years) after hospitalisation compared to pre-hospitalisation mortality. Thus, the baseline hazard is a function of age and the hazard ratios reflected the effects of the other covariates. The same model was run, stratified by sex and deprivation.

Burden

For disease blocks with at least 15,000 first admissions during the study period, the relative mortality burden of each disease block was calculated as the excess mortality in the 5 years after hospital admission (Equation 1) multiplied by the number of first-time admissions for the disease block, as follows:

$$N_{firstadmission}/N_{total} \, * \, h_{(0,5)} \quad (2)$$

Where $N_{firstadmission}$ is the total number of first hospital admissions of the disease category during the study period, N_{total} is the total number of individuals in the study, and $h_{(0,5)}$ is the mortality of individuals in the first five years following hospitalisation compared to individuals who were never hospitalised for the disease category, measured in $\log_e$ hazard ratios. The resulting value was then scaled to total 1 and provides a relative measure of the number of lives lost due to the diseases within the category, with higher values indicating a disease category with more common diseases or diseases associated with higher subsequent mortality, and lower values indicating a disease category with rare diseases or diseases associated with lower subsequent mortality. Whilst this measure may in principle be affected by differing age patterns on incidence, it was judged sufficient for our purpose – to establish broad relative weightings of the importance of each disease category.

To maintain a feasible computational burden within the national safe haven, subsequent analysis was restricted to the 25 blocks with the highest burden of death on the population (Table 2). We added C50-C50 malignant neoplasm of the breast, C60-C63, malignant neoplasms of male genital organs, and G30-G32 other diseases of the nervous system to this list, out of specific interest: in the sex-specific effects and awareness of the limitations of our method for Alzheimer's disease (see discussion). All further analyses were performed on these top 28 blocks (T-28). The use of (first) hospitalisation for a disease as our definition of incidence is imperfect (e.g. for Alzheimer's disease where hospitalisation following incidence is rare or delayed, and even first diagnosis in the community will often be preceded by a long latent period)(16).

For peer review only

Disease survival

Improvements in disease outcomes by ICD10 block were calculated by comparing 5-year all-cause mortality (Equation 1) following hospitalisation in 2001 with 5-year all-cause mortality following hospitalisation in 2011. As 5-year mortality estimates in 2011 had more uncertainty (due to fewer deaths in 2011–2016), we also calculated 5-year mortality following first-time hospitalisation for every year between 2001 and 2011 (i.e. mortality of patients admitted in those years), and used the trend in mortality over time to inform the 2011 estimate. To do so, we regressed the yearly mortality estimates against year of hospital admission, fitting a 3rd order polynomial to allow for non-linear relationships, and weighted the estimates by the inverse of their variances to account for uncertainty:

$y = \beta_1 x + \beta_2 x^2 + \beta_3 x^3 + \epsilon$ (3)

Where y is the 5-year mortality hazard after hospital admission in year x, and β_1,β_2,β_3 are the coefficients describing the relationship between y and x. We then used the value and standard error predicted for 2011 by the model as our estimate for 5-year all-cause mortality for hospitalisation in 2011.

Mortality estimates from morbidity

Estimates of the improvement in incidence of hospitalisation between decades of birth was combined into an overall improvement by performing a weighted sum of all diseases, with weights derived from the relative burden of death of each disease (see above).

These ten year improvements (I) due to incidence were added to the ten year improvements due to post incidence survival (S) to give a total improvement due to all morbidities, and proportions due to incidence/survival were calculated as the S or I / (I+S).

Ethical approval

This study was approved by the Public Benefit and Privacy Panel for Health and Social Care under application number 1617-0255/Joshi. As clinical records are provided without explicit patient consent, the panel requires the public benefit of the research to clearly outweigh any impact on individual patient privacy, and appropriate safeguards and security to be in place to protect patients. The panel granted access to de-identified patient data, accessible only through the National Safe Haven and only by University of Edinburgh researchers with data safeguarding qualifications. In addition, all results were reviewed by eDRIS before extraction from the safe haven to ensure no potentially identifiable information was made public.

Patient and public involvement

Patients and the public were not involved in the study or its design, beyond their contribution of health records. Due to the retrospective study design and anonymised nature of the records, it was not feasible to contact individual patients nor involve them in the dissemination of results.

Summary of outcomes

Mortality improvements

Age-adjusted falling mortality rates observed directly from NRS death records.

Disease burden of death

Prevalence of a disease category (total number of individuals admitted at least once 2001–2016), multiplied by the age-adjusted all-cause mortality within five years (in lnHR) after the first diagnosis of the disease category.

For peer review only

Disease weight

Disease burden of death, scaled 0-1, denoting the relative importance of a disease category.

Disease incidence improvement

Age-adjusted hazard of being admitted to hospital for a disease category (excluding subsequent hospital visits for the same disease category) from one decade of birth to the next.

Disease survival improvement

Age-adjusted hazard of dying within five years after the first hospital admission for a disease category in 2011 compared to having the first hospital admission in 2001.

Disease improvements

Linear combination of the disease survival and disease incidence (averaged across decades of birth) in units of lnHR.

Morbidity-driven mortality

The change in mortality rates expected from the improvements in morbidity (i.e. weighted sum of disease survival and incidence for all 28 diseases).

All model coefficients used in the results can be found in Supplementary File 9.

For peer review only

Results

Mortality

The population consisted of 1,967,130 Scottish individuals aged 35 years or older at the start of the study period (1 December 2000). 53.3% were female, 78.5% had been admitted to hospital at least once within the study period, and 30.6% died over the course of the study (31 January 2016). See Table 1 for detailed population characteristics.

		N			Age Entry			Age Exit			Hospital Visits		
Sex	Dead	Individuals	Admitted	Hospital Visits	Mean	Median	SD	Mean	Median	SD	Mean	Median	SD
Male	FALSE	633,953	429,659	2,315,915	50.9	49.4	10.3	65.6	64.3	9.8	3.7	2.0	6.8
Male	TRUE	283,835	283,835	2,742,686	68.0	69.3	11.7	75.6	77.1	11.4	9.7	7.0	11.4
Female	FALSE	730,671	511,632	2,764,040	52.7	51.1	11.6	67.4	66.1	10.8	3.8	2.0	7.0
Female	TRUE	318,671	318,671	2,895,443	72.1	73.8	12.0	79.7	81.7	11.5	9.1	6.0	11.0
Both	FALSE	1,364,624	941,291	5,079,955	51.9	50.3	11.1	66.6	65.3	10.4	3.7	2.0	6.9
Both	TRUE	602,506	602,506	5,638,129	70.1	71.7	12.0	77.8	79.5	11.6	9.4	6.0	11.2
Both	ALL	1,967,130	1,543,797	10,718,084	57.5	55.4	14.1	70.0	69.2	11.9	5.4	3.0	8.8

Table 1: Description of the data. The population included almost 2 million individuals (one-third of whom died during the study). See Supplementary File 3 for descriptives by deprivation including ICD10 codes. N – Number of records; Admitted – Individuals admitted to hospital at least once; Hospital Visits – All records of visiting the hospital; Age Entry – Age at the start of the study period (1 December 2000); Age Exit – Age at the end of the study period (31 January 2016) or at the end of life.

Quantifying mortality effects using Cox proportional hazard models, we observed statistically significant associations ($P < 1\times10^{-26}$) between mortality and sex, deprivation and decade of birth (Supplementary File 10). Women showed lower overall age-adjusted mortality rates compared to men (hazard ratio 0.71; 95% CI 0.70–0.71), corresponding to an expectation of life of 3.5 years longer than their male counterparts, whilst individuals from the most deprived areas (top decile) suffered mortality rates more than twice as severe (2.07; 95% CI 2.04–2.09) as those from the least deprived areas (bottom decile), corresponding to a difference in around 7 years of life. Median survival of men and women in the most deprived areas was 71.1 and 76.6 years, respectively, compared to 82.2 and 85.2 in the least deprived areas (Supplementary File 11). A wide gap between the most deprived decile and the adjacent one for men is apparent visually: the difference in median survival between deprivation deciles 1 to 9 is roughly constant (0.82/1.05 years per decile for women/men), but moving from the 9th to 10th deprivation decile has a greater effect, especially for men (1.99/2.67 years for women/men). Lastly, individuals born in the decade commencing 1935 had age-adjusted mortality rates 2.45 (95% CI 2.39–2.51) times those born three decades later, corresponding to a difference in life expectancy of around 9 years of life.

Morbidities and consequent mortality

Multiplying total number of hospitalisations during the study period (as a proxy for disease prevalence) by 5-year mortality after hospital admission (as a proxy for disease severity) provided a weight for the death burden of hospitalisation of each ICD10 block. We restricted our analyses to 28 of the top disease blocks for burden of death (T-28, see methods). Among the T-28, total cases of disease incidence (i.e. first-time admissions) during the study period ranged from 33,613 (A30-A49, "Other bacterial disease") to 225,504 (R00-R09, "Symptoms and signs involving the circulatory and respiratory systems") (Table 2). Per-person total cases of disease incidence (not age-adjusted) were 68.0% higher for the most deprived decile (188,905 individuals with 331,701 first-time admissions) compared to the least deprived decile (187,193 individuals with 195,617 first-time admissions). Between sexes, per-person incidence was 2.2% higher for men (917,788 individuals with 1,257,417 first-time admissions) compared to women (1,049,342 individuals with 1,407,223 first-time admissions) (Supplementary File 12).

For peer review only - http://bmjopen.bmj.com/site/about/guidelines.xhtml

In the first five years, the highest all-cause mortality rate was for patients admitted for C76-C80 (“Malignant neoplasms of ill defined, secondary and unspecified sites”; hazard ratio 26.1) compared to all-cause mortality rates for those not admitted for C76-C80. The lowest 5-year all-cause mortality rate was for those admitted for K20-K31 (“Diseases of oesophagus, stomach and duodenum”; hazard ratio 1.8) compared to mortality rates for those not admitted for K20-K31. Ordering diseases by their burden of death weights, we found “Influenza and pneumonia” (J09-J18), “Symptoms and signs involving circulatory and respiratory systems” (R00-R09), and “Malignant neoplasm of respiratory and intrathoracic organs” (C30-C39) were the disease categories responsible for the most death (Table 2), together accounting for 19% of the total death burden of the T-28 diseases.

For peer review only

ICD10	Disease grouping	Disease importance: Total hospital visits	5-year mortality (HR)	Relative weight	Average 10-year improvements HR (95% CI): Incidence	Survival	Combined	Survival to Incidence Ratio (SE)
J09-J18	Influenza and pneumonia	110,985	5.28	0.068	1.19 (1.11-1.27)	0.86 (0.80-0.92)	1.02 (0.92-1.12)	0.47 (0.13)
R00-R09	Symptoms and signs involving the circulatory and respiratory systems	225,504	2.08	0.061	0.88 (0.85-0.92)	0.79 (0.73-0.86)	0.70 (0.64-0.76)	0.65 (0.14)
C30-C39	Malignant neoplasm of respiratory and intrathoracic organs	54,178	21.12	0.061	0.83 (0.75-0.91)	0.81 (0.74-0.89)	0.67 (0.59-0.77)	0.52 (0.15)
R10-R19	Symptoms and signs involving the digestive system and abdomen	174,055	2.56	0.060	0.87 (0.83-0.91)	0.89 (0.82-0.97)	0.77 (0.70-0.85)	0.45 (0.19)
R50-R69	General symptoms and signs	157,357	2.67	0.057	0.90 (0.85-0.94)	0.97 (0.93-1.02)	0.87 (0.81-0.94)	0.19 (0.19)
C15-C26	Malignant neoplasms of digestive organs	71,981	8.13	0.056	0.79 (0.73-0.85)	0.67 (0.63-0.72)	0.53 (0.48-0.59)	0.63 (0.08)
I30-I52	Other forms of heart disease	142,898	2.70	0.052	0.78 (0.74-0.83)	0.84 (0.80-0.87)	0.66 (0.61-0.70)	0.43 (0.06)
C76-C80	Malignant neoplasms of ill defined, secondary and unspecified sites	39,339	26.13	0.047	0.84 (0.76-0.93)	0.69 (0.61-0.79)	0.58 (0.49-0.69)	0.68 (0.16)
Z40-Z54	Persons encountering health services for specific procedures and health care	157,841	2.15	0.044	1.00 (0.95-1.05)	0.74 (0.69-0.80)	0.74 (0.68-0.81)	1.00 (0.19)
I60-I69	Cerebrovascular diseases	100,907	3.06	0.042	0.79 (0.74-0.85)	0.82 (0.76-0.87)	0.65 (0.59-0.71)	0.46 (0.09)
K55-K63	Other diseases of intestines	206,178	1.72	0.041	0.94 (0.91-0.98)	0.80 (0.77-0.84)	0.76 (0.71-0.80)	0.80 (0.12)
J40-J47	Chronic lower respiratory diseases	78,467	3.99	0.040	0.78 (0.73-0.84)	0.79 (0.72-0.86)	0.62 (0.55-0.69)	0.49 (0.11)
I20-I25	Ischaemic heart diseases	175,605	1.83	0.039	0.65 (0.63-0.68)	0.77 (0.72-0.82)	0.50 (0.46-0.55)	0.38 (0.05)
N30-N39	Other diseases of the urinary system	126,329	2.31	0.039	1.04 (0.99-1.10)	1.24 (1.15-1.34)	1.29 (1.17-1.42)	0.84 (0.22)
K20-K31	Diseases of oesophagus, stomach and duodenum	172,206	1.83	0.038	0.72 (0.69-0.75)	0.88 (0.79-0.96)	0.63 (0.56-0.70)	0.29 (0.11)
J20-J22	Other acute lower respiratory infections	77,520	3.57	0.036	1.11 (1.03-1.20)	1.11 (1.06-1.17)	1.24 (1.13-1.36)	0.50 (0.15)
S70-S79	Injuries to the hip and thigh	78,231	2.64	0.028	0.87 (0.78-0.96)	1.02 (0.98-1.06)	0.89 (0.79-0.99)	0.12 (0.14)
A30-A49	Other bacterial diseases	33,613	6.60	0.023	1.56 (1.39-1.74)	0.94 (0.77-1.15)	1.46 (1.16-1.84)	0.13 (0.21)
T80-T88	Complications of surgical and medical care, not elsewhere classified	75,217	2.32	0.023	1.04 (0.97-1.11)	0.83 (0.74-0.94)	0.86 (0.75-0.99)	0.84 (0.40)
N17-N19	Renal failure	37,213	5.14	0.022	1.02 (0.91-1.15)	0.82 (0.73-0.91)	0.83 (0.71-0.98)	0.90 (0.42)

For peer review only

For peer review only - http://bmjopen.bmj.com/site/about/guidelines.xhtml

I80-I89	Diseases of veins, lymphatic vessels and lymph nodes, not elsewhere classified	84,073	1.94	0.021	0.80 (0.75-0.85)	0.79 (0.72-0.86)	0.63 (0.56-0.71)	0.52 (0.12)
K90-K93	Other diseases of the digestive system	47,091	2.98	0.019	0.98 (0.90-1.07)	0.83 (0.72-0.95)	0.82 (0.69-0.96)	0.91 (0.50)
I70-I79	Diseases of arteries, arterioles and capillaries	47,410	2.95	0.019	0.67 (0.61-0.74)	0.91 (0.85-0.99)	0.61 (0.54-0.69)	0.19 (0.08)
K50-K52	Non infective enteritis and colitis	59,183	2.27	0.018	0.73 (0.68-0.79)	0.89 (0.84-0.95)	0.65 (0.59-0.72)	0.27 (0.08)
S00-S09	Injuries to the head	64,925	2.09	0.018	0.95 (0.88-1.02)	0.98 (0.90-1.08)	0.93 (0.82-1.05)	0.22 (0.72)
C50-C50	Malignant neoplasm of breast	39,358	3.21	0.017	0.81 (0.74-0.89)	0.31 (0.27-0.36)	0.25 (0.21-0.30)	0.85 (0.07)
C60-C63	Malignant neoplasms of male genital organs	22,312	3.23	0.010	0.91 (0.79-1.05)	0.50 (0.44-0.57)	0.45 (0.37-0.55)	0.88 (0.14)
G30-G32	Other degenerative diseases of the central nervous system	4,655	3.29	0.002	0.75 (0.51-1.10)	0.96 (0.73-1.28)	0.78 (0.49-1.24)	0.11 (0.44)
TOTAL		2,664,631	2.77	1.000	0.89 (0.88-0.90)	0.83 (0.76-0.90)	0.74 (0.72-0.75)	0.61 (0.16)

Table 2: Relative mortality burden of hospital admission by disease grouping and improvements in hospitalisation incidence and survival. ICD10 – Diseases contained within the disease grouping, coded by International Classification of Disease Codes, Tenth Revision. See Supplementary File 4 for counts of 3-letter ICD10 records within each ICD10 block. Total hospital visits – Number of first-time admissions with main diagnosis falling within the disease block. 5-year mortality – Mortality within the first five years after admission compared to individuals who had not yet or ever been admitted for the disease group. Relative weight – Relative burden of death as a function of hospital admissions and 5-year mortality, scaled to [0-1]. Incidence – Average hazard ratio of being admitted to hospital for each subsequent decade of birth. Survival – All-cause mortality hazard ratio after being admitted for the disease in 2011 compared to 2001. Combined – linear combination of changes in disease incidence and survival. 95% confidence intervals are listed in parentheses. Ratio – the ratio of changes in disease survival to incidence of hospital admission. Standard error is listed in parentheses. See Supplementary File 12 for these data by sex and deprivation. See Supplementary File 8 for the relative burdens of all disease groupings with more than 15,000 first-time hospital admissions.

For peer review only

Apart from sex-specific cancers, we observe significant differences in burden of death between men and women for injuries to the hip and thigh (S70-S79) and head (S00-S09), with the former having a higher burden in women due to more female cases and the latter having a higher burden in men due to more male cases. For both disease blocks, the effect of hospitalisation on subsequent mortality is greater in men than women (S70-S79 hazard ratio men: 3.19, women: 2.44; S00-S09 hazard ratio men: 2.32, women: 1.88). Strikingly, 5-year mortality after hospital admission for IHD is higher for women (hazard ratio 2.01/1.70 women/men), but this is offset by the lower prevalence of hospitalisation in women (Supplementary File 12).

Trends in disease

To understand changes in disease survival rates, we next modelled the effects of a disease on all-cause mortality by year of hospital admission for admissions between 2001 and 2011 and 5-year survival subsequent to admission. We find an overall improvement over time in patient survival following hospitalisation, with a median decline between 2001 and 2011 in the 5-year hazard ratio of 16.8% for admitted cases across the T-28 diseases. The biggest improvements were for malignant neoplasms of the breast (C50) and male genital organs (C60-C63), which have seen 68.7% (95% CI 64.1%–72.7%) and 50.2% (95% CI 42.9%–56.6%) declines in the 5-year hazard ratio between 2001 and 2011 mortality, respectively. On the other hand, "Other acute lower respiratory infections" (J20-J22) and "Other diseases of the urinary system" (N30-N39) have seen increases in mortality hazard of 11.3% (95% CI 7.0%–15.7%) and 24.0% (95% CI 14.6%–34.1%), respectively (Table 2; Supplementary File 13 & 14).

We next modelled age-adjusted incidence of hospitalisation for a disease by birth decade, under the simplified model that incidence is a cohort rather than period effect – essentially modelling that current incidence is the effect of (previous) lifetime exposures, rather than current exposures. We find disease incidence has fallen decade on decade of birth for cancers, cardiovascular, and intestinal diseases, but this improvement appears to have slowed down in the last decade of birth (1955-1965) considered. Age-adjusted incidence of "Influenza and pneumonia" (J09-J18) and "Other bacterial diseases" (A30-A49) has worsened by decade on decade of birth, over the whole range of births considered (Supplementary File 6 & 7).

When taking both trends in incidence and survival into account – adding 1) the average age-adjusted incidence rate reductions between decade on decade of birth to 2) the 2001-2011 reductions in 5-year disease mortality (Supplementary File 15 & 16) – we observe the death burden of cancers is declining most (Figure 1). Notably, breast and prostate cancers have seen the largest improvement of all disease categories in the last decade. "Other diseases of the urinary system" (N30-N39), "Other bacterial diseases" (A30-A49), "Other acute lower respiratory infections" (J20-J22), and "Influenza and pneumonia" (J09-J18) have all seen increases in their effect on age-adjusted all-cause mortality.

Overall, we see broad consistency in the scale of improvements across decades of birth, except for "Malignant neoplasms of respiratory and thoracic organs" (C30-C39), where we see greater decade-on-decade improvements amongst later decades (Figure 2). Averaging these individual disease effects on death, using burden of death weightings, we can then compare the modelled death rates with those observed, and see broad correspondence, with the 1935 and 1945 decades, showing the greatest improvements. Overall, our morbidity model suggests individuals from each successive decade of birth experience an average mortality rate of 0.74 (gaining ~3 years of life) compared to the previous decade of birth (Table 2).

For peer review only - http://bmjopen.bmj.com/site/about/guidelines.xhtml

The shape of these disease-modelled mortality improvements by decade of birth broadly track the observed changes (Figure 2). This is especially apparent when stratifying the improvements by sex: Supplementary File 17 shows a reasonable relationship between the projected morbidity driven mortality and observed mortality (i.e. mortality trends in the study can largely be explained by trends in disease incidence and survival). Across sex and deprivation strata, taking into account disease survival improvements between 2001 and 2011 and all improvements in disease incidence between decades of birth, we find the largest reductions in death are due to improvements in "Ischaemic heart diseases" (I20-I25), "Malignant neoplasms of digestive organs" (C15-C26), and "Malignant neoplasm of respiratory and intrathoracic organs" (C30-C39), while the largest increases in death are due to "Other bacterial diseases" (A30-A49) and "Influenza and pneumonia" (J09-J18) (Supplementary File 18). In addition, the deterioration in "Other diseases of the urinary system" (N30-N39) morbidity shows a consistent increase with deprivation, while "Other diseases of the digestive system" (K90-K93) shows consistently larger improvements in more deprived classes (Supplementary File 12; Supplementary File 19).

Overall, we estimate 61.2% (95% CI 29.9%–92.6%) of the improvement in mortality rates was due to improvements in survival following hospital admission, with the balance arising from reduced (age-adjusted) admission rates (Table 2). Improved outcomes for cancers (C) were particularly driven by post-admission survival, especially C60-C63 (88% of mortality improvement attributable to survival rather than incidence), C50 (85%) and C76-80 (68%), whereas for cardiovascular diseases (I) the balance was more even, as seen in I80-89 (52%), I60-I69 (46%), I30-I32 (43%), I20-I25 (38%).

As previously noted, disease severity was defined as the log hazard ratio for subsequent all-cause mortality among those with a previous admission for an index group of conditions compared with those with no such admission. We regarded the rate of improvement in disease severity over time as being constant if there was the same relative fall in log hazard rate over successive time periods (so for example we regarded a fall in lnHR from 0.6 to 0.3 as equivalent to a fall from 0.3 to 0.15). Assuming the improvements in survival following hospitalisation continue for the coming decade, and differences between incidence in birth cohorts remains the same, we project a 21% slowing of improvements in mortality (-0.242 lnHR c.f. -0.305 lnHR; Table 3). Essentially, at least arithmetically, the population mortality benefits from improved cancer treatments in 2001-2011 will be hard to repeat as so much benefit has already accrued. Admittedly, this is a consequence of our model: essentially judging it equally difficult to reduce 50 excess deaths following cancer hospital admission associated to 25, as it was to reduce from 100 to 50, and as such should be considered speculative. On the other hand – our model is clearly valid *in extremis*: if all excess cancer deaths were eliminated, no further cancer driven improvement in mortality would be possible.

		Current Improvements			Projected Improvements		
Stratified	Group	Hospital Admission Rate (lnHR)	Five Year Mortality After Admission (lnHR)	Combined (lnHR)	Hospital Admission Rate (lnHR)	Five Year Mortality After Admission (lnHR)	Combined (lnHR)
None		-0.1182	-0.1866	-0.3047	-0.1182	-0.1235	-0.2418
Sex	M	-0.1428	-0.1913	-0.3340	-0.1428	-0.1273	-0.2701
Sex	F	-0.0971	-0.1823	-0.2794	-0.0971	-0.1130	-0.2101
SIMD	1	-0.1154	-0.2204	-0.3360	-0.1154	-0.1280	-0.2433
SIMD	2	-0.1157	-0.1592	-0.2743	-0.1157	-0.0456	-0.1610

For peer review only - http://bmjopen.bmj.com/site/about/guidelines.xhtml

SIMD	3	-0.1202	-0.1186	-0.2392	-0.1202	-0.0370	-0.1572
SIMD	4	-0.1418	-0.1759	-0.3201	-0.1418	-0.0541	-0.1982
SIMD	5	-0.1231	-0.1731	-0.2958	-0.1231	-0.0914	-0.2145
SIMD	6	-0.1305	-0.1928	-0.3227	-0.1305	-0.0998	-0.2303
SIMD	7	-0.1233	-0.1787	-0.3044	-0.1233	-0.1109	-0.2343
SIMD	8	-0.1305	-0.2097	-0.3402	-0.1305	-0.1422	-0.2726
SIMD	9	-0.0926	-0.1619	-0.2546	-0.0926	-0.1033	-0.1959
SIMD	10	-0.0950	-0.1970	-0.2920	-0.0950	-0.1092	-0.2043

Table 3: Mean (over birth decades) decade of birth on decade of birth improvements in morbidity for the study period, and projections into the subsequent decade by sex and deprivation. Mortality improvements were estimated from morbidity records by combining the mean improvement in hospitalisation rate across birth cohorts and the improvement in disease severity between 2001 and 2011. This was then projected forward assuming improvements in age-adjusted hospitalisation rate between birth cohorts remained constant and improvements in severity remained proportional to the (now reduced) overall mortality of the disease group.

For peer review only

For peer review only - http://bmjopen.bmj.com/site/about/guidelines.xhtml

Discussion

In a study of 1,967,120 lives and 10,718,084 hospital admissions, we observed a median age at death of 82.2/85.2 for men/women in the highest socioeconomic decile, and 11.1/8.6 years less for the lowest decile. Cancers (C), cardiovascular disease (I), respiratory diseases (J), and unclassified symptoms and signs (R) were the principal ICD10 chapters recurring in the top 28 disease blocks where hospital admission was associated with the greatest subsequent all-cause mortality, which was a product of the rate of first hospital admission with group of conditions and of all-cause mortality in the five years following admission with that condition. Specifically, our top five causes of hospitalisations associated with subsequent burden of all-cause deaths were, in descending order, 'Influenza and pneumonia' (more common and with higher subsequent mortality than the average T-28 disease), 'Symptoms and signs involving the circulatory and respiratory systems' (common), 'Malignant neoplasm of respiratory and intrathoracic organs' (higher mortality) ,'Symptoms and signs involving the digestive system and abdomen' (common), and 'General symptoms and signs' (common and higher mortality). Whilst the latter might appear a benign diagnosis, our results suggest it is a fairly strong and frequent marker of subsequent all-cause mortality.

Across decades of birth, we modelled a reduction in mortality hazard of 0.737 (95% CI 0.730–0.745) due to improvements in morbidity, which broadly tracked improvements in observed mortality. The modelled improvement was 61% accounted for by reduction in excess mortality subsequent to admission and 39% accounted for by a fall in incidences of disease (as measured by hospital admission rates). The important (i.e. burden-of-death weighted) improvements in incidence were driven by cancers and heart disease, whilst improvement in outcomes following admission were mostly driven by cancer, particularly breast and prostate cancer. In contrast, we found deteriorations in the incidence of bacterial disease and in mortality following admission for respiratory and urinary infections. Levels of morbidity and mortality varied strongly across socioeconomic groups, but patterns in **changes** of such were generally less apparent. Men showed greater rates of improvement in mortality and morbidity than women, with lung and throat cancers contributing most to male improvements and IHD contributing most to female improvements.

In conclusion, we find trends in morbidity appear to partly explain trends in mortality. The progress in prevention and cure within oncology and prevention of heart disease account for the greatest parts of mortality improvement in 2001-2016, and our model suggests mortality improvements may slow, simply because the absolute effect of progress in treatment of these diseases will be difficult to repeat. However, there is scope for further improvements in life expectancy, especially if new progress is made in the treatment of other diseases associated with death, or if prevention initiatives accelerate.

Strengths and weaknesses of the study

This study has avoided some of the known issues with cause of death recording(17) since it does not use cause-specific mortality and tracks wider disease effects and subsequent mortality (such as frailty) beyond direct causes of death, by combining hospitalisation and death records. Implicit tracking of underlying causes through an associated effect (admission to hospital for a disease) may improve estimates of trends in mortality, even if the underlying cause is obscure. We are also able to partition trends in deaths due to a disease based on trends in prevalence and incidence, which has been done for IHD(18), but not simultaneously across diseases in the same dataset. Also, our results are unaffected by population shifts as we excluded immigrants into Scotland after 2001, and instead reflect trends within 1the defined groups. Combined with the scale of our data, this consistent tracking has enabled us to

For peer review only - http://bmjopen.bmj.com/site/about/guidelines.xhtml

make like-for-like comparisons of the mortality outcomes of different disease classes across socioeconomic groups and their trends over time.

However, this study also has a number of limitations, relating to the population under study, the definition of diseases, considerations with hospital admission data, and modelling assumptions. Further discussion of these limitations can be found in Supplementary File 20.

In brief, we excluded migrants out of Scotland because their subsequent trajectory (especially death) could not be tracked. Migrants may be healthier than the average individual and excluding them could therefore overestimate the incidence of disease and death in the population we studied. However, our observed trends should remain unaffected if migration patterns did not change significantly during the study.

Secondly, for practical reasons we grouped the main diagnoses of hospital admissions by ICD-10 chapters and excluded any secondary diagnoses. As a result, we are not able to comment on the trends of individual diseases within chapters (which could offset each other) nor the trends or effects of comorbidities. The latter may affect our results if comorbidities have changed over time or by socioeconomic status; for example, a decline in lung cancer over time as a competing risk for heart disease would inflate the observed improvements for heart disease. However, this is likely to be partially mitigated by reductions in mortality for individuals not admitted for heart disease. Future work may account for comorbidities more explicitly by using competing risk regression and site-specific survival.

Thirdly, the first hospital admission on record and its date is only a proxy for incidence of severe disease. Excluding subsequent hospital admissions may understate the burden of diseases which have recurring episodes (such as influenza), although trends in these diseases are unlikely to be affected given our definitions remained constant. Conversely, diseases such as dementia and multiple sclerosis which are generally managed in the community are unlikely to result in an (immediate) admission to hospital and are therefore not captured accurately in our study. Examining trends in these chronic diseases through GP records was outside the scope of this study, but integration of our results with future work on GP records is likely to refine overall morbidity estimates.

Another consideration with hospital records is their indirect link to death. This relationship can be confounded on the one hand by other health risks and lifestyle factors, and on the other hand by coding inaccuracies and changes in admission policies and screening. Our stratified analysis by sex and socioeconomic status partially mitigates the former, and coding inaccuracies are unlikely to affect disease trends if these inaccuracies are stable over time. There is evidence that screening policies and hospital usage has changed during the study period, but their influence is limited and the opposite effects on disease incidence and survival will mostly offset each other when looking at the effect on morbidity (e.g. influenza) (Supplementary File 20). However, some caution is needed when interpreting the exact split between improvements in disease incidence and survival.

Lastly, our model assumed 1) disease incidence is a function of year of birth, 2) survival after hospital admission is a function of year of incidence, and 3) these hazards are proportionate. The first two assumptions are a simplification, but necessary given year of birth and year of incidence are completely confounded for a given age at incidence. As to the third point, while disease status itself is not always strictly a proportional hazard, trends in incidence hazard ratios between birth decades and survival hazard ratios between years of hospital admission should still be captured appropriately (Supplementary File 20).

For peer review only - http://bmjopen.bmj.com/site/about/guidelines.xhtml

Strengths and weaknesses in relation to other studies

There was a degree of correspondence in the principal burdens assessed here and a recent study by the Scottish Burden of Disease study (SBD)(19). This study used the same population and the same study period but assessed YLL (weighting young deaths more as opposed to our method which counted all deaths equally), included individuals younger than 35 years old, and used different disease groupings. Their principal burdens were IHD (ranked 13th in our list of burdens), tracheal, bronchus and lung cancers (3rd), chronic obstructive pulmonary disease (12th), stroke (10th), and Alzheimer's disease (–). Aside from Alzheimer's disease, discussed below, much of the distinction appears to arise from our observation of an association between death and admissions with indistinct diagnosis (not considered a valid specific cause of death by SBD). In the case of influenza and pneumonia, differences arise due to our study identifying a marker of frailty as well as a direct cause of death, combined with SBD grouping influenza and pneumonia under lower respiratory infections. A relative strength of our study stems from usage of incident morbidity (as marked by hospitalisation) in advance of death, based on recorded diagnosis at the time of hospital visit, thus tracking remote effects such as long term frailty rather than cause of death (which has known limited accuracy, particularly at older ages(17)). However, the principal strength arises from the ability to distinguish trends in incidence of morbidity from trends in subsequent survival. On the other hand, a relative weakness is that we are reliant on hospital admission as a marker of incidence; therefore, diagnosed or latent (presumably milder) cases in the absence of admission are not visible to us, leading for example to significant discrepancy with SBD in the apparent relative burden of Alzheimer's disease, likely due to an understatement of its importance in our results.

The closing gap in mortality between the sexes and its widening across social classes observed in our study is consistent with recent findings from the Office of National Statistics, summarised by Torjesen(20), which looked at socioeconomic deprivation in England and Wales. Similarly, a recent study of health inequality in England found rising levels of lifespan inequality across socioeconomic groupings arising from increasing inequalities across a broad span of causes of death(21). These studies had the advantage of a larger sample size (~7.5 million deaths cf. 600,000 in our study) and could therefore track trends in mortality and cause of death between stratified groups more accurately. However, Scotland's unique linkage of death records and electronic health records through eDRIS allowed us to directly model changes disease mortality at an individual level (avoiding issues with cause of death recordings and shifts in population demographics). Our study has the advantage of partly explaining these trends in mortality inequality through changes in disease incidence and survival: men experienced greater improvements in incidence of lung cancer and survival following heart disease hospitalisation compared to women, while more socially deprived individuals (men and women combined) suffered worse deteriorations in infectious disease, especially for the incidence and survival of hospitalisation for urinary tract infections. However, in contrast to Bennet *et al.*(21), we do not find a clear pattern in overall morbidity improvements across socioeconomic deciles in Scotland, and we do not observe a widening inequality in cancer, respiratory and Alzheimer's disease morbidity within our study population, although we are underpowered to detect the latter and our disease groupings were not identical.

Lastly, a recent study of coronary heart disease mortality in Scotland, using a sophisticated model to apportion improvement between prevention and treatment, found improvements for coronary heart disease between 2001-2010 were similar across social classes, and reported 33%–61% of these improvements could be attributed to advances in treatment(18). Given the very different methods, albeit studying the same population, there is reasonable concordance with our own study: we find roughly equal improvements in heart disease across social classes and estimate 38% (95% CI 28%–48%) of these improvements stem from increased survival after hospitalisation for ischaemic heart disease.

For peer review only - http://bmjopen.bmj.com/site/about/guidelines.xhtml

Hotchkiss *et al.*(18) are able to further partition improvements by uptake of primary and secondary prevention drugs and treatments. Such detailed analysis of specific diseases has been beyond the scope of our study.

Implications for clinicians and policymakers

Much of the improvements in mortality observed in Scotland between 2001–2016 can be attributed to reductions in morbidity, as captured by hospital admissions. While this study examined mortality and morbidity in the Scottish population only, there is a substantial concordance in mortality trends across high-income countries(7), as well as similarities in disease-related mortality trends between Scotland and the rest of the UK(6), warranting similar studies to be performed in other high-income countries. It is a testament to healthcare services that the majority of mortality improvements appear to stem from advances in disease survival post-admission. Observed improvements in cancer incidence and survival – especially breast and prostate – coincide with a continued effort within Scotland(22), the UK(23), and other high-income nations(24) to improve prevention and care of these diseases. However, the rapid advances in survival of both heart disease and cancer modelled by our study between 2001 and 2011 will be hard to continue to the same extent, as so much progress has already been made. At the same time, the observed deteriorations in infectious disease coincide with global increases in antimicrobial resistance(25) and emphasise the need to prioritise research in this area: infectious disease will become a larger contributor to mortality and may contribute to a widening of health inequalities between socioeconomic classes. If these current trends in morbidity continue, we expect morbidity-driven improvements in mortality to slow down. However, the life expectancy gap between Scotland and other high-income countries(26) suggests further mortality improvements are possible. The rate of this improvement will hinge upon whether advances in all major diseases categories – especially infectious disease – can catch up with the progress we have recently seen on heart disease and cancer, and whether preventable deaths from external causes (such as suicide and drug-related deaths), which cannot be accurately tracked using hospital admissions, decrease rather than rise.

Funding

The study was funded by the Lloyds Banking Group, for the creation, curation and dissemination of knowledge in the public interest, in particular to improve estimates of future population size and morbidity and mortality rates to facilitate healthcare and other government planning. All analyses stratified by socioeconomic deprivation (i.e. Table 2, Table 3, Supplementary Files 7, 9–12, 14, 16, 18, and 19) were confidentially re-analysed using 10 socioeconomic groups specified by Lloyds, rather than the Scottish Index of Multiple Deprivation. No Lloyds employees were granted access to individual patient data. Data access was granted to University of Edinburgh researchers only and only through the national safe haven by the Public Benefit and Privacy Panel for Health and Social Care under application number 1617-0255/Joshi.

The study and its design were conceived by the last author. The funder reviewed the design and said that adding stratification by socioeconomic status was a key requirement for meaningful analysis and their funding was conditional upon this, a request to which the authors readily agreed. The funder was kept informed of interim analyses and reviewed the draft manuscript, occasionally suggesting additional analyses or requesting clarifications. The funders were not permitted to and did not request the removal of any results.

For peer review only - http://bmjopen.bmj.com/site/about/guidelines.xhtml

The last author and authors affiliated with eDRIS and NHS Scotland were compensated by the University of Edinburgh using the grant from Lloyds Banking Group, in line with normal pricing for the work undertaken. PRHJT was funded by the MRC Doctoral Training Programme (MR/N013166/1) and the University of Edinburgh College of Medicine and Veterinary Medicine. JFW was funded by the MRC QTL in health and disease. Apart from Lloyds Banking Group, the funders had no role in the study design, data collection, analysis, and interpretation, or the decision to submit the work for publication.

Acknowledgements

We would like to thank Lloyds for the funding and in particular Craig Butler and Stuart McDonald for their engagement with the project. We would also like to thank Steve Pavis and Doug Kidd for their guidance with the Public Benefit and Privacy Panel for Health and Social Care.

Competing interests

PKJ reports grants from Lloyds Banking Group, PLC, during the conduct of the study. This grant was awarded to the University of Edinburgh and used in part to pay for research costs of JJK and CMF. PKJ also reports shares in Lloyds Banking Group, PLC, as part of a diversified portfolio. The remaining authors declare no competing interests.

Author contributions

PRHJT—Formal Analysis, Software, Visualisation, Writing - Original Draft, Writing - Review & Editing. JJK—Data Curation, Writing - Original Draft. JWM – Writing - Review & Editing. IG—Writing - Review & Editing. JFW—Supervision, Writing - Review & Editing. HC—Writing - Review & Editing. CMF—Conceptualization, Writing - Review & Editing. PKJ—Conceptualization, Formal Analysis, Funding Acquisition, Investigation, Methodology, Project Administration, Software, Supervision, Validation, Writing - Original Draft, Writing - Review & Editing.

Data sharing

Individual de-identified participant data cannot be shared. Statistical code and technical details are available upon request from the corresponding author.

For peer review only - http://bmjopen.bmj.com/site/about/guidelines.xhtml

References

1. Sanders S, UK Office for National Statistics. National life tables, UK: 2015 to 2017. Off Natl Stat Stat Bull [Internet]. 2018 [cited 2019 Jul 9];1–11. Available from: https://www.ons.gov.uk/peoplepopulationandcommunity/birthsdeathsandmarriages/lifeexpectancies/bulletins/nationallifetablesunitedkingdom/2015to2017
2. Lafortune, Gaetan; Balestat G. OECD Health Working Papers No.26. Trends Sev Disabil Among Elder People. 2007;(26):81.
3. Hiam L, Dorling D, Harrison D, McKee M. What caused the spike in mortality in England and Wales in January 2015? J R Soc Med. 2017;110(4):131–7.
4. Office for National Statistics. Changing trends in mortality in England and Wales:1990 to 2017 (Experimental Statistics). 2018;2017(June):1–24. Available from: https://www.ons.gov.uk/peoplepopulationandcommunity/birthsdeathsandmarriages/deaths/articles/changingtrendsinmortalityinenglandandwales1990to2017/experimentalstatistics
5. Dyer O. US life expectancy falls for third year in a row. BMJ [Internet]. 2018;363(December):k5118. Available from: http://dx.doi.org/doi:10.1136/bmj.k5118
6. Palin J. Mortality improvements in decline. The Actuary [Internet]. 2017 Aug; Available from: https://www.theactuary.com/features/2017/08/mortality-improvements-in-decline/
7. Fenton L, Minton J, Ramsay J, Kaye-Bardgett M, Fischbacher C, Wyper GM, et al. Recent adverse mortality trends in Scotland: comparison with other high-income countries. bioRxiv [Internet]. 2019;542449. Available from: https://www.biorxiv.org/content/10.1101/542449v1
8. Fenton L, Wyper G, McCartney G, Minton J. Socioeconomic inequality in recent adverse mortality trends in Scotland. bioRxiv [Internet]. 2019;542472. Available from: https://www.biorxiv.org/content/10.1101/542472v1.abstract%0Ahttps://www.biorxiv.org/content/biorxiv/early/2019/02/07/542472.full.pdf
9. Nie X, Peng X. The reporting of studies conducted using observational routinely-collected health data (RECORD) statement. Chinese J Evidence-Based Med. 2017;17(4):475–87.
10. Fleming M, Kirby B, Penny KI. Record linkage in Scotland and its applications to health research. J Clin Nurs. 2012;21(19–20):2711–21.
11. The Scottish Government. SIMD16 Technical Notes. 2016; Available from: http://www.gov.scot/Resource/0050/00504822.pdf
12. World Health Organisation. International Statistical Classification of Diseases and Related Health Problems Tenth Revision Volume 2. World Heal Organ. 2004;
13. Quaresma M, Coleman MP, Rachet B. 40-year trends in an index of survival for all cancers combined and survival adjusted for age and sex for each cancer in England and Wales, 1971-2011: A population-based study. Lancet [Internet]. 2015 Mar 28 [cited 2020 Jan 23];385(9974):1206–18. Available from: http://www.ncbi.nlm.nih.gov/pubmed/25479696
14. Joshi PK, Pirastu N, Kentistou KA, Fischer K, Hofer E, Schraut KE, et al. Genome-wide meta-analysis associates HLA-DQA1/DRB1 and LPA and lifestyle factors with human longevity. Nat Commun [Internet]. 2017 Dec 13 [cited 2019 Dec 5];8(1):910. Available from: http://www.nature.com/articles/s41467-017-00934-5
15. Cox DR. Regression Models and Life-Tables. J R Stat Soc Ser B Stat Methodol. 1972;34:187.
16. Duboisa B, Padovanib A, Scheltensc P, Rossid A, Agnello GD. Timely diagnosis for alzheimer's disease: A literature review on benefits and challenges. J Alzheimer's Dis. 2015;49(3):617–31.
17. Smith Sehdev AE, Hutchins GM. Problems with proper completion and accuracy of the COD statement. Arch Intern Med. 2001;161(2):277–84.
18. Hotchkiss JW, Davies CA, Dundas R, Hawkins N, Jhund PS, Scholes S, et al. Explaining trends in Scottish coronary heart disease mortality between 2000 and 2010 using IMPACTSEC model: Retrospective analysis using routine data. BMJ [Internet]. 2014;348(February):1–15. Available from: http://dx.doi.org/doi:10.1136/bmj.g1088
19. Mesalles-Naranjo O, Grant I, Wyper GMA, Stockton D, Dobbie R, McFadden M, et al. Trends and inequalities in the burden of mortality in Scotland 2000–2015. PLoS One. 2018;13(8):1–17.
20. Torjesen I. Inequalities in life expectancy are widening, data confirm. Bmj [Internet]. 2018;1017(March):k1017. Available from: http://dx.doi.org/doi:10.1136/bmj.k1017

For peer review only - http://bmjopen.bmj.com/site/about/guidelines.xhtml

21. Bennett JE, Pearson-Stuttard J, Kontis V, Capewell S, Wolfe I, Ezzati M. Contributions of diseases and injuries to widening life expectancy inequalities in England from 2001 to 2016: a population-based analysis of vital registration data. Lancet Public Heal [Internet]. 2018;3(12):e586–97. Available from: http://dx.doi.org/10.1016/S2468-2667(18)30214-7
22. NHS Scotland. Better Cancer Care: An Action Plan [Internet]. The Scottish Government. Edinburgh; 2008. Available from: http://www.gov.scot/resource/doc/242498/0067458.pdf
23. Department of Health. Improving Outcomes: A Strategy for Cancer. 2011;(January):101. Available from: https://www.gov.uk/government/uploads/system/uploads/attachment_data/file/213785/dh_123394.pdf
24. Harbeck N, Gnant M. Breast cancer. In: Lancet (London, England) [Internet]. Elsevier; 2017 [cited 2019 Jul 17]. p. 1134–50. Available from: http://www.ncbi.nlm.nih.gov/pubmed/27865536
25. O'Neill J. The Review on Antimicrobial Resistance [Internet]. Vol. 33, Wellcome Trust & UK Government. 2016. Available from: https://amr-review.org
26. Whyte B. Scottish mortality in a European Context 1950 – 2000. An analysis of comparative mortality trends [Internet]. Heart Disease. 2006 [cited 2019 Jul 27]. Available from: https://www.scotpho.org.uk/publications/reports-and-papers/scottish-mortality-in-a-european-context-1950-2000-an-analysis-of-comparative-mortality-trends/

For peer review only - http://bmjopen.bmj.com/site/about/guidelines.xhtml

List of Tables and Figures

Figure 1: Modelled decade-of-birth upon previous decade-of-birth hospitalisations and survival show large improvements in cancer survival and heart disease incidence but deteriorations in infectious disease. Bars represent the mean improvements in hospital admission rate across decades of birth (darker bars), added to changes from 2001 to 2011 in 5-year survival rates following hospital admission (lighter bars). Both measures are expressed in age-adjusted. For definitions of each ICD10 block, see Table 2. Width of the bars represents the relative burden of death of each disease based on total first-time hospital admissions and 5-year mortality; as such, the total area of each bar represents the relative contribution to improvements – or deteriorations – in population mortality. Error bars are standard errors of the Cox model coefficient. G30–G32 had too few hospital admissions to accurately model improvements (Survival: lnHR 0.04, SE 0.14; Hospital admission lnHR 0.29, SE 0.20). Z40-Z54 only showed improvements in survival.

Figure 2: Modelled decade-of-birth upon previous decade-of-birth mortality reductions due to morbidity changes broadly track observed trends in mortality. Panels represent the combined improvements in hospital admission rate and 5-year mortality rates following hospital admission, expressed in age-adjusted lnHR and split by decade of birth - under the model where change in incidence of disease is modelled by decade of birth and added to the survival effect is the change in subsequent 5 year survival rates from incidences in 2001 and 2011. A) Improvements for each ICD10 disease block (for definitions see Table 2). Dots here represent the relative contribution of the disease to the overall improvements in morbidity-driven mortality, with larger dots indicating a greater contribution to morbidity improvements. A red circle around the dot indicates a negative contribution (i.e. deterioration). B) Modelled trend in deaths based on the weighted morbidities from the panels above. Diseases have been ordered by their burden of death (Table 2), so smaller bars in early panels may have similar effect on the grey bar average (indicated by the dot size) as larger bars in later panels. C) Observed trend in actual deaths from death records, by decade of birth, for comparison. See Supplementary File 18 for this graph stratified by sex and deprivation.

Table 1: Description of the data. The population included almost 2 million individuals (one-third of whom died during the study). Over 90% of deceased individuals had a hospital admission record. Median projected age at death (under Kaplan-Meier curves) for males is 71.1 and 82.2, in the most and least deprived areas, respectively (females 76.6 and 85.2). See Supplementary File 3 for descriptives by deprivation including ICD10 codes. N – Number of records; Admitted – Individuals admitted to hospital at least once; Hospital Visits – All records of visiting the hospital; Age Entry – Age at the start of the study period (1 December 2000); Age Exit – Age at the end of the study period (31 January 2016) or at the end of life.

Table 2: Relative mortality burden of hospital admission by disease grouping and improvements in hospitalisation incidence and survival. ICD10 – Diseases contained within the disease grouping, coded by International Classification of Disease Codes, Tenth Revision. See Supplementary File 4 for counts of 3-letter ICD10 records within each ICD10 block. Total hospital visits – Number of first-time admissions with main diagnosis falling within the disease block. 5-year mortality – Mortality within the first five years after admission compared to individuals who had not yet or ever been admitted for the disease group. Relative weight – Relative burden of death as a function of hospital admissions and 5-year mortality, scaled to [0-1]. Incidence – Average hazard ratio of being admitted to hospital for each subsequent decade of birth. Survival – All-cause mortality hazard ratio after being admitted for the

For peer review only - http://bmjopen.bmj.com/site/about/guidelines.xhtml

disease in 2011 compared to 2001. Combined – linear combination of changes in disease incidence and survival. 95% confidence intervals are listed in parentheses. Ratio – the ratio of changes in disease survival to incidence of hospital admission. Standard error is listed in parentheses. See Supplementary File 11 for these data by sex and deprivation. See Supplementary File 8 for the relative burdens of all disease groupings with more than 15,000 first-time hospital admissions.

Table 3: Mean (over birth decades) decade of birth on decade of birth improvements in morbidity for the study period, and projections into the subsequent decade by sex and deprivation. Mortality improvements were estimated from morbidity records by combining the mean improvement in hospitalisation rate across birth cohorts and the improvement in disease severity between 2001 and 2011. This was then projected forward assuming improvements in age-adjusted hospitalisation rate between birth cohorts remained constant and improvements in severity remained proportional to the (now reduced) overall mortality of the disease group.

For peer review only

For peer review only - http://bmjopen.bmj.com/site/about/guidelines.xhtml

Supplementary Material

Supplementary File 1: Waterfall chart of individuals excluded from the study during quality control (PDF). We started with ~2.7 million primary care records from the NSS Community Health Index (CHI). Records were excluded from individuals with uncommon district postcodes (N < 5000), individuals missing from the CHI database in 2016 (and therefore likely transferred out of Scotland), individuals with record discrepancies between the CHI and National Records of Scotland databases, and individuals with records outside of the study dates or missing information on socioeconomic class.

Supplementary File 2: Details of quality control performed on the dataset (Excel Document). Primary care records from the National Health Services Scotland underwent multiple rounds of quality control. For each step, descriptive measures of the excluded individuals are provided: birth date and death date in years, and socioeconomic deprivation in deciles. Unusual postcode – District-level postcodes shared between fewer than 5,000 individuals (anomalous postcodes, often with special meanings, such as "marketing campaign"). Transferred out of Scotland – Individuals absent from NSS records at the end of the study period, suggesting transfer out of Scotland or otherwise poor tracking. CHI – Community Health Index. NRS – National Records of Scotland.

Supplementary File 3: Study descriptives by sex, deprivation, and deceased status (Excel Document). SIMD – Scottish Index of Multiple Deprivation; Age Entry – Age at the start of the study period (1 December 2000); Age Exit – Age at the end of study period (31 January 2016) or at the end of life; Admissions – Records of hospital admission, where Count represents the number of individuals with at least one hospital admission record, Mean/Median/SD represent the mean/median/standard deviation of number of hospital admission records per individual, and Sum represents the total number of admission records during the study period across all individuals; A-Z – First letter of the ICD10 main condition recorded at all hospital visits; SD – Standard Deviation

Supplementary File 4: Count of records within each ICD10 block (Excel Document). Main diagnoses of hospital admission records were grouped into ICD 10 chapter. For each ICD 10 chapter, counts of the 3-letter ICD 10 subcategories with 10 or more admissions are reported.

Supplementary File 5: Long-term trends in mortality after being admitted to hospital by disease grouping (PDF). For each individual, we identified the date at first hospital admission for a disease group in the study period and compared mortality in the following years with mortality of those who had never been admitted. Age was fitted as a covariate. Plotted here are the 1, 3, and 6 month and annual mortality rates following admission.

Supplementary File 6: Age-adjusted reductions in hospital admission rate by decade of birth (PDF). Bars represent the improvements in hospital admission rate since the previous decade of birth, expressed in age-adjusted lnHR. A) Improvements for each ICD10 disease block (for definitions see Table 2). Dots here represent the relative contribution of the disease category to the overall (i.e. burden of death weighted) improvements in mortality due to changing hospital admission rates, with larger dots indicating a greater contribution. A red circle around the dot indicates a negative contribution (i.e. deterioration). B) Combined overall improvements, modelling the effect of morbidity incidence on mortality. Diseases have been ordered by their burden of death (Table 2), so smaller bars in early panels may have similar effect on the grey bar average (indicated by the dot size) as larger bars in later panels.

For peer review only - http://bmjopen.bmj.com/site/about/guidelines.xhtml

C) Observed trend in the incidence of death from death records, by decade of birth, for comparison. See Supplementary File 7 for this graph stratified by sex and deprivation.

Supplementary File 7: Age-adjusted reductions in hospital admission rate by decade of birth, stratified by sex and deprivation (PDF).

Supplementary File 8: Relative mortality burden of all disease groupings with at least 15,000 first-time admissions (Excel Document). ICD10 – Diseases contained within the disease grouping, coded by International Classification of Disease Codes, Tenth Revision. See (Supplementary File 4) for counts of 3-letter ICD10 records within each ICD10 block. Disease – Description of the diseases within the disease grouping. Hospital visits – Number of first-time admissions with main diagnosis falling within the disease block. Mortality – Mortality compared to individuals who had not yet or ever been admitted for the disease group. Relative Burden of Death – Ratio of hospital admissions to total individuals, multiplied by the mortality in the first five years after admission. Weight - burden of death rescaled to a total value of 1.

Supplementary File 9: Improvements in incidence of hospitalisation and 5-year mortality following hospitalisation by sex and deprivation (Excel Document). Contains improvements in hospital admission rate, disease survival, and combined impact on mortality for each disease grouping, stratified by sex and deprivation.

Supplementary File 10: Kaplan-Meier survival curves show ~10 year differences in median survival between top and bottom deprivation deciles (PDF). Empirical survival curves were calculated from 1,967,130 individuals and 647,752 mortality records between 2001–2016, with median survival annotated for the least and most deprived deciles. The accompanying table quantifies the relative mortality of the sexes, birth cohorts, and deprivation deciles in log hazard ratios, with SIMD 1 males from the 1965 birth cohort as reference. See Supplementary File 11 for numerical details of the Kaplan-Meier curves including median survival of each decile.

Supplementary File 11: Kaplan-Meier survival curves show ~10 year differences in median survival between top and bottom deprivation deciles (Excel Document). SIMD – Scottish Index for Multiple Deprivation decile; Records – Total number of individuals in the decile; N Max – Total number of uncensored individuals during the study period; Events – Number of individuals who died during the study period; Mean – Mean survival time; SE – Standard error of the mean survival time; Median – median survival time (i.e. number of years 50% of individuals survive); 95% CI – 95% confidence interval around the median survival time (years).

Supplementary File 12: Disease mortality burden by sex and deprivation (Excel Document). Contains sheets for 1) total incident cases, 2) mortality in the first 5 years following disease admission, 3) mortality after 5 years following disease admission, 4) the ratio of 0-5 and >5 year mortalities, 5) the relative burden of disease, and 6) the weight of each disease, by sex and deprivation. SIMD – Decile of Scottish Index of Multiple Deprivation, from least deprived (1) to most deprived (10).

Supplementary File 13: Age-adjusted reductions in excess mortality following hospital admission between 2001–2011 (PDF). Dots represent excess mortality in the first five years after hospital admission compared non-admissions, compared to the excess mortality in 2001, expressed in age-adjusted lnHR. Fitted lines are fixed intercept 3rd power polynomials, weighted by inverse variance of

For peer review only - http://bmjopen.bmj.com/site/about/guidelines.xhtml

estimates. The polynomial fitted value at 2011 with its standard error are used in morbidity driven mortality improvement models (see also Supplementary File 15). See Supplementary File 14 for this graph stratified by sex and deprivation.

Supplementary File 14: Age-adjusted reductions in excess mortality following hospital admission between 2001–2011, stratified by sex and deprivation (PDF).

Supplementary File 15: Age-adjusted improvements in survival following hospitalisation (PDF). Bars represent the age-adjusted lnHR of survival for admissions in 2011 compared with 2001. A) Improvements for each ICD10 disease block (for definitions see Table 2). Dots represent the relative contribution of the disease category to the overall (i.e. the burden of death weighted) improvements in mortality due to changing survival following hospitalisation, with larger dots indicating a greater contribution. A red circle around the dot indicates a negative contribution (i.e. deterioration). B) Combined overall improvements, modelling the effect of morbidity survival on mortality 2001 vs 2011. Diseases have been ordered by their burden of death (Table 2), so smaller bars in early panels may have similar effect on the grey bar average (indicated by the dot size) as larger bars in later panels. C) As is obvious, there is no improvement in survival after the incidence of death. See Supplementary File 16 for this graph stratified by sex and deprivation.

Supplementary File 16: Age-adjusted increases in survival following hospitalisation, stratified by sex and deprivation (PDF).

Supplementary File 17: Improvements in mortality projected from changes in disease morbidity partially explain observed improvements in mortality (PDF). Improvements in morbidity, based on reductions in hospitalisation rate and 5-year mortality, were calculated for individuals stratified by sex and decade of birth. The dotted line represents perfect concordance between projected and observed mortality improvements, with points above this line indicating mortality improvements have been greater than projected from disease improvements, and points below this line indicating the opposite. The solid line is the regression of projected mortality on observed mortality and includes all shown data points, weighted by their uncertainty in both directions. Annotated are the regression slope (standard error), correlation, variance explained, and significance. Concordance between projected and observed mortality appears to increase with later decades of birth. M – Male; F – Female; 1 – improvements of the cohort born in the decade after 1915 compared with the cohort born in the decade before; 2 – 1925; 3 – 1935; 4 – 1945; 5 – 1955.

Supplementary File 18: Total Improvements in mortality and morbidity across all birth decades by sex and deprivation (Excel Document). Mortality improvements were estimated from morbidity records by combining the improvement in hospitalisation rate for the 1955–1965 cohort compared to the 1905–1915 cohort and the improvement in disease severity between 2001 and 2011. Actual mortality improvements of the 1955–1965 cohort vs. 1905–1915 cohort was calculated directly from mortality records. SIMD – Scottish Index of Multiple Deprivation. Lives Saved – Disease groups which have benefited the most and least from the improvements in morbidity, by taking into account the burden of death and its improvement.

Supplementary File 19: Age-adjusted reductions in combined hospital admission rate and survival following hospitalisation by decade of birth, stratified by sex and deprivation (PDF).

For peer review only - http://bmjopen.bmj.com/site/about/guidelines.xhtml

Supplementary File 20: Extended discussion (PDF).

For peer review only

For peer review only - http://bmjopen.bmj.com/site/about/guidelines.xhtml

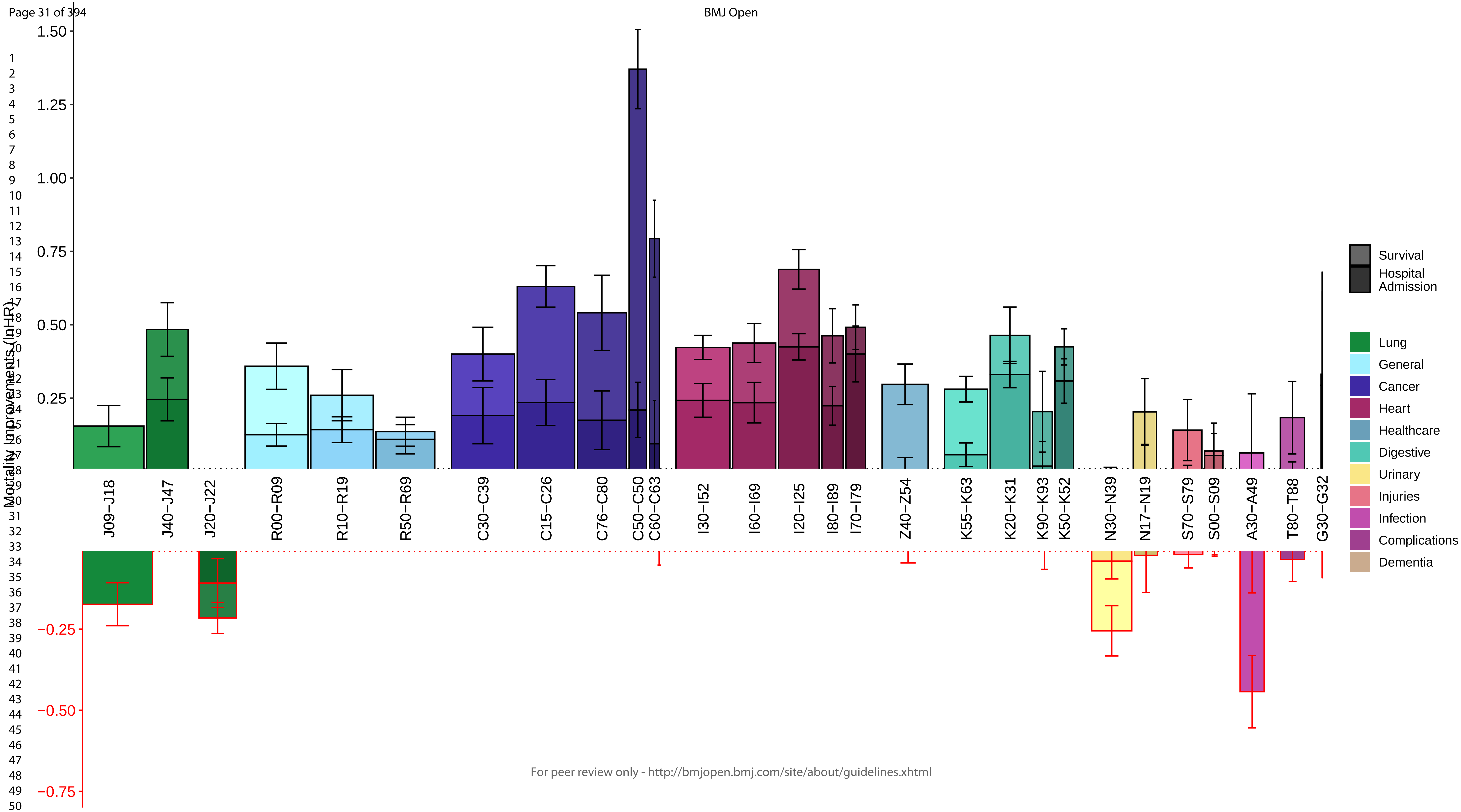
Mortality Improvements (lnHR)
1.50
1.25
1.00
0.75
0.50
0.25
−0.25
−0.50
−0.75
J09−J18
J40−J47
J20−J22
R00−R09
R10−R19
R50−R69
C30−C39
C15−C26
C76−C80
C50−C50
C60−C63
I30−I52
I60−I69
I20−I25
I80−I89
I70−I79
Z40−Z54
K55−K63
K20−K31
K90−K93
K50−K52
N30−N39
N17−N19
S70−S79
S00−S09
A30−A49
T80−T88
G30−G32
Survival
Hospital Admission
Lung
General
Cancer
Heart
Healthcare
Digestive
Urinary
Injuries
Infection
Complications
Dementia

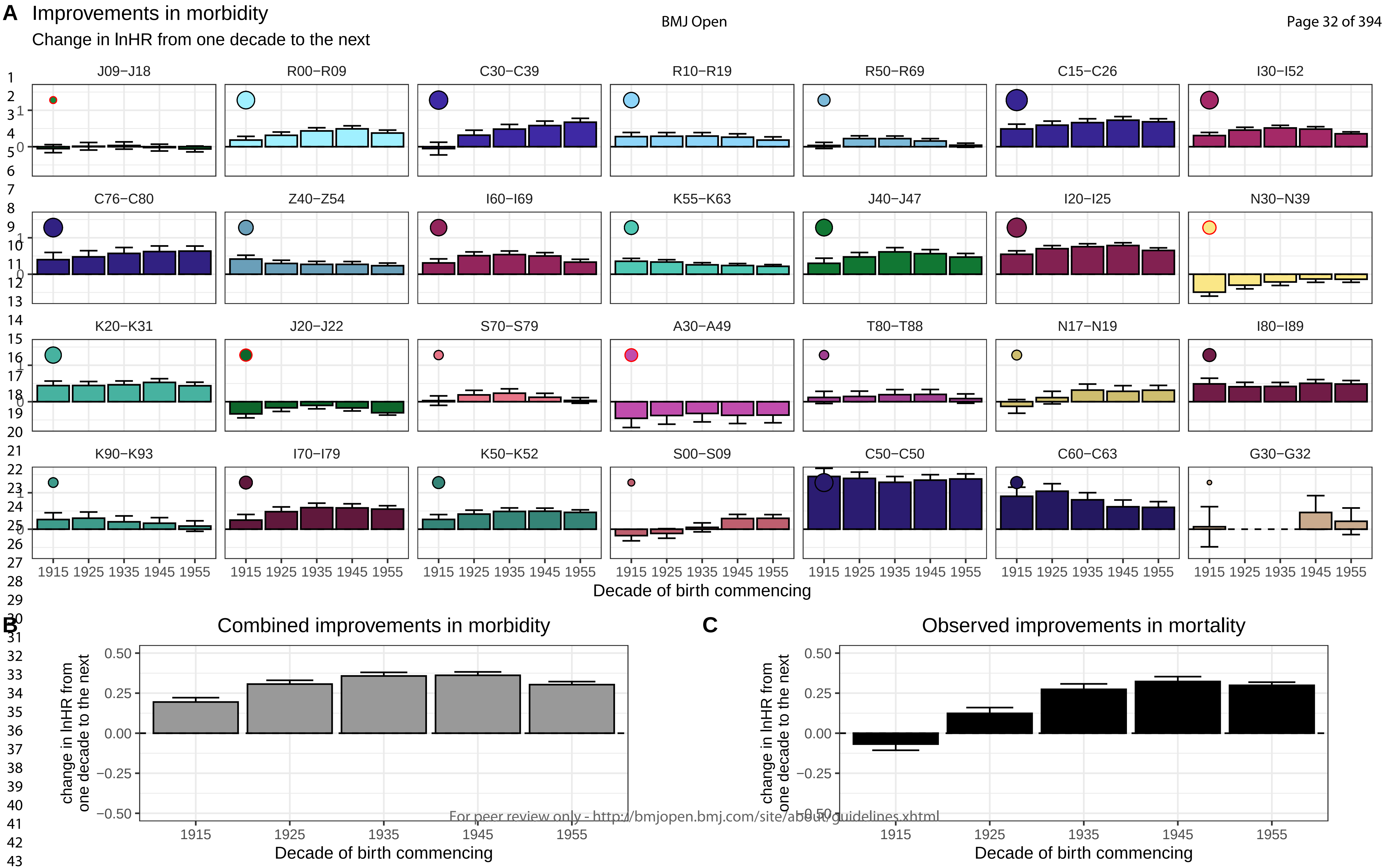
A
Improvements in morbidity
Change in lnHR from one decade to the next
J09−J18
R00−R09
C30−C39
R10−R19
R50−R69
C15−C26
I30−I52
C76−C80
Z40−Z54
I60−I69
K55−K63
J40−J47
I20−I25
N30−N39
K20−K31
J20−J22
S70−S79
A30−A49
T80−T88
N17−N19
I80−I89
K90−K93
I70−I79
K50−K52
S00−S09
C50−C50
C60−C63
G30−G32
0
1
1915 1925 1935 1945 1955
Decade of birth commencing
B
Combined improvements in morbidity
change in lnHR from one decade to the next
0.50
0.25
0.00
−0.25
−0.50
1915
1925
1935
1945
1955
Decade of birth commencing
C
Observed improvements in mortality
change in lnHR from one decade to the next
0.50
0.25
0.00
−0.25
−0.50
1915
1925
1935
1945
1955
Decade of birth commencing

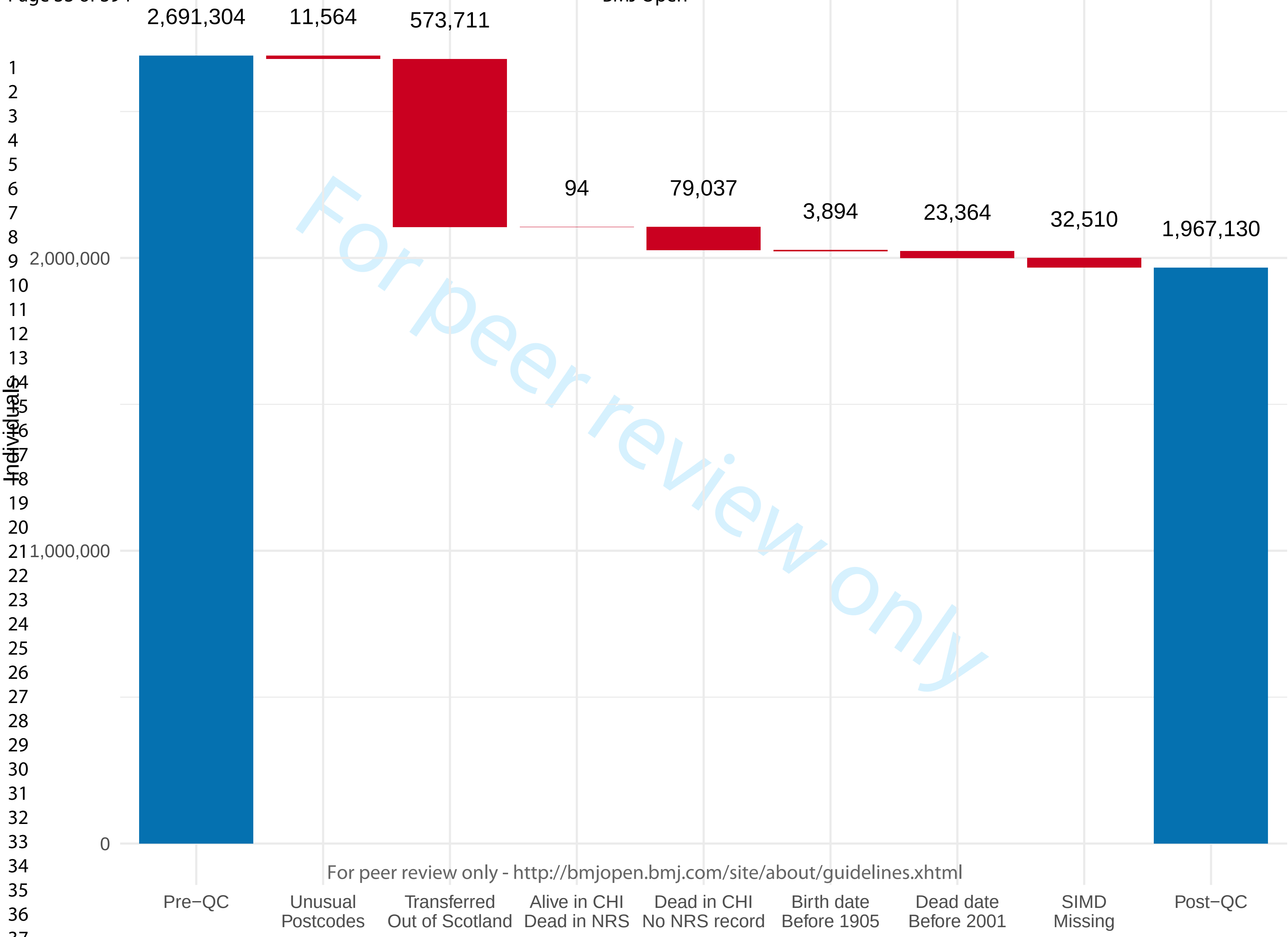

For peer review only

For peer review only - http://bmjopen.bmj.com/site/about/guidelines.xhtml

Description	Descriptive	Male			
		N	Median	Q1	Q3
Unusual Postcode	Birth Date	6,189	1941	1925	1953
	Dead Date	233	2003	2001	2009
	SIMD	162	4	2	7
Transferred out of Scotland	Birth Date	268,506	1952	1940	1959
	Dead Date	491	1998	1994	2003
	SIMD	225,372	5	2	8
Alive in CHI but Dead in NRS	Birth Date	50	1943	1933	1949
	Dead Date	50	2005	1997	2014
	SIMD	47	5	3	8
Dead in CHI but no NRS record	Birth Date	38,657	1938	1931	1947
	Dead Date	0	NA	NA	NA
	SIMD	37,570	6	3	8
Birth Date before 1905	Birth Date	526	1904	1902	1905
	Dead Date	526	2002	2001	2003
	SIMD	514	5	3	7
Dead Date before 2001	Birth Date	11,141	1926	1919	1935
	Dead Date	11,141	2001	2001	2001
	SIMD	10,897	7	4	9
Post QC	Birth Date	930,952	1947	1935	1956
	Dead Date	316,490	2008	2004	2012
	SIMD	917,788	6	3	8

For peer review only

Female			
N	Median	Q1	Q3
5,375	1938	1921	1953
296	2002	2002	2007
172	4	2	7
305,205	1952	1938	1959
599	1997	1992	2002
257,719	5	2	8
44	1933	1929	1944
44	2006	1997	2015
44	5	3	7
40,380	1934	1927	1942
0	NA	NA	NA
39,268	6	3	8
3,368	1903	1902	1904
3,368	2002	2001	2004
3,285	5	3	7
12,223	1920	1914	1929
12,223	2001	2001	2001
11,900	6	4	9
1,068,688	1944	1931	1955
354,499	2008	2004	2012
1,049,342	6	3	8

For peer review only

Statistic	Stratum			Individuals			Admissions
	Sex	SIMD	Dead	Age Entry	Age Exit	Deaths	
Count	Male	1	FALSE	67057	67057	0	42873
Count	Male	1	TRUE	19773	19773	19773	19773
Count	Male	2	FALSE	68578	68578	0	45638
Count	Male	2	TRUE	20904	20904	20904	20904
Count	Male	3	FALSE	65778	65778	0	43758
Count	Male	3	TRUE	23315	23315	23315	23315
Count	Male	4	FALSE	66278	66278	0	44121
Count	Male	4	TRUE	25194	25194	25194	25194
Count	Male	5	FALSE	64481	64481	0	43801
Count	Male	5	TRUE	27744	27744	27744	27744
Count	Male	6	FALSE	62624	62624	0	42915
Count	Male	6	TRUE	28861	28861	28861	28861
Count	Male	7	FALSE	62320	62320	0	43099
Count	Male	7	TRUE	31493	31493	31493	31493
Count	Male	8	FALSE	61306	61306	0	42554
Count	Male	8	TRUE	33924	33924	33924	33924
Count	Male	9	FALSE	58922	58922	0	41091
Count	Male	9	TRUE	35176	35176	35176	35176
Count	Male	10	FALSE	53644	53644	0	37873
Count	Male	10	TRUE	35580	35580	35580	35580
Count	Male	ALL	FALSE	630988	630988	0	427723
Count	Male	ALL	TRUE	281964	281964	281964	281964
Count	Female	1	FALSE	77585	77585	0	50045
Count	Female	1	TRUE	22778	22778	22778	22778
Count	Female	2	FALSE	78528	78528	0	52875
Count	Female	2	TRUE	23969	23969	23969	23969
Count	Female	3	FALSE	75176	75176	0	50644
Count	Female	3	TRUE	27550	27550	27550	27550
Count	Female	4	FALSE	74392	74392	0	50628
Count	Female	4	TRUE	29002	29002	29002	29002
Count	Female	5	FALSE	73595	73595	0	51276
Count	Female	5	TRUE	31315	31315	31315	31315
Count	Female	6	FALSE	72764	72764	0	51705
Count	Female	6	TRUE	31769	31769	31769	31769
Count	Female	7	FALSE	73363	73363	0	52755
Count	Female	7	TRUE	35711	35711	35711	35711
Count	Female	8	FALSE	72232	72232	0	52514
Count	Female	8	TRUE	37975	37975	37975	37975
Count	Female	9	FALSE	68635	68635	0	50778
Count	Female	9	TRUE	39108	39108	39108	39108
Count	Female	10	FALSE	61711	61711	0	46441
Count	Female	10	TRUE	37970	37970	37970	37970
Count	Female	ALL	FALSE	727981	727981	0	509661
Count	Female	ALL	TRUE	317147	317147	317147	317147
Count	ALL	1	FALSE	144642	144642	0	92918
Count	ALL	1	TRUE	42551	42551	42551	42551
Count	ALL	2	FALSE	147106	147106	0	98513
Count	ALL	2	TRUE	44873	44873	44873	44873

For peer review only

Count	ALL	3	FALSE	140954	140954	0	94402
Count	ALL	3	TRUE	50865	50865	50865	50865
Count	ALL	4	FALSE	140670	140670	0	94749
Count	ALL	4	TRUE	54196	54196	54196	54196
Count	ALL	5	FALSE	138076	138076	0	95077
Count	ALL	5	TRUE	59059	59059	59059	59059
Count	ALL	6	FALSE	135388	135388	0	94620
Count	ALL	6	TRUE	60630	60630	60630	60630
Count	ALL	7	FALSE	135683	135683	0	95854
Count	ALL	7	TRUE	67204	67204	67204	67204
Count	ALL	8	FALSE	133538	133538	0	95068
Count	ALL	8	TRUE	71899	71899	71899	71899
Count	ALL	9	FALSE	127557	127557	0	91869
Count	ALL	9	TRUE	74284	74284	74284	74284
Count	ALL	10	FALSE	115355	115355	0	84314
Count	ALL	10	TRUE	73550	73550	73550	73550
Count	ALL	ALL	FALSE	1358969	1358969	0	937384
Count	ALL	ALL	TRUE	599111	599111	599111	599111
Count	ALL	ALL	ALL	1958080	1958080	599111	1536495
Mean	Male	1	FALSE	51.42597	66.28053	0	3.022682196
Mean	Male	1	TRUE	70.28643	78.18031	1	9.638395792
Mean	Male	2	FALSE	51.03837	65.89842	0	3.247674181
Mean	Male	2	TRUE	69.47959	77.38318	1	9.913796403
Mean	Male	3	FALSE	51.20473	65.95515	0	3.275213597
Mean	Male	3	TRUE	69.67985	77.50238	1	9.254685825
Mean	Male	4	FALSE	51.15935	65.89322	0	3.264507076
Mean	Male	4	TRUE	69.26146	77.05243	1	9.325474319
Mean	Male	5	FALSE	51.15548	65.85526	0	3.488391929
Mean	Male	5	TRUE	68.88615	76.58978	1	9.352292388
Mean	Male	6	FALSE	51.04404	65.69063	0	3.74961676
Mean	Male	6	TRUE	68.37291	75.99292	1	9.493572641
Mean	Male	7	FALSE	51.12552	65.71495	0	3.856530809
Mean	Male	7	TRUE	67.96991	75.5622	1	9.641507637
Mean	Male	8	FALSE	50.78795	65.35291	0	4.040371252
Mean	Male	8	TRUE	67.42248	74.89572	1	9.831594152
Mean	Male	9	FALSE	50.5558	65.03233	0	4.281575642
Mean	Male	9	TRUE	66.58443	73.99249	1	9.876563566
Mean	Male	10	FALSE	49.7487	64.14061	0	4.579542913
Mean	Male	10	TRUE	64.88422	72.25591	1	10.06306914
Mean	Male	ALL	FALSE	50.95171	65.61792	0	3.649746112
Mean	Male	ALL	TRUE	68.0044	75.62636	1	9.66304209
Mean	Female	1	FALSE	52.84354	67.62372	0	2.971747116
Mean	Female	1	TRUE	74.03348	81.87075	1	8.8515234
Mean	Female	2	FALSE	52.34058	67.14119	0	3.217845864
Mean	Female	2	TRUE	73.2013	81.08391	1	8.994993533
Mean	Female	3	FALSE	52.74256	67.43342	0	3.257941364
Mean	Female	3	TRUE	73.65572	81.44229	1	8.581088929
Mean	Female	4	FALSE	52.8636	67.55455	0	3.346475427
Mean	Female	4	TRUE	73.11994	80.85057	1	8.586511275
Mean	Female	5	FALSE	52.93602	67.58846	0	3.533813438

For peer review only

Mean	Female	5	TRUE	72.87527	80.5496	1	8.608430465
Mean	Female	6	FALSE	52.78292	67.47188	0	3.865799021
Mean	Female	6	TRUE	72.03465	79.66025	1	8.980893324
Mean	Female	7	FALSE	53.06696	67.72345	0	4.08361163
Mean	Female	7	TRUE	71.87808	79.50544	1	9.199602363
Mean	Female	8	FALSE	52.88178	67.52256	0	4.25892956
Mean	Female	8	TRUE	71.50472	79.04223	1	9.278314681
Mean	Female	9	FALSE	52.54855	67.1636	0	4.582224812
Mean	Female	9	TRUE	70.73319	78.21541	1	9.421167025
Mean	Female	10	FALSE	51.786	66.35218	0	5.038761323
Mean	Female	10	TRUE	69.64389	77.0918	1	9.833394785
Mean	Female	ALL	FALSE	52.69305	67.37505	0	3.779152203
Mean	Female	ALL	TRUE	72.06165	79.70097	1	9.081690825
Mean	ALL	1	FALSE	52.18634	67.00101	0	2.99536096
Mean	ALL	1	TRUE	72.29227	80.15584	1	9.217174684
Mean	ALL	2	FALSE	51.73351	66.56184	0	3.231751254
Mean	ALL	2	TRUE	71.46755	79.35993	1	9.423016068
Mean	ALL	3	FALSE	52.02491	66.74356	0	3.266001674
Mean	ALL	3	TRUE	71.8333	79.63635	1	8.88984567
Mean	ALL	4	FALSE	52.06063	66.7718	0	3.307855264
Mean	ALL	4	TRUE	71.32626	79.08493	1	8.930031737
Mean	ALL	5	FALSE	52.10451	66.77906	0	3.512601756
Mean	ALL	5	TRUE	71.00131	78.6894	1	8.957872636
Mean	ALL	6	FALSE	51.9786	66.64796	0	3.812058676
Mean	ALL	6	TRUE	70.29159	77.91453	1	9.224938149
Mean	ALL	7	FALSE	52.17525	66.80093	0	3.979312073
Mean	ALL	7	TRUE	70.04664	77.65757	1	9.406687102
Mean	ALL	8	FALSE	51.92052	66.5265	0	4.158591562
Mean	ALL	8	TRUE	69.5786	77.08579	1	9.539367724
Mean	ALL	9	FALSE	51.62805	66.17911	0	4.443346896
Mean	ALL	9	TRUE	68.76861	76.21571	1	9.636812773
Mean	ALL	10	FALSE	50.83859	65.32373	0	4.825209137
Mean	ALL	10	TRUE	67.34138	74.75243	1	9.94450034
Mean	ALL	ALL	FALSE	51.88452	66.55919	0	3.719067175
Mean	ALL	ALL	TRUE	70.15216	77.78331	1	9.355296431
Mean	ALL	ALL	ALL	57.47384	69.99342	0.305969	5.443576361
Median	Male	1	FALSE	50.09	65.08	0	1
Median	Male	1	TRUE	71.67	79.75	1	7
Median	Male	2	FALSE	49.84	64.83	0	1
Median	Male	2	TRUE	70.92	79	1	7
Median	Male	3	FALSE	49.84	64.83	0	1
Median	Male	3	TRUE	71.17	79.17	1	6
Median	Male	4	FALSE	49.75	64.75	0	1
Median	Male	4	TRUE	70.67	78.66	1	6
Median	Male	5	FALSE	49.75	64.66	0	2
Median	Male	5	TRUE	70.25	78.09	1	6
Median	Male	6	FALSE	49.59	64.5	0	2
Median	Male	6	TRUE	69.67	77.5	1	7
Median	Male	7	FALSE	49.67	64.5	0	2
Median	Male	7	TRUE	69.25	76.92	1	7

For peer review only

Median	Male	8	FALSE	49.17	64	0	2
Median	Male	8	TRUE	68.67	76.33	1	7
Median	Male	9	FALSE	48.84	63.58	0	2
Median	Male	9	TRUE	67.84	75.33	1	7
Median	Male	10	FALSE	47.67	62.41	0	2
Median	Male	10	TRUE	66	73.42	1	7
Median	Male	ALL	FALSE	49.42	64.33	0	2
Median	Male	ALL	TRUE	69.34	77.09	1	7
Median	Female	1	FALSE	51.25	66.41	0	1
Median	Female	1	TRUE	75.92	84	1	6
Median	Female	2	FALSE	50.75	65.83	0	1
Median	Female	2	TRUE	75	83.33	1	6
Median	Female	3	FALSE	51	66.08	0	1
Median	Female	3	TRUE	75.59	83.59	1	6
Median	Female	4	FALSE	51.25	66.41	0	2
Median	Female	4	TRUE	74.84	82.84	1	6
Median	Female	5	FALSE	51.34	66.41	0	2
Median	Female	5	TRUE	74.67	82.58	1	6
Median	Female	6	FALSE	51.25	66.33	0	2
Median	Female	6	TRUE	73.67	81.5	1	6
Median	Female	7	FALSE	51.59	66.58	0	2
Median	Female	7	TRUE	73.5	81.33	1	6
Median	Female	8	FALSE	51.25	66.25	0	2
Median	Female	8	TRUE	73.09	80.84	1	6
Median	Female	9	FALSE	50.84	65.91	0	2
Median	Female	9	TRUE	72.25	79.92	1	6
Median	Female	10	FALSE	49.67	64.58	0	3
Median	Female	10	TRUE	71.09	78.83	1	7
Median	Female	ALL	FALSE	51.09	66.16	0	2
Median	Female	ALL	TRUE	73.84	81.67	1	6
Median	ALL	1	FALSE	50.67	65.75	0	1
Median	ALL	1	TRUE	73.92	82	1	6
Median	ALL	2	FALSE	50.25	65.33	0	1
Median	ALL	2	TRUE	73.09	81.25	1	6
Median	ALL	3	FALSE	50.42	65.5	0	1
Median	ALL	3	TRUE	73.59	81.58	1	6
Median	ALL	4	FALSE	50.5	65.5	0	2
Median	ALL	4	TRUE	72.92	80.84	1	6
Median	ALL	5	FALSE	50.59	65.58	0	2
Median	ALL	5	TRUE	72.59	80.5	1	6
Median	ALL	6	FALSE	50.5	65.41	0	2
Median	ALL	6	TRUE	71.75	79.59	1	6
Median	ALL	7	FALSE	50.67	65.58	0	2
Median	ALL	7	TRUE	71.5	79.25	1	6
Median	ALL	8	FALSE	50.25	65.16	0	2
Median	ALL	8	TRUE	71	78.67	1	6
Median	ALL	9	FALSE	49.92	64.75	0	2
Median	ALL	9	TRUE	70.17	77.75	1	6
Median	ALL	10	FALSE	48.67	63.5	0	2
Median	ALL	10	TRUE	68.59	76.16	1	7

For peer review only

Median	ALL	ALL	FALSE	50.25	65.25	0	2
Median	ALL	ALL	TRUE	71.67	79.5	1	6
Median	ALL	ALL	ALL	55.42	69.16	0	3
SD	Male	1	FALSE	10.29335	9.895881	0	5.999965825
SD	Male	1	TRUE	11.20425	10.84432	0	11.56477008
SD	Male	2	FALSE	10.02753	9.654498	0	5.937700309
SD	Male	2	TRUE	11.41291	11.05364	0	12.00967705
SD	Male	3	FALSE	10.3327	9.804563	0	5.99970456
SD	Male	3	TRUE	11.48086	11.07249	0	10.36340142
SD	Male	4	FALSE	10.33222	9.816467	0	5.706537374
SD	Male	4	TRUE	11.45496	10.99963	0	11.33085377
SD	Male	5	FALSE	10.36481	9.818106	0	6.255840399
SD	Male	5	TRUE	11.5018	11.04789	0	11.10793953
SD	Male	6	FALSE	10.37469	9.804766	0	7.046345911
SD	Male	6	TRUE	11.53179	11.13248	0	10.85321017
SD	Male	7	FALSE	10.43387	9.812677	0	6.503949308
SD	Male	7	TRUE	11.56689	11.14452	0	11.39198387
SD	Male	8	FALSE	10.38086	9.799004	0	7.39389737
SD	Male	8	TRUE	11.7453	11.31354	0	11.60403991
SD	Male	9	FALSE	10.3025	9.69833	0	7.552972389
SD	Male	9	TRUE	11.84546	11.4105	0	11.86501435
SD	Male	10	FALSE	10.09947	9.450429	0	9.092688632
SD	Male	10	TRUE	12.11585	11.79579	0	11.63274414
SD	Male	ALL	FALSE	10.30438	9.777146	0	6.769454973
SD	Male	ALL	TRUE	11.73831	11.36275	0	11.40272153
SD	Female	1	FALSE	11.57404	10.81761	0	6.140900821
SD	Female	1	TRUE	11.61165	11.17618	0	10.80280577
SD	Female	2	FALSE	11.29791	10.57343	0	6.300878901
SD	Female	2	TRUE	11.91055	11.4771	0	11.22758745
SD	Female	3	FALSE	11.72025	10.80205	0	6.099846813
SD	Female	3	TRUE	11.71637	11.27446	0	10.18372078
SD	Female	4	FALSE	11.69384	10.81516	0	5.929254745
SD	Female	4	TRUE	11.79621	11.33437	0	10.83215537
SD	Female	5	FALSE	11.75032	10.84331	0	6.169119002
SD	Female	5	TRUE	11.80434	11.35569	0	11.05748387
SD	Female	6	FALSE	11.56321	10.77253	0	6.785243756
SD	Female	6	TRUE	11.85046	11.40183	0	10.89387782
SD	Female	7	FALSE	11.63108	10.81993	0	7.980228565
SD	Female	7	TRUE	11.8501	11.33384	0	11.96747814
SD	Female	8	FALSE	11.68272	10.877	0	7.409969424
SD	Female	8	TRUE	11.88115	11.38483	0	11.05675793
SD	Female	9	FALSE	11.59512	10.75171	0	8.314135343
SD	Female	9	TRUE	12.04586	11.55406	0	10.93156282
SD	Female	10	FALSE	11.56579	10.69242	0	8.178867841
SD	Female	10	TRUE	12.40504	11.98232	0	10.94713744
SD	Female	ALL	FALSE	11.61171	10.78305	0	6.973080412
SD	Female	ALL	TRUE	11.98467	11.54057	0	11.02783173
SD	ALL	1	FALSE	11.02152	10.42197	0	6.076001052
SD	ALL	1	TRUE	11.57586	11.1757	0	11.17011719
SD	ALL	2	FALSE	10.74405	10.17427	0	6.1342454

For peer review only

SD	ALL	2	TRUE	11.82785	11.43173	0	11.60740759
SD	ALL	3	FALSE	11.12081	10.37474	0	6.053305018
SD	ALL	3	TRUE	11.77672	11.35324	0	10.27185504
SD	ALL	4	FALSE	11.10577	10.38973	0	5.825503356
SD	ALL	4	TRUE	11.79675	11.33927	0	11.07281312
SD	ALL	5	FALSE	11.16016	10.41308	0	6.209787135
SD	ALL	5	TRUE	11.8318	11.3849	0	11.0873384
SD	ALL	6	FALSE	11.06337	10.37421	0	6.907461287
SD	ALL	6	TRUE	11.84182	11.42213	0	10.87746257
SD	ALL	7	FALSE	11.1393	10.41761	0	7.339977794
SD	ALL	7	TRUE	11.87932	11.4163	0	11.703307
SD	ALL	8	FALSE	11.15289	10.45202	0	7.403368695
SD	ALL	8	TRUE	11.99159	11.53837	0	11.32156955
SD	ALL	9	FALSE	11.06155	10.33328	0	7.972949385
SD	ALL	9	TRUE	12.1295	11.67815	0	11.38532278
SD	ALL	10	FALSE	10.95563	10.19362	0	8.618896162
SD	ALL	10	TRUE	12.49441	12.13544	0	11.28451096
SD	ALL	ALL	FALSE	11.05815	10.36529	0	6.879584137
SD	ALL	ALL	TRUE	12.04087	11.63632	0	11.20957831
SD	ALL	ALL	ALL	14.14536	11.94771	0.460817	8.834008915
Sum	Male	1	FALSE	3448471	4444574	0	202692
Sum	Male	1	TRUE	1389774	1545859	19773	190580
Sum	Male	2	FALSE	3500109	4519182	0	222719
Sum	Male	2	TRUE	1452401	1617618	20904	207238
Sum	Male	3	FALSE	3368144	4338398	0	215437
Sum	Male	3	TRUE	1624586	1806968	23315	215773
Sum	Male	4	FALSE	3390740	4367271	0	216365
Sum	Male	4	TRUE	1744973	1941259	25194	234946
Sum	Male	5	FALSE	3298556	4246413	0	224935
Sum	Male	5	TRUE	1911177	2124907	27744	259470
Sum	Male	6	FALSE	3196582	4113810	0	234816
Sum	Male	6	TRUE	1973311	2193232	28861	273994
Sum	Male	7	FALSE	3186142	4095356	0	240339
Sum	Male	7	TRUE	2140576	2379681	31493	303640
Sum	Male	8	FALSE	3113606	4006526	0	247699
Sum	Male	8	TRUE	2287240	2540762	33924	333527
Sum	Male	9	FALSE	2978849	3831835	0	252279
Sum	Male	9	TRUE	2342174	2602760	35176	347418
Sum	Male	10	FALSE	2668719	3440759	0	245665
Sum	Male	10	TRUE	2308580	2570865	35580	358044
Sum	Male	ALL	FALSE	32149919	41404123	0	2302946
Sum	Male	ALL	TRUE	19174792	21323910	281964	2724630
Sum	Female	1	FALSE	4099866	5246586	0	230563
Sum	Female	1	TRUE	1686335	1864852	22778	201620
Sum	Female	2	FALSE	4110201	5272464	0	252691
Sum	Female	2	TRUE	1754562	1943500	23969	215601
Sum	Female	3	FALSE	3964975	5069375	0	244919
Sum	Female	3	TRUE	2029215	2243735	27550	236409
Sum	Female	4	FALSE	3932629	5025518	0	248951
Sum	Female	4	TRUE	2120625	2344828	29002	249026

For peer review only

Sum	Female	5	FALSE	3895827	4974173	0	260071
Sum	Female	5	TRUE	2282089	2522411	31315	269573
Sum	Female	6	FALSE	3840696	4909524	0	281291
Sum	Female	6	TRUE	2288469	2530726	31769	285314
Sum	Female	7	FALSE	3893152	4968395	0	299586
Sum	Female	7	TRUE	2566838	2839219	35711	328527
Sum	Female	8	FALSE	3819757	4877290	0	307631
Sum	Female	8	TRUE	2715392	3001629	37975	352344
Sum	Female	9	FALSE	3606670	4609774	0	314501
Sum	Female	9	TRUE	2766234	3058848	39108	368443
Sum	Female	10	FALSE	3195766	4094660	0	310947
Sum	Female	10	TRUE	2644378	2927176	37970	373374
Sum	Female	ALL	FALSE	38359538	49047757	0	2751151
Sum	Female	ALL	TRUE	22854136	25276924	317147	2880231
Sum	ALL	1	FALSE	7548337	9691160	0	433255
Sum	ALL	1	TRUE	3076108	3410711	42551	392200
Sum	ALL	2	FALSE	7610310	9791646	0	475410
Sum	ALL	2	TRUE	3206963	3561118	44873	422839
Sum	ALL	3	FALSE	7333119	9407772	0	460356
Sum	ALL	3	TRUE	3653801	4050703	50865	452182
Sum	ALL	4	FALSE	7323369	9392789	0	465316
Sum	ALL	4	TRUE	3865598	4286087	54196	483972
Sum	ALL	5	FALSE	7194383	9220586	0	485006
Sum	ALL	5	TRUE	4193267	4647317	59059	529043
Sum	ALL	6	FALSE	7037278	9023334	0	516107
Sum	ALL	6	TRUE	4261779	4723958	60630	559308
Sum	ALL	7	FALSE	7079294	9063751	0	539925
Sum	ALL	7	TRUE	4707414	5218899	67204	632167
Sum	ALL	8	FALSE	6933363	8883815	0	555330
Sum	ALL	8	TRUE	5002632	5542391	71899	685871
Sum	ALL	9	FALSE	6585519	8441609	0	566780
Sum	ALL	9	TRUE	5108407	5661608	74284	715861
Sum	ALL	10	FALSE	5864485	7535418	0	556612
Sum	ALL	10	TRUE	4952959	5498041	73550	731418
Sum	ALL	ALL	FALSE	70509457	90451880	0	5054097
Sum	ALL	ALL	TRUE	42028928	46600834	599111	5604861
Sum	ALL	ALL	ALL	1.13E+08	1.37E+08	599111	10658958

For peer review only

A	B	C	D	E	F	G	H	I
768	381	4912	4307	751	355	2680	6290	11876
1187	200	8254	2540	915	796	1577	2963	8557
947	442	5057	4910	864	387	2786	6419	12800
1307	205	8489	2889	1107	873	1714	3289	9226
904	398	4465	4753	806	466	2809	6089	12413
1439	219	8672	2994	1227	1074	1951	3659	10152
932	422	4324	4713	861	536	2993	6120	12650
1579	227	9537	3301	1410	1170	2087	3921	11082
989	388	4315	4880	943	606	2953	6114	12756
1688	278	10341	3586	1601	1328	2166	4407	12340
1023	430	4169	4982	1011	708	3052	6168	12721
1747	251	10747	3912	1678	1402	2281	4473	12687
1114	455	4091	4988	1114	845	3098	6299	13064
1946	318	11547	4154	1806	1677	2338	4835	13848
1156	434	3943	4850	1076	950	3191	6165	13071
2182	373	12407	4563	2183	1686	2435	5192	14854
1222	422	3744	4958	1209	1106	2900	6021	13041
2250	390	12585	4612	2186	2028	2551	5123	15077
1193	417	3183	4661	1239	1398	2721	5225	12022
2159	362	12620	4635	2238	2454	2508	4779	14900
10248	4189	42203	48002	9874	7357	29183	60910	126414
17484	2823	105199	37186	16351	14488	21608	42641	122723
1193	453	6203	6623	1059	447	3348	9096	8959
1370	230	7227	2504	1178	1175	1674	3717	8838
1291	517	6381	7534	1198	502	3694	9132	9766
1461	254	7507	2939	1336	1158	1748	4249	9326
1265	484	5911	7155	1220	539	3689	8872	9649
1670	269	8030	3221	1612	1542	2172	4964	10847
1279	456	5846	7166	1255	566	3781	9320	9851
1852	241	8746	3503	1679	1442	2161	5254	11583
1399	526	5765	7377	1375	610	3965	9118	10271
1885	278	9268	3889	1845	1615	2220	5813	12493
1533	541	5842	7419	1425	719	4167	9503	10746
2063	342	9884	3962	1943	1601	2226	5822	12998
1688	572	5873	7713	1572	812	4256	9891	11324
2537	354	10894	4538	2260	1651	2432	6734	14576
1771	560	5610	7678	1631	826	4388	9918	11506
2692	355	11410	4741	2439	1745	2600	6868	15761
1797	630	5387	7669	1673	813	4169	9473	11668
2801	392	11941	5022	2586	1843	2524	6954	15978
1815	573	4705	7256	1634	967	4132	8244	11054
2755	389	11506	5042	2657	1837	2475	6532	15461
15031	5312	57523	73590	14042	6801	39589	92567	104794
21086	3104	96413	39361	19535	15609	22232	56907	127861
1961	834	11115	10930	1810	802	6028	15386	20835
2557	430	15481	5044	2093	1971	3251	6680	17395
2238	959	11438	12444	2062	889	6480	15551	22566
2768	459	15996	5828	2443	2031	3462	7538	18552

For peer review only

2169	882	10376	11908	2026	1005	6498	14961	22062
3109	488	16702	6215	2839	2616	4123	8623	20999
2211	878	10170	11879	2116	1102	6774	15440	22501
3431	468	18283	6804	3089	2612	4248	9175	22665
2388	914	10080	12257	2318	1216	6918	15232	23027
3573	556	19609	7475	3446	2943	4386	10220	24833
2556	971	10011	12401	2436	1427	7219	15671	23467
3810	593	20631	7874	3621	3003	4507	10295	25685
2802	1027	9964	12701	2686	1657	7354	16190	24388
4483	672	22441	8692	4066	3328	4770	11569	28424
2927	994	9553	12528	2707	1776	7579	16083	24577
4874	728	23817	9304	4622	3431	5035	12060	30615
3019	1052	9131	12627	2882	1919	7069	15494	24709
5051	782	24526	9634	4772	3871	5075	12077	31055
3008	990	7888	11917	2873	2365	6853	13469	23076
4914	751	24126	9677	4895	4291	4983	11311	30361
25279	9501	99726	121592	23916	14158	68772	153477	231208
38570	5927	201612	76547	35886	30097	43840	99548	250584
63849	15428	301338	198139	59802	44255	112612	253025	481792
0.019029	0.008366	0.296464	0.132738	0.063856	0.010036	0.087776	0.163816	0.501603
0.112325	0.015779	2.710868	0.485055	0.11369	0.07227	0.160977	0.24842	1.415971
0.022617	0.008807	0.289772	0.143326	0.06995	0.010966	0.069439	0.163784	0.545146
0.115385	0.015499	2.649254	0.46594	0.139399	0.078119	0.156477	0.26593	1.485314
0.023123	0.009091	0.260938	0.142251	0.058941	0.012755	0.082581	0.158305	0.561221
0.116406	0.013682	2.105469	0.420459	0.125842	0.082222	0.162428	0.25653	1.447609
0.023613	0.008826	0.250822	0.135354	0.048764	0.015254	0.079423	0.162286	0.561619
0.118004	0.014646	2.139875	0.446972	0.126816	0.088593	0.173057	0.258435	1.45741
0.025946	0.01174	0.259069	0.140429	0.064143	0.020626	0.087064	0.166095	0.593369
0.110871	0.016076	2.060734	0.41454	0.129578	0.091011	0.156538	0.259624	1.49034
0.027801	0.011194	0.261513	0.171324	0.083083	0.024384	0.093015	0.168114	0.63118
0.114653	0.015661	2.079796	0.432487	0.129691	0.097017	0.168324	0.257233	1.462631
0.030568	0.010398	0.259499	0.166528	0.074358	0.028514	0.092121	0.181386	0.651621
0.118376	0.015591	2.043121	0.401137	0.151843	0.106341	0.151494	0.25339	1.511383
0.034271	0.010342	0.264101	0.161436	0.08805	0.034744	0.094281	0.178808	0.675105
0.125604	0.023464	2.019308	0.427249	0.152901	0.10721	0.152075	0.2528	1.532219
0.037813	0.011354	0.262483	0.162842	0.091036	0.042989	0.092359	0.180764	0.713808
0.12662	0.017938	1.968189	0.429242	0.144445	0.1221	0.148368	0.234506	1.504236
0.040769	0.0118	0.24575	0.176907	0.103423	0.06435	0.094046	0.169395	0.728991
0.120573	0.019084	1.943761	0.391821	0.168325	0.151124	0.144182	0.218353	1.417566
0.028127	0.010127	0.265669	0.152462	0.073648	0.025395	0.086834	0.168986	0.611913
0.11859	0.017091	2.129421	0.427519	0.140685	0.103538	0.156336	0.24884	1.47538
0.02485	0.007463	0.386544	0.149617	0.043127	0.010157	0.075414	0.205207	0.276445
0.103345	0.016595	2.489595	0.330582	0.108614	0.097594	0.148872	0.265914	1.129072
0.028321	0.00922	0.371995	0.154607	0.0394	0.010735	0.097812	0.207455	0.302987
0.109642	0.01698	2.330469	0.361675	0.10597	0.088156	0.15374	0.290083	1.130001
0.026884	0.008567	0.355313	0.161674	0.044948	0.013887	0.084588	0.210147	0.318626
0.103666	0.014955	1.971688	0.345336	0.122831	0.098984	0.153103	0.298911	1.135644
0.027503	0.00781	0.347994	0.164507	0.042545	0.013886	0.08927	0.22118	0.335103
0.11151	0.011965	2.015551	0.305082	0.116647	0.084891	0.15392	0.295635	1.168264
0.032217	0.008886	0.343108	0.166805	0.049501	0.016401	0.092778	0.216781	0.353407

For peer review only

0.109149	0.013284	1.806131	0.382341	0.119527	0.089861	0.139039	0.300655	1.171004
0.03554	0.009648	0.374814	0.182659	0.056594	0.019254	0.103581	0.23021	0.37836
0.120274	0.016903	1.955334	0.349051	0.128396	0.09141	0.146841	0.30108	1.211212
0.040811	0.010278	0.353175	0.183035	0.077055	0.022682	0.104726	0.242656	0.413737
0.131248	0.014393	1.88715	0.364594	0.141749	0.087228	0.137437	0.308532	1.246199
0.043291	0.010619	0.356352	0.195038	0.067768	0.022442	0.106504	0.240267	0.429713
0.129849	0.014957	1.802238	0.375958	0.139855	0.08661	0.13788	0.299197	1.270072
0.046274	0.011889	0.363109	0.202841	0.073243	0.022438	0.114344	0.242748	0.465652
0.135701	0.01524	1.825074	0.358341	0.149816	0.08364	0.127237	0.289225	1.275468
0.055128	0.013855	0.354005	0.217028	0.078381	0.031664	0.126785	0.238126	0.497237
0.137556	0.016223	1.782723	0.341348	0.156492	0.094101	0.137398	0.278378	1.293258
0.035544	0.00972	0.36092	0.176569	0.056536	0.017988	0.098828	0.224844	0.373224
0.121266	0.015107	1.951515	0.352817	0.131728	0.089835	0.142249	0.293429	1.213447
0.022151	0.007882	0.344782	0.141791	0.052737	0.010101	0.081145	0.186018	0.38083
0.107518	0.016216	2.592419	0.402364	0.110973	0.085826	0.154497	0.257785	1.262391
0.025662	0.009028	0.333664	0.149348	0.053642	0.010843	0.084585	0.187096	0.415877
0.112317	0.01629	2.478974	0.410247	0.121543	0.08348	0.155015	0.278831	1.295523
0.025129	0.008811	0.311272	0.15261	0.051478	0.013359	0.083651	0.185954	0.431836
0.109506	0.014371	2.033009	0.37977	0.124211	0.091301	0.157377	0.279485	1.27864
0.02567	0.008289	0.302211	0.150771	0.045475	0.01453	0.084631	0.193431	0.441828
0.114529	0.013211	2.073345	0.371042	0.121374	0.086612	0.162816	0.278342	1.302679
0.029288	0.010219	0.303862	0.154487	0.056339	0.018374	0.09011	0.193111	0.465468
0.109958	0.014596	1.925735	0.397467	0.124249	0.090401	0.14726	0.28138	1.321018
0.03196	0.010363	0.322407	0.177416	0.068847	0.021627	0.098694	0.201488	0.495302
0.117599	0.016312	2.01458	0.388768	0.129012	0.094079	0.157067	0.280208	1.330892
0.036106	0.010333	0.310149	0.175453	0.075816	0.025361	0.098936	0.214515	0.522998
0.125216	0.014954	1.96024	0.381718	0.146479	0.096185	0.144024	0.282692	1.370469
0.03915	0.010491	0.314001	0.179612	0.077079	0.028089	0.100893	0.212052	0.54237
0.127846	0.018971	1.904658	0.400159	0.14601	0.09633	0.144578	0.277306	1.393761
0.042365	0.011642	0.316627	0.184365	0.081462	0.031931	0.104189	0.214116	0.580282
0.131401	0.016518	1.892844	0.391915	0.147273	0.101852	0.137244	0.263314	1.383797
0.04845	0.012899	0.303663	0.19837	0.090026	0.046864	0.11156	0.206164	0.605011
0.129341	0.017607	1.860625	0.365765	0.162216	0.121686	0.14068	0.249341	1.353392
0.0321	0.009909	0.316694	0.165376	0.064481	0.021427	0.093259	0.198908	0.484051
0.120006	0.01604	2.035244	0.387975	0.135943	0.096284	0.148879	0.272444	1.336722
0.058997	0.011785	0.842516	0.233484	0.086346	0.044331	0.110277	0.221408	0.744941
0	0	0	0	0	0	0	0	0
0	0	0	0	0	0	0	0	0
0	0	0	0	0	0	0	0	0
0	0	0	0	0	0	0	0	0
0	0	0	0	0	0	0	0	0
0	0	0	0	0	0	0	0	0
0	0	0	0	0	0	0	0	0
0	0	0	0	0	0	0	0	0
0	0	0	0	0	0	0	0	0
0	0	0	0	0	0	0	0	0
0	0	0	0	0	0	0	0	0
0	0	0	0	0	0	0	0	0
0	0	0	0	0	0	0	0	0
0	0	0	0	0	0	0	0	0

For peer review only

0	0	0	0	0	0	0	0	0
0	0	0	0	0	0	0	0	0
0	0	0	0	0	0	0	0	0
0	0	0	0	0	0	0	0	0
0	0	0	0	0	0	0	0	0
0	0	0	0	0	0	0	0	0
0	0	0	0	0	0	0	0	0
0	0	0	0	0	0	0	0	0
0	0	0	0	0	0	0	0	0
0	0	0	0	0	0	0	0	0
0	0	0	0	0	0	0	0	0
0	0	0	0	0	0	0	0	0
0	0	0	0	0	0	0	0	0
0	0	0	0	0	0	0	0	0
0	0	0	0	0	0	0	0	0
0	0	0	0	0	0	0	0	0
0	0	0	0	0	0	0	0	0
0	0	0	0	0	0	0	0	0
0	0	0	0	0	0	0	0	0
0	0	0	0	0	0	0	0	0
0	0	0	0	0	0	0	0	0
0	0	0	0	0	0	0	0	0
0	0	0	0	0	0	0	0	0
0	0	0	0	0	0	0	0	0
0	0	0	0	0	0	0	0	0
0	0	0	0	0	0	0	0	0
0	0	0	0	0	0	0	0	0
0	0	0	0	0	0	0	0	0
0	0	0	0	0	0	0	0	0
0	0	0	0	0	0	0	0	0
0	0	0	0	0	0	0	0	0
0	0	0	0	0	0	0	0	0
0	0	0	0	0	0	0	0	0
0	0	0	0	0	0	0	0	0
0	0	0	0	0	0	0	0	0
0	0	0	0	0	0	0	0	0
0	0	0	0	0	0	0	0	0
0	0	0	0	0	0	0	0	0
0	0	0	0	0	0	0	0	0
0	0	0	0	0	0	0	0	0
0	0	0	0	0	0	0	0	0
0	0	0	0	0	0	0	0	0
0	0	0	0	0	0	0	0	0
0	0	0	0	0	0	0	0	0
0	0	0	0	0	0	0	0	0
0	0	0	0	0	0	0	0	0
0	0	0	0	0	0	0	0	0
0	0	0	0	0	0	0	0	0
0	0	0	0	0	0	0	0	0
0	0	0	0	0	0	0	0	0

For peer review only

0	0	0	0	0	0	0	0	0
0	0	0	0	0	0	0	0	0
0	0	0	0	0	0	0	0	0
0.230792	0.175965	2.333179	1.333495	1.60647	0.220566	2.134165	0.614141	1.519509
0.571489	0.187765	7.643945	4.751326	1.113488	0.418428	0.826387	0.716548	2.650713
0.231018	0.12976	2.25699	1.678535	1.608845	0.227771	0.541433	0.640879	1.613277
0.556336	0.184945	8.029279	3.608501	1.97123	0.478572	0.752077	0.782734	2.753447
0.266392	0.165634	2.262611	1.505966	1.529528	0.212216	1.147733	0.582474	1.648018
0.58012	0.179912	5.805775	4.041207	1.43015	0.460845	0.822182	0.699535	2.68662
0.247749	0.155172	2.054445	1.573823	1.077153	0.231162	0.876693	0.642487	1.647871
0.57778	0.199988	5.990244	4.505199	1.052615	0.572815	1.140847	0.705978	2.763253
0.261331	0.910854	2.062573	1.117822	1.30602	0.366319	1.600913	0.661005	1.731801
0.540771	0.216038	5.729766	3.378423	0.833186	0.521989	0.976514	0.720675	2.921626
0.292358	0.349403	1.996117	2.782163	1.545755	0.431645	0.996915	0.62131	1.821876
0.586331	0.343584	6.2395	3.792923	0.840741	0.593168	0.997021	0.754343	2.723394
0.281285	0.232587	1.913255	1.707741	1.486339	0.358611	1.164636	0.699992	1.836605
0.582371	0.19661	5.859959	3.326421	1.454984	0.626505	0.91125	0.706926	2.946004
0.321459	0.163331	2.314019	2.475764	1.668335	0.433376	0.931178	0.700122	1.894178
0.765409	1.274043	6.251932	3.285676	0.995937	0.695793	1.43561	0.709614	3.069226
0.334273	0.243425	2.077137	1.434603	1.545043	0.532511	0.768026	0.684113	2.300365
0.615184	0.269337	5.820038	3.542813	0.907194	0.711217	0.869848	0.696148	3.048583
0.373463	0.182341	1.843753	2.744422	1.788316	0.616253	0.656313	0.651455	3.02197
0.699619	0.386397	5.88204	3.726093	1.358842	0.919547	0.804935	0.654621	2.824426
0.284529	0.351715	2.124434	1.898406	1.521973	0.378522	1.185093	0.649821	1.920883
0.620343	0.505404	6.266448	3.763073	1.214683	0.641376	0.987165	0.711962	2.864078
0.29701	0.10858	2.891878	1.457956	1.078003	0.1635	0.69937	0.679934	1.096574
0.490446	0.232735	8.071861	2.888655	0.890533	0.506707	1.022772	0.708715	2.241084
0.298246	0.154371	2.560022	1.075344	0.875203	0.167377	2.825476	0.713345	1.210603
0.538871	0.252498	7.868462	3.395343	0.638288	0.47732	1.055346	0.746712	2.223521
0.25997	0.122846	2.601747	1.890895	0.917357	0.332469	0.704351	0.732196	1.594599
0.50075	0.195955	6.776997	3.13512	1.519512	0.558693	0.799565	0.823391	2.207223
0.261158	0.122617	2.577808	1.34893	0.683076	0.236381	0.821406	0.725898	1.301769
0.527947	0.162398	6.926511	2.329253	0.719958	0.476726	1.256148	0.767455	2.315119
0.295508	0.116462	2.556902	1.134573	0.900374	0.349183	0.789523	0.728222	1.315024
0.712235	0.176949	5.829402	4.757346	0.797975	0.494239	0.905876	0.751281	2.294817
0.300582	0.129127	2.8398	1.824138	0.923	0.310565	0.855584	0.745438	1.669348
0.558452	0.21496	6.376546	3.297313	0.927307	0.527944	0.894174	0.798772	2.315047
0.350719	0.133335	2.4566	1.211907	3.230293	0.387579	0.838621	0.790819	1.999533
0.609039	0.163842	6.27416	4.392373	1.491322	0.504566	0.819659	0.770184	2.955601
0.372335	0.146907	2.465375	2.213014	1.136021	0.294312	0.703398	0.738853	1.641226
0.575219	0.225647	5.919553	3.227816	0.964179	0.511163	0.730428	0.789482	2.755117
0.348778	0.142121	2.606975	1.917113	1.133772	0.303294	1.050497	0.745342	1.763048
0.615573	0.184202	6.038163	2.823089	0.980768	0.462781	0.717078	0.743733	2.545122
0.668872	0.214948	2.373401	1.832936	1.094112	0.365151	0.978128	0.766222	1.624463
0.610998	0.200608	5.816483	2.351283	0.847634	0.531521	0.992959	0.710322	2.639924
0.355575	0.140415	2.602486	1.623215	1.382264	0.297838	1.214474	0.736248	1.53847
0.583764	0.200746	6.509998	3.347996	1.025351	0.505765	0.917217	0.76208	2.496747
0.268365	0.143802	2.647933	1.40165	1.34903	0.192076	1.540763	0.650586	1.314541
0.529663	0.21302	7.876587	3.868175	1.000329	0.467927	0.936658	0.712411	2.444166
0.269018	0.143424	2.423813	1.389519	1.271129	0.197839	2.097256	0.68087	1.417827

0.547078	0.223581	7.945286	3.496607	1.424084	0.477924	0.926494	0.763791	2.490775
0.262992	0.1444	2.449779	1.722026	1.24121	0.282788	0.93772	0.667024	1.624257
0.538617	0.18877	6.350573	3.579192	1.479208	0.516212	0.810016	0.769382	2.443615
0.254935	0.13891	2.346306	1.459282	0.890743	0.233937	0.847915	0.688485	1.479313
0.551677	0.18085	6.508282	3.513321	0.890205	0.523591	1.203949	0.739739	2.537399
0.280084	0.628231	2.339458	1.126854	1.108461	0.357293	1.236575	0.698094	1.5286
0.637452	0.196287	5.784157	4.166795	0.814714	0.50746	0.939754	0.737339	2.612943
0.29683	0.255794	2.486043	2.317043	1.250293	0.371516	0.923661	0.691491	1.746111
0.571895	0.283558	6.311935	3.542108	0.887147	0.559943	0.944581	0.778241	2.520809
0.320739	0.185632	2.224068	1.460715	2.580056	0.374562	1.001642	0.751086	1.930048
0.59672	0.179942	6.084023	3.929037	1.474402	0.565071	0.863812	0.741718	2.954051
0.349926	0.154663	2.397508	2.337361	1.405688	0.364848	0.815923	0.721977	1.766101
0.671698	0.890373	6.079567	3.255322	0.979307	0.605418	1.119933	0.753206	2.910477
0.342179	0.19555	2.377472	1.711331	1.339558	0.424955	0.930793	0.718371	2.03277
0.615402	0.228507	5.936263	3.18442	0.946639	0.593838	0.793161	0.722103	2.79717
0.551587	0.200447	2.144109	2.302221	1.45868	0.498195	0.844034	0.715964	2.381565
0.655417	0.304962	5.848802	3.093687	1.124348	0.74545	0.906883	0.684596	2.731422
0.324549	0.260766	2.3929	1.756401	1.448834	0.337727	1.200936	0.698008	1.730693
0.601258	0.376231	6.397141	3.549591	1.118465	0.573637	0.950803	0.739251	2.679105
0.430529	0.300851	4.137926	2.450846	1.356722	0.425479	1.130586	0.711688	2.1046
1276	561	19880	8901	4282	673	5886	10985	33636
2221	312	53602	9591	2248	1429	3183	4912	27998
1551	604	19872	9829	4797	752	4762	11232	37385
2412	324	55380	9740	2914	1633	3271	5559	31049
1521	598	17164	9357	3877	839	5432	10413	36916
2714	319	49089	9803	2934	1917	3787	5981	33751
1565	585	16624	8971	3232	1011	5264	10756	37223
2973	369	53912	11261	3195	2232	4360	6511	36718
1673	757	16705	9055	4136	1330	5614	10710	38261
3076	446	57173	11501	3595	2525	4343	7203	41348
1741	701	16377	10729	5203	1527	5825	10528	39527
3309	452	60025	12482	3743	2800	4858	7424	42213
1905	648	16172	10378	4634	1777	5741	11304	40609
3728	491	64344	12633	4782	3349	4771	7980	47598
2101	634	16191	9897	5398	2130	5780	10962	41388
4261	796	68503	14494	5187	3637	5159	8576	51979
2228	669	15466	9595	5364	2533	5442	10651	42059
4454	631	69233	15099	5081	4295	5219	8249	52913
2187	633	13183	9490	5548	3452	5045	9087	39106
4290	679	69159	13941	5989	5377	5130	7769	50437
17748	6390	167634	96202	46471	16024	54791	106628	386110
33438	4819	600420	120545	39668	29194	44081	70164	416004
1928	579	29990	11608	3346	788	5851	15921	21448
2354	378	56708	7530	2474	2223	3391	6057	25718
2224	724	29212	12141	3094	843	7681	16291	23793
2628	407	55859	8669	2540	2113	3685	6953	27085
2021	644	26711	12154	3379	1044	6359	15798	23953
2856	412	54320	9514	3384	2727	4218	8235	31287
2046	581	25888	12238	3165	1033	6641	16454	24929
3234	347	58455	8848	3383	2462	4464	8574	33882

For peer review only

2371	654	25251	12276	3643	1207	6828	15954	26009
3418	416	56559	11973	3743	2814	4354	9415	36670
2586	702	27273	13291	4118	1401	7537	16751	27531
3821	537	62119	11089	4079	2904	4665	9565	38479
2994	754	25910	13428	5653	1664	7683	17802	30353
4687	514	67392	13020	5062	3115	4908	11018	44503
3127	767	25740	14088	4895	1621	7693	17355	31039
4931	568	68440	14277	5311	3289	5236	11362	48231
3176	816	24922	13922	5027	1540	7848	16661	31960
5307	596	71375	14014	5859	3271	4976	11311	49881
3402	855	21846	13393	4837	1954	7824	14695	30685
5223	616	67690	12961	5942	3573	5217	10570	49105
25875	7076	262743	128539	41157	13095	71945	163682	271700
38459	4791	618917	111895	41777	28491	45114	93060	384841
3204	1140	49870	20509	7628	1461	11737	26906	55084
4575	690	110310	17121	4722	3652	6574	10969	53716
3775	1328	49084	21970	7891	1595	12443	27523	61178
5040	731	111239	18409	5454	3746	6956	12512	58134
3542	1242	43875	21511	7256	1883	11791	26211	60869
5570	731	103409	19317	6318	4644	8005	14216	65038
3611	1166	42512	21209	6397	2044	11905	27210	62152
6207	716	112367	20109	6578	4694	8824	15085	70600
4044	1411	41956	21331	7779	2537	12442	26664	64270
6494	862	113732	23474	7338	5339	8697	16618	78018
4327	1403	43650	24020	9321	2928	13362	27279	67058
7130	989	122144	23571	7822	5704	9523	16989	80692
4899	1402	42082	23806	10287	3441	13424	29106	70962
8415	1005	131736	25653	9844	6464	9679	18998	92101
5228	1401	41931	23985	10293	3751	13473	28317	72427
9192	1364	136943	28771	10498	6926	10395	19938	100210
5404	1485	40388	23517	10391	4073	13290	27312	74019
9761	1227	140608	29113	10940	7566	10195	19560	102794
5589	1488	35029	22883	10385	5406	12869	23782	69791
9513	1295	136849	26902	11931	8950	10347	18339	99542
43623	13466	430377	224741	87628	29119	126736	270310	657810
71897	9610	1219337	232440	81445	57685	89195	163224	800845
115520	23076	1649714	457181	169073	86804	215931	433534	1458655

For peer review only

Hospital ICD10 Admissions

J	K	L	M	N	O	P	Q	R
3574	15522	2986	9518	7240	<10	<10	210	12744
5800	6409	1260	2721	4188	<10	<10	76	8567
4172	17435	3413	10205	7851	<10	<10	234	14252
6321	7222	1424	2967	4501	<10	<10	70	9348
4290	16820	3123	10482	7367	<10	<10	228	13495
7428	7826	1553	3116	5034	<10	<10	80	10365
4328	16820	3501	10468	7357	<10	<10	220	13365
8088	8405	1659	3529	5118	<10	<10	73	11053
4570	17133	3449	10425	7519	<10	<10	237	13854
9010	9637	1859	3756	5699	<10	<10	88	12361
4770	17098	3578	10312	7367	<10	<10	255	14358
9482	10113	2139	3961	5823	<10	<10	104	13156
5072	17155	3765	10248	7560	<10	<10	229	14886
10914	11080	2259	4130	6228	<10	<10	92	14383
5264	17079	3762	9765	7431	<10	<10	287	15658
12021	12020	2414	4344	6685	<10	<10	97	15843
5374	16918	3593	9383	7192	<10	<10	207	16036
12512	12806	2433	4280	6595	<10	<10	93	16556
5290	16069	3390	8216	6531	<10	<10	217	15737
13235	13238	2476	4203	6240	<10	<10	107	17407
46704	168049	34560	99022	73415	<10	<10	2324	144385
94811	98756	19476	37007	56111	<10	<10	880	129039
3695	15668	3016	12344	11755	428	<10	265	14018
5758	6615	1456	3668	4440	<10	<10	78	9189
4214	18276	3422	13063	12985	398	<10	269	16138
6364	7401	1557	4092	4747	<10	<10	60	10271
4254	17462	3327	12865	12628	356	<10	248	15210
7538	8421	1885	4760	5515	<10	<10	63	11733
4516	17454	3460	13002	12538	380	<10	265	15145
8160	8888	1947	4837	5645	<10	<10	59	12232
4985	18743	3704	13127	12852	342	<10	285	16016
9023	10000	2129	5063	6041	<10	<10	85	13231
5339	19675	3816	13413	13155	363	<10	272	17521
9504	10306	2216	5181	6149	<10	<10	84	13696
5982	20433	3962	13937	13694	311	<10	296	18596
11324	11659	2475	5828	7087	<10	<10	102	15600
6568	20650	4051	13628	13687	298	<10	292	19408
12587	12819	2592	6001	7511	<10	<10	106	17011
6972	20815	3880	13074	13245	302	<10	313	19694
13302	13588	2760	6026	7719	17	<10	100	17666
7353	20193	3721	11834	12551	314	<10	273	20172
13852	13685	2674	5641	7805	<10	<10	119	17916
53878	189369	36359	130287	129090	3492	<10	2778	171918
97412	103382	21691	51097	62659	70	<10	856	138545
7269	31190	6002	21862	18995	428	<10	475	26762
11558	13024	2716	6389	8628	<10	<10	154	17756
8386	35711	6835	23268	20836	398	<10	503	30390
12685	14623	2981	7059	9248	<10	<10	130	19619

For peer review only

8544	34282	6450	23347	19995	356	<10	476	28705
14966	16247	3438	7876	10549	<10	<10	143	22098
8844	34274	6961	23470	19895	380	<10	485	28510
16248	17293	3606	8366	10763	<10	<10	132	23285
9555	35876	7153	23552	20371	342	<10	522	29870
18033	19637	3988	8819	11740	<10	<10	173	25592
10109	36773	7394	23725	20522	363	<10	527	31879
18986	20419	4355	9142	11972	<10	<10	188	26852
11054	37588	7727	24185	21254	311	<10	525	33482
22238	22739	4734	9958	13315	<10	<10	194	29983
11832	37729	7813	23393	21118	298	<10	579	35066
24608	24839	5006	10345	14196	<10	<10	203	32854
12346	37733	7473	22457	20437	302	<10	520	35730
25814	26394	5193	10306	14314	17	<10	193	34222
12643	36262	7111	20050	19082	314	<10	490	35909
27087	26923	5150	9844	14045	<10	<10	226	35323
100582	357418	70919	229309	202505	3492	<10	5102	316303
192223	202138	41167	88104	118770	70	<10	1736	267584
292805	559556	112086	317413	321275	3562	<10	6838	583887
0.103106	0.449245	0.066272	0.244329	0.1964	<0.005	<0.005	0.005443	0.333522
0.825064	0.799019	0.117382	0.256815	0.471856	<0.005	<0.005	0.006676	1.007788
0.116801	0.50853	0.072341	0.261746	0.213115	<0.005	<0.005	0.005949	0.378955
0.876387	0.868925	0.124378	0.262294	0.513777	<0.005	<0.005	0.005645	1.059223
0.127459	0.50941	0.069354	0.293426	0.209538	<0.005	<0.005	0.006187	0.371948
0.943213	0.827193	0.128973	0.248938	0.48857	<0.005	<0.005	0.006262	1.042033
0.128293	0.509732	0.077914	0.286943	0.204774	<0.005	<0.005	0.005537	0.36789
0.936493	0.84024	0.112844	0.255735	0.490236	<0.005	<0.005	0.006113	1.036953
0.150075	0.542005	0.07945	0.292474	0.216482	<0.005	<0.005	0.006467	0.408322
1.002127	0.876081	0.124171	0.248775	0.4898	<0.005	<0.005	0.005046	1.05439
0.163611	0.576616	0.087219	0.309626	0.229289	<0.005	<0.005	0.006914	0.460606
1.025363	0.904993	0.139011	0.253491	0.466893	<0.005	<0.005	0.00589	1.104432
0.177712	0.598219	0.094769	0.302359	0.23206	<0.005	<0.005	0.006162	0.484194
1.1373	0.916045	0.142032	0.240784	0.464135	<0.005	<0.005	<0.005	1.15505
0.196376	0.63183	0.102616	0.295469	0.241624	<0.005	<0.005	0.007405	0.543046
1.183557	0.948031	0.158501	0.235084	0.455371	<0.005	<0.005	<0.005	1.221937
0.22635	0.661671	0.116968	0.299362	0.252045	<0.005	<0.005	0.006432	0.613727
1.238316	0.995508	0.169462	0.23172	0.453121	<0.005	<0.005	0.005458	1.257875
0.262434	0.730333	0.1158	0.294311	0.251007	<0.005	<0.005	0.006748	0.720863
1.377909	1.076363	0.13932	0.217482	0.423384	<0.005	<0.005	<0.005	1.385638
0.162068	0.566621	0.087192	0.287305	0.223537	<0.005	<0.005	0.006301	0.460666
1.085841	0.919153	0.138301	0.242843	0.467946	<0.005	<0.005	0.005221	1.152608
0.100045	0.398569	0.05541	0.317355	0.248566	0.006277	<0.005	0.006857	0.324457
0.713452	0.683335	0.116648	0.333392	0.427167	<0.005	<0.005	0.005839	0.930898
0.114252	0.467311	0.064779	0.334225	0.271037	0.006036	<0.005	0.005425	0.378056
0.763987	0.749426	0.125162	0.350912	0.427385	<0.005	<0.005	<0.005	1.021486
0.120823	0.480206	0.063824	0.341	0.270339	0.005427	<0.005	0.006026	0.380786
0.767913	0.733612	0.117132	0.335572	0.430018	<0.005	<0.005	<0.005	1.008566
0.135297	0.493897	0.069403	0.348183	0.275809	0.00621	<0.005	0.006036	0.385485
0.824185	0.73457	0.124578	0.322392	0.436866	<0.005	<0.005	<0.005	0.968864
0.14891	0.536382	0.077206	0.367036	0.285454	0.005585	<0.005	0.006278	0.425749

For peer review only

0.872872	0.770972	0.157401	0.330161	0.43088	<0.005	<0.005	0.005109	1.001022
0.174289	0.592876	0.082046	0.383775	0.313782	0.005745	<0.005	0.006171	0.488071
0.967767	0.812081	0.132519	0.337688	0.440398	<0.005	<0.005	<0.005	1.061349
0.20434	0.629663	0.082712	0.393877	0.332565	<0.005	<0.005	0.006556	0.527132
1.082272	0.836353	0.168576	0.339727	0.448797	<0.005	<0.005	0.005489	1.097281
0.235851	0.653976	0.099928	0.401872	0.343352	<0.005	<0.005	0.006175	0.582221
1.168611	0.891718	0.151942	0.329717	0.452192	<0.005	<0.005	<0.005	1.13738
0.280251	0.719152	0.113616	0.430946	0.349064	<0.005	<0.005	0.006513	0.646303
1.229621	0.92109	0.172292	0.331953	0.456198	<0.005	<0.005	<0.005	1.19303
0.364327	0.807457	0.104876	0.42174	0.384162	0.006028	<0.005	0.007276	0.79976
1.462865	0.973189	0.165051	0.322307	0.493758	<0.005	<0.005	0.005109	1.3138
0.183065	0.571027	0.080435	0.372084	0.30511	0.005591	<0.005	0.006311	0.485522
1.022747	0.825135	0.146402	0.332779	0.446995	<0.005	<0.005	<0.005	1.08958
0.101464	0.422063	0.060446	0.2835	0.224382	<0.005	<0.005	0.006202	0.32866
0.765317	0.737092	0.116989	0.297807	0.447933	<0.005	<0.005	0.006228	0.966628
0.115441	0.486527	0.068304	0.300436	0.244035	<0.005	<0.005	0.005669	0.378475
0.816348	0.805094	0.124797	0.309629	0.467631	<0.005	<0.005	<0.005	1.039066
0.12392	0.493835	0.066405	0.318799	0.241965	<0.005	<0.005	0.006101	0.376662
0.848265	0.776506	0.12256	0.295862	0.456856	<0.005	<0.005	<0.005	1.023906
0.131997	0.501358	0.073413	0.319329	0.24234	<0.005	<0.005	0.005801	0.377195
0.876393	0.783693	0.119123	0.291405	0.461676	<0.005	<0.005	<0.005	1.000517
0.149454	0.539008	0.078254	0.332216	0.253245	<0.005	<0.005	0.006366	0.417611
0.933592	0.820349	0.14179	0.291928	0.458558	<0.005	<0.005	0.00508	1.026093
0.16935	0.585355	0.084439	0.349477	0.274699	<0.005	<0.005	0.006515	0.475367
0.995184	0.856309	0.135609	0.297608	0.45301	<0.005	<0.005	0.005129	1.081857
0.19211	0.615221	0.08825	0.351842	0.286403	<0.005	<0.005	0.006375	0.507411
1.108059	0.873698	0.156137	0.293361	0.455985	<0.005	<0.005	<0.005	1.124353
0.217728	0.643809	0.101162	0.353023	0.29665	<0.005	<0.005	0.00674	0.564236
1.175663	0.918288	0.155037	0.285067	0.453692	<0.005	<0.005	<0.005	1.177276
0.255353	0.6926	0.115164	0.370164	0.304248	<0.005	<0.005	0.006476	0.631255
1.233738	0.95633	0.170952	0.284489	0.454741	<0.005	<0.005	<0.005	1.223736
0.316943	0.771592	0.109956	0.362481	0.32224	<0.005	<0.005	0.00703	0.763071
1.421768	1.0231	0.152604	0.271598	0.459714	<0.005	<0.005	<0.005	1.348552
0.173316	0.568981	0.083572	0.33272	0.267234	<0.005	<0.005	0.006306	0.473981
1.052441	0.869383	0.14259	0.290452	0.456855	<0.005	<0.005	<0.005	1.119243
0.442301	0.660895	0.10163	0.319787	0.325252	<0.005	<0.005	0.00586	0.671411
0	0	0	0	0	0	0	0	0
0	0	0	0	0	0	0	0	0
0	0	0	0	0	0	0	0	0
0	0	0	0	0	0	0	0	0
0	0	0	0	0	0	0	0	0
0	0	0	0	0	0	0	0	0
0	0	0	0	0	0	0	0	0
0	0	0	0	0	0	0	0	0
0	0	0	0	0	0	0	0	0
0	0	0	0	0	0	0	0	0
0	0	0	0	0	0	0	0	0
0	0	0	0	0	0	0	0	0
0	0	0	0	0	0	0	0	0
0	0	0	0	0	0	0	0	0

For peer review only

0	0	0	0	0	0	0	0	0
0	0	0	0	0	0	0	0	0
0	0	0	0	0	0	0	0	0
0	0	0	0	0	0	0	0	0
0	0	0	0	0	0	0	0	0
0	0	0	0	0	0	0	0	0
0	0	0	0	0	0	0	0	0
0	0	0	0	0	0	0	0	0
0	0	0	0	0	0	0	0	0
0	0	0	0	0	0	0	0	0
0	0	0	0	0	0	0	0	0
0	0	0	0	0	0	0	0	0
0	0	0	0	0	0	0	0	0
0	0	0	0	0	0	0	0	0
0	0	0	0	0	0	0	0	0
0	0	0	0	0	0	0	0	0
0	0	0	0	0	0	0	0	0
0	0	0	0	0	0	0	0	0
0	0	0	0	0	0	0	0	0
0	0	0	0	0	0	0	0	0
0	0	0	0	0	0	0	0	0
0	0	0	0	0	0	0	0	0
0	0	0	0	0	0	0	0	0
0	0	0	0	0	0	0	0	0
0	0	0	0	0	0	0	0	0
0	0	0	0	0	0	0	0	0
0	0	0	0	0	0	0	0	0
0	0	0	0	0	0	0	0	0
0	0	0	0	0	0	0	0	0
0	0	0	0	0	0	0	0	0
0	0	0	0	0	0	0	0	0
0	0	0	0	0	0	0	0	0
0	0	0	0	0	0	0	0	0
0	0	0	0	0	0	0	0	0
0	0	0	0	0	0	0	0	0
0	0	0	0	0	0	0	0	0
0	0	0	0	0	0	0	0	0
0	0	0	0	0	0	0	0	0
0	0	0	0	0	0	0	0	0
0	0	0	0	0	0	0	0	0
0	0	0	0	0	0	0	0	0
0	0	0	0	0	0	0	0	0
0	0	0	0	0	0	0	0	0
0	0	0	0	0	0	0	0	0
0	0	0	0	0	0	0	0	0
0	0	0	0	0	0	0	0	0
0	0	0	0	0	0	0	0	0
0	0	0	0	0	0	0	0	0
0	0	0	0	0	0	0	0	0
0	0	0	0	0	0	0	0	0

For peer review only

0	0	0	0	0	0	0	0	0
0	0	0	0	0	0	0	0	0
0	0	0	0	0	0	0	0	0
0.652815	1.517797	0.421715	0.883727	0.833143	0	0	0.122668	0.966247
2.112886	1.923708	1.051587	1.157877	1.302078	0	0	0.142078	1.870596
0.682466	1.401731	0.468593	0.981801	1.046524	0	0.003819	0.137978	1.145634
2.255003	2.126606	0.723463	1.084323	2.456414	0	0	0.128905	1.840903
0.721251	1.372239	0.495868	1.239466	0.852438	0	0	0.140718	1.083048
2.454465	1.960216	1.155292	0.933473	1.513284	0	0	0.167113	1.899766
0.783967	1.411689	0.450412	1.096371	0.855711	0	0	0.126045	1.106398
2.392983	2.009523	0.615244	0.897902	3.395122	0	0	0.176752	2.061826
1.010021	1.439853	0.741578	1.008903	0.975849	0	0.003938	0.159106	1.234371
2.854021	2.010175	1.128982	0.916414	4.268842	0	0	0.115061	1.898868
0.930506	1.554899	0.551886	1.372313	0.940704	0	0	0.149946	1.405429
2.683278	2.122427	0.892843	0.954391	1.494337	0	0	0.12775	2.002556
0.932285	1.597401	0.576757	1.134737	0.906604	0	0	0.133918	1.375213
3.008426	2.162549	0.946319	0.907705	3.525022	0	0	0.09799	2.478793
1.137399	1.731373	1.374992	1.161053	0.992179	0	0	0.137357	1.582533
3.028649	2.268945	1.712743	0.885946	1.34979	0	0.005429	0.085414	2.766562
1.232513	1.769006	1.662386	1.198346	1.027844	0	0	0.151511	1.775059
3.351433	2.312607	3.459342	1.430406	1.427267	0	0.021984	0.190384	2.472362
1.529114	2.051147	1.625881	1.298484	1.096122	0	0	0.147531	2.375456
3.683731	2.521988	0.9954	0.881289	1.412724	0	0	0.087174	2.789855
0.979422	1.585925	0.935821	1.140629	0.953177	0	0.00178	0.140777	1.435829
2.914442	2.17945	1.593329	1.020296	2.433226	0	0.00799	0.134731	2.303514
0.72109	1.218097	0.382294	2.478803	0.930838	0.089822	0.00359	0.219334	0.987924
2.167329	1.684347	0.614509	1.722866	1.266272	0.017528	0	0.129203	1.690673
0.795336	1.301211	0.494954	1.482452	0.979662	0.092034	0	0.119196	1.1288
2.207334	1.936438	1.269844	1.415297	1.415421	0.024166	0	0.088723	1.813781
0.836271	1.433258	0.434308	1.350182	0.876618	0.083631	0	0.153716	1.133847
2.205972	1.775914	0.609167	1.070776	1.519992	0.025559	0	0.107537	1.862705
0.898315	1.423929	0.521231	1.317526	0.943291	0.099137	0	0.135721	1.192197
2.383527	1.716992	0.777358	1.334759	4.314707	0.01313	0	0.101824	1.774899
0.921587	1.426723	0.597884	2.172975	0.910433	0.089439	0	0.135194	1.270178
2.442602	1.843041	2.965403	1.199804	3.151915	0.013841	0	0.147273	1.789454
1.027899	1.575103	0.765534	1.571088	1.092594	0.08691	0	0.134904	1.370871
2.711194	2.626119	0.783994	1.239248	3.262258	0.015867	0	0.166092	2.03061
1.261428	1.675143	0.579802	2.015062	2.126381	0.079036	0.003692	0.139021	1.451518
3.10808	2.286125	2.523567	1.918958	1.349405	0.011832	0	0.17796	2.022224
1.286477	1.691271	1.364564	1.894098	1.232514	0.07992	0	0.122913	1.552252
3.227416	3.154326	1.887995	1.431505	1.362653	0.012569	0	0.088797	2.086734
1.526945	1.768019	2.206822	2.877347	1.103227	0.077417	0	0.112852	1.717121
3.315194	2.345742	1.976407	1.919271	1.389957	0.027226	0.005057	0.114452	2.198046
1.758752	2.206486	0.667961	2.00773	1.172344	0.09053	0	0.14833	2.199161
3.931403	2.151794	1.95585	1.438418	1.481314	0.017776	0	0.116238	2.377977
1.132428	1.581662	0.948623	1.967317	1.186826	0.087104	0.001658	0.145469	1.424145
2.927259	2.253336	1.802843	1.511628	2.268316	0.018622	0.001776	0.128042	2.009902
0.690277	1.365474	0.401087	1.912908	0.887264	0.065859	0.002629	0.181054	0.977941
2.1429	1.800443	0.846165	1.487734	1.283213	0.012825	0	0.135337	1.776942
0.744848	1.349156	0.482857	1.274292	1.011791	0.06731	0.002607	0.128295	1.136675

For peer review only

2.230348	2.028102	1.051249	1.272629	1.970483	0.017663	0	0.109298	1.826542
0.7847	1.405183	0.46406	1.299901	0.865947	0.061136	0	0.147792	1.110435
2.324794	1.863222	0.901551	1.011072	1.517187	0.018811	0	0.138077	1.879839
0.84637	1.418192	0.48916	1.218717	0.903778	0.07216	0	0.131251	1.152597
2.388562	1.859445	0.706656	1.152932	3.914242	0.009605	0	0.141679	1.913924
0.963892	1.432867	0.66884	1.730162	0.942173	0.065356	0.002691	0.146846	1.253609
2.644623	1.924063	2.293822	1.07677	3.718714	0.010079	0	0.133114	1.84184
0.98406	1.565805	0.67517	1.482916	1.026	0.063778	0	0.14206	1.387025
2.698073	2.400004	0.837573	1.113568	2.576708	0.011486	0	0.149076	2.017402
1.122376	1.639962	0.578434	1.670015	1.680701	0.058167	0.002715	0.1367	1.417138
3.061884	2.229407	1.950341	1.531428	2.605855	0.008625	0	0.146042	2.247924
1.220454	1.709828	1.369357	1.6007	1.129686	0.058826	0	0.129745	1.566342
3.13519	2.772165	1.807415	1.206186	1.35659	0.009135	0.003729	0.087217	2.431648
1.398916	1.7687	1.974075	2.263269	1.070156	0.05684	0	0.132122	1.744192
3.332384	2.330391	2.779059	1.706059	1.407739	0.019756	0.015566	0.155113	2.332179
1.656701	2.135991	1.211616	1.715963	1.139466	0.066283	0	0.147959	2.283167
3.813811	2.338759	1.566611	1.202739	1.448956	0.012773	0	0.103206	2.585661
1.064176	1.583644	0.942706	1.636814	1.08538	0.063813	0.001716	0.143309	1.429635
2.921402	2.219363	1.707447	1.304435	2.347394	0.013549	0.005631	0.131233	2.153308
1.887168	1.807433	1.228631	1.542861	1.584677	0.053703	0.003427	0.139727	1.710442
6914	30125	4444	16384	13170	<10	<10	365	22365
16314	15799	2321	5078	9330	<10	<10	132	19927
8010	34874	4961	17950	14615	<10	<10	408	25988
18320	18164	2600	5483	10740	<10	<10	118	22142
8384	33508	4562	19301	13783	<10	<10	407	24466
21991	19286	3007	5804	11391	<10	<10	146	24295
8503	33784	5164	19018	13572	<10	<10	367	24383
23594	21169	2843	6443	12351	<10	<10	154	26125
9677	34949	5123	18859	13959	<10	<10	417	26329
27803	24306	3445	6902	13589	<10	<10	140	29253
10246	36110	5462	19390	14359	<10	<10	433	28845
29593	26119	4012	7316	13475	<10	<10	170	31875
11075	37281	5906	18843	14462	<10	<10	384	30175
35817	28849	4473	7583	14617	<10	<10	139	36376
12039	38735	6291	18114	14813	<10	<10	454	33292
40151	32161	5377	7975	15448	<10	<10	132	41453
13337	38987	6892	17639	14851	<10	<10	379	36162
43559	35018	5961	8151	15939	<10	<10	192	44247
14078	39178	6212	15788	13465	<10	<10	362	38670
49026	38297	4957	7738	15064	<10	<10	149	49301
102263	357531	55017	181286	141049	<10	<10	3976	290675
306168	259168	38996	68473	131944	<10	<10	1472	324994
7762	30923	4299	24622	19285	487	<10	532	25173
16251	15565	2657	7594	9730	<10	<10	133	21204
8972	36697	5087	26246	21284	474	<10	426	29688
18312	17963	3000	8411	10244	<10	<10	89	24484
9083	36100	4798	25635	20323	408	<10	453	28626
21156	20211	3227	9245	11847	10	<10	107	27786
10065	36742	5163	25902	20518	462	<10	449	28677
23903	21304	3613	9350	12670	<10	<10	95	28099

10959	39475	5682	27012	21008	411	<10	462	31333
27334	24143	4929	10339	13493	<10	<10	160	31347
12682	43140	5970	27925	22832	418	<10	449	35514
30745	25799	4210	10728	13991	<10	<10	141	33718
14991	46194	6068	28896	24398	356	<10	481	38672
38649	29867	6020	12132	16027	<10	<10	196	39185
17036	47238	7218	29028	24801	345	<10	446	42055
44378	33863	5770	12521	17172	<10	<10	148	43192
19235	49359	7798	29578	23958	337	<10	447	44359
48088	36022	6738	12982	17841	21	<10	169	46657
22483	49829	6472	26026	23707	372	<10	449	49354
55545	36952	6267	12238	18748	<10	<10	194	49885
133268	415697	58555	270870	222114	4070	<10	4594	353451
324361	261689	46431	105540	141763	82	<10	1432	345557
14676	61048	8743	41006	32455	487	<10	897	47538
32565	31364	4978	12672	19060	<10	<10	265	41131
16982	71571	10048	44196	35899	474	<10	834	55676
36632	36127	5600	13894	20984	<10	<10	207	46626
17467	69608	9360	44936	34106	408	<10	860	53092
43147	39497	6234	15049	23238	10	<10	253	52081
18568	70526	10327	44920	34090	462	<10	816	53060
47497	42473	6456	15793	25021	<10	<10	249	54224
20636	74424	10805	45871	34967	411	<10	879	57662
55137	48449	8374	17241	27082	<10	<10	300	60600
22928	79250	11432	47315	37191	418	<10	882	64359
60338	51918	8222	18044	27466	<10	<10	311	65593
26066	83475	11974	47739	38860	356	<10	865	68847
74466	58716	10493	19715	30644	<10	<10	335	75561
29075	85973	13509	47142	39614	345	<10	900	75347
84529	66024	11147	20496	32620	<10	<10	280	84645
32572	88346	14690	47217	38809	337	<10	826	80521
91647	71040	12699	21133	33780	21	<10	361	90904
36561	89007	12684	41814	37172	372	<10	811	88024
104571	75249	11224	19976	33812	<10	<10	343	99186
235531	773228	113572	452156	363163	4070	<10	8570	644126
630529	520857	85427	174013	273707	82	<10	2904	670551
866060	1294085	198999	626169	636870	4152	11	11474	1314677

For peer review only

S	T	W	Z
4597	2569	<10	7030
3009	1829	<10	3399
4984	2815	<10	7987
3272	1963	<10	3833
5257	2817	<10	7671
3793	2207	<10	4382
5721	3055	<10	7823
4054	2356	<10	4551
5921	3034	<10	7860
4431	2507	<10	5135
5864	3058	<10	7965
4672	2684	<10	5294
6105	3400	<10	8007
5219	2869	<10	5601
6517	3437	<10	7687
5561	3108	<10	5882
6572	3382	<10	7705
6251	3287	<10	5903
6984	3377	<10	6971
7081	3381	<10	5690
58522	30944	<10	76706
47343	26191	<10	49670
6576	2991	<10	6703
6528	1670	<10	2874
6769	3103	<10	7876
6819	1799	<10	3203
6623	3159	<10	7621
7897	1965	<10	3834
6814	3268	<10	7587
8075	2084	<10	3920
6937	3483	<10	8237
8486	2303	<10	4388
6949	3705	<10	8489
8128	2424	<10	4356
7419	4016	<10	8773
9176	2625	<10	4931
7409	4146	<10	8569
9610	2840	<10	4972
7232	4206	<10	8681
9617	2833	<10	5016
6921	4165	<10	7985
9284	3017	<10	4685
69649	36242	<10	80521
83620	23560	<10	42179
11173	5560	<10	13733
9537	3499	<10	6273
11753	5918	<10	15863
10091	3762	<10	7036

For peer review only

11880	5976	<10	15292
11690	4172	<10	8216
12535	6323	<10	15410
12129	4440	<10	8471
12858	6517	<10	16097
12917	4810	<10	9523
12813	6763	<10	16454
12800	5108	<10	9650
13524	7416	<10	16780
14395	5494	<10	10532
13926	7583	<10	16256
15171	5948	<10	10854
13804	7588	<10	16386
15868	6120	<10	10919
13905	7542	<10	14956
16365	6398	<10	10375
128171	67186	<10	157227
130963	49751	<10	91849
259134	116937	<10	249076
0.097708	0.06669	<0.005	0.275721
0.289991	0.191119	<0.005	0.337329
0.099959	0.074193	<0.005	0.288904
0.294202	0.190825	<0.005	0.346824
0.114278	0.075116	<0.005	0.284542
0.307656	0.183187	<0.005	0.348016
0.124838	0.083738	<0.005	0.283742
0.302096	0.187108	<0.005	0.333849
0.135017	0.085483	<0.005	0.300495
0.304859	0.177191	<0.005	0.340542
0.140154	0.088576	<0.005	0.314319
0.307439	0.184055	<0.005	0.344513
0.148203	0.10053	<0.005	0.314253
0.316356	0.183628	<0.005	0.329089
0.167977	0.104704	<0.005	0.299925
0.321189	0.185385	<0.005	0.325787
0.178117	0.109416	<0.005	0.318391
0.33864	0.181317	<0.005	0.309359
0.215905	0.124021	<0.005	0.315423
0.387071	0.189376	<0.005	0.287521
0.139927	0.090167	<0.005	0.299173
0.321112	0.18502	<0.005	0.327574
0.132912	0.065812	<0.005	0.211849
0.598867	0.143691	<0.005	0.207744
0.128438	0.06995	<0.005	0.231395
0.587592	0.149068	<0.005	0.229171
0.134192	0.073973	<0.005	0.232624
0.57637	0.134519	<0.005	0.228022
0.142314	0.077697	<0.005	0.229735
0.549204	0.137508	<0.005	0.221502
0.145186	0.085685	<0.005	0.244656

For peer review only

0.545426	0.138688	<0.005	0.224717
0.153001	0.094937	<0.005	0.253941
0.530391	0.145016	<0.005	0.228462
0.16248	0.10215	<0.005	0.262989
0.523508	0.145193	<0.005	0.233738
0.16735	0.10865	<0.005	0.251419
0.518394	0.145964	<0.005	0.221725
0.172128	0.117549	<0.005	0.269329
0.496701	0.141608	<0.005	0.214048
0.187633	0.135989	<0.005	0.275468
0.492257	0.163208	<0.005	0.204161
0.151504	0.091975	<0.005	0.246183
0.5359	0.144895	<0.005	0.221084
0.116591	0.066219	<0.005	0.24132
0.455336	0.165731	<0.005	0.267961
0.115162	0.071928	<0.005	0.258037
0.450917	0.16852	<0.005	0.283979
0.124899	0.074507	<0.005	0.256689
0.4532	0.156827	<0.005	0.283024
0.13408	0.080543	<0.005	0.254884
0.434331	0.160565	<0.005	0.273729
0.140437	0.085591	<0.005	0.270381
0.432415	0.156775	<0.005	0.279128
0.147059	0.091995	<0.005	0.281325
0.424262	0.163599	<0.005	0.283704
0.155922	0.101405	<0.005	0.286039
0.426433	0.163205	<0.005	0.278421
0.167638	0.106839	<0.005	0.273131
0.425347	0.164564	<0.005	0.270824
0.174894	0.113792	<0.005	0.291273
0.421854	0.160411	<0.005	0.259181
0.20078	0.130423	<0.005	0.293415
0.441373	0.175867	<0.005	0.244487
0.146128	0.091135	<0.005	0.270362
0.434813	0.163779	<0.005	0.271202
0.234457	0.113362	<0.005	0.270689
0	0	0	0
0	0	0	0
0	0	0	0
0	0	0	0
0	0	0	0
0	0	0	0
0	0	0	0
0	0	0	0
0	0	0	0
0	0	0	0
0	0	0	0
0	0	0	0
0	0	0	0
0	0	0	0

For peer review only

0	0	0	0
0	0	0	0
0	0	0	0
0	0	0	0
0	0	0	0
0	0	0	0
0	0	0	0
0	0	0	0
0	0	0	0
0	0	0	0
0	0	0	0
0	0	0	0
0	0	0	0
0	0	0	0
0	0	0	0
0	0	0	0
0	0	0	0
0	0	0	0
0	0	0	0
0	0	0	0
0	0	0	0
0	0	0	0
0	0	0	0
0	0	0	0
0	0	0	0
0	0	0	0
0	0	0	0
0	0	0	0
0	0	0	0
0	0	0	0
0	0	0	0
0	0	0	0
0	0	0	0
0	0	0	0
0	0	0	0
0	0	0	0
0	0	0	0
0	0	0	0
0	0	0	0
0	0	0	0
0	0	0	0
0	0	0	0
0	0	0	0
0	0	0	0
0	0	0	0
0	0	0	0
0	0	0	0
0	0	0	0
0	0	0	0
0	0	0	0

For peer review only

0	0	0	0
0	0	0	0
0	0	0	0
0.452561	0.501099	0	1.015114
0.883435	0.859217	0	1.17199
0.444013	0.579965	0	1.016633
0.875295	0.91519	0	1.142975
0.48958	0.503629	0	0.998756
0.91601	0.773771	0	1.118949
0.517787	0.700835	0	0.943663
0.895743	0.856374	0	1.118775
0.582714	0.577048	0	1.04093
0.925037	0.817414	0	1.182216
0.551007	0.599379	0	1.057303
0.919397	0.836003	0	1.402595
0.584068	0.623172	0	1.066382
0.928218	0.829354	0	1.126752
0.655159	0.641227	0	1.020794
0.972431	0.893714	0	1.152691
0.673613	0.664812	0	1.088933
0.977706	0.847549	0	1.113713
0.774231	0.76413	0	1.111306
1.06189	0.841695	0.005301	1.028374
0.575382	0.616805	0	1.035528
0.946384	0.847414	0.001883	1.156544
0.555135	0.473955	0	0.843832
1.264402	0.797015	0	0.805896
0.524303	0.535171	0	0.840878
1.237932	0.809716	0.006459	0.942173
0.540335	0.550836	0	0.879939
1.187445	0.740187	0	0.851566
0.574892	0.544419	0	0.803126
1.144657	0.688806	0	0.882908
0.594744	0.572889	0	0.79594
1.164369	0.687963	0	0.870149
0.61597	0.601573	0	0.847763
1.193543	0.736007	0.00561	0.867729
0.638488	0.730223	0	0.887514
1.176618	0.736232	0	0.950438
0.643939	0.720277	0	0.830772
1.164566	0.800766	0	0.897453
0.659376	0.743708	0	0.91776
1.151351	0.814308	0	0.895907
0.745563	0.857604	0	0.955907
1.145206	0.816963	0	0.837557
0.608903	0.637149	0	0.860874
1.178711	0.765953	0.002511	0.882827
0.510452	0.486726	0	0.927346
1.114532	0.826831	0	0.995037
0.488723	0.556504	0	0.926893

For peer review only

1.094018	0.860704	0.004721	1.042191
0.517363	0.529329	0	0.937242
1.079919	0.756148	0	0.985001
0.548795	0.623033	0	0.871804
1.043701	0.771634	0	1.001063
0.589176	0.574833	0	0.91739
1.065476	0.751796	0	1.030221
0.586848	0.600565	0	0.949025
1.077585	0.785432	0.004061	1.155131
0.61413	0.683138	0	0.972349
1.072421	0.781483	0	1.037885
0.649112	0.685119	0	0.921001
1.082664	0.846118	0	1.027133
0.665994	0.708366	0	0.99825
1.075526	0.830446	0	1.006066
0.759157	0.815488	0	1.028805
1.106927	0.829117	0.003687	0.935663
0.593602	0.627785	0	0.94495
1.080967	0.805567	0.002238	1.022214
0.787254	0.687897	0.001238	0.975805
6552	4472	<10	11821
5734	3779	<10	6670
6855	5088	<10	13185
6150	3989	<10	7250
7517	4941	<10	12451
7173	4271	<10	8114
8274	5550	<10	12519
7611	4714	<10	8411
8706	5512	<10	13162
8458	4916	<10	9448
8777	5547	<10	13489
8873	5312	<10	9943
9236	6265	<10	13544
9963	5783	<10	10364
10298	6419	<10	12763
10896	6289	<10	11052
10495	6447	<10	13083
11912	6378	<10	10882
11582	6653	<10	11946
13772	6738	<10	10230
88292	56894	<10	127963
90542	52169	<10	92364
10312	5106	<10	10602
13641	3273	<10	4732
10086	5493	<10	12235
14084	3573	<10	5493
10088	5561	<10	11781
15879	3706	<10	6282
10587	5780	<10	11631
15928	3988	<10	6424

For peer review only

10685	6306	<10	12545
17080	4343	<10	7037
11133	6908	<10	13130
16850	4607	<10	7258
11920	7494	<10	13874
18695	5185	<10	8347
12088	7848	<10	13203
19686	5543	<10	8420
11814	8068	<10	13676
19425	5538	<10	8371
11579	8392	<10	12793
18691	6197	<10	7752
110292	66956	<10	125470
169959	45953	<10	70116
16864	9578	<10	22423
19375	7052	<10	11402
16941	10581	<10	25420
20234	7562	<10	12743
17605	10502	<10	24232
23052	7977	<10	14396
18861	11330	<10	24150
23539	8702	<10	14835
19391	11818	<10	25707
25538	9259	<10	16485
19910	12455	<10	26619
25723	9919	<10	17201
21156	13759	<10	27418
28658	10968	<10	18711
22386	14267	<10	25966
30582	11832	<10	19472
22309	14515	<10	26759
31337	11916	<10	19253
23161	15045	<10	24739
32463	12935	<10	17982
198584	123850	<10	253433
260501	98122	<10	162480
459085	221972	<10	415913

For peer review only

code_block	code	cases
A00-A09	A00	<10
A00-A09	A01	61
A00-A09	A02	741
A00-A09	A03	51
A00-A09	A04	14398
A00-A09	A05	160
A00-A09	A06	39
A00-A09	A07	136
A00-A09	A08	7594
A00-A09	A09	21348
A15-A19	A15	798
A15-A19	A16	1305
A15-A19	A17	119
A15-A19	A18	474
A15-A19	A19	101
A20-A28	A20	<10
A20-A28	A21	<10
A20-A28	A22	<10
A20-A28	A23	<10
A20-A28	A24	<10
A20-A28	A25	<10
A20-A28	A26	<10
A20-A28	A27	21
A20-A28	A28	<10
A30-A49	A30	<10
A30-A49	A31	809
A30-A49	A32	139
A30-A49	A33	<10
A30-A49	A34	<10
A30-A49	A35	<10
A30-A49	A36	<10
A30-A49	A37	15
A30-A49	A38	12
A30-A49	A39	292
A30-A49	A40	1812
A30-A49	A41	59137
A30-A49	A42	111
A30-A49	A43	<10
A30-A49	A44	<10
A30-A49	A45	<10
A30-A49	A46	718
A30-A49	A47	<10

For peer review only

A30-A49	A48	757
A30-A49	A49	3528
A50-A64	A50	<10
A50-A64	A51	17
A50-A64	A52	163
A50-A64	A53	99
A50-A64	A54	22
A50-A64	A55	<10
A50-A64	A56	<10
A50-A64	A57	<10
A50-A64	A58	<10
A50-A64	A59	<10
A50-A64	A60	39
A50-A64	A61	<10
A50-A64	A62	<10
A50-A64	A63	774
A50-A64	A64	<10
A65-A69	A65	<10
A65-A69	A66	<10
A65-A69	A67	<10
A65-A69	A68	<10
A65-A69	A69	366
A70-A74	A70	13
A70-A74	A71	<10
A70-A74	A72	<10
A70-A74	A73	<10
A70-A74	A74	<10
A75-A79	A75	<10
A75-A79	A76	<10
A75-A79	A77	<10
A75-A79	A78	<10
A75-A79	A79	<10
A80-A89	A80	<10
A80-A89	A81	253
A80-A89	A82	<10
A80-A89	A83	<10
A80-A89	A84	<10
A80-A89	A85	19
A80-A89	A86	428
A80-A89	A87	538
A80-A89	A88	<10
A80-A89	A89	30
A90-A99	A90	26

For peer review only

A90-A99	A91	<10
A90-A99	A92	<10
A90-A99	A93	<10
A90-A99	A94	<10
A90-A99	A95	<10
A90-A99	A96	<10
A90-A99	A97	<10
A90-A99	A98	<10
A90-A99	A99	<10
B00-B09	B00	862
B00-B09	B01	331
B00-B09	B02	4021
B00-B09	B03	<10
B00-B09	B04	<10
B00-B09	B05	<10
B00-B09	B06	<10
B00-B09	B07	1228
B00-B09	B08	83
B00-B09	B09	29
B15-B19	B15	56
B15-B19	B16	170
B15-B19	B17	441
B15-B19	B18	894
B15-B19	B19	102
B20-B24	B20	898
B20-B24	B21	228
B20-B24	B22	196
B20-B24	B23	339
B20-B24	B24	561
B25-B34	B25	242
B25-B34	B26	40
B25-B34	B27	119
B25-B34	B28	<10
B25-B34	B29	<10
B25-B34	B30	29
B25-B34	B31	<10
B25-B34	B32	<10
B25-B34	B33	54
B25-B34	B34	7442
B35-B49	B35	259
B35-B49	B36	35
B35-B49	B37	2789
B35-B49	B38	<10

For peer review only

B35-B49	B39	<10
B35-B49	B40	<10
B35-B49	B41	<10
B35-B49	B42	<10
B35-B49	B43	<10
B35-B49	B44	917
B35-B49	B45	25
B35-B49	B46	<10
B35-B49	B47	36
B35-B49	B48	<10
B35-B49	B49	27
B50-B64	B50	102
B50-B64	B51	21
B50-B64	B52	<10
B50-B64	B53	<10
B50-B64	B54	31
B50-B64	B55	<10
B50-B64	B56	<10
B50-B64	B57	<10
B50-B64	B58	38
B50-B64	B59	413
B50-B64	B60	27
B50-B64	B61	<10
B50-B64	B62	<10
B50-B64	B63	<10
B50-B64	B64	<10
B65-B83	B65	<10
B65-B83	B66	<10
B65-B83	B67	12
B65-B83	B68	<10
B65-B83	B69	<10
B65-B83	B70	<10
B65-B83	B71	<10
B65-B83	B72	<10
B65-B83	B73	<10
B65-B83	B74	<10
B65-B83	B75	<10
B65-B83	B76	<10
B65-B83	B77	<10
B65-B83	B78	<10
B65-B83	B79	<10
B65-B83	B80	75
B65-B83	B81	<10

For peer review only

B65-B83	B82	15
B65-B83	B83	14
B85-B89	B85	<10
B85-B89	B86	66
B85-B89	B87	<10
B85-B89	B88	<10
B85-B89	B89	<10
B95-B98	B95	<10
B95-B98	B96	<10
B95-B98	B97	<10
B95-B98	B98	<10
B99-B99	B99	337
C00-C14	C00	600
C00-C14	C01	3393
C00-C14	C02	4936
C00-C14	C03	669
C00-C14	C04	2675
C00-C14	C05	1534
C00-C14	C06	2317
C00-C14	C07	1180
C00-C14	C08	398
C00-C14	C09	3780
C00-C14	C10	3029
C00-C14	C11	1236
C00-C14	C12	1907
C00-C14	C13	1739
C00-C14	C14	1984
C15-C26	C15	64713
C15-C26	C16	45622
C15-C26	C17	4248
C15-C26	C18	148739
C15-C26	C19	33263
C15-C26	C20	62367
C15-C26	C21	3890
C15-C26	C22	16066
C15-C26	C23	2790
C15-C26	C24	4795
C15-C26	C25	40633
C15-C26	C26	2466
C30-C39	C30	653
C30-C39	C31	844
C30-C39	C32	11590
C30-C39	C33	302

For peer review only

C30-C39	C34	207455
C30-C39	C35	<10
C30-C39	C36	<10
C30-C39	C37	426
C30-C39	C38	757
C30-C39	C39	23
C40-C41	C40	460
C40-C41	C41	1853
C43-C44	C43	11618
C43-C44	C44	54189
C45-C49	C45	8961
C45-C49	C46	140
C45-C49	C47	220
C45-C49	C48	6732
C45-C49	C49	4676
C50-C50	C50	230331
C51-C58	C51	4015
C51-C58	C52	1427
C51-C58	C53	12393
C51-C58	C54	21614
C51-C58	C55	2368
C51-C58	C56	67234
C51-C58	C57	993
C51-C58	C58	35
C60-C63	C60	1614
C60-C63	C61	62292
C60-C63	C62	2342
C60-C63	C63	131
C64-C68	C64	21359
C64-C68	C65	1624
C64-C68	C66	1785
C64-C68	C67	57717
C64-C68	C68	562
C69-C72	C69	1176
C69-C72	C70	279
C69-C72	C71	15898
C69-C72	C72	275
C73-C75	C73	3695
C73-C75	C74	689
C73-C75	C75	560
C76-C80	C76	2462
C76-C80	C77	8892
C76-C80	C78	45065

For peer review only

C76-C80	C79	39153
C76-C80	C80	20089
C81-C96	C81	9092
C81-C96	C82	20760
C81-C96	C83	33853
C81-C96	C84	4401
C81-C96	C85	40124
C81-C96	C86	526
C81-C96	C87	<10
C81-C96	C88	4846
C81-C96	C89	<10
C81-C96	C90	88383
C81-C96	C91	38832
C81-C96	C92	39801
C81-C96	C93	950
C81-C96	C94	759
C81-C96	C95	602
C81-C96	C96	229
C97-C97	C97	49
D00-D09	D00	945
D00-D09	D01	689
D00-D09	D02	1191
D00-D09	D03	2279
D00-D09	D04	2430
D00-D09	D05	5369
D00-D09	D06	580
D00-D09	D07	1998
D00-D09	D08	<10
D00-D09	D09	4379
D10-D36	D10	1456
D10-D36	D11	2221
D10-D36	D12	62890
D10-D36	D13	4961
D10-D36	D14	1926
D10-D36	D15	751
D10-D36	D16	727
D10-D36	D17	13392
D10-D36	D18	3327
D10-D36	D19	43
D10-D36	D20	70
D10-D36	D21	1388
D10-D36	D22	4922
D10-D36	D23	7143

For peer review only

D10-D36	D24	3472
D10-D36	D25	11816
D10-D36	D26	561
D10-D36	D27	4312
D10-D36	D28	334
D10-D36	D29	326
D10-D36	D30	978
D10-D36	D31	126
D10-D36	D32	4464
D10-D36	D33	1352
D10-D36	D34	809
D10-D36	D35	3410
D10-D36	D36	1975
D37-D48	D37	4921
D37-D48	D38	1248
D37-D48	D39	617
D37-D48	D40	209
D37-D48	D41	27615
D37-D48	D42	117
D37-D48	D43	1589
D37-D48	D44	558
D37-D48	D45	13580
D37-D48	D46	48236
D37-D48	D47	17317
D37-D48	D48	2027
D50-D53	D50	54494
D50-D53	D51	1368
D50-D53	D52	2161
D50-D53	D53	698
D55-D59	D55	33
D55-D59	D56	110
D55-D59	D57	37
D55-D59	D58	658
D55-D59	D59	4905
D60-D64	D60	1142
D60-D64	D61	9688
D60-D64	D62	135
D60-D64	D63	679
D60-D64	D64	67005
D65-D69	D65	166
D65-D69	D66	995
D65-D69	D67	175
D65-D69	D68	2853

For peer review only

D65-D69	D69	11338
D70-D77	D70	9311
D70-D77	D71	22
D70-D77	D72	575
D70-D77	D73	596
D70-D77	D74	19
D70-D77	D75	7842
D70-D77	D76	253
D70-D77	D77	<10
D80-D89	D80	4390
D80-D89	D81	66
D80-D89	D82	236
D80-D89	D83	2783
D80-D89	D84	563
D80-D89	D85	<10
D80-D89	D86	1783
D80-D89	D87	<10
D80-D89	D88	<10
D80-D89	D89	1883
E00-E07	E00	<10
E00-E07	E01	24
E00-E07	E02	<10
E00-E07	E03	985
E00-E07	E04	3761
E00-E07	E05	2536
E00-E07	E06	336
E00-E07	E07	229
E10-E14	E10	14740
E10-E14	E11	22918
E10-E14	E12	<10
E10-E14	E13	176
E10-E14	E14	3406
E15-E16	E15	82
E15-E16	E16	12554
E20-E35	E20	75
E20-E35	E21	1694
E20-E35	E22	1362
E20-E35	E23	640
E20-E35	E24	464
E20-E35	E25	<10
E20-E35	E26	124
E20-E35	E27	1248
E20-E35	E28	41

For peer review only

E20-E35	E29	460
E20-E35	E30	<10
E20-E35	E31	<10
E20-E35	E32	88
E20-E35	E33	<10
E20-E35	E34	292
E20-E35	E35	<10
E40-E46	E40	<10
E40-E46	E41	14
E40-E46	E42	<10
E40-E46	E43	56
E40-E46	E44	13
E40-E46	E45	<10
E40-E46	E46	839
E50-E64	E50	<10
E50-E64	E51	239
E50-E64	E52	<10
E50-E64	E53	1140
E50-E64	E54	<10
E50-E64	E55	58
E50-E64	E56	33
E50-E64	E57	<10
E50-E64	E58	17
E50-E64	E59	<10
E50-E64	E60	<10
E50-E64	E61	1001
E50-E64	E62	<10
E50-E64	E63	77
E50-E64	E64	<10
E65-E68	E65	122
E65-E68	E66	2216
E65-E68	E67	<10
E65-E68	E68	<10
E70-E90	E70	17
E70-E90	E71	20
E70-E90	E72	68
E70-E90	E73	40
E70-E90	E74	83
E70-E90	E75	107
E70-E90	E76	<10
E70-E90	E77	11
E70-E90	E78	513
E70-E90	E79	<10

For peer review only

E70-E90	E80	539
E70-E90	E81	<10
E70-E90	E82	<10
E70-E90	E83	60601
E70-E90	E84	395
E70-E90	E85	1311
E70-E90	E86	11524
E70-E90	E87	22513
E70-E90	E88	417
E70-E90	E89	126
E70-E90	E90	<10
F00-F09	F00	31
F00-F09	F01	5843
F00-F09	F02	14
F00-F09	F03	12131
F00-F09	F04	17
F00-F09	F05	19521
F00-F09	F06	2237
F00-F09	F07	108
F00-F09	F08	<10
F00-F09	F09	20
F10-F19	F10	36876
F10-F19	F11	200
F10-F19	F12	<10
F10-F19	F13	72
F10-F19	F14	<10
F10-F19	F15	23
F10-F19	F16	<10
F10-F19	F17	41
F10-F19	F18	<10
F10-F19	F19	53
F20-F29	F20	344
F20-F29	F21	<10
F20-F29	F22	177
F20-F29	F23	206
F20-F29	F24	<10
F20-F29	F25	104
F20-F29	F26	<10
F20-F29	F27	<10
F20-F29	F28	24
F20-F29	F29	235
F30-F39	F30	134
F30-F39	F31	305

For peer review only

F30-F39	F32	2675
F30-F39	F33	336
F30-F39	F34	29
F30-F39	F35	<10
F30-F39	F36	<10
F30-F39	F37	<10
F30-F39	F38	10
F30-F39	F39	17
F40-F48	F40	20
F40-F48	F41	4692
F40-F48	F42	15
F40-F48	F43	176
F40-F48	F44	296
F40-F48	F45	1107
F40-F48	F46	<10
F40-F48	F47	<10
F40-F48	F48	37
F50-F59	F50	154
F50-F59	F51	26
F50-F59	F52	131
F50-F59	F53	<10
F50-F59	F54	<10
F50-F59	F55	<10
F50-F59	F56	<10
F50-F59	F57	<10
F50-F59	F58	<10
F50-F59	F59	<10
F60-F69	F60	69
F60-F69	F61	<10
F60-F69	F62	11
F60-F69	F63	<10
F60-F69	F64	13
F60-F69	F65	<10
F60-F69	F66	<10
F60-F69	F67	<10
F60-F69	F68	<10
F60-F69	F69	19
F70-F79	F70	13
F70-F79	F71	<10
F70-F79	F72	<10
F70-F79	F73	<10
F70-F79	F74	<10
F70-F79	F75	<10

For peer review only

F70-F79	F76	<10
F70-F79	F77	<10
F70-F79	F78	<10
F70-F79	F79	15
F80-F89	F80	77
F80-F89	F81	23
F80-F89	F82	<10
F80-F89	F83	<10
F80-F89	F84	<10
F80-F89	F85	<10
F80-F89	F86	<10
F80-F89	F87	<10
F80-F89	F88	<10
F80-F89	F89	<10
F90-F98	F90	<10
F90-F98	F91	24
F90-F98	F92	<10
F90-F98	F93	<10
F90-F98	F94	<10
F90-F98	F95	<10
F90-F98	F96	<10
F90-F98	F97	<10
F90-F98	F98	11
F99-F99	F99	37
G00-G09	G00	1102
G00-G09	G01	<10
G00-G09	G02	<10
G00-G09	G03	420
G00-G09	G04	941
G00-G09	G05	<10
G00-G09	G06	1201
G00-G09	G07	<10
G00-G09	G08	218
G00-G09	G09	<10
G10-G13	G10	353
G10-G13	G11	454
G10-G13	G12	4387
G10-G13	G13	<10
G20-G26	G20	9724
G20-G26	G21	369
G20-G26	G22	<10
G20-G26	G23	170
G20-G26	G24	445

For peer review only

G20-G26	G25	1114
G20-G26	G26	<10
G30-G32	G30	4330
G30-G32	G31	3362
G30-G32	G32	<10
G35-G37	G35	16934
G35-G37	G36	73
G35-G37	G37	1839
G40-G47	G40	23766
G40-G47	G41	2591
G40-G47	G42	<10
G40-G47	G43	6460
G40-G47	G44	1789
G40-G47	G45	32971
G40-G47	G46	39
G40-G47	G47	15847
G50-G59	G50	2341
G50-G59	G51	3318
G50-G59	G52	168
G50-G59	G53	<10
G50-G59	G54	1004
G50-G59	G55	163
G50-G59	G56	41073
G50-G59	G57	2967
G50-G59	G58	1209
G50-G59	G59	<10
G60-G64	G60	896
G60-G64	G61	5878
G60-G64	G62	7294
G60-G64	G63	<10
G60-G64	G64	<10
G70-G73	G70	3090
G70-G73	G71	568
G70-G73	G72	564
G70-G73	G73	<10
G80-G83	G80	311
G80-G83	G81	4763
G80-G83	G82	1154
G80-G83	G83	1780
G90-G99	G90	737
G90-G99	G91	1299
G90-G99	G92	29
G90-G99	G93	4315

For peer review only

G90-G99	G94	<10
G90-G99	G95	3757
G90-G99	G96	525
G90-G99	G97	351
G90-G99	G98	124
G90-G99	G99	92
H00-H06	H00	923
H00-H06	H01	601
H00-H06	H02	20890
H00-H06	H03	<10
H00-H06	H04	6139
H00-H06	H05	1044
H00-H06	H06	70
H10-H13	H10	303
H10-H13	H11	1577
H10-H13	H12	<10
H10-H13	H13	<10
H15-H22	H15	248
H15-H22	H16	2751
H15-H22	H17	175
H15-H22	H18	2158
H15-H22	H19	<10
H15-H22	H20	1021
H15-H22	H21	352
H15-H22	H22	<10
H25-H28	H25	36162
H25-H28	H26	283044
H25-H28	H27	604
H25-H28	H28	<10
H30-H36	H30	128
H30-H36	H31	267
H30-H36	H32	<10
H30-H36	H33	8905
H30-H36	H34	1428
H30-H36	H35	23741
H30-H36	H36	507
H40-H42	H40	10279
H40-H42	H41	<10
H40-H42	H42	<10
H43-H45	H43	3417
H43-H45	H44	862
H43-H45	H45	19
H46-H48	H46	240

For peer review only

H46-H48	H47	366
H46-H48	H48	<10
H49-H52	H49	823
H49-H52	H50	1638
H49-H52	H51	94
H49-H52	H52	356
H53-H54	H53	2822
H53-H54	H54	437
H55-H59	H55	76
H55-H59	H56	<10
H55-H59	H57	910
H55-H59	H58	<10
H55-H59	H59	410
H60-H62	H60	1833
H60-H62	H61	2628
H60-H62	H62	<10
H65-H75	H65	2479
H65-H75	H66	2455
H65-H75	H67	<10
H65-H75	H68	78
H65-H75	H69	167
H65-H75	H70	238
H65-H75	H71	1245
H65-H75	H72	2070
H65-H75	H73	431
H65-H75	H74	769
H65-H75	H75	<10
H80-H83	H80	586
H80-H83	H81	2443
H80-H83	H82	<10
H80-H83	H83	3864
H90-H95	H90	791
H90-H95	H91	1229
H90-H95	H92	1177
H90-H95	H93	325
H90-H95	H94	<10
H90-H95	H95	195
I00-I02	I00	28
I00-I02	I01	40
I00-I02	I02	<10
I05-I09	I05	2332
I05-I09	I06	170
I05-I09	I07	191

For peer review only

I05-I09	I08	5145
I05-I09	I09	160
I10-I15	I10	9211
I10-I15	I11	1102
I10-I15	I12	4488
I10-I15	I13	344
I10-I15	I14	<10
I10-I15	I15	86
I20-I25	I20	118638
I20-I25	I21	202146
I20-I25	I22	17939
I20-I25	I23	304
I20-I25	I24	16721
I20-I25	I25	173177
I26-I28	I26	35328
I26-I28	I27	5307
I26-I28	I28	81
I30-I52	I30	1529
I30-I52	I31	4778
I30-I52	I32	<10
I30-I52	I33	3800
I30-I52	I34	7695
I30-I52	I35	34980
I30-I52	I36	168
I30-I52	I37	79
I30-I52	I38	1102
I30-I52	I39	<10
I30-I52	I40	166
I30-I52	I41	<10
I30-I52	I42	6027
I30-I52	I43	11
I30-I52	I44	16546
I30-I52	I45	4403
I30-I52	I46	10938
I30-I52	I47	20735
I30-I52	I48	119012
I30-I52	I49	9265
I30-I52	I50	139263
I30-I52	I51	3702
I30-I52	I52	<10
I60-I69	I60	13545
I60-I69	I61	21689
I60-I69	I62	10954

For peer review only

I60-I69	I63	98595
I60-I69	I64	66512
I60-I69	I65	8453
I60-I69	I66	2288
I60-I69	I67	16855
I60-I69	I68	<10
I60-I69	I69	<10
I70-I79	I70	10337
I70-I79	I71	26253
I70-I79	I72	3723
I70-I79	I73	54493
I70-I79	I74	7634
I70-I79	I75	<10
I70-I79	I76	<10
I70-I79	I77	10398
I70-I79	I78	6099
I70-I79	I79	64
I80-I89	I80	24269
I80-I89	I81	303
I80-I89	I82	1156
I80-I89	I83	35793
I80-I89	I84	44665
I80-I89	I85	13775
I80-I89	I86	1600
I80-I89	I87	2400
I80-I89	I88	216
I80-I89	I89	1727
I95-I99	I95	21488
I95-I99	I96	<10
I95-I99	I97	400
I95-I99	I98	465
I95-I99	I99	1748
J00-J06	J00	179
J00-J06	J01	415
J00-J06	J02	1525
J00-J06	J03	1743
J00-J06	J04	719
J00-J06	J05	422
J00-J06	J06	4633
J09-J18	J09	71
J09-J18	J10	3581
J09-J18	J11	914
J09-J18	J12	715

For peer review only

J09-J18	J13	3705
J09-J18	J14	1247
J09-J18	J15	7603
J09-J18	J16	166
J09-J18	J17	30
J09-J18	J18	222976
J20-J22	J20	955
J20-J22	J21	294
J20-J22	J22	142163
J30-J39	J30	204
J30-J39	J31	1031
J30-J39	J32	3119
J30-J39	J33	8790
J30-J39	J34	11726
J30-J39	J35	1249
J30-J39	J36	1191
J30-J39	J37	669
J30-J39	J38	10097
J30-J39	J39	3391
J40-J47	J40	1332
J40-J47	J41	80
J40-J47	J42	568
J40-J47	J43	4124
J40-J47	J44	272560
J40-J47	J45	31051
J40-J47	J46	1830
J40-J47	J47	10247
J60-J70	J60	41
J60-J70	J61	306
J60-J70	J62	48
J60-J70	J63	<10
J60-J70	J64	44
J60-J70	J65	<10
J60-J70	J66	<10
J60-J70	J67	663
J60-J70	J68	50
J60-J70	J69	29080
J60-J70	J70	356
J80-J84	J80	833
J80-J84	J81	12546
J80-J84	J82	234
J80-J84	J83	<10
J80-J84	J84	13248

For peer review only

J85-J86	J85	769
J85-J86	J86	4185
J90-J94	J90	25422
J90-J94	J91	274
J90-J94	J92	765
J90-J94	J93	8133
J90-J94	J94	1237
J95-J99	J95	1907
J95-J99	J96	15107
J95-J99	J97	<10
J95-J99	J98	10366
J95-J99	J99	15
K00-K14	K00	213
K00-K14	K01	2001
K00-K14	K02	7171
K00-K14	K03	98
K00-K14	K04	3080
K00-K14	K05	676
K00-K14	K06	669
K00-K14	K07	1047
K00-K14	K08	2333
K00-K14	K09	1024
K00-K14	K10	1536
K00-K14	K11	3597
K00-K14	K12	1701
K00-K14	K13	3908
K00-K14	K14	2507
K20-K31	K20	18489
K20-K31	K21	47676
K20-K31	K22	61869
K20-K31	K23	<10
K20-K31	K24	<10
K20-K31	K25	19140
K20-K31	K26	22841
K20-K31	K27	1453
K20-K31	K28	340
K20-K31	K29	80588
K20-K31	K30	19305
K20-K31	K31	15963
K35-K38	K35	10590
K35-K38	K36	233
K35-K38	K37	1179
K35-K38	K38	373

For peer review only

K40-K46	K40	61120
K40-K46	K41	4613
K40-K46	K42	13014
K40-K46	K43	20112
K40-K46	K44	56940
K40-K46	K45	770
K40-K46	K46	869
K50-K52	K50	22827
K50-K52	K51	19728
K50-K52	K52	69630
K55-K63	K55	16090
K55-K63	K56	40625
K55-K63	K57	126292
K55-K63	K58	5743
K55-K63	K59	52671
K55-K63	K60	11881
K55-K63	K61	8608
K55-K63	K62	67372
K55-K63	K63	37732
K65-K67	K65	6797
K65-K67	K66	3646
K65-K67	K67	<10
K70-K77	K70	36852
K70-K77	K71	343
K70-K77	K72	4934
K70-K77	K73	547
K70-K77	K74	8727
K70-K77	K75	3887
K70-K77	K76	8283
K70-K77	K77	<10
K80-K87	K80	111477
K80-K87	K81	23462
K80-K87	K82	3488
K80-K87	K83	18526
K80-K87	K84	<10
K80-K87	K85	26402
K80-K87	K86	13035
K80-K87	K87	<10
K90-K93	K90	3082
K90-K93	K91	9128
K90-K93	K92	69215
K90-K93	K93	<10
L00-L08	L00	18

For peer review only

L00-L08	L01	98
L00-L08	L02	12283
L00-L08	L03	70241
L00-L08	L04	202
L00-L08	L05	2072
L00-L08	L06	<10
L00-L08	L07	<10
L00-L08	L08	3880
L10-L14	L10	211
L10-L14	L11	100
L10-L14	L12	1308
L10-L14	L13	81
L10-L14	L14	<10
L20-L30	L20	373
L20-L30	L21	54
L20-L30	L22	<10
L20-L30	L23	150
L20-L30	L24	37
L20-L30	L25	157
L20-L30	L26	33
L20-L30	L27	784
L20-L30	L28	623
L20-L30	L29	948
L20-L30	L30	4352
L40-L45	L40	12087
L40-L45	L41	15
L40-L45	L42	<10
L40-L45	L43	647
L40-L45	L44	217
L40-L45	L45	<10
L50-L54	L50	798
L50-L54	L51	447
L50-L54	L52	117
L50-L54	L53	1109
L50-L54	L54	<10
L55-L59	L55	27
L55-L59	L56	441
L55-L59	L57	5458
L55-L59	L58	19
L55-L59	L59	113
L60-L75	L60	3250
L60-L75	L61	<10
L60-L75	L62	<10

For peer review only

L60-L75	L63	20
L60-L75	L64	<10
L60-L75	L65	47
L60-L75	L66	23
L60-L75	L67	<10
L60-L75	L68	32
L60-L75	L69	<10
L60-L75	L70	72
L60-L75	L71	671
L60-L75	L72	21898
L60-L75	L73	1783
L60-L75	L74	28
L60-L75	L75	14
L80-L99	L80	<10
L80-L99	L81	1039
L80-L99	L82	3535
L80-L99	L83	81
L80-L99	L84	210
L80-L99	L85	1123
L80-L99	L86	<10
L80-L99	L87	24
L80-L99	L88	463
L80-L99	L89	2891
L80-L99	L90	5435
L80-L99	L91	1594
L80-L99	L92	1104
L80-L99	L93	214
L80-L99	L94	319
L80-L99	L95	590
L80-L99	L96	<10
L80-L99	L97	28410
L80-L99	L98	8618
L80-L99	L99	<10
M00-M03	M00	5098
M00-M03	M01	<10
M00-M03	M02	248
M00-M03	M03	<10
M05-M14	M05	7371
M05-M14	M06	44100
M05-M14	M07	195
M05-M14	M08	156
M05-M14	M09	<10
M05-M14	M10	7801

For peer review only

M05-M14	M11	1083
M05-M14	M12	377
M05-M14	M13	5923
M05-M14	M14	<10
M15-M19	M15	1379
M15-M19	M16	72426
M15-M19	M17	80381
M15-M19	M18	4508
M15-M19	M19	19803
M20-M25	M20	24631
M20-M25	M21	2203
M20-M25	M22	755
M20-M25	M23	24731
M20-M25	M24	3350
M20-M25	M25	41115
M30-M36	M30	463
M30-M36	M31	5184
M30-M36	M32	1509
M30-M36	M33	1719
M30-M36	M34	4583
M30-M36	M35	2937
M30-M36	M36	<10
M40-M43	M40	147
M40-M43	M41	590
M40-M43	M42	<10
M40-M43	M43	1631
M45-M49	M45	1501
M45-M49	M46	3176
M45-M49	M47	6933
M45-M49	M48	10222
M45-M49	M49	16
M50-M54	M50	3098
M50-M54	M51	12249
M50-M54	M52	<10
M50-M54	M53	1234
M50-M54	M54	61197
M60-M63	M60	1550
M60-M63	M61	35
M60-M63	M62	5276
M60-M63	M63	<10
M65-M68	M65	9817
M65-M68	M66	1893
M65-M68	M67	6696

For peer review only

M65-M68	M68	<10
M70-M79	M70	4448
M70-M79	M71	2375
M70-M79	M72	19694
M70-M79	M73	<10
M70-M79	M74	<10
M70-M79	M75	20973
M70-M79	M76	736
M70-M79	M77	3176
M70-M79	M78	<10
M70-M79	M79	54568
M80-M85	M80	6497
M80-M85	M81	9212
M80-M85	M82	<10
M80-M85	M83	85
M80-M85	M84	8467
M80-M85	M85	455
M86-M90	M86	5691
M86-M90	M87	2272
M86-M90	M88	1151
M86-M90	M89	2704
M86-M90	M90	194
M91-M94	M91	38
M91-M94	M92	84
M91-M94	M93	300
M91-M94	M94	1470
M95-M99	M95	907
M95-M99	M96	2265
M95-M99	M97	<10
M95-M99	M98	<10
M95-M99	M99	<10
N00-N08	N00	158
N00-N08	N01	22
N00-N08	N02	8982
N00-N08	N03	611
N00-N08	N04	1296
N00-N08	N05	966
N00-N08	N06	17
N00-N08	N07	<10
N00-N08	N08	<10
N10-N16	N10	1183
N10-N16	N11	842
N10-N16	N12	3853

For peer review only

N10-N16	N13	16606
N10-N16	N14	100
N10-N16	N15	638
N10-N16	N16	<10
N17-N19	N17	49846
N17-N19	N18	41153
N17-N19	N19	7323
N20-N23	N20	39818
N20-N23	N21	5370
N20-N23	N22	<10
N20-N23	N23	7058
N25-N29	N25	275
N25-N29	N26	198
N25-N29	N27	31
N25-N29	N28	4123
N25-N29	N29	<10
N30-N39	N30	16966
N30-N39	N31	7352
N30-N39	N32	21571
N30-N39	N33	<10
N30-N39	N34	448
N30-N39	N35	17761
N30-N39	N36	1774
N30-N39	N37	<10
N30-N39	N38	<10
N30-N39	N39	185262
N40-N51	N40	30726
N40-N51	N41	2847
N40-N51	N42	1244
N40-N51	N43	5185
N40-N51	N44	61
N40-N51	N45	4217
N40-N51	N46	86
N40-N51	N47	9463
N40-N51	N48	5493
N40-N51	N49	1684
N40-N51	N50	6038
N40-N51	N51	<10
N60-N64	N60	3459
N60-N64	N61	2338
N60-N64	N62	1002
N60-N64	N63	1826
N60-N64	N64	2365

For peer review only

N70-N77	N70	844
N70-N77	N71	595
N70-N77	N72	259
N70-N77	N73	1742
N70-N77	N74	<10
N70-N77	N75	1513
N70-N77	N76	2140
N70-N77	N77	<10
N80-N98	N80	3008
N80-N98	N81	32139
N80-N98	N82	2451
N80-N98	N83	7052
N80-N98	N84	13289
N80-N98	N85	3244
N80-N98	N86	253
N80-N98	N87	555
N80-N98	N88	558
N80-N98	N89	1945
N80-N98	N90	3232
N80-N98	N91	105
N80-N98	N92	22483
N80-N98	N93	6963
N80-N98	N94	2318
N80-N98	N95	21071
N80-N98	N96	<10
N80-N98	N97	598
N80-N98	N98	12
N99-N99	N99	1952
O00-O08	O00	202
O00-O08	O01	17
O00-O08	O02	783
O00-O08	O03	675
O00-O08	O04	2376
O00-O08	O05	<10
O00-O08	O06	<10
O00-O08	O07	10
O00-O08	O08	30
O10-O16	O10	<10
O10-O16	O11	<10
O10-O16	O12	<10
O10-O16	O13	<10
O10-O16	O14	<10
O10-O16	O15	<10

For peer review only

O10-O16	O16	<10
O20-O29	O20	131
O20-O29	O21	30
O20-O29	O22	<10
O20-O29	O23	<10
O20-O29	O24	<10
O20-O29	O25	<10
O20-O29	O26	<10
O20-O29	O27	<10
O20-O29	O28	<10
O20-O29	O29	<10
O30-O48	O30	<10
O30-O48	O31	<10
O30-O48	O32	<10
O30-O48	O33	<10
O30-O48	O34	<10
O30-O48	O35	<10
O30-O48	O36	<10
O30-O48	O37	<10
O30-O48	O38	<10
O30-O48	O39	<10
O30-O48	O40	<10
O30-O48	O41	<10
O30-O48	O42	<10
O30-O48	O43	<10
O30-O48	O44	<10
O30-O48	O45	<10
O30-O48	O46	<10
O30-O48	O47	<10
O30-O48	O48	<10
O60-O75	O60	<10
O60-O75	O61	<10
O60-O75	O62	<10
O60-O75	O63	<10
O60-O75	O64	<10
O60-O75	O65	<10
O60-O75	O66	<10
O60-O75	O67	<10
O60-O75	O68	<10
O60-O75	O69	<10
O60-O75	O70	<10
O60-O75	O71	<10
O60-O75	O72	16

For peer review only

O60-O75	O73	<10
O60-O75	O74	<10
O60-O75	O75	<10
O80-O84	O80	<10
O80-O84	O81	<10
O80-O84	O82	<10
O80-O84	O83	<10
O80-O84	O84	<10
O85-O92	O85	<10
O85-O92	O86	<10
O85-O92	O87	<10
O85-O92	O88	<10
O85-O92	O89	<10
O85-O92	O90	<10
O85-O92	O91	<10
O85-O92	O92	<10
O95-O99	O95	<10
O95-O99	O96	<10
O95-O99	O97	<10
O95-O99	O98	<10
O95-O99	O99	<10
P05-P08	P05	<10
P05-P08	P06	<10
P05-P08	P07	<10
P05-P08	P08	<10
P10-P15	P10	<10
P10-P15	P11	<10
P10-P15	P12	<10
P10-P15	P13	<10
P10-P15	P14	<10
P10-P15	P15	<10
P20-P29	P20	<10
P20-P29	P21	<10
P20-P29	P22	<10
P20-P29	P23	<10
P20-P29	P24	<10
P20-P29	P25	<10
P20-P29	P26	<10
P20-P29	P27	<10
P20-P29	P28	<10
P20-P29	P29	<10
P35-P39	P35	<10
P35-P39	P36	<10

For peer review only

P35-P39	P37	<10
P35-P39	P38	<10
P35-P39	P39	<10
P50-P61	P50	<10
P50-P61	P51	<10
P50-P61	P52	<10
P50-P61	P53	<10
P50-P61	P54	<10
P50-P61	P55	<10
P50-P61	P56	<10
P50-P61	P57	<10
P50-P61	P58	<10
P50-P61	P59	<10
P50-P61	P60	<10
P50-P61	P61	<10
P80-P83	P80	<10
P80-P83	P81	<10
P80-P83	P82	<10
P80-P83	P83	<10
Q00-Q07	Q00	<10
Q00-Q07	Q01	14
Q00-Q07	Q02	13
Q00-Q07	Q03	90
Q00-Q07	Q04	124
Q00-Q07	Q05	114
Q00-Q07	Q06	74
Q00-Q07	Q07	236
Q10-Q18	Q10	54
Q10-Q18	Q11	<10
Q10-Q18	Q12	72
Q10-Q18	Q13	15
Q10-Q18	Q14	<10
Q10-Q18	Q15	29
Q10-Q18	Q16	14
Q10-Q18	Q17	92
Q10-Q18	Q18	352
Q20-Q28	Q20	15
Q20-Q28	Q21	1461
Q20-Q28	Q22	100
Q20-Q28	Q23	420
Q20-Q28	Q24	163
Q20-Q28	Q25	187
Q20-Q28	Q26	47

For peer review only

Q20-Q28	Q27	741
Q20-Q28	Q28	744
Q30-Q34	Q30	37
Q30-Q34	Q31	130
Q30-Q34	Q32	<10
Q30-Q34	Q33	33
Q30-Q34	Q34	12
Q35-Q37	Q35	25
Q35-Q37	Q36	10
Q35-Q37	Q37	11
Q38-Q45	Q38	1018
Q38-Q45	Q39	185
Q38-Q45	Q40	67
Q38-Q45	Q41	<10
Q38-Q45	Q42	<10
Q38-Q45	Q43	329
Q38-Q45	Q44	158
Q38-Q45	Q45	39
Q50-Q56	Q50	73
Q50-Q56	Q51	25
Q50-Q56	Q52	89
Q50-Q56	Q53	121
Q50-Q56	Q54	215
Q50-Q56	Q55	88
Q50-Q56	Q56	<10
Q60-Q64	Q60	16
Q60-Q64	Q61	839
Q60-Q64	Q62	128
Q60-Q64	Q63	67
Q60-Q64	Q64	81
Q65-Q79	Q65	201
Q65-Q79	Q66	283
Q65-Q79	Q67	90
Q65-Q79	Q68	19
Q65-Q79	Q69	<10
Q65-Q79	Q70	15
Q65-Q79	Q71	<10
Q65-Q79	Q72	<10
Q65-Q79	Q73	<10
Q65-Q79	Q74	65
Q65-Q79	Q75	17
Q65-Q79	Q76	64
Q65-Q79	Q77	51

For peer review only

Q65-Q79	Q78	82
Q65-Q79	Q79	79
Q80-Q89	Q80	<10
Q80-Q89	Q81	15
Q80-Q89	Q82	646
Q80-Q89	Q83	91
Q80-Q89	Q84	<10
Q80-Q89	Q85	413
Q80-Q89	Q86	<10
Q80-Q89	Q87	96
Q80-Q89	Q88	<10
Q80-Q89	Q89	240
Q90-Q99	Q90	133
Q90-Q99	Q91	<10
Q90-Q99	Q92	<10
Q90-Q99	Q93	<10
Q90-Q99	Q94	<10
Q90-Q99	Q95	<10
Q90-Q99	Q96	19
Q90-Q99	Q97	<10
Q90-Q99	Q98	67
Q90-Q99	Q99	17
R00-R09	R00	21072
R00-R09	R01	65
R00-R09	R02	3009
R00-R09	R03	143
R00-R09	R04	39227
R00-R09	R05	5669
R00-R09	R06	54686
R00-R09	R07	301798
R00-R09	R08	<10
R00-R09	R09	3392
R10-R19	R10	151132
R10-R19	R11	39828
R10-R19	R12	2189
R10-R19	R13	27099
R10-R19	R14	1969
R10-R19	R15	4868
R10-R19	R16	628
R10-R19	R17	5908
R10-R19	R18	9937
R10-R19	R19	31403
R20-R23	R20	5419

For peer review only

R20-R23	R21	2314
R20-R23	R22	5833
R20-R23	R23	972
R25-R29	R25	1817
R25-R29	R26	61809
R25-R29	R27	828
R25-R29	R28	<10
R25-R29	R29	27490
R30-R39	R30	1643
R30-R39	R31	55796
R30-R39	R32	5670
R30-R39	R33	35863
R30-R39	R34	151
R30-R39	R35	5426
R30-R39	R36	86
R30-R39	R37	<10
R30-R39	R38	<10
R30-R39	R39	15982
R40-R46	R40	4355
R40-R46	R41	45481
R40-R46	R42	20685
R40-R46	R43	34
R40-R46	R44	1634
R40-R46	R45	895
R40-R46	R46	668
R47-R49	R47	4910
R47-R49	R48	18
R47-R49	R49	2963
R50-R69	R50	8353
R50-R69	R51	30605
R50-R69	R52	3116
R50-R69	R53	13671
R50-R69	R54	57428
R50-R69	R55	108422
R50-R69	R56	27660
R50-R69	R57	2049
R50-R69	R58	573
R50-R69	R59	4932
R50-R69	R60	7080
R50-R69	R61	683
R50-R69	R62	38
R50-R69	R63	15747
R50-R69	R64	169

For peer review only

R50-R69	R65	<10
R50-R69	R66	<10
R50-R69	R67	<10
R50-R69	R68	1693
R50-R69	R69	3781
R70-R79	R70	123
R70-R79	R71	68
R70-R79	R72	100
R70-R79	R73	3558
R70-R79	R74	186
R70-R79	R75	<10
R70-R79	R76	716
R70-R79	R77	147
R70-R79	R78	154
R70-R79	R79	5184
R80-R82	R80	354
R80-R82	R81	10
R80-R82	R82	310
R83-R89	R83	<10
R83-R89	R84	145
R83-R89	R85	33
R83-R89	R86	21
R83-R89	R87	442
R83-R89	R88	<10
R83-R89	R89	26
R90-R94	R90	841
R90-R94	R91	18713
R90-R94	R92	130
R90-R94	R93	5822
R90-R94	R94	4510
R95-R99	R95	<10
R95-R99	R96	<10
R95-R99	R97	<10
R95-R99	R98	<10
R95-R99	R99	1944
S00-S09	S00	18337
S00-S09	S01	24541
S00-S09	S02	8599
S00-S09	S03	129
S00-S09	S04	47
S00-S09	S05	1488
S00-S09	S06	13727
S00-S09	S07	<10

For peer review only

S00-S09	S08	34
S00-S09	S09	35949
S10-S19	S10	379
S10-S19	S11	306
S10-S19	S12	3539
S10-S19	S13	470
S10-S19	S14	602
S10-S19	S15	24
S10-S19	S16	29
S10-S19	S17	<10
S10-S19	S18	<10
S10-S19	S19	652
S20-S29	S20	2820
S20-S29	S21	531
S20-S29	S22	14287
S20-S29	S23	83
S20-S29	S24	49
S20-S29	S25	41
S20-S29	S26	20
S20-S29	S27	3099
S20-S29	S28	54
S20-S29	S29	1343
S30-S39	S30	2998
S30-S39	S31	921
S30-S39	S32	22852
S30-S39	S33	91
S30-S39	S34	63
S30-S39	S35	86
S30-S39	S36	994
S30-S39	S37	430
S30-S39	S38	53
S30-S39	S39	3506
S40-S49	S40	1428
S40-S49	S41	429
S40-S49	S42	28092
S40-S49	S43	4615
S40-S49	S44	65
S40-S49	S45	50
S40-S49	S46	619
S40-S49	S47	27
S40-S49	S48	<10
S40-S49	S49	1309
S50-S59	S50	638

For peer review only

S50-S59	S51	1654
S50-S59	S52	37213
S50-S59	S53	491
S50-S59	S54	68
S50-S59	S55	43
S50-S59	S56	543
S50-S59	S57	24
S50-S59	S58	<10
S50-S59	S59	294
S60-S69	S60	820
S60-S69	S61	8375
S60-S69	S62	7364
S60-S69	S63	1407
S60-S69	S64	977
S60-S69	S65	126
S60-S69	S66	2279
S60-S69	S67	583
S60-S69	S68	1374
S60-S69	S69	964
S70-S79	S70	4456
S70-S79	S71	298
S70-S79	S72	128265
S70-S79	S73	1110
S70-S79	S74	22
S70-S79	S75	66
S70-S79	S76	1289
S70-S79	S77	22
S70-S79	S78	11
S70-S79	S79	6696
S80-S89	S80	3855
S80-S89	S81	3644
S80-S89	S82	38308
S80-S89	S83	2082
S80-S89	S84	19
S80-S89	S85	62
S80-S89	S86	1397
S80-S89	S87	78
S80-S89	S88	53
S80-S89	S89	2775
S90-S99	S90	923
S90-S99	S91	686
S90-S99	S92	4814
S90-S99	S93	823

For peer review only

S90-S99	S94	<10
S90-S99	S95	25
S90-S99	S96	118
S90-S99	S97	78
S90-S99	S98	72
S90-S99	S99	1056
T00-T07	T00	537
T00-T07	T01	126
T00-T07	T02	419
T00-T07	T03	22
T00-T07	T04	38
T00-T07	T05	<10
T00-T07	T06	29
T00-T07	T07	55
T08-T14	T08	270
T08-T14	T09	458
T08-T14	T10	20
T08-T14	T11	329
T08-T14	T12	<10
T08-T14	T13	690
T08-T14	T14	1916
T15-T19	T15	86
T15-T19	T16	84
T15-T19	T17	1763
T15-T19	T18	4624
T15-T19	T19	126
T20-T25	T20	649
T20-T25	T21	823
T20-T25	T22	489
T20-T25	T23	606
T20-T25	T24	545
T20-T25	T25	363
T26-T28	T26	59
T26-T28	T27	11
T26-T28	T28	31
T29-T32	T29	225
T29-T32	T30	78
T29-T32	T31	63
T29-T32	T32	<10
T33-T35	T33	<10
T33-T35	T34	27
T33-T35	T35	10
T36-T50	T36	344

For peer review only

T36-T50	T37	200
T36-T50	T38	1043
T36-T50	T39	13526
T36-T50	T40	6647
T36-T50	T41	49
T36-T50	T42	7832
T36-T50	T43	8970
T36-T50	T44	1166
T36-T50	T45	1213
T36-T50	T46	1791
T36-T50	T47	178
T36-T50	T48	147
T36-T50	T49	104
T36-T50	T50	3942
T51-T65	T51	432
T51-T65	T52	125
T51-T65	T53	10
T51-T65	T54	162
T51-T65	T55	18
T51-T65	T56	234
T51-T65	T57	<10
T51-T65	T58	223
T51-T65	T59	1598
T51-T65	T60	84
T51-T65	T61	24
T51-T65	T62	120
T51-T65	T63	257
T51-T65	T64	<10
T51-T65	T65	323
T66-T78	T66	139
T66-T78	T67	33
T66-T78	T68	1088
T66-T78	T69	19
T66-T78	T70	94
T66-T78	T71	110
T66-T78	T72	<10
T66-T78	T73	21
T66-T78	T74	10
T66-T78	T75	267
T66-T78	T76	<10
T66-T78	T77	<10
T66-T78	T78	4680
T79-T79	T79	1904

For peer review only

T80-T88	T80	679
T80-T88	T81	46443
T80-T88	T82	25827
T80-T88	T83	13664
T80-T88	T84	44484
T80-T88	T85	15471
T80-T88	T86	1883
T80-T88	T87	1659
T80-T88	T88	2050
W00-W19	W00	<10
W00-W19	W01	<10
W00-W19	W02	<10
W00-W19	W03	<10
W00-W19	W04	<10
W00-W19	W05	<10
W00-W19	W06	<10
W00-W19	W07	<10
W00-W19	W08	<10
W00-W19	W09	<10
W00-W19	W10	<10
W00-W19	W11	<10
W00-W19	W12	<10
W00-W19	W13	<10
W00-W19	W14	<10
W00-W19	W15	<10
W00-W19	W16	<10
W00-W19	W17	<10
W00-W19	W18	<10
W00-W19	W19	<10
Z00-Z13	Z00	542
Z00-Z13	Z01	293
Z00-Z13	Z02	<10
Z00-Z13	Z03	12713
Z00-Z13	Z04	5106
Z00-Z13	Z05	<10
Z00-Z13	Z06	<10
Z00-Z13	Z07	<10
Z00-Z13	Z08	63043
Z00-Z13	Z09	68414
Z00-Z13	Z10	<10
Z00-Z13	Z11	507
Z00-Z13	Z12	9653
Z00-Z13	Z13	8682

For peer review only

Z20-Z29	Z20	<10
Z20-Z29	Z21	<10
Z20-Z29	Z22	15
Z20-Z29	Z23	<10
Z20-Z29	Z24	<10
Z20-Z29	Z25	<10
Z20-Z29	Z26	<10
Z20-Z29	Z27	<10
Z20-Z29	Z28	<10
Z20-Z29	Z29	1198
Z30-Z39	Z30	13783
Z30-Z39	Z31	437
Z30-Z39	Z32	<10
Z30-Z39	Z33	<10
Z30-Z39	Z34	<10
Z30-Z39	Z35	<10
Z30-Z39	Z36	<10
Z30-Z39	Z37	<10
Z30-Z39	Z38	<10
Z30-Z39	Z39	<10
Z40-Z54	Z40	1433
Z40-Z54	Z41	2832
Z40-Z54	Z42	11095
Z40-Z54	Z43	12974
Z40-Z54	Z44	679
Z40-Z54	Z45	12442
Z40-Z54	Z46	23792
Z40-Z54	Z47	12123
Z40-Z54	Z48	4384
Z40-Z54	Z49	1307
Z40-Z54	Z50	7458
Z40-Z54	Z51	3707
Z40-Z54	Z52	1044
Z40-Z54	Z53	128753
Z40-Z54	Z54	3016
Z55-Z65	Z55	<10
Z55-Z65	Z56	<10
Z55-Z65	Z57	<10
Z55-Z65	Z58	<10
Z55-Z65	Z59	43
Z55-Z65	Z60	196
Z55-Z65	Z61	<10
Z55-Z65	Z62	<10

For peer review only

Z55-Z65	Z63	34
Z55-Z65	Z64	<10
Z55-Z65	Z65	<10
Z70-Z76	Z70	<10
Z70-Z76	Z71	706
Z70-Z76	Z72	103
Z70-Z76	Z73	215
Z70-Z76	Z74	4252
Z70-Z76	Z75	6190
Z70-Z76	Z76	100
Z80-Z99	Z80	<10
Z80-Z99	Z81	<10
Z80-Z99	Z82	<10
Z80-Z99	Z83	<10
Z80-Z99	Z84	<10
Z80-Z99	Z85	550
Z80-Z99	Z86	<10
Z80-Z99	Z87	<10
Z80-Z99	Z88	<10
Z80-Z99	Z89	<10
Z80-Z99	Z90	51
Z80-Z99	Z91	69
Z80-Z99	Z92	17
Z80-Z99	Z93	<10
Z80-Z99	Z94	<10
Z80-Z99	Z95	<10
Z80-Z99	Z96	<10
Z80-Z99	Z97	<10
Z80-Z99	Z98	133
Z80-Z99	Z99	<10

For peer review only

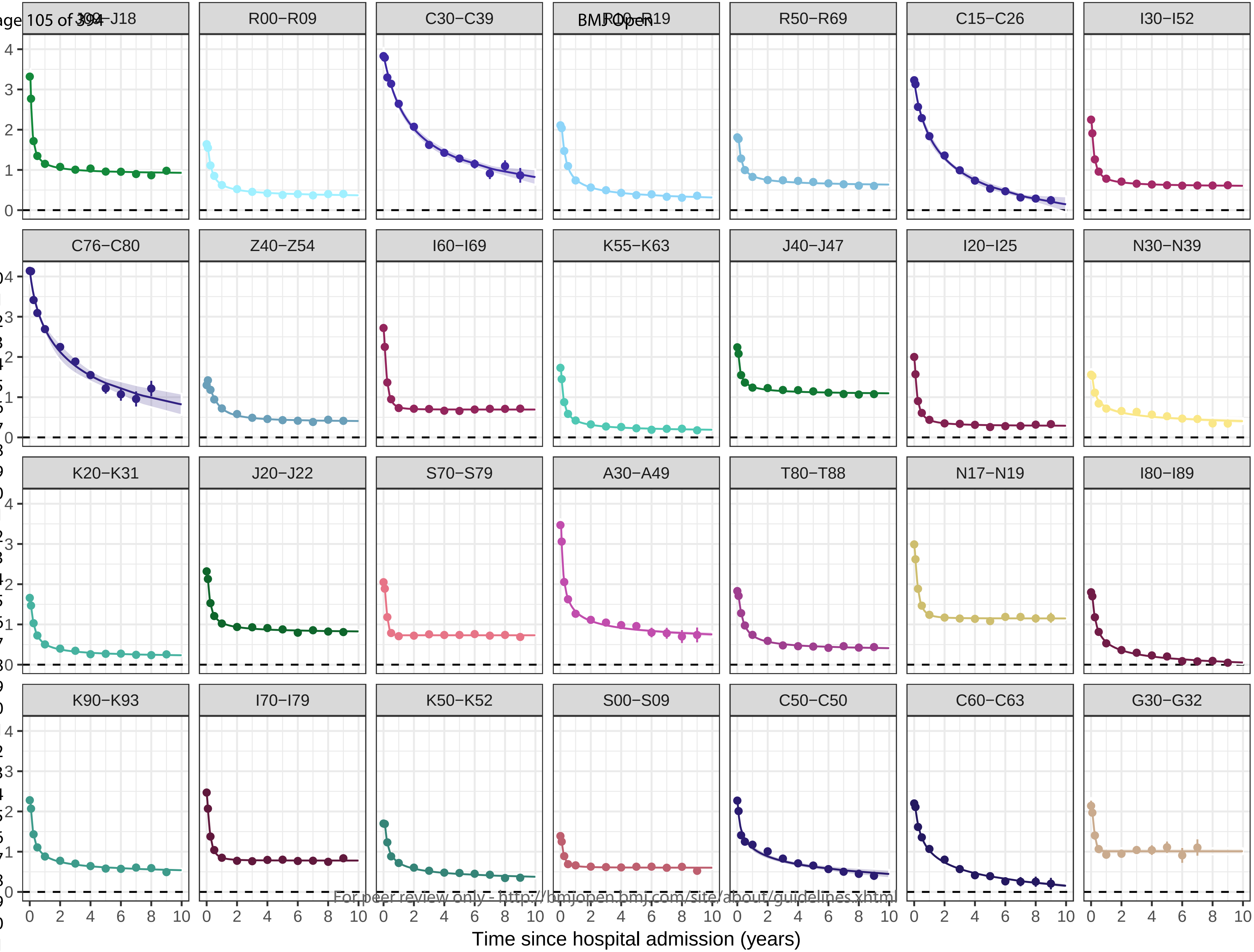
R00–R09
C30–C39
R50–R69
C15–C26
I30–I52
C76–C80
Z40–Z54
I60–I69
K55–K63
J40–J47
I20–I25
N30–N39
K20–K31
J20–J22
S70–S79
A30–A49
T80–T88
N17–N19
I80–I89
K90–K93
I70–I79
K50–K52
S00–S09
C50–C50
C60–C63
G30–G32
Mortality (lnHR)
Time since hospital admission (years)

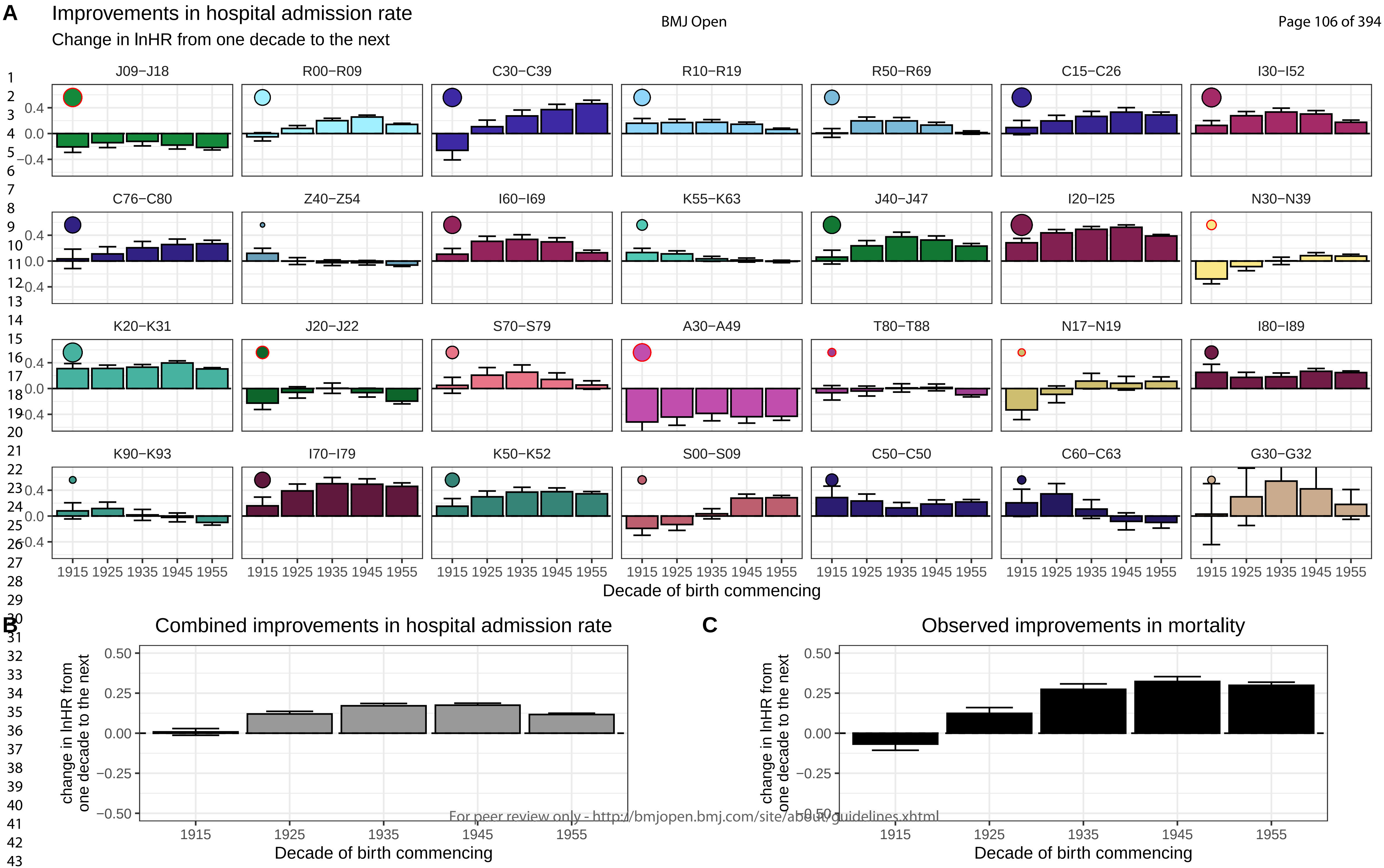
A
Improvements in hospital admission rate
Change in lnHR from one decade to the next
J09–J18
R00–R09
C30–C39
R10–R19
R50–R69
C15–C26
I30–I52
C76–C80
Z40–Z54
I60–I69
K55–K63
J40–J47
I20–I25
N30–N39
K20–K31
J20–J22
S70–S79
A30–A49
T80–T88
N17–N19
I80–I89
K90–K93
I70–I79
K50–K52
S00–S09
C50–C50
C60–C63
G30–G32
0.4
0.0
-0.4
1915 1925 1935 1945 1955
Decade of birth commencing
B
Combined improvements in hospital admission rate
change in lnHR from one decade to the next
0.50
0.25
0.00
-0.25
-0.50
1915
1925
1935
1945
1955
Decade of birth commencing
C
Observed improvements in mortality
change in lnHR from one decade to the next
0.50
0.25
0.00
-0.25
-0.50
1915
1925
1935
1945
1955
Decade of birth commencing

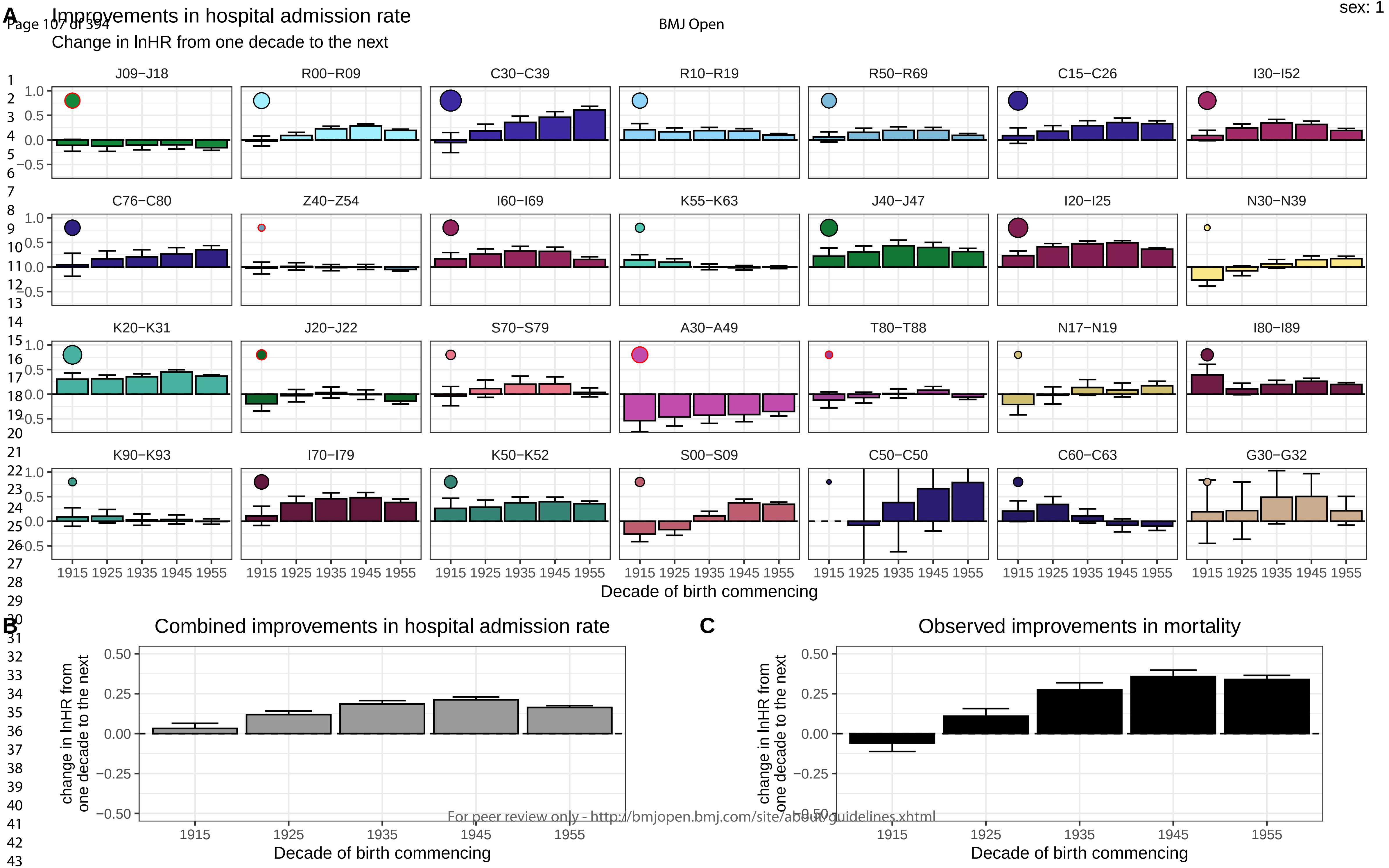
A
Improvements in hospital admission rate
Change in lnHR from one decade to the next
sex: 1
J09−J18
R00−R09
C30−C39
R10−R19
R50−R69
C15−C26
I30−I52
C76−C80
Z40−Z54
I60−I69
K55−K63
J40−J47
I20−I25
N30−N39
K20−K31
J20−J22
S70−S79
A30−A49
T80−T88
N17−N19
I80−I89
K90−K93
I70−I79
K50−K52
S00−S09
C50−C50
C60−C63
G30−G32
1.0
0.5
0.0
−0.5
1915 1925 1935 1945 1955
Decade of birth commencing
B
Combined improvements in hospital admission rate
change in lnHR from one decade to the next
0.50
0.25
0.00
−0.25
−0.50
1915
1925
1935
1945
1955
Decade of birth commencing
C
Observed improvements in mortality
change in lnHR from one decade to the next
0.50
0.25
0.00
−0.25
−0.50
1915
1925
1935
1945
1955
Decade of birth commencing

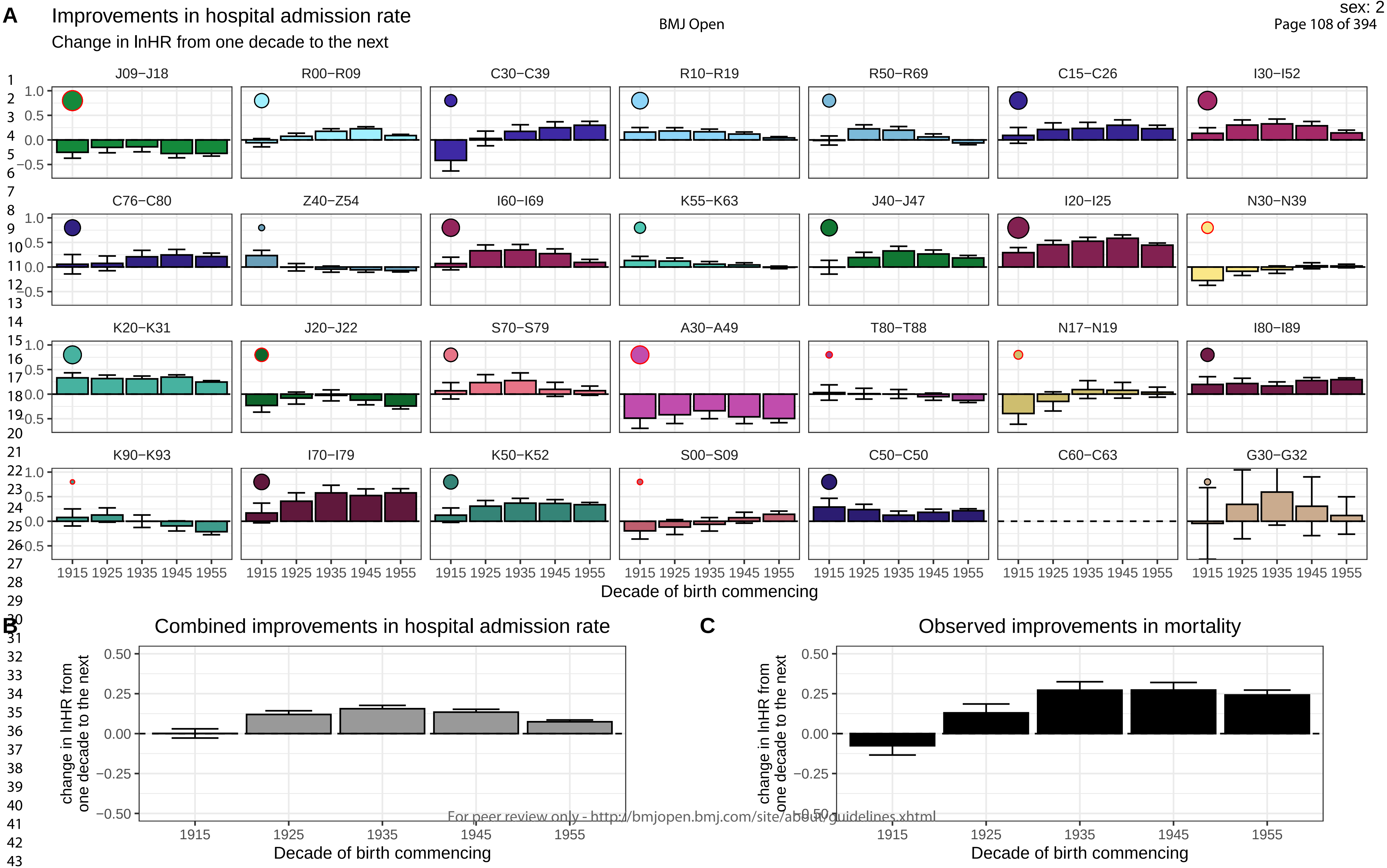
sex: 2
A
Improvements in hospital admission rate
Change in lnHR from one decade to the next
J09−J18
R00−R09
C30−C39
R10−R19
R50−R69
C15−C26
I30−I52
C76−C80
Z40−Z54
I60−I69
K55−K63
J40−J47
I20−I25
N30−N39
K20−K31
J20−J22
S70−S79
A30−A49
T80−T88
N17−N19
I80−I89
K90−K93
I70−I79
K50−K52
S00−S09
C50−C50
C60−C63
G30−G32
1.0
0.5
0.0
−0.5
1915 1925 1935 1945 1955
Decade of birth commencing
B
Combined improvements in hospital admission rate
change in lnHR from one decade to the next
0.50
0.25
0.00
−0.25
−0.50
1915
1925
1935
1945
1955
Decade of birth commencing
C
Observed improvements in mortality
change in lnHR from one decade to the next
0.50
0.25
0.00
−0.25
−0.50
1915
1925
1935
1945
1955
Decade of birth commencing

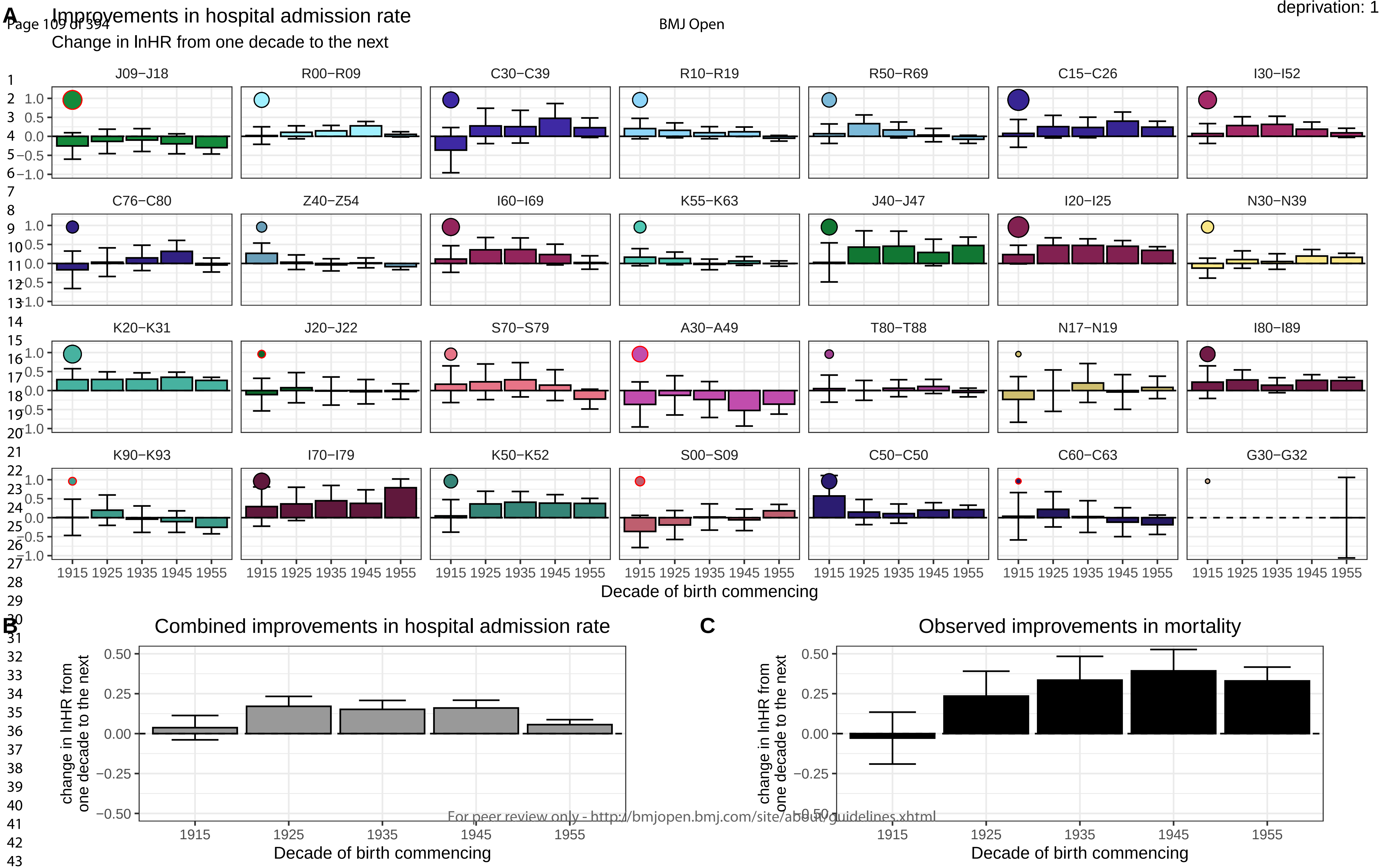
A
Improvements in hospital admission rate
Change in lnHR from one decade to the next
deprivation: 1
J09−J18
R00−R09
C30−C39
R10−R19
R50−R69
C15−C26
I30−I52
C76−C80
Z40−Z54
I60−I69
K55−K63
J40−J47
I20−I25
N30−N39
K20−K31
J20−J22
S70−S79
A30−A49
T80−T88
N17−N19
I80−I89
K90−K93
I70−I79
K50−K52
S00−S09
C50−C50
C60−C63
G30−G32
1.0
0.5
0.0
−0.5
−1.0
1915 1925 1935 1945 1955
Decade of birth commencing
B
Combined improvements in hospital admission rate
change in lnHR from one decade to the next
0.50
0.25
0.00
−0.25
−0.50
1915
1925
1935
1945
1955
Decade of birth commencing
C
Observed improvements in mortality
change in lnHR from one decade to the next
0.50
0.25
0.00
−0.25
−0.50
1915
1925
1935
1945
1955
Decade of birth commencing

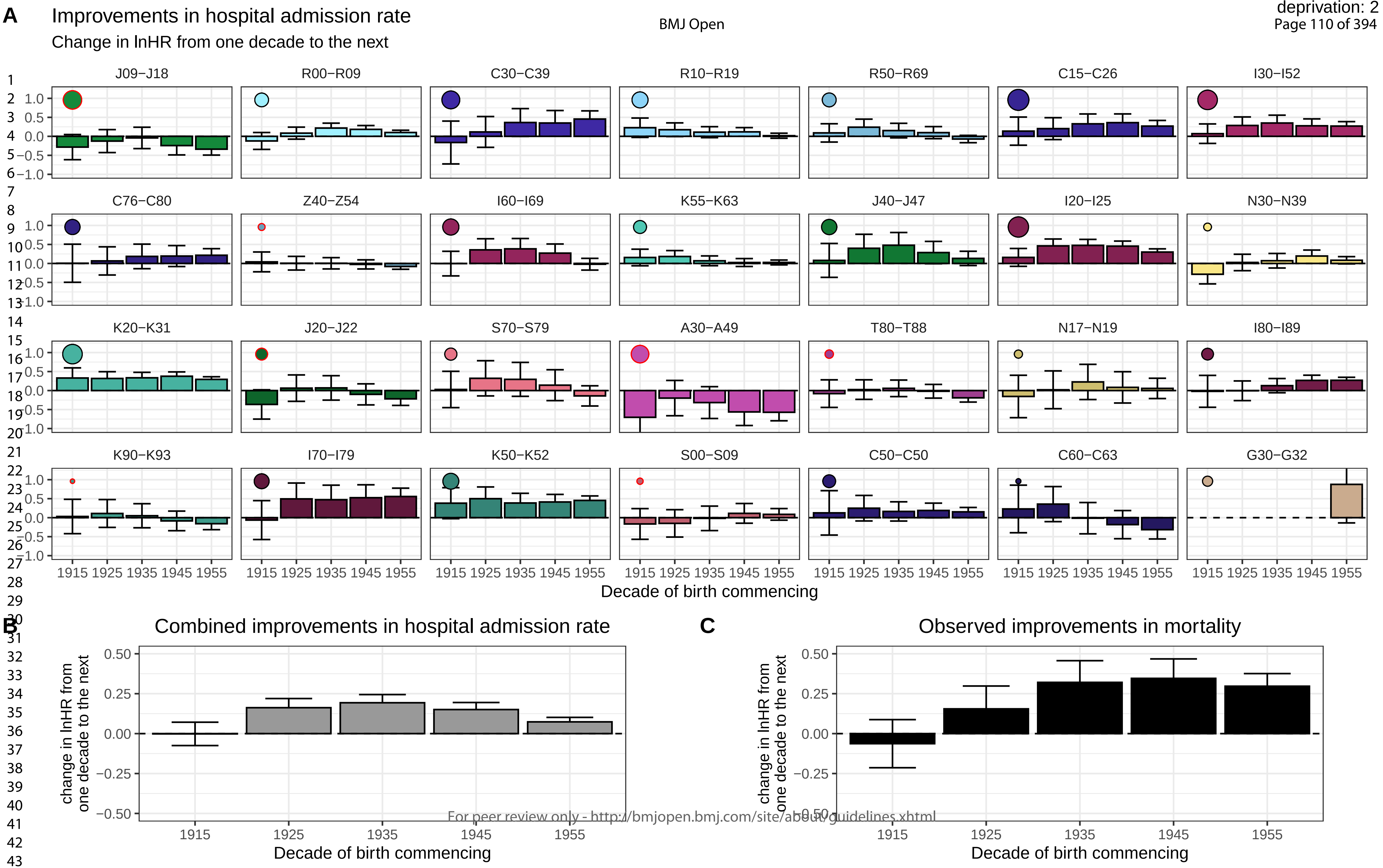

For peer review only - http://bmjopen.bmj.com/site/about/guidelines.xhtml

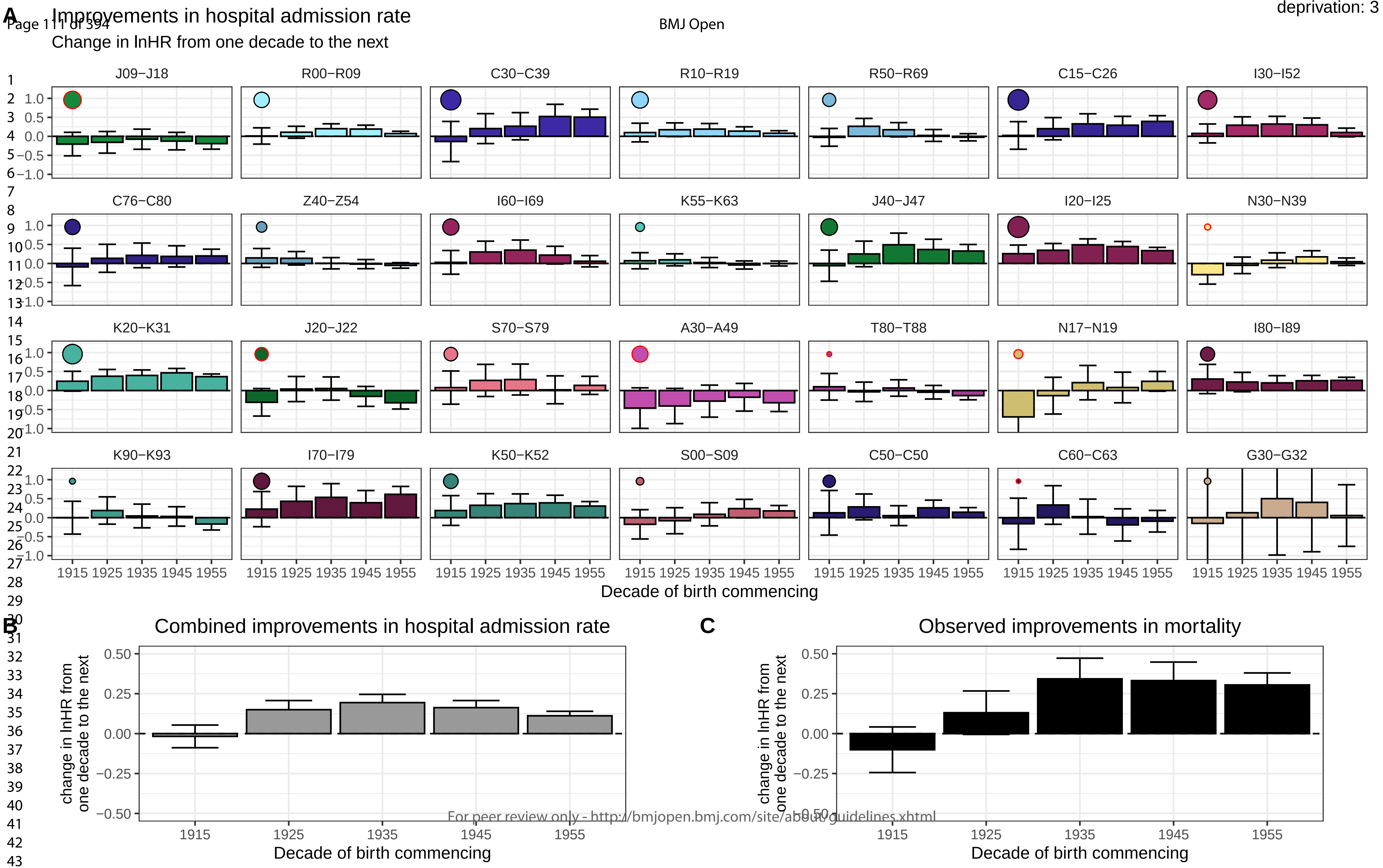

For peer review only - http://bmjopen.bmj.com/site/about/guidelines.xhtml

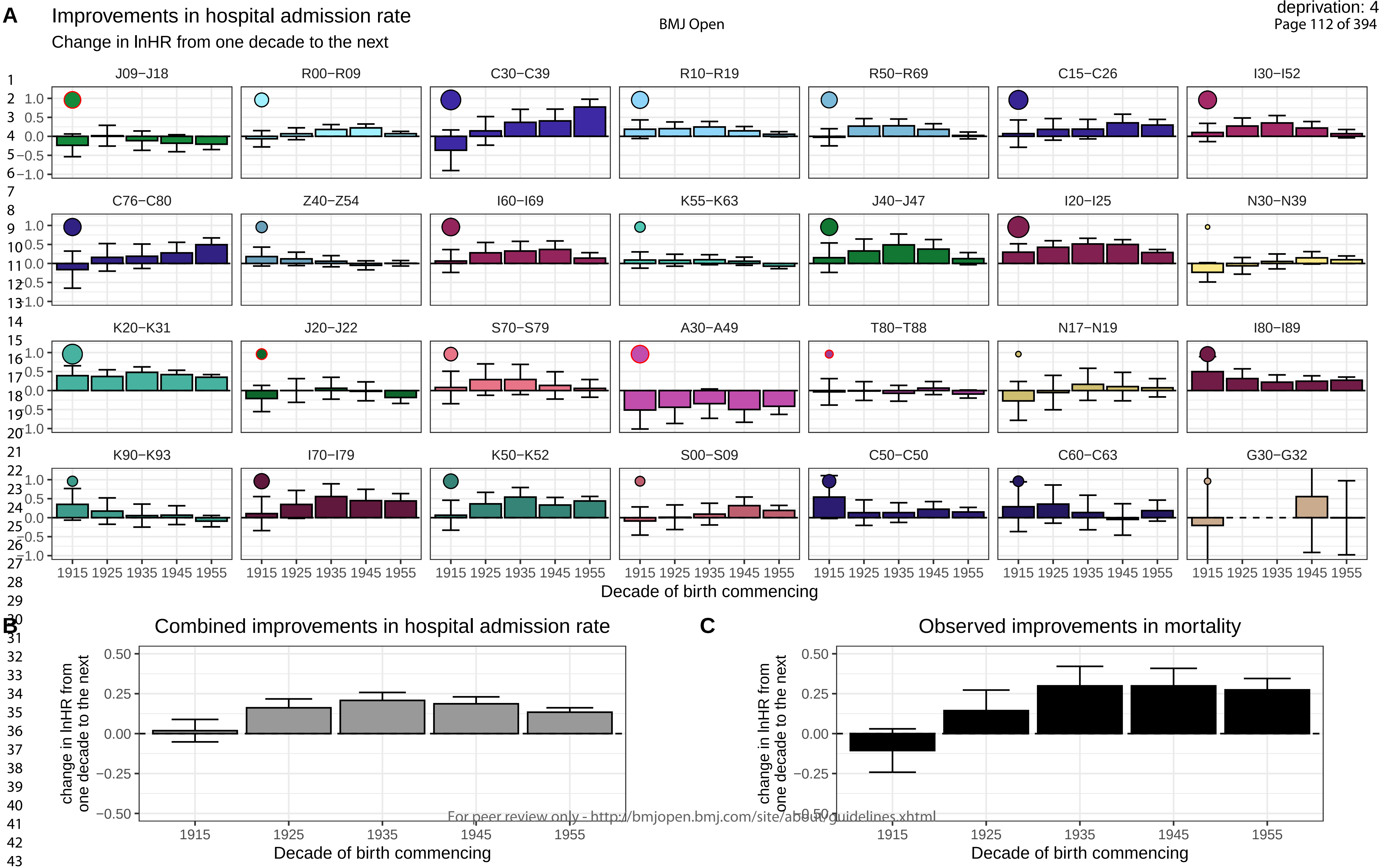

For peer review only - http://bmjopen.bmj.com/site/about/guidelines.xhtml

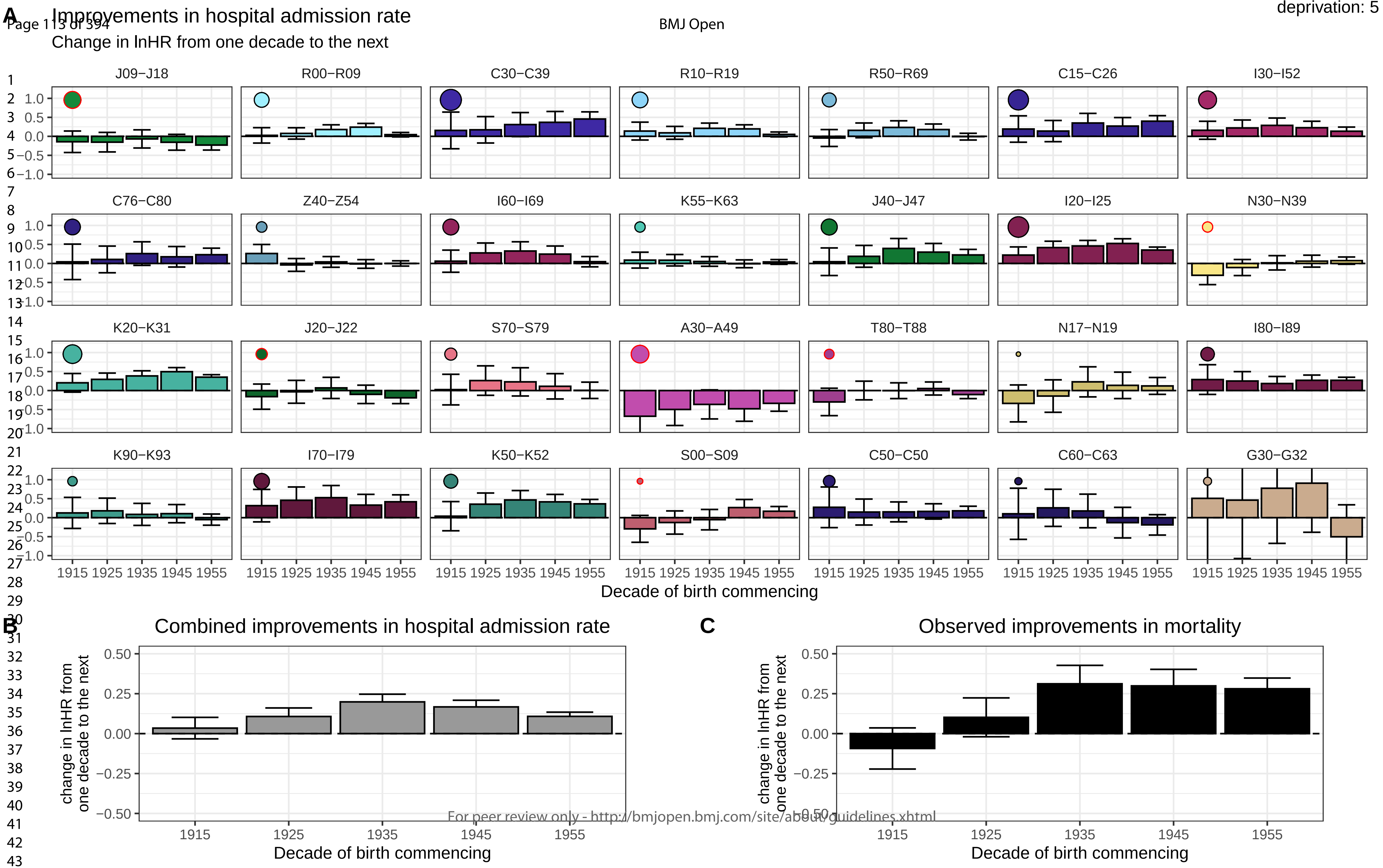
deprivation: 5
A
Improvements in hospital admission rate
Change in lnHR from one decade to the next
J09−J18
R00−R09
C30−C39
R10−R19
R50−R69
C15−C26
I30−I52
C76−C80
Z40−Z54
I60−I69
K55−K63
J40−J47
I20−I25
N30−N39
K20−K31
J20−J22
S70−S79
A30−A49
T80−T88
N17−N19
I80−I89
K90−K93
I70−I79
K50−K52
S00−S09
C50−C50
C60−C63
G30−G32
1915 1925 1935 1945 1955
Decade of birth commencing
B
Combined improvements in hospital admission rate
change in lnHR from one decade to the next
Decade of birth commencing
C
Observed improvements in mortality
change in lnHR from one decade to the next
Decade of birth commencing

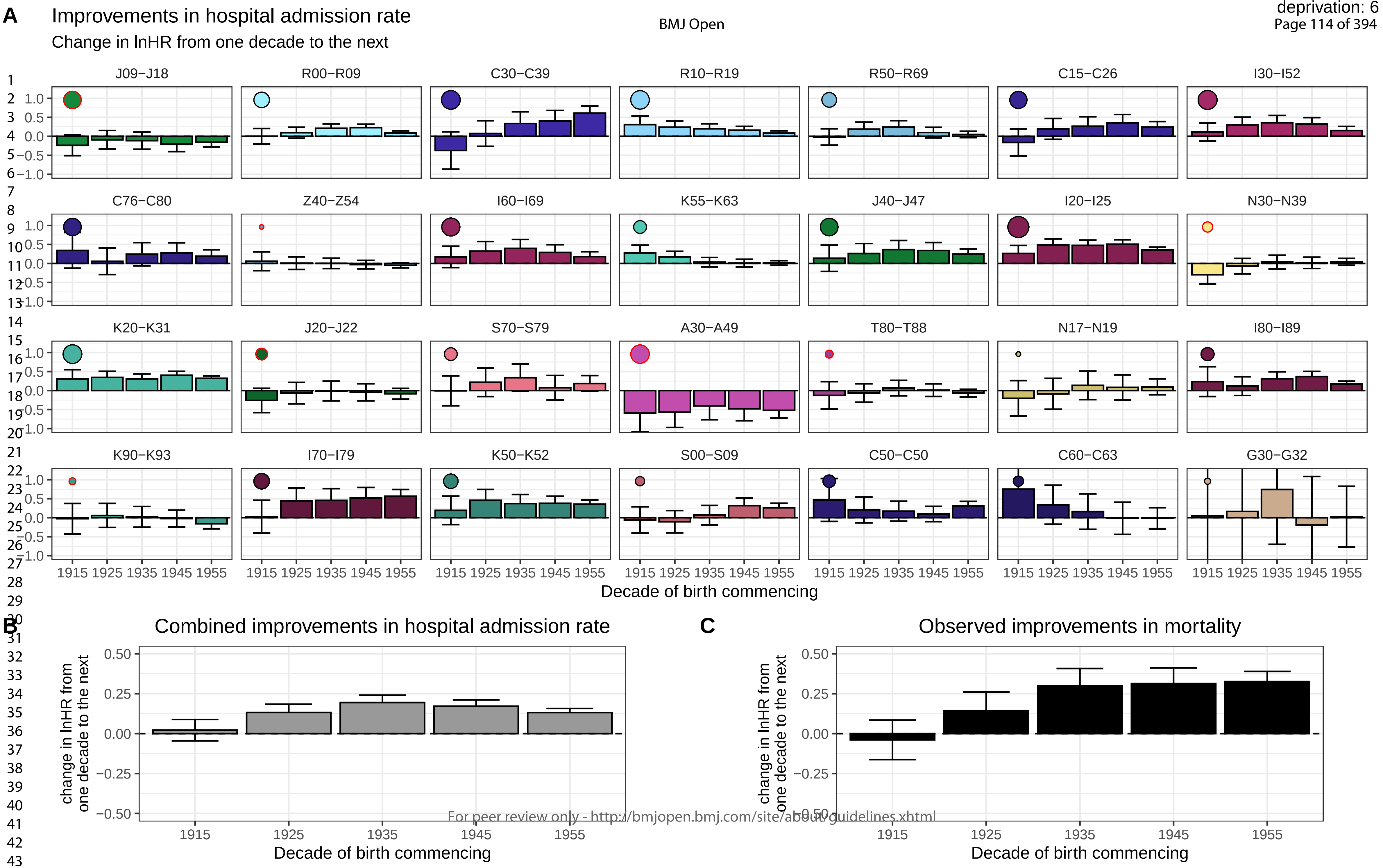

For peer review only - http://bmjopen.bmj.com/site/about/guidelines.xhtml

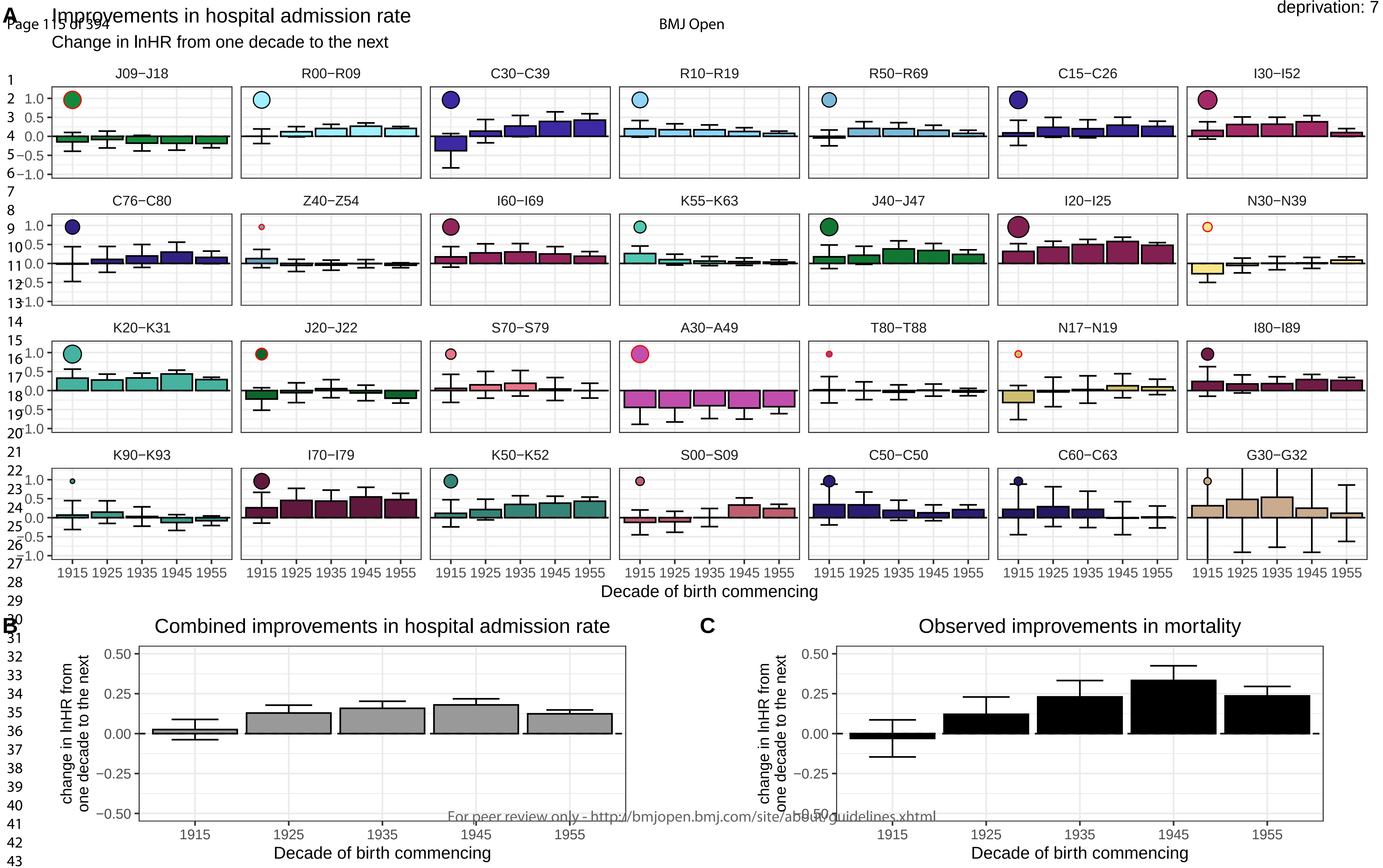

For peer review only - http://bmjopen.bmj.com/site/about/guidelines.xhtml

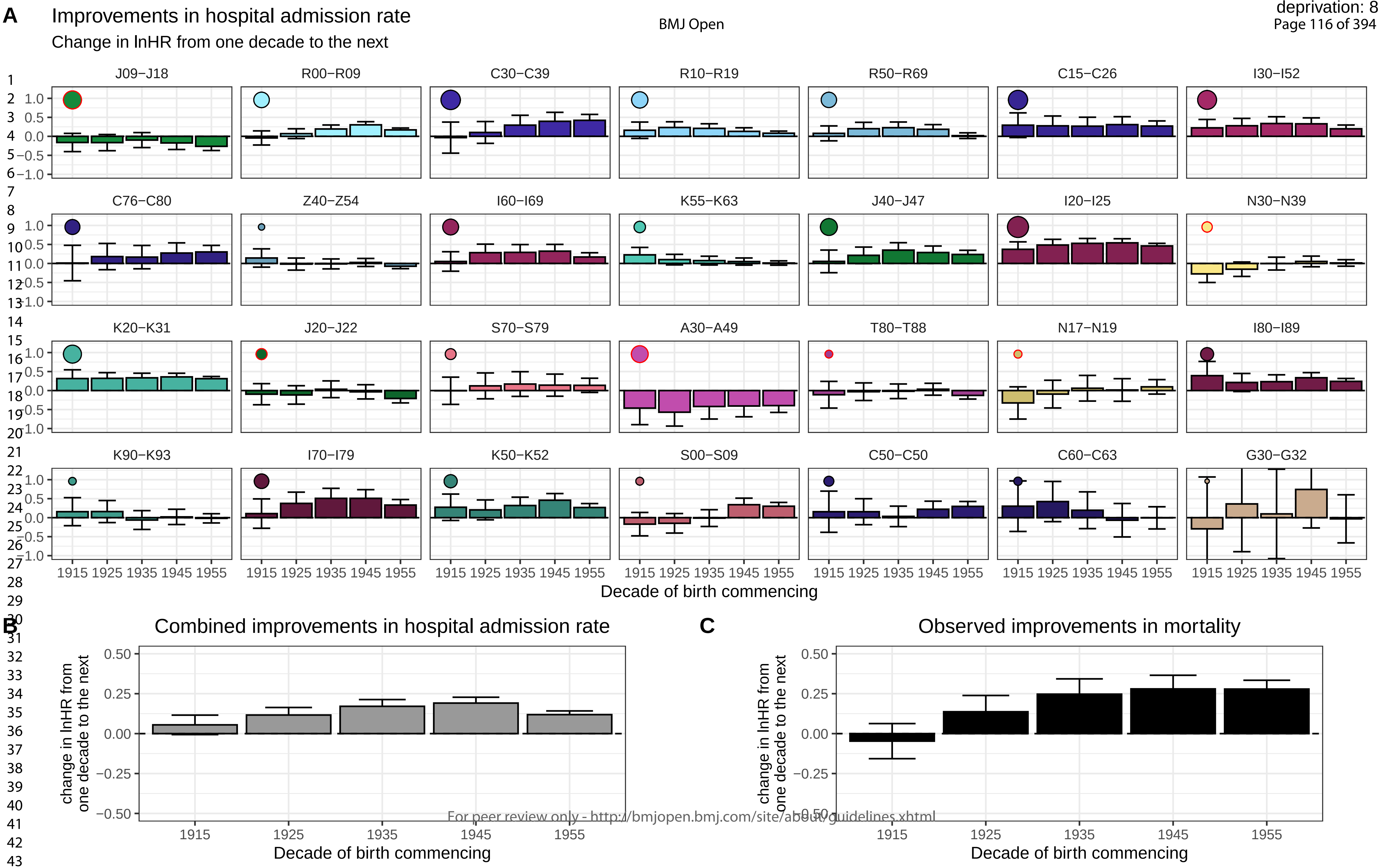

For peer review only - http://bmjopen.bmj.com/site/about/guidelines.xhtml

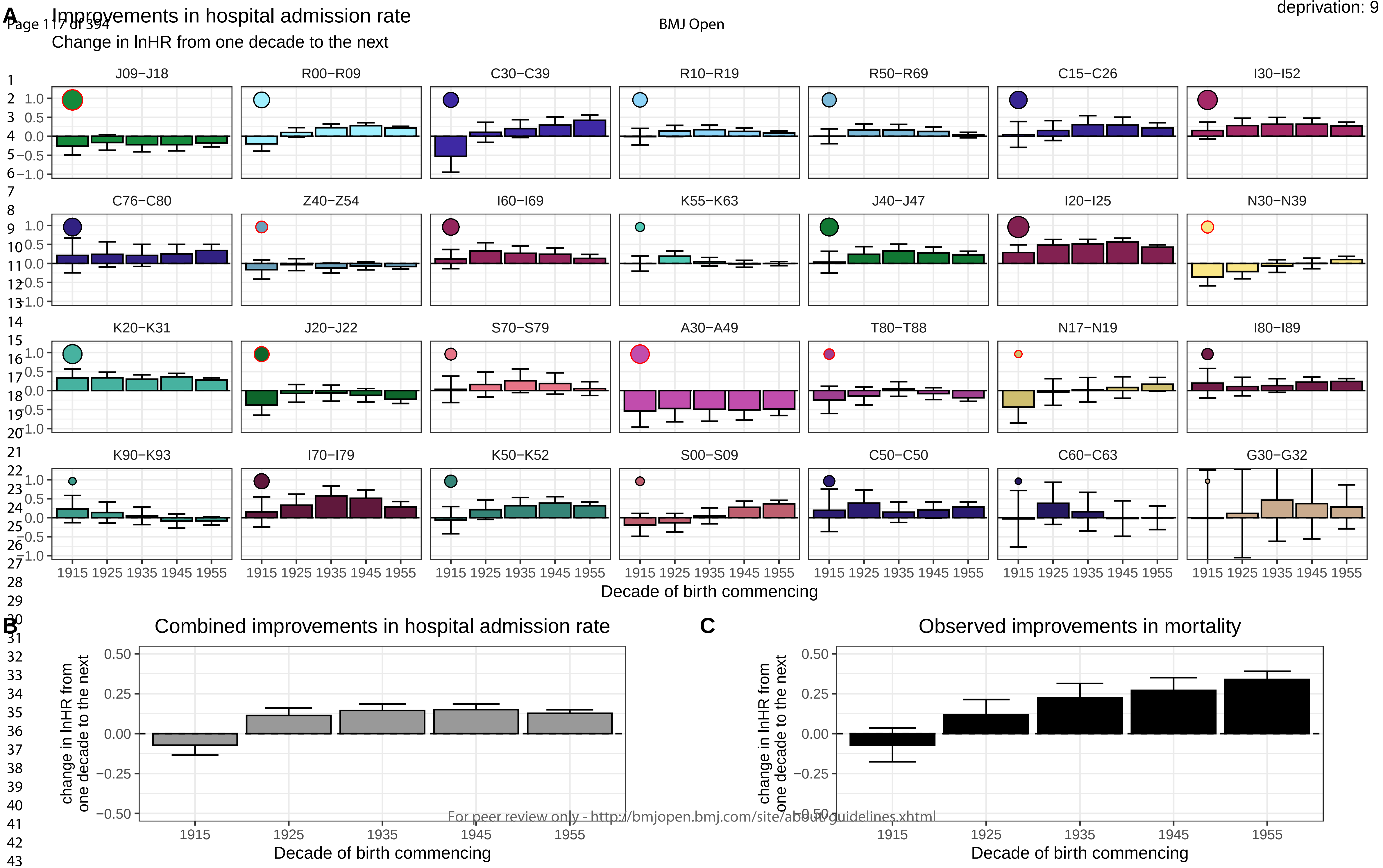

For peer review only - http://bmjopen.bmj.com/site/about/guidelines.xhtml

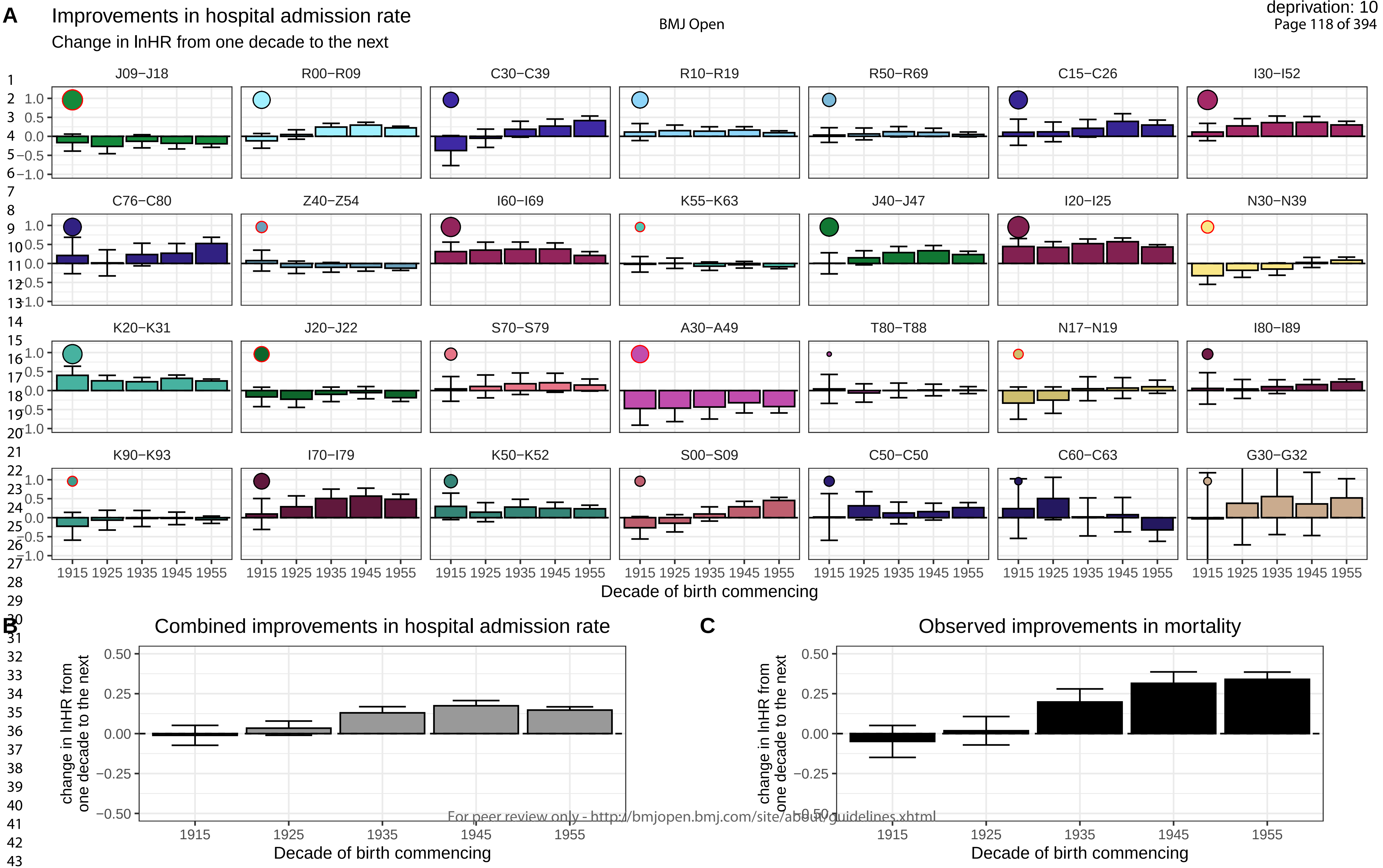

For peer review only - http://bmjopen.bmj.com/site/about/guidelines.xhtml

ICD10	Disease
J09-J18	Influenza and pneumonia
R00-R09	Symptoms and signs involving the circulatory and respiratory systems
C30-C39	Malignant neoplasm of respiratory and intrathoracic organs
R10-R19	Symptoms and signs involving the digestive system and abdomen
R50-R69	General symptoms and signs
C15-C26	Malignant neoplasms of the digestive organs
I30-I52	Other forms of heart disease
C76-C80	Malignant neoplasms of ill defined, secondary and unspecified sites
Z40-Z54	Persons encountering health services for specific procedures and health care
I60-I69	Cerebrovascular diseases
K55-K63	Other diseases of intestines
J40-J47	Chronic lower respiratory diseases
I20-I25	Ischaemic heart diseases
N30-N39	Other diseases of the urinary system
K20-K31	Diseases of oesophagus, stomach and duodenum
J20-J22	Other acute lower respiratory infections
S70-S79	Injuries to the hip and thigh
A30-A49	Other bacterial diseases
T80-T88	Complications of surgical and medical care, not elsewhere classified
N17-N19	Renal failure
I80-I89	Diseases of veins, lymphatic vessels and lymph nodes, not elsewhere classified
K90-K93	Other diseases of the digestive system
I70-I79	Diseases of arteries, arterioles and capillaries
K50-K52	Non infective enteritis and colitis
S00-S09	Injuries to the head
R25-R29	Symptoms and signs involving the nervous and musculoskeletal systems
K70-K77	Diseases of liver
C50-C50	Malignant neoplasm of breast
R30-R39	Symptoms and signs involving the urinary system
D60-D64	Aplastic and other anaemias
K80-K87	Diseases of gallbladder, biliary tract and pancreas
L00-L08	Infections of the skin and subcutaneous tissue
R40-R46	Symptoms and signs involving cognition, perception, emotional state and behaviour
E70-E90	Metabolic disorders
C81-C96	Malignant neoplasm of lymphoid, haematopoietic and related tissue
R90-R94	Abnormal findings on diagnostic imaging and in function studies, without diagnosis
J60-J70	Lung diseases due to external agents
G40-G47	Episodic and paroxysmal disorders
Z00-Z13	Persons encountering health services for examination and investigation
C51-C58	Malignant neoplasms of female genital organs
J90-J94	Other diseases of the pleura
I26-I28	Pulmonary heart disease and diseases of pulmonary circulation
T36-T50	Poisoning by, adverse effects of and underdosing of drugs, medicaments and biological substan
C64-C68	Malignant neoplasm of urinary tract
J95-J99	Other diseases of the respiratory system
M50-M54	Other dorsopathies
M70-M79	Other soft tissue disorders
F10-F19	Mental and behavioral disorders due to psychoactive substance abuse
A00-A09	Intestinal infectious diseases

For peer review only

C60-C63	Malignant neoplasms of male genital organs
D37-D48	Neoplasms of uncertain or unknown behaviour
F00-F09	Mental disorders due to known physiological conditions
D50-D53	Nutritional anaemias
E10-E14	Diabetes mellitus
L80-L99	Other disorders of the skin and subcutaneous tissue
S80-S89	Injuries to the knee and lower leg
S40-S49	Injuries to the shoulder and upper arm
K00-K14	Diseases of oral cavity and salivary glands
S30-S39	Injuries to abdomen, lower back, lumbar spine,pelvis and external genitals
M20-M25	Other joint disorders
M05-M14	Inflammatory polyarthropathies
I95-I99	Other and unspecified disorders of the circulatory system
D10-D36	Benign neoplasms
S50-S59	Injuries to the elbow and forearm
J30-J39	Other diseases of the upper respiratory tract
C43-C44	Melanoma and other malignant neoplasms of skin
S60-S69	Injuries to the wrist, hand and fingers
N20-N23	Urolithiasis
K40-K46	Hernia
N40-N51	Diseases of male genital organs
L60-L75	Disorders of skin appendages
N80-N98	Noninflammatory disorders of female genital tract
H00-H06	Disorders of the eyelid, lacrimal system and orbit
H25-H28	Disorders of the lens
M65-M68	Disorders of synovium and tendon
G50-G59	Nerve, nerve root and plexus disorders
M15-M19	Arthrosis
TOTAL	

For peer review only

Hospital Visits	Mortality (lnHR)	Relative Burden of Death	Weight
110,985	1.66	0.0939	0.0497
225,504	0.73	0.0841	0.0445
54,178	3.05	0.0840	0.0445
174,055	0.94	0.0831	0.0440
157,357	0.98	0.0786	0.0416
71,981	2.10	0.0767	0.0406
142,898	0.99	0.0720	0.0382
39,339	3.26	0.0653	0.0346
157,841	0.77	0.0614	0.0325
100,907	1.12	0.0573	0.0304
206,178	0.54	0.0569	0.0302
78,467	1.39	0.0553	0.0293
175,605	0.61	0.0542	0.0287
126,329	0.84	0.0538	0.0285
172,206	0.61	0.0530	0.0281
77,520	1.27	0.0501	0.0266
78,231	0.97	0.0386	0.0204
33,613	1.89	0.0323	0.0171
75,217	0.84	0.0322	0.0171
37,213	1.64	0.0310	0.0164
84,073	0.66	0.0283	0.0150
47,091	1.09	0.0262	0.0139
47,410	1.08	0.0261	0.0138
59,183	0.82	0.0247	0.0131
64,925	0.74	0.0243	0.0129
43,879	1.07	0.0238	0.0126
20,000	2.31	0.0235	0.0124
39,358	1.16	0.0233	0.0123
83,268	0.51	0.0217	0.0115
36,646	1.15	0.0215	0.0114
88,750	0.47	0.0211	0.0112
49,670	0.83	0.0210	0.0111
43,698	0.94	0.0209	0.0111
27,143	1.41	0.0195	0.0103
21,056	1.81	0.0193	0.0102
23,144	1.63	0.0192	0.0102
15,076	2.49	0.0191	0.0101
46,888	0.71	0.0169	0.0089
87,800	0.37	0.0164	0.0087
16,066	1.99	0.0162	0.0086
17,344	1.84	0.0162	0.0086
21,339	1.36	0.0147	0.0078
24,568	1.16	0.0145	0.0077
22,227	1.27	0.0144	0.0076
16,069	1.75	0.0143	0.0076
43,086	0.64	0.0141	0.0075
74,798	0.36	0.0136	0.0072
15,859	1.65	0.0133	0.0071
26,830	0.98	0.0133	0.0070

For peer review only

22,312	1.17	0.0133	0.0070
23,442	1.07	0.0127	0.0067
20,555	1.16	0.0122	0.0064
30,807	0.72	0.0113	0.0060
16,979	1.30	0.0112	0.0059
29,041	0.74	0.0109	0.0058
37,008	0.50	0.0094	0.0050
23,854	0.67	0.0081	0.0043
24,895	0.61	0.0077	0.0041
19,420	0.74	0.0073	0.0039
71,525	0.18	0.0065	0.0034
20,818	0.58	0.0061	0.0032
14,817	0.77	0.0058	0.0031
98,998	0.11	0.0058	0.0031
32,200	0.30	0.0050	0.0026
31,730	0.29	0.0046	0.0025
41,279	0.18	0.0037	0.0020
20,735	0.30	0.0032	0.0017
23,572	0.17	0.0020	0.0011
116,941	0.03	0.0020	0.0010
50,936	-0.01	-0.0003	-0.0002
24,006	-0.03	-0.0004	-0.0002
89,156	-0.01	-0.0007	-0.0004
22,633	-0.07	-0.0008	-0.0004
196,082	-0.02	-0.0022	-0.0012
15,091	-0.36	-0.0027	-0.0015
36,734	-0.22	-0.0040	-0.0021
112,390	-0.44	-0.0251	-0.0133
4,770,824	0.78	1.8874	1.0000

For peer review only

block	strat_level	birth_decade	burden	severity	se_severity	variable
ACT-DTH	-1	1915	NA	NA	NA	incidence
ACT-DTH	-1	1915	NA	NA	NA	impact
ACT-DTH	-1	1925	NA	NA	NA	incidence
ACT-DTH	-1	1925	NA	NA	NA	impact
ACT-DTH	-1	1935	NA	NA	NA	incidence
ACT-DTH	-1	1935	NA	NA	NA	impact
ACT-DTH	-1	1945	NA	NA	NA	incidence
ACT-DTH	-1	1945	NA	NA	NA	impact
ACT-DTH	-1	1955	NA	NA	NA	incidence
ACT-DTH	-1	1955	NA	NA	NA	impact
EST-DTH	-1	1915	1.3825	0.18660753	0.04165244	incidence
EST-DTH	-1	1915	1.3825	0.18660753	0.04165244	impact
EST-DTH	-1	1925	1.3825	0.18660753	0.04165244	incidence
EST-DTH	-1	1925	1.3825	0.18660753	0.04165244	impact
EST-DTH	-1	1935	1.3825	0.18660753	0.04165244	incidence
EST-DTH	-1	1935	1.3825	0.18660753	0.04165244	impact
EST-DTH	-1	1945	1.3825	0.18660753	0.04165244	incidence
EST-DTH	-1	1945	1.3825	0.18660753	0.04165244	impact
EST-DTH	-1	1955	1.3825	0.18660753	0.04165244	incidence
EST-DTH	-1	1955	1.3825	0.18660753	0.04165244	impact
J09-J18	-1	1915	0.09388	0.1548	0.0359	incidence
J09-J18	-1	1915	0.09388	0.1548	0.0359	impact
J09-J18	-1	1925	0.09388	0.1548	0.0359	incidence
J09-J18	-1	1925	0.09388	0.1548	0.0359	impact
J09-J18	-1	1935	0.09388	0.1548	0.0359	incidence
J09-J18	-1	1935	0.09388	0.1548	0.0359	impact
J09-J18	-1	1945	0.09388	0.1548	0.0359	incidence
J09-J18	-1	1945	0.09388	0.1548	0.0359	impact
J09-J18	-1	1955	0.09388	0.1548	0.0359	incidence
J09-J18	-1	1955	0.09388	0.1548	0.0359	impact
R00-R09	-1	1915	0.08403	0.2337	0.0402	incidence
R00-R09	-1	1915	0.08403	0.2337	0.0402	impact
R00-R09	-1	1925	0.08403	0.2337	0.0402	incidence
R00-R09	-1	1925	0.08403	0.2337	0.0402	impact
R00-R09	-1	1935	0.08403	0.2337	0.0402	incidence
R00-R09	-1	1935	0.08403	0.2337	0.0402	impact
R00-R09	-1	1945	0.08403	0.2337	0.0402	incidence
R00-R09	-1	1945	0.08403	0.2337	0.0402	impact
R00-R09	-1	1955	0.08403	0.2337	0.0402	incidence
R00-R09	-1	1955	0.08403	0.2337	0.0402	impact
C30-C39	-1	1915	0.084	0.2096	0.0466	incidence
C30-C39	-1	1915	0.084	0.2096	0.0466	impact

For peer review only

C30-C39	-1	1925	0.084	0.2096	0.0466	incidence
C30-C39	-1	1925	0.084	0.2096	0.0466	impact
C30-C39	-1	1935	0.084	0.2096	0.0466	incidence
C30-C39	-1	1935	0.084	0.2096	0.0466	impact
C30-C39	-1	1945	0.084	0.2096	0.0466	incidence
C30-C39	-1	1945	0.084	0.2096	0.0466	impact
C30-C39	-1	1955	0.084	0.2096	0.0466	incidence
C30-C39	-1	1955	0.084	0.2096	0.0466	impact
R10-R19	-1	1915	0.08308	0.1173	0.0444	incidence
R10-R19	-1	1915	0.08308	0.1173	0.0444	impact
R10-R19	-1	1925	0.08308	0.1173	0.0444	incidence
R10-R19	-1	1925	0.08308	0.1173	0.0444	impact
R10-R19	-1	1935	0.08308	0.1173	0.0444	incidence
R10-R19	-1	1935	0.08308	0.1173	0.0444	impact
R10-R19	-1	1945	0.08308	0.1173	0.0444	incidence
R10-R19	-1	1945	0.08308	0.1173	0.0444	impact
R10-R19	-1	1955	0.08308	0.1173	0.0444	incidence
R10-R19	-1	1955	0.08308	0.1173	0.0444	impact
R50-R69	-1	1915	0.07855	0.0259	0.0252	incidence
R50-R69	-1	1915	0.07855	0.0259	0.0252	impact
R50-R69	-1	1925	0.07855	0.0259	0.0252	incidence
R50-R69	-1	1925	0.07855	0.0259	0.0252	impact
R50-R69	-1	1935	0.07855	0.0259	0.0252	incidence
R50-R69	-1	1935	0.07855	0.0259	0.0252	impact
R50-R69	-1	1945	0.07855	0.0259	0.0252	incidence
R50-R69	-1	1945	0.07855	0.0259	0.0252	impact
R50-R69	-1	1955	0.07855	0.0259	0.0252	incidence
R50-R69	-1	1955	0.07855	0.0259	0.0252	impact
C15-C26	-1	1915	0.07666	0.3954	0.036	incidence
C15-C26	-1	1915	0.07666	0.3954	0.036	impact
C15-C26	-1	1925	0.07666	0.3954	0.036	incidence
C15-C26	-1	1925	0.07666	0.3954	0.036	impact
C15-C26	-1	1935	0.07666	0.3954	0.036	incidence
C15-C26	-1	1935	0.07666	0.3954	0.036	impact
C15-C26	-1	1945	0.07666	0.3954	0.036	incidence
C15-C26	-1	1945	0.07666	0.3954	0.036	impact
C15-C26	-1	1955	0.07666	0.3954	0.036	incidence
C15-C26	-1	1955	0.07666	0.3954	0.036	impact
I30-I52	-1	1915	0.07206	0.1802	0.0209	incidence
I30-I52	-1	1915	0.07206	0.1802	0.0209	impact
I30-I52	-1	1925	0.07206	0.1802	0.0209	incidence
I30-I52	-1	1925	0.07206	0.1802	0.0209	impact
I30-I52	-1	1935	0.07206	0.1802	0.0209	incidence

For peer review only

I30-I52	-1	1935	0.07206	0.1802	0.0209	impact
I30-I52	-1	1945	0.07206	0.1802	0.0209	incidence
I30-I52	-1	1945	0.07206	0.1802	0.0209	impact
I30-I52	-1	1955	0.07206	0.1802	0.0209	incidence
I30-I52	-1	1955	0.07206	0.1802	0.0209	impact
C76-C80	-1	1915	0.06525	0.3658	0.0653	incidence
C76-C80	-1	1915	0.06525	0.3658	0.0653	impact
C76-C80	-1	1925	0.06525	0.3658	0.0653	incidence
C76-C80	-1	1925	0.06525	0.3658	0.0653	impact
C76-C80	-1	1935	0.06525	0.3658	0.0653	incidence
C76-C80	-1	1935	0.06525	0.3658	0.0653	impact
C76-C80	-1	1945	0.06525	0.3658	0.0653	incidence
C76-C80	-1	1945	0.06525	0.3658	0.0653	impact
C76-C80	-1	1955	0.06525	0.3658	0.0653	incidence
C76-C80	-1	1955	0.06525	0.3658	0.0653	impact
Z40-Z54	-1	1915	0.06138	0.2963	0.0353	incidence
Z40-Z54	-1	1915	0.06138	0.2963	0.0353	impact
Z40-Z54	-1	1925	0.06138	0.2963	0.0353	incidence
Z40-Z54	-1	1925	0.06138	0.2963	0.0353	impact
Z40-Z54	-1	1935	0.06138	0.2963	0.0353	incidence
Z40-Z54	-1	1935	0.06138	0.2963	0.0353	impact
Z40-Z54	-1	1945	0.06138	0.2963	0.0353	incidence
Z40-Z54	-1	1945	0.06138	0.2963	0.0353	impact
Z40-Z54	-1	1955	0.06138	0.2963	0.0353	incidence
Z40-Z54	-1	1955	0.06138	0.2963	0.0353	impact
I60-I69	-1	1915	0.05735	0.2033	0.0338	incidence
I60-I69	-1	1915	0.05735	0.2033	0.0338	impact
I60-I69	-1	1925	0.05735	0.2033	0.0338	incidence
I60-I69	-1	1925	0.05735	0.2033	0.0338	impact
I60-I69	-1	1935	0.05735	0.2033	0.0338	incidence
I60-I69	-1	1935	0.05735	0.2033	0.0338	impact
I60-I69	-1	1945	0.05735	0.2033	0.0338	incidence
I60-I69	-1	1945	0.05735	0.2033	0.0338	impact
I60-I69	-1	1955	0.05735	0.2033	0.0338	incidence
I60-I69	-1	1955	0.05735	0.2033	0.0338	impact
K55-K63	-1	1915	0.05691	0.2234	0.0224	incidence
K55-K63	-1	1915	0.05691	0.2234	0.0224	impact
K55-K63	-1	1925	0.05691	0.2234	0.0224	incidence
K55-K63	-1	1925	0.05691	0.2234	0.0224	impact
K55-K63	-1	1935	0.05691	0.2234	0.0224	incidence
K55-K63	-1	1935	0.05691	0.2234	0.0224	impact
K55-K63	-1	1945	0.05691	0.2234	0.0224	incidence
K55-K63	-1	1945	0.05691	0.2234	0.0224	impact

For peer review only

K55-K63	-1	1955	0.05691	0.2234	0.0224 incidence
K55-K63	-1	1955	0.05691	0.2234	0.0224 impact
J40-J47	-1	1915	0.05525	0.238	0.0465 incidence
J40-J47	-1	1915	0.05525	0.238	0.0465 impact
J40-J47	-1	1925	0.05525	0.238	0.0465 incidence
J40-J47	-1	1925	0.05525	0.238	0.0465 impact
J40-J47	-1	1935	0.05525	0.238	0.0465 incidence
J40-J47	-1	1935	0.05525	0.238	0.0465 impact
J40-J47	-1	1945	0.05525	0.238	0.0465 incidence
J40-J47	-1	1945	0.05525	0.238	0.0465 impact
J40-J47	-1	1955	0.05525	0.238	0.0465 incidence
J40-J47	-1	1955	0.05525	0.238	0.0465 impact
I20-I25	-1	1915	0.05419	0.2639	0.0342 incidence
I20-I25	-1	1915	0.05419	0.2639	0.0342 impact
I20-I25	-1	1925	0.05419	0.2639	0.0342 incidence
I20-I25	-1	1925	0.05419	0.2639	0.0342 impact
I20-I25	-1	1935	0.05419	0.2639	0.0342 incidence
I20-I25	-1	1935	0.05419	0.2639	0.0342 impact
I20-I25	-1	1945	0.05419	0.2639	0.0342 incidence
I20-I25	-1	1945	0.05419	0.2639	0.0342 impact
I20-I25	-1	1955	0.05419	0.2639	0.0342 incidence
I20-I25	-1	1955	0.05419	0.2639	0.0342 impact
N30-N39	-1	1915	0.05382	-0.2148	0.0395 incidence
N30-N39	-1	1915	0.05382	-0.2148	0.0395 impact
N30-N39	-1	1925	0.05382	-0.2148	0.0395 incidence
N30-N39	-1	1925	0.05382	-0.2148	0.0395 impact
N30-N39	-1	1935	0.05382	-0.2148	0.0395 incidence
N30-N39	-1	1935	0.05382	-0.2148	0.0395 impact
N30-N39	-1	1945	0.05382	-0.2148	0.0395 incidence
N30-N39	-1	1945	0.05382	-0.2148	0.0395 impact
N30-N39	-1	1955	0.05382	-0.2148	0.0395 incidence
N30-N39	-1	1955	0.05382	-0.2148	0.0395 impact
K20-K31	-1	1915	0.05296	0.1335	0.0491 incidence
K20-K31	-1	1915	0.05296	0.1335	0.0491 impact
K20-K31	-1	1925	0.05296	0.1335	0.0491 incidence
K20-K31	-1	1925	0.05296	0.1335	0.0491 impact
K20-K31	-1	1935	0.05296	0.1335	0.0491 incidence
K20-K31	-1	1935	0.05296	0.1335	0.0491 impact
K20-K31	-1	1945	0.05296	0.1335	0.0491 incidence
K20-K31	-1	1945	0.05296	0.1335	0.0491 impact
K20-K31	-1	1955	0.05296	0.1335	0.0491 incidence
K20-K31	-1	1955	0.05296	0.1335	0.0491 impact
J20-J22	-1	1915	0.05013	-0.1073	0.0242 incidence

For peer review only

J20-J22	-1	1915	0.05013	-0.1073	0.0242 impact
J20-J22	-1	1925	0.05013	-0.1073	0.0242 incidence
J20-J22	-1	1925	0.05013	-0.1073	0.0242 impact
J20-J22	-1	1935	0.05013	-0.1073	0.0242 incidence
J20-J22	-1	1935	0.05013	-0.1073	0.0242 impact
J20-J22	-1	1945	0.05013	-0.1073	0.0242 incidence
J20-J22	-1	1945	0.05013	-0.1073	0.0242 impact
J20-J22	-1	1955	0.05013	-0.1073	0.0242 incidence
J20-J22	-1	1955	0.05013	-0.1073	0.0242 impact
S70-S79	-1	1915	0.03858	-0.0199	0.0211 incidence
S70-S79	-1	1915	0.03858	-0.0199	0.0211 impact
S70-S79	-1	1925	0.03858	-0.0199	0.0211 incidence
S70-S79	-1	1925	0.03858	-0.0199	0.0211 impact
S70-S79	-1	1935	0.03858	-0.0199	0.0211 incidence
S70-S79	-1	1935	0.03858	-0.0199	0.0211 impact
S70-S79	-1	1945	0.03858	-0.0199	0.0211 incidence
S70-S79	-1	1945	0.03858	-0.0199	0.0211 impact
S70-S79	-1	1955	0.03858	-0.0199	0.0211 incidence
S70-S79	-1	1955	0.03858	-0.0199	0.0211 impact
A30-A49	-1	1915	0.03224	0.0633	0.1027 incidence
A30-A49	-1	1915	0.03224	0.0633	0.1027 impact
A30-A49	-1	1925	0.03224	0.0633	0.1027 incidence
A30-A49	-1	1925	0.03224	0.0633	0.1027 impact
A30-A49	-1	1935	0.03224	0.0633	0.1027 incidence
A30-A49	-1	1935	0.03224	0.0633	0.1027 impact
A30-A49	-1	1945	0.03224	0.0633	0.1027 incidence
A30-A49	-1	1945	0.03224	0.0633	0.1027 impact
A30-A49	-1	1955	0.03224	0.0633	0.1027 incidence
A30-A49	-1	1955	0.03224	0.0633	0.1027 impact
T80-T88	-1	1915	0.03223	0.1835	0.0631 incidence
T80-T88	-1	1915	0.03223	0.1835	0.0631 impact
T80-T88	-1	1925	0.03223	0.1835	0.0631 incidence
T80-T88	-1	1925	0.03223	0.1835	0.0631 impact
T80-T88	-1	1935	0.03223	0.1835	0.0631 incidence
T80-T88	-1	1935	0.03223	0.1835	0.0631 impact
T80-T88	-1	1945	0.03223	0.1835	0.0631 incidence
T80-T88	-1	1945	0.03223	0.1835	0.0631 impact
T80-T88	-1	1955	0.03223	0.1835	0.0631 incidence
T80-T88	-1	1955	0.03223	0.1835	0.0631 impact
N17-N19	-1	1915	0.03099	0.2032	0.0578 incidence
N17-N19	-1	1915	0.03099	0.2032	0.0578 impact
N17-N19	-1	1925	0.03099	0.2032	0.0578 incidence
N17-N19	-1	1925	0.03099	0.2032	0.0578 impact

For peer review only

N17-N19	-1	1935	0.03099	0.2032	0.0578	incidence
N17-N19	-1	1935	0.03099	0.2032	0.0578	impact
N17-N19	-1	1945	0.03099	0.2032	0.0578	incidence
N17-N19	-1	1945	0.03099	0.2032	0.0578	impact
N17-N19	-1	1955	0.03099	0.2032	0.0578	incidence
N17-N19	-1	1955	0.03099	0.2032	0.0578	impact
I80-I89	-1	1915	0.02834	0.2381	0.0471	incidence
I80-I89	-1	1915	0.02834	0.2381	0.0471	impact
I80-I89	-1	1925	0.02834	0.2381	0.0471	incidence
I80-I89	-1	1925	0.02834	0.2381	0.0471	impact
I80-I89	-1	1935	0.02834	0.2381	0.0471	incidence
I80-I89	-1	1935	0.02834	0.2381	0.0471	impact
I80-I89	-1	1945	0.02834	0.2381	0.0471	incidence
I80-I89	-1	1945	0.02834	0.2381	0.0471	impact
I80-I89	-1	1955	0.02834	0.2381	0.0471	incidence
I80-I89	-1	1955	0.02834	0.2381	0.0471	impact
K90-K93	-1	1915	0.02617	0.1852	0.0703	incidence
K90-K93	-1	1915	0.02617	0.1852	0.0703	impact
K90-K93	-1	1925	0.02617	0.1852	0.0703	incidence
K90-K93	-1	1925	0.02617	0.1852	0.0703	impact
K90-K93	-1	1935	0.02617	0.1852	0.0703	incidence
K90-K93	-1	1935	0.02617	0.1852	0.0703	impact
K90-K93	-1	1945	0.02617	0.1852	0.0703	incidence
K90-K93	-1	1945	0.02617	0.1852	0.0703	impact
K90-K93	-1	1955	0.02617	0.1852	0.0703	incidence
K90-K93	-1	1955	0.02617	0.1852	0.0703	impact
I70-I79	-1	1915	0.02608	0.091	0.0388	incidence
I70-I79	-1	1915	0.02608	0.091	0.0388	impact
I70-I79	-1	1925	0.02608	0.091	0.0388	incidence
I70-I79	-1	1925	0.02608	0.091	0.0388	impact
I70-I79	-1	1935	0.02608	0.091	0.0388	incidence
I70-I79	-1	1935	0.02608	0.091	0.0388	impact
I70-I79	-1	1945	0.02608	0.091	0.0388	incidence
I70-I79	-1	1945	0.02608	0.091	0.0388	impact
I70-I79	-1	1955	0.02608	0.091	0.0388	incidence
I70-I79	-1	1955	0.02608	0.091	0.0388	impact
K50-K52	-1	1915	0.02464	0.1161	0.0314	incidence
K50-K52	-1	1915	0.02464	0.1161	0.0314	impact
K50-K52	-1	1925	0.02464	0.1161	0.0314	incidence
K50-K52	-1	1925	0.02464	0.1161	0.0314	impact
K50-K52	-1	1935	0.02464	0.1161	0.0314	incidence
K50-K52	-1	1935	0.02464	0.1161	0.0314	impact
K50-K52	-1	1945	0.02464	0.1161	0.0314	incidence

For peer review only

K50-K52	-1	1945	0.02464	0.1161	0.0314	impact
K50-K52	-1	1955	0.02464	0.1161	0.0314	incidence
K50-K52	-1	1955	0.02464	0.1161	0.0314	impact
S00-S09	-1	1915	0.02432	0.0155	0.0484	incidence
S00-S09	-1	1915	0.02432	0.0155	0.0484	impact
S00-S09	-1	1925	0.02432	0.0155	0.0484	incidence
S00-S09	-1	1925	0.02432	0.0155	0.0484	impact
S00-S09	-1	1935	0.02432	0.0155	0.0484	incidence
S00-S09	-1	1935	0.02432	0.0155	0.0484	impact
S00-S09	-1	1945	0.02432	0.0155	0.0484	incidence
S00-S09	-1	1945	0.02432	0.0155	0.0484	impact
S00-S09	-1	1955	0.02432	0.0155	0.0484	incidence
S00-S09	-1	1955	0.02432	0.0155	0.0484	impact
C50-C50	-1	1915	0.02331	1.1606	0.0688	incidence
C50-C50	-1	1915	0.02331	1.1606	0.0688	impact
C50-C50	-1	1925	0.02331	1.1606	0.0688	incidence
C50-C50	-1	1925	0.02331	1.1606	0.0688	impact
C50-C50	-1	1935	0.02331	1.1606	0.0688	incidence
C50-C50	-1	1935	0.02331	1.1606	0.0688	impact
C50-C50	-1	1945	0.02331	1.1606	0.0688	incidence
C50-C50	-1	1945	0.02331	1.1606	0.0688	impact
C50-C50	-1	1955	0.02331	1.1606	0.0688	incidence
C50-C50	-1	1955	0.02331	1.1606	0.0688	impact
C60-C63	-1	1915	0.01328	0.6983	0.0669	incidence
C60-C63	-1	1915	0.01328	0.6983	0.0669	impact
C60-C63	-1	1925	0.01328	0.6983	0.0669	incidence
C60-C63	-1	1925	0.01328	0.6983	0.0669	impact
C60-C63	-1	1935	0.01328	0.6983	0.0669	incidence
C60-C63	-1	1935	0.01328	0.6983	0.0669	impact
C60-C63	-1	1945	0.01328	0.6983	0.0669	incidence
C60-C63	-1	1945	0.01328	0.6983	0.0669	impact
C60-C63	-1	1955	0.01328	0.6983	0.0669	incidence
C60-C63	-1	1955	0.01328	0.6983	0.0669	impact
G30-G32	-1	1915	0.00282	0.0375	0.1439	incidence
G30-G32	-1	1915	0.00282	0.0375	0.1439	impact
G30-G32	-1	1925	0.00282	0.0375	0.1439	incidence
G30-G32	-1	1925	0.00282	0.0375	0.1439	impact
G30-G32	-1	1935	0.00282	0.0375	0.1439	incidence
G30-G32	-1	1935	0.00282	0.0375	0.1439	impact
G30-G32	-1	1945	0.00282	0.0375	0.1439	incidence
G30-G32	-1	1945	0.00282	0.0375	0.1439	impact
G30-G32	-1	1955	0.00282	0.0375	0.1439	incidence
G30-G32	-1	1955	0.00282	0.0375	0.1439	impact

For peer review only

ACT-DTH	1	1915	NA	NA	NA	incidence
ACT-DTH	1	1915	NA	NA	NA	impact
ACT-DTH	1	1925	NA	NA	NA	incidence
ACT-DTH	1	1925	NA	NA	NA	impact
ACT-DTH	1	1935	NA	NA	NA	incidence
ACT-DTH	1	1935	NA	NA	NA	impact
ACT-DTH	1	1945	NA	NA	NA	incidence
ACT-DTH	1	1945	NA	NA	NA	impact
ACT-DTH	1	1955	NA	NA	NA	incidence
ACT-DTH	1	1955	NA	NA	NA	impact
ACT-DTH	2	1915	NA	NA	NA	incidence
ACT-DTH	2	1915	NA	NA	NA	impact
ACT-DTH	2	1925	NA	NA	NA	incidence
ACT-DTH	2	1925	NA	NA	NA	impact
ACT-DTH	2	1935	NA	NA	NA	incidence
ACT-DTH	2	1935	NA	NA	NA	impact
ACT-DTH	2	1945	NA	NA	NA	incidence
ACT-DTH	2	1945	NA	NA	NA	impact
ACT-DTH	2	1955	NA	NA	NA	incidence
ACT-DTH	2	1955	NA	NA	NA	impact
EST-DTH	1	1915	1.425335	0.19129289	0.05351186	incidence
EST-DTH	1	1915	1.425335	0.19129289	0.05351186	impact
EST-DTH	1	1925	1.425335	0.19129289	0.05351186	incidence
EST-DTH	1	1925	1.425335	0.19129289	0.05351186	impact
EST-DTH	1	1935	1.425335	0.19129289	0.05351186	incidence
EST-DTH	1	1935	1.425335	0.19129289	0.05351186	impact
EST-DTH	1	1945	1.425335	0.19129289	0.05351186	incidence
EST-DTH	1	1945	1.425335	0.19129289	0.05351186	impact
EST-DTH	1	1955	1.425335	0.19129289	0.05351186	incidence
EST-DTH	1	1955	1.425335	0.19129289	0.05351186	impact
EST-DTH	2	1915	1.34704643	0.18227229	0.04817099	incidence
EST-DTH	2	1915	1.34704643	0.18227229	0.04817099	impact
EST-DTH	2	1925	1.34704643	0.18227229	0.04817099	incidence
EST-DTH	2	1925	1.34704643	0.18227229	0.04817099	impact
EST-DTH	2	1935	1.34704643	0.18227229	0.04817099	incidence
EST-DTH	2	1935	1.34704643	0.18227229	0.04817099	impact
EST-DTH	2	1945	1.34704643	0.18227229	0.04817099	incidence
EST-DTH	2	1945	1.34704643	0.18227229	0.04817099	impact
EST-DTH	2	1955	1.34704643	0.18227229	0.04817099	incidence
EST-DTH	2	1955	1.34704643	0.18227229	0.04817099	impact
J09-J18	1	1915	0.101	0.1144	0.0555	incidence
J09-J18	1	1915	0.101	0.1144	0.0555	impact
J09-J18	1	1925	0.101	0.1144	0.0555	incidence

For peer review only

J09-J18	1	1925	0.101	0.1144	0.0555	impact
J09-J18	1	1935	0.101	0.1144	0.0555	incidence
J09-J18	1	1935	0.101	0.1144	0.0555	impact
J09-J18	1	1945	0.101	0.1144	0.0555	incidence
J09-J18	1	1945	0.101	0.1144	0.0555	impact
J09-J18	1	1955	0.101	0.1144	0.0555	incidence
J09-J18	1	1955	0.101	0.1144	0.0555	impact
J09-J18	2	1915	0.0886	0.1945	0.032	incidence
J09-J18	2	1915	0.0886	0.1945	0.032	impact
J09-J18	2	1925	0.0886	0.1945	0.032	incidence
J09-J18	2	1925	0.0886	0.1945	0.032	impact
J09-J18	2	1935	0.0886	0.1945	0.032	incidence
J09-J18	2	1935	0.0886	0.1945	0.032	impact
J09-J18	2	1945	0.0886	0.1945	0.032	incidence
J09-J18	2	1945	0.0886	0.1945	0.032	impact
J09-J18	2	1955	0.0886	0.1945	0.032	incidence
J09-J18	2	1955	0.0886	0.1945	0.032	impact
R00-R09	1	1915	0.0909	0.2189	0.0504	incidence
R00-R09	1	1915	0.0909	0.2189	0.0504	impact
R00-R09	1	1925	0.0909	0.2189	0.0504	incidence
R00-R09	1	1925	0.0909	0.2189	0.0504	impact
R00-R09	1	1935	0.0909	0.2189	0.0504	incidence
R00-R09	1	1935	0.0909	0.2189	0.0504	impact
R00-R09	1	1945	0.0909	0.2189	0.0504	incidence
R00-R09	1	1945	0.0909	0.2189	0.0504	impact
R00-R09	1	1955	0.0909	0.2189	0.0504	incidence
R00-R09	1	1955	0.0909	0.2189	0.0504	impact
R00-R09	2	1915	0.0782	0.2416	0.0473	incidence
R00-R09	2	1915	0.0782	0.2416	0.0473	impact
R00-R09	2	1925	0.0782	0.2416	0.0473	incidence
R00-R09	2	1925	0.0782	0.2416	0.0473	impact
R00-R09	2	1935	0.0782	0.2416	0.0473	incidence
R00-R09	2	1935	0.0782	0.2416	0.0473	impact
R00-R09	2	1945	0.0782	0.2416	0.0473	incidence
R00-R09	2	1945	0.0782	0.2416	0.0473	impact
R00-R09	2	1955	0.0782	0.2416	0.0473	incidence
R00-R09	2	1955	0.0782	0.2416	0.0473	impact
C30-C39	1	1915	0.0942	0.213	0.0389	incidence
C30-C39	1	1915	0.0942	0.213	0.0389	impact
C30-C39	1	1925	0.0942	0.213	0.0389	incidence
C30-C39	1	1925	0.0942	0.213	0.0389	impact
C30-C39	1	1935	0.0942	0.213	0.0389	incidence
C30-C39	1	1935	0.0942	0.213	0.0389	impact

For peer review only

C30-C39	1	1945	0.0942	0.213	0.0389	incidence
C30-C39	1	1945	0.0942	0.213	0.0389	impact
C30-C39	1	1955	0.0942	0.213	0.0389	incidence
C30-C39	1	1955	0.0942	0.213	0.0389	impact
C30-C39	2	1915	0.0756	0.2028	0.0706	incidence
C30-C39	2	1915	0.0756	0.2028	0.0706	impact
C30-C39	2	1925	0.0756	0.2028	0.0706	incidence
C30-C39	2	1925	0.0756	0.2028	0.0706	impact
C30-C39	2	1935	0.0756	0.2028	0.0706	incidence
C30-C39	2	1935	0.0756	0.2028	0.0706	impact
C30-C39	2	1945	0.0756	0.2028	0.0706	incidence
C30-C39	2	1945	0.0756	0.2028	0.0706	impact
C30-C39	2	1955	0.0756	0.2028	0.0706	incidence
C30-C39	2	1955	0.0756	0.2028	0.0706	impact
R10-R19	1	1915	0.0746	0.103	0.0465	incidence
R10-R19	1	1915	0.0746	0.103	0.0465	impact
R10-R19	1	1925	0.0746	0.103	0.0465	incidence
R10-R19	1	1925	0.0746	0.103	0.0465	impact
R10-R19	1	1935	0.0746	0.103	0.0465	incidence
R10-R19	1	1935	0.0746	0.103	0.0465	impact
R10-R19	1	1945	0.0746	0.103	0.0465	incidence
R10-R19	1	1945	0.0746	0.103	0.0465	impact
R10-R19	1	1955	0.0746	0.103	0.0465	incidence
R10-R19	1	1955	0.0746	0.103	0.0465	impact
R10-R19	2	1915	0.09	0.1253	0.05	incidence
R10-R19	2	1915	0.09	0.1253	0.05	impact
R10-R19	2	1925	0.09	0.1253	0.05	incidence
R10-R19	2	1925	0.09	0.1253	0.05	impact
R10-R19	2	1935	0.09	0.1253	0.05	incidence
R10-R19	2	1935	0.09	0.1253	0.05	impact
R10-R19	2	1945	0.09	0.1253	0.05	incidence
R10-R19	2	1945	0.09	0.1253	0.05	impact
R10-R19	2	1955	0.09	0.1253	0.05	incidence
R10-R19	2	1955	0.09	0.1253	0.05	impact
R50-R69	1	1915	0.0829	0.0742	0.0334	incidence
R50-R69	1	1915	0.0829	0.0742	0.0334	impact
R50-R69	1	1925	0.0829	0.0742	0.0334	incidence
R50-R69	1	1925	0.0829	0.0742	0.0334	impact
R50-R69	1	1935	0.0829	0.0742	0.0334	incidence
R50-R69	1	1935	0.0829	0.0742	0.0334	impact
R50-R69	1	1945	0.0829	0.0742	0.0334	incidence
R50-R69	1	1945	0.0829	0.0742	0.0334	impact
R50-R69	1	1955	0.0829	0.0742	0.0334	incidence

For peer review only

R50-R69	1	1955	0.0829	0.0742	0.0334	impact
R50-R69	2	1915	0.0748	-0.0166	0.0428	incidence
R50-R69	2	1915	0.0748	-0.0166	0.0428	impact
R50-R69	2	1925	0.0748	-0.0166	0.0428	incidence
R50-R69	2	1925	0.0748	-0.0166	0.0428	impact
R50-R69	2	1935	0.0748	-0.0166	0.0428	incidence
R50-R69	2	1935	0.0748	-0.0166	0.0428	impact
R50-R69	2	1945	0.0748	-0.0166	0.0428	incidence
R50-R69	2	1945	0.0748	-0.0166	0.0428	impact
R50-R69	2	1955	0.0748	-0.0166	0.0428	incidence
R50-R69	2	1955	0.0748	-0.0166	0.0428	impact
C15-C26	1	1915	0.0913	0.366	0.0475	incidence
C15-C26	1	1915	0.0913	0.366	0.0475	impact
C15-C26	1	1925	0.0913	0.366	0.0475	incidence
C15-C26	1	1925	0.0913	0.366	0.0475	impact
C15-C26	1	1935	0.0913	0.366	0.0475	incidence
C15-C26	1	1935	0.0913	0.366	0.0475	impact
C15-C26	1	1945	0.0913	0.366	0.0475	incidence
C15-C26	1	1945	0.0913	0.366	0.0475	impact
C15-C26	1	1955	0.0913	0.366	0.0475	incidence
C15-C26	1	1955	0.0913	0.366	0.0475	impact
C15-C26	2	1915	0.064	0.4284	0.0499	incidence
C15-C26	2	1915	0.064	0.4284	0.0499	impact
C15-C26	2	1925	0.064	0.4284	0.0499	incidence
C15-C26	2	1925	0.064	0.4284	0.0499	impact
C15-C26	2	1935	0.064	0.4284	0.0499	incidence
C15-C26	2	1935	0.064	0.4284	0.0499	impact
C15-C26	2	1945	0.064	0.4284	0.0499	incidence
C15-C26	2	1945	0.064	0.4284	0.0499	impact
C15-C26	2	1955	0.064	0.4284	0.0499	incidence
C15-C26	2	1955	0.064	0.4284	0.0499	impact
I30-I52	1	1915	0.078	0.2884	0.0332	incidence
I30-I52	1	1915	0.078	0.2884	0.0332	impact
I30-I52	1	1925	0.078	0.2884	0.0332	incidence
I30-I52	1	1925	0.078	0.2884	0.0332	impact
I30-I52	1	1935	0.078	0.2884	0.0332	incidence
I30-I52	1	1935	0.078	0.2884	0.0332	impact
I30-I52	1	1945	0.078	0.2884	0.0332	incidence
I30-I52	1	1945	0.078	0.2884	0.0332	impact
I30-I52	1	1955	0.078	0.2884	0.0332	incidence
I30-I52	1	1955	0.078	0.2884	0.0332	impact
I30-I52	2	1915	0.0671	0.0783	0.0275	incidence
I30-I52	2	1915	0.0671	0.0783	0.0275	impact

For peer review only

I30-I52	2	1925	0.0671	0.0783	0.0275	incidence
I30-I52	2	1925	0.0671	0.0783	0.0275	impact
I30-I52	2	1935	0.0671	0.0783	0.0275	incidence
I30-I52	2	1935	0.0671	0.0783	0.0275	impact
I30-I52	2	1945	0.0671	0.0783	0.0275	incidence
I30-I52	2	1945	0.0671	0.0783	0.0275	impact
I30-I52	2	1955	0.0671	0.0783	0.0275	incidence
I30-I52	2	1955	0.0671	0.0783	0.0275	impact
C76-C80	1	1915	0.0626	0.2093	0.0866	incidence
C76-C80	1	1915	0.0626	0.2093	0.0866	impact
C76-C80	1	1925	0.0626	0.2093	0.0866	incidence
C76-C80	1	1925	0.0626	0.2093	0.0866	impact
C76-C80	1	1935	0.0626	0.2093	0.0866	incidence
C76-C80	1	1935	0.0626	0.2093	0.0866	impact
C76-C80	1	1945	0.0626	0.2093	0.0866	incidence
C76-C80	1	1945	0.0626	0.2093	0.0866	impact
C76-C80	1	1955	0.0626	0.2093	0.0866	incidence
C76-C80	1	1955	0.0626	0.2093	0.0866	impact
C76-C80	2	1915	0.0679	0.5031	0.0541	incidence
C76-C80	2	1915	0.0679	0.5031	0.0541	impact
C76-C80	2	1925	0.0679	0.5031	0.0541	incidence
C76-C80	2	1925	0.0679	0.5031	0.0541	impact
C76-C80	2	1935	0.0679	0.5031	0.0541	incidence
C76-C80	2	1935	0.0679	0.5031	0.0541	impact
C76-C80	2	1945	0.0679	0.5031	0.0541	incidence
C76-C80	2	1945	0.0679	0.5031	0.0541	impact
C76-C80	2	1955	0.0679	0.5031	0.0541	incidence
C76-C80	2	1955	0.0679	0.5031	0.0541	impact
Z40-Z54	1	1915	0.0656	0.364	0.0574	incidence
Z40-Z54	1	1915	0.0656	0.364	0.0574	impact
Z40-Z54	1	1925	0.0656	0.364	0.0574	incidence
Z40-Z54	1	1925	0.0656	0.364	0.0574	impact
Z40-Z54	1	1935	0.0656	0.364	0.0574	incidence
Z40-Z54	1	1935	0.0656	0.364	0.0574	impact
Z40-Z54	1	1945	0.0656	0.364	0.0574	incidence
Z40-Z54	1	1945	0.0656	0.364	0.0574	impact
Z40-Z54	1	1955	0.0656	0.364	0.0574	incidence
Z40-Z54	1	1955	0.0656	0.364	0.0574	impact
Z40-Z54	2	1915	0.0583	0.2137	0.0266	incidence
Z40-Z54	2	1915	0.0583	0.2137	0.0266	impact
Z40-Z54	2	1925	0.0583	0.2137	0.0266	incidence
Z40-Z54	2	1925	0.0583	0.2137	0.0266	impact
Z40-Z54	2	1935	0.0583	0.2137	0.0266	incidence

For peer review only

Z40-Z54	2	1935	0.0583	0.2137	0.0266	impact
Z40-Z54	2	1945	0.0583	0.2137	0.0266	incidence
Z40-Z54	2	1945	0.0583	0.2137	0.0266	impact
Z40-Z54	2	1955	0.0583	0.2137	0.0266	incidence
Z40-Z54	2	1955	0.0583	0.2137	0.0266	impact
I60-I69	1	1915	0.0534	0.2677	0.0509	incidence
I60-I69	1	1915	0.0534	0.2677	0.0509	impact
I60-I69	1	1925	0.0534	0.2677	0.0509	incidence
I60-I69	1	1925	0.0534	0.2677	0.0509	impact
I60-I69	1	1935	0.0534	0.2677	0.0509	incidence
I60-I69	1	1935	0.0534	0.2677	0.0509	impact
I60-I69	1	1945	0.0534	0.2677	0.0509	incidence
I60-I69	1	1945	0.0534	0.2677	0.0509	impact
I60-I69	1	1955	0.0534	0.2677	0.0509	incidence
I60-I69	1	1955	0.0534	0.2677	0.0509	impact
I60-I69	2	1915	0.0605	0.151	0.0368	incidence
I60-I69	2	1915	0.0605	0.151	0.0368	impact
I60-I69	2	1925	0.0605	0.151	0.0368	incidence
I60-I69	2	1925	0.0605	0.151	0.0368	impact
I60-I69	2	1935	0.0605	0.151	0.0368	incidence
I60-I69	2	1935	0.0605	0.151	0.0368	impact
I60-I69	2	1945	0.0605	0.151	0.0368	incidence
I60-I69	2	1945	0.0605	0.151	0.0368	impact
I60-I69	2	1955	0.0605	0.151	0.0368	incidence
I60-I69	2	1955	0.0605	0.151	0.0368	impact
K55-K63	1	1915	0.056	0.2964	0.0283	incidence
K55-K63	1	1915	0.056	0.2964	0.0283	impact
K55-K63	1	1925	0.056	0.2964	0.0283	incidence
K55-K63	1	1925	0.056	0.2964	0.0283	impact
K55-K63	1	1935	0.056	0.2964	0.0283	incidence
K55-K63	1	1935	0.056	0.2964	0.0283	impact
K55-K63	1	1945	0.056	0.2964	0.0283	incidence
K55-K63	1	1945	0.056	0.2964	0.0283	impact
K55-K63	1	1955	0.056	0.2964	0.0283	incidence
K55-K63	1	1955	0.056	0.2964	0.0283	impact
K55-K63	2	1915	0.0578	0.1624	0.0349	incidence
K55-K63	2	1915	0.0578	0.1624	0.0349	impact
K55-K63	2	1925	0.0578	0.1624	0.0349	incidence
K55-K63	2	1925	0.0578	0.1624	0.0349	impact
K55-K63	2	1935	0.0578	0.1624	0.0349	incidence
K55-K63	2	1935	0.0578	0.1624	0.0349	impact
K55-K63	2	1945	0.0578	0.1624	0.0349	incidence
K55-K63	2	1945	0.0578	0.1624	0.0349	impact

For peer review only

K55-K63	2	1955	0.0578	0.1624	0.0349	incidence
K55-K63	2	1955	0.0578	0.1624	0.0349	impact
J40-J47	1	1915	0.0502	0.2043	0.0751	incidence
J40-J47	1	1915	0.0502	0.2043	0.0751	impact
J40-J47	1	1925	0.0502	0.2043	0.0751	incidence
J40-J47	1	1925	0.0502	0.2043	0.0751	impact
J40-J47	1	1935	0.0502	0.2043	0.0751	incidence
J40-J47	1	1935	0.0502	0.2043	0.0751	impact
J40-J47	1	1945	0.0502	0.2043	0.0751	incidence
J40-J47	1	1945	0.0502	0.2043	0.0751	impact
J40-J47	1	1955	0.0502	0.2043	0.0751	incidence
J40-J47	1	1955	0.0502	0.2043	0.0751	impact
J40-J47	2	1915	0.0602	0.268	0.0353	incidence
J40-J47	2	1915	0.0602	0.268	0.0353	impact
J40-J47	2	1925	0.0602	0.268	0.0353	incidence
J40-J47	2	1925	0.0602	0.268	0.0353	impact
J40-J47	2	1935	0.0602	0.268	0.0353	incidence
J40-J47	2	1935	0.0602	0.268	0.0353	impact
J40-J47	2	1945	0.0602	0.268	0.0353	incidence
J40-J47	2	1945	0.0602	0.268	0.0353	impact
J40-J47	2	1955	0.0602	0.268	0.0353	incidence
J40-J47	2	1955	0.0602	0.268	0.0353	impact
I20-I25	1	1915	0.06	0.3489	0.034	incidence
I20-I25	1	1915	0.06	0.3489	0.034	impact
I20-I25	1	1925	0.06	0.3489	0.034	incidence
I20-I25	1	1925	0.06	0.3489	0.034	impact
I20-I25	1	1935	0.06	0.3489	0.034	incidence
I20-I25	1	1935	0.06	0.3489	0.034	impact
I20-I25	1	1945	0.06	0.3489	0.034	incidence
I20-I25	1	1945	0.06	0.3489	0.034	impact
I20-I25	1	1955	0.06	0.3489	0.034	incidence
I20-I25	1	1955	0.06	0.3489	0.034	impact
I20-I25	2	1915	0.0475	0.1668	0.049	incidence
I20-I25	2	1915	0.0475	0.1668	0.049	impact
I20-I25	2	1925	0.0475	0.1668	0.049	incidence
I20-I25	2	1925	0.0475	0.1668	0.049	impact
I20-I25	2	1935	0.0475	0.1668	0.049	incidence
I20-I25	2	1935	0.0475	0.1668	0.049	impact
I20-I25	2	1945	0.0475	0.1668	0.049	incidence
I20-I25	2	1945	0.0475	0.1668	0.049	impact
I20-I25	2	1955	0.0475	0.1668	0.049	incidence
I20-I25	2	1955	0.0475	0.1668	0.049	impact
N30-N39	1	1915	0.0469	-0.2899	0.0369	incidence

For peer review only

N30-N39	1	1915	0.0469	-0.2899	0.0369	impact
N30-N39	1	1925	0.0469	-0.2899	0.0369	incidence
N30-N39	1	1925	0.0469	-0.2899	0.0369	impact
N30-N39	1	1935	0.0469	-0.2899	0.0369	incidence
N30-N39	1	1935	0.0469	-0.2899	0.0369	impact
N30-N39	1	1945	0.0469	-0.2899	0.0369	incidence
N30-N39	1	1945	0.0469	-0.2899	0.0369	impact
N30-N39	1	1955	0.0469	-0.2899	0.0369	incidence
N30-N39	1	1955	0.0469	-0.2899	0.0369	impact
N30-N39	2	1915	0.06	-0.1589	0.0528	incidence
N30-N39	2	1915	0.06	-0.1589	0.0528	impact
N30-N39	2	1925	0.06	-0.1589	0.0528	incidence
N30-N39	2	1925	0.06	-0.1589	0.0528	impact
N30-N39	2	1935	0.06	-0.1589	0.0528	incidence
N30-N39	2	1935	0.06	-0.1589	0.0528	impact
N30-N39	2	1945	0.06	-0.1589	0.0528	incidence
N30-N39	2	1945	0.06	-0.1589	0.0528	impact
N30-N39	2	1955	0.06	-0.1589	0.0528	incidence
N30-N39	2	1955	0.06	-0.1589	0.0528	impact
K20-K31	1	1915	0.0595	0.1072	0.0529	incidence
K20-K31	1	1915	0.0595	0.1072	0.0529	impact
K20-K31	1	1925	0.0595	0.1072	0.0529	incidence
K20-K31	1	1925	0.0595	0.1072	0.0529	impact
K20-K31	1	1935	0.0595	0.1072	0.0529	incidence
K20-K31	1	1935	0.0595	0.1072	0.0529	impact
K20-K31	1	1945	0.0595	0.1072	0.0529	incidence
K20-K31	1	1945	0.0595	0.1072	0.0529	impact
K20-K31	1	1955	0.0595	0.1072	0.0529	incidence
K20-K31	1	1955	0.0595	0.1072	0.0529	impact
K20-K31	2	1915	0.0466	0.1561	0.074	incidence
K20-K31	2	1915	0.0466	0.1561	0.074	impact
K20-K31	2	1925	0.0466	0.1561	0.074	incidence
K20-K31	2	1925	0.0466	0.1561	0.074	impact
K20-K31	2	1935	0.0466	0.1561	0.074	incidence
K20-K31	2	1935	0.0466	0.1561	0.074	impact
K20-K31	2	1945	0.0466	0.1561	0.074	incidence
K20-K31	2	1945	0.0466	0.1561	0.074	impact
K20-K31	2	1955	0.0466	0.1561	0.074	incidence
K20-K31	2	1955	0.0466	0.1561	0.074	impact
J20-J22	1	1915	0.0529	-0.0947	0.0489	incidence
J20-J22	1	1915	0.0529	-0.0947	0.0489	impact
J20-J22	1	1925	0.0529	-0.0947	0.0489	incidence
J20-J22	1	1925	0.0529	-0.0947	0.0489	impact

For peer review only

J20-J22	1	1935	0.0529	-0.0947	0.0489 incidence
J20-J22	1	1935	0.0529	-0.0947	0.0489 impact
J20-J22	1	1945	0.0529	-0.0947	0.0489 incidence
J20-J22	1	1945	0.0529	-0.0947	0.0489 impact
J20-J22	1	1955	0.0529	-0.0947	0.0489 incidence
J20-J22	1	1955	0.0529	-0.0947	0.0489 impact
J20-J22	2	1915	0.0481	-0.1198	0.0272 incidence
J20-J22	2	1915	0.0481	-0.1198	0.0272 impact
J20-J22	2	1925	0.0481	-0.1198	0.0272 incidence
J20-J22	2	1925	0.0481	-0.1198	0.0272 impact
J20-J22	2	1935	0.0481	-0.1198	0.0272 incidence
J20-J22	2	1935	0.0481	-0.1198	0.0272 impact
J20-J22	2	1945	0.0481	-0.1198	0.0272 incidence
J20-J22	2	1945	0.0481	-0.1198	0.0272 impact
J20-J22	2	1955	0.0481	-0.1198	0.0272 incidence
J20-J22	2	1955	0.0481	-0.1198	0.0272 impact
S70-S79	1	1915	0.0278	0.0925	0.0622 incidence
S70-S79	1	1915	0.0278	0.0925	0.0622 impact
S70-S79	1	1925	0.0278	0.0925	0.0622 incidence
S70-S79	1	1925	0.0278	0.0925	0.0622 impact
S70-S79	1	1935	0.0278	0.0925	0.0622 incidence
S70-S79	1	1935	0.0278	0.0925	0.0622 impact
S70-S79	1	1945	0.0278	0.0925	0.0622 incidence
S70-S79	1	1945	0.0278	0.0925	0.0622 impact
S70-S79	1	1955	0.0278	0.0925	0.0622 incidence
S70-S79	1	1955	0.0278	0.0925	0.0622 impact
S70-S79	2	1915	0.0474	-0.0664	0.0213 incidence
S70-S79	2	1915	0.0474	-0.0664	0.0213 impact
S70-S79	2	1925	0.0474	-0.0664	0.0213 incidence
S70-S79	2	1925	0.0474	-0.0664	0.0213 impact
S70-S79	2	1935	0.0474	-0.0664	0.0213 incidence
S70-S79	2	1935	0.0474	-0.0664	0.0213 impact
S70-S79	2	1945	0.0474	-0.0664	0.0213 incidence
S70-S79	2	1945	0.0474	-0.0664	0.0213 impact
S70-S79	2	1955	0.0474	-0.0664	0.0213 incidence
S70-S79	2	1955	0.0474	-0.0664	0.0213 impact
A30-A49	1	1915	0.0318	0.02	0.1402 incidence
A30-A49	1	1915	0.0318	0.02	0.1402 impact
A30-A49	1	1925	0.0318	0.02	0.1402 incidence
A30-A49	1	1925	0.0318	0.02	0.1402 impact
A30-A49	1	1935	0.0318	0.02	0.1402 incidence
A30-A49	1	1935	0.0318	0.02	0.1402 impact
A30-A49	1	1945	0.0318	0.02	0.1402 incidence

For peer review only

A30-A49	1	1945	0.0318	0.02	0.1402	impact
A30-A49	1	1955	0.0318	0.02	0.1402	incidence
A30-A49	1	1955	0.0318	0.02	0.1402	impact
A30-A49	2	1915	0.033	0.1166	0.097	incidence
A30-A49	2	1915	0.033	0.1166	0.097	impact
A30-A49	2	1925	0.033	0.1166	0.097	incidence
A30-A49	2	1925	0.033	0.1166	0.097	impact
A30-A49	2	1935	0.033	0.1166	0.097	incidence
A30-A49	2	1935	0.033	0.1166	0.097	impact
A30-A49	2	1945	0.033	0.1166	0.097	incidence
A30-A49	2	1945	0.033	0.1166	0.097	impact
A30-A49	2	1955	0.033	0.1166	0.097	incidence
A30-A49	2	1955	0.033	0.1166	0.097	impact
T80-T88	1	1915	0.0365	0.1569	0.0961	incidence
T80-T88	1	1915	0.0365	0.1569	0.0961	impact
T80-T88	1	1925	0.0365	0.1569	0.0961	incidence
T80-T88	1	1925	0.0365	0.1569	0.0961	impact
T80-T88	1	1935	0.0365	0.1569	0.0961	incidence
T80-T88	1	1935	0.0365	0.1569	0.0961	impact
T80-T88	1	1945	0.0365	0.1569	0.0961	incidence
T80-T88	1	1945	0.0365	0.1569	0.0961	impact
T80-T88	1	1955	0.0365	0.1569	0.0961	incidence
T80-T88	1	1955	0.0365	0.1569	0.0961	impact
T80-T88	2	1915	0.0286	0.2264	0.053	incidence
T80-T88	2	1915	0.0286	0.2264	0.053	impact
T80-T88	2	1925	0.0286	0.2264	0.053	incidence
T80-T88	2	1925	0.0286	0.2264	0.053	impact
T80-T88	2	1935	0.0286	0.2264	0.053	incidence
T80-T88	2	1935	0.0286	0.2264	0.053	impact
T80-T88	2	1945	0.0286	0.2264	0.053	incidence
T80-T88	2	1945	0.0286	0.2264	0.053	impact
T80-T88	2	1955	0.0286	0.2264	0.053	incidence
T80-T88	2	1955	0.0286	0.2264	0.053	impact
N17-N19	1	1915	0.0338	0.1446	0.0628	incidence
N17-N19	1	1915	0.0338	0.1446	0.0628	impact
N17-N19	1	1925	0.0338	0.1446	0.0628	incidence
N17-N19	1	1925	0.0338	0.1446	0.0628	impact
N17-N19	1	1935	0.0338	0.1446	0.0628	incidence
N17-N19	1	1935	0.0338	0.1446	0.0628	impact
N17-N19	1	1945	0.0338	0.1446	0.0628	incidence
N17-N19	1	1945	0.0338	0.1446	0.0628	impact
N17-N19	1	1955	0.0338	0.1446	0.0628	incidence
N17-N19	1	1955	0.0338	0.1446	0.0628	impact

For peer review only

N17-N19	2	1915	0.0287	0.2572	0.0793 incidence
N17-N19	2	1915	0.0287	0.2572	0.0793 impact
N17-N19	2	1925	0.0287	0.2572	0.0793 incidence
N17-N19	2	1925	0.0287	0.2572	0.0793 impact
N17-N19	2	1935	0.0287	0.2572	0.0793 incidence
N17-N19	2	1935	0.0287	0.2572	0.0793 impact
N17-N19	2	1945	0.0287	0.2572	0.0793 incidence
N17-N19	2	1945	0.0287	0.2572	0.0793 impact
N17-N19	2	1955	0.0287	0.2572	0.0793 incidence
N17-N19	2	1955	0.0287	0.2572	0.0793 impact
I80-I89	1	1915	0.0281	0.3714	0.0539 incidence
I80-I89	1	1915	0.0281	0.3714	0.0539 impact
I80-I89	1	1925	0.0281	0.3714	0.0539 incidence
I80-I89	1	1925	0.0281	0.3714	0.0539 impact
I80-I89	1	1935	0.0281	0.3714	0.0539 incidence
I80-I89	1	1935	0.0281	0.3714	0.0539 impact
I80-I89	1	1945	0.0281	0.3714	0.0539 incidence
I80-I89	1	1945	0.0281	0.3714	0.0539 impact
I80-I89	1	1955	0.0281	0.3714	0.0539 incidence
I80-I89	1	1955	0.0281	0.3714	0.0539 impact
I80-I89	2	1915	0.0285	0.0909	0.0634 incidence
I80-I89	2	1915	0.0285	0.0909	0.0634 impact
I80-I89	2	1925	0.0285	0.0909	0.0634 incidence
I80-I89	2	1925	0.0285	0.0909	0.0634 impact
I80-I89	2	1935	0.0285	0.0909	0.0634 incidence
I80-I89	2	1935	0.0285	0.0909	0.0634 impact
I80-I89	2	1945	0.0285	0.0909	0.0634 incidence
I80-I89	2	1945	0.0285	0.0909	0.0634 impact
I80-I89	2	1955	0.0285	0.0909	0.0634 incidence
I80-I89	2	1955	0.0285	0.0909	0.0634 impact
K90-K93	1	1915	0.0302	0.2217	0.0834 incidence
K90-K93	1	1915	0.0302	0.2217	0.0834 impact
K90-K93	1	1925	0.0302	0.2217	0.0834 incidence
K90-K93	1	1925	0.0302	0.2217	0.0834 impact
K90-K93	1	1935	0.0302	0.2217	0.0834 incidence
K90-K93	1	1935	0.0302	0.2217	0.0834 impact
K90-K93	1	1945	0.0302	0.2217	0.0834 incidence
K90-K93	1	1945	0.0302	0.2217	0.0834 impact
K90-K93	1	1955	0.0302	0.2217	0.0834 incidence
K90-K93	1	1955	0.0302	0.2217	0.0834 impact
K90-K93	2	1915	0.0226	0.1189	0.0755 incidence
K90-K93	2	1915	0.0226	0.1189	0.0755 impact
K90-K93	2	1925	0.0226	0.1189	0.0755 incidence

For peer review only

K90-K93	2	1925	0.0226	0.1189	0.0755	impact
K90-K93	2	1935	0.0226	0.1189	0.0755	incidence
K90-K93	2	1935	0.0226	0.1189	0.0755	impact
K90-K93	2	1945	0.0226	0.1189	0.0755	incidence
K90-K93	2	1945	0.0226	0.1189	0.0755	impact
K90-K93	2	1955	0.0226	0.1189	0.0755	incidence
K90-K93	2	1955	0.0226	0.1189	0.0755	impact
I70-I79	1	1915	0.03	0.1357	0.0437	incidence
I70-I79	1	1915	0.03	0.1357	0.0437	impact
I70-I79	1	1925	0.03	0.1357	0.0437	incidence
I70-I79	1	1925	0.03	0.1357	0.0437	impact
I70-I79	1	1935	0.03	0.1357	0.0437	incidence
I70-I79	1	1935	0.03	0.1357	0.0437	impact
I70-I79	1	1945	0.03	0.1357	0.0437	incidence
I70-I79	1	1945	0.03	0.1357	0.0437	impact
I70-I79	1	1955	0.03	0.1357	0.0437	incidence
I70-I79	1	1955	0.03	0.1357	0.0437	impact
I70-I79	2	1915	0.0226	0.0218	0.0605	incidence
I70-I79	2	1915	0.0226	0.0218	0.0605	impact
I70-I79	2	1925	0.0226	0.0218	0.0605	incidence
I70-I79	2	1925	0.0226	0.0218	0.0605	impact
I70-I79	2	1935	0.0226	0.0218	0.0605	incidence
I70-I79	2	1935	0.0226	0.0218	0.0605	impact
I70-I79	2	1945	0.0226	0.0218	0.0605	incidence
I70-I79	2	1945	0.0226	0.0218	0.0605	impact
I70-I79	2	1955	0.0226	0.0218	0.0605	incidence
I70-I79	2	1955	0.0226	0.0218	0.0605	impact
K50-K52	1	1915	0.0216	0.1408	0.0282	incidence
K50-K52	1	1915	0.0216	0.1408	0.0282	impact
K50-K52	1	1925	0.0216	0.1408	0.0282	incidence
K50-K52	1	1925	0.0216	0.1408	0.0282	impact
K50-K52	1	1935	0.0216	0.1408	0.0282	incidence
K50-K52	1	1935	0.0216	0.1408	0.0282	impact
K50-K52	1	1945	0.0216	0.1408	0.0282	incidence
K50-K52	1	1945	0.0216	0.1408	0.0282	impact
K50-K52	1	1955	0.0216	0.1408	0.0282	incidence
K50-K52	1	1955	0.0216	0.1408	0.0282	impact
K50-K52	2	1915	0.0273	0.092	0.0373	incidence
K50-K52	2	1915	0.0273	0.092	0.0373	impact
K50-K52	2	1925	0.0273	0.092	0.0373	incidence
K50-K52	2	1925	0.0273	0.092	0.0373	impact
K50-K52	2	1935	0.0273	0.092	0.0373	incidence
K50-K52	2	1935	0.0273	0.092	0.0373	impact

For peer review only

K50-K52	2	1945	0.0273	0.092	0.0373	incidence
K50-K52	2	1945	0.0273	0.092	0.0373	impact
K50-K52	2	1955	0.0273	0.092	0.0373	incidence
K50-K52	2	1955	0.0273	0.092	0.0373	impact
S00-S09	1	1915	0.0332	0.1778	0.0483	incidence
S00-S09	1	1915	0.0332	0.1778	0.0483	impact
S00-S09	1	1925	0.0332	0.1778	0.0483	incidence
S00-S09	1	1925	0.0332	0.1778	0.0483	impact
S00-S09	1	1935	0.0332	0.1778	0.0483	incidence
S00-S09	1	1935	0.0332	0.1778	0.0483	impact
S00-S09	1	1945	0.0332	0.1778	0.0483	incidence
S00-S09	1	1945	0.0332	0.1778	0.0483	impact
S00-S09	1	1955	0.0332	0.1778	0.0483	incidence
S00-S09	1	1955	0.0332	0.1778	0.0483	impact
S00-S09	2	1915	0.0171	-0.1563	0.0589	incidence
S00-S09	2	1915	0.0171	-0.1563	0.0589	impact
S00-S09	2	1925	0.0171	-0.1563	0.0589	incidence
S00-S09	2	1925	0.0171	-0.1563	0.0589	impact
S00-S09	2	1935	0.0171	-0.1563	0.0589	incidence
S00-S09	2	1935	0.0171	-0.1563	0.0589	impact
S00-S09	2	1945	0.0171	-0.1563	0.0589	incidence
S00-S09	2	1945	0.0171	-0.1563	0.0589	impact
S00-S09	2	1955	0.0171	-0.1563	0.0589	incidence
S00-S09	2	1955	0.0171	-0.1563	0.0589	impact
C50-C50	1	1915	0.000565	1.1527	0.6432	incidence
C50-C50	1	1915	0.000565	1.1527	0.6432	impact
C50-C50	1	1925	0.000565	1.1527	0.6432	incidence
C50-C50	1	1925	0.000565	1.1527	0.6432	impact
C50-C50	1	1935	0.000565	1.1527	0.6432	incidence
C50-C50	1	1935	0.000565	1.1527	0.6432	impact
C50-C50	1	1945	0.000565	1.1527	0.6432	incidence
C50-C50	1	1945	0.000565	1.1527	0.6432	impact
C50-C50	1	1955	0.000565	1.1527	0.6432	incidence
C50-C50	1	1955	0.000565	1.1527	0.6432	impact
C50-C50	2	1915	0.0434	1.1487	0.0744	incidence
C50-C50	2	1915	0.0434	1.1487	0.0744	impact
C50-C50	2	1925	0.0434	1.1487	0.0744	incidence
C50-C50	2	1925	0.0434	1.1487	0.0744	impact
C50-C50	2	1935	0.0434	1.1487	0.0744	incidence
C50-C50	2	1935	0.0434	1.1487	0.0744	impact
C50-C50	2	1945	0.0434	1.1487	0.0744	incidence
C50-C50	2	1945	0.0434	1.1487	0.0744	impact
C50-C50	2	1955	0.0434	1.1487	0.0744	incidence

For peer review only

C50-C50	2	1955	0.0434	1.1487	0.0744	impact
C60-C63	1	1915	0.0287	0.6987	0.0671	incidence
C60-C63	1	1915	0.0287	0.6987	0.0671	impact
C60-C63	1	1925	0.0287	0.6987	0.0671	incidence
C60-C63	1	1925	0.0287	0.6987	0.0671	impact
C60-C63	1	1935	0.0287	0.6987	0.0671	incidence
C60-C63	1	1935	0.0287	0.6987	0.0671	impact
C60-C63	1	1945	0.0287	0.6987	0.0671	incidence
C60-C63	1	1945	0.0287	0.6987	0.0671	impact
C60-C63	1	1955	0.0287	0.6987	0.0671	incidence
C60-C63	1	1955	0.0287	0.6987	0.0671	impact
C60-C63	2	1915	-3.57E-06	NA	NA	incidence
C60-C63	2	1915	-3.57E-06	NA	NA	impact
C60-C63	2	1925	-3.57E-06	NA	NA	incidence
C60-C63	2	1925	-3.57E-06	NA	NA	impact
C60-C63	2	1935	-3.57E-06	NA	NA	incidence
C60-C63	2	1935	-3.57E-06	NA	NA	impact
C60-C63	2	1945	-3.57E-06	NA	NA	incidence
C60-C63	2	1945	-3.57E-06	NA	NA	impact
C60-C63	2	1955	-3.57E-06	NA	NA	incidence
C60-C63	2	1955	-3.57E-06	NA	NA	impact
G30-G32	1	1915	0.00307	-0.0377	0.2699	incidence
G30-G32	1	1915	0.00307	-0.0377	0.2699	impact
G30-G32	1	1925	0.00307	-0.0377	0.2699	incidence
G30-G32	1	1925	0.00307	-0.0377	0.2699	impact
G30-G32	1	1935	0.00307	-0.0377	0.2699	incidence
G30-G32	1	1935	0.00307	-0.0377	0.2699	impact
G30-G32	1	1945	0.00307	-0.0377	0.2699	incidence
G30-G32	1	1945	0.00307	-0.0377	0.2699	impact
G30-G32	1	1955	0.00307	-0.0377	0.2699	incidence
G30-G32	1	1955	0.00307	-0.0377	0.2699	impact
G30-G32	2	1915	0.00265	0.064	0.1112	incidence
G30-G32	2	1915	0.00265	0.064	0.1112	impact
G30-G32	2	1925	0.00265	0.064	0.1112	incidence
G30-G32	2	1925	0.00265	0.064	0.1112	impact
G30-G32	2	1935	0.00265	0.064	0.1112	incidence
G30-G32	2	1935	0.00265	0.064	0.1112	impact
G30-G32	2	1945	0.00265	0.064	0.1112	incidence
G30-G32	2	1945	0.00265	0.064	0.1112	impact
G30-G32	2	1955	0.00265	0.064	0.1112	incidence
G30-G32	2	1955	0.00265	0.064	0.1112	impact
ACT-DTH	1	1915	NA	NA	NA	incidence
ACT-DTH	1	1915	NA	NA	NA	impact

For peer review only

ACT-DTH	1	1925	NA	NA	NA	incidence
ACT-DTH	1	1925	NA	NA	NA	impact
ACT-DTH	1	1935	NA	NA	NA	incidence
ACT-DTH	1	1935	NA	NA	NA	impact
ACT-DTH	1	1945	NA	NA	NA	incidence
ACT-DTH	1	1945	NA	NA	NA	impact
ACT-DTH	1	1955	NA	NA	NA	incidence
ACT-DTH	1	1955	NA	NA	NA	impact
ACT-DTH	2	1915	NA	NA	NA	incidence
ACT-DTH	2	1915	NA	NA	NA	impact
ACT-DTH	2	1925	NA	NA	NA	incidence
ACT-DTH	2	1925	NA	NA	NA	impact
ACT-DTH	2	1935	NA	NA	NA	incidence
ACT-DTH	2	1935	NA	NA	NA	impact
ACT-DTH	2	1945	NA	NA	NA	incidence
ACT-DTH	2	1945	NA	NA	NA	impact
ACT-DTH	2	1955	NA	NA	NA	incidence
ACT-DTH	2	1955	NA	NA	NA	impact
ACT-DTH	3	1915	NA	NA	NA	incidence
ACT-DTH	3	1915	NA	NA	NA	impact
ACT-DTH	3	1925	NA	NA	NA	incidence
ACT-DTH	3	1925	NA	NA	NA	impact
ACT-DTH	3	1935	NA	NA	NA	incidence
ACT-DTH	3	1935	NA	NA	NA	impact
ACT-DTH	3	1945	NA	NA	NA	incidence
ACT-DTH	3	1945	NA	NA	NA	impact
ACT-DTH	3	1955	NA	NA	NA	incidence
ACT-DTH	3	1955	NA	NA	NA	impact
ACT-DTH	4	1915	NA	NA	NA	incidence
ACT-DTH	4	1915	NA	NA	NA	impact
ACT-DTH	4	1925	NA	NA	NA	incidence
ACT-DTH	4	1925	NA	NA	NA	impact
ACT-DTH	4	1935	NA	NA	NA	incidence
ACT-DTH	4	1935	NA	NA	NA	impact
ACT-DTH	4	1945	NA	NA	NA	incidence
ACT-DTH	4	1945	NA	NA	NA	impact
ACT-DTH	4	1955	NA	NA	NA	incidence
ACT-DTH	4	1955	NA	NA	NA	impact
ACT-DTH	5	1915	NA	NA	NA	incidence
ACT-DTH	5	1915	NA	NA	NA	impact
ACT-DTH	5	1925	NA	NA	NA	incidence
ACT-DTH	5	1925	NA	NA	NA	impact
ACT-DTH	5	1935	NA	NA	NA	incidence

For peer review only

ACT-DTH	5	1935	NA	NA	NA	impact
ACT-DTH	5	1945	NA	NA	NA	incidence
ACT-DTH	5	1945	NA	NA	NA	impact
ACT-DTH	5	1955	NA	NA	NA	incidence
ACT-DTH	5	1955	NA	NA	NA	impact
ACT-DTH	6	1915	NA	NA	NA	incidence
ACT-DTH	6	1915	NA	NA	NA	impact
ACT-DTH	6	1925	NA	NA	NA	incidence
ACT-DTH	6	1925	NA	NA	NA	impact
ACT-DTH	6	1935	NA	NA	NA	incidence
ACT-DTH	6	1935	NA	NA	NA	impact
ACT-DTH	6	1945	NA	NA	NA	incidence
ACT-DTH	6	1945	NA	NA	NA	impact
ACT-DTH	6	1955	NA	NA	NA	incidence
ACT-DTH	6	1955	NA	NA	NA	impact
ACT-DTH	7	1915	NA	NA	NA	incidence
ACT-DTH	7	1915	NA	NA	NA	impact
ACT-DTH	7	1925	NA	NA	NA	incidence
ACT-DTH	7	1925	NA	NA	NA	impact
ACT-DTH	7	1935	NA	NA	NA	incidence
ACT-DTH	7	1935	NA	NA	NA	impact
ACT-DTH	7	1945	NA	NA	NA	incidence
ACT-DTH	7	1945	NA	NA	NA	impact
ACT-DTH	7	1955	NA	NA	NA	incidence
ACT-DTH	7	1955	NA	NA	NA	impact
ACT-DTH	8	1915	NA	NA	NA	incidence
ACT-DTH	8	1915	NA	NA	NA	impact
ACT-DTH	8	1925	NA	NA	NA	incidence
ACT-DTH	8	1925	NA	NA	NA	impact
ACT-DTH	8	1935	NA	NA	NA	incidence
ACT-DTH	8	1935	NA	NA	NA	impact
ACT-DTH	8	1945	NA	NA	NA	incidence
ACT-DTH	8	1945	NA	NA	NA	impact
ACT-DTH	8	1955	NA	NA	NA	incidence
ACT-DTH	8	1955	NA	NA	NA	impact
ACT-DTH	9	1915	NA	NA	NA	incidence
ACT-DTH	9	1915	NA	NA	NA	impact
ACT-DTH	9	1925	NA	NA	NA	incidence
ACT-DTH	9	1925	NA	NA	NA	impact
ACT-DTH	9	1935	NA	NA	NA	incidence
ACT-DTH	9	1935	NA	NA	NA	impact
ACT-DTH	9	1945	NA	NA	NA	incidence
ACT-DTH	9	1945	NA	NA	NA	impact

For peer review only

ACT-DTH	9	1955	NA	NA	NA	incidence
ACT-DTH	9	1955	NA	NA	NA	impact
ACT-DTH	10	1915	NA	NA	NA	incidence
ACT-DTH	10	1915	NA	NA	NA	impact
ACT-DTH	10	1925	NA	NA	NA	incidence
ACT-DTH	10	1925	NA	NA	NA	impact
ACT-DTH	10	1935	NA	NA	NA	incidence
ACT-DTH	10	1935	NA	NA	NA	impact
ACT-DTH	10	1945	NA	NA	NA	incidence
ACT-DTH	10	1945	NA	NA	NA	impact
ACT-DTH	10	1955	NA	NA	NA	incidence
ACT-DTH	10	1955	NA	NA	NA	impact
EST-DTH	1	1915	1.09419	0.22037548	0.15620343	incidence
EST-DTH	1	1915	1.09419	0.22037548	0.15620343	impact
EST-DTH	1	1925	1.09419	0.22037548	0.15620343	incidence
EST-DTH	1	1925	1.09419	0.22037548	0.15620343	impact
EST-DTH	1	1935	1.09419	0.22037548	0.15620343	incidence
EST-DTH	1	1935	1.09419	0.22037548	0.15620343	impact
EST-DTH	1	1945	1.09419	0.22037548	0.15620343	incidence
EST-DTH	1	1945	1.09419	0.22037548	0.15620343	impact
EST-DTH	1	1955	1.09419	0.22037548	0.15620343	incidence
EST-DTH	1	1955	1.09419	0.22037548	0.15620343	impact
EST-DTH	2	1915	1.16032	0.15917636	0.13497034	incidence
EST-DTH	2	1915	1.16032	0.15917636	0.13497034	impact
EST-DTH	2	1925	1.16032	0.15917636	0.13497034	incidence
EST-DTH	2	1925	1.16032	0.15917636	0.13497034	impact
EST-DTH	2	1935	1.16032	0.15917636	0.13497034	incidence
EST-DTH	2	1935	1.16032	0.15917636	0.13497034	impact
EST-DTH	2	1945	1.16032	0.15917636	0.13497034	incidence
EST-DTH	2	1945	1.16032	0.15917636	0.13497034	impact
EST-DTH	2	1955	1.16032	0.15917636	0.13497034	incidence
EST-DTH	2	1955	1.16032	0.15917636	0.13497034	impact
EST-DTH	3	1915	1.17692	0.11862247	0.14295061	incidence
EST-DTH	3	1915	1.17692	0.11862247	0.14295061	impact
EST-DTH	3	1925	1.17692	0.11862247	0.14295061	incidence
EST-DTH	3	1925	1.17692	0.11862247	0.14295061	impact
EST-DTH	3	1935	1.17692	0.11862247	0.14295061	incidence
EST-DTH	3	1935	1.17692	0.11862247	0.14295061	impact
EST-DTH	3	1945	1.17692	0.11862247	0.14295061	incidence
EST-DTH	3	1945	1.17692	0.11862247	0.14295061	impact
EST-DTH	3	1955	1.17692	0.11862247	0.14295061	incidence
EST-DTH	3	1955	1.17692	0.11862247	0.14295061	impact
EST-DTH	4	1915	1.23779	0.17586043	0.11924127	incidence

For peer review only

EST-DTH	4	1915	1.23779	0.17586043	0.11924127	impact
EST-DTH	4	1925	1.23779	0.17586043	0.11924127	incidence
EST-DTH	4	1925	1.23779	0.17586043	0.11924127	impact
EST-DTH	4	1935	1.23779	0.17586043	0.11924127	incidence
EST-DTH	4	1935	1.23779	0.17586043	0.11924127	impact
EST-DTH	4	1945	1.23779	0.17586043	0.11924127	incidence
EST-DTH	4	1945	1.23779	0.17586043	0.11924127	impact
EST-DTH	4	1955	1.23779	0.17586043	0.11924127	incidence
EST-DTH	4	1955	1.23779	0.17586043	0.11924127	impact
EST-DTH	5	1915	1.31797	0.17306388	0.11751678	incidence
EST-DTH	5	1915	1.31797	0.17306388	0.11751678	impact
EST-DTH	5	1925	1.31797	0.17306388	0.11751678	incidence
EST-DTH	5	1925	1.31797	0.17306388	0.11751678	impact
EST-DTH	5	1935	1.31797	0.17306388	0.11751678	incidence
EST-DTH	5	1935	1.31797	0.17306388	0.11751678	impact
EST-DTH	5	1945	1.31797	0.17306388	0.11751678	incidence
EST-DTH	5	1945	1.31797	0.17306388	0.11751678	impact
EST-DTH	5	1955	1.31797	0.17306388	0.11751678	incidence
EST-DTH	5	1955	1.31797	0.17306388	0.11751678	impact
EST-DTH	6	1915	1.41303	0.19278868	0.1171426	incidence
EST-DTH	6	1915	1.41303	0.19278868	0.1171426	impact
EST-DTH	6	1925	1.41303	0.19278868	0.1171426	incidence
EST-DTH	6	1925	1.41303	0.19278868	0.1171426	impact
EST-DTH	6	1935	1.41303	0.19278868	0.1171426	incidence
EST-DTH	6	1935	1.41303	0.19278868	0.1171426	impact
EST-DTH	6	1945	1.41303	0.19278868	0.1171426	incidence
EST-DTH	6	1945	1.41303	0.19278868	0.1171426	impact
EST-DTH	6	1955	1.41303	0.19278868	0.1171426	incidence
EST-DTH	6	1955	1.41303	0.19278868	0.1171426	impact
EST-DTH	7	1915	1.49752	0.17870892	0.10998368	incidence
EST-DTH	7	1915	1.49752	0.17870892	0.10998368	impact
EST-DTH	7	1925	1.49752	0.17870892	0.10998368	incidence
EST-DTH	7	1925	1.49752	0.17870892	0.10998368	impact
EST-DTH	7	1935	1.49752	0.17870892	0.10998368	incidence
EST-DTH	7	1935	1.49752	0.17870892	0.10998368	impact
EST-DTH	7	1945	1.49752	0.17870892	0.10998368	incidence
EST-DTH	7	1945	1.49752	0.17870892	0.10998368	impact
EST-DTH	7	1955	1.49752	0.17870892	0.10998368	incidence
EST-DTH	7	1955	1.49752	0.17870892	0.10998368	impact
EST-DTH	8	1915	1.54785	0.20973753	0.09527479	incidence
EST-DTH	8	1915	1.54785	0.20973753	0.09527479	impact
EST-DTH	8	1925	1.54785	0.20973753	0.09527479	incidence
EST-DTH	8	1925	1.54785	0.20973753	0.09527479	impact

For peer review only

EST-DTH	8	1935	1.54785	0.20973753	0.09527479	incidence
EST-DTH	8	1935	1.54785	0.20973753	0.09527479	impact
EST-DTH	8	1945	1.54785	0.20973753	0.09527479	incidence
EST-DTH	8	1945	1.54785	0.20973753	0.09527479	impact
EST-DTH	8	1955	1.54785	0.20973753	0.09527479	incidence
EST-DTH	8	1955	1.54785	0.20973753	0.09527479	impact
EST-DTH	9	1915	1.61129	0.16192717	0.10840535	incidence
EST-DTH	9	1915	1.61129	0.16192717	0.10840535	impact
EST-DTH	9	1925	1.61129	0.16192717	0.10840535	incidence
EST-DTH	9	1925	1.61129	0.16192717	0.10840535	impact
EST-DTH	9	1935	1.61129	0.16192717	0.10840535	incidence
EST-DTH	9	1935	1.61129	0.16192717	0.10840535	impact
EST-DTH	9	1945	1.61129	0.16192717	0.10840535	incidence
EST-DTH	9	1945	1.61129	0.16192717	0.10840535	impact
EST-DTH	9	1955	1.61129	0.16192717	0.10840535	incidence
EST-DTH	9	1955	1.61129	0.16192717	0.10840535	impact
EST-DTH	10	1915	1.71953	0.19697031	0.10725023	incidence
EST-DTH	10	1915	1.71953	0.19697031	0.10725023	impact
EST-DTH	10	1925	1.71953	0.19697031	0.10725023	incidence
EST-DTH	10	1925	1.71953	0.19697031	0.10725023	impact
EST-DTH	10	1935	1.71953	0.19697031	0.10725023	incidence
EST-DTH	10	1935	1.71953	0.19697031	0.10725023	impact
EST-DTH	10	1945	1.71953	0.19697031	0.10725023	incidence
EST-DTH	10	1945	1.71953	0.19697031	0.10725023	impact
EST-DTH	10	1955	1.71953	0.19697031	0.10725023	incidence
EST-DTH	10	1955	1.71953	0.19697031	0.10725023	impact
J09-J18	1	1915	0.06736	-0.0811	0.2164	incidence
J09-J18	1	1915	0.06736	-0.0811	0.2164	impact
J09-J18	1	1925	0.06736	-0.0811	0.2164	incidence
J09-J18	1	1925	0.06736	-0.0811	0.2164	impact
J09-J18	1	1935	0.06736	-0.0811	0.2164	incidence
J09-J18	1	1935	0.06736	-0.0811	0.2164	impact
J09-J18	1	1945	0.06736	-0.0811	0.2164	incidence
J09-J18	1	1945	0.06736	-0.0811	0.2164	impact
J09-J18	1	1955	0.06736	-0.0811	0.2164	incidence
J09-J18	1	1955	0.06736	-0.0811	0.2164	impact
J09-J18	2	1915	0.06656	0.186	0.1322	incidence
J09-J18	2	1915	0.06656	0.186	0.1322	impact
J09-J18	2	1925	0.06656	0.186	0.1322	incidence
J09-J18	2	1925	0.06656	0.186	0.1322	impact
J09-J18	2	1935	0.06656	0.186	0.1322	incidence
J09-J18	2	1935	0.06656	0.186	0.1322	impact
J09-J18	2	1945	0.06656	0.186	0.1322	incidence

For peer review only

J09-J18	2	1945	0.06656	0.186	0.1322	impact
J09-J18	2	1955	0.06656	0.186	0.1322	incidence
J09-J18	2	1955	0.06656	0.186	0.1322	impact
J09-J18	3	1915	0.07515	0.0534	0.2479	incidence
J09-J18	3	1915	0.07515	0.0534	0.2479	impact
J09-J18	3	1925	0.07515	0.0534	0.2479	incidence
J09-J18	3	1925	0.07515	0.0534	0.2479	impact
J09-J18	3	1935	0.07515	0.0534	0.2479	incidence
J09-J18	3	1935	0.07515	0.0534	0.2479	impact
J09-J18	3	1945	0.07515	0.0534	0.2479	incidence
J09-J18	3	1945	0.07515	0.0534	0.2479	impact
J09-J18	3	1955	0.07515	0.0534	0.2479	incidence
J09-J18	3	1955	0.07515	0.0534	0.2479	impact
J09-J18	4	1915	0.07726	-0.0173	0.1405	incidence
J09-J18	4	1915	0.07726	-0.0173	0.1405	impact
J09-J18	4	1925	0.07726	-0.0173	0.1405	incidence
J09-J18	4	1925	0.07726	-0.0173	0.1405	impact
J09-J18	4	1935	0.07726	-0.0173	0.1405	incidence
J09-J18	4	1935	0.07726	-0.0173	0.1405	impact
J09-J18	4	1945	0.07726	-0.0173	0.1405	incidence
J09-J18	4	1945	0.07726	-0.0173	0.1405	impact
J09-J18	4	1955	0.07726	-0.0173	0.1405	incidence
J09-J18	4	1955	0.07726	-0.0173	0.1405	impact
J09-J18	5	1915	0.0863	0.196	0.1213	incidence
J09-J18	5	1915	0.0863	0.196	0.1213	impact
J09-J18	5	1925	0.0863	0.196	0.1213	incidence
J09-J18	5	1925	0.0863	0.196	0.1213	impact
J09-J18	5	1935	0.0863	0.196	0.1213	incidence
J09-J18	5	1935	0.0863	0.196	0.1213	impact
J09-J18	5	1945	0.0863	0.196	0.1213	incidence
J09-J18	5	1945	0.0863	0.196	0.1213	impact
J09-J18	5	1955	0.0863	0.196	0.1213	incidence
J09-J18	5	1955	0.0863	0.196	0.1213	impact
J09-J18	6	1915	0.09347	0.321	0.1602	incidence
J09-J18	6	1915	0.09347	0.321	0.1602	impact
J09-J18	6	1925	0.09347	0.321	0.1602	incidence
J09-J18	6	1925	0.09347	0.321	0.1602	impact
J09-J18	6	1935	0.09347	0.321	0.1602	incidence
J09-J18	6	1935	0.09347	0.321	0.1602	impact
J09-J18	6	1945	0.09347	0.321	0.1602	incidence
J09-J18	6	1945	0.09347	0.321	0.1602	impact
J09-J18	6	1955	0.09347	0.321	0.1602	incidence
J09-J18	6	1955	0.09347	0.321	0.1602	impact

For peer review only

J09-J18	7	1915	0.10779	0.191	0.0632 incidence
J09-J18	7	1915	0.10779	0.191	0.0632 impact
J09-J18	7	1925	0.10779	0.191	0.0632 incidence
J09-J18	7	1925	0.10779	0.191	0.0632 impact
J09-J18	7	1935	0.10779	0.191	0.0632 incidence
J09-J18	7	1935	0.10779	0.191	0.0632 impact
J09-J18	7	1945	0.10779	0.191	0.0632 incidence
J09-J18	7	1945	0.10779	0.191	0.0632 impact
J09-J18	7	1955	0.10779	0.191	0.0632 incidence
J09-J18	7	1955	0.10779	0.191	0.0632 impact
J09-J18	8	1915	0.11486	0.19	0.0861 incidence
J09-J18	8	1915	0.11486	0.19	0.0861 impact
J09-J18	8	1925	0.11486	0.19	0.0861 incidence
J09-J18	8	1925	0.11486	0.19	0.0861 impact
J09-J18	8	1935	0.11486	0.19	0.0861 incidence
J09-J18	8	1935	0.11486	0.19	0.0861 impact
J09-J18	8	1945	0.11486	0.19	0.0861 incidence
J09-J18	8	1945	0.11486	0.19	0.0861 impact
J09-J18	8	1955	0.11486	0.19	0.0861 incidence
J09-J18	8	1955	0.11486	0.19	0.0861 impact
J09-J18	9	1915	0.11924	0.106	0.1007 incidence
J09-J18	9	1915	0.11924	0.106	0.1007 impact
J09-J18	9	1925	0.11924	0.106	0.1007 incidence
J09-J18	9	1925	0.11924	0.106	0.1007 impact
J09-J18	9	1935	0.11924	0.106	0.1007 incidence
J09-J18	9	1935	0.11924	0.106	0.1007 impact
J09-J18	9	1945	0.11924	0.106	0.1007 incidence
J09-J18	9	1945	0.11924	0.106	0.1007 impact
J09-J18	9	1955	0.11924	0.106	0.1007 incidence
J09-J18	9	1955	0.11924	0.106	0.1007 impact
J09-J18	10	1915	0.12938	0.2	0.0684 incidence
J09-J18	10	1915	0.12938	0.2	0.0684 impact
J09-J18	10	1925	0.12938	0.2	0.0684 incidence
J09-J18	10	1925	0.12938	0.2	0.0684 impact
J09-J18	10	1935	0.12938	0.2	0.0684 incidence
J09-J18	10	1935	0.12938	0.2	0.0684 impact
J09-J18	10	1945	0.12938	0.2	0.0684 incidence
J09-J18	10	1945	0.12938	0.2	0.0684 impact
J09-J18	10	1955	0.12938	0.2	0.0684 incidence
J09-J18	10	1955	0.12938	0.2	0.0684 impact
R00-R09	1	1915	0.05824	0.182	0.1512 incidence
R00-R09	1	1915	0.05824	0.182	0.1512 impact
R00-R09	1	1925	0.05824	0.182	0.1512 incidence

For peer review only

R00-R09	1	1925	0.05824	0.182	0.1512	impact
R00-R09	1	1935	0.05824	0.182	0.1512	incidence
R00-R09	1	1935	0.05824	0.182	0.1512	impact
R00-R09	1	1945	0.05824	0.182	0.1512	incidence
R00-R09	1	1945	0.05824	0.182	0.1512	impact
R00-R09	1	1955	0.05824	0.182	0.1512	incidence
R00-R09	1	1955	0.05824	0.182	0.1512	impact
R00-R09	2	1915	0.06563	0.24	0.1145	incidence
R00-R09	2	1915	0.06563	0.24	0.1145	impact
R00-R09	2	1925	0.06563	0.24	0.1145	incidence
R00-R09	2	1925	0.06563	0.24	0.1145	impact
R00-R09	2	1935	0.06563	0.24	0.1145	incidence
R00-R09	2	1935	0.06563	0.24	0.1145	impact
R00-R09	2	1945	0.06563	0.24	0.1145	incidence
R00-R09	2	1945	0.06563	0.24	0.1145	impact
R00-R09	2	1955	0.06563	0.24	0.1145	incidence
R00-R09	2	1955	0.06563	0.24	0.1145	impact
R00-R09	3	1915	0.06941	0.24	0.1174	incidence
R00-R09	3	1915	0.06941	0.24	0.1174	impact
R00-R09	3	1925	0.06941	0.24	0.1174	incidence
R00-R09	3	1925	0.06941	0.24	0.1174	impact
R00-R09	3	1935	0.06941	0.24	0.1174	incidence
R00-R09	3	1935	0.06941	0.24	0.1174	impact
R00-R09	3	1945	0.06941	0.24	0.1174	incidence
R00-R09	3	1945	0.06941	0.24	0.1174	impact
R00-R09	3	1955	0.06941	0.24	0.1174	incidence
R00-R09	3	1955	0.06941	0.24	0.1174	impact
R00-R09	4	1915	0.07076	0.279	0.0989	incidence
R00-R09	4	1915	0.07076	0.279	0.0989	impact
R00-R09	4	1925	0.07076	0.279	0.0989	incidence
R00-R09	4	1925	0.07076	0.279	0.0989	impact
R00-R09	4	1935	0.07076	0.279	0.0989	incidence
R00-R09	4	1935	0.07076	0.279	0.0989	impact
R00-R09	4	1945	0.07076	0.279	0.0989	incidence
R00-R09	4	1945	0.07076	0.279	0.0989	impact
R00-R09	4	1955	0.07076	0.279	0.0989	incidence
R00-R09	4	1955	0.07076	0.279	0.0989	impact
R00-R09	5	1915	0.07736	0.0266	0.0866	incidence
R00-R09	5	1915	0.07736	0.0266	0.0866	impact
R00-R09	5	1925	0.07736	0.0266	0.0866	incidence
R00-R09	5	1925	0.07736	0.0266	0.0866	impact
R00-R09	5	1935	0.07736	0.0266	0.0866	incidence
R00-R09	5	1935	0.07736	0.0266	0.0866	impact

For peer review only

R00-R09	5	1945	0.07736	0.0266	0.0866 incidence
R00-R09	5	1945	0.07736	0.0266	0.0866 impact
R00-R09	5	1955	0.07736	0.0266	0.0866 incidence
R00-R09	5	1955	0.07736	0.0266	0.0866 impact
R00-R09	6	1915	0.08497	0.22	0.0933 incidence
R00-R09	6	1915	0.08497	0.22	0.0933 impact
R00-R09	6	1925	0.08497	0.22	0.0933 incidence
R00-R09	6	1925	0.08497	0.22	0.0933 impact
R00-R09	6	1935	0.08497	0.22	0.0933 incidence
R00-R09	6	1935	0.08497	0.22	0.0933 impact
R00-R09	6	1945	0.08497	0.22	0.0933 incidence
R00-R09	6	1945	0.08497	0.22	0.0933 impact
R00-R09	6	1955	0.08497	0.22	0.0933 incidence
R00-R09	6	1955	0.08497	0.22	0.0933 impact
R00-R09	7	1915	0.094	0.19	0.0637 incidence
R00-R09	7	1915	0.094	0.19	0.0637 impact
R00-R09	7	1925	0.094	0.19	0.0637 incidence
R00-R09	7	1925	0.094	0.19	0.0637 impact
R00-R09	7	1935	0.094	0.19	0.0637 incidence
R00-R09	7	1935	0.094	0.19	0.0637 impact
R00-R09	7	1945	0.094	0.19	0.0637 incidence
R00-R09	7	1945	0.094	0.19	0.0637 impact
R00-R09	7	1955	0.094	0.19	0.0637 incidence
R00-R09	7	1955	0.094	0.19	0.0637 impact
R00-R09	8	1915	0.09576	0.295	0.0714 incidence
R00-R09	8	1915	0.09576	0.295	0.0714 impact
R00-R09	8	1925	0.09576	0.295	0.0714 incidence
R00-R09	8	1925	0.09576	0.295	0.0714 impact
R00-R09	8	1935	0.09576	0.295	0.0714 incidence
R00-R09	8	1935	0.09576	0.295	0.0714 impact
R00-R09	8	1945	0.09576	0.295	0.0714 incidence
R00-R09	8	1945	0.09576	0.295	0.0714 impact
R00-R09	8	1955	0.09576	0.295	0.0714 incidence
R00-R09	8	1955	0.09576	0.295	0.0714 impact
R00-R09	9	1915	0.10204	0.189	0.0585 incidence
R00-R09	9	1915	0.10204	0.189	0.0585 impact
R00-R09	9	1925	0.10204	0.189	0.0585 incidence
R00-R09	9	1925	0.10204	0.189	0.0585 impact
R00-R09	9	1935	0.10204	0.189	0.0585 incidence
R00-R09	9	1935	0.10204	0.189	0.0585 impact
R00-R09	9	1945	0.10204	0.189	0.0585 incidence
R00-R09	9	1945	0.10204	0.189	0.0585 impact
R00-R09	9	1955	0.10204	0.189	0.0585 incidence

For peer review only

R00-R09	9	1955	0.10204	0.189	0.0585	impact
R00-R09	10	1915	0.11344	0.344	0.0891	incidence
R00-R09	10	1915	0.11344	0.344	0.0891	impact
R00-R09	10	1925	0.11344	0.344	0.0891	incidence
R00-R09	10	1925	0.11344	0.344	0.0891	impact
R00-R09	10	1935	0.11344	0.344	0.0891	incidence
R00-R09	10	1935	0.11344	0.344	0.0891	impact
R00-R09	10	1945	0.11344	0.344	0.0891	incidence
R00-R09	10	1945	0.11344	0.344	0.0891	impact
R00-R09	10	1955	0.11344	0.344	0.0891	incidence
R00-R09	10	1955	0.11344	0.344	0.0891	impact
C30-C39	1	1915	0.04823	0.218	0.226	incidence
C30-C39	1	1915	0.04823	0.218	0.226	impact
C30-C39	1	1925	0.04823	0.218	0.226	incidence
C30-C39	1	1925	0.04823	0.218	0.226	impact
C30-C39	1	1935	0.04823	0.218	0.226	incidence
C30-C39	1	1935	0.04823	0.218	0.226	impact
C30-C39	1	1945	0.04823	0.218	0.226	incidence
C30-C39	1	1945	0.04823	0.218	0.226	impact
C30-C39	1	1955	0.04823	0.218	0.226	incidence
C30-C39	1	1955	0.04823	0.218	0.226	impact
C30-C39	2	1915	0.05585	0.154	0.1857	incidence
C30-C39	2	1915	0.05585	0.154	0.1857	impact
C30-C39	2	1925	0.05585	0.154	0.1857	incidence
C30-C39	2	1925	0.05585	0.154	0.1857	impact
C30-C39	2	1935	0.05585	0.154	0.1857	incidence
C30-C39	2	1935	0.05585	0.154	0.1857	impact
C30-C39	2	1945	0.05585	0.154	0.1857	incidence
C30-C39	2	1945	0.05585	0.154	0.1857	impact
C30-C39	2	1955	0.05585	0.154	0.1857	incidence
C30-C39	2	1955	0.05585	0.154	0.1857	impact
C30-C39	3	1915	0.0601	0.342	0.1827	incidence
C30-C39	3	1915	0.0601	0.342	0.1827	impact
C30-C39	3	1925	0.0601	0.342	0.1827	incidence
C30-C39	3	1925	0.0601	0.342	0.1827	impact
C30-C39	3	1935	0.0601	0.342	0.1827	incidence
C30-C39	3	1935	0.0601	0.342	0.1827	impact
C30-C39	3	1945	0.0601	0.342	0.1827	incidence
C30-C39	3	1945	0.0601	0.342	0.1827	impact
C30-C39	3	1955	0.0601	0.342	0.1827	incidence
C30-C39	3	1955	0.0601	0.342	0.1827	impact
C30-C39	4	1915	0.06682	0.387	0.2018	incidence
C30-C39	4	1915	0.06682	0.387	0.2018	impact

For peer review only

C30-C39	4	1925	0.06682	0.387	0.2018	incidence
C30-C39	4	1925	0.06682	0.387	0.2018	impact
C30-C39	4	1935	0.06682	0.387	0.2018	incidence
C30-C39	4	1935	0.06682	0.387	0.2018	impact
C30-C39	4	1945	0.06682	0.387	0.2018	incidence
C30-C39	4	1945	0.06682	0.387	0.2018	impact
C30-C39	4	1955	0.06682	0.387	0.2018	incidence
C30-C39	4	1955	0.06682	0.387	0.2018	impact
C30-C39	5	1915	0.07719	0.116	0.1521	incidence
C30-C39	5	1915	0.07719	0.116	0.1521	impact
C30-C39	5	1925	0.07719	0.116	0.1521	incidence
C30-C39	5	1925	0.07719	0.116	0.1521	impact
C30-C39	5	1935	0.07719	0.116	0.1521	incidence
C30-C39	5	1935	0.07719	0.116	0.1521	impact
C30-C39	5	1945	0.07719	0.116	0.1521	incidence
C30-C39	5	1945	0.07719	0.116	0.1521	impact
C30-C39	5	1955	0.07719	0.116	0.1521	incidence
C30-C39	5	1955	0.07719	0.116	0.1521	impact
C30-C39	6	1915	0.08462	0.339	0.1115	incidence
C30-C39	6	1915	0.08462	0.339	0.1115	impact
C30-C39	6	1925	0.08462	0.339	0.1115	incidence
C30-C39	6	1925	0.08462	0.339	0.1115	impact
C30-C39	6	1935	0.08462	0.339	0.1115	incidence
C30-C39	6	1935	0.08462	0.339	0.1115	impact
C30-C39	6	1945	0.08462	0.339	0.1115	incidence
C30-C39	6	1945	0.08462	0.339	0.1115	impact
C30-C39	6	1955	0.08462	0.339	0.1115	incidence
C30-C39	6	1955	0.08462	0.339	0.1115	impact
C30-C39	7	1915	0.09461	-0.11	0.1279	incidence
C30-C39	7	1915	0.09461	-0.11	0.1279	impact
C30-C39	7	1925	0.09461	-0.11	0.1279	incidence
C30-C39	7	1925	0.09461	-0.11	0.1279	impact
C30-C39	7	1935	0.09461	-0.11	0.1279	incidence
C30-C39	7	1935	0.09461	-0.11	0.1279	impact
C30-C39	7	1945	0.09461	-0.11	0.1279	incidence
C30-C39	7	1945	0.09461	-0.11	0.1279	impact
C30-C39	7	1955	0.09461	-0.11	0.1279	incidence
C30-C39	7	1955	0.09461	-0.11	0.1279	impact
C30-C39	8	1915	0.10066	0.224	0.1223	incidence
C30-C39	8	1915	0.10066	0.224	0.1223	impact
C30-C39	8	1925	0.10066	0.224	0.1223	incidence
C30-C39	8	1925	0.10066	0.224	0.1223	impact
C30-C39	8	1935	0.10066	0.224	0.1223	incidence

For peer review only

C30-C39	8	1935	0.10066	0.224	0.1223	impact
C30-C39	8	1945	0.10066	0.224	0.1223	incidence
C30-C39	8	1945	0.10066	0.224	0.1223	impact
C30-C39	8	1955	0.10066	0.224	0.1223	incidence
C30-C39	8	1955	0.10066	0.224	0.1223	impact
C30-C39	9	1915	0.11459	0.26	0.1417	incidence
C30-C39	9	1915	0.11459	0.26	0.1417	impact
C30-C39	9	1925	0.11459	0.26	0.1417	incidence
C30-C39	9	1925	0.11459	0.26	0.1417	impact
C30-C39	9	1935	0.11459	0.26	0.1417	incidence
C30-C39	9	1935	0.11459	0.26	0.1417	impact
C30-C39	9	1945	0.11459	0.26	0.1417	incidence
C30-C39	9	1945	0.11459	0.26	0.1417	impact
C30-C39	9	1955	0.11459	0.26	0.1417	incidence
C30-C39	9	1955	0.11459	0.26	0.1417	impact
C30-C39	10	1915	0.13172	0.189	0.1183	incidence
C30-C39	10	1915	0.13172	0.189	0.1183	impact
C30-C39	10	1925	0.13172	0.189	0.1183	incidence
C30-C39	10	1925	0.13172	0.189	0.1183	impact
C30-C39	10	1935	0.13172	0.189	0.1183	incidence
C30-C39	10	1935	0.13172	0.189	0.1183	impact
C30-C39	10	1945	0.13172	0.189	0.1183	incidence
C30-C39	10	1945	0.13172	0.189	0.1183	impact
C30-C39	10	1955	0.13172	0.189	0.1183	incidence
C30-C39	10	1955	0.13172	0.189	0.1183	impact
R10-R19	1	1915	0.06568	0.327	0.1147	incidence
R10-R19	1	1915	0.06568	0.327	0.1147	impact
R10-R19	1	1925	0.06568	0.327	0.1147	incidence
R10-R19	1	1925	0.06568	0.327	0.1147	impact
R10-R19	1	1935	0.06568	0.327	0.1147	incidence
R10-R19	1	1935	0.06568	0.327	0.1147	impact
R10-R19	1	1945	0.06568	0.327	0.1147	incidence
R10-R19	1	1945	0.06568	0.327	0.1147	impact
R10-R19	1	1955	0.06568	0.327	0.1147	incidence
R10-R19	1	1955	0.06568	0.327	0.1147	impact
R10-R19	2	1915	0.07283	-0.0707	0.0794	incidence
R10-R19	2	1915	0.07283	-0.0707	0.0794	impact
R10-R19	2	1925	0.07283	-0.0707	0.0794	incidence
R10-R19	2	1925	0.07283	-0.0707	0.0794	impact
R10-R19	2	1935	0.07283	-0.0707	0.0794	incidence
R10-R19	2	1935	0.07283	-0.0707	0.0794	impact
R10-R19	2	1945	0.07283	-0.0707	0.0794	incidence
R10-R19	2	1945	0.07283	-0.0707	0.0794	impact

For peer review only

R10-R19	2	1955	0.07283	-0.0707	0.0794	incidence
R10-R19	2	1955	0.07283	-0.0707	0.0794	impact
R10-R19	3	1915	0.07177	-0.0812	0.1762	incidence
R10-R19	3	1915	0.07177	-0.0812	0.1762	impact
R10-R19	3	1925	0.07177	-0.0812	0.1762	incidence
R10-R19	3	1925	0.07177	-0.0812	0.1762	impact
R10-R19	3	1935	0.07177	-0.0812	0.1762	incidence
R10-R19	3	1935	0.07177	-0.0812	0.1762	impact
R10-R19	3	1945	0.07177	-0.0812	0.1762	incidence
R10-R19	3	1945	0.07177	-0.0812	0.1762	impact
R10-R19	3	1955	0.07177	-0.0812	0.1762	incidence
R10-R19	3	1955	0.07177	-0.0812	0.1762	impact
R10-R19	4	1915	0.07134	0.171	0.0898	incidence
R10-R19	4	1915	0.07134	0.171	0.0898	impact
R10-R19	4	1925	0.07134	0.171	0.0898	incidence
R10-R19	4	1925	0.07134	0.171	0.0898	impact
R10-R19	4	1935	0.07134	0.171	0.0898	incidence
R10-R19	4	1935	0.07134	0.171	0.0898	impact
R10-R19	4	1945	0.07134	0.171	0.0898	incidence
R10-R19	4	1945	0.07134	0.171	0.0898	impact
R10-R19	4	1955	0.07134	0.171	0.0898	incidence
R10-R19	4	1955	0.07134	0.171	0.0898	impact
R10-R19	5	1915	0.07757	0.0212	0.0662	incidence
R10-R19	5	1915	0.07757	0.0212	0.0662	impact
R10-R19	5	1925	0.07757	0.0212	0.0662	incidence
R10-R19	5	1925	0.07757	0.0212	0.0662	impact
R10-R19	5	1935	0.07757	0.0212	0.0662	incidence
R10-R19	5	1935	0.07757	0.0212	0.0662	impact
R10-R19	5	1945	0.07757	0.0212	0.0662	incidence
R10-R19	5	1945	0.07757	0.0212	0.0662	impact
R10-R19	5	1955	0.07757	0.0212	0.0662	incidence
R10-R19	5	1955	0.07757	0.0212	0.0662	impact
R10-R19	6	1915	0.08707	0.152	0.1132	incidence
R10-R19	6	1915	0.08707	0.152	0.1132	impact
R10-R19	6	1925	0.08707	0.152	0.1132	incidence
R10-R19	6	1925	0.08707	0.152	0.1132	impact
R10-R19	6	1935	0.08707	0.152	0.1132	incidence
R10-R19	6	1935	0.08707	0.152	0.1132	impact
R10-R19	6	1945	0.08707	0.152	0.1132	incidence
R10-R19	6	1945	0.08707	0.152	0.1132	impact
R10-R19	6	1955	0.08707	0.152	0.1132	incidence
R10-R19	6	1955	0.08707	0.152	0.1132	impact
R10-R19	7	1915	0.08726	0.148	0.0699	incidence

For peer review only

R10-R19	7	1915	0.08726	0.148	0.0699	impact
R10-R19	7	1925	0.08726	0.148	0.0699	incidence
R10-R19	7	1925	0.08726	0.148	0.0699	impact
R10-R19	7	1935	0.08726	0.148	0.0699	incidence
R10-R19	7	1935	0.08726	0.148	0.0699	impact
R10-R19	7	1945	0.08726	0.148	0.0699	incidence
R10-R19	7	1945	0.08726	0.148	0.0699	impact
R10-R19	7	1955	0.08726	0.148	0.0699	incidence
R10-R19	7	1955	0.08726	0.148	0.0699	impact
R10-R19	8	1915	0.09335	0.158	0.1092	incidence
R10-R19	8	1915	0.09335	0.158	0.1092	impact
R10-R19	8	1925	0.09335	0.158	0.1092	incidence
R10-R19	8	1925	0.09335	0.158	0.1092	impact
R10-R19	8	1935	0.09335	0.158	0.1092	incidence
R10-R19	8	1935	0.09335	0.158	0.1092	impact
R10-R19	8	1945	0.09335	0.158	0.1092	incidence
R10-R19	8	1945	0.09335	0.158	0.1092	impact
R10-R19	8	1955	0.09335	0.158	0.1092	incidence
R10-R19	8	1955	0.09335	0.158	0.1092	impact
R10-R19	9	1915	0.09705	0.0947	0.1415	incidence
R10-R19	9	1915	0.09705	0.0947	0.1415	impact
R10-R19	9	1925	0.09705	0.0947	0.1415	incidence
R10-R19	9	1925	0.09705	0.0947	0.1415	impact
R10-R19	9	1935	0.09705	0.0947	0.1415	incidence
R10-R19	9	1935	0.09705	0.0947	0.1415	impact
R10-R19	9	1945	0.09705	0.0947	0.1415	incidence
R10-R19	9	1945	0.09705	0.0947	0.1415	impact
R10-R19	9	1955	0.09705	0.0947	0.1415	incidence
R10-R19	9	1955	0.09705	0.0947	0.1415	impact
R10-R19	10	1915	0.10533	0.163	0.0842	incidence
R10-R19	10	1915	0.10533	0.163	0.0842	impact
R10-R19	10	1925	0.10533	0.163	0.0842	incidence
R10-R19	10	1925	0.10533	0.163	0.0842	impact
R10-R19	10	1935	0.10533	0.163	0.0842	incidence
R10-R19	10	1935	0.10533	0.163	0.0842	impact
R10-R19	10	1945	0.10533	0.163	0.0842	incidence
R10-R19	10	1945	0.10533	0.163	0.0842	impact
R10-R19	10	1955	0.10533	0.163	0.0842	incidence
R10-R19	10	1955	0.10533	0.163	0.0842	impact
R50-R69	1	1915	0.05948	0.0606	0.1279	incidence
R50-R69	1	1915	0.05948	0.0606	0.1279	impact
R50-R69	1	1925	0.05948	0.0606	0.1279	incidence
R50-R69	1	1925	0.05948	0.0606	0.1279	impact

For peer review only

R50-R69	1	1935	0.05948	0.0606	0.1279	incidence
R50-R69	1	1935	0.05948	0.0606	0.1279	impact
R50-R69	1	1945	0.05948	0.0606	0.1279	incidence
R50-R69	1	1945	0.05948	0.0606	0.1279	impact
R50-R69	1	1955	0.05948	0.0606	0.1279	incidence
R50-R69	1	1955	0.05948	0.0606	0.1279	impact
R50-R69	2	1915	0.06175	-0.126	0.1108	incidence
R50-R69	2	1915	0.06175	-0.126	0.1108	impact
R50-R69	2	1925	0.06175	-0.126	0.1108	incidence
R50-R69	2	1925	0.06175	-0.126	0.1108	impact
R50-R69	2	1935	0.06175	-0.126	0.1108	incidence
R50-R69	2	1935	0.06175	-0.126	0.1108	impact
R50-R69	2	1945	0.06175	-0.126	0.1108	incidence
R50-R69	2	1945	0.06175	-0.126	0.1108	impact
R50-R69	2	1955	0.06175	-0.126	0.1108	incidence
R50-R69	2	1955	0.06175	-0.126	0.1108	impact
R50-R69	3	1915	0.06402	-0.158	0.0825	incidence
R50-R69	3	1915	0.06402	-0.158	0.0825	impact
R50-R69	3	1925	0.06402	-0.158	0.0825	incidence
R50-R69	3	1925	0.06402	-0.158	0.0825	impact
R50-R69	3	1935	0.06402	-0.158	0.0825	incidence
R50-R69	3	1935	0.06402	-0.158	0.0825	impact
R50-R69	3	1945	0.06402	-0.158	0.0825	incidence
R50-R69	3	1945	0.06402	-0.158	0.0825	impact
R50-R69	3	1955	0.06402	-0.158	0.0825	incidence
R50-R69	3	1955	0.06402	-0.158	0.0825	impact
R50-R69	4	1915	0.06989	-0.156	0.0743	incidence
R50-R69	4	1915	0.06989	-0.156	0.0743	impact
R50-R69	4	1925	0.06989	-0.156	0.0743	incidence
R50-R69	4	1925	0.06989	-0.156	0.0743	impact
R50-R69	4	1935	0.06989	-0.156	0.0743	incidence
R50-R69	4	1935	0.06989	-0.156	0.0743	impact
R50-R69	4	1945	0.06989	-0.156	0.0743	incidence
R50-R69	4	1945	0.06989	-0.156	0.0743	impact
R50-R69	4	1955	0.06989	-0.156	0.0743	incidence
R50-R69	4	1955	0.06989	-0.156	0.0743	impact
R50-R69	5	1915	0.0726	-0.112	0.0409	incidence
R50-R69	5	1915	0.0726	-0.112	0.0409	impact
R50-R69	5	1925	0.0726	-0.112	0.0409	incidence
R50-R69	5	1925	0.0726	-0.112	0.0409	impact
R50-R69	5	1935	0.0726	-0.112	0.0409	incidence
R50-R69	5	1935	0.0726	-0.112	0.0409	impact
R50-R69	5	1945	0.0726	-0.112	0.0409	incidence

For peer review only

R50-R69	5	1945	0.0726	-0.112	0.0409	impact
R50-R69	5	1955	0.0726	-0.112	0.0409	incidence
R50-R69	5	1955	0.0726	-0.112	0.0409	impact
R50-R69	6	1915	0.08086	-0.0859	0.074	incidence
R50-R69	6	1915	0.08086	-0.0859	0.074	impact
R50-R69	6	1925	0.08086	-0.0859	0.074	incidence
R50-R69	6	1925	0.08086	-0.0859	0.074	impact
R50-R69	6	1935	0.08086	-0.0859	0.074	incidence
R50-R69	6	1935	0.08086	-0.0859	0.074	impact
R50-R69	6	1945	0.08086	-0.0859	0.074	incidence
R50-R69	6	1945	0.08086	-0.0859	0.074	impact
R50-R69	6	1955	0.08086	-0.0859	0.074	incidence
R50-R69	6	1955	0.08086	-0.0859	0.074	impact
R50-R69	7	1915	0.08435	0.219	0.05	incidence
R50-R69	7	1915	0.08435	0.219	0.05	impact
R50-R69	7	1925	0.08435	0.219	0.05	incidence
R50-R69	7	1925	0.08435	0.219	0.05	impact
R50-R69	7	1935	0.08435	0.219	0.05	incidence
R50-R69	7	1935	0.08435	0.219	0.05	impact
R50-R69	7	1945	0.08435	0.219	0.05	incidence
R50-R69	7	1945	0.08435	0.219	0.05	impact
R50-R69	7	1955	0.08435	0.219	0.05	incidence
R50-R69	7	1955	0.08435	0.219	0.05	impact
R50-R69	8	1915	0.09114	0.143	0.0592	incidence
R50-R69	8	1915	0.09114	0.143	0.0592	impact
R50-R69	8	1925	0.09114	0.143	0.0592	incidence
R50-R69	8	1925	0.09114	0.143	0.0592	impact
R50-R69	8	1935	0.09114	0.143	0.0592	incidence
R50-R69	8	1935	0.09114	0.143	0.0592	impact
R50-R69	8	1945	0.09114	0.143	0.0592	incidence
R50-R69	8	1945	0.09114	0.143	0.0592	impact
R50-R69	8	1955	0.09114	0.143	0.0592	incidence
R50-R69	8	1955	0.09114	0.143	0.0592	impact
R50-R69	9	1915	0.09458	-0.00997	0.0592	incidence
R50-R69	9	1915	0.09458	-0.00997	0.0592	impact
R50-R69	9	1925	0.09458	-0.00997	0.0592	incidence
R50-R69	9	1925	0.09458	-0.00997	0.0592	impact
R50-R69	9	1935	0.09458	-0.00997	0.0592	incidence
R50-R69	9	1935	0.09458	-0.00997	0.0592	impact
R50-R69	9	1945	0.09458	-0.00997	0.0592	incidence
R50-R69	9	1945	0.09458	-0.00997	0.0592	impact
R50-R69	9	1955	0.09458	-0.00997	0.0592	incidence
R50-R69	9	1955	0.09458	-0.00997	0.0592	impact

For peer review only

R50-R69	10	1915	0.10377	0.229	0.0593	incidence
R50-R69	10	1915	0.10377	0.229	0.0593	impact
R50-R69	10	1925	0.10377	0.229	0.0593	incidence
R50-R69	10	1925	0.10377	0.229	0.0593	impact
R50-R69	10	1935	0.10377	0.229	0.0593	incidence
R50-R69	10	1935	0.10377	0.229	0.0593	impact
R50-R69	10	1945	0.10377	0.229	0.0593	incidence
R50-R69	10	1945	0.10377	0.229	0.0593	impact
R50-R69	10	1955	0.10377	0.229	0.0593	incidence
R50-R69	10	1955	0.10377	0.229	0.0593	impact
C15-C26	1	1915	0.07182	0.503	0.0999	incidence
C15-C26	1	1915	0.07182	0.503	0.0999	impact
C15-C26	1	1925	0.07182	0.503	0.0999	incidence
C15-C26	1	1925	0.07182	0.503	0.0999	impact
C15-C26	1	1935	0.07182	0.503	0.0999	incidence
C15-C26	1	1935	0.07182	0.503	0.0999	impact
C15-C26	1	1945	0.07182	0.503	0.0999	incidence
C15-C26	1	1945	0.07182	0.503	0.0999	impact
C15-C26	1	1955	0.07182	0.503	0.0999	incidence
C15-C26	1	1955	0.07182	0.503	0.0999	impact
C15-C26	2	1915	0.07297	0.504	0.1334	incidence
C15-C26	2	1915	0.07297	0.504	0.1334	impact
C15-C26	2	1925	0.07297	0.504	0.1334	incidence
C15-C26	2	1925	0.07297	0.504	0.1334	impact
C15-C26	2	1935	0.07297	0.504	0.1334	incidence
C15-C26	2	1935	0.07297	0.504	0.1334	impact
C15-C26	2	1945	0.07297	0.504	0.1334	incidence
C15-C26	2	1945	0.07297	0.504	0.1334	impact
C15-C26	2	1955	0.07297	0.504	0.1334	incidence
C15-C26	2	1955	0.07297	0.504	0.1334	impact
C15-C26	3	1915	0.0707	0.448	0.1607	incidence
C15-C26	3	1915	0.0707	0.448	0.1607	impact
C15-C26	3	1925	0.0707	0.448	0.1607	incidence
C15-C26	3	1925	0.0707	0.448	0.1607	impact
C15-C26	3	1935	0.0707	0.448	0.1607	incidence
C15-C26	3	1935	0.0707	0.448	0.1607	impact
C15-C26	3	1945	0.0707	0.448	0.1607	incidence
C15-C26	3	1945	0.0707	0.448	0.1607	impact
C15-C26	3	1955	0.0707	0.448	0.1607	incidence
C15-C26	3	1955	0.0707	0.448	0.1607	impact
C15-C26	4	1915	0.07253	0.446	0.0749	incidence
C15-C26	4	1915	0.07253	0.446	0.0749	impact
C15-C26	4	1925	0.07253	0.446	0.0749	incidence

For peer review only

C15-C26	4	1925	0.07253	0.446	0.0749	impact
C15-C26	4	1935	0.07253	0.446	0.0749	incidence
C15-C26	4	1935	0.07253	0.446	0.0749	impact
C15-C26	4	1945	0.07253	0.446	0.0749	incidence
C15-C26	4	1945	0.07253	0.446	0.0749	impact
C15-C26	4	1955	0.07253	0.446	0.0749	incidence
C15-C26	4	1955	0.07253	0.446	0.0749	impact
C15-C26	5	1915	0.07538	0.535	0.1503	incidence
C15-C26	5	1915	0.07538	0.535	0.1503	impact
C15-C26	5	1925	0.07538	0.535	0.1503	incidence
C15-C26	5	1925	0.07538	0.535	0.1503	impact
C15-C26	5	1935	0.07538	0.535	0.1503	incidence
C15-C26	5	1935	0.07538	0.535	0.1503	impact
C15-C26	5	1945	0.07538	0.535	0.1503	incidence
C15-C26	5	1945	0.07538	0.535	0.1503	impact
C15-C26	5	1955	0.07538	0.535	0.1503	incidence
C15-C26	5	1955	0.07538	0.535	0.1503	impact
C15-C26	6	1915	0.07905	0.259	0.0994	incidence
C15-C26	6	1915	0.07905	0.259	0.0994	impact
C15-C26	6	1925	0.07905	0.259	0.0994	incidence
C15-C26	6	1925	0.07905	0.259	0.0994	impact
C15-C26	6	1935	0.07905	0.259	0.0994	incidence
C15-C26	6	1935	0.07905	0.259	0.0994	impact
C15-C26	6	1945	0.07905	0.259	0.0994	incidence
C15-C26	6	1945	0.07905	0.259	0.0994	impact
C15-C26	6	1955	0.07905	0.259	0.0994	incidence
C15-C26	6	1955	0.07905	0.259	0.0994	impact
C15-C26	7	1915	0.08115	0.215	0.1191	incidence
C15-C26	7	1915	0.08115	0.215	0.1191	impact
C15-C26	7	1925	0.08115	0.215	0.1191	incidence
C15-C26	7	1925	0.08115	0.215	0.1191	impact
C15-C26	7	1935	0.08115	0.215	0.1191	incidence
C15-C26	7	1935	0.08115	0.215	0.1191	impact
C15-C26	7	1945	0.08115	0.215	0.1191	incidence
C15-C26	7	1945	0.08115	0.215	0.1191	impact
C15-C26	7	1955	0.08115	0.215	0.1191	incidence
C15-C26	7	1955	0.08115	0.215	0.1191	impact
C15-C26	8	1915	0.08203	0.294	0.1213	incidence
C15-C26	8	1915	0.08203	0.294	0.1213	impact
C15-C26	8	1925	0.08203	0.294	0.1213	incidence
C15-C26	8	1925	0.08203	0.294	0.1213	impact
C15-C26	8	1935	0.08203	0.294	0.1213	incidence
C15-C26	8	1935	0.08203	0.294	0.1213	impact

For peer review only

C15-C26	8	1945	0.08203	0.294	0.1213	incidence
C15-C26	8	1945	0.08203	0.294	0.1213	impact
C15-C26	8	1955	0.08203	0.294	0.1213	incidence
C15-C26	8	1955	0.08203	0.294	0.1213	impact
C15-C26	9	1915	0.0805	0.293	0.1242	incidence
C15-C26	9	1915	0.0805	0.293	0.1242	impact
C15-C26	9	1925	0.0805	0.293	0.1242	incidence
C15-C26	9	1925	0.0805	0.293	0.1242	impact
C15-C26	9	1935	0.0805	0.293	0.1242	incidence
C15-C26	9	1935	0.0805	0.293	0.1242	impact
C15-C26	9	1945	0.0805	0.293	0.1242	incidence
C15-C26	9	1945	0.0805	0.293	0.1242	impact
C15-C26	9	1955	0.0805	0.293	0.1242	incidence
C15-C26	9	1955	0.0805	0.293	0.1242	impact
C15-C26	10	1915	0.08054	0.486	0.1388	incidence
C15-C26	10	1915	0.08054	0.486	0.1388	impact
C15-C26	10	1925	0.08054	0.486	0.1388	incidence
C15-C26	10	1925	0.08054	0.486	0.1388	impact
C15-C26	10	1935	0.08054	0.486	0.1388	incidence
C15-C26	10	1935	0.08054	0.486	0.1388	impact
C15-C26	10	1945	0.08054	0.486	0.1388	incidence
C15-C26	10	1945	0.08054	0.486	0.1388	impact
C15-C26	10	1955	0.08054	0.486	0.1388	incidence
C15-C26	10	1955	0.08054	0.486	0.1388	impact
I30-I52	1	1915	0.05901	0.385	0.1052	incidence
I30-I52	1	1915	0.05901	0.385	0.1052	impact
I30-I52	1	1925	0.05901	0.385	0.1052	incidence
I30-I52	1	1925	0.05901	0.385	0.1052	impact
I30-I52	1	1935	0.05901	0.385	0.1052	incidence
I30-I52	1	1935	0.05901	0.385	0.1052	impact
I30-I52	1	1945	0.05901	0.385	0.1052	incidence
I30-I52	1	1945	0.05901	0.385	0.1052	impact
I30-I52	1	1955	0.05901	0.385	0.1052	incidence
I30-I52	1	1955	0.05901	0.385	0.1052	impact
I30-I52	2	1915	0.06281	0.204	0.0691	incidence
I30-I52	2	1915	0.06281	0.204	0.0691	impact
I30-I52	2	1925	0.06281	0.204	0.0691	incidence
I30-I52	2	1925	0.06281	0.204	0.0691	impact
I30-I52	2	1935	0.06281	0.204	0.0691	incidence
I30-I52	2	1935	0.06281	0.204	0.0691	impact
I30-I52	2	1945	0.06281	0.204	0.0691	incidence
I30-I52	2	1945	0.06281	0.204	0.0691	impact
I30-I52	2	1955	0.06281	0.204	0.0691	incidence

For peer review only

I30-I52	2	1955	0.06281	0.204	0.0691	impact
I30-I52	3	1915	0.06088	-0.0376	0.1333	incidence
I30-I52	3	1915	0.06088	-0.0376	0.1333	impact
I30-I52	3	1925	0.06088	-0.0376	0.1333	incidence
I30-I52	3	1925	0.06088	-0.0376	0.1333	impact
I30-I52	3	1935	0.06088	-0.0376	0.1333	incidence
I30-I52	3	1935	0.06088	-0.0376	0.1333	impact
I30-I52	3	1945	0.06088	-0.0376	0.1333	incidence
I30-I52	3	1945	0.06088	-0.0376	0.1333	impact
I30-I52	3	1955	0.06088	-0.0376	0.1333	incidence
I30-I52	3	1955	0.06088	-0.0376	0.1333	impact
I30-I52	4	1915	0.06727	0.284	0.1168	incidence
I30-I52	4	1915	0.06727	0.284	0.1168	impact
I30-I52	4	1925	0.06727	0.284	0.1168	incidence
I30-I52	4	1925	0.06727	0.284	0.1168	impact
I30-I52	4	1935	0.06727	0.284	0.1168	incidence
I30-I52	4	1935	0.06727	0.284	0.1168	impact
I30-I52	4	1945	0.06727	0.284	0.1168	incidence
I30-I52	4	1945	0.06727	0.284	0.1168	impact
I30-I52	4	1955	0.06727	0.284	0.1168	incidence
I30-I52	4	1955	0.06727	0.284	0.1168	impact
I30-I52	5	1915	0.07252	0.23	0.0366	incidence
I30-I52	5	1915	0.07252	0.23	0.0366	impact
I30-I52	5	1925	0.07252	0.23	0.0366	incidence
I30-I52	5	1925	0.07252	0.23	0.0366	impact
I30-I52	5	1935	0.07252	0.23	0.0366	incidence
I30-I52	5	1935	0.07252	0.23	0.0366	impact
I30-I52	5	1945	0.07252	0.23	0.0366	incidence
I30-I52	5	1945	0.07252	0.23	0.0366	impact
I30-I52	5	1955	0.07252	0.23	0.0366	incidence
I30-I52	5	1955	0.07252	0.23	0.0366	impact
I30-I52	6	1915	0.07431	0.253	0.0737	incidence
I30-I52	6	1915	0.07431	0.253	0.0737	impact
I30-I52	6	1925	0.07431	0.253	0.0737	incidence
I30-I52	6	1925	0.07431	0.253	0.0737	impact
I30-I52	6	1935	0.07431	0.253	0.0737	incidence
I30-I52	6	1935	0.07431	0.253	0.0737	impact
I30-I52	6	1945	0.07431	0.253	0.0737	incidence
I30-I52	6	1945	0.07431	0.253	0.0737	impact
I30-I52	6	1955	0.07431	0.253	0.0737	incidence
I30-I52	6	1955	0.07431	0.253	0.0737	impact
I30-I52	7	1915	0.0814	0.264	0.0761	incidence
I30-I52	7	1915	0.0814	0.264	0.0761	impact

For peer review only

I30-I52	7	1925	0.0814	0.264	0.0761	incidence
I30-I52	7	1925	0.0814	0.264	0.0761	impact
I30-I52	7	1935	0.0814	0.264	0.0761	incidence
I30-I52	7	1935	0.0814	0.264	0.0761	impact
I30-I52	7	1945	0.0814	0.264	0.0761	incidence
I30-I52	7	1945	0.0814	0.264	0.0761	impact
I30-I52	7	1955	0.0814	0.264	0.0761	incidence
I30-I52	7	1955	0.0814	0.264	0.0761	impact
I30-I52	8	1915	0.08139	0.116	0.0487	incidence
I30-I52	8	1915	0.08139	0.116	0.0487	impact
I30-I52	8	1925	0.08139	0.116	0.0487	incidence
I30-I52	8	1925	0.08139	0.116	0.0487	impact
I30-I52	8	1935	0.08139	0.116	0.0487	incidence
I30-I52	8	1935	0.08139	0.116	0.0487	impact
I30-I52	8	1945	0.08139	0.116	0.0487	incidence
I30-I52	8	1945	0.08139	0.116	0.0487	impact
I30-I52	8	1955	0.08139	0.116	0.0487	incidence
I30-I52	8	1955	0.08139	0.116	0.0487	impact
I30-I52	9	1915	0.08033	0.0407	0.0909	incidence
I30-I52	9	1915	0.08033	0.0407	0.0909	impact
I30-I52	9	1925	0.08033	0.0407	0.0909	incidence
I30-I52	9	1925	0.08033	0.0407	0.0909	impact
I30-I52	9	1935	0.08033	0.0407	0.0909	incidence
I30-I52	9	1935	0.08033	0.0407	0.0909	impact
I30-I52	9	1945	0.08033	0.0407	0.0909	incidence
I30-I52	9	1945	0.08033	0.0407	0.0909	impact
I30-I52	9	1955	0.08033	0.0407	0.0909	incidence
I30-I52	9	1955	0.08033	0.0407	0.0909	impact
I30-I52	10	1915	0.07841	0.072	0.0981	incidence
I30-I52	10	1915	0.07841	0.072	0.0981	impact
I30-I52	10	1925	0.07841	0.072	0.0981	incidence
I30-I52	10	1925	0.07841	0.072	0.0981	impact
I30-I52	10	1935	0.07841	0.072	0.0981	incidence
I30-I52	10	1935	0.07841	0.072	0.0981	impact
I30-I52	10	1945	0.07841	0.072	0.0981	incidence
I30-I52	10	1945	0.07841	0.072	0.0981	impact
I30-I52	10	1955	0.07841	0.072	0.0981	incidence
I30-I52	10	1955	0.07841	0.072	0.0981	impact
C76-C80	1	1915	0.06351	0.353	0.1424	incidence
C76-C80	1	1915	0.06351	0.353	0.1424	impact
C76-C80	1	1925	0.06351	0.353	0.1424	incidence
C76-C80	1	1925	0.06351	0.353	0.1424	impact
C76-C80	1	1935	0.06351	0.353	0.1424	incidence

For peer review only

C76-C80	1	1935	0.06351	0.353	0.1424	impact
C76-C80	1	1945	0.06351	0.353	0.1424	incidence
C76-C80	1	1945	0.06351	0.353	0.1424	impact
C76-C80	1	1955	0.06351	0.353	0.1424	incidence
C76-C80	1	1955	0.06351	0.353	0.1424	impact
C76-C80	2	1915	0.06043	0.062	0.2428	incidence
C76-C80	2	1915	0.06043	0.062	0.2428	impact
C76-C80	2	1925	0.06043	0.062	0.2428	incidence
C76-C80	2	1925	0.06043	0.062	0.2428	impact
C76-C80	2	1935	0.06043	0.062	0.2428	incidence
C76-C80	2	1935	0.06043	0.062	0.2428	impact
C76-C80	2	1945	0.06043	0.062	0.2428	incidence
C76-C80	2	1945	0.06043	0.062	0.2428	impact
C76-C80	2	1955	0.06043	0.062	0.2428	incidence
C76-C80	2	1955	0.06043	0.062	0.2428	impact
C76-C80	3	1915	0.06169	0.461	0.1858	incidence
C76-C80	3	1915	0.06169	0.461	0.1858	impact
C76-C80	3	1925	0.06169	0.461	0.1858	incidence
C76-C80	3	1925	0.06169	0.461	0.1858	impact
C76-C80	3	1935	0.06169	0.461	0.1858	incidence
C76-C80	3	1935	0.06169	0.461	0.1858	impact
C76-C80	3	1945	0.06169	0.461	0.1858	incidence
C76-C80	3	1945	0.06169	0.461	0.1858	impact
C76-C80	3	1955	0.06169	0.461	0.1858	incidence
C76-C80	3	1955	0.06169	0.461	0.1858	impact
C76-C80	4	1915	0.06455	0.429	0.2195	incidence
C76-C80	4	1915	0.06455	0.429	0.2195	impact
C76-C80	4	1925	0.06455	0.429	0.2195	incidence
C76-C80	4	1925	0.06455	0.429	0.2195	impact
C76-C80	4	1935	0.06455	0.429	0.2195	incidence
C76-C80	4	1935	0.06455	0.429	0.2195	impact
C76-C80	4	1945	0.06455	0.429	0.2195	incidence
C76-C80	4	1945	0.06455	0.429	0.2195	impact
C76-C80	4	1955	0.06455	0.429	0.2195	incidence
C76-C80	4	1955	0.06455	0.429	0.2195	impact
C76-C80	5	1915	0.06494	0.28	0.1804	incidence
C76-C80	5	1915	0.06494	0.28	0.1804	impact
C76-C80	5	1925	0.06494	0.28	0.1804	incidence
C76-C80	5	1925	0.06494	0.28	0.1804	impact
C76-C80	5	1935	0.06494	0.28	0.1804	incidence
C76-C80	5	1935	0.06494	0.28	0.1804	impact
C76-C80	5	1945	0.06494	0.28	0.1804	incidence
C76-C80	5	1945	0.06494	0.28	0.1804	impact

For peer review only

C76-C80	5	1955	0.06494	0.28	0.1804	incidence
C76-C80	5	1955	0.06494	0.28	0.1804	impact
C76-C80	6	1915	0.06744	0.582	0.1907	incidence
C76-C80	6	1915	0.06744	0.582	0.1907	impact
C76-C80	6	1925	0.06744	0.582	0.1907	incidence
C76-C80	6	1925	0.06744	0.582	0.1907	impact
C76-C80	6	1935	0.06744	0.582	0.1907	incidence
C76-C80	6	1935	0.06744	0.582	0.1907	impact
C76-C80	6	1945	0.06744	0.582	0.1907	incidence
C76-C80	6	1945	0.06744	0.582	0.1907	impact
C76-C80	6	1955	0.06744	0.582	0.1907	incidence
C76-C80	6	1955	0.06744	0.582	0.1907	impact
C76-C80	7	1915	0.06698	0.479	0.1987	incidence
C76-C80	7	1915	0.06698	0.479	0.1987	impact
C76-C80	7	1925	0.06698	0.479	0.1987	incidence
C76-C80	7	1925	0.06698	0.479	0.1987	impact
C76-C80	7	1935	0.06698	0.479	0.1987	incidence
C76-C80	7	1935	0.06698	0.479	0.1987	impact
C76-C80	7	1945	0.06698	0.479	0.1987	incidence
C76-C80	7	1945	0.06698	0.479	0.1987	impact
C76-C80	7	1955	0.06698	0.479	0.1987	incidence
C76-C80	7	1955	0.06698	0.479	0.1987	impact
C76-C80	8	1915	0.06417	0.518	0.181	incidence
C76-C80	8	1915	0.06417	0.518	0.181	impact
C76-C80	8	1925	0.06417	0.518	0.181	incidence
C76-C80	8	1925	0.06417	0.518	0.181	impact
C76-C80	8	1935	0.06417	0.518	0.181	incidence
C76-C80	8	1935	0.06417	0.518	0.181	impact
C76-C80	8	1945	0.06417	0.518	0.181	incidence
C76-C80	8	1945	0.06417	0.518	0.181	impact
C76-C80	8	1955	0.06417	0.518	0.181	incidence
C76-C80	8	1955	0.06417	0.518	0.181	impact
C76-C80	9	1915	0.07071	0.411	0.1457	incidence
C76-C80	9	1915	0.07071	0.411	0.1457	impact
C76-C80	9	1925	0.07071	0.411	0.1457	incidence
C76-C80	9	1925	0.07071	0.411	0.1457	impact
C76-C80	9	1935	0.07071	0.411	0.1457	incidence
C76-C80	9	1935	0.07071	0.411	0.1457	impact
C76-C80	9	1945	0.07071	0.411	0.1457	incidence
C76-C80	9	1945	0.07071	0.411	0.1457	impact
C76-C80	9	1955	0.07071	0.411	0.1457	incidence
C76-C80	9	1955	0.07071	0.411	0.1457	impact
C76-C80	10	1915	0.06817	-0.137	0.2519	incidence

For peer review only

C76-C80	10	1915	0.06817	-0.137	0.2519	impact
C76-C80	10	1925	0.06817	-0.137	0.2519	incidence
C76-C80	10	1925	0.06817	-0.137	0.2519	impact
C76-C80	10	1935	0.06817	-0.137	0.2519	incidence
C76-C80	10	1935	0.06817	-0.137	0.2519	impact
C76-C80	10	1945	0.06817	-0.137	0.2519	incidence
C76-C80	10	1945	0.06817	-0.137	0.2519	impact
C76-C80	10	1955	0.06817	-0.137	0.2519	incidence
C76-C80	10	1955	0.06817	-0.137	0.2519	impact
Z40-Z54	1	1915	0.05436	0.508	0.1214	incidence
Z40-Z54	1	1915	0.05436	0.508	0.1214	impact
Z40-Z54	1	1925	0.05436	0.508	0.1214	incidence
Z40-Z54	1	1925	0.05436	0.508	0.1214	impact
Z40-Z54	1	1935	0.05436	0.508	0.1214	incidence
Z40-Z54	1	1935	0.05436	0.508	0.1214	impact
Z40-Z54	1	1945	0.05436	0.508	0.1214	incidence
Z40-Z54	1	1945	0.05436	0.508	0.1214	impact
Z40-Z54	1	1955	0.05436	0.508	0.1214	incidence
Z40-Z54	1	1955	0.05436	0.508	0.1214	impact
Z40-Z54	2	1915	0.05805	0.296	0.1157	incidence
Z40-Z54	2	1915	0.05805	0.296	0.1157	impact
Z40-Z54	2	1925	0.05805	0.296	0.1157	incidence
Z40-Z54	2	1925	0.05805	0.296	0.1157	impact
Z40-Z54	2	1935	0.05805	0.296	0.1157	incidence
Z40-Z54	2	1935	0.05805	0.296	0.1157	impact
Z40-Z54	2	1945	0.05805	0.296	0.1157	incidence
Z40-Z54	2	1945	0.05805	0.296	0.1157	impact
Z40-Z54	2	1955	0.05805	0.296	0.1157	incidence
Z40-Z54	2	1955	0.05805	0.296	0.1157	impact
Z40-Z54	3	1915	0.05967	0.136	0.0591	incidence
Z40-Z54	3	1915	0.05967	0.136	0.0591	impact
Z40-Z54	3	1925	0.05967	0.136	0.0591	incidence
Z40-Z54	3	1925	0.05967	0.136	0.0591	impact
Z40-Z54	3	1935	0.05967	0.136	0.0591	incidence
Z40-Z54	3	1935	0.05967	0.136	0.0591	impact
Z40-Z54	3	1945	0.05967	0.136	0.0591	incidence
Z40-Z54	3	1945	0.05967	0.136	0.0591	impact
Z40-Z54	3	1955	0.05967	0.136	0.0591	incidence
Z40-Z54	3	1955	0.05967	0.136	0.0591	impact
Z40-Z54	4	1915	0.05692	0.288	0.0692	incidence
Z40-Z54	4	1915	0.05692	0.288	0.0692	impact
Z40-Z54	4	1925	0.05692	0.288	0.0692	incidence
Z40-Z54	4	1925	0.05692	0.288	0.0692	impact

For peer review only

Z40-Z54	4	1935	0.05692	0.288	0.0692	incidence
Z40-Z54	4	1935	0.05692	0.288	0.0692	impact
Z40-Z54	4	1945	0.05692	0.288	0.0692	incidence
Z40-Z54	4	1945	0.05692	0.288	0.0692	impact
Z40-Z54	4	1955	0.05692	0.288	0.0692	incidence
Z40-Z54	4	1955	0.05692	0.288	0.0692	impact
Z40-Z54	5	1915	0.06141	0.41	0.0748	incidence
Z40-Z54	5	1915	0.06141	0.41	0.0748	impact
Z40-Z54	5	1925	0.06141	0.41	0.0748	incidence
Z40-Z54	5	1925	0.06141	0.41	0.0748	impact
Z40-Z54	5	1935	0.06141	0.41	0.0748	incidence
Z40-Z54	5	1935	0.06141	0.41	0.0748	impact
Z40-Z54	5	1945	0.06141	0.41	0.0748	incidence
Z40-Z54	5	1945	0.06141	0.41	0.0748	impact
Z40-Z54	5	1955	0.06141	0.41	0.0748	incidence
Z40-Z54	5	1955	0.06141	0.41	0.0748	impact
Z40-Z54	6	1915	0.06444	0.184	0.0853	incidence
Z40-Z54	6	1915	0.06444	0.184	0.0853	impact
Z40-Z54	6	1925	0.06444	0.184	0.0853	incidence
Z40-Z54	6	1925	0.06444	0.184	0.0853	impact
Z40-Z54	6	1935	0.06444	0.184	0.0853	incidence
Z40-Z54	6	1935	0.06444	0.184	0.0853	impact
Z40-Z54	6	1945	0.06444	0.184	0.0853	incidence
Z40-Z54	6	1945	0.06444	0.184	0.0853	impact
Z40-Z54	6	1955	0.06444	0.184	0.0853	incidence
Z40-Z54	6	1955	0.06444	0.184	0.0853	impact
Z40-Z54	7	1915	0.06444	0.342	0.0583	incidence
Z40-Z54	7	1915	0.06444	0.342	0.0583	impact
Z40-Z54	7	1925	0.06444	0.342	0.0583	incidence
Z40-Z54	7	1925	0.06444	0.342	0.0583	impact
Z40-Z54	7	1935	0.06444	0.342	0.0583	incidence
Z40-Z54	7	1935	0.06444	0.342	0.0583	impact
Z40-Z54	7	1945	0.06444	0.342	0.0583	incidence
Z40-Z54	7	1945	0.06444	0.342	0.0583	impact
Z40-Z54	7	1955	0.06444	0.342	0.0583	incidence
Z40-Z54	7	1955	0.06444	0.342	0.0583	impact
Z40-Z54	8	1915	0.06346	0.404	0.0808	incidence
Z40-Z54	8	1915	0.06346	0.404	0.0808	impact
Z40-Z54	8	1925	0.06346	0.404	0.0808	incidence
Z40-Z54	8	1925	0.06346	0.404	0.0808	impact
Z40-Z54	8	1935	0.06346	0.404	0.0808	incidence
Z40-Z54	8	1935	0.06346	0.404	0.0808	impact
Z40-Z54	8	1945	0.06346	0.404	0.0808	incidence

For peer review only

Z40-Z54	8	1945	0.06346	0.404	0.0808	impact
Z40-Z54	8	1955	0.06346	0.404	0.0808	incidence
Z40-Z54	8	1955	0.06346	0.404	0.0808	impact
Z40-Z54	9	1915	0.06254	0.178	0.1077	incidence
Z40-Z54	9	1915	0.06254	0.178	0.1077	impact
Z40-Z54	9	1925	0.06254	0.178	0.1077	incidence
Z40-Z54	9	1925	0.06254	0.178	0.1077	impact
Z40-Z54	9	1935	0.06254	0.178	0.1077	incidence
Z40-Z54	9	1935	0.06254	0.178	0.1077	impact
Z40-Z54	9	1945	0.06254	0.178	0.1077	incidence
Z40-Z54	9	1945	0.06254	0.178	0.1077	impact
Z40-Z54	9	1955	0.06254	0.178	0.1077	incidence
Z40-Z54	9	1955	0.06254	0.178	0.1077	impact
Z40-Z54	10	1915	0.06599	0.237	0.0504	incidence
Z40-Z54	10	1915	0.06599	0.237	0.0504	impact
Z40-Z54	10	1925	0.06599	0.237	0.0504	incidence
Z40-Z54	10	1925	0.06599	0.237	0.0504	impact
Z40-Z54	10	1935	0.06599	0.237	0.0504	incidence
Z40-Z54	10	1935	0.06599	0.237	0.0504	impact
Z40-Z54	10	1945	0.06599	0.237	0.0504	incidence
Z40-Z54	10	1945	0.06599	0.237	0.0504	impact
Z40-Z54	10	1955	0.06599	0.237	0.0504	incidence
Z40-Z54	10	1955	0.06599	0.237	0.0504	impact
I60-I69	1	1915	0.04602	0.335	0.1035	incidence
I60-I69	1	1915	0.04602	0.335	0.1035	impact
I60-I69	1	1925	0.04602	0.335	0.1035	incidence
I60-I69	1	1925	0.04602	0.335	0.1035	impact
I60-I69	1	1935	0.04602	0.335	0.1035	incidence
I60-I69	1	1935	0.04602	0.335	0.1035	impact
I60-I69	1	1945	0.04602	0.335	0.1035	incidence
I60-I69	1	1945	0.04602	0.335	0.1035	impact
I60-I69	1	1955	0.04602	0.335	0.1035	incidence
I60-I69	1	1955	0.04602	0.335	0.1035	impact
I60-I69	2	1915	0.04864	0.113	0.0658	incidence
I60-I69	2	1915	0.04864	0.113	0.0658	impact
I60-I69	2	1925	0.04864	0.113	0.0658	incidence
I60-I69	2	1925	0.04864	0.113	0.0658	impact
I60-I69	2	1935	0.04864	0.113	0.0658	incidence
I60-I69	2	1935	0.04864	0.113	0.0658	impact
I60-I69	2	1945	0.04864	0.113	0.0658	incidence
I60-I69	2	1945	0.04864	0.113	0.0658	impact
I60-I69	2	1955	0.04864	0.113	0.0658	incidence
I60-I69	2	1955	0.04864	0.113	0.0658	impact

For peer review only

I60-I69	3	1915	0.04845	-0.0343	0.0859	incidence
I60-I69	3	1915	0.04845	-0.0343	0.0859	impact
I60-I69	3	1925	0.04845	-0.0343	0.0859	incidence
I60-I69	3	1925	0.04845	-0.0343	0.0859	impact
I60-I69	3	1935	0.04845	-0.0343	0.0859	incidence
I60-I69	3	1935	0.04845	-0.0343	0.0859	impact
I60-I69	3	1945	0.04845	-0.0343	0.0859	incidence
I60-I69	3	1945	0.04845	-0.0343	0.0859	impact
I60-I69	3	1955	0.04845	-0.0343	0.0859	incidence
I60-I69	3	1955	0.04845	-0.0343	0.0859	impact
I60-I69	4	1915	0.05517	0.0596	0.0747	incidence
I60-I69	4	1915	0.05517	0.0596	0.0747	impact
I60-I69	4	1925	0.05517	0.0596	0.0747	incidence
I60-I69	4	1925	0.05517	0.0596	0.0747	impact
I60-I69	4	1935	0.05517	0.0596	0.0747	incidence
I60-I69	4	1935	0.05517	0.0596	0.0747	impact
I60-I69	4	1945	0.05517	0.0596	0.0747	incidence
I60-I69	4	1945	0.05517	0.0596	0.0747	impact
I60-I69	4	1955	0.05517	0.0596	0.0747	incidence
I60-I69	4	1955	0.05517	0.0596	0.0747	impact
I60-I69	5	1915	0.0566	-0.0936	0.1751	incidence
I60-I69	5	1915	0.0566	-0.0936	0.1751	impact
I60-I69	5	1925	0.0566	-0.0936	0.1751	incidence
I60-I69	5	1925	0.0566	-0.0936	0.1751	impact
I60-I69	5	1935	0.0566	-0.0936	0.1751	incidence
I60-I69	5	1935	0.0566	-0.0936	0.1751	impact
I60-I69	5	1945	0.0566	-0.0936	0.1751	incidence
I60-I69	5	1945	0.0566	-0.0936	0.1751	impact
I60-I69	5	1955	0.0566	-0.0936	0.1751	incidence
I60-I69	5	1955	0.0566	-0.0936	0.1751	impact
I60-I69	6	1915	0.05977	0.326	0.0758	incidence
I60-I69	6	1915	0.05977	0.326	0.0758	impact
I60-I69	6	1925	0.05977	0.326	0.0758	incidence
I60-I69	6	1925	0.05977	0.326	0.0758	impact
I60-I69	6	1935	0.05977	0.326	0.0758	incidence
I60-I69	6	1935	0.05977	0.326	0.0758	impact
I60-I69	6	1945	0.05977	0.326	0.0758	incidence
I60-I69	6	1945	0.05977	0.326	0.0758	impact
I60-I69	6	1955	0.05977	0.326	0.0758	incidence
I60-I69	6	1955	0.05977	0.326	0.0758	impact
I60-I69	7	1915	0.06041	0.224	0.1172	incidence
I60-I69	7	1915	0.06041	0.224	0.1172	impact
I60-I69	7	1925	0.06041	0.224	0.1172	incidence

For peer review only

I60-I69	7	1925	0.06041	0.224	0.1172	impact
I60-I69	7	1935	0.06041	0.224	0.1172	incidence
I60-I69	7	1935	0.06041	0.224	0.1172	impact
I60-I69	7	1945	0.06041	0.224	0.1172	incidence
I60-I69	7	1945	0.06041	0.224	0.1172	impact
I60-I69	7	1955	0.06041	0.224	0.1172	incidence
I60-I69	7	1955	0.06041	0.224	0.1172	impact
I60-I69	8	1915	0.06284	0.162	0.1262	incidence
I60-I69	8	1915	0.06284	0.162	0.1262	impact
I60-I69	8	1925	0.06284	0.162	0.1262	incidence
I60-I69	8	1925	0.06284	0.162	0.1262	impact
I60-I69	8	1935	0.06284	0.162	0.1262	incidence
I60-I69	8	1935	0.06284	0.162	0.1262	impact
I60-I69	8	1945	0.06284	0.162	0.1262	incidence
I60-I69	8	1945	0.06284	0.162	0.1262	impact
I60-I69	8	1955	0.06284	0.162	0.1262	incidence
I60-I69	8	1955	0.06284	0.162	0.1262	impact
I60-I69	9	1915	0.06622	0.344	0.1757	incidence
I60-I69	9	1915	0.06622	0.344	0.1757	impact
I60-I69	9	1925	0.06622	0.344	0.1757	incidence
I60-I69	9	1925	0.06622	0.344	0.1757	impact
I60-I69	9	1935	0.06622	0.344	0.1757	incidence
I60-I69	9	1935	0.06622	0.344	0.1757	impact
I60-I69	9	1945	0.06622	0.344	0.1757	incidence
I60-I69	9	1945	0.06622	0.344	0.1757	impact
I60-I69	9	1955	0.06622	0.344	0.1757	incidence
I60-I69	9	1955	0.06622	0.344	0.1757	impact
I60-I69	10	1915	0.0661	0.387	0.0974	incidence
I60-I69	10	1915	0.0661	0.387	0.0974	impact
I60-I69	10	1925	0.0661	0.387	0.0974	incidence
I60-I69	10	1925	0.0661	0.387	0.0974	impact
I60-I69	10	1935	0.0661	0.387	0.0974	incidence
I60-I69	10	1935	0.0661	0.387	0.0974	impact
I60-I69	10	1945	0.0661	0.387	0.0974	incidence
I60-I69	10	1945	0.0661	0.387	0.0974	impact
I60-I69	10	1955	0.0661	0.387	0.0974	incidence
I60-I69	10	1955	0.0661	0.387	0.0974	impact
K55-K63	1	1915	0.05313	0.372	0.0952	incidence
K55-K63	1	1915	0.05313	0.372	0.0952	impact
K55-K63	1	1925	0.05313	0.372	0.0952	incidence
K55-K63	1	1925	0.05313	0.372	0.0952	impact
K55-K63	1	1935	0.05313	0.372	0.0952	incidence
K55-K63	1	1935	0.05313	0.372	0.0952	impact

For peer review only

K55-K63	1	1945	0.05313	0.372	0.0952	incidence
K55-K63	1	1945	0.05313	0.372	0.0952	impact
K55-K63	1	1955	0.05313	0.372	0.0952	incidence
K55-K63	1	1955	0.05313	0.372	0.0952	impact
K55-K63	2	1915	0.0558	0.352	0.1217	incidence
K55-K63	2	1915	0.0558	0.352	0.1217	impact
K55-K63	2	1925	0.0558	0.352	0.1217	incidence
K55-K63	2	1925	0.0558	0.352	0.1217	impact
K55-K63	2	1935	0.0558	0.352	0.1217	incidence
K55-K63	2	1935	0.0558	0.352	0.1217	impact
K55-K63	2	1945	0.0558	0.352	0.1217	incidence
K55-K63	2	1945	0.0558	0.352	0.1217	impact
K55-K63	2	1955	0.0558	0.352	0.1217	incidence
K55-K63	2	1955	0.0558	0.352	0.1217	impact
K55-K63	3	1915	0.05091	0.156	0.1045	incidence
K55-K63	3	1915	0.05091	0.156	0.1045	impact
K55-K63	3	1925	0.05091	0.156	0.1045	incidence
K55-K63	3	1925	0.05091	0.156	0.1045	impact
K55-K63	3	1935	0.05091	0.156	0.1045	incidence
K55-K63	3	1935	0.05091	0.156	0.1045	impact
K55-K63	3	1945	0.05091	0.156	0.1045	incidence
K55-K63	3	1945	0.05091	0.156	0.1045	impact
K55-K63	3	1955	0.05091	0.156	0.1045	incidence
K55-K63	3	1955	0.05091	0.156	0.1045	impact
K55-K63	4	1915	0.05251	0.133	0.0466	incidence
K55-K63	4	1915	0.05251	0.133	0.0466	impact
K55-K63	4	1925	0.05251	0.133	0.0466	incidence
K55-K63	4	1925	0.05251	0.133	0.0466	impact
K55-K63	4	1935	0.05251	0.133	0.0466	incidence
K55-K63	4	1935	0.05251	0.133	0.0466	impact
K55-K63	4	1945	0.05251	0.133	0.0466	incidence
K55-K63	4	1945	0.05251	0.133	0.0466	impact
K55-K63	4	1955	0.05251	0.133	0.0466	incidence
K55-K63	4	1955	0.05251	0.133	0.0466	impact
K55-K63	5	1915	0.05646	0.322	0.0821	incidence
K55-K63	5	1915	0.05646	0.322	0.0821	impact
K55-K63	5	1925	0.05646	0.322	0.0821	incidence
K55-K63	5	1925	0.05646	0.322	0.0821	impact
K55-K63	5	1935	0.05646	0.322	0.0821	incidence
K55-K63	5	1935	0.05646	0.322	0.0821	impact
K55-K63	5	1945	0.05646	0.322	0.0821	incidence
K55-K63	5	1945	0.05646	0.322	0.0821	impact
K55-K63	5	1955	0.05646	0.322	0.0821	incidence

For peer review only

K55-K63	5	1955	0.05646	0.322	0.0821	impact
K55-K63	6	1915	0.0608	0.108	0.0607	incidence
K55-K63	6	1915	0.0608	0.108	0.0607	impact
K55-K63	6	1925	0.0608	0.108	0.0607	incidence
K55-K63	6	1925	0.0608	0.108	0.0607	impact
K55-K63	6	1935	0.0608	0.108	0.0607	incidence
K55-K63	6	1935	0.0608	0.108	0.0607	impact
K55-K63	6	1945	0.0608	0.108	0.0607	incidence
K55-K63	6	1945	0.0608	0.108	0.0607	impact
K55-K63	6	1955	0.0608	0.108	0.0607	incidence
K55-K63	6	1955	0.0608	0.108	0.0607	impact
K55-K63	7	1915	0.05797	0.231	0.076	incidence
K55-K63	7	1915	0.05797	0.231	0.076	impact
K55-K63	7	1925	0.05797	0.231	0.076	incidence
K55-K63	7	1925	0.05797	0.231	0.076	impact
K55-K63	7	1935	0.05797	0.231	0.076	incidence
K55-K63	7	1935	0.05797	0.231	0.076	impact
K55-K63	7	1945	0.05797	0.231	0.076	incidence
K55-K63	7	1945	0.05797	0.231	0.076	impact
K55-K63	7	1955	0.05797	0.231	0.076	incidence
K55-K63	7	1955	0.05797	0.231	0.076	impact
K55-K63	8	1915	0.06053	0.16	0.0355	incidence
K55-K63	8	1915	0.06053	0.16	0.0355	impact
K55-K63	8	1925	0.06053	0.16	0.0355	incidence
K55-K63	8	1925	0.06053	0.16	0.0355	impact
K55-K63	8	1935	0.06053	0.16	0.0355	incidence
K55-K63	8	1935	0.06053	0.16	0.0355	impact
K55-K63	8	1945	0.06053	0.16	0.0355	incidence
K55-K63	8	1945	0.06053	0.16	0.0355	impact
K55-K63	8	1955	0.06053	0.16	0.0355	incidence
K55-K63	8	1955	0.06053	0.16	0.0355	impact
K55-K63	9	1915	0.06093	0.0284	0.0519	incidence
K55-K63	9	1915	0.06093	0.0284	0.0519	impact
K55-K63	9	1925	0.06093	0.0284	0.0519	incidence
K55-K63	9	1925	0.06093	0.0284	0.0519	impact
K55-K63	9	1935	0.06093	0.0284	0.0519	incidence
K55-K63	9	1935	0.06093	0.0284	0.0519	impact
K55-K63	9	1945	0.06093	0.0284	0.0519	incidence
K55-K63	9	1945	0.06093	0.0284	0.0519	impact
K55-K63	9	1955	0.06093	0.0284	0.0519	incidence
K55-K63	9	1955	0.06093	0.0284	0.0519	impact
K55-K63	10	1915	0.06045	0.438	0.0651	incidence
K55-K63	10	1915	0.06045	0.438	0.0651	impact

For peer review only

K55-K63	10	1925	0.06045	0.438	0.0651 incidence
K55-K63	10	1925	0.06045	0.438	0.0651 impact
K55-K63	10	1935	0.06045	0.438	0.0651 incidence
K55-K63	10	1935	0.06045	0.438	0.0651 impact
K55-K63	10	1945	0.06045	0.438	0.0651 incidence
K55-K63	10	1945	0.06045	0.438	0.0651 impact
K55-K63	10	1955	0.06045	0.438	0.0651 incidence
K55-K63	10	1955	0.06045	0.438	0.0651 impact
J40-J47	1	1915	0.02506	-0.0521	0.2423 incidence
J40-J47	1	1915	0.02506	-0.0521	0.2423 impact
J40-J47	1	1925	0.02506	-0.0521	0.2423 incidence
J40-J47	1	1925	0.02506	-0.0521	0.2423 impact
J40-J47	1	1935	0.02506	-0.0521	0.2423 incidence
J40-J47	1	1935	0.02506	-0.0521	0.2423 impact
J40-J47	1	1945	0.02506	-0.0521	0.2423 incidence
J40-J47	1	1945	0.02506	-0.0521	0.2423 impact
J40-J47	1	1955	0.02506	-0.0521	0.2423 incidence
J40-J47	1	1955	0.02506	-0.0521	0.2423 impact
J40-J47	2	1915	0.02988	0.302	0.0996 incidence
J40-J47	2	1915	0.02988	0.302	0.0996 impact
J40-J47	2	1925	0.02988	0.302	0.0996 incidence
J40-J47	2	1925	0.02988	0.302	0.0996 impact
J40-J47	2	1935	0.02988	0.302	0.0996 incidence
J40-J47	2	1935	0.02988	0.302	0.0996 impact
J40-J47	2	1945	0.02988	0.302	0.0996 incidence
J40-J47	2	1945	0.02988	0.302	0.0996 impact
J40-J47	2	1955	0.02988	0.302	0.0996 incidence
J40-J47	2	1955	0.02988	0.302	0.0996 impact
J40-J47	3	1915	0.03616	0.219	0.1306 incidence
J40-J47	3	1915	0.03616	0.219	0.1306 impact
J40-J47	3	1925	0.03616	0.219	0.1306 incidence
J40-J47	3	1925	0.03616	0.219	0.1306 impact
J40-J47	3	1935	0.03616	0.219	0.1306 incidence
J40-J47	3	1935	0.03616	0.219	0.1306 impact
J40-J47	3	1945	0.03616	0.219	0.1306 incidence
J40-J47	3	1945	0.03616	0.219	0.1306 impact
J40-J47	3	1955	0.03616	0.219	0.1306 incidence
J40-J47	3	1955	0.03616	0.219	0.1306 impact
J40-J47	4	1915	0.04058	0.302	0.1523 incidence
J40-J47	4	1915	0.04058	0.302	0.1523 impact
J40-J47	4	1925	0.04058	0.302	0.1523 incidence
J40-J47	4	1925	0.04058	0.302	0.1523 impact
J40-J47	4	1935	0.04058	0.302	0.1523 incidence

For peer review only

J40-J47	4	1935	0.04058	0.302	0.1523	impact
J40-J47	4	1945	0.04058	0.302	0.1523	incidence
J40-J47	4	1945	0.04058	0.302	0.1523	impact
J40-J47	4	1955	0.04058	0.302	0.1523	incidence
J40-J47	4	1955	0.04058	0.302	0.1523	impact
J40-J47	5	1915	0.04837	0.108	0.1366	incidence
J40-J47	5	1915	0.04837	0.108	0.1366	impact
J40-J47	5	1925	0.04837	0.108	0.1366	incidence
J40-J47	5	1925	0.04837	0.108	0.1366	impact
J40-J47	5	1935	0.04837	0.108	0.1366	incidence
J40-J47	5	1935	0.04837	0.108	0.1366	impact
J40-J47	5	1945	0.04837	0.108	0.1366	incidence
J40-J47	5	1945	0.04837	0.108	0.1366	impact
J40-J47	5	1955	0.04837	0.108	0.1366	incidence
J40-J47	5	1955	0.04837	0.108	0.1366	impact
J40-J47	6	1915	0.056	0.0917	0.1844	incidence
J40-J47	6	1915	0.056	0.0917	0.1844	impact
J40-J47	6	1925	0.056	0.0917	0.1844	incidence
J40-J47	6	1925	0.056	0.0917	0.1844	impact
J40-J47	6	1935	0.056	0.0917	0.1844	incidence
J40-J47	6	1935	0.056	0.0917	0.1844	impact
J40-J47	6	1945	0.056	0.0917	0.1844	incidence
J40-J47	6	1945	0.056	0.0917	0.1844	impact
J40-J47	6	1955	0.056	0.0917	0.1844	incidence
J40-J47	6	1955	0.056	0.0917	0.1844	impact
J40-J47	7	1915	0.06357	0.225	0.0885	incidence
J40-J47	7	1915	0.06357	0.225	0.0885	impact
J40-J47	7	1925	0.06357	0.225	0.0885	incidence
J40-J47	7	1925	0.06357	0.225	0.0885	impact
J40-J47	7	1935	0.06357	0.225	0.0885	incidence
J40-J47	7	1935	0.06357	0.225	0.0885	impact
J40-J47	7	1945	0.06357	0.225	0.0885	incidence
J40-J47	7	1945	0.06357	0.225	0.0885	impact
J40-J47	7	1955	0.06357	0.225	0.0885	incidence
J40-J47	7	1955	0.06357	0.225	0.0885	impact
J40-J47	8	1915	0.07319	0.18	0.0345	incidence
J40-J47	8	1915	0.07319	0.18	0.0345	impact
J40-J47	8	1925	0.07319	0.18	0.0345	incidence
J40-J47	8	1925	0.07319	0.18	0.0345	impact
J40-J47	8	1935	0.07319	0.18	0.0345	incidence
J40-J47	8	1935	0.07319	0.18	0.0345	impact
J40-J47	8	1945	0.07319	0.18	0.0345	incidence
J40-J47	8	1945	0.07319	0.18	0.0345	impact

For peer review only

J40-J47	8	1955	0.07319	0.18	0.0345 incidence
J40-J47	8	1955	0.07319	0.18	0.0345 impact
J40-J47	9	1915	0.08131	0.372	0.0696 incidence
J40-J47	9	1915	0.08131	0.372	0.0696 impact
J40-J47	9	1925	0.08131	0.372	0.0696 incidence
J40-J47	9	1925	0.08131	0.372	0.0696 impact
J40-J47	9	1935	0.08131	0.372	0.0696 incidence
J40-J47	9	1935	0.08131	0.372	0.0696 impact
J40-J47	9	1945	0.08131	0.372	0.0696 incidence
J40-J47	9	1945	0.08131	0.372	0.0696 impact
J40-J47	9	1955	0.08131	0.372	0.0696 incidence
J40-J47	9	1955	0.08131	0.372	0.0696 impact
J40-J47	10	1915	0.09425	0.32	0.0923 incidence
J40-J47	10	1915	0.09425	0.32	0.0923 impact
J40-J47	10	1925	0.09425	0.32	0.0923 incidence
J40-J47	10	1925	0.09425	0.32	0.0923 impact
J40-J47	10	1935	0.09425	0.32	0.0923 incidence
J40-J47	10	1935	0.09425	0.32	0.0923 impact
J40-J47	10	1945	0.09425	0.32	0.0923 incidence
J40-J47	10	1945	0.09425	0.32	0.0923 impact
J40-J47	10	1955	0.09425	0.32	0.0923 incidence
J40-J47	10	1955	0.09425	0.32	0.0923 impact
I20-I25	1	1915	0.04147	0.272	0.2127 incidence
I20-I25	1	1915	0.04147	0.272	0.2127 impact
I20-I25	1	1925	0.04147	0.272	0.2127 incidence
I20-I25	1	1925	0.04147	0.272	0.2127 impact
I20-I25	1	1935	0.04147	0.272	0.2127 incidence
I20-I25	1	1935	0.04147	0.272	0.2127 impact
I20-I25	1	1945	0.04147	0.272	0.2127 incidence
I20-I25	1	1945	0.04147	0.272	0.2127 impact
I20-I25	1	1955	0.04147	0.272	0.2127 incidence
I20-I25	1	1955	0.04147	0.272	0.2127 impact
I20-I25	2	1915	0.04704	0.274	0.1164 incidence
I20-I25	2	1915	0.04704	0.274	0.1164 impact
I20-I25	2	1925	0.04704	0.274	0.1164 incidence
I20-I25	2	1925	0.04704	0.274	0.1164 impact
I20-I25	2	1935	0.04704	0.274	0.1164 incidence
I20-I25	2	1935	0.04704	0.274	0.1164 impact
I20-I25	2	1945	0.04704	0.274	0.1164 incidence
I20-I25	2	1945	0.04704	0.274	0.1164 impact
I20-I25	2	1955	0.04704	0.274	0.1164 incidence
I20-I25	2	1955	0.04704	0.274	0.1164 impact
I20-I25	3	1915	0.04845	0.0976	0.0974 incidence

For peer review only

I20-I25	3	1915	0.04845	0.0976	0.0974	impact
I20-I25	3	1925	0.04845	0.0976	0.0974	incidence
I20-I25	3	1925	0.04845	0.0976	0.0974	impact
I20-I25	3	1935	0.04845	0.0976	0.0974	incidence
I20-I25	3	1935	0.04845	0.0976	0.0974	impact
I20-I25	3	1945	0.04845	0.0976	0.0974	incidence
I20-I25	3	1945	0.04845	0.0976	0.0974	impact
I20-I25	3	1955	0.04845	0.0976	0.0974	incidence
I20-I25	3	1955	0.04845	0.0976	0.0974	impact
I20-I25	4	1915	0.05142	0.373	0.0591	incidence
I20-I25	4	1915	0.05142	0.373	0.0591	impact
I20-I25	4	1925	0.05142	0.373	0.0591	incidence
I20-I25	4	1925	0.05142	0.373	0.0591	impact
I20-I25	4	1935	0.05142	0.373	0.0591	incidence
I20-I25	4	1935	0.05142	0.373	0.0591	impact
I20-I25	4	1945	0.05142	0.373	0.0591	incidence
I20-I25	4	1945	0.05142	0.373	0.0591	impact
I20-I25	4	1955	0.05142	0.373	0.0591	incidence
I20-I25	4	1955	0.05142	0.373	0.0591	impact
I20-I25	5	1915	0.0542	0.227	0.1244	incidence
I20-I25	5	1915	0.0542	0.227	0.1244	impact
I20-I25	5	1925	0.0542	0.227	0.1244	incidence
I20-I25	5	1925	0.0542	0.227	0.1244	impact
I20-I25	5	1935	0.0542	0.227	0.1244	incidence
I20-I25	5	1935	0.0542	0.227	0.1244	impact
I20-I25	5	1945	0.0542	0.227	0.1244	incidence
I20-I25	5	1945	0.0542	0.227	0.1244	impact
I20-I25	5	1955	0.0542	0.227	0.1244	incidence
I20-I25	5	1955	0.0542	0.227	0.1244	impact
I20-I25	6	1915	0.05741	0.288	0.0802	incidence
I20-I25	6	1915	0.05741	0.288	0.0802	impact
I20-I25	6	1925	0.05741	0.288	0.0802	incidence
I20-I25	6	1925	0.05741	0.288	0.0802	impact
I20-I25	6	1935	0.05741	0.288	0.0802	incidence
I20-I25	6	1935	0.05741	0.288	0.0802	impact
I20-I25	6	1945	0.05741	0.288	0.0802	incidence
I20-I25	6	1945	0.05741	0.288	0.0802	impact
I20-I25	6	1955	0.05741	0.288	0.0802	incidence
I20-I25	6	1955	0.05741	0.288	0.0802	impact
I20-I25	7	1915	0.06044	0.245	0.1318	incidence
I20-I25	7	1915	0.06044	0.245	0.1318	impact
I20-I25	7	1925	0.06044	0.245	0.1318	incidence
I20-I25	7	1925	0.06044	0.245	0.1318	impact

For peer review only

I20-I25	7	1935	0.06044	0.245	0.1318	incidence
I20-I25	7	1935	0.06044	0.245	0.1318	impact
I20-I25	7	1945	0.06044	0.245	0.1318	incidence
I20-I25	7	1945	0.06044	0.245	0.1318	impact
I20-I25	7	1955	0.06044	0.245	0.1318	incidence
I20-I25	7	1955	0.06044	0.245	0.1318	impact
I20-I25	8	1915	0.0609	0.411	0.041	incidence
I20-I25	8	1915	0.0609	0.411	0.041	impact
I20-I25	8	1925	0.0609	0.411	0.041	incidence
I20-I25	8	1925	0.0609	0.411	0.041	impact
I20-I25	8	1935	0.0609	0.411	0.041	incidence
I20-I25	8	1935	0.0609	0.411	0.041	impact
I20-I25	8	1945	0.0609	0.411	0.041	incidence
I20-I25	8	1945	0.0609	0.411	0.041	impact
I20-I25	8	1955	0.0609	0.411	0.041	incidence
I20-I25	8	1955	0.0609	0.411	0.041	impact
I20-I25	9	1915	0.05894	0.183	0.0856	incidence
I20-I25	9	1915	0.05894	0.183	0.0856	impact
I20-I25	9	1925	0.05894	0.183	0.0856	incidence
I20-I25	9	1925	0.05894	0.183	0.0856	impact
I20-I25	9	1935	0.05894	0.183	0.0856	incidence
I20-I25	9	1935	0.05894	0.183	0.0856	impact
I20-I25	9	1945	0.05894	0.183	0.0856	incidence
I20-I25	9	1945	0.05894	0.183	0.0856	impact
I20-I25	9	1955	0.05894	0.183	0.0856	incidence
I20-I25	9	1955	0.05894	0.183	0.0856	impact
I20-I25	10	1915	0.05665	0.236	0.0807	incidence
I20-I25	10	1915	0.05665	0.236	0.0807	impact
I20-I25	10	1925	0.05665	0.236	0.0807	incidence
I20-I25	10	1925	0.05665	0.236	0.0807	impact
I20-I25	10	1935	0.05665	0.236	0.0807	incidence
I20-I25	10	1935	0.05665	0.236	0.0807	impact
I20-I25	10	1945	0.05665	0.236	0.0807	incidence
I20-I25	10	1945	0.05665	0.236	0.0807	impact
I20-I25	10	1955	0.05665	0.236	0.0807	incidence
I20-I25	10	1955	0.05665	0.236	0.0807	impact
N30-N39	1	1915	0.04904	0.00162	0.0681	incidence
N30-N39	1	1915	0.04904	0.00162	0.0681	impact
N30-N39	1	1925	0.04904	0.00162	0.0681	incidence
N30-N39	1	1925	0.04904	0.00162	0.0681	impact
N30-N39	1	1935	0.04904	0.00162	0.0681	incidence
N30-N39	1	1935	0.04904	0.00162	0.0681	impact
N30-N39	1	1945	0.04904	0.00162	0.0681	incidence

For peer review only

N30-N39	1	1945	0.04904	0.00162	0.0681	impact
N30-N39	1	1955	0.04904	0.00162	0.0681	incidence
N30-N39	1	1955	0.04904	0.00162	0.0681	impact
N30-N39	2	1915	0.05089	-0.132	0.113	incidence
N30-N39	2	1915	0.05089	-0.132	0.113	impact
N30-N39	2	1925	0.05089	-0.132	0.113	incidence
N30-N39	2	1925	0.05089	-0.132	0.113	impact
N30-N39	2	1935	0.05089	-0.132	0.113	incidence
N30-N39	2	1935	0.05089	-0.132	0.113	impact
N30-N39	2	1945	0.05089	-0.132	0.113	incidence
N30-N39	2	1945	0.05089	-0.132	0.113	impact
N30-N39	2	1955	0.05089	-0.132	0.113	incidence
N30-N39	2	1955	0.05089	-0.132	0.113	impact
N30-N39	3	1915	0.05158	-0.258	0.0691	incidence
N30-N39	3	1915	0.05158	-0.258	0.0691	impact
N30-N39	3	1925	0.05158	-0.258	0.0691	incidence
N30-N39	3	1925	0.05158	-0.258	0.0691	impact
N30-N39	3	1935	0.05158	-0.258	0.0691	incidence
N30-N39	3	1935	0.05158	-0.258	0.0691	impact
N30-N39	3	1945	0.05158	-0.258	0.0691	incidence
N30-N39	3	1945	0.05158	-0.258	0.0691	impact
N30-N39	3	1955	0.05158	-0.258	0.0691	incidence
N30-N39	3	1955	0.05158	-0.258	0.0691	impact
N30-N39	4	1915	0.05025	-0.276	0.0693	incidence
N30-N39	4	1915	0.05025	-0.276	0.0693	impact
N30-N39	4	1925	0.05025	-0.276	0.0693	incidence
N30-N39	4	1925	0.05025	-0.276	0.0693	impact
N30-N39	4	1935	0.05025	-0.276	0.0693	incidence
N30-N39	4	1935	0.05025	-0.276	0.0693	impact
N30-N39	4	1945	0.05025	-0.276	0.0693	incidence
N30-N39	4	1945	0.05025	-0.276	0.0693	impact
N30-N39	4	1955	0.05025	-0.276	0.0693	incidence
N30-N39	4	1955	0.05025	-0.276	0.0693	impact
N30-N39	5	1915	0.05282	-0.167	0.074	incidence
N30-N39	5	1915	0.05282	-0.167	0.074	impact
N30-N39	5	1925	0.05282	-0.167	0.074	incidence
N30-N39	5	1925	0.05282	-0.167	0.074	impact
N30-N39	5	1935	0.05282	-0.167	0.074	incidence
N30-N39	5	1935	0.05282	-0.167	0.074	impact
N30-N39	5	1945	0.05282	-0.167	0.074	incidence
N30-N39	5	1945	0.05282	-0.167	0.074	impact
N30-N39	5	1955	0.05282	-0.167	0.074	incidence
N30-N39	5	1955	0.05282	-0.167	0.074	impact

For peer review only

N30-N39	6	1915	0.05613	-0.164	0.1009	incidence
N30-N39	6	1915	0.05613	-0.164	0.1009	impact
N30-N39	6	1925	0.05613	-0.164	0.1009	incidence
N30-N39	6	1925	0.05613	-0.164	0.1009	impact
N30-N39	6	1935	0.05613	-0.164	0.1009	incidence
N30-N39	6	1935	0.05613	-0.164	0.1009	impact
N30-N39	6	1945	0.05613	-0.164	0.1009	incidence
N30-N39	6	1945	0.05613	-0.164	0.1009	impact
N30-N39	6	1955	0.05613	-0.164	0.1009	incidence
N30-N39	6	1955	0.05613	-0.164	0.1009	impact
N30-N39	7	1915	0.05793	-0.298	0.139	incidence
N30-N39	7	1915	0.05793	-0.298	0.139	impact
N30-N39	7	1925	0.05793	-0.298	0.139	incidence
N30-N39	7	1925	0.05793	-0.298	0.139	impact
N30-N39	7	1935	0.05793	-0.298	0.139	incidence
N30-N39	7	1935	0.05793	-0.298	0.139	impact
N30-N39	7	1945	0.05793	-0.298	0.139	incidence
N30-N39	7	1945	0.05793	-0.298	0.139	impact
N30-N39	7	1955	0.05793	-0.298	0.139	incidence
N30-N39	7	1955	0.05793	-0.298	0.139	impact
N30-N39	8	1915	0.05605	-0.17	0.0693	incidence
N30-N39	8	1915	0.05605	-0.17	0.0693	impact
N30-N39	8	1925	0.05605	-0.17	0.0693	incidence
N30-N39	8	1925	0.05605	-0.17	0.0693	impact
N30-N39	8	1935	0.05605	-0.17	0.0693	incidence
N30-N39	8	1935	0.05605	-0.17	0.0693	impact
N30-N39	8	1945	0.05605	-0.17	0.0693	incidence
N30-N39	8	1945	0.05605	-0.17	0.0693	impact
N30-N39	8	1955	0.05605	-0.17	0.0693	incidence
N30-N39	8	1955	0.05605	-0.17	0.0693	impact
N30-N39	9	1915	0.05751	-0.28	0.0692	incidence
N30-N39	9	1915	0.05751	-0.28	0.0692	impact
N30-N39	9	1925	0.05751	-0.28	0.0692	incidence
N30-N39	9	1925	0.05751	-0.28	0.0692	impact
N30-N39	9	1935	0.05751	-0.28	0.0692	incidence
N30-N39	9	1935	0.05751	-0.28	0.0692	impact
N30-N39	9	1945	0.05751	-0.28	0.0692	incidence
N30-N39	9	1945	0.05751	-0.28	0.0692	impact
N30-N39	9	1955	0.05751	-0.28	0.0692	incidence
N30-N39	9	1955	0.05751	-0.28	0.0692	impact
N30-N39	10	1915	0.05699	-0.318	0.0632	incidence
N30-N39	10	1915	0.05699	-0.318	0.0632	impact
N30-N39	10	1925	0.05699	-0.318	0.0632	incidence

For peer review only

N30-N39	10	1925	0.05699	-0.318	0.0632	impact
N30-N39	10	1935	0.05699	-0.318	0.0632	incidence
N30-N39	10	1935	0.05699	-0.318	0.0632	impact
N30-N39	10	1945	0.05699	-0.318	0.0632	incidence
N30-N39	10	1945	0.05699	-0.318	0.0632	impact
N30-N39	10	1955	0.05699	-0.318	0.0632	incidence
N30-N39	10	1955	0.05699	-0.318	0.0632	impact
K20-K31	1	1915	0.03766	-0.0496	0.0955	incidence
K20-K31	1	1915	0.03766	-0.0496	0.0955	impact
K20-K31	1	1925	0.03766	-0.0496	0.0955	incidence
K20-K31	1	1925	0.03766	-0.0496	0.0955	impact
K20-K31	1	1935	0.03766	-0.0496	0.0955	incidence
K20-K31	1	1935	0.03766	-0.0496	0.0955	impact
K20-K31	1	1945	0.03766	-0.0496	0.0955	incidence
K20-K31	1	1945	0.03766	-0.0496	0.0955	impact
K20-K31	1	1955	0.03766	-0.0496	0.0955	incidence
K20-K31	1	1955	0.03766	-0.0496	0.0955	impact
K20-K31	2	1915	0.04783	-0.106	0.1136	incidence
K20-K31	2	1915	0.04783	-0.106	0.1136	impact
K20-K31	2	1925	0.04783	-0.106	0.1136	incidence
K20-K31	2	1925	0.04783	-0.106	0.1136	impact
K20-K31	2	1935	0.04783	-0.106	0.1136	incidence
K20-K31	2	1935	0.04783	-0.106	0.1136	impact
K20-K31	2	1945	0.04783	-0.106	0.1136	incidence
K20-K31	2	1945	0.04783	-0.106	0.1136	impact
K20-K31	2	1955	0.04783	-0.106	0.1136	incidence
K20-K31	2	1955	0.04783	-0.106	0.1136	impact
K20-K31	3	1915	0.04096	0.0126	0.1419	incidence
K20-K31	3	1915	0.04096	0.0126	0.1419	impact
K20-K31	3	1925	0.04096	0.0126	0.1419	incidence
K20-K31	3	1925	0.04096	0.0126	0.1419	impact
K20-K31	3	1935	0.04096	0.0126	0.1419	incidence
K20-K31	3	1935	0.04096	0.0126	0.1419	impact
K20-K31	3	1945	0.04096	0.0126	0.1419	incidence
K20-K31	3	1945	0.04096	0.0126	0.1419	impact
K20-K31	3	1955	0.04096	0.0126	0.1419	incidence
K20-K31	3	1955	0.04096	0.0126	0.1419	impact
K20-K31	4	1915	0.04399	0.388	0.1158	incidence
K20-K31	4	1915	0.04399	0.388	0.1158	impact
K20-K31	4	1925	0.04399	0.388	0.1158	incidence
K20-K31	4	1925	0.04399	0.388	0.1158	impact
K20-K31	4	1935	0.04399	0.388	0.1158	incidence
K20-K31	4	1935	0.04399	0.388	0.1158	impact

For peer review only

K20-K31	4	1945	0.04399	0.388	0.1158	incidence
K20-K31	4	1945	0.04399	0.388	0.1158	impact
K20-K31	4	1955	0.04399	0.388	0.1158	incidence
K20-K31	4	1955	0.04399	0.388	0.1158	impact
K20-K31	5	1915	0.04809	0.0629	0.1036	incidence
K20-K31	5	1915	0.04809	0.0629	0.1036	impact
K20-K31	5	1925	0.04809	0.0629	0.1036	incidence
K20-K31	5	1925	0.04809	0.0629	0.1036	impact
K20-K31	5	1935	0.04809	0.0629	0.1036	incidence
K20-K31	5	1935	0.04809	0.0629	0.1036	impact
K20-K31	5	1945	0.04809	0.0629	0.1036	incidence
K20-K31	5	1945	0.04809	0.0629	0.1036	impact
K20-K31	5	1955	0.04809	0.0629	0.1036	incidence
K20-K31	5	1955	0.04809	0.0629	0.1036	impact
K20-K31	6	1915	0.05308	0.107	0.0414	incidence
K20-K31	6	1915	0.05308	0.107	0.0414	impact
K20-K31	6	1925	0.05308	0.107	0.0414	incidence
K20-K31	6	1925	0.05308	0.107	0.0414	impact
K20-K31	6	1935	0.05308	0.107	0.0414	incidence
K20-K31	6	1935	0.05308	0.107	0.0414	impact
K20-K31	6	1945	0.05308	0.107	0.0414	incidence
K20-K31	6	1945	0.05308	0.107	0.0414	impact
K20-K31	6	1955	0.05308	0.107	0.0414	incidence
K20-K31	6	1955	0.05308	0.107	0.0414	impact
K20-K31	7	1915	0.05566	0.0204	0.101	incidence
K20-K31	7	1915	0.05566	0.0204	0.101	impact
K20-K31	7	1925	0.05566	0.0204	0.101	incidence
K20-K31	7	1925	0.05566	0.0204	0.101	impact
K20-K31	7	1935	0.05566	0.0204	0.101	incidence
K20-K31	7	1935	0.05566	0.0204	0.101	impact
K20-K31	7	1945	0.05566	0.0204	0.101	incidence
K20-K31	7	1945	0.05566	0.0204	0.101	impact
K20-K31	7	1955	0.05566	0.0204	0.101	incidence
K20-K31	7	1955	0.05566	0.0204	0.101	impact
K20-K31	8	1915	0.0597	0.16	0.1355	incidence
K20-K31	8	1915	0.0597	0.16	0.1355	impact
K20-K31	8	1925	0.0597	0.16	0.1355	incidence
K20-K31	8	1925	0.0597	0.16	0.1355	impact
K20-K31	8	1935	0.0597	0.16	0.1355	incidence
K20-K31	8	1935	0.0597	0.16	0.1355	impact
K20-K31	8	1945	0.0597	0.16	0.1355	incidence
K20-K31	8	1945	0.0597	0.16	0.1355	impact
K20-K31	8	1955	0.0597	0.16	0.1355	incidence

For peer review only

K20-K31	8	1955	0.0597	0.16	0.1355	impact
K20-K31	9	1915	0.06451	0.176	0.086	incidence
K20-K31	9	1915	0.06451	0.176	0.086	impact
K20-K31	9	1925	0.06451	0.176	0.086	incidence
K20-K31	9	1925	0.06451	0.176	0.086	impact
K20-K31	9	1935	0.06451	0.176	0.086	incidence
K20-K31	9	1935	0.06451	0.176	0.086	impact
K20-K31	9	1945	0.06451	0.176	0.086	incidence
K20-K31	9	1945	0.06451	0.176	0.086	impact
K20-K31	9	1955	0.06451	0.176	0.086	incidence
K20-K31	9	1955	0.06451	0.176	0.086	impact
K20-K31	10	1915	0.07311	0.282	0.0986	incidence
K20-K31	10	1915	0.07311	0.282	0.0986	impact
K20-K31	10	1925	0.07311	0.282	0.0986	incidence
K20-K31	10	1925	0.07311	0.282	0.0986	impact
K20-K31	10	1935	0.07311	0.282	0.0986	incidence
K20-K31	10	1935	0.07311	0.282	0.0986	impact
K20-K31	10	1945	0.07311	0.282	0.0986	incidence
K20-K31	10	1945	0.07311	0.282	0.0986	impact
K20-K31	10	1955	0.07311	0.282	0.0986	incidence
K20-K31	10	1955	0.07311	0.282	0.0986	impact
J20-J22	1	1915	0.03207	-0.316	0.1645	incidence
J20-J22	1	1915	0.03207	-0.316	0.1645	impact
J20-J22	1	1925	0.03207	-0.316	0.1645	incidence
J20-J22	1	1925	0.03207	-0.316	0.1645	impact
J20-J22	1	1935	0.03207	-0.316	0.1645	incidence
J20-J22	1	1935	0.03207	-0.316	0.1645	impact
J20-J22	1	1945	0.03207	-0.316	0.1645	incidence
J20-J22	1	1945	0.03207	-0.316	0.1645	impact
J20-J22	1	1955	0.03207	-0.316	0.1645	incidence
J20-J22	1	1955	0.03207	-0.316	0.1645	impact
J20-J22	2	1915	0.03618	-0.221	0.0764	incidence
J20-J22	2	1915	0.03618	-0.221	0.0764	impact
J20-J22	2	1925	0.03618	-0.221	0.0764	incidence
J20-J22	2	1925	0.03618	-0.221	0.0764	impact
J20-J22	2	1935	0.03618	-0.221	0.0764	incidence
J20-J22	2	1935	0.03618	-0.221	0.0764	impact
J20-J22	2	1945	0.03618	-0.221	0.0764	incidence
J20-J22	2	1945	0.03618	-0.221	0.0764	impact
J20-J22	2	1955	0.03618	-0.221	0.0764	incidence
J20-J22	2	1955	0.03618	-0.221	0.0764	impact
J20-J22	3	1915	0.03897	-0.0651	0.0942	incidence
J20-J22	3	1915	0.03897	-0.0651	0.0942	impact

For peer review only

J20-J22	3	1925	0.03897	-0.0651	0.0942	incidence
J20-J22	3	1925	0.03897	-0.0651	0.0942	impact
J20-J22	3	1935	0.03897	-0.0651	0.0942	incidence
J20-J22	3	1935	0.03897	-0.0651	0.0942	impact
J20-J22	3	1945	0.03897	-0.0651	0.0942	incidence
J20-J22	3	1945	0.03897	-0.0651	0.0942	impact
J20-J22	3	1955	0.03897	-0.0651	0.0942	incidence
J20-J22	3	1955	0.03897	-0.0651	0.0942	impact
J20-J22	4	1915	0.04249	-0.236	0.0827	incidence
J20-J22	4	1915	0.04249	-0.236	0.0827	impact
J20-J22	4	1925	0.04249	-0.236	0.0827	incidence
J20-J22	4	1925	0.04249	-0.236	0.0827	impact
J20-J22	4	1935	0.04249	-0.236	0.0827	incidence
J20-J22	4	1935	0.04249	-0.236	0.0827	impact
J20-J22	4	1945	0.04249	-0.236	0.0827	incidence
J20-J22	4	1945	0.04249	-0.236	0.0827	impact
J20-J22	4	1955	0.04249	-0.236	0.0827	incidence
J20-J22	4	1955	0.04249	-0.236	0.0827	impact
J20-J22	5	1915	0.04629	-0.169	0.1004	incidence
J20-J22	5	1915	0.04629	-0.169	0.1004	impact
J20-J22	5	1925	0.04629	-0.169	0.1004	incidence
J20-J22	5	1925	0.04629	-0.169	0.1004	impact
J20-J22	5	1935	0.04629	-0.169	0.1004	incidence
J20-J22	5	1935	0.04629	-0.169	0.1004	impact
J20-J22	5	1945	0.04629	-0.169	0.1004	incidence
J20-J22	5	1945	0.04629	-0.169	0.1004	impact
J20-J22	5	1955	0.04629	-0.169	0.1004	incidence
J20-J22	5	1955	0.04629	-0.169	0.1004	impact
J20-J22	6	1915	0.05025	-0.175	0.1064	incidence
J20-J22	6	1915	0.05025	-0.175	0.1064	impact
J20-J22	6	1925	0.05025	-0.175	0.1064	incidence
J20-J22	6	1925	0.05025	-0.175	0.1064	impact
J20-J22	6	1935	0.05025	-0.175	0.1064	incidence
J20-J22	6	1935	0.05025	-0.175	0.1064	impact
J20-J22	6	1945	0.05025	-0.175	0.1064	incidence
J20-J22	6	1945	0.05025	-0.175	0.1064	impact
J20-J22	6	1955	0.05025	-0.175	0.1064	incidence
J20-J22	6	1955	0.05025	-0.175	0.1064	impact
J20-J22	7	1915	0.05574	-0.108	0.0707	incidence
J20-J22	7	1915	0.05574	-0.108	0.0707	impact
J20-J22	7	1925	0.05574	-0.108	0.0707	incidence
J20-J22	7	1925	0.05574	-0.108	0.0707	impact
J20-J22	7	1935	0.05574	-0.108	0.0707	incidence

For peer review only

J20-J22	7	1935	0.05574	-0.108	0.0707	impact
J20-J22	7	1945	0.05574	-0.108	0.0707	incidence
J20-J22	7	1945	0.05574	-0.108	0.0707	impact
J20-J22	7	1955	0.05574	-0.108	0.0707	incidence
J20-J22	7	1955	0.05574	-0.108	0.0707	impact
J20-J22	8	1915	0.05951	0.143	0.0706	incidence
J20-J22	8	1915	0.05951	0.143	0.0706	impact
J20-J22	8	1925	0.05951	0.143	0.0706	incidence
J20-J22	8	1925	0.05951	0.143	0.0706	impact
J20-J22	8	1935	0.05951	0.143	0.0706	incidence
J20-J22	8	1935	0.05951	0.143	0.0706	impact
J20-J22	8	1945	0.05951	0.143	0.0706	incidence
J20-J22	8	1945	0.05951	0.143	0.0706	impact
J20-J22	8	1955	0.05951	0.143	0.0706	incidence
J20-J22	8	1955	0.05951	0.143	0.0706	impact
J20-J22	9	1915	0.06303	-0.165	0.0647	incidence
J20-J22	9	1915	0.06303	-0.165	0.0647	impact
J20-J22	9	1925	0.06303	-0.165	0.0647	incidence
J20-J22	9	1925	0.06303	-0.165	0.0647	impact
J20-J22	9	1935	0.06303	-0.165	0.0647	incidence
J20-J22	9	1935	0.06303	-0.165	0.0647	impact
J20-J22	9	1945	0.06303	-0.165	0.0647	incidence
J20-J22	9	1945	0.06303	-0.165	0.0647	impact
J20-J22	9	1955	0.06303	-0.165	0.0647	incidence
J20-J22	9	1955	0.06303	-0.165	0.0647	impact
J20-J22	10	1915	0.07659	-0.0308	0.1224	incidence
J20-J22	10	1915	0.07659	-0.0308	0.1224	impact
J20-J22	10	1925	0.07659	-0.0308	0.1224	incidence
J20-J22	10	1925	0.07659	-0.0308	0.1224	impact
J20-J22	10	1935	0.07659	-0.0308	0.1224	incidence
J20-J22	10	1935	0.07659	-0.0308	0.1224	impact
J20-J22	10	1945	0.07659	-0.0308	0.1224	incidence
J20-J22	10	1945	0.07659	-0.0308	0.1224	impact
J20-J22	10	1955	0.07659	-0.0308	0.1224	incidence
J20-J22	10	1955	0.07659	-0.0308	0.1224	impact
S70-S79	1	1915	0.03132	0.257	0.148	incidence
S70-S79	1	1915	0.03132	0.257	0.148	impact
S70-S79	1	1925	0.03132	0.257	0.148	incidence
S70-S79	1	1925	0.03132	0.257	0.148	impact
S70-S79	1	1935	0.03132	0.257	0.148	incidence
S70-S79	1	1935	0.03132	0.257	0.148	impact
S70-S79	1	1945	0.03132	0.257	0.148	incidence
S70-S79	1	1945	0.03132	0.257	0.148	impact

For peer review only

S70-S79	1	1955	0.03132	0.257	0.148	incidence
S70-S79	1	1955	0.03132	0.257	0.148	impact
S70-S79	2	1915	0.03294	-0.027	0.0959	incidence
S70-S79	2	1915	0.03294	-0.027	0.0959	impact
S70-S79	2	1925	0.03294	-0.027	0.0959	incidence
S70-S79	2	1925	0.03294	-0.027	0.0959	impact
S70-S79	2	1935	0.03294	-0.027	0.0959	incidence
S70-S79	2	1935	0.03294	-0.027	0.0959	impact
S70-S79	2	1945	0.03294	-0.027	0.0959	incidence
S70-S79	2	1945	0.03294	-0.027	0.0959	impact
S70-S79	2	1955	0.03294	-0.027	0.0959	incidence
S70-S79	2	1955	0.03294	-0.027	0.0959	impact
S70-S79	3	1915	0.03598	0.0994	0.1019	incidence
S70-S79	3	1915	0.03598	0.0994	0.1019	impact
S70-S79	3	1925	0.03598	0.0994	0.1019	incidence
S70-S79	3	1925	0.03598	0.0994	0.1019	impact
S70-S79	3	1935	0.03598	0.0994	0.1019	incidence
S70-S79	3	1935	0.03598	0.0994	0.1019	impact
S70-S79	3	1945	0.03598	0.0994	0.1019	incidence
S70-S79	3	1945	0.03598	0.0994	0.1019	impact
S70-S79	3	1955	0.03598	0.0994	0.1019	incidence
S70-S79	3	1955	0.03598	0.0994	0.1019	impact
S70-S79	4	1915	0.03695	-0.219	0.1176	incidence
S70-S79	4	1915	0.03695	-0.219	0.1176	impact
S70-S79	4	1925	0.03695	-0.219	0.1176	incidence
S70-S79	4	1925	0.03695	-0.219	0.1176	impact
S70-S79	4	1935	0.03695	-0.219	0.1176	incidence
S70-S79	4	1935	0.03695	-0.219	0.1176	impact
S70-S79	4	1945	0.03695	-0.219	0.1176	incidence
S70-S79	4	1945	0.03695	-0.219	0.1176	impact
S70-S79	4	1955	0.03695	-0.219	0.1176	incidence
S70-S79	4	1955	0.03695	-0.219	0.1176	impact
S70-S79	5	1915	0.03831	-0.0924	0.0706	incidence
S70-S79	5	1915	0.03831	-0.0924	0.0706	impact
S70-S79	5	1925	0.03831	-0.0924	0.0706	incidence
S70-S79	5	1925	0.03831	-0.0924	0.0706	impact
S70-S79	5	1935	0.03831	-0.0924	0.0706	incidence
S70-S79	5	1935	0.03831	-0.0924	0.0706	impact
S70-S79	5	1945	0.03831	-0.0924	0.0706	incidence
S70-S79	5	1945	0.03831	-0.0924	0.0706	impact
S70-S79	5	1955	0.03831	-0.0924	0.0706	incidence
S70-S79	5	1955	0.03831	-0.0924	0.0706	impact
S70-S79	6	1915	0.03745	-0.122	0.0613	incidence

For peer review only

S70-S79	6	1915	0.03745	-0.122	0.0613	impact
S70-S79	6	1925	0.03745	-0.122	0.0613	incidence
S70-S79	6	1925	0.03745	-0.122	0.0613	impact
S70-S79	6	1935	0.03745	-0.122	0.0613	incidence
S70-S79	6	1935	0.03745	-0.122	0.0613	impact
S70-S79	6	1945	0.03745	-0.122	0.0613	incidence
S70-S79	6	1945	0.03745	-0.122	0.0613	impact
S70-S79	6	1955	0.03745	-0.122	0.0613	incidence
S70-S79	6	1955	0.03745	-0.122	0.0613	impact
S70-S79	7	1915	0.04192	0.157	0.1052	incidence
S70-S79	7	1915	0.04192	0.157	0.1052	impact
S70-S79	7	1925	0.04192	0.157	0.1052	incidence
S70-S79	7	1925	0.04192	0.157	0.1052	impact
S70-S79	7	1935	0.04192	0.157	0.1052	incidence
S70-S79	7	1935	0.04192	0.157	0.1052	impact
S70-S79	7	1945	0.04192	0.157	0.1052	incidence
S70-S79	7	1945	0.04192	0.157	0.1052	impact
S70-S79	7	1955	0.04192	0.157	0.1052	incidence
S70-S79	7	1955	0.04192	0.157	0.1052	impact
S70-S79	8	1915	0.04218	-0.0917	0.1111	incidence
S70-S79	8	1915	0.04218	-0.0917	0.1111	impact
S70-S79	8	1925	0.04218	-0.0917	0.1111	incidence
S70-S79	8	1925	0.04218	-0.0917	0.1111	impact
S70-S79	8	1935	0.04218	-0.0917	0.1111	incidence
S70-S79	8	1935	0.04218	-0.0917	0.1111	impact
S70-S79	8	1945	0.04218	-0.0917	0.1111	incidence
S70-S79	8	1945	0.04218	-0.0917	0.1111	impact
S70-S79	8	1955	0.04218	-0.0917	0.1111	incidence
S70-S79	8	1955	0.04218	-0.0917	0.1111	impact
S70-S79	9	1915	0.04231	0.00191	0.1198	incidence
S70-S79	9	1915	0.04231	0.00191	0.1198	impact
S70-S79	9	1925	0.04231	0.00191	0.1198	incidence
S70-S79	9	1925	0.04231	0.00191	0.1198	impact
S70-S79	9	1935	0.04231	0.00191	0.1198	incidence
S70-S79	9	1935	0.04231	0.00191	0.1198	impact
S70-S79	9	1945	0.04231	0.00191	0.1198	incidence
S70-S79	9	1945	0.04231	0.00191	0.1198	impact
S70-S79	9	1955	0.04231	0.00191	0.1198	incidence
S70-S79	9	1955	0.04231	0.00191	0.1198	impact
S70-S79	10	1915	0.04409	-0.178	0.1147	incidence
S70-S79	10	1915	0.04409	-0.178	0.1147	impact
S70-S79	10	1925	0.04409	-0.178	0.1147	incidence
S70-S79	10	1925	0.04409	-0.178	0.1147	impact

For peer review only

S70-S79	10	1935	0.04409	-0.178	0.1147	incidence
S70-S79	10	1935	0.04409	-0.178	0.1147	impact
S70-S79	10	1945	0.04409	-0.178	0.1147	incidence
S70-S79	10	1945	0.04409	-0.178	0.1147	impact
S70-S79	10	1955	0.04409	-0.178	0.1147	incidence
S70-S79	10	1955	0.04409	-0.178	0.1147	impact
A30-A49	1	1915	0.02452	0.324	0.2369	incidence
A30-A49	1	1915	0.02452	0.324	0.2369	impact
A30-A49	1	1925	0.02452	0.324	0.2369	incidence
A30-A49	1	1925	0.02452	0.324	0.2369	impact
A30-A49	1	1935	0.02452	0.324	0.2369	incidence
A30-A49	1	1935	0.02452	0.324	0.2369	impact
A30-A49	1	1945	0.02452	0.324	0.2369	incidence
A30-A49	1	1945	0.02452	0.324	0.2369	impact
A30-A49	1	1955	0.02452	0.324	0.2369	incidence
A30-A49	1	1955	0.02452	0.324	0.2369	impact
A30-A49	2	1915	0.02561	-0.304	0.2255	incidence
A30-A49	2	1915	0.02561	-0.304	0.2255	impact
A30-A49	2	1925	0.02561	-0.304	0.2255	incidence
A30-A49	2	1925	0.02561	-0.304	0.2255	impact
A30-A49	2	1935	0.02561	-0.304	0.2255	incidence
A30-A49	2	1935	0.02561	-0.304	0.2255	impact
A30-A49	2	1945	0.02561	-0.304	0.2255	incidence
A30-A49	2	1945	0.02561	-0.304	0.2255	impact
A30-A49	2	1955	0.02561	-0.304	0.2255	incidence
A30-A49	2	1955	0.02561	-0.304	0.2255	impact
A30-A49	3	1915	0.02707	-0.328	0.2641	incidence
A30-A49	3	1915	0.02707	-0.328	0.2641	impact
A30-A49	3	1925	0.02707	-0.328	0.2641	incidence
A30-A49	3	1925	0.02707	-0.328	0.2641	impact
A30-A49	3	1935	0.02707	-0.328	0.2641	incidence
A30-A49	3	1935	0.02707	-0.328	0.2641	impact
A30-A49	3	1945	0.02707	-0.328	0.2641	incidence
A30-A49	3	1945	0.02707	-0.328	0.2641	impact
A30-A49	3	1955	0.02707	-0.328	0.2641	incidence
A30-A49	3	1955	0.02707	-0.328	0.2641	impact
A30-A49	4	1915	0.02927	-0.286	0.1399	incidence
A30-A49	4	1915	0.02927	-0.286	0.1399	impact
A30-A49	4	1925	0.02927	-0.286	0.1399	incidence
A30-A49	4	1925	0.02927	-0.286	0.1399	impact
A30-A49	4	1935	0.02927	-0.286	0.1399	incidence
A30-A49	4	1935	0.02927	-0.286	0.1399	impact
A30-A49	4	1945	0.02927	-0.286	0.1399	incidence

For peer review only

A30-A49	4	1945	0.02927	-0.286	0.1399	impact
A30-A49	4	1955	0.02927	-0.286	0.1399	incidence
A30-A49	4	1955	0.02927	-0.286	0.1399	impact
A30-A49	5	1915	0.03087	0.423	0.2242	incidence
A30-A49	5	1915	0.03087	0.423	0.2242	impact
A30-A49	5	1925	0.03087	0.423	0.2242	incidence
A30-A49	5	1925	0.03087	0.423	0.2242	impact
A30-A49	5	1935	0.03087	0.423	0.2242	incidence
A30-A49	5	1935	0.03087	0.423	0.2242	impact
A30-A49	5	1945	0.03087	0.423	0.2242	incidence
A30-A49	5	1945	0.03087	0.423	0.2242	impact
A30-A49	5	1955	0.03087	0.423	0.2242	incidence
A30-A49	5	1955	0.03087	0.423	0.2242	impact
A30-A49	6	1915	0.03366	-0.608	0.1778	incidence
A30-A49	6	1915	0.03366	-0.608	0.1778	impact
A30-A49	6	1925	0.03366	-0.608	0.1778	incidence
A30-A49	6	1925	0.03366	-0.608	0.1778	impact
A30-A49	6	1935	0.03366	-0.608	0.1778	incidence
A30-A49	6	1935	0.03366	-0.608	0.1778	impact
A30-A49	6	1945	0.03366	-0.608	0.1778	incidence
A30-A49	6	1945	0.03366	-0.608	0.1778	impact
A30-A49	6	1955	0.03366	-0.608	0.1778	incidence
A30-A49	6	1955	0.03366	-0.608	0.1778	impact
A30-A49	7	1915	0.03746	0.531	0.3313	incidence
A30-A49	7	1915	0.03746	0.531	0.3313	impact
A30-A49	7	1925	0.03746	0.531	0.3313	incidence
A30-A49	7	1925	0.03746	0.531	0.3313	impact
A30-A49	7	1935	0.03746	0.531	0.3313	incidence
A30-A49	7	1935	0.03746	0.531	0.3313	impact
A30-A49	7	1945	0.03746	0.531	0.3313	incidence
A30-A49	7	1945	0.03746	0.531	0.3313	impact
A30-A49	7	1955	0.03746	0.531	0.3313	incidence
A30-A49	7	1955	0.03746	0.531	0.3313	impact
A30-A49	8	1915	0.03675	0.366	0.1353	incidence
A30-A49	8	1915	0.03675	0.366	0.1353	impact
A30-A49	8	1925	0.03675	0.366	0.1353	incidence
A30-A49	8	1925	0.03675	0.366	0.1353	impact
A30-A49	8	1935	0.03675	0.366	0.1353	incidence
A30-A49	8	1935	0.03675	0.366	0.1353	impact
A30-A49	8	1945	0.03675	0.366	0.1353	incidence
A30-A49	8	1945	0.03675	0.366	0.1353	impact
A30-A49	8	1955	0.03675	0.366	0.1353	incidence
A30-A49	8	1955	0.03675	0.366	0.1353	impact

For peer review only

A30-A49	9	1915	0.03863	0.134	0.1894	incidence
A30-A49	9	1915	0.03863	0.134	0.1894	impact
A30-A49	9	1925	0.03863	0.134	0.1894	incidence
A30-A49	9	1925	0.03863	0.134	0.1894	impact
A30-A49	9	1935	0.03863	0.134	0.1894	incidence
A30-A49	9	1935	0.03863	0.134	0.1894	impact
A30-A49	9	1945	0.03863	0.134	0.1894	incidence
A30-A49	9	1945	0.03863	0.134	0.1894	impact
A30-A49	9	1955	0.03863	0.134	0.1894	incidence
A30-A49	9	1955	0.03863	0.134	0.1894	impact
A30-A49	10	1915	0.03867	-0.218	0.1681	incidence
A30-A49	10	1915	0.03867	-0.218	0.1681	impact
A30-A49	10	1925	0.03867	-0.218	0.1681	incidence
A30-A49	10	1925	0.03867	-0.218	0.1681	impact
A30-A49	10	1935	0.03867	-0.218	0.1681	incidence
A30-A49	10	1935	0.03867	-0.218	0.1681	impact
A30-A49	10	1945	0.03867	-0.218	0.1681	incidence
A30-A49	10	1945	0.03867	-0.218	0.1681	impact
A30-A49	10	1955	0.03867	-0.218	0.1681	incidence
A30-A49	10	1955	0.03867	-0.218	0.1681	impact
T80-T88	1	1915	0.02975	0.189	0.248	incidence
T80-T88	1	1915	0.02975	0.189	0.248	impact
T80-T88	1	1925	0.02975	0.189	0.248	incidence
T80-T88	1	1925	0.02975	0.189	0.248	impact
T80-T88	1	1935	0.02975	0.189	0.248	incidence
T80-T88	1	1935	0.02975	0.189	0.248	impact
T80-T88	1	1945	0.02975	0.189	0.248	incidence
T80-T88	1	1945	0.02975	0.189	0.248	impact
T80-T88	1	1955	0.02975	0.189	0.248	incidence
T80-T88	1	1955	0.02975	0.189	0.248	impact
T80-T88	2	1915	0.03112	0.0583	0.1508	incidence
T80-T88	2	1915	0.03112	0.0583	0.1508	impact
T80-T88	2	1925	0.03112	0.0583	0.1508	incidence
T80-T88	2	1925	0.03112	0.0583	0.1508	impact
T80-T88	2	1935	0.03112	0.0583	0.1508	incidence
T80-T88	2	1935	0.03112	0.0583	0.1508	impact
T80-T88	2	1945	0.03112	0.0583	0.1508	incidence
T80-T88	2	1945	0.03112	0.0583	0.1508	impact
T80-T88	2	1955	0.03112	0.0583	0.1508	incidence
T80-T88	2	1955	0.03112	0.0583	0.1508	impact
T80-T88	3	1915	0.03032	0.0515	0.0582	incidence
T80-T88	3	1915	0.03032	0.0515	0.0582	impact
T80-T88	3	1925	0.03032	0.0515	0.0582	incidence

For peer review only

T80-T88	3	1925	0.03032	0.0515	0.0582	impact
T80-T88	3	1935	0.03032	0.0515	0.0582	incidence
T80-T88	3	1935	0.03032	0.0515	0.0582	impact
T80-T88	3	1945	0.03032	0.0515	0.0582	incidence
T80-T88	3	1945	0.03032	0.0515	0.0582	impact
T80-T88	3	1955	0.03032	0.0515	0.0582	incidence
T80-T88	3	1955	0.03032	0.0515	0.0582	impact
T80-T88	4	1915	0.03201	0.201	0.1644	incidence
T80-T88	4	1915	0.03201	0.201	0.1644	impact
T80-T88	4	1925	0.03201	0.201	0.1644	incidence
T80-T88	4	1925	0.03201	0.201	0.1644	impact
T80-T88	4	1935	0.03201	0.201	0.1644	incidence
T80-T88	4	1935	0.03201	0.201	0.1644	impact
T80-T88	4	1945	0.03201	0.201	0.1644	incidence
T80-T88	4	1945	0.03201	0.201	0.1644	impact
T80-T88	4	1955	0.03201	0.201	0.1644	incidence
T80-T88	4	1955	0.03201	0.201	0.1644	impact
T80-T88	5	1915	0.0326	0.172	0.0617	incidence
T80-T88	5	1915	0.0326	0.172	0.0617	impact
T80-T88	5	1925	0.0326	0.172	0.0617	incidence
T80-T88	5	1925	0.0326	0.172	0.0617	impact
T80-T88	5	1935	0.0326	0.172	0.0617	incidence
T80-T88	5	1935	0.0326	0.172	0.0617	impact
T80-T88	5	1945	0.0326	0.172	0.0617	incidence
T80-T88	5	1945	0.0326	0.172	0.0617	impact
T80-T88	5	1955	0.0326	0.172	0.0617	incidence
T80-T88	5	1955	0.0326	0.172	0.0617	impact
T80-T88	6	1915	0.03287	0.434	0.1027	incidence
T80-T88	6	1915	0.03287	0.434	0.1027	impact
T80-T88	6	1925	0.03287	0.434	0.1027	incidence
T80-T88	6	1925	0.03287	0.434	0.1027	impact
T80-T88	6	1935	0.03287	0.434	0.1027	incidence
T80-T88	6	1935	0.03287	0.434	0.1027	impact
T80-T88	6	1945	0.03287	0.434	0.1027	incidence
T80-T88	6	1945	0.03287	0.434	0.1027	impact
T80-T88	6	1955	0.03287	0.434	0.1027	incidence
T80-T88	6	1955	0.03287	0.434	0.1027	impact
T80-T88	7	1915	0.03332	0.198	0.1504	incidence
T80-T88	7	1915	0.03332	0.198	0.1504	impact
T80-T88	7	1925	0.03332	0.198	0.1504	incidence
T80-T88	7	1925	0.03332	0.198	0.1504	impact
T80-T88	7	1935	0.03332	0.198	0.1504	incidence
T80-T88	7	1935	0.03332	0.198	0.1504	impact

For peer review only

T80-T88	7	1945	0.03332	0.198	0.1504	incidence
T80-T88	7	1945	0.03332	0.198	0.1504	impact
T80-T88	7	1955	0.03332	0.198	0.1504	incidence
T80-T88	7	1955	0.03332	0.198	0.1504	impact
T80-T88	8	1915	0.03245	0.261	0.1197	incidence
T80-T88	8	1915	0.03245	0.261	0.1197	impact
T80-T88	8	1925	0.03245	0.261	0.1197	incidence
T80-T88	8	1925	0.03245	0.261	0.1197	impact
T80-T88	8	1935	0.03245	0.261	0.1197	incidence
T80-T88	8	1935	0.03245	0.261	0.1197	impact
T80-T88	8	1945	0.03245	0.261	0.1197	incidence
T80-T88	8	1945	0.03245	0.261	0.1197	impact
T80-T88	8	1955	0.03245	0.261	0.1197	incidence
T80-T88	8	1955	0.03245	0.261	0.1197	impact
T80-T88	9	1915	0.03284	2.14E-05	0.1148	incidence
T80-T88	9	1915	0.03284	2.14E-05	0.1148	impact
T80-T88	9	1925	0.03284	2.14E-05	0.1148	incidence
T80-T88	9	1925	0.03284	2.14E-05	0.1148	impact
T80-T88	9	1935	0.03284	2.14E-05	0.1148	incidence
T80-T88	9	1935	0.03284	2.14E-05	0.1148	impact
T80-T88	9	1945	0.03284	2.14E-05	0.1148	incidence
T80-T88	9	1945	0.03284	2.14E-05	0.1148	impact
T80-T88	9	1955	0.03284	2.14E-05	0.1148	incidence
T80-T88	9	1955	0.03284	2.14E-05	0.1148	impact
T80-T88	10	1915	0.03329	0.204	0.0876	incidence
T80-T88	10	1915	0.03329	0.204	0.0876	impact
T80-T88	10	1925	0.03329	0.204	0.0876	incidence
T80-T88	10	1925	0.03329	0.204	0.0876	impact
T80-T88	10	1935	0.03329	0.204	0.0876	incidence
T80-T88	10	1935	0.03329	0.204	0.0876	impact
T80-T88	10	1945	0.03329	0.204	0.0876	incidence
T80-T88	10	1945	0.03329	0.204	0.0876	impact
T80-T88	10	1955	0.03329	0.204	0.0876	incidence
T80-T88	10	1955	0.03329	0.204	0.0876	impact
N17-N19	1	1915	0.02326	0.0207	0.2501	incidence
N17-N19	1	1915	0.02326	0.0207	0.2501	impact
N17-N19	1	1925	0.02326	0.0207	0.2501	incidence
N17-N19	1	1925	0.02326	0.0207	0.2501	impact
N17-N19	1	1935	0.02326	0.0207	0.2501	incidence
N17-N19	1	1935	0.02326	0.0207	0.2501	impact
N17-N19	1	1945	0.02326	0.0207	0.2501	incidence
N17-N19	1	1945	0.02326	0.0207	0.2501	impact
N17-N19	1	1955	0.02326	0.0207	0.2501	incidence

For peer review only

N17-N19	1	1955	0.02326	0.0207	0.2501	impact
N17-N19	2	1915	0.02443	-0.0883	0.2134	incidence
N17-N19	2	1915	0.02443	-0.0883	0.2134	impact
N17-N19	2	1925	0.02443	-0.0883	0.2134	incidence
N17-N19	2	1925	0.02443	-0.0883	0.2134	impact
N17-N19	2	1935	0.02443	-0.0883	0.2134	incidence
N17-N19	2	1935	0.02443	-0.0883	0.2134	impact
N17-N19	2	1945	0.02443	-0.0883	0.2134	incidence
N17-N19	2	1945	0.02443	-0.0883	0.2134	impact
N17-N19	2	1955	0.02443	-0.0883	0.2134	incidence
N17-N19	2	1955	0.02443	-0.0883	0.2134	impact
N17-N19	3	1915	0.02593	0.231	0.1435	incidence
N17-N19	3	1915	0.02593	0.231	0.1435	impact
N17-N19	3	1925	0.02593	0.231	0.1435	incidence
N17-N19	3	1925	0.02593	0.231	0.1435	impact
N17-N19	3	1935	0.02593	0.231	0.1435	incidence
N17-N19	3	1935	0.02593	0.231	0.1435	impact
N17-N19	3	1945	0.02593	0.231	0.1435	incidence
N17-N19	3	1945	0.02593	0.231	0.1435	impact
N17-N19	3	1955	0.02593	0.231	0.1435	incidence
N17-N19	3	1955	0.02593	0.231	0.1435	impact
N17-N19	4	1915	0.02765	0.00575	0.064	incidence
N17-N19	4	1915	0.02765	0.00575	0.064	impact
N17-N19	4	1925	0.02765	0.00575	0.064	incidence
N17-N19	4	1925	0.02765	0.00575	0.064	impact
N17-N19	4	1935	0.02765	0.00575	0.064	incidence
N17-N19	4	1935	0.02765	0.00575	0.064	impact
N17-N19	4	1945	0.02765	0.00575	0.064	incidence
N17-N19	4	1945	0.02765	0.00575	0.064	impact
N17-N19	4	1955	0.02765	0.00575	0.064	incidence
N17-N19	4	1955	0.02765	0.00575	0.064	impact
N17-N19	5	1915	0.02914	0.534	0.2105	incidence
N17-N19	5	1915	0.02914	0.534	0.2105	impact
N17-N19	5	1925	0.02914	0.534	0.2105	incidence
N17-N19	5	1925	0.02914	0.534	0.2105	impact
N17-N19	5	1935	0.02914	0.534	0.2105	incidence
N17-N19	5	1935	0.02914	0.534	0.2105	impact
N17-N19	5	1945	0.02914	0.534	0.2105	incidence
N17-N19	5	1945	0.02914	0.534	0.2105	impact
N17-N19	5	1955	0.02914	0.534	0.2105	incidence
N17-N19	5	1955	0.02914	0.534	0.2105	impact
N17-N19	6	1915	0.03191	0.176	0.1943	incidence
N17-N19	6	1915	0.03191	0.176	0.1943	impact

For peer review only

N17-N19	6	1925	0.03191	0.176	0.1943	incidence
N17-N19	6	1925	0.03191	0.176	0.1943	impact
N17-N19	6	1935	0.03191	0.176	0.1943	incidence
N17-N19	6	1935	0.03191	0.176	0.1943	impact
N17-N19	6	1945	0.03191	0.176	0.1943	incidence
N17-N19	6	1945	0.03191	0.176	0.1943	impact
N17-N19	6	1955	0.03191	0.176	0.1943	incidence
N17-N19	6	1955	0.03191	0.176	0.1943	impact
N17-N19	7	1915	0.03594	0.196	0.1471	incidence
N17-N19	7	1915	0.03594	0.196	0.1471	impact
N17-N19	7	1925	0.03594	0.196	0.1471	incidence
N17-N19	7	1925	0.03594	0.196	0.1471	impact
N17-N19	7	1935	0.03594	0.196	0.1471	incidence
N17-N19	7	1935	0.03594	0.196	0.1471	impact
N17-N19	7	1945	0.03594	0.196	0.1471	incidence
N17-N19	7	1945	0.03594	0.196	0.1471	impact
N17-N19	7	1955	0.03594	0.196	0.1471	incidence
N17-N19	7	1955	0.03594	0.196	0.1471	impact
N17-N19	8	1915	0.03673	0.114	0.1066	incidence
N17-N19	8	1915	0.03673	0.114	0.1066	impact
N17-N19	8	1925	0.03673	0.114	0.1066	incidence
N17-N19	8	1925	0.03673	0.114	0.1066	impact
N17-N19	8	1935	0.03673	0.114	0.1066	incidence
N17-N19	8	1935	0.03673	0.114	0.1066	impact
N17-N19	8	1945	0.03673	0.114	0.1066	incidence
N17-N19	8	1945	0.03673	0.114	0.1066	impact
N17-N19	8	1955	0.03673	0.114	0.1066	incidence
N17-N19	8	1955	0.03673	0.114	0.1066	impact
N17-N19	9	1915	0.03657	0.428	0.1679	incidence
N17-N19	9	1915	0.03657	0.428	0.1679	impact
N17-N19	9	1925	0.03657	0.428	0.1679	incidence
N17-N19	9	1925	0.03657	0.428	0.1679	impact
N17-N19	9	1935	0.03657	0.428	0.1679	incidence
N17-N19	9	1935	0.03657	0.428	0.1679	impact
N17-N19	9	1945	0.03657	0.428	0.1679	incidence
N17-N19	9	1945	0.03657	0.428	0.1679	impact
N17-N19	9	1955	0.03657	0.428	0.1679	incidence
N17-N19	9	1955	0.03657	0.428	0.1679	impact
N17-N19	10	1915	0.03761	0.151	0.1776	incidence
N17-N19	10	1915	0.03761	0.151	0.1776	impact
N17-N19	10	1925	0.03761	0.151	0.1776	incidence
N17-N19	10	1925	0.03761	0.151	0.1776	impact
N17-N19	10	1935	0.03761	0.151	0.1776	incidence

For peer review only

N17-N19	10	1935	0.03761	0.151	0.1776	impact
N17-N19	10	1945	0.03761	0.151	0.1776	incidence
N17-N19	10	1945	0.03761	0.151	0.1776	impact
N17-N19	10	1955	0.03761	0.151	0.1776	incidence
N17-N19	10	1955	0.03761	0.151	0.1776	impact
I80-I89	1	1915	0.03016	0.415	0.2227	incidence
I80-I89	1	1915	0.03016	0.415	0.2227	impact
I80-I89	1	1925	0.03016	0.415	0.2227	incidence
I80-I89	1	1925	0.03016	0.415	0.2227	impact
I80-I89	1	1935	0.03016	0.415	0.2227	incidence
I80-I89	1	1935	0.03016	0.415	0.2227	impact
I80-I89	1	1945	0.03016	0.415	0.2227	incidence
I80-I89	1	1945	0.03016	0.415	0.2227	impact
I80-I89	1	1955	0.03016	0.415	0.2227	incidence
I80-I89	1	1955	0.03016	0.415	0.2227	impact
I80-I89	2	1915	0.02989	0.0507	0.2263	incidence
I80-I89	2	1915	0.02989	0.0507	0.2263	impact
I80-I89	2	1925	0.02989	0.0507	0.2263	incidence
I80-I89	2	1925	0.02989	0.0507	0.2263	impact
I80-I89	2	1935	0.02989	0.0507	0.2263	incidence
I80-I89	2	1935	0.02989	0.0507	0.2263	impact
I80-I89	2	1945	0.02989	0.0507	0.2263	incidence
I80-I89	2	1945	0.02989	0.0507	0.2263	impact
I80-I89	2	1955	0.02989	0.0507	0.2263	incidence
I80-I89	2	1955	0.02989	0.0507	0.2263	impact
I80-I89	3	1915	0.02537	0.101	0.1917	incidence
I80-I89	3	1915	0.02537	0.101	0.1917	impact
I80-I89	3	1925	0.02537	0.101	0.1917	incidence
I80-I89	3	1925	0.02537	0.101	0.1917	impact
I80-I89	3	1935	0.02537	0.101	0.1917	incidence
I80-I89	3	1935	0.02537	0.101	0.1917	impact
I80-I89	3	1945	0.02537	0.101	0.1917	incidence
I80-I89	3	1945	0.02537	0.101	0.1917	impact
I80-I89	3	1955	0.02537	0.101	0.1917	incidence
I80-I89	3	1955	0.02537	0.101	0.1917	impact
I80-I89	4	1915	0.02707	0.617	0.3081	incidence
I80-I89	4	1915	0.02707	0.617	0.3081	impact
I80-I89	4	1925	0.02707	0.617	0.3081	incidence
I80-I89	4	1925	0.02707	0.617	0.3081	impact
I80-I89	4	1935	0.02707	0.617	0.3081	incidence
I80-I89	4	1935	0.02707	0.617	0.3081	impact
I80-I89	4	1945	0.02707	0.617	0.3081	incidence
I80-I89	4	1945	0.02707	0.617	0.3081	impact

For peer review only

I80-I89	4	1955	0.02707	0.617	0.3081	incidence
I80-I89	4	1955	0.02707	0.617	0.3081	impact
I80-I89	5	1915	0.02685	0.213	0.1902	incidence
I80-I89	5	1915	0.02685	0.213	0.1902	impact
I80-I89	5	1925	0.02685	0.213	0.1902	incidence
I80-I89	5	1925	0.02685	0.213	0.1902	impact
I80-I89	5	1935	0.02685	0.213	0.1902	incidence
I80-I89	5	1935	0.02685	0.213	0.1902	impact
I80-I89	5	1945	0.02685	0.213	0.1902	incidence
I80-I89	5	1945	0.02685	0.213	0.1902	impact
I80-I89	5	1955	0.02685	0.213	0.1902	incidence
I80-I89	5	1955	0.02685	0.213	0.1902	impact
I80-I89	6	1915	0.0274	0.0794	0.1808	incidence
I80-I89	6	1915	0.0274	0.0794	0.1808	impact
I80-I89	6	1925	0.0274	0.0794	0.1808	incidence
I80-I89	6	1925	0.0274	0.0794	0.1808	impact
I80-I89	6	1935	0.0274	0.0794	0.1808	incidence
I80-I89	6	1935	0.0274	0.0794	0.1808	impact
I80-I89	6	1945	0.0274	0.0794	0.1808	incidence
I80-I89	6	1945	0.0274	0.0794	0.1808	impact
I80-I89	6	1955	0.0274	0.0794	0.1808	incidence
I80-I89	6	1955	0.0274	0.0794	0.1808	impact
I80-I89	7	1915	0.02688	0.489	0.1499	incidence
I80-I89	7	1915	0.02688	0.489	0.1499	impact
I80-I89	7	1925	0.02688	0.489	0.1499	incidence
I80-I89	7	1925	0.02688	0.489	0.1499	impact
I80-I89	7	1935	0.02688	0.489	0.1499	incidence
I80-I89	7	1935	0.02688	0.489	0.1499	impact
I80-I89	7	1945	0.02688	0.489	0.1499	incidence
I80-I89	7	1945	0.02688	0.489	0.1499	impact
I80-I89	7	1955	0.02688	0.489	0.1499	incidence
I80-I89	7	1955	0.02688	0.489	0.1499	impact
I80-I89	8	1915	0.02879	0.284	0.1301	incidence
I80-I89	8	1915	0.02879	0.284	0.1301	impact
I80-I89	8	1925	0.02879	0.284	0.1301	incidence
I80-I89	8	1925	0.02879	0.284	0.1301	impact
I80-I89	8	1935	0.02879	0.284	0.1301	incidence
I80-I89	8	1935	0.02879	0.284	0.1301	impact
I80-I89	8	1945	0.02879	0.284	0.1301	incidence
I80-I89	8	1945	0.02879	0.284	0.1301	impact
I80-I89	8	1955	0.02879	0.284	0.1301	incidence
I80-I89	8	1955	0.02879	0.284	0.1301	impact
I80-I89	9	1915	0.02884	0.057	0.1173	incidence

For peer review only

I80-I89	9	1915	0.02884	0.057	0.1173	impact
I80-I89	9	1925	0.02884	0.057	0.1173	incidence
I80-I89	9	1925	0.02884	0.057	0.1173	impact
I80-I89	9	1935	0.02884	0.057	0.1173	incidence
I80-I89	9	1935	0.02884	0.057	0.1173	impact
I80-I89	9	1945	0.02884	0.057	0.1173	incidence
I80-I89	9	1945	0.02884	0.057	0.1173	impact
I80-I89	9	1955	0.02884	0.057	0.1173	incidence
I80-I89	9	1955	0.02884	0.057	0.1173	impact
I80-I89	10	1915	0.0327	0.138	0.1618	incidence
I80-I89	10	1915	0.0327	0.138	0.1618	impact
I80-I89	10	1925	0.0327	0.138	0.1618	incidence
I80-I89	10	1925	0.0327	0.138	0.1618	impact
I80-I89	10	1935	0.0327	0.138	0.1618	incidence
I80-I89	10	1935	0.0327	0.138	0.1618	impact
I80-I89	10	1945	0.0327	0.138	0.1618	incidence
I80-I89	10	1945	0.0327	0.138	0.1618	impact
I80-I89	10	1955	0.0327	0.138	0.1618	incidence
I80-I89	10	1955	0.0327	0.138	0.1618	impact
K90-K93	1	1915	0.01809	-0.348	0.1264	incidence
K90-K93	1	1915	0.01809	-0.348	0.1264	impact
K90-K93	1	1925	0.01809	-0.348	0.1264	incidence
K90-K93	1	1925	0.01809	-0.348	0.1264	impact
K90-K93	1	1935	0.01809	-0.348	0.1264	incidence
K90-K93	1	1935	0.01809	-0.348	0.1264	impact
K90-K93	1	1945	0.01809	-0.348	0.1264	incidence
K90-K93	1	1945	0.01809	-0.348	0.1264	impact
K90-K93	1	1955	0.01809	-0.348	0.1264	incidence
K90-K93	1	1955	0.01809	-0.348	0.1264	impact
K90-K93	2	1915	0.01987	-0.0753	0.2466	incidence
K90-K93	2	1915	0.01987	-0.0753	0.2466	impact
K90-K93	2	1925	0.01987	-0.0753	0.2466	incidence
K90-K93	2	1925	0.01987	-0.0753	0.2466	impact
K90-K93	2	1935	0.01987	-0.0753	0.2466	incidence
K90-K93	2	1935	0.01987	-0.0753	0.2466	impact
K90-K93	2	1945	0.01987	-0.0753	0.2466	incidence
K90-K93	2	1945	0.01987	-0.0753	0.2466	impact
K90-K93	2	1955	0.01987	-0.0753	0.2466	incidence
K90-K93	2	1955	0.01987	-0.0753	0.2466	impact
K90-K93	3	1915	0.01998	-0.164	0.2035	incidence
K90-K93	3	1915	0.01998	-0.164	0.2035	impact
K90-K93	3	1925	0.01998	-0.164	0.2035	incidence
K90-K93	3	1925	0.01998	-0.164	0.2035	impact

For peer review only

K90-K93	3	1935	0.01998	-0.164	0.2035	incidence
K90-K93	3	1935	0.01998	-0.164	0.2035	impact
K90-K93	3	1945	0.01998	-0.164	0.2035	incidence
K90-K93	3	1945	0.01998	-0.164	0.2035	impact
K90-K93	3	1955	0.01998	-0.164	0.2035	incidence
K90-K93	3	1955	0.01998	-0.164	0.2035	impact
K90-K93	4	1915	0.02234	0.0764	0.1642	incidence
K90-K93	4	1915	0.02234	0.0764	0.1642	impact
K90-K93	4	1925	0.02234	0.0764	0.1642	incidence
K90-K93	4	1925	0.02234	0.0764	0.1642	impact
K90-K93	4	1935	0.02234	0.0764	0.1642	incidence
K90-K93	4	1935	0.02234	0.0764	0.1642	impact
K90-K93	4	1945	0.02234	0.0764	0.1642	incidence
K90-K93	4	1945	0.02234	0.0764	0.1642	impact
K90-K93	4	1955	0.02234	0.0764	0.1642	incidence
K90-K93	4	1955	0.02234	0.0764	0.1642	impact
K90-K93	5	1915	0.02331	0.0467	0.1623	incidence
K90-K93	5	1915	0.02331	0.0467	0.1623	impact
K90-K93	5	1925	0.02331	0.0467	0.1623	incidence
K90-K93	5	1925	0.02331	0.0467	0.1623	impact
K90-K93	5	1935	0.02331	0.0467	0.1623	incidence
K90-K93	5	1935	0.02331	0.0467	0.1623	impact
K90-K93	5	1945	0.02331	0.0467	0.1623	incidence
K90-K93	5	1945	0.02331	0.0467	0.1623	impact
K90-K93	5	1955	0.02331	0.0467	0.1623	incidence
K90-K93	5	1955	0.02331	0.0467	0.1623	impact
K90-K93	6	1915	0.02622	0.253	0.1297	incidence
K90-K93	6	1915	0.02622	0.253	0.1297	impact
K90-K93	6	1925	0.02622	0.253	0.1297	incidence
K90-K93	6	1925	0.02622	0.253	0.1297	impact
K90-K93	6	1935	0.02622	0.253	0.1297	incidence
K90-K93	6	1935	0.02622	0.253	0.1297	impact
K90-K93	6	1945	0.02622	0.253	0.1297	incidence
K90-K93	6	1945	0.02622	0.253	0.1297	impact
K90-K93	6	1955	0.02622	0.253	0.1297	incidence
K90-K93	6	1955	0.02622	0.253	0.1297	impact
K90-K93	7	1915	0.02917	0.16	0.0941	incidence
K90-K93	7	1915	0.02917	0.16	0.0941	impact
K90-K93	7	1925	0.02917	0.16	0.0941	incidence
K90-K93	7	1925	0.02917	0.16	0.0941	impact
K90-K93	7	1935	0.02917	0.16	0.0941	incidence
K90-K93	7	1935	0.02917	0.16	0.0941	impact
K90-K93	7	1945	0.02917	0.16	0.0941	incidence

For peer review only

K90-K93	7	1945	0.02917	0.16	0.0941	impact
K90-K93	7	1955	0.02917	0.16	0.0941	incidence
K90-K93	7	1955	0.02917	0.16	0.0941	impact
K90-K93	8	1915	0.02958	0.212	0.1361	incidence
K90-K93	8	1915	0.02958	0.212	0.1361	impact
K90-K93	8	1925	0.02958	0.212	0.1361	incidence
K90-K93	8	1925	0.02958	0.212	0.1361	impact
K90-K93	8	1935	0.02958	0.212	0.1361	incidence
K90-K93	8	1935	0.02958	0.212	0.1361	impact
K90-K93	8	1945	0.02958	0.212	0.1361	incidence
K90-K93	8	1945	0.02958	0.212	0.1361	impact
K90-K93	8	1955	0.02958	0.212	0.1361	incidence
K90-K93	8	1955	0.02958	0.212	0.1361	impact
K90-K93	9	1915	0.03216	0.26	0.1528	incidence
K90-K93	9	1915	0.03216	0.26	0.1528	impact
K90-K93	9	1925	0.03216	0.26	0.1528	incidence
K90-K93	9	1925	0.03216	0.26	0.1528	impact
K90-K93	9	1935	0.03216	0.26	0.1528	incidence
K90-K93	9	1935	0.03216	0.26	0.1528	impact
K90-K93	9	1945	0.03216	0.26	0.1528	incidence
K90-K93	9	1945	0.03216	0.26	0.1528	impact
K90-K93	9	1955	0.03216	0.26	0.1528	incidence
K90-K93	9	1955	0.03216	0.26	0.1528	impact
K90-K93	10	1915	0.03873	0.631	0.1607	incidence
K90-K93	10	1915	0.03873	0.631	0.1607	impact
K90-K93	10	1925	0.03873	0.631	0.1607	incidence
K90-K93	10	1925	0.03873	0.631	0.1607	impact
K90-K93	10	1935	0.03873	0.631	0.1607	incidence
K90-K93	10	1935	0.03873	0.631	0.1607	impact
K90-K93	10	1945	0.03873	0.631	0.1607	incidence
K90-K93	10	1945	0.03873	0.631	0.1607	impact
K90-K93	10	1955	0.03873	0.631	0.1607	incidence
K90-K93	10	1955	0.03873	0.631	0.1607	impact
I70-I79	1	1915	0.01949	0.426	0.2025	incidence
I70-I79	1	1915	0.01949	0.426	0.2025	impact
I70-I79	1	1925	0.01949	0.426	0.2025	incidence
I70-I79	1	1925	0.01949	0.426	0.2025	impact
I70-I79	1	1935	0.01949	0.426	0.2025	incidence
I70-I79	1	1935	0.01949	0.426	0.2025	impact
I70-I79	1	1945	0.01949	0.426	0.2025	incidence
I70-I79	1	1945	0.01949	0.426	0.2025	impact
I70-I79	1	1955	0.01949	0.426	0.2025	incidence
I70-I79	1	1955	0.01949	0.426	0.2025	impact

For peer review only

I70-I79	2	1915	0.01936	-0.11	0.124	incidence
I70-I79	2	1915	0.01936	-0.11	0.124	impact
I70-I79	2	1925	0.01936	-0.11	0.124	incidence
I70-I79	2	1925	0.01936	-0.11	0.124	impact
I70-I79	2	1935	0.01936	-0.11	0.124	incidence
I70-I79	2	1935	0.01936	-0.11	0.124	impact
I70-I79	2	1945	0.01936	-0.11	0.124	incidence
I70-I79	2	1945	0.01936	-0.11	0.124	impact
I70-I79	2	1955	0.01936	-0.11	0.124	incidence
I70-I79	2	1955	0.01936	-0.11	0.124	impact
I70-I79	3	1915	0.02112	0.088	0.1238	incidence
I70-I79	3	1915	0.02112	0.088	0.1238	impact
I70-I79	3	1925	0.02112	0.088	0.1238	incidence
I70-I79	3	1925	0.02112	0.088	0.1238	impact
I70-I79	3	1935	0.02112	0.088	0.1238	incidence
I70-I79	3	1935	0.02112	0.088	0.1238	impact
I70-I79	3	1945	0.02112	0.088	0.1238	incidence
I70-I79	3	1945	0.02112	0.088	0.1238	impact
I70-I79	3	1955	0.02112	0.088	0.1238	incidence
I70-I79	3	1955	0.02112	0.088	0.1238	impact
I70-I79	4	1915	0.02318	0.257	0.1528	incidence
I70-I79	4	1915	0.02318	0.257	0.1528	impact
I70-I79	4	1925	0.02318	0.257	0.1528	incidence
I70-I79	4	1925	0.02318	0.257	0.1528	impact
I70-I79	4	1935	0.02318	0.257	0.1528	incidence
I70-I79	4	1935	0.02318	0.257	0.1528	impact
I70-I79	4	1945	0.02318	0.257	0.1528	incidence
I70-I79	4	1945	0.02318	0.257	0.1528	impact
I70-I79	4	1955	0.02318	0.257	0.1528	incidence
I70-I79	4	1955	0.02318	0.257	0.1528	impact
I70-I79	5	1915	0.02498	0.261	0.1831	incidence
I70-I79	5	1915	0.02498	0.261	0.1831	impact
I70-I79	5	1925	0.02498	0.261	0.1831	incidence
I70-I79	5	1925	0.02498	0.261	0.1831	impact
I70-I79	5	1935	0.02498	0.261	0.1831	incidence
I70-I79	5	1935	0.02498	0.261	0.1831	impact
I70-I79	5	1945	0.02498	0.261	0.1831	incidence
I70-I79	5	1945	0.02498	0.261	0.1831	impact
I70-I79	5	1955	0.02498	0.261	0.1831	incidence
I70-I79	5	1955	0.02498	0.261	0.1831	impact
I70-I79	6	1915	0.02721	0.0406	0.1895	incidence
I70-I79	6	1915	0.02721	0.0406	0.1895	impact
I70-I79	6	1925	0.02721	0.0406	0.1895	incidence

For peer review only

I70-I79	6	1925	0.02721	0.0406	0.1895	impact
I70-I79	6	1935	0.02721	0.0406	0.1895	incidence
I70-I79	6	1935	0.02721	0.0406	0.1895	impact
I70-I79	6	1945	0.02721	0.0406	0.1895	incidence
I70-I79	6	1945	0.02721	0.0406	0.1895	impact
I70-I79	6	1955	0.02721	0.0406	0.1895	incidence
I70-I79	6	1955	0.02721	0.0406	0.1895	impact
I70-I79	7	1915	0.02886	-0.00355	0.135	incidence
I70-I79	7	1915	0.02886	-0.00355	0.135	impact
I70-I79	7	1925	0.02886	-0.00355	0.135	incidence
I70-I79	7	1925	0.02886	-0.00355	0.135	impact
I70-I79	7	1935	0.02886	-0.00355	0.135	incidence
I70-I79	7	1935	0.02886	-0.00355	0.135	impact
I70-I79	7	1945	0.02886	-0.00355	0.135	incidence
I70-I79	7	1945	0.02886	-0.00355	0.135	impact
I70-I79	7	1955	0.02886	-0.00355	0.135	incidence
I70-I79	7	1955	0.02886	-0.00355	0.135	impact
I70-I79	8	1915	0.03078	-0.048	0.1019	incidence
I70-I79	8	1915	0.03078	-0.048	0.1019	impact
I70-I79	8	1925	0.03078	-0.048	0.1019	incidence
I70-I79	8	1925	0.03078	-0.048	0.1019	impact
I70-I79	8	1935	0.03078	-0.048	0.1019	incidence
I70-I79	8	1935	0.03078	-0.048	0.1019	impact
I70-I79	8	1945	0.03078	-0.048	0.1019	incidence
I70-I79	8	1945	0.03078	-0.048	0.1019	impact
I70-I79	8	1955	0.03078	-0.048	0.1019	incidence
I70-I79	8	1955	0.03078	-0.048	0.1019	impact
I70-I79	9	1915	0.03059	0.0584	0.0619	incidence
I70-I79	9	1915	0.03059	0.0584	0.0619	impact
I70-I79	9	1925	0.03059	0.0584	0.0619	incidence
I70-I79	9	1925	0.03059	0.0584	0.0619	impact
I70-I79	9	1935	0.03059	0.0584	0.0619	incidence
I70-I79	9	1935	0.03059	0.0584	0.0619	impact
I70-I79	9	1945	0.03059	0.0584	0.0619	incidence
I70-I79	9	1945	0.03059	0.0584	0.0619	impact
I70-I79	9	1955	0.03059	0.0584	0.0619	incidence
I70-I79	9	1955	0.03059	0.0584	0.0619	impact
I70-I79	10	1915	0.03226	0.0655	0.163	incidence
I70-I79	10	1915	0.03226	0.0655	0.163	impact
I70-I79	10	1925	0.03226	0.0655	0.163	incidence
I70-I79	10	1925	0.03226	0.0655	0.163	impact
I70-I79	10	1935	0.03226	0.0655	0.163	incidence
I70-I79	10	1935	0.03226	0.0655	0.163	impact

For peer review only

I70-I79	10	1945	0.03226	0.0655	0.163	incidence
I70-I79	10	1945	0.03226	0.0655	0.163	impact
I70-I79	10	1955	0.03226	0.0655	0.163	incidence
I70-I79	10	1955	0.03226	0.0655	0.163	impact
K50-K52	1	1915	0.01668	-0.17	0.164	incidence
K50-K52	1	1915	0.01668	-0.17	0.164	impact
K50-K52	1	1925	0.01668	-0.17	0.164	incidence
K50-K52	1	1925	0.01668	-0.17	0.164	impact
K50-K52	1	1935	0.01668	-0.17	0.164	incidence
K50-K52	1	1935	0.01668	-0.17	0.164	impact
K50-K52	1	1945	0.01668	-0.17	0.164	incidence
K50-K52	1	1945	0.01668	-0.17	0.164	impact
K50-K52	1	1955	0.01668	-0.17	0.164	incidence
K50-K52	1	1955	0.01668	-0.17	0.164	impact
K50-K52	2	1915	0.02159	0.288	0.1331	incidence
K50-K52	2	1915	0.02159	0.288	0.1331	impact
K50-K52	2	1925	0.02159	0.288	0.1331	incidence
K50-K52	2	1925	0.02159	0.288	0.1331	impact
K50-K52	2	1935	0.02159	0.288	0.1331	incidence
K50-K52	2	1935	0.02159	0.288	0.1331	impact
K50-K52	2	1945	0.02159	0.288	0.1331	incidence
K50-K52	2	1945	0.02159	0.288	0.1331	impact
K50-K52	2	1955	0.02159	0.288	0.1331	incidence
K50-K52	2	1955	0.02159	0.288	0.1331	impact
K50-K52	3	1915	0.02204	-0.124	0.24	incidence
K50-K52	3	1915	0.02204	-0.124	0.24	impact
K50-K52	3	1925	0.02204	-0.124	0.24	incidence
K50-K52	3	1925	0.02204	-0.124	0.24	impact
K50-K52	3	1935	0.02204	-0.124	0.24	incidence
K50-K52	3	1935	0.02204	-0.124	0.24	impact
K50-K52	3	1945	0.02204	-0.124	0.24	incidence
K50-K52	3	1945	0.02204	-0.124	0.24	impact
K50-K52	3	1955	0.02204	-0.124	0.24	incidence
K50-K52	3	1955	0.02204	-0.124	0.24	impact
K50-K52	4	1915	0.02233	-0.375	0.2026	incidence
K50-K52	4	1915	0.02233	-0.375	0.2026	impact
K50-K52	4	1925	0.02233	-0.375	0.2026	incidence
K50-K52	4	1925	0.02233	-0.375	0.2026	impact
K50-K52	4	1935	0.02233	-0.375	0.2026	incidence
K50-K52	4	1935	0.02233	-0.375	0.2026	impact
K50-K52	4	1945	0.02233	-0.375	0.2026	incidence
K50-K52	4	1945	0.02233	-0.375	0.2026	impact
K50-K52	4	1955	0.02233	-0.375	0.2026	incidence

For peer review only

K50-K52	4	1955	0.02233	-0.375	0.2026	impact
K50-K52	5	1915	0.02267	0.353	0.1282	incidence
K50-K52	5	1915	0.02267	0.353	0.1282	impact
K50-K52	5	1925	0.02267	0.353	0.1282	incidence
K50-K52	5	1925	0.02267	0.353	0.1282	impact
K50-K52	5	1935	0.02267	0.353	0.1282	incidence
K50-K52	5	1935	0.02267	0.353	0.1282	impact
K50-K52	5	1945	0.02267	0.353	0.1282	incidence
K50-K52	5	1945	0.02267	0.353	0.1282	impact
K50-K52	5	1955	0.02267	0.353	0.1282	incidence
K50-K52	5	1955	0.02267	0.353	0.1282	impact
K50-K52	6	1915	0.02537	0.149	0.1138	incidence
K50-K52	6	1915	0.02537	0.149	0.1138	impact
K50-K52	6	1925	0.02537	0.149	0.1138	incidence
K50-K52	6	1925	0.02537	0.149	0.1138	impact
K50-K52	6	1935	0.02537	0.149	0.1138	incidence
K50-K52	6	1935	0.02537	0.149	0.1138	impact
K50-K52	6	1945	0.02537	0.149	0.1138	incidence
K50-K52	6	1945	0.02537	0.149	0.1138	impact
K50-K52	6	1955	0.02537	0.149	0.1138	incidence
K50-K52	6	1955	0.02537	0.149	0.1138	impact
K50-K52	7	1915	0.028	0.0934	0.082	incidence
K50-K52	7	1915	0.028	0.0934	0.082	impact
K50-K52	7	1925	0.028	0.0934	0.082	incidence
K50-K52	7	1925	0.028	0.0934	0.082	impact
K50-K52	7	1935	0.028	0.0934	0.082	incidence
K50-K52	7	1935	0.028	0.0934	0.082	impact
K50-K52	7	1945	0.028	0.0934	0.082	incidence
K50-K52	7	1945	0.028	0.0934	0.082	impact
K50-K52	7	1955	0.028	0.0934	0.082	incidence
K50-K52	7	1955	0.028	0.0934	0.082	impact
K50-K52	8	1915	0.02751	0.0265	0.1333	incidence
K50-K52	8	1915	0.02751	0.0265	0.1333	impact
K50-K52	8	1925	0.02751	0.0265	0.1333	incidence
K50-K52	8	1925	0.02751	0.0265	0.1333	impact
K50-K52	8	1935	0.02751	0.0265	0.1333	incidence
K50-K52	8	1935	0.02751	0.0265	0.1333	impact
K50-K52	8	1945	0.02751	0.0265	0.1333	incidence
K50-K52	8	1945	0.02751	0.0265	0.1333	impact
K50-K52	8	1955	0.02751	0.0265	0.1333	incidence
K50-K52	8	1955	0.02751	0.0265	0.1333	impact
K50-K52	9	1915	0.02794	0.245	0.1126	incidence
K50-K52	9	1915	0.02794	0.245	0.1126	impact

For peer review only

K50-K52	9	1925	0.02794	0.245	0.1126	incidence
K50-K52	9	1925	0.02794	0.245	0.1126	impact
K50-K52	9	1935	0.02794	0.245	0.1126	incidence
K50-K52	9	1935	0.02794	0.245	0.1126	impact
K50-K52	9	1945	0.02794	0.245	0.1126	incidence
K50-K52	9	1945	0.02794	0.245	0.1126	impact
K50-K52	9	1955	0.02794	0.245	0.1126	incidence
K50-K52	9	1955	0.02794	0.245	0.1126	impact
K50-K52	10	1915	0.03151	0.24	0.1034	incidence
K50-K52	10	1915	0.03151	0.24	0.1034	impact
K50-K52	10	1925	0.03151	0.24	0.1034	incidence
K50-K52	10	1925	0.03151	0.24	0.1034	impact
K50-K52	10	1935	0.03151	0.24	0.1034	incidence
K50-K52	10	1935	0.03151	0.24	0.1034	impact
K50-K52	10	1945	0.03151	0.24	0.1034	incidence
K50-K52	10	1945	0.03151	0.24	0.1034	impact
K50-K52	10	1955	0.03151	0.24	0.1034	incidence
K50-K52	10	1955	0.03151	0.24	0.1034	impact
S00-S09	1	1915	0.01798	-0.152	0.183	incidence
S00-S09	1	1915	0.01798	-0.152	0.183	impact
S00-S09	1	1925	0.01798	-0.152	0.183	incidence
S00-S09	1	1925	0.01798	-0.152	0.183	impact
S00-S09	1	1935	0.01798	-0.152	0.183	incidence
S00-S09	1	1935	0.01798	-0.152	0.183	impact
S00-S09	1	1945	0.01798	-0.152	0.183	incidence
S00-S09	1	1945	0.01798	-0.152	0.183	impact
S00-S09	1	1955	0.01798	-0.152	0.183	incidence
S00-S09	1	1955	0.01798	-0.152	0.183	impact
S00-S09	2	1915	0.01603	0.248	0.1632	incidence
S00-S09	2	1915	0.01603	0.248	0.1632	impact
S00-S09	2	1925	0.01603	0.248	0.1632	incidence
S00-S09	2	1925	0.01603	0.248	0.1632	impact
S00-S09	2	1935	0.01603	0.248	0.1632	incidence
S00-S09	2	1935	0.01603	0.248	0.1632	impact
S00-S09	2	1945	0.01603	0.248	0.1632	incidence
S00-S09	2	1945	0.01603	0.248	0.1632	impact
S00-S09	2	1955	0.01603	0.248	0.1632	incidence
S00-S09	2	1955	0.01603	0.248	0.1632	impact
S00-S09	3	1915	0.01769	-0.0459	0.1171	incidence
S00-S09	3	1915	0.01769	-0.0459	0.1171	impact
S00-S09	3	1925	0.01769	-0.0459	0.1171	incidence
S00-S09	3	1925	0.01769	-0.0459	0.1171	impact
S00-S09	3	1935	0.01769	-0.0459	0.1171	incidence

For peer review only

S00-S09	3	1935	0.01769	-0.0459	0.1171	impact
S00-S09	3	1945	0.01769	-0.0459	0.1171	incidence
S00-S09	3	1945	0.01769	-0.0459	0.1171	impact
S00-S09	3	1955	0.01769	-0.0459	0.1171	incidence
S00-S09	3	1955	0.01769	-0.0459	0.1171	impact
S00-S09	4	1915	0.0202	-0.336	0.1536	incidence
S00-S09	4	1915	0.0202	-0.336	0.1536	impact
S00-S09	4	1925	0.0202	-0.336	0.1536	incidence
S00-S09	4	1925	0.0202	-0.336	0.1536	impact
S00-S09	4	1935	0.0202	-0.336	0.1536	incidence
S00-S09	4	1935	0.0202	-0.336	0.1536	impact
S00-S09	4	1945	0.0202	-0.336	0.1536	incidence
S00-S09	4	1945	0.0202	-0.336	0.1536	impact
S00-S09	4	1955	0.0202	-0.336	0.1536	incidence
S00-S09	4	1955	0.0202	-0.336	0.1536	impact
S00-S09	5	1915	0.02099	-0.213	0.1882	incidence
S00-S09	5	1915	0.02099	-0.213	0.1882	impact
S00-S09	5	1925	0.02099	-0.213	0.1882	incidence
S00-S09	5	1925	0.02099	-0.213	0.1882	impact
S00-S09	5	1935	0.02099	-0.213	0.1882	incidence
S00-S09	5	1935	0.02099	-0.213	0.1882	impact
S00-S09	5	1945	0.02099	-0.213	0.1882	incidence
S00-S09	5	1945	0.02099	-0.213	0.1882	impact
S00-S09	5	1955	0.02099	-0.213	0.1882	incidence
S00-S09	5	1955	0.02099	-0.213	0.1882	impact
S00-S09	6	1915	0.0223	0.222	0.1938	incidence
S00-S09	6	1915	0.0223	0.222	0.1938	impact
S00-S09	6	1925	0.0223	0.222	0.1938	incidence
S00-S09	6	1925	0.0223	0.222	0.1938	impact
S00-S09	6	1935	0.0223	0.222	0.1938	incidence
S00-S09	6	1935	0.0223	0.222	0.1938	impact
S00-S09	6	1945	0.0223	0.222	0.1938	incidence
S00-S09	6	1945	0.0223	0.222	0.1938	impact
S00-S09	6	1955	0.0223	0.222	0.1938	incidence
S00-S09	6	1955	0.0223	0.222	0.1938	impact
S00-S09	7	1915	0.0262	-0.00577	0.1761	incidence
S00-S09	7	1915	0.0262	-0.00577	0.1761	impact
S00-S09	7	1925	0.0262	-0.00577	0.1761	incidence
S00-S09	7	1925	0.0262	-0.00577	0.1761	impact
S00-S09	7	1935	0.0262	-0.00577	0.1761	incidence
S00-S09	7	1935	0.0262	-0.00577	0.1761	impact
S00-S09	7	1945	0.0262	-0.00577	0.1761	incidence
S00-S09	7	1945	0.0262	-0.00577	0.1761	impact

For peer review only

S00-S09	7	1955	0.0262	-0.00577	0.1761	incidence
S00-S09	7	1955	0.0262	-0.00577	0.1761	impact
S00-S09	8	1915	0.02803	0.0104	0.0798	incidence
S00-S09	8	1915	0.02803	0.0104	0.0798	impact
S00-S09	8	1925	0.02803	0.0104	0.0798	incidence
S00-S09	8	1925	0.02803	0.0104	0.0798	impact
S00-S09	8	1935	0.02803	0.0104	0.0798	incidence
S00-S09	8	1935	0.02803	0.0104	0.0798	impact
S00-S09	8	1945	0.02803	0.0104	0.0798	incidence
S00-S09	8	1945	0.02803	0.0104	0.0798	impact
S00-S09	8	1955	0.02803	0.0104	0.0798	incidence
S00-S09	8	1955	0.02803	0.0104	0.0798	impact
S00-S09	9	1915	0.03226	-0.093	0.1162	incidence
S00-S09	9	1915	0.03226	-0.093	0.1162	impact
S00-S09	9	1925	0.03226	-0.093	0.1162	incidence
S00-S09	9	1925	0.03226	-0.093	0.1162	impact
S00-S09	9	1935	0.03226	-0.093	0.1162	incidence
S00-S09	9	1935	0.03226	-0.093	0.1162	impact
S00-S09	9	1945	0.03226	-0.093	0.1162	incidence
S00-S09	9	1945	0.03226	-0.093	0.1162	impact
S00-S09	9	1955	0.03226	-0.093	0.1162	incidence
S00-S09	9	1955	0.03226	-0.093	0.1162	impact
S00-S09	10	1915	0.039	0.181	0.143	incidence
S00-S09	10	1915	0.039	0.181	0.143	impact
S00-S09	10	1925	0.039	0.181	0.143	incidence
S00-S09	10	1925	0.039	0.181	0.143	impact
S00-S09	10	1935	0.039	0.181	0.143	incidence
S00-S09	10	1935	0.039	0.181	0.143	impact
S00-S09	10	1945	0.039	0.181	0.143	incidence
S00-S09	10	1945	0.039	0.181	0.143	impact
S00-S09	10	1955	0.039	0.181	0.143	incidence
S00-S09	10	1955	0.039	0.181	0.143	impact
C50-C50	1	1915	0.02952	0.565	0.2398	incidence
C50-C50	1	1915	0.02952	0.565	0.2398	impact
C50-C50	1	1925	0.02952	0.565	0.2398	incidence
C50-C50	1	1925	0.02952	0.565	0.2398	impact
C50-C50	1	1935	0.02952	0.565	0.2398	incidence
C50-C50	1	1935	0.02952	0.565	0.2398	impact
C50-C50	1	1945	0.02952	0.565	0.2398	incidence
C50-C50	1	1945	0.02952	0.565	0.2398	impact
C50-C50	1	1955	0.02952	0.565	0.2398	incidence
C50-C50	1	1955	0.02952	0.565	0.2398	impact
C50-C50	2	1915	0.02828	1.74	0.2432	incidence

For peer review only

C50-C50	2	1915	0.02828	1.74	0.2432 impact
C50-C50	2	1925	0.02828	1.74	0.2432 incidence
C50-C50	2	1925	0.02828	1.74	0.2432 impact
C50-C50	2	1935	0.02828	1.74	0.2432 incidence
C50-C50	2	1935	0.02828	1.74	0.2432 impact
C50-C50	2	1945	0.02828	1.74	0.2432 incidence
C50-C50	2	1945	0.02828	1.74	0.2432 impact
C50-C50	2	1955	0.02828	1.74	0.2432 incidence
C50-C50	2	1955	0.02828	1.74	0.2432 impact
C50-C50	3	1915	0.02502	1.7	0.2848 incidence
C50-C50	3	1915	0.02502	1.7	0.2848 impact
C50-C50	3	1925	0.02502	1.7	0.2848 incidence
C50-C50	3	1925	0.02502	1.7	0.2848 impact
C50-C50	3	1935	0.02502	1.7	0.2848 incidence
C50-C50	3	1935	0.02502	1.7	0.2848 impact
C50-C50	3	1945	0.02502	1.7	0.2848 incidence
C50-C50	3	1945	0.02502	1.7	0.2848 impact
C50-C50	3	1955	0.02502	1.7	0.2848 incidence
C50-C50	3	1955	0.02502	1.7	0.2848 impact
C50-C50	4	1915	0.02499	1.45	0.1209 incidence
C50-C50	4	1915	0.02499	1.45	0.1209 impact
C50-C50	4	1925	0.02499	1.45	0.1209 incidence
C50-C50	4	1925	0.02499	1.45	0.1209 impact
C50-C50	4	1935	0.02499	1.45	0.1209 incidence
C50-C50	4	1935	0.02499	1.45	0.1209 impact
C50-C50	4	1945	0.02499	1.45	0.1209 incidence
C50-C50	4	1945	0.02499	1.45	0.1209 impact
C50-C50	4	1955	0.02499	1.45	0.1209 incidence
C50-C50	4	1955	0.02499	1.45	0.1209 impact
C50-C50	5	1915	0.02305	1.17	0.1704 incidence
C50-C50	5	1915	0.02305	1.17	0.1704 impact
C50-C50	5	1925	0.02305	1.17	0.1704 incidence
C50-C50	5	1925	0.02305	1.17	0.1704 impact
C50-C50	5	1935	0.02305	1.17	0.1704 incidence
C50-C50	5	1935	0.02305	1.17	0.1704 impact
C50-C50	5	1945	0.02305	1.17	0.1704 incidence
C50-C50	5	1945	0.02305	1.17	0.1704 impact
C50-C50	5	1955	0.02305	1.17	0.1704 incidence
C50-C50	5	1955	0.02305	1.17	0.1704 impact
C50-C50	6	1915	0.02338	1.59	0.3056 incidence
C50-C50	6	1915	0.02338	1.59	0.3056 impact
C50-C50	6	1925	0.02338	1.59	0.3056 incidence
C50-C50	6	1925	0.02338	1.59	0.3056 impact

For peer review only

C50-C50	6	1935	0.02338	1.59	0.3056 incidence
C50-C50	6	1935	0.02338	1.59	0.3056 impact
C50-C50	6	1945	0.02338	1.59	0.3056 incidence
C50-C50	6	1945	0.02338	1.59	0.3056 impact
C50-C50	6	1955	0.02338	1.59	0.3056 incidence
C50-C50	6	1955	0.02338	1.59	0.3056 impact
C50-C50	7	1915	0.02101	0.955	0.2127 incidence
C50-C50	7	1915	0.02101	0.955	0.2127 impact
C50-C50	7	1925	0.02101	0.955	0.2127 incidence
C50-C50	7	1925	0.02101	0.955	0.2127 impact
C50-C50	7	1935	0.02101	0.955	0.2127 incidence
C50-C50	7	1935	0.02101	0.955	0.2127 impact
C50-C50	7	1945	0.02101	0.955	0.2127 incidence
C50-C50	7	1945	0.02101	0.955	0.2127 impact
C50-C50	7	1955	0.02101	0.955	0.2127 incidence
C50-C50	7	1955	0.02101	0.955	0.2127 impact
C50-C50	8	1915	0.02088	0.876	0.1875 incidence
C50-C50	8	1915	0.02088	0.876	0.1875 impact
C50-C50	8	1925	0.02088	0.876	0.1875 incidence
C50-C50	8	1925	0.02088	0.876	0.1875 impact
C50-C50	8	1935	0.02088	0.876	0.1875 incidence
C50-C50	8	1935	0.02088	0.876	0.1875 impact
C50-C50	8	1945	0.02088	0.876	0.1875 incidence
C50-C50	8	1945	0.02088	0.876	0.1875 impact
C50-C50	8	1955	0.02088	0.876	0.1875 incidence
C50-C50	8	1955	0.02088	0.876	0.1875 impact
C50-C50	9	1915	0.02093	1.03	0.2063 incidence
C50-C50	9	1915	0.02093	1.03	0.2063 impact
C50-C50	9	1925	0.02093	1.03	0.2063 incidence
C50-C50	9	1925	0.02093	1.03	0.2063 impact
C50-C50	9	1935	0.02093	1.03	0.2063 incidence
C50-C50	9	1935	0.02093	1.03	0.2063 impact
C50-C50	9	1945	0.02093	1.03	0.2063 incidence
C50-C50	9	1945	0.02093	1.03	0.2063 impact
C50-C50	9	1955	0.02093	1.03	0.2063 incidence
C50-C50	9	1955	0.02093	1.03	0.2063 impact
C50-C50	10	1915	0.01881	0.806	0.1659 incidence
C50-C50	10	1915	0.01881	0.806	0.1659 impact
C50-C50	10	1925	0.01881	0.806	0.1659 incidence
C50-C50	10	1925	0.01881	0.806	0.1659 impact
C50-C50	10	1935	0.01881	0.806	0.1659 incidence
C50-C50	10	1935	0.01881	0.806	0.1659 impact
C50-C50	10	1945	0.01881	0.806	0.1659 incidence

For peer review only

C50-C50	10	1945	0.01881	0.806	0.1659	impact
C50-C50	10	1955	0.01881	0.806	0.1659	incidence
C50-C50	10	1955	0.01881	0.806	0.1659	impact
C60-C63	1	1915	0.01835	0.552	0.2666	incidence
C60-C63	1	1915	0.01835	0.552	0.2666	impact
C60-C63	1	1925	0.01835	0.552	0.2666	incidence
C60-C63	1	1925	0.01835	0.552	0.2666	impact
C60-C63	1	1935	0.01835	0.552	0.2666	incidence
C60-C63	1	1935	0.01835	0.552	0.2666	impact
C60-C63	1	1945	0.01835	0.552	0.2666	incidence
C60-C63	1	1945	0.01835	0.552	0.2666	impact
C60-C63	1	1955	0.01835	0.552	0.2666	incidence
C60-C63	1	1955	0.01835	0.552	0.2666	impact
C60-C63	2	1915	0.01534	0.923	0.1908	incidence
C60-C63	2	1915	0.01534	0.923	0.1908	impact
C60-C63	2	1925	0.01534	0.923	0.1908	incidence
C60-C63	2	1925	0.01534	0.923	0.1908	impact
C60-C63	2	1935	0.01534	0.923	0.1908	incidence
C60-C63	2	1935	0.01534	0.923	0.1908	impact
C60-C63	2	1945	0.01534	0.923	0.1908	incidence
C60-C63	2	1945	0.01534	0.923	0.1908	impact
C60-C63	2	1955	0.01534	0.923	0.1908	incidence
C60-C63	2	1955	0.01534	0.923	0.1908	impact
C60-C63	3	1915	0.01468	0.234	0.193	incidence
C60-C63	3	1915	0.01468	0.234	0.193	impact
C60-C63	3	1925	0.01468	0.234	0.193	incidence
C60-C63	3	1925	0.01468	0.234	0.193	impact
C60-C63	3	1935	0.01468	0.234	0.193	incidence
C60-C63	3	1935	0.01468	0.234	0.193	impact
C60-C63	3	1945	0.01468	0.234	0.193	incidence
C60-C63	3	1945	0.01468	0.234	0.193	impact
C60-C63	3	1955	0.01468	0.234	0.193	incidence
C60-C63	3	1955	0.01468	0.234	0.193	impact
C60-C63	4	1915	0.01534	0.624	0.1919	incidence
C60-C63	4	1915	0.01534	0.624	0.1919	impact
C60-C63	4	1925	0.01534	0.624	0.1919	incidence
C60-C63	4	1925	0.01534	0.624	0.1919	impact
C60-C63	4	1935	0.01534	0.624	0.1919	incidence
C60-C63	4	1935	0.01534	0.624	0.1919	impact
C60-C63	4	1945	0.01534	0.624	0.1919	incidence
C60-C63	4	1945	0.01534	0.624	0.1919	impact
C60-C63	4	1955	0.01534	0.624	0.1919	incidence
C60-C63	4	1955	0.01534	0.624	0.1919	impact

For peer review only

C60-C63	5	1915	0.0146	0.876	0.2221 incidence
C60-C63	5	1915	0.0146	0.876	0.2221 impact
C60-C63	5	1925	0.0146	0.876	0.2221 incidence
C60-C63	5	1925	0.0146	0.876	0.2221 impact
C60-C63	5	1935	0.0146	0.876	0.2221 incidence
C60-C63	5	1935	0.0146	0.876	0.2221 impact
C60-C63	5	1945	0.0146	0.876	0.2221 incidence
C60-C63	5	1945	0.0146	0.876	0.2221 impact
C60-C63	5	1955	0.0146	0.876	0.2221 incidence
C60-C63	5	1955	0.0146	0.876	0.2221 impact
C60-C63	6	1915	0.01287	0.86	0.187 incidence
C60-C63	6	1915	0.01287	0.86	0.187 impact
C60-C63	6	1925	0.01287	0.86	0.187 incidence
C60-C63	6	1925	0.01287	0.86	0.187 impact
C60-C63	6	1935	0.01287	0.86	0.187 incidence
C60-C63	6	1935	0.01287	0.86	0.187 impact
C60-C63	6	1945	0.01287	0.86	0.187 incidence
C60-C63	6	1945	0.01287	0.86	0.187 impact
C60-C63	6	1955	0.01287	0.86	0.187 incidence
C60-C63	6	1955	0.01287	0.86	0.187 impact
C60-C63	7	1915	0.01208	0.38	0.244 incidence
C60-C63	7	1915	0.01208	0.38	0.244 impact
C60-C63	7	1925	0.01208	0.38	0.244 incidence
C60-C63	7	1925	0.01208	0.38	0.244 impact
C60-C63	7	1935	0.01208	0.38	0.244 incidence
C60-C63	7	1935	0.01208	0.38	0.244 impact
C60-C63	7	1945	0.01208	0.38	0.244 incidence
C60-C63	7	1945	0.01208	0.38	0.244 impact
C60-C63	7	1955	0.01208	0.38	0.244 incidence
C60-C63	7	1955	0.01208	0.38	0.244 impact
C60-C63	8	1915	0.01184	0.772	0.1313 incidence
C60-C63	8	1915	0.01184	0.772	0.1313 impact
C60-C63	8	1925	0.01184	0.772	0.1313 incidence
C60-C63	8	1925	0.01184	0.772	0.1313 impact
C60-C63	8	1935	0.01184	0.772	0.1313 incidence
C60-C63	8	1935	0.01184	0.772	0.1313 impact
C60-C63	8	1945	0.01184	0.772	0.1313 incidence
C60-C63	8	1945	0.01184	0.772	0.1313 impact
C60-C63	8	1955	0.01184	0.772	0.1313 incidence
C60-C63	8	1955	0.01184	0.772	0.1313 impact
C60-C63	9	1915	0.01124	1.29	0.2817 incidence
C60-C63	9	1915	0.01124	1.29	0.2817 impact
C60-C63	9	1925	0.01124	1.29	0.2817 incidence

For peer review only

C60-C63	9	1925	0.01124	1.29	0.2817 impact
C60-C63	9	1935	0.01124	1.29	0.2817 incidence
C60-C63	9	1935	0.01124	1.29	0.2817 impact
C60-C63	9	1945	0.01124	1.29	0.2817 incidence
C60-C63	9	1945	0.01124	1.29	0.2817 impact
C60-C63	9	1955	0.01124	1.29	0.2817 incidence
C60-C63	9	1955	0.01124	1.29	0.2817 impact
C60-C63	10	1915	0.00884	0.618	0.1586 incidence
C60-C63	10	1915	0.00884	0.618	0.1586 impact
C60-C63	10	1925	0.00884	0.618	0.1586 incidence
C60-C63	10	1925	0.00884	0.618	0.1586 impact
C60-C63	10	1935	0.00884	0.618	0.1586 incidence
C60-C63	10	1935	0.00884	0.618	0.1586 impact
C60-C63	10	1945	0.00884	0.618	0.1586 incidence
C60-C63	10	1945	0.00884	0.618	0.1586 impact
C60-C63	10	1955	0.00884	0.618	0.1586 incidence
C60-C63	10	1955	0.00884	0.618	0.1586 impact
G30-G32	1	1915	0.00293	0.146	0.326 incidence
G30-G32	1	1915	0.00293	0.146	0.326 impact
G30-G32	1	1925	0.00293	0.146	0.326 incidence
G30-G32	1	1925	0.00293	0.146	0.326 impact
G30-G32	1	1935	0.00293	0.146	0.326 incidence
G30-G32	1	1935	0.00293	0.146	0.326 impact
G30-G32	1	1945	0.00293	0.146	0.326 incidence
G30-G32	1	1945	0.00293	0.146	0.326 impact
G30-G32	1	1955	0.00293	0.146	0.326 incidence
G30-G32	1	1955	0.00293	0.146	0.326 impact
G30-G32	2	1915	0.00272	0.248	0.4579 incidence
G30-G32	2	1915	0.00272	0.248	0.4579 impact
G30-G32	2	1925	0.00272	0.248	0.4579 incidence
G30-G32	2	1925	0.00272	0.248	0.4579 impact
G30-G32	2	1935	0.00272	0.248	0.4579 incidence
G30-G32	2	1935	0.00272	0.248	0.4579 impact
G30-G32	2	1945	0.00272	0.248	0.4579 incidence
G30-G32	2	1945	0.00272	0.248	0.4579 impact
G30-G32	2	1955	0.00272	0.248	0.4579 incidence
G30-G32	2	1955	0.00272	0.248	0.4579 impact
G30-G32	3	1915	0.00285	-0.361	0.3347 incidence
G30-G32	3	1915	0.00285	-0.361	0.3347 impact
G30-G32	3	1925	0.00285	-0.361	0.3347 incidence
G30-G32	3	1925	0.00285	-0.361	0.3347 impact
G30-G32	3	1935	0.00285	-0.361	0.3347 incidence
G30-G32	3	1935	0.00285	-0.361	0.3347 impact

For peer review only

G30-G32	3	1945	0.00285	-0.361	0.3347	incidence
G30-G32	3	1945	0.00285	-0.361	0.3347	impact
G30-G32	3	1955	0.00285	-0.361	0.3347	incidence
G30-G32	3	1955	0.00285	-0.361	0.3347	impact
G30-G32	4	1915	0.00271	-1.27	0.284	incidence
G30-G32	4	1915	0.00271	-1.27	0.284	impact
G30-G32	4	1925	0.00271	-1.27	0.284	incidence
G30-G32	4	1925	0.00271	-1.27	0.284	impact
G30-G32	4	1935	0.00271	-1.27	0.284	incidence
G30-G32	4	1935	0.00271	-1.27	0.284	impact
G30-G32	4	1945	0.00271	-1.27	0.284	incidence
G30-G32	4	1945	0.00271	-1.27	0.284	impact
G30-G32	4	1955	0.00271	-1.27	0.284	incidence
G30-G32	4	1955	0.00271	-1.27	0.284	impact
G30-G32	5	1915	0.0025	0.876	0.3529	incidence
G30-G32	5	1915	0.0025	0.876	0.3529	impact
G30-G32	5	1925	0.0025	0.876	0.3529	incidence
G30-G32	5	1925	0.0025	0.876	0.3529	impact
G30-G32	5	1935	0.0025	0.876	0.3529	incidence
G30-G32	5	1935	0.0025	0.876	0.3529	impact
G30-G32	5	1945	0.0025	0.876	0.3529	incidence
G30-G32	5	1945	0.0025	0.876	0.3529	impact
G30-G32	5	1955	0.0025	0.876	0.3529	incidence
G30-G32	5	1955	0.0025	0.876	0.3529	impact
G30-G32	6	1915	0.00272	0.451	0.4303	incidence
G30-G32	6	1915	0.00272	0.451	0.4303	impact
G30-G32	6	1925	0.00272	0.451	0.4303	incidence
G30-G32	6	1925	0.00272	0.451	0.4303	impact
G30-G32	6	1935	0.00272	0.451	0.4303	incidence
G30-G32	6	1935	0.00272	0.451	0.4303	impact
G30-G32	6	1945	0.00272	0.451	0.4303	incidence
G30-G32	6	1945	0.00272	0.451	0.4303	impact
G30-G32	6	1955	0.00272	0.451	0.4303	incidence
G30-G32	6	1955	0.00272	0.451	0.4303	impact
G30-G32	7	1915	0.00298	-1.24	0.588	incidence
G30-G32	7	1915	0.00298	-1.24	0.588	impact
G30-G32	7	1925	0.00298	-1.24	0.588	incidence
G30-G32	7	1925	0.00298	-1.24	0.588	impact
G30-G32	7	1935	0.00298	-1.24	0.588	incidence
G30-G32	7	1935	0.00298	-1.24	0.588	impact
G30-G32	7	1945	0.00298	-1.24	0.588	incidence
G30-G32	7	1945	0.00298	-1.24	0.588	impact
G30-G32	7	1955	0.00298	-1.24	0.588	incidence

For peer review only

G30-G32	7	1955	0.00298	-1.24	0.588	impact
G30-G32	8	1915	0.00279	0.552	0.4202	incidence
G30-G32	8	1915	0.00279	0.552	0.4202	impact
G30-G32	8	1925	0.00279	0.552	0.4202	incidence
G30-G32	8	1925	0.00279	0.552	0.4202	impact
G30-G32	8	1935	0.00279	0.552	0.4202	incidence
G30-G32	8	1935	0.00279	0.552	0.4202	impact
G30-G32	8	1945	0.00279	0.552	0.4202	incidence
G30-G32	8	1945	0.00279	0.552	0.4202	impact
G30-G32	8	1955	0.00279	0.552	0.4202	incidence
G30-G32	8	1955	0.00279	0.552	0.4202	impact
G30-G32	9	1915	0.00295	0.304	0.2768	incidence
G30-G32	9	1915	0.00295	0.304	0.2768	impact
G30-G32	9	1925	0.00295	0.304	0.2768	incidence
G30-G32	9	1925	0.00295	0.304	0.2768	impact
G30-G32	9	1935	0.00295	0.304	0.2768	incidence
G30-G32	9	1935	0.00295	0.304	0.2768	impact
G30-G32	9	1945	0.00295	0.304	0.2768	incidence
G30-G32	9	1945	0.00295	0.304	0.2768	impact
G30-G32	9	1955	0.00295	0.304	0.2768	incidence
G30-G32	9	1955	0.00295	0.304	0.2768	impact
G30-G32	10	1915	0.00313	-0.429	0.12	incidence
G30-G32	10	1915	0.00313	-0.429	0.12	impact
G30-G32	10	1925	0.00313	-0.429	0.12	incidence
G30-G32	10	1925	0.00313	-0.429	0.12	impact
G30-G32	10	1935	0.00313	-0.429	0.12	incidence
G30-G32	10	1935	0.00313	-0.429	0.12	impact
G30-G32	10	1945	0.00313	-0.429	0.12	incidence
G30-G32	10	1945	0.00313	-0.429	0.12	impact
G30-G32	10	1955	0.00313	-0.429	0.12	incidence
G30-G32	10	1955	0.00313	-0.429	0.12	impact

For peer review only

beta	se	strat_var
-0.068	0.01967587	none
-0.068	0.01967587	none
0.124	0.01838967	none
0.124	0.01838967	none
0.274	0.0173358	none
0.274	0.0173358	none
0.323	0.01544801	none
0.323	0.01544801	none
0.299	0.00997	none
0.299	0.00997	none
0.00812434	0.01069376	none
0.19473187	0.01383358	none
0.12017855	0.00829215	none
0.3067276	0.01206114	none
0.17119761	0.0073964	none
0.35735305	0.01146477	none
0.17496596	0.00637669	none
0.36157349	0.01084774	none
0.11665286	0.00404314	none
0.3032604	0.00966222	none
-0.20744	0.04331108	none
-0.05264	0.05625531	none
-0.1417	0.0388434	none
0.0131	0.05289253	none
-0.1213	0.03588718	none
0.0335	0.05076121	none
-0.1787	0.03121728	none
-0.0239	0.04757445	none
-0.2161	0.01972	none
-0.0613	0.0409596	none
-0.051	0.03267415	none
0.1827	0.05180386	none
0.079	0.02295844	none
0.3127	0.04629395	none
0.19984	0.01875793	none
0.43354	0.04436102	none
0.25636	0.0147411	none
0.49006	0.04281752	none
0.1418	0.0087	none
0.3755	0.04113065	none
-0.26062	0.07552801	none
-0.05102	0.08874706	none

For peer review only

0.1066	0.05192004	none
0.3162	0.06976568	none
0.2727	0.04692004	none
0.4823	0.06612904	none
0.3708	0.04223837	none
0.5804	0.06289388	none
0.4629	0.0278	none
0.6725	0.05426233	none
0.15981	0.03755423	none
0.27711	0.05815221	none
0.17	0.02651886	none
0.2873	0.05171663	none
0.1744	0.02147417	none
0.2917	0.04932038	none
0.1442	0.01669378	none
0.2615	0.04743461	none
0.0644	0.00982	none
0.1817	0.04547299	none
0.00907	0.03465977	none
0.03497	0.04285254	none
0.1971	0.0298734	none
0.223	0.03908273	none
0.1963	0.02671647	none
0.2222	0.03672615	none
0.131	0.02207825	none
0.1569	0.03350357	none
0.0154	0.01333	none
0.0413	0.0285084	none
0.093	0.05622962	none
0.4884	0.06676653	none
0.196	0.04432268	none
0.5914	0.05710079	none
0.26561	0.04013839	none
0.66101	0.05391744	none
0.33219	0.03544885	none
0.72759	0.05052347	none
0.2882	0.02297	none
0.6836	0.04270388	none
0.126	0.03819346	none
0.3062	0.04353792	none
0.276	0.0333997	none
0.4562	0.03939987	none
0.33362	0.03078847	none

For peer review only

0.51382	0.0372121	none
0.30278	0.02708621	none
0.48298	0.03421217	none
0.1746	0.01735	none
0.3548	0.02716307	none
0.03395	0.07671617	none
0.39975	0.10074453	none
0.1104	0.05691371	none
0.4762	0.08662136	none
0.2055	0.050143	none
0.5713	0.0823311	none
0.2557	0.0433663	none
0.6215	0.0783883	none
0.2683	0.02766	none
0.6341	0.07091661	none
0.11957	0.04027034	none
0.41587	0.05355175	none
-5.00E-04	0.02722223	none
0.2958	0.04457735	none
-0.0261	0.02243212	none
0.2702	0.04182451	none
-0.0263	0.01806621	none
0.27	0.03965448	none
-0.0625	0.01091	none
0.2338	0.03694751	none
0.1062	0.04539273	none
0.3095	0.05659452	none
0.3056	0.04032989	none
0.5089	0.05262072	none
0.3354	0.03723251	none
0.5387	0.05028618	none
0.2974	0.03247106	none
0.5007	0.04687014	none
0.1283	0.0206	none
0.3316	0.03958283	none
0.132	0.03333767	none
0.3554	0.04016416	none
0.111	0.02386629	none
0.3344	0.03273164	none
0.03424	0.01987159	none
0.25764	0.02994395	none
0.01546	0.01608439	none
0.23886	0.02757658	none

For peer review only

-0.0067	0.00974	none
0.2167	0.02442596	none
0.061	0.05475263	none
0.299	0.07183384	none
0.236	0.04143151	none
0.474	0.06228017	none
0.37465	0.03723923	none
0.61265	0.05957357	none
0.32565	0.03240655	none
0.56365	0.05667834	none
0.2317	0.02062	none
0.4697	0.05086683	none
0.283	0.03449072	none
0.5469	0.04857211	none
0.437	0.02631369	none
0.7009	0.04315148	none
0.49165	0.02261526	none
0.75555	0.0410011	none
0.52295	0.01901588	none
0.78685	0.03913111	none
0.3884	0.01194	none
0.6523	0.03622435	none
-0.27757	0.03882525	none
-0.49237	0.05538637	none
-0.0859	0.03285362	none
-0.3007	0.05137714	none
0.0024	0.0292	none
-0.2124	0.04912118	none
0.0832	0.02420553	none
-0.1316	0.04632664	none
0.0762	0.01474	none
-0.1386	0.04216062	none
0.30913	0.0400451	none
0.44263	0.06335945	none
0.3116	0.02625281	none
0.4451	0.05567782	none
0.3308	0.0212417	none
0.4643	0.05349785	none
0.3963	0.016866	none
0.5298	0.05191601	none
0.304	0.01011	none
0.4375	0.05013005	none
-0.22614	0.05050149	none

For peer review only

-0.33344	0.05600036	none
-0.0604	0.04472136	none
-0.1677	0.05084919	none
0.0052	0.0408589	none
-0.1021	0.04748779	none
-0.0634	0.03492985	none
-0.1707	0.04249393	none
-0.1954	0.02179	none
-0.3027	0.03256446	none
0.05001	0.06302801	none
0.03011	0.06646608	none
0.2069	0.06025363	none
0.187	0.06384129	none
0.2531	0.05758351	none
0.2332	0.06132756	none
0.1413	0.05168306	none
0.1214	0.05582427	none
0.0546	0.03333	none
0.0347	0.03944742	none
-0.5186	0.07772837	none
-0.4553	0.12879825	none
-0.4419	0.06526944	none
-0.3786	0.12168562	none
-0.3852	0.05892113	none
-0.3219	0.11840182	none
-0.4369	0.05061719	none
-0.3736	0.11449624	none
-0.4306	0.0319	none
-0.3673	0.10754023	none
-0.0659	0.05732469	none
0.1176	0.08525098	none
-0.039	0.03942296	none
0.1445	0.07440282	none
0.0098	0.0329196	none
0.1933	0.07117099	none
0.0174	0.02694327	none
0.2009	0.06861159	none
-0.0975	0.0165	none
0.086	0.06522162	none
-0.3314	0.07665722	none
-0.1282	0.09600609	none
-0.0903	0.0665115	none
0.1129	0.08811708	none

For peer review only

0.1154	0.06150325	none
0.3186	0.08440077	none
0.0823	0.05410841	none
0.2855	0.07917424	none
0.1126	0.0346	none
0.3158	0.06736468	none
0.25092	0.06373257	none
0.48902	0.07924803	none
0.1729	0.0401005	none
0.411	0.06185839	none
0.1818	0.02998033	none
0.4199	0.05583216	none
0.2675	0.02212833	none
0.5056	0.05203915	none
0.2474	0.01273	none
0.4855	0.04878999	none
0.0801	0.06406005	none
0.2653	0.09510931	none
0.1164	0.05083788	none
0.3016	0.08675586	none
0.0173	0.04343087	none
0.2025	0.08263371	none
-0.0215	0.03512036	none
0.1637	0.07858454	none
-0.0991	0.0212	none
0.0861	0.07342704	none
0.15853	0.06886755	none
0.24953	0.07904543	none
0.3895	0.05438695	none
0.4805	0.06680853	none
0.5012	0.04899082	none
0.5922	0.06249432	none
0.4928	0.04289243	none
0.5838	0.05783771	none
0.46	0.02752	none
0.551	0.0475688	none
0.15185	0.06062384	none
0.26795	0.06827306	none
0.2981	0.0457739	none
0.4142	0.05550865	none
0.3709	0.03821963	none
0.487	0.04946413	none
0.3769	0.03014557	none

For peer review only

0.493	0.04352833	none
0.3444	0.01784	none
0.4605	0.03611406	none
-0.1914	0.05464842	none
-0.1759	0.07300007	none
-0.1319	0.04638933	none
-0.1164	0.06704126	none
0.035	0.04025481	none
0.0505	0.06295244	none
0.2775	0.03168911	none
0.293	0.05785119	none
0.2837	0.0184	none
0.2992	0.05177953	none
0.28644	0.09024217	none
1.44704	0.11347727	none
0.2329	0.05508176	none
1.3935	0.08813308	none
0.1266	0.0425737	none
1.2872	0.08090711	none
0.1857	0.03301801	none
1.3463	0.0763127	none
0.2174	0.01983	none
1.378	0.07160076	none
0.2049	0.1078416	none
0.9032	0.12690713	none
0.3433	0.08165886	none
1.0416	0.1055641	none
0.108	0.07402297	none
0.8063	0.0997748	none
-0.0828	0.06728358	none
0.6155	0.09488251	none
-0.0995	0.0448	none
0.5988	0.08051491	none
0.02977	0.24127671	none
0.06727	0.28092999	none
0.2979	0.22680249	none
NA	NA	none
0.54149	0.21435916	none
NA	NA	none
0.42091	0.18726128	none
0.45841	0.23616519	none
0.1798	0.11813	none
0.2173	0.18617709	none

For peer review only

-0.059	0.02723564	sex
-0.059	0.02723564	sex
0.109	0.02433475	sex
0.109	0.02433475	sex
0.274	0.02271673	sex
0.274	0.02271673	sex
0.358	0.02015341	sex
0.358	0.02015341	sex
0.339	0.013	sex
0.339	0.013	sex
-0.076	0.02963798	sex
-0.076	0.02963798	sex
0.13	0.0283593	sex
0.13	0.0283593	sex
0.272	0.02695645	sex
0.272	0.02695645	sex
0.273	0.02412302	sex
0.273	0.02412302	sex
0.242	0.0156	sex
0.242	0.0156	sex
0.03269788	0.01610284	sex
0.22360952	0.01967766	sex
0.1187832	0.01180346	sex
0.31007609	0.01634917	sex
0.18644042	0.01046499	sex
0.37773331	0.0154107	sex
0.21233446	0.0090729	sex
0.40362734	0.01450143	sex
0.16377289	0.00579663	sex
0.35506578	0.01271121	sex
0.00114746	0.01472157	sex
0.18341975	0.01796179	sex
0.11986523	0.01182827	sex
0.30213752	0.0156783	sex
0.15590977	0.01062114	sex
0.33818206	0.01478884	sex
0.13444135	0.00913484	sex
0.31671364	0.01376032	sex
0.07415494	0.00576279	sex
0.25642723	0.01179453	sex
-0.1097	0.06177783	sex
0.0047	0.08304667	sex
-0.12721	0.0539179	sex

For peer review only

-0.01281	0.07737823	sex
-0.10449	0.04975791	sex
0.00991	0.07453925	sex
-0.09908	0.04354595	sex
0.01532	0.07054431	sex
-0.15792	0.0277	sex
-0.04352	0.06202854	sex
-0.2518	0.06133743	sex
-0.0573	0.06918295	sex
-0.15187	0.05602856	sex
0.04263	0.06452286	sex
-0.14063	0.05188998	sex
0.05387	0.06096368	sex
-0.27624	0.04480647	sex
-0.08174	0.05506015	sex
-0.27376	0.0281	sex
-0.07926	0.0425865	sex
-0.0228	0.05199548	sex
0.1961	0.07241333	sex
0.08933	0.03287461	sex
0.30823	0.06017392	sex
0.22697	0.02637821	sex
0.44587	0.05688559	sex
0.28377	0.02060097	sex
0.50267	0.05444777	sex
0.19423	0.0122	sex
0.41313	0.05185557	sex
-0.057	0.04300151	sex
0.1846	0.06392511	sex
0.07366	0.03216287	sex
0.31526	0.05719913	sex
0.17524	0.0267088	sex
0.41684	0.05431989	sex
0.2264	0.02104186	sex
0.468	0.0517692	sex
0.0886	0.0124	sex
0.3302	0.04889836	sex
-0.0538	0.10411767	sex
0.1592	0.1111472	sex
0.18086	0.07118659	sex
0.39386	0.08112176	sex
0.35554	0.06439705	sex
0.56854	0.07523423	sex

For peer review only

0.46193	0.05824036	sex
0.67493	0.07003678	sex
0.60807	0.0385	sex
0.82107	0.0547308	sex
-0.4167	0.10976083	sex
-0.2139	0.13050594	sex
0.02981	0.07621916	sex
0.23261	0.10389283	sex
0.17359	0.06872125	sex
0.37639	0.09852396	sex
0.24838	0.06153324	sex
0.45118	0.09365202	sex
0.29862	0.0403	sex
0.50142	0.08129237	sex
0.2061	0.06410772	sex
0.3091	0.07919628	sex
0.16362	0.04169844	sex
0.26662	0.06245807	sex
0.18888	0.03348447	sex
0.29188	0.05730148	sex
0.17844	0.02630019	sex
0.28144	0.05342237	sex
0.09856	0.0157	sex
0.20156	0.04907892	sex
0.1598	0.04679541	sex
0.2851	0.06848219	sex
0.1815	0.03432856	sex
0.3068	0.06065023	sex
0.1651	0.02787723	sex
0.2904	0.05724631	sex
0.11918	0.02156409	sex
0.24448	0.05445191	sex
0.04182	0.0126	sex
0.16712	0.05156317	sex
0.0601	0.05286	sex
0.1343	0.06252791	sex
0.15465	0.04242794	sex
0.22885	0.05399713	sex
0.19415	0.03742192	sex
0.26835	0.05015935	sex
0.19373	0.03102209	sex
0.26793	0.04558432	sex
0.09327	0.0189	sex

For peer review only

0.16747	0.03837669	sex
-0.0119	0.04728097	sex
-0.0285	0.06377562	sex
0.22479	0.04224559	sex
0.20819	0.06013759	sex
0.1980031	0.03819332	sex
0.1814031	0.05736349	sex
0.0608969	0.0314401	sex
0.0442969	0.05310669	sex
-0.06099	0.0188	sex
-0.07759	0.04674698	sex
0.0876	0.08094566	sex
0.4536	0.09385334	sex
0.17702	0.05816537	sex
0.54302	0.07509634	sex
0.28948	0.05203124	sex
0.65548	0.07045211	sex
0.35387	0.04609772	sex
0.71987	0.06619101	sex
0.33013	0.03	sex
0.69613	0.05618051	sex
0.0923	0.08186745	sex
0.5207	0.09587643	sex
0.21209	0.0689362	sex
0.64049	0.08510118	sex
0.23351	0.06313731	sex
0.66191	0.08047565	sex
0.29841	0.05570969	sex
0.72681	0.07479024	sex
0.22859	0.0359	sex
0.65699	0.06147211	sex
0.09	0.05380009	sex
0.3784	0.06321938	sex
0.24098	0.04333405	sex
0.52938	0.05459011	sex
0.34122	0.03908248	sex
0.62962	0.05128041	sex
0.31449	0.03401955	sex
0.60289	0.04753494	sex
0.19151	0.0217	sex
0.47991	0.0396627	sex
0.1342	0.05814061	sex
0.2125	0.06431625	sex

For peer review only

0.30216	0.05340721	sex
0.38046	0.06007146	sex
0.32624	0.05016503	sex
0.40454	0.05720822	sex
0.28752	0.04471107	sex
0.36582	0.05249124	sex
0.14248	0.0288	sex
0.22078	0.03982072	sex
0.0463	0.11944756	sex
0.2556	0.14753739	sex
0.16267	0.08567152	sex
0.37197	0.12181613	sex
0.20203	0.07624756	sex
0.41133	0.11538306	sex
0.26424	0.067184	sex
0.47354	0.10960497	sex
0.35176	0.0435	sex
0.56106	0.09691135	sex
0.0566	0.10083938	sex
0.5597	0.11443509	sex
0.07494	0.07648091	sex
0.57804	0.09368106	sex
0.20836	0.0669517	sex
0.71146	0.08607752	sex
0.24605	0.05708319	sex
0.74915	0.07864668	sex
0.21295	0.036	sex
0.71605	0.06498315	sex
-0.0204	0.06123961	sex
0.3436	0.0839348	sex
0.0138	0.03851493	sex
0.3778	0.06912424	sex
-0.0121	0.03199766	sex
0.3519	0.06571613	sex
0.00148	0.02628555	sex
0.36548	0.06313232	sex
-0.05008	0.0162	sex
0.31392	0.05964227	sex
0.2334	0.05439017	sex
0.4471	0.06054626	sex
-0.00426	0.03857836	sex
0.20944	0.0468599	sex
-0.04284	0.03175358	sex

For peer review only

0.17086	0.04142282	sex
-0.05733	0.02504157	sex
0.15637	0.03653273	sex
-0.07167	0.0148	sex
0.14203	0.03044011	sex
0.1651	0.06660068	sex
0.4328	0.08382398	sex
0.26358	0.05436543	sex
0.53128	0.07447422	sex
0.32482	0.04934744	sex
0.59252	0.07089415	sex
0.31811	0.04296941	sex
0.58581	0.06661216	sex
0.15489	0.0274	sex
0.42259	0.05780632	sex
0.0712	0.06570723	sex
0.2222	0.07531056	sex
0.33121	0.06062087	sex
0.48221	0.07091636	sex
0.34719	0.05675509	sex
0.49819	0.06764156	sex
0.2721	0.04955492	sex
0.4231	0.06172463	sex
0.0939	0.0312	sex
0.2449	0.04824604	sex
0.1428	0.05564243	sex
0.4392	0.06242572	sex
0.09951	0.03563888	sex
0.39591	0.04550846	sex
0.00349	0.02903877	sex
0.29989	0.040548	sex
-0.01481	0.02340086	sex
0.28159	0.03672179	sex
-0.00509	0.0142	sex
0.29131	0.03166275	sex
0.1333	0.0426169	sex
0.2957	0.05508366	sex
0.12058	0.03215043	sex
0.28298	0.04745166	sex
0.05862	0.02717076	sex
0.22102	0.04422963	sex
0.04294	0.02212058	sex
0.20534	0.04131985	sex

For peer review only

-0.00794	0.0134	sex
0.15446	0.03738409	sex
0.2205	0.08554093	sex
0.4248	0.11382996	sex
0.30273	0.06506804	sex
0.50703	0.0993673	sex
0.43187	0.05910135	sex
0.63617	0.09556663	sex
0.39794	0.05229197	sex
0.60224	0.09151208	sex
0.31406	0.0338	sex
0.51836	0.08235563	sex
-0.0041	0.07139811	sex
0.2639	0.07964785	sex
0.19374	0.05412079	sex
0.46174	0.0646154	sex
0.32766	0.04822499	sex
0.59566	0.05976404	sex
0.26532	0.04144937	sex
0.53332	0.05444392	sex
0.18468	0.0261	sex
0.45268	0.04390103	sex
0.2305	0.05014619	sex
0.5794	0.06058581	sex
0.41256	0.03332942	sex
0.76146	0.04761145	sex
0.47224	0.02760525	sex
0.82114	0.04379555	sex
0.49211	0.02277016	sex
0.84101	0.04092041	sex
0.36289	0.0142	sex
0.71179	0.03684617	sex
0.2925	0.05283418	sex
0.4593	0.07205866	sex
0.4541	0.0447464	sex
0.6209	0.06635691	sex
0.5249	0.04029404	sex
0.6917	0.06343981	sex
0.58498	0.03475068	sex
0.75178	0.06007171	sex
0.44502	0.022	sex
0.61182	0.0537122	sex
-0.2618	0.06322001	sex

For peer review only

-0.5517	0.07320096	sex
-0.07632	0.05091572	sex
-0.36622	0.062881	sex
0.06462	0.04547571	sex
-0.22528	0.05856321	sex
0.14991	0.03869987	sex
-0.13999	0.05347233	sex
0.17109	0.0242	sex
-0.11881	0.04412766	sex
-0.2739	0.05003729	sex
-0.4328	0.07274318	sex
-0.08617	0.04346815	sex
-0.24507	0.06839094	sex
-0.05313	0.03850519	sex
-0.21203	0.06534899	sex
0.02599	0.03124052	sex
-0.13291	0.0613499	sex
0.02101	0.0186	sex
-0.13789	0.05598035	sex
0.3032	0.06371224	sex
0.4104	0.08281099	sex
0.31114	0.03845101	sex
0.41834	0.06539794	sex
0.35236	0.03070505	sex
0.45956	0.06116543	sex
0.44923	0.0243403	sex
0.55643	0.05823109	sex
0.36777	0.0147	sex
0.47497	0.05490446	sex
0.3316	0.05218448	sex
0.4877	0.09054954	sex
0.31704	0.03596804	sex
0.47314	0.08227819	sex
0.31066	0.02934706	sex
0.46676	0.07960685	sex
0.34669	0.02328003	sex
0.50279	0.07757551	sex
0.24631	0.014	sex
0.40241	0.07531268	sex
-0.1956	0.07518224	sex
-0.2903	0.08968601	sex
-0.03153	0.06404662	sex
-0.12623	0.08058027	sex

For peer review only

0.03403	0.05848248	sex
-0.06067	0.0762326	sex
-0.00971	0.05047653	sex
-0.10441	0.07027866	sex
-0.14029	0.0318	sex
-0.23499	0.05833052	sex
-0.2303	0.06915273	sex
-0.3501	0.07430976	sex
-0.08025	0.062476	sex
-0.20005	0.06814022	sex
-0.02545	0.05721888	sex
-0.14525	0.06335487	sex
-0.12188	0.04857201	sex
-0.24168	0.05566938	sex
-0.24212	0.03	sex
-0.36192	0.04049494	sex
-0.0393	0.09982991	sex
0.0532	0.11762164	sex
0.11241	0.09082819	sex
0.20491	0.11008451	sex
0.20199	0.08451485	sex
0.29449	0.10493617	sex
0.20731	0.07349803	sex
0.29981	0.09628499	sex
0.03569	0.0464	sex
0.12819	0.07760026	sex
0.0683	0.08508049	sex
0.0019	0.08770621	sex
0.23515	0.08252448	sex
0.16875	0.08522899	sex
0.27575	0.07985193	sex
0.20935	0.08264394	sex
0.09764	0.07308522	sex
0.03124	0.07612582	sex
0.07036	0.0479	sex
0.00396	0.05242232	sex
-0.5393	0.11700107	sex
-0.5193	0.18260693	sex
-0.46406	0.09395983	sex
-0.44406	0.16877349	sex
-0.42934	0.08468353	sex
-0.40934	0.16379054	sex
-0.41551	0.07351959	sex

For peer review only

-0.39551	0.1583072	sex
-0.35449	0.0468	sex
-0.33449	0.14780487	sex
-0.4889	0.10541395	sex
-0.3723	0.14325188	sex
-0.41942	0.09095257	sex
-0.30282	0.13297131	sex
-0.33818	0.08223169	sex
-0.22158	0.12716544	sex
-0.46189	0.07007517	sex
-0.34529	0.11966424	sex
-0.49511	0.0438	sex
-0.37851	0.10643045	sex
-0.1183	0.08303987	sex
0.0386	0.12700721	sex
-0.07091	0.05568357	sex
0.08599	0.11106696	sex
0.01491	0.04686801	sex
0.17181	0.10691969	sex
0.07947	0.0391372	sex
0.23637	0.10376382	sex
-0.06007	0.0244	sex
0.09683	0.09914923	sex
0.0327	0.07971976	sex
0.2591	0.09573004	sex
0.00919	0.05597857	sex
0.23559	0.07708826	sex
0.00311	0.04642629	sex
0.22951	0.0704585	sex
-0.05197	0.03734019	sex
0.17443	0.06483279	sex
-0.12703	0.0225	sex
0.09937	0.05757821	sex
-0.2104	0.10769406	sex
-0.0658	0.12466696	sex
-0.02535	0.08966878	sex
0.11925	0.10947297	sex
0.13495	0.08231555	sex
0.27955	0.10353594	sex
0.08463	0.07241768	sex
0.22923	0.09585489	sex
0.17037	0.0464	sex
0.31497	0.07808201	sex

For peer review only

-0.3939	0.11118786	sex
-0.1367	0.13656951	sex
-0.14805	0.09946788	sex
0.10915	0.12720987	sex
0.09315	0.09270394	sex
0.35035	0.12199389	sex
0.07863	0.08167509	sex
0.33583	0.11383897	sex
0.04037	0.0521	sex
0.29757	0.09488361	sex
0.3881	0.11145169	sex
0.7595	0.12380101	sex
0.10496	0.0595336	sex
0.47636	0.08030853	sex
0.19824	0.04270012	sex
0.56964	0.06876416	sex
0.26099	0.03149175	sex
0.63239	0.06242548	sex
0.19801	0.0182	sex
0.56941	0.05688981	sex
0.1976	0.08016433	sex
0.2885	0.10220509	sex
0.21587	0.05476422	sex
0.30677	0.08377756	sex
0.16693	0.04202154	sex
0.25783	0.07606162	sex
0.27575	0.03115542	sex
0.36665	0.07064149	sex
0.29525	0.0179	sex
0.38615	0.06587845	sex
0.0864	0.09662877	sex
0.3081	0.12764278	sex
0.10234	0.06998543	sex
0.32404	0.10887387	sex
0.03256	0.05864094	sex
0.25426	0.10195254	sex
0.03636	0.04724108	sex
0.25806	0.0958503	sex
-0.00546	0.0286	sex
0.21624	0.08816757	sex
0.0774	0.08867632	sex
0.1963	0.11646347	sex
0.12696	0.07431776	sex

For peer review only

0.24586	0.10594046	sex
0.00024	0.06468879	sex
0.11914	0.09942278	sex
-0.09527	0.05256006	sex
0.02363	0.09199353	sex
-0.21273	0.0316	sex
-0.09383	0.08184626	sex
0.1092	0.09970381	sex
0.2449	0.10886019	sex
0.36925	0.06994262	sex
0.50495	0.08247218	sex
0.45645	0.06216189	sex
0.59215	0.07598546	sex
0.47805	0.05469963	sex
0.61375	0.07001243	sex
0.38195	0.0354	sex
0.51765	0.05623922	sex
0.168	0.10231134	sex
0.1898	0.11886068	sex
0.40739	0.08727296	sex
0.42919	0.10619237	sex
0.57661	0.07957047	sex
0.59841	0.09995854	sex
0.52262	0.06895288	sex
0.54442	0.09173195	sex
0.57738	0.0435	sex
0.59918	0.0745151	sex
0.2617	0.10522001	sex
0.4025	0.10893342	sex
0.28652	0.07325169	sex
0.42732	0.07849236	sex
0.37488	0.0599257	sex
0.51568	0.06622937	sex
0.39694	0.04680385	sex
0.53774	0.05464284	sex
0.35606	0.0276	sex
0.49686	0.03945884	sex
0.1237	0.07514153	sex
0.2157	0.08389005	sex
0.30682	0.05879907	sex
0.39882	0.06963203	sex
0.36828	0.04970392	sex
0.46028	0.06214314	sex

For peer review only

0.36265	0.03948164	sex
0.45465	0.05431473	sex
0.33635	0.0234	sex
0.42835	0.04403237	sex
-0.2562	0.08041673	sex
-0.0784	0.09380693	sex
-0.1698	0.05976788	sex
0.008	0.07684458	sex
0.1059	0.04947727	sex
0.2837	0.06914398	sex
0.37286	0.03829204	sex
0.55066	0.06163741	sex
0.34514	0.0222	sex
0.52294	0.0531576	sex
-0.1956	0.08477317	sex
-0.3519	0.10322645	sex
-0.11854	0.07733104	sex
-0.27484	0.09720751	sex
-0.06236	0.07002742	sex
-0.21866	0.09150437	sex
0.0723	0.05655449	sex
-0.084	0.0816555	sex
0.1417	0.0329	sex
-0.0146	0.0674657	sex
NA	NA	sex
NA	NA	sex
-0.08219	0.6105647	sex
1.07051	0.88684581	sex
0.37979	0.51015488	sex
1.53249	0.82095325	sex
0.66213	0.4400659	sex
1.81483	0.77933577	sex
0.78787	0.283	sex
1.94057	0.70270566	sex
0.2888	0.09060155	sex
1.4375	0.11723481	sex
0.23679	0.05530145	sex
1.38549	0.09270173	sex
0.12331	0.0426522	sex
1.27201	0.08575879	sex
0.18162	0.0330601	sex
1.33032	0.08141456	sex
0.21438	0.0199	sex

For peer review only

	1.36308	0.07701539	sex
	0.205	0.1078416	sex
	0.9037	0.12701268	sex
	0.3432	0.08165886	sex
	1.0419	0.10569097	sex
	0.1078	0.07402297	sex
	0.8065	0.09990901	sex
	-0.0825	0.06728358	sex
	0.6162	0.09502363	sex
	-0.0995	0.0448	sex
	0.5992	0.08068116	sex
NA		0	sex
NA		0	sex
NA	NA		sex
NA	NA		sex
NA	NA		sex
NA	NA		sex
NA	NA		sex
NA	NA		sex
NA	NA		sex
NA	NA		sex
	0.1939	0.32876527	sex
	0.1562	0.42536174	sex
	0.21645	0.29750355	sex
	0.17875	0.4016894	sex
	0.48805	0.27613403	sex
	0.45035	0.38612953	sex
	0.50373	0.23754158	sex
	0.46603	0.35954417	sex
	0.21327	0.149	sex
	0.17557	0.30829695	sex
	-0.0443	0.37164957	sex
	0.0197	0.38792891	sex
	0.3435	0.35753512	sex
	0.4075	0.37442863	sex
	0.5937	0.34311077	sex
	0.6577	0.36068052	sex
	0.30487	0.30473103	sex
	0.36887	0.32438625	sex
	0.11713	0.194	sex
	0.18113	0.22361002	sex
	-0.028	0.08290006	deprivation
	-0.028	0.08290006	deprivation

For peer review only

0.235	0.07948899	deprivation
0.235	0.07948899	deprivation
0.335	0.07590843	deprivation
0.335	0.07590843	deprivation
0.393	0.06824691	deprivation
0.393	0.06824691	deprivation
0.33	0.0442	deprivation
0.33	0.0442	deprivation
-0.063	0.07682942	deprivation
-0.063	0.07682942	deprivation
0.155	0.07298712	deprivation
0.155	0.07298712	deprivation
0.321	0.06926507	deprivation
0.321	0.06926507	deprivation
0.346	0.06214	deprivation
0.346	0.06214	deprivation
0.297	0.0403	deprivation
0.297	0.0403	deprivation
-0.101	0.07286289	deprivation
-0.101	0.07286289	deprivation
0.131	0.06944955	deprivation
0.131	0.06944955	deprivation
0.343	0.06600947	deprivation
0.343	0.06600947	deprivation
0.332	0.0591684	deprivation
0.332	0.0591684	deprivation
0.305	0.0383	deprivation
0.305	0.0383	deprivation
-0.106	0.0693334	deprivation
-0.106	0.0693334	deprivation
0.144	0.06570396	deprivation
0.144	0.06570396	deprivation
0.299	0.06232985	deprivation
0.299	0.06232985	deprivation
0.299	0.0559683	deprivation
0.299	0.0559683	deprivation
0.274	0.0363	deprivation
0.274	0.0363	deprivation
-0.093	0.06573165	deprivation
-0.093	0.06573165	deprivation
0.102	0.06209702	deprivation
0.102	0.06209702	deprivation
0.312	0.05886459	deprivation

For peer review only

0.312	0.05886459	deprivation
0.299	0.05277954	deprivation
0.299	0.05277954	deprivation
0.281	0.0342	deprivation
0.281	0.0342	deprivation
-0.039	0.06313113	deprivation
-0.039	0.06313113	deprivation
0.144	0.05912783	deprivation
0.144	0.05912783	deprivation
0.298	0.05596472	deprivation
0.298	0.05596472	deprivation
0.314	0.05008992	deprivation
0.314	0.05008992	deprivation
0.326	0.0324	deprivation
0.326	0.0324	deprivation
-0.03	0.05924264	deprivation
-0.03	0.05924264	deprivation
0.121	0.0553104	deprivation
0.121	0.0553104	deprivation
0.23	0.05228508	deprivation
0.23	0.05228508	deprivation
0.333	0.04682499	deprivation
0.333	0.04682499	deprivation
0.236	0.0303	deprivation
0.236	0.0303	deprivation
-0.047	0.05607424	deprivation
-0.047	0.05607424	deprivation
0.137	0.05191724	deprivation
0.137	0.05191724	deprivation
0.247	0.04889305	deprivation
0.247	0.04889305	deprivation
0.28	0.04357178	deprivation
0.28	0.04357178	deprivation
0.279	0.0281	deprivation
0.279	0.0281	deprivation
-0.071	0.05376365	deprivation
-0.071	0.05376365	deprivation
0.117	0.04909358	deprivation
0.117	0.04909358	deprivation
0.224	0.04592527	deprivation
0.224	0.04592527	deprivation
0.271	0.04067739	deprivation
0.271	0.04067739	deprivation

For peer review only

0.339	0.0261	deprivation
0.339	0.0261	deprivation
-0.049	0.05105468	deprivation
-0.049	0.05105468	deprivation
0.018	0.04542797	deprivation
0.018	0.04542797	deprivation
0.198	0.0418336	deprivation
0.198	0.0418336	deprivation
0.315	0.03651685	deprivation
0.315	0.03651685	deprivation
0.34	0.0232	deprivation
0.34	0.0232	deprivation
0.03726323	0.03888358	deprivation
0.2578384	0.05060186	deprivation
0.17098525	0.03167267	deprivation
0.39156042	0.04529651	deprivation
0.15179069	0.02857175	deprivation
0.37236587	0.04318519	deprivation
0.16052393	0.0247997	deprivation
0.38109911	0.04078775	deprivation
0.05626703	0.0158493	deprivation
0.27699293	0.03602387	deprivation
-0.0016554	0.03729818	deprivation
0.15731228	0.0469925	deprivation
0.16232845	0.02926308	deprivation
0.3212961	0.04090805	deprivation
0.19329026	0.02607073	deprivation
0.35225792	0.03868881	deprivation
0.1507855	0.02246522	deprivation
0.30975316	0.03635694	deprivation
0.07356991	0.0143283	deprivation
0.23065138	0.03195262	deprivation
-0.0173544	0.03624262	deprivation
0.10126803	0.04844415	deprivation
0.14989213	0.0292034	deprivation
0.26972709	0.04338	deprivation
0.19420497	0.02623974	deprivation
0.31324796	0.04144652	deprivation
0.16273094	0.02265466	deprivation
0.28135341	0.03932609	deprivation
0.1116226	0.01437945	deprivation
0.23024507	0.03521472	deprivation
0.01846075	0.0358598	deprivation

For peer review only

0.19798335	0.04439087	deprivation
0.16170411	0.02835109	deprivation
0.34073703	0.03863039	deprivation
0.20790548	0.02547151	deprivation
0.3869384	0.0365694	deprivation
0.18676172	0.02216746	deprivation
0.36498652	0.03431066	deprivation
0.13407451	0.01414644	deprivation
0.30993493	0.02981663	deprivation
0.03445481	0.03432329	deprivation
0.20751869	0.04256493	deprivation
0.1072803	0.02728864	deprivation
0.27833428	0.03709035	deprivation
0.19873836	0.02452019	deprivation
0.37180224	0.0351415	deprivation
0.16728422	0.0212511	deprivation
0.3403481	0.03294381	deprivation
0.10793977	0.01352545	deprivation
0.28100365	0.02857662	deprivation
0.02169445	0.03410132	deprivation
0.21393247	0.04233349	deprivation
0.13264539	0.02634128	deprivation
0.32487562	0.03637821	deprivation
0.19464855	0.02360134	deprivation
0.38588278	0.034449	deprivation
0.1716582	0.02045723	deprivation
0.36464378	0.03238299	deprivation
0.13160582	0.01302283	deprivation
0.32409864	0.02829663	deprivation
0.02543045	0.03234132	deprivation
0.20639272	0.03975699	deprivation
0.12899493	0.0250878	deprivation
0.30983176	0.03412178	deprivation
0.15846557	0.02249894	deprivation
0.33924828	0.03226935	deprivation
0.17979233	0.0195015	deprivation
0.36119069	0.03026237	deprivation
0.12400393	0.01240865	deprivation
0.30555543	0.02627356	deprivation
0.05480491	0.03110766	deprivation
0.26454244	0.03742956	deprivation
0.11638762	0.02412038	deprivation
0.32612515	0.0318603	deprivation

For peer review only

0.17099511	0.02157485	deprivation
0.38073264	0.02997932	deprivation
0.19125794	0.01866584	deprivation
0.40099548	0.02795889	deprivation
0.11880491	0.01186415	deprivation
0.32854244	0.02395921	deprivation
-0.072566	0.03183265	deprivation
0.08936119	0.03968441	deprivation
0.11338274	0.02353432	deprivation
0.27530991	0.03339759	deprivation
0.14456694	0.02086118	deprivation
0.30649411	0.03157093	deprivation
0.15054276	0.01793074	deprivation
0.31246993	0.02971609	deprivation
0.12725103	0.01134615	deprivation
0.2891782	0.02627299	deprivation
-0.0107495	0.03181105	deprivation
0.18622076	0.03943098	deprivation
0.03414097	0.02271102	deprivation
0.23111128	0.0325369	deprivation
0.13004237	0.01969557	deprivation
0.32701268	0.0305086	deprivation
0.17431774	0.01670215	deprivation
0.37128805	0.02866742	deprivation
0.14732794	0.01046898	deprivation
0.34429825	0.02554327	deprivation
-0.25274	0.17764743	deprivation
-0.33384	0.2799778	deprivation
-0.1329	0.16403478	deprivation
-0.214	0.27154442	deprivation
-0.09771	0.15336509	deprivation
-0.17881	0.26523539	deprivation
-0.196809	0.13416199	deprivation
-0.277909	0.25461422	deprivation
-0.297931	0.085	deprivation
-0.379031	0.23249508	deprivation
-0.28315	0.16887303	deprivation
-0.09715	0.21446431	deprivation
-0.12495	0.15421161	deprivation
0.06105	0.2031208	deprivation
-0.04126	0.1435424	deprivation
0.14474	0.1951442	deprivation
-0.243141	0.12533898	deprivation

For peer review only

-0.057141	0.18217217	deprivation
-0.337499	0.0795	deprivation
-0.151499	0.15426306	deprivation
-0.20366	0.15805417	deprivation
-0.15026	0.2939992	deprivation
-0.15718	0.14522896	deprivation
-0.10378	0.28730795	deprivation
-0.07526	0.13505295	deprivation
-0.02186	0.28230074	deprivation
-0.125819	0.11749404	deprivation
-0.072419	0.27433421	deprivation
-0.192171	0.0742	deprivation
-0.138771	0.2587664	deprivation
-0.23646	0.15257949	deprivation
-0.25376	0.20741444	deprivation
0.01704	0.13935631	deprivation
-0.00026	0.19788994	deprivation
-0.11377	0.12994191	deprivation
-0.13107	0.19137698	deprivation
-0.181218	0.11394148	deprivation
-0.198518	0.18089475	deprivation
-0.205792	0.0725	deprivation
-0.223092	0.15810282	deprivation
-0.14255	0.14431732	deprivation
0.05345	0.18852369	deprivation
-0.15324	0.13108886	deprivation
0.04276	0.17860006	deprivation
-0.06779	0.12180858	deprivation
0.12821	0.1719041	deprivation
-0.156126	0.10625032	deprivation
0.039874	0.1612539	deprivation
-0.229254	0.0672	deprivation
-0.033254	0.13867058	deprivation
-0.23769	0.13830622	deprivation
0.08331	0.21164274	deprivation
-0.09018	0.12416268	deprivation
0.23082	0.20268303	deprivation
-0.11295	0.11502074	deprivation
0.20805	0.19721514	deprivation
-0.205507	0.10001685	deprivation
0.115493	0.18885817	deprivation
-0.154313	0.0631	deprivation
0.166687	0.17217912	deprivation

For peer review only

-0.14491	0.1262555	deprivation
0.04609	0.14119026	deprivation
-0.08373	0.1132015	deprivation
0.10727	0.12964883	deprivation
-0.17946	0.10462094	deprivation
0.01154	0.12222839	deprivation
-0.184831	0.09129211	deprivation
0.006169	0.11103373	deprivation
-0.188179	0.058	deprivation
0.002821	0.08578018	deprivation
-0.16189	0.12234889	deprivation
0.02811	0.14960769	deprivation
-0.16481	0.10881516	deprivation
0.02519	0.1387586	deprivation
-0.09828	0.10065237	deprivation
0.09172	0.13245418	deprivation
-0.173728	0.0879501	deprivation
0.016272	0.12307896	deprivation
-0.263072	0.0559	deprivation
-0.073072	0.10265486	deprivation
-0.25883	0.11912565	deprivation
-0.15283	0.15598529	deprivation
-0.16269	0.10402557	deprivation
-0.05669	0.14478194	deprivation
-0.21843	0.09556003	deprivation
-0.11243	0.13882439	deprivation
-0.217941	0.08296174	deprivation
-0.111941	0.13047276	deprivation
-0.173989	0.0523	deprivation
-0.067989	0.11347149	deprivation
-0.1645	0.11422946	deprivation
0.0355	0.13314252	deprivation
-0.26477	0.09747702	deprivation
-0.06477	0.11908119	deprivation
-0.13044	0.08822902	deprivation
0.06956	0.11163745	deprivation
-0.183303	0.07568197	deprivation
0.016697	0.10201137	deprivation
-0.196577	0.0474	deprivation
0.003423	0.08321851	deprivation
0.02092	0.11756807	deprivation
0.20292	0.19152987	deprivation
0.10596	0.0876797	deprivation

For peer review only

0.28796	0.17478321	deprivation
0.14444	0.07307072	deprivation
0.32644	0.16793085	deprivation
0.276299	0.05836	deprivation
0.458299	0.16207199	deprivation
0.052211	0.035	deprivation
0.234211	0.15519807	deprivation
-0.12135	0.11346515	deprivation
0.11865	0.16119736	deprivation
0.08422	0.08085753	deprivation
0.32422	0.140172	deprivation
0.21745	0.06611036	deprivation
0.45745	0.13221509	deprivation
0.183291	0.05184139	deprivation
0.423291	0.12568922	deprivation
0.100679	0.0308	deprivation
0.340679	0.11857019	deprivation
0.00995	0.10906218	deprivation
0.24995	0.16024144	deprivation
0.10814	0.08009994	deprivation
0.34814	0.14212234	deprivation
0.20023	0.06628612	deprivation
0.44023	0.13482066	deprivation
0.191618	0.05246046	deprivation
0.431618	0.12858795	deprivation
0.072652	0.0313	deprivation
0.312652	0.12150082	deprivation
-0.06407	0.10938944	deprivation
0.21493	0.14746952	deprivation
0.0683	0.07967748	deprivation
0.3473	0.1270028	deprivation
0.18082	0.06592738	deprivation
0.45982	0.11885971	deprivation
0.222224	0.05212111	deprivation
0.501224	0.11179365	deprivation
0.069536	0.031	deprivation
0.348536	0.10364463	deprivation
0.02566	0.10337626	deprivation
0.05226	0.13485626	deprivation
0.07666	0.07623359	deprivation
0.10326	0.11537383	deprivation
0.17937	0.06350717	deprivation
0.20597	0.1073905	deprivation

0.241856	0.05020159	deprivation
0.268456	0.10009875	deprivation
0.044334	0.0298	deprivation
0.070934	0.09158384	deprivation
0.00207	0.10365259	deprivation
0.22207	0.13945878	deprivation
0.09594	0.07304752	deprivation
0.31594	0.11849401	deprivation
0.21254	0.05989733	deprivation
0.43254	0.11087191	deprivation
0.228018	0.04726108	deprivation
0.448018	0.10458728	deprivation
0.091182	0.0281	deprivation
0.311182	0.09743973	deprivation
0.00292	0.09791364	deprivation
0.19292	0.11681083	deprivation
0.1214	0.06884715	deprivation
0.3114	0.09379563	deprivation
0.20566	0.05649186	deprivation
0.39566	0.08514118	deprivation
0.265992	0.04454088	deprivation
0.455992	0.0777276	deprivation
0.207888	0.0265	deprivation
0.397888	0.06899232	deprivation
-0.04213	0.09485067	deprivation
0.25287	0.11872072	deprivation
0.07058	0.06559642	deprivation
0.36558	0.09695798	deprivation
0.1935	0.05371276	deprivation
0.4885	0.08934775	deprivation
0.302638	0.04234171	deprivation
0.597638	0.08301072	deprivation
0.169582	0.0251	deprivation
0.464582	0.07568335	deprivation
-0.19682	0.09820082	deprivation
-0.00782	0.11430507	deprivation
0.10251	0.06443757	deprivation
0.29151	0.08703132	deprivation
0.22646	0.05197548	deprivation
0.41546	0.07825407	deprivation
0.28041	0.04032679	deprivation
0.46941	0.0710528	deprivation
0.21841	0.0236	deprivation

0.40741	0.06308098	deprivation
-0.11833	0.09849391	deprivation
0.22567	0.13281514	deprivation
0.04859	0.06342886	deprivation
0.39259	0.10937107	deprivation
0.24401	0.04980382	deprivation
0.58801	0.10207463	deprivation
0.295551	0.03803709	deprivation
0.639551	0.09687946	deprivation
0.223149	0.0219	deprivation
0.567149	0.09175195	deprivation
-0.36311	0.30365759	deprivation
-0.14511	0.37852864	deprivation
0.27449	0.23744374	deprivation
0.49249	0.3278041	deprivation
0.25447	0.21962372	deprivation
0.47247	0.31513581	deprivation
0.473859	0.1990566	deprivation
0.691859	0.30116363	deprivation
0.226781	0.1312	deprivation
0.444781	0.26132248	deprivation
-0.16222	0.28761956	deprivation
-0.00822	0.34235873	deprivation
0.11578	0.20702437	deprivation
0.26978	0.27810714	deprivation
0.36382	0.18692386	deprivation
0.51782	0.26348628	deprivation
0.351074	0.16768357	deprivation
0.505074	0.25020446	deprivation
0.454386	0.1103	deprivation
0.608386	0.21598745	deprivation
-0.13592	0.26939029	deprivation
0.20608	0.32550026	deprivation
0.20359	0.20094228	deprivation
0.54559	0.27158257	deprivation
0.26622	0.18280823	deprivation
0.60822	0.25845336	deprivation
0.52238	0.16368729	deprivation
0.86438	0.24530149	deprivation
0.50519	0.1072	deprivation
0.84719	0.21182807	deprivation
-0.36573	0.27276657	deprivation
0.02127	0.33930052	deprivation

For peer review only

0.14385	0.19190383	deprivation
0.53085	0.27847858	deprivation
0.36827	0.17478181	deprivation
0.75527	0.26696801	deprivation
0.405984	0.15841869	deprivation
0.792984	0.25655354	deprivation
0.772006	0.1048	deprivation
1.159006	0.22739015	deprivation
0.15615	0.24626492	deprivation
0.27215	0.28944917	deprivation
0.17237	0.17745411	deprivation
0.28837	0.23371857	deprivation
0.30958	0.16148721	deprivation
0.42558	0.22183897	deprivation
0.369006	0.14576478	deprivation
0.485006	0.21066984	deprivation
0.455934	0.0961	deprivation
0.571934	0.17991559	deprivation
-0.37247	0.25030663	deprivation
-0.03347	0.27401763	deprivation
0.07421	0.17170105	deprivation
0.41321	0.20472787	deprivation
0.33879	0.1570796	deprivation
0.67779	0.19262983	deprivation
0.400848	0.14322025	deprivation
0.739848	0.18150562	deprivation
0.611222	0.0952	deprivation
0.950222	0.14661272	deprivation
-0.37855	0.23029876	deprivation
-0.48855	0.26343107	deprivation
0.13657	0.15650853	deprivation
0.02657	0.20212207	deprivation
0.27056	0.14243936	deprivation
0.16056	0.19143505	deprivation
0.391158	0.12936035	deprivation
0.281158	0.18191347	deprivation
0.426712	0.0857	deprivation
0.316712	0.15395746	deprivation
-0.03347	0.20870872	deprivation
0.19053	0.24190209	deprivation
0.1025	0.14646754	deprivation
0.3265	0.19081412	deprivation
0.29439	0.13282428	deprivation

For peer review only

0.51839	0.18055354	deprivation
0.395962	0.12023431	deprivation
0.619962	0.17150388	deprivation
0.421288	0.0795	deprivation
0.645288	0.14586823	deprivation
-0.52792	0.21318924	deprivation
-0.26792	0.25598543	deprivation
0.10558	0.13441075	deprivation
0.36558	0.19530781	deprivation
0.20351	0.12046327	deprivation
0.46351	0.18598465	deprivation
0.294619	0.10807946	deprivation
0.554619	0.17821352	deprivation
0.420801	0.0711	deprivation
0.680801	0.15853738	deprivation
-0.3745	0.20156113	deprivation
-0.1855	0.23371303	deprivation
-0.05072	0.12298167	deprivation
0.13828	0.17064402	deprivation
0.18749	0.10704252	deprivation
0.37649	0.15953993	deprivation
0.268954	0.09480955	deprivation
0.457954	0.15160389	deprivation
0.414586	0.0618	deprivation
0.603586	0.13346959	deprivation
0.20386	0.13707939	deprivation
0.53086	0.17873682	deprivation
0.15831	0.09995379	deprivation
0.48531	0.15214089	deprivation
0.09563	0.08124463	deprivation
0.42263	0.14055881	deprivation
0.120298	0.06384113	deprivation
0.447298	0.13126988	deprivation
-0.050158	0.038	deprivation
0.276842	0.12083083	deprivation
0.22686	0.1301624	deprivation
0.15616	0.15246839	deprivation
0.17429	0.0919557	deprivation
0.10359	0.12149161	deprivation
0.10994	0.07379221	deprivation
0.03924	0.1083958	deprivation
0.118018	0.05702359	deprivation
0.047318	0.09775505	deprivation

For peer review only

0.016632	0.0337	deprivation
-0.054068	0.08625572	deprivation
0.09969	0.12529585	deprivation
0.01849	0.21620705	deprivation
0.17384	0.09208692	deprivation
0.09264	0.19881258	deprivation
0.19065	0.07496372	deprivation
0.10945	0.19148368	deprivation
0.137405	0.05806729	deprivation
0.056205	0.18552156	deprivation
0.081915	0.0341	deprivation
0.000715	0.17946936	deprivation
0.18718	0.12500292	deprivation
0.35818	0.15391481	deprivation
0.20175	0.0904339	deprivation
0.37275	0.1274454	deprivation
0.2461	0.07401223	deprivation
0.4171	0.11636946	deprivation
0.144978	0.05792167	deprivation
0.315978	0.10685953	deprivation
0.054972	0.0344	deprivation
0.225972	0.0961634	deprivation
0.13867	0.11992664	deprivation
0.15987	0.13698482	deprivation
0.09192	0.08640185	deprivation
0.11312	0.10884723	deprivation
0.21039	0.07052943	deprivation
0.23159	0.09673076	deprivation
0.195739	0.05508285	deprivation
0.216939	0.08611945	deprivation
0.050801	0.0326	deprivation
0.072001	0.0737916	deprivation
0.30937	0.11385298	deprivation
0.46137	0.16055136	deprivation
0.2395	0.08194809	deprivation
0.3915	0.13974881	deprivation
0.20023	0.06681377	deprivation
0.35223	0.13144702	deprivation
0.160791	0.05236258	deprivation
0.312791	0.12472402	deprivation
0.085889	0.031	deprivation
0.237889	0.11736797	deprivation
0.19817	0.11163409	deprivation

For peer review only

0.34617	0.13171249	deprivation
0.17451	0.07952767	deprivation
0.32251	0.1058804	deprivation
0.17351	0.06501946	deprivation
0.32151	0.09546486	deprivation
0.127333	0.05102401	deprivation
0.275333	0.08654167	deprivation
0.077947	0.0301	deprivation
0.225947	0.07610532	deprivation
0.16015	0.11017645	deprivation
0.31815	0.15512411	deprivation
0.23288	0.0767918	deprivation
0.39088	0.13349764	deprivation
0.20888	0.06233177	deprivation
0.36688	0.12573739	deprivation
0.129992	0.04874474	deprivation
0.287992	0.11958549	deprivation
0.081818	0.0287	deprivation
0.239818	0.1129085	deprivation
-0.00856	0.1117775	deprivation
0.08614	0.18032321	deprivation
0.14075	0.07560403	deprivation
0.23545	0.16043136	deprivation
0.17524	0.06100262	deprivation
0.26994	0.15408949	deprivation
0.129073	0.04734818	deprivation
0.223773	0.1492116	deprivation
0.086667	0.0277	deprivation
0.181367	0.14418578	deprivation
0.11311	0.11415078	deprivation
0.27611	0.14184513	deprivation
0.1499	0.07497093	deprivation
0.3129	0.11273988	deprivation
0.13599	0.05884633	deprivation
0.29899	0.10272551	deprivation
0.165848	0.04491882	deprivation
0.328848	0.09543238	deprivation
0.094942	0.0259	deprivation
0.257942	0.08809342	deprivation
0.0698	0.13078964	deprivation
0.1304	0.18293261	deprivation
0.33378	0.11716868	deprivation
0.39438	0.17345579	deprivation

For peer review only

0.16848	0.10695429	deprivation
0.22908	0.16672621	deprivation
0.032505	0.0894634	deprivation
0.093105	0.15608366	deprivation
-0.078705	0.0543	deprivation
-0.018105	0.13894927	deprivation
0.09048	0.12288181	deprivation
-0.03552	0.1654587	deprivation
0.24078	0.10813482	deprivation
0.11478	0.15482177	deprivation
0.15178	0.09757177	deprivation
0.02578	0.1476377	deprivation
0.097603	0.0810432	deprivation
-0.028397	0.13727578	deprivation
-0.070783	0.0492	deprivation
-0.196783	0.12123234	deprivation
-0.02635	0.11935514	deprivation
-0.18435	0.14509273	deprivation
0.26404	0.10579475	deprivation
0.10604	0.13415953	deprivation
0.17384	0.09565291	deprivation
0.01584	0.12631599	deprivation
0.023281	0.07960402	deprivation
-0.134719	0.11464314	deprivation
-0.024391	0.0484	deprivation
-0.182391	0.09564941	deprivation
-0.02477	0.1149957	deprivation
-0.18077	0.13691055	deprivation
0.26841	0.10120524	deprivation
0.11241	0.12555075	deprivation
0.2769	0.0911937	deprivation
0.1209	0.11762984	deprivation
0.184001	0.07576305	deprivation
0.028001	0.10611564	deprivation
0.023429	0.046	deprivation
-0.132571	0.08738701	deprivation
-0.04379	0.11344091	deprivation
-0.15579	0.12058876	deprivation
0.15768	0.09880324	deprivation
0.04568	0.10693405	deprivation
0.23468	0.08905869	deprivation
0.12268	0.09800133	deprivation
0.179245	0.07418369	deprivation

For peer review only

0.067245	0.08471145	deprivation
-0.008065	0.0451	deprivation
-0.120065	0.06088366	deprivation
-0.01488	0.10995927	deprivation
-0.10078	0.13254071	deprivation
0.18986	0.09471241	deprivation
0.10396	0.12019334	deprivation
0.24522	0.08495675	deprivation
0.15932	0.1126661	deprivation
0.099416	0.07062308	deprivation
0.013516	0.10229184	deprivation
0.050334	0.0429	deprivation
-0.035566	0.08553602	deprivation
-0.04133	0.1062362	deprivation
0.17767	0.11741435	deprivation
0.20765	0.09110022	deprivation
0.42665	0.10391944	deprivation
0.19958	0.0818338	deprivation
0.41858	0.09589979	deprivation
0.158688	0.06818402	deprivation
0.377688	0.08455211	deprivation
0.078412	0.0415	deprivation
0.297412	0.06497884	deprivation
0.07823	0.10020639	deprivation
0.22123	0.11638711	deprivation
0.20086	0.08565816	deprivation
0.34386	0.10412473	deprivation
0.22708	0.07660294	deprivation
0.37008	0.09681245	deprivation
0.185102	0.06332053	deprivation
0.328102	0.08668408	deprivation
0.018028	0.0382	deprivation
0.161028	0.07045481	deprivation
0.00186	0.09968576	deprivation
-0.00811	0.11593917	deprivation
0.1654	0.08349449	deprivation
0.15543	0.10235219	deprivation
0.16923	0.07379058	deprivation
0.15926	0.0946028	deprivation
0.127822	0.06056005	deprivation
0.117852	0.08468861	deprivation
0.034698	0.0364	deprivation
0.024728	0.06949532	deprivation

For peer review only

0.03379	0.09858448	deprivation
0.26279	0.11504517	deprivation
0.06358	0.07965086	deprivation
0.29258	0.09930131	deprivation
0.12174	0.06914658	deprivation
0.35074	0.09109193	deprivation
0.104863	0.05574415	deprivation
0.333863	0.08138735	deprivation
0.050077	0.0329	deprivation
0.279077	0.06781519	deprivation
0.07774	0.18676124	deprivation
0.58074	0.21180125	deprivation
0.25467	0.15193012	deprivation
0.75767	0.18183171	deprivation
0.23355	0.13803261	deprivation
0.73655	0.17039076	deprivation
0.400112	0.12228393	deprivation
0.903112	0.15790304	deprivation
0.242328	0.0794	deprivation
0.745328	0.12761023	deprivation
0.13706	0.18890304	deprivation
0.64106	0.23125726	deprivation
0.20281	0.14694206	deprivation
0.70681	0.19846292	deprivation
0.32935	0.13252328	deprivation
0.83335	0.18803718	deprivation
0.360394	0.11717517	deprivation
0.864394	0.17755444	deprivation
0.269166	0.0761	deprivation
0.773166	0.15357985	deprivation
0.02371	0.18530097	deprivation
0.47171	0.24527727	deprivation
0.20086	0.14924644	deprivation
0.64886	0.21931482	deprivation
0.32865	0.13541584	deprivation
0.77665	0.21014742	deprivation
0.292453	0.11948757	deprivation
0.740453	0.20025426	deprivation
0.392807	0.0772	deprivation
0.840807	0.1782816	deprivation
0.07148	0.18356105	deprivation
0.51748	0.19825405	deprivation
0.18377	0.14515791	deprivation

For peer review only

0.62977	0.16334268	deprivation
0.19018	0.13124519	deprivation
0.63618	0.15111357	deprivation
0.354925	0.11637238	deprivation
0.800925	0.1383927	deprivation
0.297015	0.0758	deprivation
0.743015	0.10656289	deprivation
0.19278	0.17718163	deprivation
0.72778	0.23234332	deprivation
0.13871	0.14238557	deprivation
0.67371	0.2070356	deprivation
0.35098	0.12911297	deprivation
0.88598	0.198142	deprivation
0.269528	0.11460436	deprivation
0.804528	0.1890086	deprivation
0.398452	0.0746	deprivation
0.933452	0.16779526	deprivation
-0.16212	0.18140259	deprivation
0.09688	0.20685082	deprivation
0.1963	0.14004435	deprivation
0.4553	0.17173462	deprivation
0.26634	0.1273311	deprivation
0.52534	0.16153504	deprivation
0.352256	0.11280514	deprivation
0.611256	0.15035079	deprivation
0.244964	0.073	deprivation
0.503964	0.12332623	deprivation
0.09167	0.16906286	deprivation
0.30667	0.20680198	deprivation
0.23726	0.13360722	deprivation
0.45226	0.1789852	deprivation
0.19947	0.12131719	deprivation
0.41447	0.17000785	deprivation
0.292953	0.10728513	deprivation
0.507953	0.16029632	deprivation
0.262277	0.0693	deprivation
0.477277	0.13779441	deprivation
0.29319	0.16570591	deprivation
0.58719	0.20535856	deprivation
0.27641	0.13247011	deprivation
0.57041	0.17961631	deprivation
0.26739	0.12026076	deprivation
0.56139	0.17081083	deprivation

For peer review only

0.309029	0.10641626	deprivation
0.603029	0.16136329	deprivation
0.270641	0.0689	deprivation
0.564641	0.13950233	deprivation
0.04861	0.17366093	deprivation
0.34161	0.21350354	deprivation
0.15249	0.13395671	deprivation
0.44549	0.18267468	deprivation
0.30791	0.12147131	deprivation
0.60091	0.17372657	deprivation
0.294787	0.10707964	deprivation
0.587787	0.16398686	deprivation
0.223573	0.0691	deprivation
0.516573	0.14212829	deprivation
0.10824	0.17571232	deprivation
0.59424	0.22392021	deprivation
0.11775	0.13239958	deprivation
0.60375	0.19182046	deprivation
0.21195	0.11844446	deprivation
0.69795	0.18246789	deprivation
0.393053	0.10377447	deprivation
0.879053	0.17330488	deprivation
0.298027	0.0667	deprivation
0.784027	0.15399458	deprivation
0.0742	0.13334737	deprivation
0.4592	0.16984864	deprivation
0.28391	0.11837251	deprivation
0.66891	0.15836379	deprivation
0.31469	0.10935689	deprivation
0.69969	0.1517431	deprivation
0.188708	0.09606607	deprivation
0.573708	0.14246308	deprivation
0.092032	0.0615	deprivation
0.477032	0.12185766	deprivation
0.07017	0.1308804	deprivation
0.27417	0.14800166	deprivation
0.28593	0.11470854	deprivation
0.48993	0.13391363	deprivation
0.35049	0.10540119	deprivation
0.55449	0.12603261	deprivation
0.277257	0.09282801	deprivation
0.481257	0.11572316	deprivation
0.270313	0.0598	deprivation

For peer review only

0.474313	0.09138299	deprivation
0.07614	0.12704586	deprivation
0.03854	0.18414543	deprivation
0.29304	0.11257287	deprivation
0.25544	0.17447504	deprivation
0.3234	0.10370598	deprivation
0.2858	0.16888996	deprivation
0.30371	0.09094465	deprivation
0.26611	0.16136858	deprivation
0.10156	0.0583	deprivation
0.06396	0.14549151	deprivation
0.10138	0.12236	deprivation
0.38538	0.16915735	deprivation
0.27111	0.10826505	deprivation
0.55511	0.15925941	deprivation
0.35227	0.09966343	deprivation
0.63627	0.15354166	deprivation
0.218173	0.08741081	deprivation
0.502173	0.14588657	deprivation
0.070537	0.0559	deprivation
0.354537	0.12948764	deprivation
0.15943	0.12088548	deprivation
0.38943	0.12630463	deprivation
0.2209	0.10691459	deprivation
0.4509	0.11300571	deprivation
0.28811	0.09880324	deprivation
0.51811	0.10536432	deprivation
0.226504	0.08703953	deprivation
0.456504	0.09442161	deprivation
0.134706	0.0558	deprivation
0.364706	0.0667323	deprivation
0.11339	0.12159708	deprivation
0.36639	0.1421884	deprivation
0.29687	0.10663194	deprivation
0.54987	0.12962276	deprivation
0.35698	0.09845309	deprivation
0.60998	0.12298252	deprivation
0.323109	0.08687382	deprivation
0.576109	0.11392432	deprivation
0.151221	0.0559	deprivation
0.404221	0.09250135	deprivation
0.15529	0.11607829	deprivation
0.41929	0.13879978	deprivation

For peer review only

0.31119	0.10160709	deprivation
0.57519	0.1269457	deprivation
0.31878	0.09385201	deprivation
0.58278	0.12082802	deprivation
0.382809	0.08289222	deprivation
0.646809	0.11252702	deprivation
0.099671	0.0534	deprivation
0.363671	0.0929665	deprivation
0.22278	0.11295419	deprivation
0.33878	0.12300545	deprivation
0.27883	0.09871256	deprivation
0.39483	0.11007207	deprivation
0.33754	0.09108771	deprivation
0.45354	0.10328921	deprivation
0.329227	0.08009994	deprivation
0.445227	0.09374268	deprivation
0.197893	0.0512	deprivation
0.313893	0.07066208	deprivation
0.15166	0.1142366	deprivation
0.19236	0.14598908	deprivation
0.28231	0.0987915	deprivation
0.32301	0.13424817	deprivation
0.31957	0.09087882	deprivation
0.36027	0.12853704	deprivation
0.31993	0.08007447	deprivation
0.36063	0.1211393	deprivation
0.27352	0.0514	deprivation
0.31422	0.10442591	deprivation
0.11357	0.1161152	deprivation
0.18557	0.15200773	deprivation
0.27465	0.09797479	deprivation
0.34665	0.13864584	deprivation
0.36052	0.08905515	deprivation
0.43252	0.13249313	deprivation
0.370911	0.07757899	deprivation
0.442911	0.12506842	deprivation
0.300299	0.0493	deprivation
0.372299	0.10979117	deprivation
-0.16709	0.2516272	deprivation
0.18591	0.28912629	deprivation
0.0336	0.19224591	deprivation
0.3866	0.23924099	deprivation
0.14633	0.17017782	deprivation

For peer review only

0.49933	0.22189694	deprivation
0.318316	0.14724364	deprivation
0.671316	0.20483762	deprivation
-0.040836	0.0938	deprivation
0.312164	0.17051745	deprivation
0.00567	0.25585938	deprivation
0.06767	0.35272633	deprivation
0.06689	0.18921958	deprivation
0.12889	0.30782445	deprivation
0.18554	0.16494678	deprivation
0.24754	0.29352901	deprivation
0.194471	0.14071958	deprivation
0.256471	0.28063115	deprivation
0.215009	0.089	deprivation
0.277009	0.25859783	deprivation
-0.09045	0.25090654	deprivation
0.37055	0.31221103	deprivation
0.13537	0.18807834	deprivation
0.59637	0.26437681	deprivation
0.21269	0.16568407	deprivation
0.67369	0.24894347	deprivation
0.186059	0.1425074	deprivation
0.647059	0.23415807	deprivation
0.197771	0.0906	deprivation
0.658771	0.20671236	deprivation
-0.16288	0.24903434	deprivation
0.26612	0.33196137	deprivation
0.16065	0.18506891	deprivation
0.58965	0.28710756	deprivation
0.18959	0.1639571	deprivation
0.61859	0.27397478	deprivation
0.277776	0.14233879	deprivation
0.706776	0.26161151	deprivation
0.493504	0.0913	deprivation
0.922504	0.23773081	deprivation
0.0424	0.23818417	deprivation
0.3224	0.29879066	deprivation
0.10603	0.17916032	deprivation
0.38603	0.25424905	deprivation
0.25956	0.15834425	deprivation
0.53956	0.24003554	deprivation
0.175451	0.13766528	deprivation
0.455451	0.22692706	deprivation

For peer review only

0.229619	0.0882	deprivation
0.509619	0.20080687	deprivation
0.34348	0.23935699	deprivation
0.92548	0.30603637	deprivation
0.0557	0.17742088	deprivation
0.6377	0.26047008	deprivation
0.24247	0.15629283	deprivation
0.82447	0.24656427	deprivation
0.277423	0.13550181	deprivation
0.859423	0.23393852	deprivation
0.190157	0.0865	deprivation
0.772157	0.20940091	deprivation
-0.01655	0.23375235	deprivation
0.46245	0.30679284	deprivation
0.10669	0.17353504	deprivation
0.58569	0.26381073	deprivation
0.19728	0.15394369	deprivation
0.67628	0.25135702	deprivation
0.298944	0.1332125	deprivation
0.777944	0.2392222	deprivation
0.159326	0.0846	deprivation
0.638326	0.2159603	deprivation
0.0111	0.23802227	deprivation
0.5291	0.29902441	deprivation
0.18136	0.17635762	deprivation
0.69936	0.25271132	deprivation
0.1664	0.15676817	deprivation
0.6844	0.239452	deprivation
0.273645	0.13679492	deprivation
0.791645	0.22687849	deprivation
0.302835	0.0878	deprivation
0.820835	0.20117117	deprivation
0.21164	0.23362076	deprivation
0.62264	0.27533098	deprivation
0.23906	0.16948112	deprivation
0.65006	0.2235002	deprivation
0.21144	0.14888398	deprivation
0.62244	0.2083145	deprivation
0.250318	0.1285496	deprivation
0.661318	0.19430257	deprivation
0.341642	0.082	deprivation
0.752642	0.16718998	deprivation
0.20966	0.24427282	deprivation

For peer review only

0.07266	0.35088862	deprivation
0.016	0.17543275	deprivation
-0.121	0.30696948	deprivation
0.23443	0.15214763	deprivation
0.09743	0.29428304	deprivation
0.268284	0.13131508	deprivation
0.131284	0.28407263	deprivation
0.524956	0.0838	deprivation
0.387956	0.26547326	deprivation
0.26653	0.13804206	deprivation
0.77453	0.18383028	deprivation
0.03265	0.09815355	deprivation
0.54065	0.1561156	deprivation
-0.03664	0.08189383	deprivation
0.47136	0.14643961	deprivation
0.015488	0.06660481	deprivation
0.523488	0.13847079	deprivation
-0.084348	0.0406	deprivation
0.423652	0.12800906	deprivation
0.04114	0.13306228	deprivation
0.33714	0.17632941	deprivation
0.00632	0.09172813	deprivation
0.30232	0.14765006	deprivation
0.00445	0.07588761	deprivation
0.30045	0.13836698	deprivation
-0.025173	0.06128238	deprivation
0.270827	0.13092754	deprivation
-0.080067	0.0372	deprivation
0.215933	0.12153325	deprivation
0.14597	0.12628262	deprivation
0.28197	0.1394278	deprivation
0.1359	0.09034407	deprivation
0.2719	0.10795768	deprivation
0.00531	0.07588761	deprivation
0.14131	0.09618597	deprivation
-0.017164	0.06134313	deprivation
0.118836	0.08518093	deprivation
-0.051336	0.0373	deprivation
0.084664	0.06988634	deprivation
0.18134	0.12700744	deprivation
0.46934	0.14463585	deprivation
0.12067	0.08984392	deprivation
0.40867	0.11340445	deprivation

For peer review only

0.05896	0.07481243	deprivation
0.34696	0.10190947	deprivation
-0.049703	0.06056278	deprivation
0.238297	0.09195918	deprivation
0.004753	0.0368	deprivation
0.292753	0.07837653	deprivation
0.26153	0.12146119	deprivation
0.67153	0.14264593	deprivation
-0.0388	0.08592567	deprivation
0.3712	0.11392217	deprivation
0.04062	0.07181713	deprivation
0.45062	0.10369542	deprivation
-0.01316	0.05816124	deprivation
0.39684	0.0947511	deprivation
3.00E-04	0.0352	deprivation
0.4103	0.08266849	deprivation
0.057	0.12617278	deprivation
0.241	0.15230121	deprivation
0.00725	0.08455838	deprivation
0.19125	0.12010916	deprivation
0.00151	0.07004977	deprivation
0.18551	0.1103769	deprivation
-0.030004	0.05666127	deprivation
0.153996	0.10240405	deprivation
-0.050006	0.0343	deprivation
0.133994	0.09193791	deprivation
0.12862	0.12272441	deprivation
0.47062	0.13586821	deprivation
-0.05119	0.08184278	deprivation
0.29081	0.10048448	deprivation
-0.04509	0.06792415	deprivation
0.29691	0.08951302	deprivation
-0.005859	0.0547603	deprivation
0.336141	0.07998487	deprivation
-0.047461	0.033	deprivation
0.294539	0.06699172	deprivation
0.14382	0.12277659	deprivation
0.54782	0.14697867	deprivation
-0.0167	0.08102642	deprivation
0.3873	0.11442867	deprivation
-0.0122	0.06679708	deprivation
0.3918	0.10483554	deprivation
0.026182	0.05388107	deprivation

For peer review only

0.430182	0.09711751	deprivation
-0.073292	0.0326	deprivation
0.330708	0.08712864	deprivation
-0.16262	0.12869538	deprivation
0.01538	0.16781475	deprivation
-0.03119	0.0802268	deprivation
0.14681	0.1342968	deprivation
-0.12005	0.06546526	deprivation
0.05795	0.12603567	deprivation
-0.065832	0.05242003	deprivation
0.112168	0.11977959	deprivation
-0.082148	0.0315	deprivation
0.095852	0.11221203	deprivation
0.07477	0.14029412	deprivation
0.31177	0.14907247	deprivation
-0.10096	0.08316466	deprivation
0.13604	0.09724464	deprivation
-0.10201	0.06531501	deprivation
0.13499	0.08249976	deprivation
-0.102115	0.05128206	deprivation
0.134885	0.07190278	deprivation
-0.123925	0.0304	deprivation
0.113075	0.05885847	deprivation
0.11634	0.17878459	deprivation
0.45134	0.20658214	deprivation
0.3588	0.16536898	deprivation
0.6938	0.19508754	deprivation
0.36692	0.15580764	deprivation
0.70192	0.18705152	deprivation
0.234405	0.1391949	deprivation
0.569405	0.1734574	deprivation
0.024945	0.0901	deprivation
0.359945	0.1372234	deprivation
-0.004	0.16515363	deprivation
0.109	0.17777896	deprivation
0.35773	0.14904982	deprivation
0.47073	0.16292787	deprivation
0.38417	0.13906448	deprivation
0.49717	0.15384593	deprivation
0.271516	0.12274905	deprivation
0.384516	0.13927301	deprivation
-0.020436	0.0787	deprivation
0.092564	0.10258328	deprivation

For peer review only

0.0286	0.15920832	deprivation
-0.0057	0.18090357	deprivation
0.30234	0.1448696	deprivation
0.26804	0.16842212	deprivation
0.35101	0.13538482	deprivation
0.31671	0.16033671	deprivation
0.218668	0.11897281	deprivation
0.184368	0.14674243	deprivation
0.058432	0.0758	deprivation
0.024132	0.11456199	deprivation
0.06428	0.15347892	deprivation
0.12388	0.17069233	deprivation
0.27982	0.13915046	deprivation
0.33942	0.15793334	deprivation
0.32731	0.12972112	deprivation
0.38691	0.14969188	deprivation
0.368247	0.11451624	deprivation
0.427847	0.13672622	deprivation
0.140233	0.0734	deprivation
0.199833	0.10472655	deprivation
0.05974	0.14866422	deprivation
-0.03386	0.22969776	deprivation
0.27714	0.13349626	deprivation
0.18354	0.2201846	deprivation
0.32715	0.12413686	deprivation
0.23355	0.21463916	deprivation
0.245696	0.10870603	deprivation
0.152096	0.20609952	deprivation
0.046484	0.069	deprivation
-0.047116	0.1882047	deprivation
0.17266	0.14368389	deprivation
0.49866	0.16245215	deprivation
0.32483	0.12812498	deprivation
0.65083	0.14886789	deprivation
0.39747	0.11870034	deprivation
0.72347	0.14083824	deprivation
0.290796	0.10385191	deprivation
0.616796	0.12857239	deprivation
0.179684	0.0661	deprivation
0.505684	0.10057261	deprivation
0.17323	0.13774012	deprivation
0.39723	0.18085403	deprivation
0.27813	0.12219857	deprivation

For peer review only

0.50213	0.16931725	deprivation
0.30292	0.11282145	deprivation
0.52692	0.16267919	deprivation
0.249883	0.09887083	deprivation
0.473883	0.15333388	deprivation
0.189237	0.063	deprivation
0.413237	0.13305954	deprivation
0.05217	0.13077691	deprivation
0.21417	0.18173893	deprivation
0.28415	0.11421121	deprivation
0.44615	0.17020764	deprivation
0.29143	0.10484279	deprivation
0.45343	0.16406843	deprivation
0.323187	0.0909121	deprivation
0.485187	0.15553601	deprivation
0.169693	0.0574	deprivation
0.331693	0.13864054	deprivation
0.11531	0.12884502	deprivation
0.45931	0.21787962	deprivation
0.33137	0.10991583	deprivation
0.67537	0.2072486	deprivation
0.26723	0.10004744	deprivation
0.61123	0.20218798	deprivation
0.240802	0.08624088	deprivation
0.584802	0.19572425	deprivation
0.131778	0.0543	deprivation
0.475778	0.18389938	deprivation
0.31221	0.12717272	deprivation
0.69921	0.16018633	deprivation
0.35037	0.10740619	deprivation
0.73737	0.14499259	deprivation
0.37446	0.09653233	deprivation
0.76146	0.13713224	deprivation
0.379224	0.08244422	deprivation
0.766224	0.12760803	deprivation
0.210386	0.0516	deprivation
0.597386	0.11022395	deprivation
0.16481	0.11489826	deprivation
0.53681	0.14921344	deprivation
0.1328	0.08527256	deprivation
0.5048	0.1278063	deprivation
-0.02573	0.07145887	deprivation
0.34627	0.11903533	deprivation

For peer review only

0.064532	0.05824577	deprivation
0.436532	0.1116047	deprivation
-0.002582	0.0356	deprivation
0.369418	0.10163858	deprivation
0.15724	0.11138532	deprivation
0.50924	0.16497751	deprivation
0.18325	0.07942254	deprivation
0.53525	0.14532319	deprivation
0.0696	0.06568295	deprivation
0.4216	0.13829367	deprivation
0.02466	0.05304489	deprivation
0.37666	0.13275786	deprivation
0.0265	0.0324	deprivation
0.3785	0.12593907	deprivation
0.0715	0.10846659	deprivation
0.2275	0.15061623	deprivation
0.09562	0.08072726	deprivation
0.25162	0.13204976	deprivation
0.02416	0.06750148	deprivation
0.18016	0.12440539	deprivation
-0.040384	0.05464247	deprivation
0.115616	0.11792392	deprivation
-0.000796	0.0332	deprivation
0.155204	0.10964712	deprivation
0.09006	0.10925772	deprivation
0.22306	0.11878051	deprivation
0.08543	0.08038159	deprivation
0.21843	0.09291265	deprivation
0.09841	0.06727199	deprivation
0.23141	0.08183569	deprivation
0.059151	0.05482527	deprivation
0.192151	0.07195394	deprivation
-0.071941	0.0335	deprivation
0.061059	0.05739172	deprivation
0.08743	0.10653023	deprivation
0.40943	0.13449573	deprivation
0.08515	0.07695037	deprivation
0.40715	0.11252453	deprivation
0.05228	0.06464712	deprivation
0.37428	0.10449718	deprivation
-0.008988	0.0525632	deprivation
0.313012	0.09748487	deprivation
0.039858	0.032	deprivation

For peer review only

0.361858	0.08811589	deprivation
0.27748	0.10411767	deprivation
0.38548	0.12051963	deprivation
0.17274	0.07483756	deprivation
0.28074	0.09635948	deprivation
0.03499	0.06272687	deprivation
0.14299	0.08728774	deprivation
0.010616	0.05088114	deprivation
0.118616	0.07920467	deprivation
0.013564	0.0308	deprivation
0.121564	0.0680671	deprivation
0.26153	0.10059553	deprivation
0.49253	0.1260772	deprivation
0.10128	0.07286604	deprivation
0.33228	0.10528751	deprivation
0.06319	0.06107708	deprivation
0.29419	0.09750082	deprivation
0.04778	0.04992314	deprivation
0.27878	0.0909303	deprivation
0.03564	0.0304	deprivation
0.26664	0.0818545	deprivation
0.22605	0.09990896	deprivation
0.38605	0.10602853	deprivation
0.1011	0.07066378	deprivation
0.2611	0.07907983	deprivation
0.075	0.05903914	deprivation
0.235	0.06889028	deprivation
0.05034	0.04788121	deprivation
0.21034	0.05960587	deprivation
0.01177	0.029	deprivation
0.17177	0.04583939	deprivation
-0.00377	0.10198323	deprivation
0.02463	0.11442985	deprivation
0.19071	0.06955092	deprivation
0.21911	0.08678099	deprivation
0.04469	0.05777093	deprivation
0.07309	0.07766009	deprivation
-0.009761	0.04662028	deprivation
0.018639	0.06976432	deprivation
-0.003819	0.0281	deprivation
0.024581	0.05901881	deprivation
-0.02273	0.10449483	deprivation
0.41527	0.1231145	deprivation

For peer review only

0.00523	0.0692943	deprivation
0.44323	0.09507739	deprivation
-0.07153	0.05624678	deprivation
0.36647	0.08603319	deprivation
-0.034066	0.0446213	deprivation
0.403934	0.07892446	deprivation
-0.085644	0.0265	deprivation
0.352356	0.07028698	deprivation
0.02809	0.26238308	deprivation
-0.02401	0.35714727	deprivation
0.43055	0.21864464	deprivation
0.37845	0.32636601	deprivation
0.45318	0.20233104	deprivation
0.40108	0.31566935	deprivation
0.289603	0.17804879	deprivation
0.237503	0.30068365	deprivation
0.471947	0.1136	deprivation
0.419847	0.26760839	deprivation
0.08036	0.22763554	deprivation
0.38236	0.24847153	deprivation
0.39953	0.18779044	deprivation
0.70153	0.2125686	deprivation
0.47901	0.17123566	deprivation
0.78101	0.19809546	deprivation
0.286284	0.14990574	deprivation
0.588284	0.17997747	deprivation
0.133186	0.0958	deprivation
0.435186	0.13819479	deprivation
-0.05922	0.20922509	deprivation
0.15978	0.24664043	deprivation
0.25046	0.17178152	deprivation
0.46946	0.21578983	deprivation
0.49121	0.15686733	deprivation
0.71021	0.20411693	deprivation
0.367141	0.13752443	deprivation
0.586141	0.18965582	deprivation
0.326989	0.0881	deprivation
0.545989	0.1575372	deprivation
0.15184	0.19806045	deprivation
0.45384	0.24984641	deprivation
0.32878	0.16039907	deprivation
0.63078	0.22118578	deprivation
0.48842	0.14644811	deprivation

For peer review only

0.79042	0.21128734	deprivation
0.378132	0.12814055	deprivation
0.680132	0.1990359	deprivation
0.126878	0.0816	deprivation
0.428878	0.17278267	deprivation
0.04533	0.18533497	deprivation
0.15333	0.23023599	deprivation
0.18723	0.14639378	deprivation
0.29523	0.20022662	deprivation
0.39549	0.13342507	deprivation
0.50349	0.19094976	deprivation
0.297718	0.11696192	deprivation
0.405718	0.17983228	deprivation
0.223222	0.0747	deprivation
0.331222	0.15569088	deprivation
0.13652	0.1772413	deprivation
0.22822	0.25576912	deprivation
0.26147	0.13475181	deprivation
0.35317	0.22838873	deprivation
0.36612	0.12182779	deprivation
0.45782	0.22100989	deprivation
0.341374	0.10691099	deprivation
0.433074	0.21315093	deprivation
0.247546	0.0686	deprivation
0.339246	0.19674684	deprivation
0.17574	0.15839902	deprivation
0.40074	0.18144558	deprivation
0.21502	0.12125486	deprivation
0.44002	0.15011659	deprivation
0.38217	0.10902009	deprivation
0.60717	0.14041948	deprivation
0.340247	0.09529795	deprivation
0.565247	0.13005364	deprivation
0.238343	0.0609	deprivation
0.463343	0.10742933	deprivation
0.05435	0.15193897	deprivation
0.23435	0.15580661	deprivation
0.21284	0.11133921	deprivation
0.39284	0.11656187	deprivation
0.35065	0.09996609	deprivation
0.53065	0.10575193	deprivation
0.287578	0.08726998	deprivation
0.467578	0.09384189	deprivation

For peer review only

0.236642	0.0558	deprivation
0.416642	0.06560404	deprivation
0.03434	0.14553161	deprivation
0.40634	0.16131835	deprivation
0.2394	0.10394984	deprivation
0.6114	0.12509888	deprivation
0.32759	0.09233201	deprivation
0.69959	0.11562595	deprivation
0.275387	0.07993579	deprivation
0.647387	0.10599005	deprivation
0.221263	0.0507	deprivation
0.593263	0.08610836	deprivation
0.00365	0.14181428	deprivation
0.32365	0.16920573	deprivation
0.14901	0.09485384	deprivation
0.46901	0.13235007	deprivation
0.28583	0.08203243	deprivation
0.60583	0.12348526	deprivation
0.334637	0.07002978	deprivation
0.654637	0.11585966	deprivation
0.234223	0.0441	deprivation
0.554223	0.10229418	deprivation
0.23326	0.12504207	deprivation
0.50526	0.24673226	deprivation
0.47813	0.1006857	deprivation
0.75013	0.23532722	deprivation
0.47419	0.08890023	deprivation
0.74619	0.230531	deprivation
0.451977	0.07682213	deprivation
0.723977	0.22614803	deprivation
0.344883	0.0492	deprivation
0.616883	0.21831612	deprivation
0.15926	0.11994482	deprivation
0.43326	0.16713982	deprivation
0.4622	0.09258704	deprivation
0.7362	0.14873238	deprivation
0.47575	0.08030971	deprivation
0.74975	0.14141644	deprivation
0.455276	0.06820689	deprivation
0.729276	0.1349116	deprivation
0.299724	0.0433	deprivation
0.573724	0.12419279	deprivation
0.25766	0.11558136	deprivation

For peer review only

0.35526	0.15114831	deprivation
0.34688	0.09142877	deprivation
0.44448	0.13358885	deprivation
0.49087	0.0798672	deprivation
0.58847	0.12595845	deprivation
0.444557	0.06812966	deprivation
0.542157	0.11886299	deprivation
0.337513	0.0433	deprivation
0.435113	0.10659104	deprivation
0.29661	0.11278945	deprivation
0.66961	0.12733527	deprivation
0.42579	0.08797733	deprivation
0.79879	0.105985	deprivation
0.5124	0.0764055	deprivation
0.8854	0.09659508	deprivation
0.499935	0.06456315	deprivation
0.872935	0.08752834	deprivation
0.288295	0.0406	deprivation
0.661295	0.07170195	deprivation
0.21958	0.10991474	deprivation
0.44658	0.16600184	deprivation
0.41766	0.08548368	deprivation
0.64466	0.15093979	deprivation
0.46006	0.07448631	deprivation
0.68706	0.14499507	deprivation
0.526283	0.06325346	deprivation
0.753283	0.13955773	deprivation
0.353127	0.04	deprivation
0.580127	0.13067272	deprivation
0.26279	0.10812331	deprivation
0.55079	0.13462054	deprivation
0.4854	0.08239053	deprivation
0.7734	0.1149793	deprivation
0.47584	0.07124135	deprivation
0.76384	0.10727241	deprivation
0.506955	0.06014399	deprivation
0.794955	0.1002464	deprivation
0.355925	0.0379	deprivation
0.643925	0.08870428	deprivation
0.31751	0.10399558	deprivation
0.56251	0.16788782	deprivation
0.42944	0.0793532	deprivation
0.67444	0.15384463	deprivation

For peer review only

0.49845	0.06860685	deprivation
0.74345	0.14858715	deprivation
0.577794	0.05783122	deprivation
0.822794	0.14392946	deprivation
0.477466	0.0362	deprivation
0.722466	0.13668094	deprivation
0.37173	0.10028684	deprivation
0.78273	0.10834413	deprivation
0.48465	0.07673917	deprivation
0.89565	0.08700517	deprivation
0.52876	0.06593649	deprivation
0.93976	0.07764419	deprivation
0.542288	0.05526997	deprivation
0.953288	0.06881693	deprivation
0.461432	0.0346	deprivation
0.872432	0.05364849	deprivation
0.28777	0.10289762	deprivation
0.47077	0.13384797	deprivation
0.48374	0.07508209	deprivation
0.66674	0.11386255	deprivation
0.50974	0.06362586	deprivation
0.69274	0.10665651	deprivation
0.563166	0.05261606	deprivation
0.746166	0.10047791	deprivation
0.426194	0.0326	deprivation
0.609194	0.0915976	deprivation
0.44614	0.1068367	deprivation
0.68214	0.13389014	deprivation
0.42394	0.07584095	deprivation
0.65994	0.11074448	deprivation
0.52125	0.06240521	deprivation
0.75725	0.10201422	deprivation
0.571923	0.05036229	deprivation
0.807923	0.09512544	deprivation
0.433867	0.0306	deprivation
0.669867	0.08630672	deprivation
-0.12334	0.13404481	deprivation
-0.12172	0.15035165	deprivation
0.10281	0.1168769	deprivation
0.10443	0.13526944	deprivation
0.05093	0.10458948	deprivation
0.05255	0.12480613	deprivation
0.195006	0.08737254	deprivation

For peer review only

0.196626	0.11077712	deprivation
0.161754	0.0536	deprivation
0.163374	0.08666355	deprivation
-0.28449	0.12947602	deprivation
-0.41649	0.1718518	deprivation
0.02627	0.11031972	deprivation
-0.10573	0.15792226	deprivation
0.0715	0.0975545	deprivation
-0.0605	0.14928456	deprivation
0.195348	0.08026288	deprivation
0.063348	0.13860422	deprivation
0.084222	0.0487	deprivation
-0.047778	0.12304751	deprivation
-0.29514	0.12718058	deprivation
-0.55314	0.14474015	deprivation
-0.04983	0.11091731	deprivation
-0.30783	0.13068076	deprivation
0.08558	0.09935895	deprivation
-0.17242	0.12102483	deprivation
0.173524	0.08238428	deprivation
-0.084476	0.10752665	deprivation
0.045926	0.0501	deprivation
-0.212074	0.08535116	deprivation
-0.23343	0.12983004	deprivation
-0.50943	0.14716769	deprivation
-0.06284	0.11185205	deprivation
-0.33884	0.13158028	deprivation
0.05312	0.10005519	deprivation
-0.22288	0.12171085	deprivation
0.147792	0.08353107	deprivation
-0.128208	0.10853539	deprivation
0.097318	0.0512	deprivation
-0.178682	0.08616223	deprivation
-0.31168	0.12523438	deprivation
-0.47868	0.14546357	deprivation
-0.10752	0.10767474	deprivation
-0.27452	0.13065164	deprivation
0.0168	0.0961699	deprivation
-0.1502	0.12134517	deprivation
0.060536	0.07992703	deprivation
-0.106464	0.10892351	deprivation
0.073974	0.0488	deprivation
-0.093026	0.0886422	deprivation

For peer review only

-0.29688	0.12434649	deprivation
-0.46088	0.16013388	deprivation
-0.07081	0.10388287	deprivation
-0.23481	0.14481871	deprivation
0.03671	0.09213425	deprivation
-0.12729	0.13663649	deprivation
0.015466	0.07626303	deprivation
-0.148534	0.12647869	deprivation
0.042694	0.0463	deprivation
-0.121306	0.11101577	deprivation
-0.26962	0.11741231	deprivation
-0.56762	0.18195233	deprivation
-0.05333	0.09926701	deprivation
-0.35133	0.17080673	deprivation
0.00806	0.08850316	deprivation
-0.28994	0.16478413	deprivation
0.014254	0.07360435	deprivation
-0.283746	0.15728509	deprivation
0.086826	0.0448	deprivation
-0.211174	0.14604123	deprivation
-0.27473	0.11547489	deprivation
-0.44473	0.13467346	deprivation
-0.15153	0.09694045	deprivation
-0.32153	0.1191635	deprivation
-0.00365	0.08617006	deprivation
-0.17365	0.11057925	deprivation
0.053115	0.0716654	deprivation
-0.116885	0.09969163	deprivation
0.013585	0.0437	deprivation
-0.156415	0.0819279	deprivation
-0.3588	0.11725413	deprivation
-0.6388	0.13615128	deprivation
-0.2139	0.09638325	deprivation
-0.4939	0.11865231	deprivation
-0.06928	0.0854645	deprivation
-0.34928	0.10996736	deprivation
0.000867	0.07090346	deprivation
-0.279133	0.09907543	deprivation
0.101903	0.0431	deprivation
-0.178097	0.08152454	deprivation
-0.32226	0.11630843	deprivation
-0.64026	0.13237028	deprivation
-0.18056	0.09464724	deprivation

For peer review only

-0.49856	0.11380835	deprivation
-0.14799	0.08287901	deprivation
-0.46599	0.10422653	deprivation
0.026327	0.06772001	deprivation
-0.291673	0.09262959	deprivation
0.086963	0.0406	deprivation
-0.231037	0.07511724	deprivation
0.28819	0.14663618	deprivation
0.23859	0.17499263	deprivation
0.29246	0.10216271	deprivation
0.24286	0.13984802	deprivation
0.30091	0.08449154	deprivation
0.25131	0.12751106	deprivation
0.350702	0.06830593	deprivation
0.301102	0.11741359	deprivation
0.267258	0.0417	deprivation
0.217658	0.1042072	deprivation
0.33064	0.13614118	deprivation
0.22464	0.17731153	deprivation
0.31824	0.09100247	deprivation
0.21224	0.14555552	deprivation
0.33575	0.07321667	deprivation
0.22975	0.13515044	deprivation
0.376857	0.05814233	deprivation
0.270857	0.12761461	deprivation
0.294223	0.0353	deprivation
0.188223	0.11895819	deprivation
0.24583	0.13339059	deprivation
0.25843	0.19475282	deprivation
0.37706	0.09083458	deprivation
0.38966	0.16848303	deprivation
0.39748	0.07418733	deprivation
0.41008	0.16012298	deprivation
0.465997	0.05914229	deprivation
0.478597	0.15373165	deprivation
0.365913	0.0359	deprivation
0.378513	0.14637083	deprivation
0.39214	0.13289251	deprivation
0.78014	0.17626701	deprivation
0.37082	0.09022333	deprivation
0.75882	0.14679881	deprivation
0.47949	0.07353319	deprivation
0.86749	0.13717423	deprivation

For peer review only

0.420007	0.0587218	deprivation
0.808007	0.12983794	deprivation
0.350973	0.0356	deprivation
0.738973	0.12114867	deprivation
0.20382	0.12496472	deprivation
0.26672	0.16232418	deprivation
0.29443	0.08466434	deprivation
0.35733	0.13379466	deprivation
0.38529	0.06912865	deprivation
0.44819	0.1245461	deprivation
0.496742	0.05504044	deprivation
0.559642	0.1173133	deprivation
0.352678	0.0332	deprivation
0.415578	0.10878971	deprivation
0.30135	0.12695393	deprivation
0.40835	0.13353374	deprivation
0.34704	0.08206985	deprivation
0.45404	0.09192073	deprivation
0.30595	0.0667084	deprivation
0.41295	0.07851095	deprivation
0.403242	0.05306006	deprivation
0.510242	0.0673003	deprivation
0.322328	0.0319	deprivation
0.429328	0.05226442	deprivation
0.3267	0.12121848	deprivation
0.3471	0.15778124	deprivation
0.2779	0.07914954	deprivation
0.2983	0.12831855	deprivation
0.33179	0.06442895	deprivation
0.35219	0.11980021	deprivation
0.436694	0.05146076	deprivation
0.457094	0.11335436	deprivation
0.289526	0.0311	deprivation
0.309926	0.10567975	deprivation
0.3168	0.11736	deprivation
0.4768	0.17925853	deprivation
0.32144	0.07646287	deprivation
0.48144	0.15558541	deprivation
0.33486	0.06202234	deprivation
0.49486	0.1490202	deprivation
0.3594	0.04922002	deprivation
0.5194	0.14416262	deprivation
0.31297	0.0295	deprivation

For peer review only

0.47297	0.13867408	deprivation
0.33447	0.11851793	deprivation
0.51047	0.14643258	deprivation
0.33696	0.07416751	deprivation
0.51296	0.11356417	deprivation
0.2979	0.05961585	deprivation
0.4739	0.10464249	deprivation
0.360789	0.04704083	deprivation
0.536789	0.09802469	deprivation
0.281461	0.028	deprivation
0.457461	0.09044335	deprivation
0.40081	0.12229121	deprivation
0.68281	0.15708947	deprivation
0.25662	0.07352503	deprivation
0.53862	0.12299549	deprivation
0.23166	0.05740949	deprivation
0.51366	0.11409562	deprivation
0.321451	0.04432618	deprivation
0.603451	0.10810537	deprivation
0.252879	0.026	deprivation
0.534879	0.10197039	deprivation
-0.10658	0.21896176	deprivation
-0.42258	0.27386949	deprivation
0.07548	0.20273184	deprivation
-0.24052	0.26107556	deprivation
-0.01256	0.18871195	deprivation
-0.32856	0.25034466	deprivation
-0.0302	0.16386192	deprivation
-0.3462	0.23218738	deprivation
-0.02538	0.1033	deprivation
-0.34138	0.19424505	deprivation
-0.36493	0.19681781	deprivation
-0.58593	0.21112605	deprivation
0.06369	0.17750101	deprivation
-0.15731	0.19324485	deprivation
0.07008	0.16409857	deprivation
-0.15092	0.18101188	deprivation
-0.099865	0.14135293	deprivation
-0.320865	0.16067859	deprivation
-0.217615	0.0884	deprivation
-0.438615	0.11683972	deprivation
-0.30909	0.18478477	deprivation
-0.37419	0.20741034	deprivation

For peer review only

0.03984	0.1687968	deprivation
-0.02526	0.19330287	deprivation
0.05339	0.15576495	deprivation
-0.01171	0.18203395	deprivation
-0.153593	0.13399761	deprivation
-0.218693	0.1637956	deprivation
-0.321427	0.084	deprivation
-0.386527	0.12621268	deprivation
-0.20901	0.17600028	deprivation
-0.44501	0.1944618	deprivation
0.002	0.15925806	deprivation
-0.234	0.17945033	deprivation
0.06139	0.1468312	deprivation
-0.17461	0.16851911	deprivation
-0.020366	0.12697228	deprivation
-0.256366	0.1515297	deprivation
-0.182524	0.08	deprivation
-0.418524	0.11506211	deprivation
-0.16017	0.16912377	deprivation
-0.32917	0.19667997	deprivation
-0.03276	0.15337614	deprivation
-0.20176	0.18331503	deprivation
0.07003	0.14206861	deprivation
-0.09897	0.17396451	deprivation
-0.09983	0.12333422	deprivation
-0.26883	0.15903298	deprivation
-0.19055	0.0778	deprivation
-0.35955	0.12701575	deprivation
-0.26043	0.16315416	deprivation
-0.43543	0.19478255	deprivation
-0.06757	0.1444179	deprivation
-0.24257	0.17938085	deprivation
-0.01151	0.13260426	deprivation
-0.18651	0.17001426	deprivation
-0.045587	0.11460297	deprivation
-0.220587	0.15638031	deprivation
-0.083313	0.0722	deprivation
-0.258313	0.12858383	deprivation
-0.22184	0.1510861	deprivation
-0.32984	0.16680977	deprivation
-0.05684	0.13288687	deprivation
-0.16484	0.15052379	deprivation
0.04976	0.12164752	deprivation

For peer review only

-0.05824	0.14070043	deprivation
-0.062303	0.10429286	deprivation
-0.170303	0.12599798	deprivation
-0.201677	0.0652	deprivation
-0.309677	0.09617448	deprivation
-0.09538	0.14175038	deprivation
0.04762	0.15835886	deprivation
-0.1151	0.12378065	deprivation
0.0279	0.14249916	deprivation
0.03413	0.11239417	deprivation
0.17713	0.13272833	deprivation
-0.03313	0.09559482	deprivation
0.10987	0.11883909	deprivation
-0.20837	0.0594	deprivation
-0.06537	0.0922644	deprivation
-0.37625	0.13941926	deprivation
-0.54125	0.15370042	deprivation
-0.07467	0.11891543	deprivation
-0.23967	0.13537714	deprivation
-0.06752	0.10771096	deprivation
-0.23252	0.12564927	deprivation
-0.12571	0.09150503	deprivation
-0.29071	0.1120681	deprivation
-0.22928	0.0566	deprivation
-0.39428	0.08596307	deprivation
-0.16761	0.13067127	deprivation
-0.19841	0.17904396	deprivation
-0.22863	0.10926431	deprivation
-0.25943	0.16407453	deprivation
-0.09969	0.09754532	deprivation
-0.13049	0.1565147	deprivation
-0.055496	0.0819523	deprivation
-0.086296	0.14730221	deprivation
-0.188504	0.0503	deprivation
-0.219304	0.13233235	deprivation
0.16711	0.24632306	deprivation
0.42411	0.28736571	deprivation
0.23069	0.23930002	deprivation
0.48769	0.28136898	deprivation
0.28451	0.23039065	deprivation
0.54151	0.27383179	deprivation
0.143054	0.20689652	deprivation
0.400054	0.25438194	deprivation

For peer review only

-0.224944	0.1331	deprivation
0.032056	0.19904675	deprivation
0.02873	0.24371368	deprivation
0.00173	0.26190298	deprivation
0.32238	0.23640028	deprivation
0.29538	0.25511154	deprivation
0.29486	0.22790722	deprivation
0.26786	0.24726203	deprivation
0.142019	0.2073764	deprivation
0.115019	0.22847709	deprivation
-0.140299	0.1356	deprivation
-0.167299	0.16608483	deprivation
0.07825	0.222926	deprivation
0.17765	0.24511142	deprivation
0.26675	0.21575414	deprivation
0.36615	0.23860733	deprivation
0.29044	0.20754224	deprivation
0.38984	0.23120854	deprivation
0.019728	0.18741809	deprivation
0.119128	0.21332874	deprivation
0.136512	0.1215	deprivation
0.235912	0.15857446	deprivation
0.08103	0.21798027	deprivation
-0.13797	0.24767955	deprivation
0.28882	0.21066336	deprivation
0.06982	0.24126502	deprivation
0.29131	0.20265925	deprivation
0.07231	0.23430862	deprivation
0.13551	0.18328492	deprivation
-0.08349	0.2177685	deprivation
0.05755	0.119	deprivation
-0.16145	0.16730439	deprivation
0.02549	0.20561724	deprivation
-0.06691	0.21740012	deprivation
0.26299	0.19786672	deprivation
0.17059	0.21008475	deprivation
0.2289	0.18958903	deprivation
0.1365	0.20230759	deprivation
0.112045	0.16997132	deprivation
0.019645	0.18405056	deprivation
0.006325	0.1095	deprivation
-0.086075	0.13028665	deprivation
-0.00678	0.20111611	deprivation

For peer review only

-0.12878	0.21025076	deprivation
0.21877	0.19221457	deprivation
0.09677	0.20175265	deprivation
0.33895	0.18385769	deprivation
0.21695	0.19380748	deprivation
0.075513	0.16486252	deprivation
-0.046487	0.17589014	deprivation
0.185827	0.1062	deprivation
0.063827	0.1226219	deprivation
0.05741	0.18839398	deprivation
0.21441	0.21577611	deprivation
0.15137	0.17955782	deprivation
0.30837	0.20810586	deprivation
0.19178	0.17161964	deprivation
0.34878	0.20129665	deprivation
0.04088	0.1540573	deprivation
0.19788	0.18654943	deprivation
-0.00234	0.0994	deprivation
0.15466	0.14473217	deprivation
-0.0055	0.18281756	deprivation
-0.0972	0.21392866	deprivation
0.12344	0.17369082	deprivation
0.03174	0.20618368	deprivation
0.17133	0.16581972	deprivation
0.07963	0.19959807	deprivation
0.142365	0.14848549	deprivation
0.050665	0.18544851	deprivation
0.137565	0.0955	deprivation
0.045865	0.14650413	deprivation
0.03261	0.17796418	deprivation
0.03452	0.21453039	deprivation
0.15828	0.167968	deprivation
0.16019	0.20631357	deprivation
0.25988	0.15974586	deprivation
0.26179	0.19967669	deprivation
0.186267	0.14351129	deprivation
0.188177	0.18694259	deprivation
0.051193	0.093	deprivation
0.053103	0.15166094	deprivation
0.04348	0.1660228	deprivation
-0.13452	0.20179113	deprivation
0.10887	0.15363463	deprivation
-0.06913	0.19172817	deprivation

For peer review only

0.17962	0.14448599	deprivation
0.00162	0.18447843	deprivation
0.205817	0.12757743	deprivation
0.027817	0.17155783	deprivation
0.145173	0.0812	deprivation
-0.032827	0.14053302	deprivation
-0.36484	0.30233434	deprivation
-0.04084	0.3840933	deprivation
-0.12572	0.2636988	deprivation
0.19828	0.35448367	deprivation
-0.23588	0.24098519	deprivation
0.08812	0.3379282	deprivation
-0.523434	0.20958822	deprivation
-0.199434	0.31630496	deprivation
-0.358476	0.1331	deprivation
-0.034476	0.27173005	deprivation
-0.70373	0.28109788	deprivation
-1.00773	0.36036963	deprivation
-0.19828	0.23705706	deprivation
-0.50228	0.32717931	deprivation
-0.31638	0.21384212	deprivation
-0.62038	0.31077114	deprivation
-0.562626	0.18230208	deprivation
-0.866626	0.28997293	deprivation
-0.571574	0.1142	deprivation
-0.875574	0.25276845	deprivation
-0.46163	0.27239872	deprivation
-0.78963	0.37940726	deprivation
-0.40633	0.23631938	deprivation
-0.73433	0.35439478	deprivation
-0.27809	0.21490866	deprivation
-0.60609	0.34049162	deprivation
-0.176485	0.18594002	deprivation
-0.504485	0.32298994	deprivation
-0.320815	0.118	deprivation
-0.648815	0.28926253	deprivation
-0.51216	0.25497774	deprivation
-0.79816	0.29083614	deprivation
-0.43985	0.21753568	deprivation
-0.72585	0.25863832	deprivation
-0.34437	0.19712169	deprivation
-0.63037	0.24172085	deprivation
-0.499051	0.17105976	deprivation

For peer review only

-0.785051	0.22098292	deprivation
-0.414419	0.1088	deprivation
-0.700419	0.17722711	deprivation
-0.67625	0.25520443	deprivation
-0.25325	0.33969831	deprivation
-0.49728	0.21531674	deprivation
-0.07428	0.31084874	deprivation
-0.36428	0.19510031	deprivation
0.05872	0.29720325	deprivation
-0.478934	0.16789294	deprivation
-0.055934	0.28009584	deprivation
-0.336496	0.106	deprivation
0.086504	0.24799524	deprivation
-0.59255	0.24903213	deprivation
-1.20055	0.30598993	deprivation
-0.56719	0.20587861	deprivation
-1.17519	0.27202728	deprivation
-0.40407	0.18574189	deprivation
-1.01207	0.25712427	deprivation
-0.479749	0.1600688	deprivation
-1.087749	0.23923808	deprivation
-0.521711	0.1011	deprivation
-1.129711	0.20453374	deprivation
-0.44288	0.22759545	deprivation
0.08812	0.4019445	deprivation
-0.45213	0.19085073	deprivation
0.07887	0.38233976	deprivation
-0.39859	0.17263754	deprivation
0.13241	0.37358187	deprivation
-0.459816	0.14858708	deprivation
0.071184	0.36309477	deprivation
-0.424244	0.0936	deprivation
0.106756	0.34426828	deprivation
-0.462	0.22209732	deprivation
-0.096	0.26006405	deprivation
-0.56972	0.18632364	deprivation
-0.20372	0.23026635	deprivation
-0.41916	0.16846023	deprivation
-0.05316	0.21606698	deprivation
-0.405884	0.14521587	deprivation
-0.039884	0.19847856	deprivation
-0.395756	0.0917	deprivation
-0.029756	0.16344718	deprivation

For peer review only

-0.53571	0.2181972	deprivation
-0.40171	0.28893318	deprivation
-0.47106	0.17914134	deprivation
-0.33706	0.26069902	deprivation
-0.49158	0.16083485	deprivation
-0.35758	0.24847577	deprivation
-0.509548	0.13762097	deprivation
-0.375548	0.23411939	deprivation
-0.486552	0.0863	deprivation
-0.352552	0.20813469	deprivation
-0.47161	0.22426103	deprivation
-0.68961	0.28026884	deprivation
-0.46231	0.1810311	deprivation
-0.68031	0.24704224	deprivation
-0.43353	0.16101705	deprivation
-0.65153	0.23277478	deprivation
-0.321208	0.13687074	deprivation
-0.539208	0.21677456	deprivation
-0.421432	0.0856	deprivation
-0.639432	0.18863979	deprivation
0.05126	0.18188879	deprivation
0.24026	0.30755086	deprivation
0.00497	0.13344606	deprivation
0.19397	0.2816236	deprivation
0.06191	0.11414955	deprivation
0.25091	0.27300938	deprivation
0.108431	0.0949255	deprivation
0.297431	0.26554632	deprivation
-0.050501	0.0587	deprivation
0.138499	0.25485229	deprivation
-0.08057	0.18525412	deprivation
-0.02227	0.23887179	deprivation
0.02571	0.13155793	deprivation
0.08401	0.20012029	deprivation
0.05884	0.11105755	deprivation
0.11714	0.18728166	deprivation
-0.018378	0.09206677	deprivation
0.039922	0.17668313	deprivation
-0.191182	0.057	deprivation
-0.132882	0.16121303	deprivation
0.09987	0.17850347	deprivation
0.15137	0.18775178	deprivation
-0.03343	0.13008724	deprivation

For peer review only

0.01807	0.14251291	deprivation
0.06758	0.11035253	deprivation
0.11908	0.12475945	deprivation
-0.043718	0.09117938	deprivation
0.007782	0.10817079	deprivation
-0.132902	0.0562	deprivation
-0.081402	0.08090538	deprivation
-0.03409	0.17756985	deprivation
0.16691	0.24198845	deprivation
-0.01214	0.12616755	deprivation
0.18886	0.20723323	deprivation
-0.07175	0.10625272	deprivation
0.12925	0.19574729	deprivation
0.065428	0.08768016	deprivation
0.266428	0.18632007	deprivation
-0.091398	0.0541	deprivation
0.109602	0.17307273	deprivation
-0.29949	0.18416194	deprivation
-0.12749	0.19422284	deprivation
0.00144	0.12536132	deprivation
0.17344	0.1397224	deprivation
-0.00359	0.10576488	deprivation
0.16841	0.12244632	deprivation
0.053988	0.08748971	deprivation
0.225988	0.10705765	deprivation
-0.104788	0.0543	deprivation
0.067212	0.08219112	deprivation
-0.12677	0.18405412	deprivation
0.30723	0.21076814	deprivation
-0.06414	0.12379972	deprivation
0.36986	0.16085291	deprivation
0.06617	0.10363904	deprivation
0.50017	0.14590524	deprivation
0.010706	0.08509219	deprivation
0.444706	0.13337155	deprivation
-0.068166	0.0522	deprivation
0.365834	0.11520473	deprivation
0.02111	0.17741728	deprivation
0.21911	0.23258773	deprivation
-0.0045	0.1192042	deprivation
0.1935	0.19191092	deprivation
-0.04415	0.09939884	deprivation
0.15385	0.18027837	deprivation

For peer review only

0.012467	0.08126857	deprivation
0.210467	0.17095245	deprivation
-0.039247	0.0497	deprivation
0.158753	0.15839902	deprivation
-0.10966	0.17908088	deprivation
0.15134	0.21540207	deprivation
-0.03016	0.11810589	deprivation
0.23084	0.16815793	deprivation
-0.01862	0.09805407	deprivation
0.24238	0.15473426	deprivation
0.035	0.08000625	deprivation
0.296	0.143976	deprivation
-0.1278	0.0488	deprivation
0.1332	0.12926535	deprivation
-0.24529	0.18285191	deprivation
-0.2452686	0.21590243	deprivation
-0.14386	0.12061385	deprivation
-0.1438386	0.16651348	deprivation
0.04033	0.09887088	deprivation
0.0403514	0.15150739	deprivation
-0.079979	0.07998256	deprivation
-0.0799576	0.13991515	deprivation
-0.188831	0.0485	deprivation
-0.1888096	0.1246246	deprivation
0.04348	0.19452529	deprivation
0.24748	0.21333975	deprivation
-0.06386	0.12326184	deprivation
0.14014	0.15121918	deprivation
0.00435	0.09824403	deprivation
0.20835	0.13162694	deprivation
0.01559	0.07808002	deprivation
0.21959	0.11734671	deprivation
0.01201	0.0468	deprivation
0.21601	0.09931767	deprivation
-0.23299	0.3066665	deprivation
-0.21229	0.39572004	deprivation
-0.00241	0.27836532	deprivation
0.01829	0.37421553	deprivation
0.19947	0.26108428	deprivation
0.22017	0.36154531	deprivation
-0.037397	0.232968	deprivation
-0.016697	0.34179541	deprivation
0.083387	0.1503	deprivation

For peer review only

0.104087	0.29178776	deprivation
-0.15528	0.28476476	deprivation
-0.24358	0.35585184	deprivation
0.02032	0.25383794	deprivation
-0.06798	0.33162217	deprivation
0.22676	0.23622807	deprivation
0.13846	0.31834456	deprivation
0.084195	0.21013144	deprivation
-0.004105	0.29949087	deprivation
0.057155	0.1361	deprivation
-0.031145	0.25310624	deprivation
-0.69375	0.28022164	deprivation
-0.46275	0.3148276	deprivation
-0.13442	0.24674846	deprivation
0.09658	0.28544185	deprivation
0.20841	0.23095558	deprivation
0.43941	0.27190574	deprivation
0.080852	0.20578552	deprivation
0.311852	0.25087832	deprivation
0.242648	0.1328	deprivation
0.473648	0.19552005	deprivation
-0.2722	0.26084325	deprivation
-0.26645	0.26857997	deprivation
-0.05292	0.23105367	deprivation
-0.04717	0.23975362	deprivation
0.16418	0.21541049	deprivation
0.16993	0.22471689	deprivation
0.104484	0.19105873	deprivation
0.110234	0.20149303	deprivation
0.073166	0.123	deprivation
0.078916	0.13865425	deprivation
-0.33817	0.24794453	deprivation
0.19583	0.32524874	deprivation
-0.14442	0.21791783	deprivation
0.38958	0.30298256	deprivation
0.22923	0.20198658	deprivation
0.76323	0.29173418	deprivation
0.137246	0.17754937	deprivation
0.671246	0.27537979	deprivation
0.121924	0.1133	deprivation
0.655924	0.23905468	deprivation
-0.20389	0.23815014	deprivation
-0.02789	0.30735644	deprivation

For peer review only

-0.08484	0.20724973	deprivation
0.09116	0.28408615	deprivation
0.13669	0.1917218	deprivation
0.31269	0.27296472	deprivation
0.083557	0.16802658	deprivation
0.259557	0.25687627	deprivation
0.097993	0.1067	deprivation
0.273993	0.22166953	deprivation
-0.31519	0.22882399	deprivation
-0.11919	0.27202726	deprivation
-0.0358	0.19817934	deprivation
0.1602	0.24680652	deprivation
0.02749	0.18365601	deprivation
0.22349	0.23530393	deprivation
0.126935	0.16181301	deprivation
0.322935	0.2186821	deprivation
0.096205	0.1036	deprivation
0.292205	0.17992046	deprivation
-0.32584	0.21637098	deprivation
-0.21184	0.24120522	deprivation
-0.09319	0.18624479	deprivation
0.02081	0.21459422	deprivation
0.06106	0.17211368	deprivation
0.17506	0.20245167	deprivation
0.015446	0.15186458	deprivation
0.129446	0.18554355	deprivation
0.097994	0.0973	deprivation
0.211994	0.14432897	deprivation
-0.43597	0.21436233	deprivation
-0.00797	0.27228959	deprivation
-0.03788	0.17954078	deprivation
0.39012	0.24581558	deprivation
0.02042	0.16478501	deprivation
0.44842	0.23525414	deprivation
0.080302	0.14399226	deprivation
0.508302	0.22118811	deprivation
0.166938	0.0916	deprivation
0.594938	0.19126152	deprivation
-0.32981	0.21627099	deprivation
-0.17881	0.27984799	deprivation
-0.25185	0.17741776	deprivation
-0.10085	0.2510355	deprivation
0.04824	0.16077565	deprivation

For peer review only

0.19924	0.23956329	deprivation
0.067258	0.13962897	deprivation
0.218258	0.22591594	deprivation
0.100902	0.0885	deprivation
0.251902	0.19842885	deprivation
0.22136	0.21879728	deprivation
0.63636	0.31219792	deprivation
0.27978	0.13480449	deprivation
0.69478	0.26032199	deprivation
0.14092	0.10078537	deprivation
0.55592	0.24444423	deprivation
0.269865	0.07539828	deprivation
0.684865	0.2351174	deprivation
0.262125	0.0439	deprivation
0.677125	0.22698568	deprivation
-0.02032	0.214052	deprivation
0.03038	0.31149631	deprivation
-0.0056	0.13205734	deprivation
0.0451	0.26201303	deprivation
0.12943	0.09550131	deprivation
0.18013	0.24562612	deprivation
0.267793	0.06966814	deprivation
0.318493	0.23678121	deprivation
0.270367	0.0402	deprivation
0.321067	0.22984284	deprivation
0.30356	0.19621654	deprivation
0.40456	0.27431701	deprivation
0.22408	0.13000096	deprivation
0.32508	0.23162284	deprivation
0.20194	0.09724053	deprivation
0.30294	0.21495258	deprivation
0.257849	0.0718646	deprivation
0.358849	0.20472765	deprivation
0.267661	0.0416	deprivation
0.368661	0.1961618	deprivation
0.4988	0.20071086	deprivation
1.1158	0.36770975	deprivation
0.31534	0.13090015	deprivation
0.93234	0.33475433	deprivation
0.2198	0.09842002	deprivation
0.8368	0.32343795	deprivation
0.245986	0.07273631	deprivation
0.862986	0.31656939	deprivation

For peer review only

0.272134	0.0424	deprivation
0.889134	0.31100381	deprivation
0.28958	0.1995432	deprivation
0.50258	0.27566924	deprivation
0.25119	0.12626266	deprivation
0.46419	0.22829433	deprivation
0.18566	0.09446322	deprivation
0.39866	0.21236605	deprivation
0.270045	0.07039957	deprivation
0.483045	0.2028106	deprivation
0.270275	0.0409	deprivation
0.483275	0.19454781	deprivation
0.23578	0.20044044	deprivation
0.31518	0.2699352	deprivation
0.11726	0.12564872	deprivation
0.19666	0.22017321	deprivation
0.3108	0.0933231	deprivation
0.3902	0.20346459	deprivation
0.369068	0.06902036	deprivation
0.448468	0.19352636	deprivation
0.170332	0.0395	deprivation
0.249732	0.18506456	deprivation
0.24008	0.19857948	deprivation
0.72908	0.24880478	deprivation
0.17455	0.12102054	deprivation
0.66355	0.19265508	deprivation
0.18275	0.09204809	deprivation
0.67175	0.17590583	deprivation
0.289567	0.06903948	deprivation
0.778567	0.16503472	deprivation
0.267583	0.0401	deprivation
0.756583	0.15517094	deprivation
0.39248	0.19075146	deprivation
0.67648	0.2308942	deprivation
0.21198	0.1212238	deprivation
0.49598	0.17782356	deprivation
0.23331	0.09264562	deprivation
0.51731	0.15971606	deprivation
0.3376	0.06857084	deprivation
0.6216	0.14706451	deprivation
0.24189	0.039	deprivation
0.52589	0.13581977	deprivation
0.19349	0.19764716	deprivation

For peer review only

0.25049	0.22983405	deprivation
0.10582	0.12406341	deprivation
0.16282	0.1707367	deprivation
0.1327	0.09232037	deprivation
0.1897	0.1492727	deprivation
0.220998	0.06774253	deprivation
0.277998	0.13545605	deprivation
0.240402	0.0387	deprivation
0.297402	0.12351915	deprivation
0.05683	0.21109062	deprivation
0.19483	0.26596708	deprivation
0.04219	0.12720008	deprivation
0.18019	0.20581327	deprivation
0.1034	0.09296026	deprivation
0.2414	0.18660346	deprivation
0.158194	0.06712019	deprivation
0.296194	0.17516952	deprivation
0.229036	0.0376	deprivation
0.367036	0.16611141	deprivation
0.0095	0.24347544	deprivation
-0.3385	0.27433055	deprivation
0.1975	0.20386844	deprivation
-0.1505	0.23987351	deprivation
-0.03937	0.17763474	deprivation
-0.38737	0.21801619	deprivation
-0.103799	0.14458306	deprivation
-0.451799	0.19204484	deprivation
-0.253941	0.0875	deprivation
-0.601941	0.15373097	deprivation
0.03124	0.23067228	deprivation
-0.04406	0.33767034	deprivation
0.11048	0.18691265	deprivation
0.03518	0.30943158	deprivation
0.05088	0.16171459	deprivation
-0.02442	0.29489519	deprivation
-0.084923	0.13164878	deprivation
-0.160223	0.27954062	deprivation
-0.158377	0.0802	deprivation
-0.233677	0.25931371	deprivation
-0.00093	0.22037706	deprivation
-0.16493	0.29996383	deprivation
0.18896	0.18328243	deprivation
0.02496	0.27386986	deprivation

For peer review only

0.04501	0.15913105	deprivation
-0.11899	0.25833107	deprivation
0.032663	0.13035724	deprivation
-0.131337	0.2416718	deprivation
-0.167653	0.0799	deprivation
-0.331653	0.21862356	deprivation
0.35104	0.2123183	deprivation
0.42744	0.26840399	deprivation
0.17381	0.17716097	deprivation
0.25021	0.24155258	deprivation
0.05481	0.15456342	deprivation
0.13121	0.22550275	deprivation
0.066199	0.12620479	deprivation
0.142599	0.2070973	deprivation
-0.091469	0.0766	deprivation
-0.015069	0.1811883	deprivation
0.12508	0.20860837	deprivation
0.17178	0.26430804	deprivation
0.18062	0.17020488	deprivation
0.22732	0.23518289	deprivation
0.08632	0.14787065	deprivation
0.13302	0.21956097	deprivation
0.106037	0.12198131	deprivation
0.152737	0.20302889	deprivation
-0.051087	0.075	deprivation
-0.004387	0.17879119	deprivation
-0.02606	0.20440157	deprivation
0.22694	0.24207869	deprivation
0.05888	0.16161683	deprivation
0.31188	0.20722473	deprivation
0.02345	0.13918348	deprivation
0.27645	0.19024755	deprivation
-0.023928	0.11350441	deprivation
0.229072	0.17235237	deprivation
-0.159752	0.0689	deprivation
0.093248	0.1468649	deprivation
0.06862	0.19420929	deprivation
0.22862	0.21580561	deprivation
0.14625	0.15187376	deprivation
0.30625	0.17866295	deprivation
0.031	0.13005172	deprivation
0.191	0.16052495	deprivation
-0.128034	0.10604702	deprivation

For peer review only

0.031966	0.14177722	deprivation
-0.080326	0.0646	deprivation
0.079674	0.11414013	deprivation
0.15799	0.18804162	deprivation
0.36999	0.23212682	deprivation
0.16115	0.14799017	deprivation
0.37315	0.20105795	deprivation
-0.06221	0.12637567	deprivation
0.14979	0.18572566	deprivation
0.020329	0.10254253	deprivation
0.232329	0.17040593	deprivation
-0.017259	0.0621	deprivation
0.194741	0.14959819	deprivation
0.22643	0.18256117	deprivation
0.48643	0.2380681	deprivation
0.1359	0.14024044	deprivation
0.3959	0.20740111	deprivation
0.04865	0.11796143	deprivation
0.30865	0.19303559	deprivation
-0.088222	0.09444178	deprivation
0.171778	0.17963043	deprivation
-0.085138	0.0562	deprivation
0.174862	0.16280749	deprivation
-0.22614	0.18660214	deprivation
0.40486	0.24626175	deprivation
-0.06523	0.13290974	deprivation
0.56577	0.20854134	deprivation
-0.02173	0.10826306	deprivation
0.60927	0.1937663	deprivation
-0.018717	0.08411718	deprivation
0.612283	0.1813841	deprivation
-0.054593	0.0491	deprivation
0.576407	0.16803363	deprivation
0.29208	0.26360571	deprivation
0.71808	0.33240671	deprivation
0.36252	0.22357292	deprivation
0.78852	0.30164731	deprivation
0.44519	0.20579587	deprivation
0.87119	0.28871818	deprivation
0.376434	0.18206936	deprivation
0.802434	0.27231508	deprivation
0.788776	0.117	deprivation
1.214776	0.23387016	deprivation

For peer review only

-0.06331	0.26116148	deprivation
-0.17331	0.28910434	deprivation
0.49402	0.21313013	deprivation
0.38402	0.24657747	deprivation
0.47266	0.19526016	deprivation
0.36266	0.23130614	deprivation
0.525666	0.17339458	deprivation
0.415666	0.21317054	deprivation
0.557344	0.1122	deprivation
0.447344	0.16722691	deprivation
0.22488	0.23625971	deprivation
0.31288	0.26673037	deprivation
0.43142	0.20057363	deprivation
0.51942	0.23570367	deprivation
0.53508	0.1840244	deprivation
0.62308	0.22179139	deprivation
0.394118	0.16329513	deprivation
0.482118	0.20491886	deprivation
0.614432	0.1059	deprivation
0.702432	0.16291486	deprivation
0.10749	0.22845282	deprivation
0.36449	0.27484274	deprivation
0.34887	0.18767581	deprivation
0.60587	0.2420125	deprivation
0.55614	0.1710689	deprivation
0.81314	0.22937395	deprivation
0.4481	0.15206841	deprivation
0.7051	0.21557514	deprivation
0.44037	0.0988	deprivation
0.69737	0.18195956	deprivation
0.31652	0.21804678	deprivation
0.57752	0.28472796	deprivation
0.45785	0.17860809	deprivation
0.71885	0.25578597	deprivation
0.52672	0.16306471	deprivation
0.78772	0.24518505	deprivation
0.330566	0.14433884	deprivation
0.591566	0.23315083	deprivation
0.418824	0.0931	deprivation
0.679824	0.20540988	deprivation
0.02548	0.22153386	deprivation
0.06608	0.29152616	deprivation
0.44258	0.17303208	deprivation

For peer review only

0.48318	0.25661323	deprivation
0.45558	0.1574807	deprivation
0.49618	0.24639485	deprivation
0.521651	0.13968253	deprivation
0.562251	0.23541763	deprivation
0.564239	0.0905	deprivation
0.604839	0.21000119	deprivation
0.26157	0.20658831	deprivation
0.25802	0.24678681	deprivation
0.45291	0.16280396	deprivation
0.44936	0.21149499	deprivation
0.43699	0.14702615	deprivation
0.43344	0.19960383	deprivation
0.545891	0.12964062	deprivation
0.542341	0.18716755	deprivation
0.475479	0.0837	deprivation
0.471929	0.15884171	deprivation
0.10717	0.19725496	deprivation
0.05917	0.22202056	deprivation
0.3756	0.15125994	deprivation
0.3276	0.18238196	deprivation
0.50837	0.13454375	deprivation
0.46037	0.16877687	deprivation
0.509014	0.11669349	deprivation
0.461014	0.1549225	deprivation
0.331806	0.0744	deprivation
0.283806	0.1261704	deprivation
0.15103	0.20188326	deprivation
0.20943	0.2111598	deprivation
0.32817	0.14827677	deprivation
0.38657	0.16067859	deprivation
0.57528	0.13046456	deprivation
0.63368	0.14440433	deprivation
0.509837	0.11252022	deprivation
0.568237	0.12842278	deprivation
0.285393	0.0716	deprivation
0.343793	0.09464761	deprivation
0.09688	0.20770183	deprivation
0.16238	0.26402472	deprivation
0.28767	0.145889	deprivation
0.35317	0.21875237	deprivation
0.50534	0.12548327	deprivation
0.57084	0.20570622	deprivation

For peer review only

0.567386	0.10696808	deprivation
0.632886	0.19496454	deprivation
0.484944	0.0676	deprivation
0.550444	0.17646178	deprivation
0.04693	0.21849275	deprivation
-0.12307	0.27319422	deprivation
0.36177	0.17033391	deprivation
0.19177	0.23645219	deprivation
0.40669	0.14404249	deprivation
0.23669	0.21827561	deprivation
0.382692	0.11400439	deprivation
0.212692	0.19973232	deprivation
0.375278	0.0676	deprivation
0.205278	0.17738591	deprivation
0.38164	0.20828099	deprivation
0.66964	0.24717722	deprivation
0.49965	0.15766499	deprivation
0.78765	0.20633434	deprivation
0.38646	0.13	deprivation
0.67446	0.18605271	deprivation
0.413404	0.10152202	deprivation
0.701404	0.16739872	deprivation
0.453756	0.0604	deprivation
0.741756	0.1461635	deprivation
0.1886	0.19998185	deprivation
0.0646	0.31239837	deprivation
0.32598	0.15611848	deprivation
0.20198	0.28630924	deprivation
0.37006	0.13024304	deprivation
0.24606	0.27306272	deprivation
0.39043	0.10226852	deprivation
0.26643	0.26088091	deprivation
0.30606	0.0603	deprivation
0.18206	0.24745927	deprivation
0.06475	0.20103047	deprivation
-0.31025	0.285412	deprivation
0.36375	0.15445663	deprivation
-0.01125	0.25476187	deprivation
0.54114	0.12924399	deprivation
0.16614	0.2403139	deprivation
0.333	0.10208154	deprivation
-0.042	0.22686428	deprivation
0.43961	0.0608	deprivation

For peer review only

0.06461	0.21152636	deprivation
0.03916	0.19601801	deprivation
0.39216	0.23421849	deprivation
0.35487	0.15012881	deprivation
0.70787	0.19741808	deprivation
0.46689	0.12587462	deprivation
0.81989	0.17966541	deprivation
0.41676	0.09966193	deprivation
0.76976	0.16238146	deprivation
0.36396	0.0593	deprivation
0.71696	0.14125059	deprivation
0.19252	0.19174238	deprivation
0.34152	0.22296991	deprivation
0.45853	0.14500848	deprivation
0.60753	0.18433095	deprivation
0.37158	0.12175163	deprivation
0.52058	0.16665503	deprivation
0.377346	0.096145	deprivation
0.526346	0.14897752	deprivation
0.354884	0.0569	deprivation
0.503884	0.12723227	deprivation
0.11498	0.18233456	deprivation
0.20838	0.19992471	deprivation
0.21385	0.13870735	deprivation
0.30725	0.16113265	deprivation
0.34847	0.11673123	deprivation
0.44187	0.14265406	deprivation
0.382362	0.09324017	deprivation
0.475762	0.12416815	deprivation
0.432868	0.0558	deprivation
0.526268	0.09918488	deprivation
0.27406	0.17714294	deprivation
0.30056	0.22169463	deprivation
0.20486	0.13346929	deprivation
0.23136	0.18863441	deprivation
0.31974	0.11224994	deprivation
0.34624	0.17426686	deprivation
0.459544	0.08944188	deprivation
0.486044	0.16052645	deprivation
0.267476	0.0532	deprivation
0.293976	0.14352397	deprivation
-0.06529	0.18233072	deprivation
0.17971	0.21429711	deprivation

For peer review only

0.21158	0.13139197	deprivation
0.45658	0.17303933	deprivation
0.31543	0.10971495	deprivation
0.56043	0.15721364	deprivation
0.38381	0.08639595	deprivation
0.62881	0.14192611	deprivation
0.31434	0.0505	deprivation
0.55934	0.12340588	deprivation
0.2971	0.17783813	deprivation
0.5371	0.2057133	deprivation
0.14486	0.12813216	deprivation
0.38486	0.16464936	deprivation
0.27869	0.10494175	deprivation
0.51869	0.1473239	deprivation
0.245172	0.08203639	deprivation
0.485172	0.13199064	deprivation
0.234218	0.0479	deprivation
0.474218	0.113956	deprivation
-0.36309	0.21626266	deprivation
-0.51509	0.28329938	deprivation
-0.19284	0.19466137	deprivation
-0.34484	0.26717419	deprivation
0.018	0.17629158	deprivation
-0.134	0.25410179	deprivation
-0.056748	0.1444864	deprivation
-0.208748	0.23316372	deprivation
0.181508	0.0856	deprivation
0.029508	0.20203059	deprivation
-0.16553	0.20614194	deprivation
0.08247	0.26292345	deprivation
-0.15019	0.18378566	deprivation
0.09781	0.24578733	deprivation
-0.01759	0.16430329	deprivation
0.23041	0.23158111	deprivation
0.113794	0.13237541	deprivation
0.361794	0.21013684	deprivation
0.087186	0.0778	deprivation
0.335186	0.18079569	deprivation
-0.1755	0.19658497	deprivation
-0.2214	0.22881884	deprivation
-0.08027	0.17481013	deprivation
-0.12617	0.21040673	deprivation
0.08953	0.15555028	deprivation

For peer review only

0.04363	0.19470054	deprivation
0.238871	0.12421067	deprivation
0.192971	0.17070647	deprivation
0.178589	0.0723	deprivation
0.132689	0.13762158	deprivation
-0.08736	0.1890908	deprivation
-0.42336	0.24361505	deprivation
0.01151	0.16535658	deprivation
-0.32449	0.22568952	deprivation
0.09397	0.14595359	deprivation
-0.24203	0.21188537	deprivation
0.316901	0.1159687	deprivation
-0.019099	0.1924622	deprivation
0.190619	0.0675	deprivation
-0.145381	0.16777726	deprivation
-0.29433	0.1804936	deprivation
-0.50733	0.26076269	deprivation
-0.12783	0.15582493	deprivation
-0.34083	0.24433717	deprivation
-0.05251	0.13675807	deprivation
-0.26551	0.23264138	deprivation
0.266171	0.10859954	deprivation
0.053171	0.21728576	deprivation
0.169469	0.0635	deprivation
-0.043531	0.19862399	deprivation
-0.05954	0.17698328	deprivation
0.16246	0.26245289	deprivation
-0.10736	0.14993082	deprivation
0.11464	0.2450259	deprivation
0.06683	0.1303292	deprivation
0.28883	0.23354687	deprivation
0.31758	0.10310058	deprivation
0.53958	0.21951804	deprivation
0.26111	0.0602	deprivation
0.48311	0.20293467	deprivation
-0.12361	0.1667675	deprivation
-0.12938	0.24253373	deprivation
-0.10853	0.14074548	deprivation
-0.1143	0.22543403	deprivation
0.00336	0.12163507	deprivation
-0.00241	0.21402406	deprivation
0.333403	0.09591689	deprivation
0.327633	0.20052745	deprivation

For peer review only

0.242587	0.0561	deprivation
0.236817	0.18481997	deprivation
-0.17078	0.15715766	deprivation
-0.16038	0.17625711	deprivation
-0.14808	0.13106289	deprivation
-0.13768	0.15344549	deprivation
-0.01129	0.11258388	deprivation
-0.00089	0.13799699	deprivation
0.341535	0.08804601	deprivation
0.351935	0.1188282	deprivation
0.301275	0.0511	deprivation
0.311675	0.09475891	deprivation
-0.18885	0.15400263	deprivation
-0.28185	0.19292291	deprivation
-0.13306	0.12531377	deprivation
-0.22606	0.17089757	deprivation
0.048	0.10644031	deprivation
-0.045	0.15758166	deprivation
0.272973	0.08265313	deprivation
0.179973	0.14259727	deprivation
0.364287	0.0477	deprivation
0.271287	0.12560943	deprivation
-0.26592	0.15064727	deprivation
-0.08492	0.20771038	deprivation
-0.14767	0.11609104	deprivation
0.03333	0.18419047	deprivation
0.09772	0.09562704	deprivation
0.27872	0.1720277	deprivation
0.286395	0.07317513	deprivation
0.467395	0.16063499	deprivation
0.452375	0.0416	deprivation
0.633375	0.14892804	deprivation
0.57093	0.2751891	deprivation
1.13593	0.36501107	deprivation
0.14759	0.16896023	deprivation
0.71259	0.29334553	deprivation
0.10716	0.12900702	deprivation
0.67216	0.27229919	deprivation
0.200385	0.09924016	deprivation
0.765385	0.25952389	deprivation
0.212105	0.0594	deprivation
0.777105	0.24704736	deprivation
0.12662	0.29801612	deprivation

For peer review only

1.86662	0.3846555	deprivation
0.24929	0.17150073	deprivation
1.98929	0.29758821	deprivation
0.16444	0.12910654	deprivation
1.90444	0.27534477	deprivation
0.190072	0.09814	deprivation
1.930072	0.26225503	deprivation
0.153448	0.0589	deprivation
1.893448	0.25023079	deprivation
0.12831	0.29948564	deprivation
1.82831	0.41328282	deprivation
0.28344	0.17336632	deprivation
1.98344	0.33341704	deprivation
0.05314	0.13344456	deprivation
1.75314	0.3145131	deprivation
0.259794	0.10282033	deprivation
1.959794	0.30279211	deprivation
0.143936	0.0619	deprivation
1.843936	0.29144922	deprivation
0.54233	0.28825976	deprivation
1.99233	0.31258679	deprivation
0.13248	0.17126407	deprivation
1.58248	0.20963824	deprivation
0.13397	0.13220322	deprivation
1.58397	0.17914938	deprivation
0.222236	0.10256003	deprivation
1.672236	0.15854138	deprivation
0.149594	0.0616	deprivation
1.599594	0.1356885	deprivation
0.27318	0.27280808	deprivation
1.44318	0.32165262	deprivation
0.14826	0.17436275	deprivation
1.31826	0.24380019	deprivation
0.15257	0.13366376	deprivation
1.32257	0.21656907	deprivation
0.164898	0.10278001	deprivation
1.334898	0.19899721	deprivation
0.180402	0.0617	deprivation
1.350402	0.18122652	deprivation
0.46622	0.28841264	deprivation
2.05622	0.42020615	deprivation
0.20515	0.17324194	deprivation
1.79515	0.35128924	deprivation

For peer review only

0.17093	0.13328136	deprivation
1.76093	0.33339958	deprivation
0.096701	0.10298063	deprivation
1.686701	0.32248468	deprivation
0.308739	0.0615	deprivation
1.898739	0.31172682	deprivation
0.34472	0.27342246	deprivation
1.29972	0.34641179	deprivation
0.33799	0.17218409	deprivation
1.29299	0.27365791	deprivation
0.19384	0.13549675	deprivation
1.14884	0.25219171	deprivation
0.128898	0.10644083	deprivation
1.083898	0.23784646	deprivation
0.211702	0.0642	deprivation
1.166702	0.2221777	deprivation
0.15661	0.27661038	deprivation
1.03261	0.33416994	deprivation
0.15711	0.17432412	deprivation
1.03311	0.25601787	deprivation
0.03363	0.13757605	deprivation
0.90963	0.23255842	deprivation
0.221135	0.10882265	deprivation
1.097135	0.21679165	deprivation
0.297235	0.0659	deprivation
1.173235	0.1987437	deprivation
0.19309	0.28527226	deprivation
1.22309	0.35205106	deprivation
0.38261	0.17695655	deprivation
1.41261	0.27179645	deprivation
0.14365	0.13863567	deprivation
1.17365	0.2485549	deprivation
0.201289	0.10928495	deprivation
1.231289	0.23345854	deprivation
0.280731	0.0664	deprivation
1.310731	0.21672252	deprivation
0.01846	0.31373597	deprivation
0.82446	0.35489868	deprivation
0.31314	0.18860448	deprivation
1.11914	0.25118611	deprivation
0.12398	0.14583597	deprivation
0.92998	0.22088671	deprivation
0.159037	0.11312051	deprivation

For peer review only

0.965037	0.20079607	deprivation
0.264673	0.0676	deprivation
1.070673	0.17914399	deprivation
0.0374	0.3178443	deprivation
0.5894	0.41485005	deprivation
0.2197	0.2365967	deprivation
0.7717	0.35644573	deprivation
0.02896	0.21303756	deprivation
0.58096	0.34126318	deprivation
-0.11864	0.194	deprivation
0.43336	0.32971436	deprivation
-0.18632	0.13	deprivation
0.36568	0.29660674	deprivation
0.2296	0.31950743	deprivation
1.1526	0.37214196	deprivation
0.3571	0.23527431	deprivation
1.2801	0.30291689	deprivation
-0.01347	0.2102118	deprivation
0.90953	0.28389019	deprivation
-0.18307	0.18917981	deprivation
0.73993	0.26868874	deprivation
-0.31546	0.125	deprivation
0.60754	0.22810007	deprivation
-0.1596	0.34337298	deprivation
0.0744	0.39389593	deprivation
0.3335	0.25918719	deprivation
0.5675	0.32315167	deprivation
0.02698	0.23564592	deprivation
0.26098	0.30459481	deprivation
-0.19047	0.21592823	deprivation
0.04353	0.28961008	deprivation
-0.09351	0.145	deprivation
0.14049	0.24140008	deprivation
0.2886	0.3340015	deprivation
0.9126	0.38520463	deprivation
0.3574	0.25636302	deprivation
0.9814	0.32023056	deprivation
0.13604	0.2321422	deprivation
0.76004	0.30119032	deprivation
-0.04672	0.21102133	deprivation
0.57728	0.28522905	deprivation
0.18568	0.141	deprivation
0.80968	0.2381315	deprivation

For peer review only

0.1024	0.34368154	deprivation
0.9784	0.40920094	deprivation
0.2593	0.24930303	deprivation
1.1353	0.33388682	deprivation
0.17694	0.22575429	deprivation
1.05294	0.31669135	deprivation
-0.13092	0.20537283	deprivation
0.74508	0.30250026	deprivation
-0.18902	0.137	deprivation
0.68698	0.26095481	deprivation
0.7519	0.33712016	deprivation
1.6119	0.38551135	deprivation
0.3402	0.26201145	deprivation
1.2002	0.32189905	deprivation
0.16037	0.23779403	deprivation
1.02037	0.30251446	deprivation
-0.01612	0.21600232	deprivation
0.84388	0.28570264	deprivation
-0.01925	0.144	deprivation
0.84075	0.23601907	deprivation
0.2181	0.33944955	deprivation
0.5981	0.41804545	deprivation
0.2901	0.26766023	deprivation
0.6701	0.36218504	deprivation
0.21835	0.24412497	deprivation
0.59835	0.34515649	deprivation
-0.01326	0.22173182	deprivation
0.36674	0.32969835	deprivation
0.01891	0.147	deprivation
0.39891	0.28485962	deprivation
0.3018	0.33922854	deprivation
1.0738	0.36375224	deprivation
0.4242	0.26974247	deprivation
1.1962	0.30000115	deprivation
0.19544	0.24695141	deprivation
0.96744	0.27968677	deprivation
-0.06707	0.22455512	deprivation
0.70493	0.26012437	deprivation
-0.00437	0.149	deprivation
0.76763	0.1985968	deprivation
-0.0299	0.3802486	deprivation
1.2601	0.4732271	deprivation
0.3773	0.28319605	deprivation

For peer review only

1.6673	0.39944323	deprivation
0.15734	0.25967095	deprivation
1.44734	0.38312386	deprivation
-0.02303	0.23792856	deprivation
1.26697	0.36873417	deprivation
-0.00231	0.159	deprivation
1.28769	0.32347471	deprivation
0.2386	0.40036234	deprivation
0.8566	0.43063205	deprivation
0.5046	0.28410033	deprivation
1.1226	0.32537203	deprivation
0.02038	0.25548581	deprivation
0.63838	0.30071076	deprivation
0.07874	0.23095021	deprivation
0.69674	0.28016417	deprivation
-0.32212	0.153	deprivation
0.29588	0.22037005	deprivation
NA	NA	deprivation
NA	NA	deprivation
NA	NA	deprivation
NA	NA	deprivation
NA	NA	deprivation
NA	NA	deprivation
NA	NA	deprivation
NA	NA	deprivation
0.000132	0.5407	deprivation
NA	NA	deprivation
NA	NA	deprivation
NA	NA	deprivation
NA	NA	deprivation
NA	NA	deprivation
NA	NA	deprivation
NA	NA	deprivation
NA	NA	deprivation
NA	NA	deprivation
0.876307	0.5174	deprivation
NA	NA	deprivation
-0.15082	0.82803847	deprivation
-0.51182	0.89312473	deprivation
0.13002	0.79053934	deprivation
NA	NA	deprivation
0.50059	0.75704566	deprivation
NA	NA	deprivation

For peer review only

0.402828	0.66316105	deprivation
0.041828	0.74283691	deprivation
0.056312	0.4137	deprivation
-0.304688	0.53213887	deprivation
-0.20471	0.90194069	deprivation
NA	NA	deprivation
NA	NA	deprivation
NA	NA	deprivation
NA	NA	deprivation
NA	NA	deprivation
0.555061	0.75026716	deprivation
NA	NA	deprivation
-0.002811	0.4975	deprivation
-1.272811	0.57285448	deprivation
0.5084	0.82826641	deprivation
1.3844	0.90031309	deprivation
0.46193	0.78434317	deprivation
NA	NA	deprivation
0.77429	0.74035479	deprivation
1.65029	0.82016073	deprivation
0.90804	0.65919807	deprivation
1.78404	0.74771687	deprivation
-0.50319	0.4297	deprivation
0.37281	0.55604002	deprivation
0.049	0.8211843	deprivation
NA	NA	deprivation
0.16399	0.77256028	deprivation
NA	NA	deprivation
0.74241	0.73700265	deprivation
NA	NA	deprivation
-0.188646	0.64948818	deprivation
NA	NA	deprivation
0.026796	0.4083	deprivation
NA	NA	deprivation
0.31403	0.75141723	deprivation
NA	NA	deprivation
0.48051	0.70990537	deprivation
NA	NA	deprivation
0.53712	0.67141838	deprivation
NA	NA	deprivation
0.249681	0.59265764	deprivation
NA	NA	deprivation
0.117089	0.3788	deprivation

For peer review only

NA	NA	deprivation
-0.2937	0.69639473	deprivation
0.2583	0.8133472	deprivation
0.36212	0.64155874	deprivation
0.91412	0.76691959	deprivation
0.09948	0.60036057	deprivation
0.65148	0.73280342	deprivation
0.743215	0.5172632	deprivation
1.295215	0.66643024	deprivation
-0.030285	0.3229	deprivation
0.521715	0.52993627	deprivation
-0.02172	0.65270983	deprivation
0.28228	0.70897698	deprivation
0.11319	0.59371054	deprivation
0.41719	0.65506522	deprivation
0.46207	0.55326027	deprivation
0.76607	0.61863977	deprivation
0.370378	0.47497303	deprivation
0.674378	0.54974323	deprivation
0.285492	0.2957	deprivation
0.589492	0.40503917	deprivation
-0.03039	0.61902019	deprivation
-0.45939	0.63054421	deprivation
0.37902	0.5581889	deprivation
-0.04998	0.57094207	deprivation
0.5566	0.51094466	deprivation
0.1276	0.52484707	deprivation
0.361747	0.42437427	deprivation
-0.067253	0.4410142	deprivation
0.520203	0.2574	deprivation
0.091203	0.28399782	deprivation

For peer review only

Effect	lnHR	SE
Female	-0.35	0.003
1915	0.95	0.015
1925	1.02	0.013
1935	0.90	0.013
1945	0.62	0.012
1955	0.30	0.010
SIMD 2	0.07	0.007
SIMD 3	0.16	0.006
SIMD 4	0.22	0.006
SIMD 5	0.29	0.006
SIMD 6	0.35	0.006
SIMD 7	0.41	0.006
SIMD 8	0.49	0.006
SIMD 9	0.59	0.006
SIMD 10	0.73	0.006

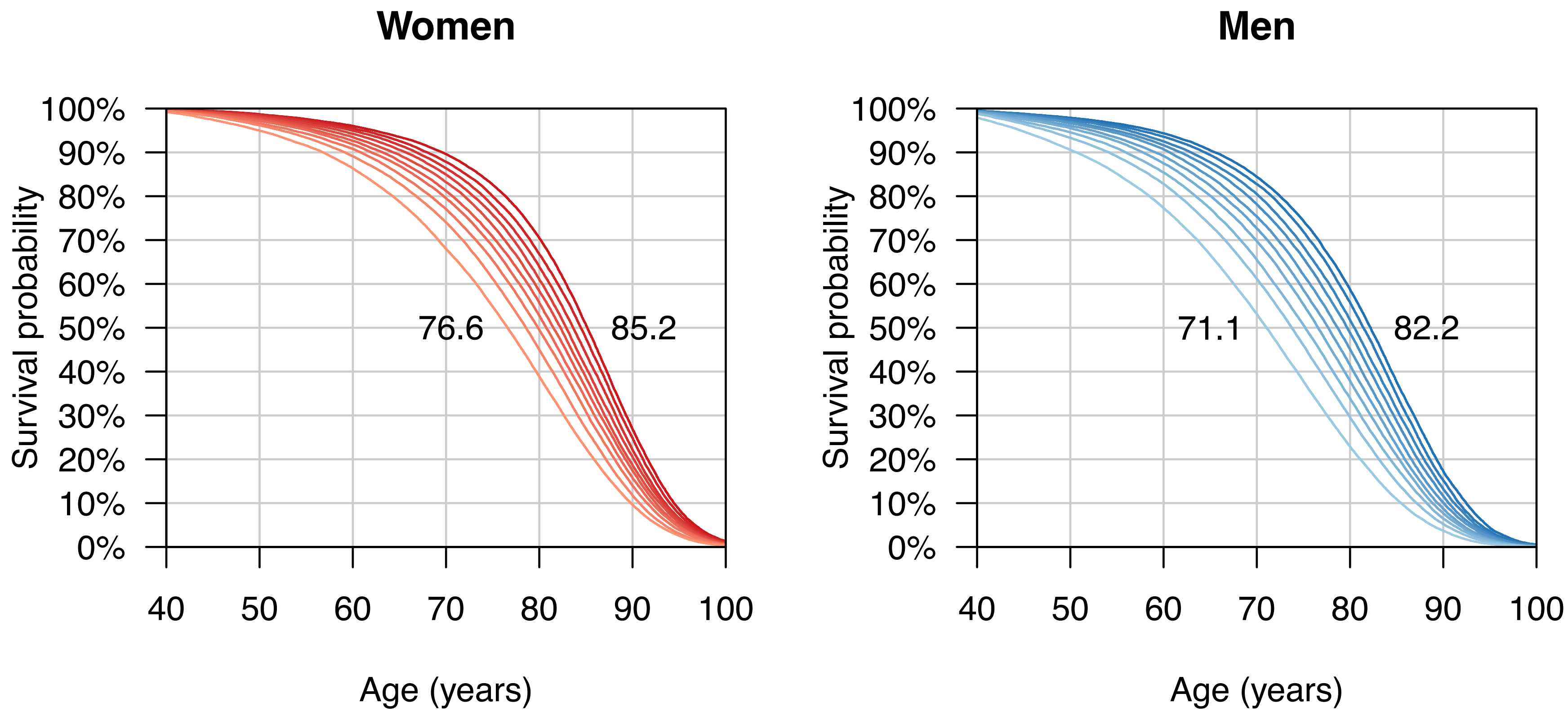

For peer review only - http://bmjopen.bmj.com/site/about/guidelines.xhtml

SIMD	Sex	Records	N Max	Events	Mean	SE	Median	95% CI
1	Female	100,383	40,153	24,811	83.27	0.06	85.17	(85.00 - 85.25)
2	Female	102,599	42,224	25,838	82.45	0.07	84.42	(84.33 - 84.59)
3	Female	102,821	39,962	29,961	81.67	0.07	83.66	(83.50 - 83.75)
4	Female	103,757	39,061	31,518	80.93	0.07	82.91	(82.75 - 83.00)
5	Female	105,156	38,529	33,927	80.21	0.07	82.25	(82.16 - 82.42)
6	Female	104,629	38,337	34,087	79.45	0.07	81.50	(81.34 - 81.66)
7	Female	109,393	38,245	38,311	78.73	0.08	80.75	(80.59 - 80.92)
8	Female	110,980	38,602	40,759	77.79	0.08	79.84	(79.75 - 80.00)
9	Female	108,027	37,650	41,626	76.59	0.08	78.58	(78.42 - 78.75)
10	Female	101,597	36,901	41,221	74.76	0.08	76.59	(76.42 - 76.75)
1	Male	86,838	37,598	21,157	80.27	0.11	82.17	(82.00 - 82.33)
2	Male	89,592	39,461	22,285	79.46	0.09	81.42	(81.25 - 81.59)
3	Male	89,210	37,738	25,213	78.51	0.08	80.50	(80.33 - 80.59)
4	Male	91,885	38,420	27,322	77.67	0.10	79.67	(79.50 - 79.83)
5	Male	92,520	37,718	29,886	76.72	0.08	78.67	(78.50 - 78.83)
6	Male	91,619	36,850	31,135	75.74	0.08	77.67	(77.50 - 77.83)
7	Male	94,206	36,950	34,148	74.77	0.09	76.58	(76.42 - 76.75)
8	Male	96,038	37,801	36,772	73.50	0.09	75.17	(75.00 - 75.33)
9	Male	94,456	37,026	38,175	72.09	0.09	73.75	(73.58 - 73.92)
10	Male	91,424	37,172	39,600	69.57	0.12	71.08	(70.84 - 71.25)

For peer review only

ICD10	Unstratified	Male	Female	SIMD 1	SIMD 2	SIMD 3	SIMD 4
J09-J18	110,985	54,896	56,089	7,252	7,776	8,751	9,459
R00-R09	225,504	109,642	115,862	15,272	18,020	18,360	18,633
C30-C39	54,178	29,657	24,521	2,895	3,368	3,643	4,210
R10-R19	174,055	68,258	105,797	12,079	14,415	14,372	14,847
R50-R69	157,357	71,014	86,343	11,363	12,493	13,392	14,408
C15-C26	71,981	40,064	31,917	6,269	6,442	6,434	6,747
I30-I52	142,898	73,086	69,812	11,666	12,084	12,791	13,737
C76-C80	39,339	18,077	21,262	3,490	3,437	3,549	3,828
Z40-Z54	157,841	77,906	79,935	12,101	13,893	14,324	14,538
I60-I69	100,907	47,573	53,334	7,498	8,086	8,810	9,378
K55-K63	206,178	91,114	115,064	16,228	18,523	18,172	18,214
J40-J47	78,467	33,777	44,690	3,337	4,159	5,065	5,654
I20-I25	175,605	104,230	71,375	12,769	14,306	15,055	15,919
N30-N39	126,329	51,830	74,499	10,422	11,152	11,750	11,300
K20-K31	172,206	80,887	91,319	11,156	14,273	14,354	14,637
J20-J22	77,520	35,833	41,687	4,647	5,420	6,174	6,656
S70-S79	78,231	22,042	56,189	6,346	6,608	7,494	7,665
A30-A49	33,613	15,982	17,631	2,244	2,533	2,688	3,109
T80-T88	75,217	37,899	37,318	6,647	6,827	7,006	7,368
N17-N19	37,213	19,032	18,181	2,462	2,760	3,053	3,327
I80-I89	84,073	39,544	44,529	7,315	8,149	7,999	7,857
K90-K93	47,091	23,934	23,157	3,048	3,542	3,754	4,054
I70-I79	47,410	27,229	20,181	3,235	3,392	3,922	4,307
K50-K52	59,183	22,753	36,430	4,277	4,976	5,123	5,226
S00-S09	64,925	36,484	28,441	4,311	4,563	4,997	5,435
C50-C50	39,358	339	39,019	4,213	4,168	3,981	4,077
C60-C63	22,312	22,311	<10	2,663	2,499	2,329	2,326
G30-G32	4,655	2,024	2,631	412	431	473	430
TOTAL	2,664,631	1,257,417	<1,407,223	195,617	218,295	227,815	237,346

For peer review only

SIMD 5	SIMD 6	SIMD 7	SIMD 8	SIMD 9	SIMD 10
10,358	11,004	12,978	13,930	14,406	15,071
20,418	22,175	25,017	27,572	28,979	31,058
4,779	5,418	6,287	7,040	7,831	8,707
16,364	18,102	19,207	20,624	21,429	22,616
14,842	15,727	17,003	18,829	19,170	20,130
7,147	7,280	7,966	8,129	7,844	7,723
14,379	14,312	15,845	16,391	16,281	15,412
4,028	4,029	4,251	4,216	4,389	4,122
15,932	16,360	17,423	17,777	18,091	17,402
9,805	10,290	10,952	11,903	12,069	12,116
19,789	20,934	22,228	23,380	24,145	24,565
6,762	7,675	9,291	10,862	11,983	13,679
17,142	18,025	19,688	20,874	21,235	20,592
12,142	12,490	13,844	14,379	14,377	14,473
16,561	17,478	18,855	20,361	21,734	22,797
7,380	7,662	8,743	9,513	10,049	11,276
7,939	7,703	8,451	8,705	8,716	8,604
3,195	3,363	3,929	4,164	4,298	4,090
7,477	7,579	8,212	8,293	8,020	7,788
3,578	3,802	4,254	4,650	4,738	4,589
8,384	8,603	8,884	8,948	8,943	8,991
4,311	4,639	5,270	5,536	6,098	6,839
4,698	4,926	5,415	5,809	5,821	5,885
5,609	5,907	6,494	6,936	7,135	7,500
5,927	6,164	6,991	7,960	8,607	9,970
3,958	3,966	3,939	3,869	3,787	3,400
2,347	2,177	2,120	2,113	1,996	1,742
414	423	483	488	537	564
255,665	268,213	294,020	313,251	322,708	331,701

For peer review only

ICD10	Jnstratifiec	Male	Female	SIMD 1	SIMD 2	SIMD 3	SIMD 4	SIMD 5
J09-J18	1.66	1.68	1.66	1.74	1.64	1.65	1.60	1.65
R00-R09	0.73	0.76	0.71	0.71	0.70	0.73	0.74	0.75
C30-C39	3.05	2.92	3.23	3.12	3.19	3.17	3.10	3.19
R10-R19	0.94	1.00	0.89	1.02	0.97	0.96	0.94	0.94
R50-R69	0.98	1.07	0.91	0.98	0.95	0.92	0.95	0.97
C15-C26	2.10	2.09	2.10	2.15	2.18	2.11	2.10	2.08
I30-I52	0.99	0.98	1.01	0.95	1.00	0.91	0.96	1.00
C76-C80	3.26	3.18	3.35	3.41	3.38	3.34	3.30	3.19
Z40-Z54	0.76	0.77	0.76	0.84	0.80	0.80	0.77	0.76
I60-I69	1.12	1.03	1.19	1.15	1.16	1.06	1.15	1.14
K55-K63	0.54	0.56	0.53	0.61	0.58	0.54	0.56	0.56
J40-J47	1.38	1.36	1.41	1.41	1.38	1.37	1.40	1.41
I20-I25	0.61	0.53	0.70	0.61	0.63	0.62	0.63	0.62
N30-N39	0.84	0.83	0.84	0.88	0.88	0.84	0.87	0.86
K20-K31	0.60	0.68	0.54	0.63	0.64	0.55	0.59	0.57
J20-J22	1.27	1.35	1.21	1.29	1.28	1.21	1.25	1.24
S70-S79	0.97	1.16	0.89	0.92	0.96	0.92	0.94	0.95
A30-A49	1.89	1.83	1.96	2.05	1.94	1.93	1.84	1.91
T80-T88	0.84	0.88	0.80	0.84	0.88	0.83	0.85	0.86
N17-N19	1.64	1.63	1.66	1.77	1.70	1.63	1.63	1.61
I80-I89	0.66	0.65	0.67	0.77	0.70	0.61	0.67	0.63
K90-K93	1.09	1.16	1.02	1.11	1.08	1.02	1.08	1.07
I70-I79	1.08	1.01	1.18	1.13	1.10	1.03	1.05	1.05
K50-K52	0.82	0.87	0.78	0.73	0.83	0.83	0.84	0.80
S00-S09	0.74	0.84	0.63	0.78	0.68	0.68	0.73	0.70
C50-C50	1.17	1.53	1.17	1.31	1.30	1.21	1.20	1.15
C60-C63	1.17	1.18	NA	1.29	1.18	1.21	1.29	1.23
G30-G32	1.19	1.39	1.06	1.33	1.21	1.16	1.23	1.19

For peer review only

SIMD 6	SIMD 7	SIMD 8	SIMD 9	SIMD 10
1.67	1.69	1.71	1.68	1.66
0.75	0.76	0.72	0.71	0.70
3.06	3.06	2.96	2.96	2.92
0.94	0.92	0.94	0.92	0.90
1.01	1.01	1.00	1.00	1.00
2.13	2.07	2.09	2.08	2.01
1.02	1.05	1.03	1.00	0.98
3.29	3.21	3.15	3.26	3.19
0.77	0.75	0.74	0.70	0.73
1.14	1.12	1.09	1.11	1.05
0.57	0.53	0.54	0.51	0.48
1.43	1.39	1.40	1.37	1.33
0.62	0.62	0.60	0.56	0.53
0.88	0.85	0.81	0.81	0.76
0.60	0.60	0.61	0.60	0.62
1.29	1.30	1.29	1.27	1.31
0.95	1.01	1.00	0.98	0.99
1.96	1.94	1.83	1.82	1.82
0.85	0.83	0.81	0.83	0.82
1.65	1.72	1.64	1.56	1.58
0.62	0.62	0.67	0.65	0.70
1.11	1.13	1.11	1.07	1.09
1.08	1.08	1.10	1.06	1.06
0.84	0.88	0.82	0.79	0.81
0.71	0.76	0.73	0.76	0.76
1.16	1.09	1.12	1.12	1.07
1.16	1.16	1.16	1.14	0.98
1.26	1.26	1.18	1.11	1.07

For peer review only

ICD10	Jnstratifiec	Male	Female	SIMD 1	SIMD 2	SIMD 3	SIMD 4	SIMD 5
J09-J18	0.0340	0.0354	0.0329	0.0308	0.0287	0.0319	0.0312	0.0327
R00-R09	0.0304	0.0319	0.0290	0.0266	0.0283	0.0295	0.0286	0.0293
C30-C39	0.0304	0.0331	0.0281	0.0220	0.0241	0.0255	0.0270	0.0293
R10-R19	0.0300	0.0262	0.0334	0.0300	0.0314	0.0305	0.0288	0.0294
R50-R69	0.0284	0.0291	0.0278	0.0272	0.0266	0.0272	0.0282	0.0275
C15-C26	0.0277	0.0320	0.0238	0.0328	0.0314	0.0300	0.0293	0.0286
I30-I52	0.0261	0.0274	0.0249	0.0270	0.0271	0.0259	0.0272	0.0275
C76-C80	0.0236	0.0220	0.0252	0.0290	0.0260	0.0262	0.0261	0.0246
Z40-Z54	0.0222	0.0230	0.0216	0.0248	0.0250	0.0254	0.0230	0.0233
I60-I69	0.0207	0.0187	0.0225	0.0210	0.0210	0.0206	0.0223	0.0215
K55-K63	0.0206	0.0197	0.0215	0.0243	0.0240	0.0216	0.0212	0.0214
J40-J47	0.0200	0.0176	0.0223	0.0115	0.0129	0.0154	0.0164	0.0184
I20-I25	0.0196	0.0211	0.0176	0.0190	0.0203	0.0206	0.0208	0.0206
N30-N39	0.0195	0.0165	0.0223	0.0224	0.0219	0.0219	0.0203	0.0200
K20-K31	0.0192	0.0209	0.0173	0.0172	0.0206	0.0174	0.0178	0.0182
J20-J22	0.0181	0.0186	0.0179	0.0147	0.0156	0.0166	0.0172	0.0176
S70-S79	0.0140	0.0098	0.0176	0.0143	0.0142	0.0153	0.0149	0.0145
A30-A49	0.0117	0.0112	0.0122	0.0112	0.0110	0.0115	0.0118	0.0117
T80-T88	0.0117	0.0128	0.0106	0.0136	0.0134	0.0129	0.0129	0.0124
N17-N19	0.0112	0.0119	0.0107	0.0106	0.0105	0.0110	0.0112	0.0111
I80-I89	0.0102	0.0099	0.0106	0.0138	0.0129	0.0108	0.0109	0.0102
K90-K93	0.0095	0.0106	0.0084	0.0083	0.0086	0.0085	0.0090	0.0088
I70-I79	0.0094	0.0105	0.0084	0.0089	0.0083	0.0090	0.0094	0.0095
K50-K52	0.0089	0.0076	0.0101	0.0076	0.0093	0.0094	0.0090	0.0086
S00-S09	0.0088	0.0117	0.0063	0.0082	0.0069	0.0075	0.0082	0.0080
C50-C50	0.0084	NA	0.0161	0.0135	0.0122	0.0106	0.0101	0.0087
C60-C63	0.0048	0.0101	NA	0.0084	0.0066	0.0062	0.0062	0.0055
G30-G32	0.0010	0.0011	0.0010	0.0013	0.0012	0.0012	0.0011	0.0009
TOTAL	1.0000	1.0000	1.0000	1.0000	1.0000	1.0000	1.0000	1.0000

For peer review only

SIMD 6	SIMD 7	SIMD 8	SIMD 9	SIMD 10
0.0331	0.0360	0.0371	0.0370	0.0376
0.0301	0.0314	0.0309	0.0317	0.0330
0.0299	0.0316	0.0325	0.0356	0.0383
0.0308	0.0291	0.0302	0.0301	0.0306
0.0286	0.0282	0.0294	0.0293	0.0302
0.0280	0.0271	0.0265	0.0250	0.0234
0.0263	0.0272	0.0263	0.0249	0.0228
0.0239	0.0224	0.0207	0.0219	0.0198
0.0228	0.0215	0.0205	0.0194	0.0192
0.0211	0.0202	0.0203	0.0205	0.0192
0.0215	0.0194	0.0196	0.0189	0.0176
0.0198	0.0212	0.0236	0.0252	0.0274
0.0203	0.0202	0.0197	0.0183	0.0165
0.0199	0.0193	0.0181	0.0178	0.0166
0.0188	0.0186	0.0193	0.0200	0.0213
0.0178	0.0186	0.0192	0.0196	0.0223
0.0133	0.0140	0.0136	0.0131	0.0128
0.0119	0.0125	0.0119	0.0120	0.0112
0.0116	0.0111	0.0105	0.0102	0.0097
0.0113	0.0120	0.0119	0.0113	0.0109
0.0097	0.0090	0.0093	0.0089	0.0095
0.0093	0.0097	0.0096	0.0100	0.0113
0.0096	0.0096	0.0099	0.0095	0.0094
0.0090	0.0093	0.0089	0.0087	0.0092
0.0079	0.0087	0.0091	0.0100	0.0113
0.0083	0.0070	0.0067	0.0065	0.0055
0.0046	0.0040	0.0038	0.0035	0.0026
0.0010	0.0010	0.0009	0.0009	0.0009
1.0000	1.0000	1.0000	1.0000	1.0000

For peer review only

ICD10	Unstratified	Male	Female	SIMD 1	SIMD 2	SIMD 3
J09-J18	-0.19 (0.01)	-0.13 (0.02)	-0.24 (0.02)	-0.23 (0.06)	-0.25 (0.05)	-0.16 (0.05)
R00-R09	0.16 (0.01)	0.20 (0.01)	0.12 (0.01)	0.11 (0.03)	0.12 (0.02)	0.11 (0.02)
C30-C39	0.32 (0.02)	0.44 (0.03)	0.19 (0.03)	0.24 (0.09)	0.34 (0.07)	0.39 (0.07)
R10-R19	0.11 (0.01)	0.13 (0.01)	0.09 (0.01)	0.03 (0.03)	0.07 (0.03)	0.11 (0.03)
R50-R69	0.08 (0.01)	0.13 (0.01)	0.03 (0.01)	0.03 (0.04)	0.03 (0.03)	0.04 (0.03)
C15-C26	0.27 (0.02)	0.29 (0.02)	0.23 (0.02)	0.26 (0.05)	0.28 (0.05)	0.31 (0.05)
I30-I52	0.23 (0.01)	0.23 (0.01)	0.21 (0.02)	0.16 (0.04)	0.27 (0.04)	0.19 (0.04)
C76-C80	0.22 (0.02)	0.27 (0.03)	0.19 (0.03)	0.06 (0.06)	0.18 (0.06)	0.17 (0.06)
Z40-Z54	-0.04 (0.01)	-0.03 (0.01)	-0.05 (0.01)	-0.03 (0.03)	-0.05 (0.03)	-0.01 (0.03)
I60-I69	0.21 (0.01)	0.22 (0.02)	0.19 (0.02)	0.17 (0.06)	0.14 (0.05)	0.16 (0.05)
K55-K63	0.02 (0.01)	0.01 (0.01)	0.03 (0.01)	0.03 (0.03)	0.05 (0.02)	0.01 (0.02)
J40-J47	0.26 (0.01)	0.34 (0.02)	0.21 (0.02)	0.39 (0.08)	0.24 (0.07)	0.32 (0.06)
I20-I25	0.43 (0.01)	0.40 (0.01)	0.47 (0.02)	0.39 (0.03)	0.36 (0.03)	0.38 (0.03)
N30-N39	0.03 (0.01)	0.09 (0.02)	-0.02 (0.01)	0.12 (0.04)	0.07 (0.03)	0.04 (0.04)
K20-K31	0.33 (0.01)	0.38 (0.01)	0.28 (0.01)	0.29 (0.03)	0.32 (0.03)	0.39 (0.03)
J20-J22	-0.13 (0.02)	-0.08 (0.02)	-0.17 (0.02)	-0.02 (0.07)	-0.14 (0.06)	-0.20 (0.06)
S70-S79	0.12 (0.02)	0.09 (0.03)	0.13 (0.03)	0.02 (0.09)	0.06 (0.09)	0.15 (0.08)
A30-A49	-0.43 (0.02)	-0.40 (0.03)	-0.46 (0.03)	-0.35 (0.09)	-0.50 (0.08)	-0.31 (0.08)
T80-T88	-0.05 (0.01)	-0.03 (0.02)	-0.08 (0.02)	0.01 (0.04)	-0.10 (0.04)	-0.07 (0.04)
N17-N19	0.04 (0.02)	0.09 (0.03)	-0.01 (0.03)	0.03 (0.10)	0.06 (0.09)	0.07 (0.09)
I80-I89	0.24 (0.01)	0.21 (0.01)	0.27 (0.01)	0.25 (0.03)	0.23 (0.03)	0.26 (0.03)
K90-K93	-0.04 (0.02)	0.02 (0.02)	-0.11 (0.02)	-0.14 (0.06)	-0.08 (0.06)	-0.06 (0.06)
I70-I79	0.44 (0.02)	0.39 (0.02)	0.51 (0.03)	0.56 (0.08)	0.48 (0.08)	0.50 (0.07)
K50-K52	0.34 (0.01)	0.36 (0.02)	0.33 (0.02)	0.36 (0.05)	0.44 (0.05)	0.33 (0.04)
S00-S09	0.19 (0.01)	0.26 (0.02)	0.05 (0.02)	0.04 (0.06)	0.04 (0.06)	0.13 (0.05)
C50-C50	0.20 (0.01)	NA	0.20 (0.02)	0.20 (0.05)	0.17 (0.04)	0.17 (0.05)
C60-C63	0.02 (0.03)	0.02 (0.03)	NA	-0.07 (0.09)	-0.12 (0.08)	-0.04 (0.10)
G30-G32	0.27 (0.08)	0.30 (0.10)	0.23 (0.13)	NA	NA	0.16 (0.28)
TOTAL	0.13 (0.00)	0.16 (0.00)	0.10 (0.00)	0.10 (0.01)	0.11 (0.01)	0.13 (0.01)

For peer review only

SIMD 4	SIMD 5	SIMD 6	SIMD 7	SIMD 8	SIMD 9
-0.16 (0.05)	-0.18 (0.05)	-0.16 (0.04)	-0.17 (0.04)	-0.20 (0.04)	-0.19 (0.04)
0.11 (0.02)	0.10 (0.02)	0.13 (0.02)	0.20 (0.02)	0.18 (0.02)	0.21 (0.02)
0.48 (0.07)	0.36 (0.06)	0.39 (0.06)	0.31 (0.06)	0.32 (0.05)	0.27 (0.05)
0.11 (0.03)	0.11 (0.02)	0.14 (0.02)	0.11 (0.02)	0.12 (0.02)	0.11 (0.02)
0.11 (0.03)	0.07 (0.03)	0.09 (0.03)	0.11 (0.03)	0.10 (0.03)	0.08 (0.03)
0.26 (0.05)	0.32 (0.05)	0.23 (0.05)	0.24 (0.05)	0.28 (0.05)	0.23 (0.05)
0.17 (0.04)	0.19 (0.04)	0.23 (0.04)	0.22 (0.04)	0.25 (0.03)	0.28 (0.03)
0.33 (0.06)	0.20 (0.06)	0.21 (0.06)	0.17 (0.06)	0.24 (0.06)	0.29 (0.06)
0.02 (0.03)	0.01 (0.03)	-0.03 (0.03)	-0.03 (0.02)	-0.03 (0.02)	-0.08 (0.02)
0.22 (0.05)	0.15 (0.05)	0.25 (0.04)	0.23 (0.04)	0.22 (0.04)	0.19 (0.04)
-0.00 (0.02)	0.04 (0.02)	0.04 (0.02)	0.06 (0.02)	0.05 (0.02)	0.02 (0.02)
0.25 (0.06)	0.24 (0.05)	0.28 (0.05)	0.27 (0.04)	0.25 (0.04)	0.24 (0.04)
0.38 (0.03)	0.40 (0.03)	0.41 (0.03)	0.49 (0.03)	0.48 (0.02)	0.46 (0.02)
0.06 (0.04)	0.02 (0.03)	0.00 (0.03)	0.02 (0.03)	-0.02 (0.03)	-0.00 (0.03)
0.38 (0.03)	0.37 (0.02)	0.34 (0.02)	0.32 (0.02)	0.33 (0.02)	0.31 (0.02)
-0.10 (0.05)	-0.12 (0.05)	-0.08 (0.05)	-0.13 (0.04)	-0.12 (0.04)	-0.18 (0.04)
0.14 (0.08)	0.09 (0.07)	0.17 (0.07)	0.06 (0.07)	0.12 (0.06)	0.12 (0.06)
-0.43 (0.07)	-0.41 (0.07)	-0.51 (0.07)	-0.43 (0.06)	-0.43 (0.06)	-0.49 (0.06)
-0.05 (0.04)	-0.06 (0.04)	-0.04 (0.04)	-0.02 (0.04)	-0.07 (0.04)	-0.14 (0.04)
0.04 (0.08)	0.06 (0.08)	0.05 (0.07)	0.04 (0.07)	0.01 (0.07)	0.05 (0.06)
0.27 (0.03)	0.26 (0.03)	0.22 (0.03)	0.26 (0.03)	0.26 (0.03)	0.22 (0.03)
0.01 (0.06)	0.03 (0.05)	-0.08 (0.05)	-0.05 (0.05)	0.01 (0.05)	-0.03 (0.04)
0.42 (0.07)	0.41 (0.06)	0.48 (0.06)	0.46 (0.06)	0.38 (0.05)	0.37 (0.05)
0.41 (0.05)	0.37 (0.04)	0.36 (0.04)	0.38 (0.04)	0.31 (0.04)	0.30 (0.04)
0.17 (0.05)	0.10 (0.05)	0.20 (0.04)	0.18 (0.04)	0.21 (0.04)	0.24 (0.04)
0.17 (0.05)	0.17 (0.05)	0.25 (0.05)	0.21 (0.05)	0.23 (0.05)	0.25 (0.05)
0.16 (0.09)	-0.04 (0.09)	0.12 (0.10)	0.10 (0.10)	0.10 (0.10)	0.07 (0.10)
NA	0.18 (0.28)	0.11 (0.27)	0.27 (0.25)	0.15 (0.22)	0.27 (0.20)
0.15 (0.01)	0.13 (0.01)	0.14 (0.01)	0.13 (0.01)	0.14 (0.01)	0.12 (0.01)

For peer review only

SIMD 10
-0.19 (0.03)
0.22 (0.02)
0.26 (0.04)
0.12 (0.02)
0.07 (0.02)
0.27 (0.05)
0.30 (0.03)
0.36 (0.06)
-0.11 (0.02)
0.29 (0.04)
-0.06 (0.02)
0.24 (0.03)
0.47 (0.02)
-0.01 (0.03)
0.27 (0.02)
-0.15 (0.04)
0.15 (0.05)
-0.41 (0.06)
0.01 (0.03)
0.01 (0.06)
0.19 (0.03)
-0.05 (0.04)
0.46 (0.05)
0.24 (0.04)
0.31 (0.03)
0.22 (0.05)
-0.04 (0.10)
0.43 (0.18)
0.13 (0.01)

For peer review only

ICD10	Unstratified	Male	Female	SIMD 1	SIMD 2	SIMD 3
J09-J18	0.15 (0.04)	0.11 (0.06)	0.19 (0.03)	-0.08 (0.22)	0.19 (0.13)	0.05 (0.25)
R00-R09	0.23 (0.04)	0.22 (0.05)	0.24 (0.05)	0.18 (0.15)	0.24 (0.11)	0.24 (0.12)
C30-C39	0.21 (0.05)	0.21 (0.04)	0.20 (0.07)	0.22 (0.23)	0.15 (0.19)	0.34 (0.18)
R10-R19	0.12 (0.04)	0.10 (0.05)	0.13 (0.05)	0.33 (0.11)	-0.07 (0.08)	-0.08 (0.18)
R50-R69	0.03 (0.03)	0.07 (0.03)	-0.02 (0.04)	0.06 (0.13)	-0.13 (0.11)	-0.16 (0.08)
C15-C26	0.40 (0.04)	0.37 (0.05)	0.43 (0.05)	0.50 (0.10)	0.50 (0.13)	0.45 (0.16)
I30-I52	0.18 (0.02)	0.29 (0.03)	0.08 (0.03)	0.38 (0.11)	0.20 (0.07)	-0.04 (0.13)
C76-C80	0.37 (0.07)	0.21 (0.09)	0.50 (0.05)	0.35 (0.14)	0.06 (0.24)	0.46 (0.19)
Z40-Z54	0.30 (0.04)	0.36 (0.06)	0.21 (0.03)	0.51 (0.12)	0.30 (0.12)	0.14 (0.06)
I60-I69	0.20 (0.03)	0.27 (0.05)	0.15 (0.04)	0.34 (0.10)	0.11 (0.07)	-0.03 (0.09)
K55-K63	0.22 (0.02)	0.30 (0.03)	0.16 (0.03)	0.37 (0.10)	0.35 (0.12)	0.16 (0.10)
J40-J47	0.24 (0.05)	0.20 (0.08)	0.27 (0.04)	-0.05 (0.24)	0.30 (0.10)	0.22 (0.13)
I20-I25	0.26 (0.03)	0.35 (0.03)	0.17 (0.05)	0.27 (0.21)	0.27 (0.12)	0.10 (0.10)
N30-N39	-0.21 (0.04)	-0.29 (0.04)	-0.16 (0.05)	0.00 (0.07)	-0.13 (0.11)	-0.26 (0.07)
K20-K31	0.13 (0.05)	0.11 (0.05)	0.16 (0.07)	-0.05 (0.10)	-0.11 (0.11)	0.01 (0.14)
J20-J22	-0.11 (0.02)	-0.09 (0.05)	-0.12 (0.03)	-0.32 (0.16)	-0.22 (0.08)	-0.07 (0.09)
S70-S79	-0.02 (0.02)	0.09 (0.06)	-0.07 (0.02)	0.26 (0.15)	-0.03 (0.10)	0.10 (0.10)
A30-A49	0.06 (0.10)	0.02 (0.14)	0.12 (0.10)	0.32 (0.24)	-0.30 (0.23)	-0.33 (0.26)
T80-T88	0.18 (0.06)	0.16 (0.10)	0.23 (0.05)	0.19 (0.25)	0.06 (0.15)	0.05 (0.06)
N17-N19	0.20 (0.06)	0.14 (0.06)	0.26 (0.08)	0.02 (0.25)	-0.09 (0.21)	0.23 (0.14)
I80-I89	0.24 (0.05)	0.37 (0.05)	0.09 (0.06)	0.42 (0.22)	0.05 (0.23)	0.10 (0.19)
K90-K93	0.19 (0.07)	0.22 (0.08)	0.12 (0.08)	-0.35 (0.13)	-0.08 (0.25)	-0.16 (0.20)
I70-I79	0.09 (0.04)	0.14 (0.04)	0.02 (0.06)	0.43 (0.20)	-0.11 (0.12)	0.09 (0.12)
K50-K52	0.12 (0.03)	0.14 (0.03)	0.09 (0.04)	-0.17 (0.16)	0.29 (0.13)	-0.12 (0.24)
S00-S09	0.02 (0.05)	0.18 (0.05)	-0.16 (0.06)	-0.15 (0.18)	0.25 (0.16)	-0.05 (0.12)
C50-C50	1.16 (0.07)	1.15 (0.64)	1.15 (0.07)	0.56 (0.24)	1.74 (0.24)	1.70 (0.28)
C60-C63	0.70 (0.07)	0.70 (0.07)	NA	0.55 (0.27)	0.92 (0.19)	0.23 (0.19)
G30-G32	0.04 (0.14)	-0.04 (0.27)	0.06 (0.11)	0.15 (0.33)	0.25 (0.46)	-0.36 (0.33)
TOTAL	0.19 (0.04)	0.19 (0.05)	0.18 (0.05)	0.22 (0.16)	0.16 (0.13)	0.12 (0.14)

For peer review only

SIMD 4	SIMD 5	SIMD 6	SIMD 7	SIMD 8	SIMD 9
-0.02 (0.14)	0.20 (0.12)	0.32 (0.16)	0.19 (0.06)	0.19 (0.09)	0.11 (0.10)
0.28 (0.10)	0.03 (0.09)	0.22 (0.09)	0.19 (0.06)	0.30 (0.07)	0.19 (0.06)
0.39 (0.20)	0.12 (0.15)	0.34 (0.11)	-0.11 (0.13)	0.22 (0.12)	0.26 (0.14)
0.17 (0.09)	0.02 (0.07)	0.15 (0.11)	0.15 (0.07)	0.16 (0.11)	0.09 (0.14)
-0.16 (0.07)	-0.11 (0.04)	-0.09 (0.07)	0.22 (0.05)	0.14 (0.06)	-0.01 (0.06)
0.45 (0.07)	0.54 (0.15)	0.26 (0.10)	0.22 (0.12)	0.29 (0.12)	0.29 (0.12)
0.28 (0.12)	0.23 (0.04)	0.25 (0.07)	0.26 (0.08)	0.12 (0.05)	0.04 (0.09)
0.43 (0.22)	0.28 (0.18)	0.58 (0.19)	0.48 (0.20)	0.52 (0.18)	0.41 (0.15)
0.29 (0.07)	0.41 (0.07)	0.18 (0.09)	0.34 (0.06)	0.40 (0.08)	0.18 (0.11)
0.06 (0.07)	-0.09 (0.18)	0.33 (0.08)	0.22 (0.12)	0.16 (0.13)	0.34 (0.18)
0.13 (0.05)	0.32 (0.08)	0.11 (0.06)	0.23 (0.08)	0.16 (0.04)	0.03 (0.05)
0.30 (0.15)	0.11 (0.14)	0.09 (0.18)	0.22 (0.09)	0.18 (0.03)	0.37 (0.07)
0.37 (0.06)	0.23 (0.12)	0.29 (0.08)	0.24 (0.13)	0.41 (0.04)	0.18 (0.09)
-0.28 (0.07)	-0.17 (0.07)	-0.16 (0.10)	-0.30 (0.14)	-0.17 (0.07)	-0.28 (0.07)
0.39 (0.12)	0.06 (0.10)	0.11 (0.04)	0.02 (0.10)	0.16 (0.14)	0.18 (0.09)
-0.24 (0.08)	-0.17 (0.10)	-0.18 (0.11)	-0.11 (0.07)	0.14 (0.07)	-0.16 (0.06)
-0.22 (0.12)	-0.09 (0.07)	-0.12 (0.06)	0.16 (0.11)	-0.09 (0.11)	0.00 (0.12)
-0.29 (0.14)	0.42 (0.22)	-0.61 (0.18)	0.53 (0.33)	0.37 (0.14)	0.13 (0.19)
0.20 (0.16)	0.17 (0.06)	0.43 (0.10)	0.20 (0.15)	0.26 (0.12)	0.00 (0.11)
0.01 (0.06)	0.53 (0.21)	0.18 (0.19)	0.20 (0.15)	0.11 (0.11)	0.43 (0.17)
0.62 (0.31)	0.21 (0.19)	0.08 (0.18)	0.49 (0.15)	0.28 (0.13)	0.06 (0.12)
0.08 (0.16)	0.05 (0.16)	0.25 (0.13)	0.16 (0.09)	0.21 (0.14)	0.26 (0.15)
0.26 (0.15)	0.26 (0.18)	0.04 (0.19)	-0.00 (0.14)	-0.05 (0.10)	0.06 (0.06)
-0.38 (0.20)	0.35 (0.13)	0.15 (0.11)	0.09 (0.08)	0.03 (0.13)	0.24 (0.11)
-0.34 (0.15)	-0.21 (0.19)	0.22 (0.19)	-0.01 (0.18)	0.01 (0.08)	-0.09 (0.12)
1.45 (0.12)	1.17 (0.17)	1.59 (0.31)	0.96 (0.21)	0.88 (0.19)	1.03 (0.21)
0.62 (0.19)	0.88 (0.22)	0.86 (0.19)	0.38 (0.24)	0.77 (0.13)	1.29 (0.28)
-1.27 (0.28)	0.88 (0.35)	0.45 (0.43)	-1.24 (0.59)	0.55 (0.42)	0.30 (0.28)
0.18 (0.12)	0.17 (0.12)	0.19 (0.12)	0.18 (0.11)	0.21 (0.10)	0.16 (0.11)

For peer review only

SIMD 10
0.20 (0.07)
0.34 (0.09)
0.19 (0.12)
0.16 (0.08)
0.23 (0.06)
0.49 (0.14)
0.07 (0.10)
-0.14 (0.25)
0.24 (0.05)
0.39 (0.10)
0.44 (0.07)
0.32 (0.09)
0.24 (0.08)
-0.32 (0.06)
0.28 (0.10)
-0.03 (0.12)
-0.18 (0.11)
-0.22 (0.17)
0.20 (0.09)
0.15 (0.18)
0.14 (0.16)
0.63 (0.16)
0.07 (0.16)
0.24 (0.10)
0.18 (0.14)
0.81 (0.17)
0.62 (0.16)
-0.43 (0.12)
0.20 (0.11)

For peer review only

ICD10	Unstratified	Male	Female	SIMD 1	SIMD 2	SIMD 3
J09-J18	-0.02 (0.02)	-0.01 (0.03)	-0.04 (0.03)	-0.28 (0.12)	-0.03 (0.08)	-0.10 (0.12)
R00-R09	0.37 (0.02)	0.39 (0.03)	0.35 (0.02)	0.31 (0.08)	0.35 (0.06)	0.36 (0.06)
C30-C39	0.47 (0.03)	0.62 (0.03)	0.34 (0.04)	0.43 (0.14)	0.44 (0.12)	0.67 (0.11)
R10-R19	0.26 (0.02)	0.26 (0.03)	0.25 (0.03)	0.41 (0.06)	0.03 (0.05)	0.06 (0.09)
R50-R69	0.13 (0.02)	0.21 (0.02)	0.05 (0.02)	0.15 (0.07)	-0.04 (0.06)	-0.09 (0.05)
C15-C26	0.65 (0.02)	0.64 (0.03)	0.65 (0.03)	0.76 (0.07)	0.78 (0.08)	0.72 (0.09)
I30-I52	0.42 (0.02)	0.53 (0.02)	0.30 (0.02)	0.57 (0.07)	0.47 (0.05)	0.18 (0.07)
C76-C80	0.56 (0.04)	0.44 (0.05)	0.68 (0.04)	0.42 (0.10)	0.21 (0.13)	0.61 (0.11)
Z40-Z54	0.28 (0.02)	0.35 (0.03)	0.19 (0.02)	0.52 (0.07)	0.28 (0.06)	0.15 (0.04)
I60-I69	0.43 (0.02)	0.51 (0.03)	0.36 (0.03)	0.53 (0.08)	0.27 (0.06)	0.14 (0.07)
K55-K63	0.26 (0.01)	0.32 (0.02)	0.22 (0.02)	0.42 (0.05)	0.43 (0.06)	0.18 (0.06)
J40-J47	0.50 (0.03)	0.54 (0.04)	0.48 (0.03)	0.30 (0.14)	0.56 (0.08)	0.52 (0.09)
I20-I25	0.70 (0.02)	0.76 (0.02)	0.64 (0.03)	0.67 (0.10)	0.65 (0.06)	0.48 (0.06)
N30-N39	-0.23 (0.02)	-0.23 (0.03)	-0.22 (0.03)	0.11 (0.05)	-0.09 (0.06)	-0.23 (0.05)
K20-K31	0.47 (0.02)	0.47 (0.03)	0.46 (0.04)	0.25 (0.06)	0.22 (0.06)	0.39 (0.07)
J20-J22	-0.23 (0.02)	-0.16 (0.03)	-0.28 (0.03)	-0.34 (0.11)	-0.35 (0.07)	-0.23 (0.07)
S70-S79	0.10 (0.02)	0.19 (0.04)	0.06 (0.03)	0.32 (0.11)	0.05 (0.10)	0.25 (0.09)
A30-A49	-0.38 (0.05)	-0.41 (0.07)	-0.33 (0.06)	-0.01 (0.15)	-0.78 (0.13)	-0.65 (0.15)
T80-T88	0.15 (0.03)	0.13 (0.05)	0.18 (0.03)	0.22 (0.12)	0.01 (0.08)	0.00 (0.05)
N17-N19	0.21 (0.04)	0.21 (0.04)	0.22 (0.05)	0.04 (0.16)	-0.03 (0.14)	0.25 (0.11)
I80-I89	0.46 (0.03)	0.58 (0.03)	0.33 (0.03)	0.65 (0.11)	0.20 (0.11)	0.35 (0.10)
K90-K93	0.19 (0.04)	0.26 (0.05)	0.07 (0.04)	-0.44 (0.09)	-0.10 (0.13)	-0.16 (0.11)
I70-I79	0.52 (0.03)	0.53 (0.03)	0.51 (0.04)	0.92 (0.13)	0.34 (0.10)	0.57 (0.09)
K50-K52	0.45 (0.02)	0.50 (0.03)	0.42 (0.03)	0.17 (0.10)	0.72 (0.08)	0.20 (0.12)
S00-S09	0.11 (0.03)	0.34 (0.03)	-0.15 (0.04)	-0.19 (0.11)	0.25 (0.10)	0.05 (0.08)
C50-C50	1.36 (0.04)	NA	1.35 (0.04)	0.78 (0.13)	1.92 (0.13)	1.88 (0.14)
C60-C63	0.76 (0.04)	0.76 (0.04)	NA	0.53 (0.15)	0.87 (0.13)	0.21 (0.13)
G30-G32	NA	0.28 (0.17)	0.30 (0.14)	NA	NA	NA
TOTAL	0.31 (0.01)	0.35 (0.01)	0.28 (0.01)	0.34 (0.02)	0.28 (0.02)	0.25 (0.02)

For peer review only

SIMD 4	SIMD 5	SIMD 6	SIMD 7	SIMD 8	SIMD 9
-0.17 (0.08)	0.04 (0.07)	0.16 (0.09)	0.03 (0.05)	0.01 (0.06)	-0.10 (0.06)
0.39 (0.05)	0.15 (0.05)	0.36 (0.05)	0.38 (0.04)	0.46 (0.04)	0.37 (0.03)
0.74 (0.12)	0.44 (0.10)	0.67 (0.08)	0.14 (0.08)	0.51 (0.08)	0.45 (0.08)
0.32 (0.05)	0.15 (0.04)	0.34 (0.06)	0.28 (0.04)	0.31 (0.06)	0.20 (0.07)
-0.02 (0.05)	-0.03 (0.04)	0.02 (0.05)	0.34 (0.04)	0.27 (0.04)	0.09 (0.04)
0.70 (0.06)	0.82 (0.09)	0.48 (0.07)	0.45 (0.07)	0.58 (0.07)	0.51 (0.08)
0.48 (0.07)	0.42 (0.04)	0.50 (0.05)	0.51 (0.05)	0.38 (0.04)	0.32 (0.06)
0.67 (0.12)	0.46 (0.11)	0.80 (0.11)	0.64 (0.11)	0.73 (0.11)	0.68 (0.09)
0.33 (0.04)	0.44 (0.05)	0.17 (0.05)	0.32 (0.04)	0.40 (0.05)	0.09 (0.06)
0.29 (0.06)	0.09 (0.09)	0.59 (0.06)	0.46 (0.07)	0.39 (0.07)	0.56 (0.09)
0.16 (0.03)	0.37 (0.05)	0.18 (0.04)	0.31 (0.04)	0.22 (0.03)	0.06 (0.03)
0.59 (0.09)	0.35 (0.08)	0.37 (0.10)	0.50 (0.06)	0.43 (0.04)	0.61 (0.05)
0.77 (0.04)	0.63 (0.06)	0.71 (0.05)	0.71 (0.07)	0.90 (0.03)	0.65 (0.05)
-0.24 (0.05)	-0.18 (0.05)	-0.19 (0.06)	-0.32 (0.07)	-0.21 (0.05)	-0.33 (0.05)
0.79 (0.06)	0.43 (0.06)	0.45 (0.03)	0.35 (0.05)	0.49 (0.07)	0.49 (0.05)
-0.32 (0.07)	-0.27 (0.07)	-0.26 (0.07)	-0.22 (0.06)	0.04 (0.05)	-0.34 (0.05)
-0.07 (0.10)	0.01 (0.08)	0.05 (0.08)	0.23 (0.08)	0.03 (0.08)	0.13 (0.08)
-0.72 (0.10)	-0.03 (0.13)	-1.12 (0.11)	0.10 (0.17)	-0.07 (0.09)	-0.36 (0.11)
0.17 (0.09)	0.12 (0.05)	0.41 (0.06)	0.18 (0.08)	0.21 (0.07)	-0.12 (0.07)
0.04 (0.09)	0.56 (0.13)	0.20 (0.12)	0.21 (0.10)	0.11 (0.08)	0.43 (0.10)
0.92 (0.15)	0.46 (0.10)	0.32 (0.09)	0.72 (0.08)	0.56 (0.07)	0.25 (0.07)
0.15 (0.10)	0.12 (0.10)	0.21 (0.08)	0.14 (0.07)	0.25 (0.08)	0.28 (0.09)
0.66 (0.10)	0.67 (0.11)	0.48 (0.11)	0.45 (0.09)	0.34 (0.07)	0.44 (0.06)
-0.01 (0.11)	0.71 (0.08)	0.51 (0.07)	0.44 (0.06)	0.34 (0.08)	0.52 (0.07)
-0.20 (0.09)	-0.19 (0.10)	0.34 (0.10)	0.09 (0.09)	0.16 (0.06)	0.04 (0.07)
1.64 (0.08)	1.34 (0.10)	1.82 (0.15)	1.18 (0.12)	1.06 (0.11)	1.27 (0.11)
0.79 (0.13)	0.89 (0.14)	1.03 (0.13)	0.50 (0.15)	0.89 (0.12)	1.38 (0.17)
NA	NA	NA	NA	0.73 (0.30)	0.57 (0.25)
0.33 (0.02)	0.30 (0.02)	0.33 (0.02)	0.31 (0.01)	0.35 (0.01)	0.27 (0.01)

For peer review only

SIMD 10
0.01 (0.05)
0.51 (0.05)
0.37 (0.07)
0.29 (0.05)
0.30 (0.04)
0.73 (0.08)
0.37 (0.06)
0.13 (0.13)
0.14 (0.04)
0.70 (0.06)
0.39 (0.04)
0.55 (0.06)
0.72 (0.05)
-0.37 (0.04)
0.56 (0.05)
-0.18 (0.07)
-0.04 (0.08)
-0.63 (0.10)
0.21 (0.06)
0.11 (0.10)
0.27 (0.09)
0.57 (0.09)
0.49 (0.09)
0.48 (0.06)
0.32 (0.08)
1.00 (0.10)
0.63 (0.13)
-0.00 (0.19)
0.31 (0.01)

For peer review only

ICD10	Unstratified	Male	Female	SIMD 1	SIMD 2	SIMD 3
J09-J18	0.45 (0.12)	0.47 (0.25)	0.45 (0.08)	0.26 (0.73)	0.43 (0.34)	0.25 (1.19)
R00-R09	0.60 (0.12)	0.52 (0.14)	0.67 (0.16)	0.62 (0.61)	0.67 (0.38)	0.68 (0.41)
C30-C39	0.39 (0.09)	0.33 (0.06)	0.52 (0.20)	0.48 (0.56)	0.31 (0.40)	0.47 (0.28)
R10-R19	0.53 (0.23)	0.43 (0.21)	0.59 (0.27)	0.92 (0.44)	0.51 (0.65)	0.42 (0.98)
R50-R69	0.25 (0.25)	0.36 (0.18)	0.39 (1.08)	0.67 (1.72)	0.79 (0.90)	0.80 (0.55)
C15-C26	0.60 (0.06)	0.55 (0.08)	0.65 (0.09)	0.66 (0.16)	0.64 (0.21)	0.59 (0.25)
I30-I52	0.44 (0.06)	0.55 (0.07)	0.27 (0.10)	0.70 (0.24)	0.43 (0.16)	0.16 (0.59)
C76-C80	0.62 (0.13)	0.44 (0.20)	0.72 (0.10)	0.86 (0.48)	0.26 (1.05)	0.73 (0.37)
Z40-Z54	0.89 (0.14)	0.93 (0.20)	0.82 (0.14)	0.94 (0.31)	0.87 (0.45)	0.93 (0.57)
I60-I69	0.49 (0.09)	0.54 (0.12)	0.45 (0.12)	0.67 (0.26)	0.45 (0.30)	0.18 (0.46)
K55-K63	0.92 (0.13)	0.97 (0.13)	0.84 (0.24)	0.93 (0.33)	0.87 (0.40)	0.96 (0.90)
J40-J47	0.48 (0.10)	0.38 (0.15)	0.56 (0.09)	0.12 (0.55)	0.56 (0.22)	0.41 (0.27)
I20-I25	0.38 (0.05)	0.46 (0.05)	0.26 (0.08)	0.41 (0.35)	0.43 (0.20)	0.21 (0.21)
N30-N39	0.89 (0.22)	0.76 (0.12)	0.89 (0.40)	0.01 (0.54)	0.65 (0.67)	0.87 (0.33)
K20-K31	0.29 (0.11)	0.22 (0.11)	0.35 (0.18)	0.15 (0.28)	0.25 (0.28)	0.03 (0.36)
J20-J22	0.45 (0.11)	0.53 (0.32)	0.41 (0.10)	0.94 (0.70)	0.62 (0.27)	0.25 (0.37)
S70-S79	0.14 (0.16)	0.50 (0.39)	0.34 (0.13)	0.92 (0.78)	0.32 (1.23)	0.40 (0.46)
A30-A49	0.13 (0.21)	0.05 (0.33)	0.20 (0.17)	0.48 (0.40)	0.38 (0.30)	0.51 (0.47)
T80-T88	0.77 (0.34)	0.86 (0.70)	0.75 (0.22)	0.96 (1.77)	0.38 (1.05)	0.44 (0.56)
N17-N19	0.83 (0.32)	0.61 (0.32)	0.96 (0.43)	0.38 (4.95)	0.59 (1.70)	0.78 (0.65)
I80-I89	0.50 (0.11)	0.64 (0.11)	0.25 (0.18)	0.62 (0.40)	0.18 (0.81)	0.28 (0.56)
K90-K93	0.83 (0.41)	0.91 (0.47)	0.51 (0.37)	0.72 (0.33)	0.49 (1.78)	0.75 (1.17)
I70-I79	0.17 (0.07)	0.26 (0.09)	0.04 (0.11)	0.43 (0.23)	0.19 (0.22)	0.15 (0.21)
K50-K52	0.25 (0.07)	0.28 (0.06)	0.22 (0.09)	0.32 (0.32)	0.40 (0.20)	0.28 (0.55)
S00-S09	0.08 (0.24)	0.41 (0.12)	0.74 (0.36)	0.80 (1.27)	0.87 (0.77)	0.26 (0.69)
C50-C50	0.85 (0.07)	NA	0.85 (0.07)	0.74 (0.39)	0.91 (0.17)	0.91 (0.21)
C60-C63	0.97 (0.14)	0.97 (0.14)	NA	0.89 (0.59)	0.89 (0.25)	0.85 (0.97)
G30-G32	0.12 (0.47)	0.11 (0.80)	0.22 (0.40)	NA	NA	0.69 (0.86)
TOTAL	0.59 (0.15)	0.54 (0.17)	0.65 (0.21)	0.68 (0.59)	0.59 (0.58)	0.48 (0.64)

For peer review only

SIMD 4	SIMD 5	SIMD 6	SIMD 7	SIMD 8	SIMD 9
0.10 (0.78)	0.53 (0.37)	0.67 (0.41)	0.53 (0.21)	0.48 (0.25)	0.35 (0.36)
0.72 (0.32)	0.21 (0.69)	0.63 (0.32)	0.48 (0.18)	0.62 (0.18)	0.48 (0.16)
0.45 (0.26)	0.24 (0.33)	0.47 (0.17)	0.26 (0.32)	0.41 (0.24)	0.49 (0.30)
0.60 (0.37)	0.17 (0.53)	0.53 (0.45)	0.57 (0.31)	0.57 (0.45)	0.47 (0.78)
0.59 (0.34)	0.61 (0.28)	0.48 (0.46)	0.66 (0.19)	0.59 (0.29)	0.11 (0.66)
0.63 (0.13)	0.63 (0.21)	0.53 (0.23)	0.47 (0.29)	0.51 (0.24)	0.56 (0.28)
0.63 (0.31)	0.55 (0.11)	0.53 (0.18)	0.55 (0.19)	0.31 (0.14)	0.13 (0.29)
0.57 (0.34)	0.59 (0.44)	0.74 (0.30)	0.73 (0.38)	0.68 (0.29)	0.59 (0.25)
0.94 (0.32)	0.97 (0.25)	0.86 (0.54)	0.91 (0.22)	0.93 (0.26)	0.68 (0.51)
0.21 (0.28)	0.38 (0.77)	0.57 (0.16)	0.50 (0.29)	0.43 (0.36)	0.64 (0.39)
0.99 (0.52)	0.89 (0.31)	0.72 (0.51)	0.80 (0.34)	0.78 (0.23)	0.61 (1.32)
0.54 (0.32)	0.31 (0.41)	0.25 (0.52)	0.45 (0.20)	0.42 (0.10)	0.61 (0.14)
0.50 (0.09)	0.36 (0.21)	0.41 (0.13)	0.34 (0.19)	0.46 (0.05)	0.28 (0.14)
0.82 (0.28)	0.91 (0.57)	0.99 (0.88)	0.93 (0.60)	0.90 (0.51)	0.99 (0.36)
0.50 (0.17)	0.14 (0.24)	0.24 (0.10)	0.06 (0.29)	0.33 (0.29)	0.37 (0.19)
0.70 (0.32)	0.59 (0.42)	0.68 (0.52)	0.46 (0.34)	0.54 (0.31)	0.47 (0.21)
0.61 (0.41)	0.50 (0.47)	0.42 (0.25)	0.72 (0.63)	0.42 (0.57)	0.02 (1.00)
0.40 (0.21)	0.51 (0.30)	0.54 (0.18)	0.55 (0.39)	0.46 (0.19)	0.21 (0.31)
0.81 (0.86)	0.75 (0.36)	0.92 (0.31)	0.89 (0.92)	0.79 (0.47)	0.00 (0.85)
0.12 (1.36)	0.89 (0.49)	0.78 (1.11)	0.84 (0.86)	0.89 (1.19)	0.89 (0.48)
0.70 (0.42)	0.45 (0.44)	0.26 (0.62)	0.66 (0.24)	0.52 (0.27)	0.21 (0.44)
0.86 (2.48)	0.59 (2.44)	0.76 (0.50)	0.78 (0.61)	0.95 (0.86)	0.88 (0.71)
0.38 (0.24)	0.39 (0.29)	0.08 (0.36)	0.01 (0.29)	0.11 (0.24)	0.14 (0.15)
0.48 (0.29)	0.49 (0.20)	0.29 (0.23)	0.20 (0.18)	0.08 (0.40)	0.45 (0.23)
0.67 (0.37)	0.67 (0.72)	0.53 (0.53)	0.03 (0.96)	0.05 (0.37)	0.28 (0.36)
0.89 (0.10)	0.87 (0.17)	0.87 (0.22)	0.82 (0.24)	0.79 (0.22)	0.80 (0.21)
0.79 (0.32)	0.96 (0.35)	0.88 (0.27)	0.80 (0.68)	0.89 (0.23)	0.95 (0.29)
NA	0.83 (0.49)	0.81 (1.07)	0.82 (0.52)	0.79 (0.80)	0.53 (0.57)
0.54 (0.42)	0.58 (0.45)	0.58 (0.41)	0.57 (0.41)	0.61 (0.32)	0.57 (0.44)

For peer review only

SIMD 10
0.51 (0.20)
0.61 (0.19)
0.42 (0.29)
0.58 (0.35)
0.77 (0.26)
0.64 (0.22)
0.19 (0.27)
0.28 (0.53)
0.68 (0.18)
0.57 (0.17)
0.87 (0.18)
0.57 (0.19)
0.33 (0.12)
0.98 (0.29)
0.51 (0.20)
0.17 (0.67)
0.55 (0.41)
0.35 (0.28)
0.97 (0.60)
0.93 (1.53)
0.42 (0.54)
0.92 (0.32)
0.12 (0.31)
0.50 (0.25)
0.37 (0.31)
0.78 (0.21)
0.93 (0.36)
0.50 (0.19)
0.60 (0.38)

For peer review only

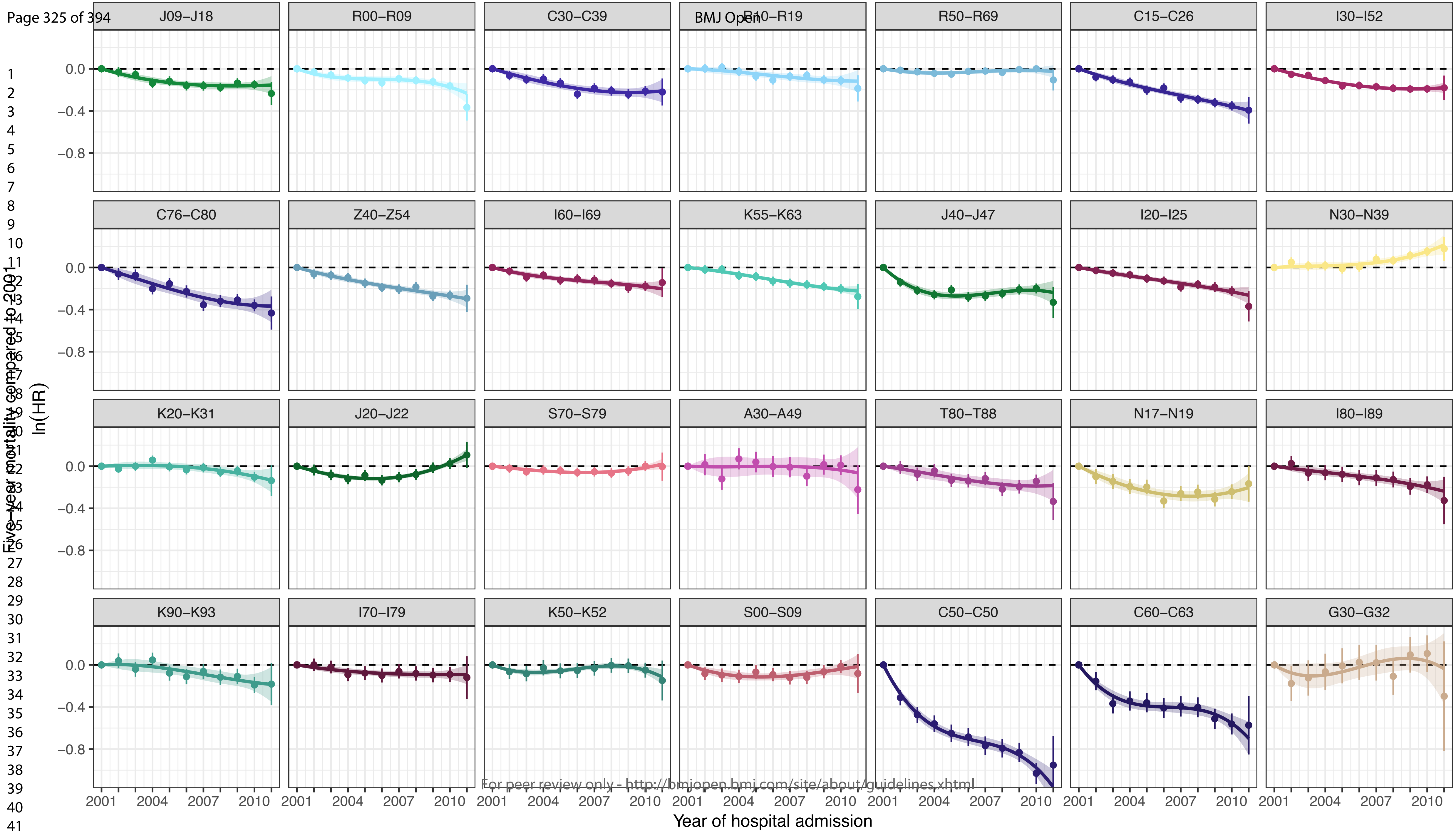
J09–J18
R00–R09
C30–C39
R10–R19
R50–R69
C15–C26
I30–I52
C76–C80
Z40–Z54
I60–I69
K55–K63
J40–J47
I20–I25
N30–N39
K20–K31
J20–J22
S70–S79
A30–A49
T80–T88
N17–N19
I80–I89
K90–K93
I70–I79
K50–K52
S00–S09
C50–C50
C60–C63
G30–G32
0.0
−0.4
−0.8
2001
2004
2007
2010
Five−year mortality compared to 2001
ln(HR)
Year of hospital admission

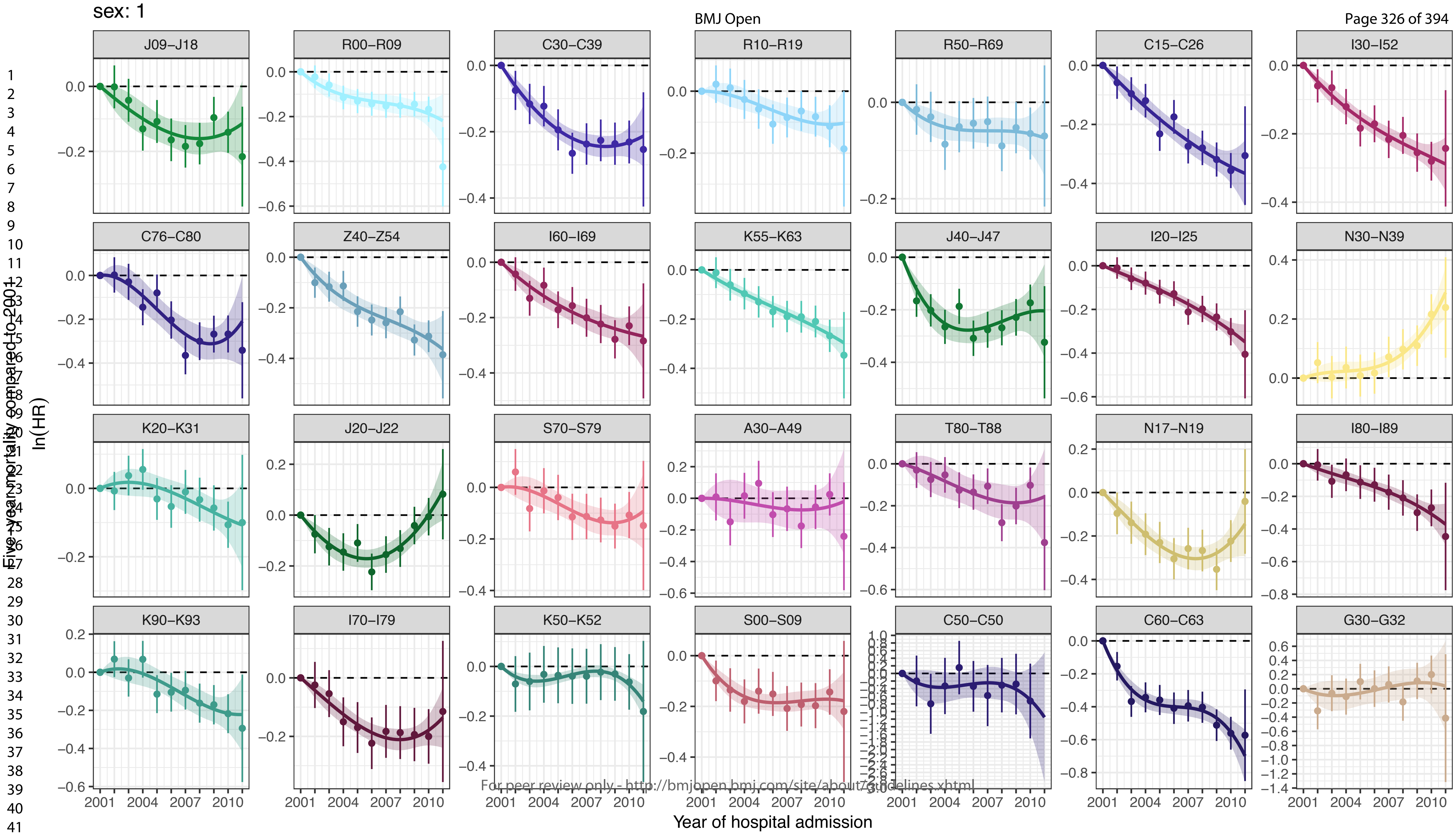
sex: 1
J09–J18
R00–R09
C30–C39
R10–R19
R50–R69
C15–C26
I30–I52
C76–C80
Z40–Z54
I60–I69
K55–K63
J40–J47
I20–I25
N30–N39
K20–K31
J20–J22
S70–S79
A30–A49
T80–T88
N17–N19
I80–I89
K90–K93
I70–I79
K50–K52
S00–S09
C50–C50
C60–C63
G30–G32
Five-year mortality compared to 2001
ln(HR)
Year of hospital admission

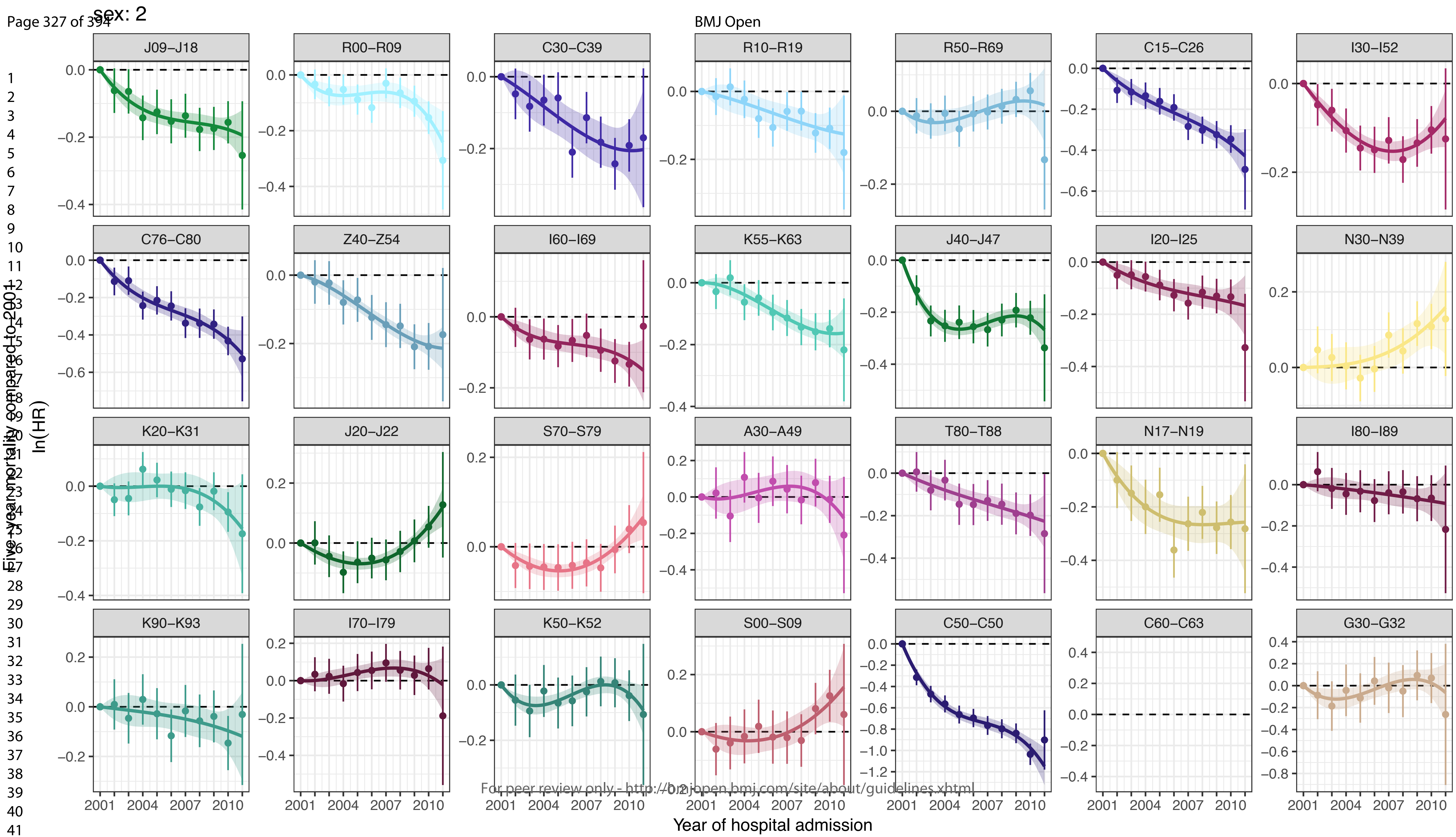

For peer review only - http://bmjopen.bmj.com/site/about/guidelines.xhtml

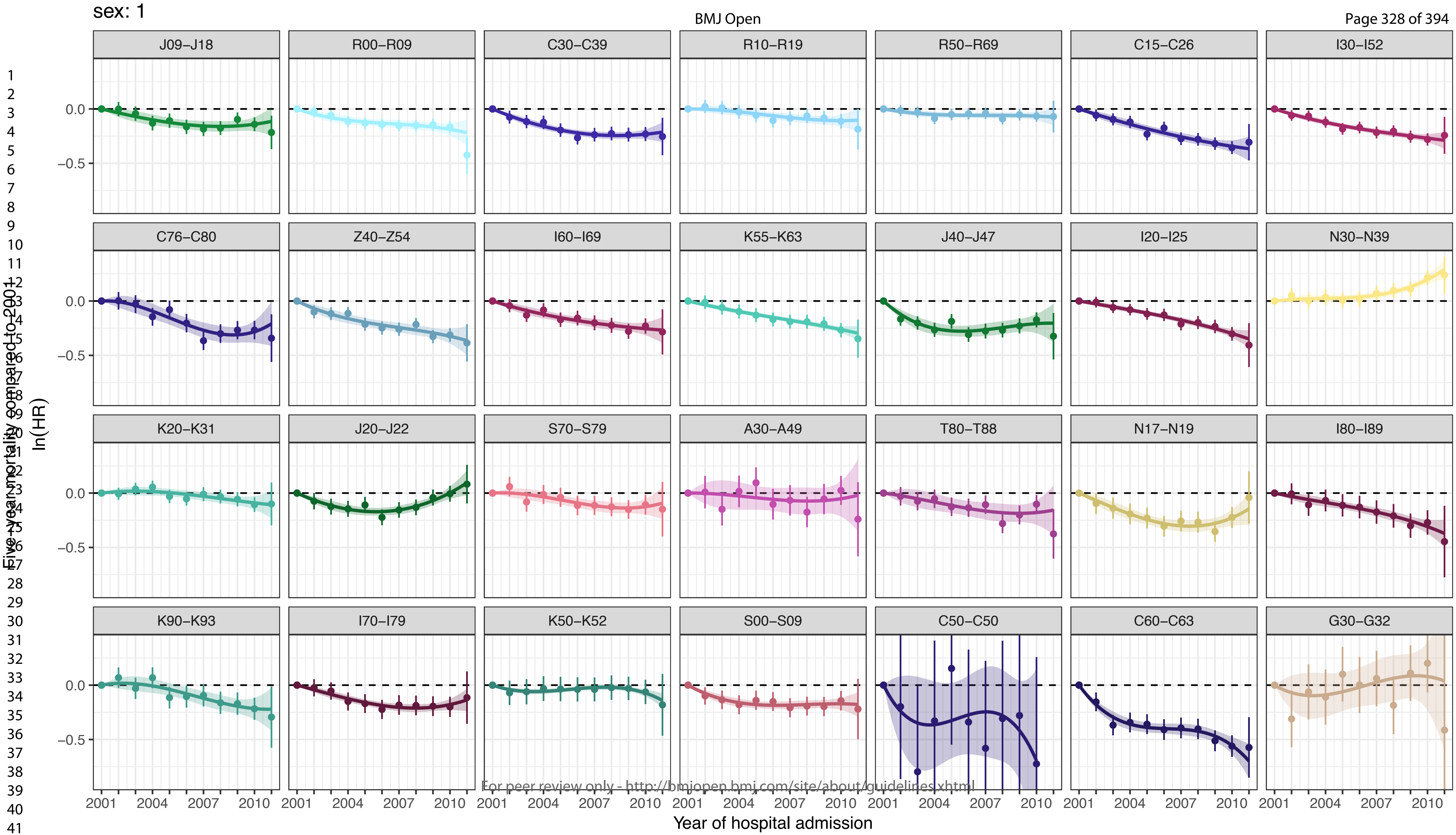

For peer review only - http://bmjopen.bmj.com/site/about/guidelines.xhtml

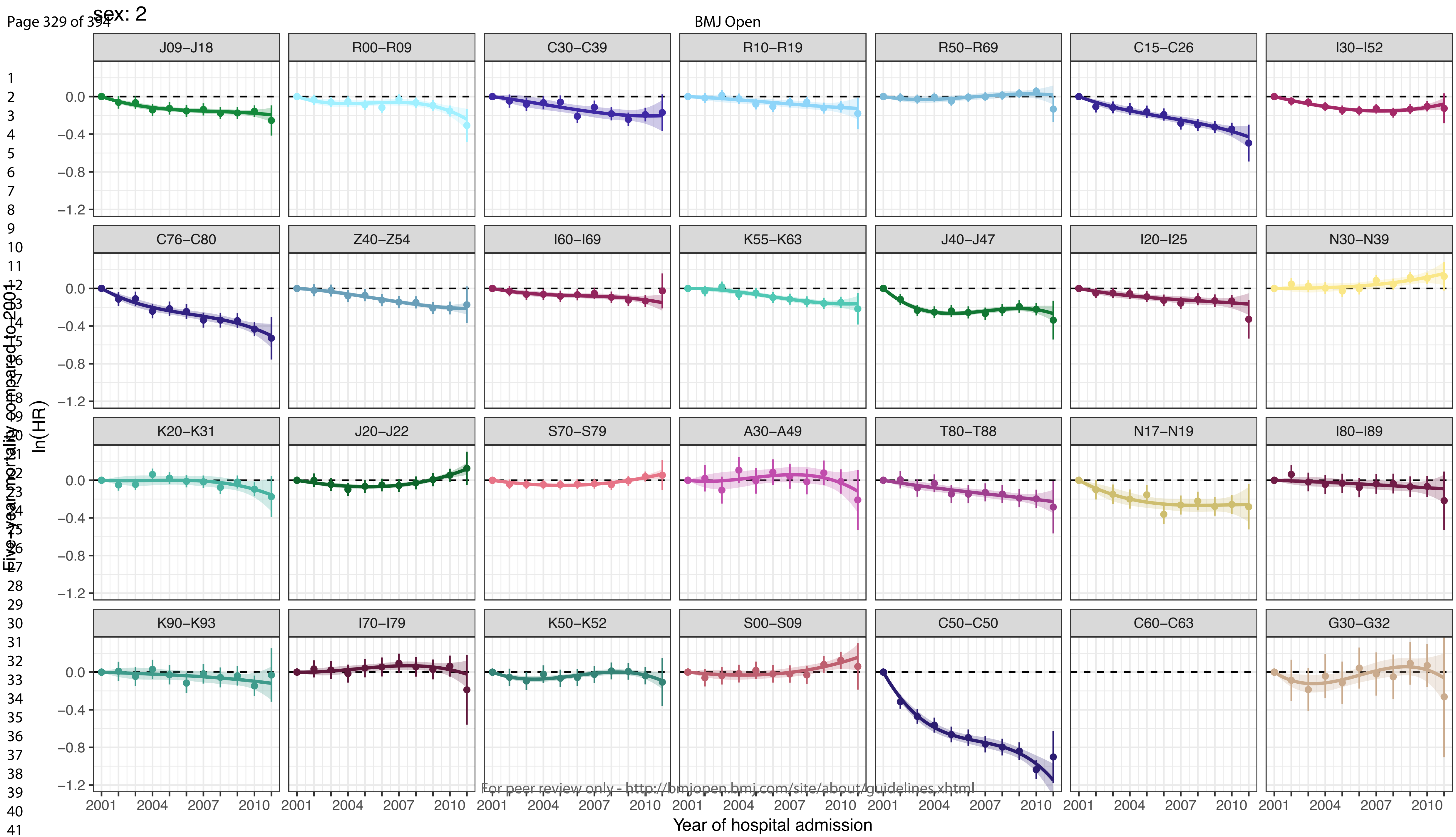

For peer review only - http://bmjopen.bmj.com/site/about/guidelines.xhtml

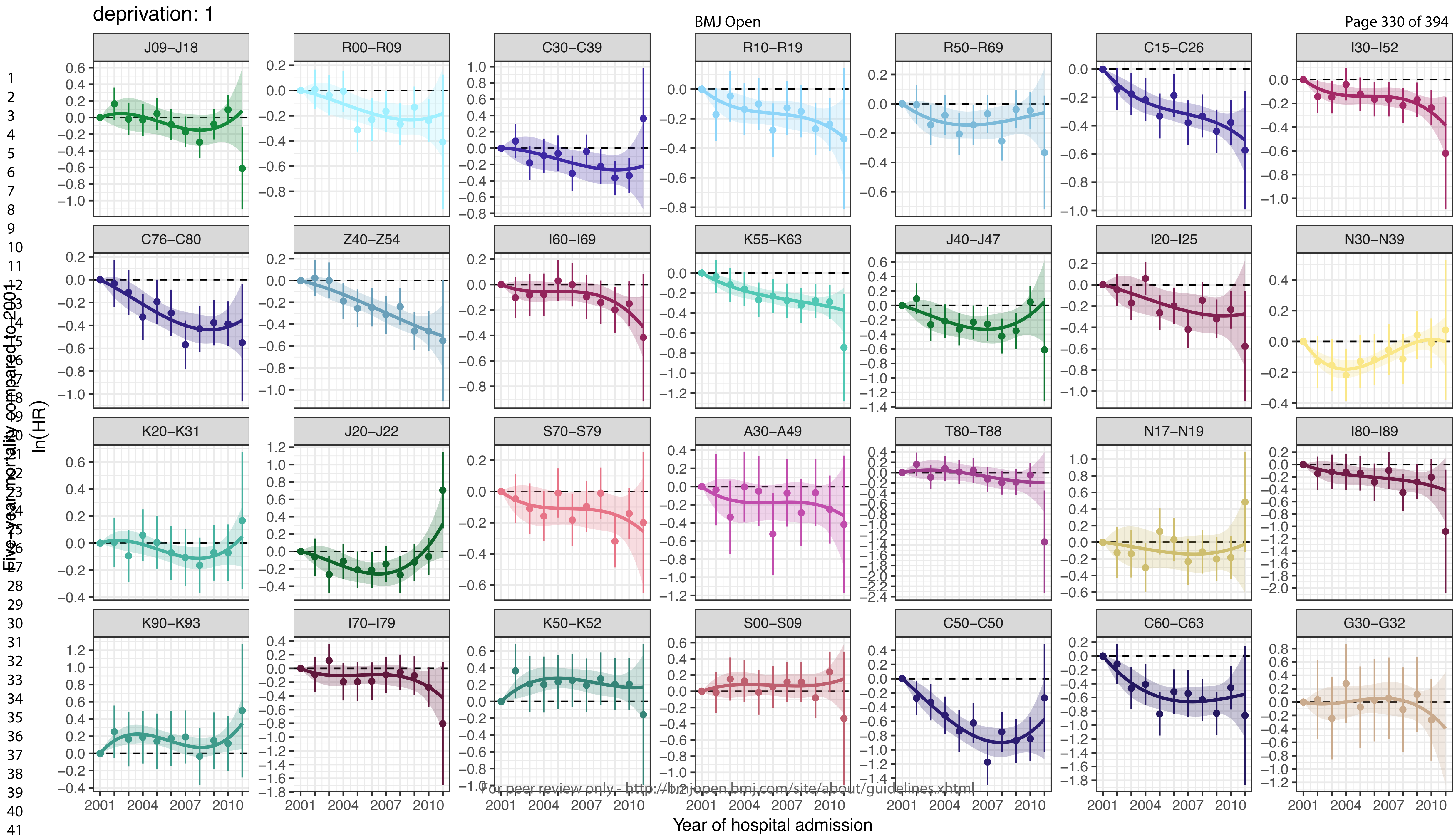
deprivation: 1
J09–J18
R00–R09
C30–C39
R10–R19
R50–R69
C15–C26
I30–I52
C76–C80
Z40–Z54
I60–I69
K55–K63
J40–J47
I20–I25
N30–N39
K20–K31
J20–J22
S70–S79
A30–A49
T80–T88
N17–N19
I80–I89
K90–K93
I70–I79
K50–K52
S00–S09
C50–C50
C60–C63
G30–G32
Five-year mortality compared to 2001
ln(HR)
Year of hospital admission

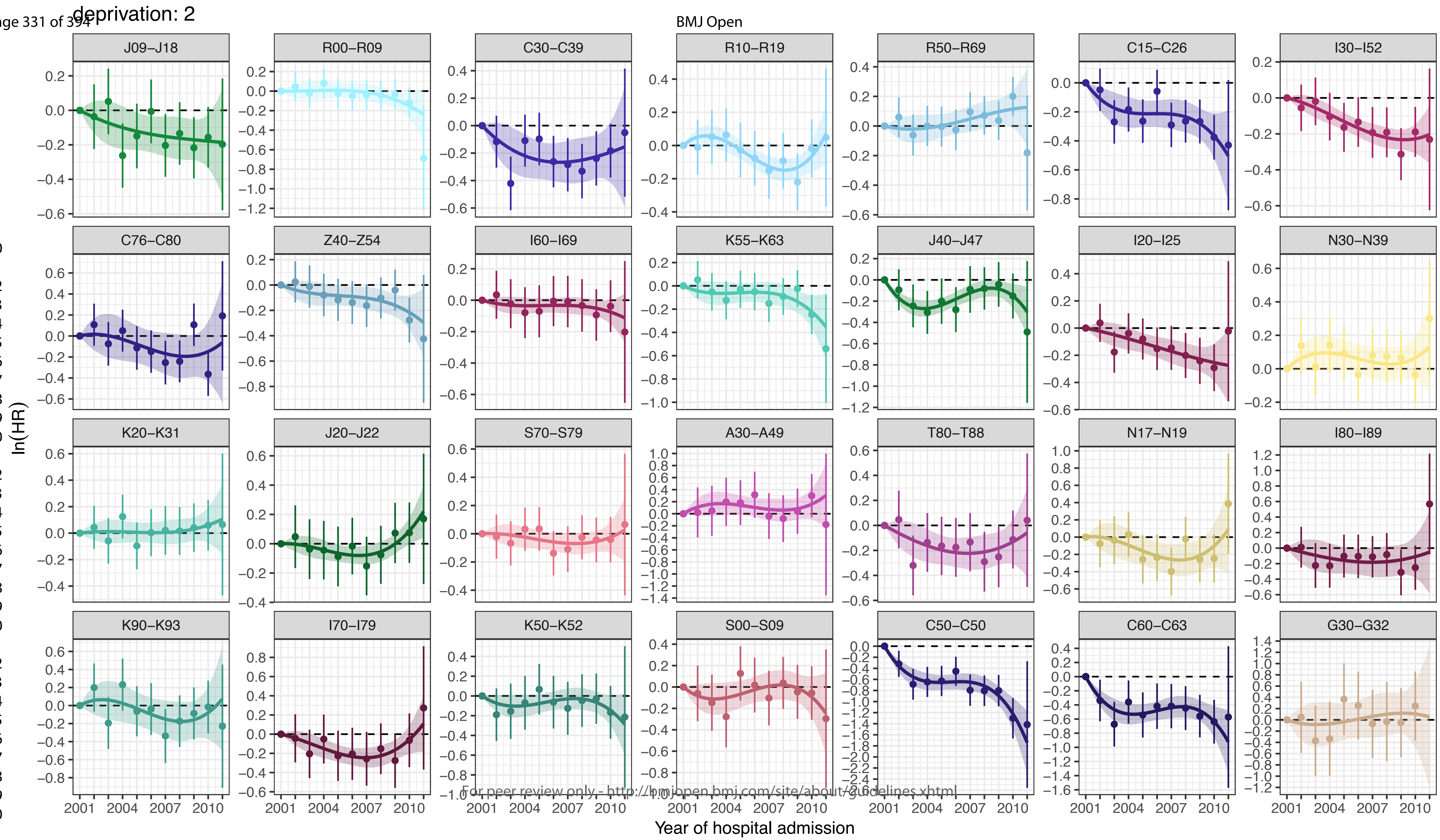

For peer review only - http://bmjopen.bmj.com/site/about/guidelines.xhtml

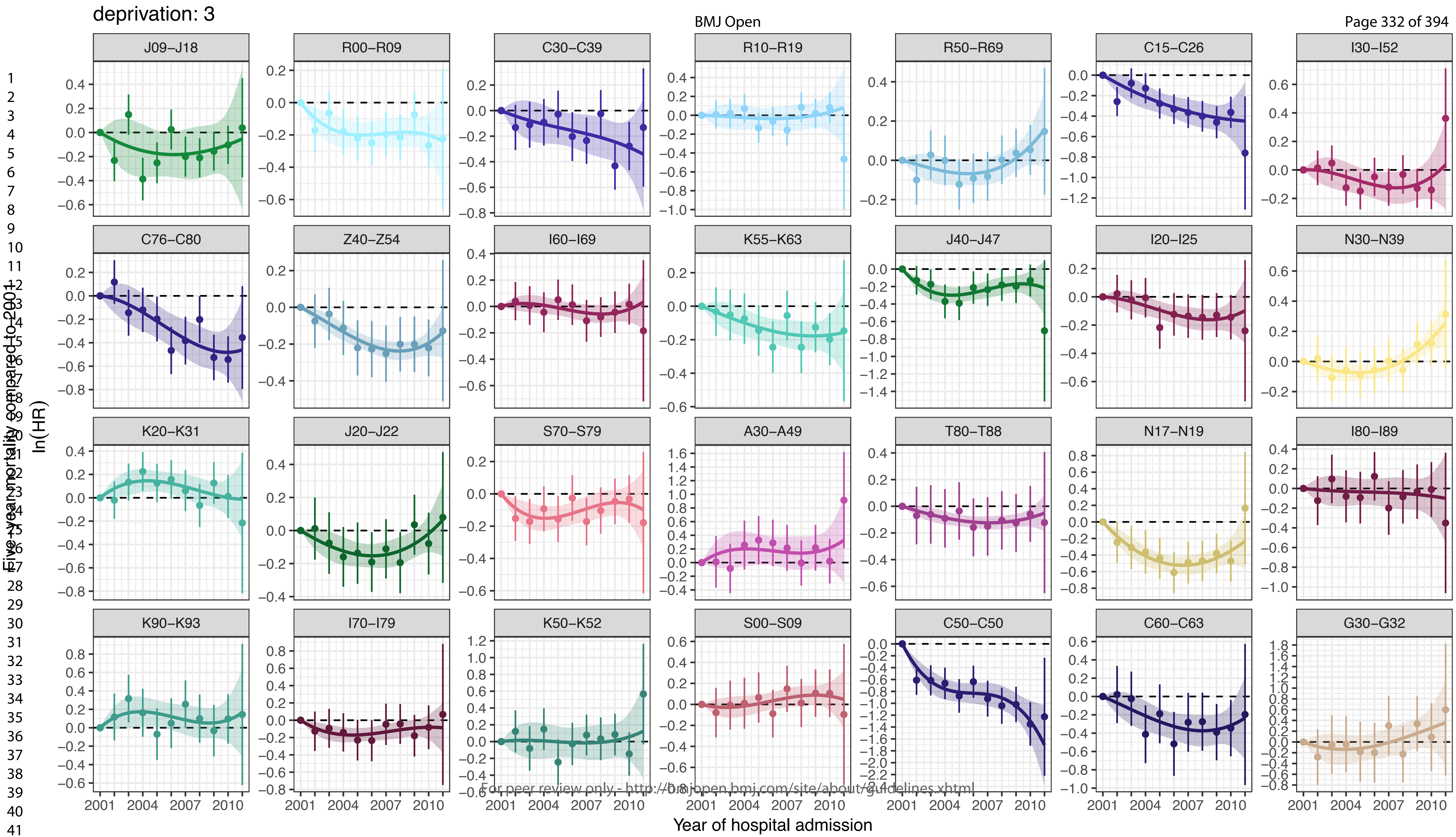
deprivation: 3
J09–J18
R00–R09
C30–C39
R10–R19
R50–R69
C15–C26
I30–I52
C76–C80
Z40–Z54
I60–I69
K55–K63
J40–J47
I20–I25
N30–N39
K20–K31
J20–J22
S70–S79
A30–A49
T80–T88
N17–N19
I80–I89
K90–K93
I70–I79
K50–K52
S00–S09
C50–C50
C60–C63
G30–G32
Five-year mortality compared to 2001
ln(HR)
Year of hospital admission

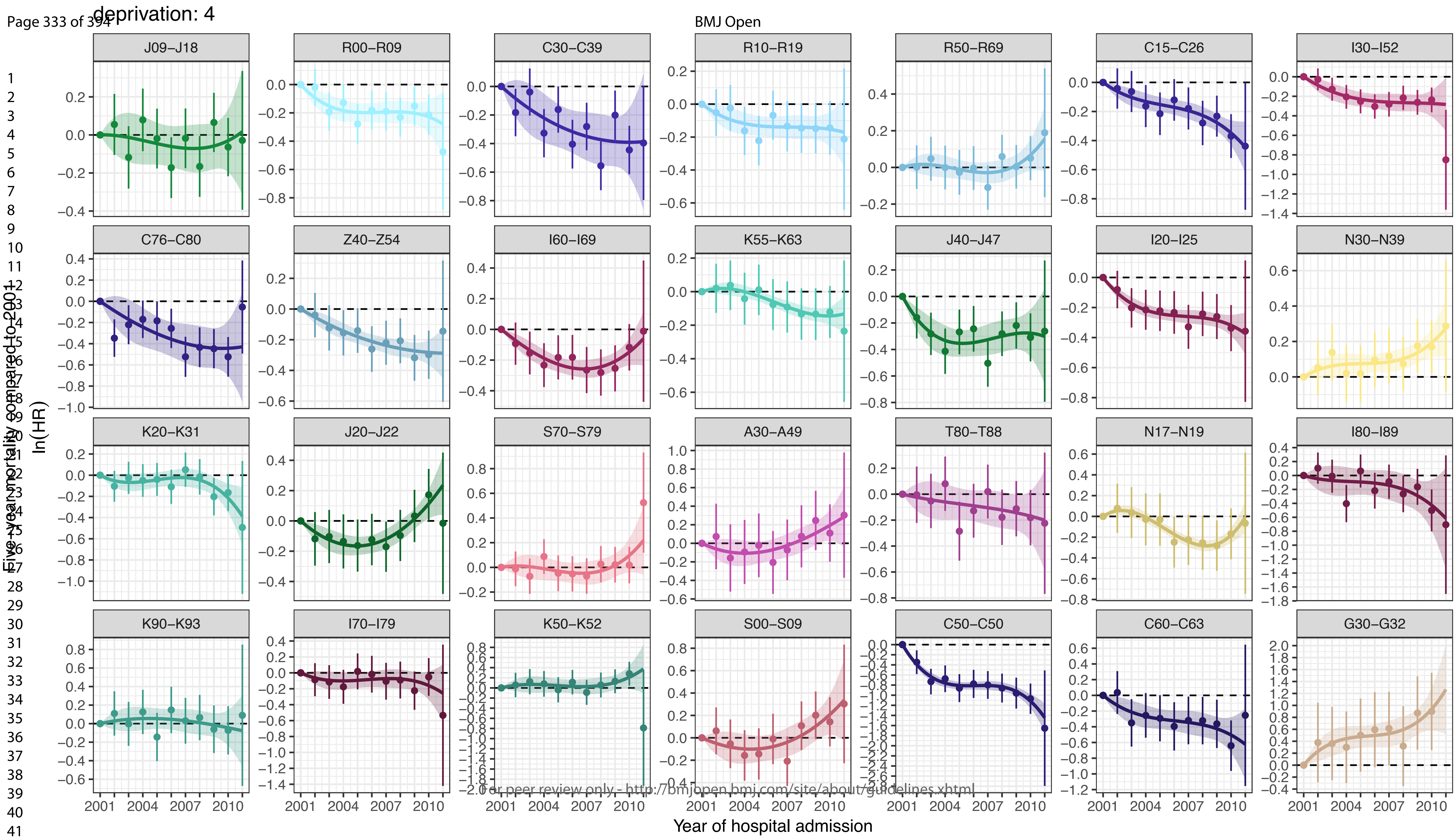
deprivation: 4
J09–J18
R00–R09
C30–C39
R10–R19
R50–R69
C15–C26
I30–I52
C76–C80
Z40–Z54
I60–I69
K55–K63
J40–J47
I20–I25
N30–N39
K20–K31
J20–J22
S70–S79
A30–A49
T80–T88
N17–N19
I80–I89
K90–K93
I70–I79
K50–K52
S00–S09
C50–C50
C60–C63
G30–G32
Five-year mortality compared to 2001
ln(HR)
Year of hospital admission

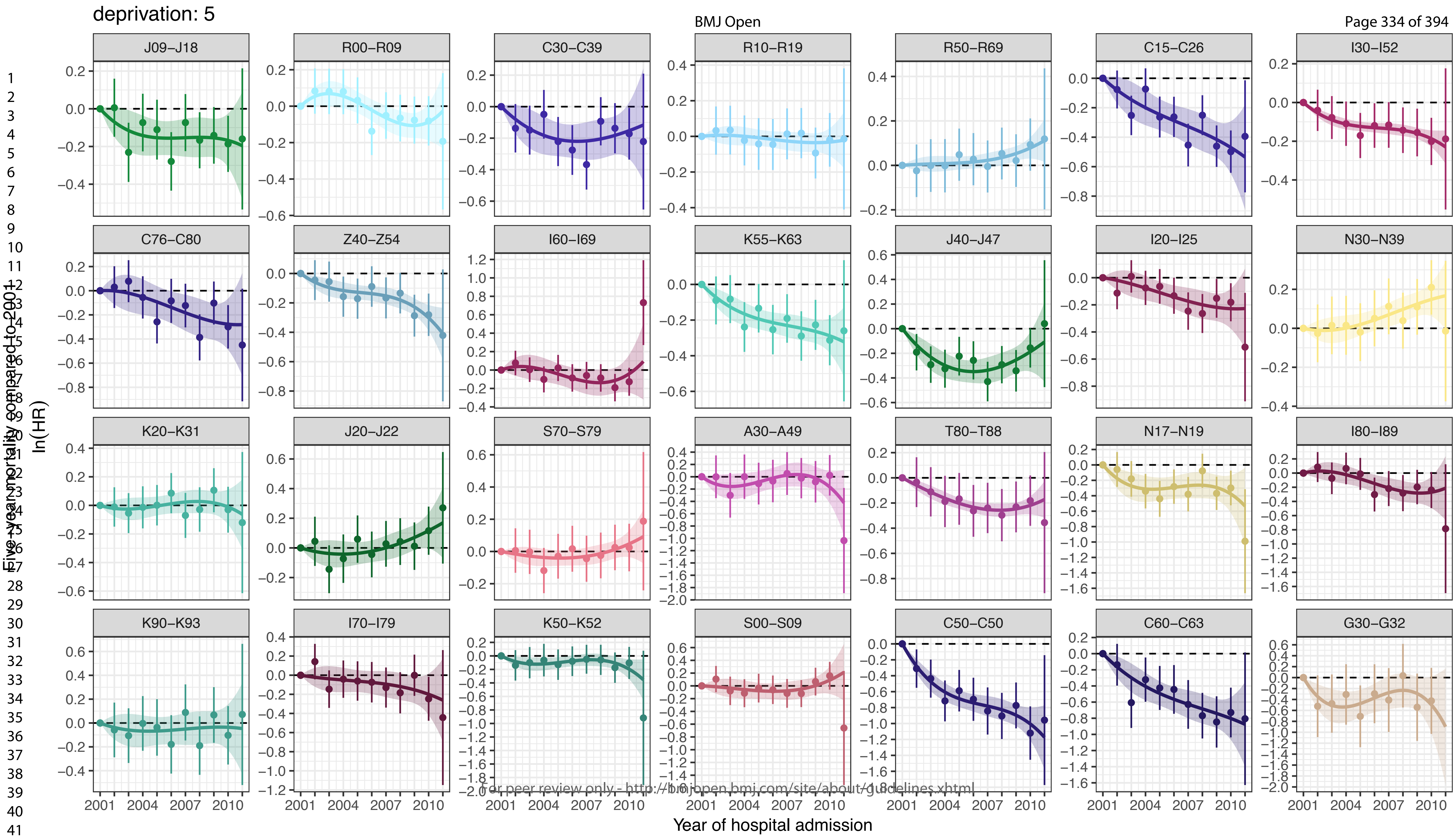
deprivation: 5
J09–J18
R00–R09
C30–C39
R10–R19
R50–R69
C15–C26
I30–I52
C76–C80
Z40–Z54
I60–I69
K55–K63
J40–J47
I20–I25
N30–N39
K20–K31
J20–J22
S70–S79
A30–A49
T80–T88
N17–N19
I80–I89
K90–K93
I70–I79
K50–K52
S00–S09
C50–C50
C60–C63
G30–G32
Five-year mortality compared to 2001
ln(HR)
Year of hospital admission
2001
2004
2007
2010

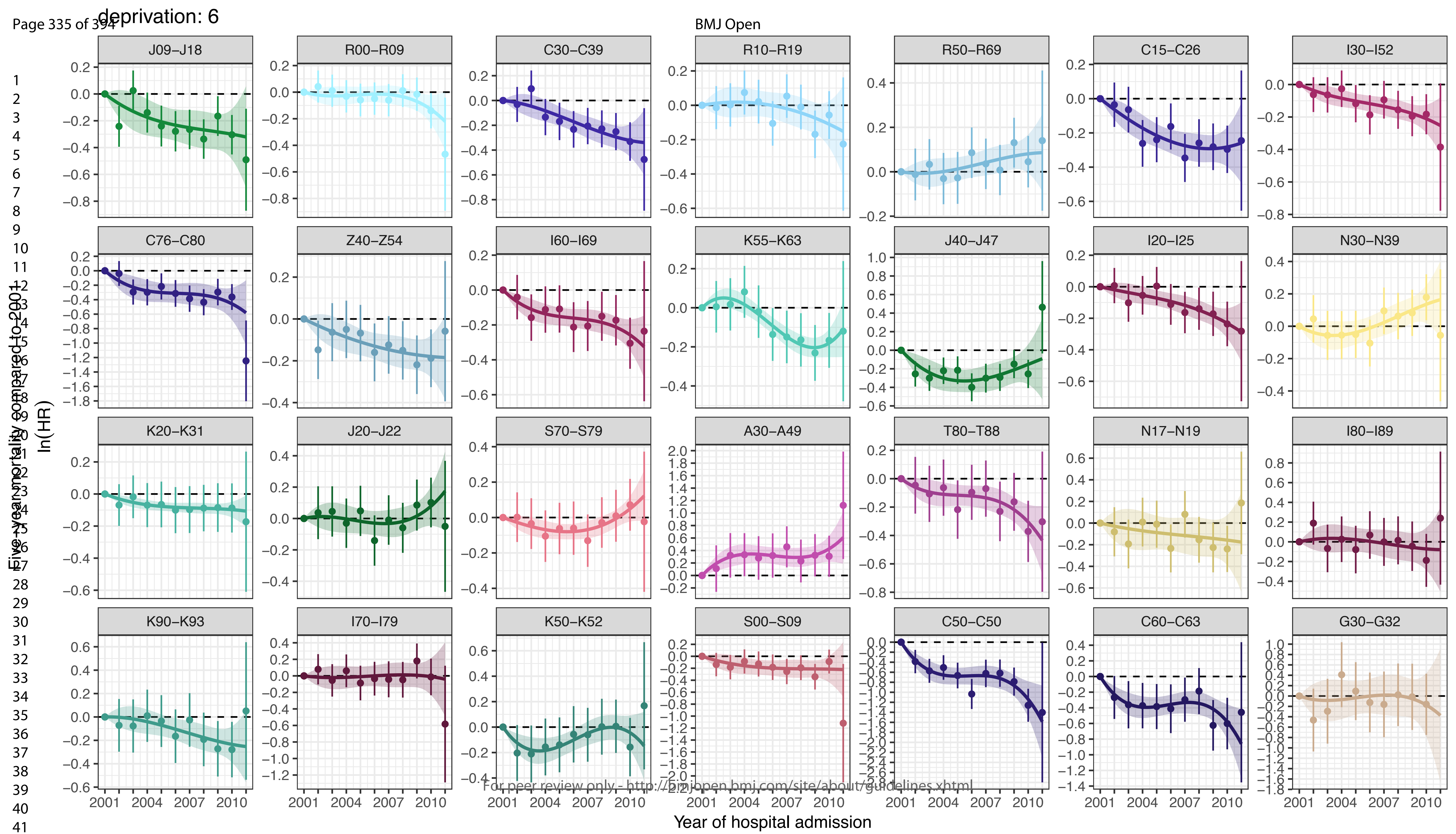
deprivation: 6
J09–J18
R00–R09
C30–C39
R10–R19
R50–R69
C15–C26
I30–I52
C76–C80
Z40–Z54
I60–I69
K55–K63
J40–J47
I20–I25
N30–N39
K20–K31
J20–J22
S70–S79
A30–A49
T80–T88
N17–N19
I80–I89
K90–K93
I70–I79
K50–K52
S00–S09
C50–C50
C60–C63
G30–G32
Five-year mortality compared to 2001
ln(HR)
Year of hospital admission
2001
2004
2007
2010

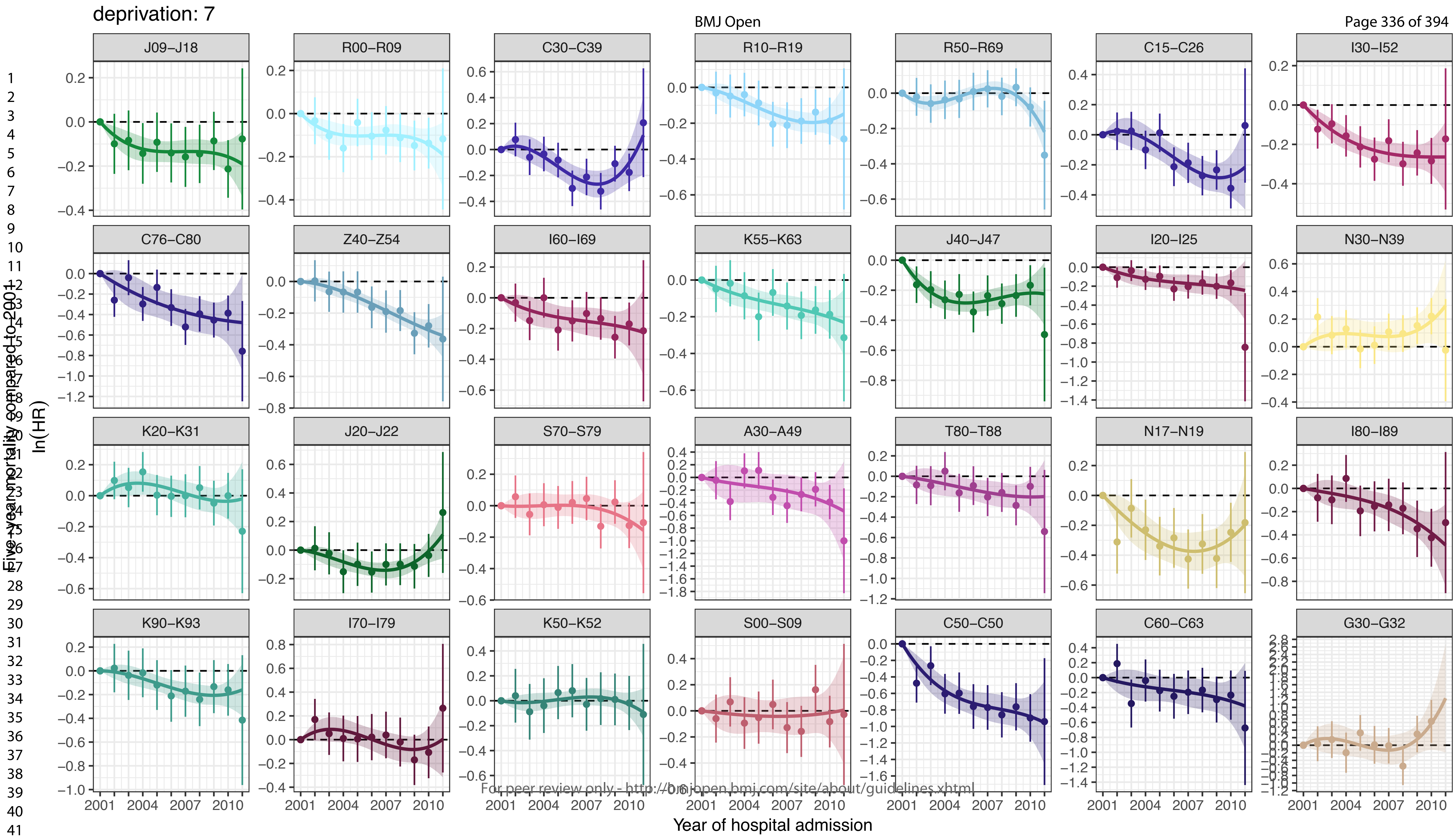
deprivation: 7
J09–J18
R00–R09
C30–C39
R10–R19
R50–R69
C15–C26
I30–I52
C76–C80
Z40–Z54
I60–I69
K55–K63
J40–J47
I20–I25
N30–N39
K20–K31
J20–J22
S70–S79
A30–A49
T80–T88
N17–N19
I80–I89
K90–K93
I70–I79
K50–K52
S00–S09
C50–C50
C60–C63
G30–G32
Five-year mortality compared to 2001
ln(HR)
Year of hospital admission

deprivation: 8

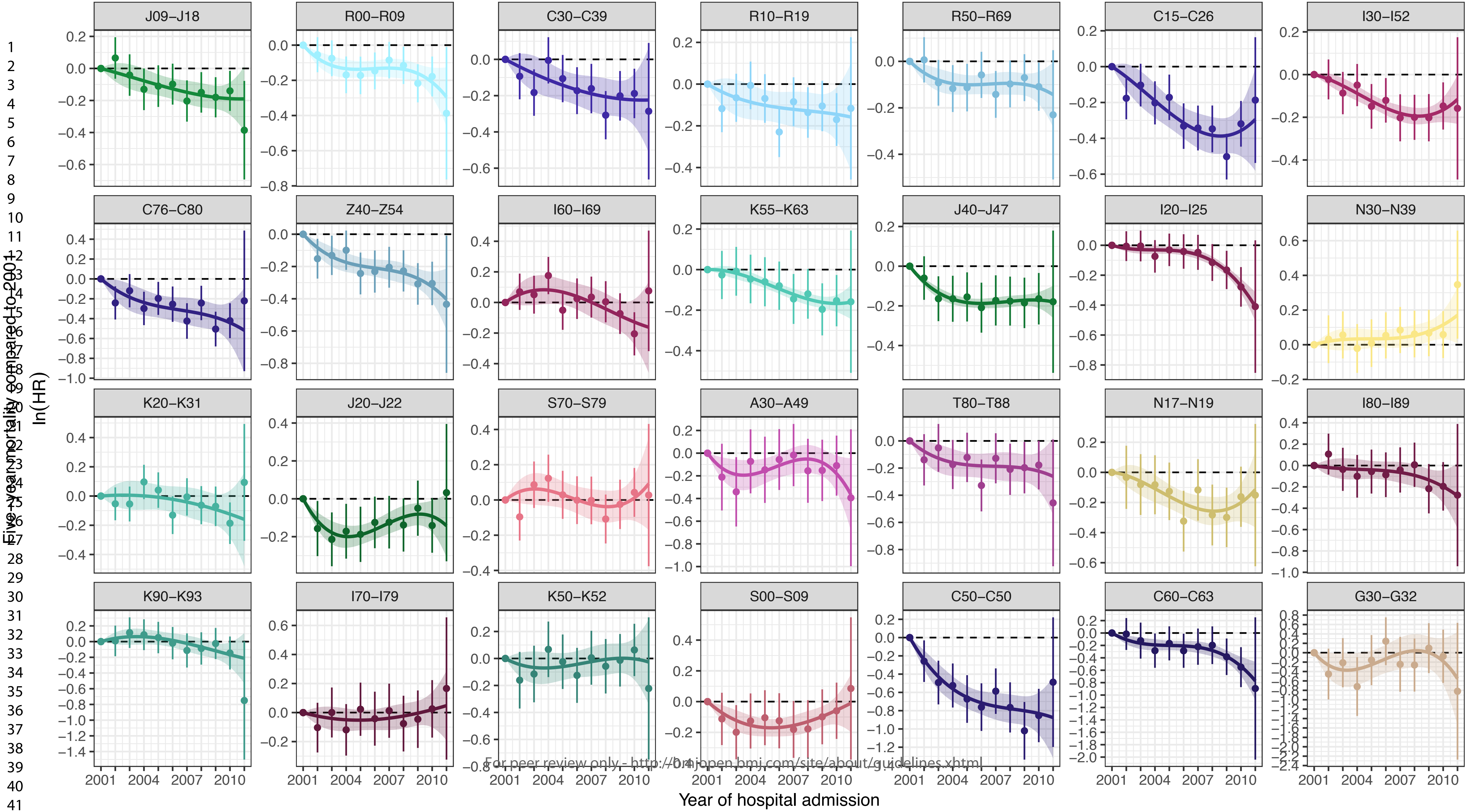

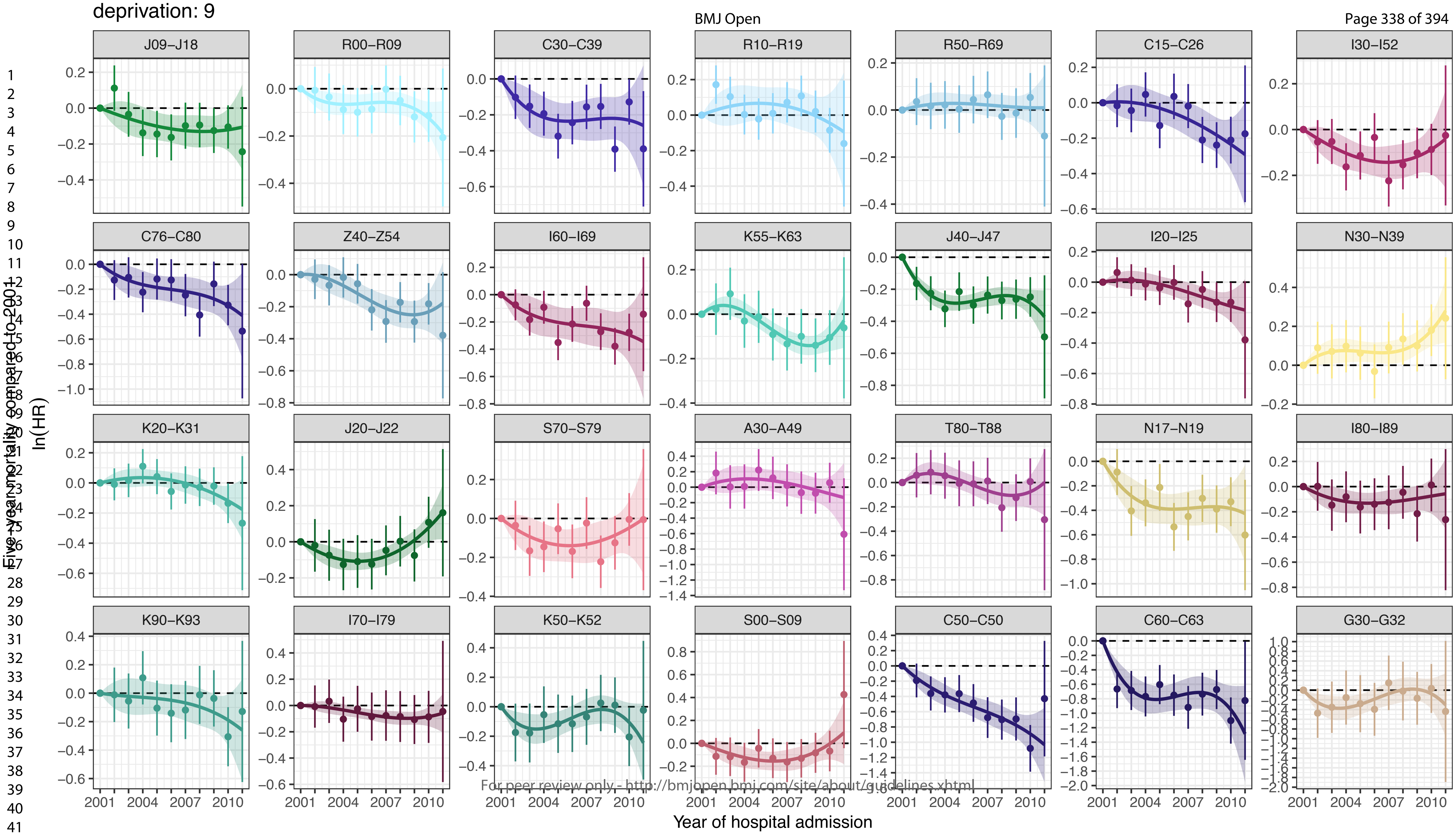
deprivation: 9
J09–J18
R00–R09
C30–C39
R10–R19
R50–R69
C15–C26
I30–I52
C76–C80
Z40–Z54
I60–I69
K55–K63
J40–J47
I20–I25
N30–N39
K20–K31
J20–J22
S70–S79
A30–A49
T80–T88
N17–N19
I80–I89
K90–K93
I70–I79
K50–K52
S00–S09
C50–C50
C60–C63
G30–G32
Five-year mortality compared to 2001:
ln(HR)
Year of hospital admission

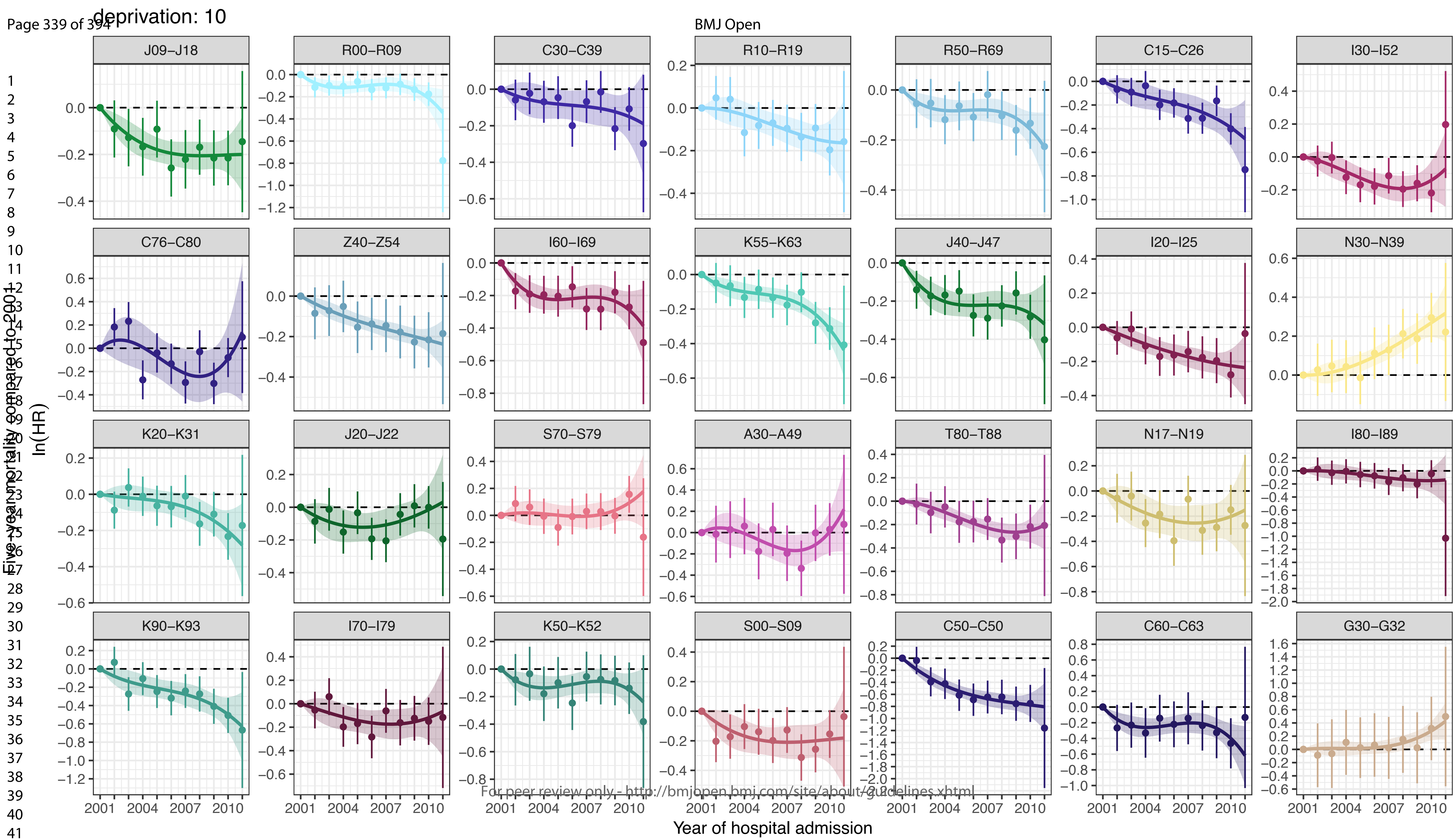

For peer review only - http://bmjopen.bmj.com/site/about/guidelines.xhtml

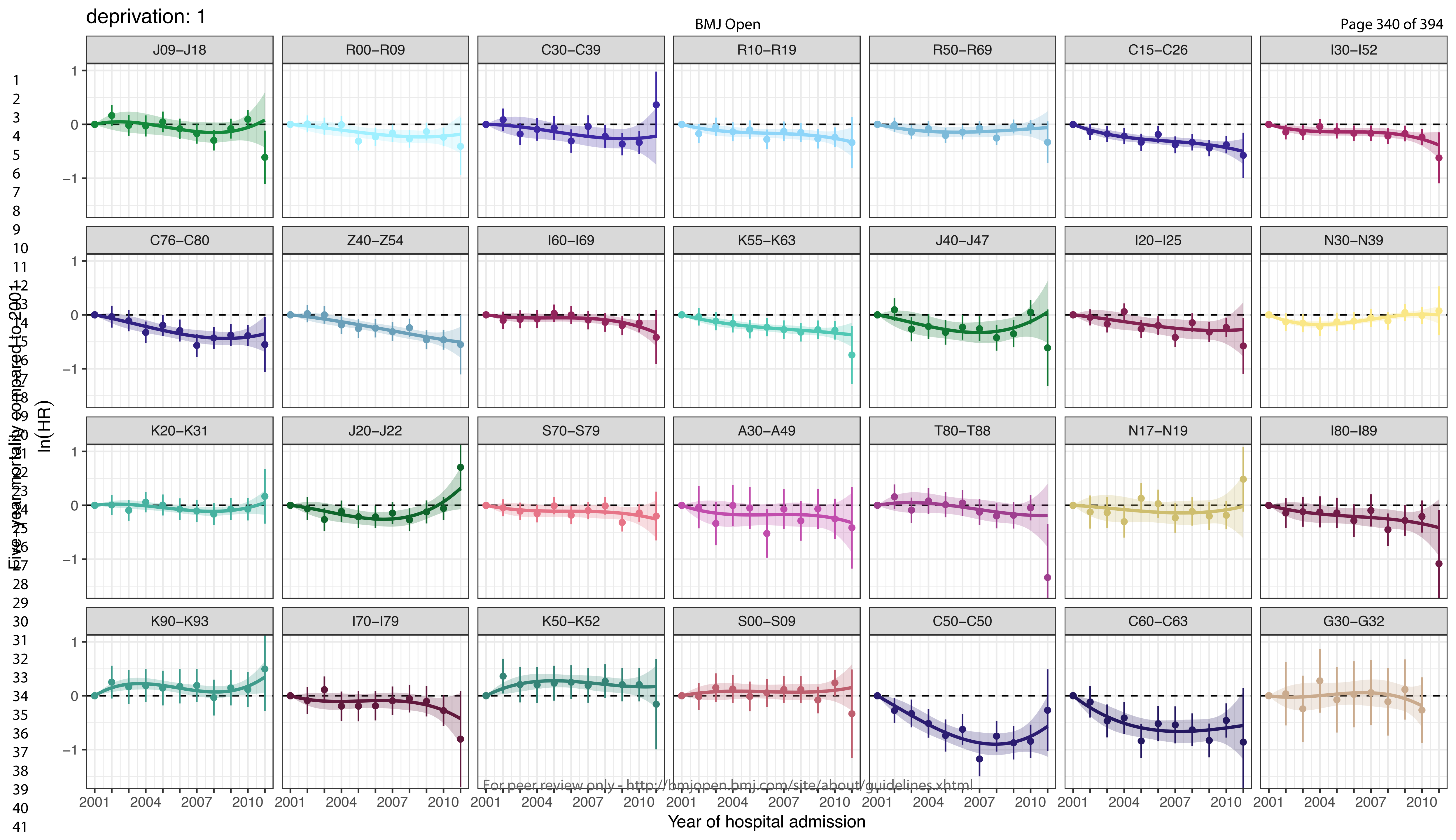

For peer review only - http://bmjopen.bmj.com/site/about/guidelines.xhtml

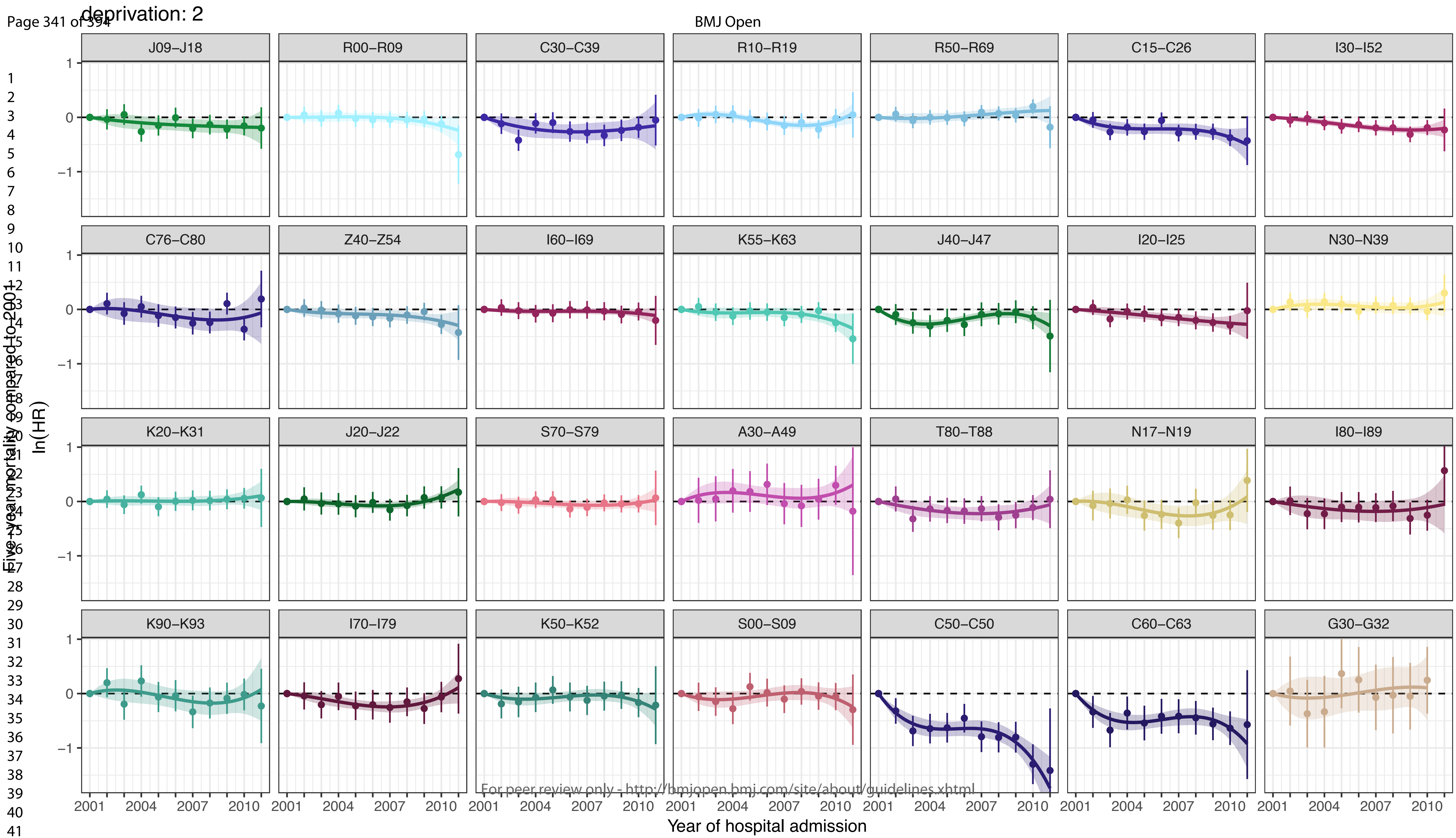
deprivation: 2
J09–J18
R00–R09
C30–C39
R10–R19
R50–R69
C15–C26
I30–I52
C76–C80
Z40–Z54
I60–I69
K55–K63
J40–J47
I20–I25
N30–N39
K20–K31
J20–J22
S70–S79
A30–A49
T80–T88
N17–N19
I80–I89
K90–K93
I70–I79
K50–K52
S00–S09
C50–C50
C60–C63
G30–G32
1
0
−1
2001
2004
2007
2010
Five-year mortality compared to 2001:
ln(HR)
Year of hospital admission

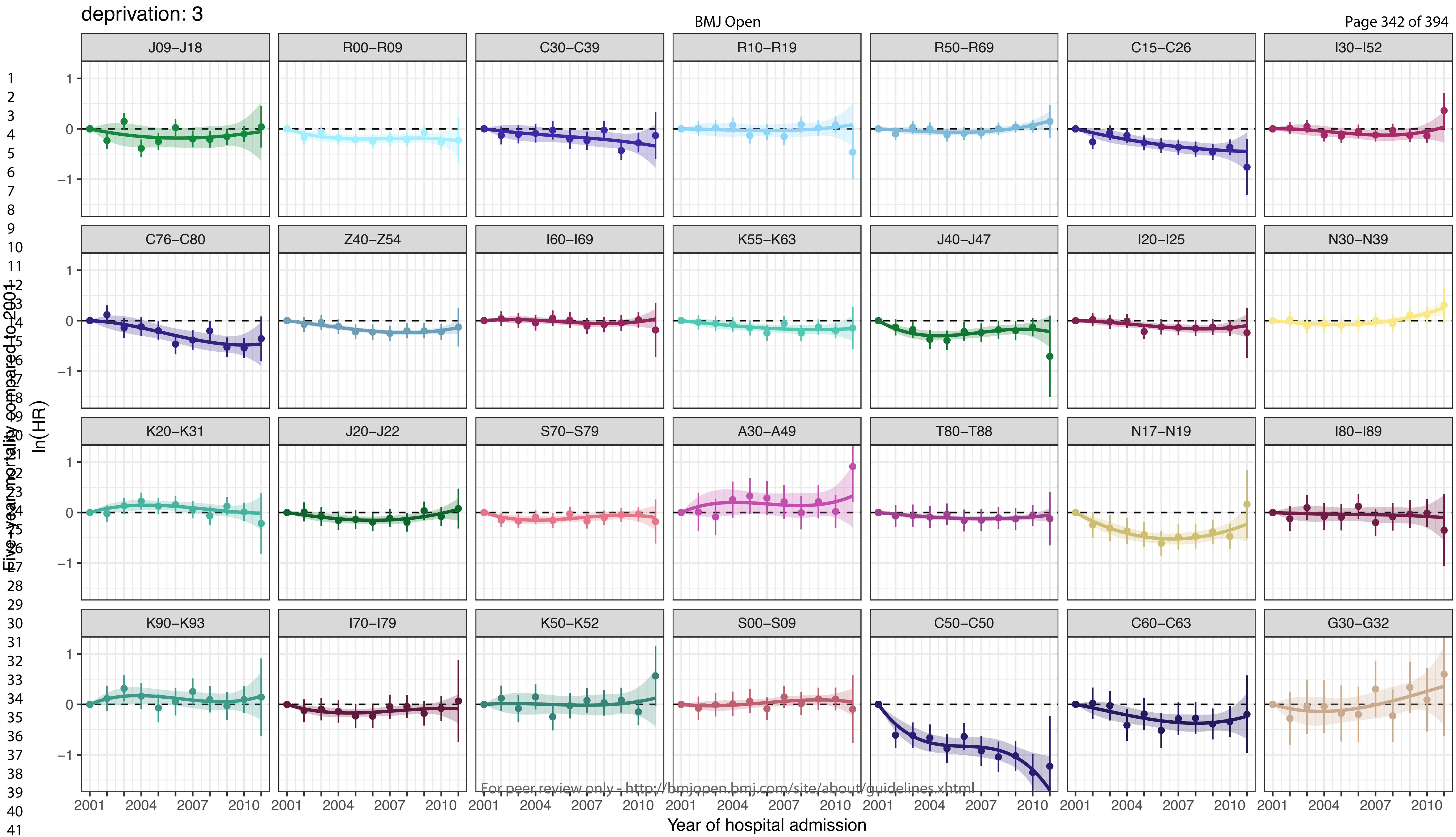

For peer review only - http://bmjopen.bmj.com/site/about/guidelines.xhtml

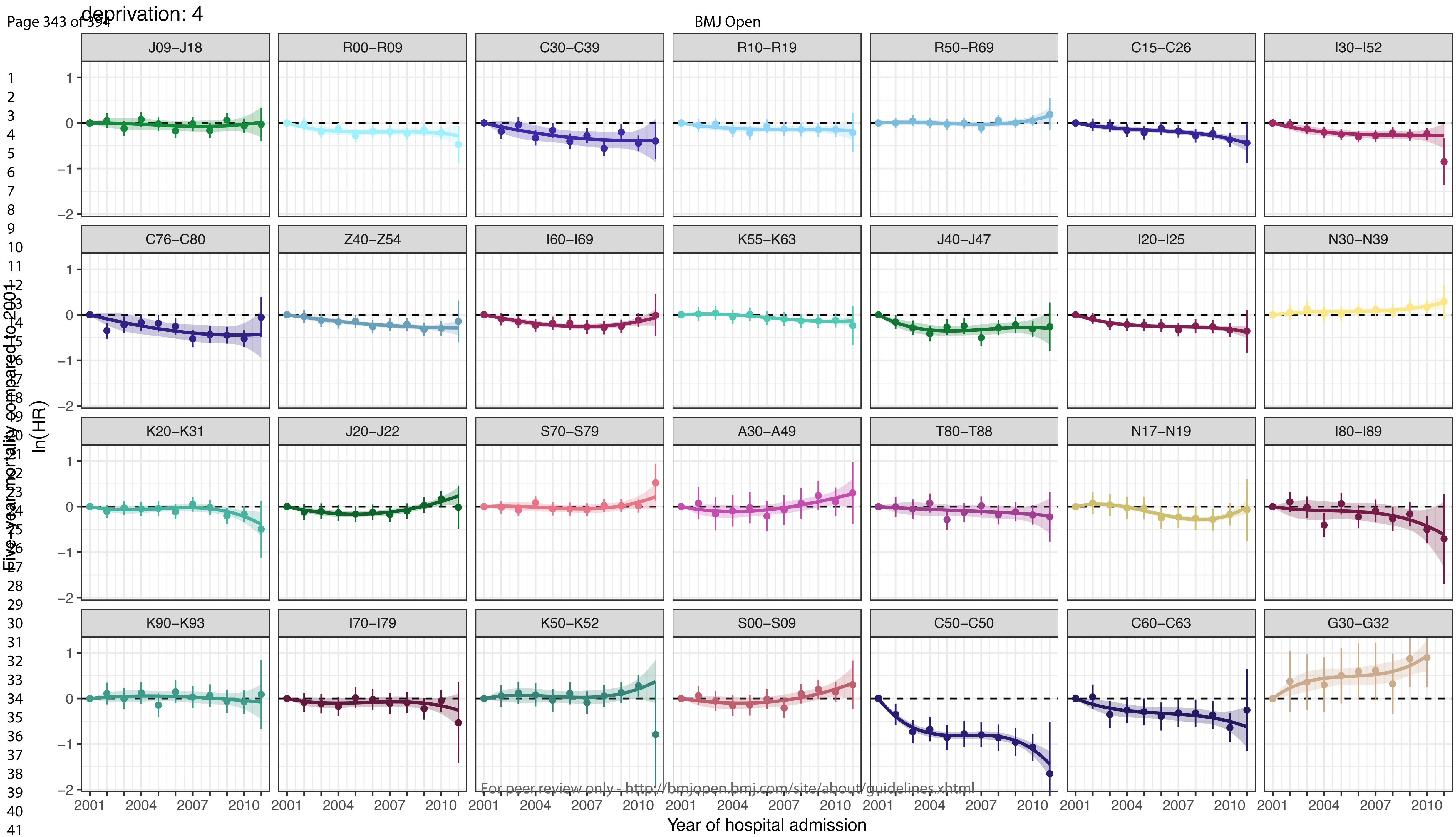

For peer review only - http://bmjopen.bmj.com/site/about/guidelines.xhtml

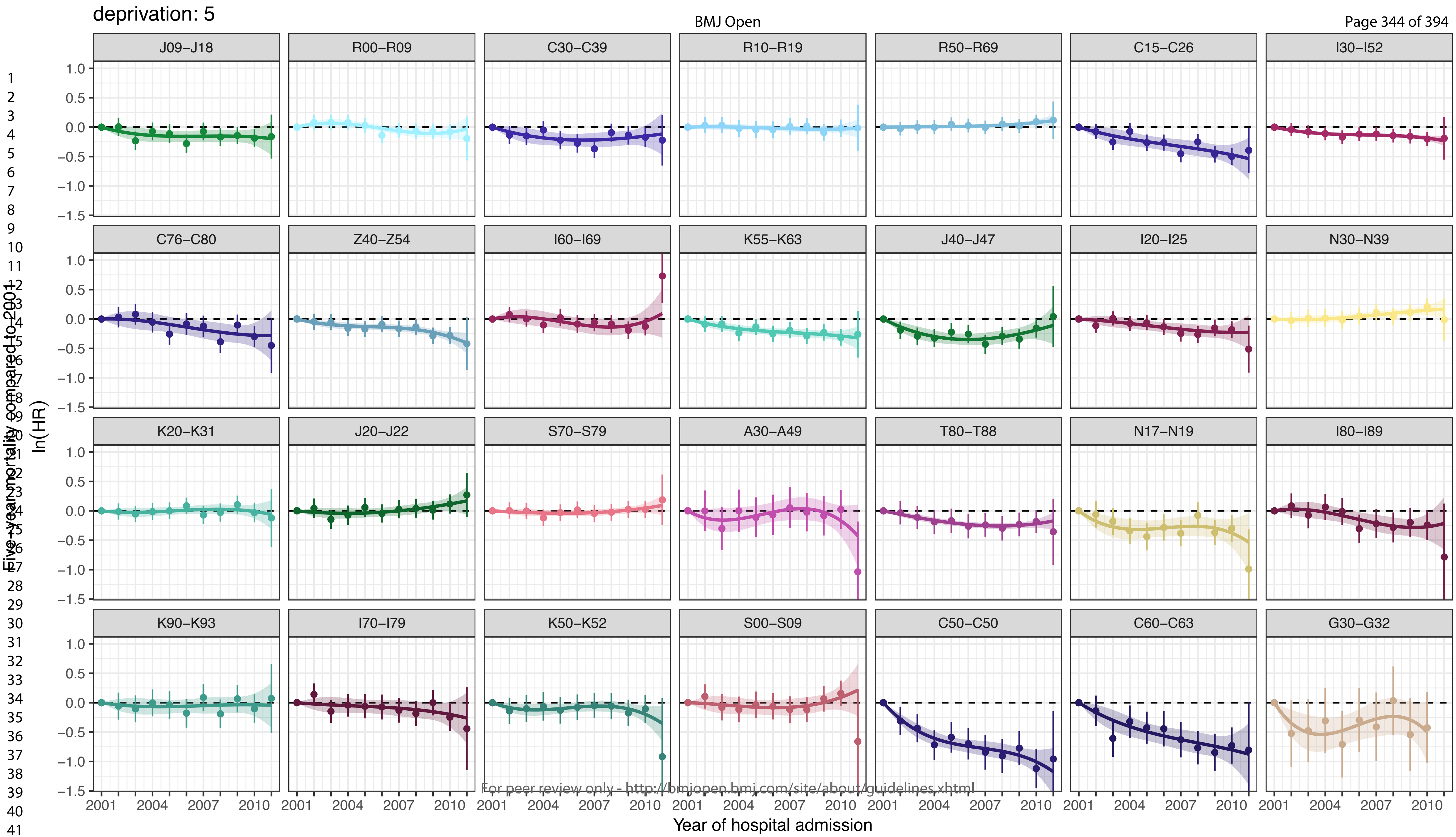
deprivation: 5
J09–J18
R00–R09
C30–C39
R10–R19
R50–R69
C15–C26
I30–I52
C76–C80
Z40–Z54
I60–I69
K55–K63
J40–J47
I20–I25
N30–N39
K20–K31
J20–J22
S70–S79
A30–A49
T80–T88
N17–N19
I80–I89
K90–K93
I70–I79
K50–K52
S00–S09
C50–C50
C60–C63
G30–G32
1.0
0.5
0.0
−0.5
−1.0
−1.5
2001
2004
2007
2010
Five-year mortality compared to 2001
ln(HR)
Year of hospital admission

deprivation: 6

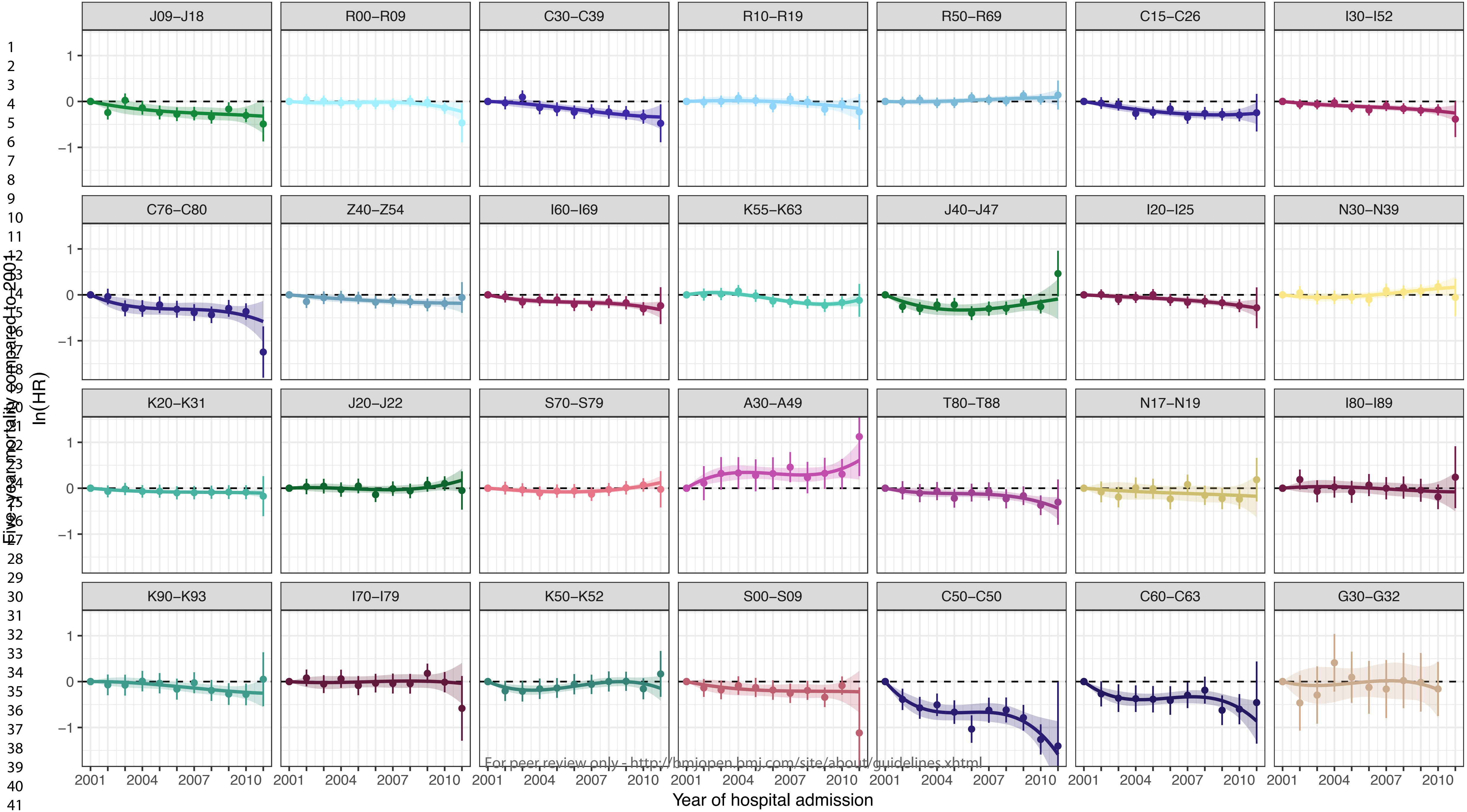

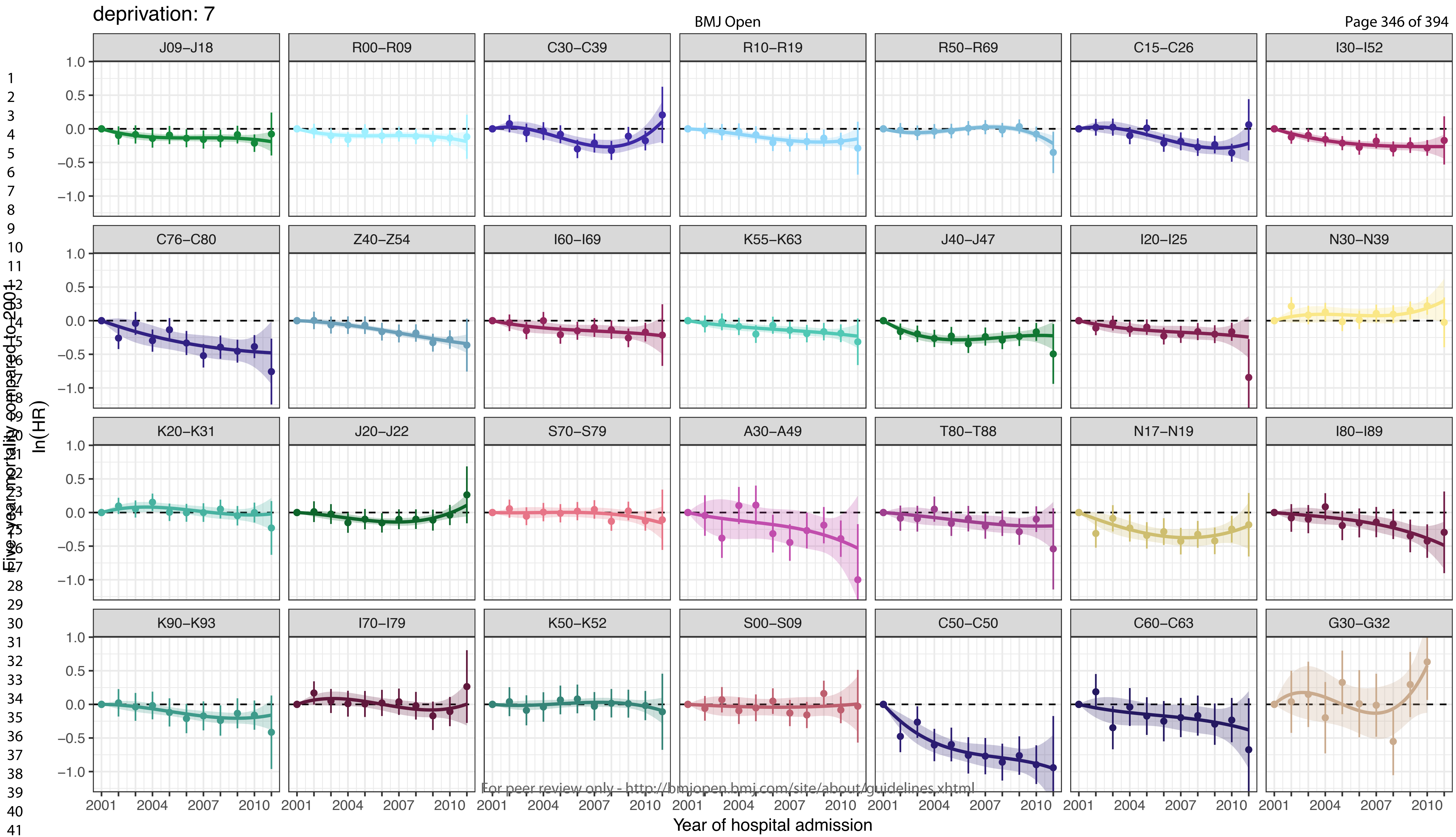
deprivation: 7
J09–J18
R00–R09
C30–C39
R10–R19
R50–R69
C15–C26
I30–I52
C76–C80
Z40–Z54
I60–I69
K55–K63
J40–J47
I20–I25
N30–N39
K20–K31
J20–J22
S70–S79
A30–A49
T80–T88
N17–N19
I80–I89
K90–K93
I70–I79
K50–K52
S00–S09
C50–C50
C60–C63
G30–G32
1.0
0.5
0.0
−0.5
−1.0
2001
2004
2007
2010
Five-year mortality compared to 2001
ln(HR)
Year of hospital admission

deprivation: 8

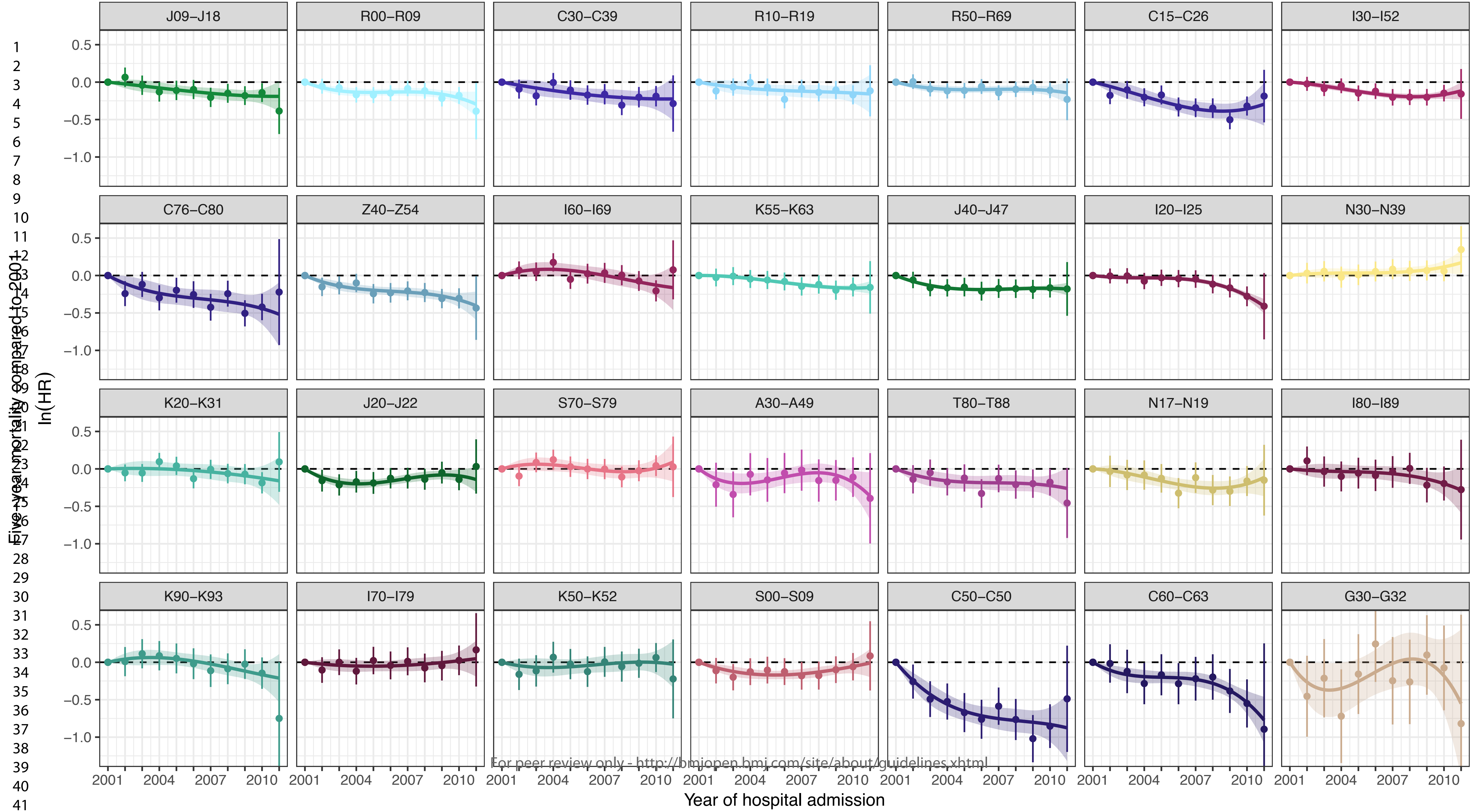

For peer review only - http://bmjopen.bmj.com/site/about/guidelines.xhtml

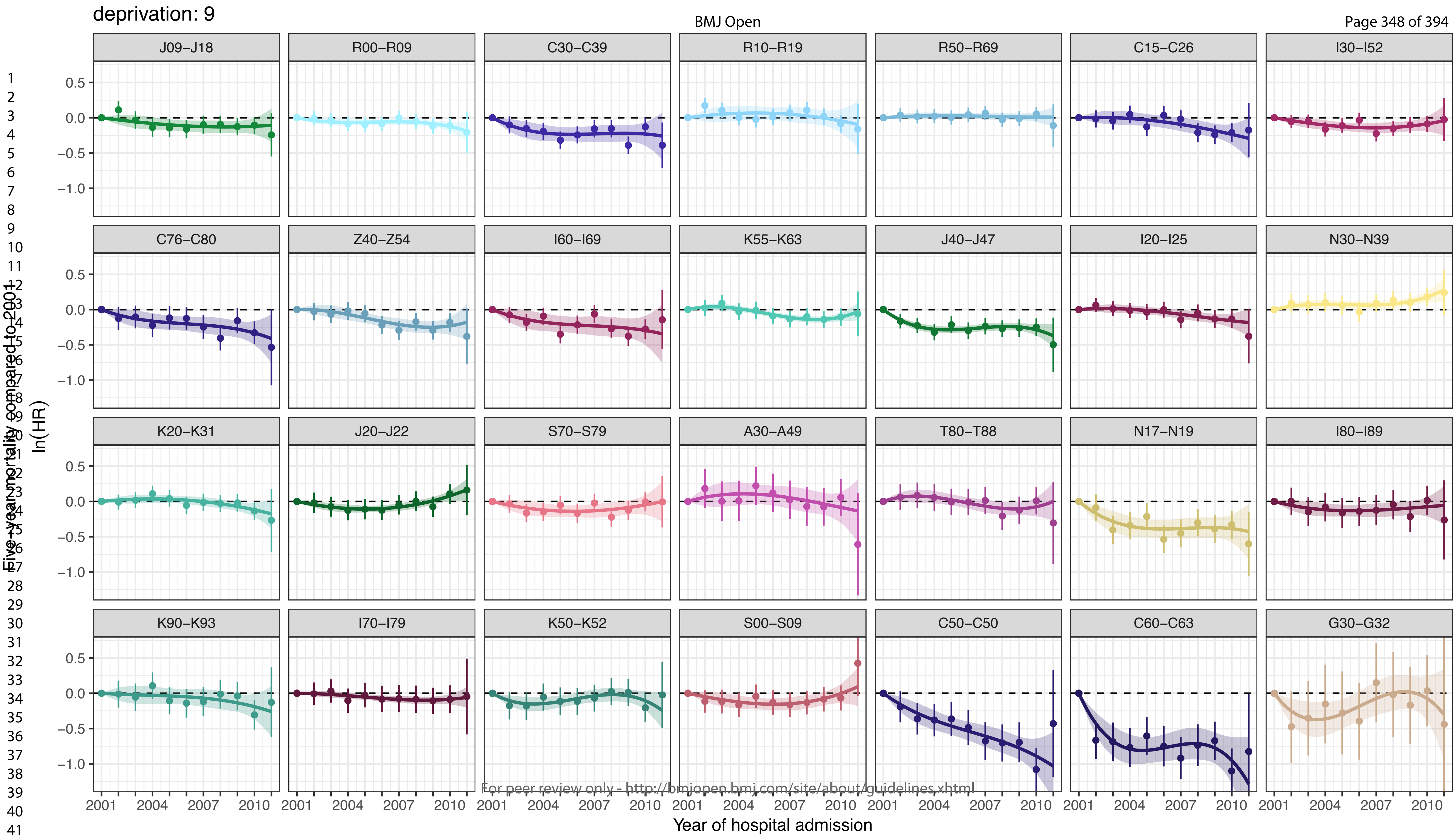

For peer review only - http://bmjopen.bmj.com/site/about/guidelines.xhtml

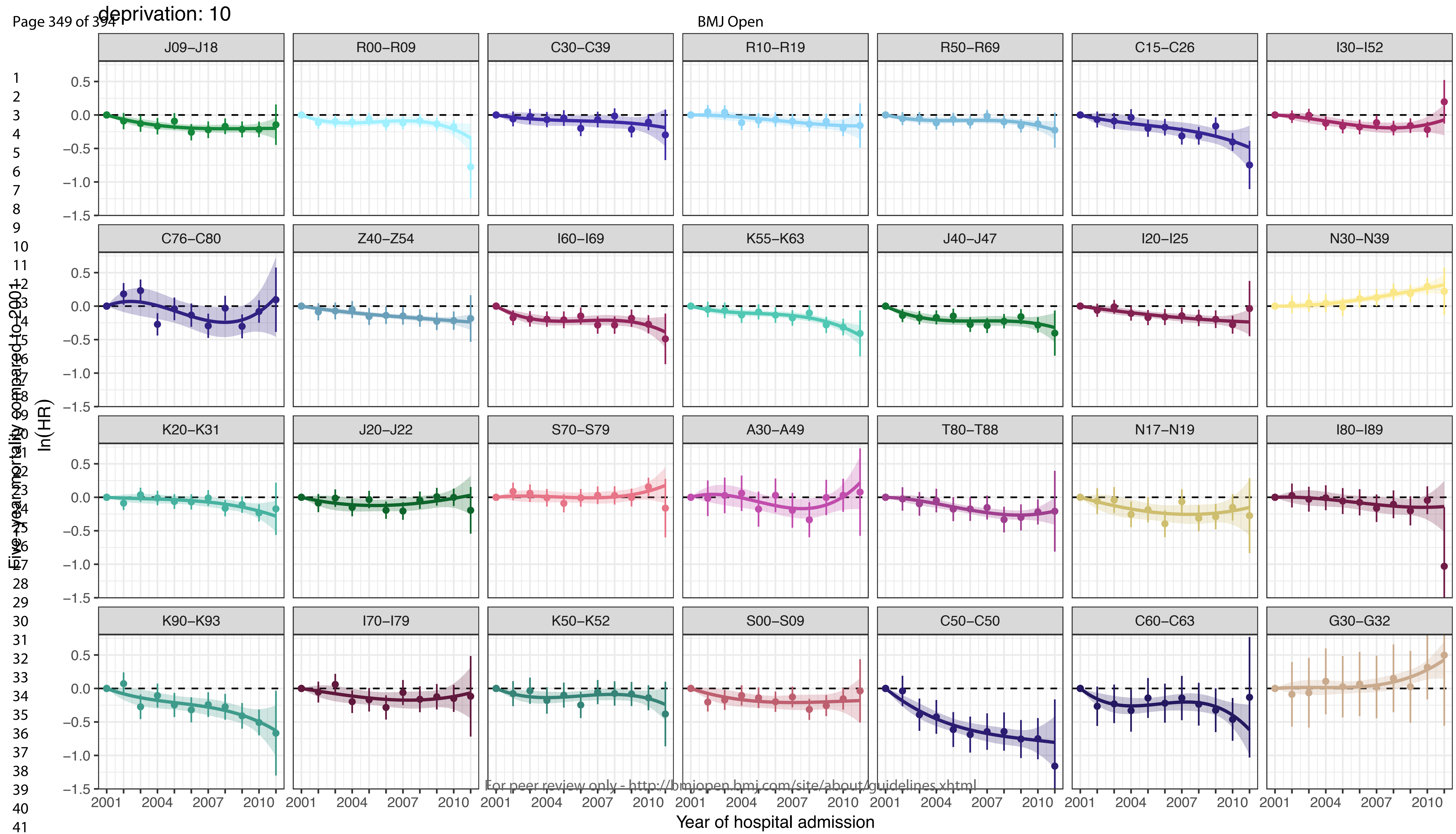

For peer review only - http://bmjopen.bmj.com/site/about/guidelines.xhtml

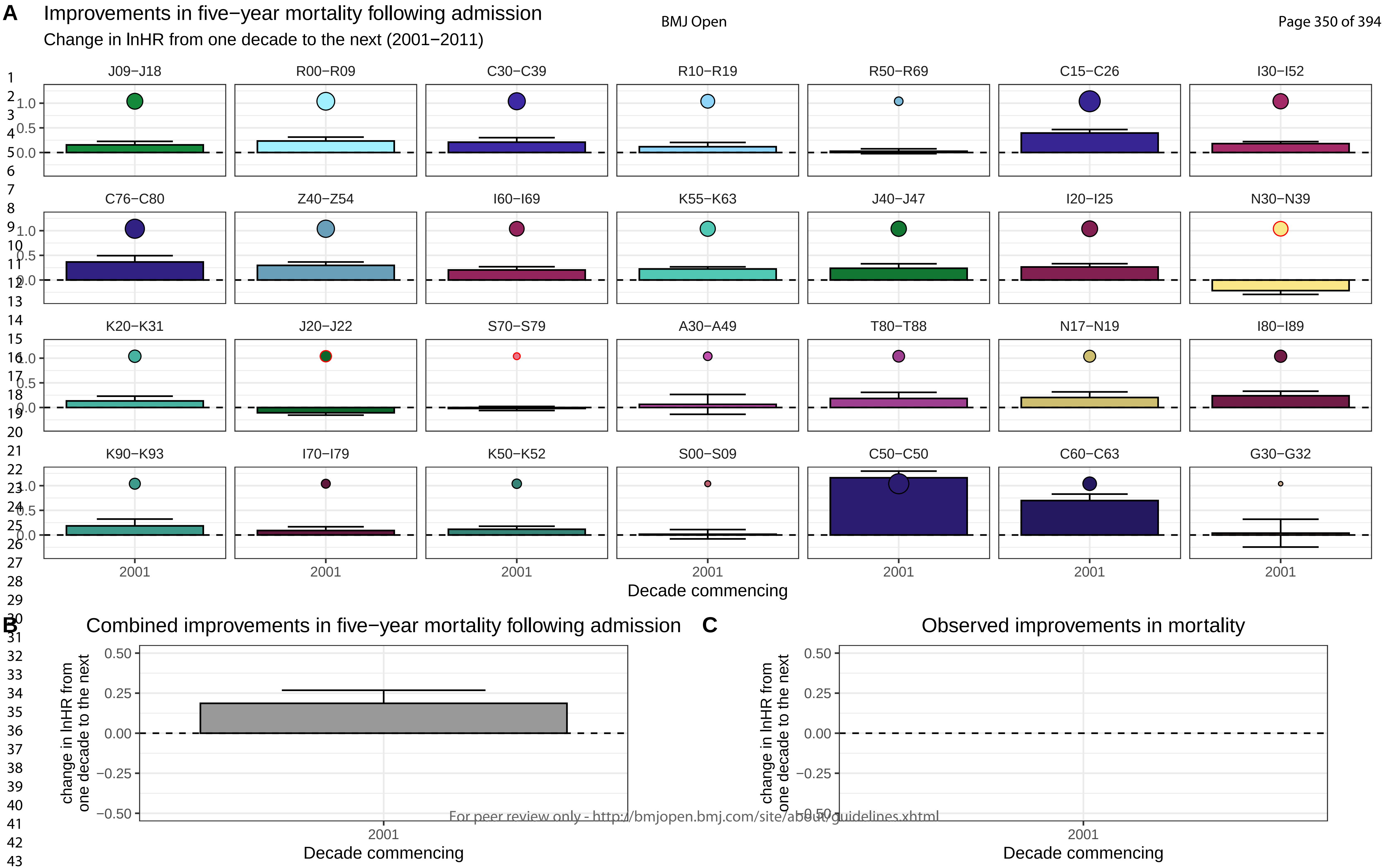
A
Improvements in five−year mortality following admission
Change in lnHR from one decade to the next (2001−2011)
J09−J18
R00−R09
C30−C39
R10−R19
R50−R69
C15−C26
I30−I52
C76−C80
Z40−Z54
I60−I69
K55−K63
J40−J47
I20−I25
N30−N39
K20−K31
J20−J22
S70−S79
A30−A49
T80−T88
N17−N19
I80−I89
K90−K93
I70−I79
K50−K52
S00−S09
C50−C50
C60−C63
G30−G32
1.0
0.5
0.0
2001
Decade commencing
B
Combined improvements in five−year mortality following admission
change in lnHR from one decade to the next
0.50
0.25
0.00
−0.25
−0.50
2001
Decade commencing
C
Observed improvements in mortality
change in lnHR from one decade to the next
0.50
0.25
0.00
−0.25
−0.50
2001
Decade commencing

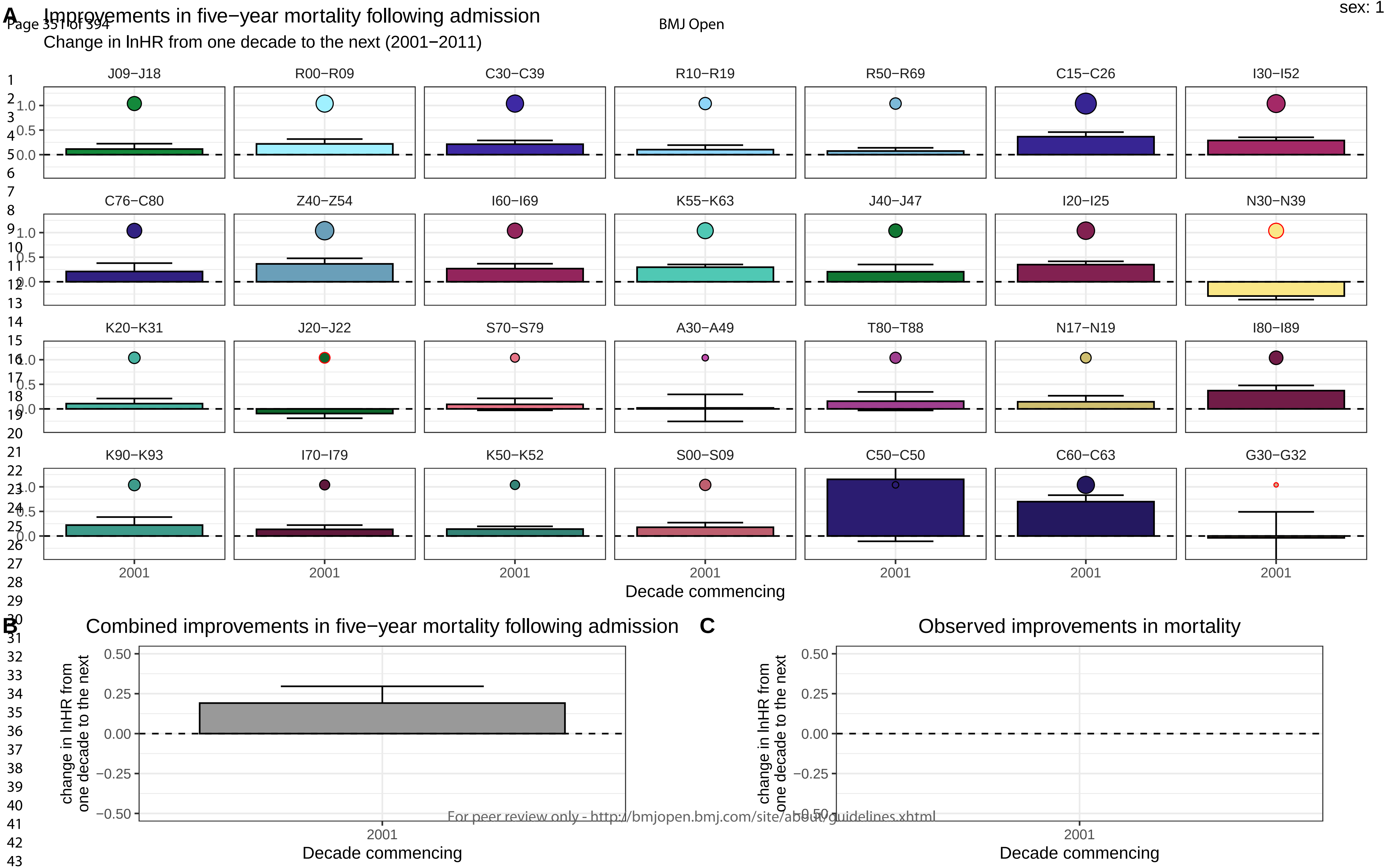
A
Improvements in five−year mortality following admission
Change in lnHR from one decade to the next (2001−2011)
sex: 1
J09−J18
R00−R09
C30−C39
R10−R19
R50−R69
C15−C26
I30−I52
C76−C80
Z40−Z54
I60−I69
K55−K63
J40−J47
I20−I25
N30−N39
K20−K31
J20−J22
S70−S79
A30−A49
T80−T88
N17−N19
I80−I89
K90−K93
I70−I79
K50−K52
S00−S09
C50−C50
C60−C63
G30−G32
1.0
0.5
0.0
2001
Decade commencing
B
Combined improvements in five−year mortality following admission
C
Observed improvements in mortality
change in lnHR from one decade to the next
0.50
0.25
0.00
−0.25
−0.50
2001
Decade commencing

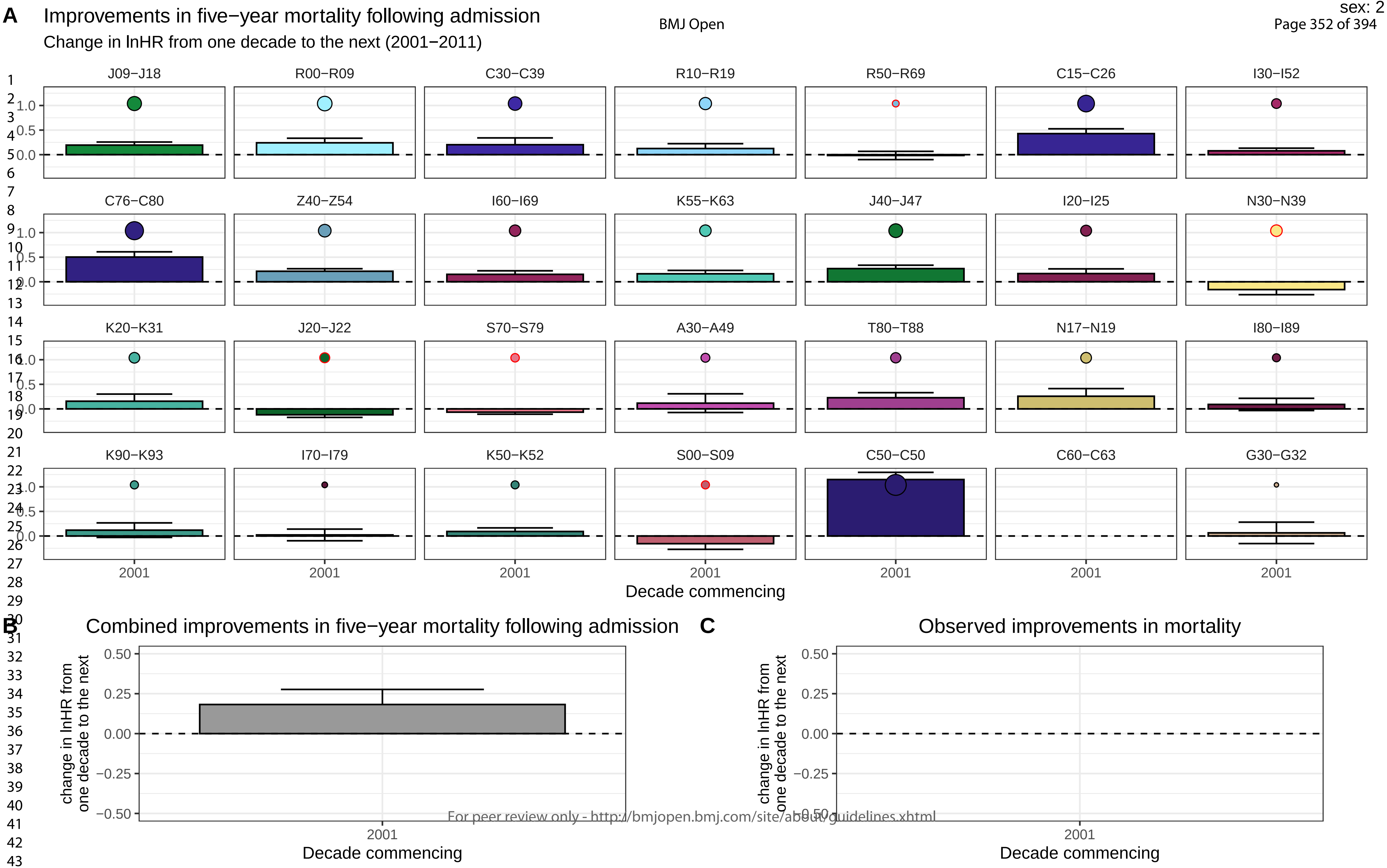
sex: 2
A
Improvements in five−year mortality following admission
Change in lnHR from one decade to the next (2001−2011)
J09−J18
R00−R09
C30−C39
R10−R19
R50−R69
C15−C26
I30−I52
C76−C80
Z40−Z54
I60−I69
K55−K63
J40−J47
I20−I25
N30−N39
K20−K31
J20−J22
S70−S79
A30−A49
T80−T88
N17−N19
I80−I89
K90−K93
I70−I79
K50−K52
S00−S09
C50−C50
C60−C63
G30−G32
1.0
0.5
0.0
2001
Decade commencing
B
Combined improvements in five−year mortality following admission
change in lnHR from one decade to the next
0.50
0.25
0.00
−0.25
−0.50
2001
Decade commencing
C
Observed improvements in mortality
change in lnHR from one decade to the next
0.50
0.25
0.00
−0.25
−0.50
2001
Decade commencing

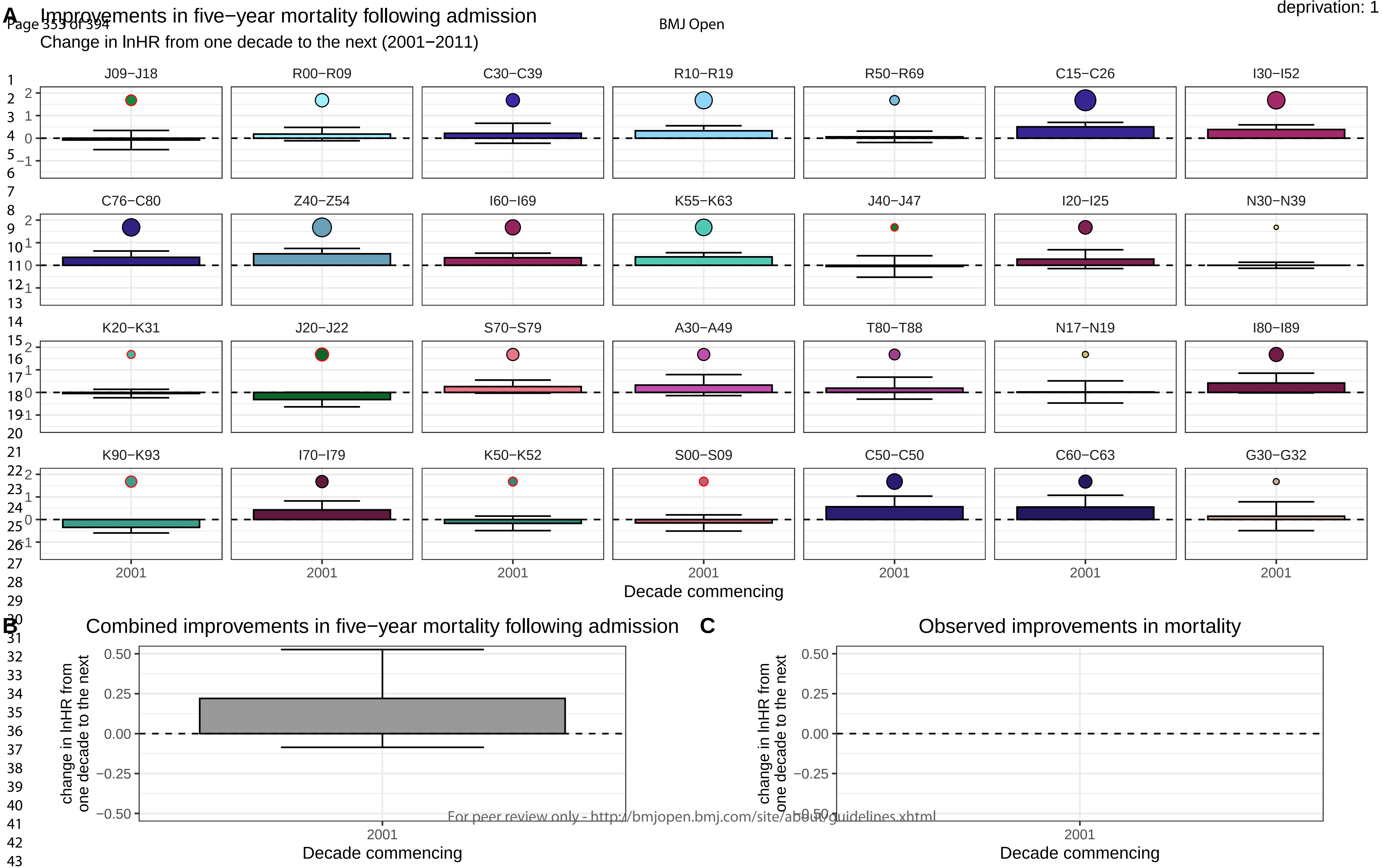
A Improvements in five−year mortality following admission
Change in lnHR from one decade to the next (2001−2011)
deprivation: 1
J09−J18
R00−R09
C30−C39
R10−R19
R50−R69
C15−C26
I30−I52
C76−C80
Z40−Z54
I60−I69
K55−K63
J40−J47
I20−I25
N30−N39
K20−K31
J20−J22
S70−S79
A30−A49
T80−T88
N17−N19
I80−I89
K90−K93
I70−I79
K50−K52
S00−S09
C50−C50
C60−C63
G30−G32
2
1
0
−1
2001
Decade commencing
B Combined improvements in five−year mortality following admission
change in lnHR from one decade to the next
0.50
0.25
0.00
−0.25
−0.50
2001
Decade commencing
C Observed improvements in mortality
change in lnHR from one decade to the next
0.50
0.25
0.00
−0.25
−0.50
2001
Decade commencing

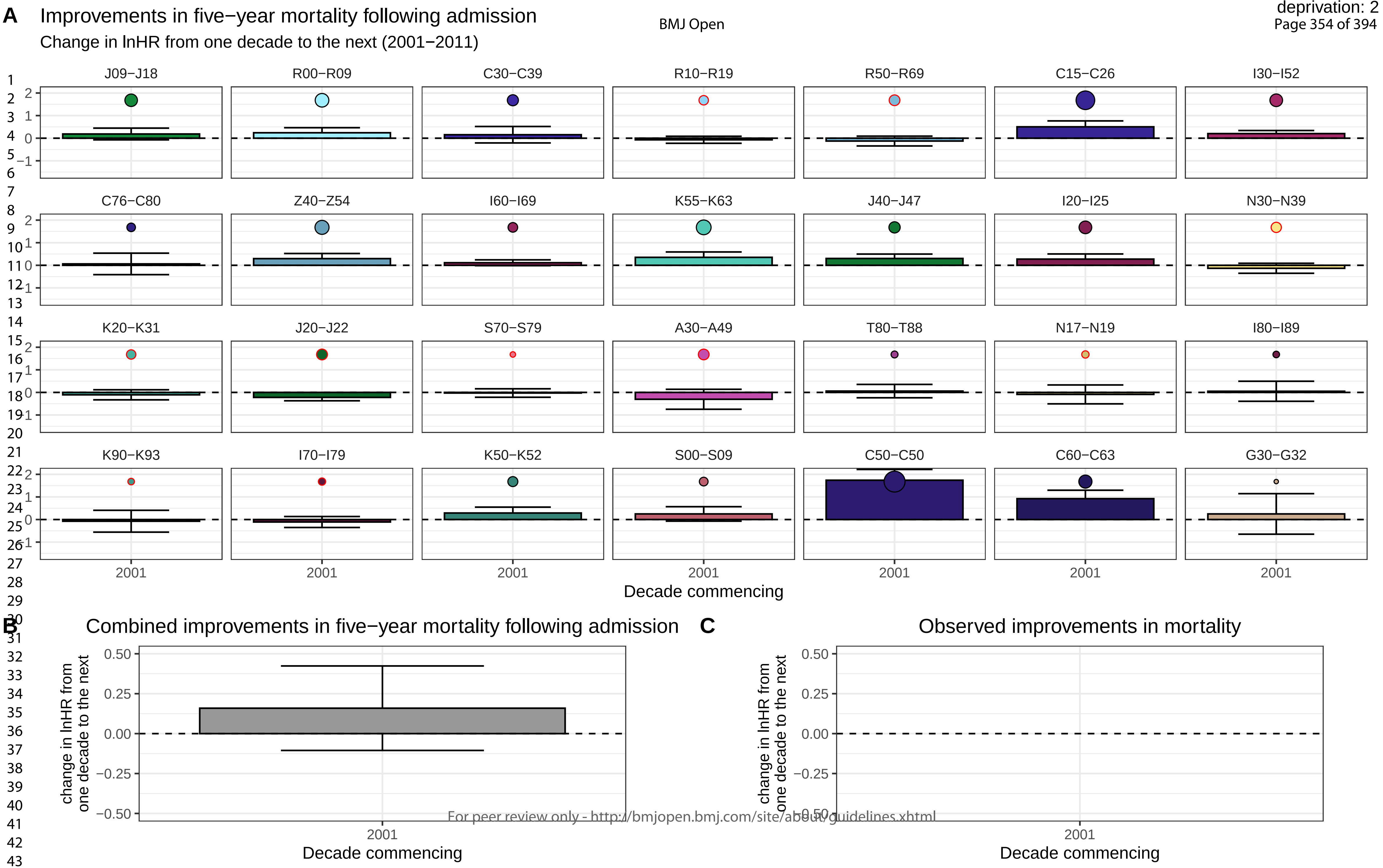
deprivation: 2
A
Improvements in five−year mortality following admission
Change in lnHR from one decade to the next (2001−2011)
J09−J18
R00−R09
C30−C39
R10−R19
R50−R69
C15−C26
I30−I52
C76−C80
Z40−Z54
I60−I69
K55−K63
J40−J47
I20−I25
N30−N39
K20−K31
J20−J22
S70−S79
A30−A49
T80−T88
N17−N19
I80−I89
K90−K93
I70−I79
K50−K52
S00−S09
C50−C50
C60−C63
G30−G32
2
1
0
−1
2001
Decade commencing
B
Combined improvements in five−year mortality following admission
change in lnHR from one decade to the next
0.50
0.25
0.00
−0.25
−0.50
2001
Decade commencing
C
Observed improvements in mortality
change in lnHR from one decade to the next
0.50
0.25
0.00
−0.25
−0.50
2001
Decade commencing

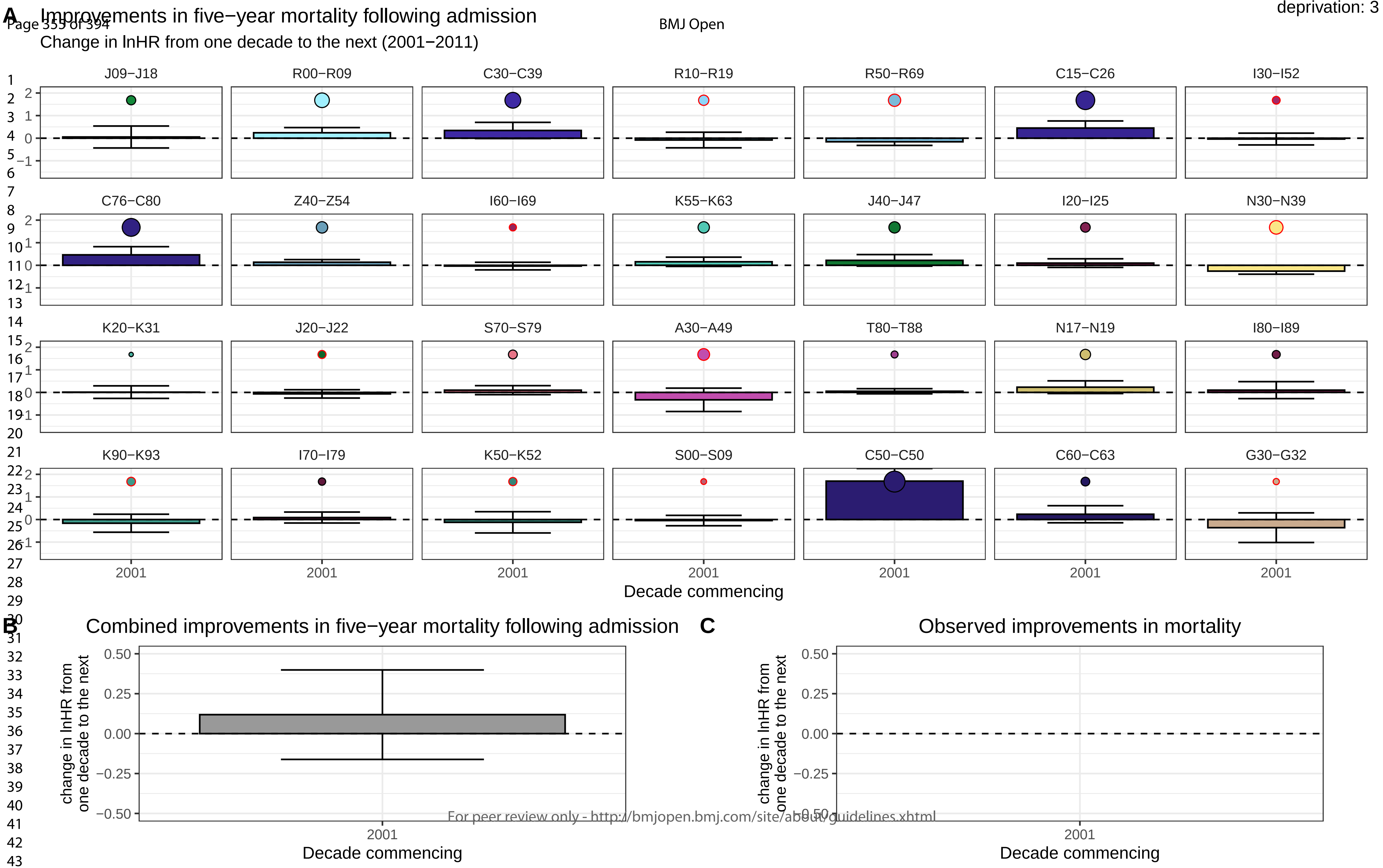
A
Improvements in five−year mortality following admission
Change in lnHR from one decade to the next (2001−2011)
deprivation: 3
J09−J18
R00−R09
C30−C39
R10−R19
R50−R69
C15−C26
I30−I52
C76−C80
Z40−Z54
I60−I69
K55−K63
J40−J47
I20−I25
N30−N39
K20−K31
J20−J22
S70−S79
A30−A49
T80−T88
N17−N19
I80−I89
K90−K93
I70−I79
K50−K52
S00−S09
C50−C50
C60−C63
G30−G32
2001
Decade commencing
B
Combined improvements in five−year mortality following admission
change in lnHR from one decade to the next
Decade commencing
C
Observed improvements in mortality
change in lnHR from one decade to the next
Decade commencing

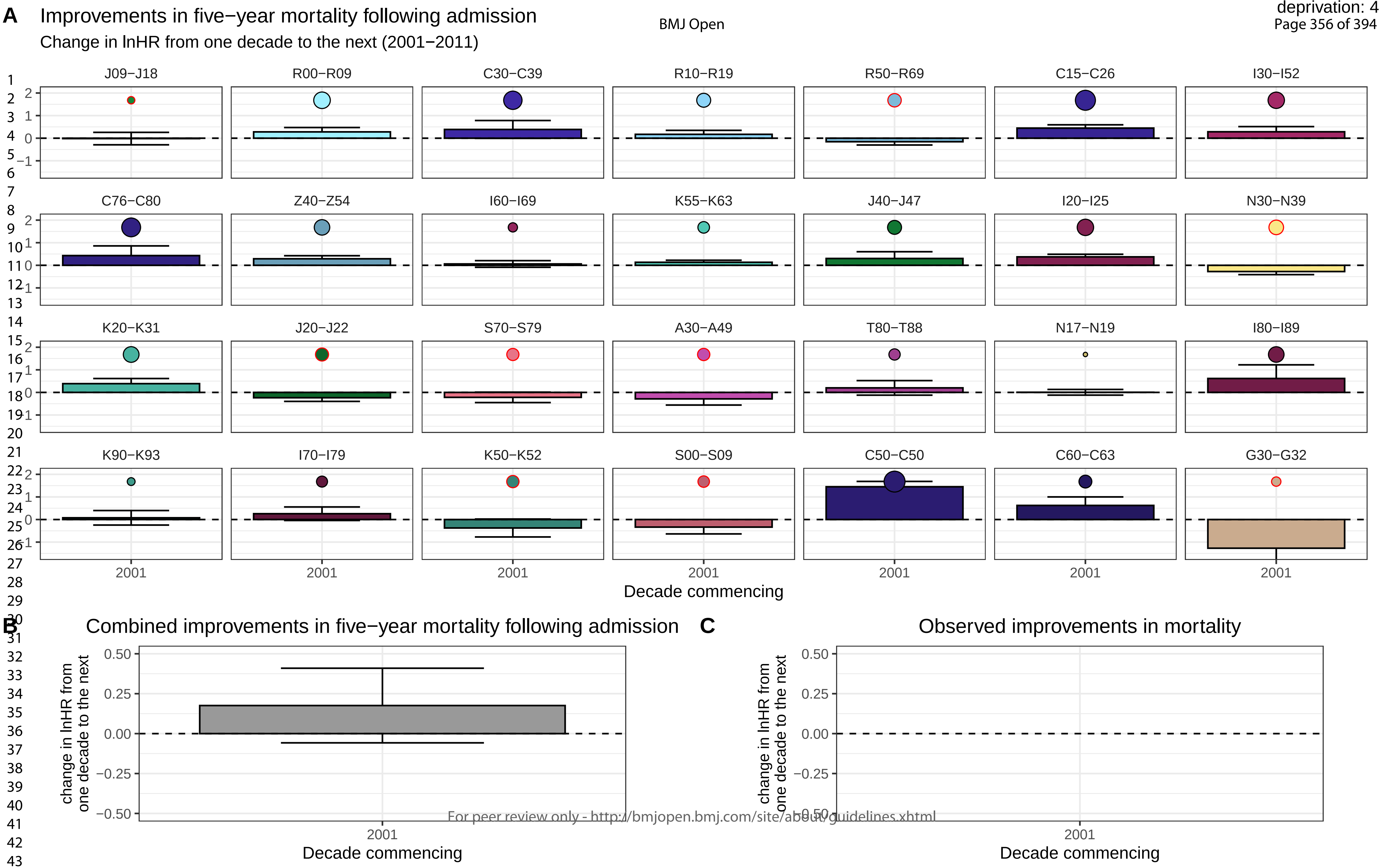
deprivation: 4
A
Improvements in five−year mortality following admission
Change in lnHR from one decade to the next (2001−2011)
J09−J18
R00−R09
C30−C39
R10−R19
R50−R69
C15−C26
I30−I52
C76−C80
Z40−Z54
I60−I69
K55−K63
J40−J47
I20−I25
N30−N39
K20−K31
J20−J22
S70−S79
A30−A49
T80−T88
N17−N19
I80−I89
K90−K93
I70−I79
K50−K52
S00−S09
C50−C50
C60−C63
G30−G32
2
1
0
−1
2001
Decade commencing
B
Combined improvements in five−year mortality following admission
change in lnHR from one decade to the next
0.50
0.25
0.00
−0.25
−0.50
2001
Decade commencing
C
Observed improvements in mortality
change in lnHR from one decade to the next
0.50
0.25
0.00
−0.25
−0.50
2001
Decade commencing

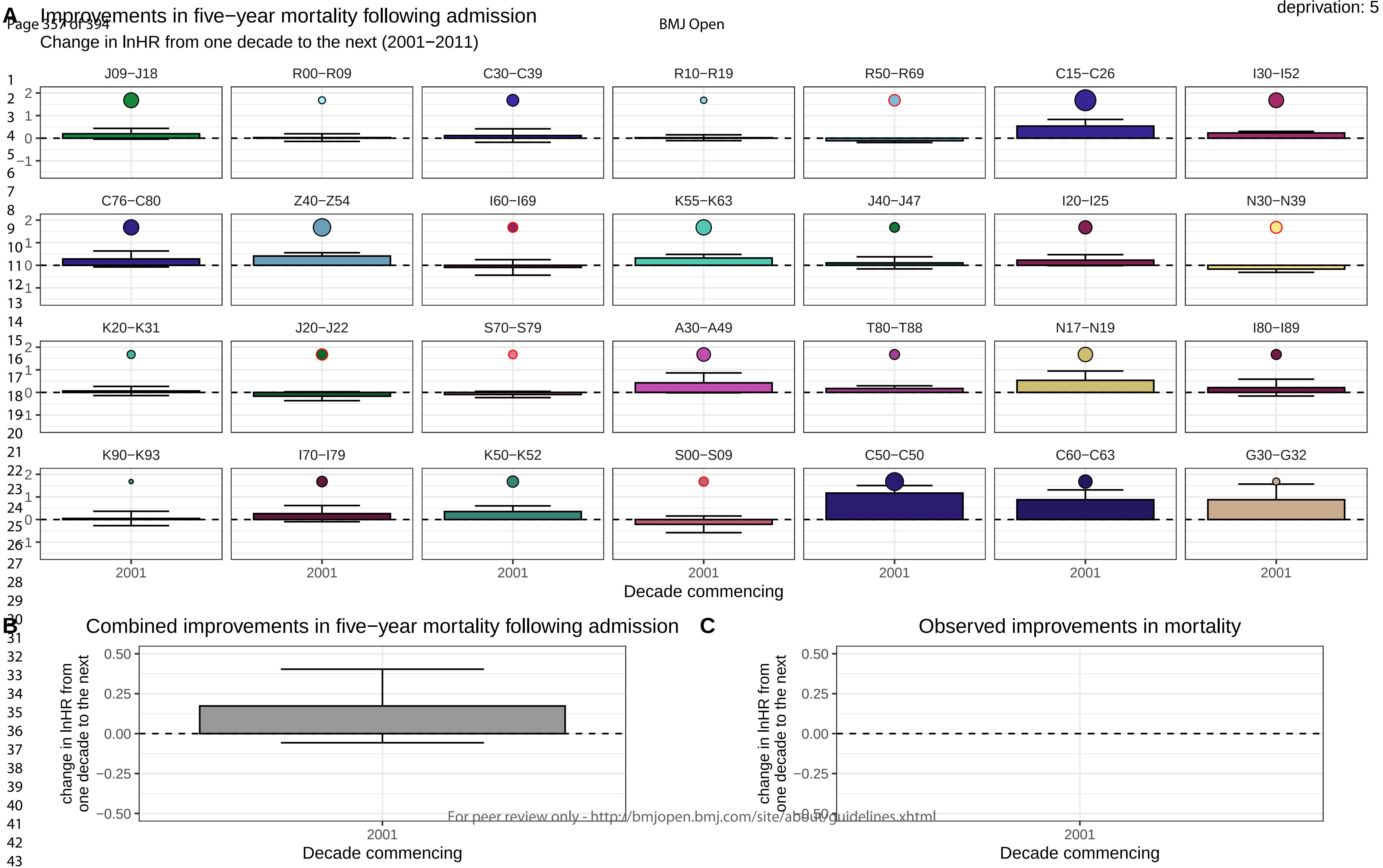
deprivation: 5
A
Improvements in five−year mortality following admission
Change in lnHR from one decade to the next (2001−2011)
J09−J18
R00−R09
C30−C39
R10−R19
R50−R69
C15−C26
I30−I52
C76−C80
Z40−Z54
I60−I69
K55−K63
J40−J47
I20−I25
N30−N39
K20−K31
J20−J22
S70−S79
A30−A49
T80−T88
N17−N19
I80−I89
K90−K93
I70−I79
K50−K52
S00−S09
C50−C50
C60−C63
G30−G32
2
1
0
−1
2001
Decade commencing
B
Combined improvements in five−year mortality following admission
change in lnHR from one decade to the next
0.50
0.25
0.00
−0.25
−0.50
2001
Decade commencing
C
Observed improvements in mortality
change in lnHR from one decade to the next
0.50
0.25
0.00
−0.25
−0.50
2001
Decade commencing

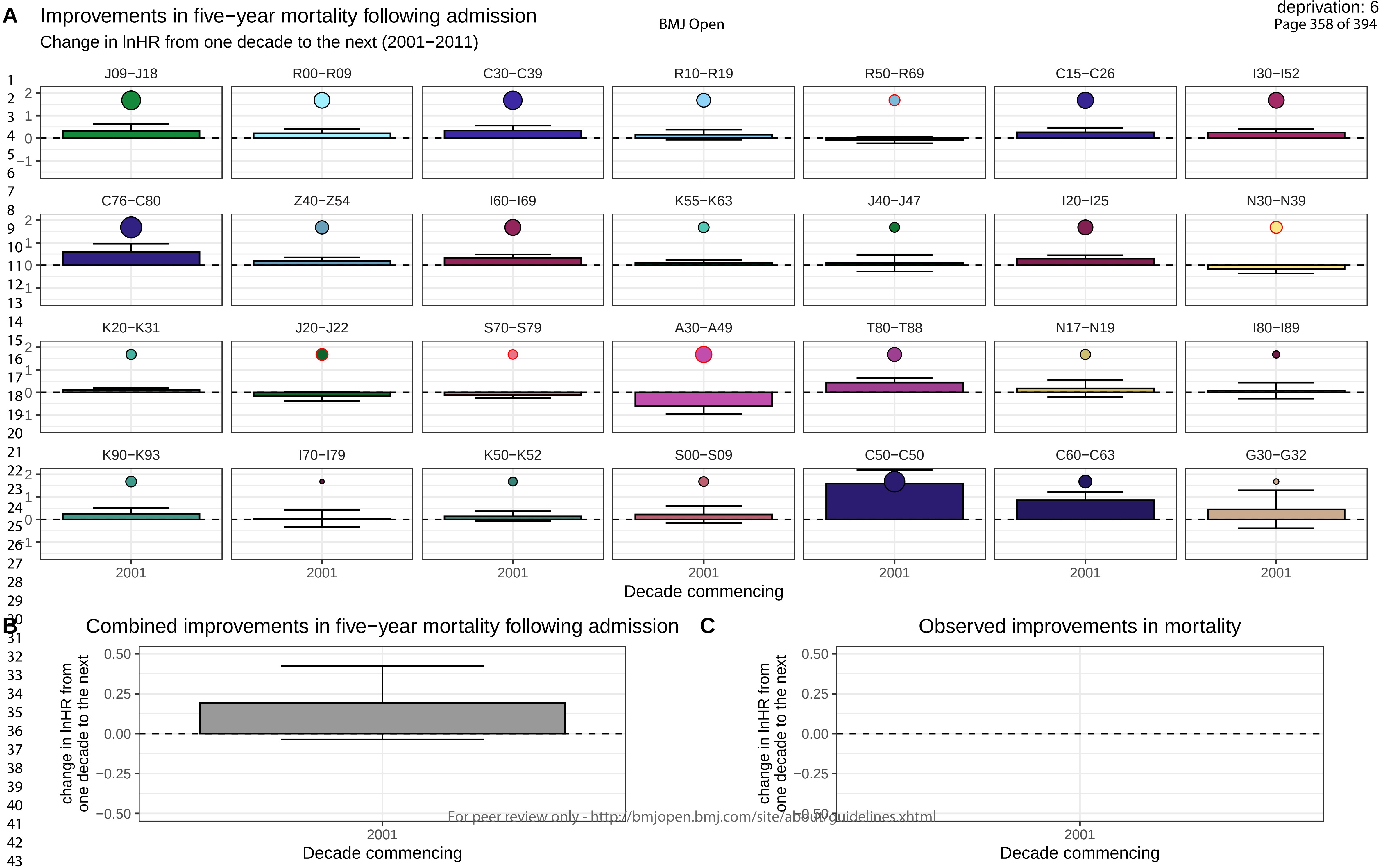
A
Improvements in five−year mortality following admission
Change in lnHR from one decade to the next (2001−2011)
J09−J18
R00−R09
C30−C39
R10−R19
R50−R69
C15−C26
I30−I52
C76−C80
Z40−Z54
I60−I69
K55−K63
J40−J47
I20−I25
N30−N39
K20−K31
J20−J22
S70−S79
A30−A49
T80−T88
N17−N19
I80−I89
K90−K93
I70−I79
K50−K52
S00−S09
C50−C50
C60−C63
G30−G32
2001
Decade commencing
B
Combined improvements in five−year mortality following admission
change in lnHR from one decade to the next
0.50
0.25
0.00
−0.25
−0.50
2001
Decade commencing
C
Observed improvements in mortality
change in lnHR from one decade to the next
0.50
0.25
0.00
−0.25
−0.50
2001
Decade commencing

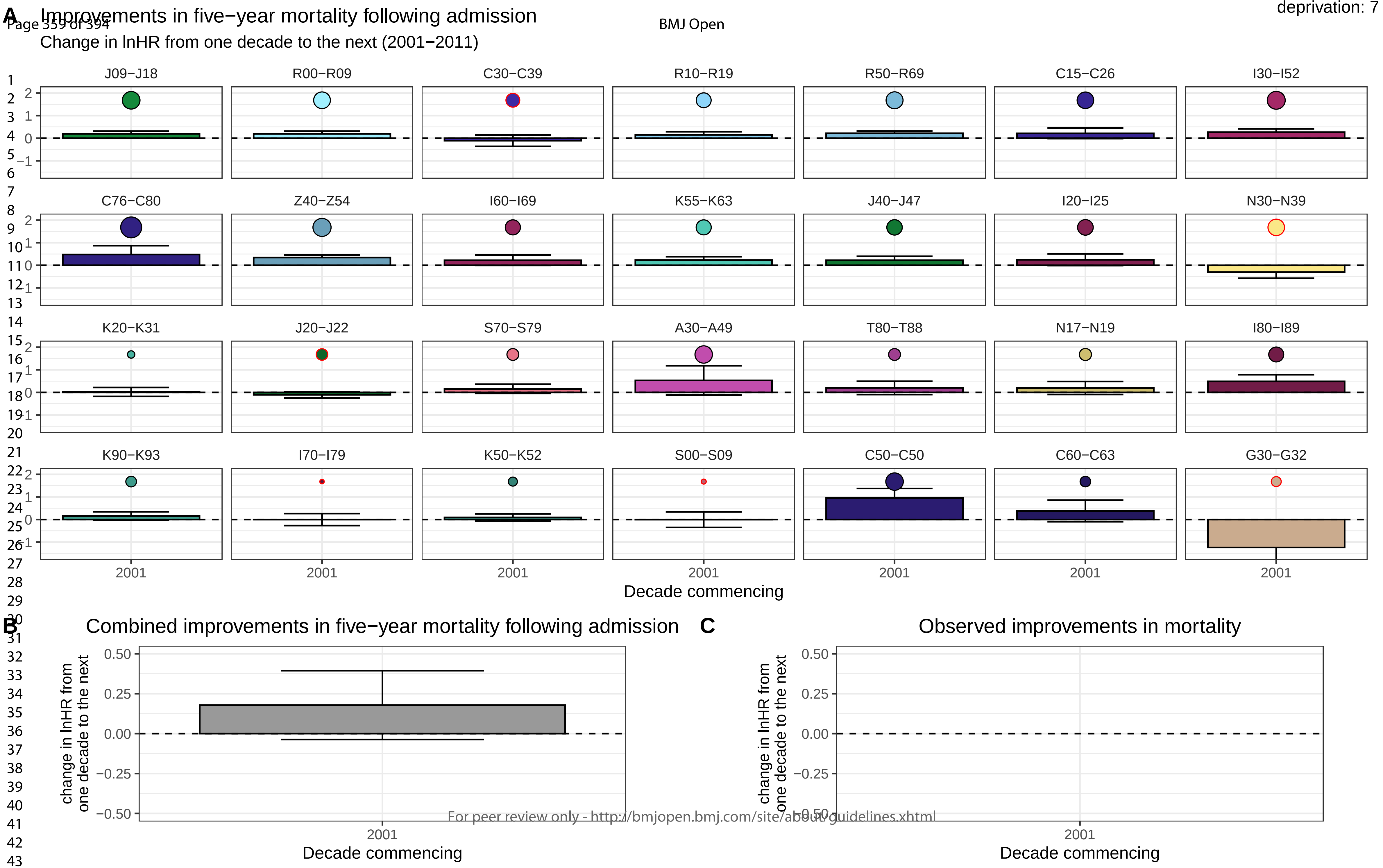
deprivation: 7
A
Improvements in five−year mortality following admission
Change in lnHR from one decade to the next (2001−2011)
J09−J18
R00−R09
C30−C39
R10−R19
R50−R69
C15−C26
I30−I52
C76−C80
Z40−Z54
I60−I69
K55−K63
J40−J47
I20−I25
N30−N39
K20−K31
J20−J22
S70−S79
A30−A49
T80−T88
N17−N19
I80−I89
K90−K93
I70−I79
K50−K52
S00−S09
C50−C50
C60−C63
G30−G32
2
1
0
−1
2001
Decade commencing
B
Combined improvements in five−year mortality following admission
C
Observed improvements in mortality
change in lnHR from one decade to the next
0.50
0.25
0.00
−0.25
−0.50
2001
Decade commencing

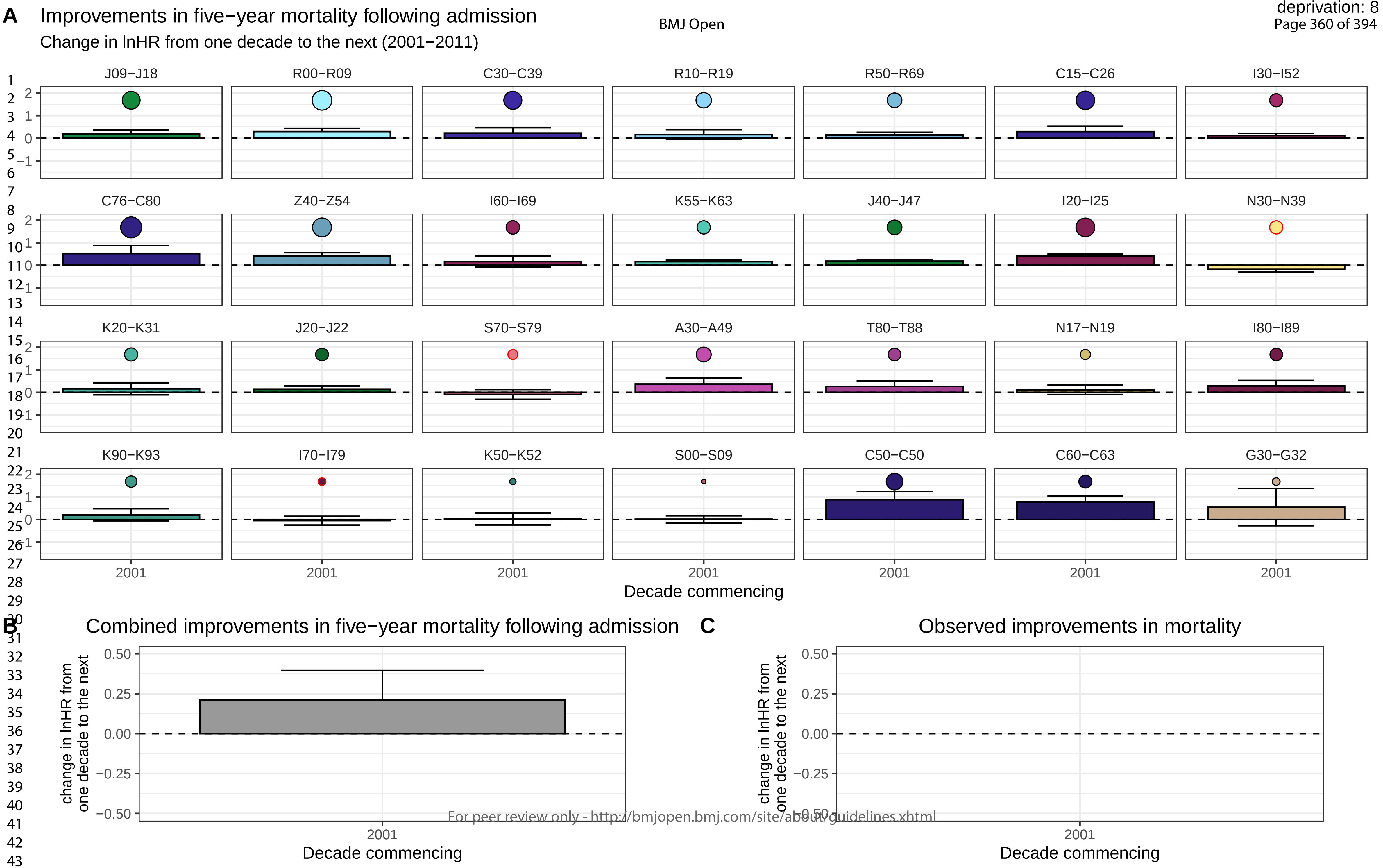

For peer review only - http://bmjopen.bmj.com/site/about/guidelines.xhtml

deprivation: 9

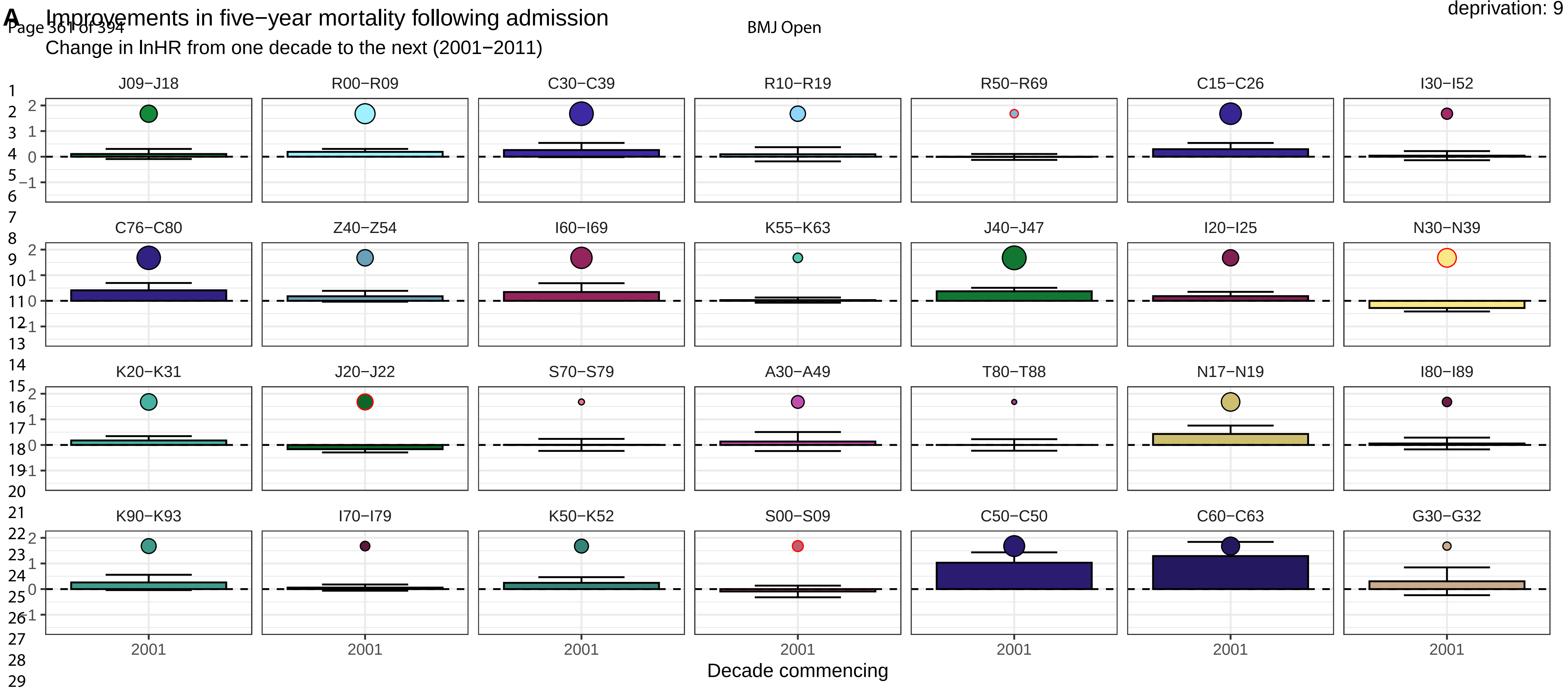

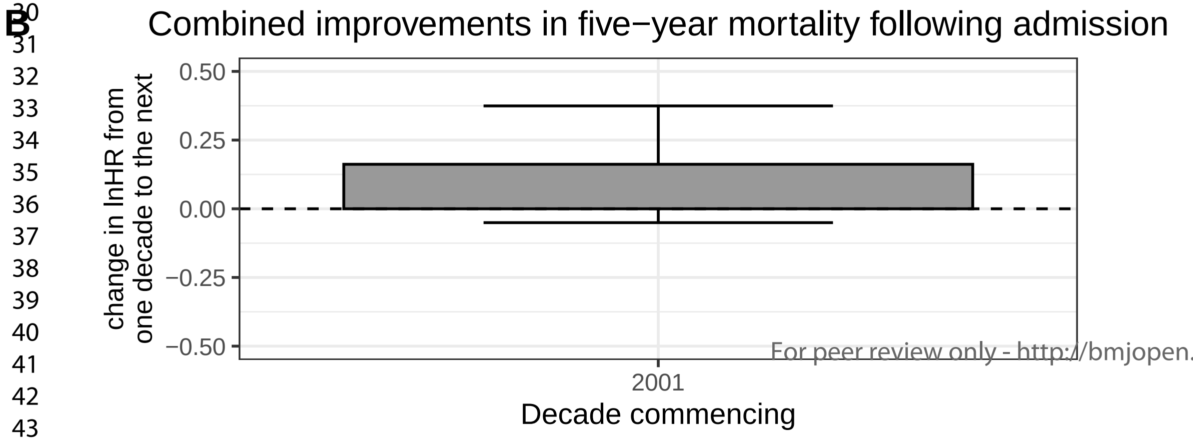

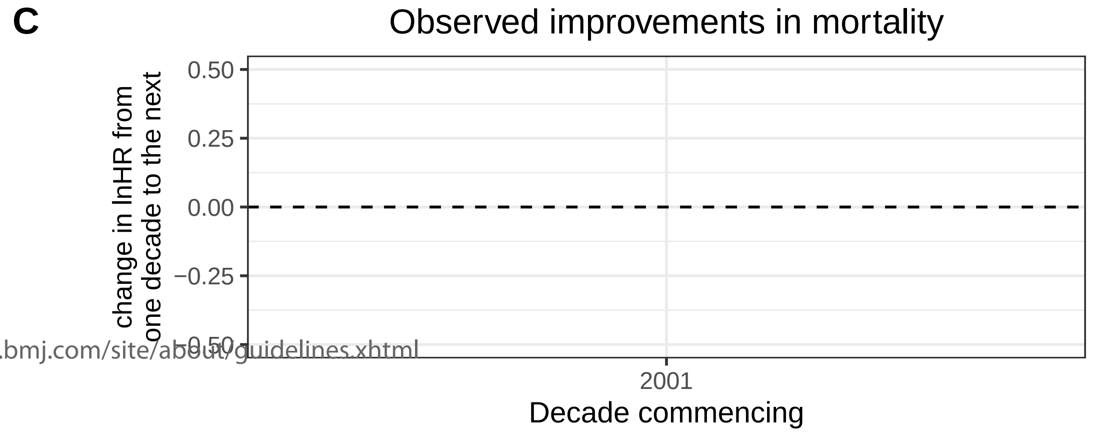

For peer review only - http://bmjopen.bmj.com/site/about/guidelines.xhtml

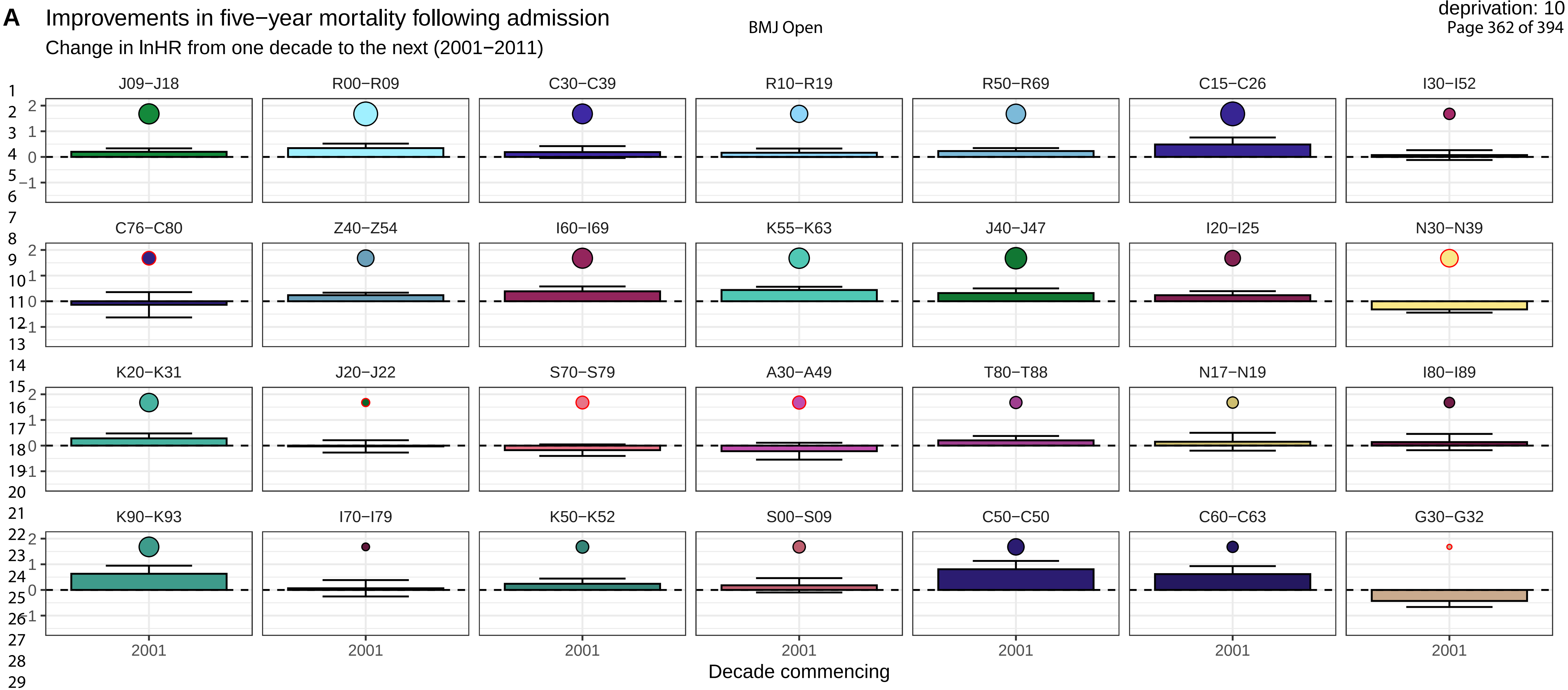
A
Improvements in five−year mortality following admission
Change in lnHR from one decade to the next (2001−2011)
J09−J18
R00−R09
C30−C39
R10−R19
R50−R69
C15−C26
I30−I52
C76−C80
Z40−Z54
I60−I69
K55−K63
J40−J47
I20−I25
N30−N39
K20−K31
J20−J22
S70−S79
A30−A49
T80−T88
N17−N19
I80−I89
K90−K93
I70−I79
K50−K52
S00−S09
C50−C50
C60−C63
G30−G32
2
1
0
−1
2001
Decade commencing

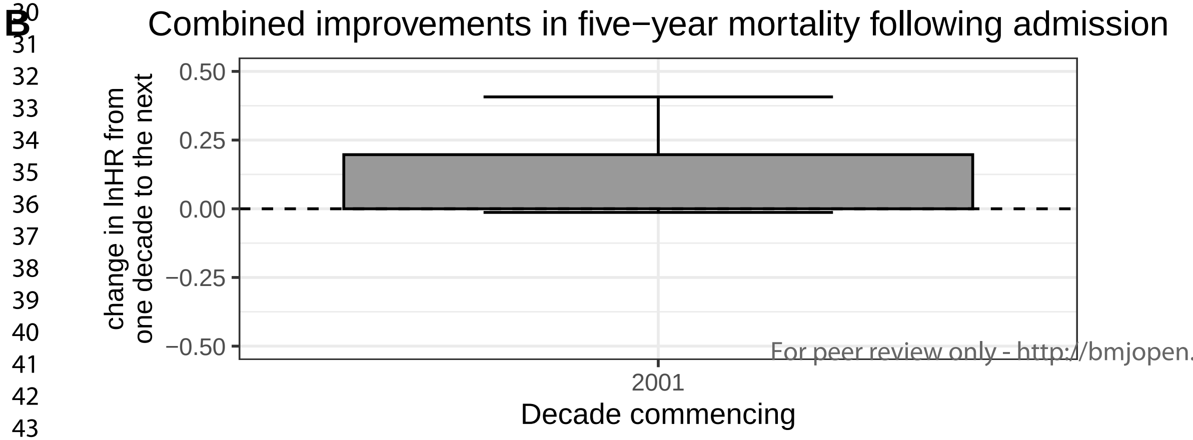
B
Combined improvements in five−year mortality following admission
change in lnHR from one decade to the next
0.50
0.25
0.00
−0.25
−0.50
2001
Decade commencing

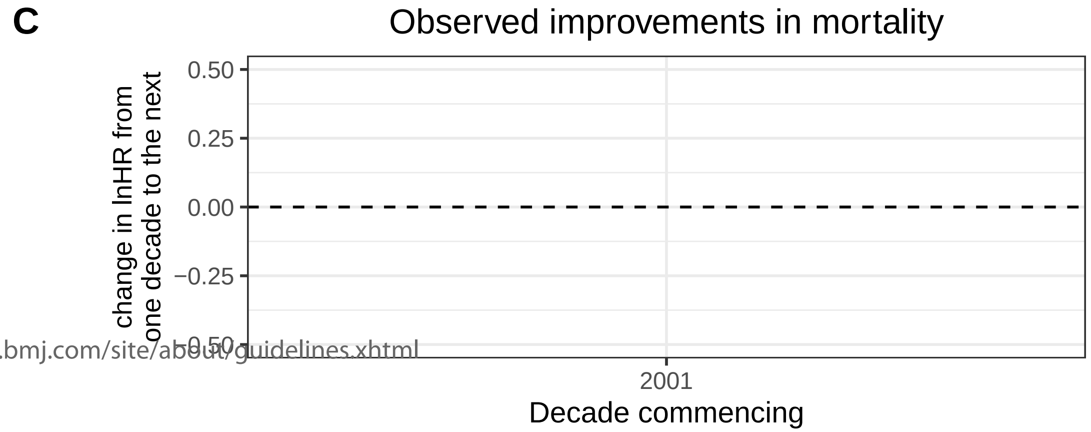
C
Observed improvements in mortality
change in lnHR from one decade to the next
0.50
0.25
0.00
−0.25
−0.50
2001
Decade commencing

Impact

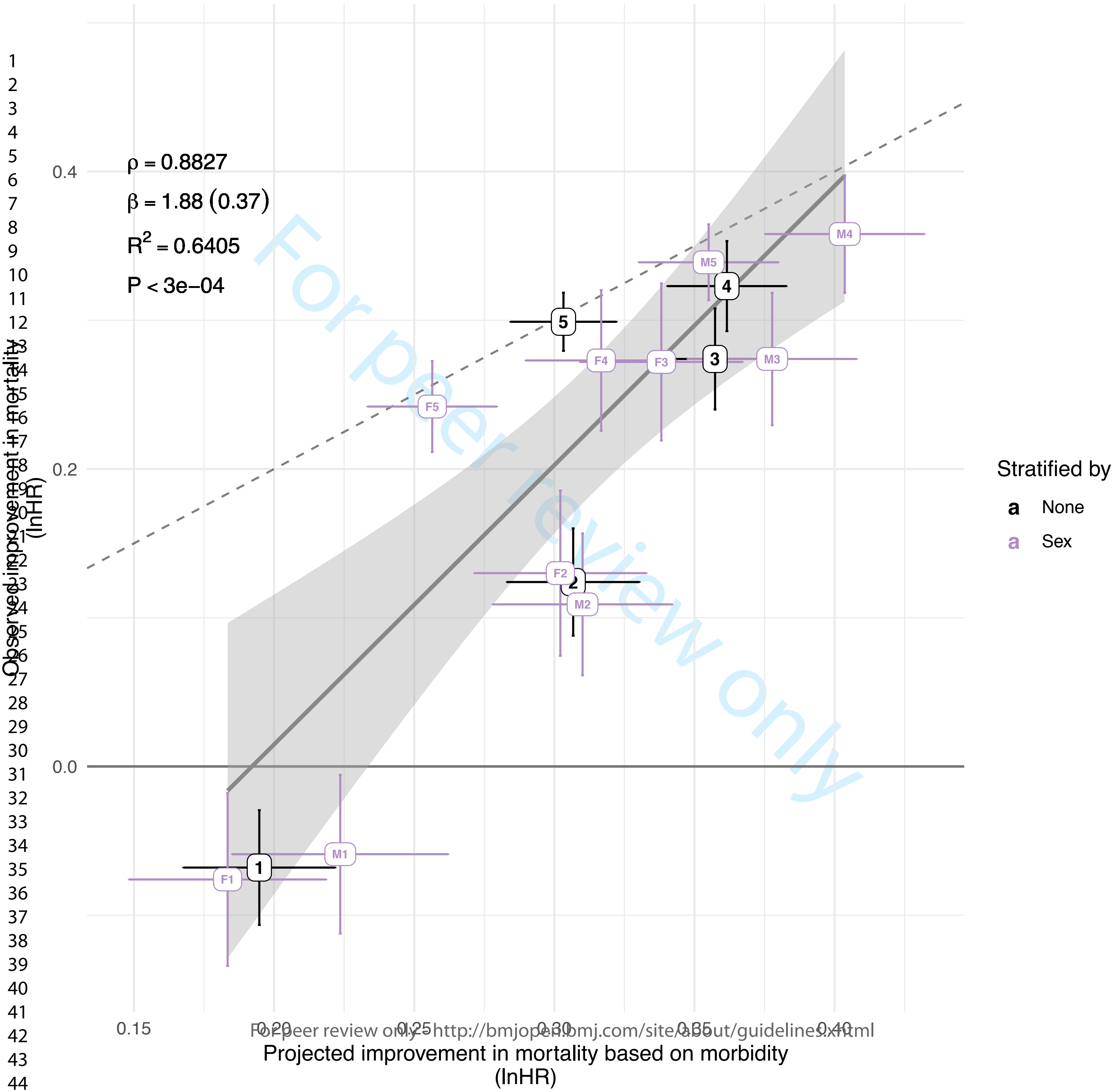

For peer review only

Stratified	Group	Mortality Improvements Observed From Death Records		Mortality Improvements Estimated from Morbidity Records		Lives Saved	
		lnHR	SE	lnHR	SE	Most	Least
None		0.95	0.0145	0.78	0.0124	I20-I25	A30-A49
Sex	M	1.02	0.0207	0.91	0.0176	C30-C39	A30-A49
	F	0.84	0.0215	0.67	0.0157	I20-I25	J09-J18
SIMD	1	1.27	0.0601	0.80	0.0449	C15-C26	J09-J18
	2	1.06	0.0560	0.74	0.0416	C15-C26	A30-A49
	3	1.01	0.0530	0.72	0.0433	C15-C26	A30-A49
	4	0.91	0.0506	0.89	0.0391	I20-I25	A30-A49
	5	0.90	0.0481	0.79	0.0374	C15-C26	A30-A49
	6	1.04	0.0465	0.85	0.0375	I20-I25	A30-A49
	7	0.89	0.0437	0.80	0.0351	I20-I25	J09-J18
	8	0.90	0.0416	0.86	0.0328	I20-I25	J09-J18
	9	0.88	0.0402	0.63	0.0355	I20-I25	J09-J18
	10	0.82	0.0387	0.67	0.0355	I20-I25	J09-J18

For peer review only

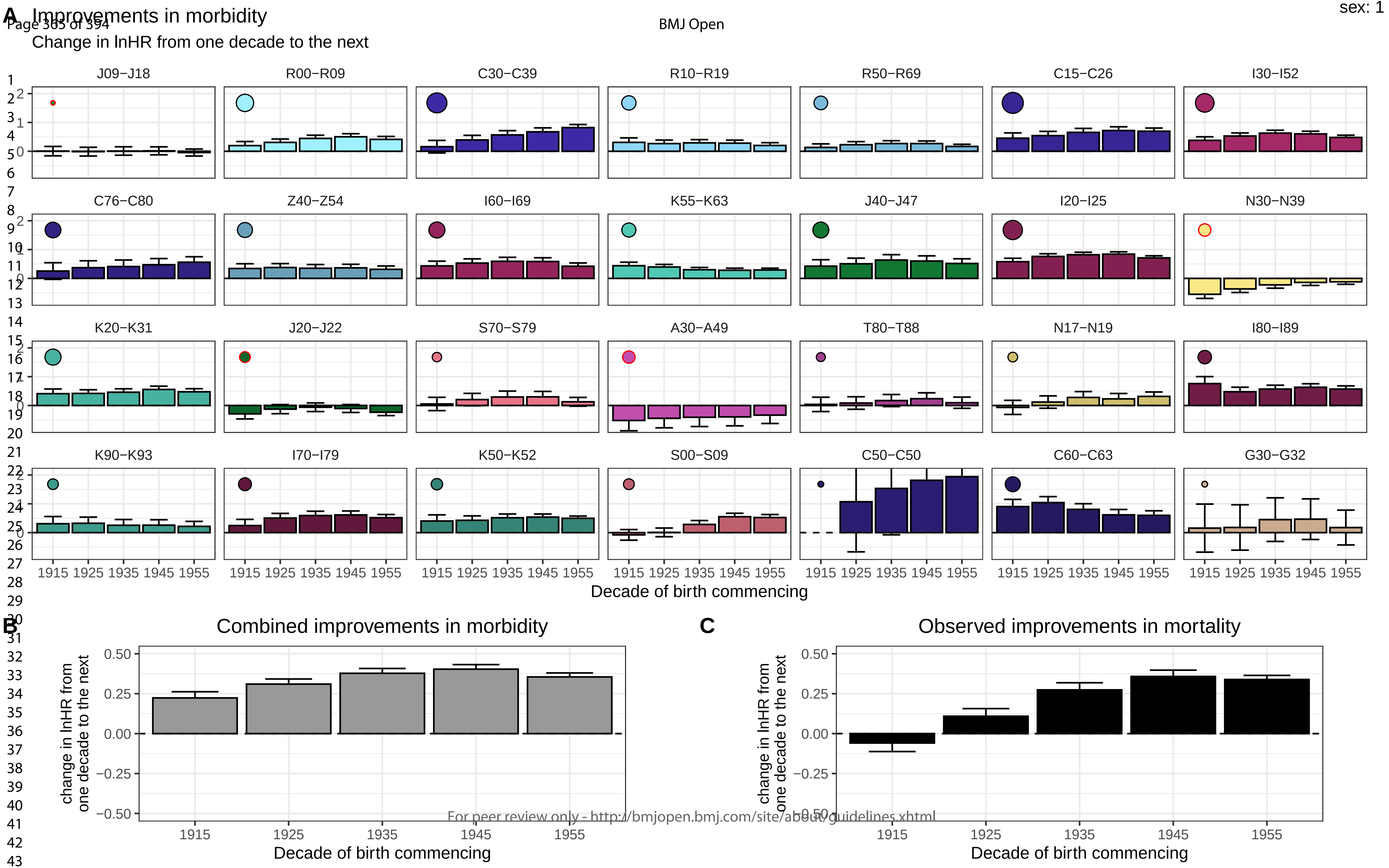

For peer review only - http://bmjopen.bmj.com/site/about/guidelines.xhtml

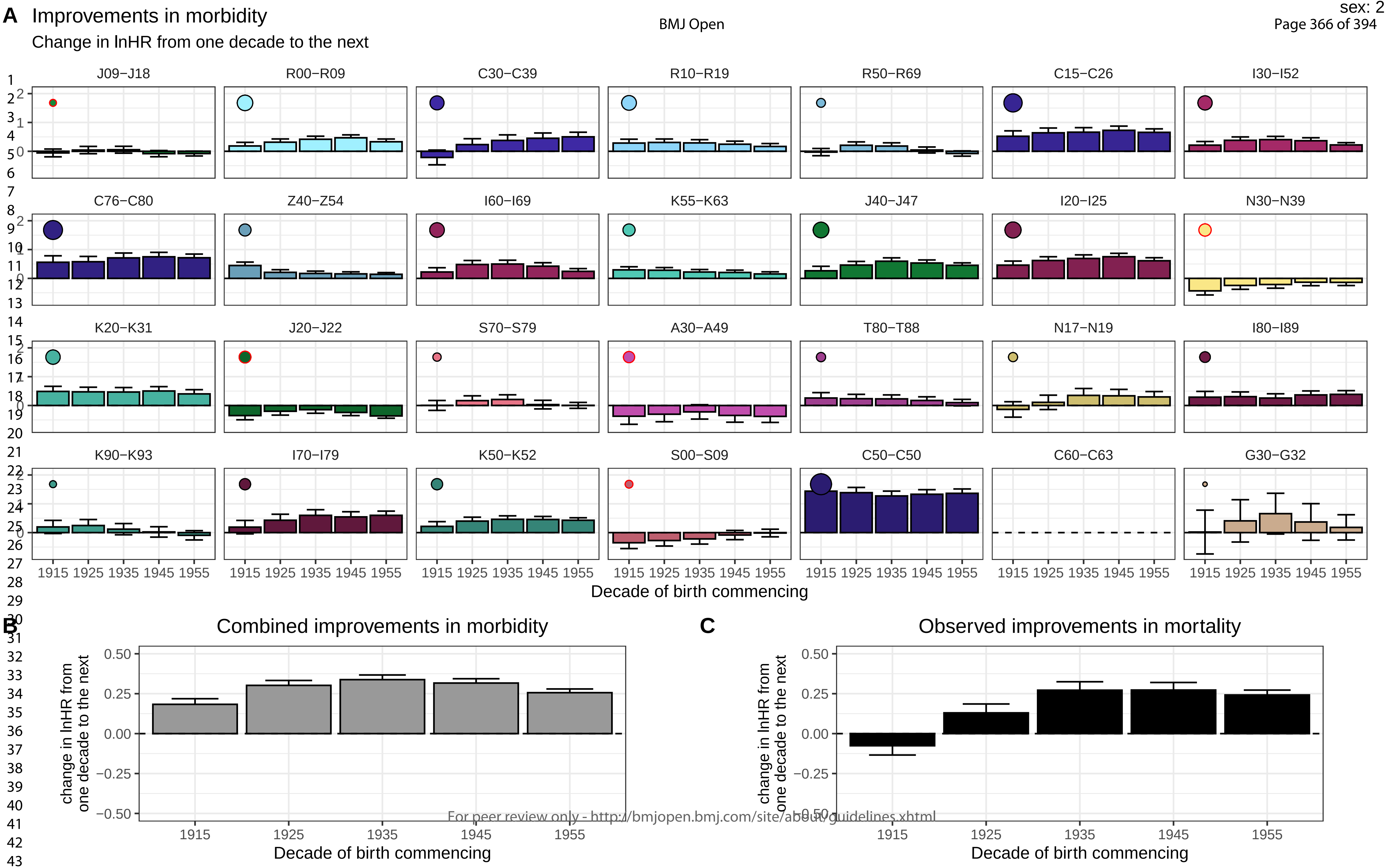
sex: 2
A
Improvements in morbidity
Change in lnHR from one decade to the next
J09−J18
R00−R09
C30−C39
R10−R19
R50−R69
C15−C26
I30−I52
C76−C80
Z40−Z54
I60−I69
K55−K63
J40−J47
I20−I25
N30−N39
K20−K31
J20−J22
S70−S79
A30−A49
T80−T88
N17−N19
I80−I89
K90−K93
I70−I79
K50−K52
S00−S09
C50−C50
C60−C63
G30−G32
1915 1925 1935 1945 1955
Decade of birth commencing
B
Combined improvements in morbidity
change in lnHR from one decade to the next
0.50
0.25
0.00
−0.25
−0.50
Decade of birth commencing
C
Observed improvements in mortality
change in lnHR from one decade to the next
Decade of birth commencing

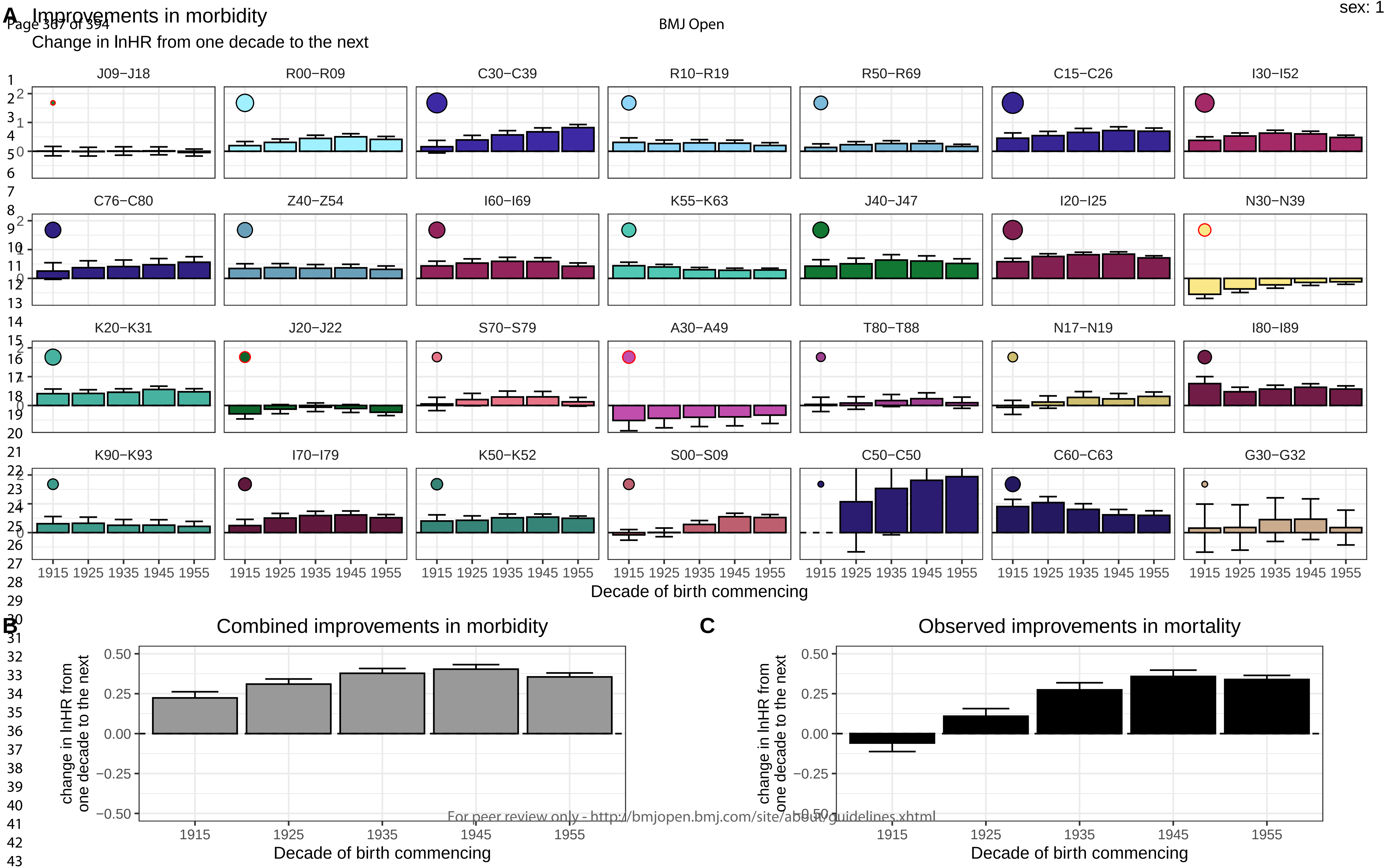
A Improvements in morbidity
Change in lnHR from one decade to the next
sex: 1
J09−J18
R00−R09
C30−C39
R10−R19
R50−R69
C15−C26
I30−I52
C76−C80
Z40−Z54
I60−I69
K55−K63
J40−J47
I20−I25
N30−N39
K20−K31
J20−J22
S70−S79
A30−A49
T80−T88
N17−N19
I80−I89
K90−K93
I70−I79
K50−K52
S00−S09
C50−C50
C60−C63
G30−G32
0
1
2
1915 1925 1935 1945 1955
Decade of birth commencing
B Combined improvements in morbidity
C Observed improvements in mortality
change in lnHR from one decade to the next
0.50
0.25
0.00
−0.25
−0.50
1915
1925
1935
1945
1955
Decade of birth commencing

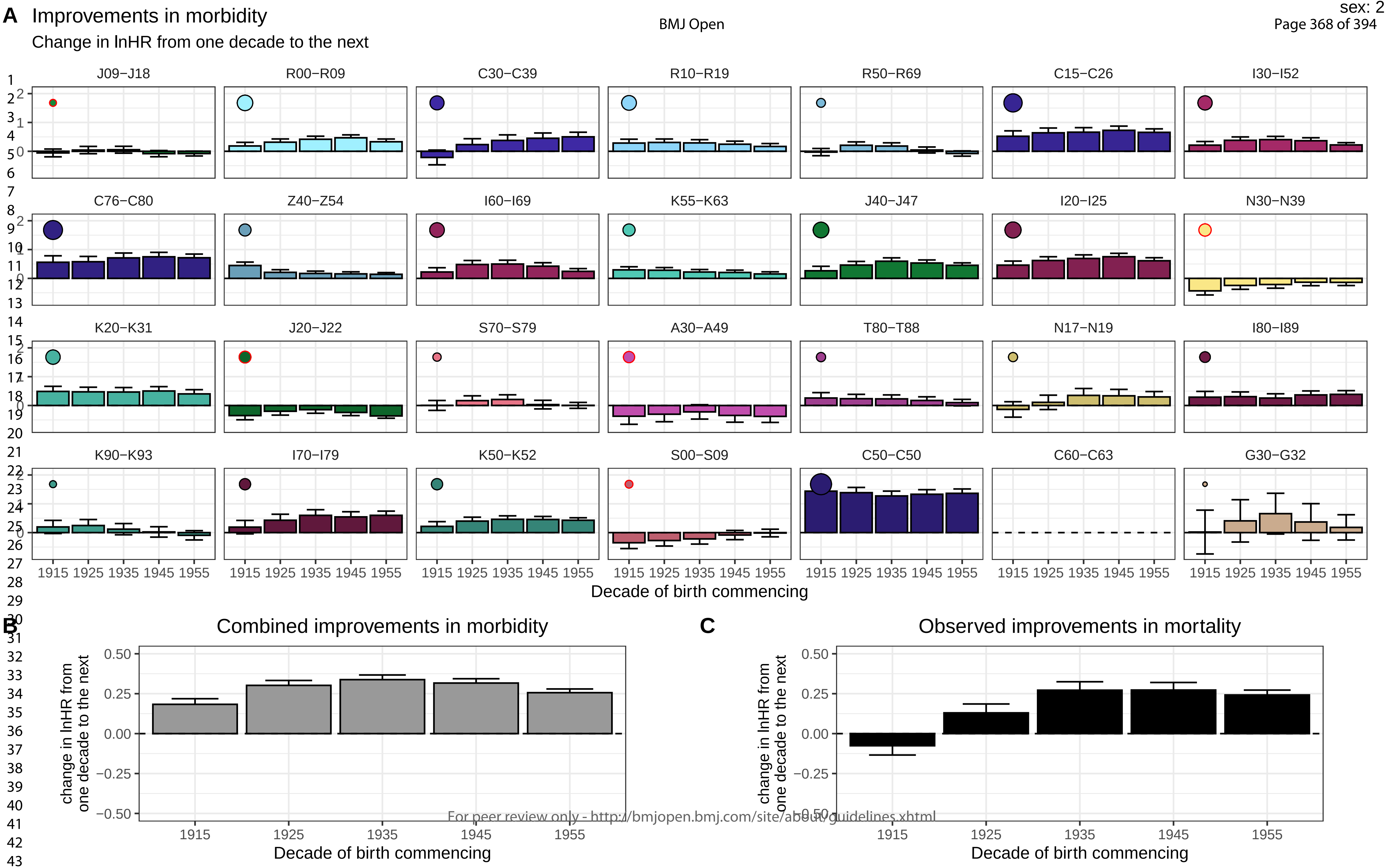

For peer review only - http://bmjopen.bmj.com/site/about/guidelines.xhtml

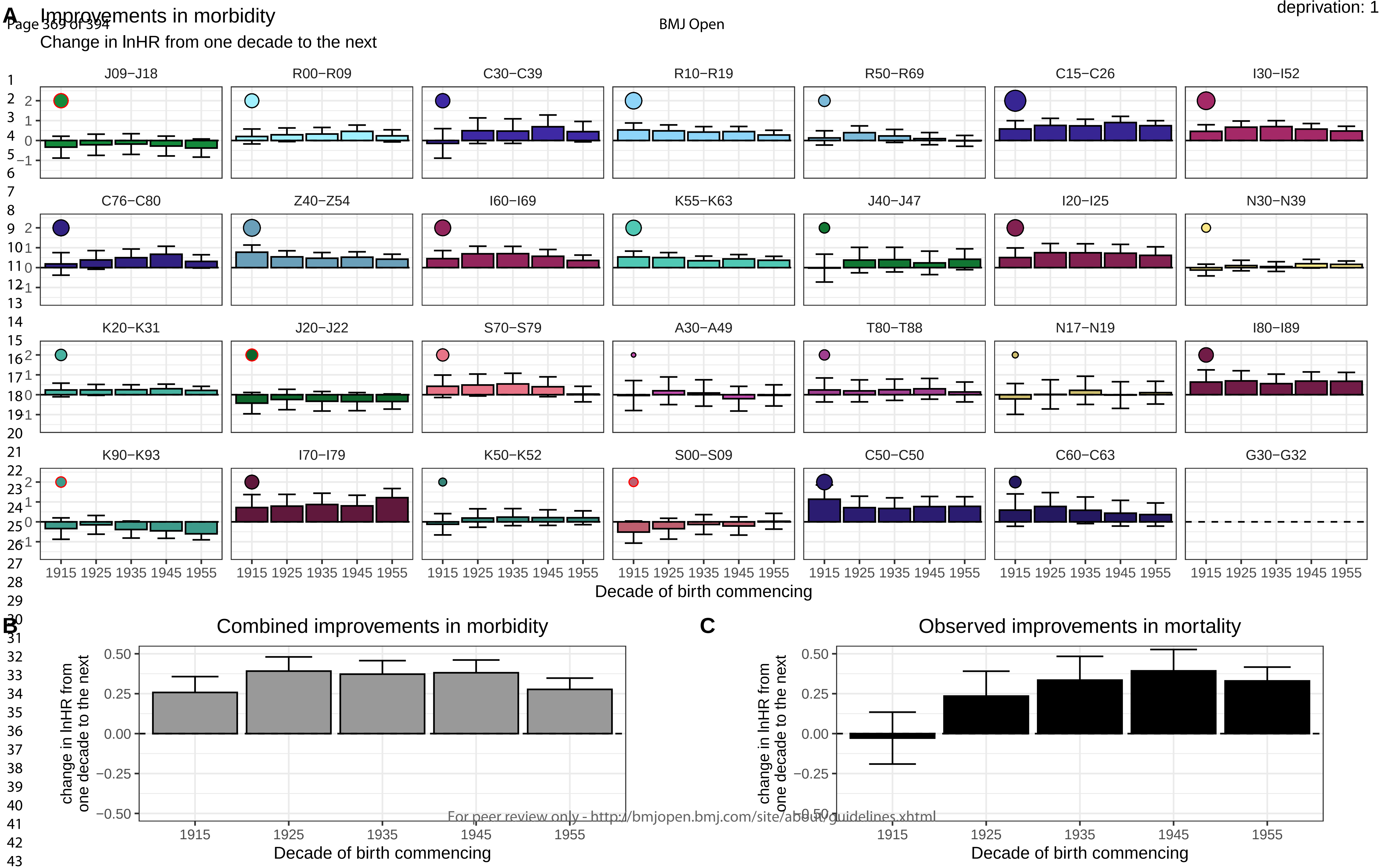
A Improvements in morbidity
Change in lnHR from one decade to the next
deprivation: 1
J09−J18
R00−R09
C30−C39
R10−R19
R50−R69
C15−C26
I30−I52
C76−C80
Z40−Z54
I60−I69
K55−K63
J40−J47
I20−I25
N30−N39
K20−K31
J20−J22
S70−S79
A30−A49
T80−T88
N17−N19
I80−I89
K90−K93
I70−I79
K50−K52
S00−S09
C50−C50
C60−C63
G30−G32
2
1
0
−1
1915 1925 1935 1945 1955
Decade of birth commencing
B Combined improvements in morbidity
change in lnHR from one decade to the next
0.50
0.25
0.00
−0.25
−0.50
1915 1925 1935 1945 1955
Decade of birth commencing
C Observed improvements in mortality
change in lnHR from one decade to the next
0.50
0.25
0.00
−0.25
−0.50
1915 1925 1935 1945 1955
Decade of birth commencing

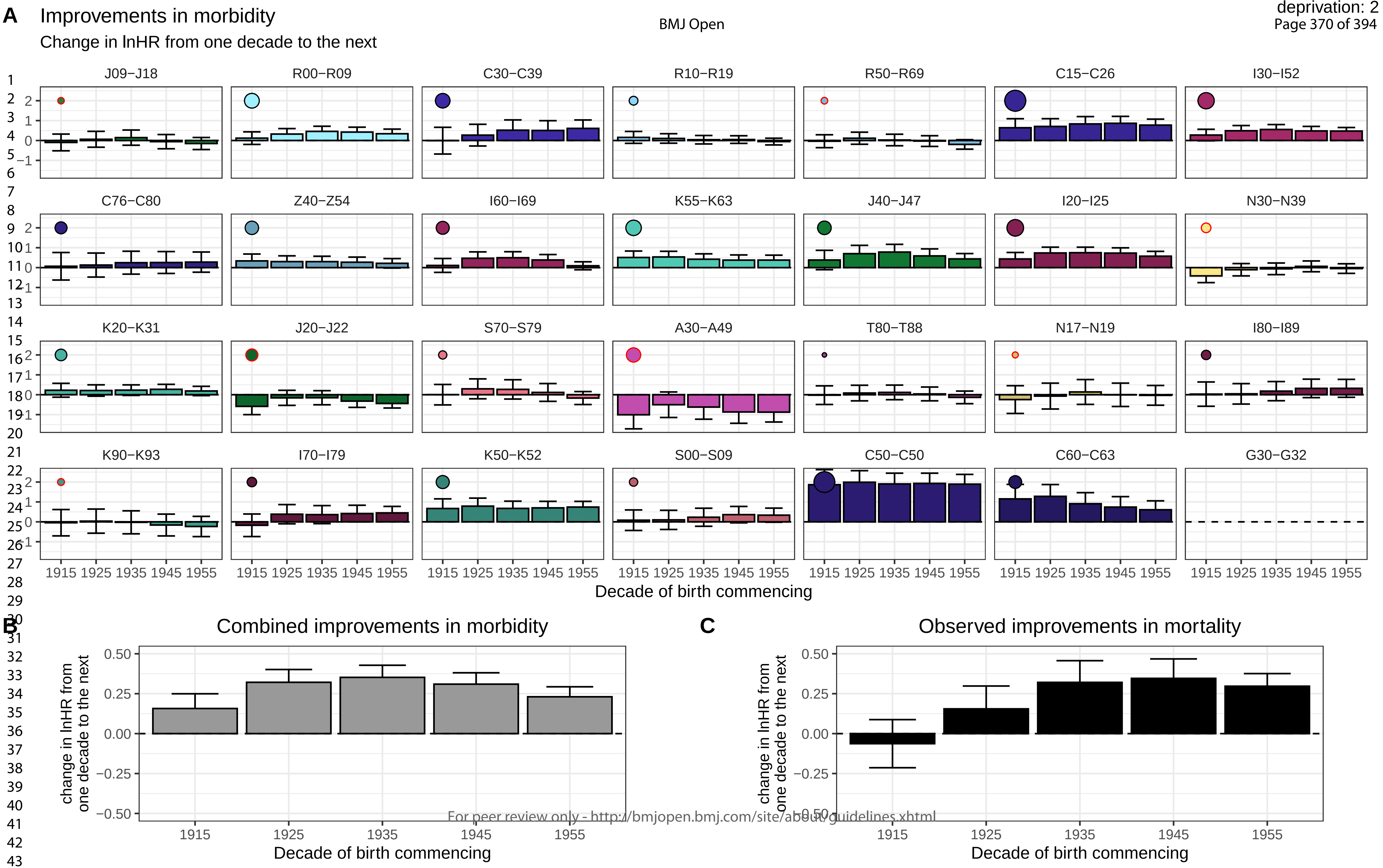

For peer review only - http://bmjopen.bmj.com/site/about/guidelines.xhtml

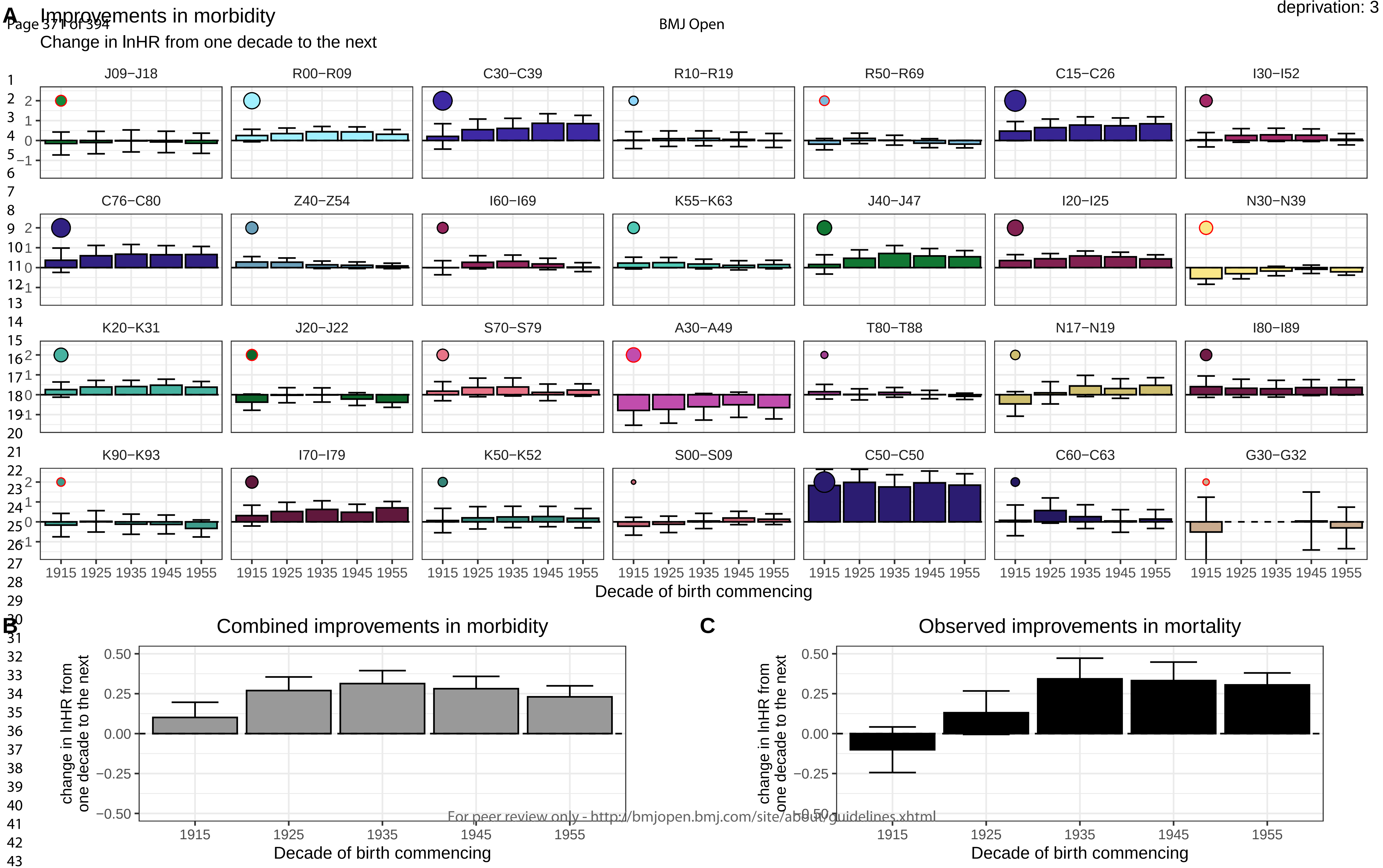

A Improvements in morbidity
Change in lnHR from one decade to the next
deprivation: 3
J09−J18
R00−R09
C30−C39
R10−R19
R50−R69
C15−C26
I30−I52
C76−C80
Z40−Z54
I60−I69
K55−K63
J40−J47
I20−I25
N30−N39
K20−K31
J20−J22
S70−S79
A30−A49
T80−T88
N17−N19
I80−I89
K90−K93
I70−I79
K50−K52
S00−S09
C50−C50
C60−C63
G30−G32
2
1
0
−1
1915 1925 1935 1945 1955
Decade of birth commencing
B
Combined improvements in morbidity
change in lnHR from one decade to the next
0.50
0.25
0.00
−0.25
−0.50
1915
1925
1935
1945
1955
Decade of birth commencing
C
Observed improvements in mortality
change in lnHR from one decade to the next
0.50
0.25
0.00
−0.25
−0.50
1915
1925
1935
1945
1955
Decade of birth commencing

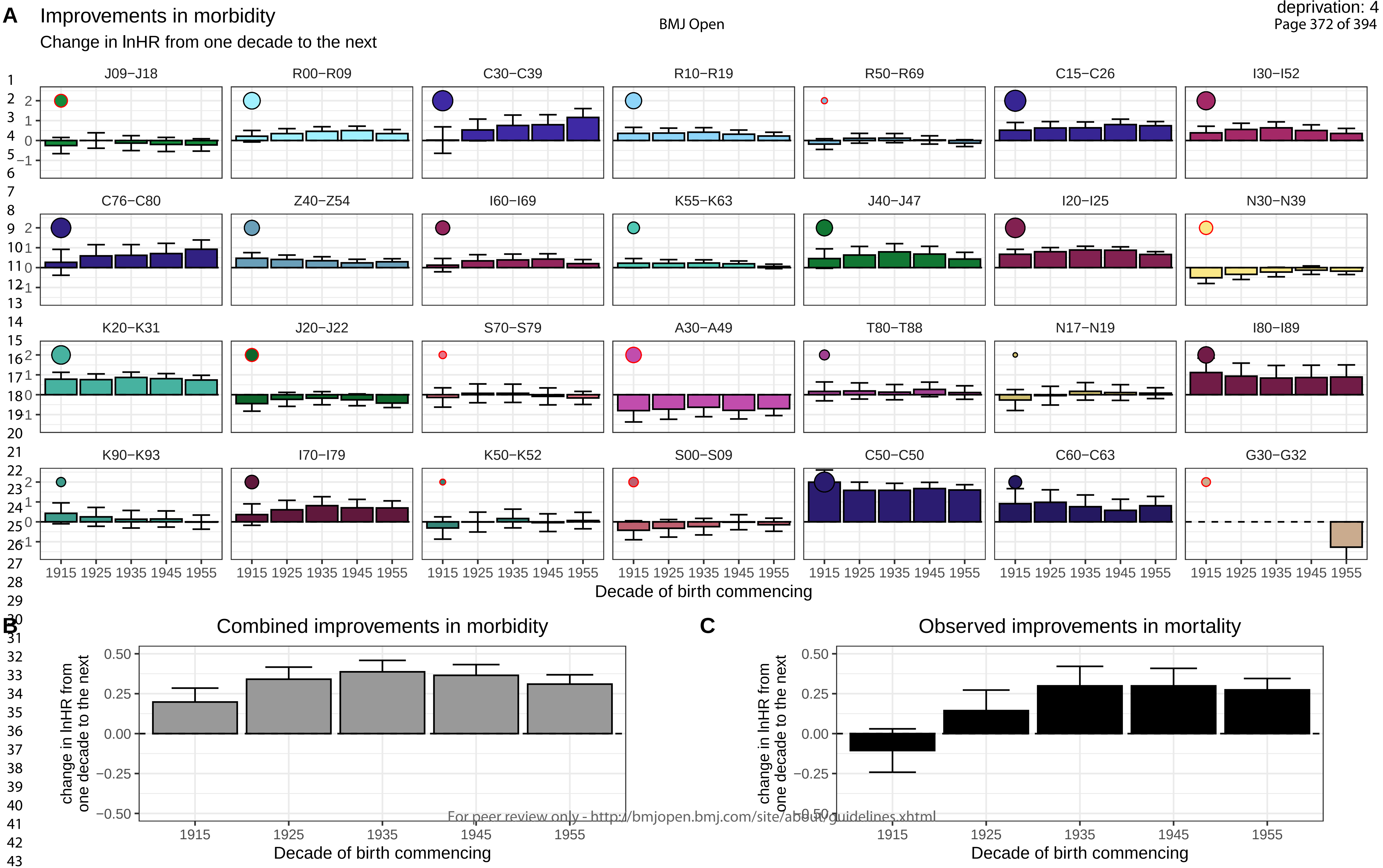

For peer review only - http://bmjopen.bmj.com/site/about/guidelines.xhtml

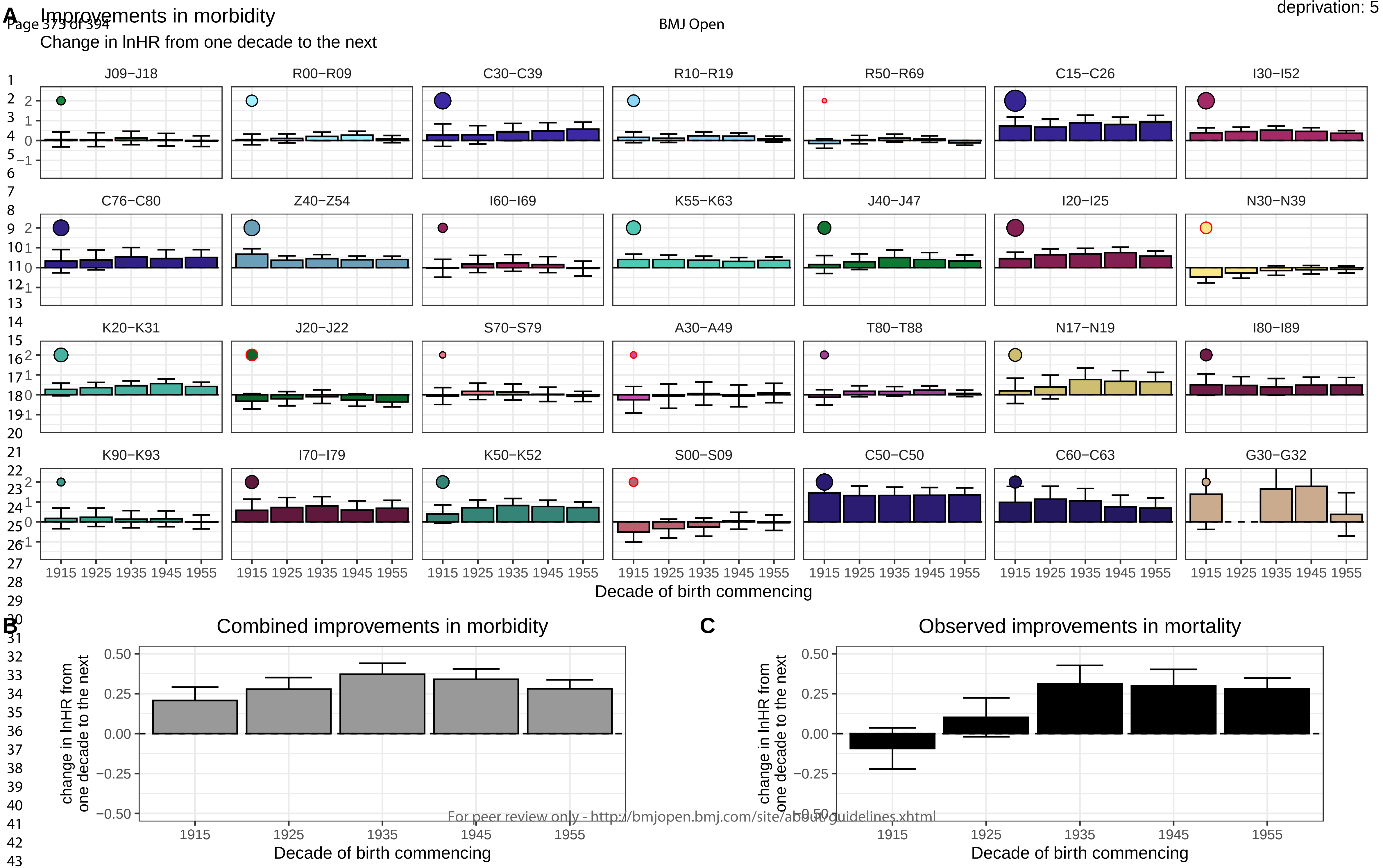
A
Improvements in morbidity
Change in lnHR from one decade to the next
deprivation: 5
J09−J18
R00−R09
C30−C39
R10−R19
R50−R69
C15−C26
I30−I52
C76−C80
Z40−Z54
I60−I69
K55−K63
J40−J47
I20−I25
N30−N39
K20−K31
J20−J22
S70−S79
A30−A49
T80−T88
N17−N19
I80−I89
K90−K93
I70−I79
K50−K52
S00−S09
C50−C50
C60−C63
G30−G32
2
1
0
−1
1915 1925 1935 1945 1955
Decade of birth commencing
B
Combined improvements in morbidity
change in lnHR from one decade to the next
0.50
0.25
0.00
−0.25
−0.50
1915
1925
1935
1945
1955
Decade of birth commencing
C
Observed improvements in mortality
change in lnHR from one decade to the next
0.50
0.25
0.00
−0.25
−0.50
1915
1925
1935
1945
1955
Decade of birth commencing

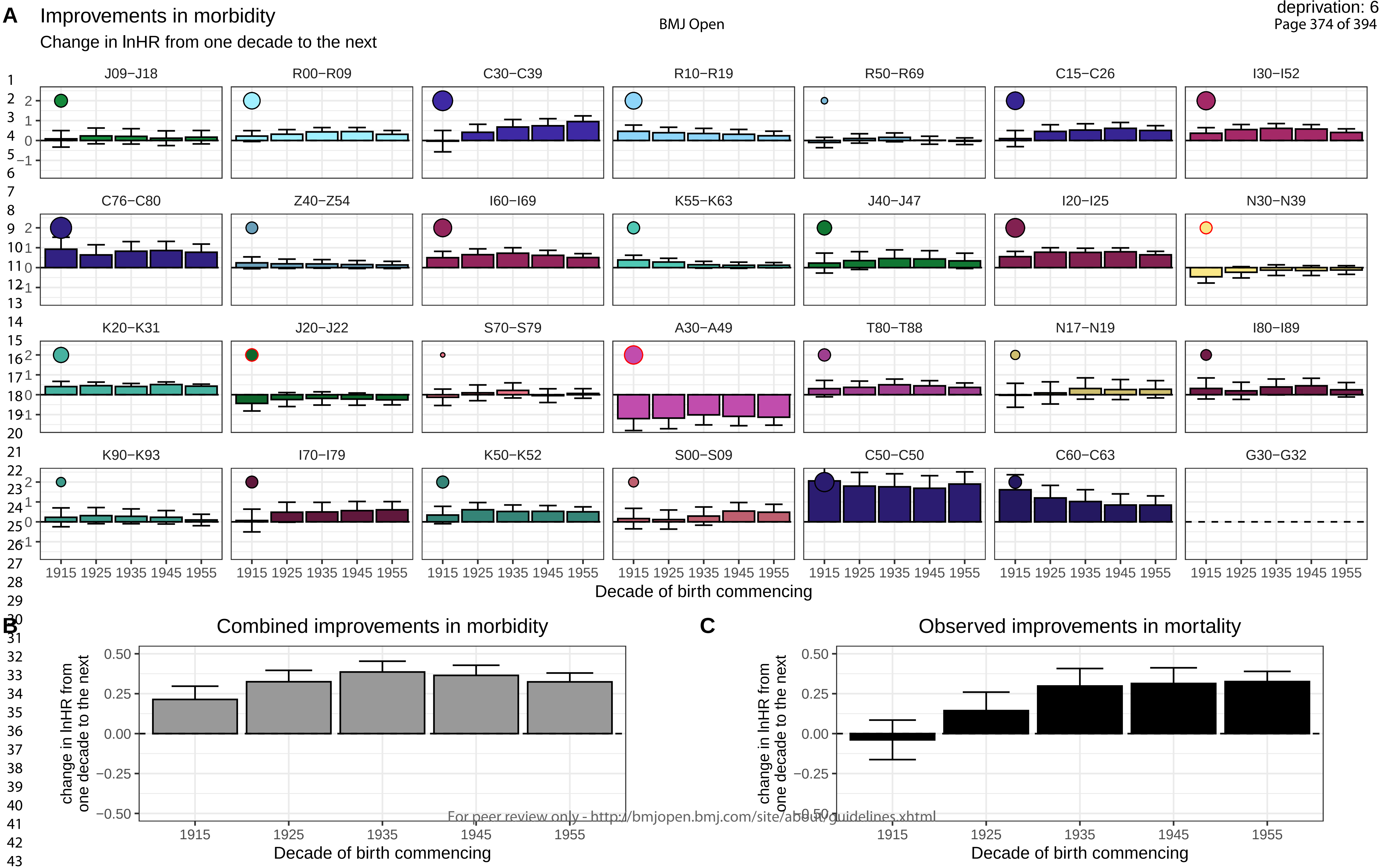

For peer review only - http://bmjopen.bmj.com/site/about/guidelines.xhtml

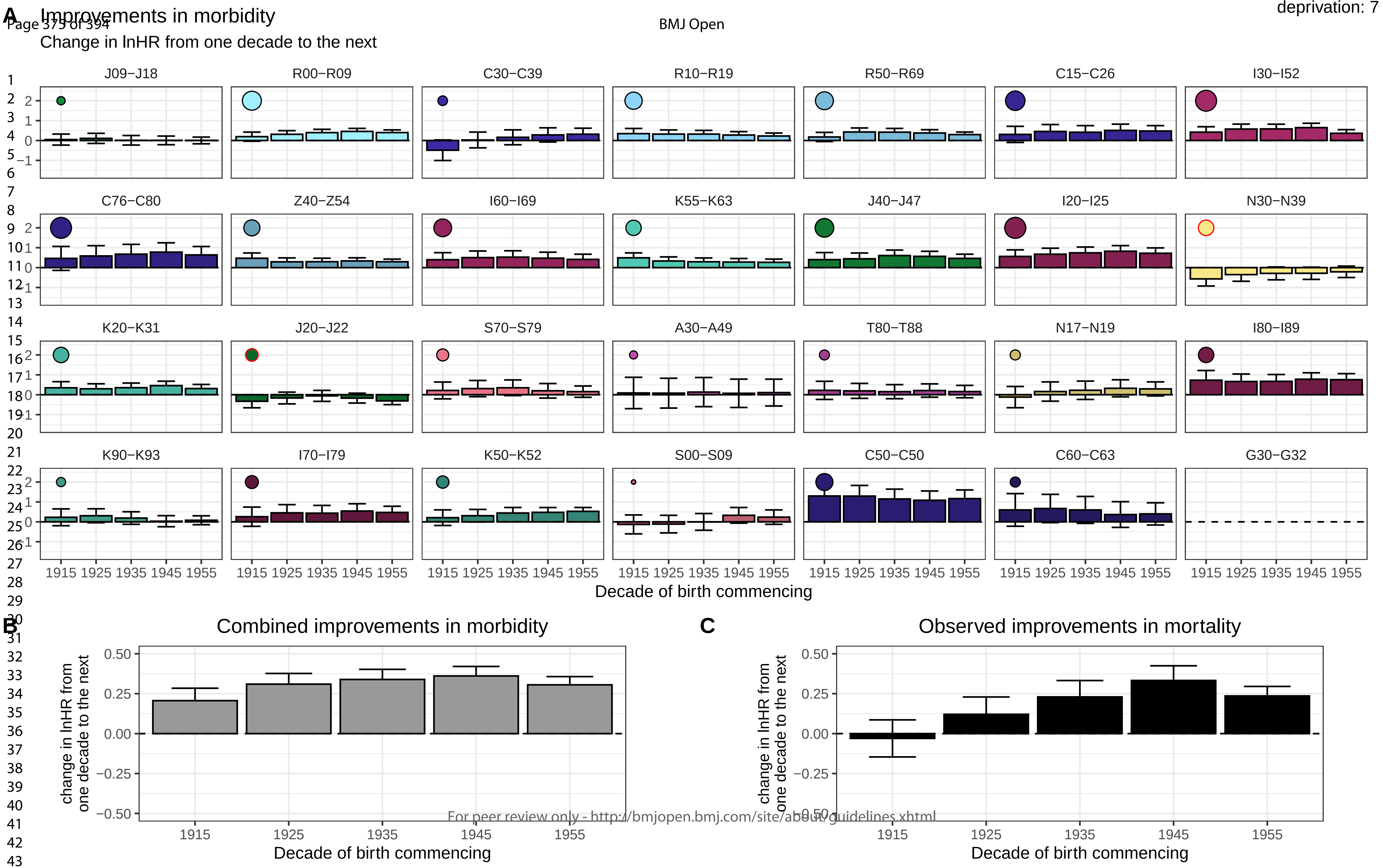

For peer review only - http://bmjopen.bmj.com/site/about/guidelines.xhtml

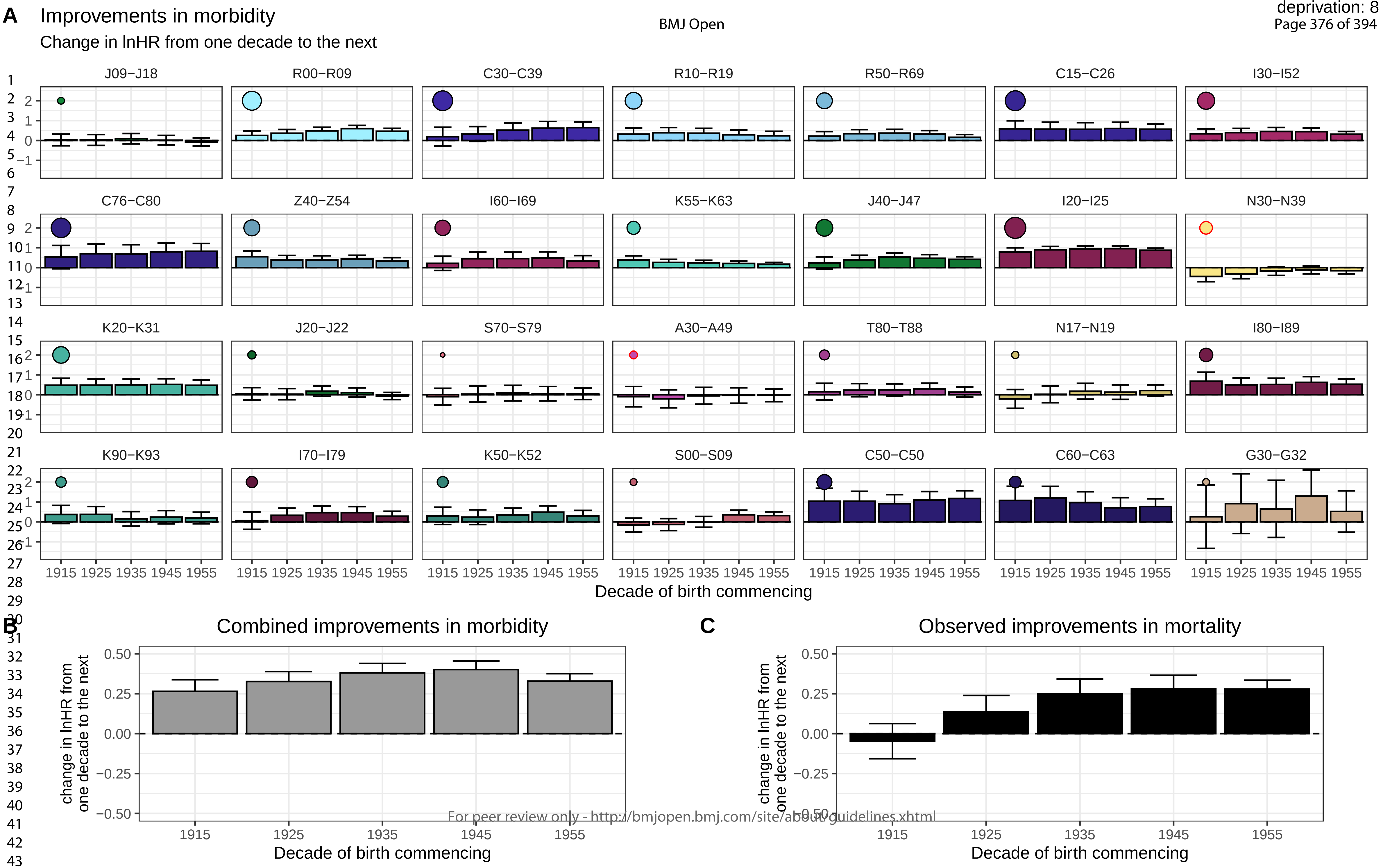

For peer review only - http://bmjopen.bmj.com/site/about/guidelines.xhtml

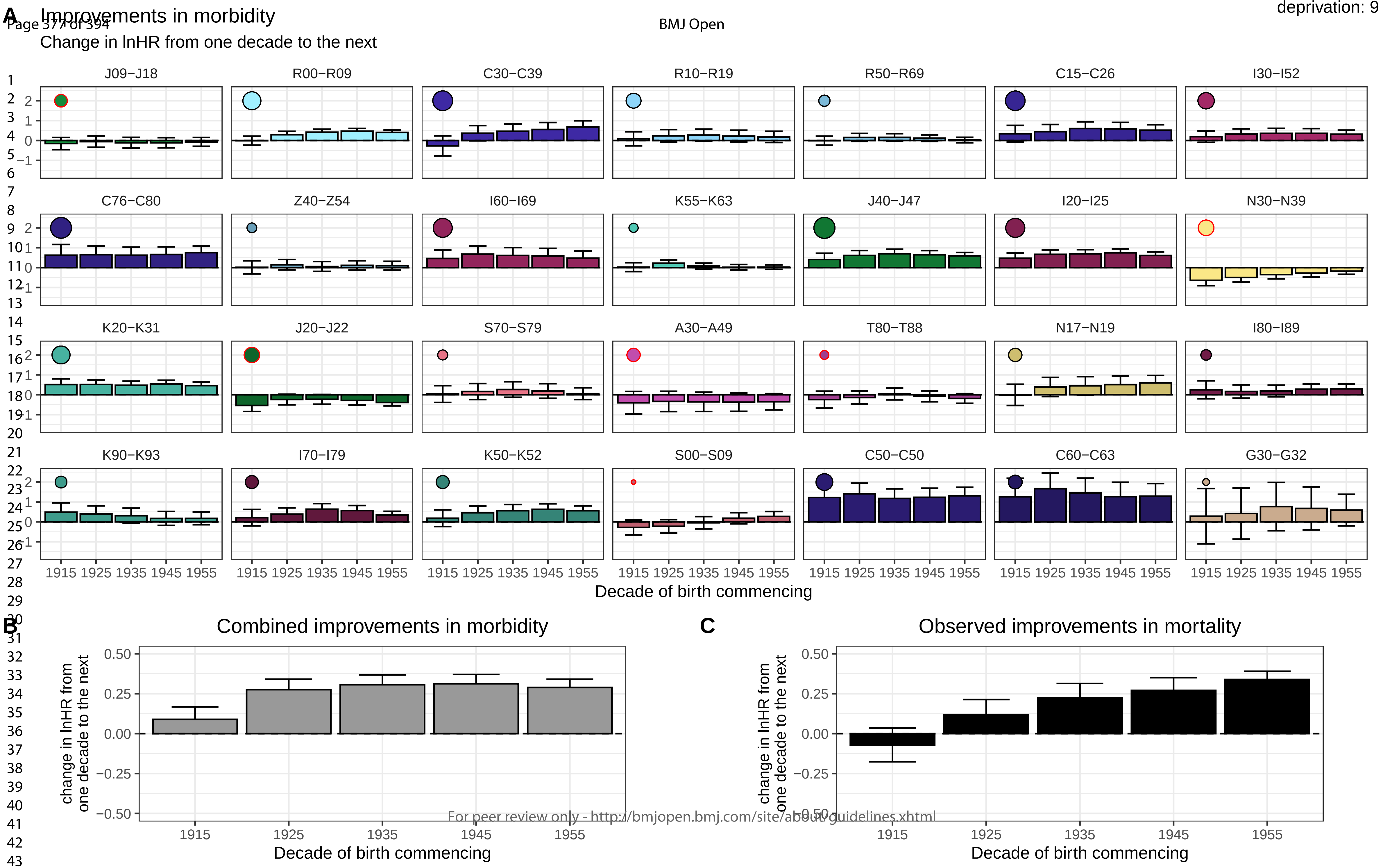
deprivation: 9
A
Improvements in morbidity
Change in lnHR from one decade to the next
J09–J18
R00–R09
C30–C39
R10–R19
R50–R69
C15–C26
I30–I52
C76–C80
Z40–Z54
I60–I69
K55–K63
J40–J47
I20–I25
N30–N39
K20–K31
J20–J22
S70–S79
A30–A49
T80–T88
N17–N19
I80–I89
K90–K93
I70–I79
K50–K52
S00–S09
C50–C50
C60–C63
G30–G32
2
1
0
−1
1915 1925 1935 1945 1955
Decade of birth commencing
B
Combined improvements in morbidity
change in lnHR from one decade to the next
0.50
0.25
0.00
−0.25
−0.50
1915
1925
1935
1945
1955
Decade of birth commencing
C
Observed improvements in mortality
change in lnHR from one decade to the next
0.50
0.25
0.00
−0.25
−0.50
1915
1925
1935
1945
1955
Decade of birth commencing

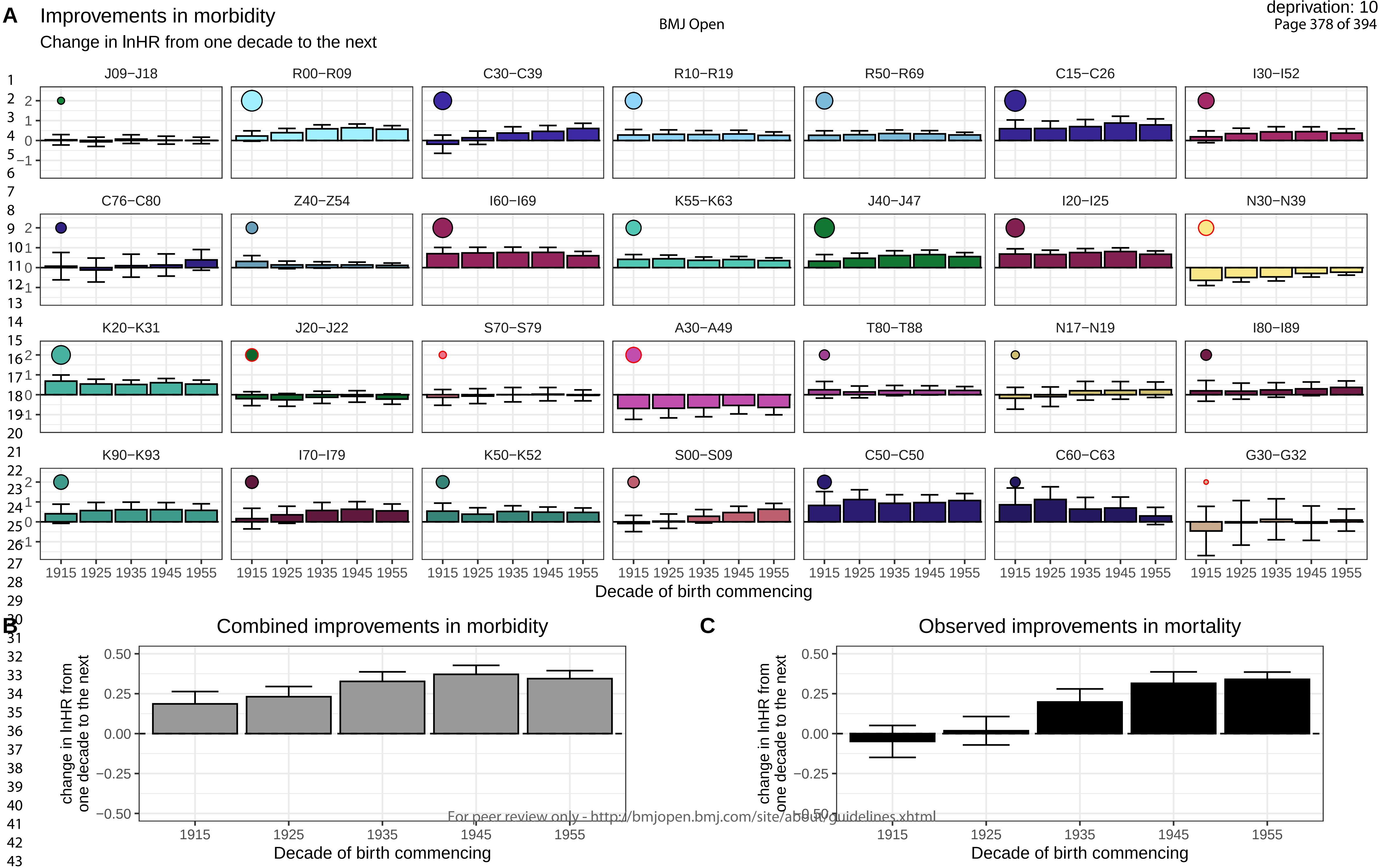
deprivation: 10
A
Improvements in morbidity
Change in lnHR from one decade to the next
J09−J18
R00−R09
C30−C39
R10−R19
R50−R69
C15−C26
I30−I52
C76−C80
Z40−Z54
I60−I69
K55−K63
J40−J47
I20−I25
N30−N39
K20−K31
J20−J22
S70−S79
A30−A49
T80−T88
N17−N19
I80−I89
K90−K93
I70−I79
K50−K52
S00−S09
C50−C50
C60−C63
G30−G32
1915 1925 1935 1945 1955
Decade of birth commencing
B
Combined improvements in morbidity
change in lnHR from one decade to the next
0.50
0.25
0.00
−0.25
−0.50
1915
1925
1935
1945
1955
Decade of birth commencing
C
Observed improvements in mortality
change in lnHR from one decade to the next
0.50
0.25
0.00
−0.25
−0.50
1915
1925
1935
1945
1955
Decade of birth commencing

Study population

Whilst necessary given the data structure, excluding all migrants during the study period will in many cases have excluded healthy people moving for economic or lifestyle opportunity. When health status influences the propensity to migrate, it forms a competing risk for both mortality and morbidity, which can bias results[1]. Due to the lack of mortality records on migrants, we are unable to assess the magnitude of this bias. Nevertheless, as we are principally concerned in the trend of morbidity and mortality, only changes in migration biases —not their absolute level—should affect our results materially.

Definition of diseases

We used recorded main position coding of first hospital admission of a disease block as the index event, excluding secondary diagnoses and any subsequent admissions for a disease within the same block. Hospital admission is only a proxy for the incidence of severe disease, and likely works better for chronic conditions like breast cancer where there is a single disease process that can result in a number of hospital admissions and may result in mortality in the timeframe of the study. It works perhaps less well for acute conditions, such as influenza, which can have distinct occurrences over the study period. Missing subsequent episodes of this type likely understates the burden of such diseases, although we expect trends in incidence and severity to be less affected by underreporting. Hospital admissions also have difficulty to capture chronic conditions which are managed in general practice or the community but eventually lead to death, such as dementia and multiple sclerosis. Although the CHI database captures this community illness through GP records, we only sought approval to access CHI data on patient date of birth and deprivation for this study, as GP records come with their own drawbacks (intractable scale, lack of precision, and heterogeneity between practices) and hospital admissions capture the incidence of most serious diseases. Future research may integrate GP records with our results to improve trend estimates of the diseases we were unable to model accurately.

Grouping of diseases into categories was another practical necessity; however, this grouping prevents us from assessing the trends of individual diseases, including the possibility of opposing trends offsetting each other. For example, the improvement seen in "General symptoms and signs involving the circulatory and respiratory systems" (R00-R09) does not reveal whether trends in "Pain in throat and chest" (R07) and "Abnormalities of breathing" (R06), the two most common diagnoses within the category (Table 2—Source Data 2), were directionally concordant.

Considerations with hospital admission data

Association between hospital admission for a condition and the latter occurrence of death does not necessarily imply causation, and may be confounded by other health risks, such as socioeconomic status, a second disease, or behavioural factors, although our analysis stratified by socioeconomic status may partly mitigate these effects. Should a patient have been admitted for several diseases prior to death, they would have been recorded as dying with all of those diseases. This is a strength in that diseases causing other diseases and in turn death are captured, although diseases not leading to death (DNLD) are also potentially captured as a cause of death. The latter is mitigated by the fact that subjects without DNLD should have been subject to similar other morbidity and mortality risks. The effect of DNLD across the population is thus properly measured. In any case, modelling comorbidities

For peer review only - http://bmjopen.bmj.com/site/about/guidelines.xhtml

simultaneously or modelling the causal effects of one morbidity on another and consequently on death has been beyond the scope of this study. Where an unanalysed comorbidity (for example, lung cancer amongst heart disease patients) is reducing across a dimension (such as over time, or increasing socioeconomic status), the reduction in the competing risk (lung cancer) will have led to a decrease in measured mortality amongst those with heart disease, but this would be counterbalanced to an unknown degree by a similar effect amongst those without heart disease. Future work might seek to consider this point using competing risk regression or site-specific survival.

By relying on routine data, we have been able to create a very large dataset, at the expense of being unable to externally check individual fields, such as primary diagnosis, where there are known to be inaccuracies[2]. However, our conclusions will be less affected if these inaccuracies are reasonably stable over time.

Changes in hospital admission rates could have been influenced by changes in referrals, waiting times, and disease screenings, especially if these changes differed by sex or socioeconomic status. For example, hospital waiting times in Scotland have fluctuated during the study period[3], coinciding with an increase in the demand for private healthcare[4], and an increase in the likelihood of patients' disease being managed in community care[5]. However, less than 10% of Scottish patients have private healthcare insurance and the NSS has not made a concerted change in hospital access for acute diseases[6]. Differences in access may well contribute to socioeconomic differences, but we would see them as a way in which the impact of deprivation on health is mediated rather than as a reason to discount the importance of deprivation as a health determinant. Conversely, changes in disease screening are likely to influence both hospital admission rates and subsequent survival. The UK has a long-established breast cancer screening programme, which evolved during the study period: the upper age for women to be screened was extended from 64 years old to 70 years old in 2004 and attendance rates fluctuated between 71%-76%, with its highest point in 2008[7]. The rate of overdiagnosis is around 11%[8], but whether this has changed over the study period is unknown.

Any increases in rate of screening and overdiagnosis would be reflected in our study by an improvement in disease survival and a deterioration in the incidence. One such example is the observed increase in incidence of influenza and pneumonia, which was offset by increased survival and may reflect changes in coding practices and/or frequency of referral[9]. For breast cancer, we observe an improvement in both incidence and survival, and this improvement is similar in shape those of prostate cancer, which does not have a national screening programme. As such, a degree of caution is needed in interpreting the exact partition between disease incidence and survival, but changes due to screening will have offset each other in terms of the overall change in morbidity.

Modelling assumptions

Our model assumed that (age-adjusted) incidence is a function of year of birth, but that mortality post-incidence is a function of the year of incidence. This is clearly an oversimplification, but necessary given that year of birth and year of incidence are completely confounded for a given age at incidence. Furthermore, we have used a simple definition of burden of disease: the total number of cases multiplied by the excess hazard across all ages. More complex definitions might have looked at Years of Life Lost (YLL) and taken into account

For peer review only

any age-related pattern in incidence. However, our measure is simple and is predominantly used to weight the diseases and especially their improvements to calculate an overall improvement in all-disease impact, rather than as an absolute measure of burden.

Our model also assumed disease incidence and survival hazards were proportionate. Disease status hazards were not always proportional across the baseline (age defined) hazard. Burden is thus sensitive to the population's age distribution, but is captured for the population under study (as the mean hazard increase across ages is captured). Furthermore, trends in hazard ratios measured should be robust given 1) the reasonably steady age distribution and 2) the shapes of mortality and incidence curves were constant over the study period. Conversely, recently doubt was cast on the effect of using decade of birth groupings in a standardised mortality analysis[10] due to changing patterns of births within the decade: that concern does not apply here as the Cox baseline hazard used full (not rounded) age information, with decade of birth fitted as a covariate. Using the cox model also allowed us to infer a baseline hazard across the whole range of ages (50+) , despite individuals only having been observed for 15 years, akin to constructing a period mortality table. However, further complexity was introduced by then calculating hazard ratios between successive decades of birth, relying upon a five year overlap in observed ages over 15 years in subjects born 10 years apart. Whilst if all the model assumptions hold, this is correct, it should not be used unthinkingly to infer trends in outcomes and especially differences between decades of birth prior to the study start. As such, there may be survivor biases in attempts to infer whole-of-life decade of birth inferences from our study. Nonetheless, we believe inferences of trends in disease and mortality and causal links remain valid with the period of study.

References

1 Andersen PK, Geskus RB, De witte T, *et al.* Competing risks in epidemiology: Possibilities and pitfalls. *Int J Epidemiol* 2012;**41**:861–70. doi:10.1093/ije/dyr213
2 ISD Scotland. Assessment of SMR01 Data Scotland 2014-2015. 2012.
3 Information Services Division. Inpatient and Day Case Waiting Times Trends: Long term trends. 1 April 1997 to 30 September 2018. 2019. https://beta.isdscotland.org/find-publications-and-data/healthcare-resources/waiting-times/inpatient-and-day-case-waiting-times-trends/ (accessed 20 Jan 2020).
4 Bíró A, Hellowell M. Public–private sector interactions and the demand for supplementary health insurance in the United Kingdom. *Health Policy (New York)* 2016;**120**:840–7. doi:10.1016/j.healthpol.2016.05.002
5 Information Services Devision. National Service Scotland: Acute Hospital Activity and NHS Beds Information. 2018.
6 Steel D, Cylus J. United Kingdom (Scotland): Health system review. *Health Syst Transit* 2012;**14**:xv–xxii, 1–150.http://www.ncbi.nlm.nih.gov/pubmed/23579054 (accessed 20 Jan 2020).
7 Information Services Division. Scottish Breast Screening Programme. 2018. https://www.isdscotland.org/Health-Topics/Cancer/Breast-Screening/ (accessed 20 Jan 2020).
8 Marmot MG, Altman DG, Cameron DA, *et al.* The benefits and harms of breast cancer screening: An independent review. *Br J Cancer* 2013;**108**:2205–40. doi:10.1038/bjc.2013.177
9 Nair H, Watts AT, Williams LJ, *et al.* Pneumonia hospitalisations in Scotland following the introduction of pneumococcal conjugate vaccination in young children. *BMC Infect*

Dis 2016;**16**:1–11. doi:10.1186/s12879-016-1693-x

10 Minton J, Green M, McCartney G, *et al.* Two cheers for a small giant? Why we need better ways of seeing data: A commentary on: 'Rising morbidity and mortality in midlife among White non-Hispanic Americans in the 21st century'. *Int J Epidemiol* 2017;**46**:356–61. doi:10.1093/ije/dyw095

For peer review only

The RECORD statement – checklist of items, extended from the STROBE statement, that should be reported in observational studies using routinely collected health data.

	Item No.	STROBE items	Location in manuscript where items are reported	RECORD items	Location in manuscript where items are reported
**Title and abstract**					
	1	(a) Indicate the study's design with a commonly used term in the title or the abstract (b) Provide in the abstract an informative and balanced summary of what was done and what was found	Page 1: Title & Abstract<br><br>(a) "nationwide cohort study of linked hospital admission and death records"<br><br>(b) See "Main outcome measures" "Results" "Conclusion"	RECORD 1.1: The type of data used should be specified in the title or abstract. When possible, the name of the databases used should be included.<br><br>RECORD 1.2: If applicable, the geographic region and timeframe within which the study took place should be reported in the title or abstract.<br><br>RECORD 1.3: If linkage between databases was conducted for the study, this should be clearly stated in the title or abstract.	Page 1: Title<br><br>1.1/1.2/1.3 "Scotland: nationwide cohort study of linked hospital admission and death records 2001–2016"
**Introduction**					
Background rationale	2	Explain the scientific background and rationale for the investigation being reported	Page 4: "…several studies have suggested that there has been slowdown in [life expectancy] improvements… however, the causes are less clear…"<br><br>Page 4: "Understanding the trends … has the potential to reveal		

For peer review only

			whether investment in healthcare and research is directed at the most urgent diseases and most affected individuals."		
Objectives	3	State specific objectives, including any prespecified hypotheses	Page 4: "…explore how trends in longevity were driven by different trends in broad classes of disease incidence or survival, and highlight diseases which have shown more or less improvement in their contribution to overall mortality."		
**Methods**					
Study Design	4	Present key elements of study design early in the paper	See Page 5: Data Sources & Page 6: Design		
Setting	5	Describe the setting, locations, and relevant dates, including periods of recruitment, exposure, follow-up, and data collection	See Page 5: Data Sources		
Participants	6	*(a) Cohort study* - Give the eligibility criteria, and the sources and methods of selection of participants. Describe methods of follow-up *Case-control study* - Give the eligibility criteria, and the sources and methods of case	(a) Page 5: Data Sources "The final study population included all 1,967,130 individuals born between 1905 and 1965 who registered	RECORD 6.1: The methods of study population selection (such as codes or algorithms used to identify subjects) should be listed in detail. If this is not possible, an explanation should be provided.	6.1/6.2 Page 5: Data Sources "The CHI number, contained in the dataset, is effectively a

For peer review only

		ascertainment and control selection. Give the rationale for the choice of cases and controls<br>*Cross-sectional study* - Give the eligibility criteria, and the sources and methods of selection of participants<br><br>*(b) Cohort study* - For matched studies, give matching criteria and number of exposed and unexposed<br>*Case-control study* - For matched studies, give matching criteria and the number of controls per case	with the NSS, were resident in Scotland during the study period, and had complete and reliable records on their date of birth, socioeconomic status, and death (if applicable)."<br><br>(b) N/A	RECORD 6.2: Any validation studies of the codes or algorithms used to select the population should be referenced. If validation was conducted for this study and not published elsewhere, detailed methods and results should be provided.<br><br>RECORD 6.3: If the study involved linkage of databases, consider use of a flow diagram or other graphical display to demonstrate the data linkage process, including the number of individuals with linked data at each stage.	patient identifier and added to other health datasets to make linkage possible, for instance between hospital admissions, death records and the Scottish cancer registry (reference to paper describing linkage)"<br><br>6.3<br>Linkage described in Page 5: Data Sources, with S Figure 1 showing QC.
Variables	7	Clearly define all outcomes, exposures, predictors, potential confounders, and effect modifiers. Give diagnostic criteria, if applicable.	Page 9: Summary of outcomes, with details of each of the outcomes, predictors, and covariates in the sections under Statistical Analysis. (Page 6-8)	RECORD 7.1: A complete list of codes and algorithms used to classify exposures, outcomes, confounders, and effect modifiers should be provided. If these cannot be reported, an explanation should be provided.	7.1<br>Page 6: "main diagnoses of acute hospital admission records … as laid out by the ICD10 Chapters". Sections under Statistical Analysis describe algorithms (Page 6-8)
Data sources/ measurement	8	For each variable of interest, give sources of data and details of methods of assessment (measurement).	Page 6, 7:<br>"A Cox proportional hazards model using NRS mortality data"		

For peer review only

		Describe comparability of assessment methods if there is more than one group	"We grouped the main diagnoses of each NSS hospital admission into categories, as laid out by the ICD10 Chapters"<br><br>"relative mortality burden of each disease block was calculated as the excess mortality (lnHR) in the 5 years after hospital admission (Equation 1) multiplied by the number of first-time admissions for the disease block"		
Bias	9	Describe any efforts to address potential sources of bias	Page 8: "Whilst this measure may in principle be affected by differing age patterns on incidence, it was judged sufficient for our purpose – to establish broad relative weightings of the importance of each disease."<br><br>Page 8: "The use of (first) hospitalisation for a disease as our definition of		

For peer review only

			incidence is imperfect"		
Study size	10	Explain how the study size was arrived at	Page 7: Data Sources – Community Health Index dataset "we excluded individuals with district codes with less than 5,000 individuals … individuals missing from the CHI database in 2016, but not recorded as dead … individuals with record discrepancies … individuals with records outside of the study dates … giving 1,967,130 individuals for analysis post-QC"		
Quantitative variables	11	Explain how quantitative variables were handled in the analyses. If applicable, describe which groupings were chosen, and why	Page 7: "In order to quantify mortality in the 5 years following hospitalisation, person-time of individuals was divided into phases, corresponding to the study start until hospitalisation, the first 5 years after hospitalisation, and		

For peer review only

			the remaining time in the study."<br><br>"The status of each phase was fitted as a covariate in a Cox proportional hazards model with death as the event, adjusting for sex and deprivation"		
Statistical methods	12	(a) Describe all statistical methods, including those used to control for confounding<br>(b) Describe any methods used to examine subgroups and interactions<br>(c) Explain how missing data were addressed<br>(d) *Cohort study* - If applicable, explain how loss to follow-up was addressed<br>*Case-control study* - If applicable, explain how matching of cases and controls was addressed<br>*Cross-sectional study* - If applicable, describe analytical methods taking account of sampling strategy<br>(e) Describe any sensitivity analyses	Page 6-8:<br>(a) "Cox proportional hazard models"<br><br>(b) "we fit sex, deprivation, and decade of birth as covariates. Again, the same analyses were performed when stratifying by sex and deprivation"<br><br>(c) "only individuals with complete records were included in the analysis."<br><br>(d) "We also excluded individuals missing from the CHI database in 2016, but not recorded as dead (and therefore likely		

For peer review only

			transferred out of Scotland; N = 573,711)"<br><br>(e) N/A		
Data access and cleaning methods		..		RECORD 12.1: Authors should describe the extent to which the investigators had access to the database population used to create the study population.<br><br>RECORD 12.2: Authors should provide information on the data cleaning methods used in the study.	12.1 Page 5: "The dataset we received contained only records with district-level postcodes and SIMD deciles"<br><br>12.2 Page 5: "we excluded individuals…"
Linkage		..		RECORD 12.3: State whether the study included person-level, institutional-level, or other data linkage across two or more databases. The methods of linkage and methods of linkage quality evaluation should be provided.	12.3 Page 5: "The CHI number, contained in the dataset, is effectively a patient identifier and added to other health datasets to make linkage possible, for instance between hospital admissions, death records and the Scottish cancer registry"
**Results**					

For peer review only

Participants	13	(a) Report the numbers of individuals at each stage of the study (*e.g.*, numbers potentially eligible, examined for eligibility, confirmed eligible, included in the study, completing follow-up, and analysed)<br>(b) Give reasons for non-participation at each stage.<br>(c) Consider use of a flow diagram	Page 5: Community Health Index dataset & S Figure 1 & S Figure 1—Source Data 1	RECORD 13.1: Describe in detail the selection of the persons included in the study (*i.e.*, study population selection) including filtering based on data quality, data availability and linkage. The selection of included persons can be described in the text and/or by means of the study flow diagram.	13.1 Page 5: "We also excluded individuals missing from the CHI database in 2016, but not recorded as dead (and therefore likely transferred out of Scotland; N = 573,711), individuals with record discrepancies between the CHI and National Records of Scotland databases (N = 79,131), and individuals with records outside of the study dates or missing information on socioeconomic class (N = 59,767),"
Descriptive data	14	(a) Give characteristics of study participants (*e.g.*, demographic, clinical, social) and information on exposures and potential confounders<br>(b) Indicate the number of participants with missing data for each variable of interest	Table 1, Table 1—Source Data 1, S Figure 1, S Figure 1—Source Data 1		

For peer review only

		(c) *Cohort study* - summarise follow-up time (*e.g.*, average and total amount)			
Outcome data	15	*Cohort study* - Report numbers of outcome events or summary measures over time<br>*Case-control study* - Report numbers in each exposure category, or summary measures of exposure<br>*Cross-sectional study* - Report numbers of outcome events or summary measures	Table 1, Table 1—Source Data 1		
Main results	16	(a) Give unadjusted estimates and, if applicable, confounder-adjusted estimates and their precision (e.g., 95% confidence interval). Make clear which confounders were adjusted for and why they were included<br>(b) Report category boundaries when continuous variables were categorized<br>(c) If relevant, consider translating estimates of relative risk into absolute risk for a meaningful time period	(a) Page 10: "age-adjusted mortality rates compared to men (hazard ratio 0.71; 95% CI 0.70–0.71)"<br><br>(b) N/A<br><br>(c) Page 11: "68.0% higher for the most deprived decile (188,905 individuals with 331,701 first-time admissions)"		
Other analyses	17	Report other analyses done—e.g., analyses of subgroups and interactions, and sensitivity analyses	Page 12: "Across sex and deprivation strata"		
**Discussion**					
Key results	18	Summarise key results with reference to study objectives	Page 14: "Cancers (C), cardiovascular disease (I),		

For peer review only

			respiratory diseases (J), and unclassified symptoms and signs (R) were the principal ICD10 chapters recurring in the top 28 disease blocks associated with total death "<br><br>"we modelled a reduction in mortality hazard of 0.737 (95% CI 0.730–0.745) due to improvements in morbidity"		
Limitations	19	Discuss limitations of the study, taking into account sources of potential bias or imprecision. Discuss both direction and magnitude of any potential bias	Page 15: "However, this study also has a number of limitations." & Supplementary Note 1	RECORD 19.1: Discuss the implications of using data that were not created or collected to answer the specific research question(s). Include discussion of misclassification bias, unmeasured confounding, missing data, and changing eligibility over time, as they pertain to the study being reported.	Page 15: "the first hospital admission on record and its date is only a proxy for incidence of severe disease."<br><br>Supplementary Note 1: "By relying on routine data, we have been able to create a very large dataset, at the expense of being unable to externally check individual fields, such as primary diagnosis"

For peer review only

					Page 16: "Another consideration with hospital records is their indirect link to death. This relationship can be confounded on the one hand by other health risks and lifestyle factors, and on the other hand by coding inaccuracies and changes in admission policies and screening"
Interpretation	20	Give a cautious overall interpretation of results considering objectives, limitations, multiplicity of analyses, results from similar studies, and other relevant evidence	Page 17: "Much of the improvements in mortality observed in Scotland between 2001–2016 can be attributed to improved morbidity"<br><br>Page 17: "coincide with a continued effort within Scotland, the UK, and other developed nations to improve prevention and care"<br><br>Page 17: "global increases in		

For peer review only

			antimicrobial resistance”		
Generalisability	21	Discuss the generalisability (external validity) of the study results	Page 17: “While this study examined mortality and morbidity in the Scottish population only, there is a substantial concordance in mortality trends across high-income countries, as well as similarities in disease-related mortality trends between Scotland and the rest of the UK, warranting similar studies to be performed in other high-income countries”		
**Other Information**					
Funding	22	Give the source of funding and the role of the funders for the present study and, if applicable, for the original study on which the present article is based	Page 18: “[Lloyds Banking Group] reviewed the design and said that adding stratification by socioeconomic status was a key requirement for meaningful analysis and their funding was conditional upon this, a request to which the authors readily agreed. The		

For peer review only

			funder was kept informed of interim analyses and reviewed the draft manuscript, occasionally suggesting additional analyses or requesting clarifications. The funders were not permitted to and did not request the removal of any results."		
Accessibility of protocol, raw data, and programming code		..		RECORD 22.1: Authors should provide information on how to access any supplemental information such as the study protocol, raw data, or programming code.	22.1 Page 18: "Individual de-identified participant data cannot be shared."

*Reference: Benchimol EI, Smeeth L, Guttmann A, Harron K, Moher D, Petersen I, Sørensen HT, von Elm E, Langan SM, the RECORD Working Committee. The REporting of studies Conducted using Observational Routinely-collected health Data (RECORD) Statement. *PLoS Medicine* 2015; in press.

*Checklist is protected under Creative Commons Attribution (CC BY) license.

For peer review only
